# An Integrated Approach for the Analysis of Biological Pathways using Mixed Models

**DOI:** 10.1371/journal.pgen.1000115

**Published:** 2008-07-04

**Authors:** Lily Wang, Bing Zhang, Russell D. Wolfinger, Xi Chen

**Affiliations:** 1 Department of Biostatistics, Vanderbilt University, Nashville, Tennessee, United States of America; 2 Department of Biomedical Informatics, Vanderbilt University, Nashville, Tennessee, United States of America; 3 SAS Institute, Inc., Cary, North Carolina, United States of America; 4 Department of Quantitative Health Sciences, The Cleveland Clinic, Cleveland, Ohio, United States of America; University of Alabama at Birmingham, United States of America

## Abstract

Gene class, ontology, or pathway testing analysis has become increasingly popular in microarray data analysis. Such approaches allow the integration of gene annotation databases, such as Gene Ontology and KEGG Pathway, to formally test for subtle but coordinated changes at a system level. Higher power in gene class testing is gained by combining weak signals from a number of individual genes in each pathway. We propose an alternative approach for gene-class testing based on mixed models, a class of statistical models that:

- provides the ability to model and borrow strength across genes that are both up and down in a pathway,
- operates within a well-established statistical framework amenable to direct control of false positive or false discovery rates,
- exhibits improved power over widely used methods under normal location-based alternative hypotheses, and
- handles complex experimental designs for which permutation resampling is difficult.

We compare the properties of this mixed models approach with nonparametric method GSEA and parametric method PAGE using a simulation study, and illustrate its application with a diabetes data set and a dose-response data set.

## Author Summary

In microarray data analysis, when statistical testing is applied to each gene individually, one is often left with too many significant genes that are difficult to interpret or too few genes after a multiple comparison adjustment. Gene-class, or pathway-level testing, integrates gene annotation data such as Gene Ontology and tests for coordinated changes at the system level. These approaches can both increase power for detecting differential expression and allow for better understanding of the underlying biological processes associated with variations in outcome. We propose an alternative pathway analysis method based on mixed models, and show this method provides useful inferences beyond those available in currently popular methods, with improved power and the ability to handle complex experimental designs.

## Introduction

To help increase power to detect microarray differential expression and to better interpret findings, gene-class testing or pathway analysis has become increasingly popular [1]. These approaches allow the integration of gene annotation databases such as Gene Ontology [2] and KEGG Pathway [3] to formally test for subtle but coordinated changes at the system level. Improved power of gene-class testing is gained by combining weak signals from a number of individual genes in each pathway. In addition, pathway analysis has been effectively used to examine common features between data sets [4].

The most commonly used approach for pathway analysis, the enrichment or overrepresentation analysis, uses Fisher's exact test. This method starts with a list of differentially expressed genes based on an arbitrary cutoff of nominal p-values, and compares the number of significant genes in the pathway to the rest of the genes to determine if any gene-set is overrepresented in the significant gene list. The Fisher's exact test is implemented in a number of software packages such as GOTM [5], WebGestalt [6], GENMAPP [7], ChipInfo [8], ONTO-TOOLS [9], GOstat [10], DAVID [11], and JMP Genomics (http://www.jmp.com/genomics). Although straightforward to implement and interpret, this method loses information by using only the significant genes resulted from arbitrarily dichotomizing p-values at some threshold.

More recent approaches such as Gene Set Enrichment Analysis (GSEA) [12],[13] and its extensions use continuous distributions of evidence for differential expression and are based on a modified version of the Kolmogorov-Smirnov test that compares the distribution of test statistics in a pathway to the test statistics for the rest of the genes. However, as explained in [14], the specific alternative hypothesis for coordinated association between genes in a gene-set with phenotype is likely to be a location change from background distribution. The Kolmogorov-Smirnov test used by GSEA, which detects any changes in the distribution, is often not optimally powerful for detecting specific location changes. In addition, false positives may result when genes in a gene-set have different variances compared with genes outside the pathway. Methods that test for location changes include PAGE [15] and Functional Class Scoring [16]. PAGE uses normal distribution to approximate test statistics based on differences in means for gene-set genes and other genes; Functional Class Scoring method computes mean (-log(p-value)) from p-values for all genes in a gene-set, and compares this raw score to an empirically derived distribution of raw scores for randomly selected gene-sets of the same size using a statistical resampling approach.

Other examples of permutation- and bootstrap-based methods include SAFE [17], iGA [18] and GSA [19]. However, resampling-based methods rely on exchangeability that may be hard to achieve in complex experimental designs. For example, in designs with multiple random effects and/or time-series covariance structures, great care must be taken to achieve an appropriate resampling-based null distribution. In this paper, we propose an alternative, parametric approach for gene-class testing based on mixed linear models [20], which can readily accommodate complex designs under standard parametric assumptions.

Some parametric methods and their comparisons with the proposed method are in order. Wolfinger et al. [21] and Chu et al. [22] considered using mixed models for detecting differentially expressed genes for cDNA and Affymetrix microarrays. Ng et al. [23] proposed random effects models to cluster gene expression profiles, but their gene-sets are derived by statistical learning, not based on biological knowledge. Other parametric models include the random effect model of Goeman et al. [24] and the ANCOVA model of Mansmann [25] for testing whether a particular gene-set contains any gene associated with outcome. There is an important distinction between these models and our proposed model. Tian et al. [14] formulated two statistical hypothesis for testing coordinated association between a group of genes with a phenotype of interest: hypothesis Q1 - The genes in a gene-set show the same pattern of associations with the phenotype compared with the rest of the genes; and hypothesis Q2 - The gene-set does not contain any genes whose expression levels are associated with the phenotype of interest. Goeman et al. [24] and Mansmann et al. [25] both test Q2 whereas our proposed model tests Q1. The most similar parametric method with our proposed model that tests Q1 is PAGE [15] mentioned above; test statistics for both PAGE and the proposed method are based on differences in means for gene-set genes and other genes. Our method can be viewed as an extension of PAGE with the ability to account for design of experiment (e.g. covariate adjustment) and modeling dependency between genes with a more general covariance structure.

In Materials and Methods, we describe the proposed mixed model, including assumptions and interpretations. This model incorporates both fixed effects (e.g. type of tissues, cases vs. controls) and random effects which are assumed to be sampled from a normal distribution and naturally fall into a hierarchical empirical Bayes framework. The inclusion of random effects both facilitates inferences to be made to the underlying population represented by the observed samples and is a simple mechanism for modeling a covariance structure within groups of correlated observations. Another advantage is that mixed models provide a powerful, unified and flexible framework that allows one to conduct hypothesis testing for gene-sets and accounting for other design factors at the same time. With mixed models, between-arrays normalization, adjusting for covariates and gene-set testing are achieved in a single step; in contrast, other gene-class testing methods usually require separate data processing steps for normalization, assessing statistical significance of individual genes using a test statistics such as the t-score, and comparison of the test statistics for genes in the pathway with non-pathway genes. In the Results section, we first confirm the increased power over the nonparametric method GSEA and parametric method PAGE using simulations and then illustrate the method using two microarray datasets, a human diabetic muscle dataset [12] and a dose-response study [26]. In the Discussion section, we provide some concluding comments.

## Materials and Methods

Given two groups of samples and an *a priori* defined set of genes from a particular pathway, we are interested in testing whether the differential expression between the groups are significantly different for genes in the pathway compared with the rest of the genes. For sake of concreteness we assume without loss of generality the two groups of samples are from patients with a disease phenotype (cases) and otherwise (controls).

### Data Preprocessing

We assume reliable numerical values are obtained from gene expression intensities and are on the log2 scale. In single colored arrays, the expression values for each gene are derived from each spot on the array; in two-colored arrays, the expression values for each gene can be the original intensities or the ratios of expression values for experimental sample compared to reference sample. When multiple probe sets for a gene are present, they can be mapped to some standard gene IDs such as the Ensembl Gene IDs (http://www.ensembl.org) and the median is used for further analysis. This is often done for computational efficiencies of larger arrays. In the following discussion, we assume there is one value for each gene, at the end of the Discussion section, we discuss extensions of basic mixed model to accommodate multiple gene expression values per gene.

Next, to homogenize variances for all the genes included in mixed model and to make their means comparable, we standardize values for each gene with control group mean and standard deviations. Specifically, the mean and standard deviation of each gene from control patients are calculated, and all the gene values are standardized by subtracting the control group mean and dividing by the control group standard deviation. The standardized gene expression values then represent the number of standard deviations away from the “normal” gene expression values. In a time course experiment, expression values at baseline can be used similarly as control group data to standardize all measurements in the time course.

### Linear Mixed Model

Linear mixed models is a class of statistical models that handles data where observations are not independent, such as gene expression values from the same array. They include both fixed effects and random effects, and thus are called mixed effect models. The fixed effects model the systematic effects or the mean structure of data, and the random effects account for complex covariance structure of observations, such as those between genes. In addition, they also allow inferences to be made to the entire population of samples from which the observed samples arise.

Assuming after data pre-processing, there are one measurement per gene from each array, we propose the following basic linear mixed models for comparing differential expression pattern in the pathway (or gene-set) *m* and the rest of genes:
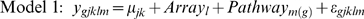
Here, *y* represents log transformed gene expression values, *j* = 1 if gene *g* is from the pathway *m* and *j* = 0 otherwise; *k* = 1 for case values and *k* = 0 for control values. The parameters *μ_jk_* model systematic effects or fixed effects affecting gene expression values, and correspond to a classical cell-means model [27]. The fixed effects portion of Model 1 is equivalent to a model with intercept, indicator variable Group (case or control), indicator variable Pathway *m* (yes or no), and Group×Pathway *m* interaction effects. Although Model 1 does not include gene-specific fixed effects, we account these through standardization of gene values (Data Preprocessing in Materials and Methods) which makes expression values from different genes comparable and homogenizes their variances.

While *μ_jk_* are fixed unknown parameters to be estimated from data, the terms *Array_l_* and *Pathway_m_*_(*g*)_ for *l*-th array and *m*-th pathway are random variables, we use the subscript *(g)* to emphasize values for *Pathway* random effects vary according to genes. We discuss in detail the construction of these random effects and the specific covariance structure accounted by them in the Materials and Methods section. Finally, *ε* represents variations due to measurement error and we assume *ε_gjklm_*∼*N*(0, *σ* ^2^). Parameters from the mixed model are estimated using the method of restricted maximum likelihood (REML) along with appropriate standard errors.

The hypothesis we are testing is whether the amount of differential expression between cases and controls for gene-set genes are significantly different from the other genes. This is essentially the interaction effect between gene-set and group. In terms of Model 1, we want to test *H*_0_:(*μ*_11_−*μ*_10_)−(*μ*_01_−*μ*_00_) = 0. Here, *μ*_11_−*μ*_10_ represents differential expression for genes in the pathway and *μ*_01_−*μ*_00_ represents differential expression for the rest of the genes.

In feedback or reverse regulation, in response to an input signal, genes in a gene-set may shift in both directions, that is, a fraction of gene-set genes are up-regulated and another fraction of gene-set genes are down-regulated, then testing changes in the entire gene-set will not be effective as the changes in different directions will cancel each other out. Instead, we propose modeling reverse regulation with
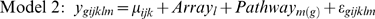
where *i* indicates direction of changes for gene *g*, *i* = 1 for up-regulated genes and *i* = 0 for down-regulated genes. With this model, we estimate <1?show=[to]?>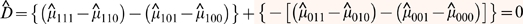 where 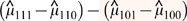 estimates amount of up-regulation and 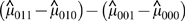 estimates amount of down-regulation.

Because the direction of change *i* for each gene is estimated from data, the hypothesis we are testing in this case is equivalent to *H*_0_:{[(*μ*_11_−*μ*_10_)−(*μ*_01_−*μ*_00_)|*i* = 1]−[(*μ*_11_−*μ*_10_)−(*μ*_01_−*μ*_00_)|*i* = 0]} = 0. Therefore, 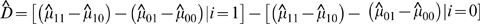 is the difference of two conditional random variables, its distribution is a skewed unimodal distribution and can not be approximated well using normal distribution. We propose a Box-Cox transformation [28] of the test statistics to account for this. Specifically, to test for significance of *n* (e.g. 500) gene-sets, we follow these steps:

1. Generate gene expression values for *n* “null gene-sets”, see details below.
2. For each null gene-set, fit Model 2 to data and calculate t-statistics *T_D_* corresponding to estimate *Dˆ*.
3. Consider t-statistics for all null gene-sets, let *T_D_*_+_ = *T_D_*−min(*T_D_*) where min(*T_D_*) = minimum over all t-statistics, so that *T_D_*_+_≥0. The Box-Cox transformation of 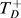 is defined by 
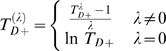
 where *λ* maximizes the function 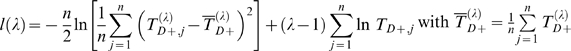 and 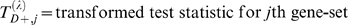. The Box-Cox transformation ensures the transformed variable 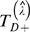 can be well approximated by a normal distribution.
4. With estimated 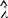, apply the Box-Cox transformation to t-statistics corresponding to those gene-sets to be tested to obtain 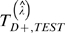. Here, *T_D_*_+,*TEST*_ is calculated by subtracting minimum from t-statistics of all gene-sets to be tested. The p-value for a particular gene-set *j* with t-statistics *t* can then be approximated by 
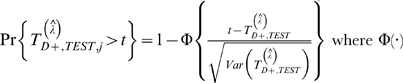
 where Φ(·) is c.d.f. of standard normal distribution.

We use the Monte Carlo simulation approach [29] to simulate gene expression values with the same covariance structure as those in real microarray data. First, we fit Model 2 to real microarray data and estimate covariance parameters corresponding to variance components for random effects and residual errors ***ε***. Next, we simulate gene values with random effects and errors generated according to the estimated covariance parameters. Because the dependency between genes are captured approximately by random effects and covariance parameters in mixed models (Materials and Methods), the simulated gene expression values will have essentially the same covariance structure as gene values in real microarray data. Also, since no fixed effects were added, the simulated data do not depend on outcome and therefore correspond to null gene-sets values.

Once we obtain nominal p-values from steps described above, we next calculate adjusted p-values to control for False Discovery Rate (FDR). An adjusted p-value of 0.05 for a gene set indicates that among all significant gene sets selected at this threshold, 5 out 100 of them are expected to be false leads.

### Random Effects and Covariance Structure Modeled by Mixed Model

In Models 1 and 2, we assume normal distributions for the random effects: 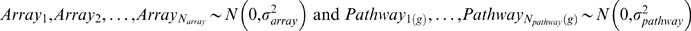. Here, the *Array* random effects model effects due to sample variations and *Pathway* random effects represent variations associated with different biological processes defined by pathways. The random effects have the advantage of requiring only a single parameter (e.g. 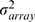), the variance component, to be estimated. In the simulation study we accommodate 50 pathways simultaneously. For real microarray dataset, one can also construct a separate pathway “other” to include all genes not belonging to any gene-sets to be tested.

Another important advantage of random effects is that they help capture the heterogeneous covariations across genes. In particular, the *Array* random effects account for covariance among all observations from the same array and *Pathway* random effects account for covariance among genes from the same pathway. Note that the random *Pathway* effects vary according to genes, to model different amount of dependencies between pairs of genes. We discuss the specific covariance structure accounted by these random effects and their constructions in details next.

The *Array* random effects are constructed as indicator variables for each array, that is, *Array_l_* = *I*{array *l*}. To construct the *Pathway* random effects, first, calculate t-statistics for each gene based on observed data. Let 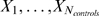 be gene expression values from control samples, and 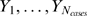 be gene expression values from case samples. Compute 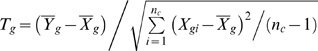 where *X̅* *_g_* and *Y̅* *_g_* are average gene values for control samples and case samples respectively. Next, we construct *Pathway_m_*_(*g*)_ = *T_g_*×I{pathway *m*}, where I(pathway *m*) is indicator variable for a gene belonging to pathway *m*. Therefore, for genes within pathway *m*, *Pathway_m_*_(*g*)_ varies depending on *T_g_* and it is 0 for genes outside pathway *m*.

Using matrix algebra, it can be shown that *Array* and *Pathway* random effects induce a covariance structure in the marginal model that accommodates different amount of dependencies between genes (see for example, [29], page 737). More specifically, let *y_glm_* be gene expression value for gene *g* from pathway *m* on array *l*, then 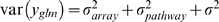 where *σ* ^2^ is residual variance associated with measurement errors and
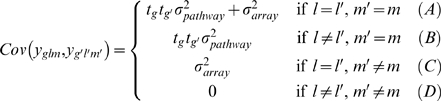
Here, *t_g_* denotes value of statistic *T* for gene *g*. In (B), for genes from the same pathway, the correlations between genes depend on directions and magnitudes of their differential expression changes. So two genes are highly positively correlated if there are large differential expression changes for both genes and their changes are in the same direction. In (C), assuming most of covariations between genes come from those genes within the same pathway and genes from different pathways but on the same array are only weakly correlated, we model a common covariance between these genes. In practice, we found assuming heterogenous covariances 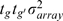 tend to be too strong for genes from different pathways and tests for gene-sets based on it lose too much power. Comparing (A) and (B), (C), genes from the same arrays and pathways are more correlated than those from different arrays or from different pathways. In (D), we assume genes from different arrays and different pathways are independent given the arrays are from independent patients.

## Results

### Simulation Study

We performed a simulation study to assess the sensitivity and specificity of a mixed model approach compared with GSEA and PAGE which also test hypothesis Q1 in Tian et al. [14], that is, the association of gene-set genes with outcome is similar with the association for the rest of the genes. For each scenario in Table 1, two sets of 50 microarray samples were simulated for treatment and control groups. Each sample consisted of 1500 values generated from the standard normal distribution as an approximation to log transformed gene expression values. These values were assigned to 50 gene-sets, each with 30 genes. Treatment effects were added to gene-set 1 according to the parameters *p*, *up*, *μ* where

*p* = Proportion of genes with treatment effect added to case group,
*up* = Among treated genes, the proportion of genes for which positive treatment effect *μ* were added,
1−*up* = Among treated genes, the proportion of genes for which negative treatment effects −*μ* were added.

**Table 1:** Area Under ROC Curve (AUC) for the comparison of Mixed Model, PAGE and GSEA methods using simulated data.

Scene	tot_p	up_p	mu	Mixed Model	GSEA	PAGE
1	0.3	0.5	0.2	0.6158	0.5468	0.5453
2	0.3	0.5	0.4	0.9346	0.6762	0.5852
3	0.3	0.5	0.6	0.9986	0.7349	0.6230
4	0.5	0.5	0.2	0.7735	0.7417	0.5452
5	0.5	0.5	0.4	0.9868	0.7321	0.5851
6	0.5	0.5	0.6	1.0000	0.7373	0.6225
7	0.8	0.5	0.2	0.9106	0.7394	0.5063
8	0.8	0.5	0.4	1.0000	0.7373	0.5064
9	0.8	0.5	0.6	1.0000	0.7373	0.5062
10	0.3	0.8	0.2	0.7074	0.6395	0.7002
11	0.3	0.8	0.4	0.8814	0.8484	0.8755
12	0.3	0.8	0.6	0.9718	0.9710	0.9683
13	0.5	0.8	0.2	0.8472	0.7173	0.8456
14	0.5	0.8	0.4	0.9872	0.9750	0.9888
15	0.5	0.8	0.6	0.9999	0.9957	1.0000
16	0.8	0.8	0.2	0.9551	0.8969	0.9572
17	0.8	0.8	0.4	1.0000	0.9956	1.0000
18	0.8	0.8	0.6	1.0000	0.9964	1.0000

tot_p = proportion of genes with treatment effect added to treatment group.

up_p = among treated genes, the proportion of genes for which positive treatment effect *mu* was added.

1-up = among treated genes, the proportion of genes for which negative treatment effect - *mu* was added.

Therefore, among all the genes in the gene-set, there were 30×*p*×*up* up-regulated genes and 30×*p*×(1−*up*) down-regulated genes. For example, for Scenario 1 in Table 1, there were 9 ( = 30×0.3) genes in gene-set 1 with treatment effect added, among them 5 (≈30×0.3×0.5) gene values were increased with 0.2 units and the remaining 4 genes were decreased with −0.2 units. In scenes 4–6 and 7–9, the total proportions of genes with treatment effects were changed to 0.5 and 0.8 respectively. In scenes 10–18, among treated genes, 80% of genes were moved up and 20% genes were moved down. These parameters were chosen to represent different degrees of feedback and reverse regulation. For each scenario, only the first gene-set was associated with treatment-control groups and the other gene-sets were null gene-sets by design of experiment.

The javaGSEA implementation was used for GSEA analysis and the algorithm described on page 10 of [15] was used for PAGE. SAS PROC MIXED [29] was used for mixed model analysis. For datasets with up_p = 0.5, GSEA algorithm was implemented with gene list sorting mode “abs”, so genes were sorted based on absolute values; the mixed model was implemented with Model 2. For each scenario with up_p = 0.5, 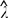 was estimated by applying Box-Cox transformation (Linear Mixed Model in Materials and Methods) to t-statistics of the 49 null gene-sets. The results showed the estimated 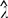 was 0.7 for all scenarios for the transformed t-statistics to achieve approximate normality.

To compare the performances of Mixed Model 1 with GSEA and PAGE, we generated 20 datasets for each set of parameters *p*, *up*, *μ* and computed the Area Under the receiver operating characteristic Curve (AUC) for each method. The receiver operating characteristic (ROC) curves show trade-off between sensitivity and 1-specificity as the significance cutoff is varied. The AUC assesses the overall discriminative ability of the methods at determining whether a given gene-set is associated with outcome over all possible cutoffs. In addition, we calculated the test sizes of each method (the proportions of p-values less than 0.05 for null gene-sets). Because under the null hypothesis we expect the p-values to follow a uniform distribution, a method with test size equal to or less than the significance cutoff (e.g. 0.05) is desirable.

**Figure 1:**
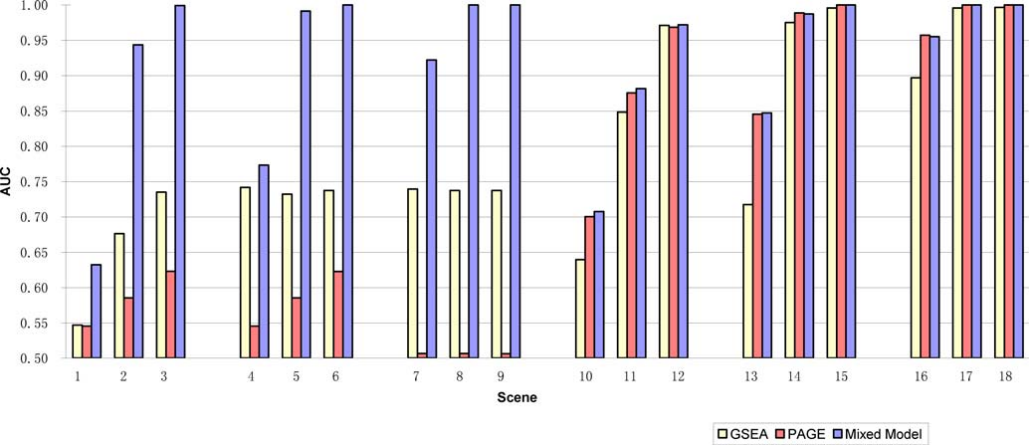
Area under ROC Curves (AUC) for the comparison of Mixed Model, PAGE and GSEA methods using simulated data. For each scene, there were 20 simulated datasets, each with 1500 genes assigned to 50 gene-sets, among them only the first gene-set (gene-set 1) include genes associated with outcome by design. The test results from each method were compared with true classification of the gene-sets. The AUC measures the ability of a test to correctly classify whether a gene-set is associated with outcome. In scenes 1–9, when genes were shifted in both directions equally (up_p = 0.5), mixed model outperformed both GSEA and PAGE. In scenes 10–18, when most of genes were shifted in one direction (up_p = 0.8), mixed model and PAGE performed similar, and they both outperformed GSEA, especially when the magnitude of differential expression in gene-set 1 is small (scenes 10, 13, 16).

In terms of AUC, when most genes are shifted in one direction (up_p = 0.8), the mixed model and PAGE performed similarly, and they both outperformed GSEA consistently across scenarios 10–18 (Table 1, Figure 1). These results show that improved power can be gained over GSEA, which tests for any differences in distributions, by using approaches such as the mixed model or PAGE that test for location changes. When genes are shifted in both directions equally (up_p = 0.5), the mixed model performed better than both GSEA and PAGE. The better performance of the mixed model vs. PAGE shows that combining signals for up-regulation and down-regulation by Mixed Model 2 is more effective in this setting because signals from genes shifted in different directions may be cancelled out. We note also that all methods maintained proper test sizes for all scenarios.

### Reanalysis of Diabetes Study Data

Mootha et al. [12] compared gene expression of skeletal muscle biopsy samples from human diabetes patients and patients with normal glucose tolerance. There were 17 control patients (group NGT) and 18 diabetic patients (group DM2) in this study and 149 curated gene-sets were tested for enrichment using GSEA. They found that genes involved in oxidative phosphorylation were coordinately down regulated in human diabetes. To compare the results of the mixed model approach with GSEA and to confirm that mixed models can also detect subtle but coordinated changes in gene expression within gene-sets, we reanalyzed this data set.

Table 2 tabulates analysis results for gene-sets selected by mixed models and the GSEA method. The results for GSEA were obtained from http://www-genome.wi.mit.edu/mpg/oxphos/. For the mixed model method, the nominal p-value were estimated by fitting Model 1 and testing the interaction term Type×Pathway. Because the Pathway random effects were also included in Model 1, they induce a more general covariance structure between genes, so mixed model analysis accounts for heterogeneous variances of different pathways and gene-gene correlations. False discovery rate (FDR) adjusted p-values were also calculated, an adjusted p-value of 0.05 for a pathway indicates that among all significant pathways selected at this threshold, 5 out 100 of them are expected to be false leads.

**Table 2:** Comparison of Mixed Model and GSEA Results for the Analysis of Diabetes Dataset from Mootha et al. (2003).

Pathway		Nominal p-values		FDR Adj. p-value
	Size	GSEA	Mixed Model	Mixed Model
OXPHOS_HG_U133A_probes	114	0.003	1.40E-12	2.11E-10
c18_U133_probes	248	0.932	4.43E-07	3.34E-05
human_mitoDB_6_2002_HG_U133A_probes	594	0.091	6.97E-06	3.51E-04
mitochondr_HG_U133A_probes	615	0.087	2.03E-05	7.68E-04
c25_U133_probes	64	0.246	9.07E-04	0.027
MAP00350_Tyrosine_metabolism	47	0.965	0.00110	0.028
c19_U133_probes	203	0.778	0.00253	0.048
MAP00010_Glycolysis_Gluconeogenesis	91	0.759	0.00255	0.048
MAP00500_Starch_and_sucrose_metabolism	30	1	0.00294	0.049

Size refers to the number of genes in the gene-set. Both mixed model and GSEA selected the pathway “OXPHOS_HG_U133A_probes” as the most significantly changed pathway and ranked the pathways “human_mitoDB_6_2002_HG_U133A_pro”, “mitochondr_HG_U133A_probes” high on their significant pathways list. Mixed model selected 6 additional gene-sets at 5% FDR level.

The results show that both the mixed model and GSEA selected the pathway “OXPHOS_HG_U133A_probes” as the most significantly changed pathway and ranked the pathways “human_mitoDB_6_2002_HG_U133A_pro”, “mitochondr_HG_U133A_probes” high on their significant pathways list. While mixed model selected 9 gene-sets at 5% FDR level, all FDR adjusted p-values for GSEA method were greater than 0.2 (the minimum was 0.447). As diabetes is primarily a chronic disorder of carbohydrate metabolism, additional pathways identified by the mixed model, such as the “Glycolysis/Gluconeogenesis” and “Starch and sucrose metabolism” make biological sense. Chronic diabetes has also been associated with changes in “Tyrosine metabolism” [30], another pathway identified by the mixed model.

### A Dose Response Study

We next applied the mixed model method to a dose-response microarray experiment. West et al. [26] conducted experiments to study the effect of HNE (4-hydroxy-2-nonenal) on RKO human colorectal carcinoma cells. It is postulated that HNE induces cellular dysfunction in a variety of disorders such as inflammation, cancer, neurodegenerative, cardiovascular disease [31],[32]. In this study, Affymetrix U133 Plus 2.0 chips were used with RKO cells to explore transcriptional changes induced following treatment for 6 or 24 hours with 5,20, or 60 *µ*M HNE. Figure S1 shows the dose response relationships averaged over all genes for each gene set for each treatment duration.

Our main objective was to identify gene sets with significant monotone changes over doses and to assess whether the changes were similar for the two treatment durations. With permutation based methods such as GSEA, one needs to decide *a priori* whether to test for trends of gene expression over different doses of HNE for each treatment duration separately or to test for trends by pooling data from both treatment durations. In contrast, the mixed model framework provides a more efficient way to incorporate information from both treatment durations, and standard methods apply for testing polynomial trends of gene expressions over different doses of HNE and for testing trend by treatment duration interaction.

We next describe the analysis workflow. First, probe sets were mapped to Ensembl Gene IDs and median expression levels for multiple probe sets corresponding to the same gene were calculated. After this step, we were left with 17278 genes and they were tested for enrichment against gene sets generated based on the biological process categories in Gene Ontology. Genes in the human genome were mapped to GO categories according to Ensembl annotation (http://www.ensembl.org). We focused on GO categories with 10 to 200 genes by removing all the other categories. Note that this is the size of a gene set when all of the genes in the genome are considered. For genes on a specific array, the gene counts for a gene set will be slightly smaller. In order to reduce the redundancy in GO, we further removed all child-categories if corresponding parent-category was within the size limitation. After the above processes, 444 remaining gene sets were used for the enrichment analysis.

Next, we calculated means and standard deviations for each gene at dose 0 for each treatment duration separately and then used these values to standardize all gene expression values. That is, the values for each gene were standardized by subtracting the dose 0 means and dividing by dose 0 standard deviations. The standardized gene expression values then represented the number of standard deviation away from the “normal” gene expression at dose 0.

Finally, we applied the mixed model with fixed effects Dose, Treatment Duration, Dose×Treatment Duration to the gene expression values. Because the data were collected at different times, the variable Batch was also added to adjust for the effects of different batches. In addition, a random Array effect was included in the model to account for correlations of genes from the same array and to facilitate inference to an entire population of arrays, not only to those considered in this study. Contrasts of parameters from this model based orthogonal polynomial coefficients were then used to test for linear trend of expression values over doses and Duration×Linear trend effect. The orthogonal polynomial coefficients are linear transformations of the natural polynomial scores and they alleviate collinearity problems of natural polynomial scores. Adjusted p-values were then computed using the R *multtest* package [33] to control for False Discovery Rate (FDR) using the method of Benjamini and Hochberg [34].

**Figure 2:**
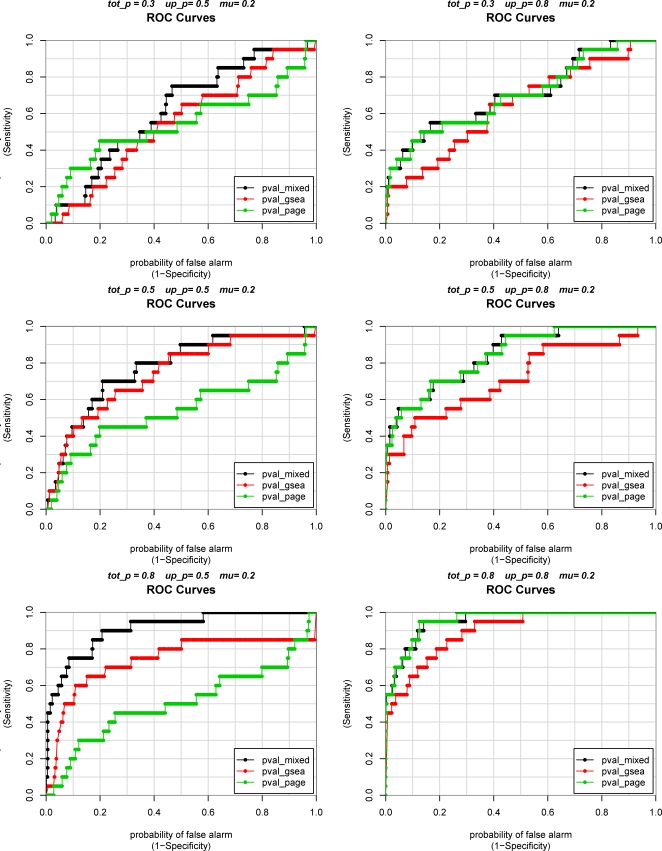
Receiver Operating Characteristic Curves for Mixed Model, GSEA and PAGE using simulated data. tot_p = proportion of genes with treatment effect added to treatment group in gene–set 1; up_p = among treated genes, the proportion of genes for which positive treatment effect mu was added; 1−up = among treated genes, the proportion of genes for which negative treatment effect –mu was added. See text for details of simulation experiment.

**Figure 3:**
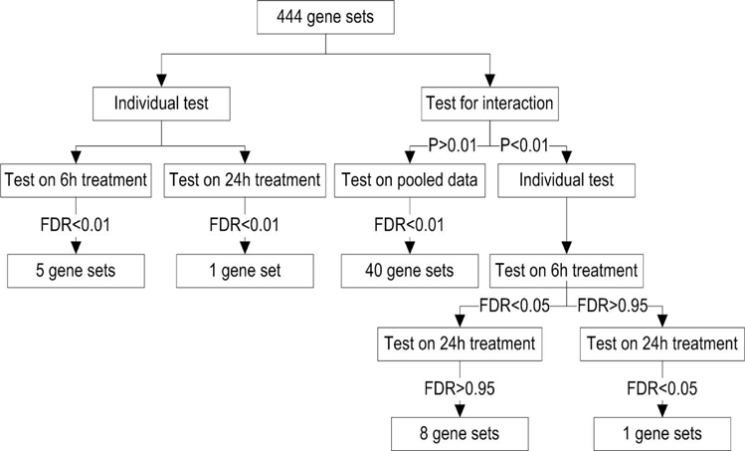
Workflow and results for Mixed Model analysis of HNE dataset. When individual tests were conducted with 6 hr treatment samples and 24 hr samples separately, only 5 and 1 gene-sets were significant at 0.01 FDR level. However, when all samples were used, for testing gene-sets with non-significant Duration×Linear Trend interaction, 40 gene-sets were significant at 0.01 FDR level. This shows pooling data with similar trends from both treatment durations improves the statistical power for identifying biologically meaningful gene sets.

Because we were mainly interested in gene sets directly responding to changes in HNE, our analysis focused on gene sets with significant linear trends of expression values corresponding to monotone changes over doses. At the adjusted p-value level of 0.01, we identified 5 and 1 responsive gene sets for 6 h and 24 h treatment, respectively (Figure 2). However, after testing for a Duration×Linear Trend interaction, and refitting gene sets for which the interaction was nonsignificant, we identified 40 responsive gene sets at the adjusted p-value level of 0.01 (Figure 3). Among them, 36 out of the 40 gene sets were not identified in the individual test. These 36 gene sets represented some important biological processes that are known to be responsive to HNE treatment, such as “mismatch repair”, “double-strand break repair”, and “response to inorganic substance” (Table S1). These results demonstrated that pooling data with similar trends from both treatment durations is helpful for improving statistical power and identifying biologically meaningful gene sets.

On the other hand, the interaction tests were also used to select gene sets showing different response trends for the 6 h and 24 h treatments. Among the 12 gene sets with significant interactions (p-value<0.01), 8 of them were responsive for 6 h treatment (adjusted p-value<0.05) but not for 24 h treatment (adjusted p-value>0.95, see Figure 3). These gene sets represented biological processes that responded to HNE in a quick manner, including “cytoplasmic sequestering of protein”, “negative regulation of transcription factor import”, and “cellular response to stimulus” etc. (Table S1). Down-regulation of the biological processes “cytoplasmic sequestering of protein” and “negative regulation of transcription factor import” at 6 h will lead to the release of transcription factors that are sequestered in the cytosol, which is consistent with the significant increase in overall transcription after 6 h of HNE treatment. One gene set, “pyrimidine deoxyribonucleotide metabolism”, showed a significant response for the 24 h treatment (adjusted p-value = 0) but not for 6 h treatment (adjusted p-value = 0.33). These results indicated that although both signaling and metabolic changes were involved in oxidative stress, metabolism response was slower than the signaling response, e.g. transcription factor import.

## Discussion

In this paper, we have proposed linear mixed models for the analysis of microarray data at the pathway-level. This flexible, unified and practical approach can be easily implemented in common statistical software packages. The proposed model makes three main improvements over popular methods for gene-set testing: improved power through testing location shift of gene-set genes, more refined modeling of covariance structure between genes through specification of random effects, and the ability to account for complicated experimental designs through inclusion of design factors and covariate effects.

As suggested by Tian et al. [14], power is lost when GSEA tests Q1 (genes in a gene-set show the same pattern of associations with the phenotype compared with the rest of the genes) but generates the null distribution of test statistic under hypothesis Q2 (all genes in gene-set are not associated with outcome) by permuting samples. In addition, the alternative hypothesis that is of interest for Q1 is more likely to be location shift for genes in the gene-set compared to background genes; the use of an omnibus test such as the Kolmogorov test by GSEA may result in further loss in power and produce false positives for tightly correlated gene-sets. Our proposed method provides a simple way to test for location shifts in Q1 while accounting for covariance structure between genes at the same time. It provides increased power while still maintaining control of the false positive rate.

The use of random effects to account for a general covariance structure that varies according to genes in the proposed models represent our efforts for improving covariance structure modeling of current parametric methods. False positives are likely to result when dependency between genes are not accounted for [15], or through the use of homogenous correlation between all genes on the same array [23]. Our proposed model, although may not be perfect, provides a way to capture the primary heterogeneous covariance structure between genes. As genes operate with complex covariation patterns, covariance structure modeling is a challenge for parametric methods and future study with further refined modeling of dependencies between genes will extend the power and potential of mixed models and other parametric methods.

On the other hand, the strength of parametric methods such as the proposed mixed models lie in their ability to account for complicated design information. When there are multiple sources of covariation in the data, permutation or resampling methods are often difficult to employ. In contrast, mixed Models 1 and 2 can be easily extended to handle a variety of more complex designs. For example, for two-color arrays and other arrays with multiple measurements per gene on each array, Model 1 can be augmented with additional random effects corresponding to spot or block effects. When arrays are processed in multiple batches, a batch effect can be added to the model to adjust for systematic effects from different batches. Similarly, other random effects from blocks and sites where the experiments were performed can also be incorporated into the models. In the A Dose Response Study section, although we have analyzed a dose response study, time-course experiments can also be analyzed in a similar way. For example, for a time-course study with two treatments and four time points, a mixed model with fixed effects Treatment, Time and Treatment×Time plus random effects can be constructed. In addition, these models can be further extended to accommodate design information such as matched case-control pairs. Littell et al. [29] provides a comprehensive set of examples covering a wide range of mixed models and related covariance structures. Tests for multiple interaction effects in these and numerous other mixed model settings can provide valuable sentinels for scientific discovery.

## Supporting Information

Figure S1 Average standardized gene expression values for each dose and each treatment duration.
(0.77 MB PDF)

Table S1 Supplementary table for HNE data.
(0.24 MB XLS)
