# Supplementary material for: An Integrated Approach for the Analysis of Biological Pathways using Mixed Models
Source: PLoS Genet. 2008 Jul 4;4(7):e1000115. doi: 10.1371/journal.pgen.1000115 (PMC2565842; doi:10.1371/journal.pgen.1000115)

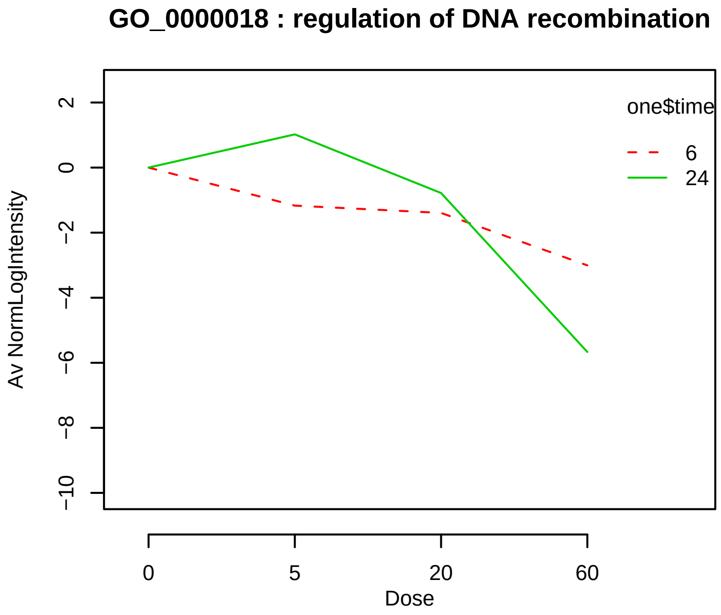
GO_0000018 : regulation of DNA recombination
Av NormLogIntensity
Dose
one$time
6
24

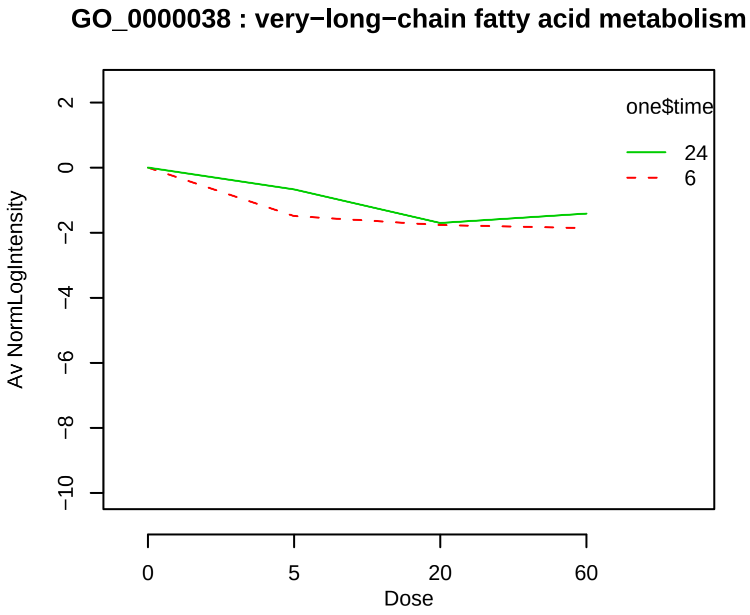
GO_0000038 : very−long−chain fatty acid metabolism
Av NormLogIntensity
Dose
one$time
24
6

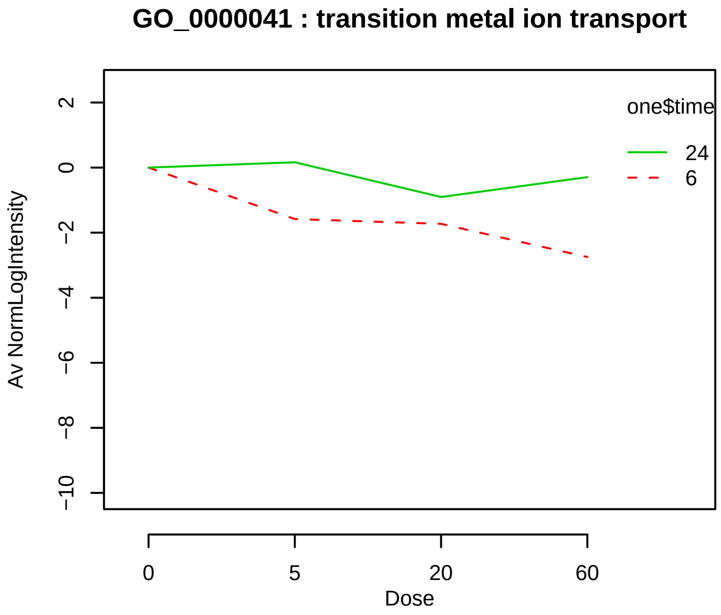
GO_0000041 : transition metal ion transport
Av NormLogIntensity
Dose
one$time
24
6

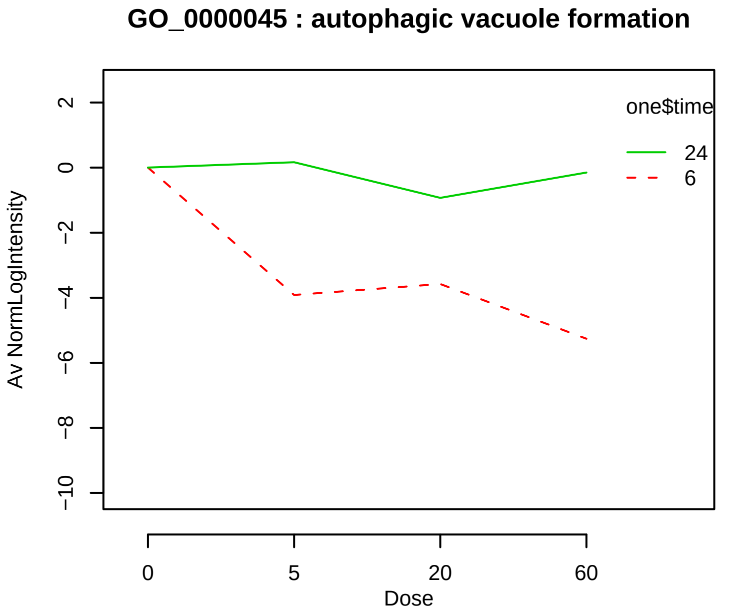
GO_0000045 : autophagic vacuole formation
Av NormLogIntensity
Dose
one$time
24
6

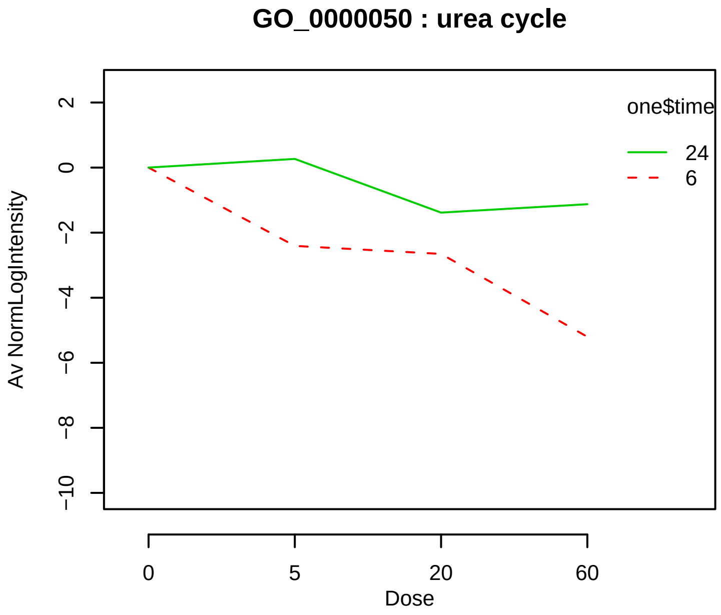
GO_0000050 : urea cycle
Av NormLogIntensity
Dose
one$time
24
6

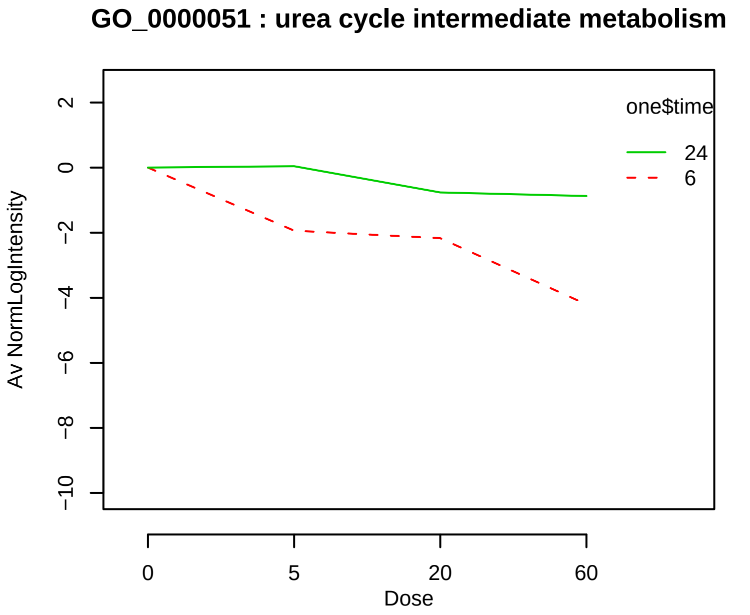
GO_0000051 : urea cycle intermediate metabolism
Av NormLogIntensity
Dose
one$time
24
6

GO_0000059 : protein import into nucleus\, docking

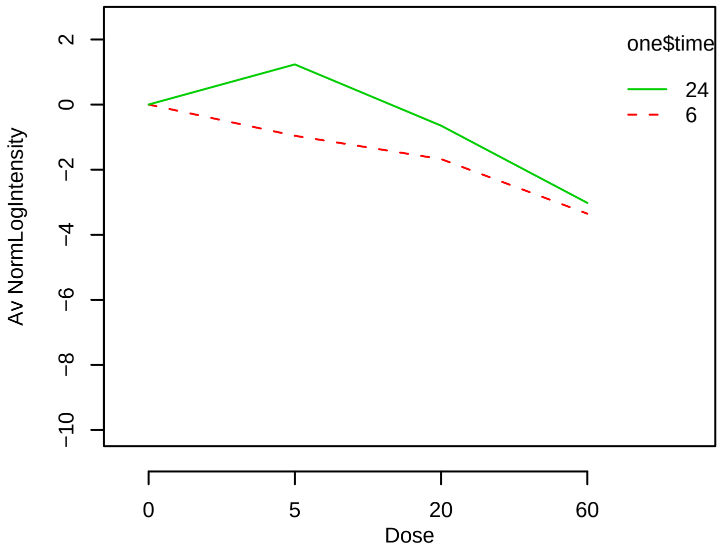

GO_0000060 : protein import into nucleus\, translocation

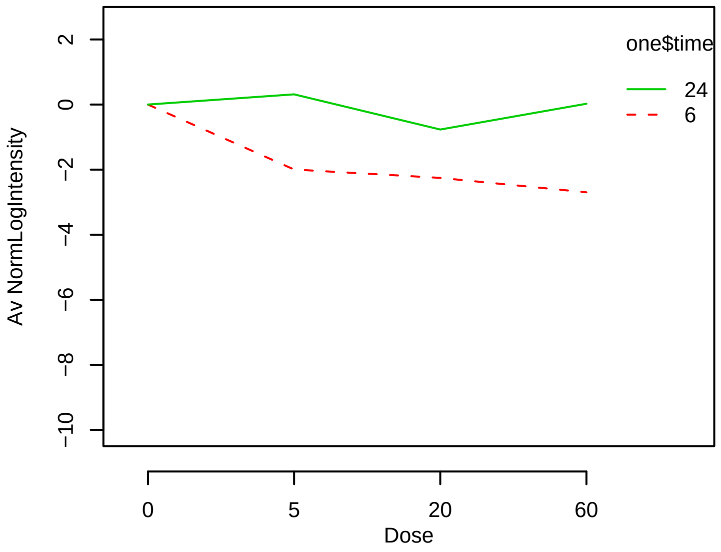

GO_0000070 : mitotic sister chromatid segregation

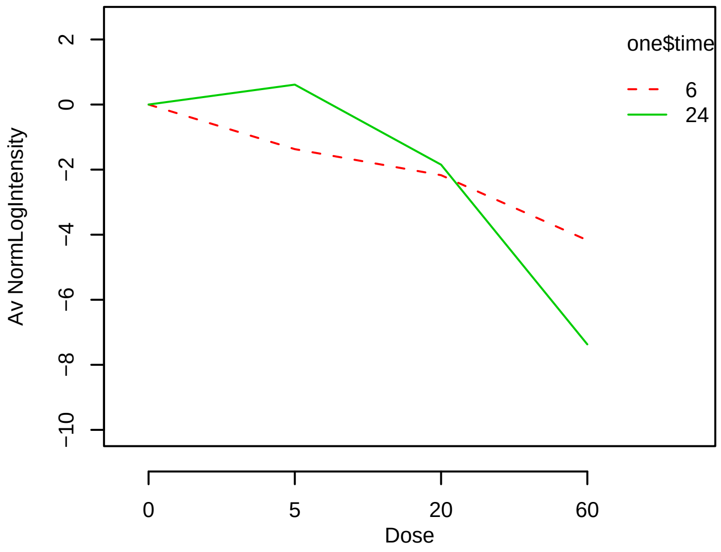

GO_0000075 : cell cycle checkpoint

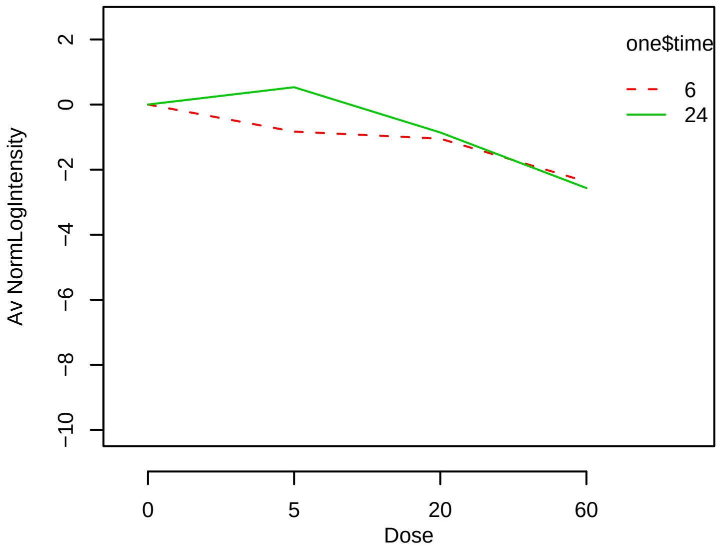

GO_0000076 : DNA replication checkpoint

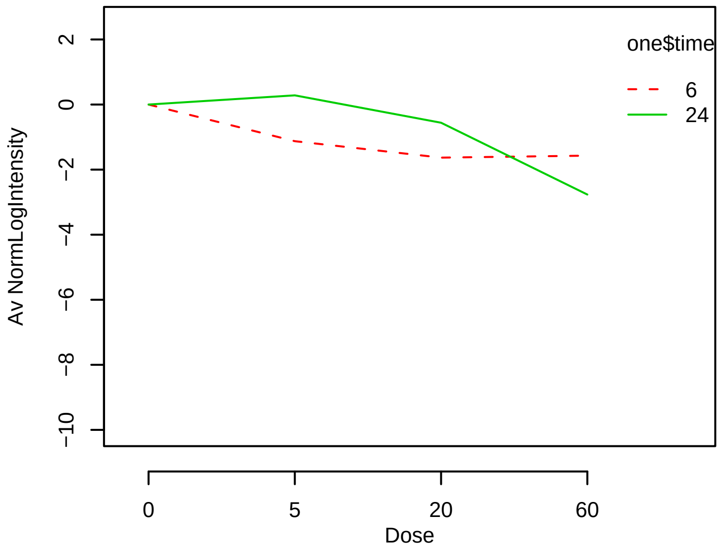

GO_0000077 : DNA damage checkpoint

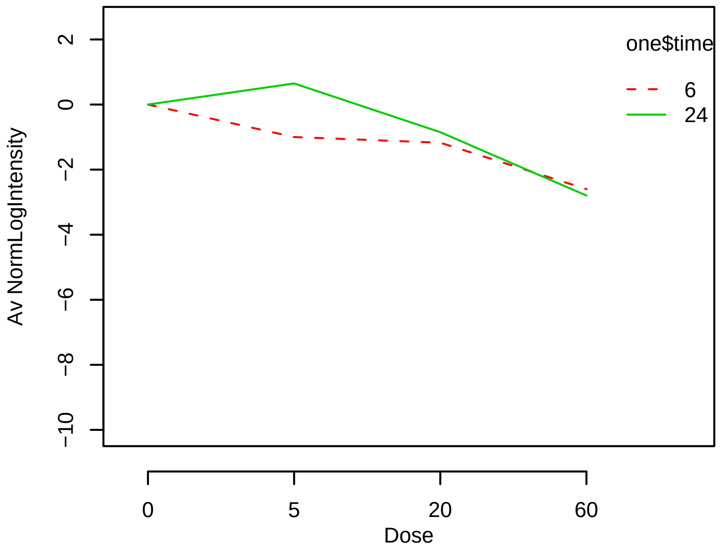

GO_0000079 : regulation of cyclin−dependent protein kinase

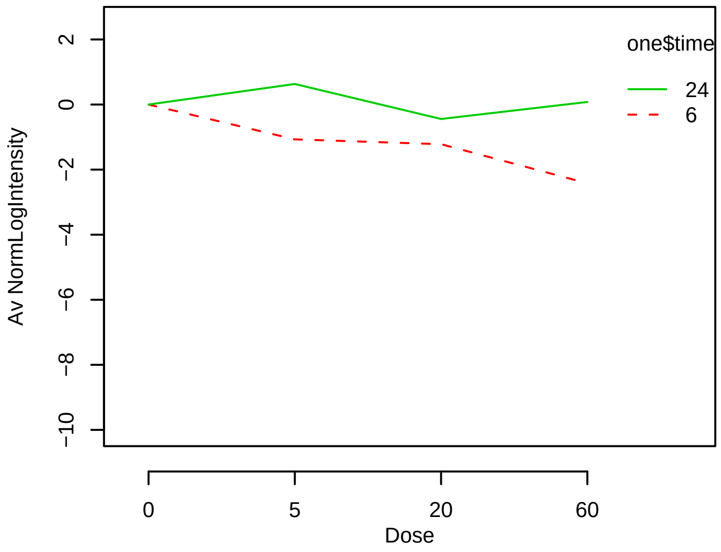

GO_0000080 : G1 phase of mitotic cell cycle

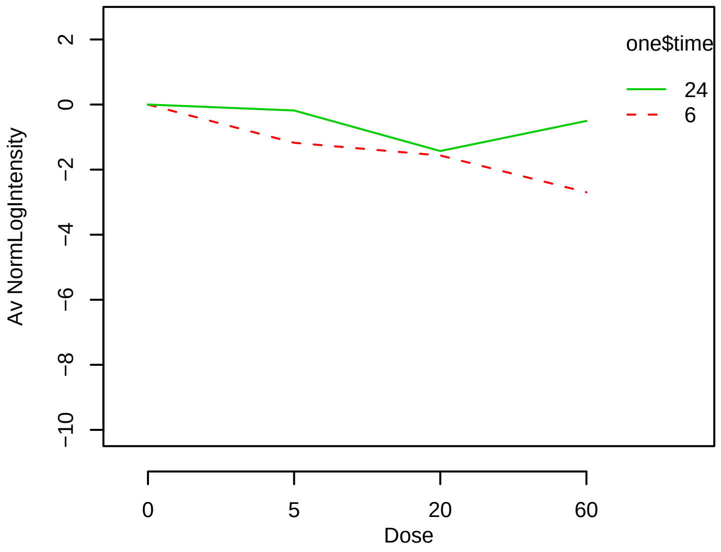

GO_0000082 : G1/S transition of mitotic cell cycle

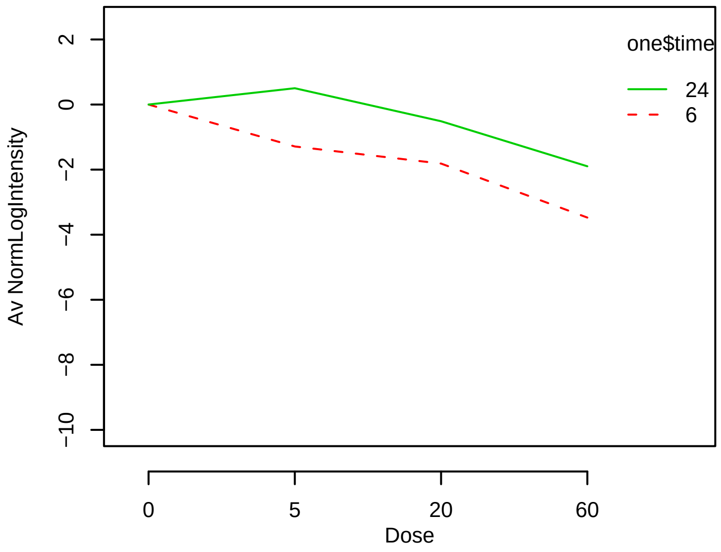

GO_0000084 : S phase of mitotic cell cycle

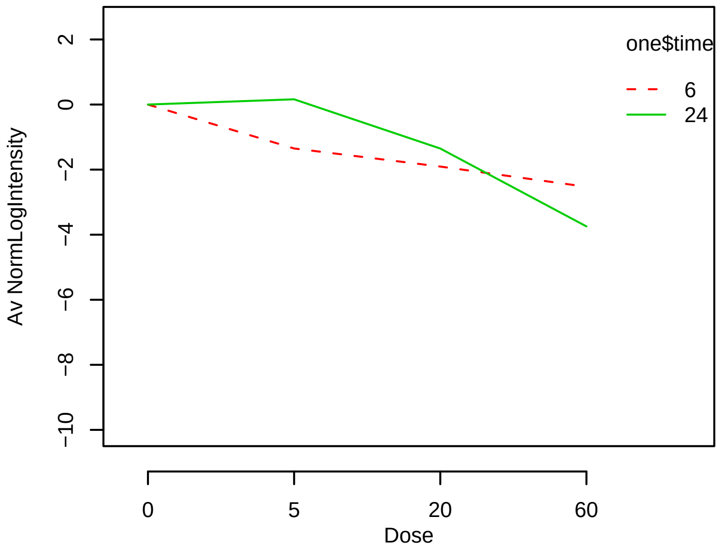

GO_0000085 : G2 phase of mitotic cell cycle

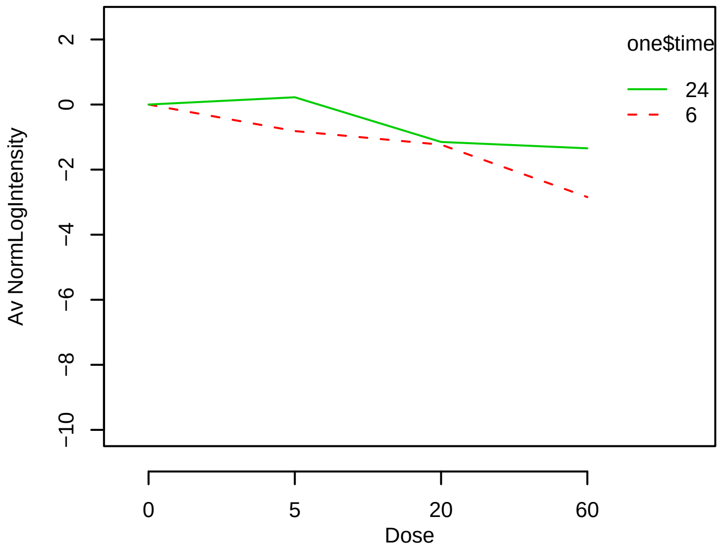

GO_0000086 : G2/M transition of mitotic cell cycle

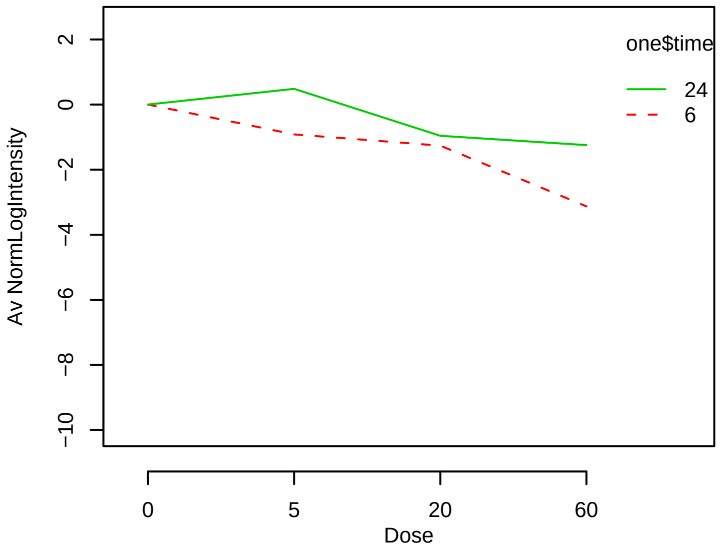

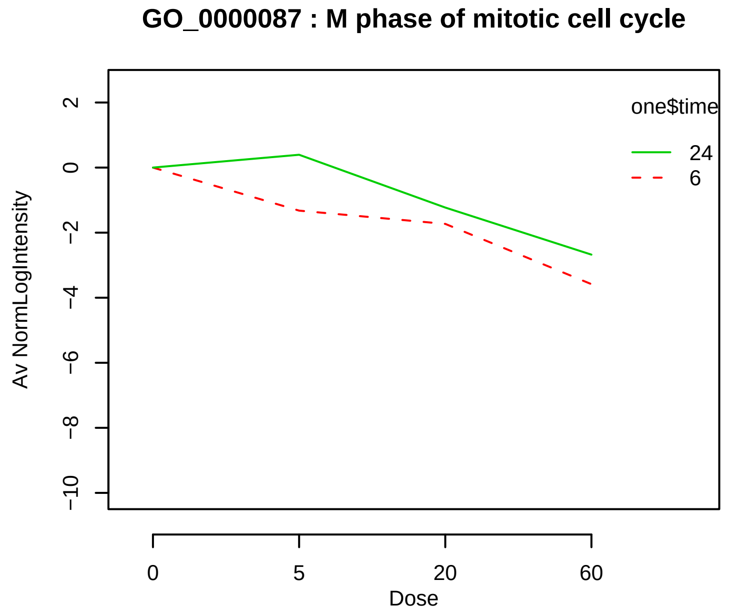
GO_0000087 : M phase of mitotic cell cycle
Av NormLogIntensity
2
0
−2
−4
−6
−8
−10
one$time
24
6
0
5
20
60
Dose

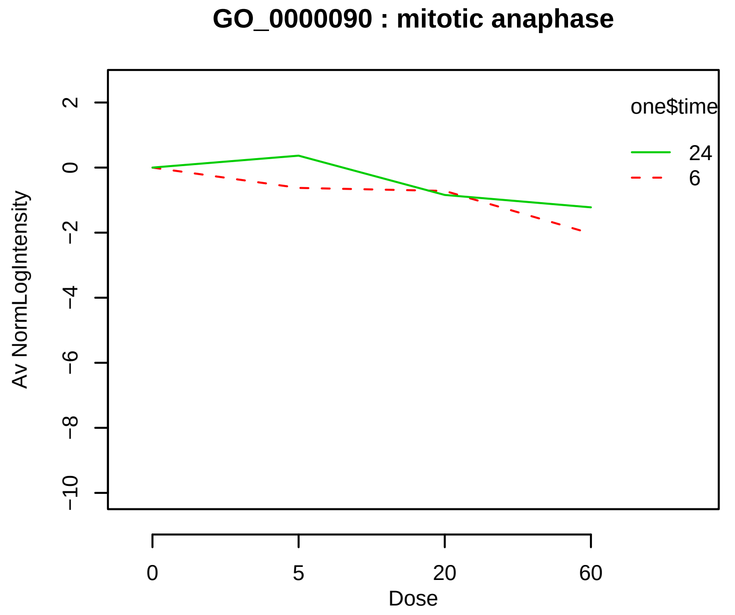
GO_0000090 : mitotic anaphase
Av NormLogIntensity
2
0
−2
−4
−6
−8
−10
one$time
24
6
0
5
20
60
Dose

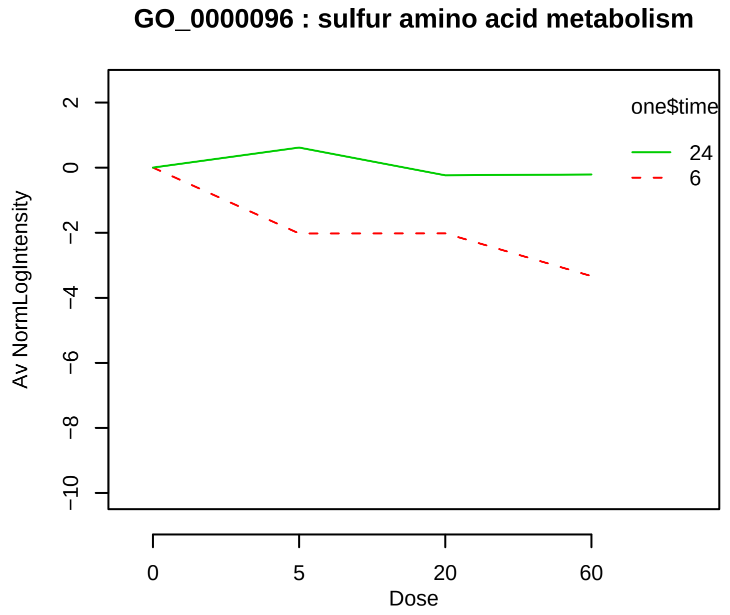
GO_0000096 : sulfur amino acid metabolism
Av NormLogIntensity
2
0
−2
−4
−6
−8
−10
one$time
24
6
0
5
20
60
Dose

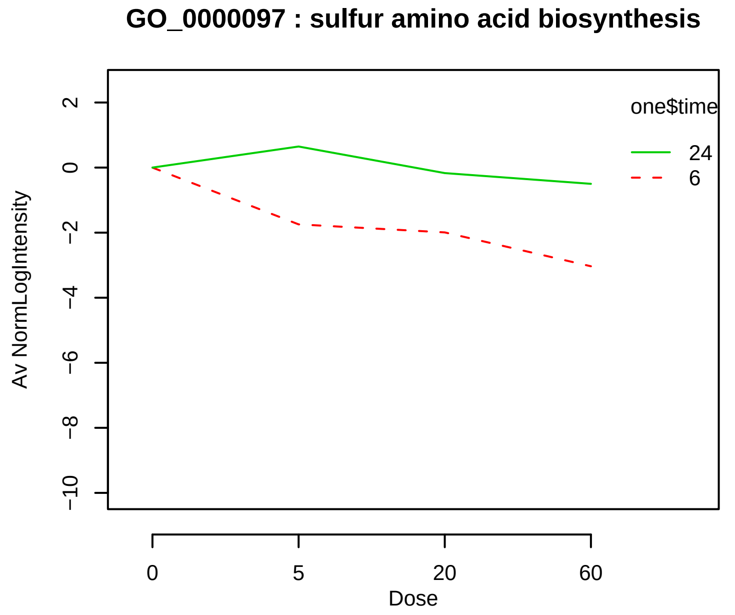
GO_0000097 : sulfur amino acid biosynthesis
Av NormLogIntensity
2
0
−2
−4
−6
−8
−10
one$time
24
6
0
5
20
60
Dose

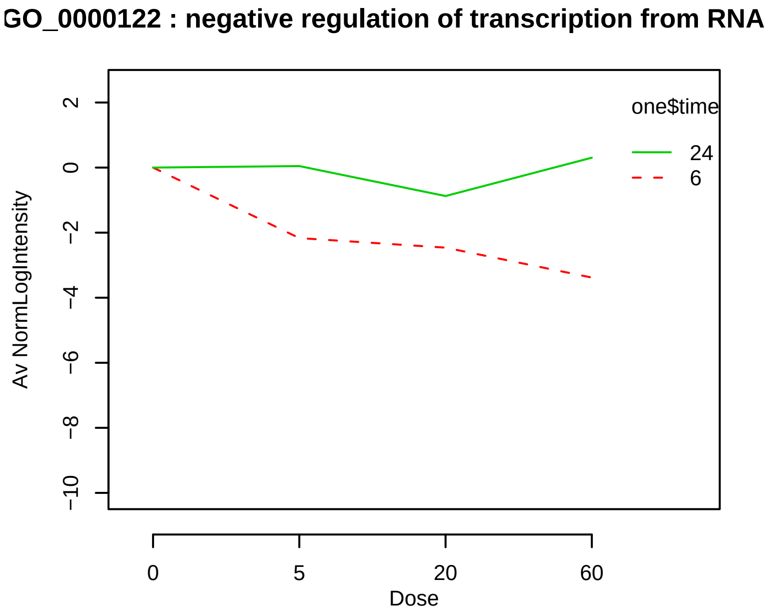
GO_0000122 : negative regulation of transcription from RNA
Av NormLogIntensity
2
0
−2
−4
−6
−8
−10
one$time
24
6
0
5
20
60
Dose

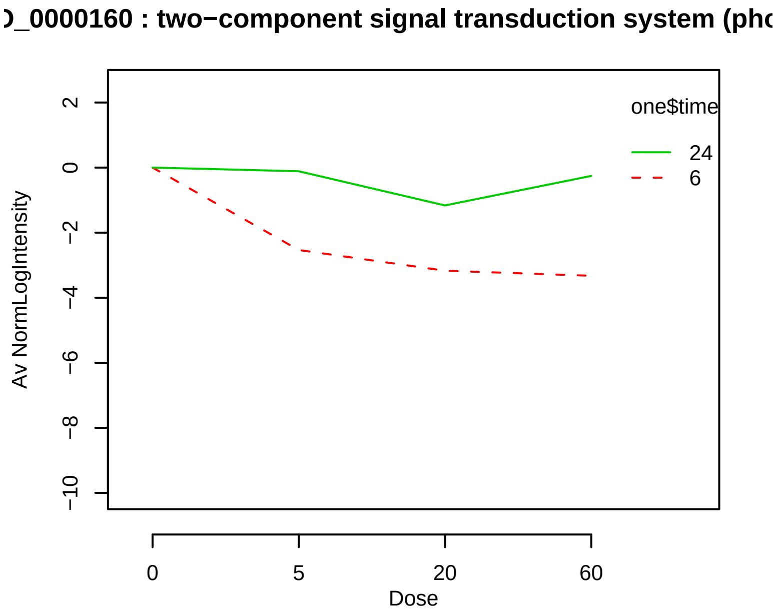
)_0000160 : two−component signal transduction system (pho
Av NormLogIntensity
2
0
−2
−4
−6
−8
−10
one$time
24
6
0
5
20
60
Dose

GO_0000165 : MAPKKK cascade

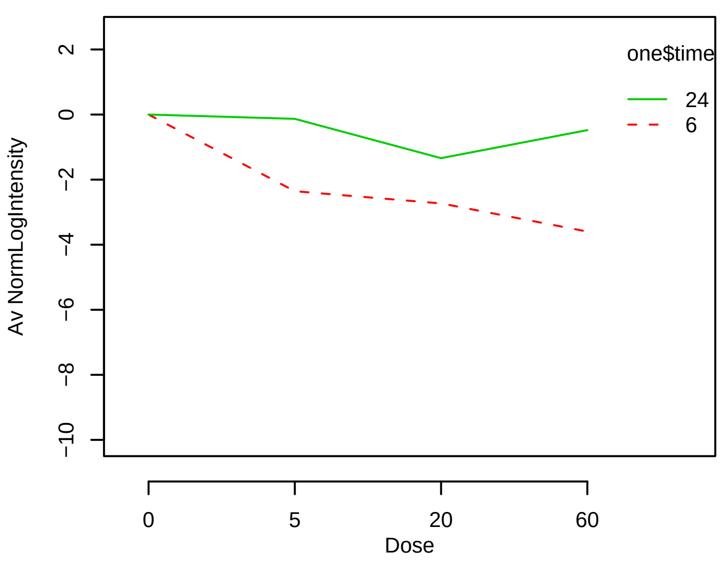

GO_0000184 : mRNA catabolism\, nonsense−mediated dec

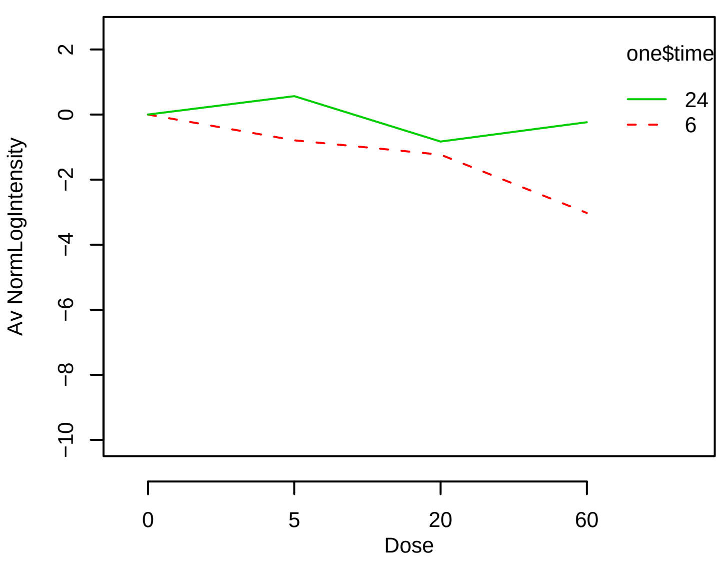

GO_0000185 : activation of MAPKKK activity

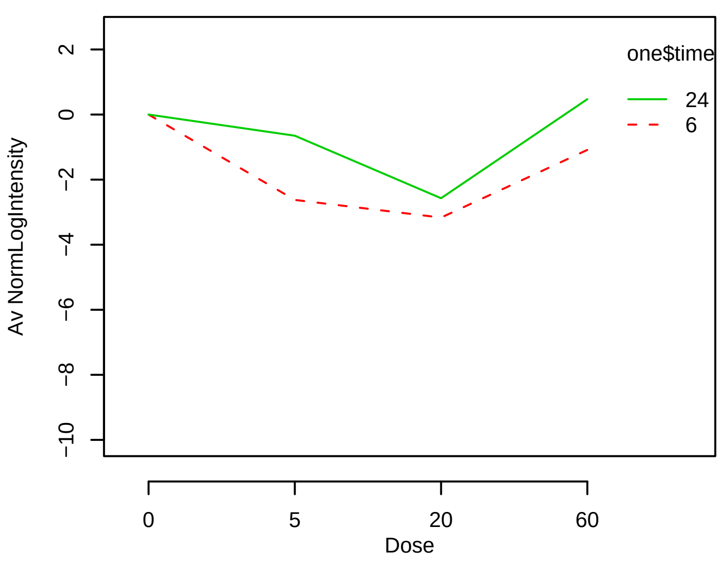

GO_0000186 : activation of MAPKK activity

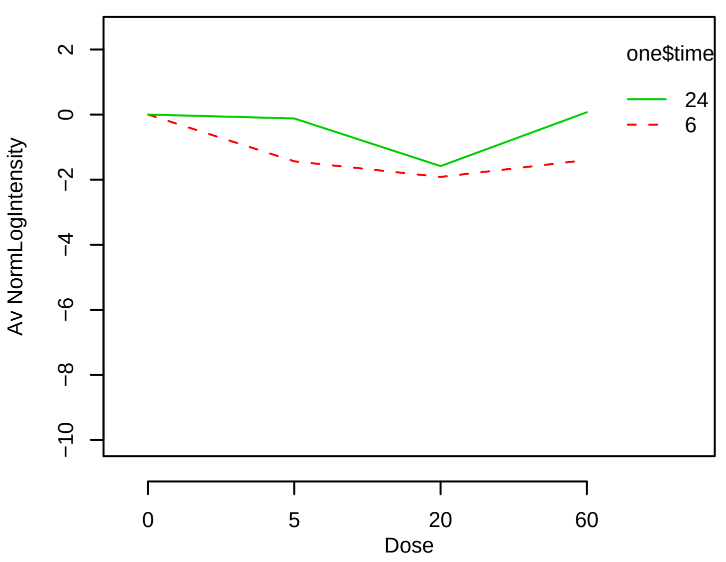

GO_0000187 : activation of MAPK activity

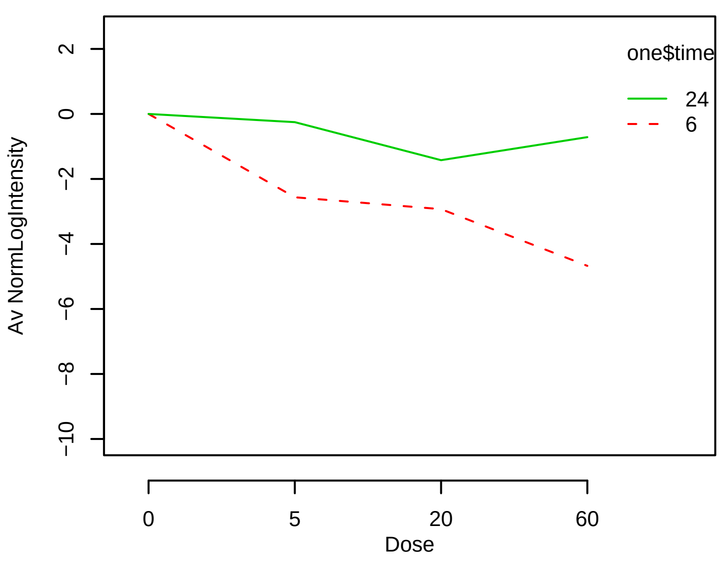

GO_0000188 : inactivation of MAPK activity

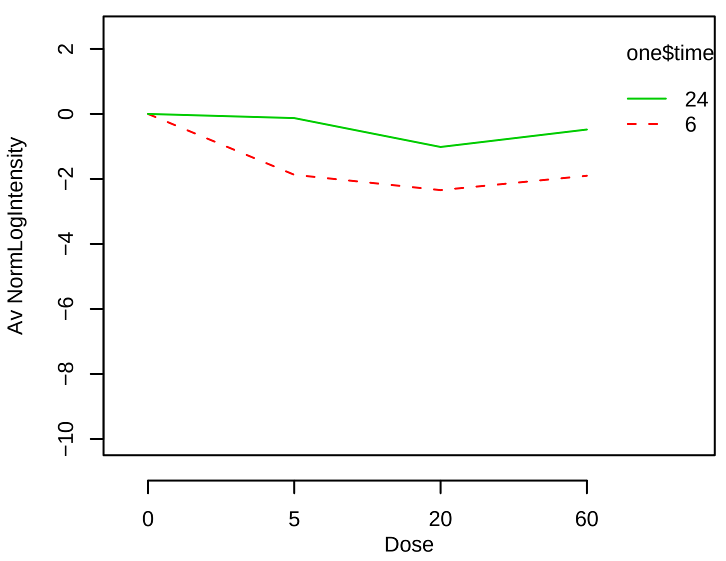

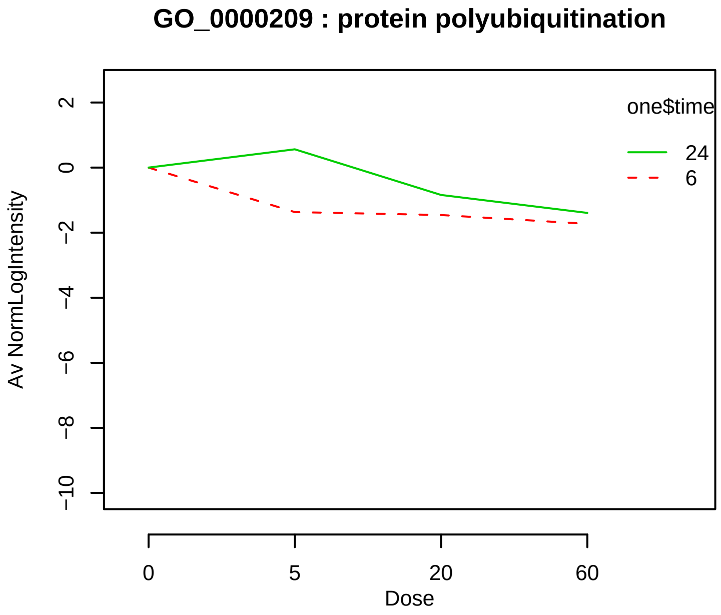
GO_0000209 : protein polyubiquitination
one$time
24
6
Av NormLogIntensity
Dose
0
5
20
60

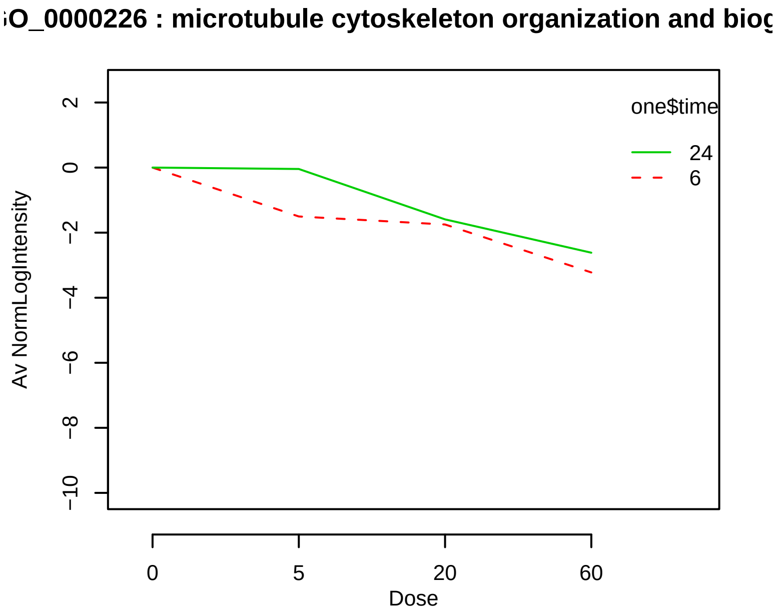
GO_0000226 : microtubule cytoskeleton organization and biog
one$time
24
6
Av NormLogIntensity
Dose

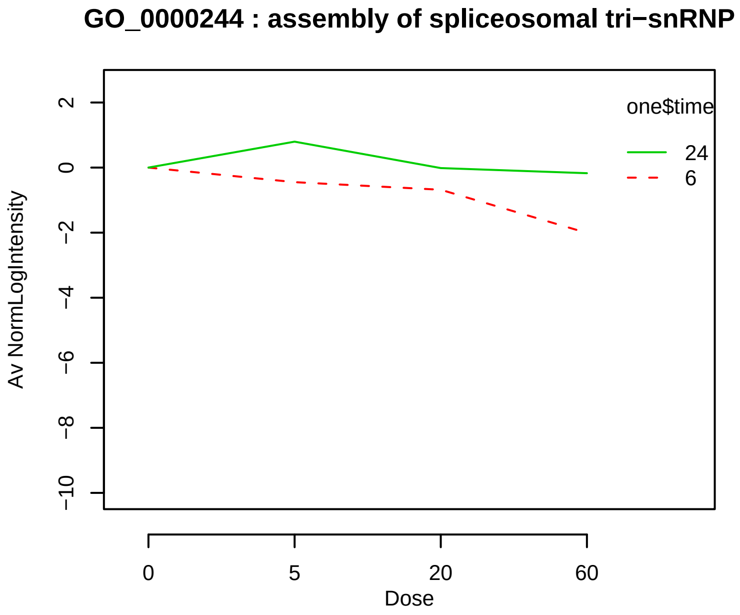
GO_0000244 : assembly of spliceosomal tri−snRNP
one$time
24
6
Av NormLogIntensity
Dose

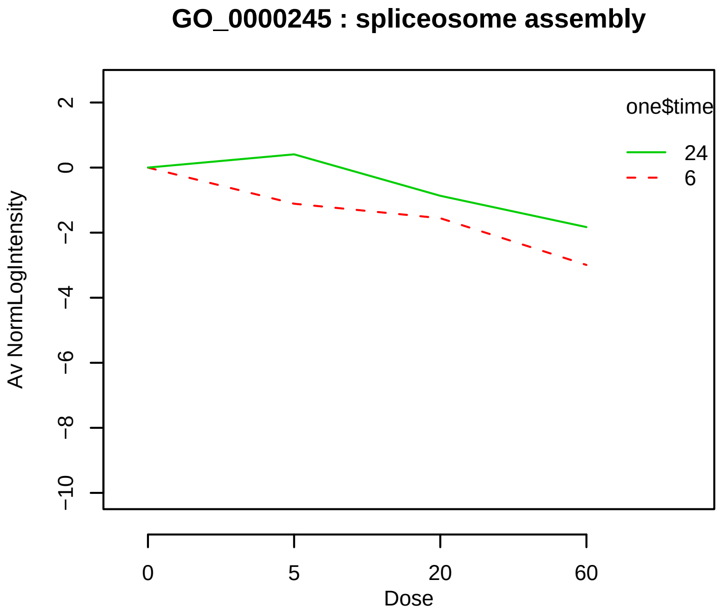
GO_0000245 : spliceosome assembly
one$time
24
6
Av NormLogIntensity
Dose

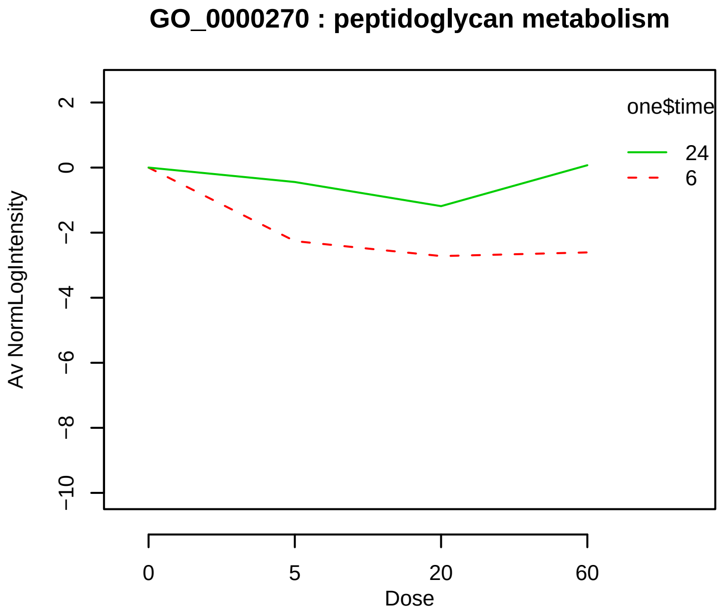
GO_0000270 : peptidoglycan metabolism
one$time
24
6
Av NormLogIntensity
Dose

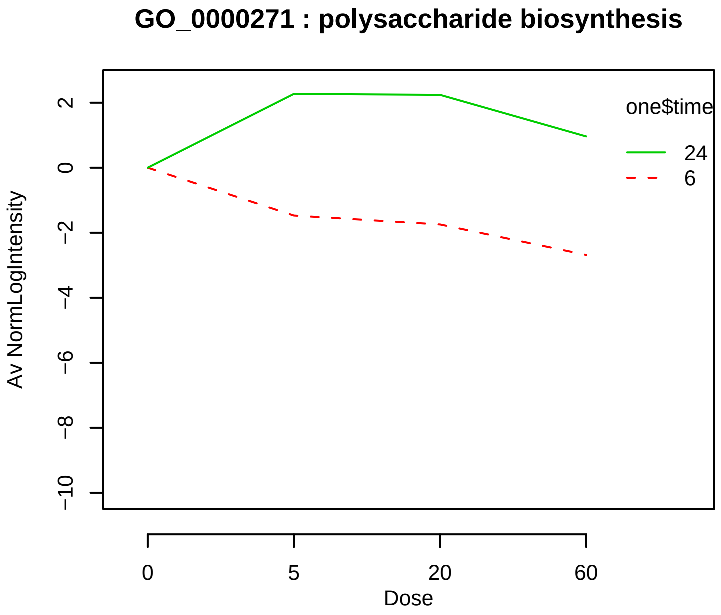
GO_0000271 : polysaccharide biosynthesis
one$time
24
6
Av NormLogIntensity
Dose

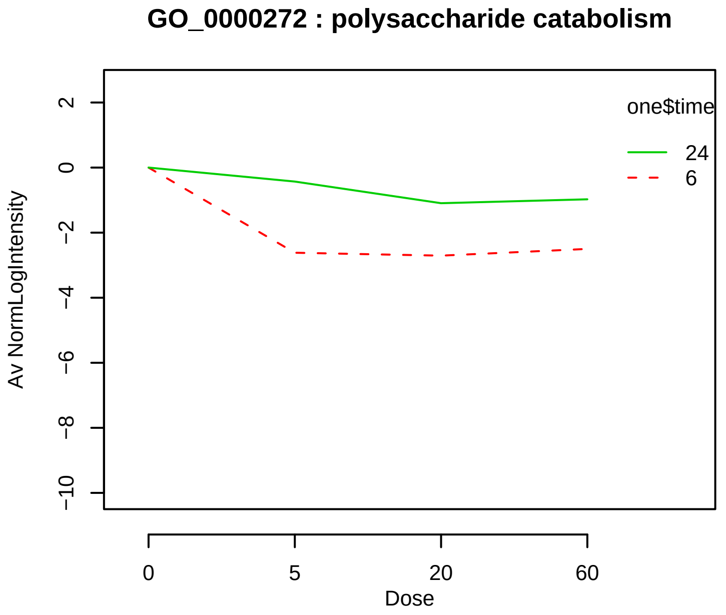
GO_0000272 : polysaccharide catabolism
Av NormLogIntensity
2
0
−2
−4
−6
−8
−10
one$time
24
6
0
5
20
60
Dose

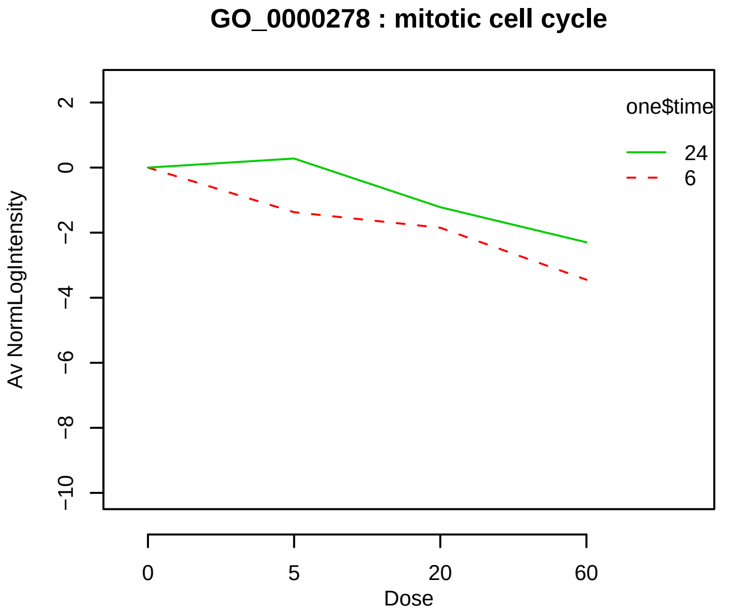
GO_0000278 : mitotic cell cycle
Av NormLogIntensity
2
0
−2
−4
−6
−8
−10
one$time
24
6
0
5
20
60
Dose

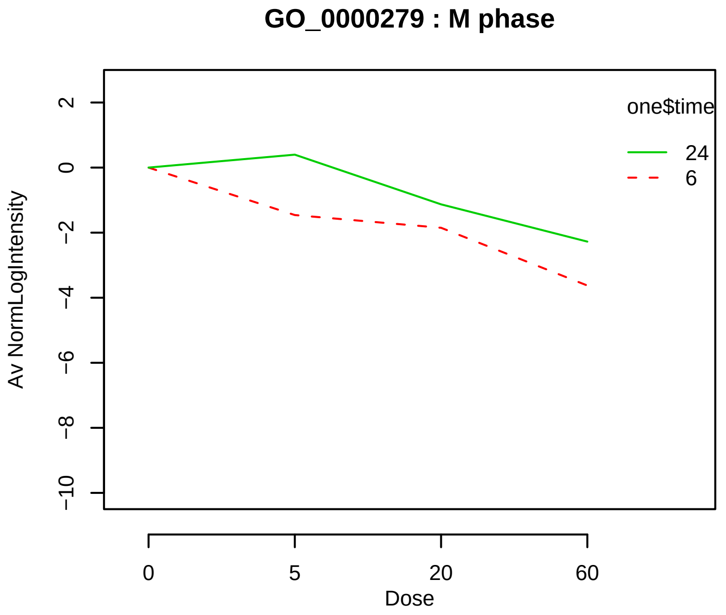
GO_0000279 : M phase
Av NormLogIntensity
2
0
−2
−4
−6
−8
−10
one$time
24
6
0
5
20
60
Dose

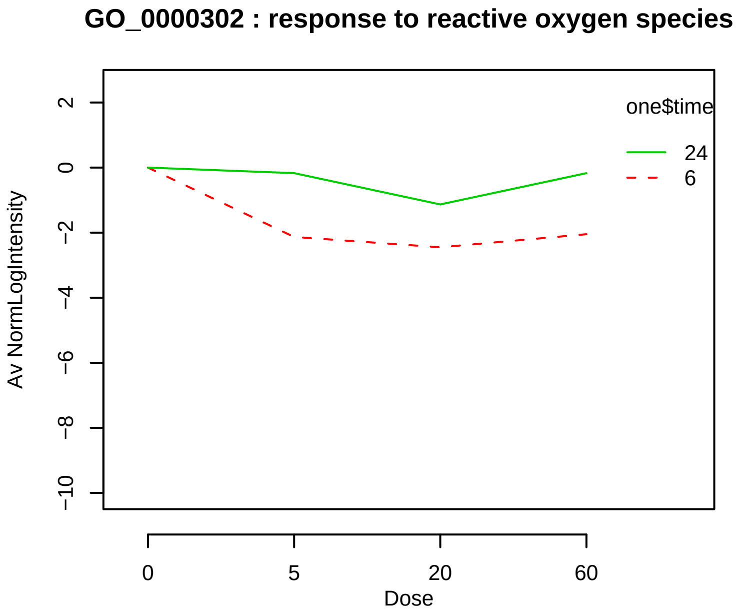
GO_0000302 : response to reactive oxygen species
Av NormLogIntensity
2
0
−2
−4
−6
−8
−10
one$time
24
6
0
5
20
60
Dose

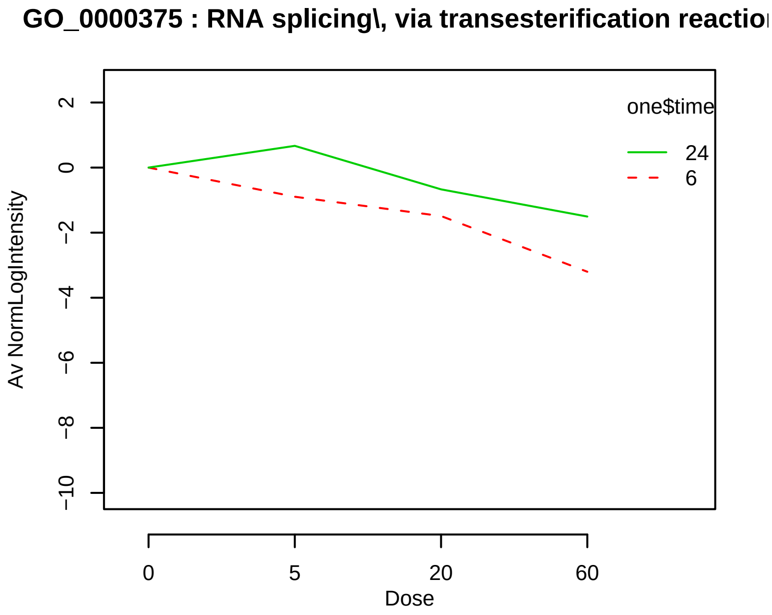
GO_0000375 : RNA splicing\, via transesterification reactio
Av NormLogIntensity
2
0
−2
−4
−6
−8
−10
one$time
24
6
0
5
20
60
Dose

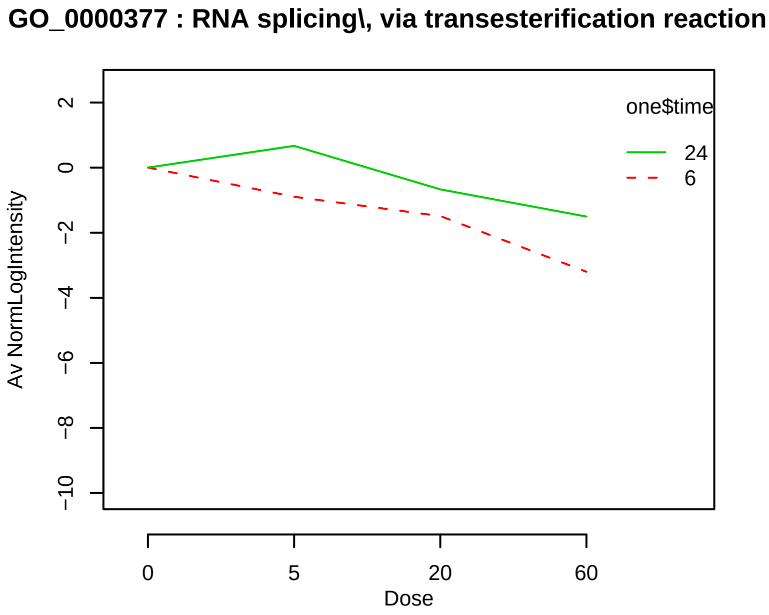
GO_0000377 : RNA splicing\, via transesterification reaction
Av NormLogIntensity
2
0
−2
−4
−6
−8
−10
one$time
24
6
0
5
20
60
Dose

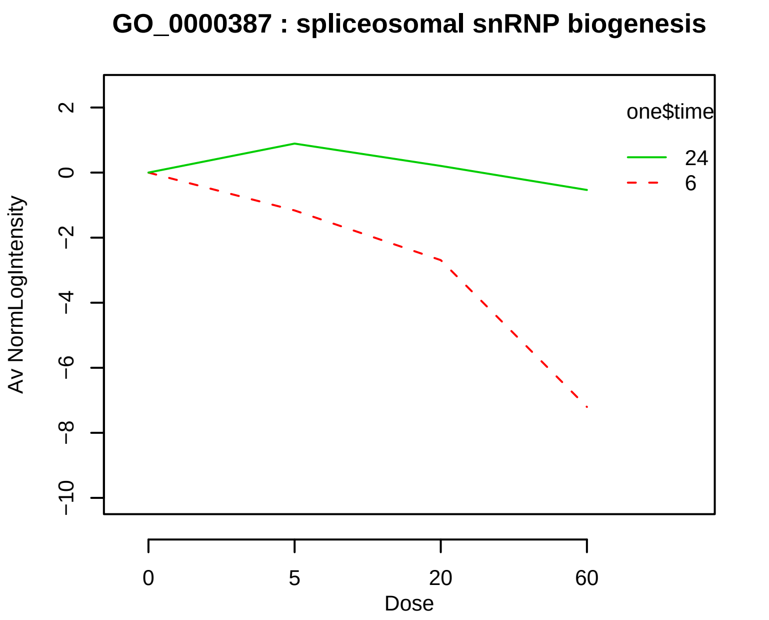
GO_0000387 : spliceosomal snRNP biogenesis
Av NormLogIntensity
Dose
one$time
24
6

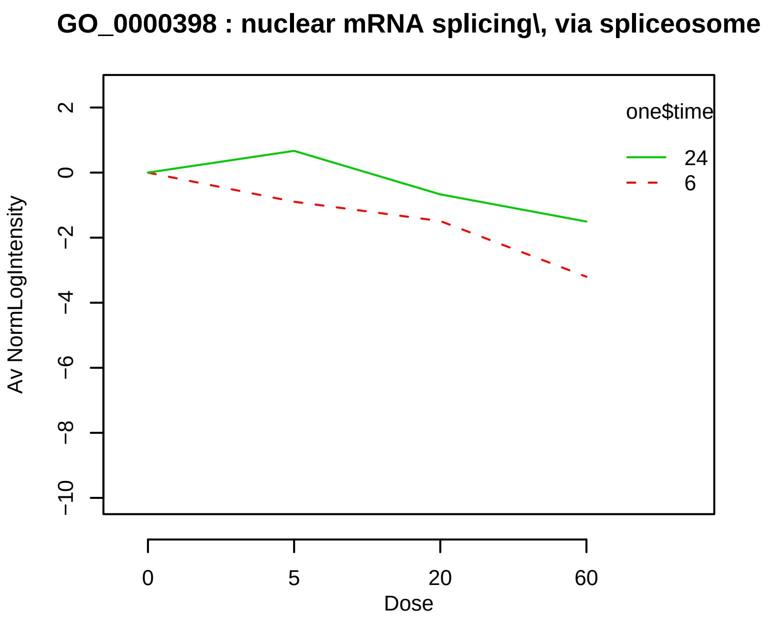
GO_0000398 : nuclear mRNA splicing\, via spliceosome
Av NormLogIntensity
Dose
one$time
24
6

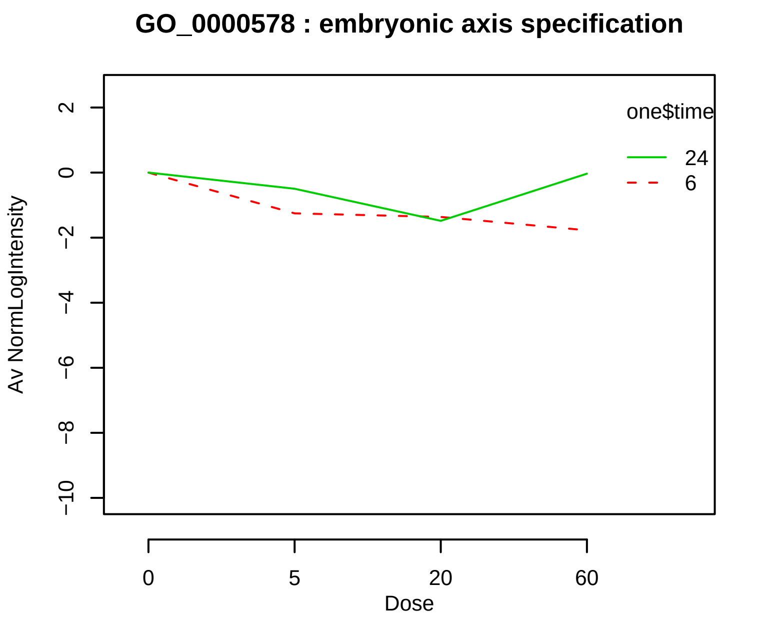
GO_0000578 : embryonic axis specification
Av NormLogIntensity
Dose
one$time
24
6

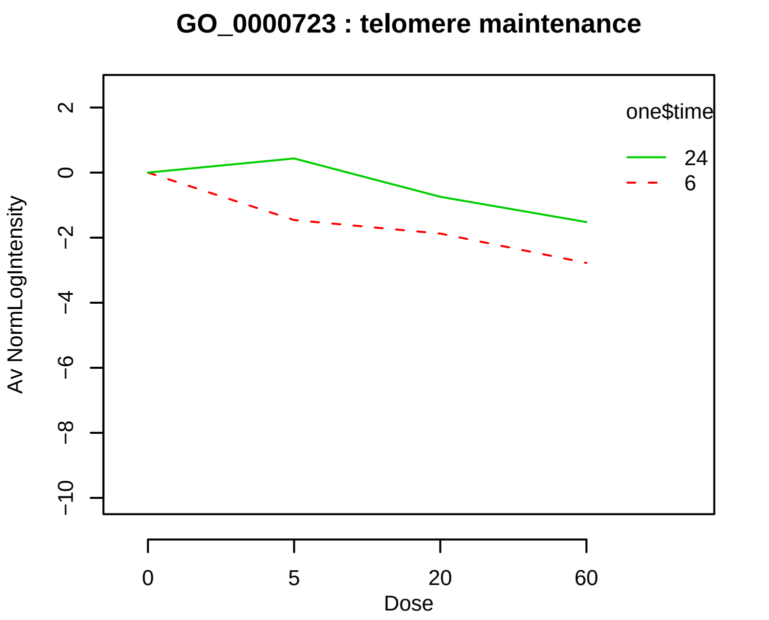
GO_0000723 : telomere maintenance
Av NormLogIntensity
Dose
one$time
24
6

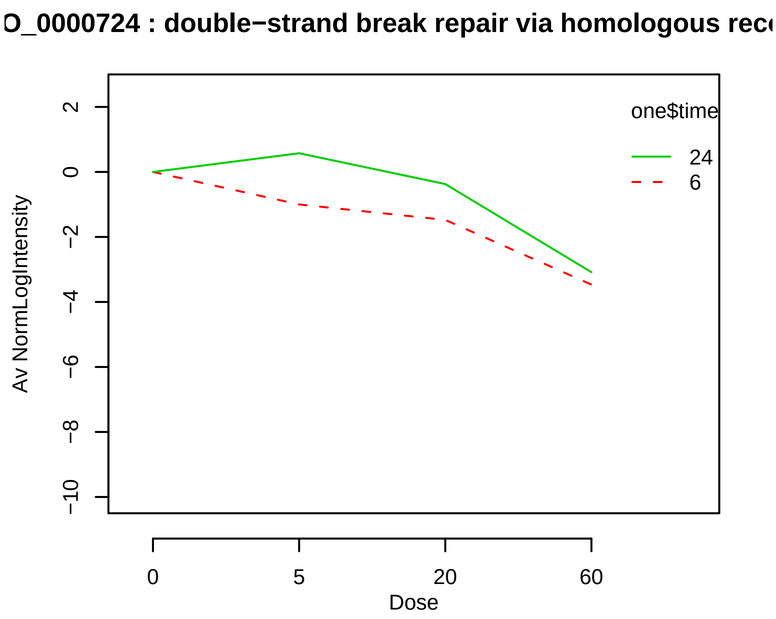
O_0000724 : double−strand break repair via homologous rec
Av NormLogIntensity
Dose
one$time
24
6

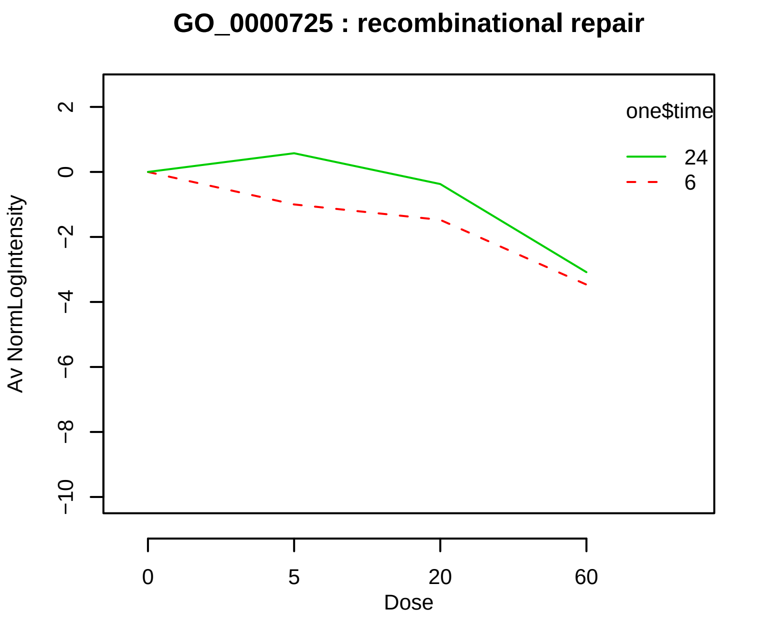
GO_0000725 : recombinational repair
Av NormLogIntensity
Dose
one$time
24
6

GO_0000726 : non−recombinational repair

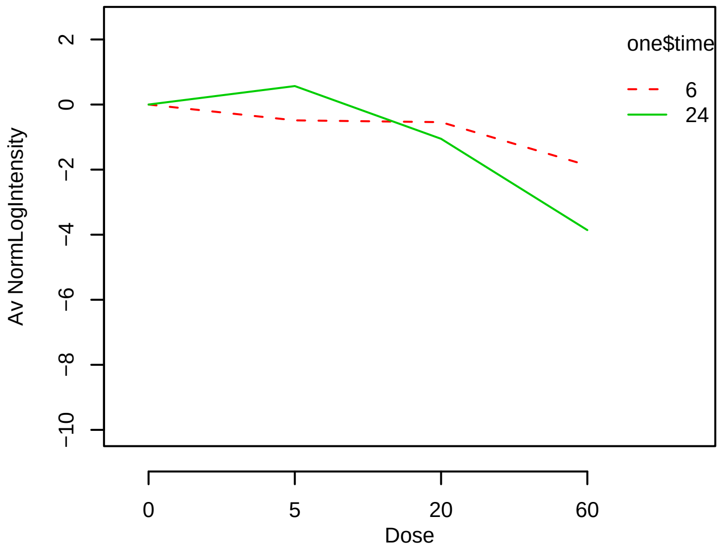

GO_0000731 : DNA synthesis during DNA repair

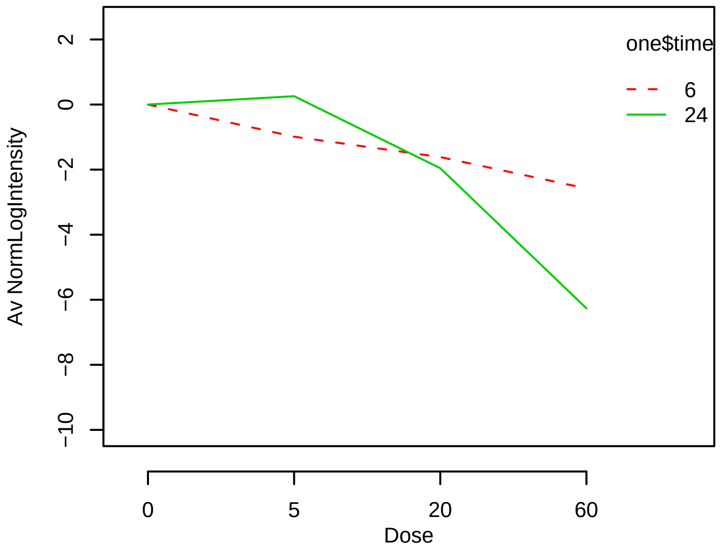

GO_0000819 : sister chromatid segregation

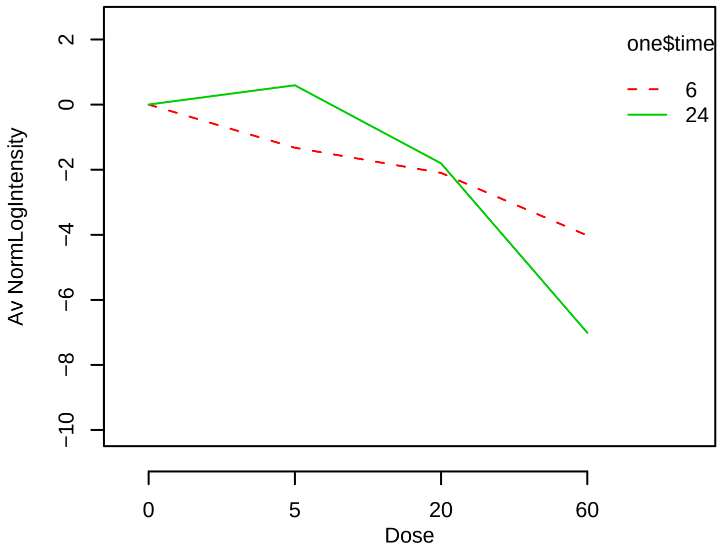

GO_0000904 : cellular morphogenesis during differentiatio

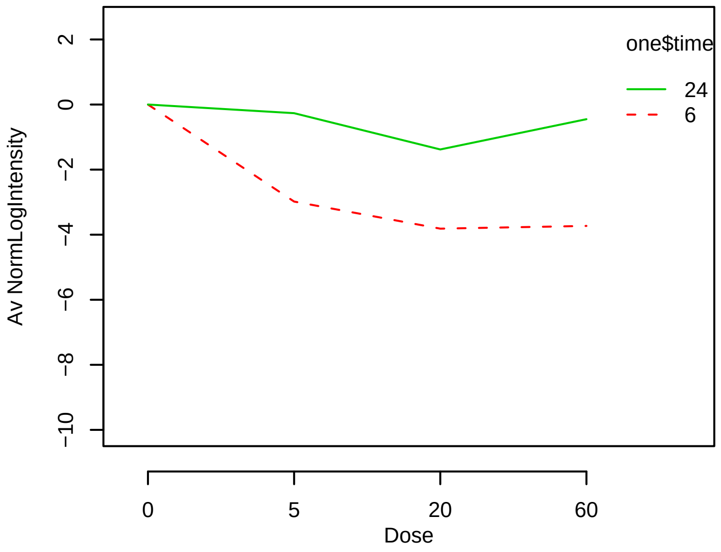

GO_0000910 : cytokinesis

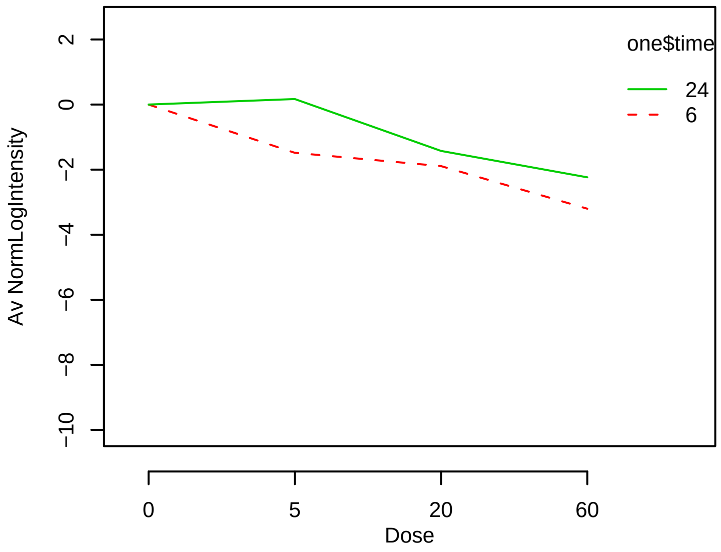

GO_0001501 : skeletal development

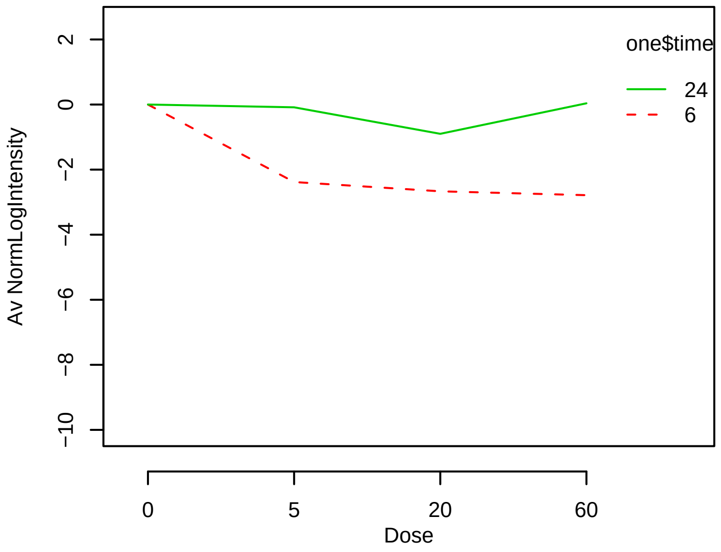

GO_0001502 : cartilage condensation

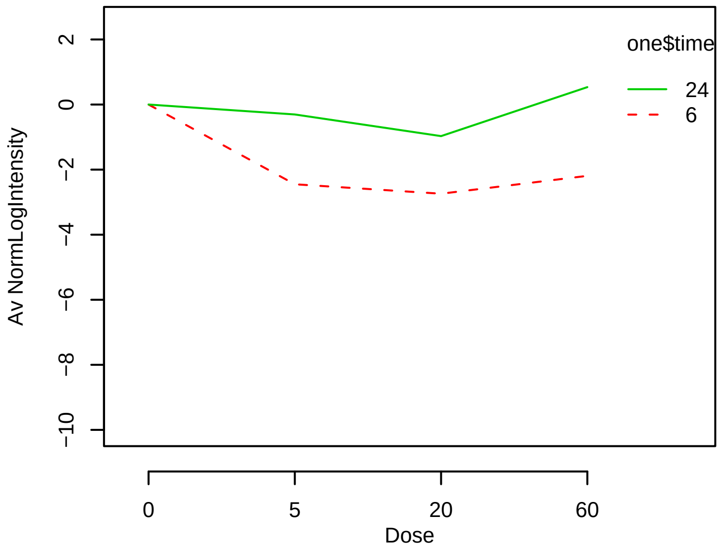

GO_0001503 : ossification

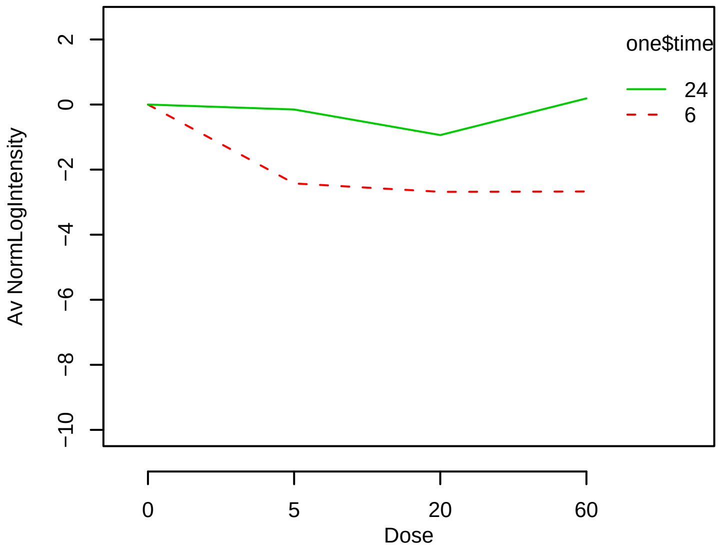

GO_0001504 : neurotransmitter uptake

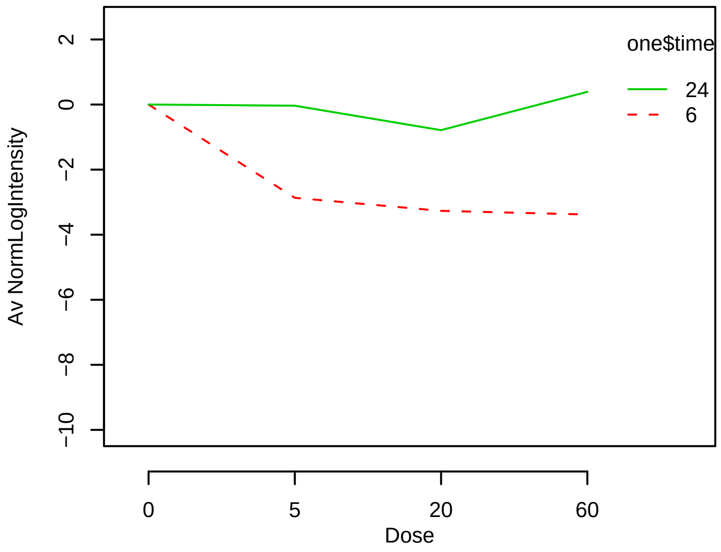

GO_0001505 : regulation of neurotransmitter levels

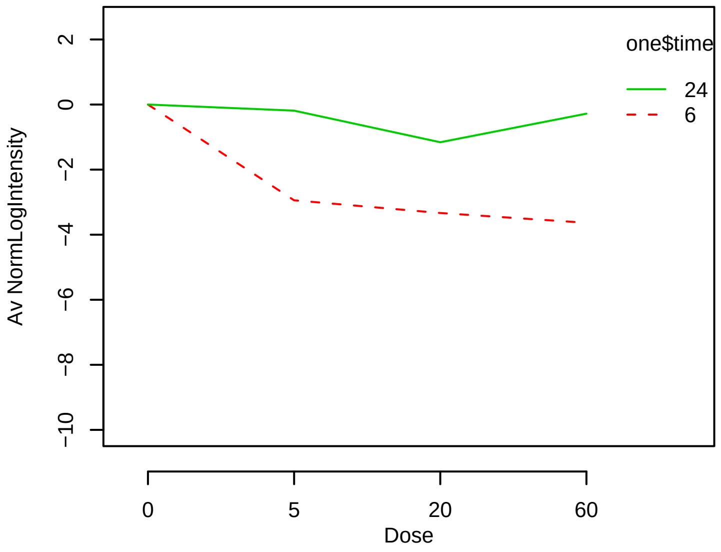

GO_0001508 : regulation of action potential

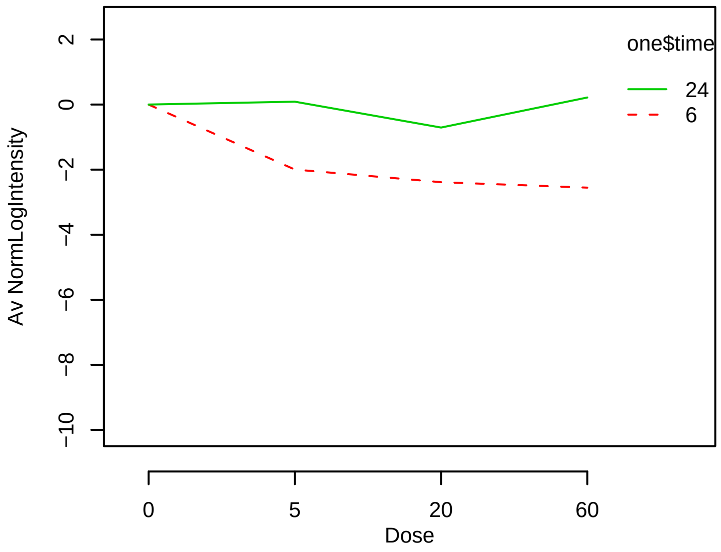

GO_0001514 : selenocysteine incorporation

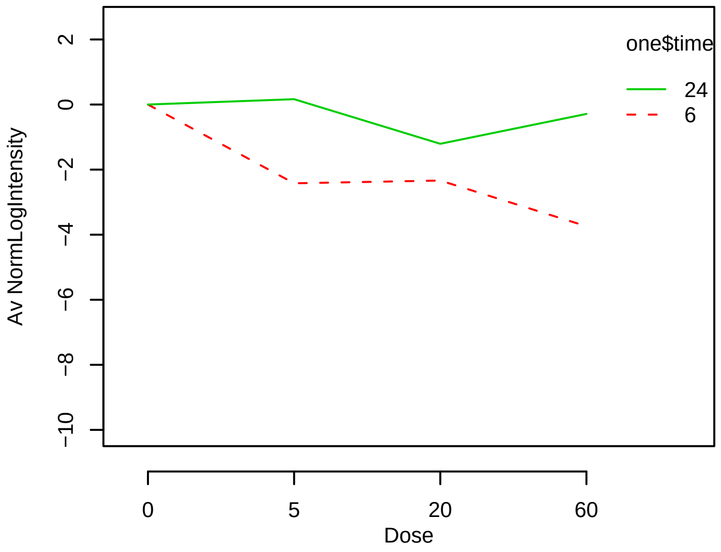

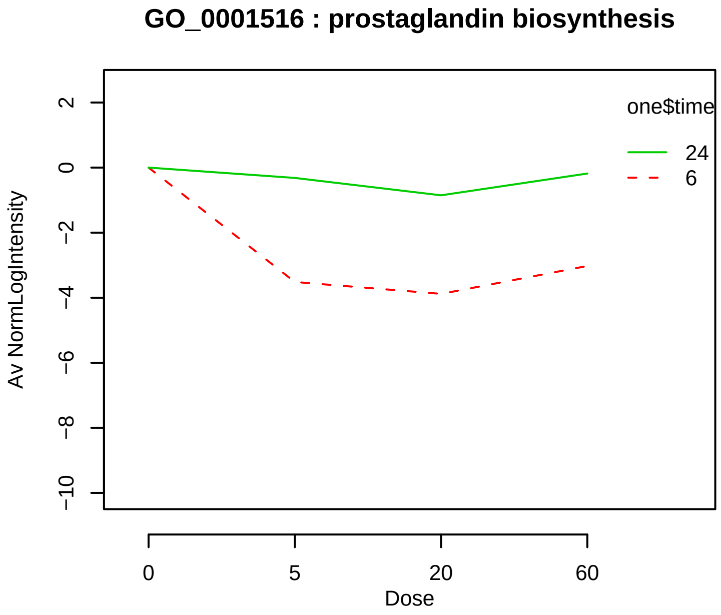
GO_0001516 : prostaglandin biosynthesis
one$time
24
6
Av NormLogIntensity
2
0
−2
−4
−6
−8
−10
0
5
20
60
Dose

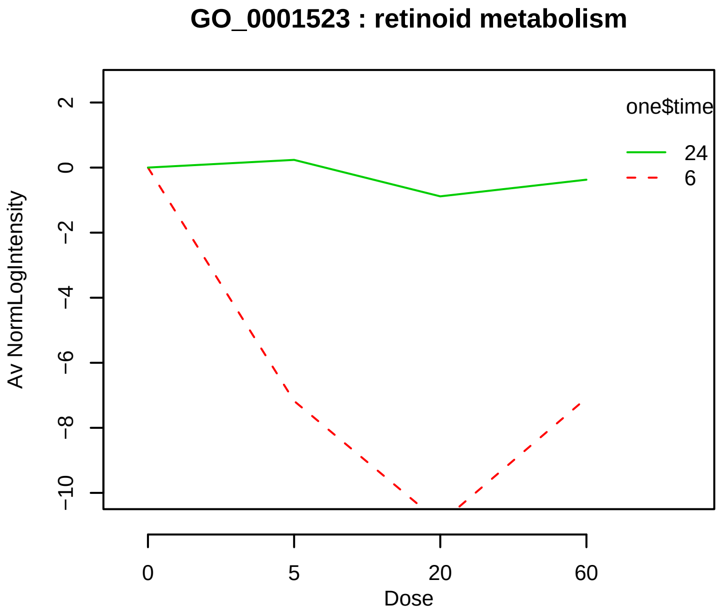
GO_0001523 : retinoid metabolism
one$time
24
6
Av NormLogIntensity
2
0
−2
−4
−6
−8
−10
0
5
20
60
Dose

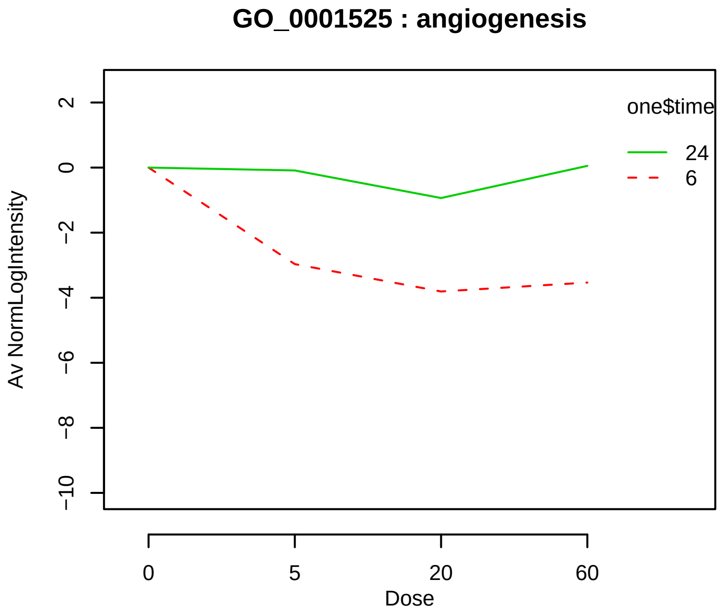
GO_0001525 : angiogenesis
one$time
24
6
Av NormLogIntensity
2
0
−2
−4
−6
−8
−10
0
5
20
60
Dose

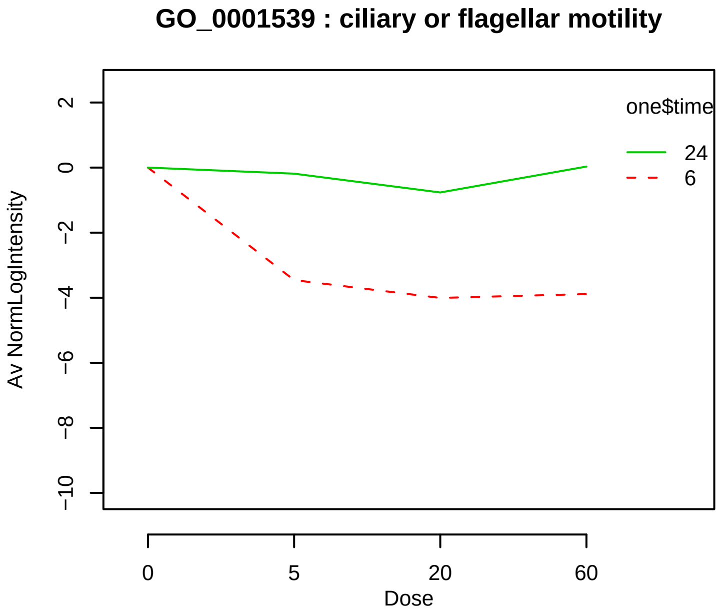
GO_0001539 : ciliary or flagellar motility
one$time
24
6
Av NormLogIntensity
2
0
−2
−4
−6
−8
−10
0
5
20
60
Dose

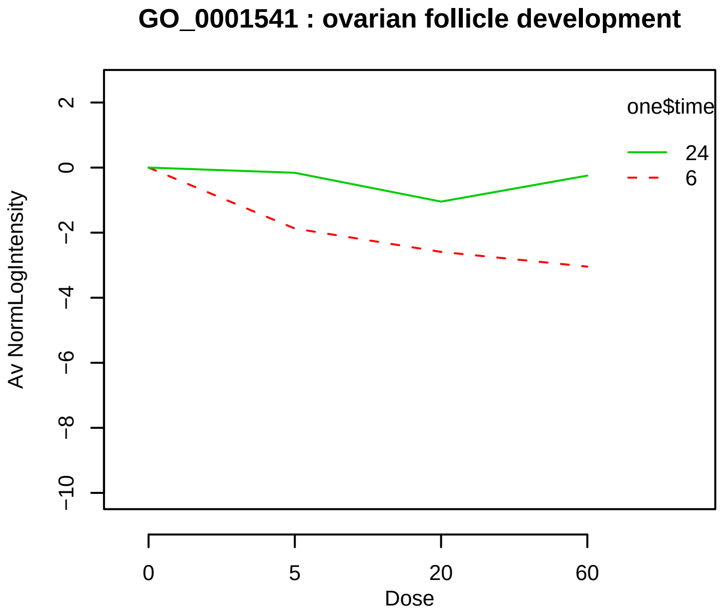
GO_0001541 : ovarian follicle development
one$time
24
6
Av NormLogIntensity
2
0
−2
−4
−6
−8
−10
0
5
20
60
Dose

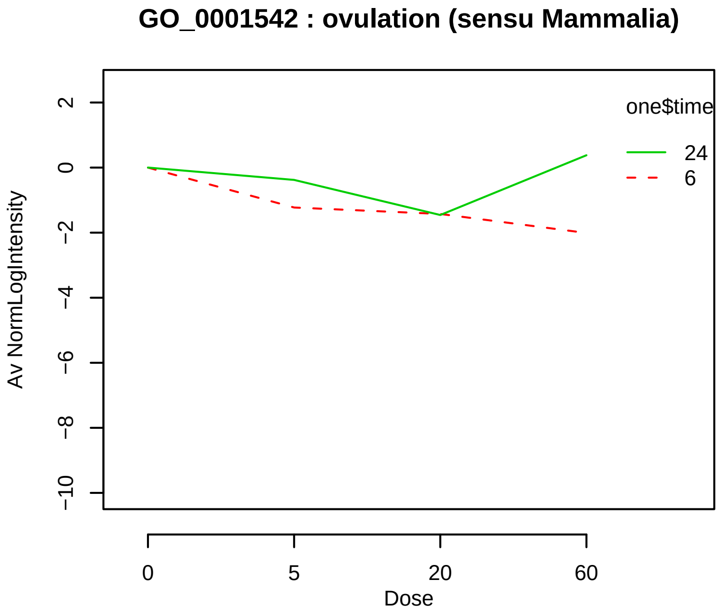
GO_0001542 : ovulation (sensu Mammalia)
one$time
24
6
Av NormLogIntensity
2
0
−2
−4
−6
−8
−10
0
5
20
60
Dose

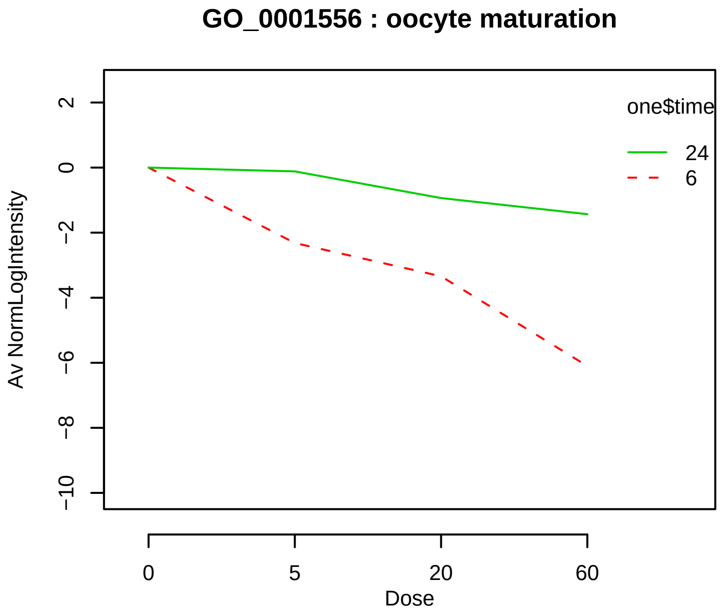
GO_0001556 : oocyte maturation
Av NormLogIntensity
2
0
−2
−4
−6
−8
−10
one$time
24
6
0
5
20
60
Dose

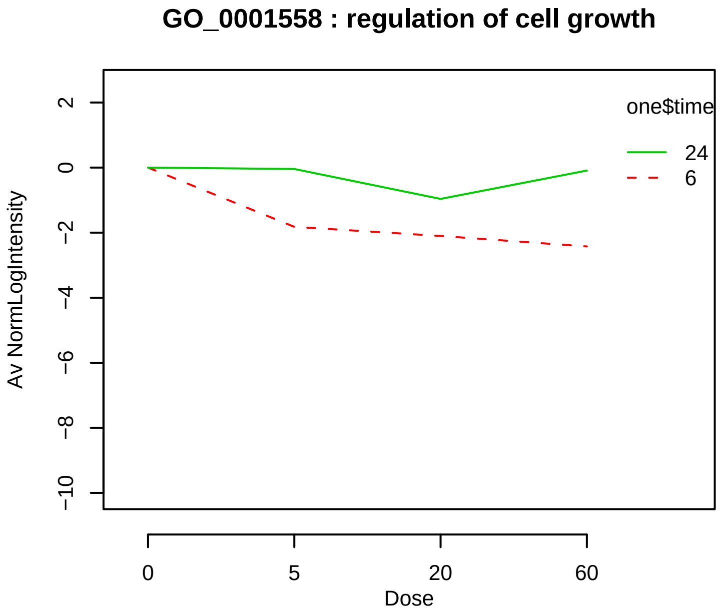
GO_0001558 : regulation of cell growth
Av NormLogIntensity
2
0
−2
−4
−6
−8
−10
one$time
24
6
0
5
20
60
Dose

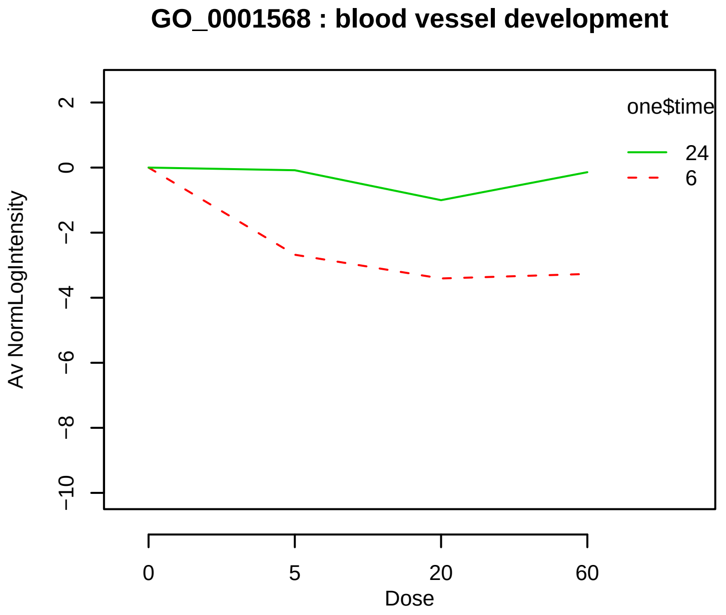
GO_0001568 : blood vessel development
Av NormLogIntensity
2
0
−2
−4
−6
−8
−10
one$time
24
6
0
5
20
60
Dose

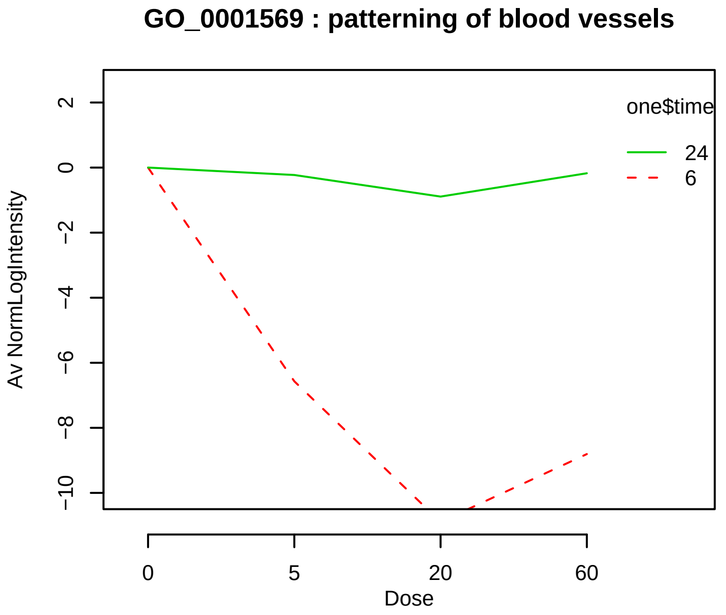
GO_0001569 : patterning of blood vessels
Av NormLogIntensity
2
0
−2
−4
−6
−8
−10
one$time
24
6
0
5
20
60
Dose

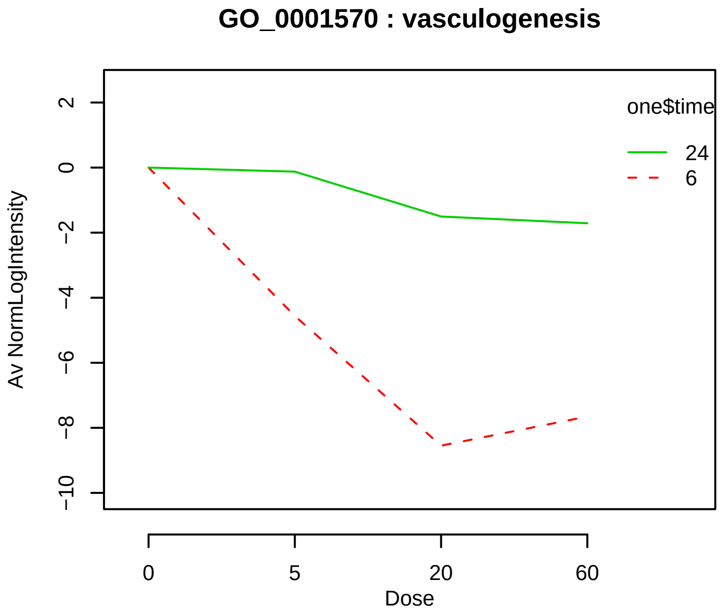
GO_0001570 : vasculogenesis
Av NormLogIntensity
2
0
−2
−4
−6
−8
−10
one$time
24
6
0
5
20
60
Dose

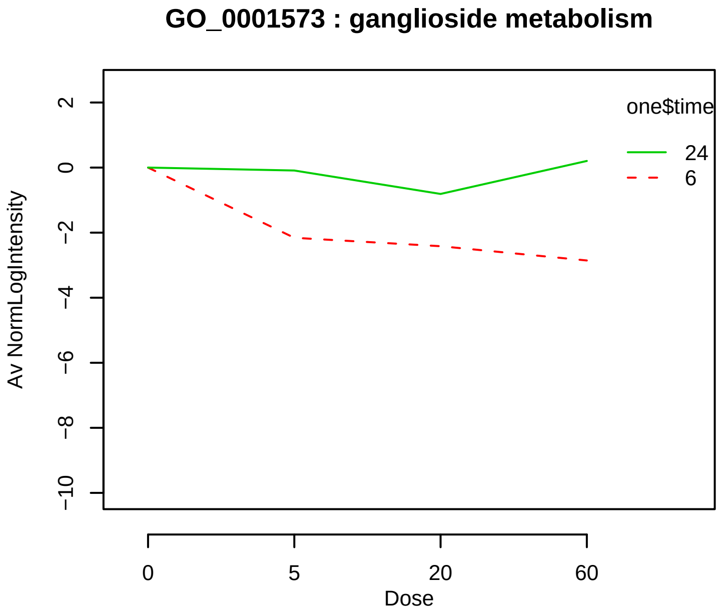
GO_0001573 : ganglioside metabolism
Av NormLogIntensity
2
0
−2
−4
−6
−8
−10
one$time
24
6
0
5
20
60
Dose

GO_0001578 : microtubule bundle formation

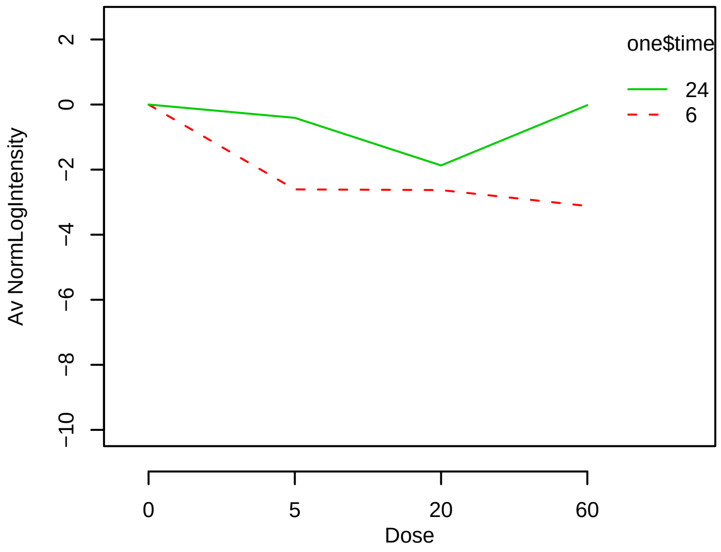

GO_0001649 : osteoblast differentiation

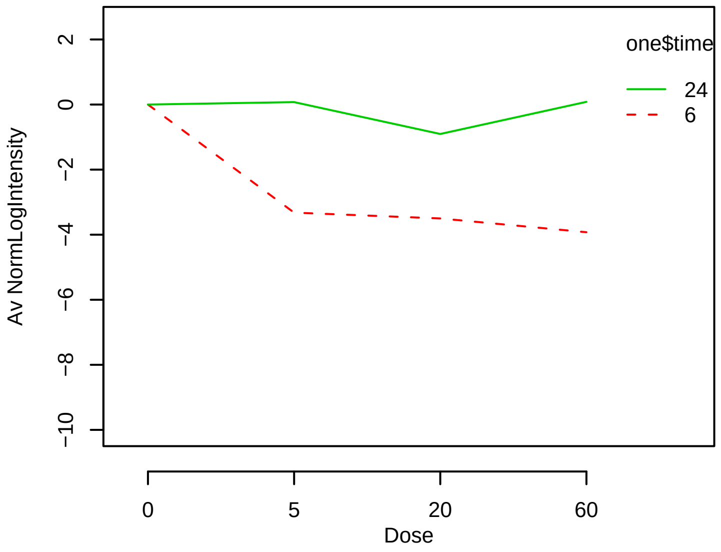

GO_0001654 : eye development

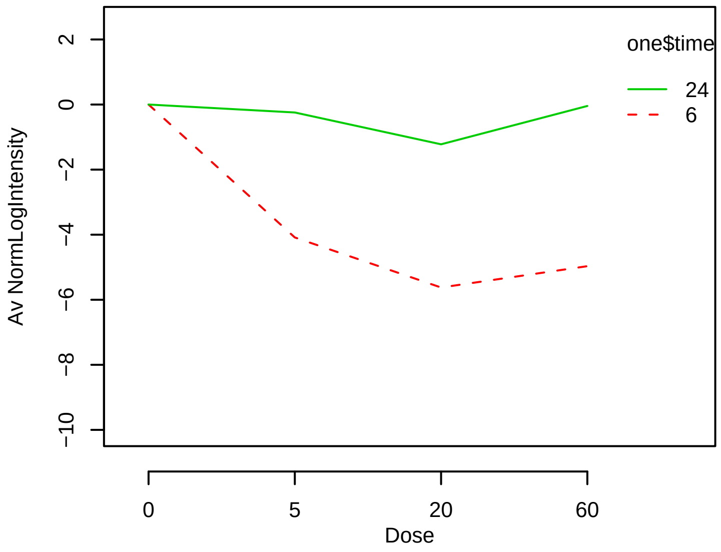

GO_0001655 : urogenital system development

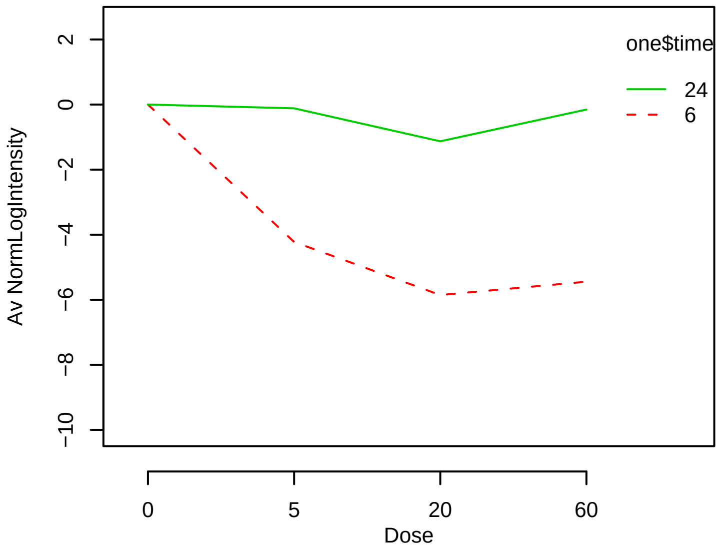

GO_0001656 : metanephros development

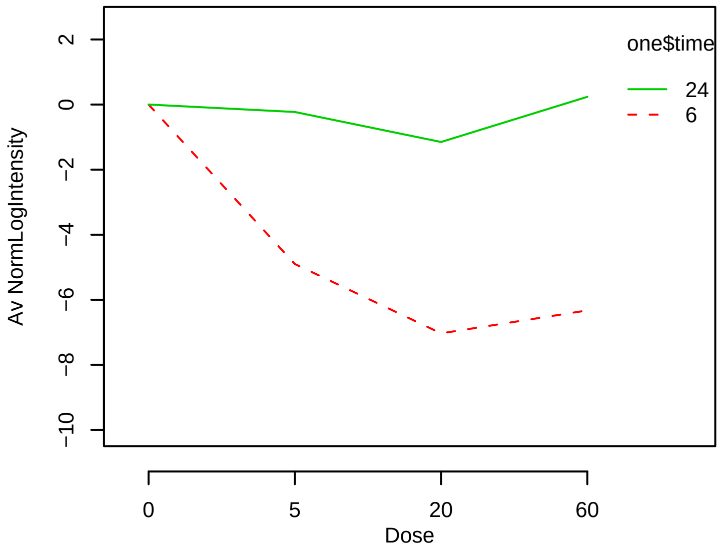

GO_0001657 : ureteric bud development

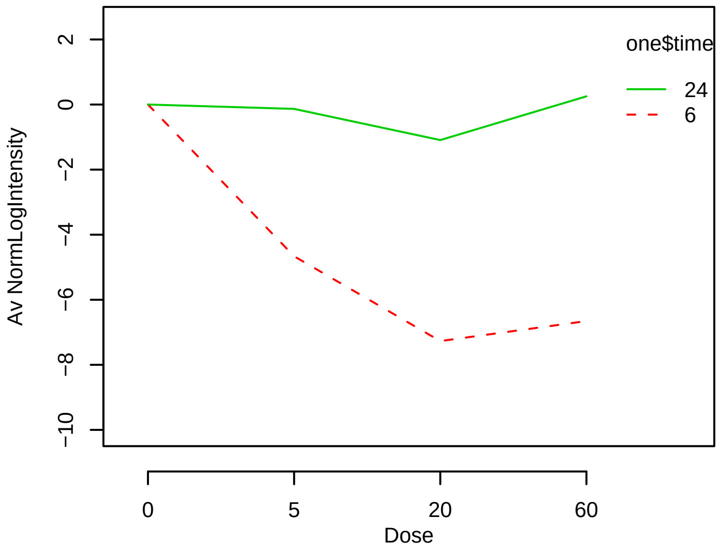

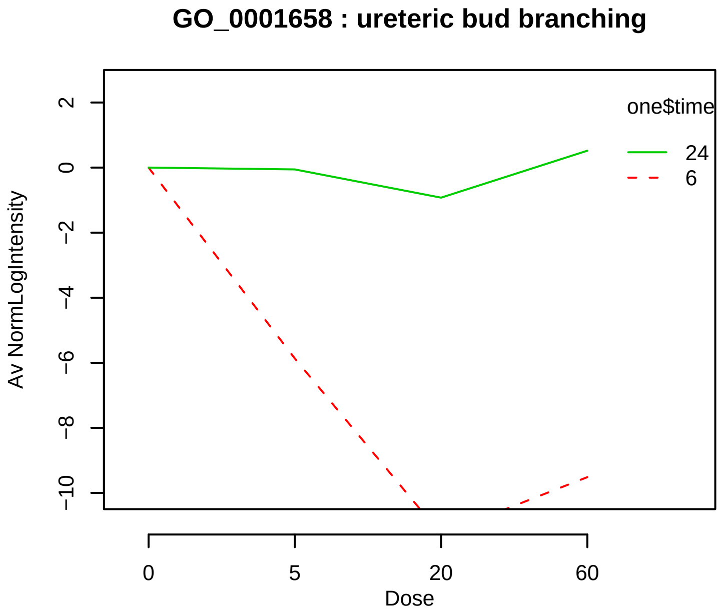
GO_0001658 : ureteric bud branching
one$time
24
6
Av NormLogIntensity
Dose
0
5
20
60
2
0
−2
−4
−6
−8
−10

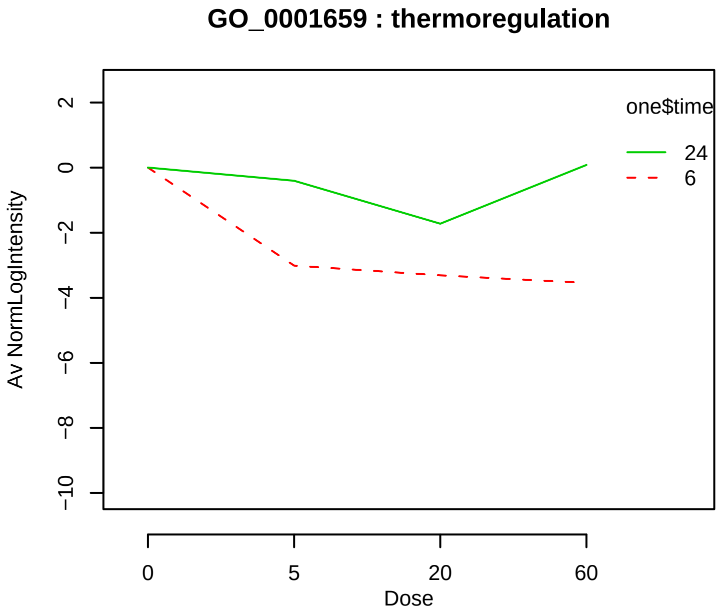
GO_0001659 : thermoregulation
one$time
24
6
Av NormLogIntensity
Dose
0
5
20
60
2
0
−2
−4
−6
−8
−10

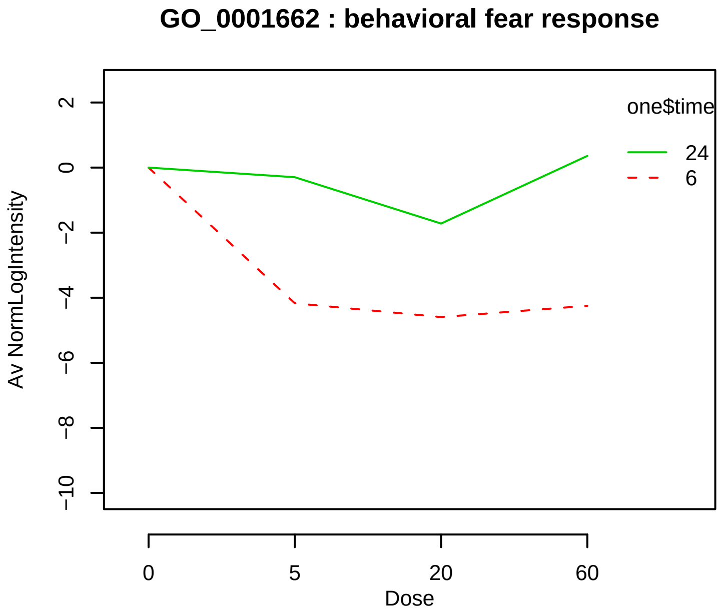
GO_0001662 : behavioral fear response
one$time
24
6
Av NormLogIntensity
Dose
0
5
20
60
2
0
−2
−4
−6
−8
−10

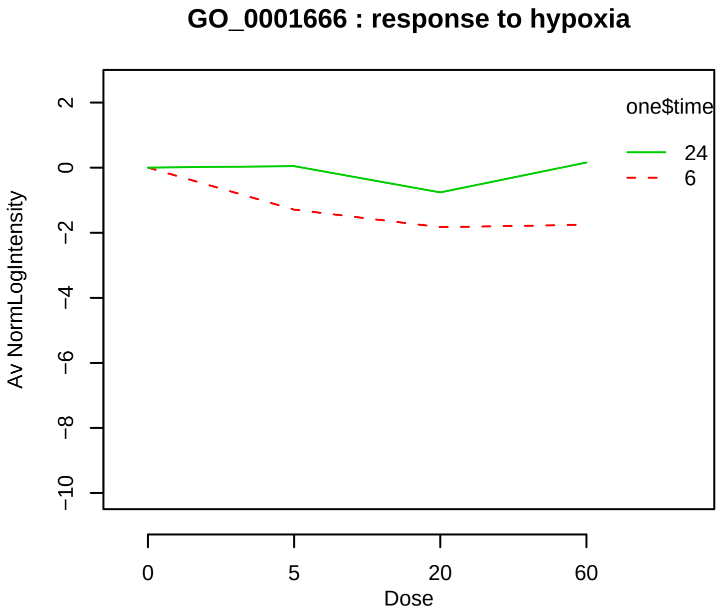
GO_0001666 : response to hypoxia
one$time
24
6
Av NormLogIntensity
Dose
0
5
20
60
2
0
−2
−4
−6
−8
−10

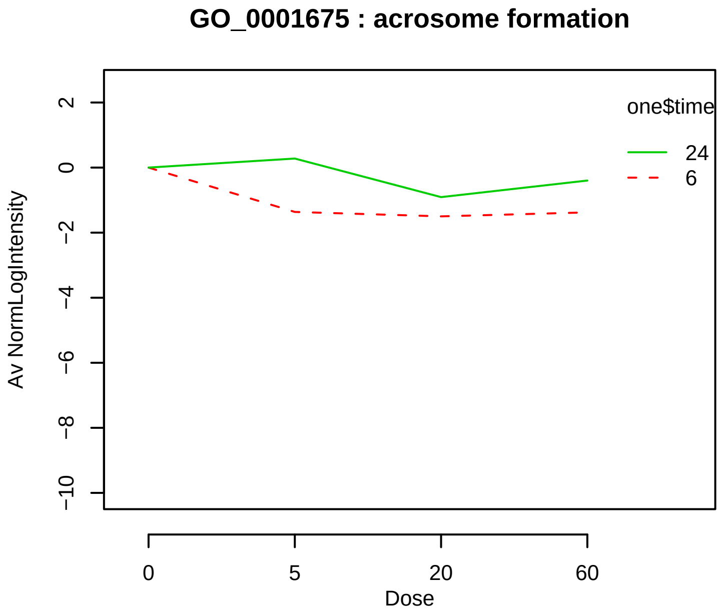
GO_0001675 : acrosome formation
one$time
24
6
Av NormLogIntensity
Dose
0
5
20
60
2
0
−2
−4
−6
−8
−10

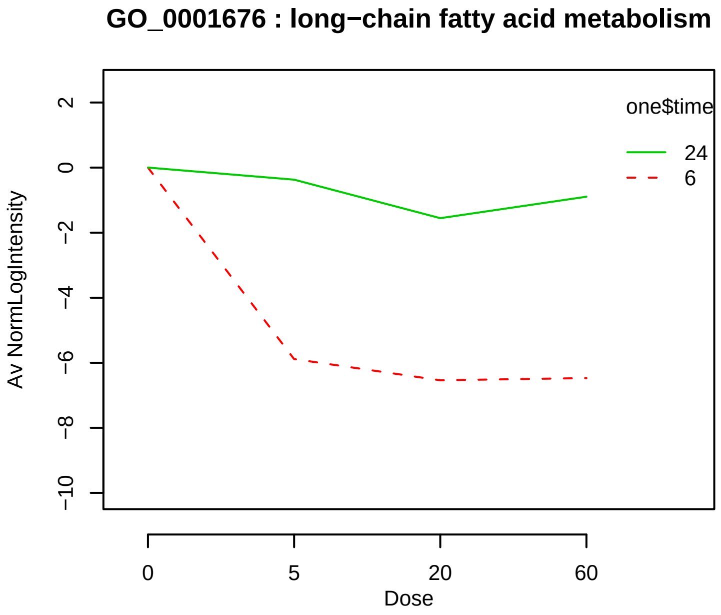
GO_0001676 : long−chain fatty acid metabolism
one$time
24
6
Av NormLogIntensity
Dose
0
5
20
60
2
0
−2
−4
−6
−8
−10

GO_0001701 : embryonic development (sensu Mammalia)

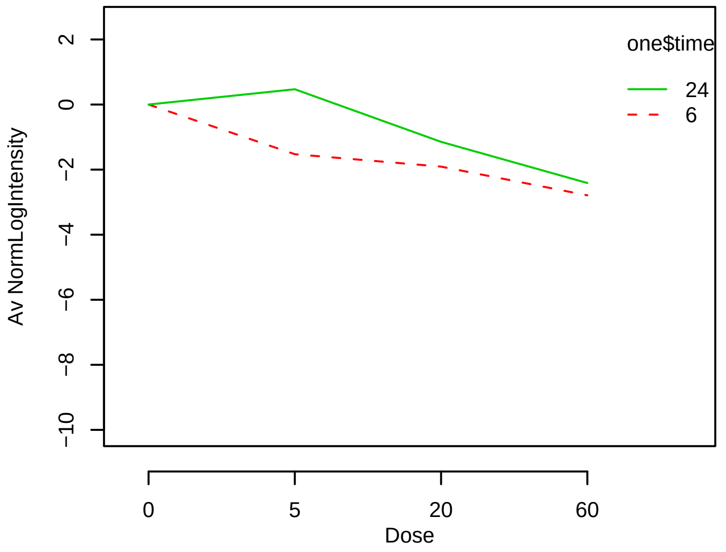

GO_0001702 : gastrulation (sensu Deuterostomia)

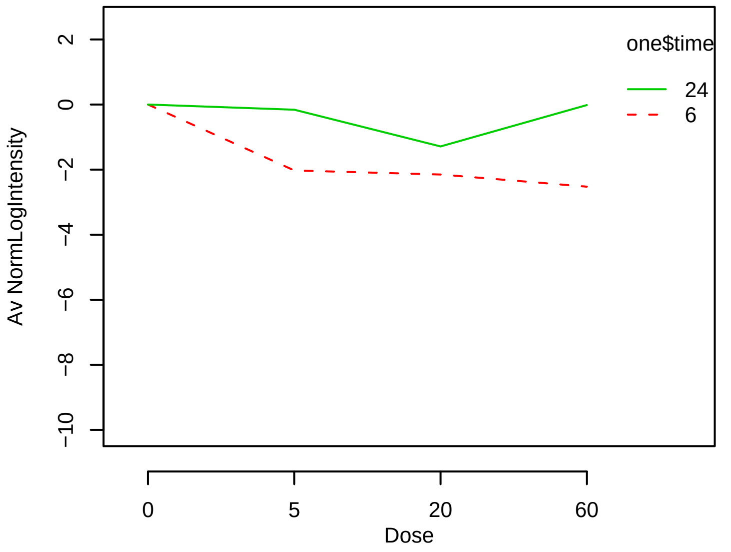

GO_0001704 : formation of primary germ layer

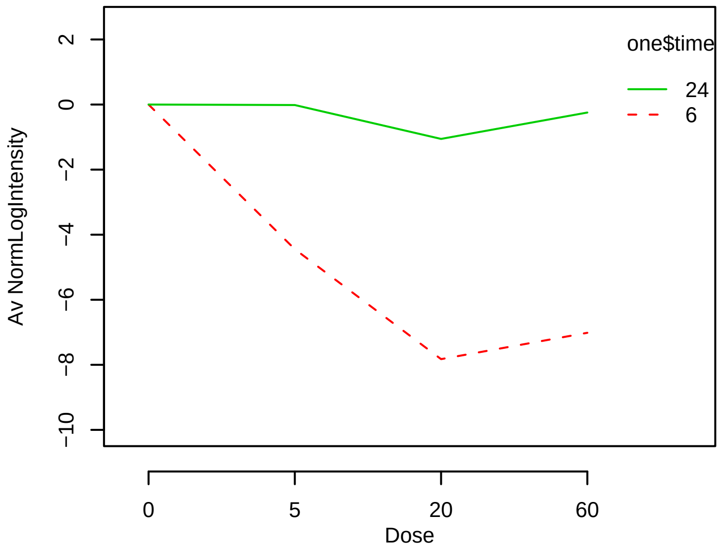

GO_0001707 : mesoderm formation

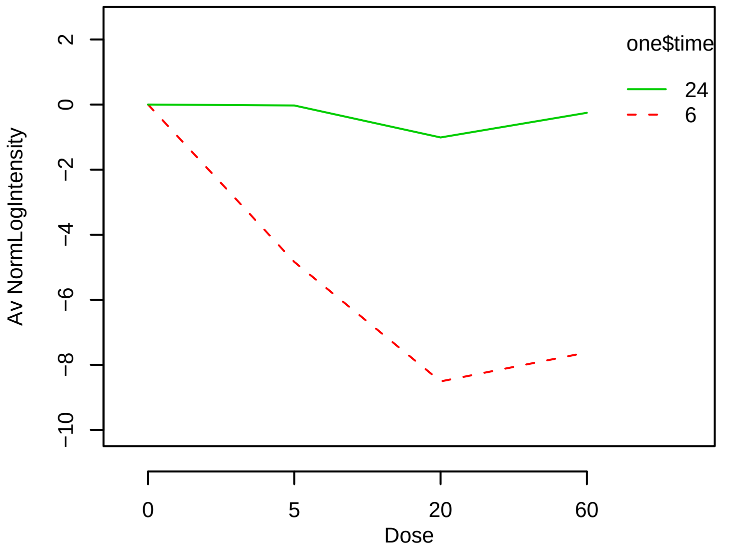

GO_0001708 : cell fate specification

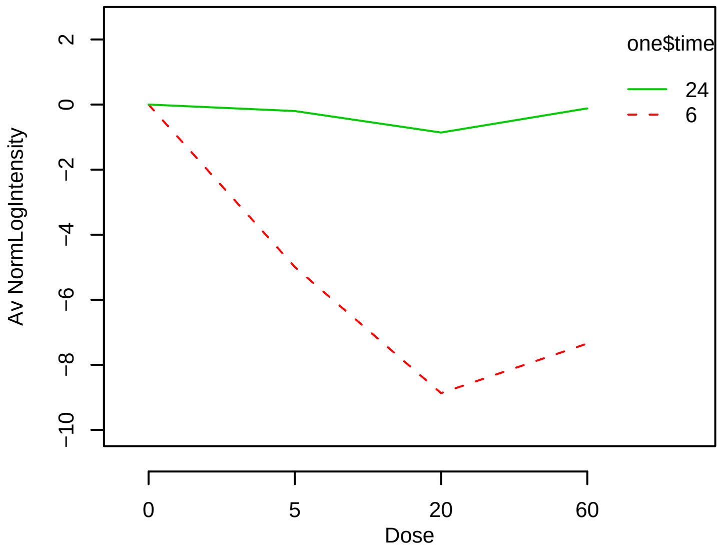

GO_0001709 : cell fate determination

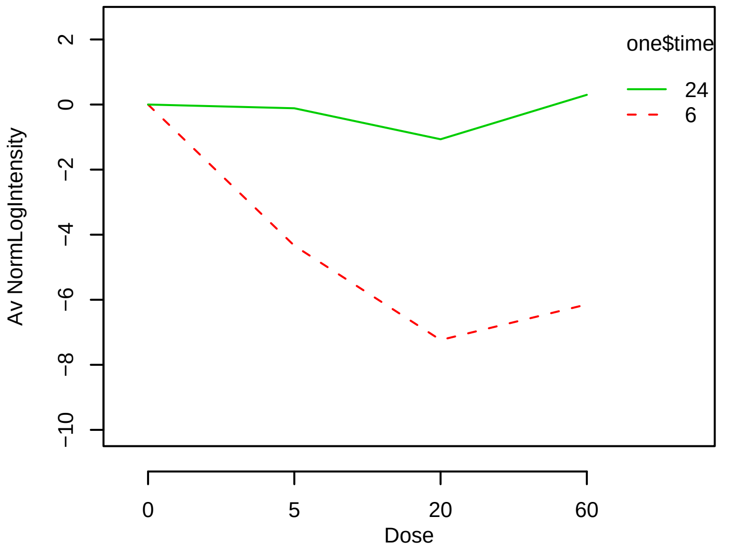

GO_0001736 : establishment of planar polarity

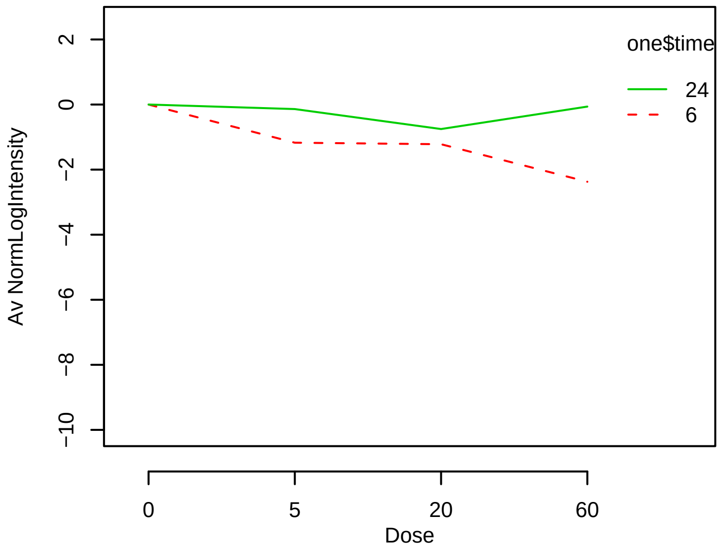

GO_0001738 : morphogenesis of a polarized epithelium

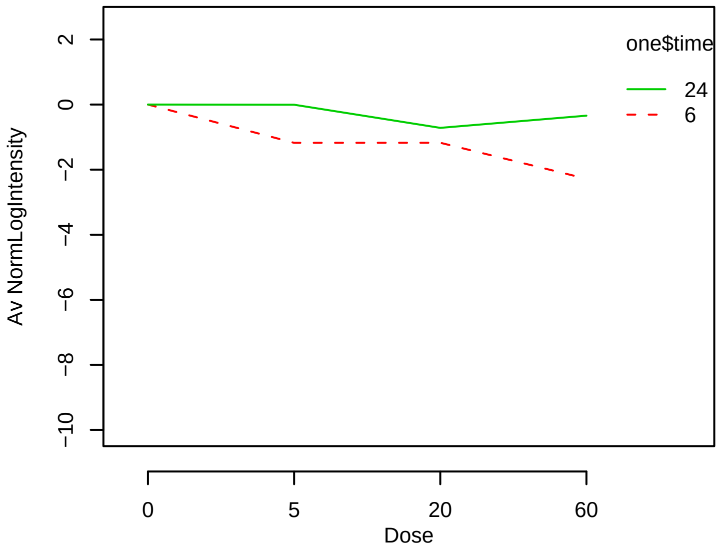

GO_0001747 : eye development (sensu Mammalia)

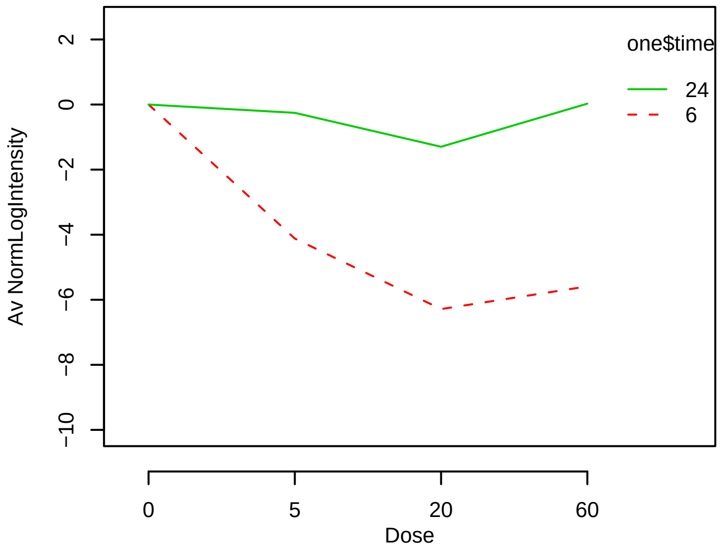

GO_0001754 : eye photoreceptor cell differentiation

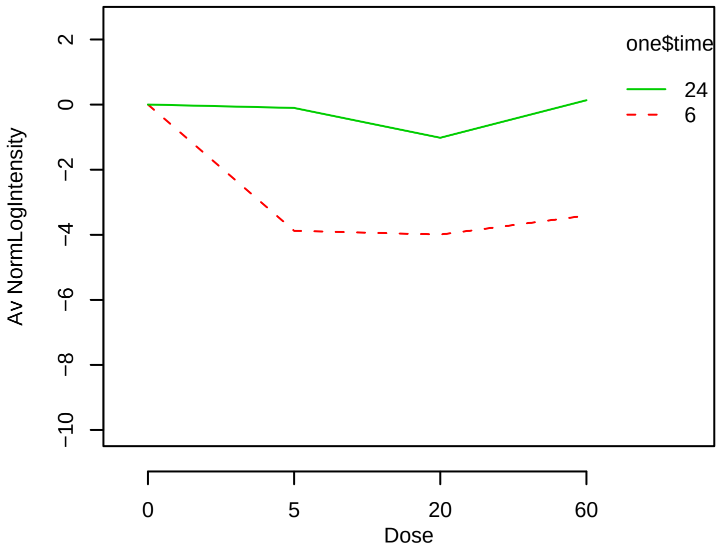

GO_0001755 : neural crest cell migration

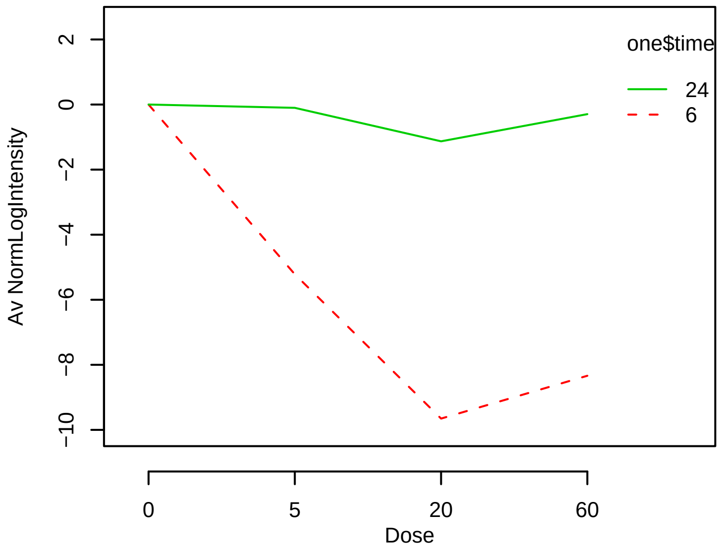

GO_0001756 : somitogenesis

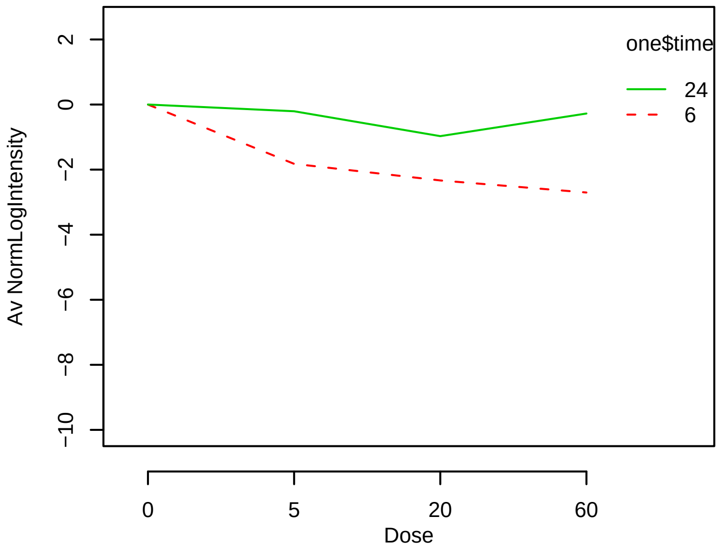

GO_0001759 : induction of an organ

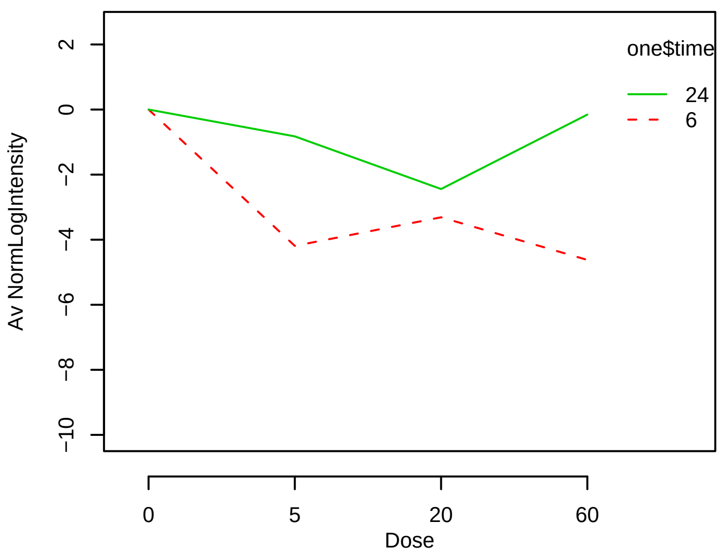

GO_0001763 : morphogenesis of a branching structure

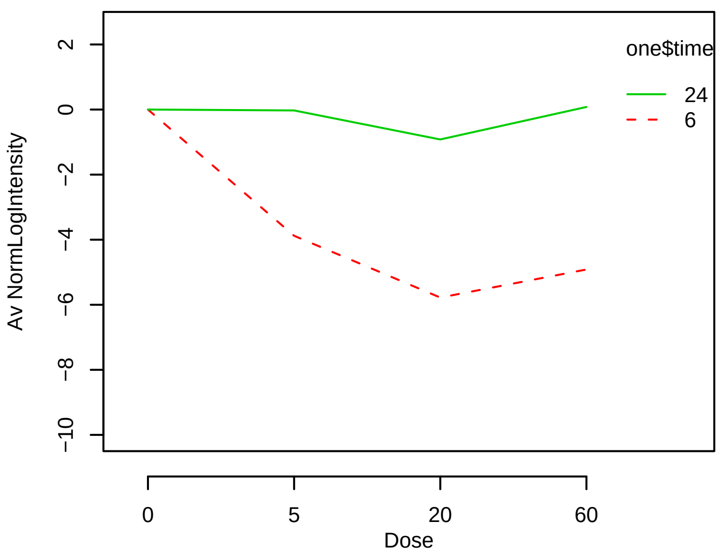

GO_0001764 : neuron migration

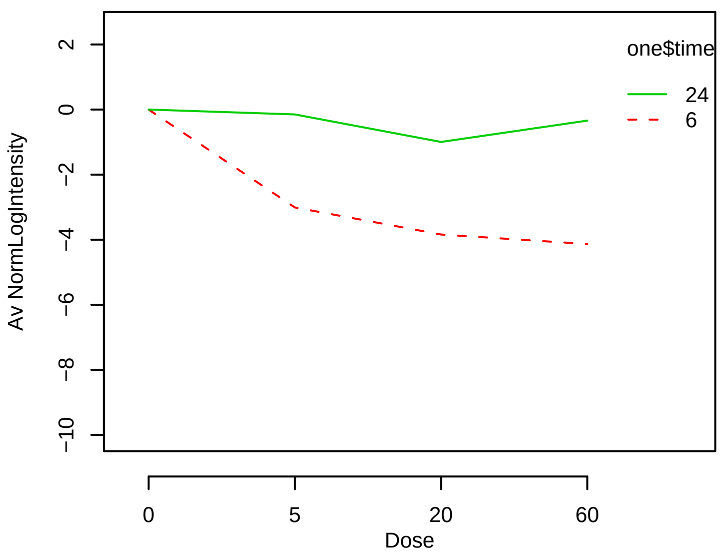

GO_0001773 : myeloid dendritic cell activation

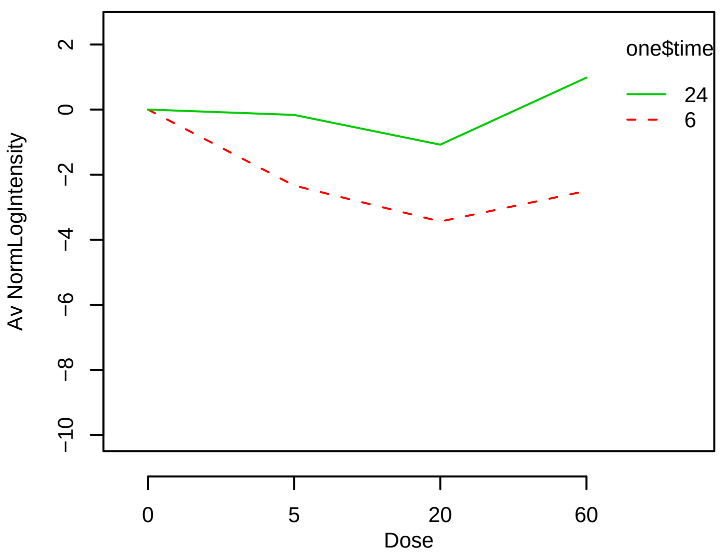

GO_0001775 : cell activation

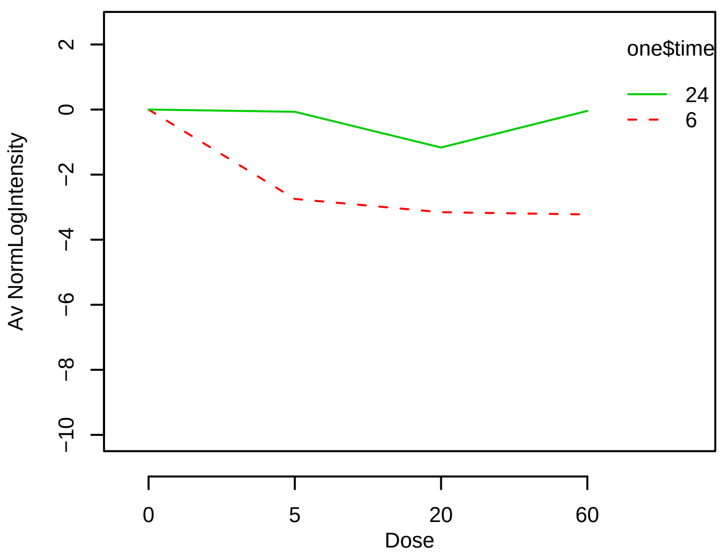

GO_0001776 : leukocyte homeostasis

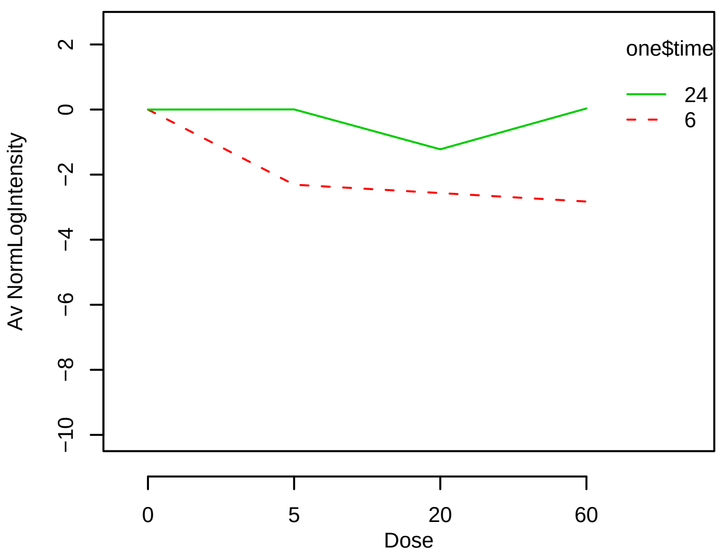

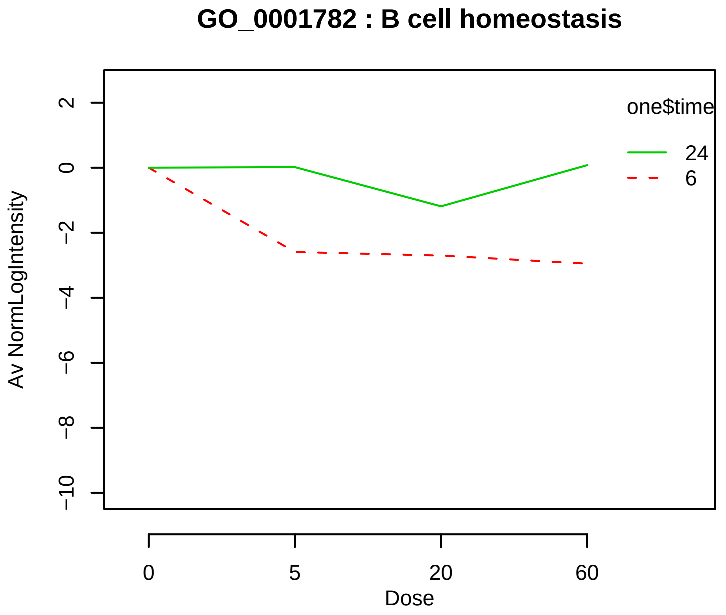

GO_0001782 : B cell homeostasis
one$time
24
6
Av NormLogIntensity
2
0
−2
−4
−6
−8
−10
0
5
20
60
Dose

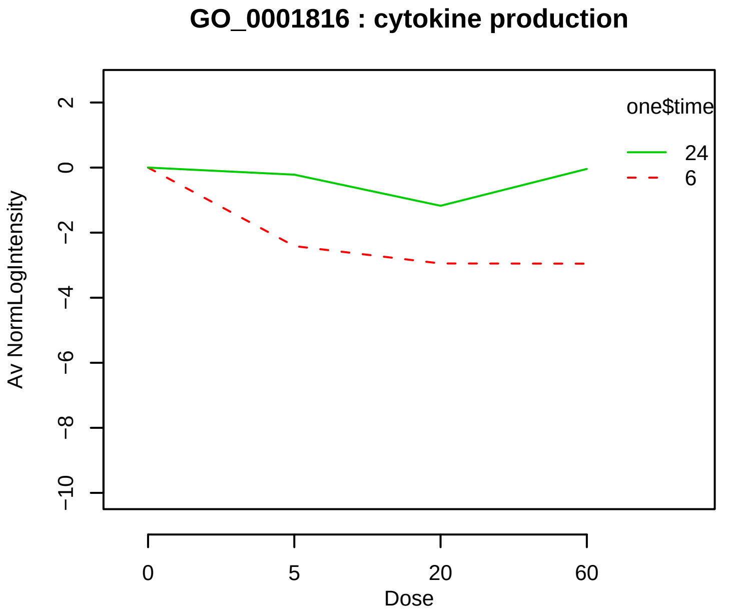

GO_0001816 : cytokine production
one$time
24
6
Av NormLogIntensity
2
0
−2
−4
−6
−8
−10
0
5
20
60
Dose

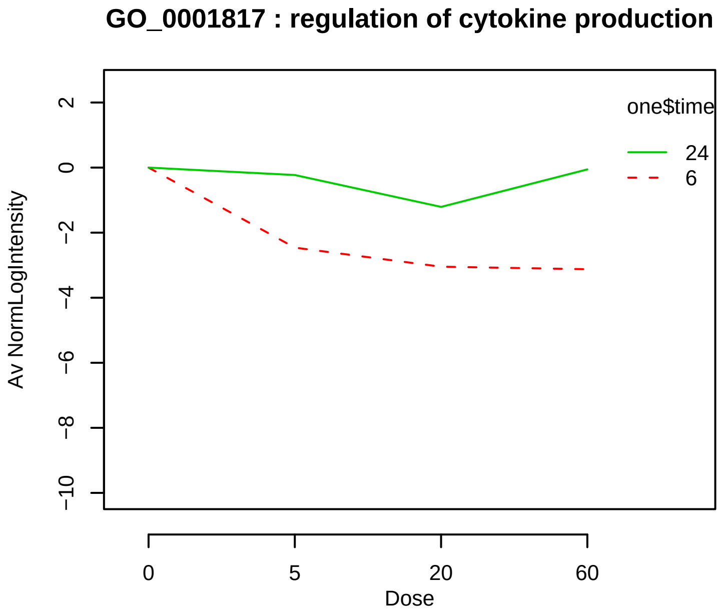

GO_0001817 : regulation of cytokine production
one$time
24
6
Av NormLogIntensity
2
0
−2
−4
−6
−8
−10
0
5
20
60
Dose

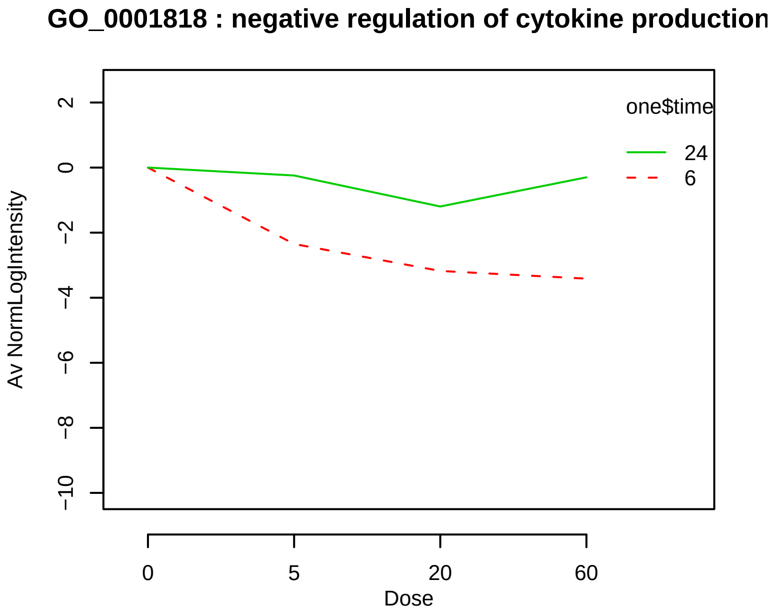

GO_0001818 : negative regulation of cytokine production
one$time
24
6
Av NormLogIntensity
2
0
−2
−4
−6
−8
−10
0
5
20
60
Dose

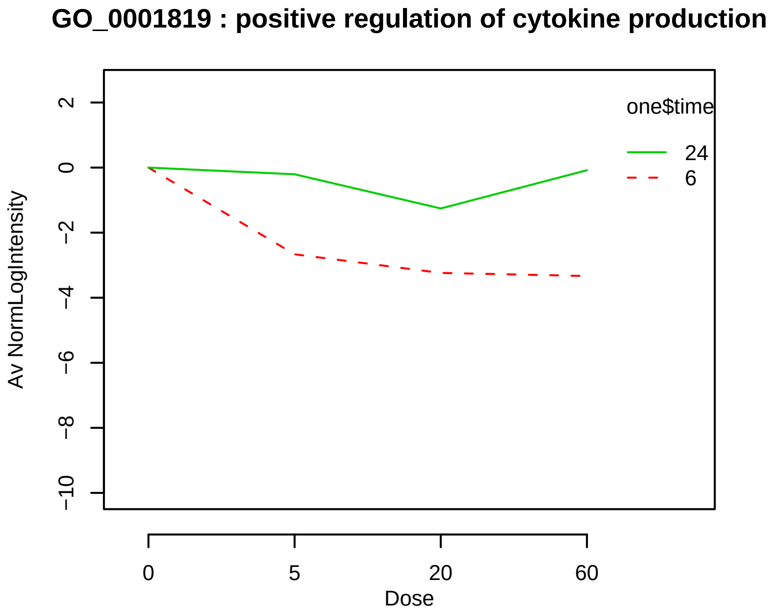

GO_0001819 : positive regulation of cytokine production
one$time
24
6
Av NormLogIntensity
2
0
−2
−4
−6
−8
−10
0
5
20
60
Dose

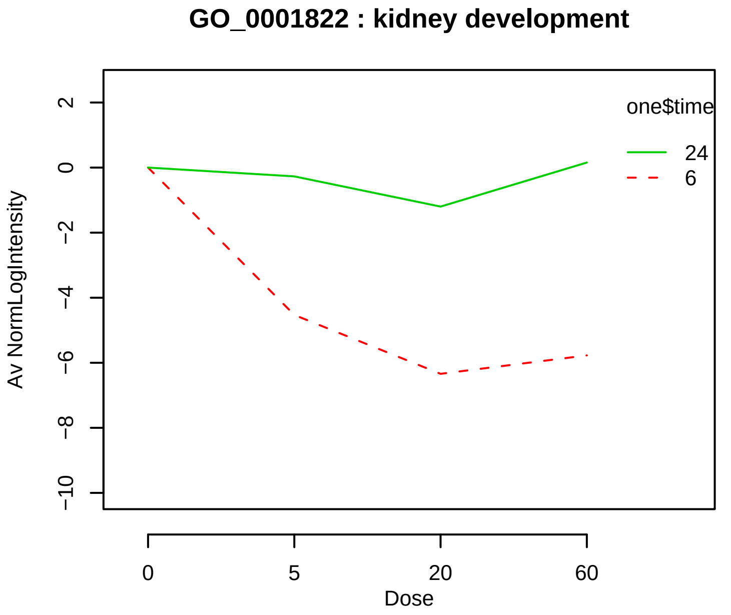

GO_0001822 : kidney development
one$time
24
6
Av NormLogIntensity
2
0
−2
−4
−6
−8
−10
0
5
20
60
Dose

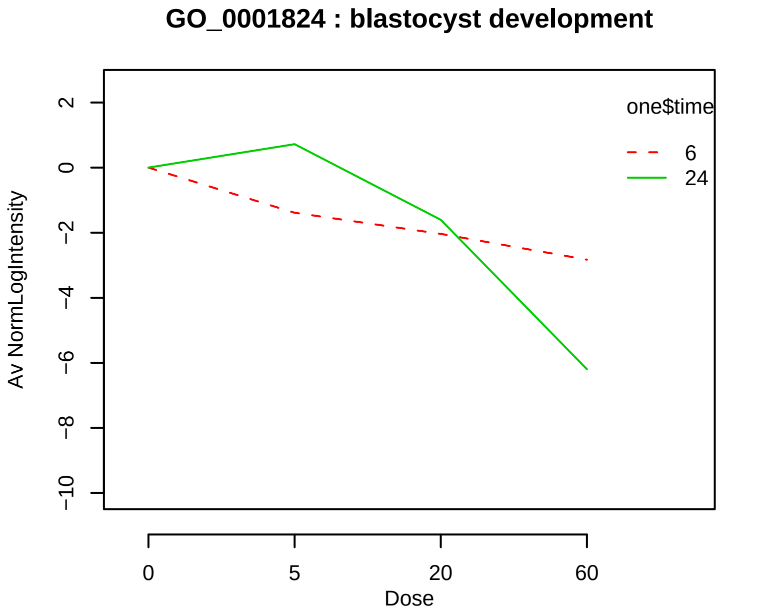
GO_0001824 : blastocyst development
one$time
6
24
Av NormLogIntensity
2
0
−2
−4
−6
−8
−10
0
5
20
60
Dose

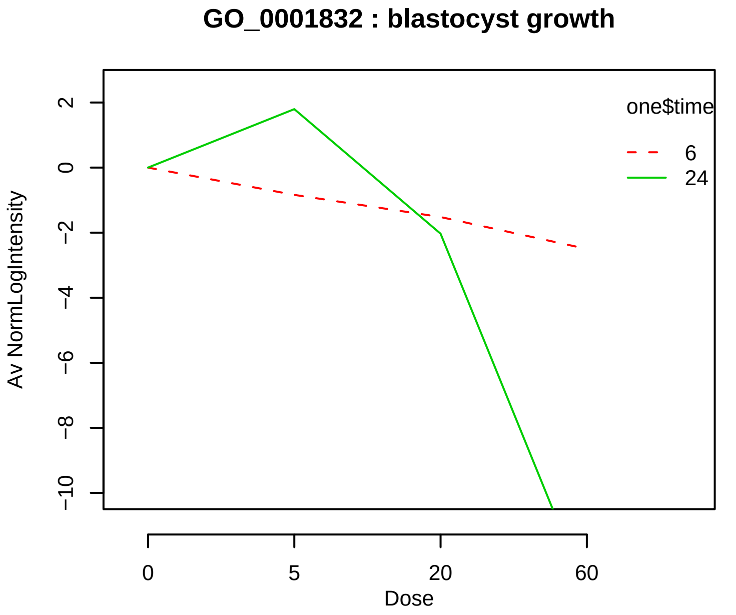
GO_0001832 : blastocyst growth
one$time
6
24
Av NormLogIntensity
2
0
−2
−4
−6
−8
−10
0
5
20
60
Dose

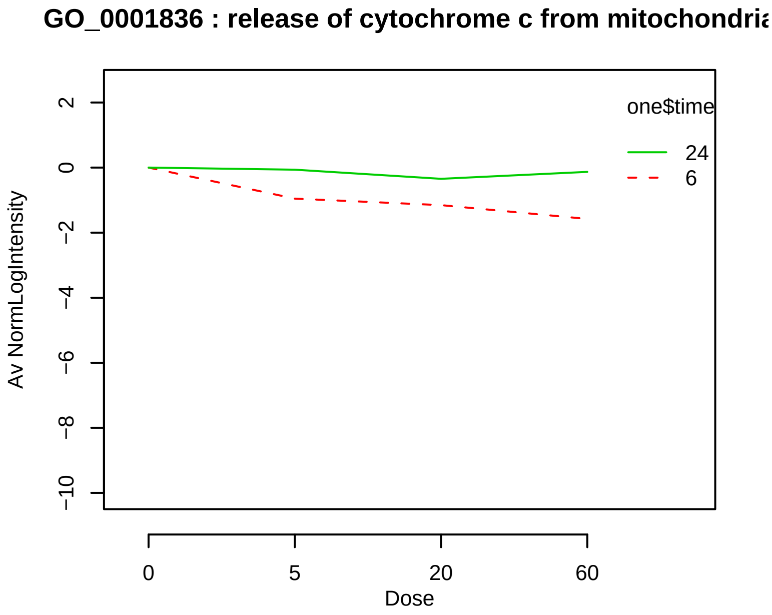
GO_0001836 : release of cytochrome c from mitochondria
one$time
24
6
Av NormLogIntensity
2
0
−2
−4
−6
−8
−10
0
5
20
60
Dose

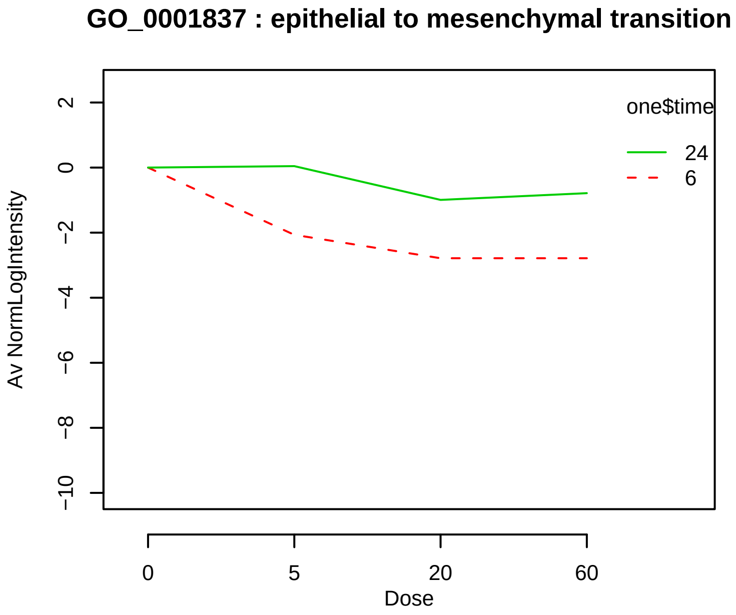
GO_0001837 : epithelial to mesenchymal transition
one$time
24
6
Av NormLogIntensity
2
0
−2
−4
−6
−8
−10
0
5
20
60
Dose

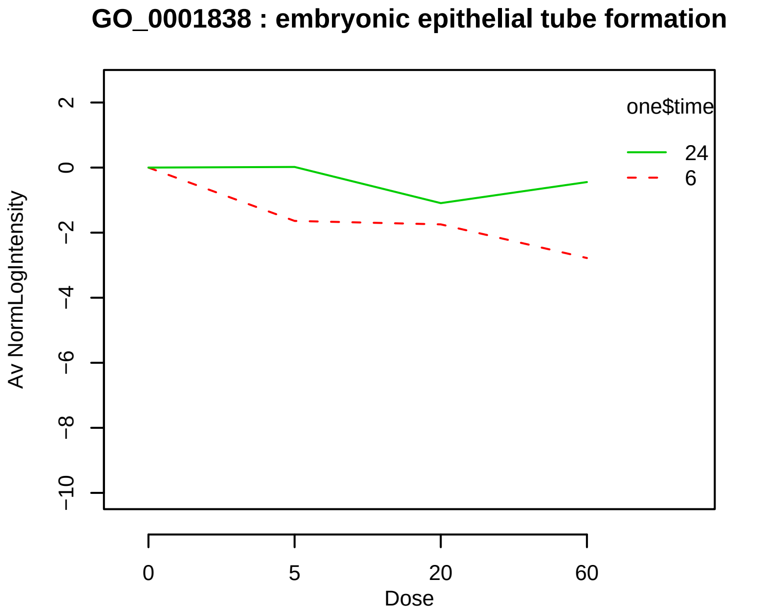
GO_0001838 : embryonic epithelial tube formation
one$time
24
6
Av NormLogIntensity
2
0
−2
−4
−6
−8
−10
0
5
20
60
Dose

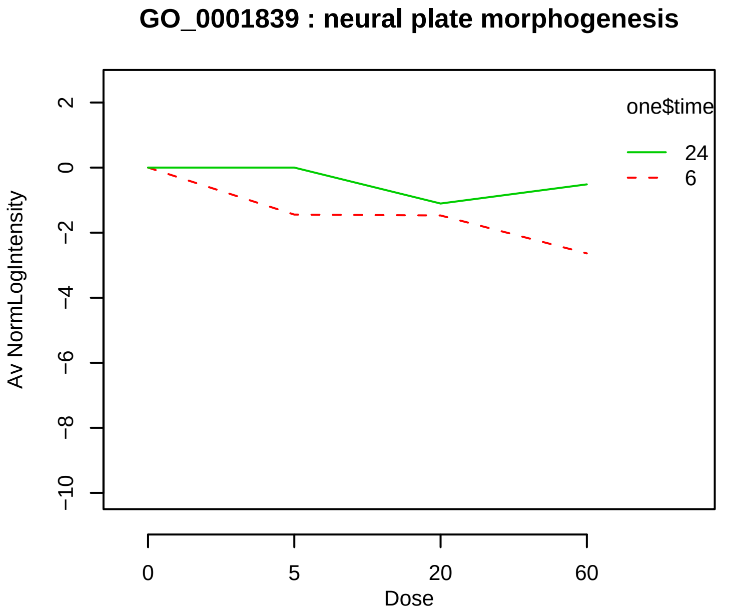
GO_0001839 : neural plate morphogenesis
one$time
24
6
Av NormLogIntensity
2
0
−2
−4
−6
−8
−10
0
5
20
60
Dose

GO_0001841 : neural tube formation

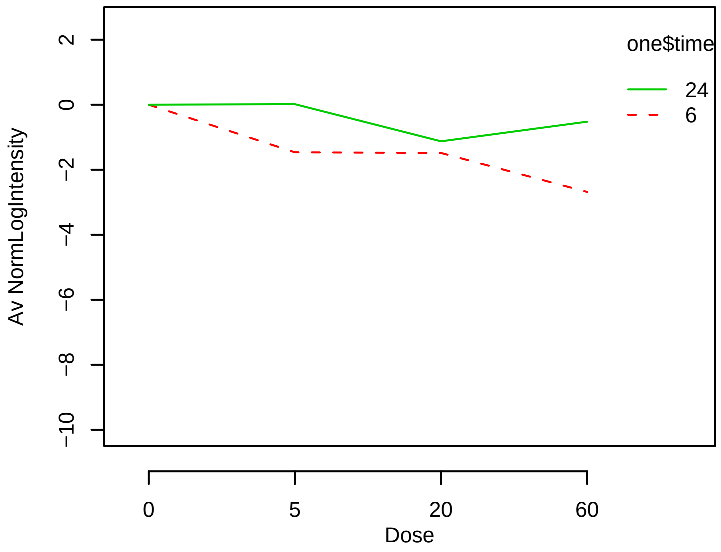

GO_0001843 : neural tube closure

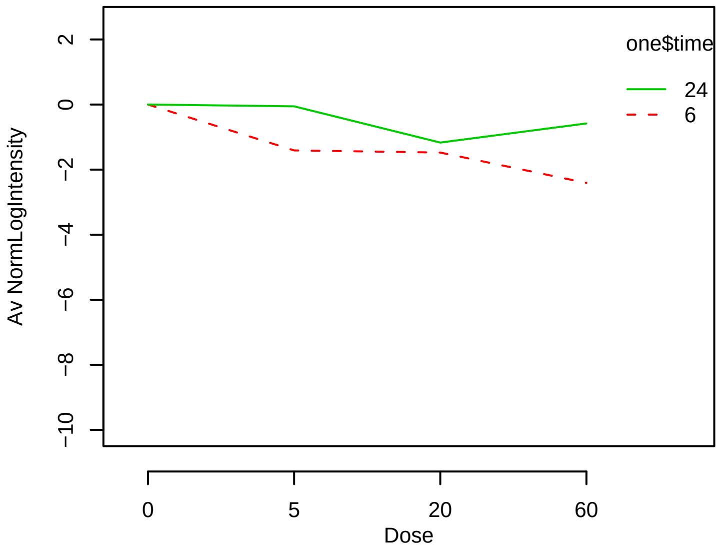

GO_0001889 : liver development

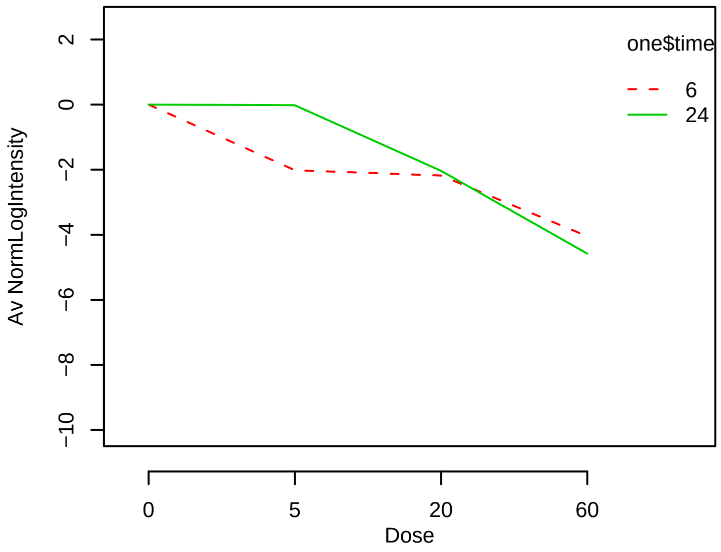

GO_0001890 : placenta development

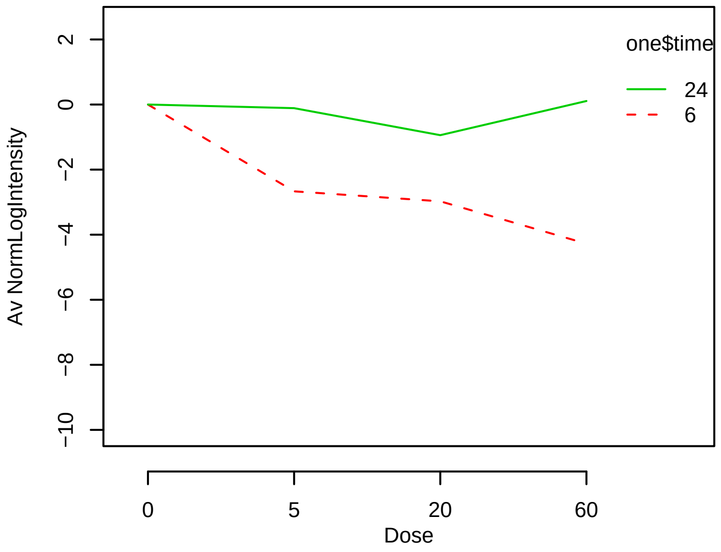

GO_0001892 : embryonic placenta development

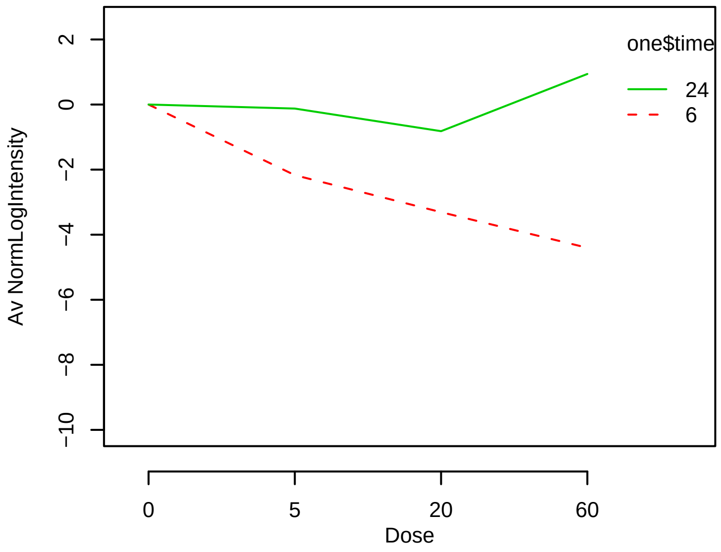

GO_0001894 : tissue homeostasis

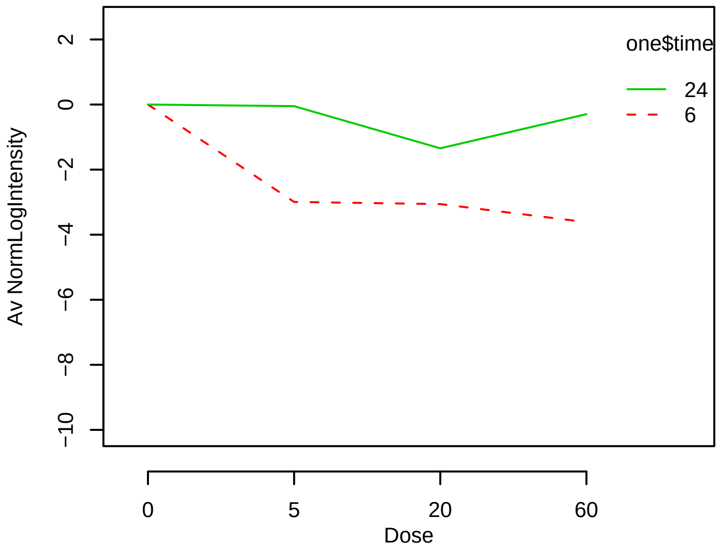

GO_0001906 : cell killing

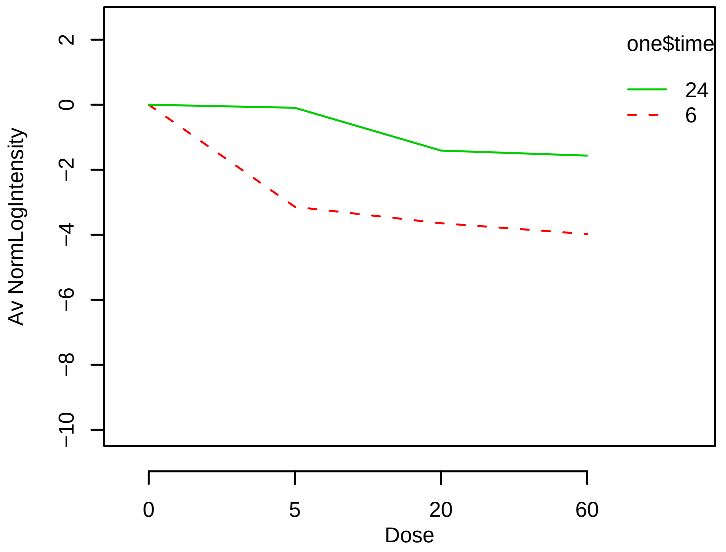

GO_0001909 : leukocyte mediated cytotoxicity

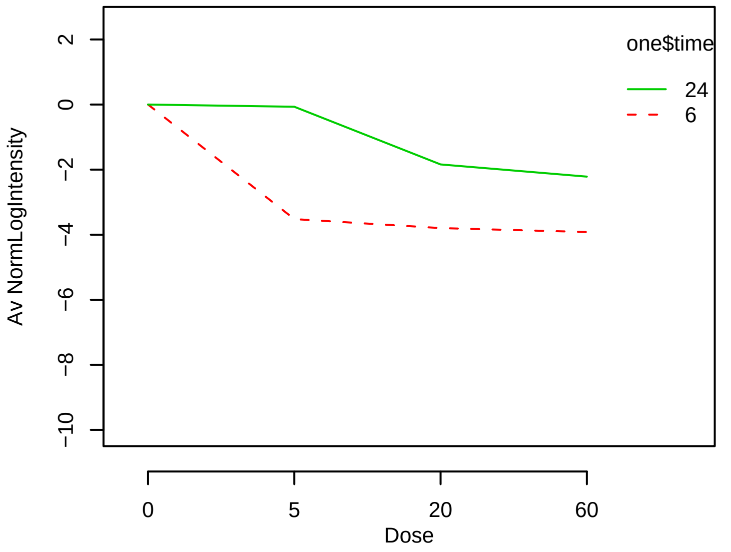

GO_0001932 : regulation of protein amino acid phosphoryla

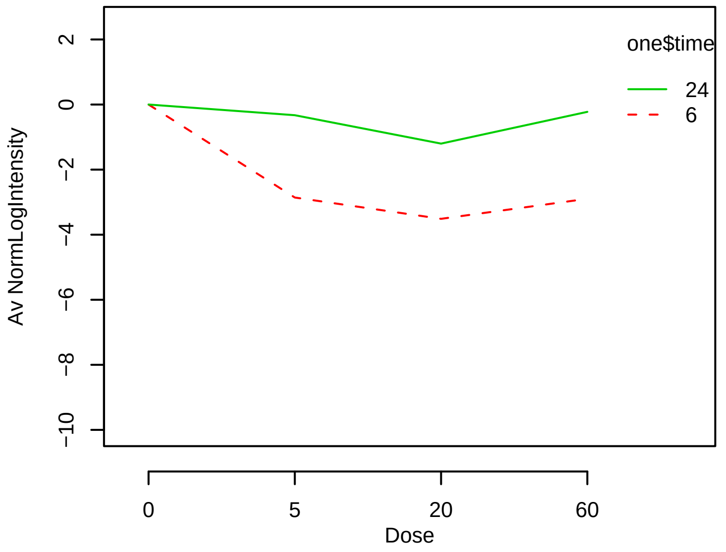

GO_0001933 : negative regulation of protein amino acid phos

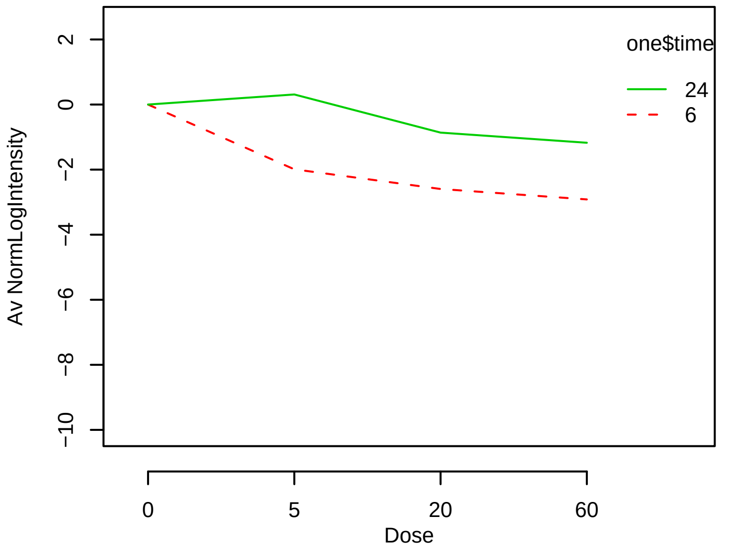

GO_0001934 : positive regulation of protein amino acid phos

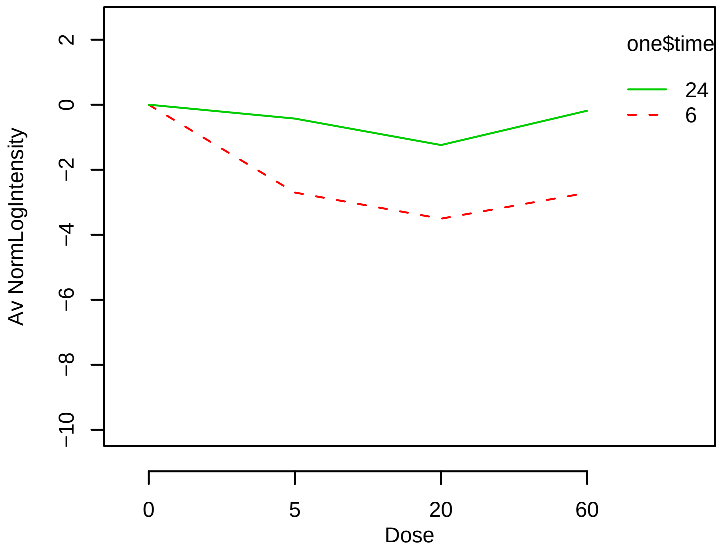

GO_0001935 : endothelial cell proliferation

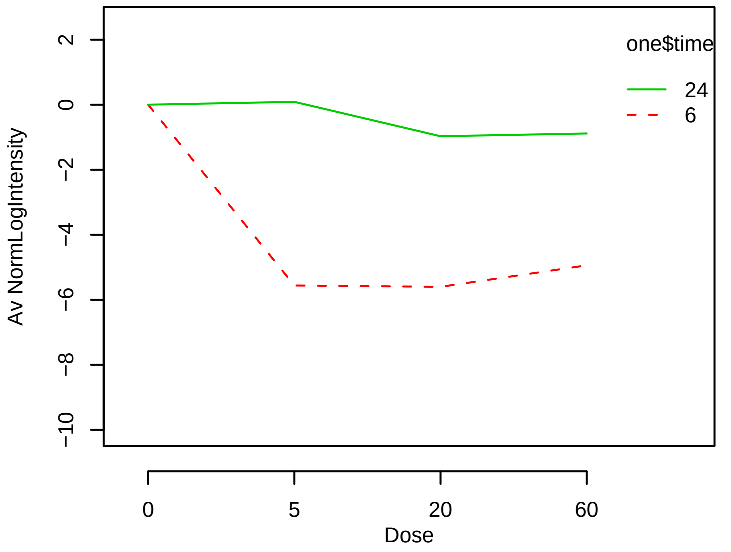

GO_0001936 : regulation of endothelial cell proliferation

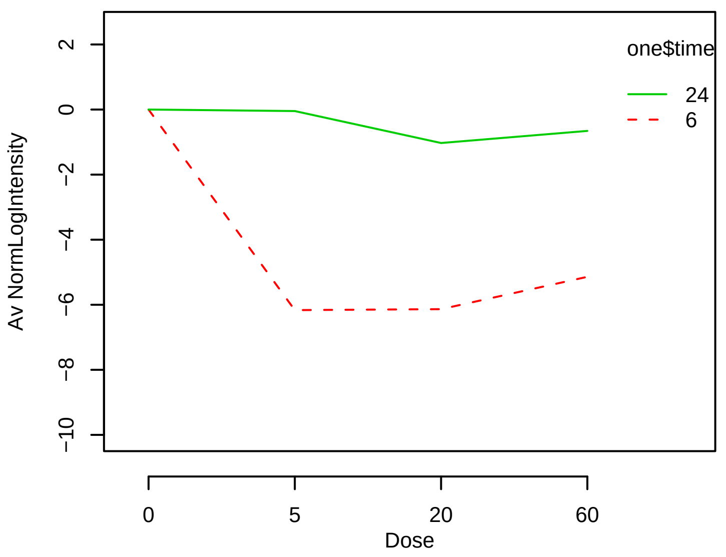

GO_0001937 : negative regulation of endothelial cell prolife

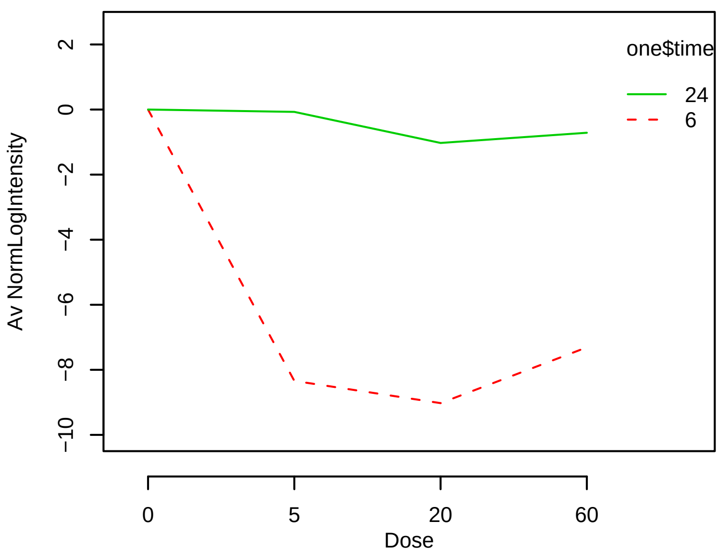

GO_0001942 : hair follicle development

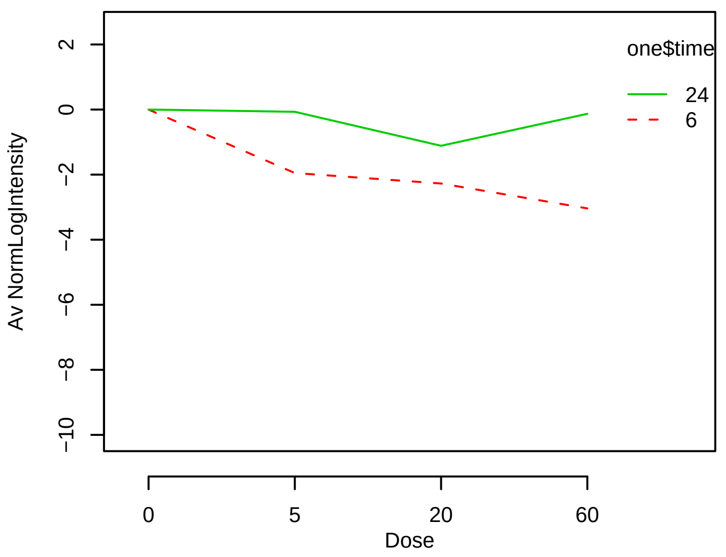

GO_0001944 : vasculature development

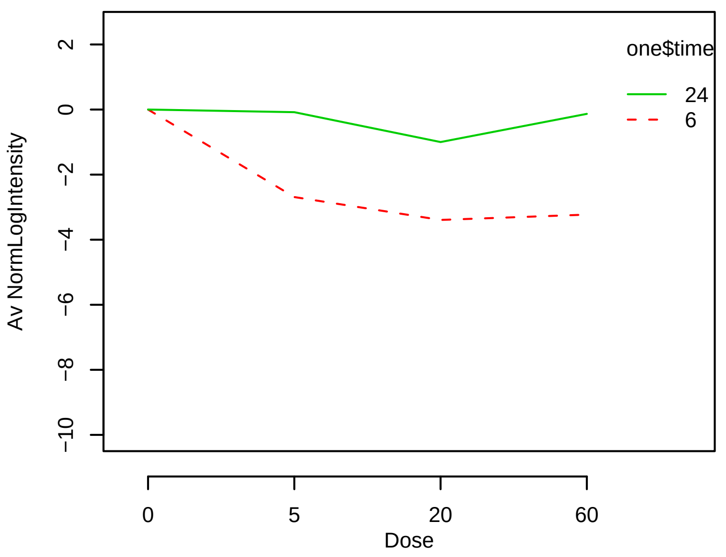

GO_0001947 : heart looping

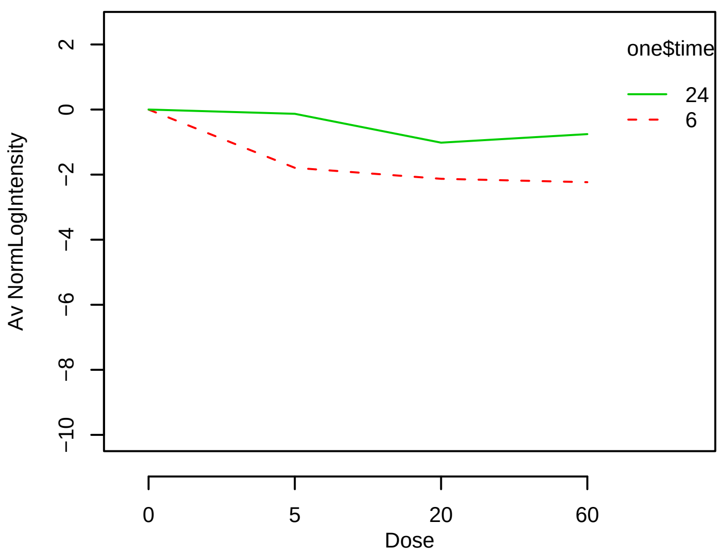

GO_0001952 : regulation of cell−matrix adhesion

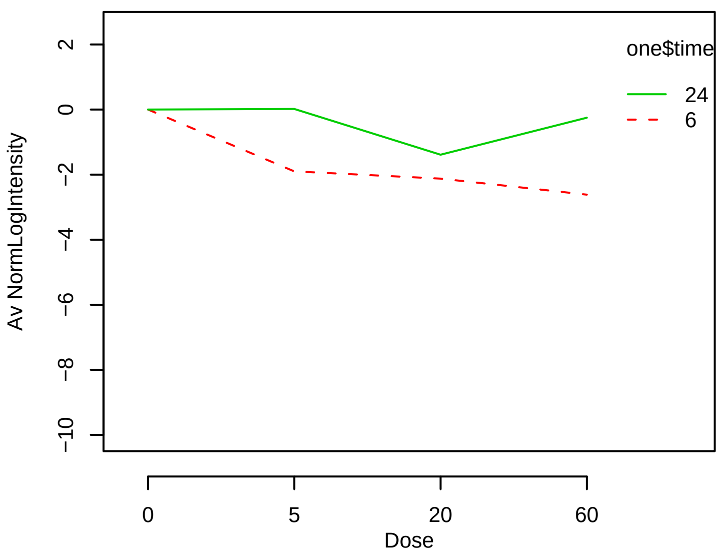

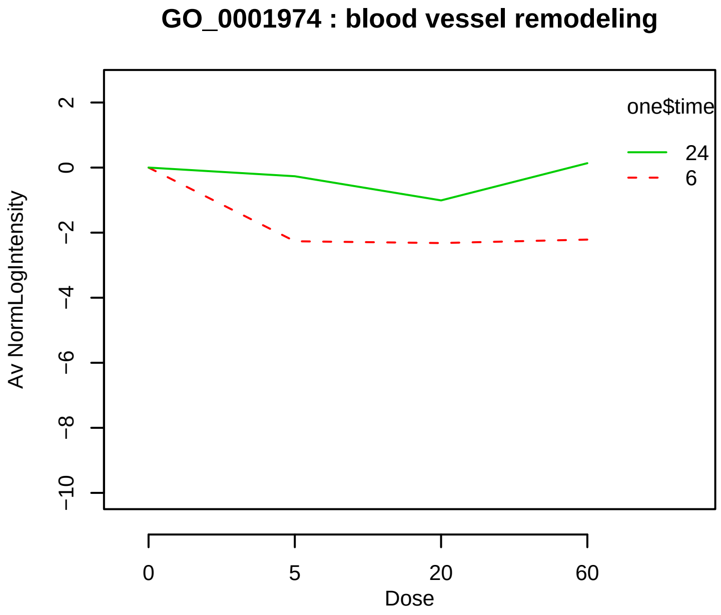
GO_0001974 : blood vessel remodeling
one$time
24
6
Av NormLogIntensity
Dose
0
5
20
60
2
0
−2
−4
−6
−8
−10

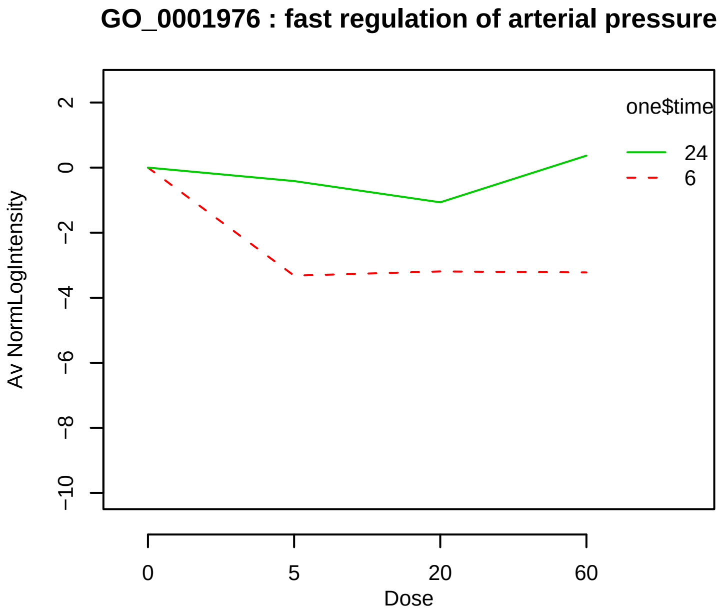
GO_0001976 : fast regulation of arterial pressure
one$time
24
6
Av NormLogIntensity
Dose
0
5
20
60
2
0
−2
−4
−6
−8
−10

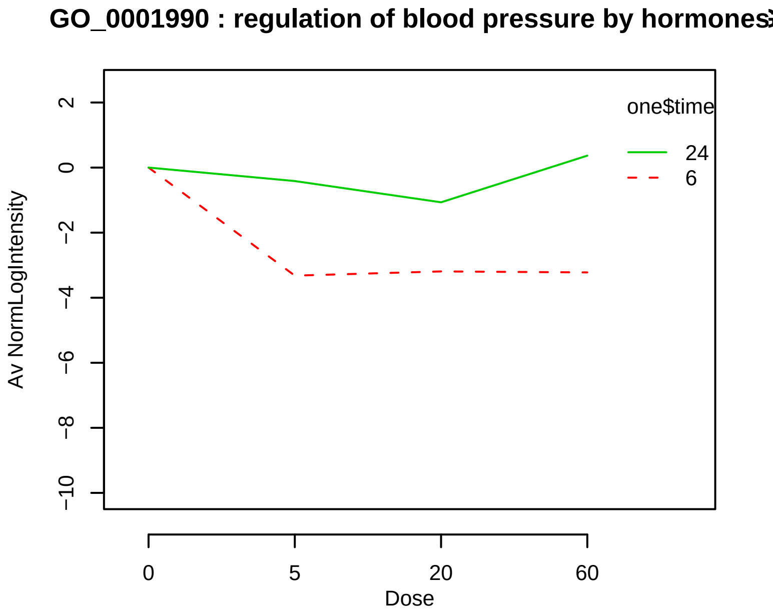
GO_0001990 : regulation of blood pressure by hormones
one$time
24
6
Av NormLogIntensity
Dose
0
5
20
60
2
0
−2
−4
−6
−8
−10

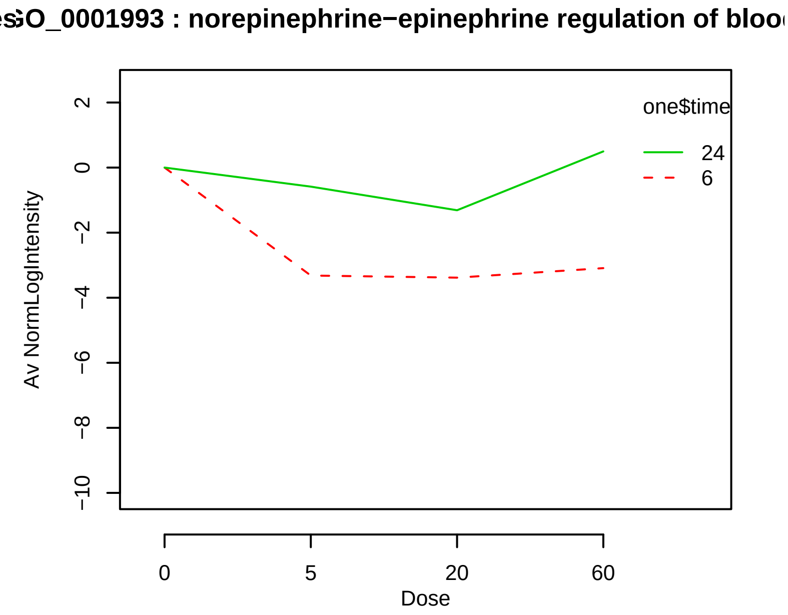
GO_0001993 : norepinephrine−epinephrine regulation of bloo
one$time
24
6
Av NormLogIntensity
Dose
0
5
20
60
2
0
−2
−4
−6
−8
−10

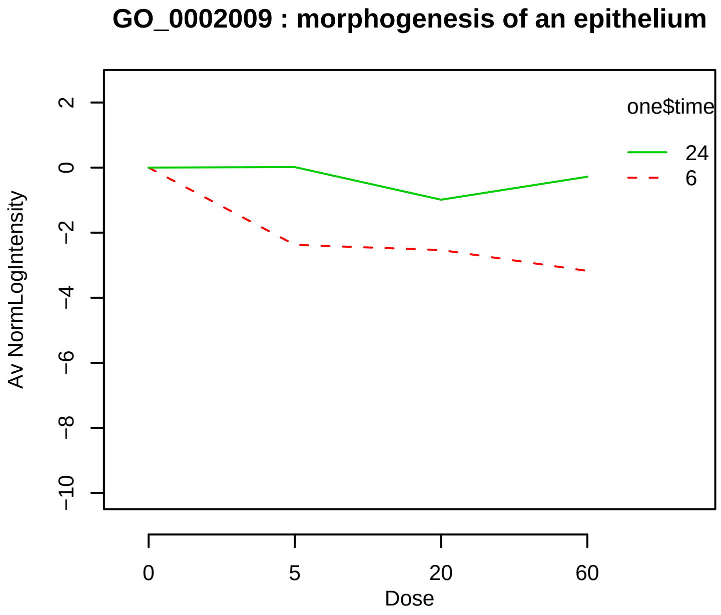
GO_0002009 : morphogenesis of an epithelium
one$time
24
6
Av NormLogIntensity
Dose
0
5
20
60
2
0
−2
−4
−6
−8
−10

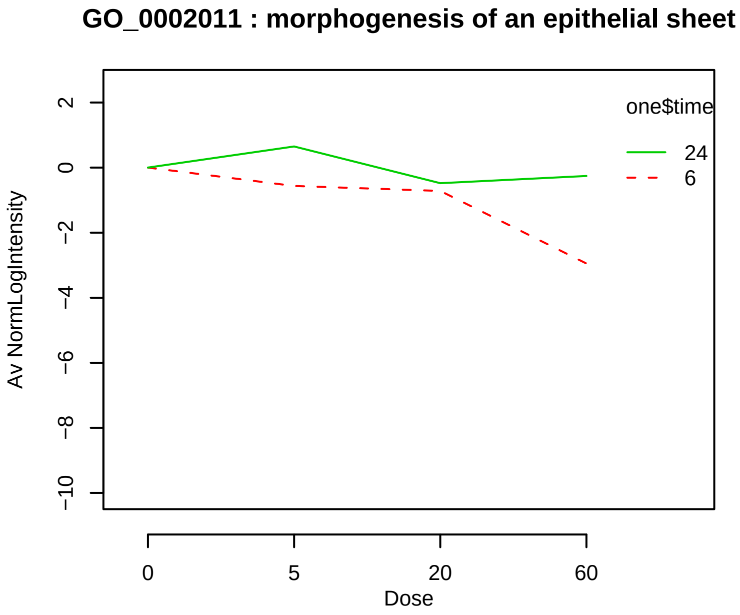
GO_0002011 : morphogenesis of an epithelial sheet
one$time
24
6
Av NormLogIntensity
Dose
0
5
20
60
2
0
−2
−4
−6
−8
−10

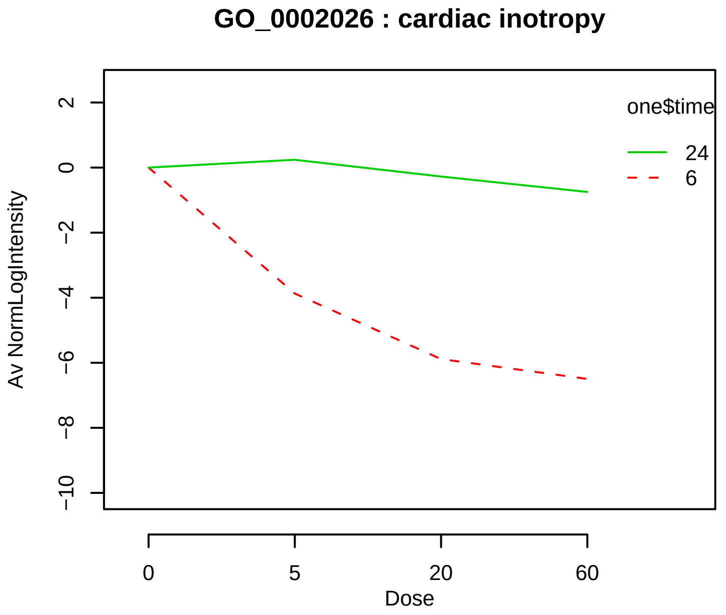
GO_0002026 : cardiac inotropy
Av NormLogIntensity
2
0
−2
−4
−6
−8
−10
0
5
20
60
Dose
one$time
24
6

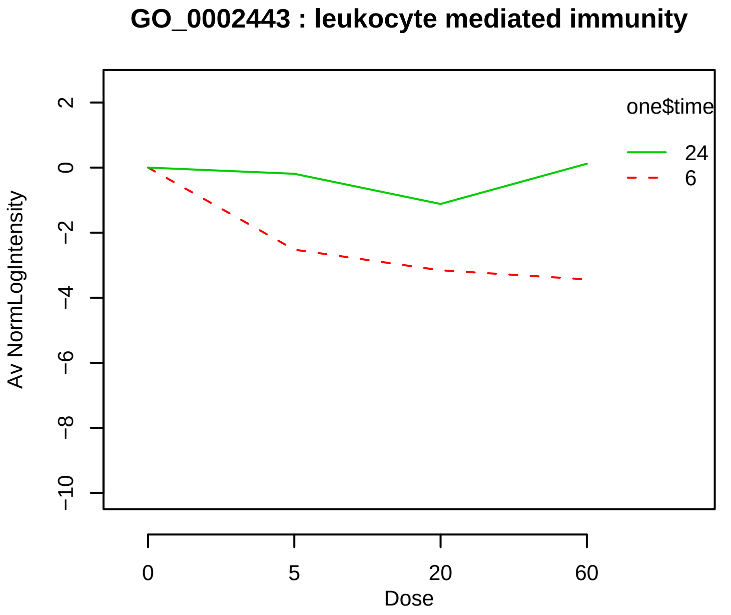
GO_0002443 : leukocyte mediated immunity
Av NormLogIntensity
2
0
−2
−4
−6
−8
−10
0
5
20
60
Dose
one$time
24
6

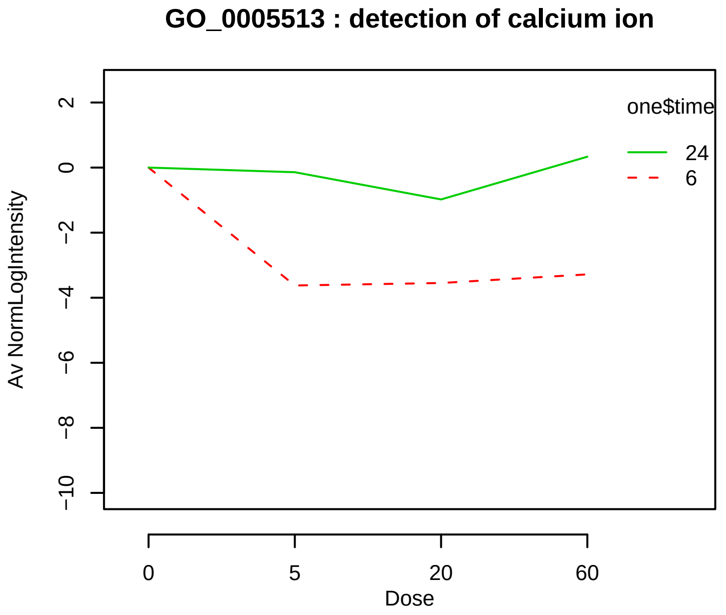
GO_0005513 : detection of calcium ion
Av NormLogIntensity
2
0
−2
−4
−6
−8
−10
0
5
20
60
Dose
one$time
24
6

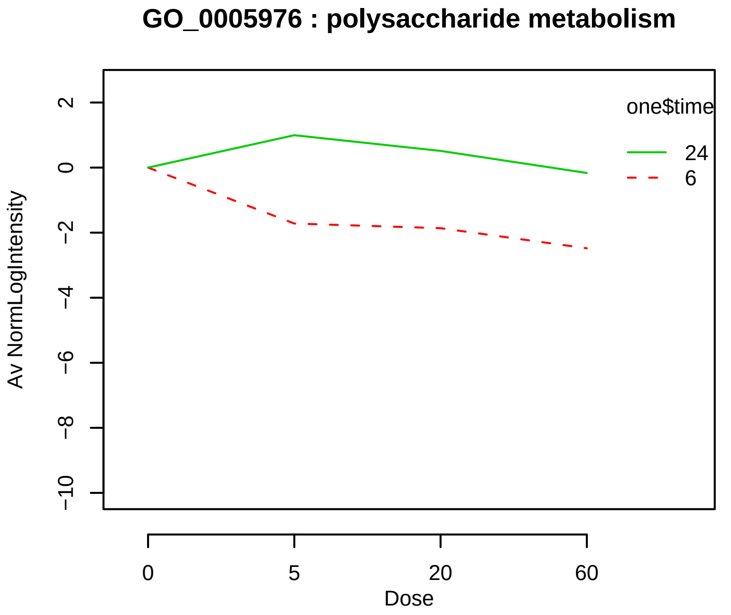
GO_0005976 : polysaccharide metabolism
Av NormLogIntensity
2
0
−2
−4
−6
−8
−10
0
5
20
60
Dose
one$time
24
6

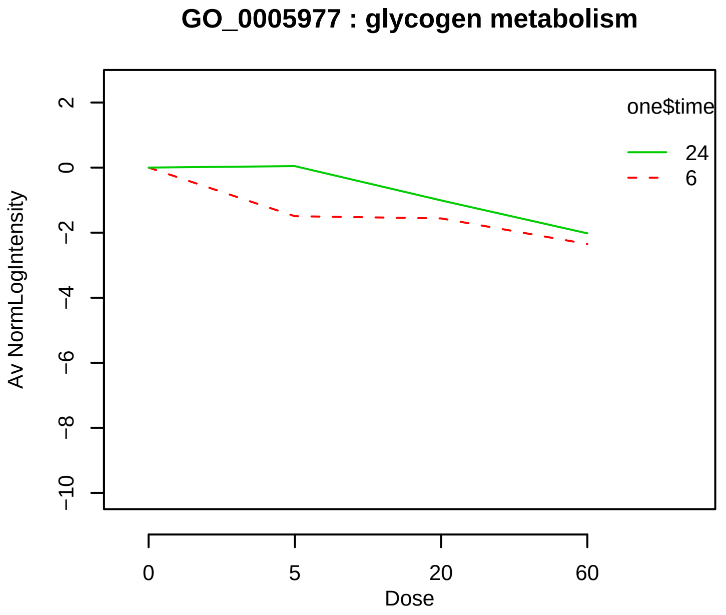
GO_0005977 : glycogen metabolism
Av NormLogIntensity
2
0
−2
−4
−6
−8
−10
0
5
20
60
Dose
one$time
24
6

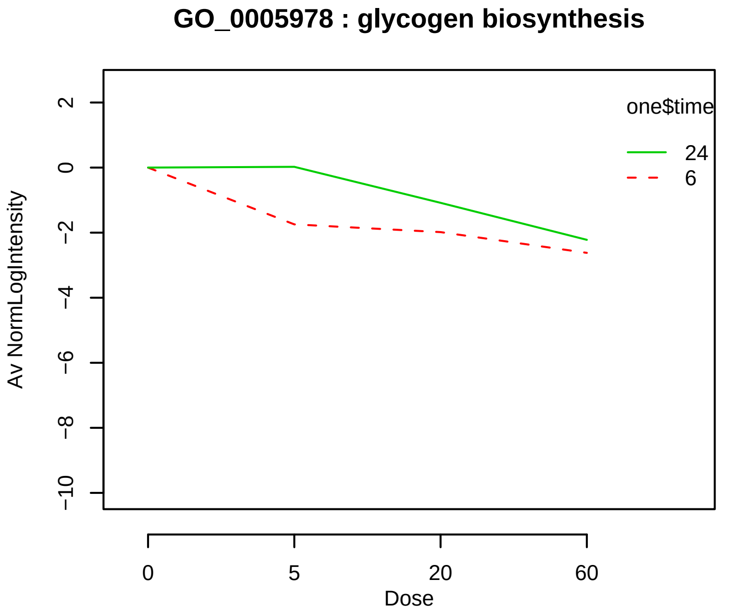
GO_0005978 : glycogen biosynthesis
Av NormLogIntensity
2
0
−2
−4
−6
−8
−10
0
5
20
60
Dose
one$time
24
6

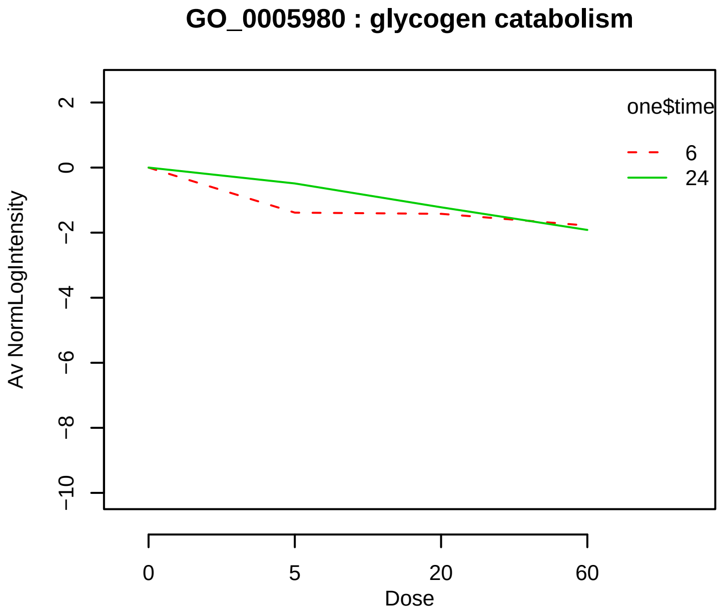
GO_0005980 : glycogen catabolism
Av NormLogIntensity
2
0
−2
−4
−6
−8
−10
one$time
6
24
0
5
20
60
Dose

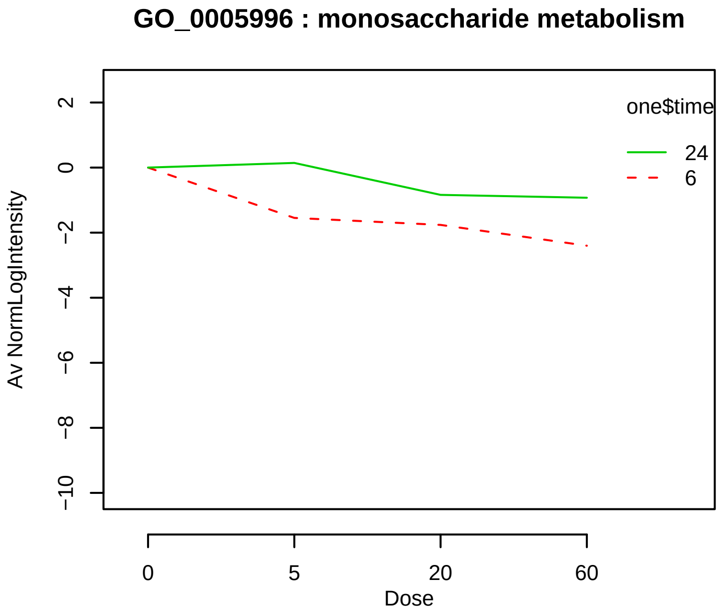
GO_0005996 : monosaccharide metabolism
Av NormLogIntensity
2
0
−2
−4
−6
−8
−10
one$time
24
6
0
5
20
60
Dose

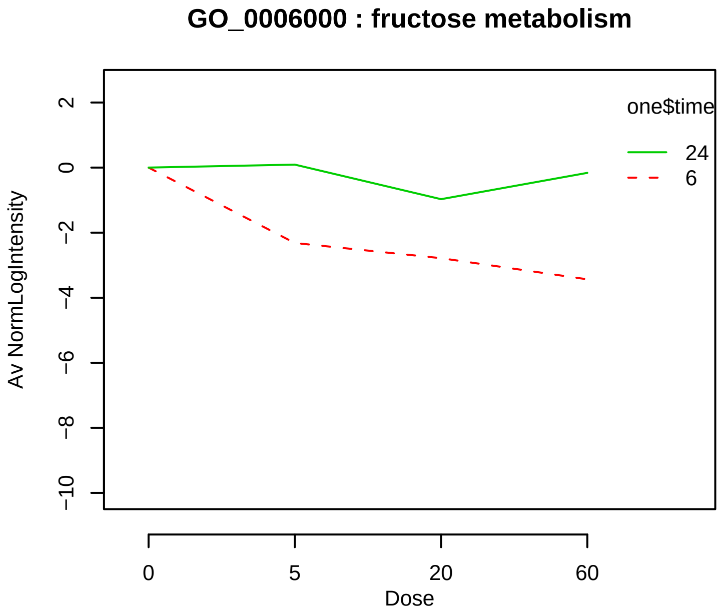
GO_0006000 : fructose metabolism
Av NormLogIntensity
2
0
−2
−4
−6
−8
−10
one$time
24
6
0
5
20
60
Dose

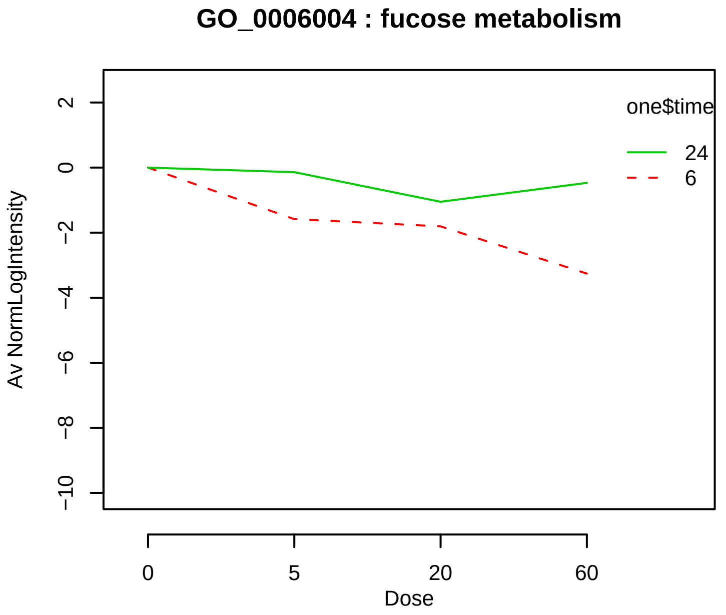
GO_0006004 : fucose metabolism
Av NormLogIntensity
2
0
−2
−4
−6
−8
−10
one$time
24
6
0
5
20
60
Dose

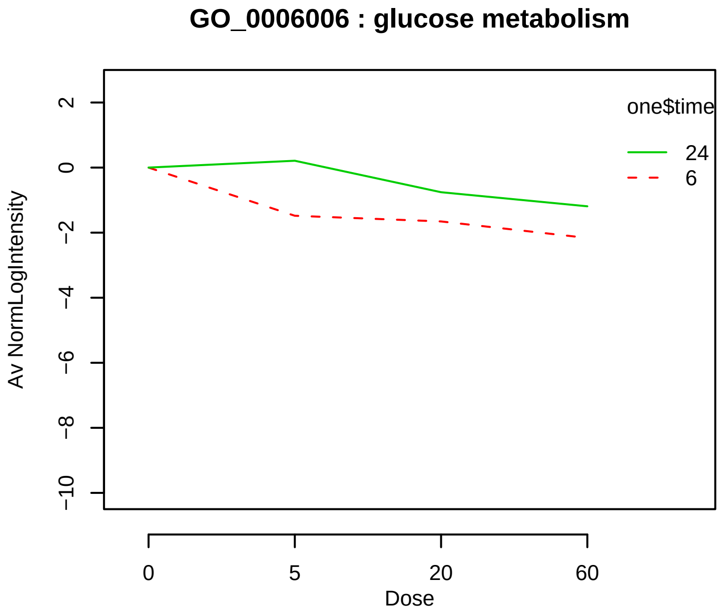
GO_0006006 : glucose metabolism
Av NormLogIntensity
2
0
−2
−4
−6
−8
−10
one$time
24
6
0
5
20
60
Dose

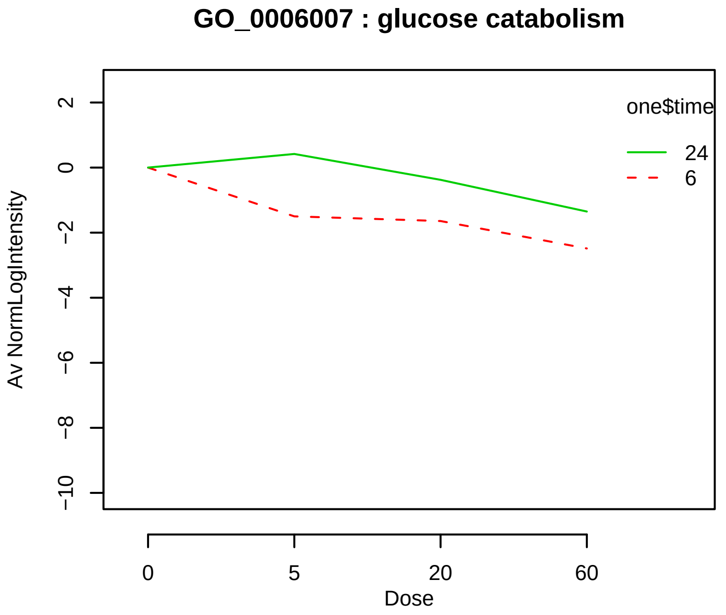
GO_0006007 : glucose catabolism
Av NormLogIntensity
2
0
−2
−4
−6
−8
−10
one$time
24
6
0
5
20
60
Dose

GO_0006012 : galactose metabolism

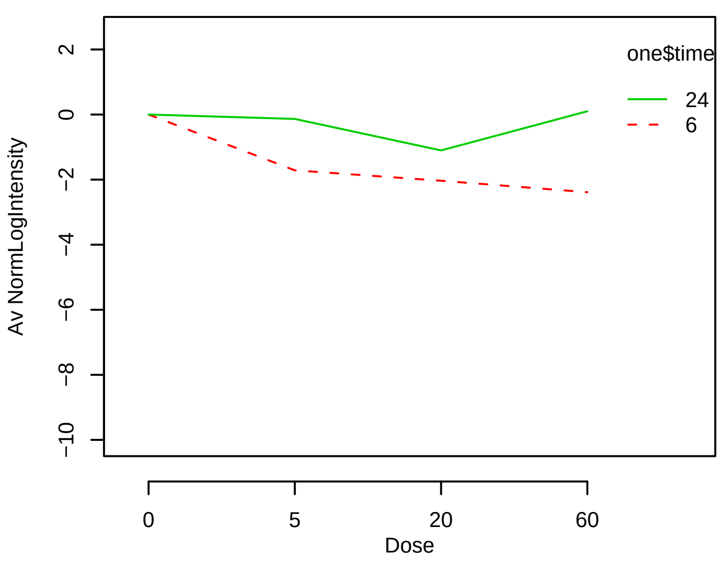

GO_0006013 : mannose metabolism

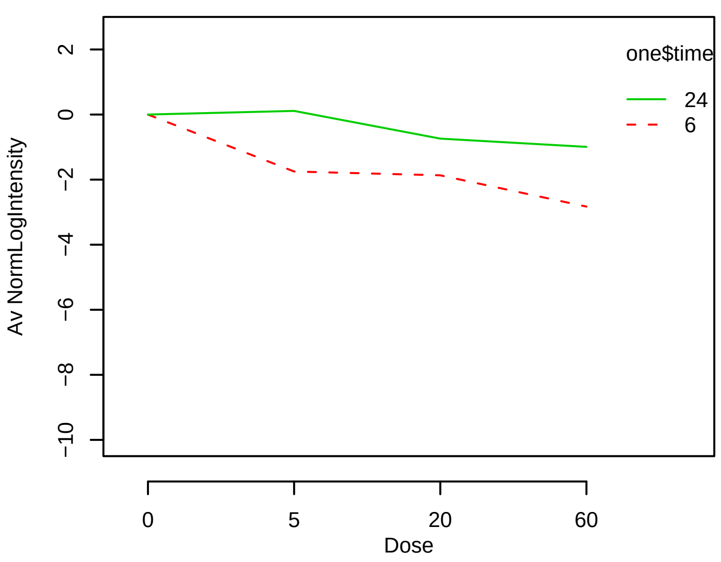

GO_0006020 : myo−inositol metabolism

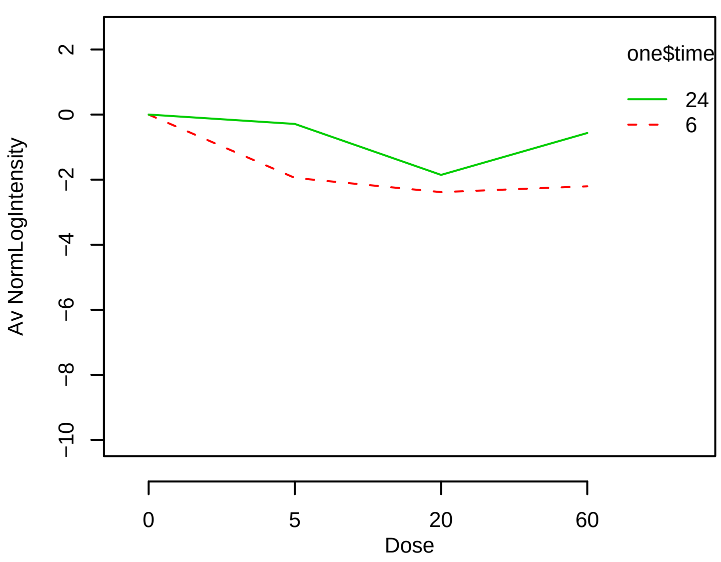

GO_0006022 : aminoglycan metabolism

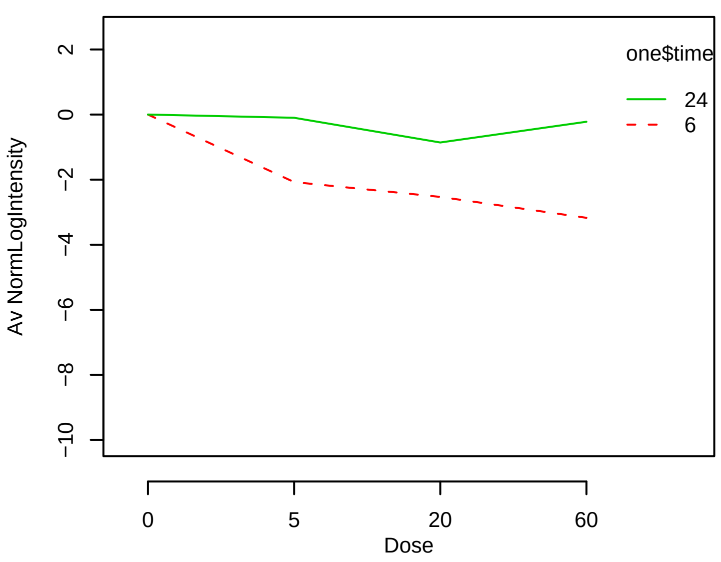

GO_0006023 : aminoglycan biosynthesis

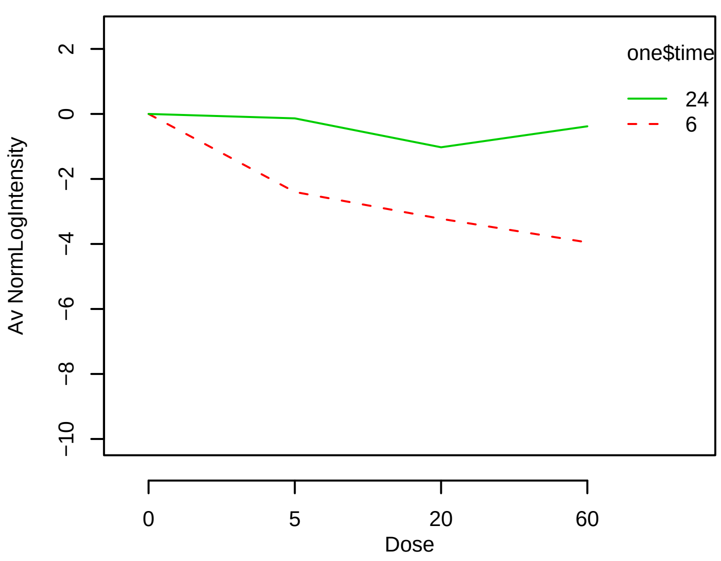

GO_0006024 : glycosaminoglycan biosynthesis

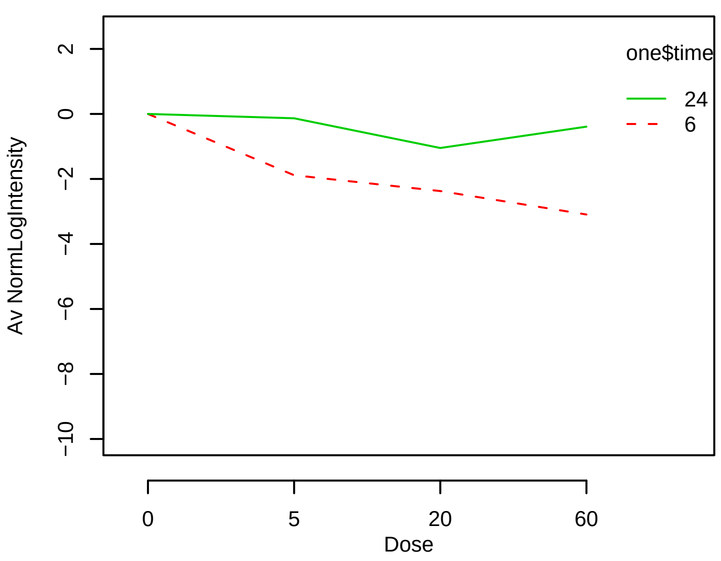

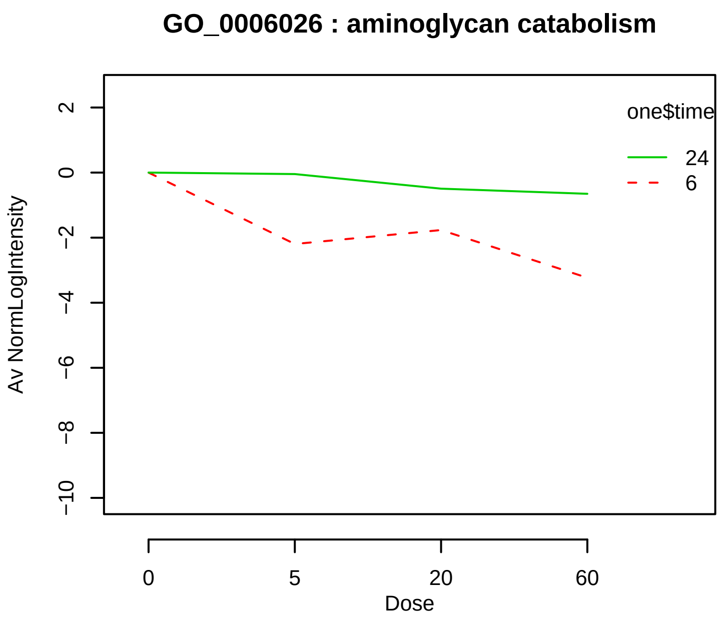
GO_0006026 : aminoglycan catabolism
one$time
24
6
Av NormLogIntensity
2
0
−2
−4
−6
−8
−10
0
5
20
60
Dose

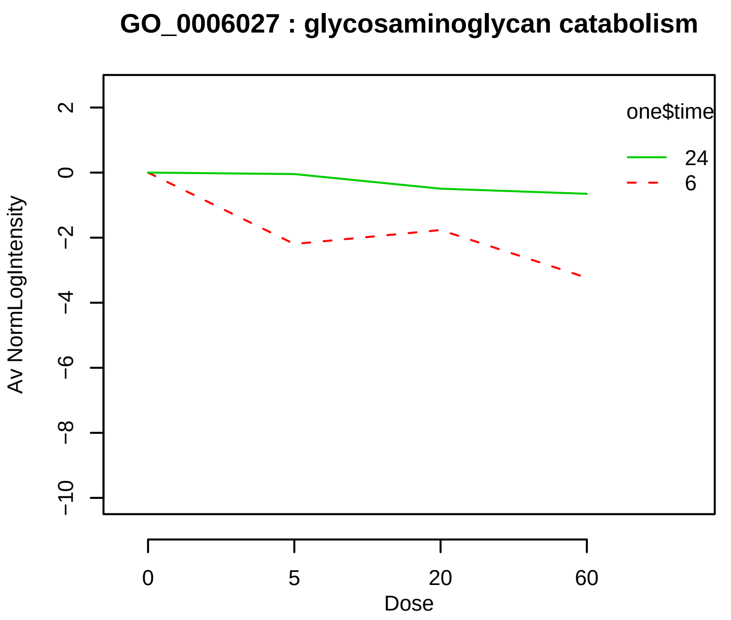
GO_0006027 : glycosaminoglycan catabolism
one$time
24
6
Av NormLogIntensity
2
0
−2
−4
−6
−8
−10
0
5
20
60
Dose

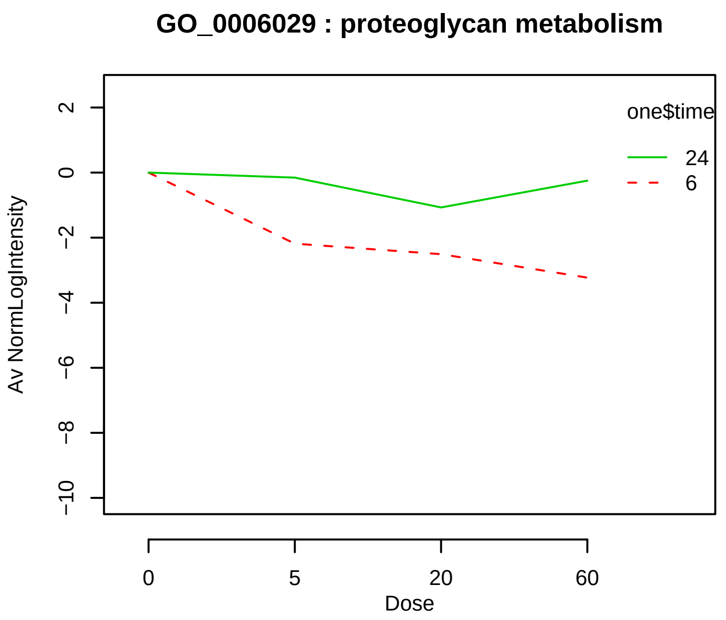
GO_0006029 : proteoglycan metabolism
one$time
24
6
Av NormLogIntensity
2
0
−2
−4
−6
−8
−10
0
5
20
60
Dose

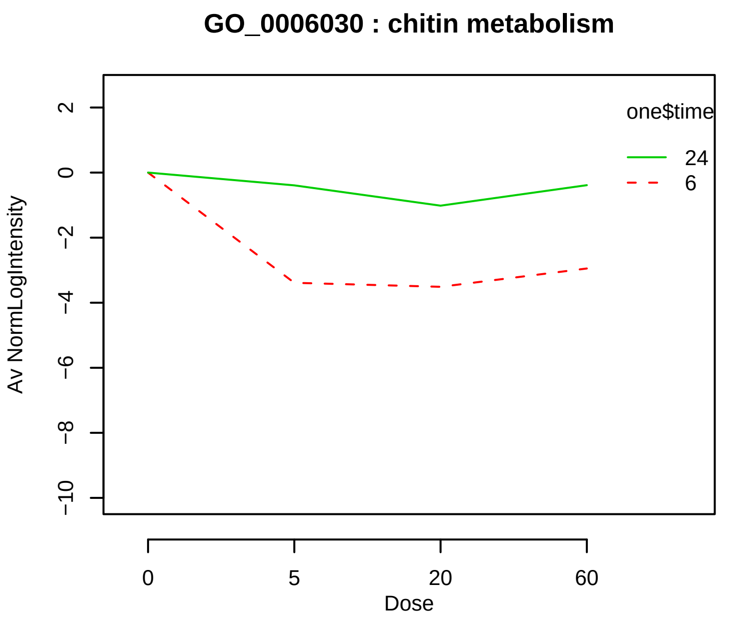
GO_0006030 : chitin metabolism
one$time
24
6
Av NormLogIntensity
2
0
−2
−4
−6
−8
−10
0
5
20
60
Dose

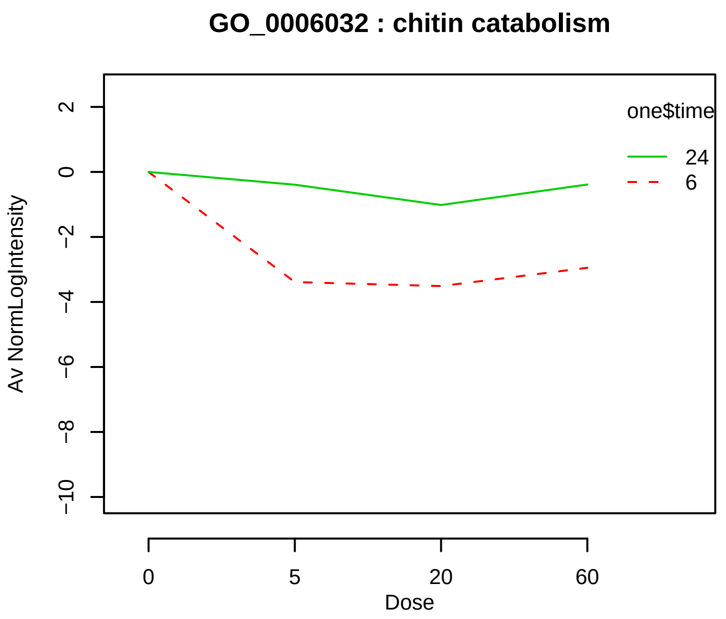
GO_0006032 : chitin catabolism
one$time
24
6
Av NormLogIntensity
2
0
−2
−4
−6
−8
−10
0
5
20
60
Dose

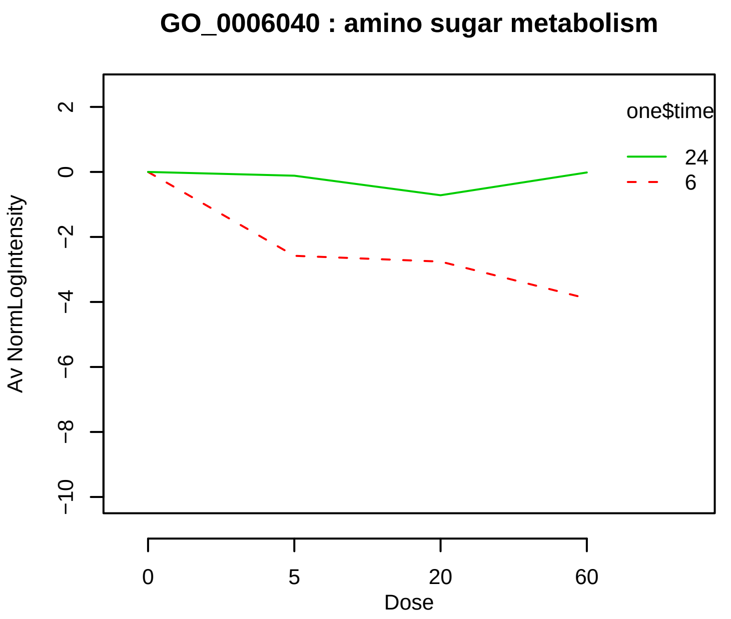
GO_0006040 : amino sugar metabolism
one$time
24
6
Av NormLogIntensity
2
0
−2
−4
−6
−8
−10
0
5
20
60
Dose

GO_0006041 : glucosamine metabolism

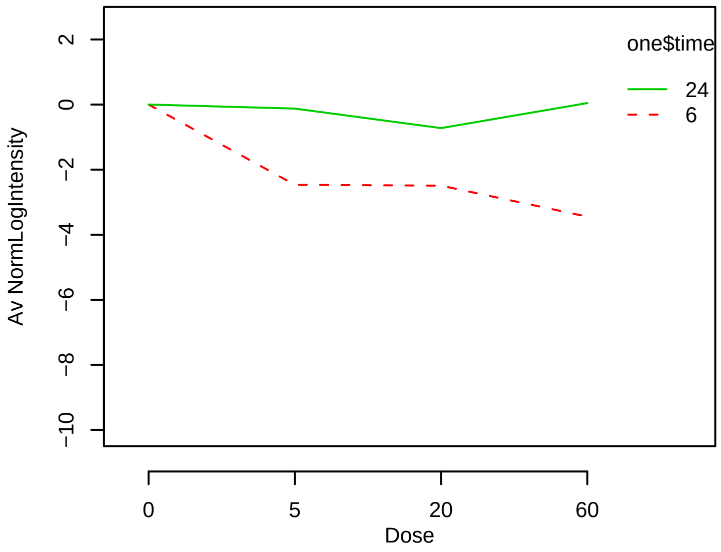

GO_0006043 : glucosamine catabolism

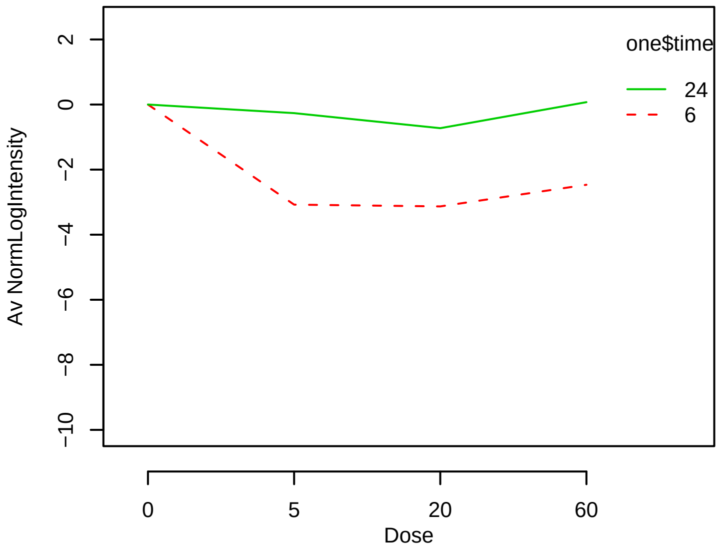

GO_0006044 : N−acetylglucosamine metabolism

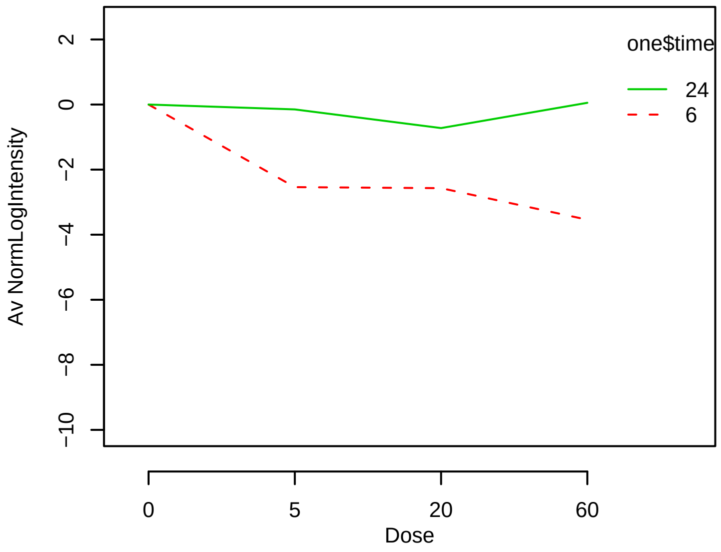

GO_0006046 : N−acetylglucosamine catabolism

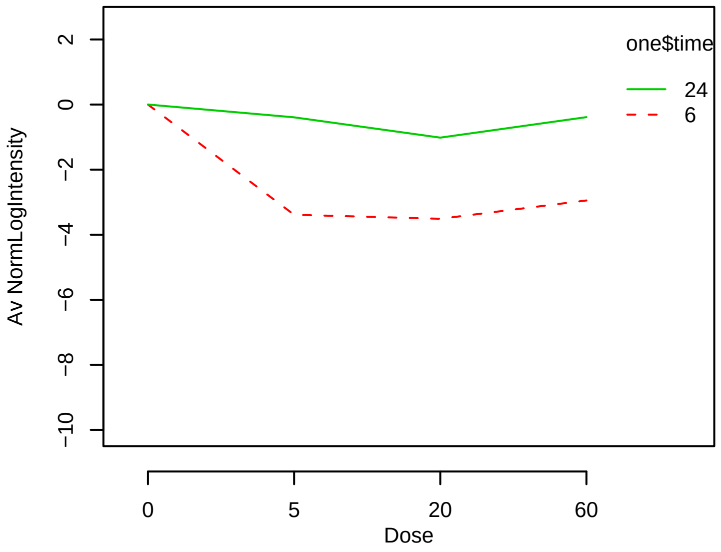

GO_0006067 : ethanol metabolism

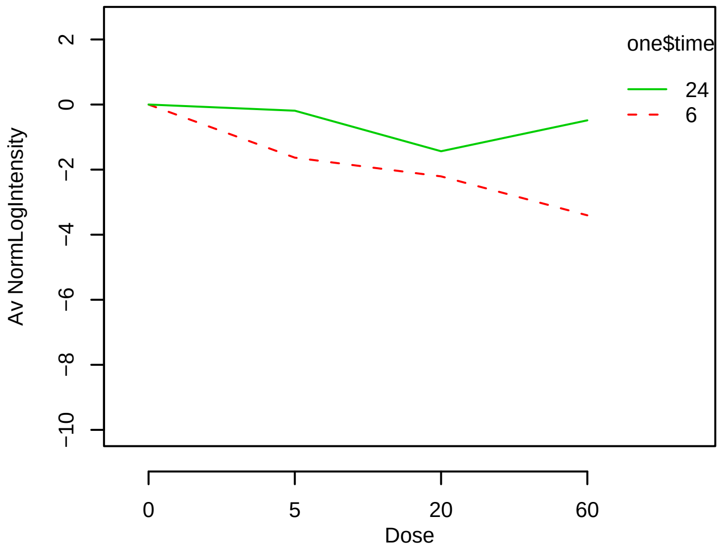

GO_0006069 : ethanol oxidation

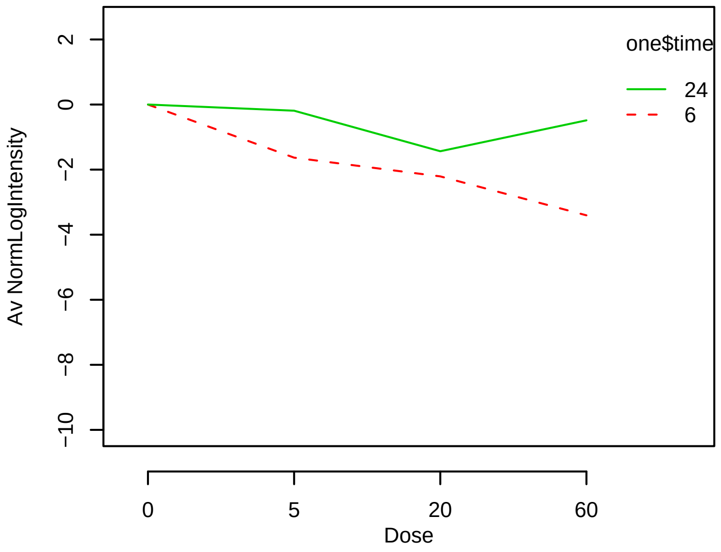

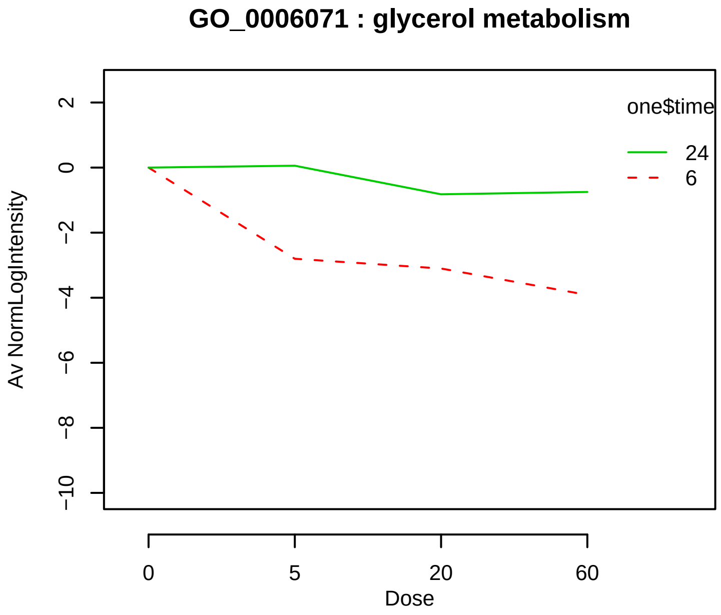
GO_0006071 : glycerol metabolism
one$time
24
6
Av NormLogIntensity
2
0
−2
−4
−6
−8
−10
0
5
20
60
Dose

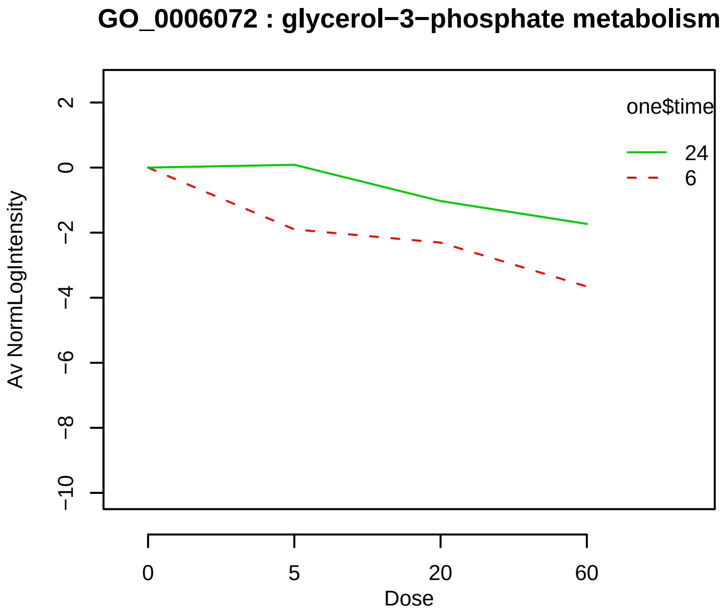
GO_0006072 : glycerol−3−phosphate metabolism
one$time
24
6
Av NormLogIntensity
2
0
−2
−4
−6
−8
−10
0
5
20
60
Dose

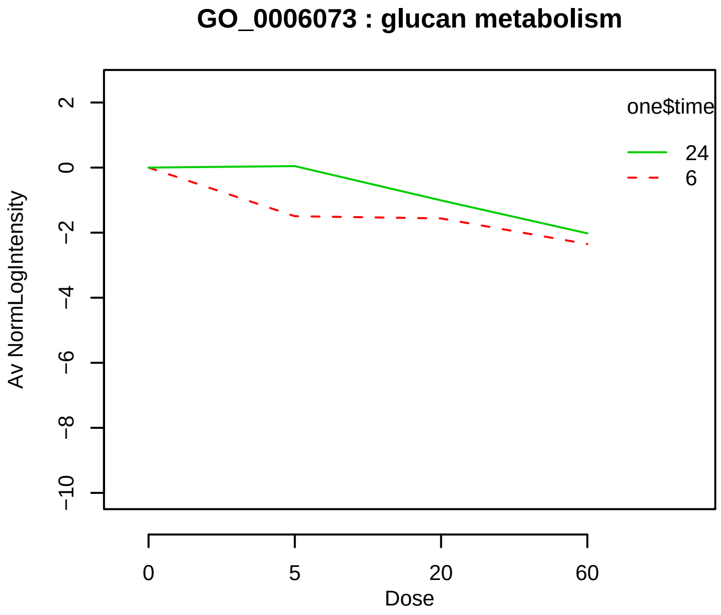
GO_0006073 : glucan metabolism
one$time
24
6
Av NormLogIntensity
2
0
−2
−4
−6
−8
−10
0
5
20
60
Dose

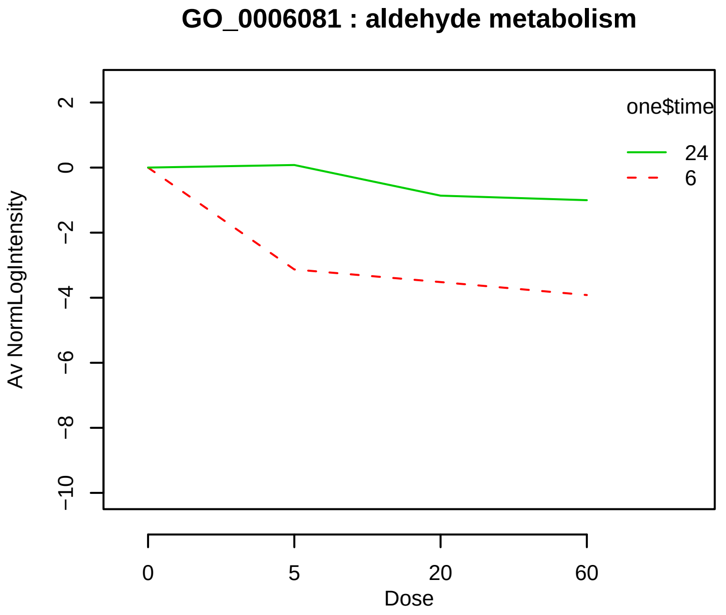
GO_0006081 : aldehyde metabolism
one$time
24
6
Av NormLogIntensity
2
0
−2
−4
−6
−8
−10
0
5
20
60
Dose

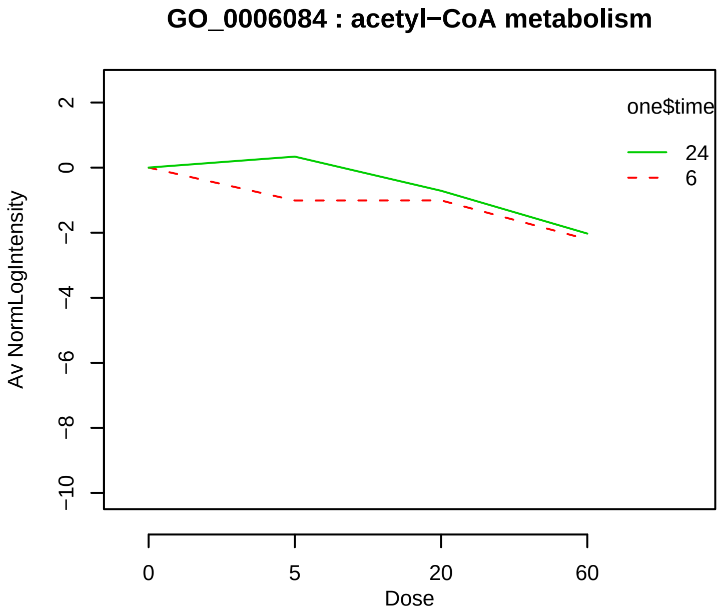
GO_0006084 : acetyl−CoA metabolism
one$time
24
6
Av NormLogIntensity
2
0
−2
−4
−6
−8
−10
0
5
20
60
Dose

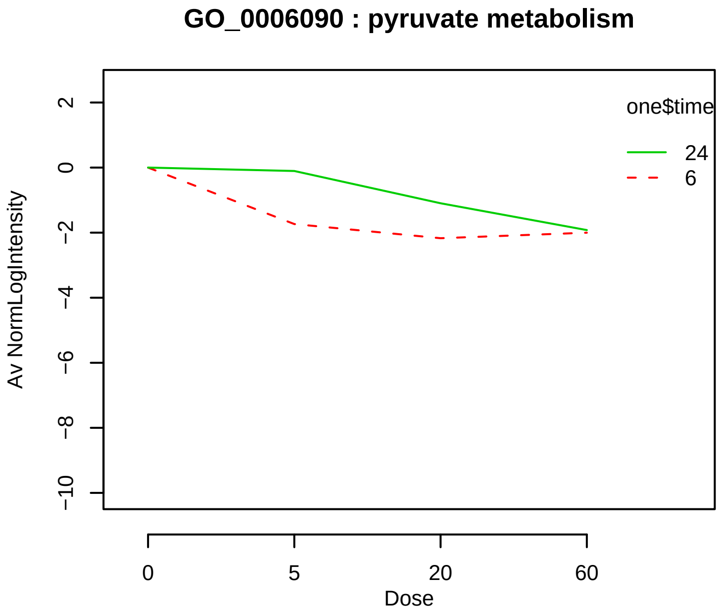
GO_0006090 : pyruvate metabolism
one$time
24
6
Av NormLogIntensity
2
0
−2
−4
−6
−8
−10
0
5
20
60
Dose

GO_0006092 : main pathways of carbohydrate metabolism

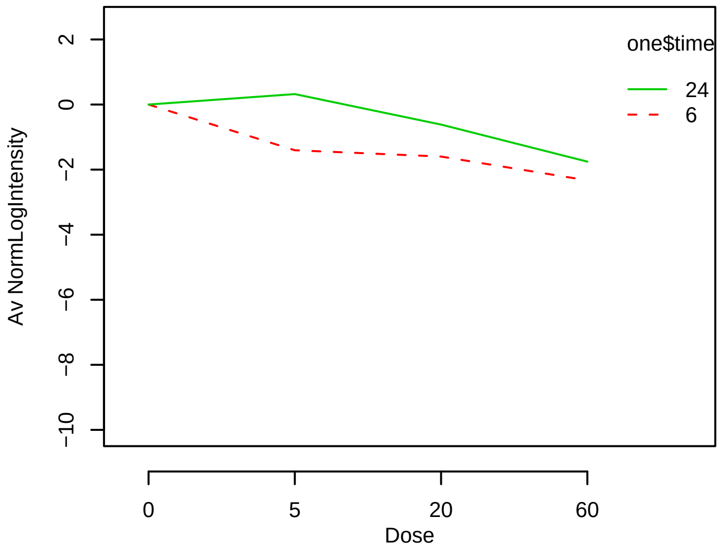

GO_0006094 : gluconeogenesis

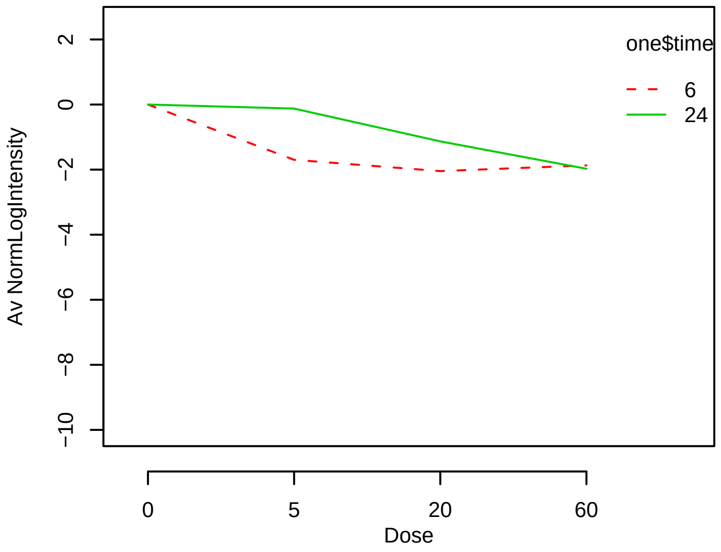

GO_0006096 : glycolysis

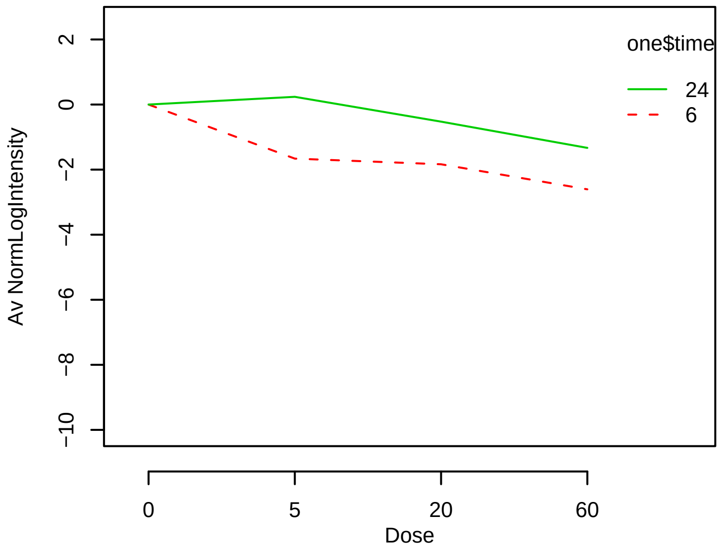

GO_0006098 : pentose−phosphate shunt

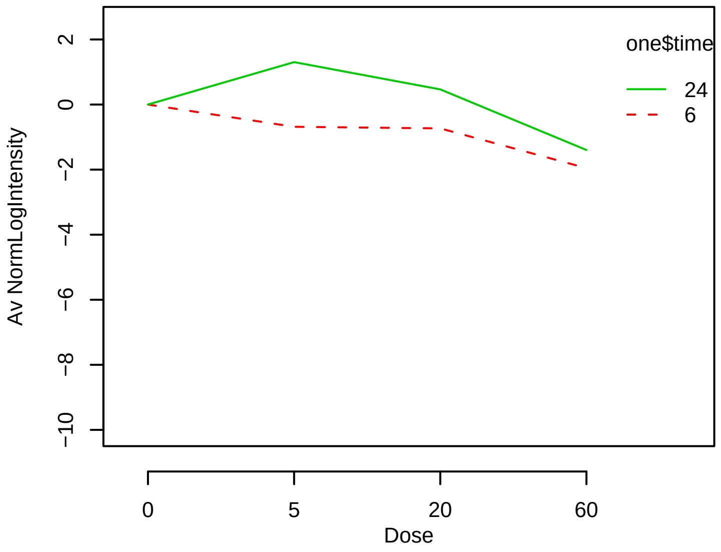

GO_0006099 : tricarboxylic acid cycle

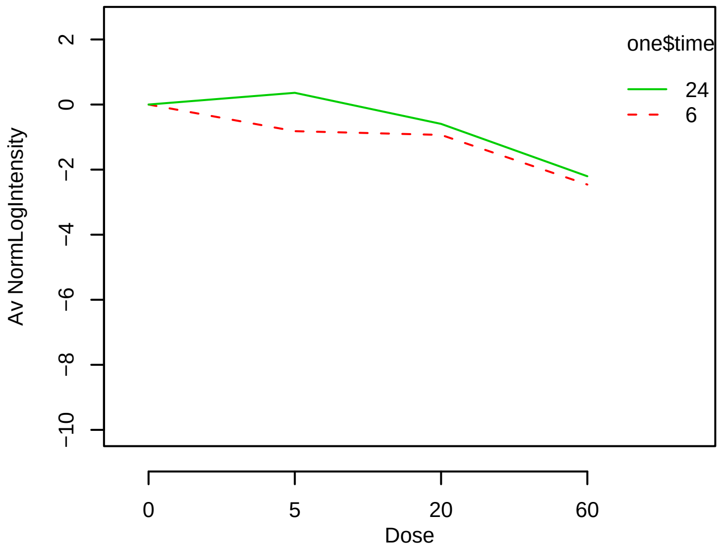

GO_0006100 : tricarboxylic acid cycle intermediate metaboli

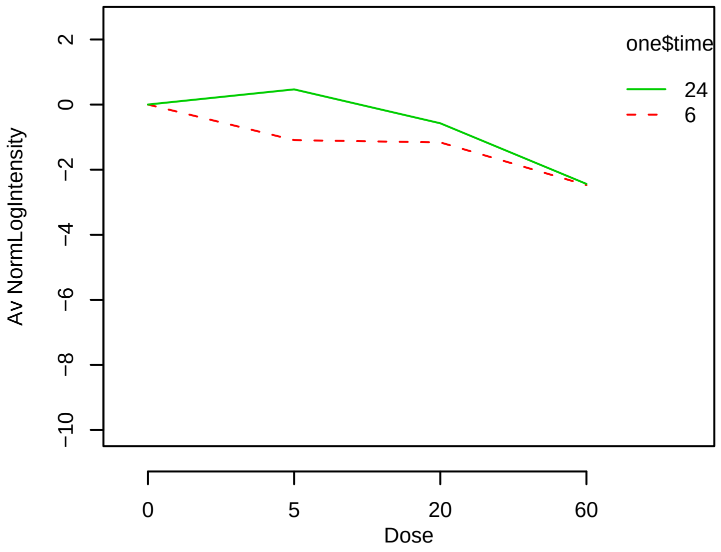

GO_0006108 : malate metabolism

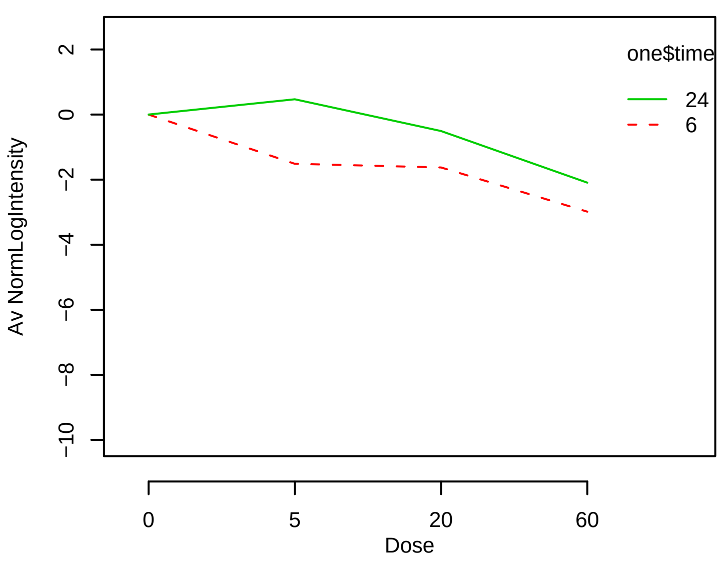

GO_0006109 : regulation of carbohydrate metabolism

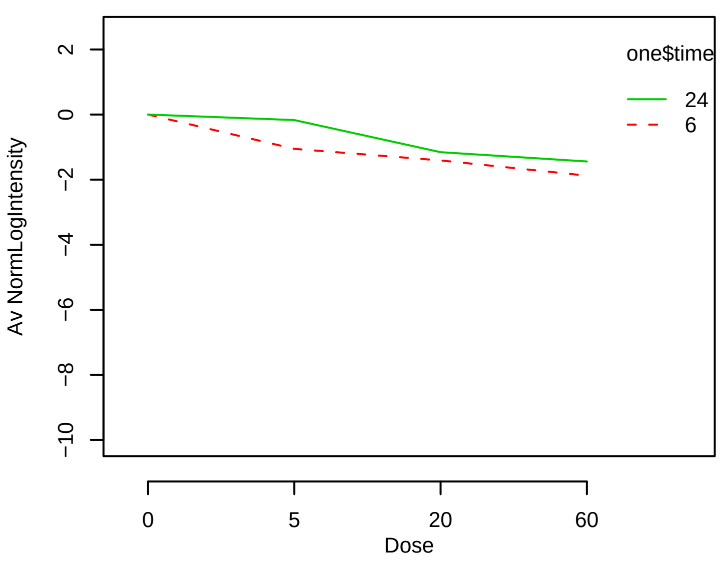

GO_0006112 : energy reserve metabolism

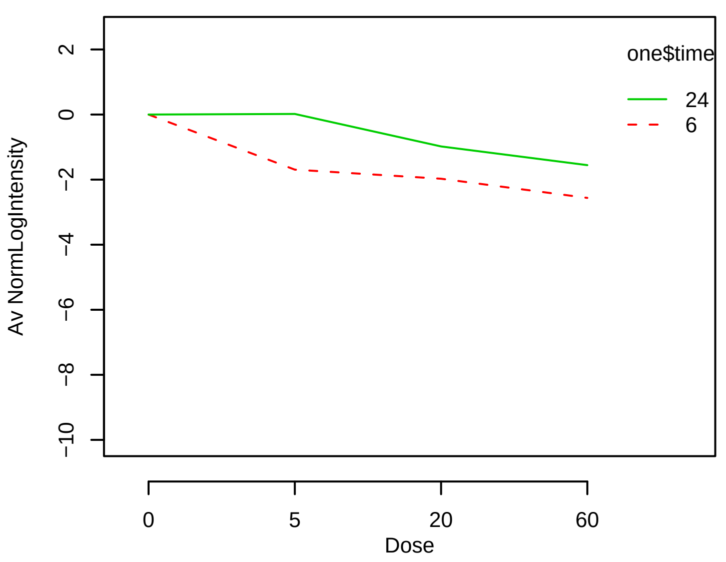

GO_0006119 : oxidative phosphorylation

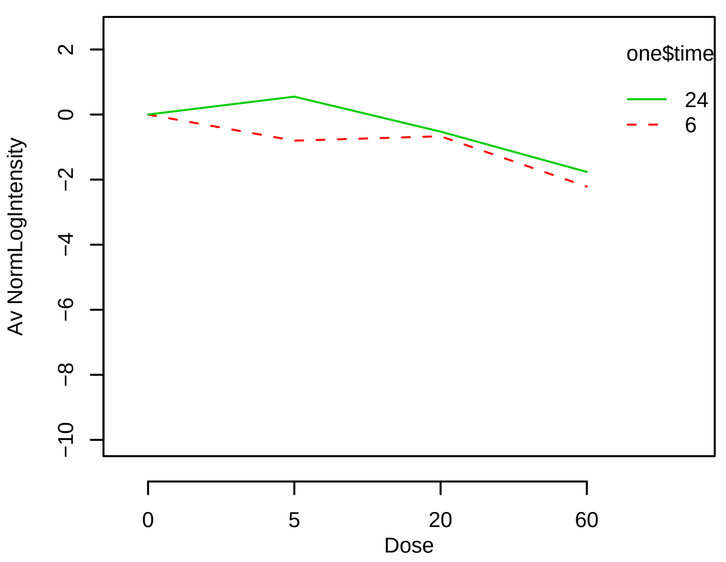

GO_0006120 : mitochondrial electron transport\, NADH to ubi

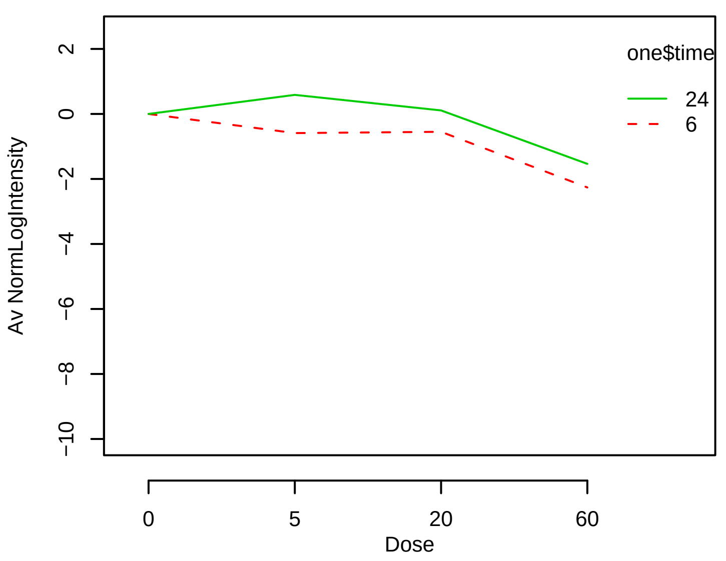

GO_0006140 : regulation of nucleotide metabolism

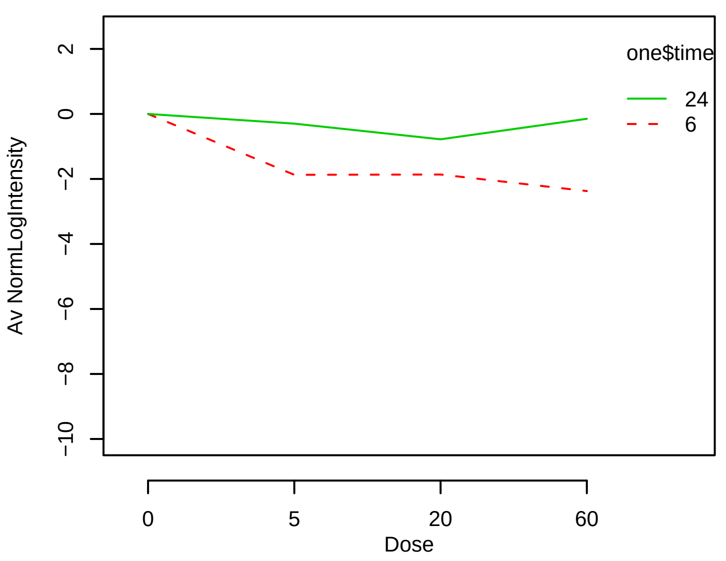

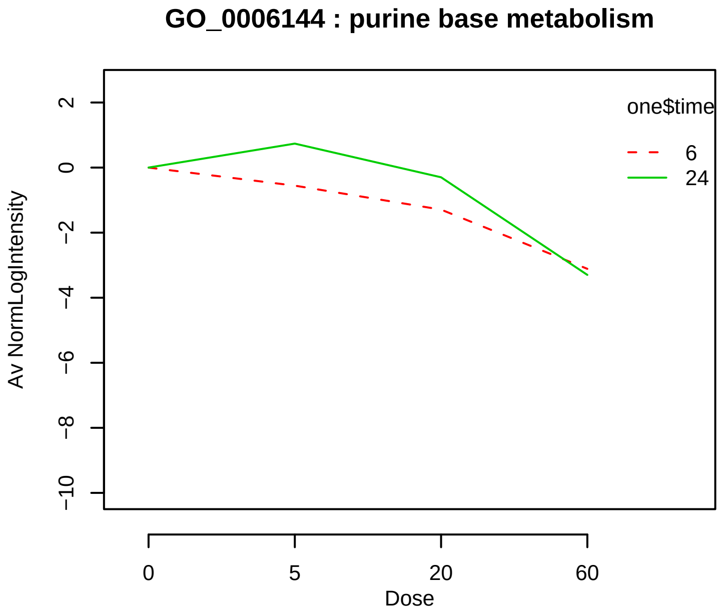
GO_0006144 : purine base metabolism
Av NormLogIntensity
Dose
one$time
6
24
2
0
−2
−4
−6
−8
−10
0
5
20
60

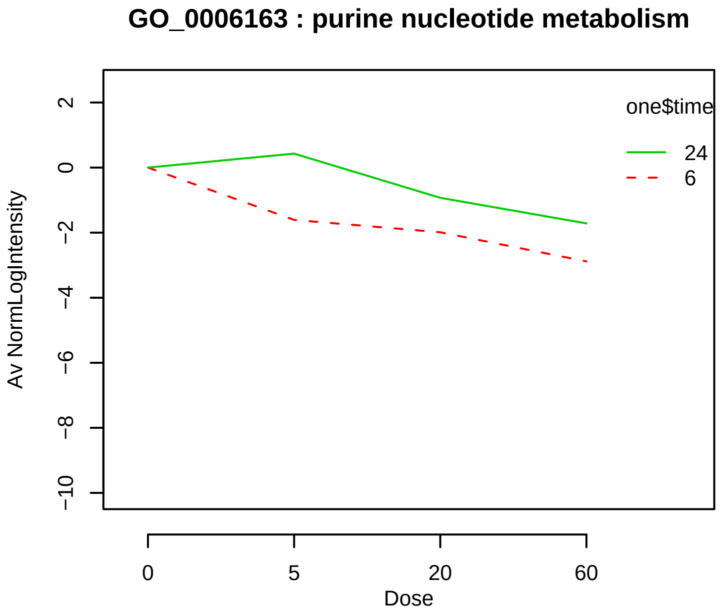
GO_0006163 : purine nucleotide metabolism
Av NormLogIntensity
Dose
one$time
24
6
2
0
−2
−4
−6
−8
−10
0
5
20
60

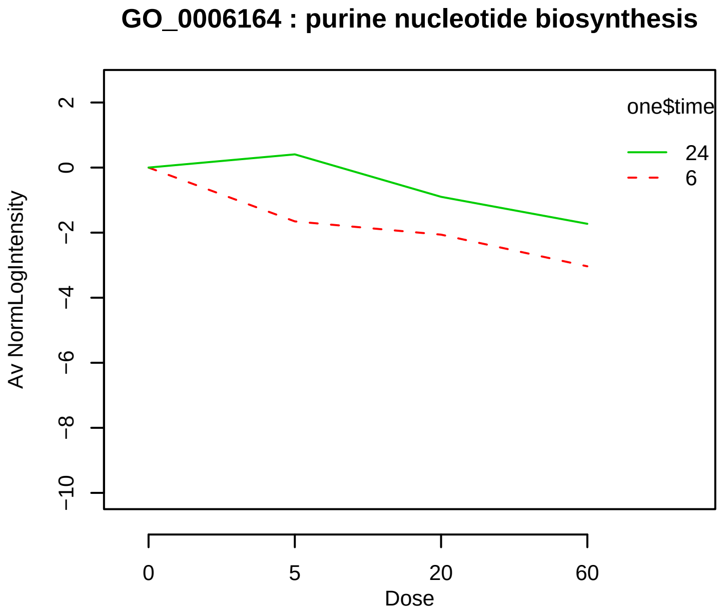
GO_0006164 : purine nucleotide biosynthesis
Av NormLogIntensity
Dose
one$time
24
6
2
0
−2
−4
−6
−8
−10
0
5
20
60

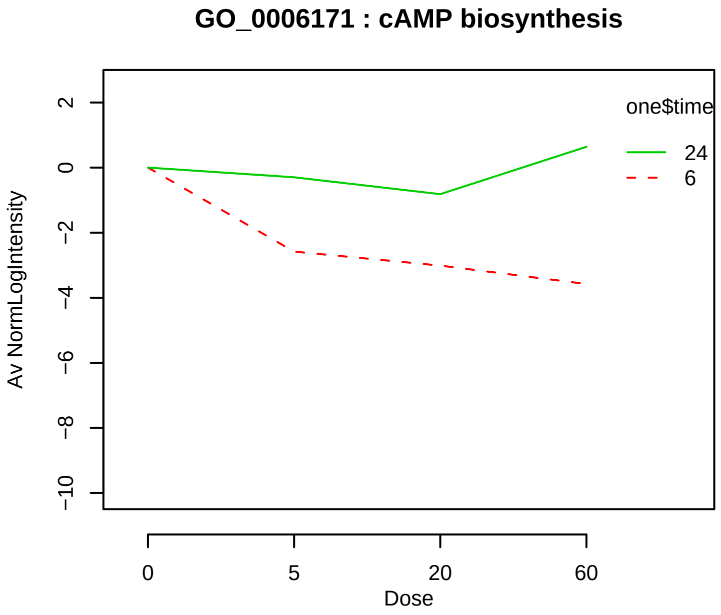
GO_0006171 : cAMP biosynthesis
Av NormLogIntensity
Dose
one$time
24
6
2
0
−2
−4
−6
−8
−10
0
5
20
60

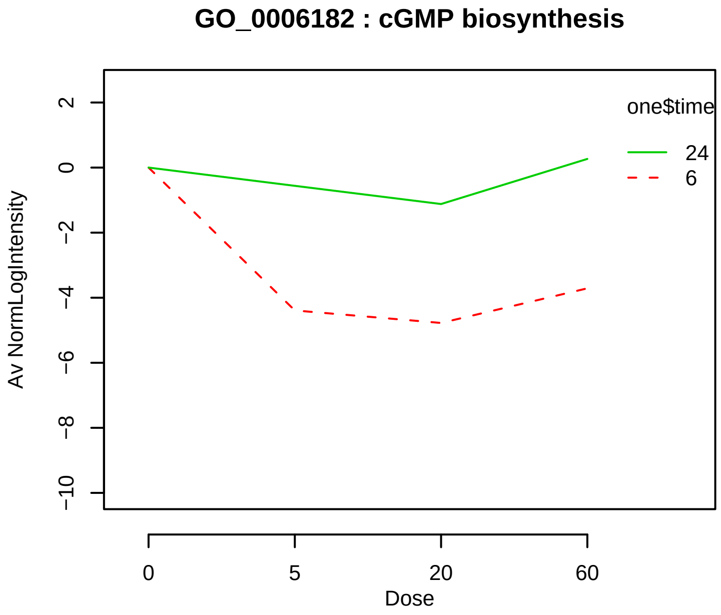
GO_0006182 : cGMP biosynthesis
Av NormLogIntensity
Dose
one$time
24
6
2
0
−2
−4
−6
−8
−10
0
5
20
60

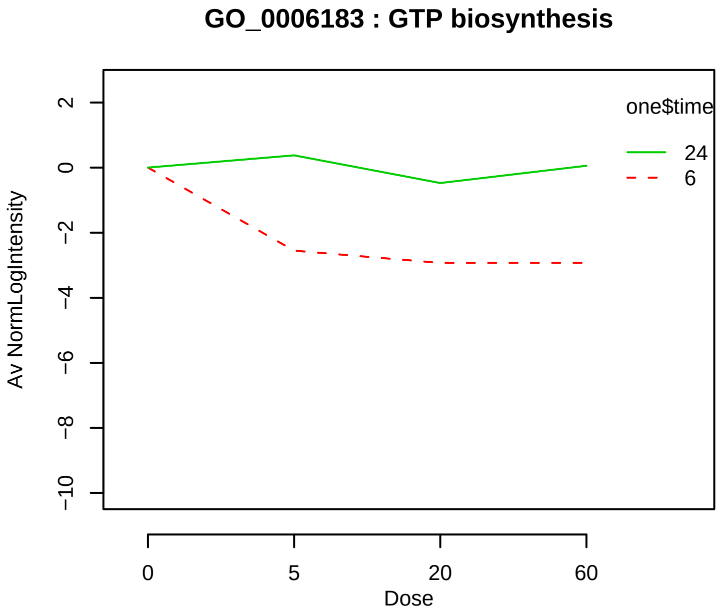
GO_0006183 : GTP biosynthesis
Av NormLogIntensity
Dose
one$time
24
6
2
0
−2
−4
−6
−8
−10
0
5
20
60

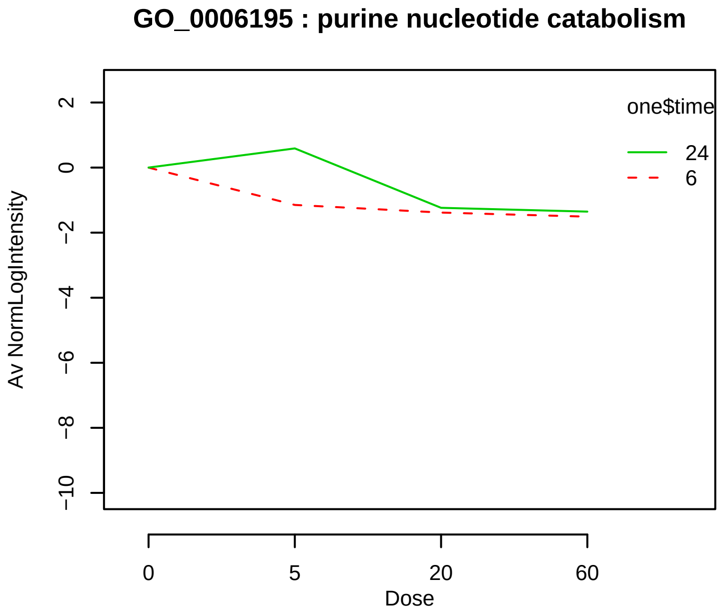
GO_0006195 : purine nucleotide catabolism
one$time
24
6
Av NormLogIntensity
Dose
2
0
−2
−4
−6
−8
−10
0
5
20
60

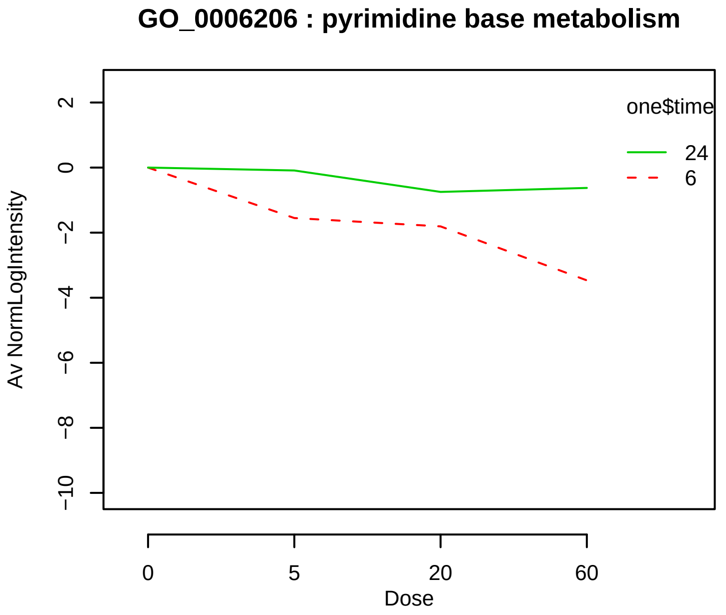
GO_0006206 : pyrimidine base metabolism
one$time
24
6
Av NormLogIntensity
Dose
2
0
−2
−4
−6
−8
−10
0
5
20
60

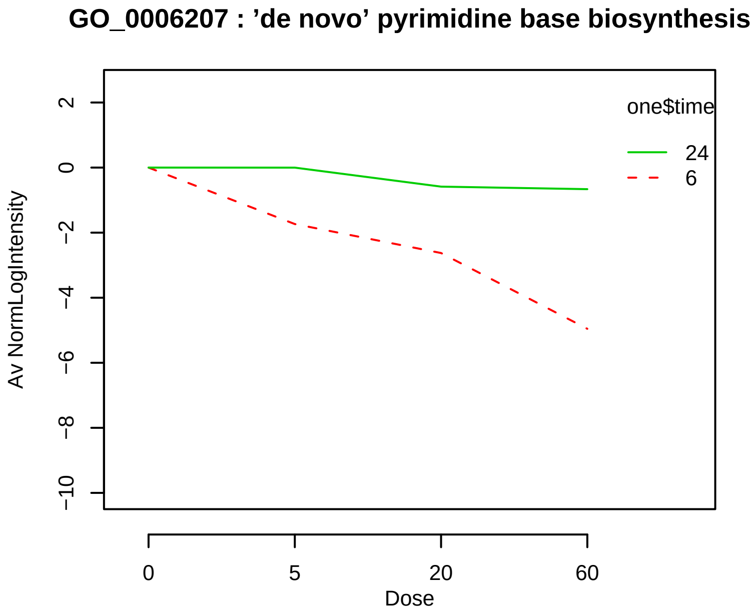
GO_0006207 : 'de novo' pyrimidine base biosynthesis
one$time
24
6
Av NormLogIntensity
Dose
2
0
−2
−4
−6
−8
−10
0
5
20
60

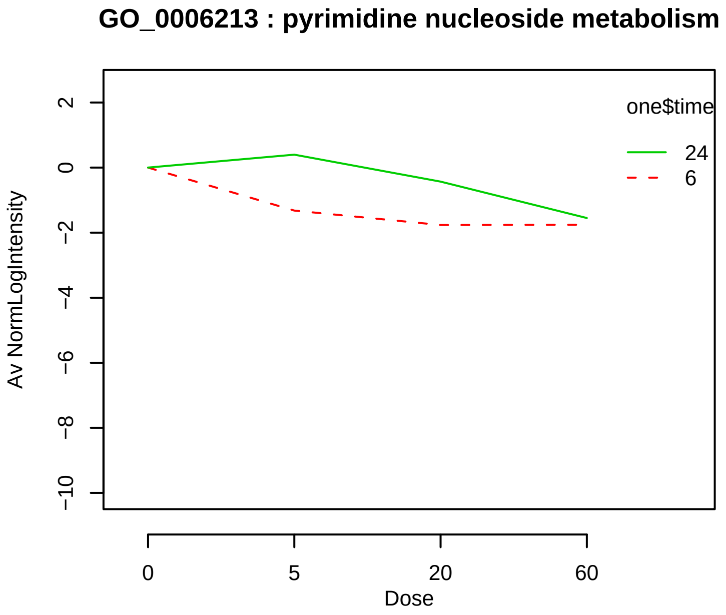
GO_0006213 : pyrimidine nucleoside metabolism
one$time
24
6
Av NormLogIntensity
Dose
2
0
−2
−4
−6
−8
−10
0
5
20
60

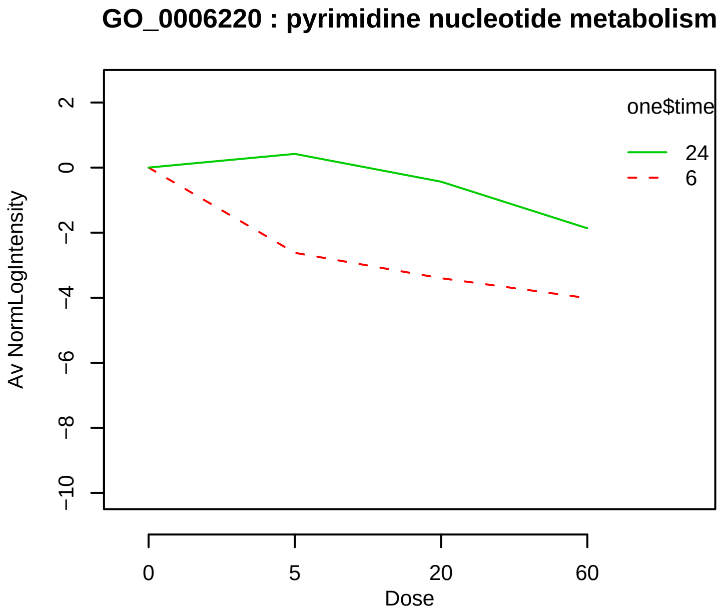
GO_0006220 : pyrimidine nucleotide metabolism
one$time
24
6
Av NormLogIntensity
Dose
2
0
−2
−4
−6
−8
−10
0
5
20
60

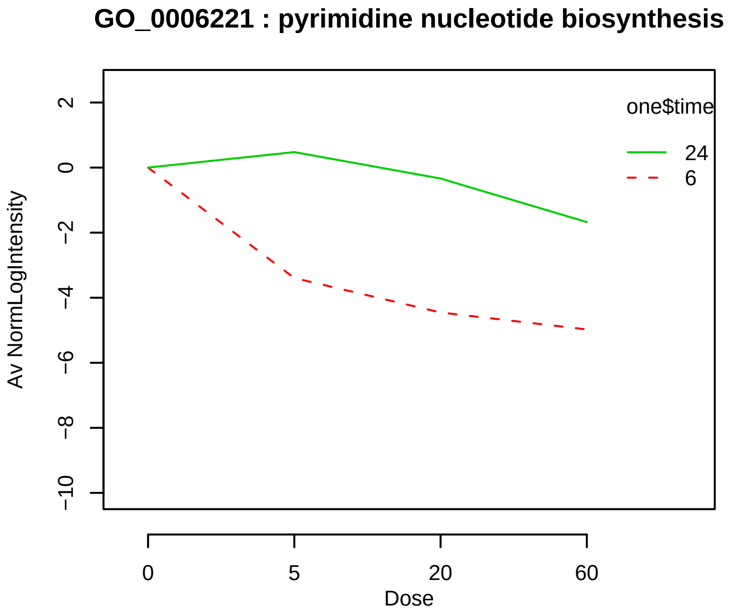
GO_0006221 : pyrimidine nucleotide biosynthesis
one$time
24
6
Av NormLogIntensity
Dose
2
0
−2
−4
−6
−8
−10
0
5
20
60

GO_0006228 : UTP biosynthesis

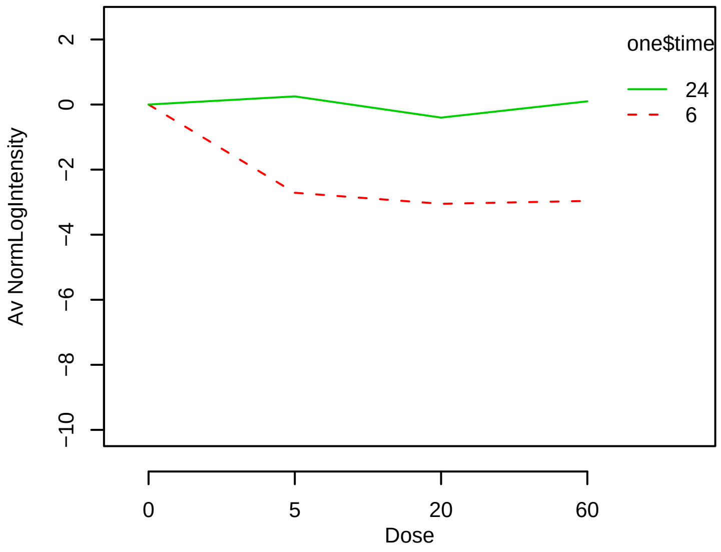

GO_0006241 : CTP biosynthesis

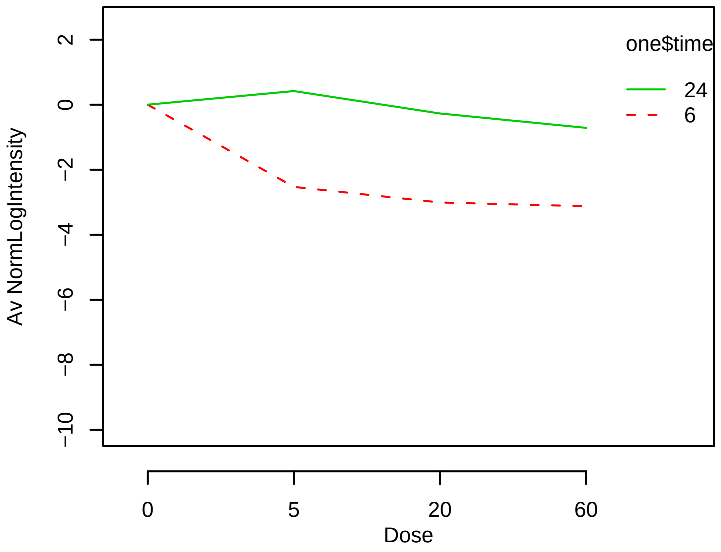

GO_0006260 : DNA replication

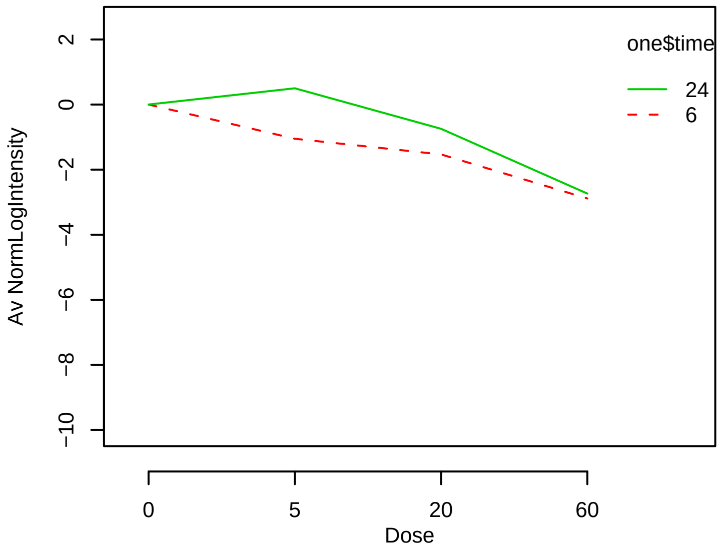

GO_0006261 : DNA−dependent DNA replication

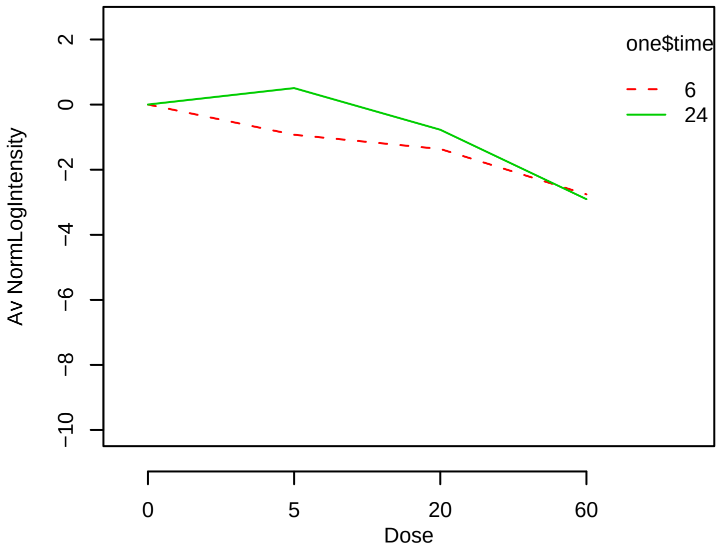

GO_0006265 : DNA topological change

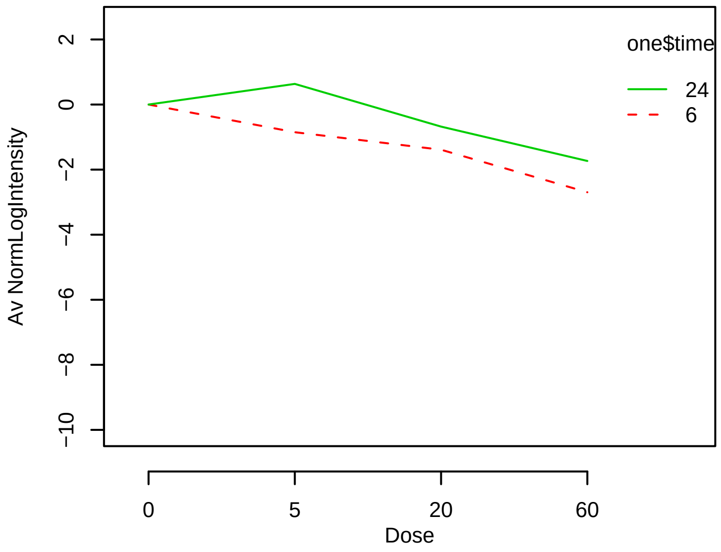

GO_0006268 : DNA unwinding during replication

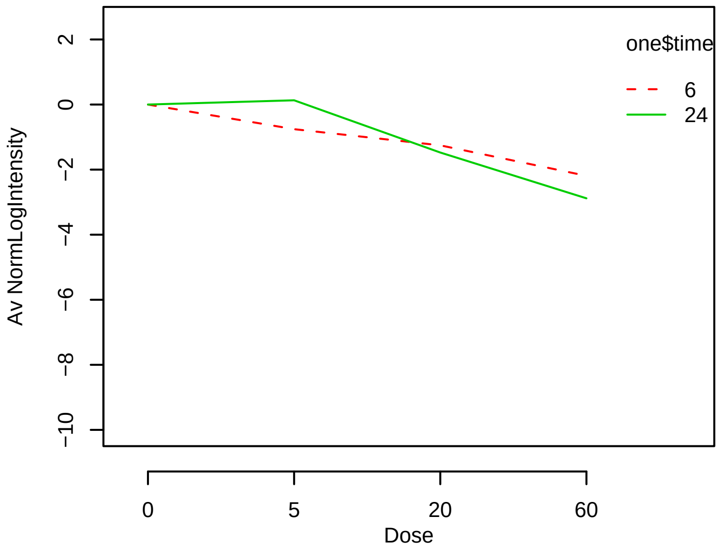

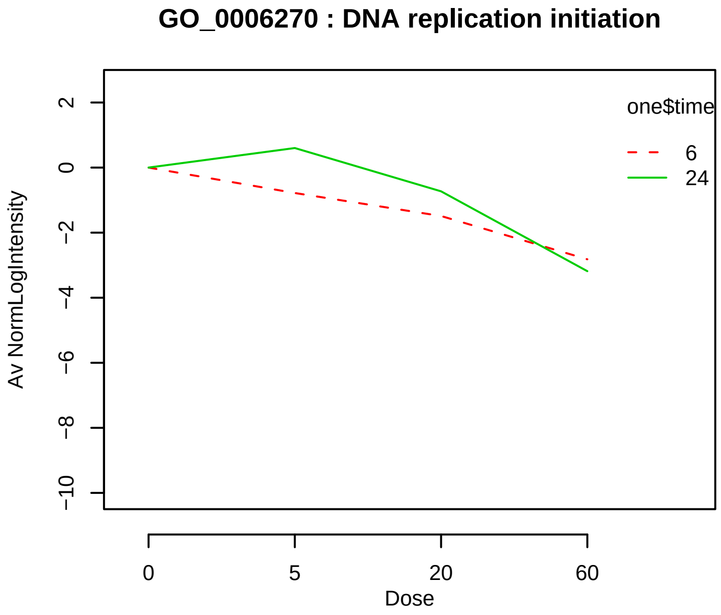
GO_0006270 : DNA replication initiation
Av NormLogIntensity
Dose
one$time
6
24
0
5
20
60
2
0
−2
−4
−6
−8
−10

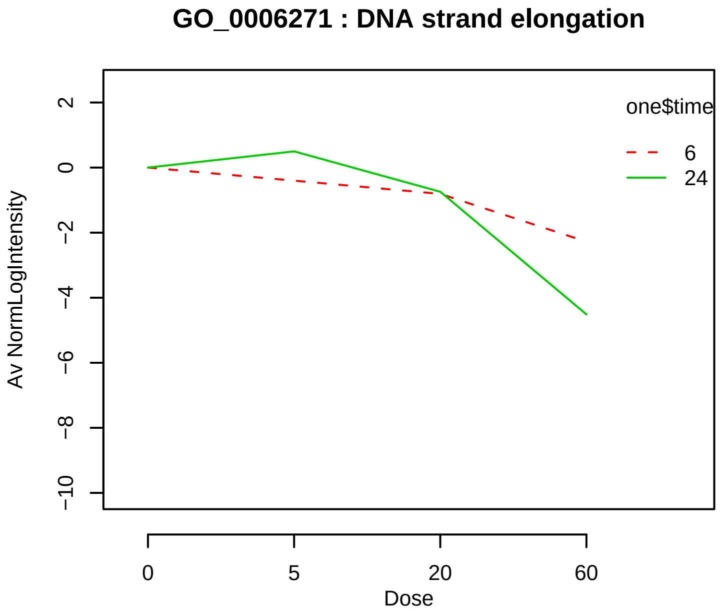
GO_0006271 : DNA strand elongation
Av NormLogIntensity
Dose
one$time
6
24
0
5
20
60
2
0
−2
−4
−6
−8
−10

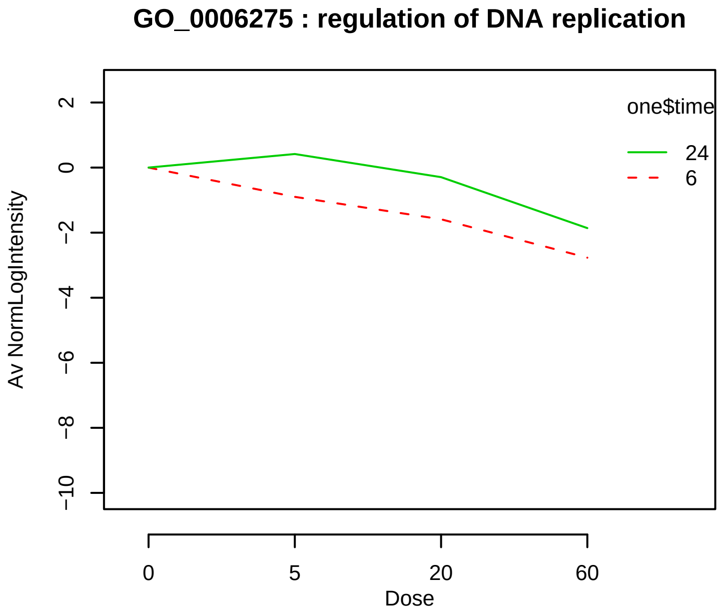
GO_0006275 : regulation of DNA replication
Av NormLogIntensity
Dose
one$time
24
6
0
5
20
60
2
0
−2
−4
−6
−8
−10

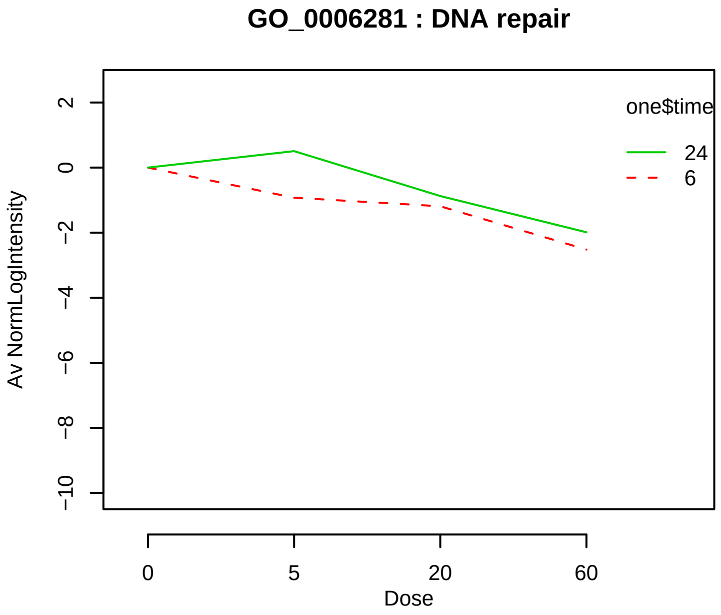
GO_0006281 : DNA repair
Av NormLogIntensity
Dose
one$time
24
6
0
5
20
60
2
0
−2
−4
−6
−8
−10

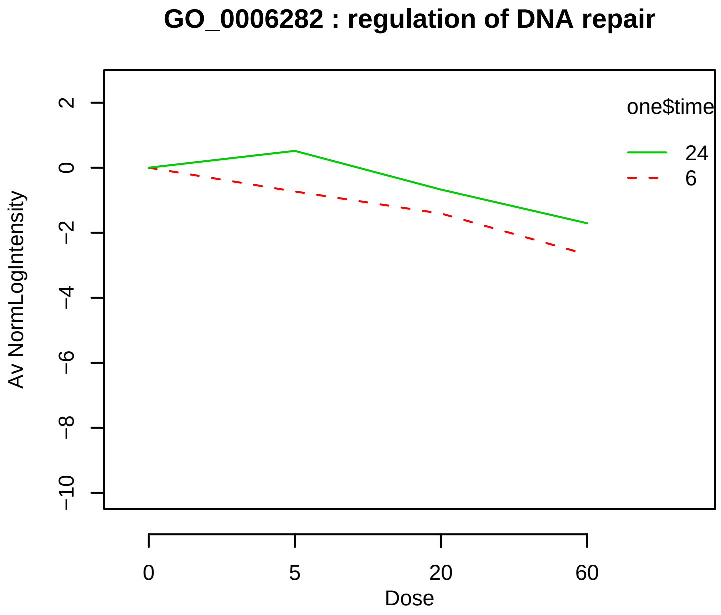
GO_0006282 : regulation of DNA repair
Av NormLogIntensity
Dose
one$time
24
6
0
5
20
60
2
0
−2
−4
−6
−8
−10

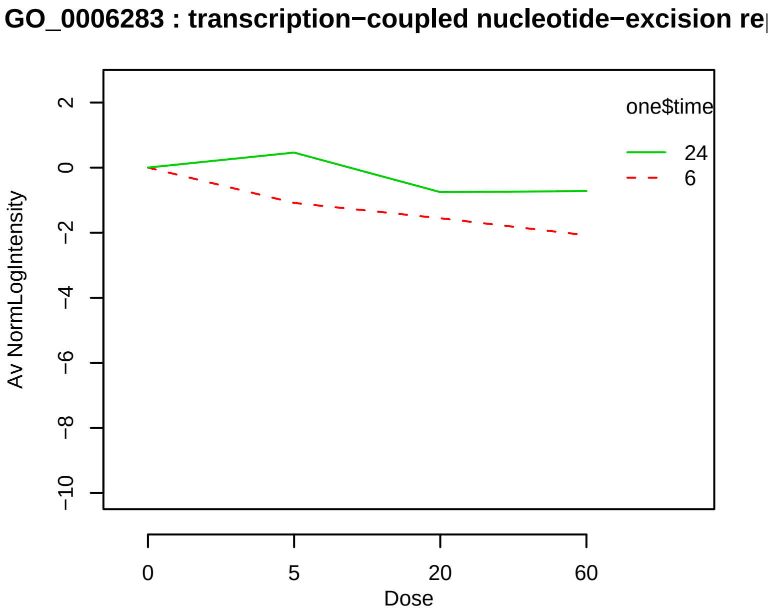
GO_0006283 : transcription−coupled nucleotide−excision re
Av NormLogIntensity
Dose
one$time
24
6
0
5
20
60
2
0
−2
−4
−6
−8
−10

GO_0006284 : base−excision repair

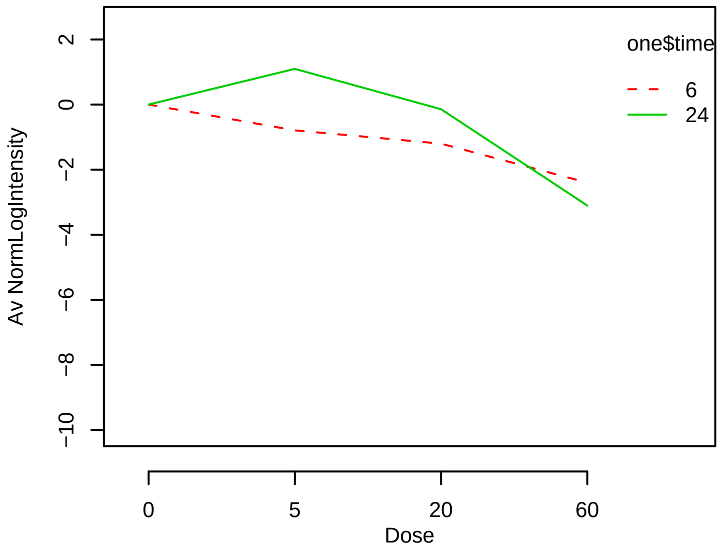

GO_0006289 : nucleotide−excision repair

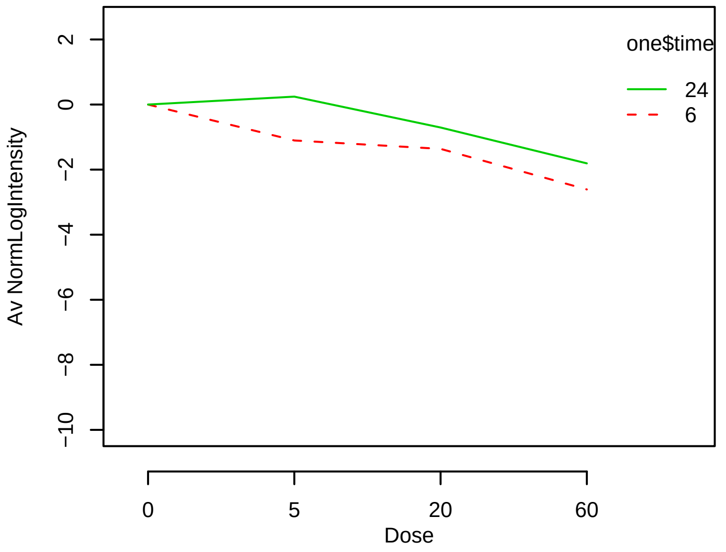

GO_0006298 : mismatch repair

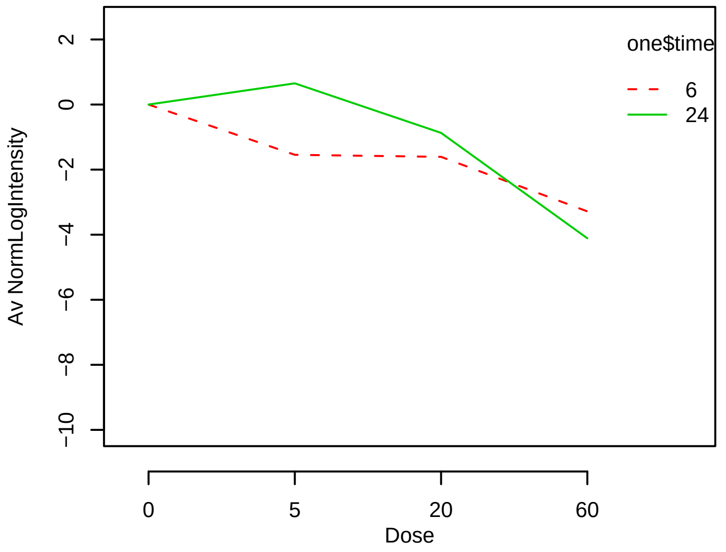

GO_0006301 : postreplication repair

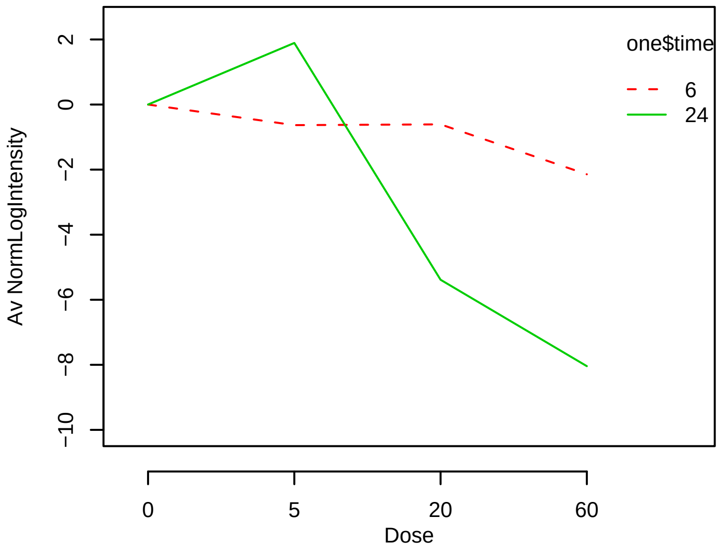

GO_0006302 : double−strand break repair

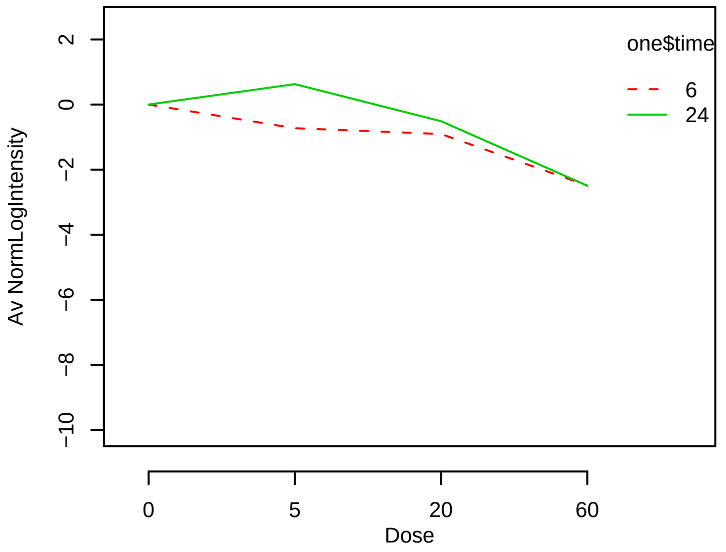

O_0006303 : double−strand break repair via nonhomologous

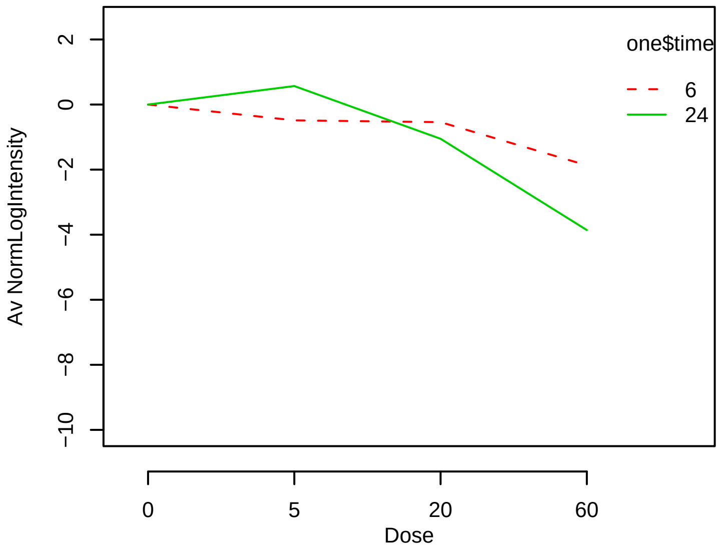

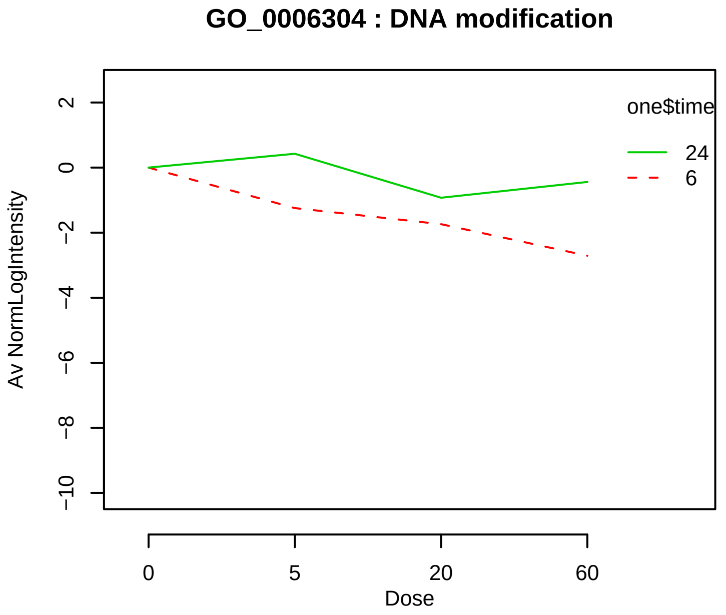
GO_0006304 : DNA modification
Av NormLogIntensity
2
0
−2
−4
−6
−8
−10
one$time
24
6
0
5
20
60
Dose

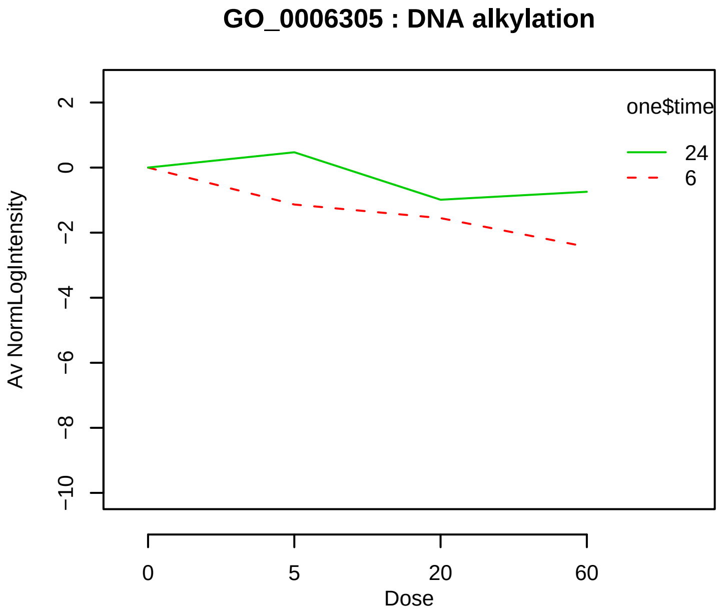
GO_0006305 : DNA alkylation
Av NormLogIntensity
2
0
−2
−4
−6
−8
−10
one$time
24
6
0
5
20
60
Dose

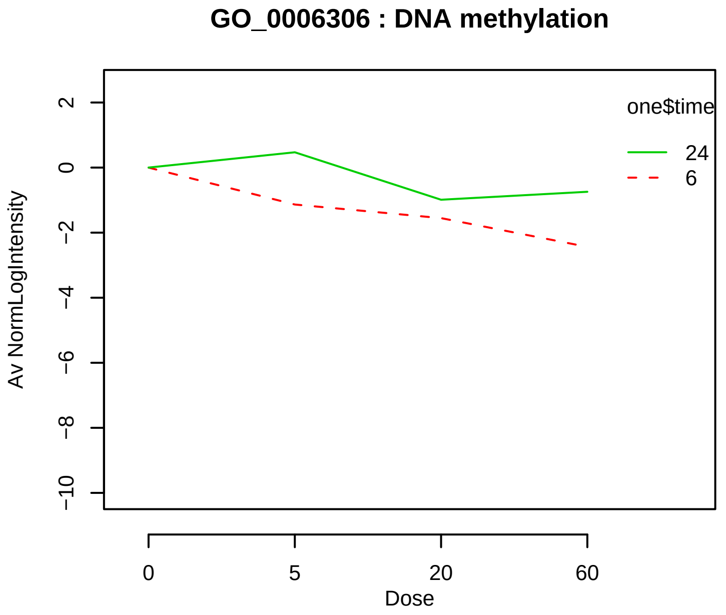
GO_0006306 : DNA methylation
Av NormLogIntensity
2
0
−2
−4
−6
−8
−10
one$time
24
6
0
5
20
60
Dose

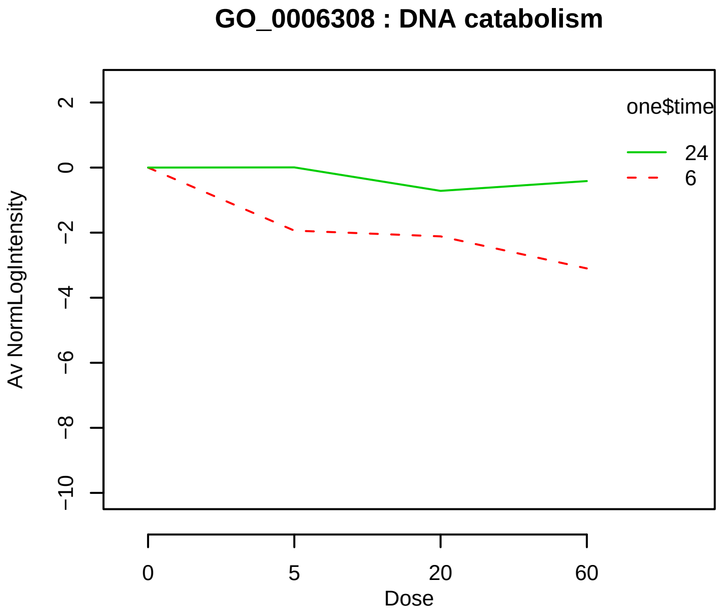
GO_0006308 : DNA catabolism
Av NormLogIntensity
2
0
−2
−4
−6
−8
−10
one$time
24
6
0
5
20
60
Dose

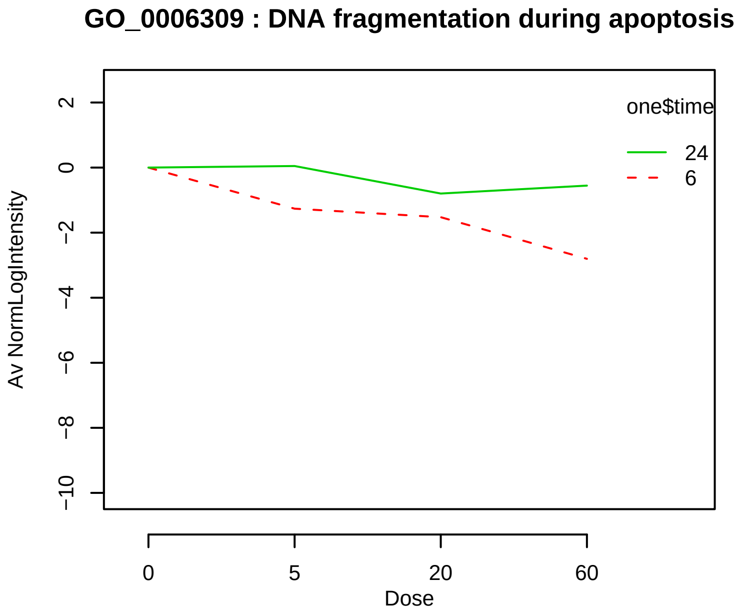
GO_0006309 : DNA fragmentation during apoptosis
Av NormLogIntensity
2
0
−2
−4
−6
−8
−10
one$time
24
6
0
5
20
60
Dose

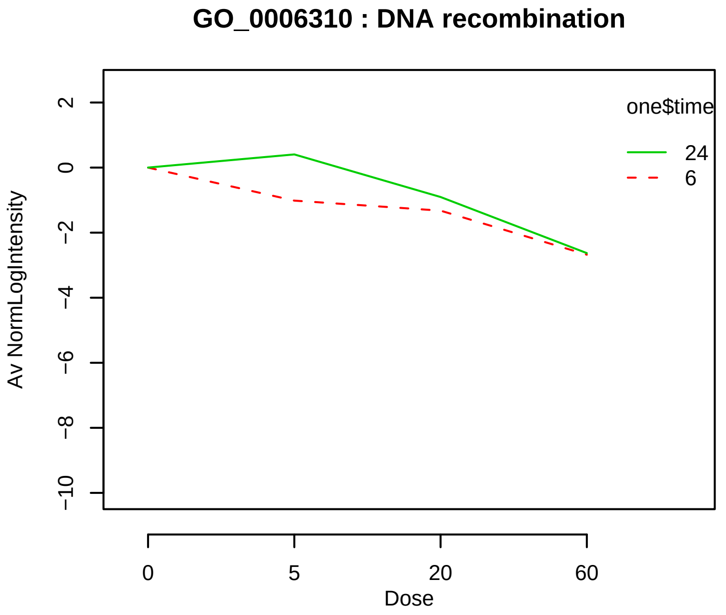
GO_0006310 : DNA recombination
Av NormLogIntensity
2
0
−2
−4
−6
−8
−10
one$time
24
6
0
5
20
60
Dose

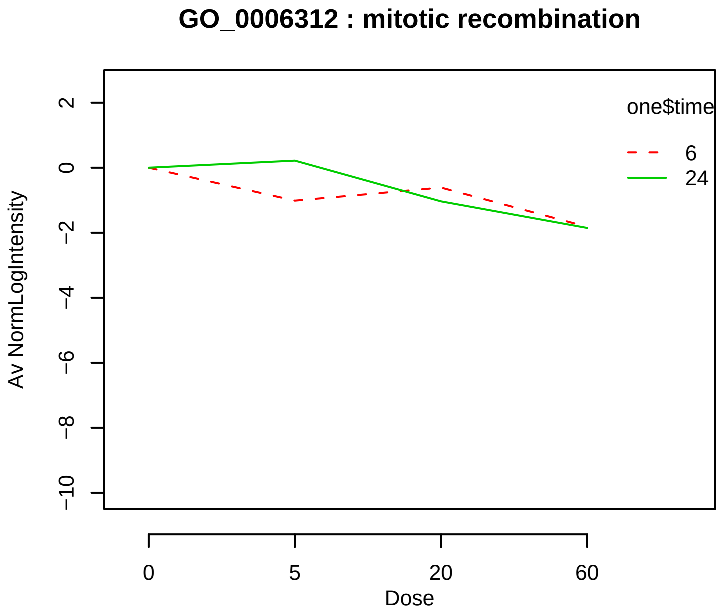
GO_0006312 : mitotic recombination
Av NormLogIntensity
2
0
−2
−4
−6
−8
−10
one$time
6
24
0
5
20
60
Dose

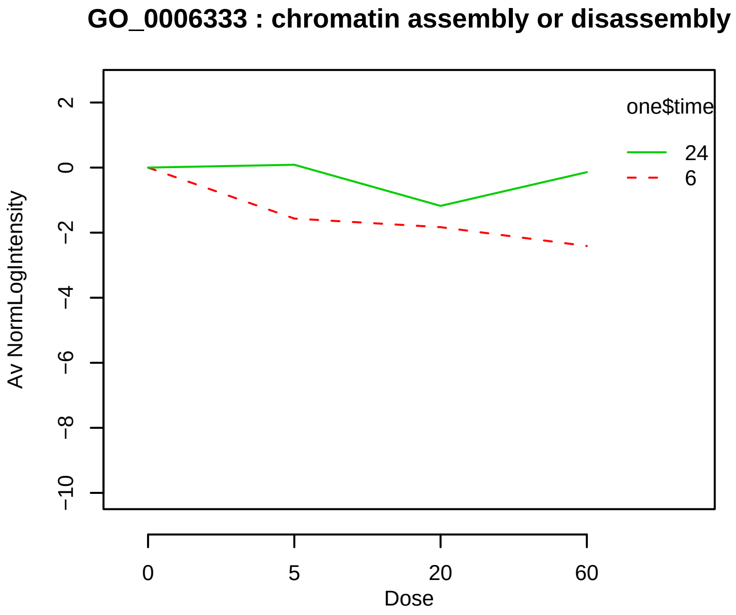
GO_0006333 : chromatin assembly or disassembly
Av NormLogIntensity
2
0
−2
−4
−6
−8
−10
one$time
24
6
0
5
20
60
Dose

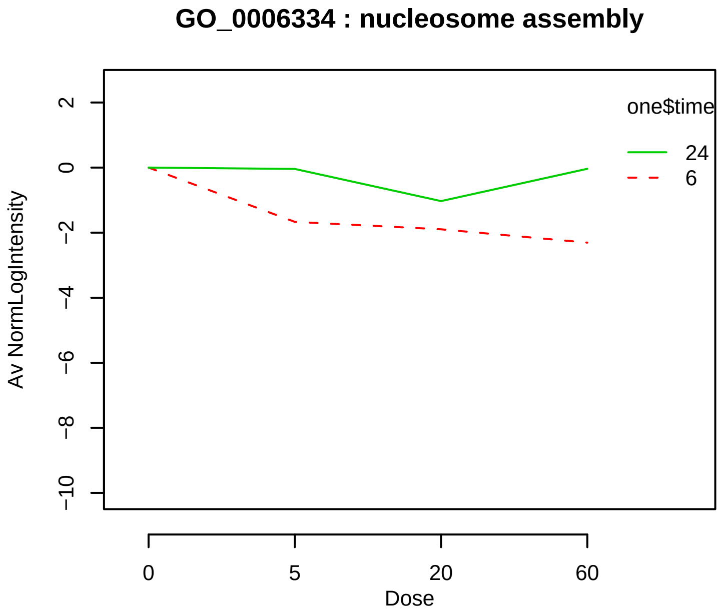
GO_0006334 : nucleosome assembly
Av NormLogIntensity
2
0
−2
−4
−6
−8
−10
one$time
24
6
0
5
20
60
Dose

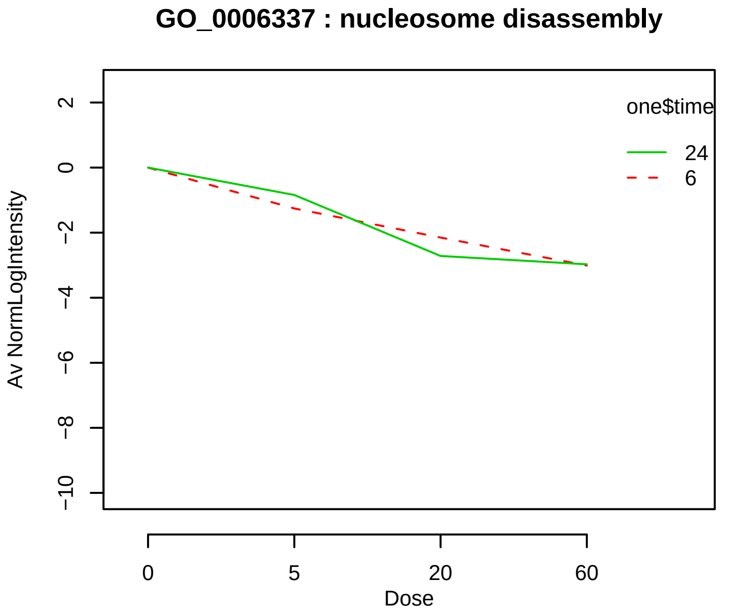
GO_0006337 : nucleosome disassembly
Av NormLogIntensity
2
0
−2
−4
−6
−8
−10
one$time
24
6
0
5
20
60
Dose

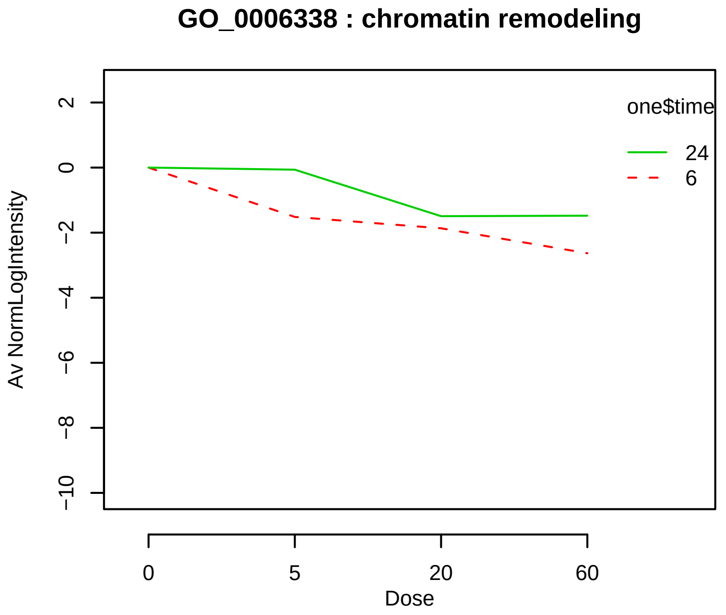
GO_0006338 : chromatin remodeling
Av NormLogIntensity
2
0
−2
−4
−6
−8
−10
one$time
24
6
0
5
20
60
Dose

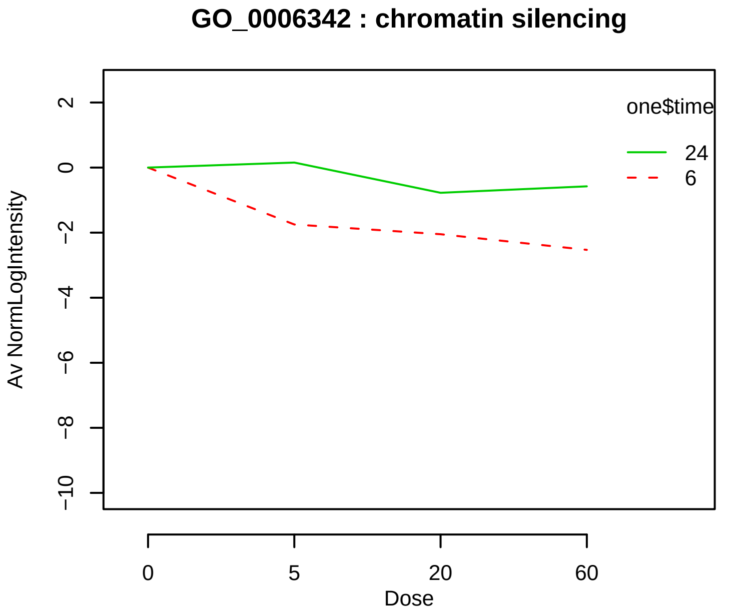
GO_0006342 : chromatin silencing
Av NormLogIntensity
2
0
−2
−4
−6
−8
−10
one$time
24
6
0
5
20
60
Dose

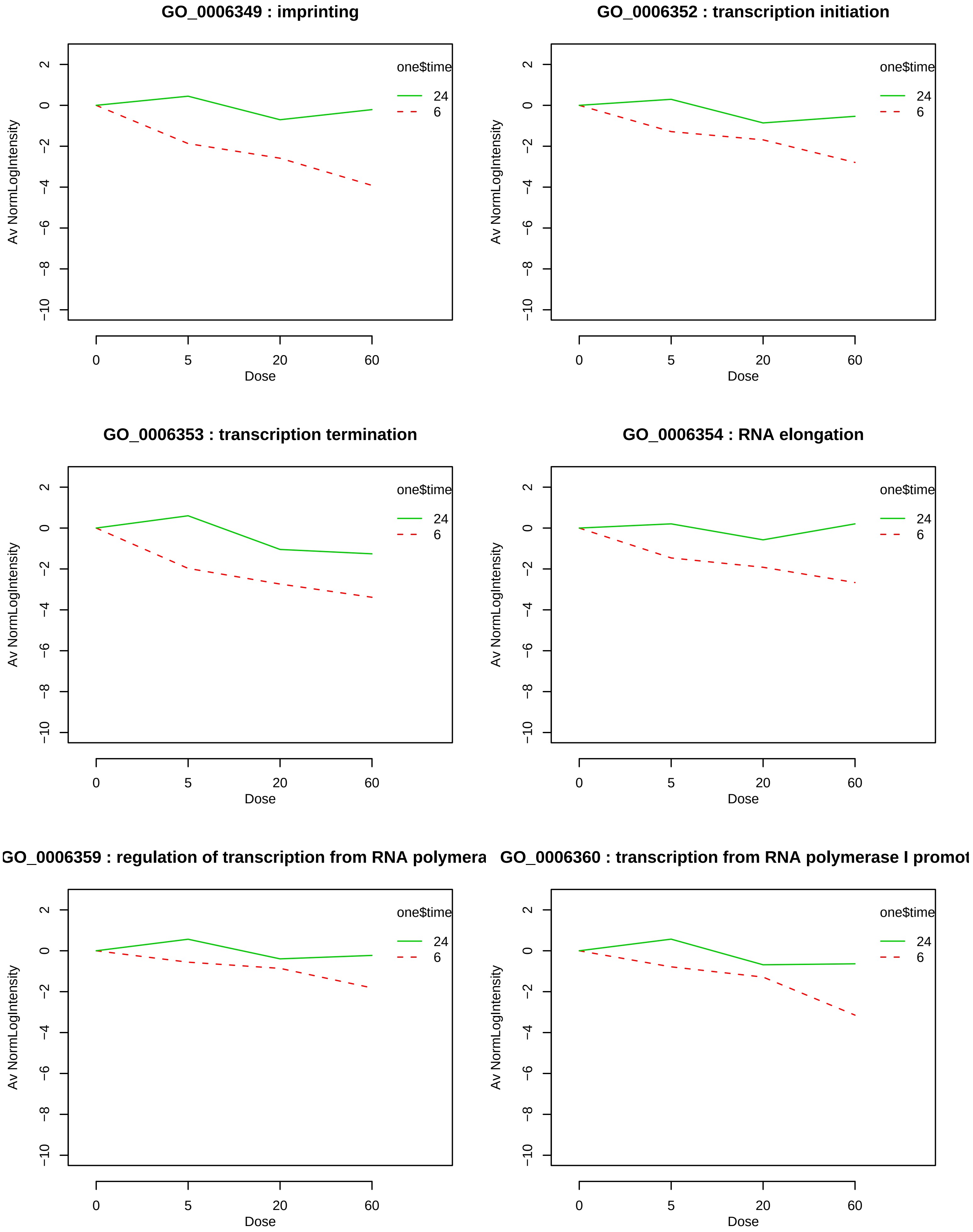
GO_0006349 : imprinting
GO_0006352 : transcription initiation
GO_0006353 : transcription termination
GO_0006354 : RNA elongation
GO_0006359 : regulation of transcription from RNA polymera
GO_0006360 : transcription from RNA polymerase I promot
one$time
24
6
Av NormLogIntensity
Dose
0
5
20
60
2
0
−2
−4
−6
−8
−10

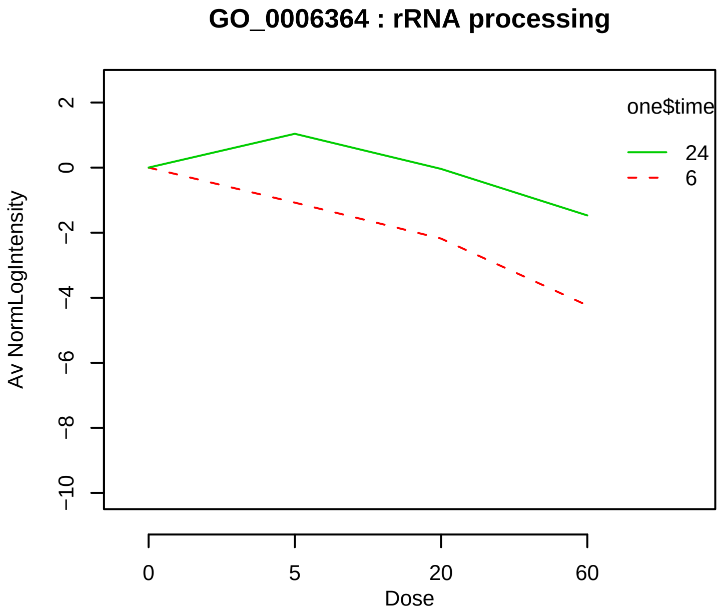
GO_0006364 : rRNA processing
Av NormLogIntensity
2 0 −2 −4 −6 −8 −10
Dose
0 5 20 60
one$time
24
6

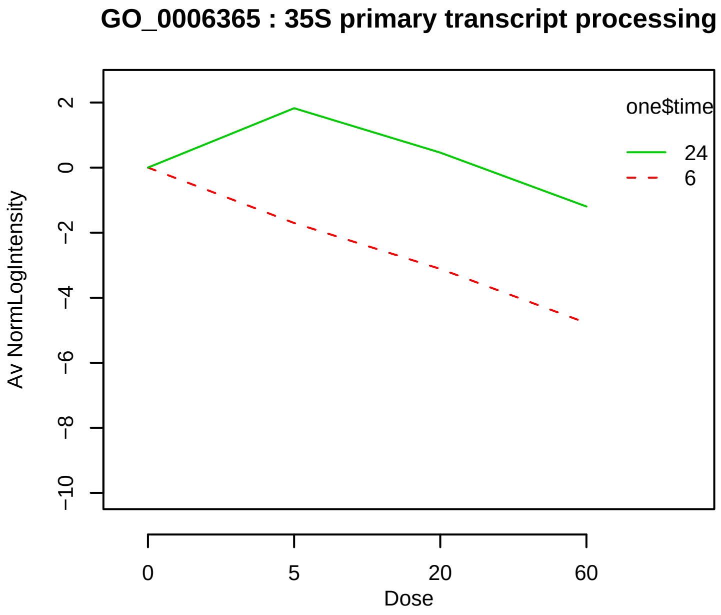
GO_0006365 : 35S primary transcript processing
Av NormLogIntensity
2 0 −2 −4 −6 −8 −10
Dose
0 5 20 60
one$time
24
6

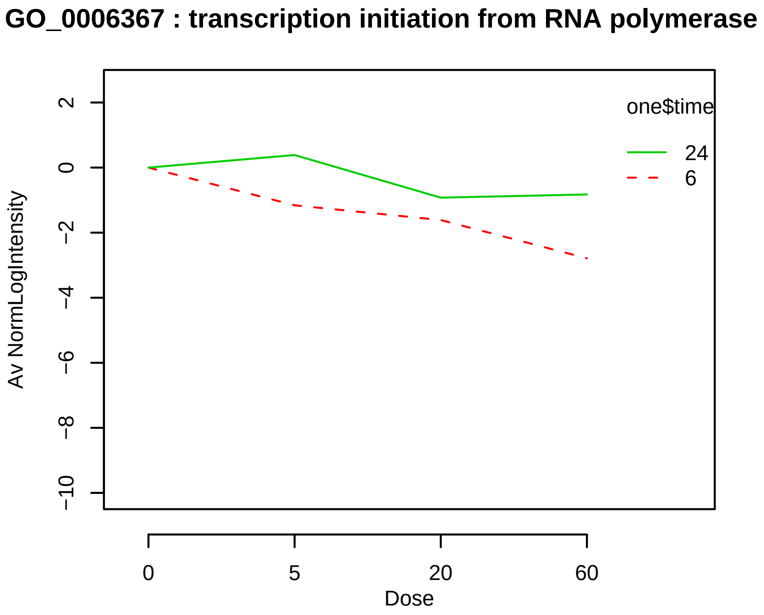
GO_0006367 : transcription initiation from RNA polymerase
Av NormLogIntensity
2 0 −2 −4 −6 −8 −10
Dose
0 5 20 60
one$time
24
6

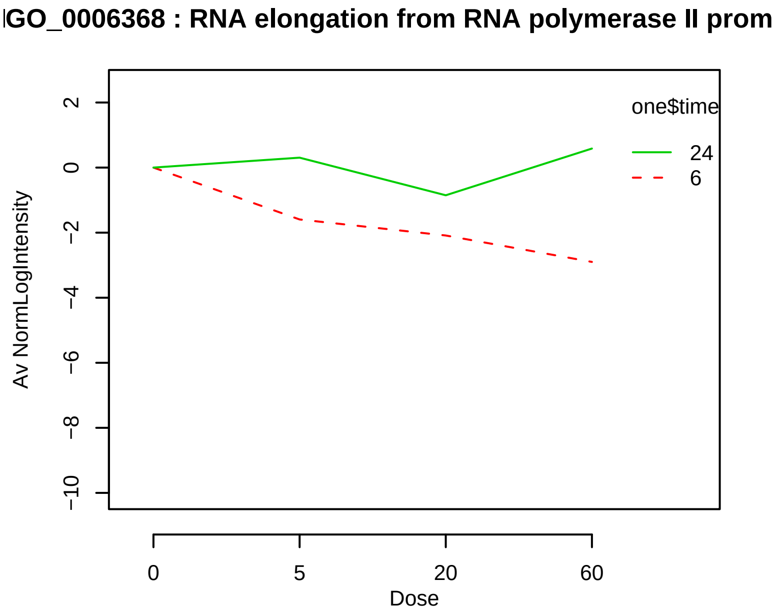
GO_0006368 : RNA elongation from RNA polymerase II prom
Av NormLogIntensity
2 0 −2 −4 −6 −8 −10
Dose
0 5 20 60
one$time
24
6

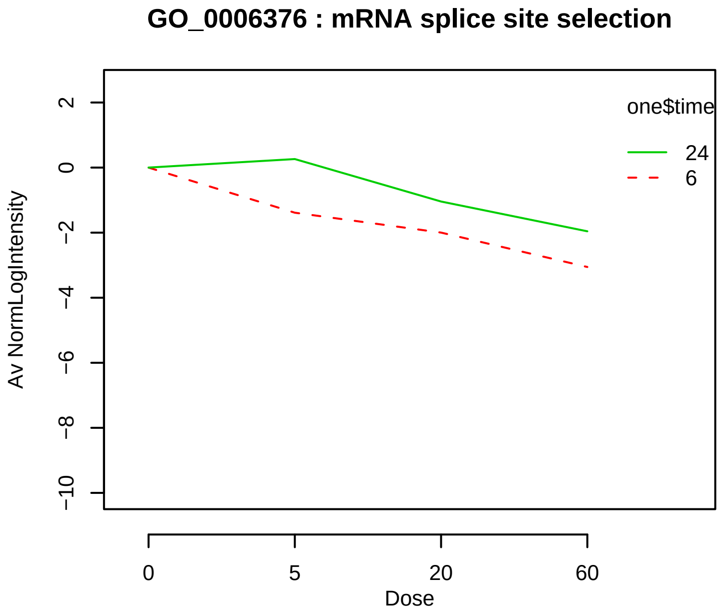
GO_0006376 : mRNA splice site selection
Av NormLogIntensity
2 0 −2 −4 −6 −8 −10
Dose
0 5 20 60
one$time
24
6

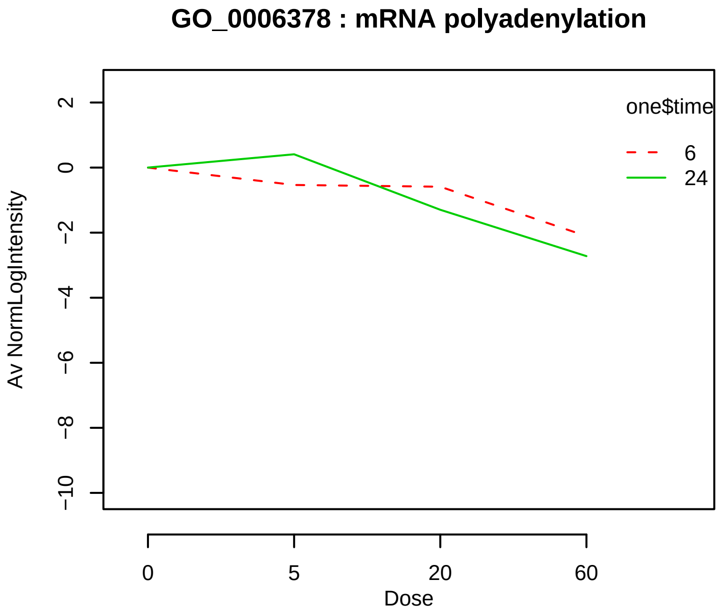
GO_0006378 : mRNA polyadenylation
Av NormLogIntensity
2 0 −2 −4 −6 −8 −10
Dose
0 5 20 60
one$time
6
24

GO_0006379 : mRNA cleavage

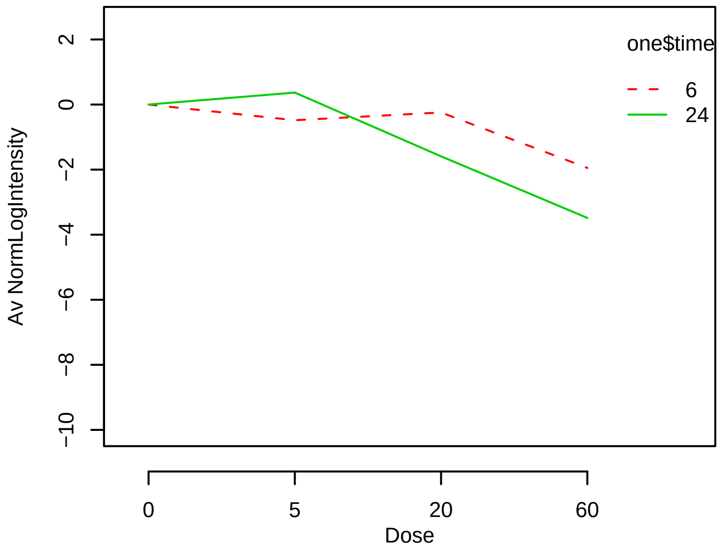

GO_0006381 : mRNA editing

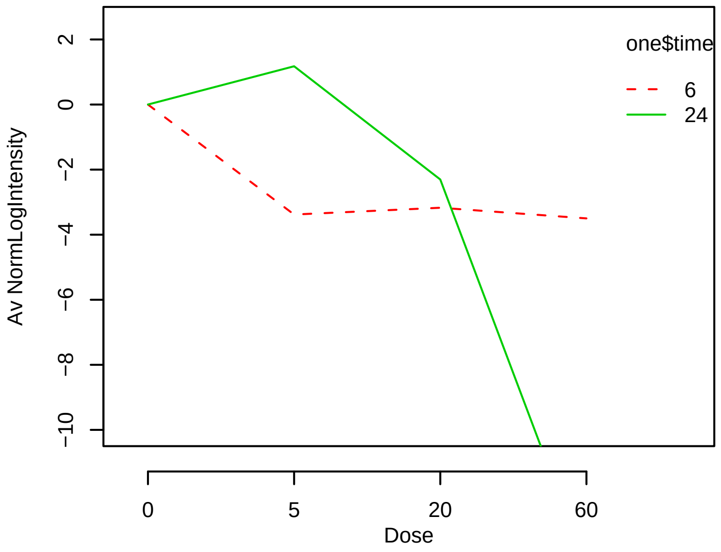

GO_0006383 : transcription from RNA polymerase III promo

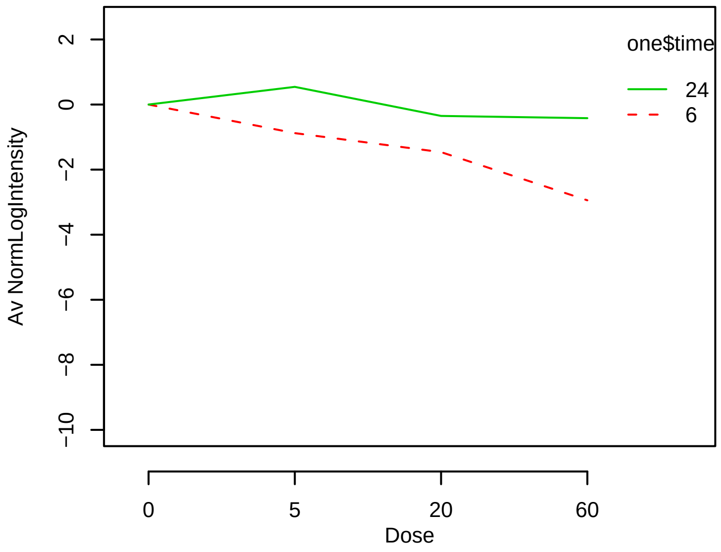

GO_0006397 : mRNA processing

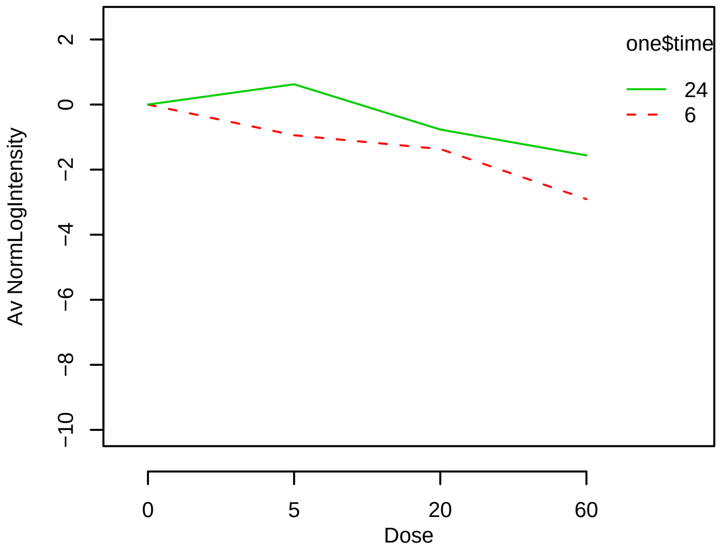

GO_0006399 : tRNA metabolism

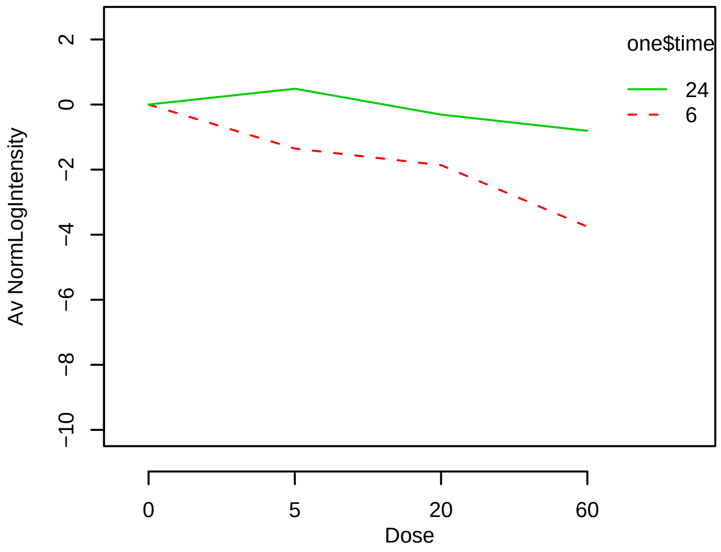

GO_0006400 : tRNA modification

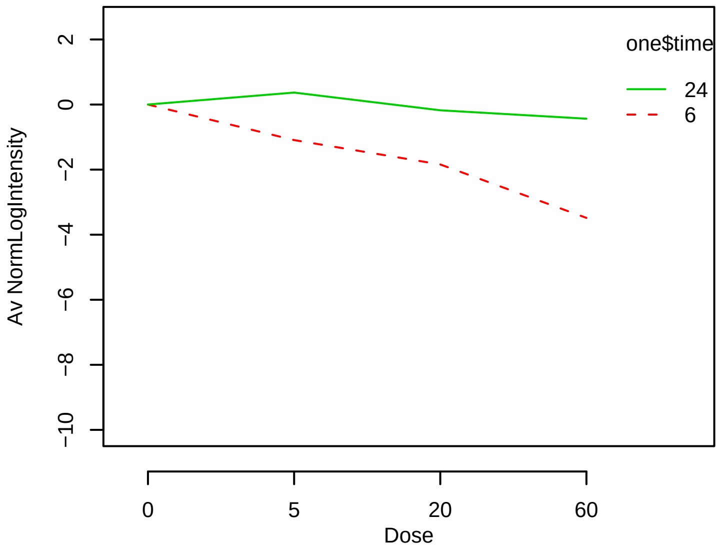

GO_0006401 : RNA catabolism

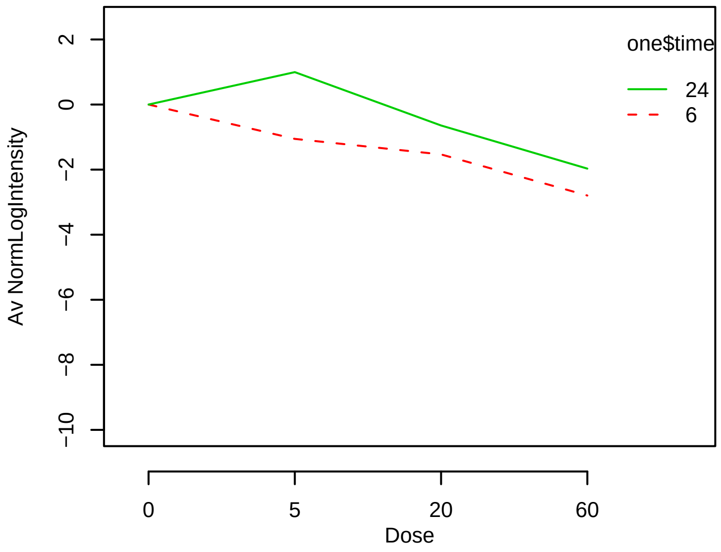

GO_0006402 : mRNA catabolism

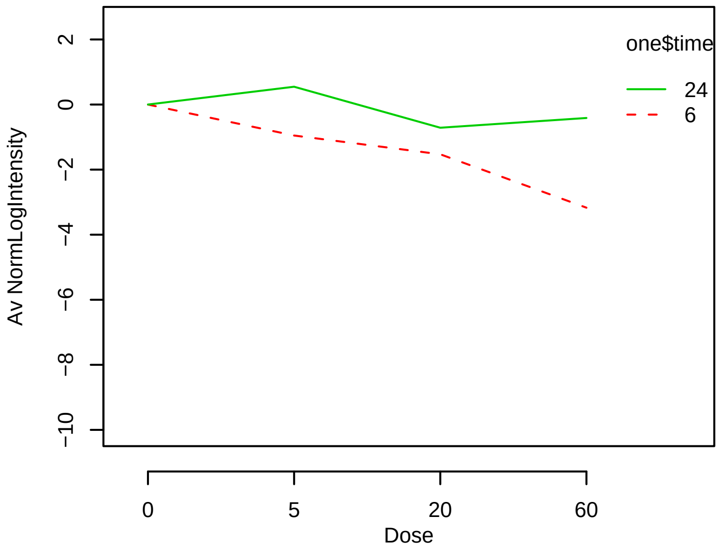

GO_0006403 : RNA localization

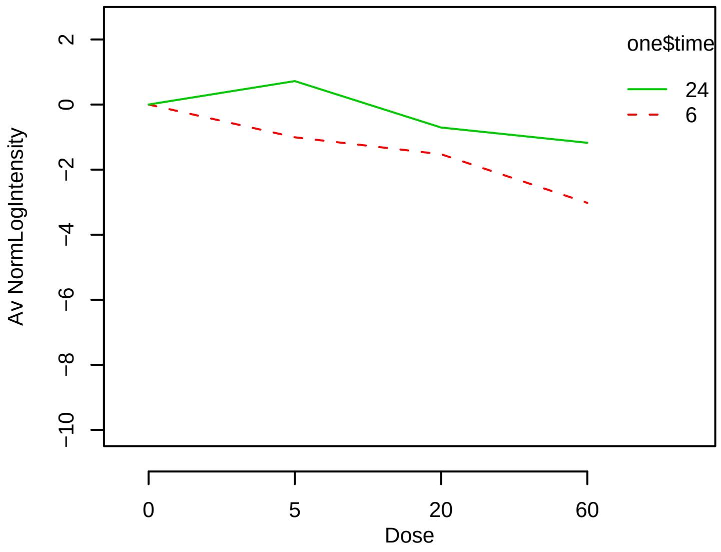

GO_0006405 : RNA export from nucleus

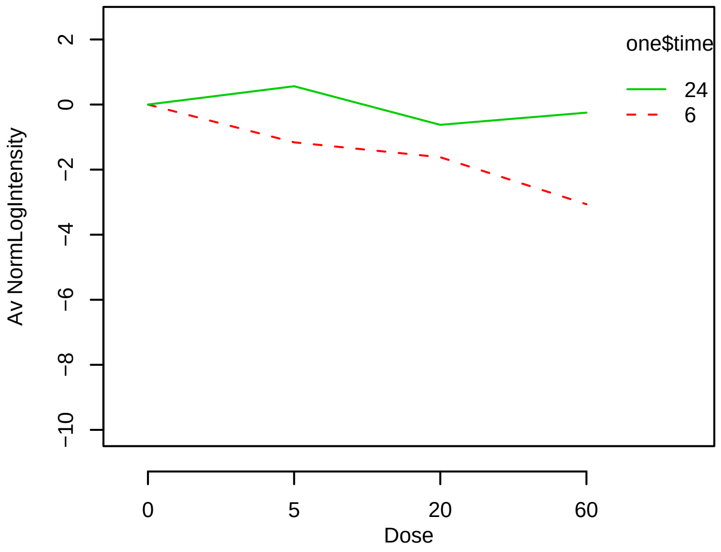

GO_0006406 : mRNA export from nucleus

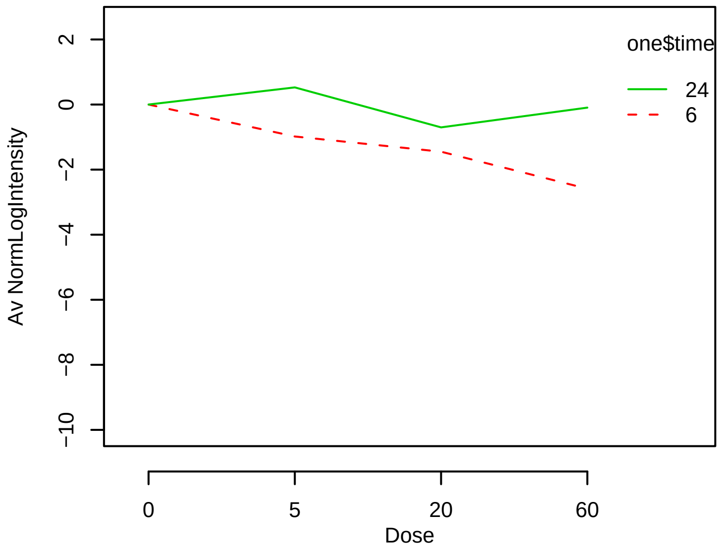

GO_0006413 : translational initiation

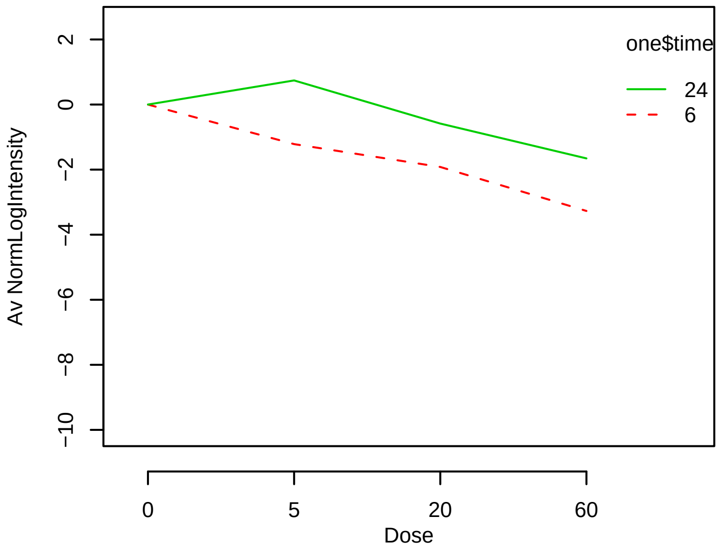

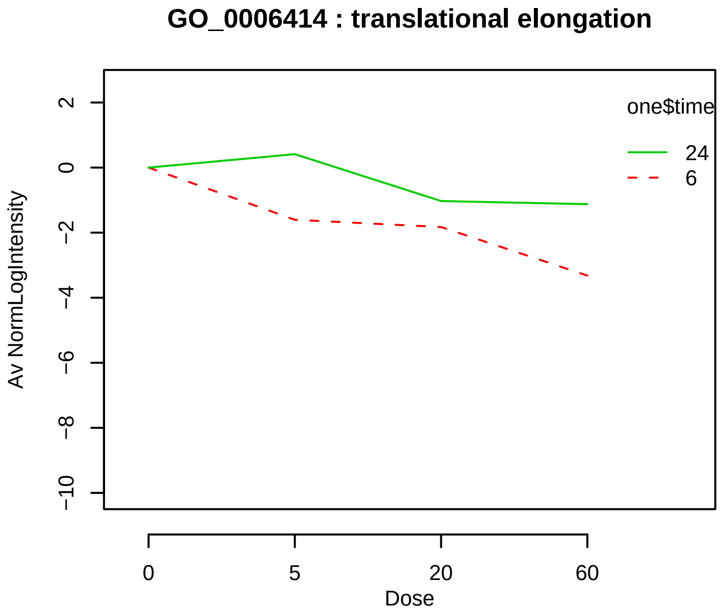
GO_0006414 : translational elongation
Av NormLogIntensity
Dose
one$time
24
6

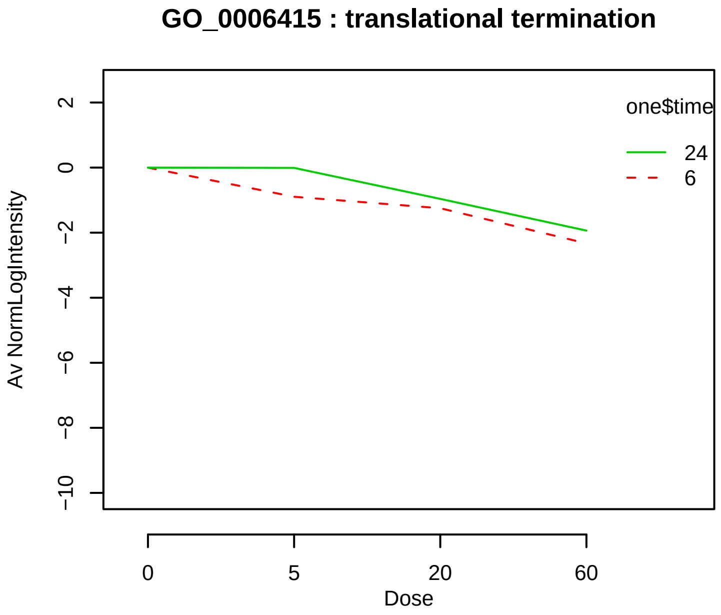
GO_0006415 : translational termination
Av NormLogIntensity
Dose
one$time
24
6

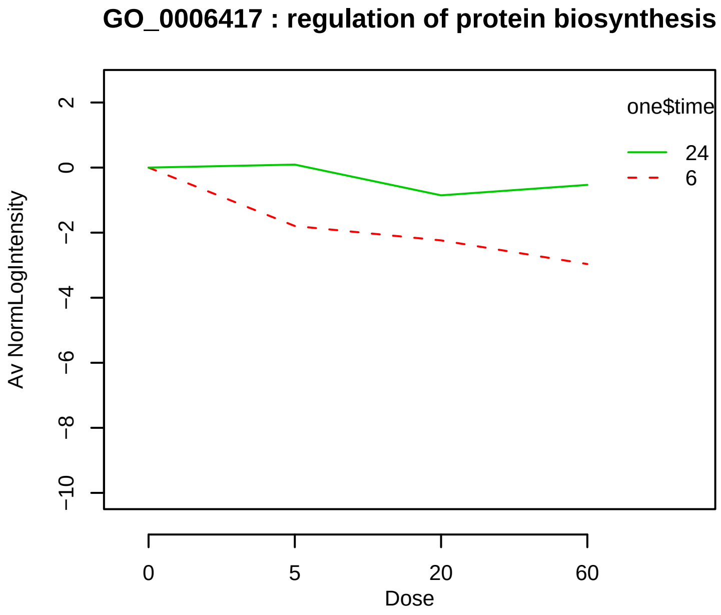
GO_0006417 : regulation of protein biosynthesis
Av NormLogIntensity
Dose
one$time
24
6

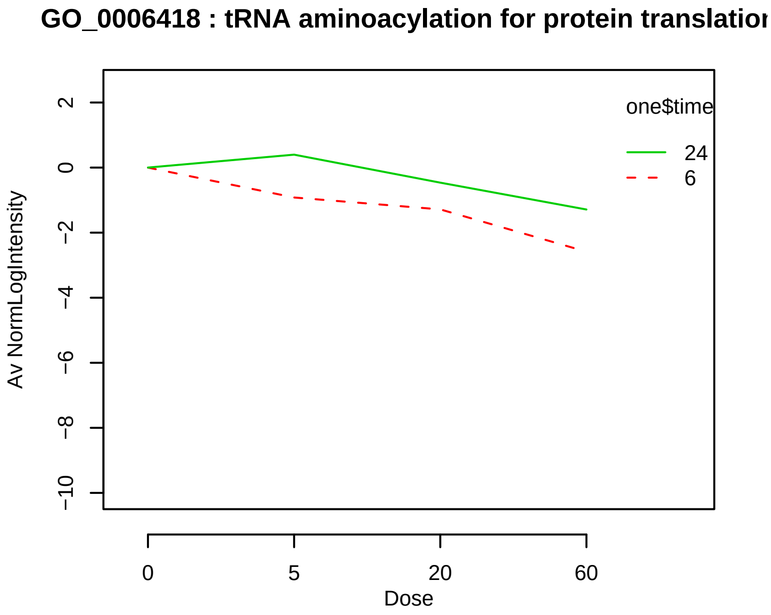
GO_0006418 : tRNA aminoacylation for protein translatior
Av NormLogIntensity
Dose
one$time
24
6

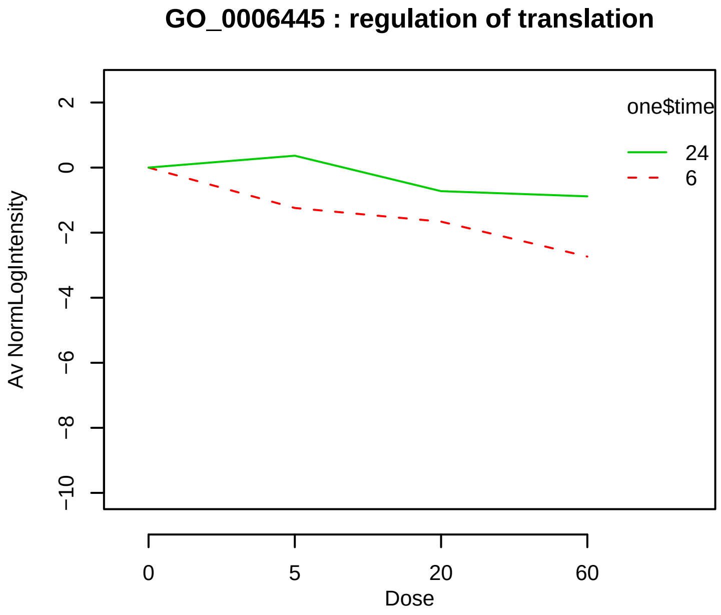
GO_0006445 : regulation of translation
Av NormLogIntensity
Dose
one$time
24
6

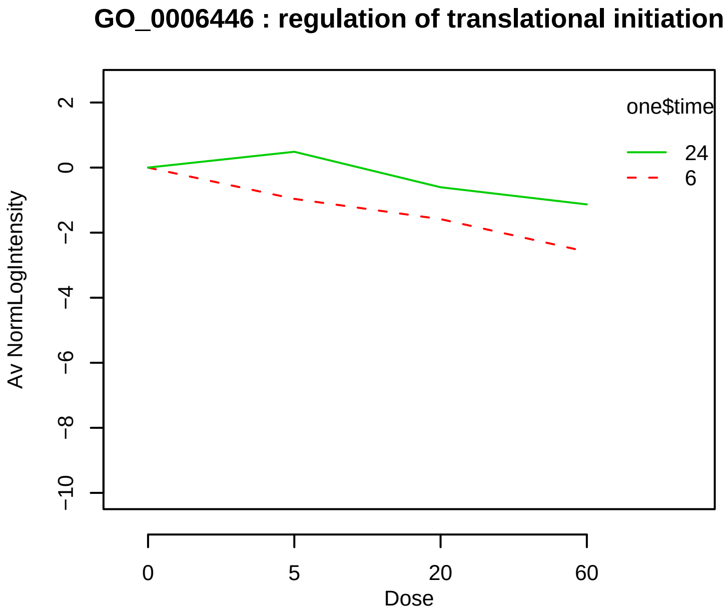
GO_0006446 : regulation of translational initiation
Av NormLogIntensity
Dose
one$time
24
6

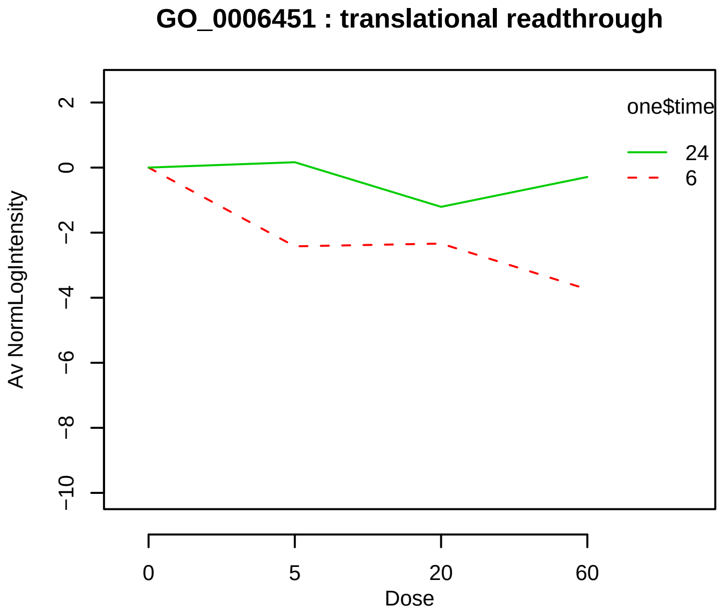
GO_0006451 : translational readthrough
one$time
24
6
Av NormLogIntensity
2
0
−2
−4
−6
−8
−10
0
5
20
60
Dose

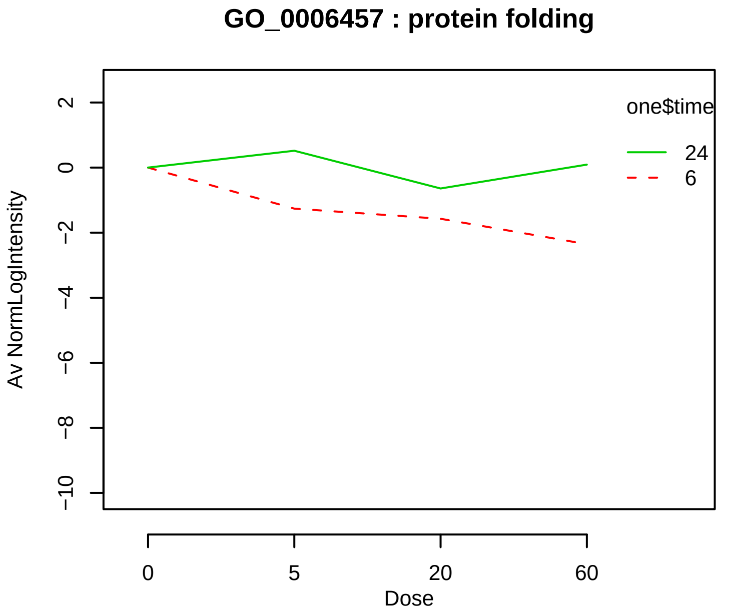
GO_0006457 : protein folding
one$time
24
6
Av NormLogIntensity
2
0
−2
−4
−6
−8
−10
0
5
20
60
Dose

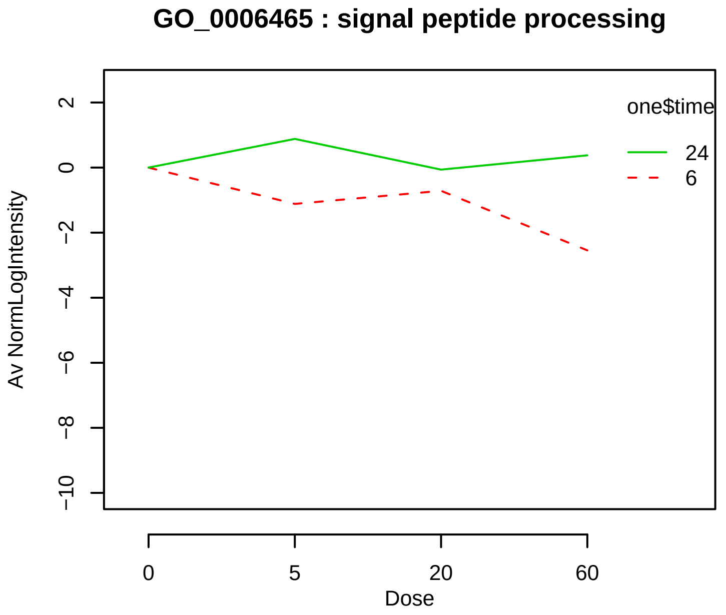
GO_0006465 : signal peptide processing
one$time
24
6
Av NormLogIntensity
2
0
−2
−4
−6
−8
−10
0
5
20
60
Dose

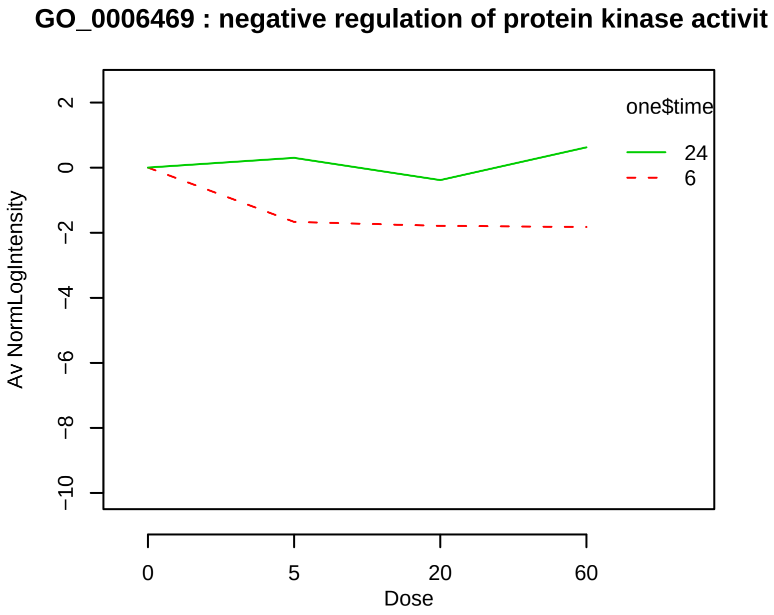
GO_0006469 : negative regulation of protein kinase activit
one$time
24
6
Av NormLogIntensity
2
0
−2
−4
−6
−8
−10
0
5
20
60
Dose

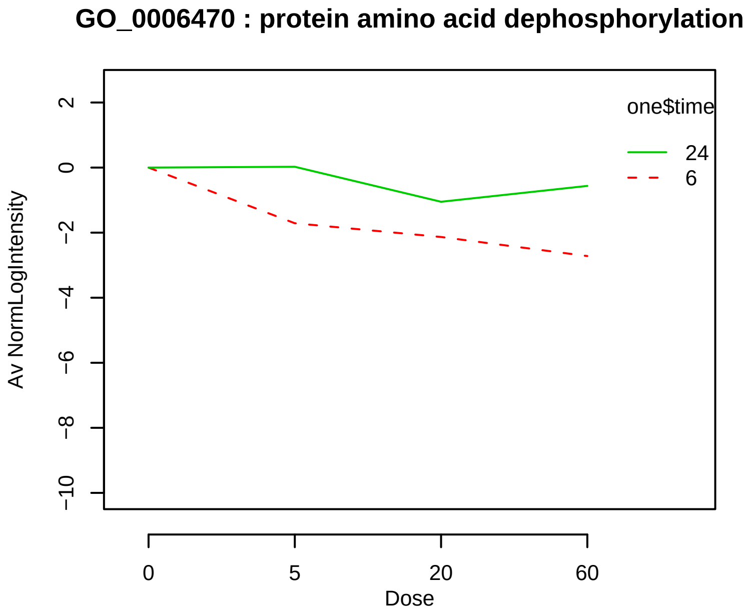
GO_0006470 : protein amino acid dephosphorylation
one$time
24
6
Av NormLogIntensity
2
0
−2
−4
−6
−8
−10
0
5
20
60
Dose

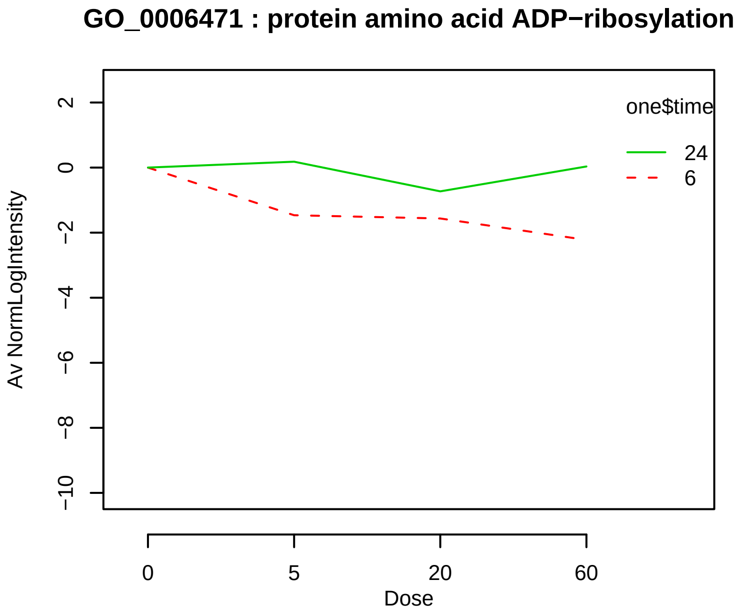
GO_0006471 : protein amino acid ADP−ribosylation
one$time
24
6
Av NormLogIntensity
2
0
−2
−4
−6
−8
−10
0
5
20
60
Dose

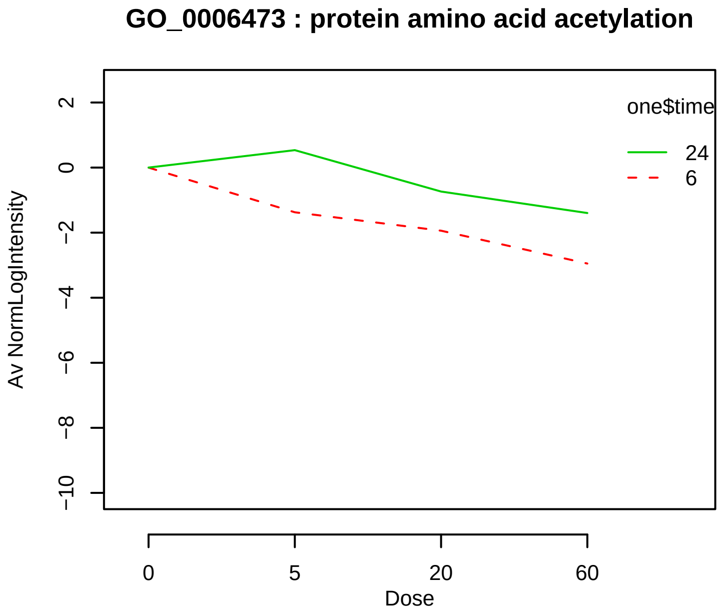
GO_0006473 : protein amino acid acetylation
one$time
24
6
Av NormLogIntensity
Dose
0
5
20
60

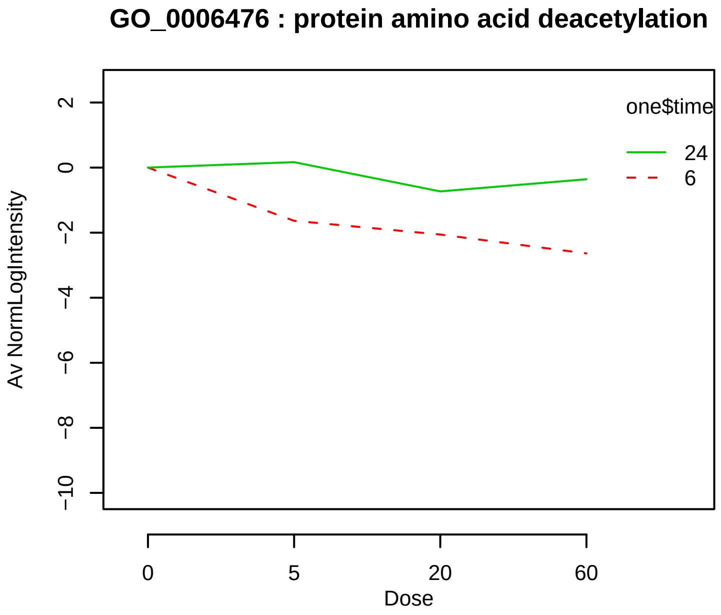
GO_0006476 : protein amino acid deacetylation
one$time
24
6
Av NormLogIntensity
Dose
0
5
20
60

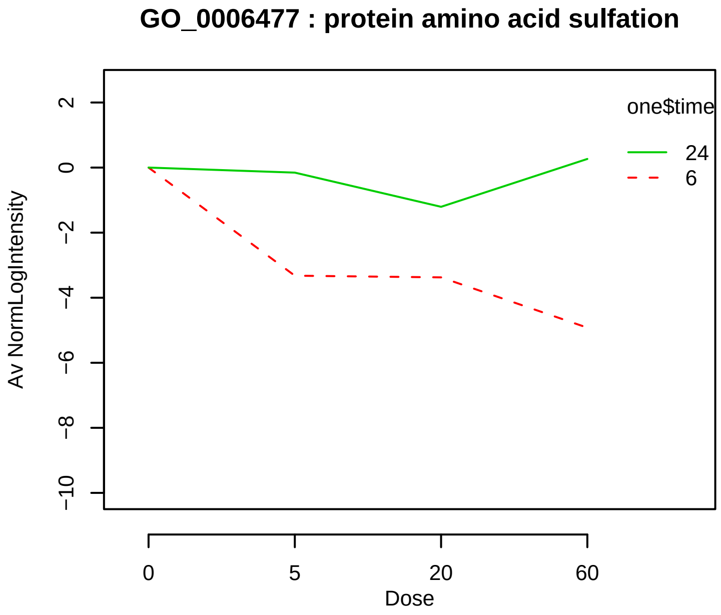
GO_0006477 : protein amino acid sulfation
one$time
24
6
Av NormLogIntensity
Dose
0
5
20
60

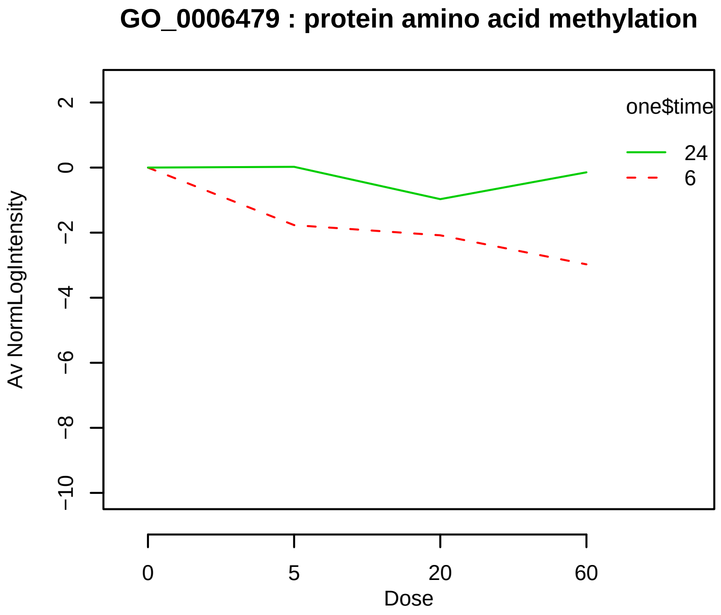
GO_0006479 : protein amino acid methylation
one$time
24
6
Av NormLogIntensity
Dose
0
5
20
60

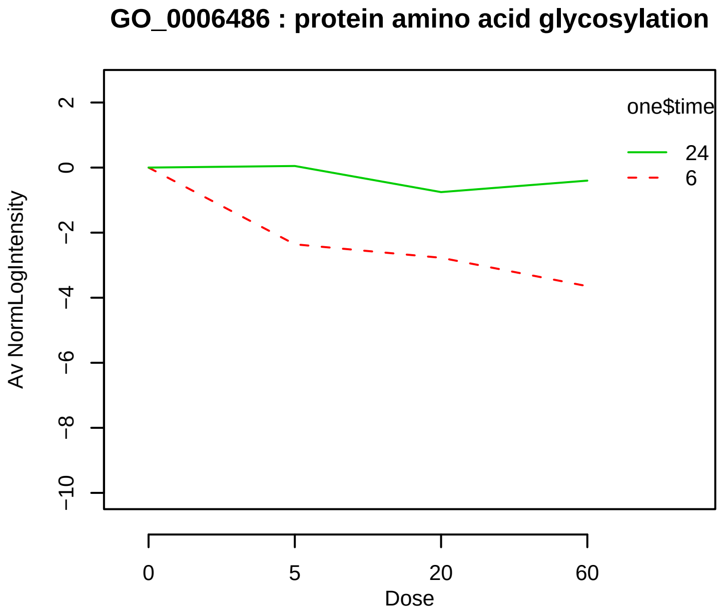
GO_0006486 : protein amino acid glycosylation
one$time
24
6
Av NormLogIntensity
Dose
0
5
20
60

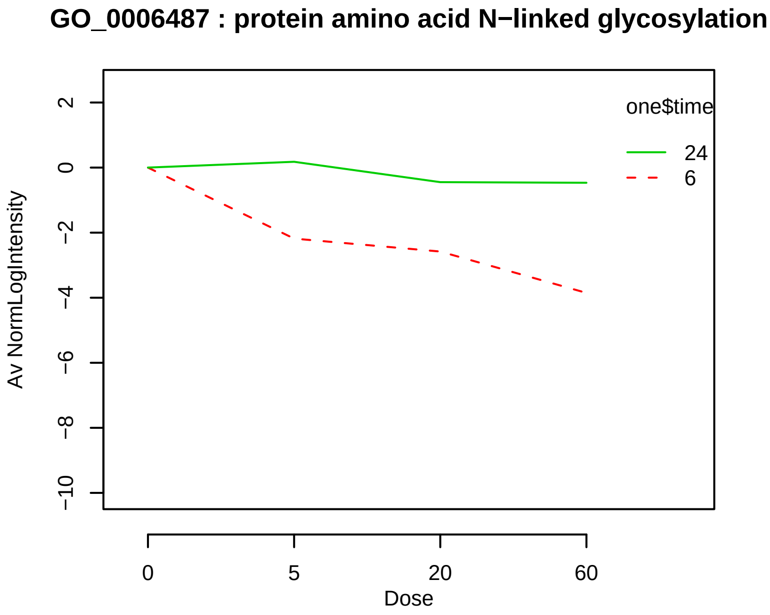
GO_0006487 : protein amino acid N−linked glycosylation
one$time
24
6
Av NormLogIntensity
Dose
0
5
20
60

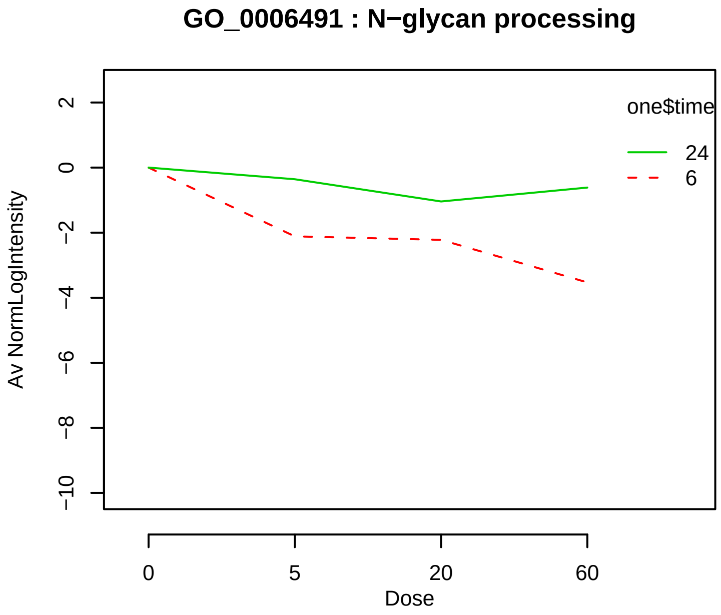
GO_0006491 : N−glycan processing
Av NormLogIntensity
2
0
−2
−4
−6
−8
−10
one$time
24
6
0
5
20
60
Dose

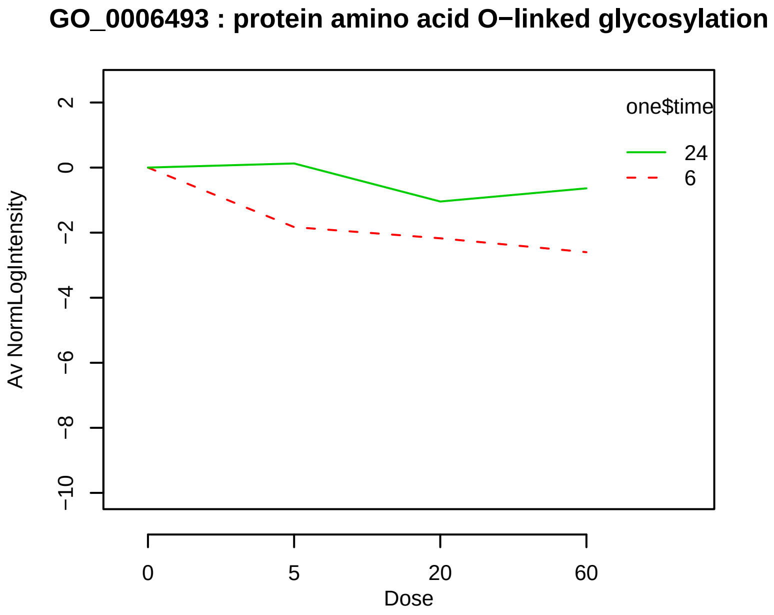
GO_0006493 : protein amino acid O−linked glycosylation
Av NormLogIntensity
2
0
−2
−4
−6
−8
−10
one$time
24
6
0
5
20
60
Dose

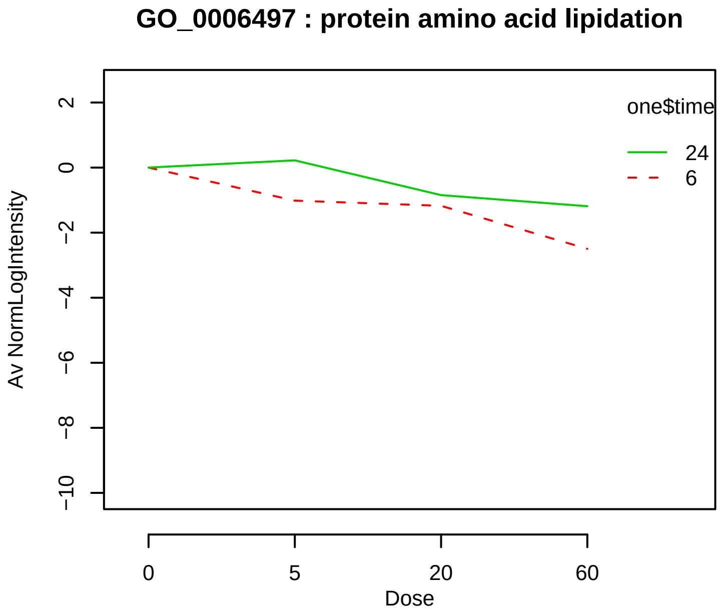
GO_0006497 : protein amino acid lipidation
Av NormLogIntensity
2
0
−2
−4
−6
−8
−10
one$time
24
6
0
5
20
60
Dose

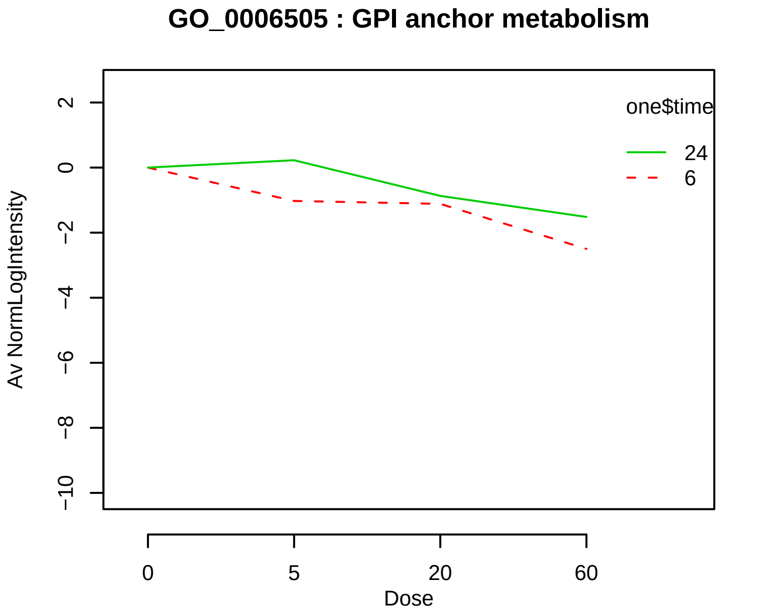
GO_0006505 : GPI anchor metabolism
Av NormLogIntensity
2
0
−2
−4
−6
−8
−10
one$time
24
6
0
5
20
60
Dose

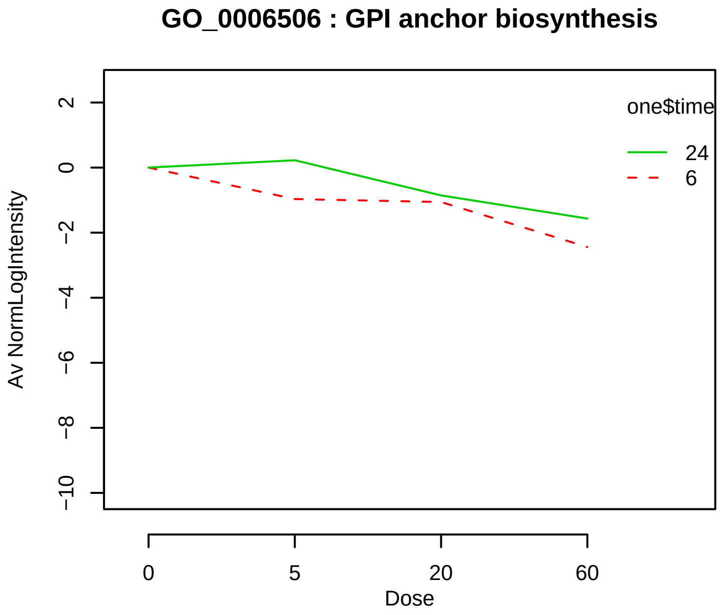
GO_0006506 : GPI anchor biosynthesis
Av NormLogIntensity
2
0
−2
−4
−6
−8
−10
one$time
24
6
0
5
20
60
Dose

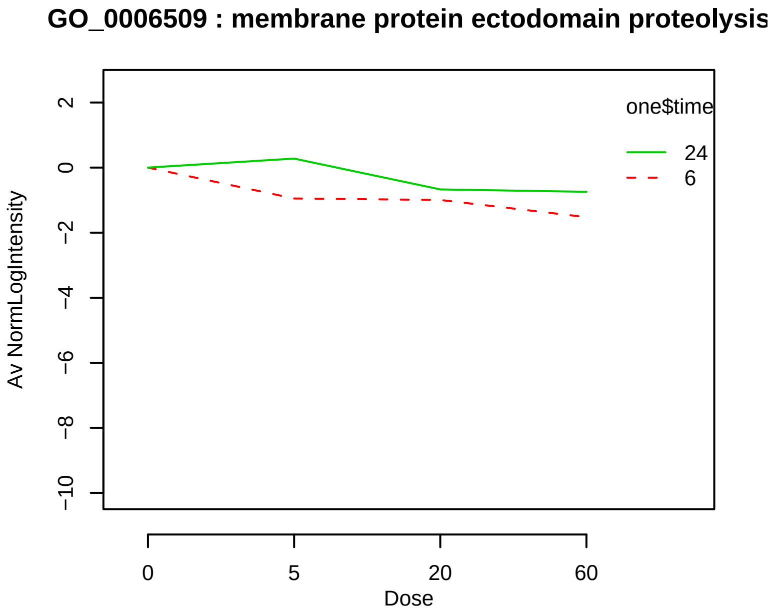
GO_0006509 : membrane protein ectodomain proteolysis
Av NormLogIntensity
2
0
−2
−4
−6
−8
−10
one$time
24
6
0
5
20
60
Dose

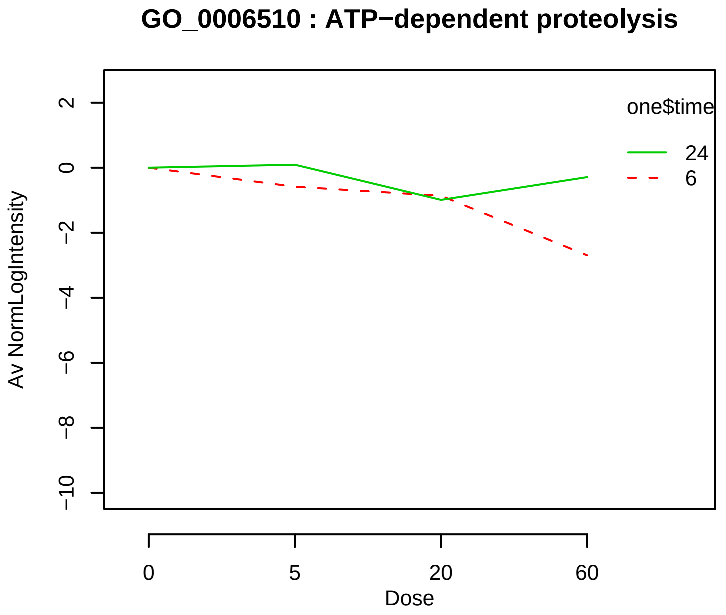
GO_0006510 : ATP−dependent proteolysis
one$time
24
6
Av NormLogIntensity
2
0
−2
−4
−6
−8
−10
0
5
20
60
Dose

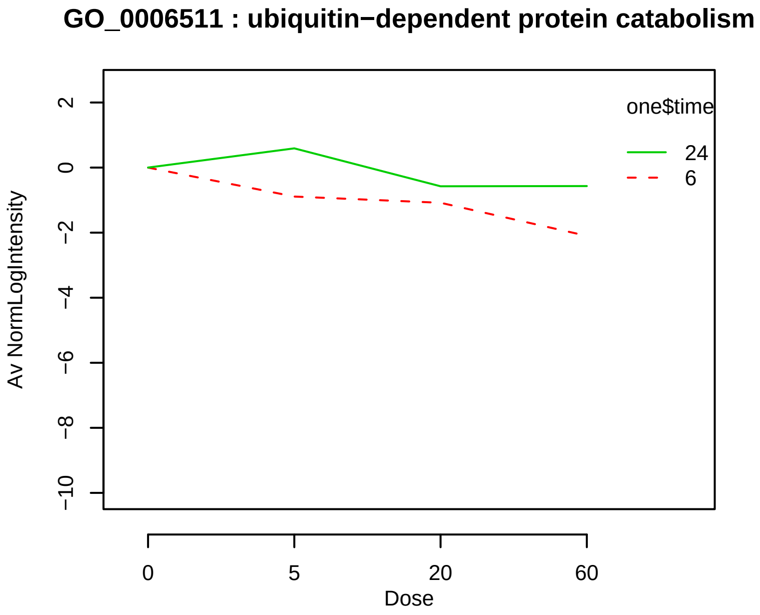
GO_0006511 : ubiquitin−dependent protein catabolism
one$time
24
6
Av NormLogIntensity
2
0
−2
−4
−6
−8
−10
0
5
20
60
Dose

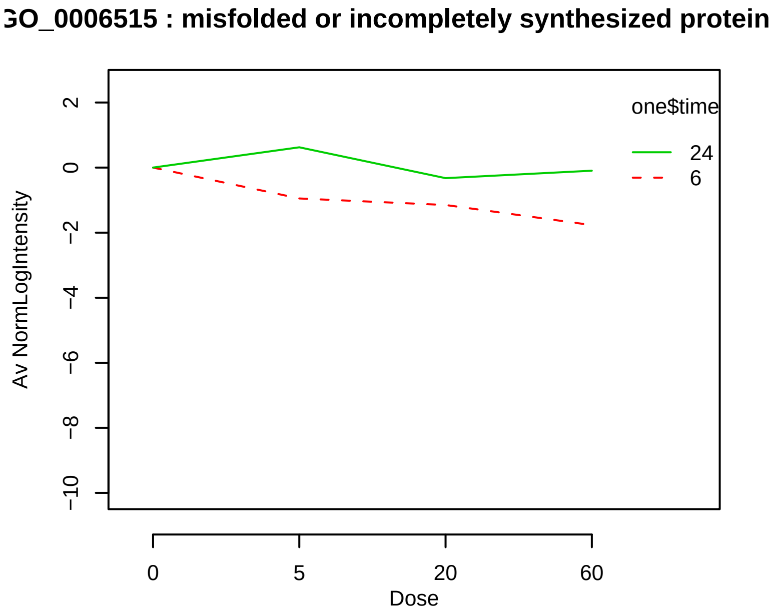
GO_0006515 : misfolded or incompletely synthesized protein
one$time
24
6
Av NormLogIntensity
2
0
−2
−4
−6
−8
−10
0
5
20
60
Dose

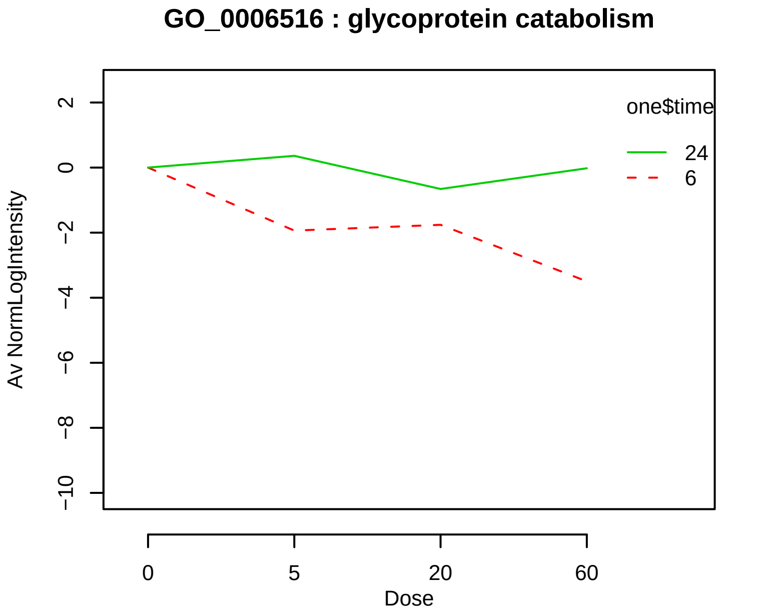
GO_0006516 : glycoprotein catabolism
one$time
24
6
Av NormLogIntensity
2
0
−2
−4
−6
−8
−10
0
5
20
60
Dose

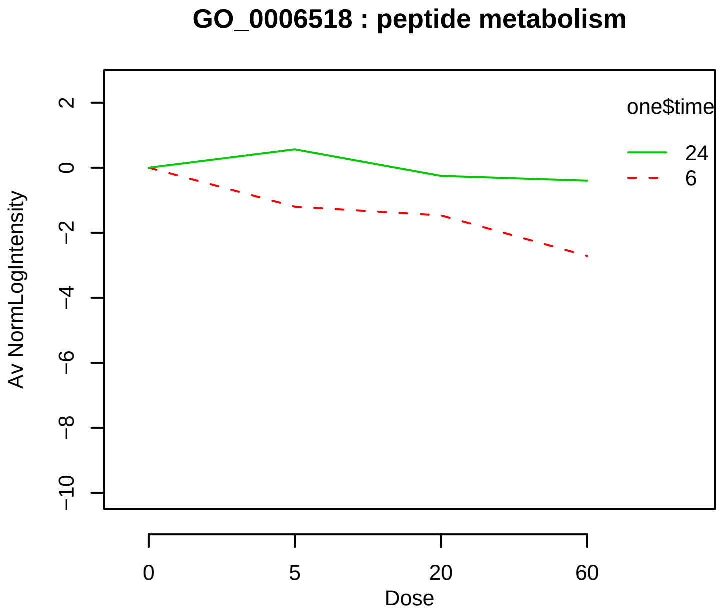
GO_0006518 : peptide metabolism
one$time
24
6
Av NormLogIntensity
2
0
−2
−4
−6
−8
−10
0
5
20
60
Dose

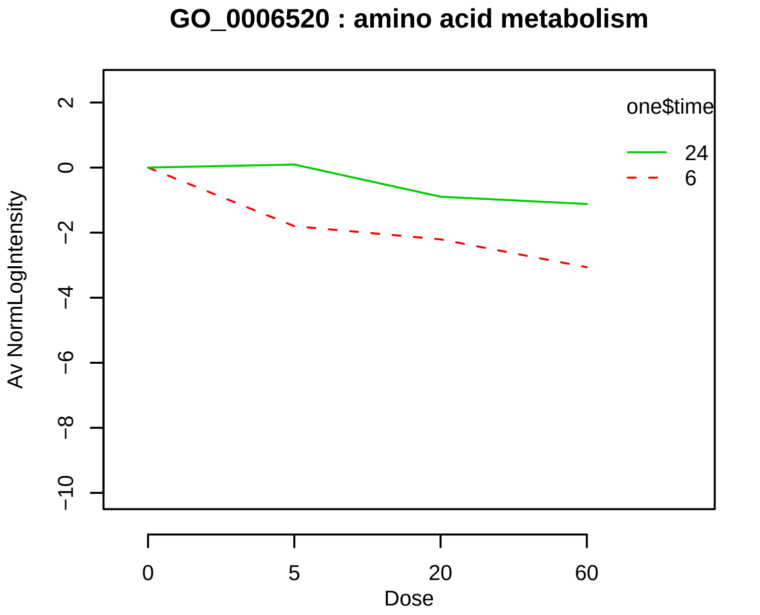
GO_0006520 : amino acid metabolism
one$time
24
6
Av NormLogIntensity
2
0
−2
−4
−6
−8
−10
0
5
20
60
Dose

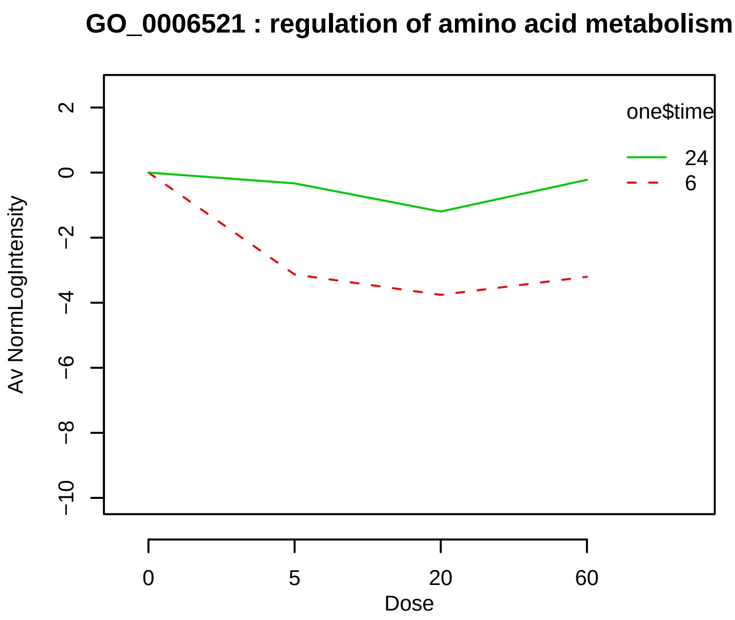
GO_0006521 : regulation of amino acid metabolism
Av NormLogIntensity
Dose
one$time
24
6
2
0
−2
−4
−6
−8
−10
0
5
20
60

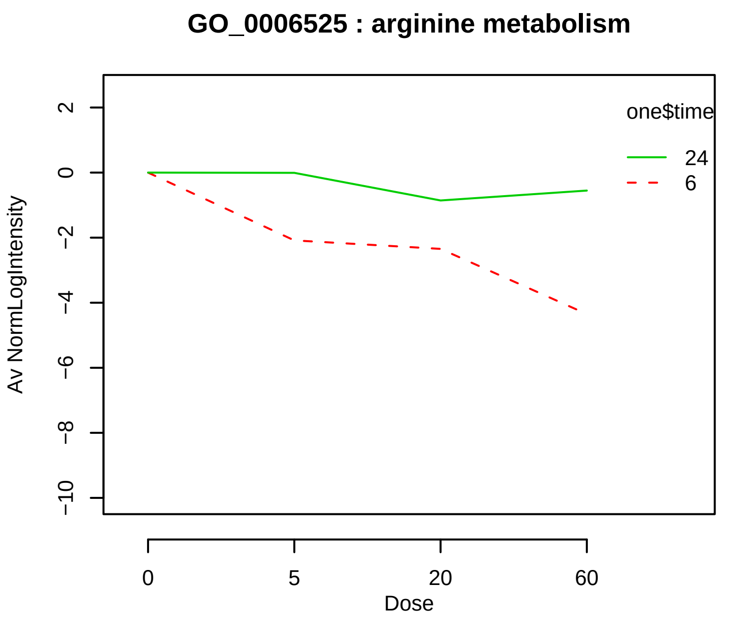
GO_0006525 : arginine metabolism
Av NormLogIntensity
Dose
one$time
24
6
2
0
−2
−4
−6
−8
−10
0
5
20
60

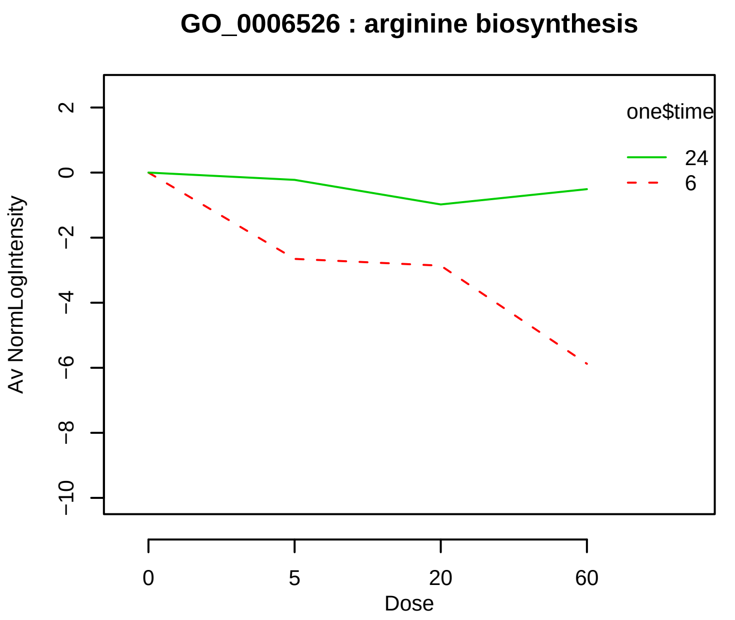
GO_0006526 : arginine biosynthesis
Av NormLogIntensity
Dose
one$time
24
6
2
0
−2
−4
−6
−8
−10
0
5
20
60

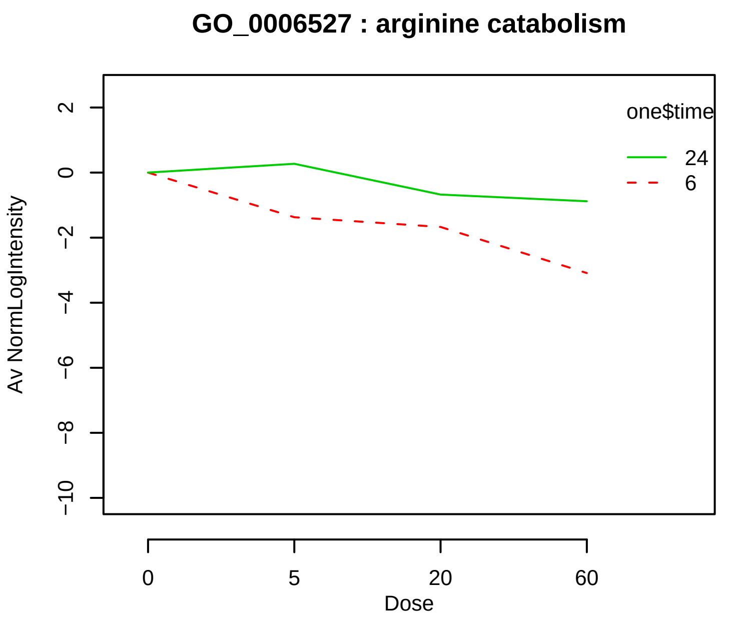
GO_0006527 : arginine catabolism
Av NormLogIntensity
Dose
one$time
24
6
2
0
−2
−4
−6
−8
−10
0
5
20
60

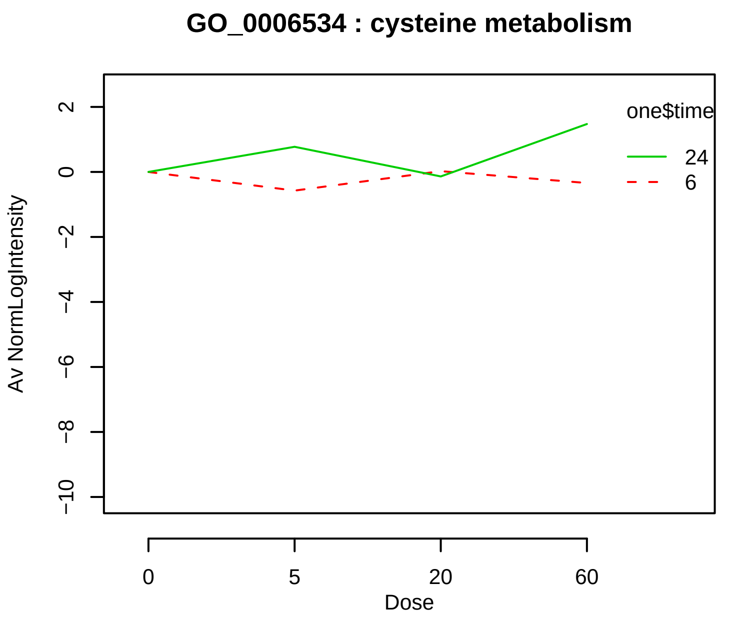
GO_0006534 : cysteine metabolism
Av NormLogIntensity
Dose
one$time
24
6
2
0
−2
−4
−6
−8
−10
0
5
20
60

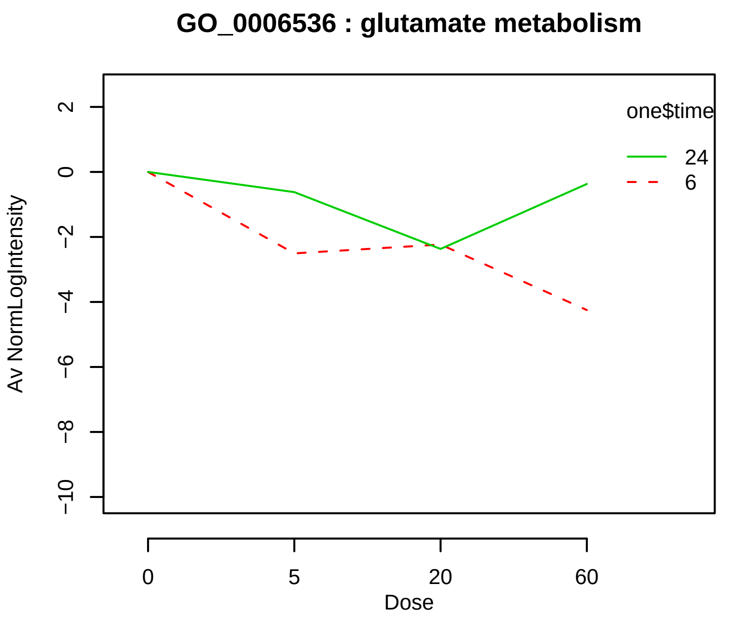
GO_0006536 : glutamate metabolism
Av NormLogIntensity
Dose
one$time
24
6
2
0
−2
−4
−6
−8
−10
0
5
20
60

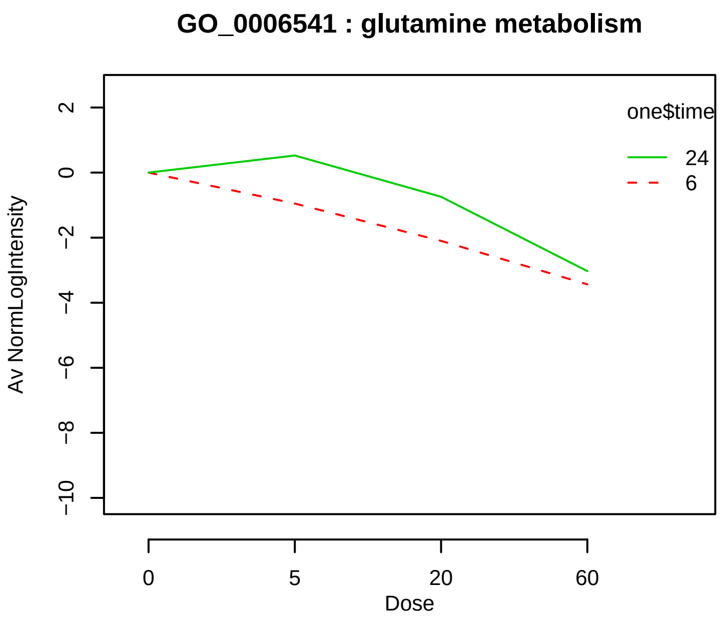

GO_0006541 : glutamine metabolism
Av NormLogIntensity
2
0
−2
−4
−6
−8
−10
one$time
24
6
0
5
20
60
Dose

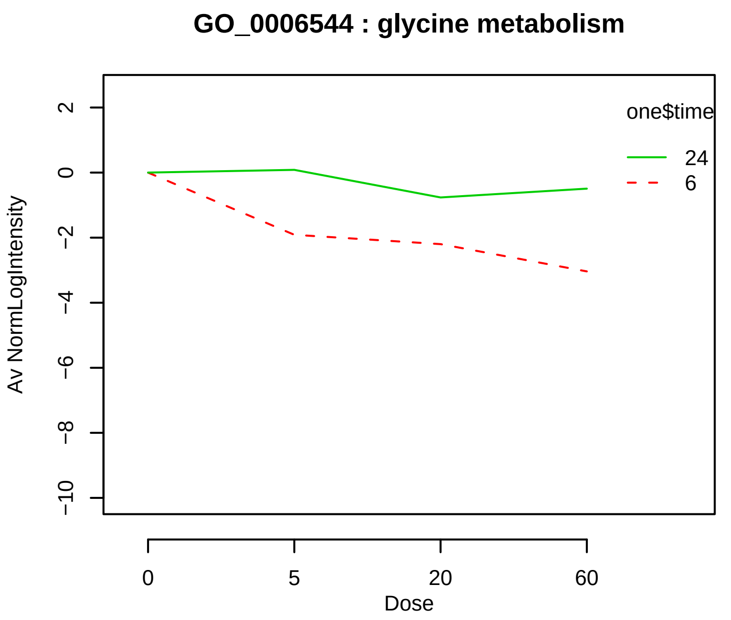

GO_0006544 : glycine metabolism
Av NormLogIntensity
2
0
−2
−4
−6
−8
−10
one$time
24
6
0
5
20
60
Dose

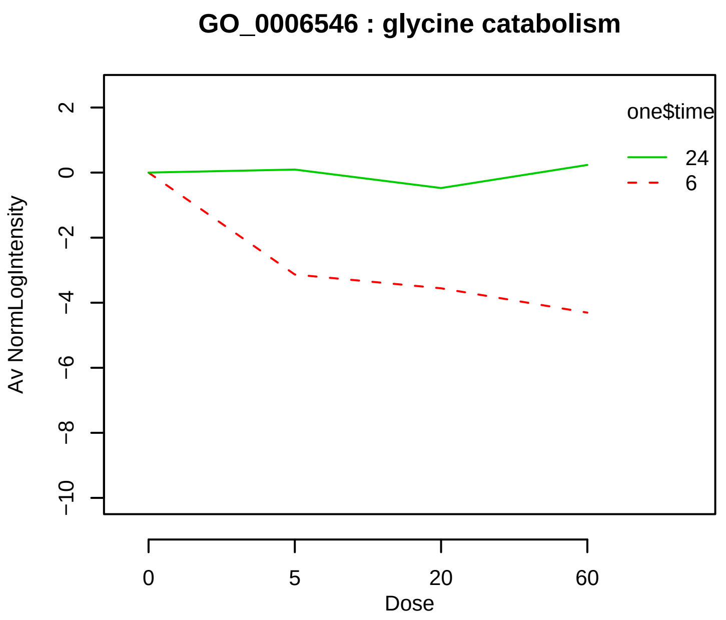

GO_0006546 : glycine catabolism
Av NormLogIntensity
2
0
−2
−4
−6
−8
−10
one$time
24
6
0
5
20
60
Dose

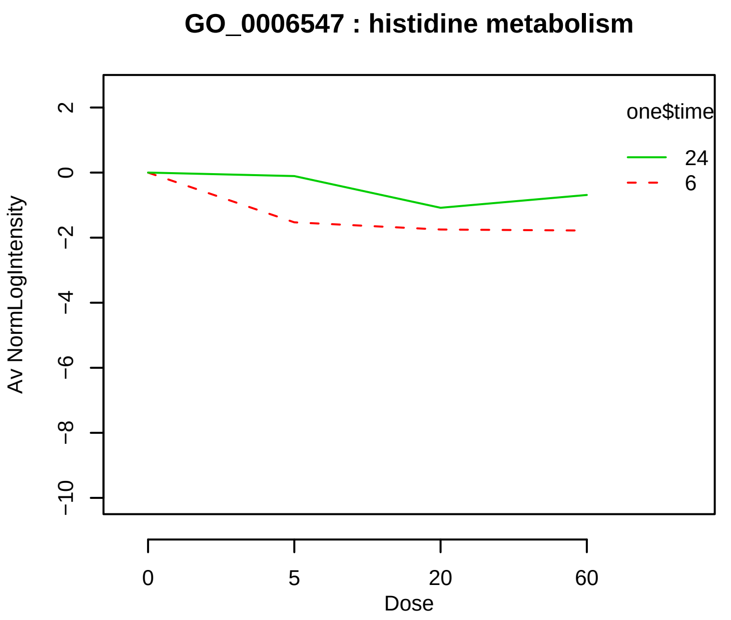

GO_0006547 : histidine metabolism
Av NormLogIntensity
2
0
−2
−4
−6
−8
−10
one$time
24
6
0
5
20
60
Dose

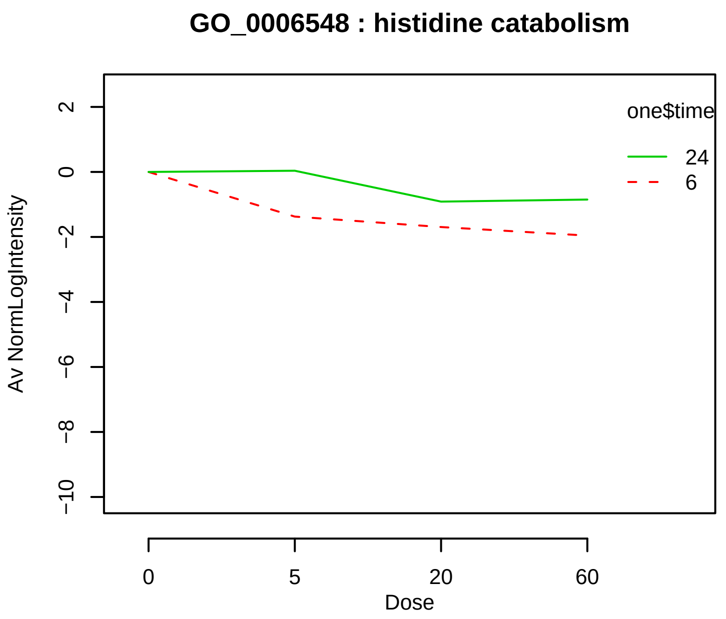

GO_0006548 : histidine catabolism
Av NormLogIntensity
2
0
−2
−4
−6
−8
−10
one$time
24
6
0
5
20
60
Dose

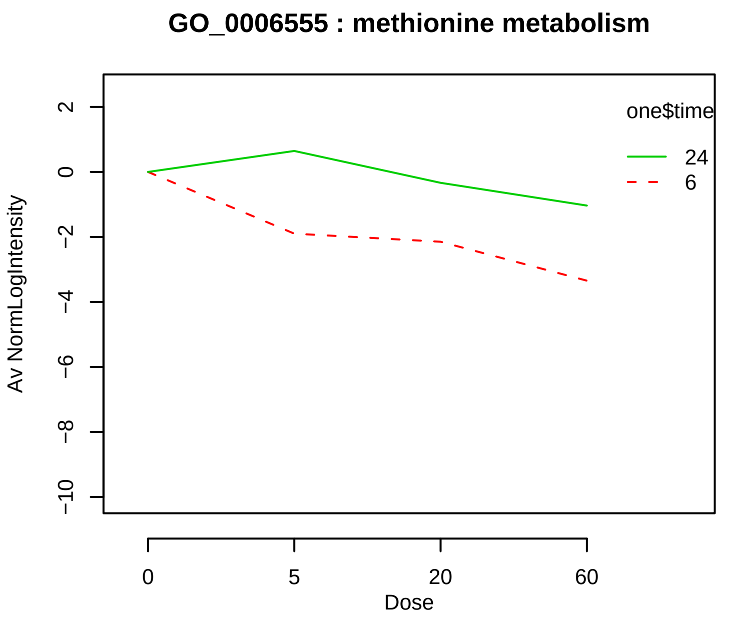

GO_0006555 : methionine metabolism
Av NormLogIntensity
2
0
−2
−4
−6
−8
−10
one$time
24
6
0
5
20
60
Dose

GO_0006558 : L−phenylalanine metabolism

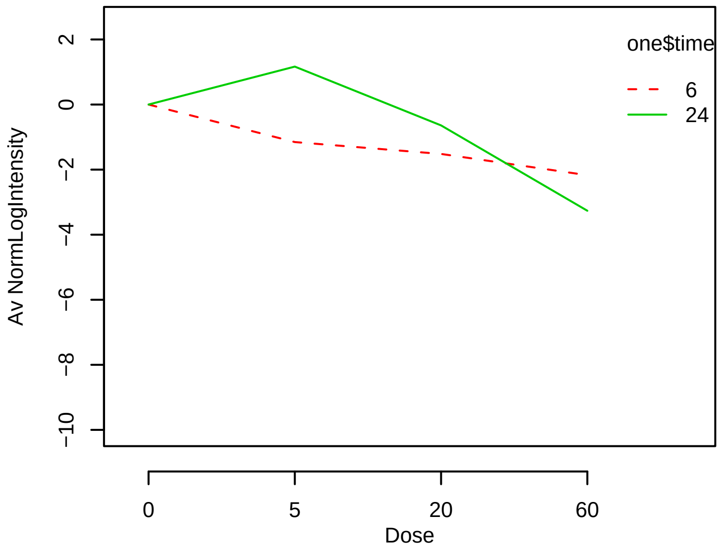

GO_0006559 : L−phenylalanine catabolism

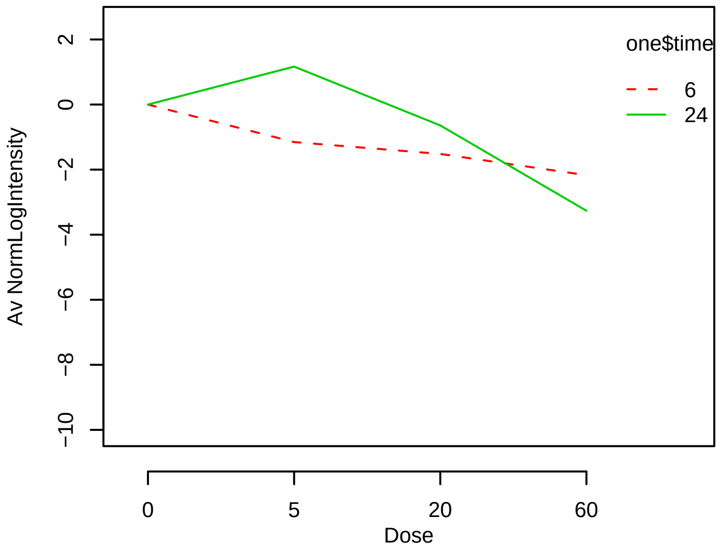

GO_0006560 : proline metabolism

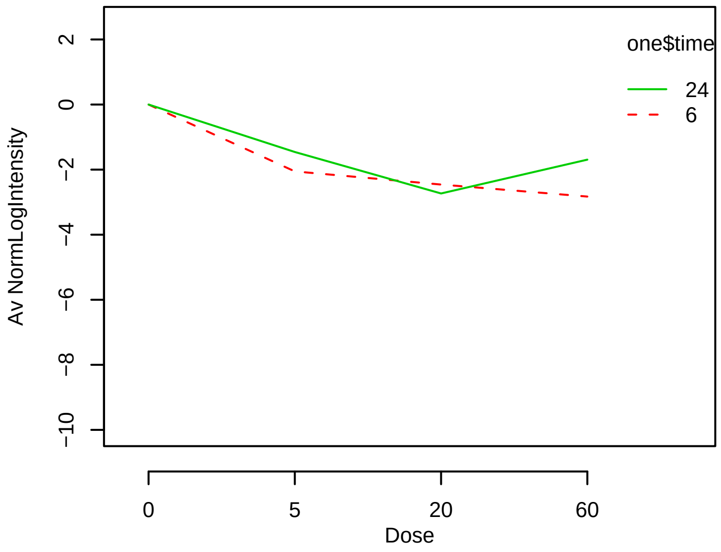

GO_0006561 : proline biosynthesis

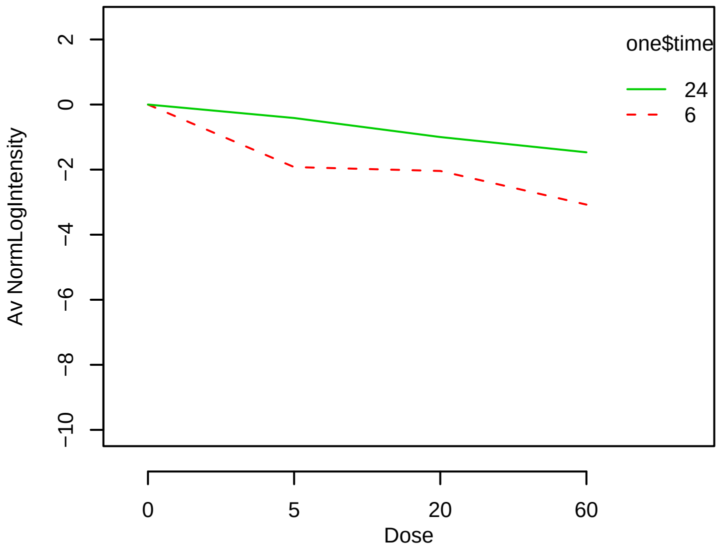

GO_0006563 : L−serine metabolism

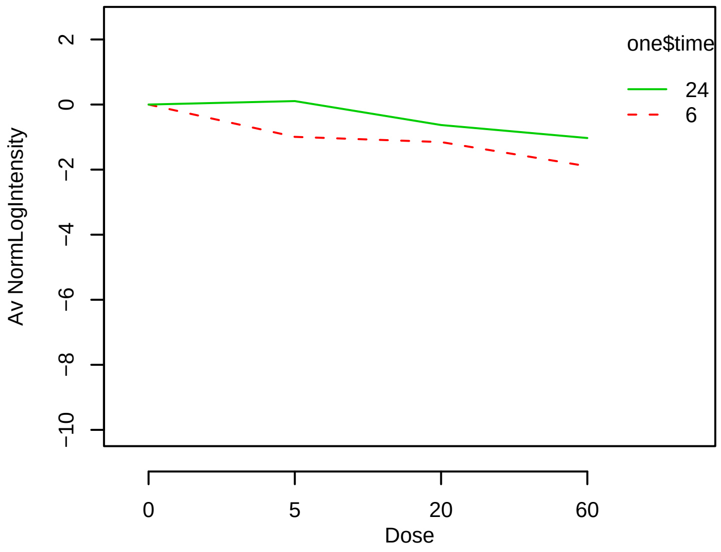

GO_0006564 : L−serine biosynthesis

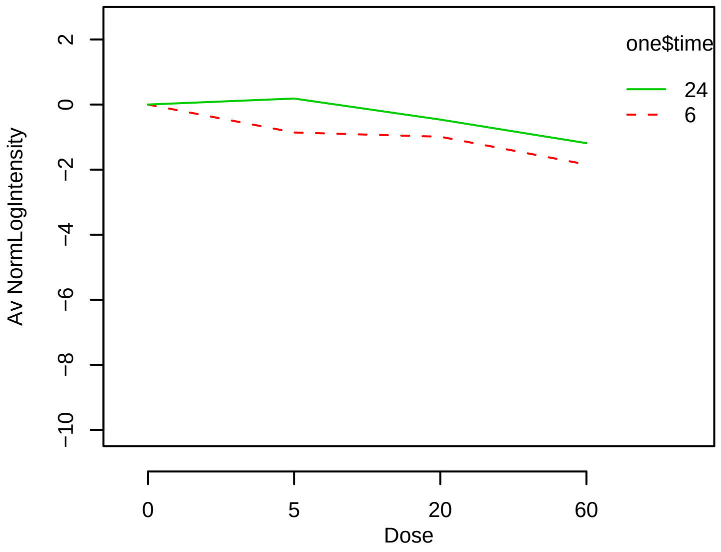

GO_0006568 : tryptophan metabolism

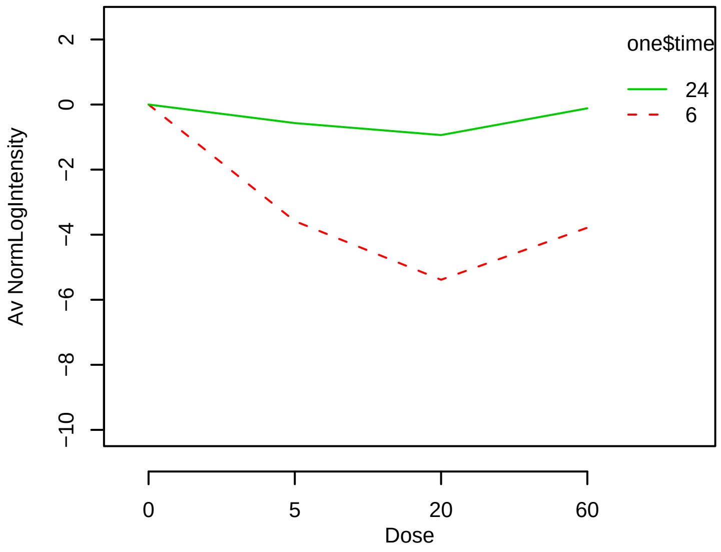

GO_0006570 : tyrosine metabolism

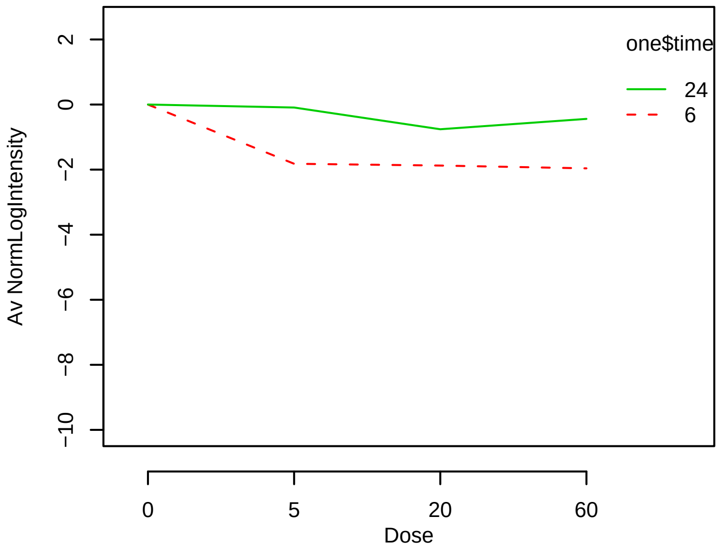

GO_0006572 : tyrosine catabolism

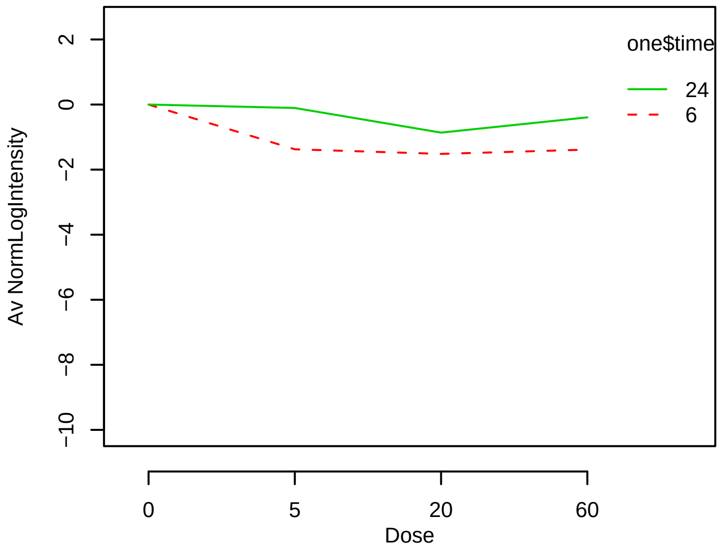

GO_0006575 : amino acid derivative metabolism

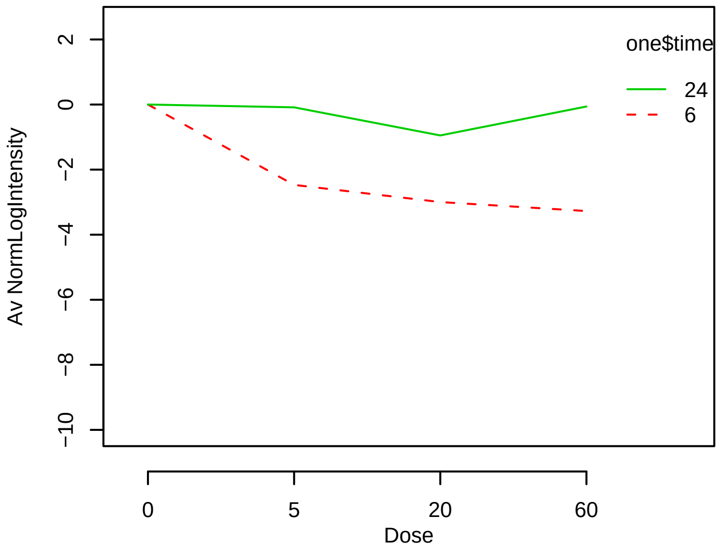

GO_0006576 : biogenic amine metabolism

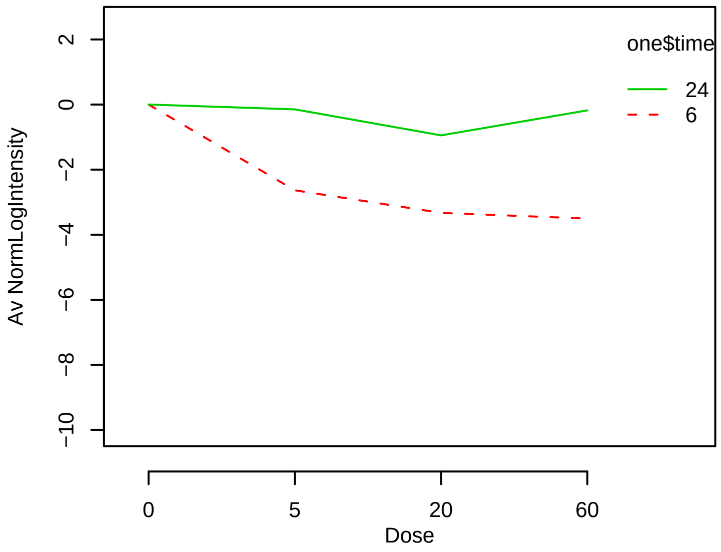

GO_0006582 : melanin metabolism

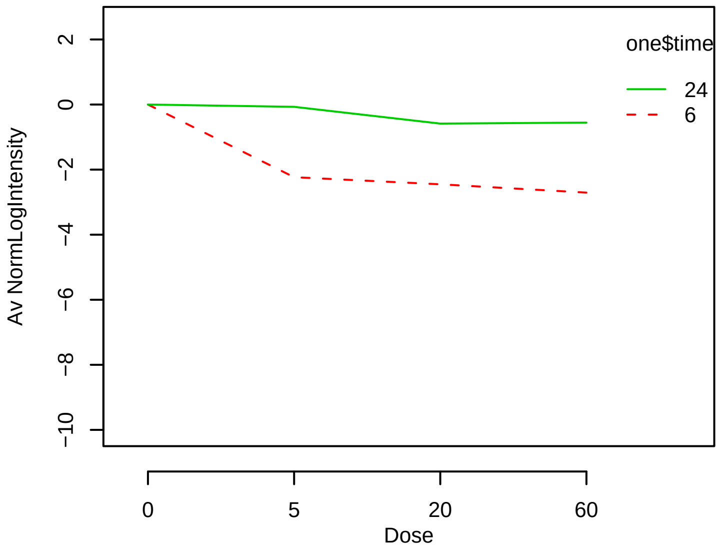

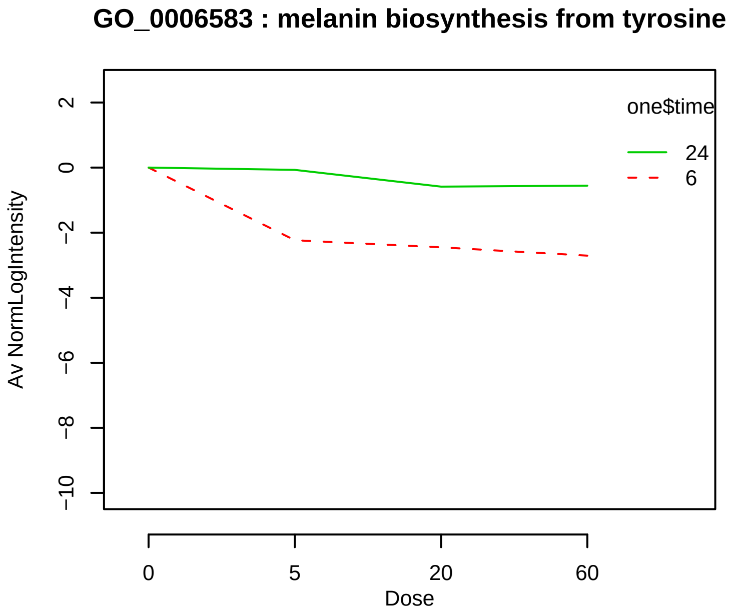
GO_0006583 : melanin biosynthesis from tyrosine
Av NormLogIntensity
2
0
−2
−4
−6
−8
−10
one$time
24
6
0
5
20
60
Dose

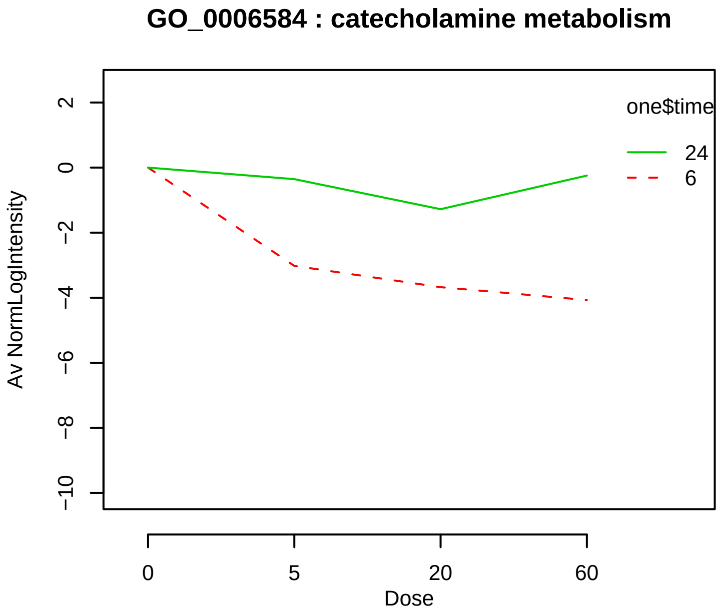
GO_0006584 : catecholamine metabolism
Av NormLogIntensity
2
0
−2
−4
−6
−8
−10
one$time
24
6
0
5
20
60
Dose

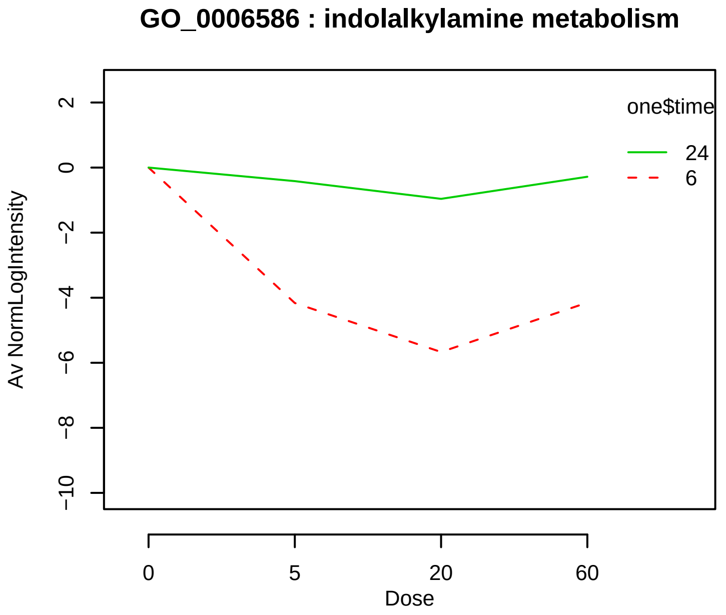
GO_0006586 : indolalkylamine metabolism
Av NormLogIntensity
2
0
−2
−4
−6
−8
−10
one$time
24
6
0
5
20
60
Dose

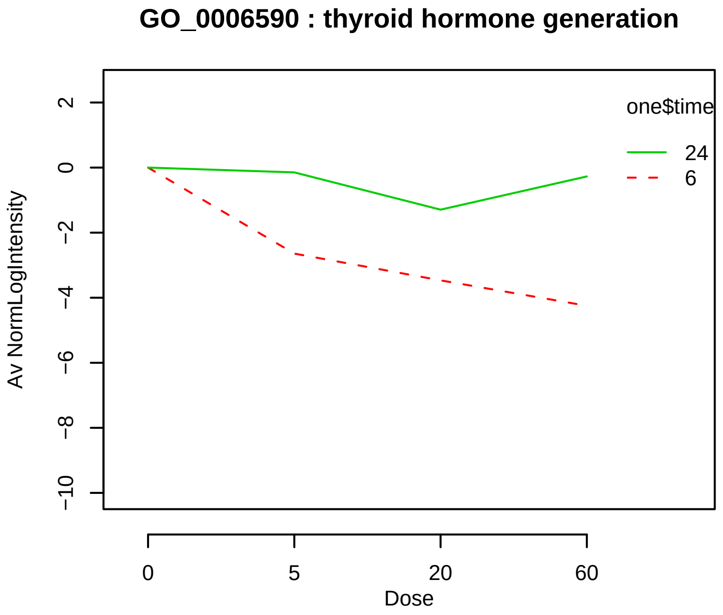
GO_0006590 : thyroid hormone generation
Av NormLogIntensity
2
0
−2
−4
−6
−8
−10
one$time
24
6
0
5
20
60
Dose

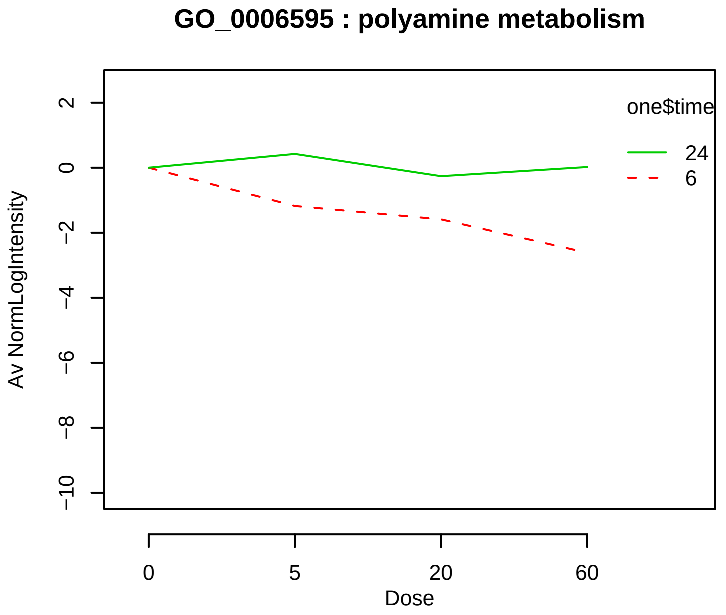
GO_0006595 : polyamine metabolism
Av NormLogIntensity
2
0
−2
−4
−6
−8
−10
one$time
24
6
0
5
20
60
Dose

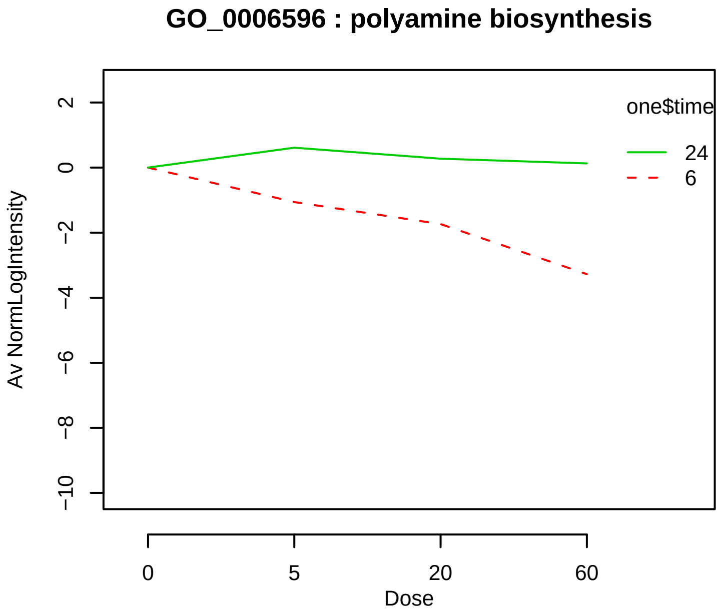
GO_0006596 : polyamine biosynthesis
Av NormLogIntensity
2
0
−2
−4
−6
−8
−10
one$time
24
6
0
5
20
60
Dose

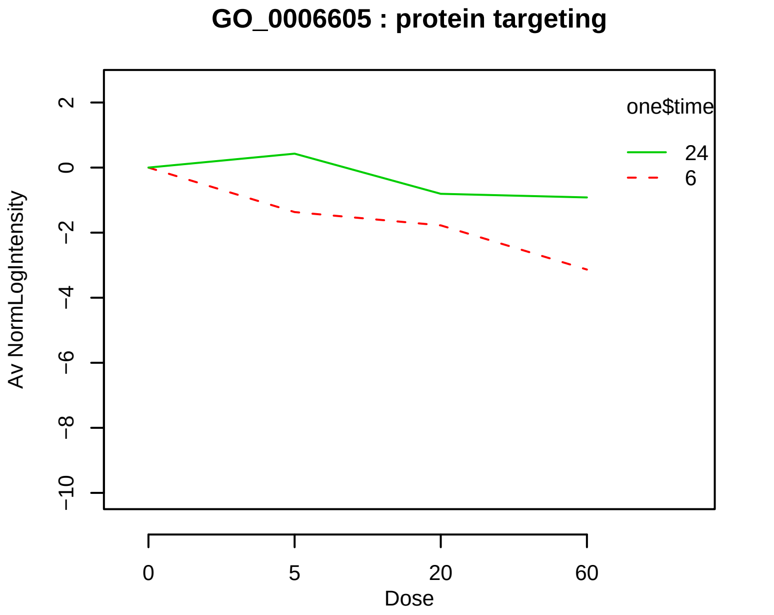
GO_0006605 : protein targeting
Av NormLogIntensity
Dose
one$time
24
6
2
0
−2
−4
−6
−8
−10
0
5
20
60

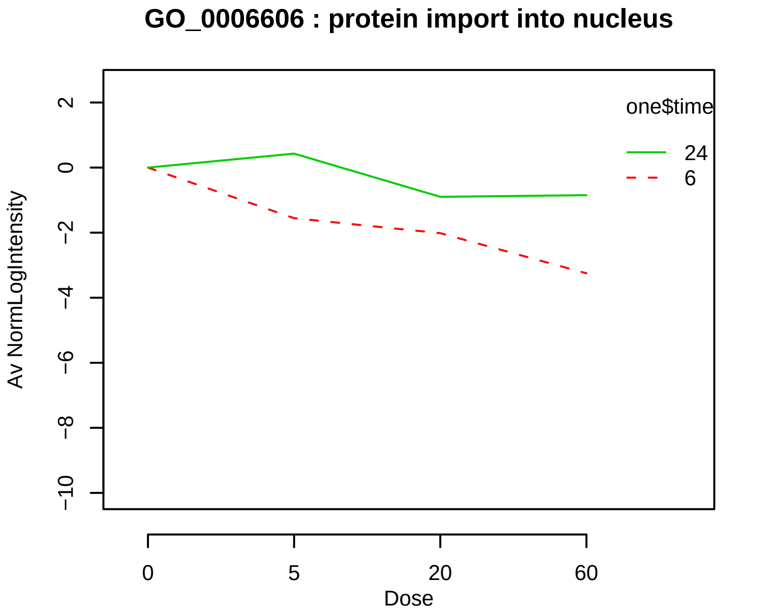
GO_0006606 : protein import into nucleus
Av NormLogIntensity
Dose
one$time
24
6
2
0
−2
−4
−6
−8
−10
0
5
20
60

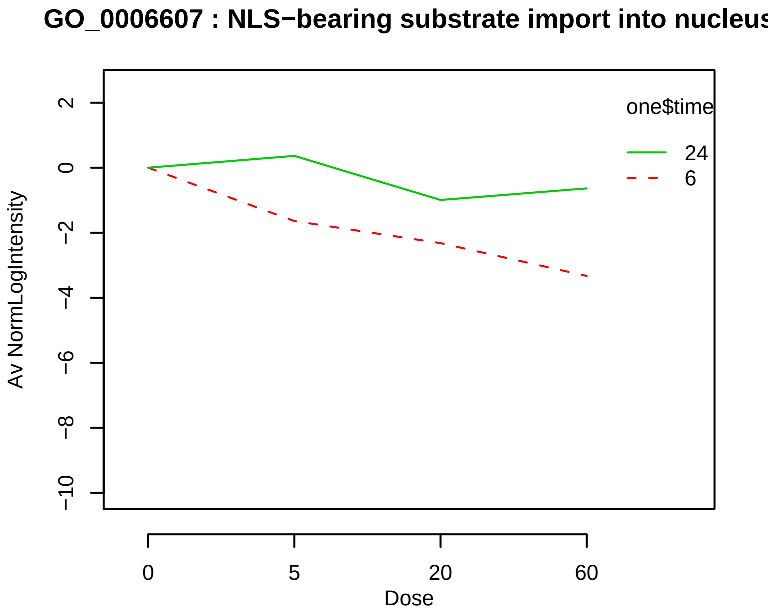
GO_0006607 : NLS−bearing substrate import into nucleus
Av NormLogIntensity
Dose
one$time
24
6
2
0
−2
−4
−6
−8
−10
0
5
20
60

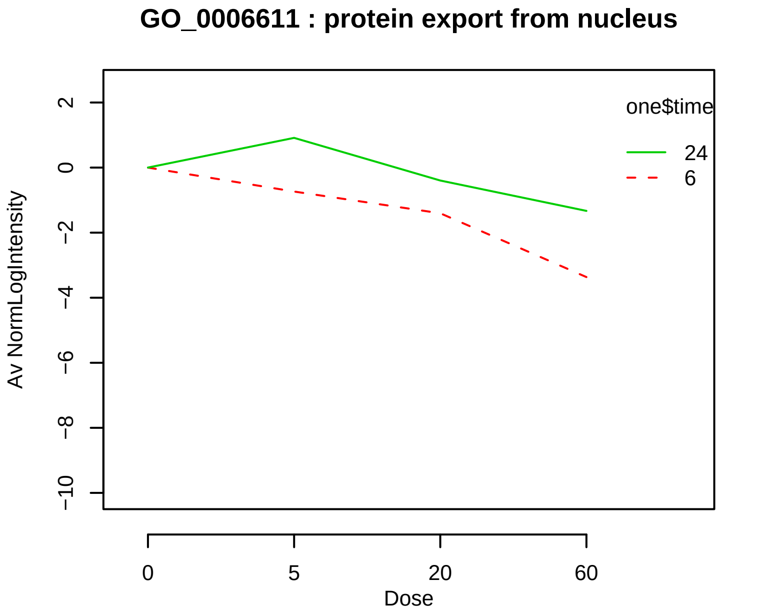
GO_0006611 : protein export from nucleus
Av NormLogIntensity
Dose
one$time
24
6
2
0
−2
−4
−6
−8
−10
0
5
20
60

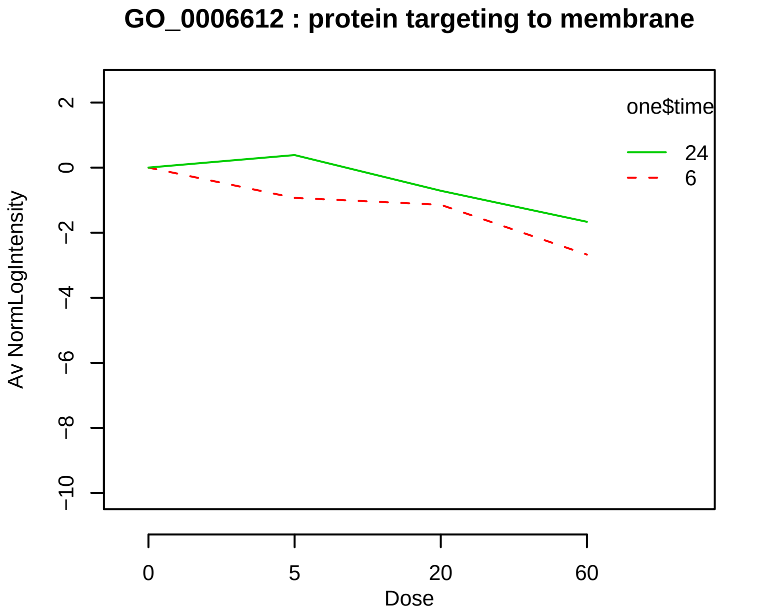
GO_0006612 : protein targeting to membrane
Av NormLogIntensity
Dose
one$time
24
6
2
0
−2
−4
−6
−8
−10
0
5
20
60

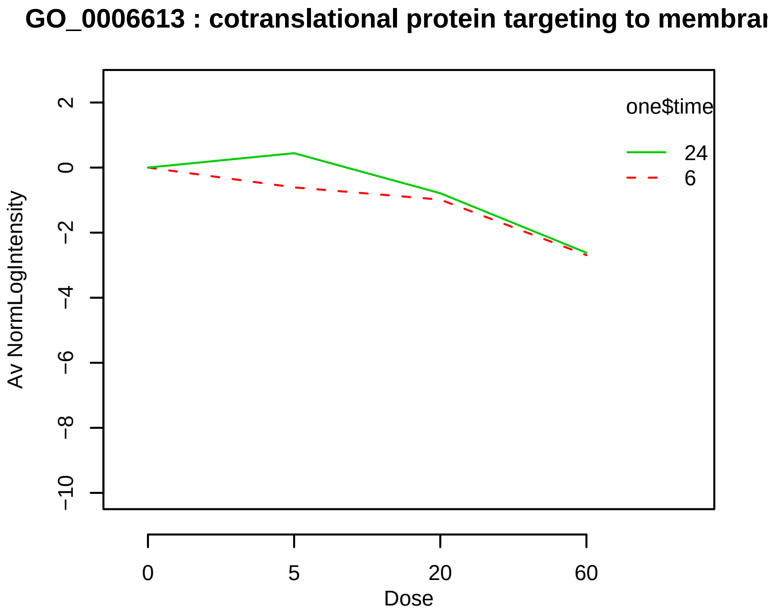
GO_0006613 : cotranslational protein targeting to membra
Av NormLogIntensity
Dose
one$time
24
6
2
0
−2
−4
−6
−8
−10
0
5
20
60

GO_0006621 : protein retention in ER

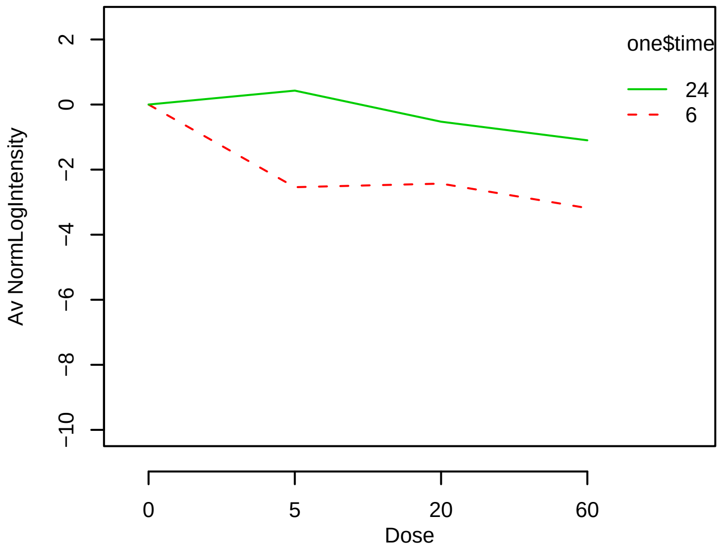

GO_0006625 : protein targeting to peroxisome

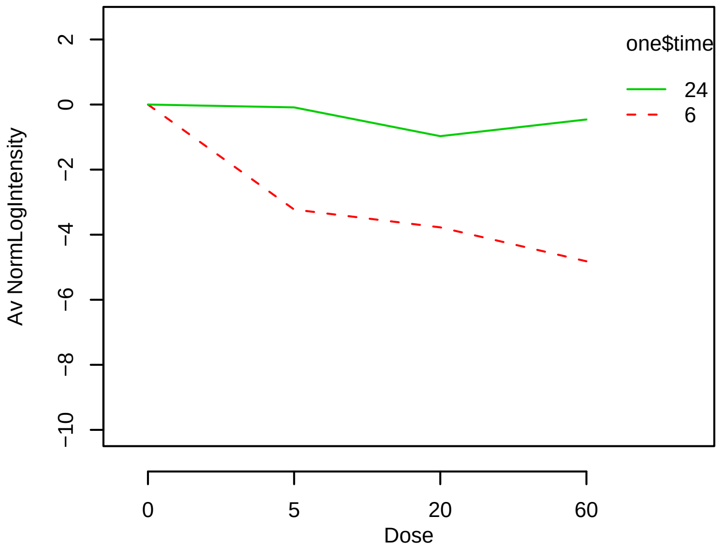

GO_0006626 : protein targeting to mitochondrion

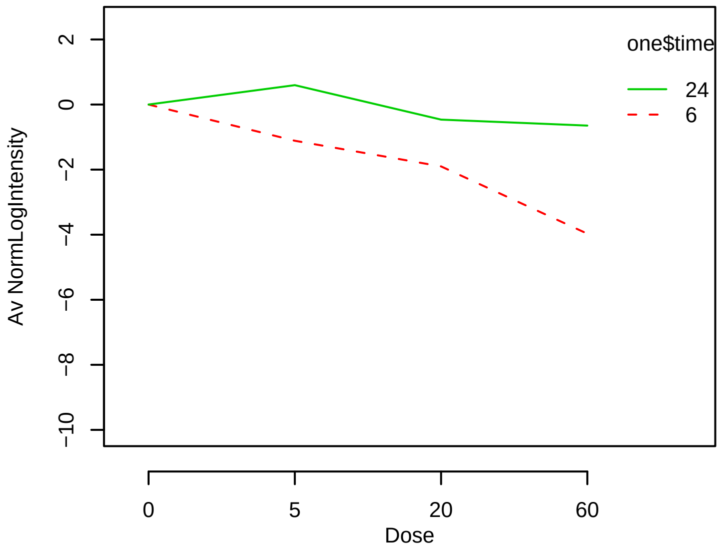

GO_0006631 : fatty acid metabolism

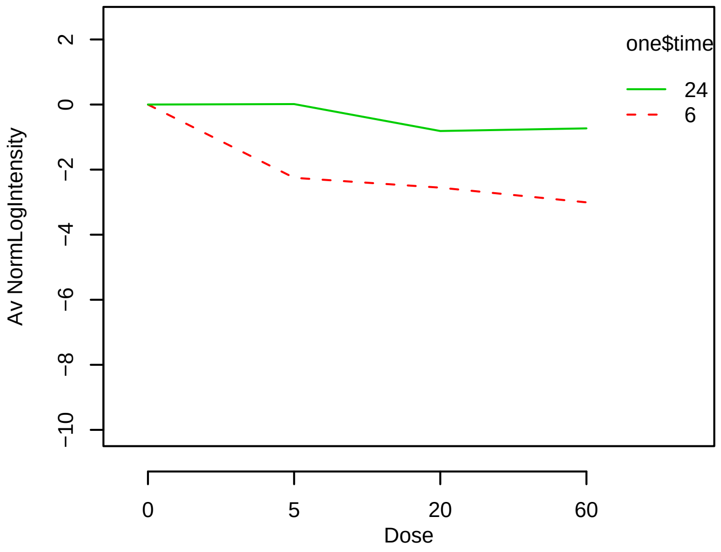

GO_0006633 : fatty acid biosynthesis

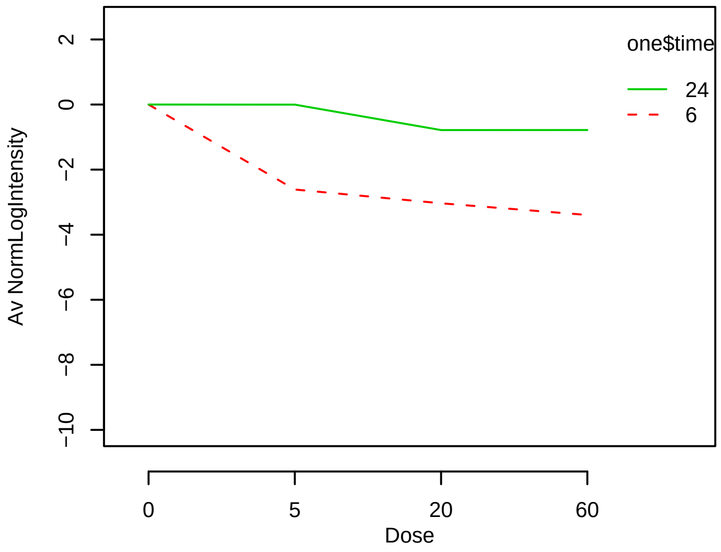

GO_0006635 : fatty acid beta−oxidation

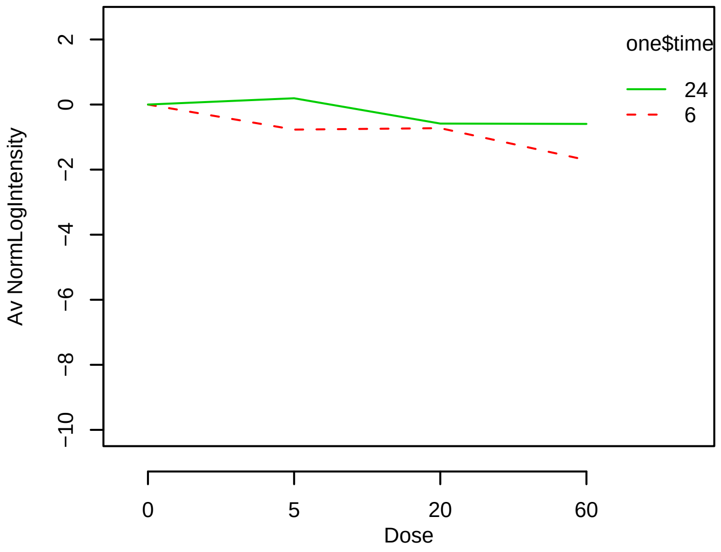

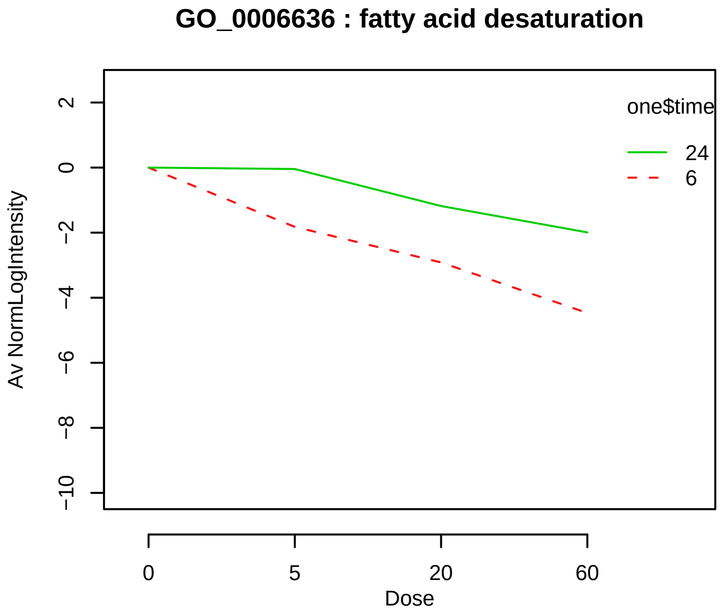
GO_0006636 : fatty acid desaturation
one$time
24
6
Av NormLogIntensity
2
0
−2
−4
−6
−8
−10
0
5
20
60
Dose

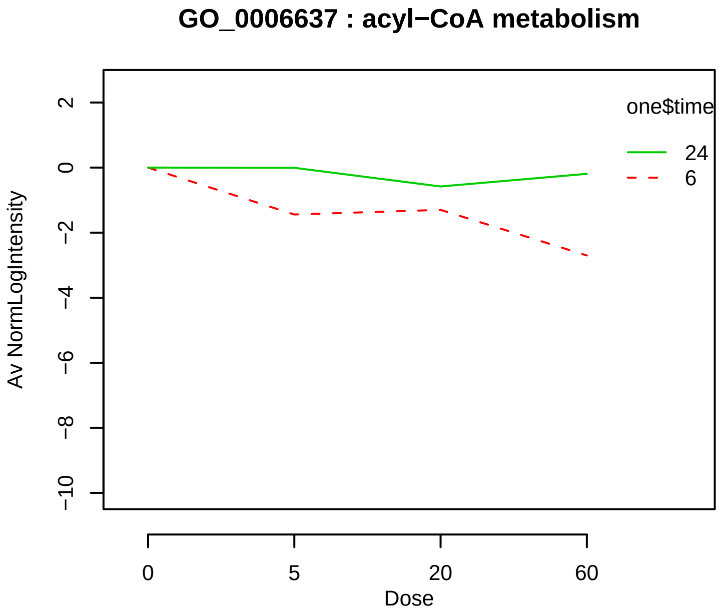
GO_0006637 : acyl−CoA metabolism
one$time
24
6
Av NormLogIntensity
2
0
−2
−4
−6
−8
−10
0
5
20
60
Dose

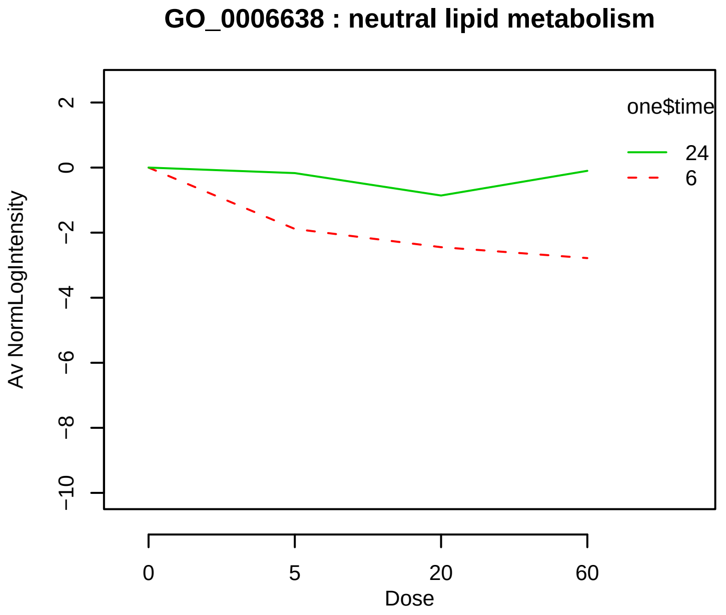
GO_0006638 : neutral lipid metabolism
one$time
24
6
Av NormLogIntensity
2
0
−2
−4
−6
−8
−10
0
5
20
60
Dose

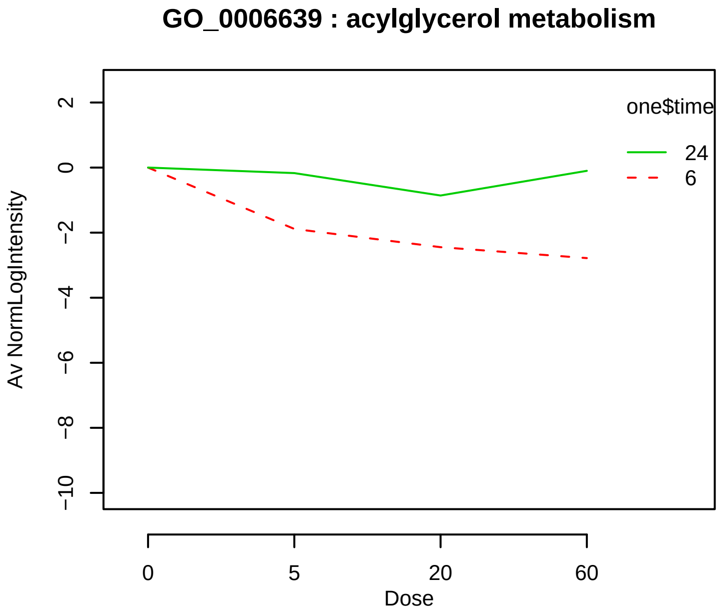
GO_0006639 : acylglycerol metabolism
one$time
24
6
Av NormLogIntensity
2
0
−2
−4
−6
−8
−10
0
5
20
60
Dose

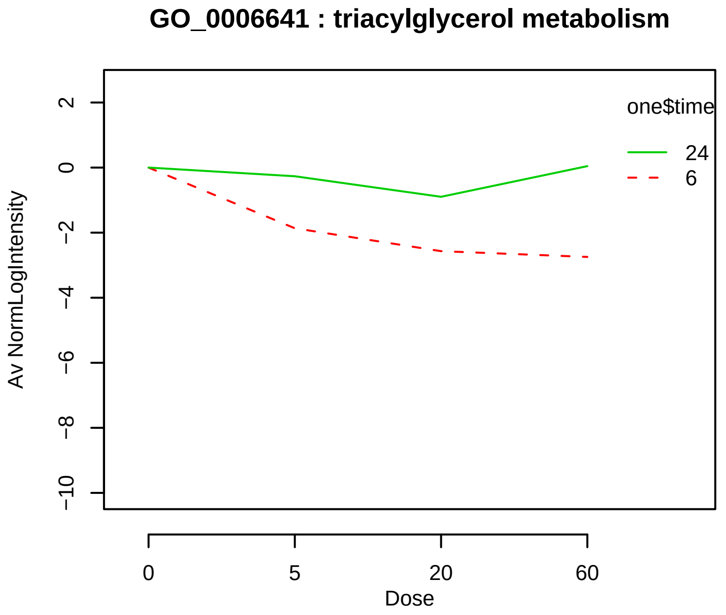
GO_0006641 : triacylglycerol metabolism
one$time
24
6
Av NormLogIntensity
2
0
−2
−4
−6
−8
−10
0
5
20
60
Dose

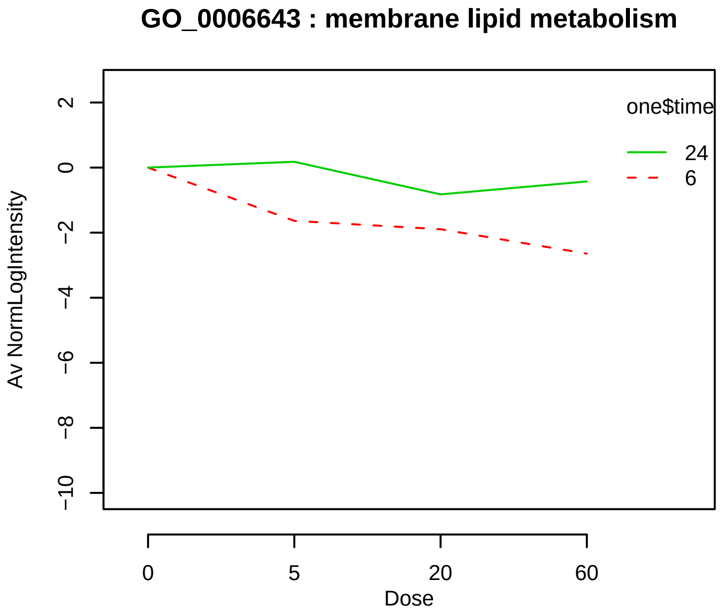
GO_0006643 : membrane lipid metabolism
one$time
24
6
Av NormLogIntensity
2
0
−2
−4
−6
−8
−10
0
5
20
60
Dose

GO_0006644 : phospholipid metabolism

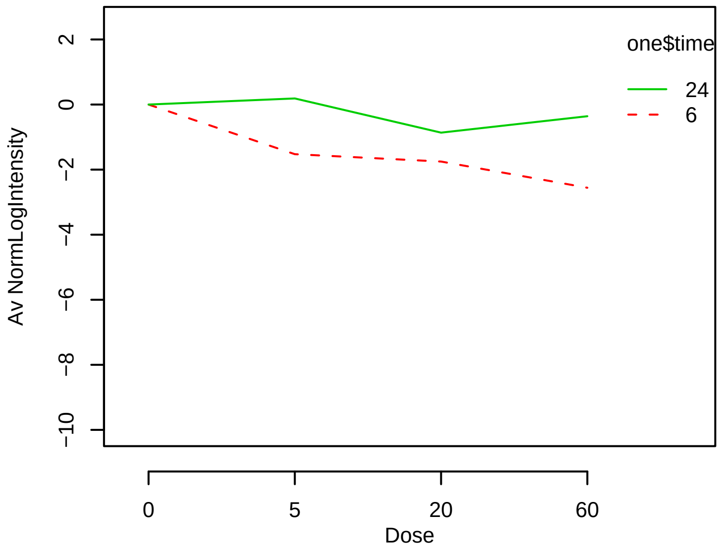

GO_0006650 : glycerophospholipid metabolism

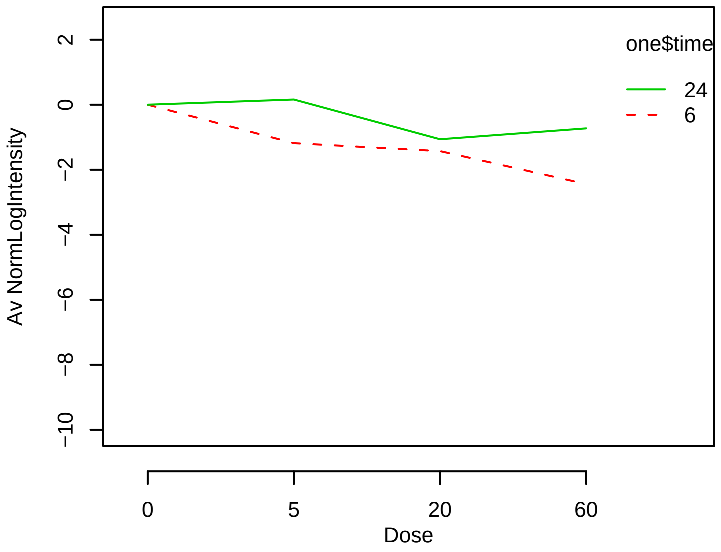

GO_0006656 : phosphatidylcholine biosynthesis

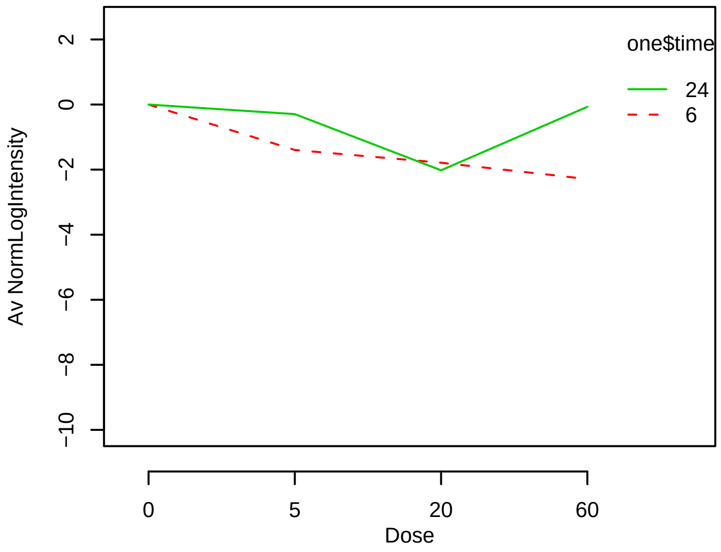

GO_0006661 : phosphatidylinositol biosynthesis

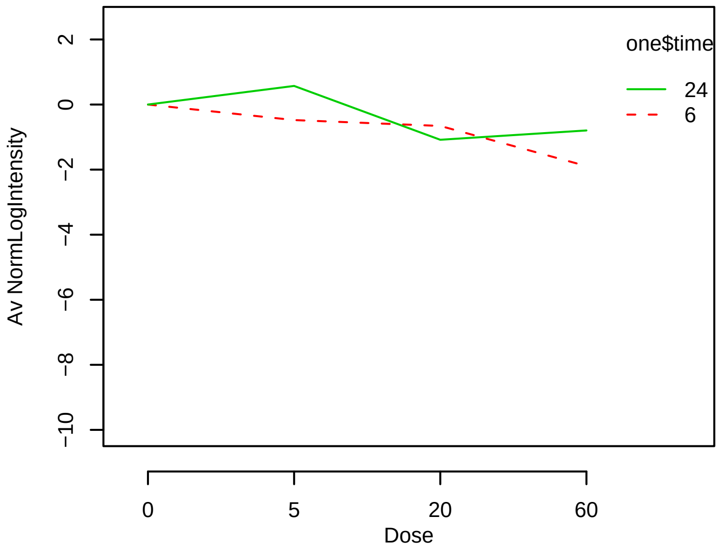

GO_0006662 : glycerol ether metabolism

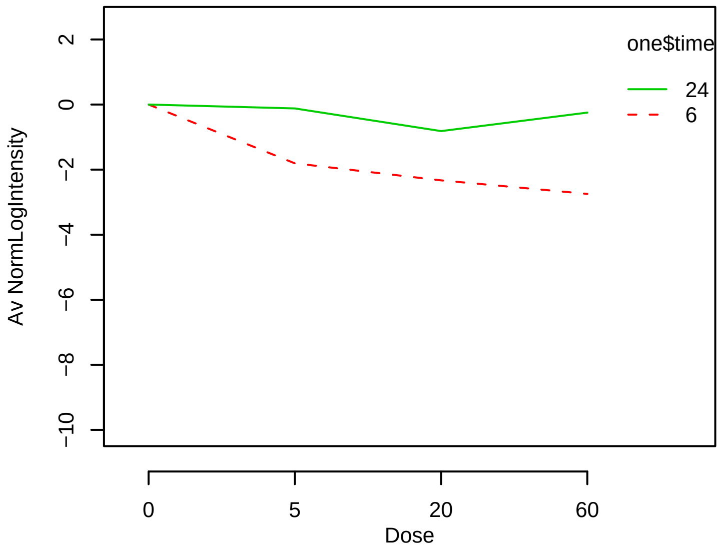

GO_0006664 : glycolipid metabolism

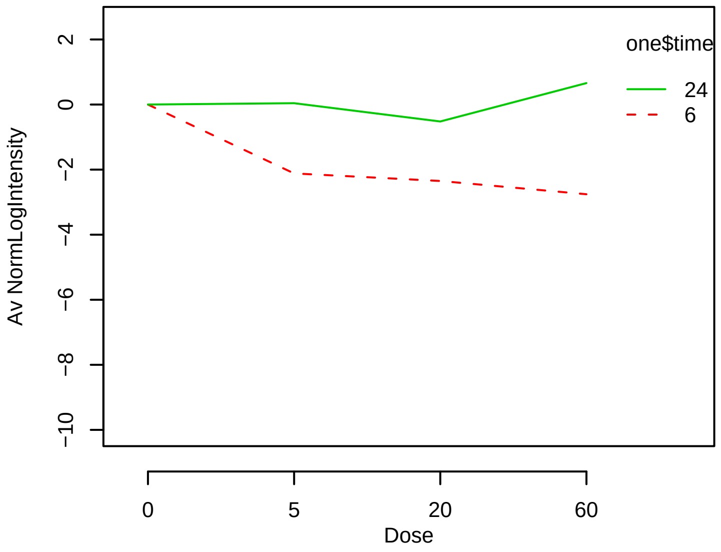

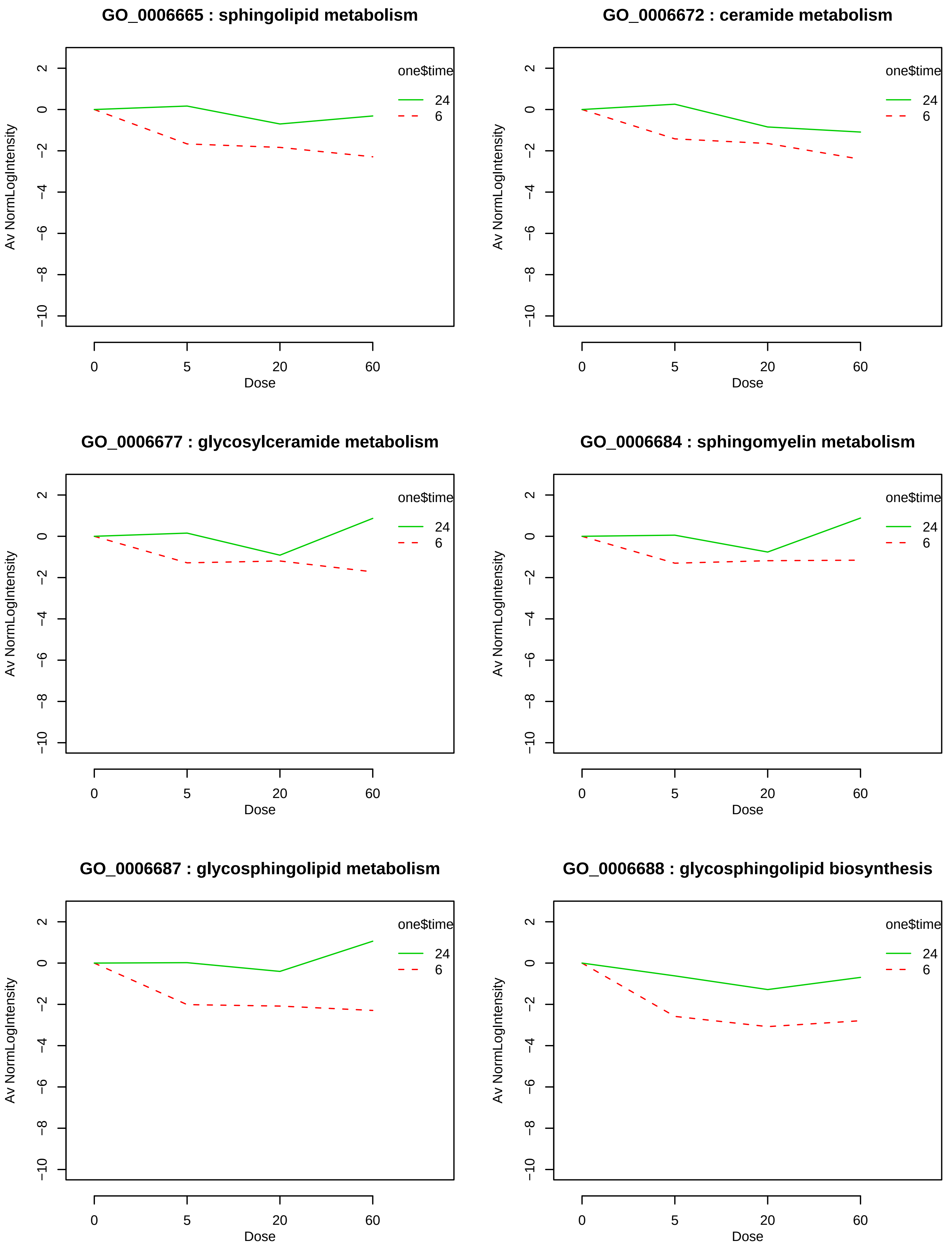
GO_0006665 : sphingolipid metabolism
GO_0006672 : ceramide metabolism
GO_0006677 : glycosylceramide metabolism
GO_0006684 : sphingomyelin metabolism
GO_0006687 : glycosphingolipid metabolism
GO_0006688 : glycosphingolipid biosynthesis
Av NormLogIntensity
Dose
one$time
24
6
2
0
−2
−4
−6
−8
−10
0
5
20
60

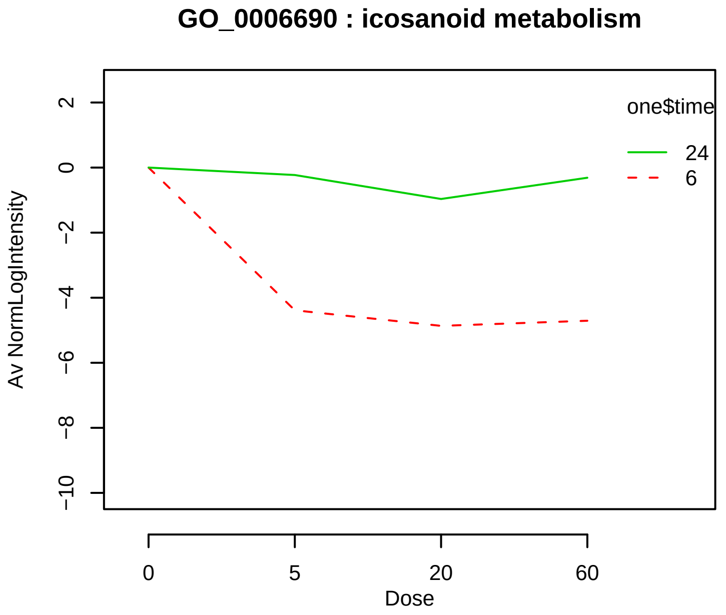
GO_0006690 : icosanoid metabolism
Av NormLogIntensity
2
0
−2
−4
−6
−8
−10
one$time
24
6
0
5
20
60
Dose

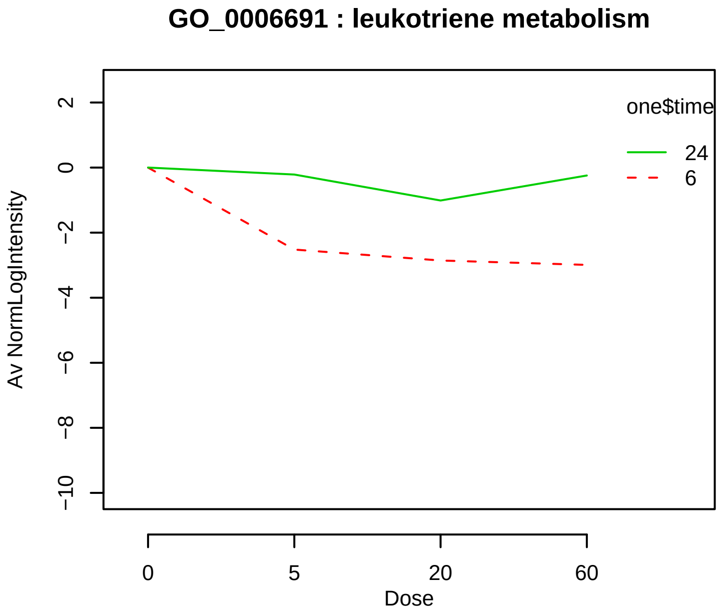
GO_0006691 : leukotriene metabolism
Av NormLogIntensity
2
0
−2
−4
−6
−8
−10
one$time
24
6
0
5
20
60
Dose

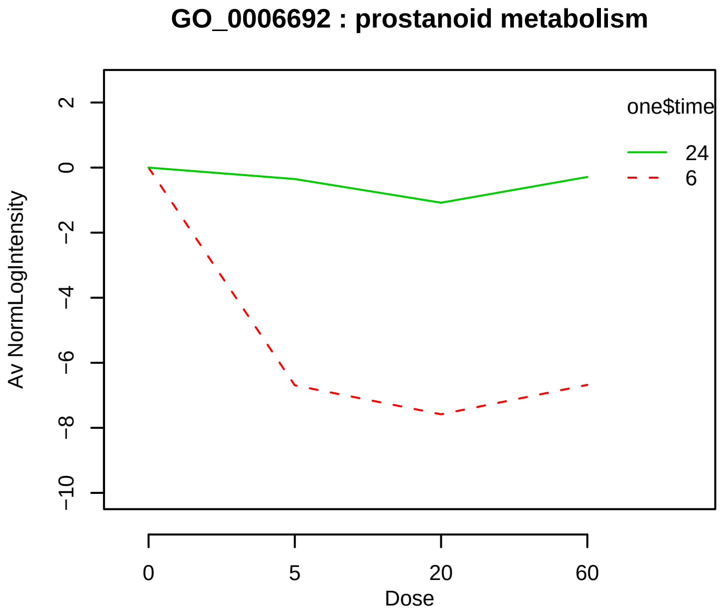
GO_0006692 : prostanoid metabolism
Av NormLogIntensity
2
0
−2
−4
−6
−8
−10
one$time
24
6
0
5
20
60
Dose

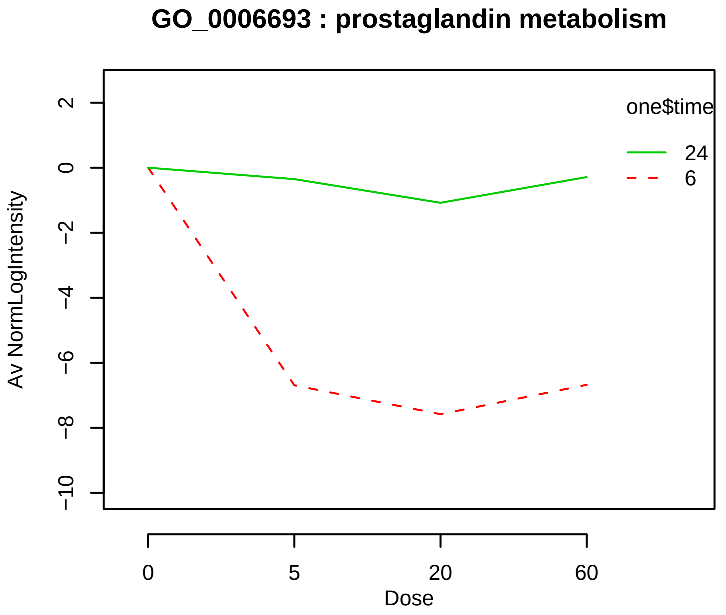
GO_0006693 : prostaglandin metabolism
Av NormLogIntensity
2
0
−2
−4
−6
−8
−10
one$time
24
6
0
5
20
60
Dose

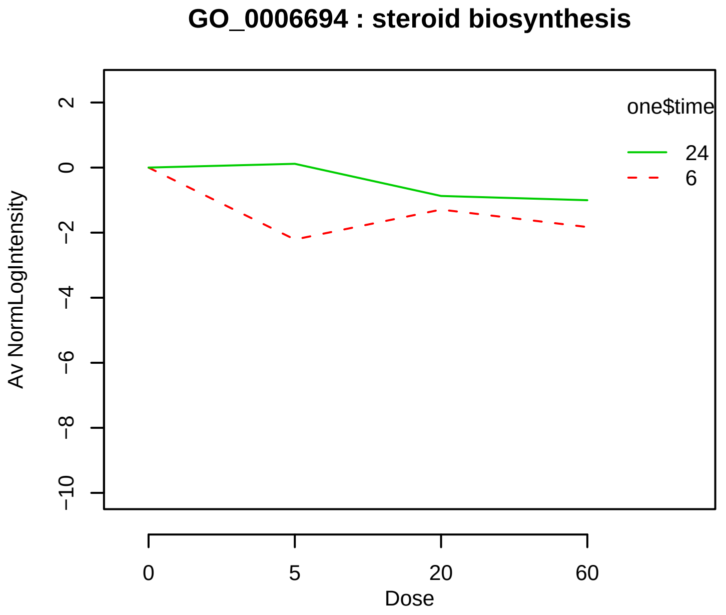
GO_0006694 : steroid biosynthesis
Av NormLogIntensity
2
0
−2
−4
−6
−8
−10
one$time
24
6
0
5
20
60
Dose

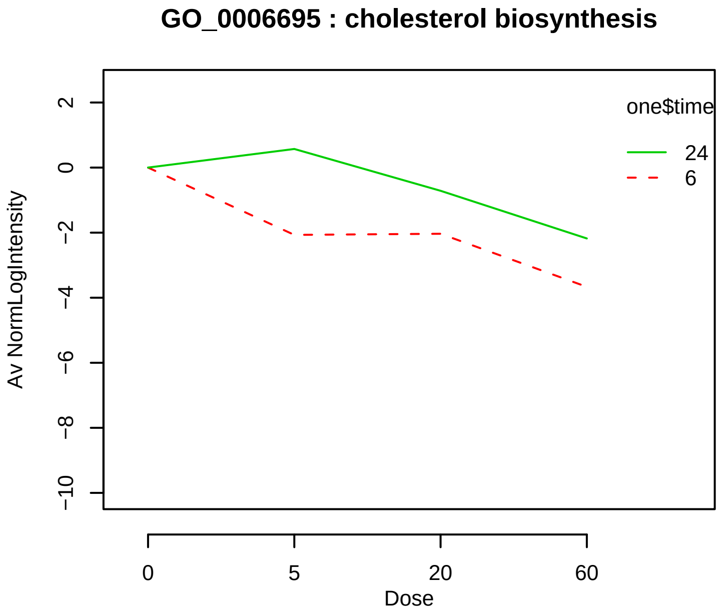
GO_0006695 : cholesterol biosynthesis
Av NormLogIntensity
2
0
−2
−4
−6
−8
−10
one$time
24
6
0
5
20
60
Dose

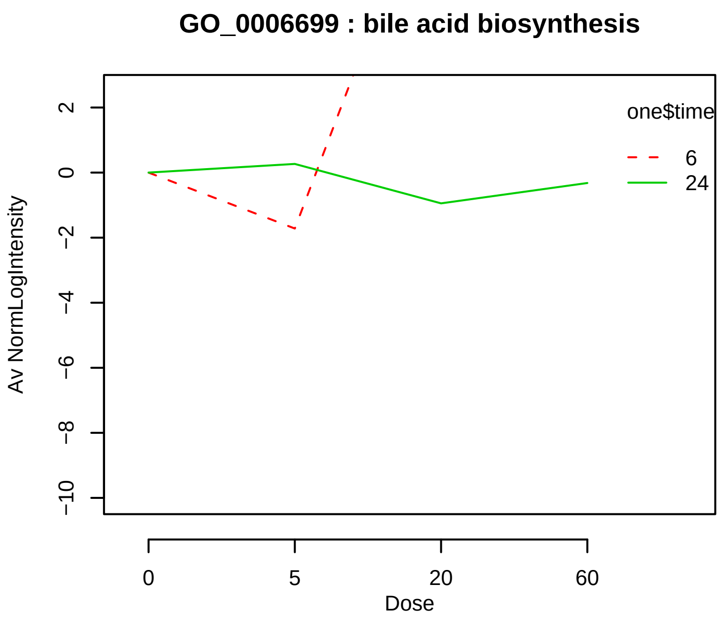
GO_0006699 : bile acid biosynthesis
Av NormLogIntensity
2
0
−2
−4
−6
−8
−10
one$time
6
24
0
5
20
60
Dose

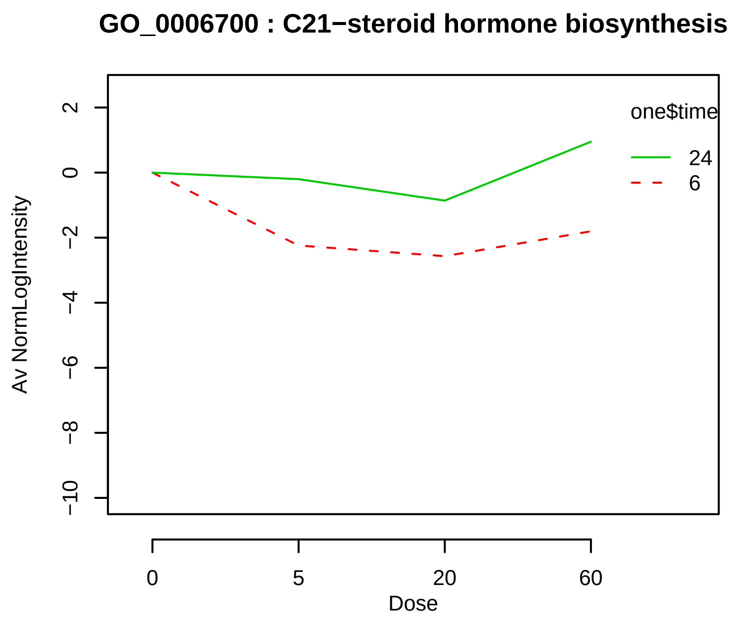
GO_0006700 : C21−steroid hormone biosynthesis
Av NormLogIntensity
2
0
−2
−4
−6
−8
−10
one$time
24
6
0
5
20
60
Dose

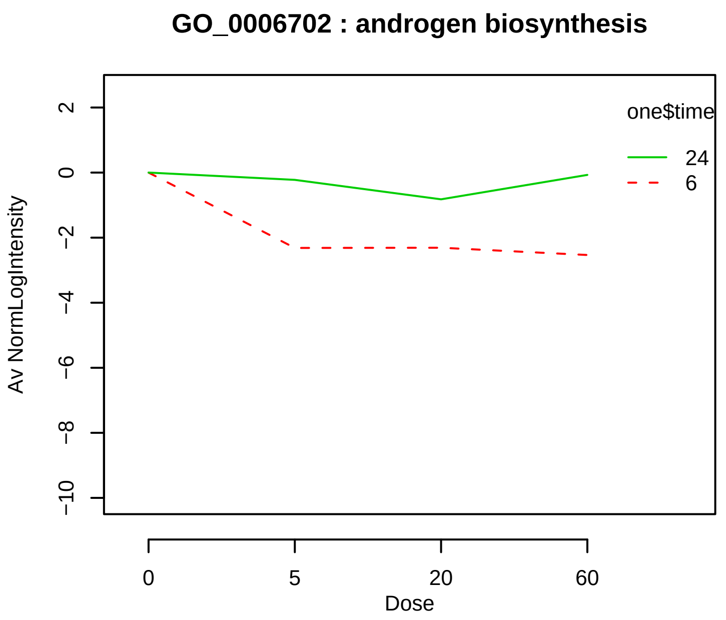
GO_0006702 : androgen biosynthesis
Av NormLogIntensity
2
0
−2
−4
−6
−8
−10
one$time
24
6
0
5
20
60
Dose

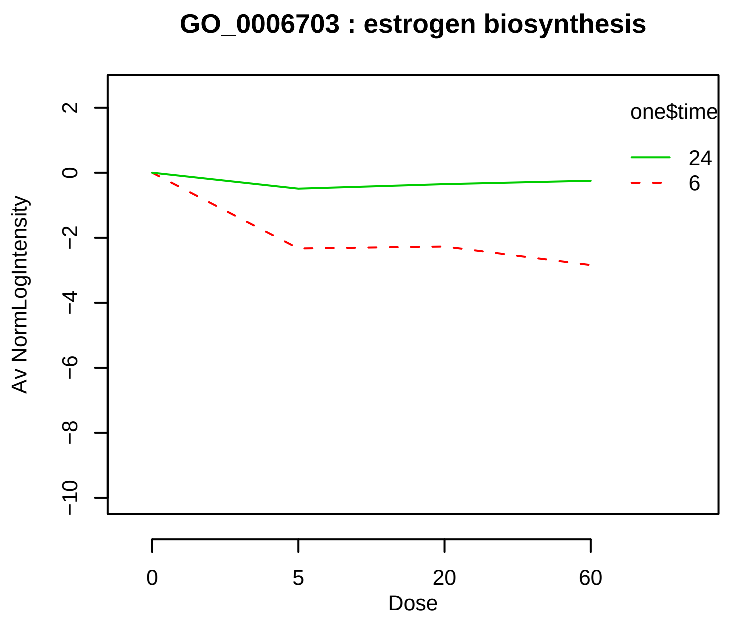
GO_0006703 : estrogen biosynthesis
Av NormLogIntensity
2
0
−2
−4
−6
−8
−10
one$time
24
6
0
5
20
60
Dose

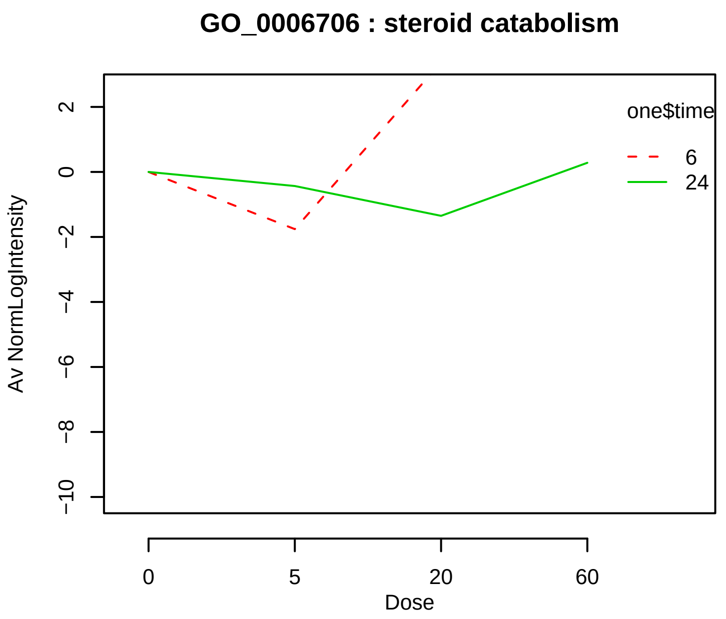
GO_0006706 : steroid catabolism
Av NormLogIntensity
2
0
−2
−4
−6
−8
−10
one$time
6
24
0
5
20
60
Dose

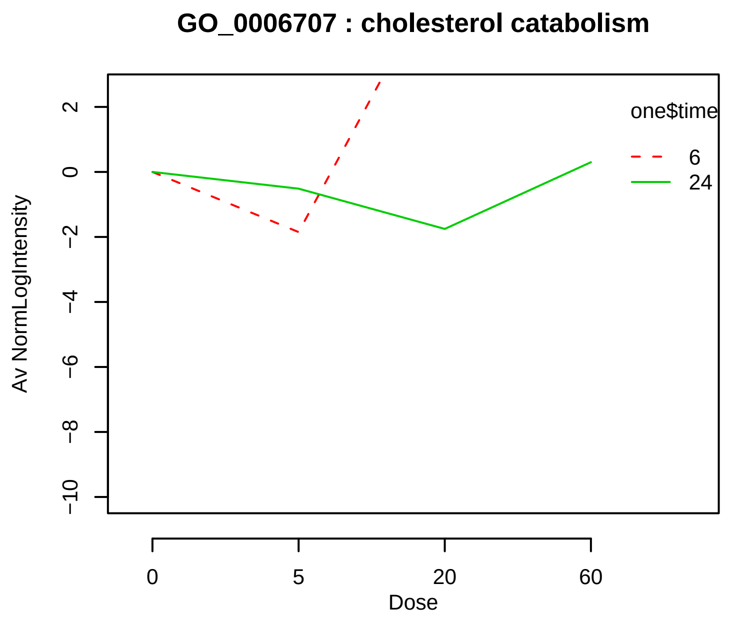
GO_0006707 : cholesterol catabolism
Av NormLogIntensity
2
0
−2
−4
−6
−8
−10
one$time
6
24
0
5
20
60
Dose

GO_0006720 : isoprenoid metabolism

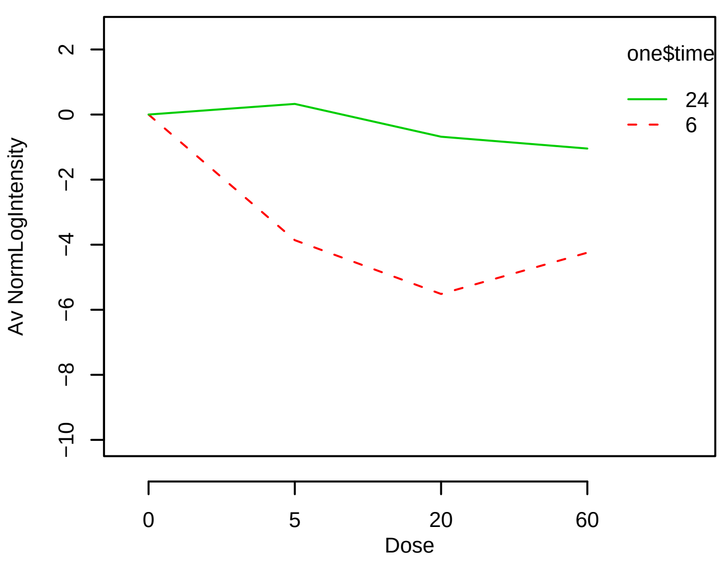

GO_0006721 : terpenoid metabolism

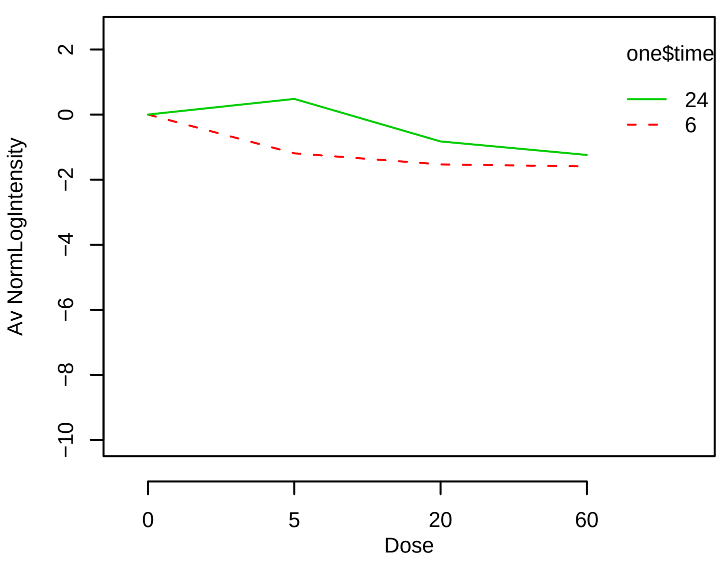

GO_0006725 : aromatic compound metabolism

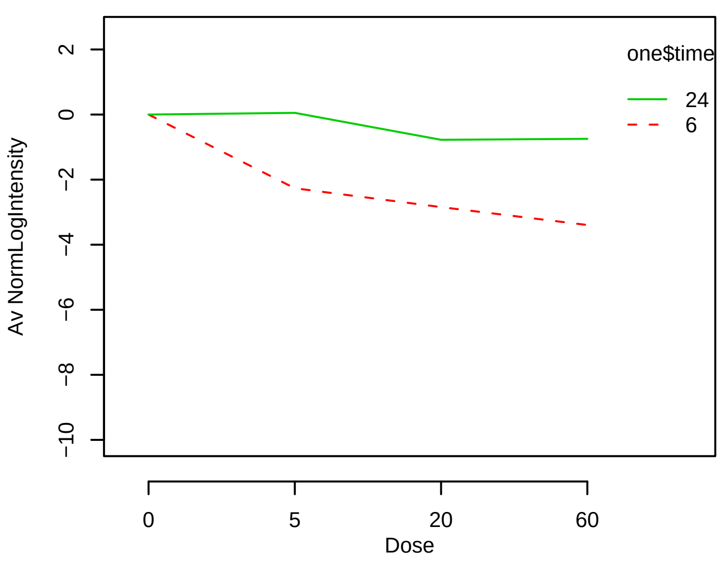

GO_0006729 : tetrahydrobiopterin biosynthesis

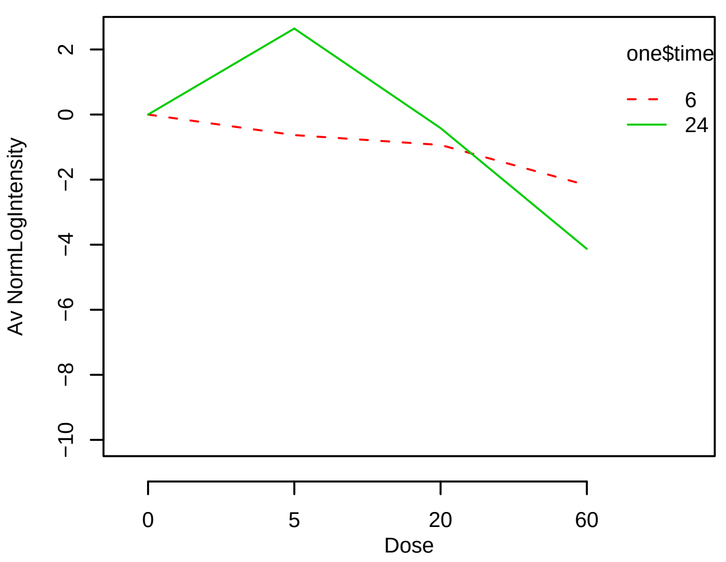

GO_0006730 : one−carbon compound metabolism

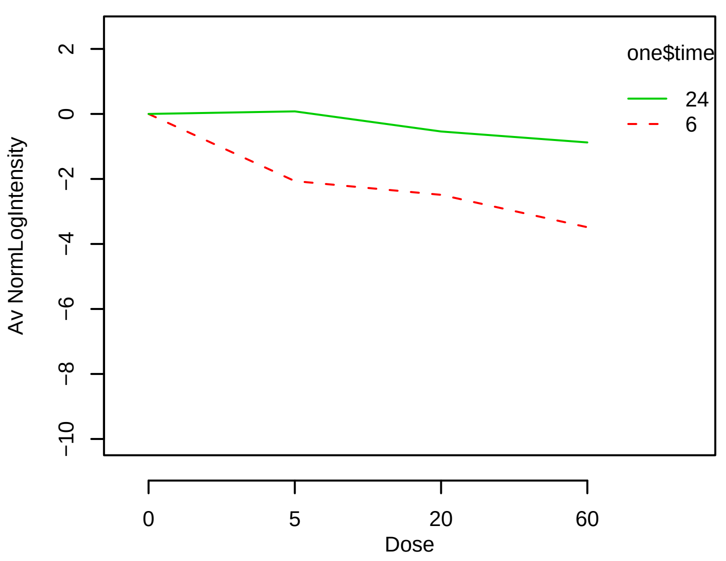

GO_0006732 : coenzyme metabolism

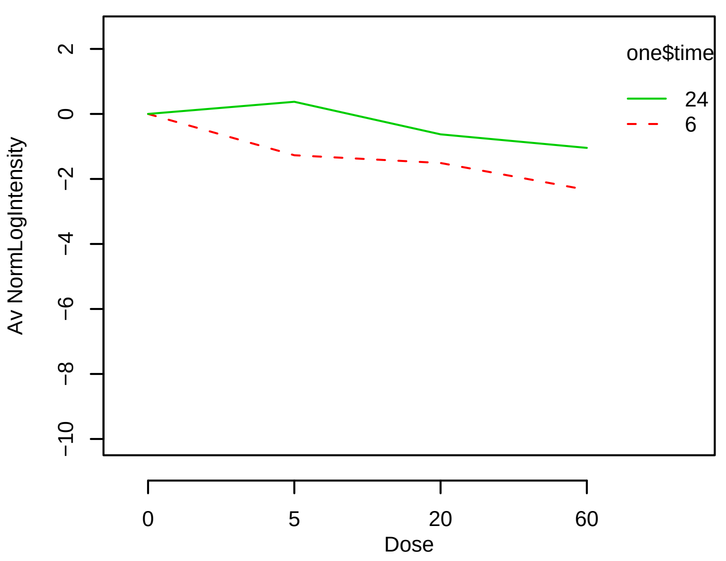

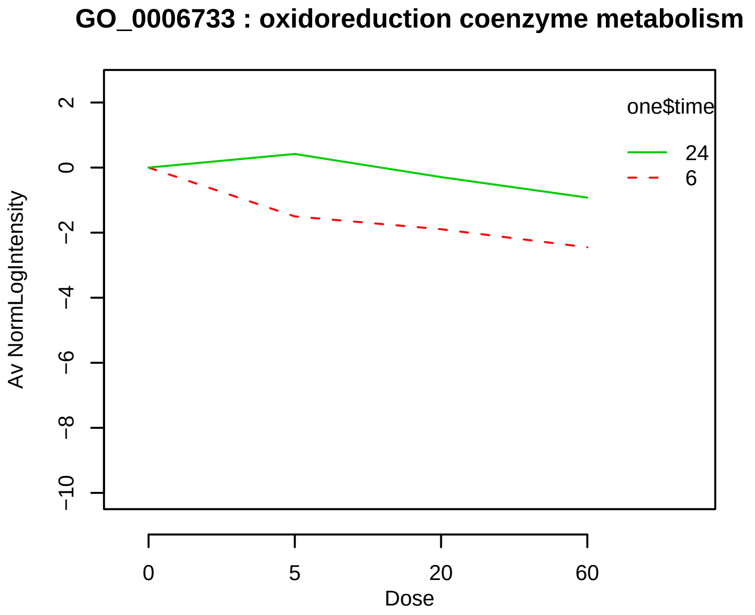
GO_0006733 : oxidoreduction coenzyme metabolism
Av NormLogIntensity
2
0
−2
−4
−6
−8
−10
one$time
24
6
0
5
20
60
Dose

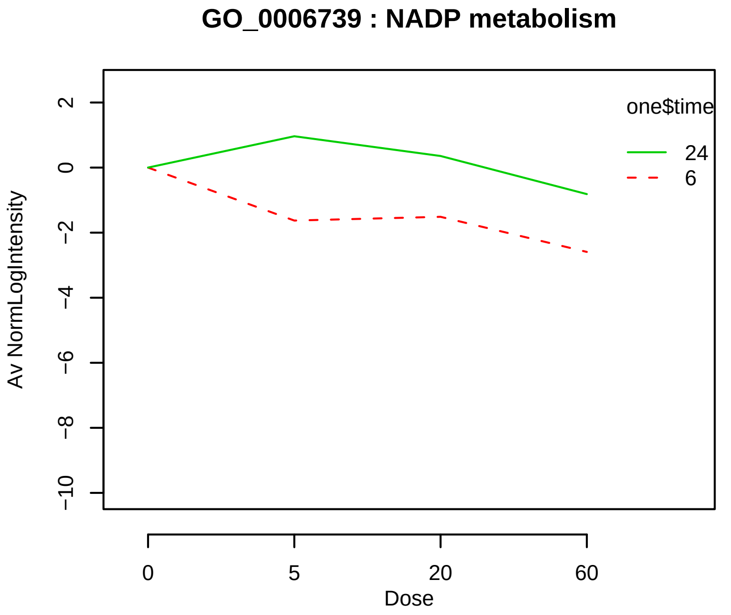
GO_0006739 : NADP metabolism
Av NormLogIntensity
2
0
−2
−4
−6
−8
−10
one$time
24
6
0
5
20
60
Dose

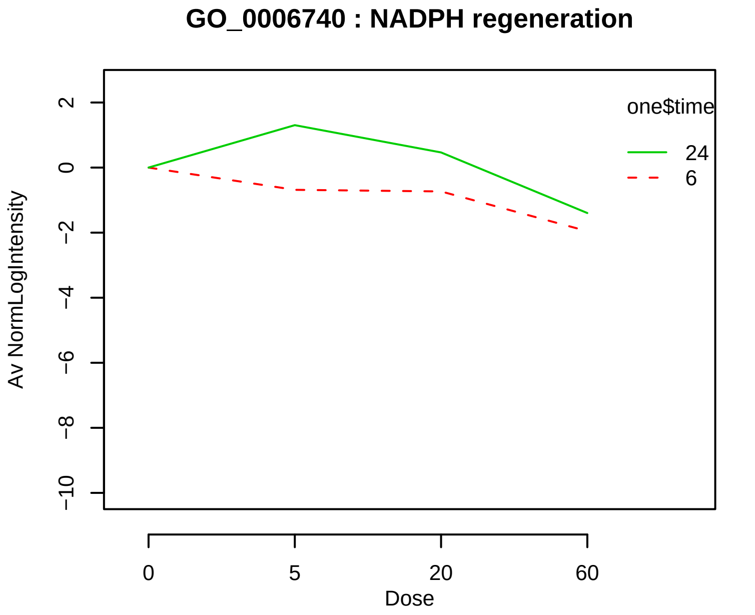
GO_0006740 : NADPH regeneration
Av NormLogIntensity
2
0
−2
−4
−6
−8
−10
one$time
24
6
0
5
20
60
Dose

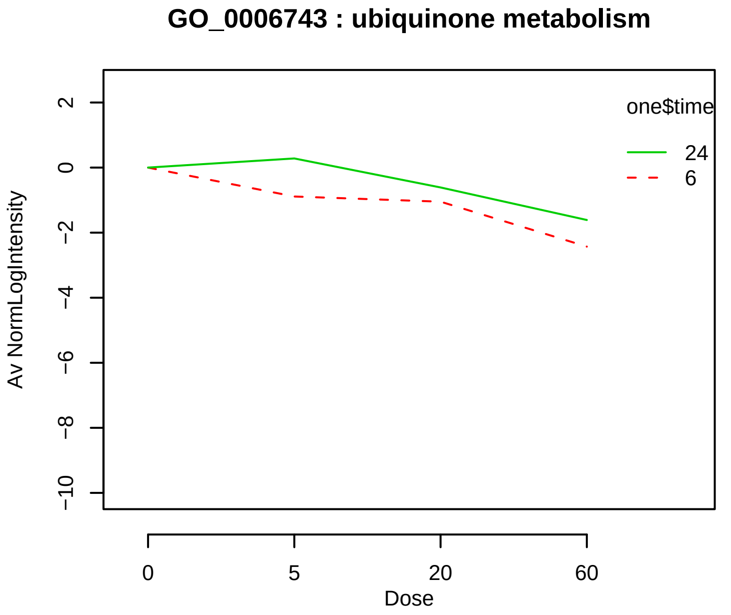
GO_0006743 : ubiquinone metabolism
Av NormLogIntensity
2
0
−2
−4
−6
−8
−10
one$time
24
6
0
5
20
60
Dose

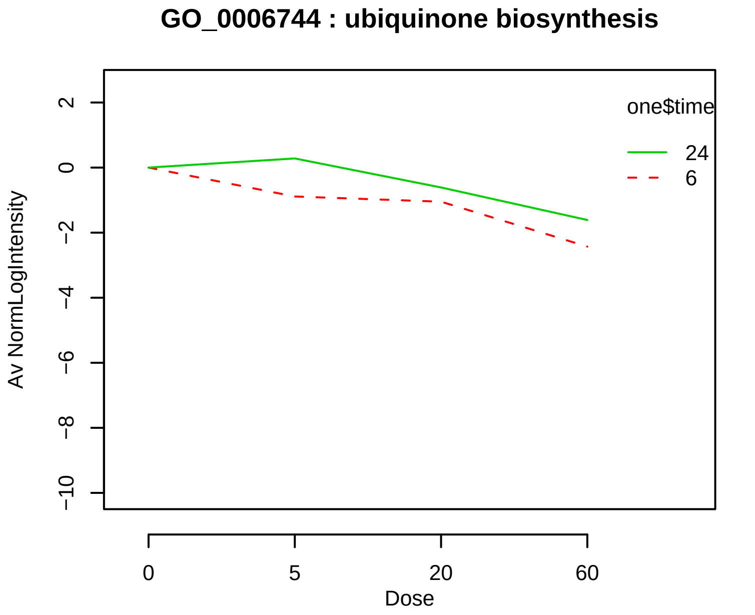
GO_0006744 : ubiquinone biosynthesis
Av NormLogIntensity
2
0
−2
−4
−6
−8
−10
one$time
24
6
0
5
20
60
Dose

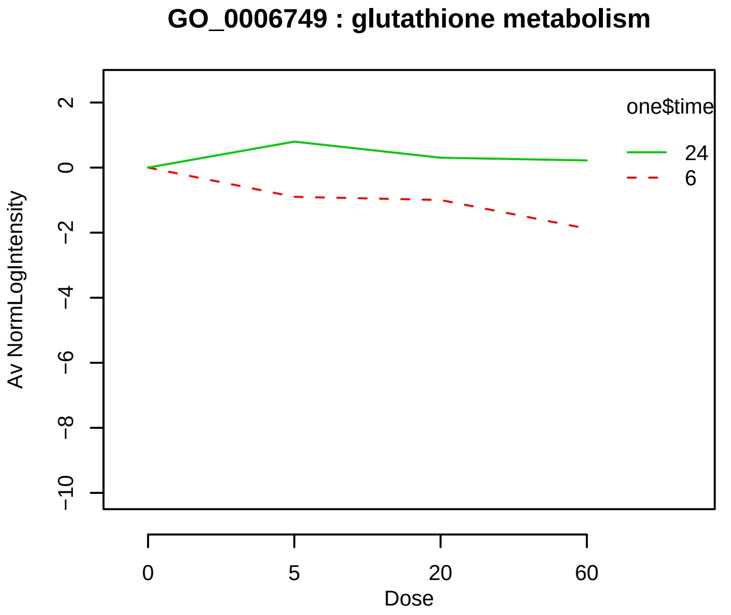
GO_0006749 : glutathione metabolism
Av NormLogIntensity
2
0
−2
−4
−6
−8
−10
one$time
24
6
0
5
20
60
Dose

GO_0006750 : glutathione biosynthesis

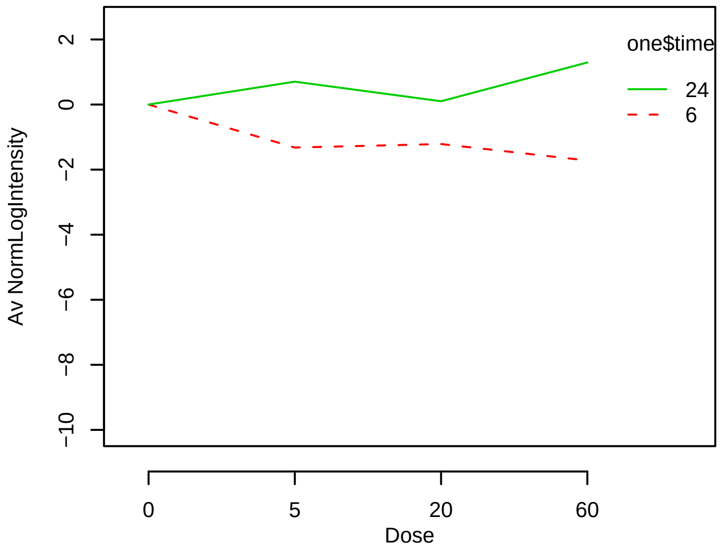

GO_0006752 : group transfer coenzyme metabolism

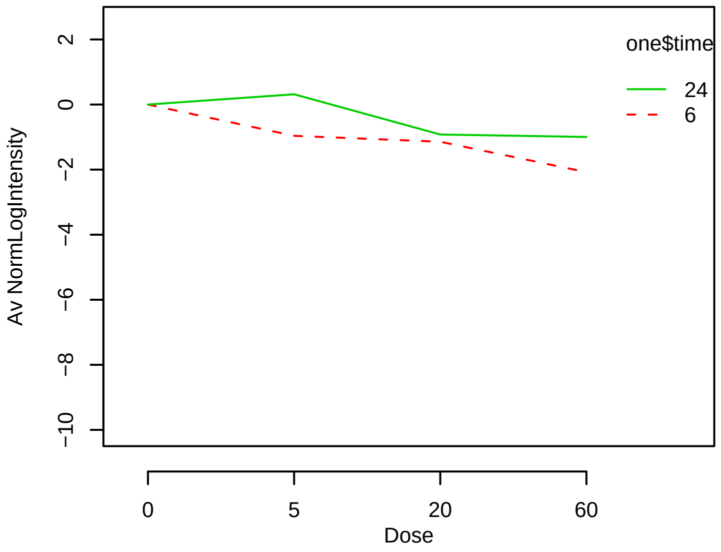

GO_0006753 : nucleoside phosphate metabolism

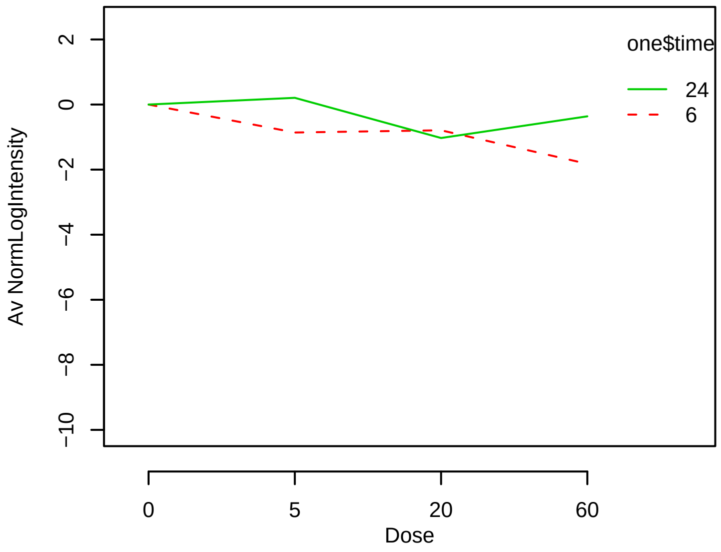

GO_0006754 : ATP biosynthesis

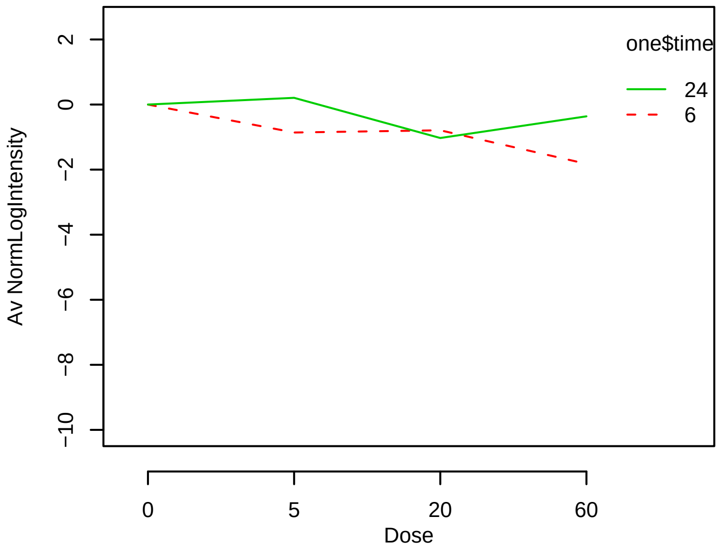

GO_0006760 : folic acid and derivative metabolism

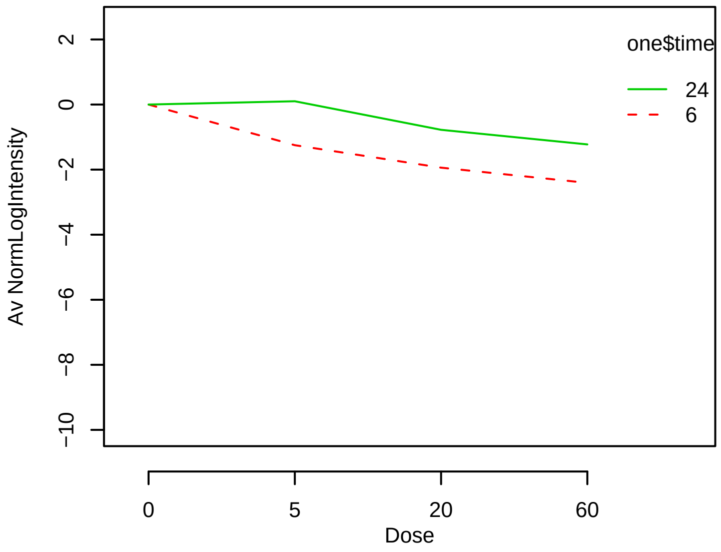

GO_0006766 : vitamin metabolism

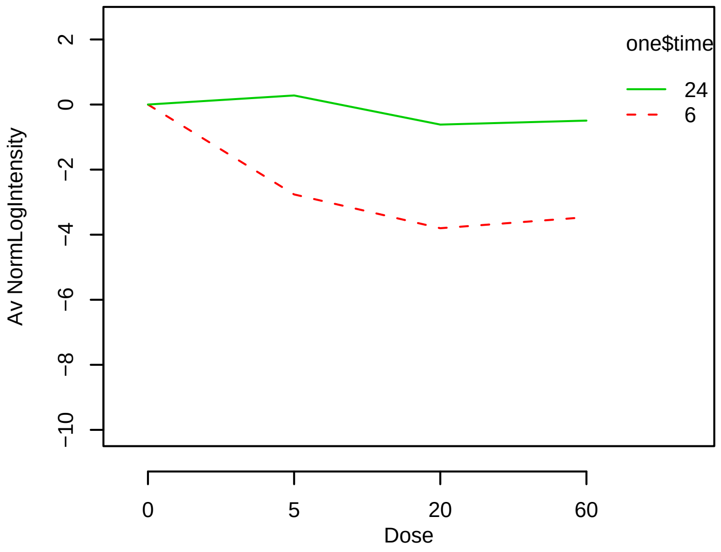

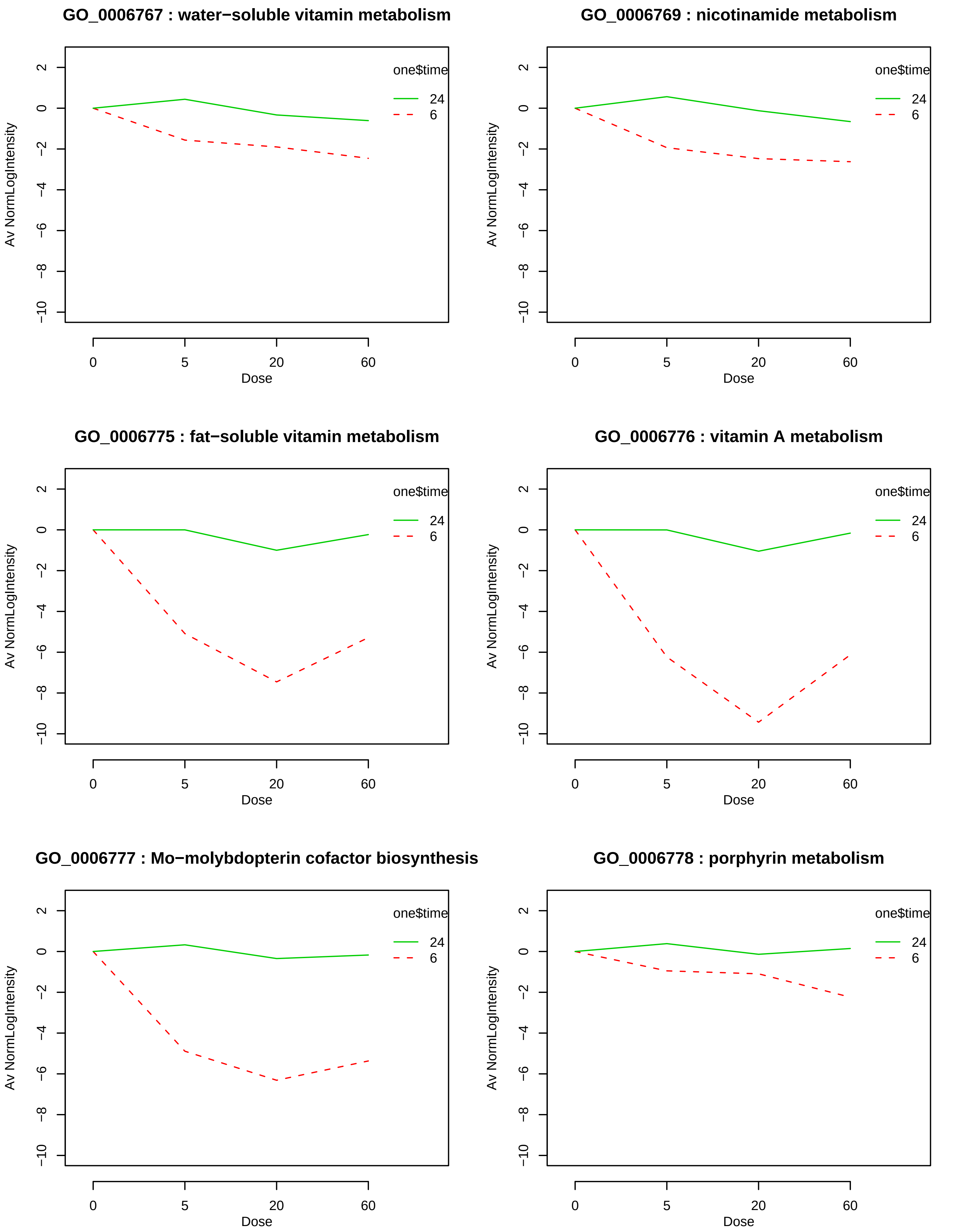
GO_0006767 : water−soluble vitamin metabolism
GO_0006769 : nicotinamide metabolism
GO_0006775 : fat−soluble vitamin metabolism
GO_0006776 : vitamin A metabolism
GO_0006777 : Mo−molybdopterin cofactor biosynthesis
GO_0006778 : porphyrin metabolism
Av NormLogIntensity
Dose
one$time
24
6
0
5
20
60
2
0
−2
−4
−6
−8
−10

GO_0006779 : porphyrin biosynthesis

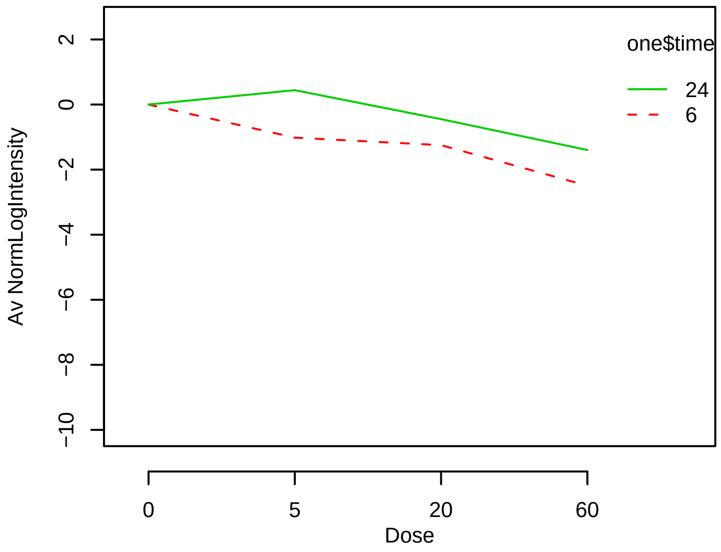

GO_0006783 : heme biosynthesis

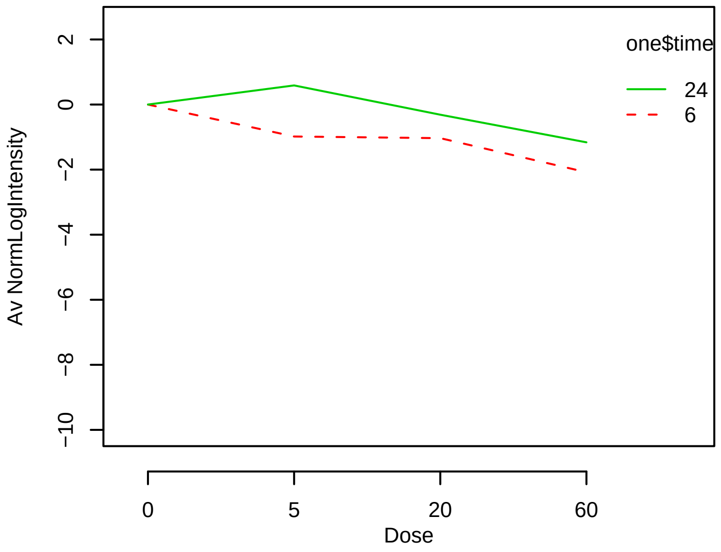

GO_0006787 : porphyrin catabolism

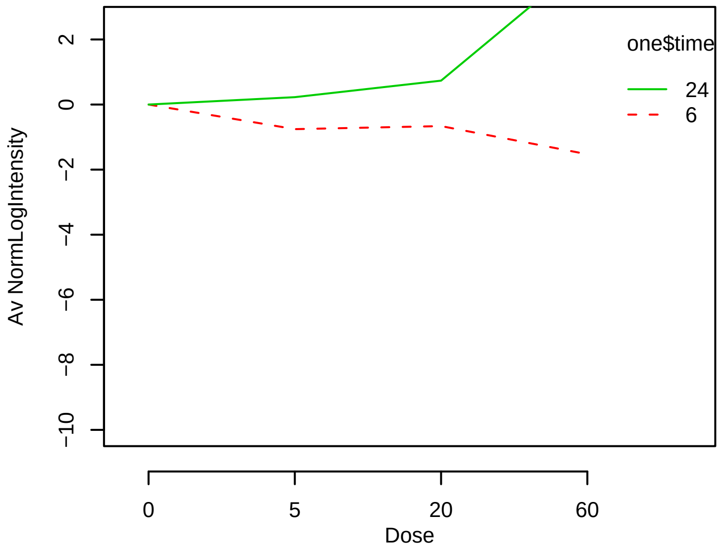

GO_0006790 : sulfur metabolism

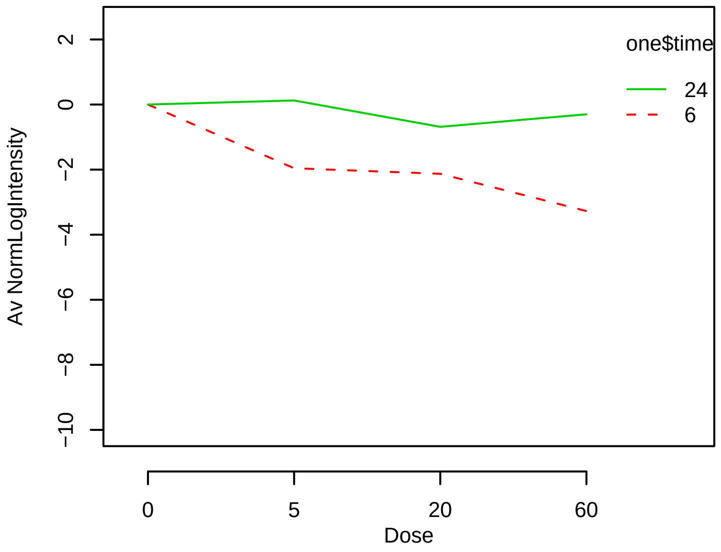

GO_0006800 : oxygen and reactive oxygen species metaboli

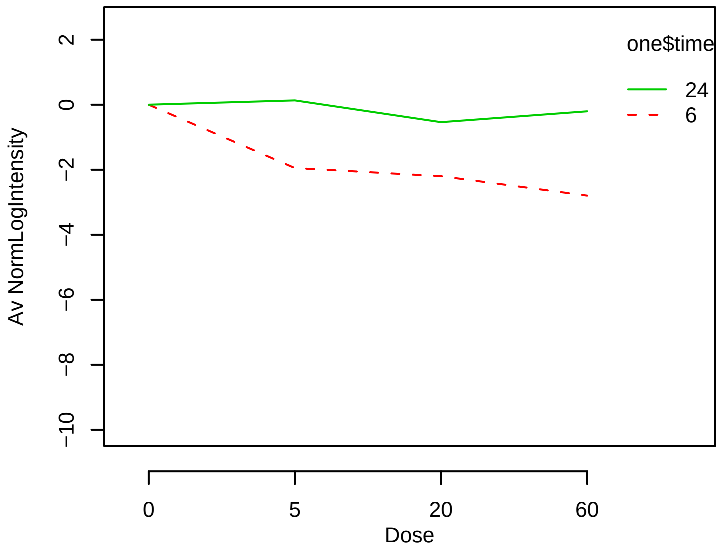

GO_0006801 : superoxide metabolism

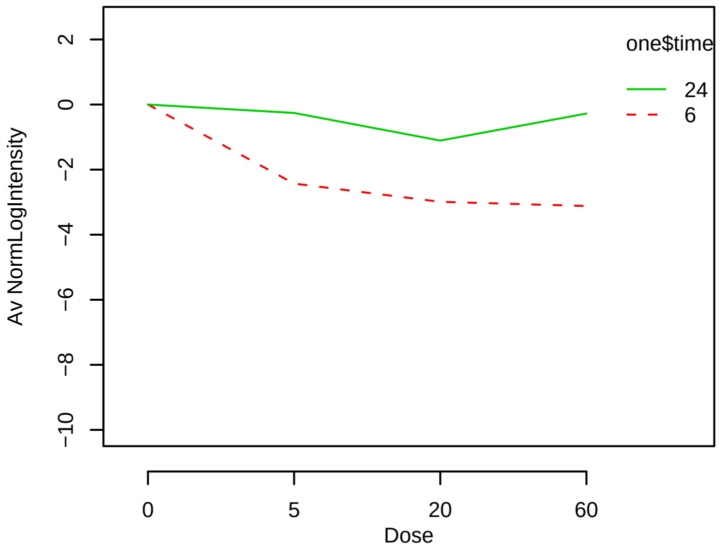

GO_0006805 : xenobiotic metabolism

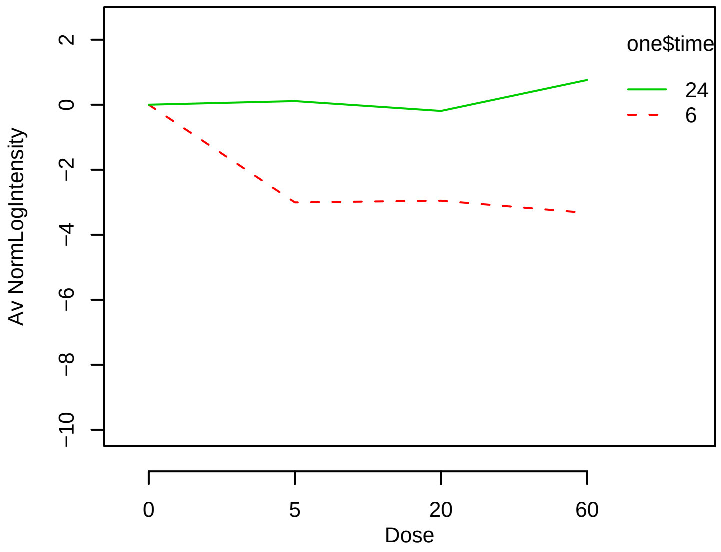

GO_0006809 : nitric oxide biosynthesis

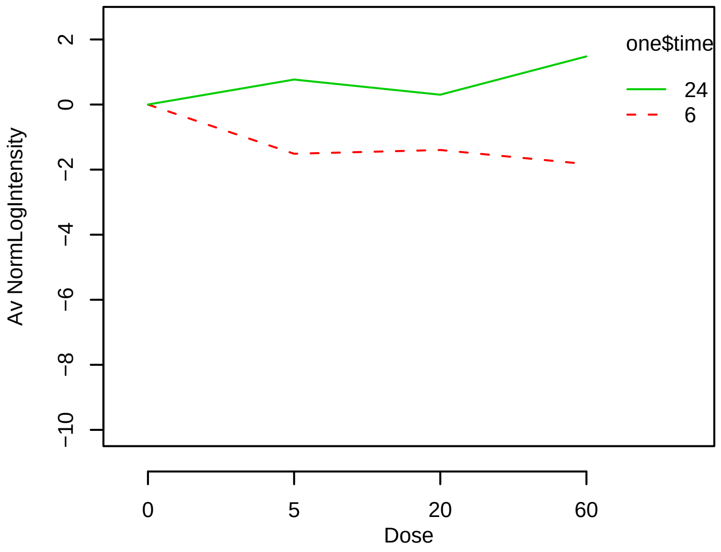

GO_0006813 : potassium ion transport

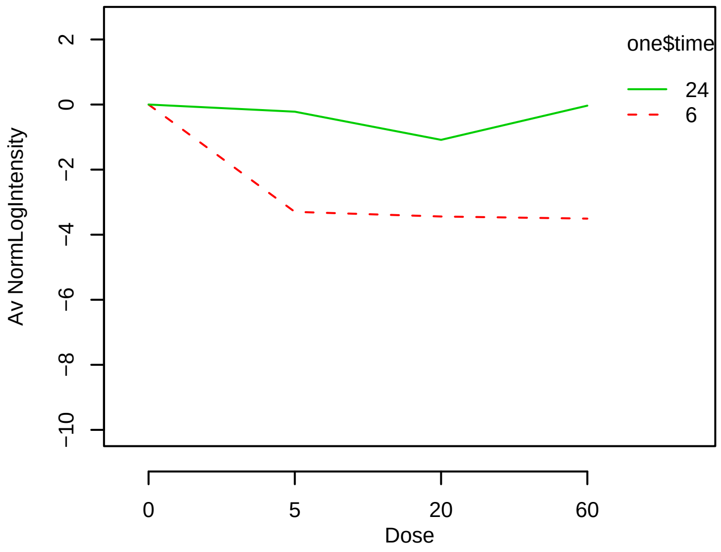

GO_0006814 : sodium ion transport

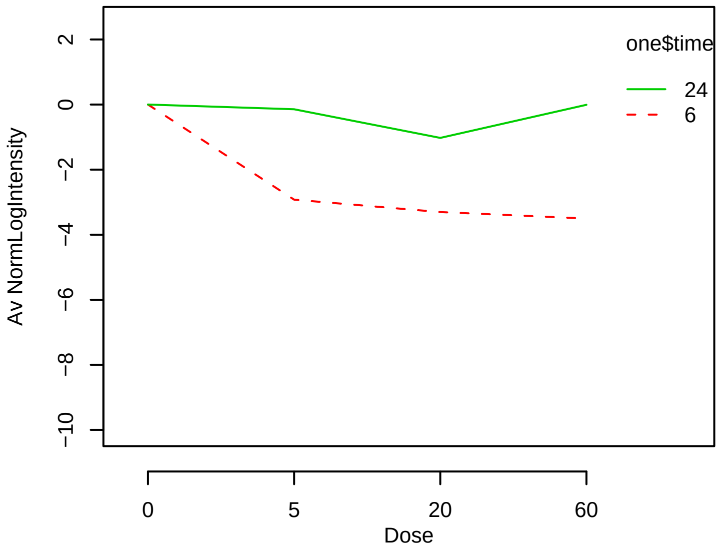

GO_0006816 : calcium ion transport

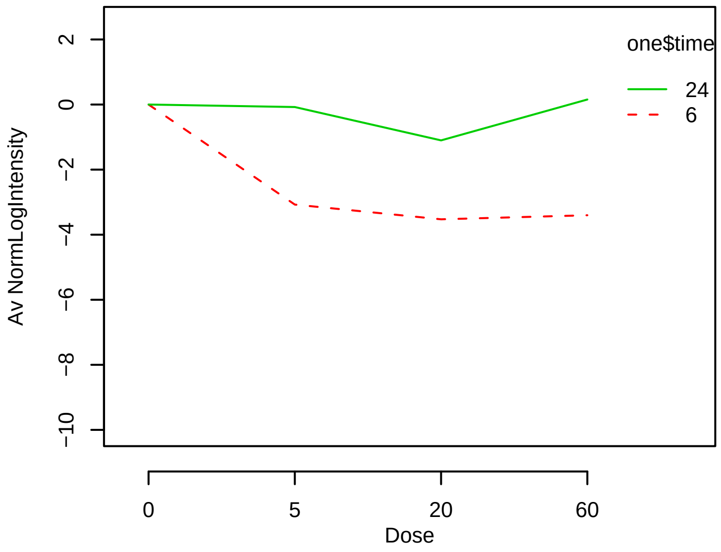

GO_0006817 : phosphate transport

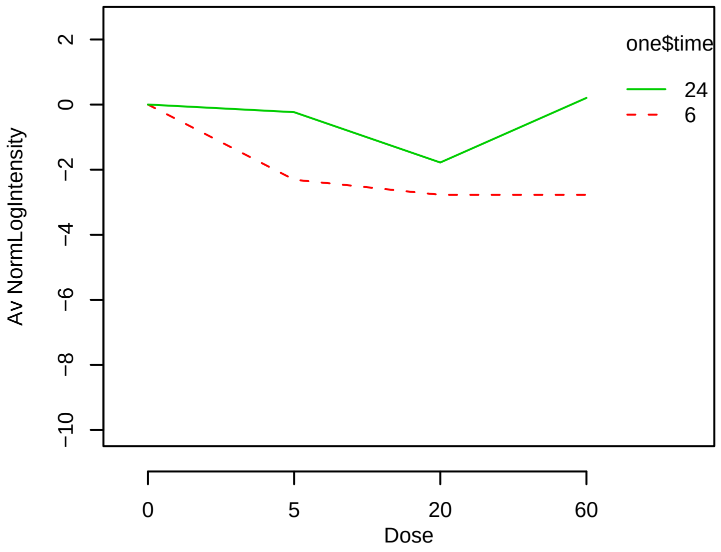

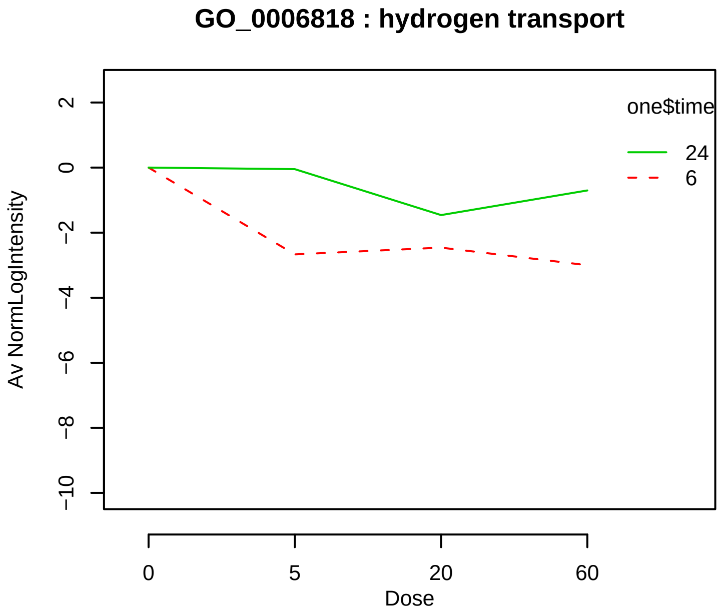
GO_0006818 : hydrogen transport
one$time
24
6
Av NormLogIntensity
2
0
−2
−4
−6
−8
−10
0
5
20
60
Dose
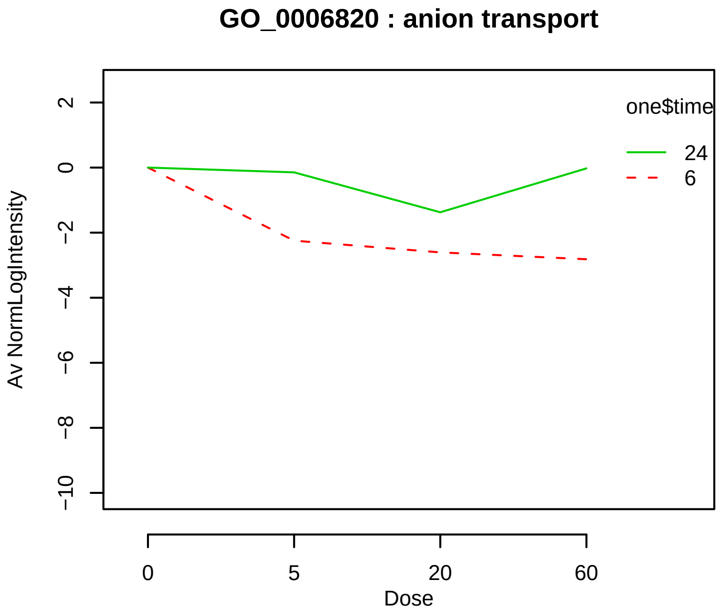
GO_0006820 : anion transport
one$time
24
6
Av NormLogIntensity
2
0
−2
−4
−6
−8
−10
0
5
20
60
Dose
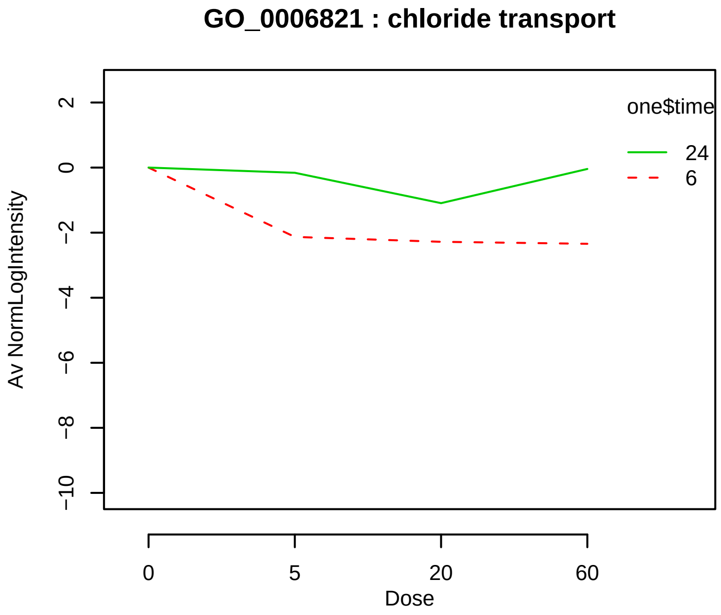
GO_0006821 : chloride transport
one$time
24
6
Av NormLogIntensity
2
0
−2
−4
−6
−8
−10
0
5
20
60
Dose
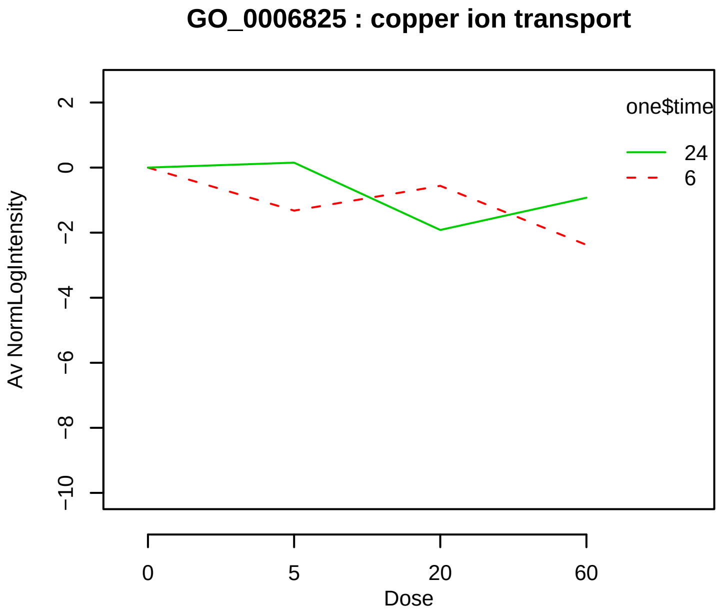
GO_0006825 : copper ion transport
one$time
24
6
Av NormLogIntensity
2
0
−2
−4
−6
−8
−10
0
5
20
60
Dose
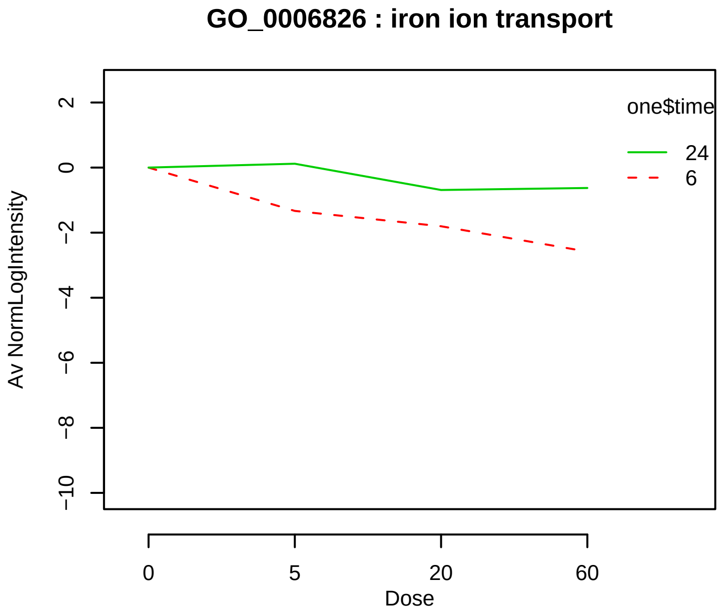
GO_0006826 : iron ion transport
one$time
24
6
Av NormLogIntensity
2
0
−2
−4
−6
−8
−10
0
5
20
60
Dose
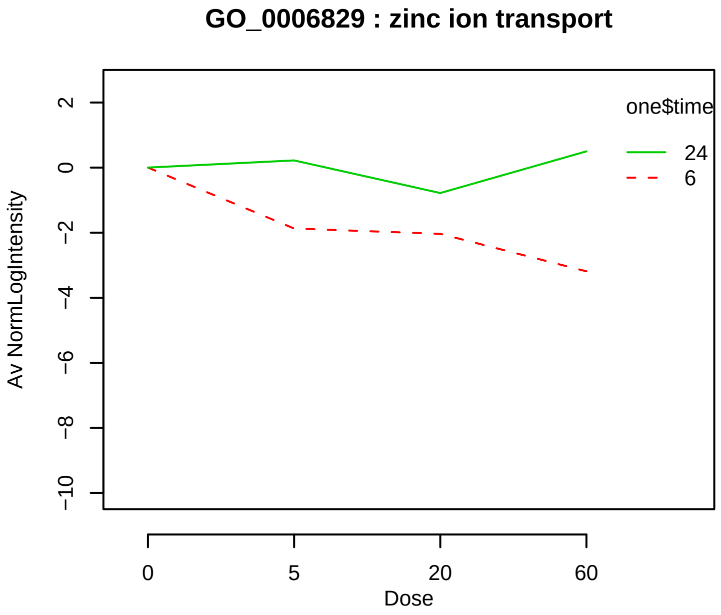
GO_0006829 : zinc ion transport
one$time
24
6
Av NormLogIntensity
2
0
−2
−4
−6
−8
−10
0
5
20
60
Dose

GO_0006833 : water transport

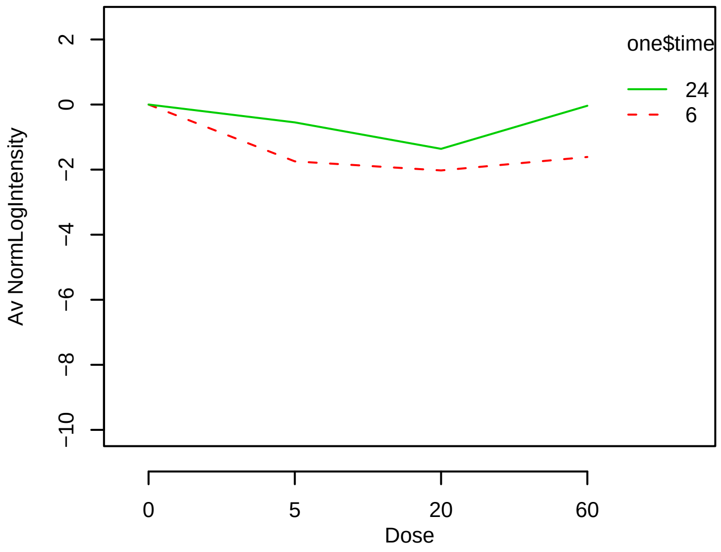

GO_0006835 : dicarboxylic acid transport

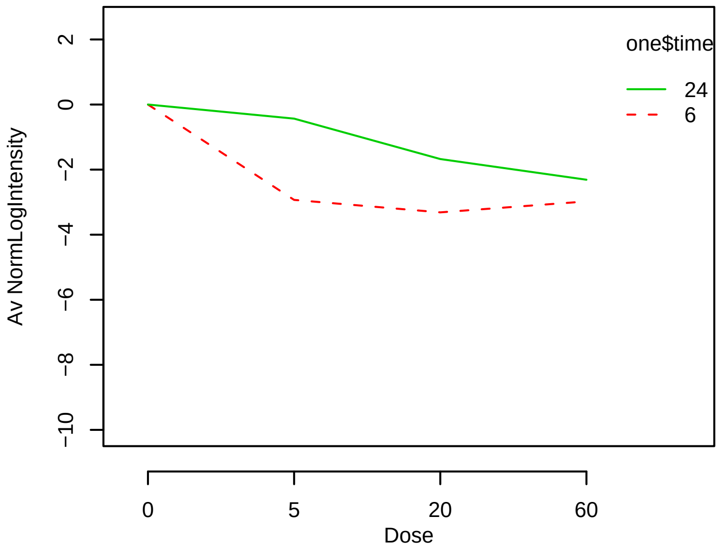

GO_0006836 : neurotransmitter transport

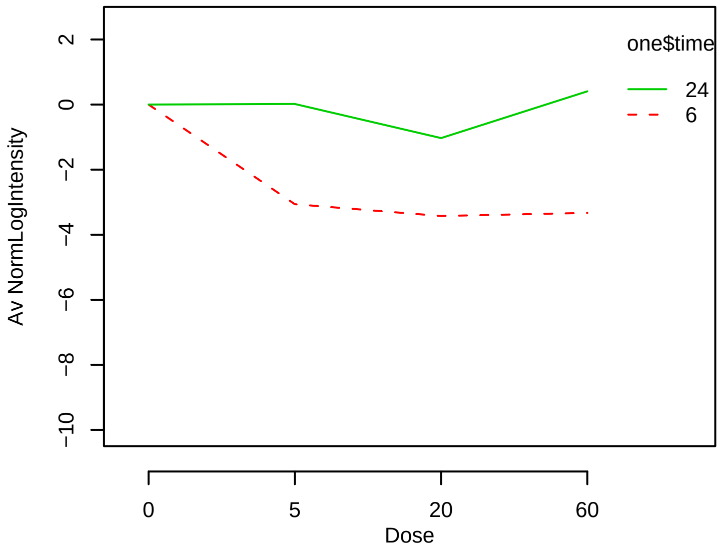

GO_0006839 : mitochondrial transport

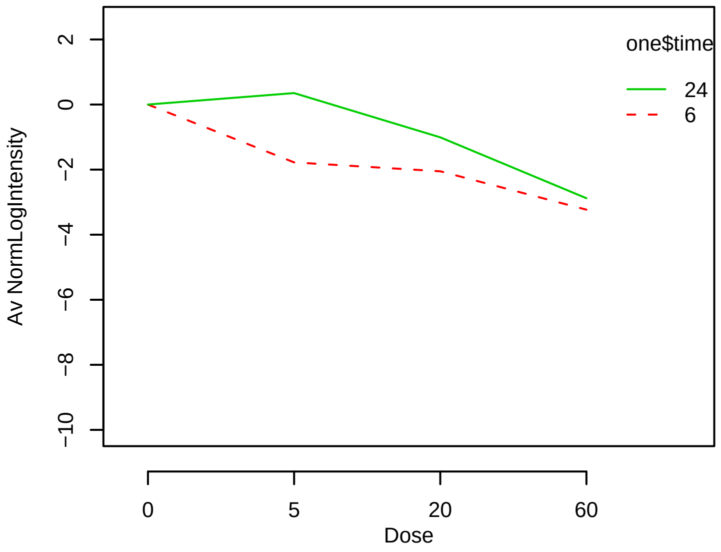

GO_0006857 : oligopeptide transport

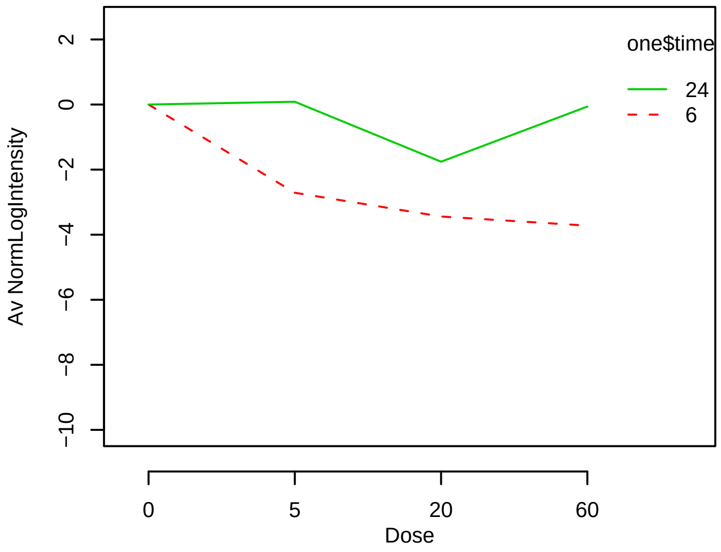

GO_0006865 : amino acid transport

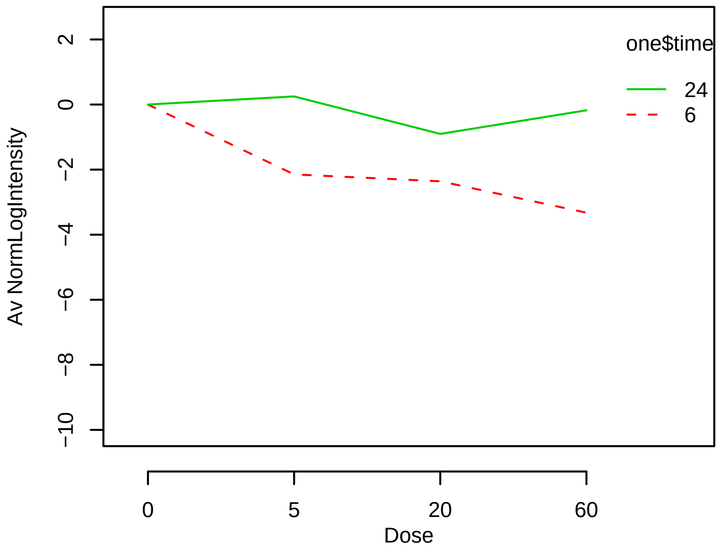

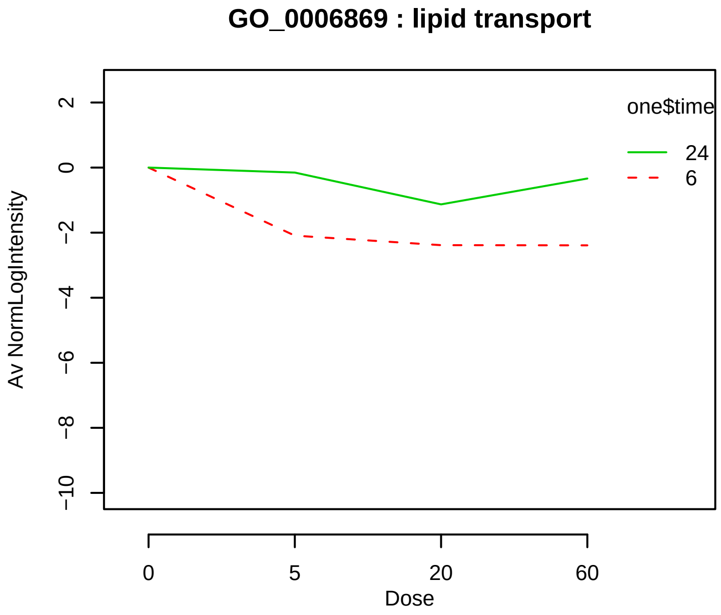
GO_0006869 : lipid transport
Av NormLogIntensity
2
0
−2
−4
−6
−8
−10
one$time
24
6
0
5
20
60
Dose

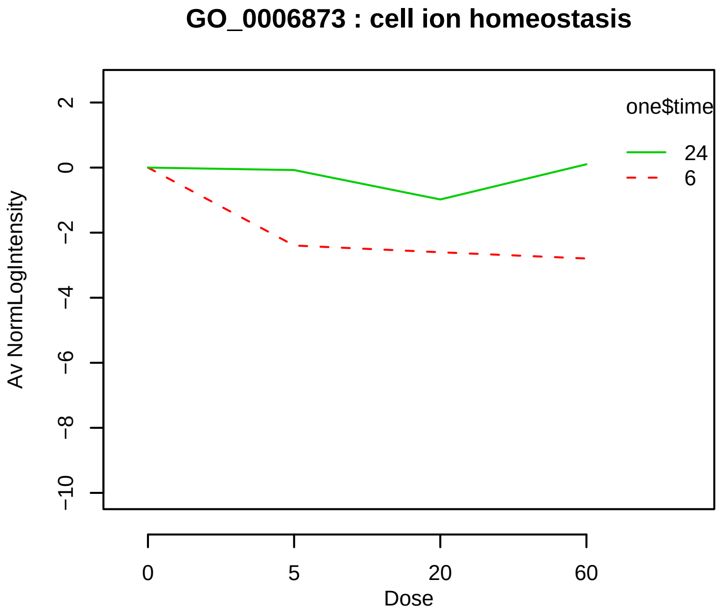
GO_0006873 : cell ion homeostasis
Av NormLogIntensity
2
0
−2
−4
−6
−8
−10
one$time
24
6
0
5
20
60
Dose

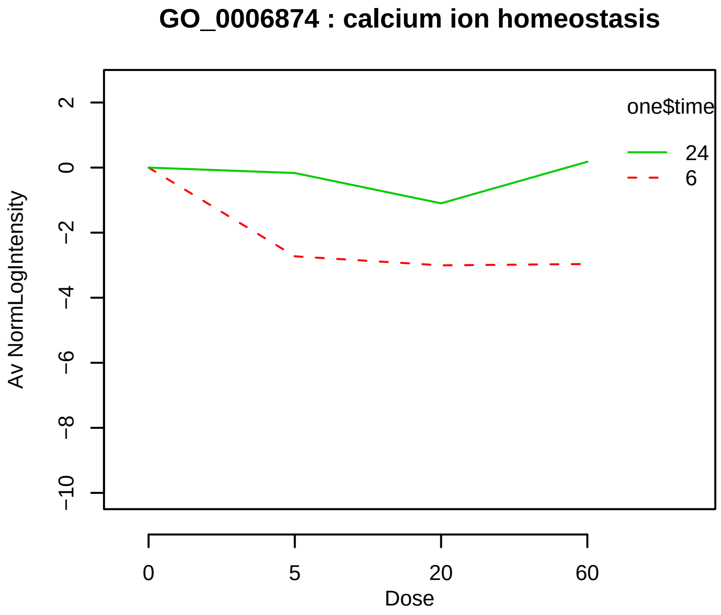
GO_0006874 : calcium ion homeostasis
Av NormLogIntensity
2
0
−2
−4
−6
−8
−10
one$time
24
6
0
5
20
60
Dose

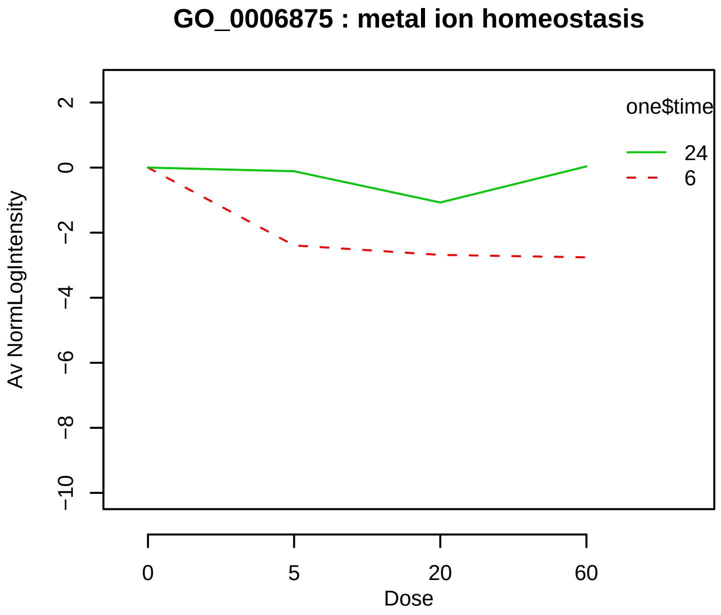
GO_0006875 : metal ion homeostasis
Av NormLogIntensity
2
0
−2
−4
−6
−8
−10
one$time
24
6
0
5
20
60
Dose

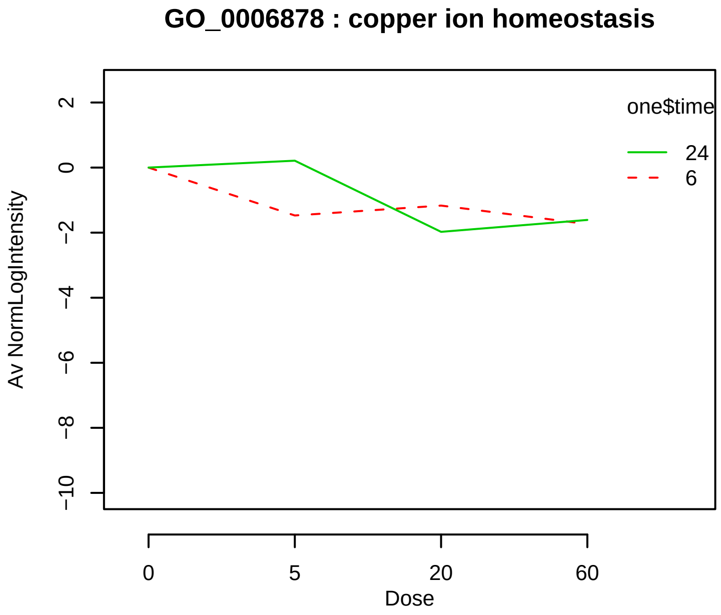
GO_0006878 : copper ion homeostasis
Av NormLogIntensity
2
0
−2
−4
−6
−8
−10
one$time
24
6
0
5
20
60
Dose

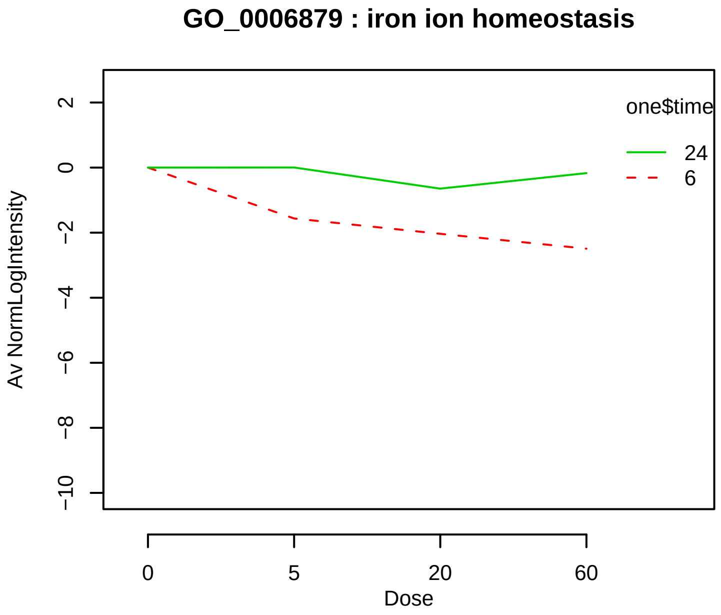
GO_0006879 : iron ion homeostasis
Av NormLogIntensity
2
0
−2
−4
−6
−8
−10
one$time
24
6
0
5
20
60
Dose

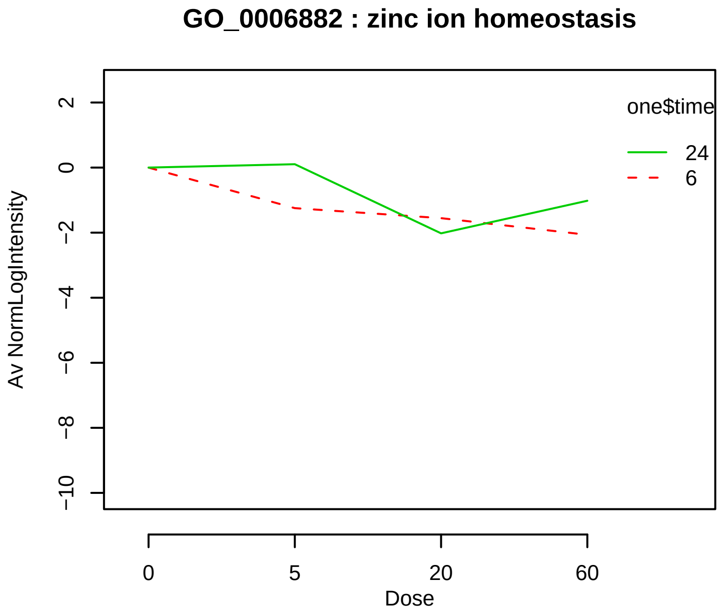
GO_0006882 : zinc ion homeostasis
one$time
24
6
Av NormLogIntensity
Dose
0
5
20
60

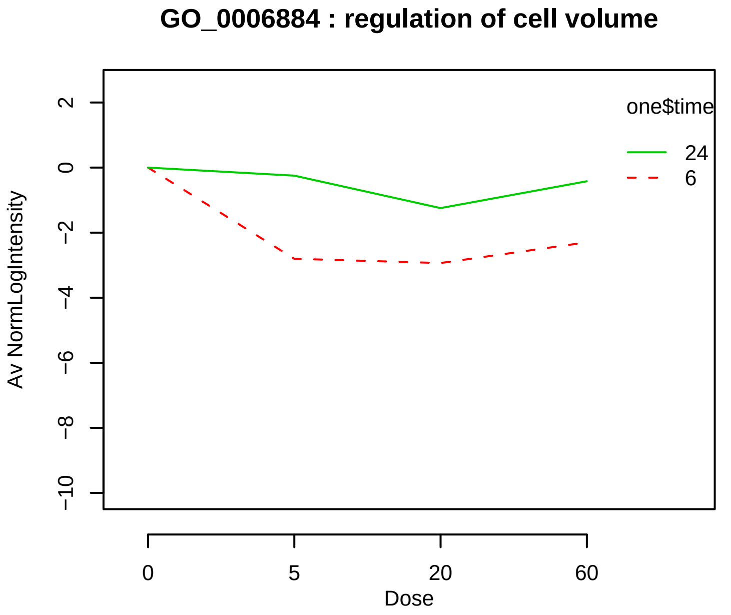
GO_0006884 : regulation of cell volume
one$time
24
6
Av NormLogIntensity
Dose
0
5
20
60

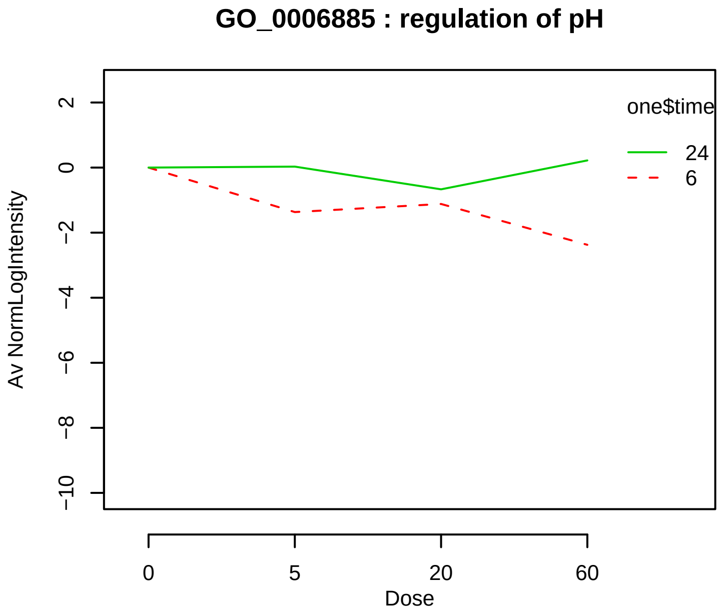
GO_0006885 : regulation of pH
one$time
24
6
Av NormLogIntensity
Dose
0
5
20
60

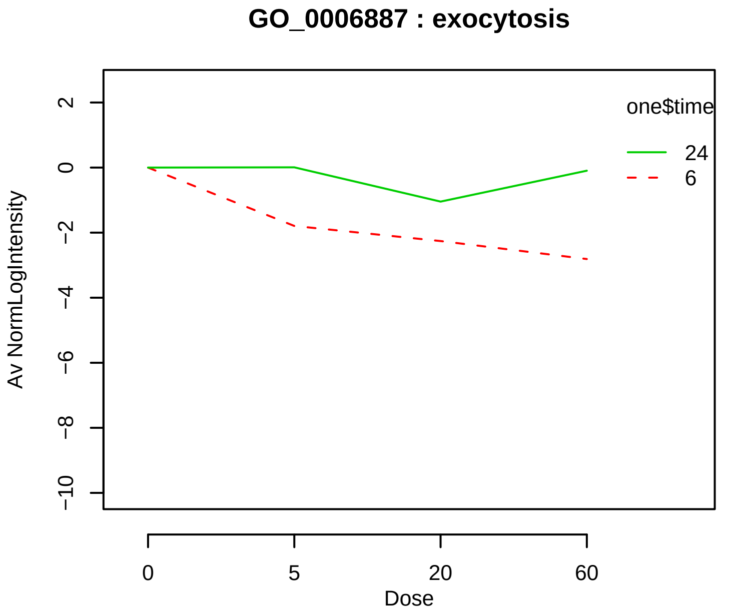
GO_0006887 : exocytosis
one$time
24
6
Av NormLogIntensity
Dose
0
5
20
60

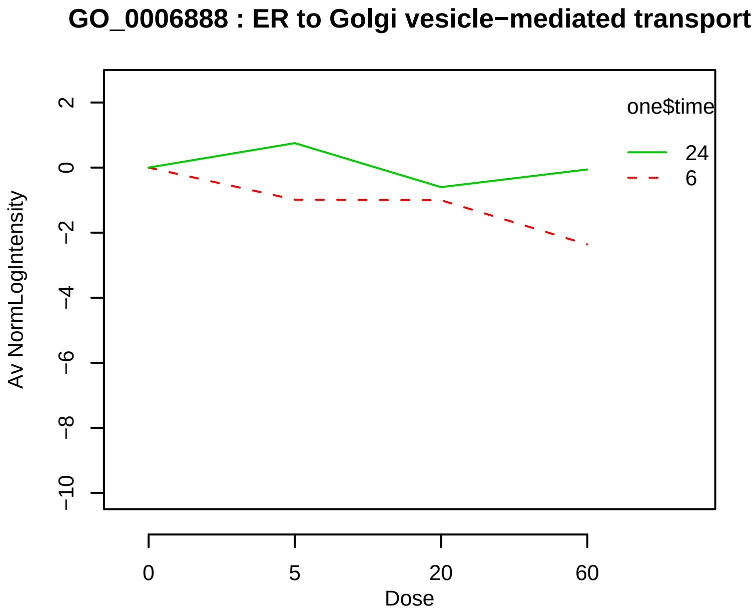
GO_0006888 : ER to Golgi vesicle−mediated transport
one$time
24
6
Av NormLogIntensity
Dose
0
5
20
60

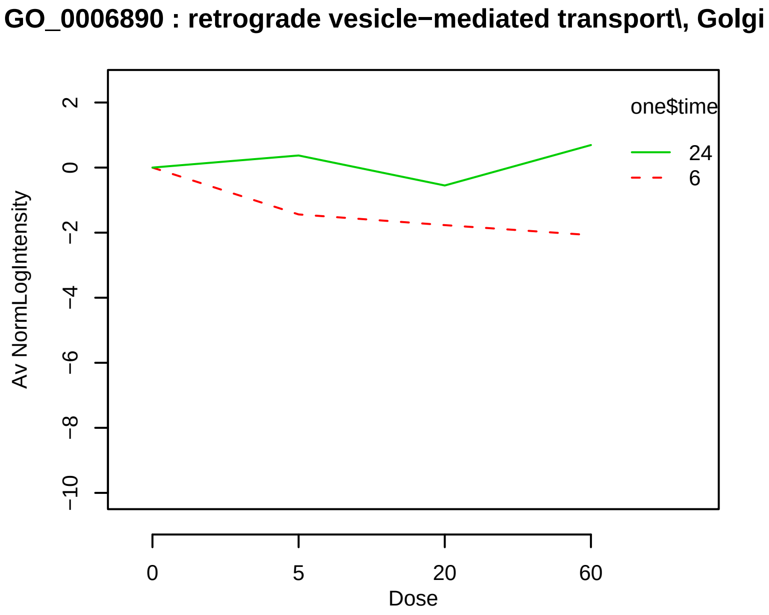
GO_0006890 : retrograde vesicle−mediated transport\, Golgi
one$time
24
6
Av NormLogIntensity
Dose
0
5
20
60

GO_0006891 : intra−Golgi vesicle−mediated transport

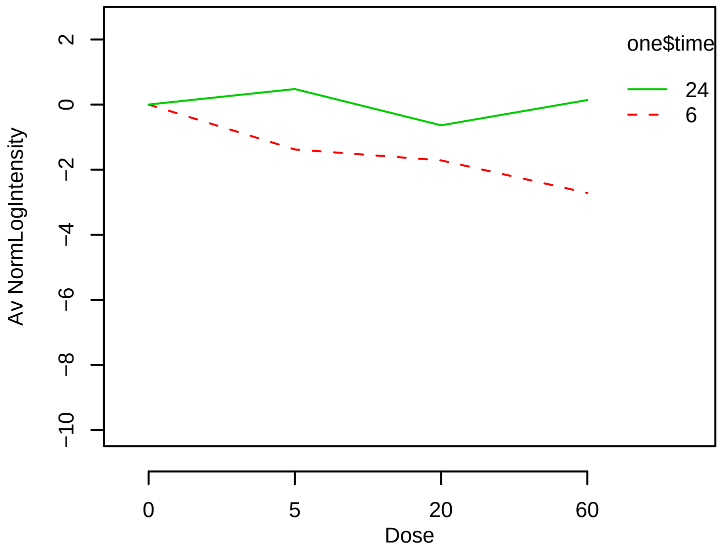

GO_0006892 : post−Golgi vesicle−mediated transport

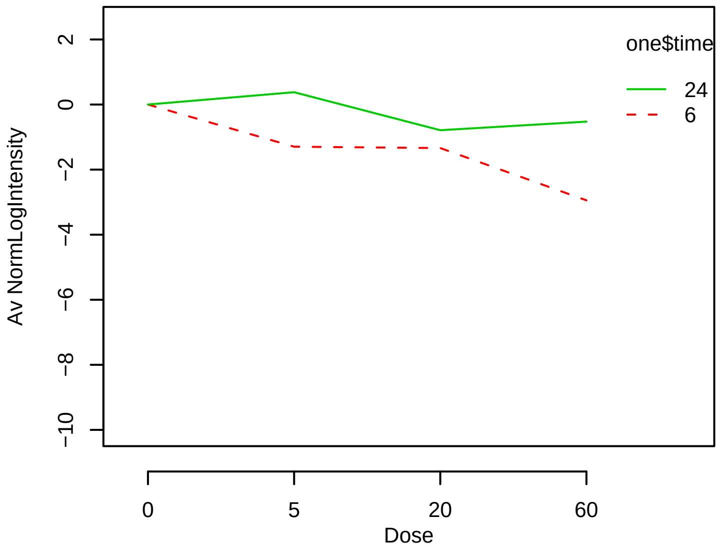

GO_0006895 : Golgi to endosome transport

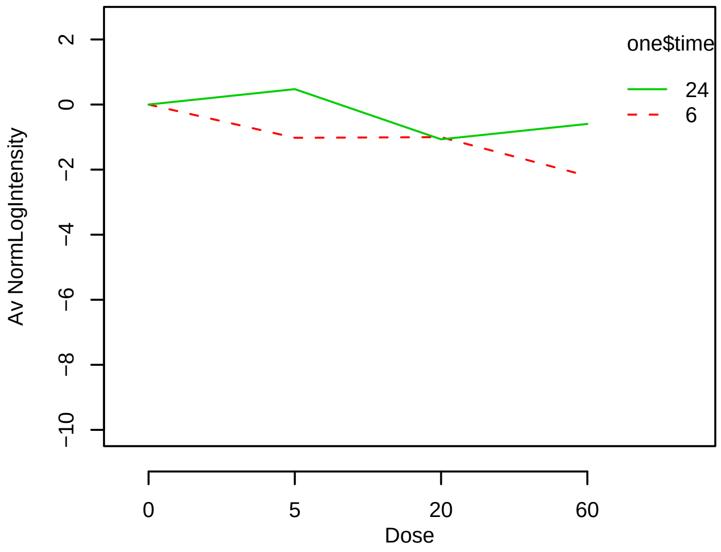

GO_0006897 : endocytosis

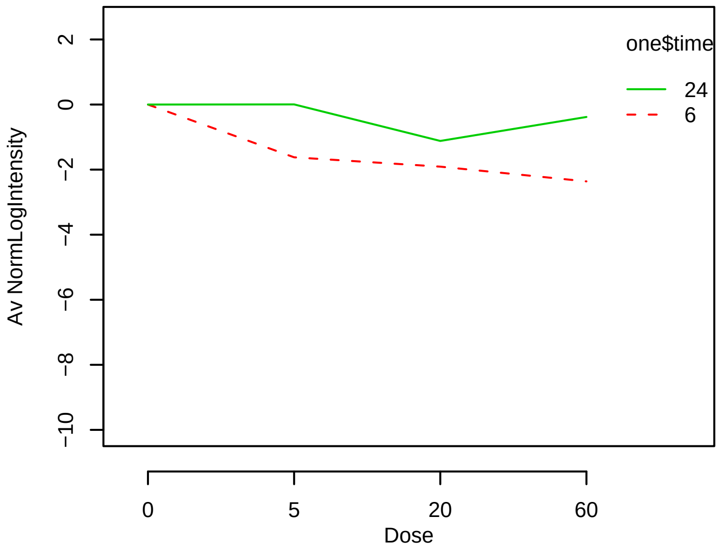

GO_0006898 : receptor−mediated endocytosis

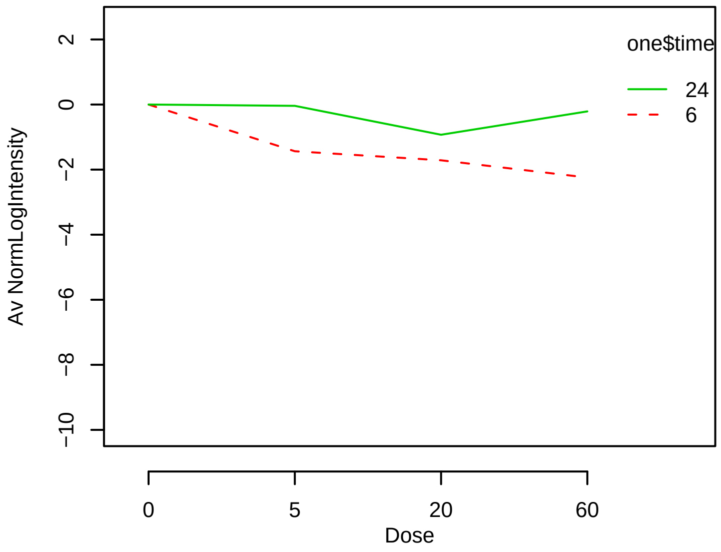

GO_0006903 : vesicle targeting

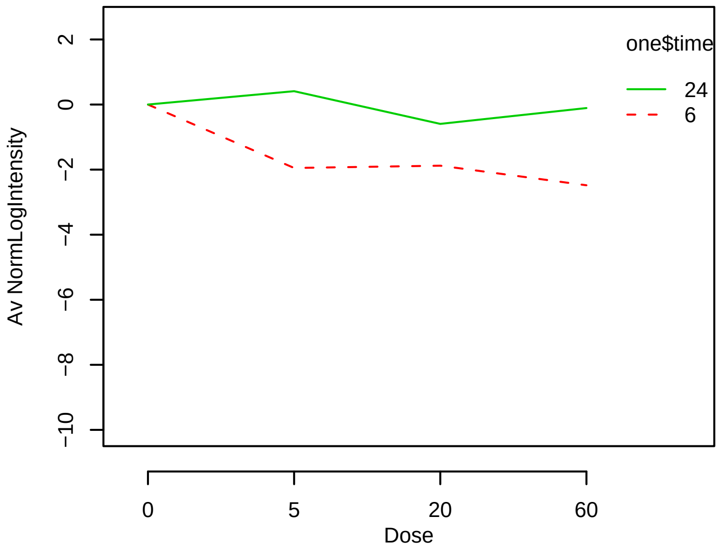

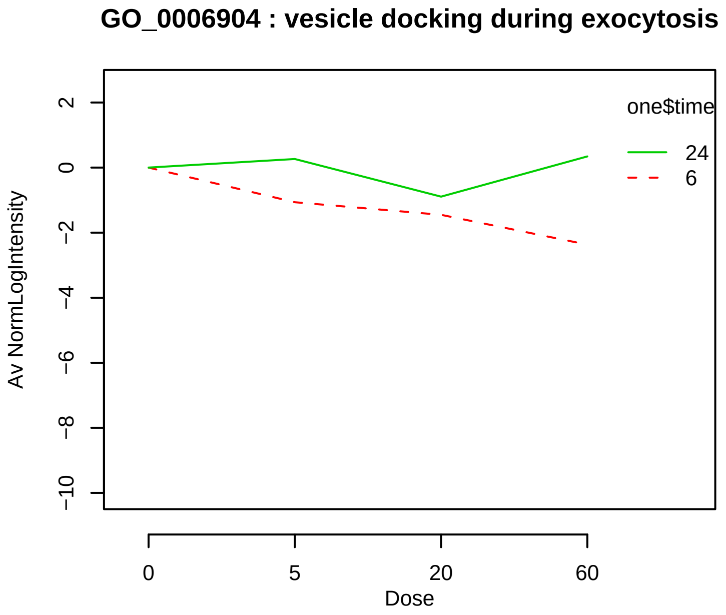
GO_0006904 : vesicle docking during exocytosis
Av NormLogIntensity
Dose
one$time
24
6

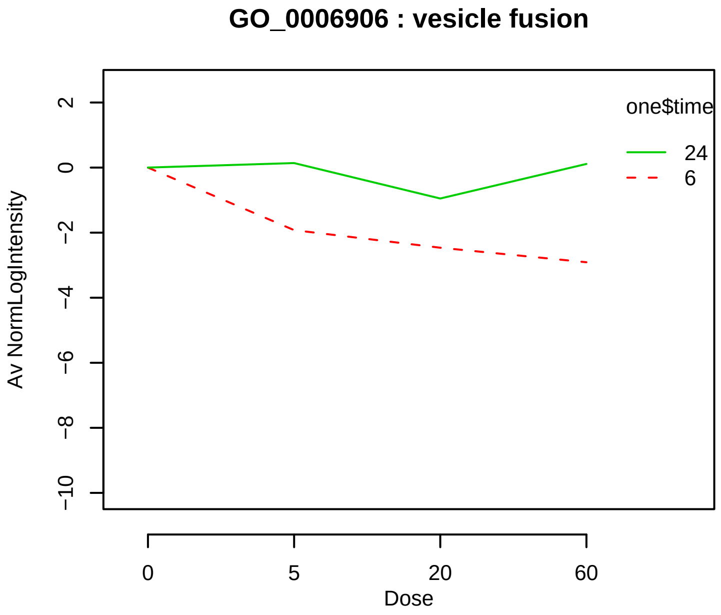
GO_0006906 : vesicle fusion
Av NormLogIntensity
Dose
one$time
24
6

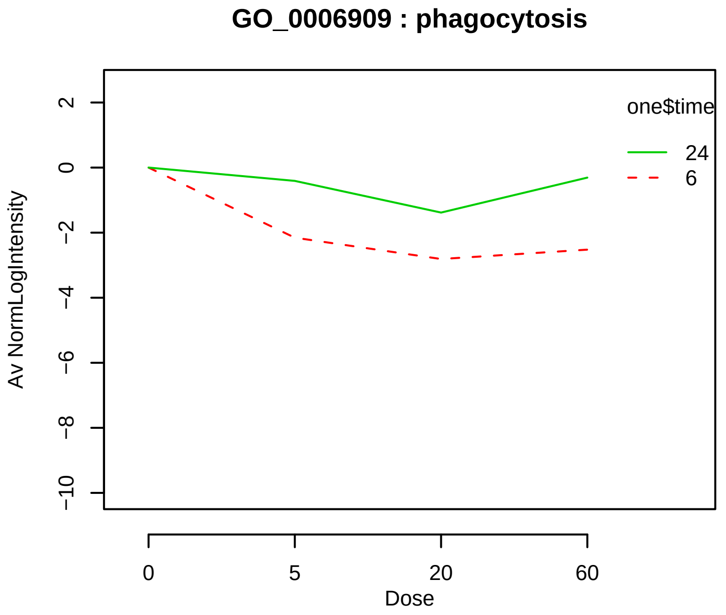
GO_0006909 : phagocytosis
Av NormLogIntensity
Dose
one$time
24
6

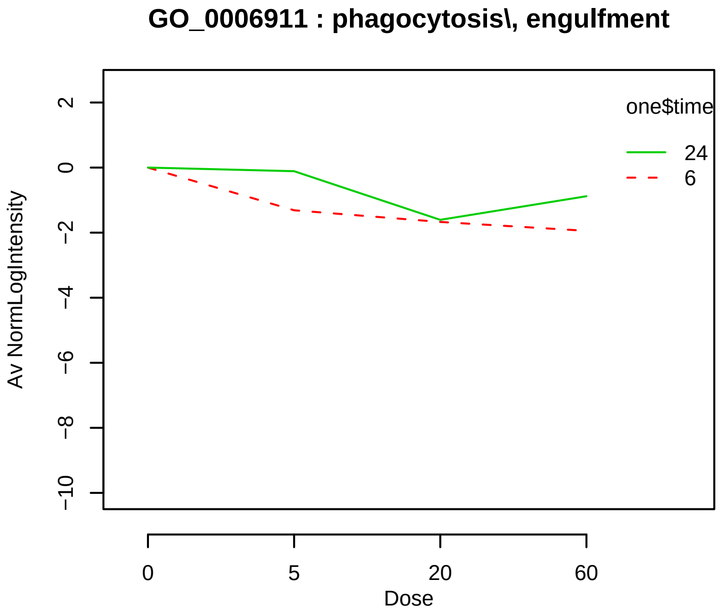
GO_0006911 : phagocytosis\, engulfment
Av NormLogIntensity
Dose
one$time
24
6

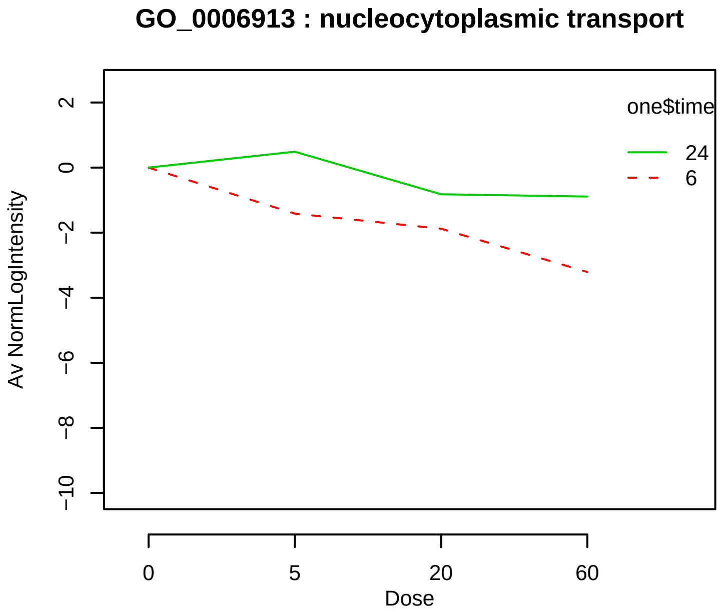
GO_0006913 : nucleocytoplasmic transport
Av NormLogIntensity
Dose
one$time
24
6

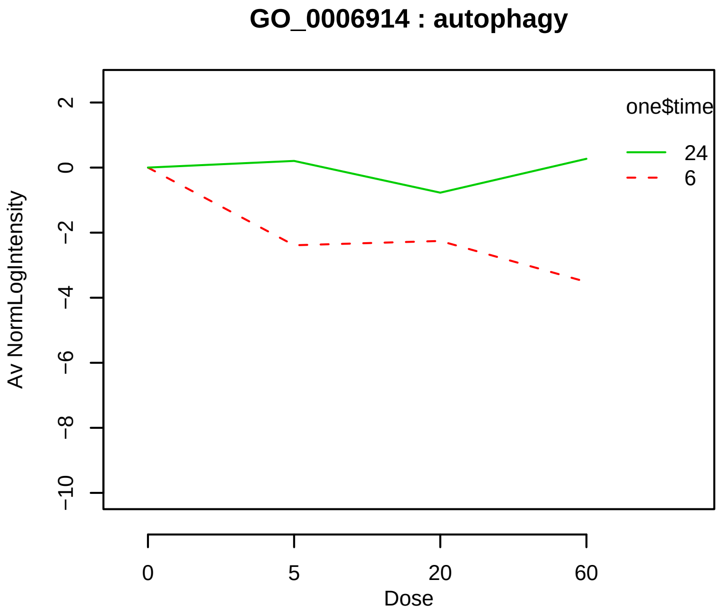
GO_0006914 : autophagy
Av NormLogIntensity
Dose
one$time
24
6

GO_0006916 : anti−apoptosis

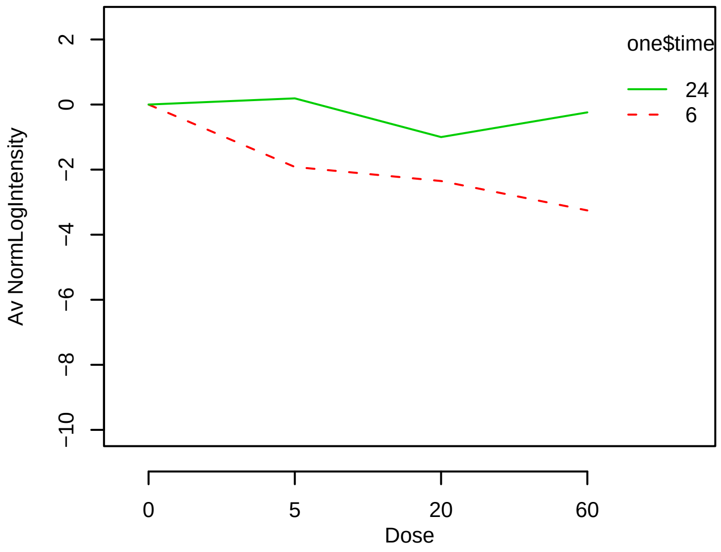

GO_0006917 : induction of apoptosis

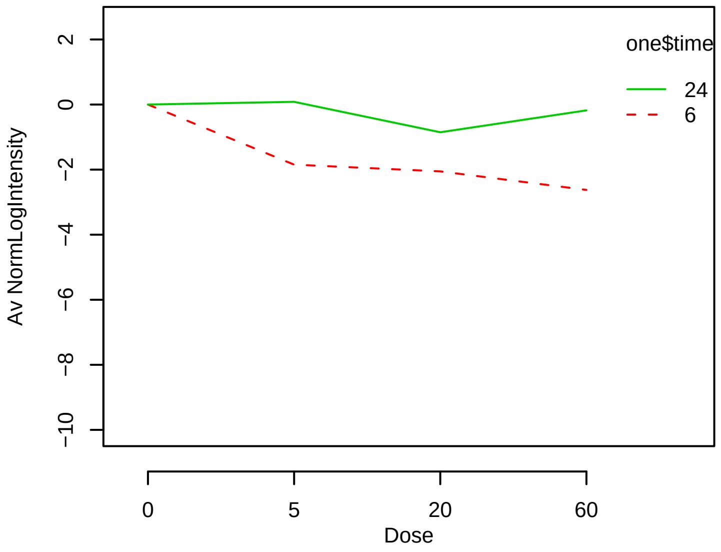

GO_0006919 : caspase activation

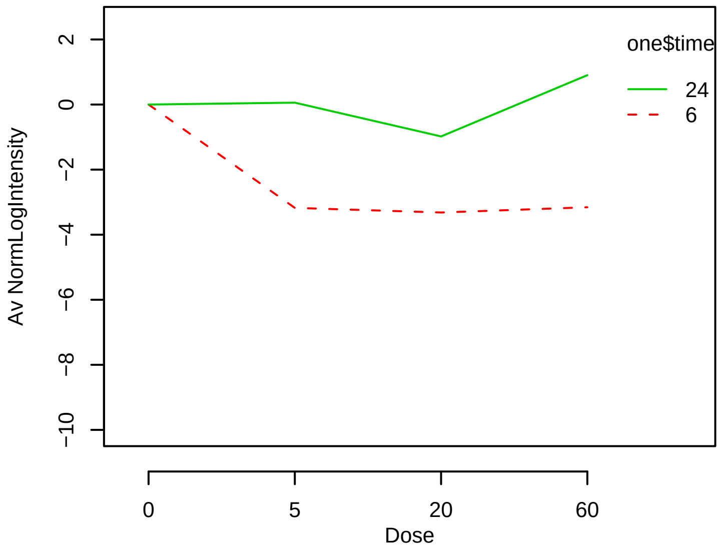

GO_0006921 : disassembly of cell structures during apopto

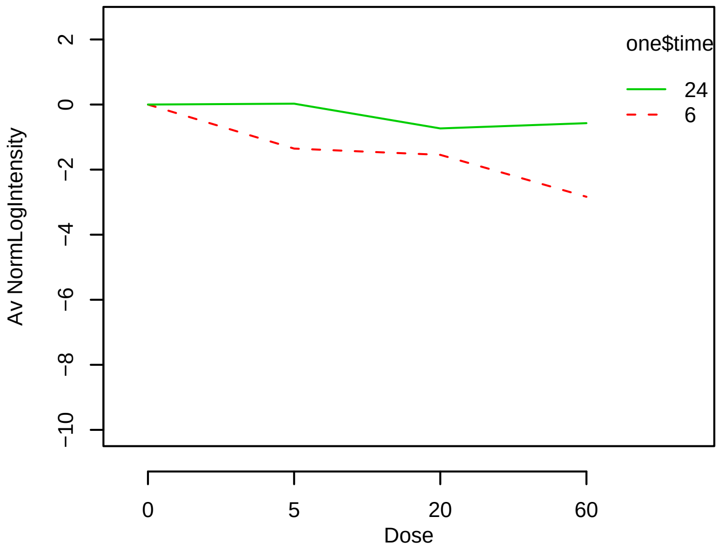

GO_0006929 : substrate−bound cell migration

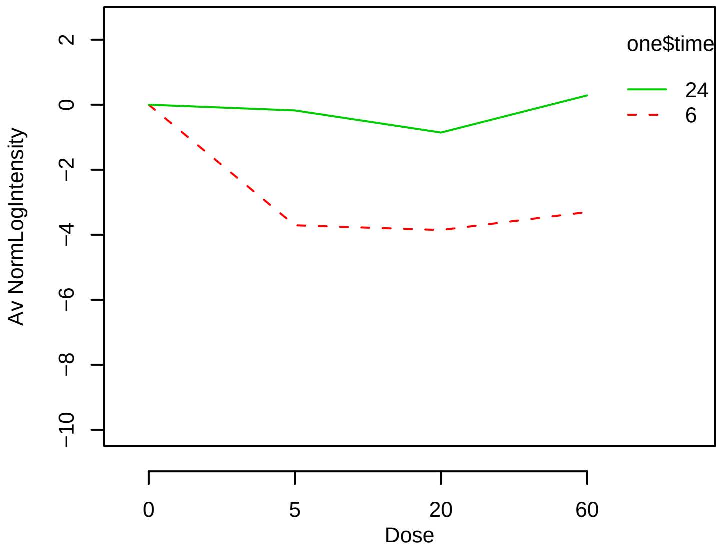

GO_0006935 : chemotaxis

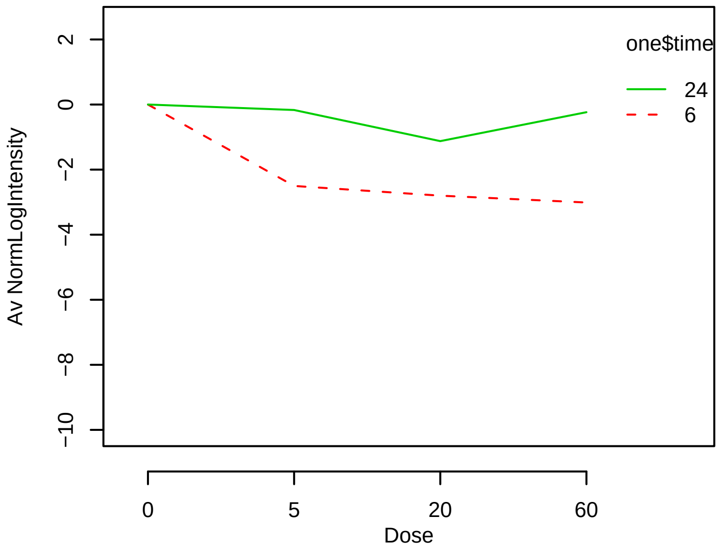

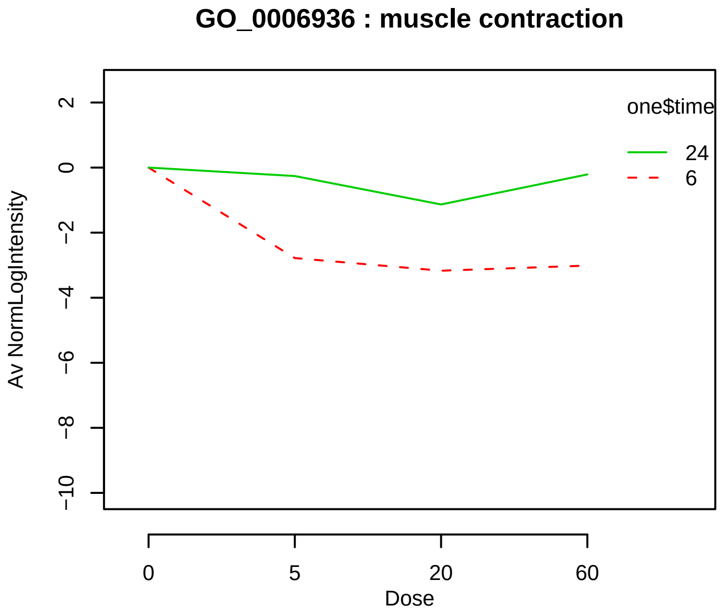

GO_0006936 : muscle contraction
Av NormLogIntensity
Dose
one$time
24
6

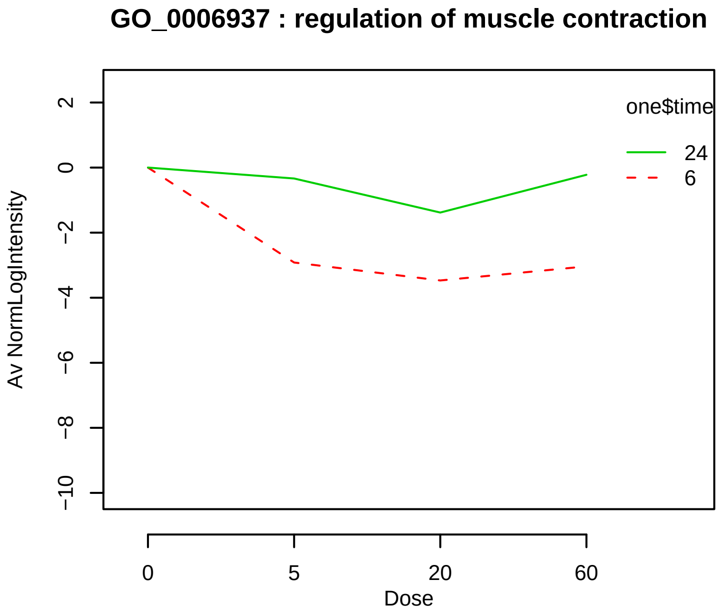

GO_0006937 : regulation of muscle contraction
Av NormLogIntensity
Dose
one$time
24
6

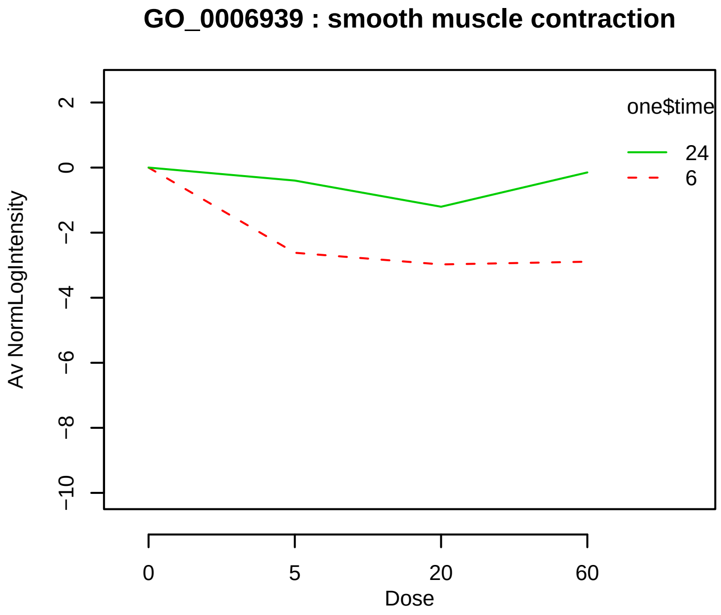

GO_0006939 : smooth muscle contraction
Av NormLogIntensity
Dose
one$time
24
6

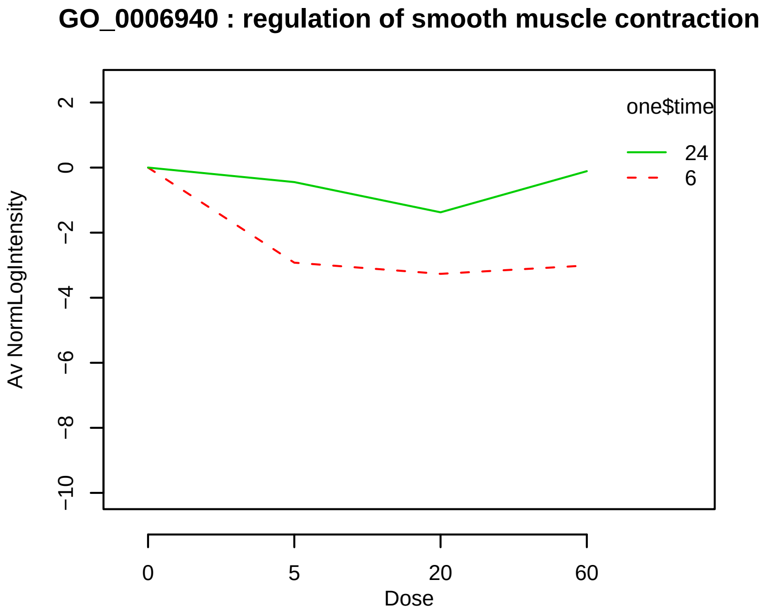

GO_0006940 : regulation of smooth muscle contraction
Av NormLogIntensity
Dose
one$time
24
6

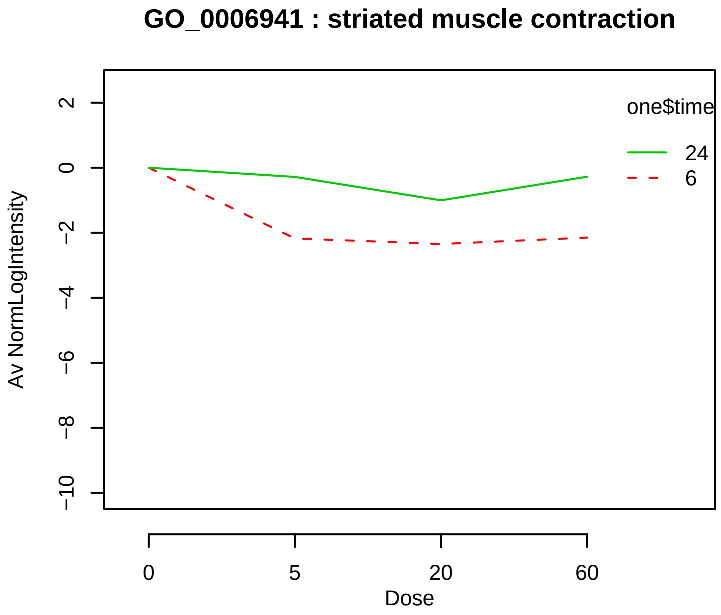

GO_0006941 : striated muscle contraction
Av NormLogIntensity
Dose
one$time
24
6

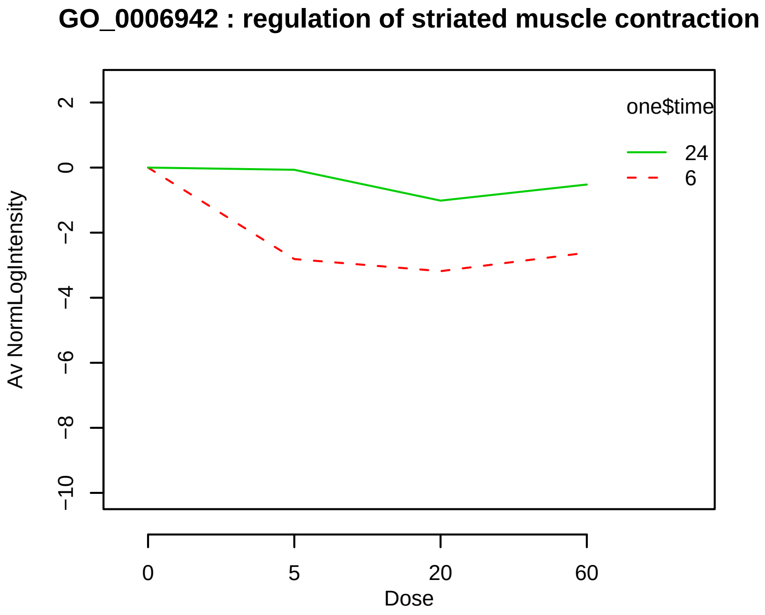

GO_0006942 : regulation of striated muscle contraction
Av NormLogIntensity
Dose
one$time
24
6

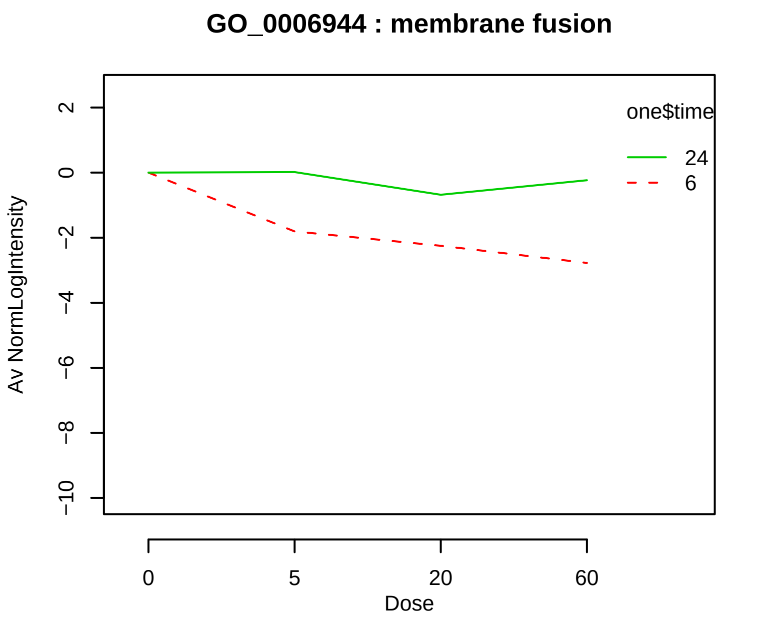
GO_0006944 : membrane fusion
one$time
24
6
Av NormLogIntensity
2
0
−2
−4
−6
−8
−10
0
5
20
60
Dose

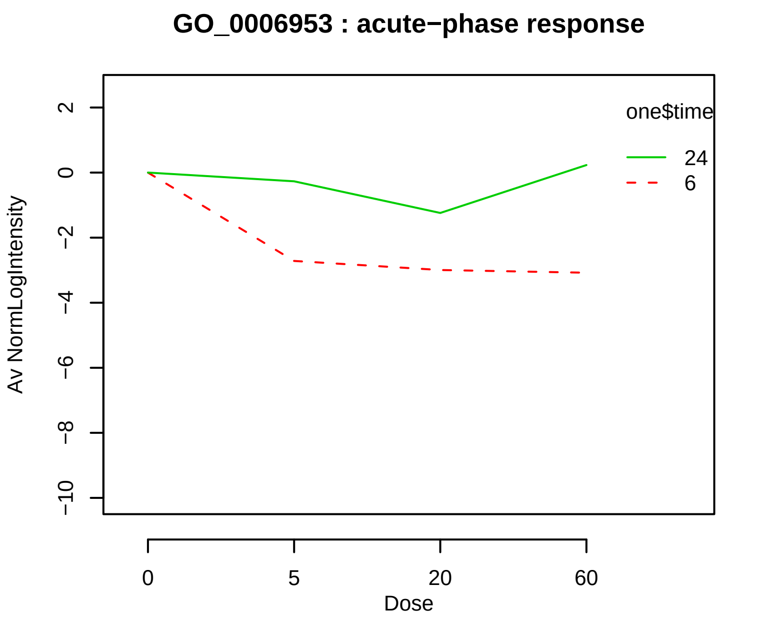
GO_0006953 : acute−phase response
one$time
24
6
Av NormLogIntensity
2
0
−2
−4
−6
−8
−10
0
5
20
60
Dose

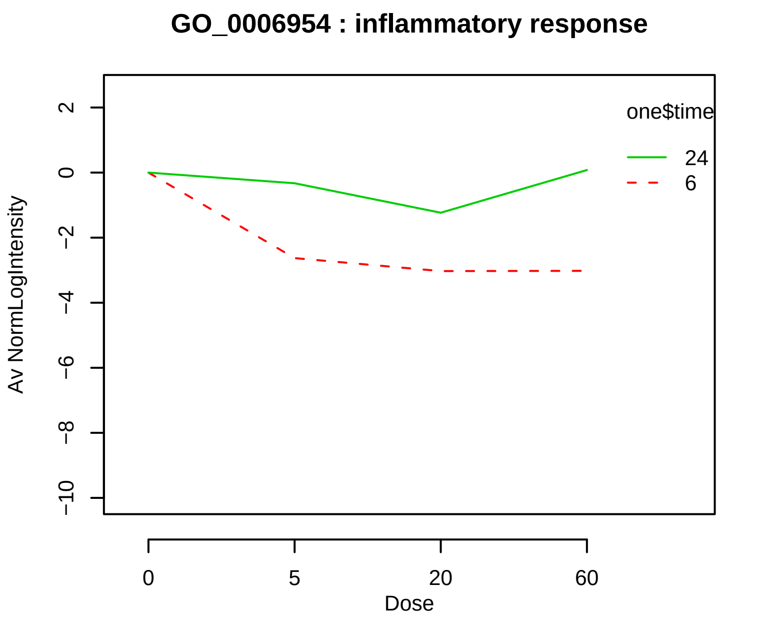
GO_0006954 : inflammatory response
one$time
24
6
Av NormLogIntensity
2
0
−2
−4
−6
−8
−10
0
5
20
60
Dose

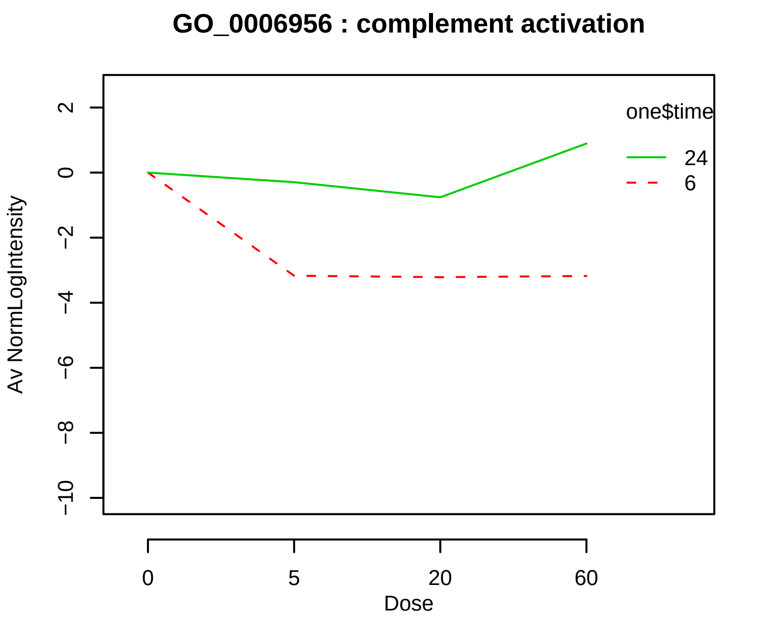
GO_0006956 : complement activation
one$time
24
6
Av NormLogIntensity
2
0
−2
−4
−6
−8
−10
0
5
20
60
Dose

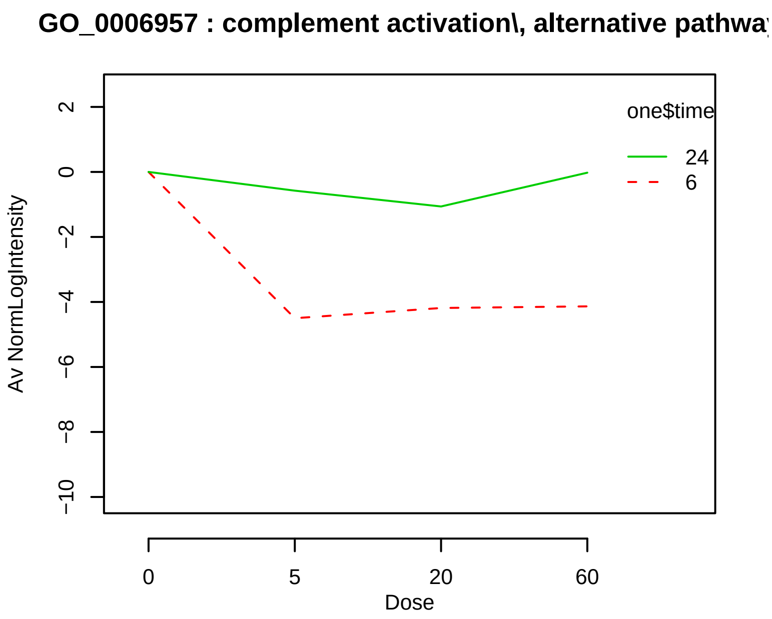
GO_0006957 : complement activation\, alternative pathwa
one$time
24
6
Av NormLogIntensity
2
0
−2
−4
−6
−8
−10
0
5
20
60
Dose

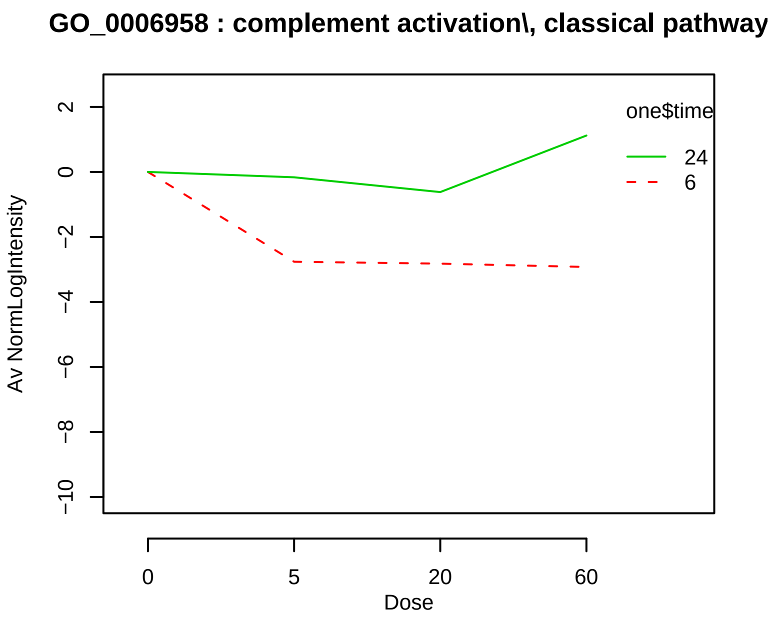
GO_0006958 : complement activation\, classical pathway
one$time
24
6
Av NormLogIntensity
2
0
−2
−4
−6
−8
−10
0
5
20
60
Dose

GO_0006959 : humoral immune response

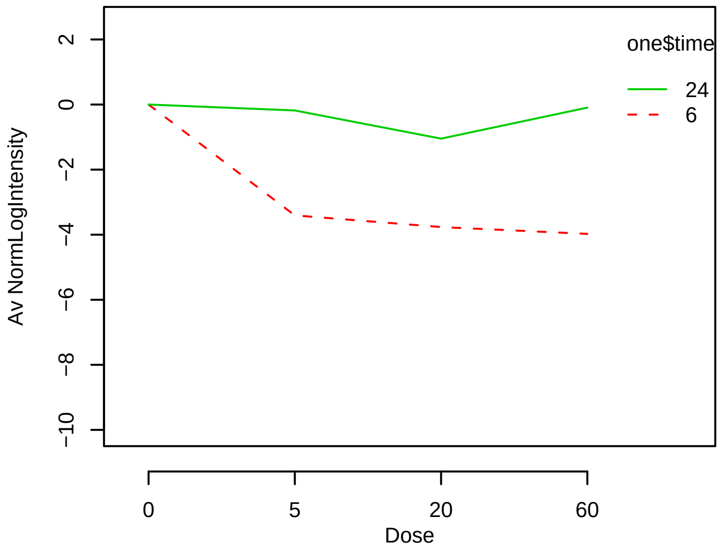

GO_0006968 : cellular defense response

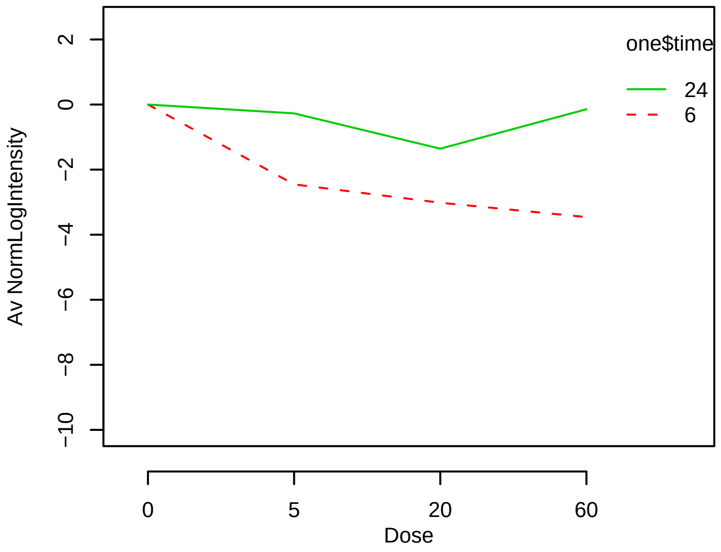

GO_0006970 : response to osmotic stress

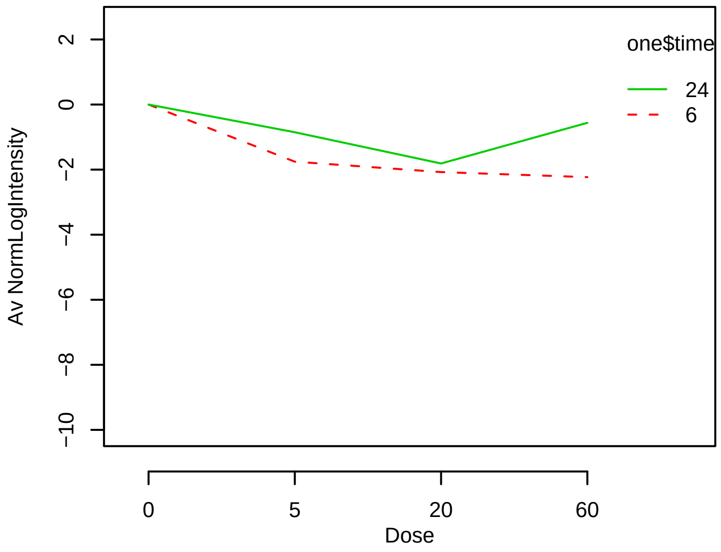

GO_0006972 : hyperosmotic response

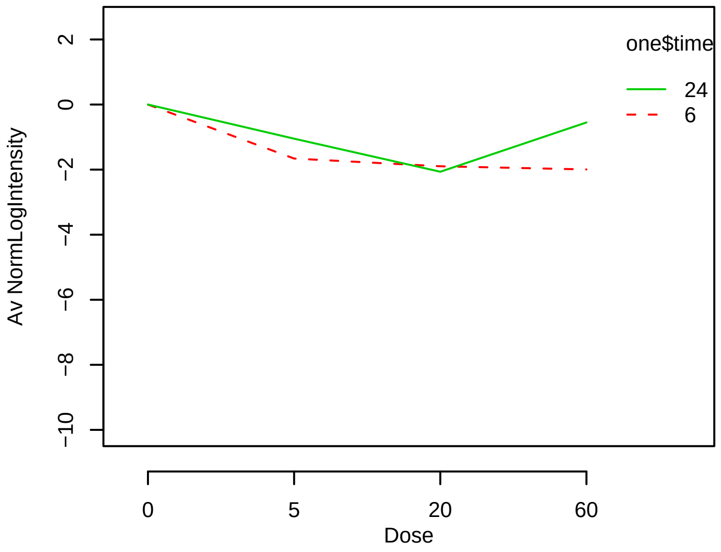

O_0006977 : DNA damage response\, signal transduction by

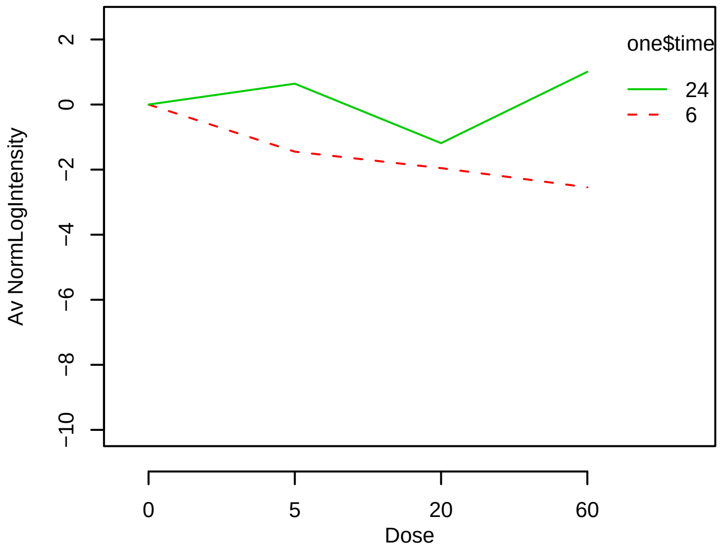

GO_0006979 : response to oxidative stress

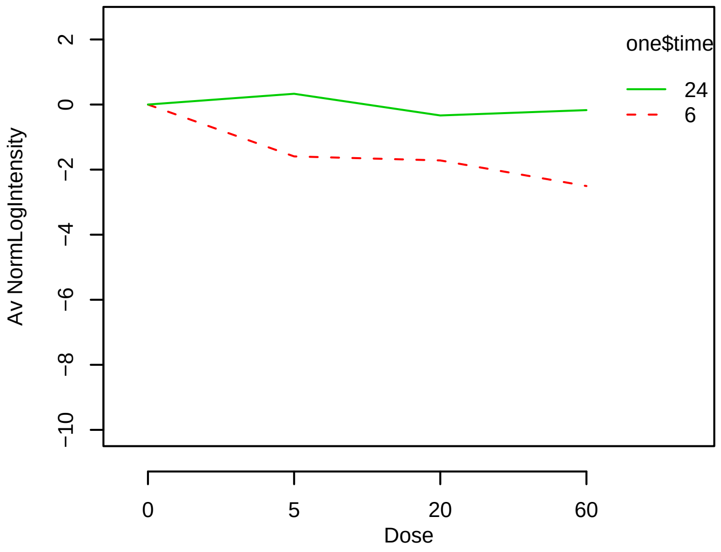

GO_0006984 : ER−nuclear signaling pathway

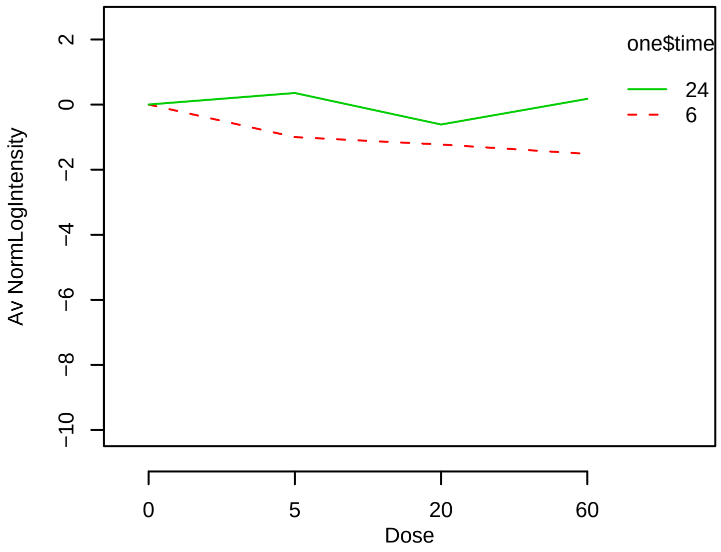

GO_0006986 : response to unfolded protein

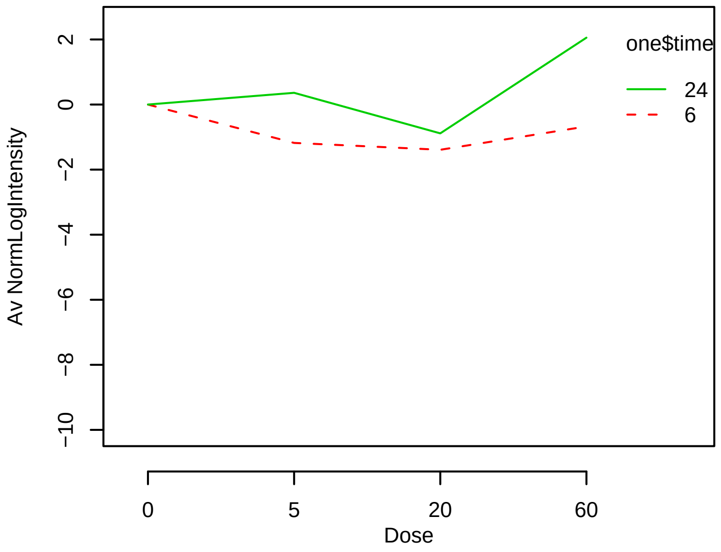

GO_0006997 : nuclear organization and biogenesis

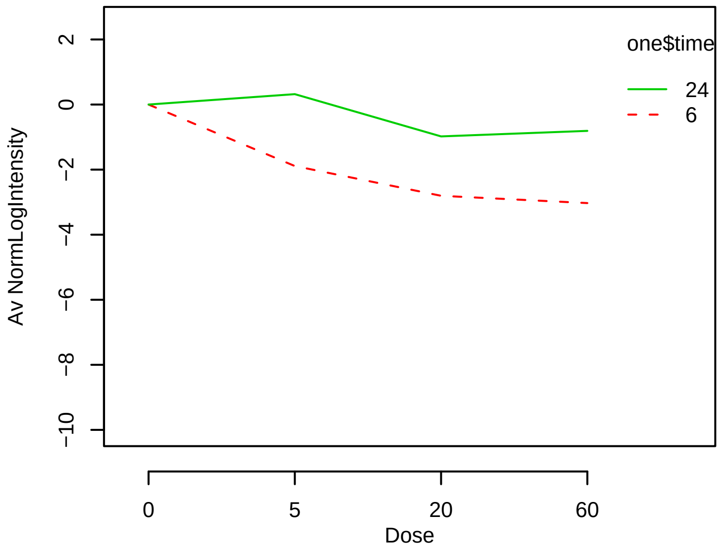

GO_0007004 : telomere maintenance via telomerase

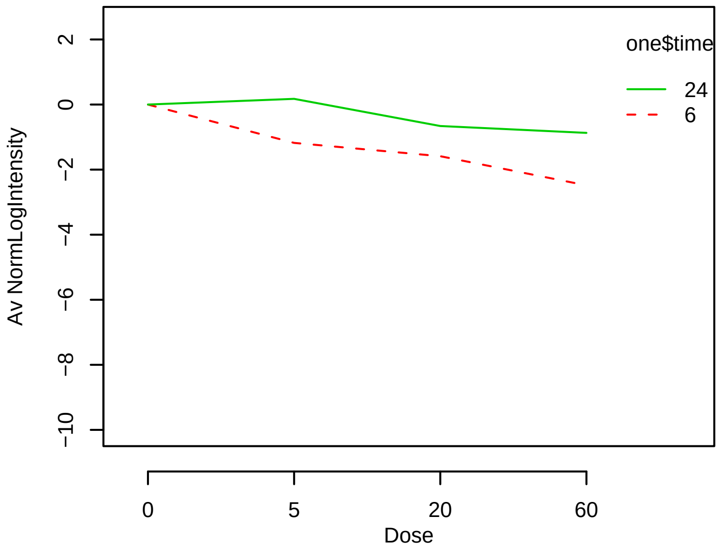

GO_0007005 : mitochondrion organization and biogenesiO_0007006 : mitochondrial membrane organization and bioge

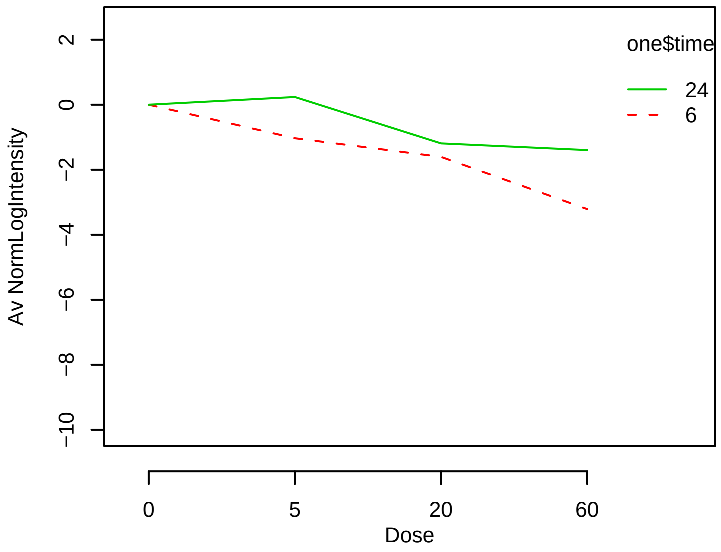

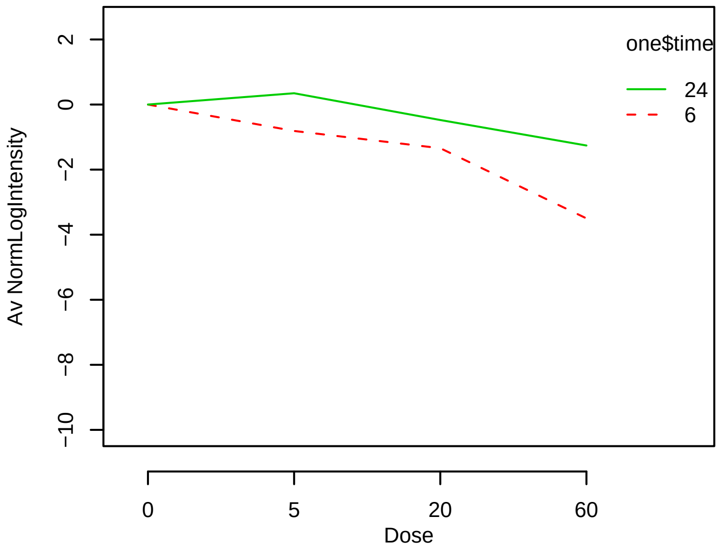

O_0007007 : inner mitochondrial membrane organization anc

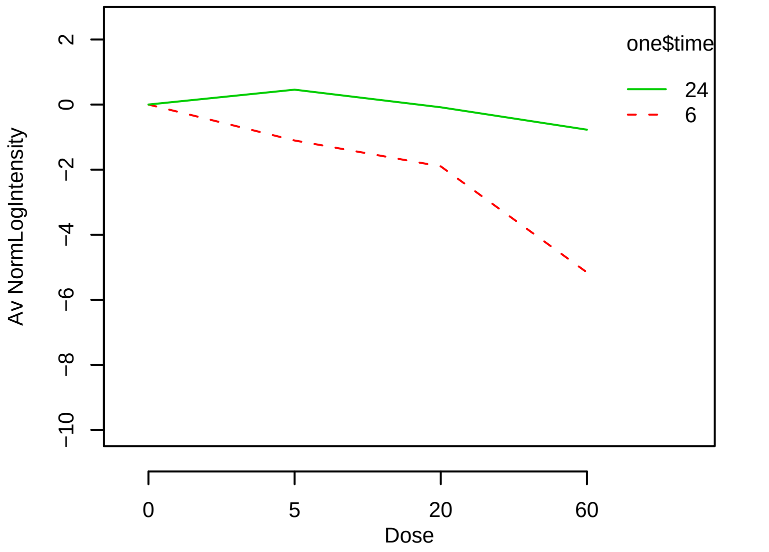

GO_0007009 : plasma membrane organization and biogenes

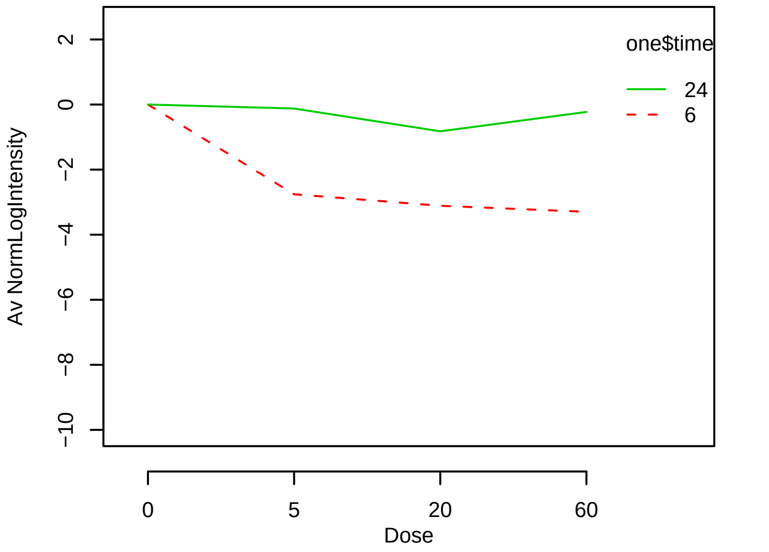

GO_0007015 : actin filament organization

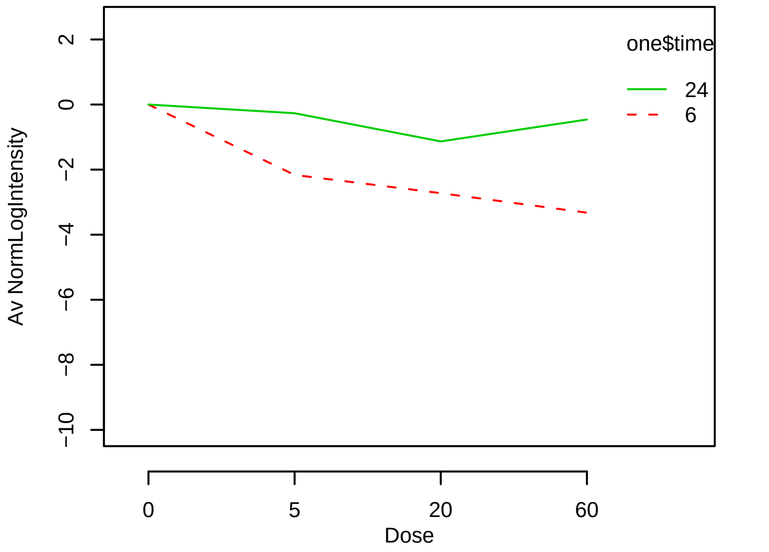

GO_0007016 : cytoskeletal anchoring

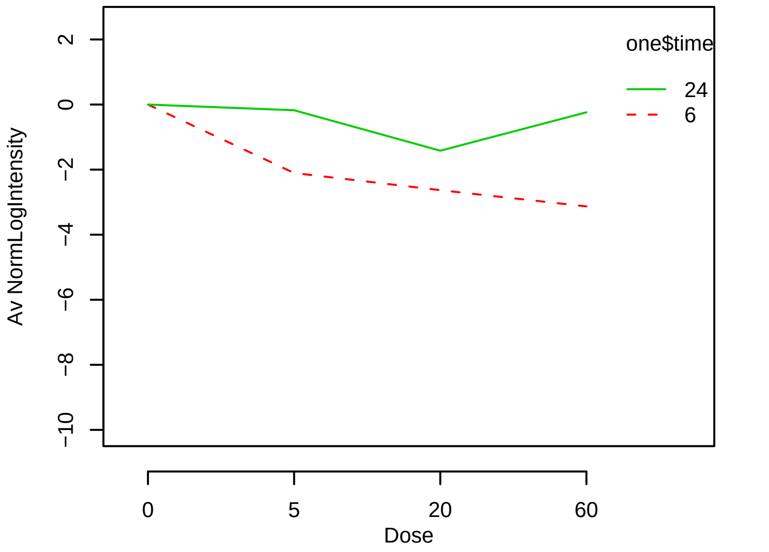

GO_0007017 : microtubule−based process

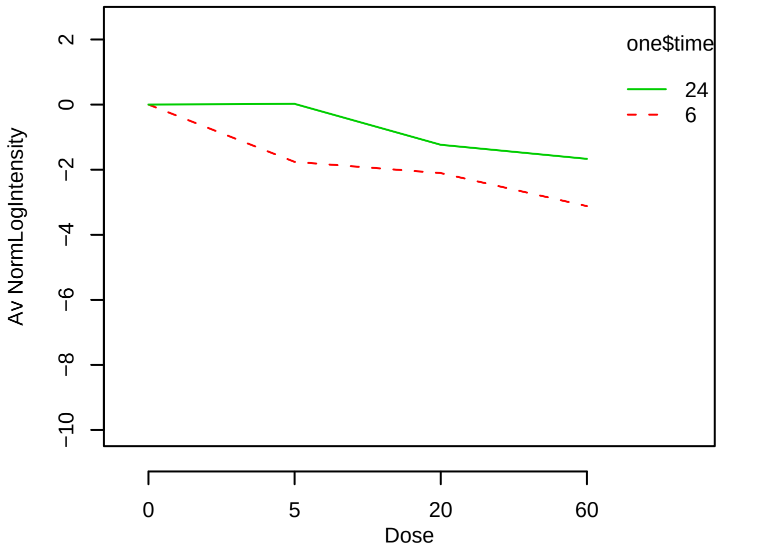

GO_0007018 : microtubule−based movement

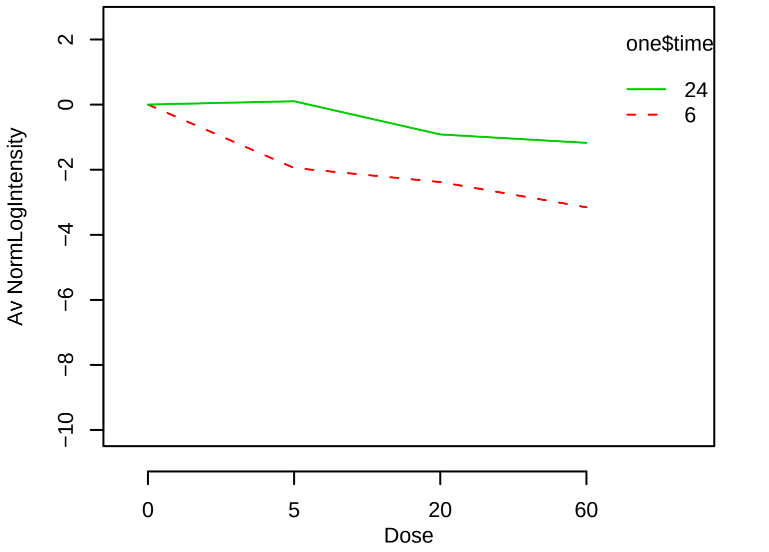

GO_0007019 : microtubule depolymerization

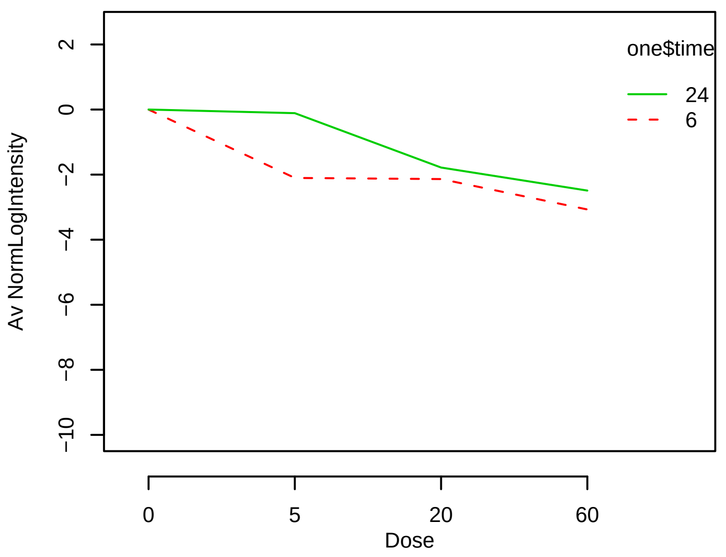

GO_0007020 : microtubule nucleation

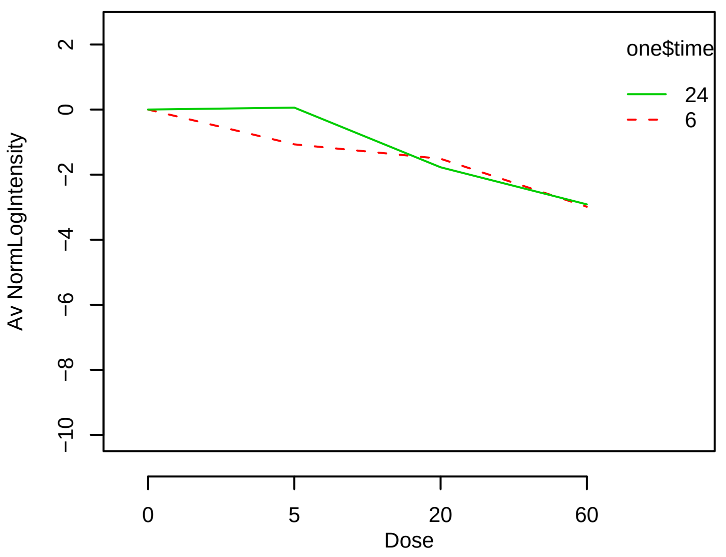

GO_0007021 : tubulin folding

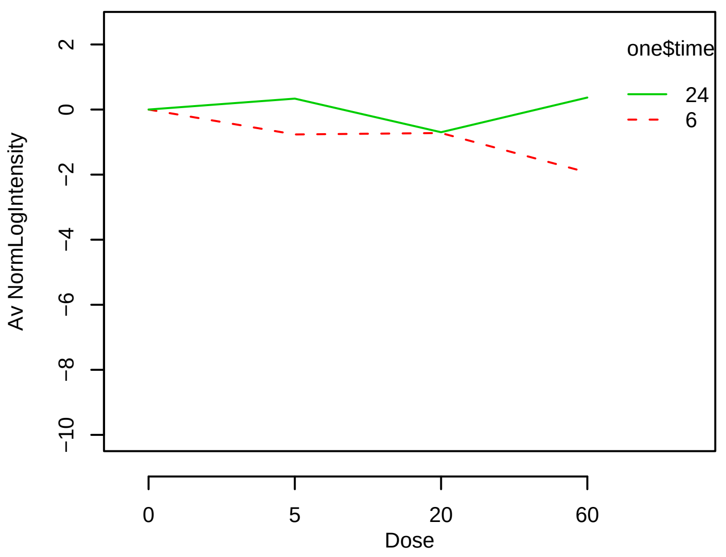

GO_0007025 : beta−tubulin folding

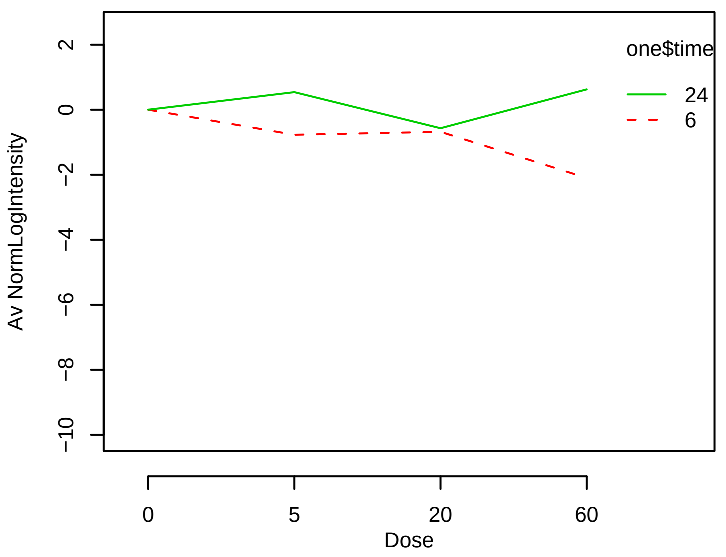

GO_0007026 : negative regulation of microtubule depolymeri

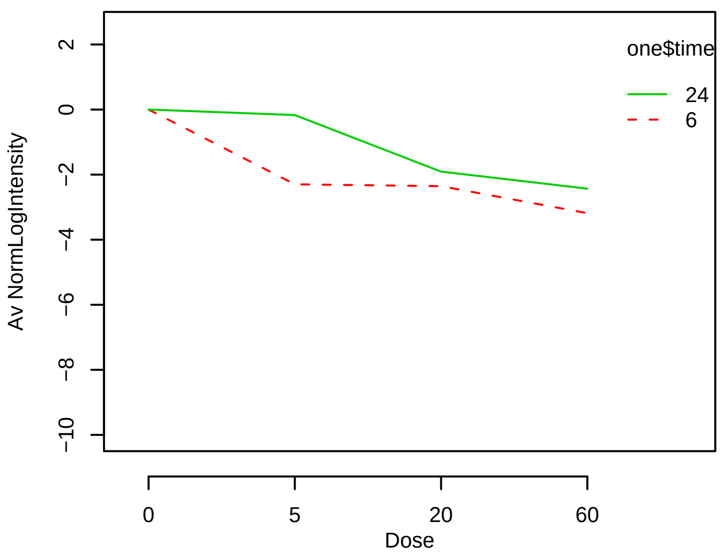

GO_0007028 : cytoplasm organization and biogenesis

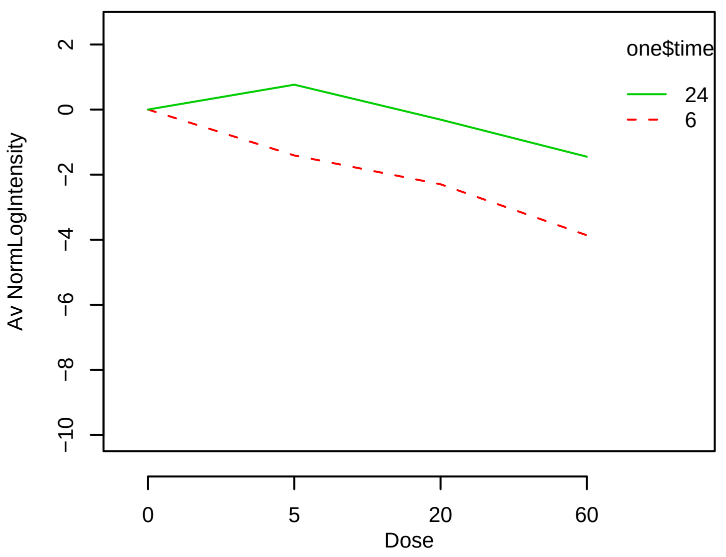

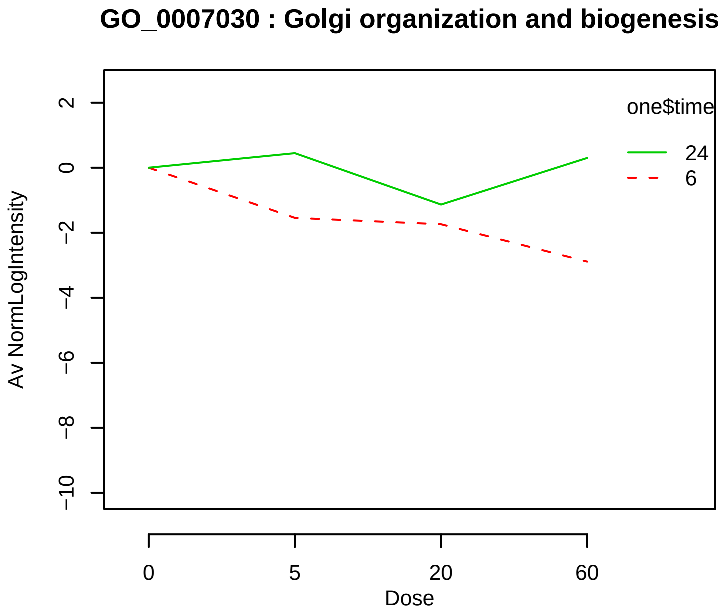
GO_0007030 : Golgi organization and biogenesis
Av NormLogIntensity
Dose
one$time
24
6
2
0
−2
−4
−6
−8
−10
0
5
20
60

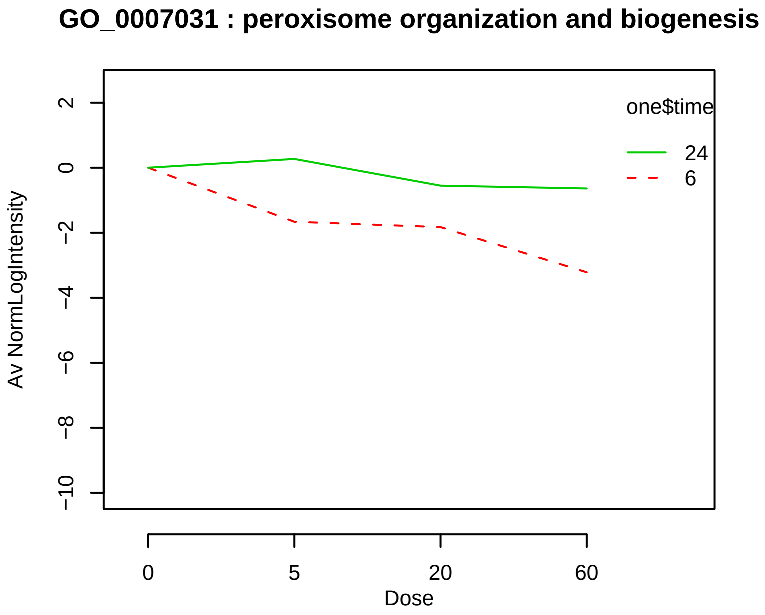
GO_0007031 : peroxisome organization and biogenesis
Av NormLogIntensity
Dose
one$time
24
6
2
0
−2
−4
−6
−8
−10
0
5
20
60

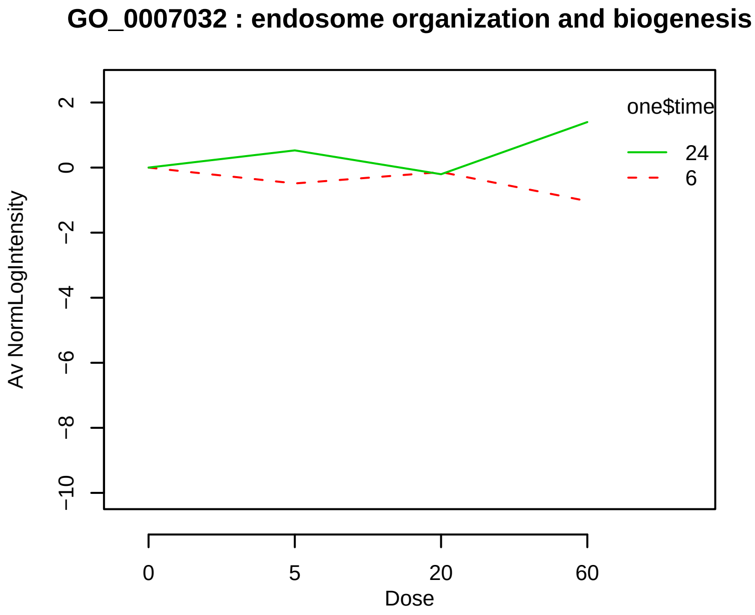
GO_0007032 : endosome organization and biogenesis
Av NormLogIntensity
Dose
one$time
24
6
2
0
−2
−4
−6
−8
−10
0
5
20
60

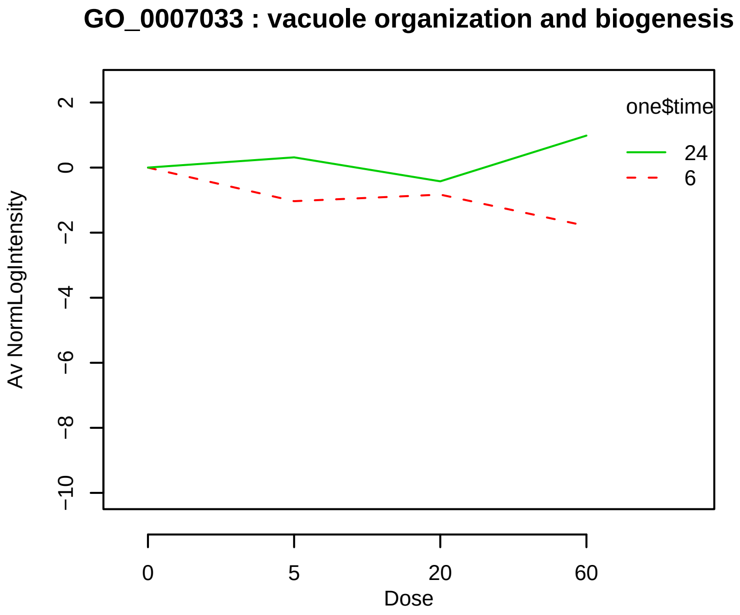
GO_0007033 : vacuole organization and biogenesis
Av NormLogIntensity
Dose
one$time
24
6
2
0
−2
−4
−6
−8
−10
0
5
20
60

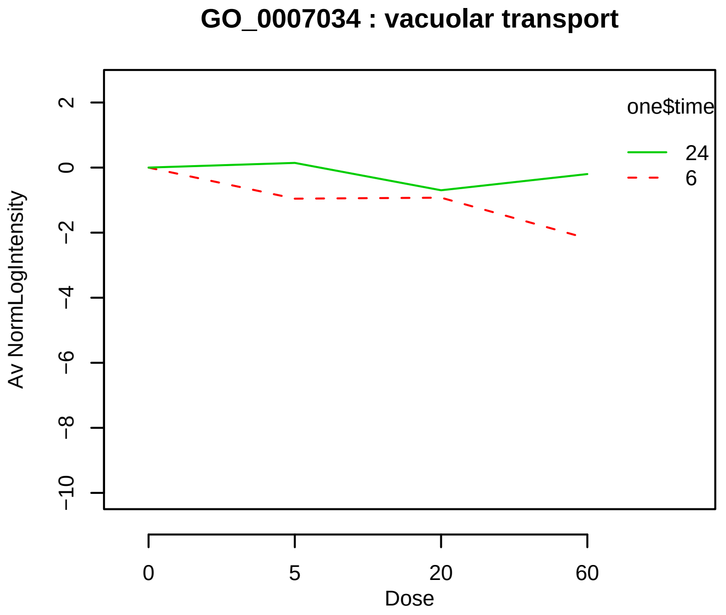
GO_0007034 : vacuolar transport
Av NormLogIntensity
Dose
one$time
24
6
2
0
−2
−4
−6
−8
−10
0
5
20
60

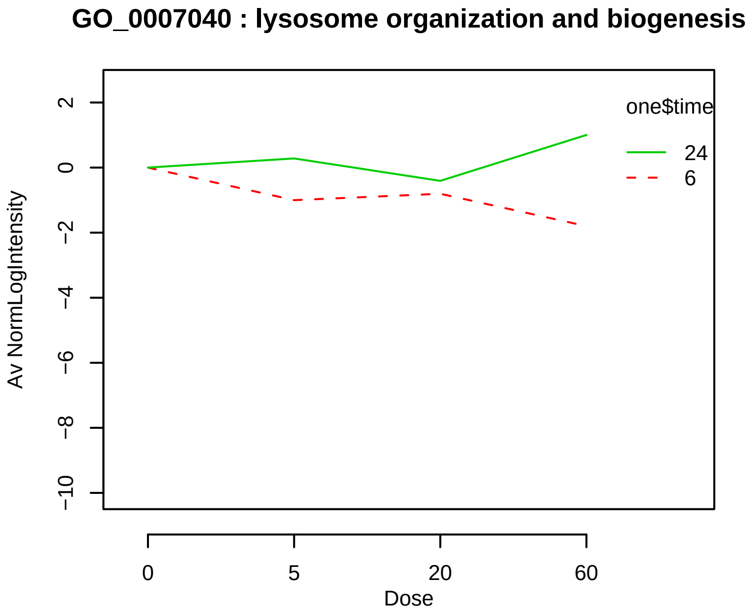
GO_0007040 : lysosome organization and biogenesis
Av NormLogIntensity
Dose
one$time
24
6
2
0
−2
−4
−6
−8
−10
0
5
20
60

GO_0007041 : lysosomal transport

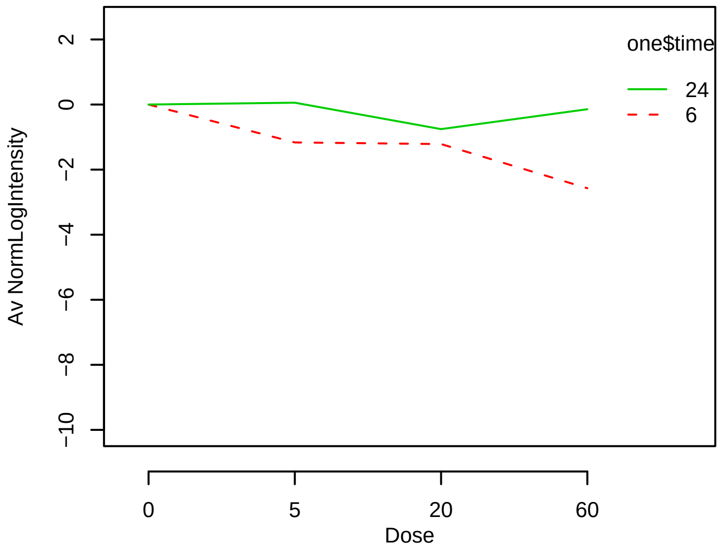

GO_0007043 : intercellular junction assembly

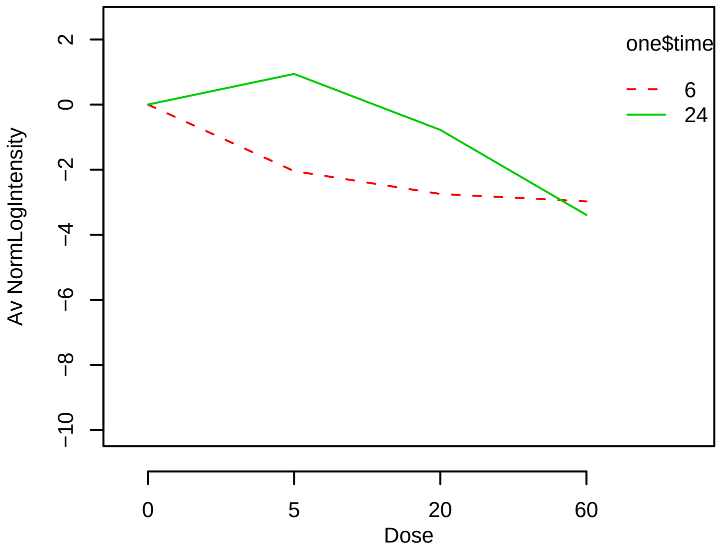

GO_0007044 : cell−substrate junction assembly

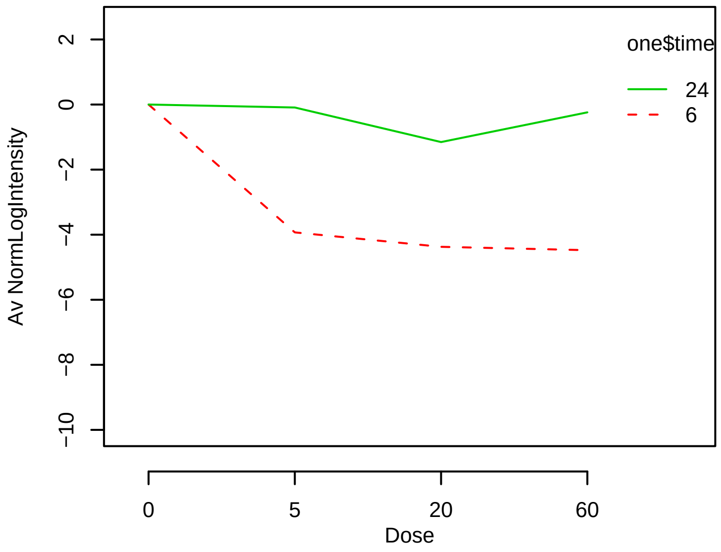

GO_0007046 : ribosome biogenesis

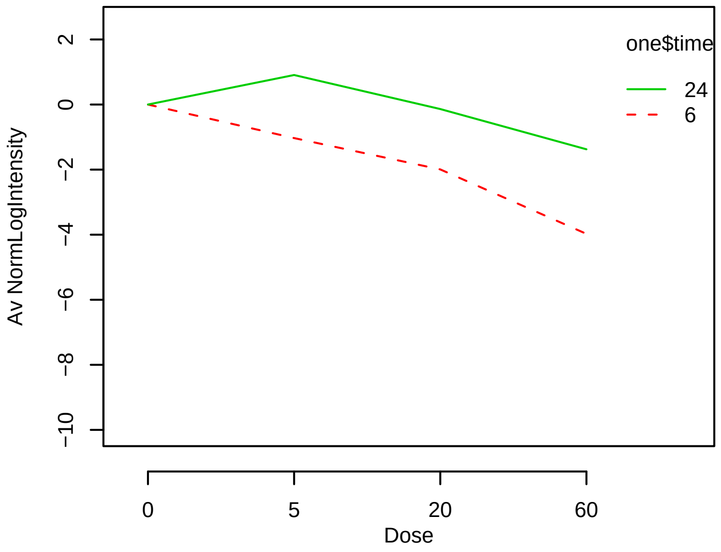

GO_0007050 : cell cycle arrest

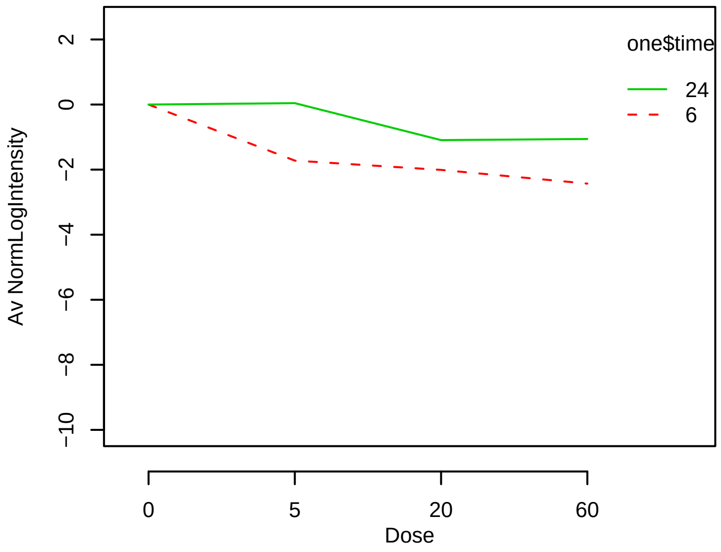

GO_0007051 : spindle organization and biogenesis

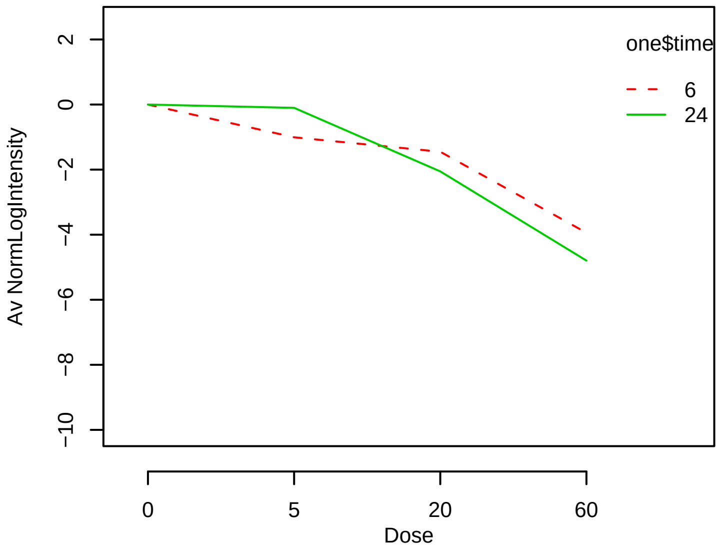

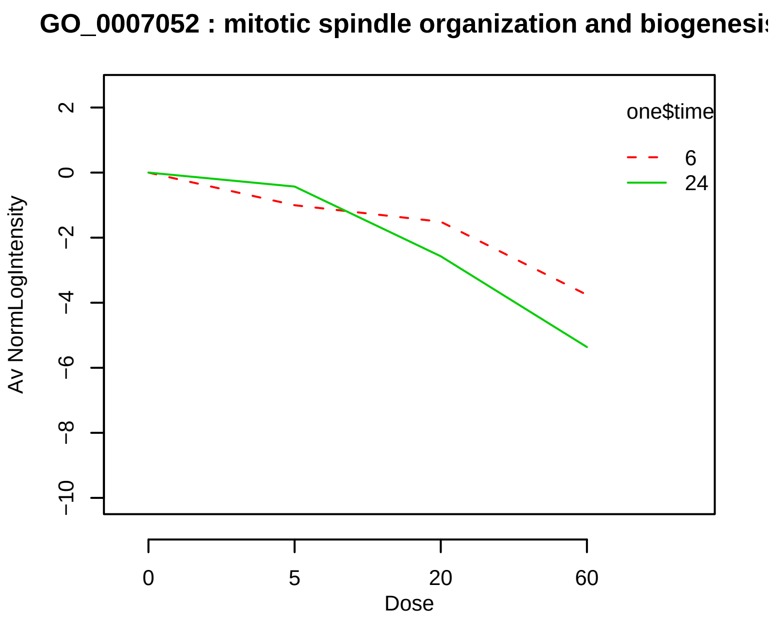
GO_0007052 : mitotic spindle organization and biogenesis
Av NormLogIntensity
2
0
−2
−4
−6
−8
−10
one$time
6
24
0
5
20
60
Dose

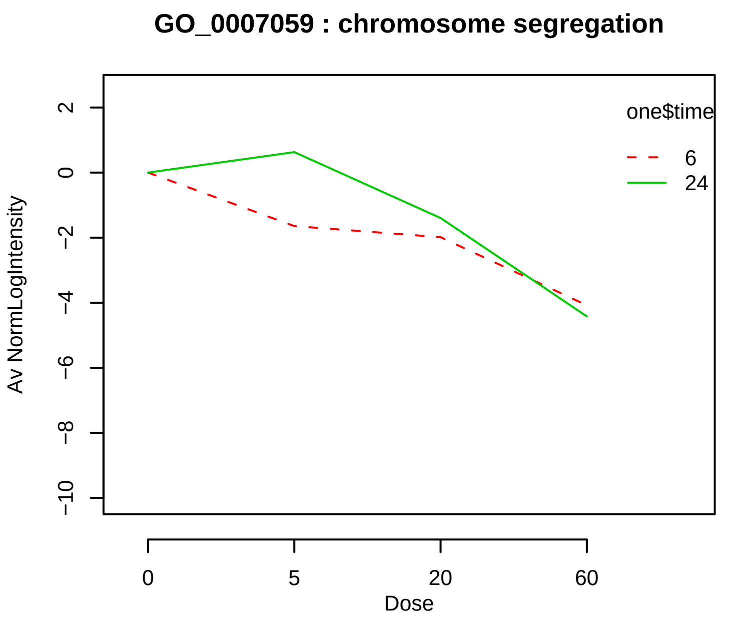
GO_0007059 : chromosome segregation
Av NormLogIntensity
2
0
−2
−4
−6
−8
−10
one$time
6
24
0
5
20
60
Dose

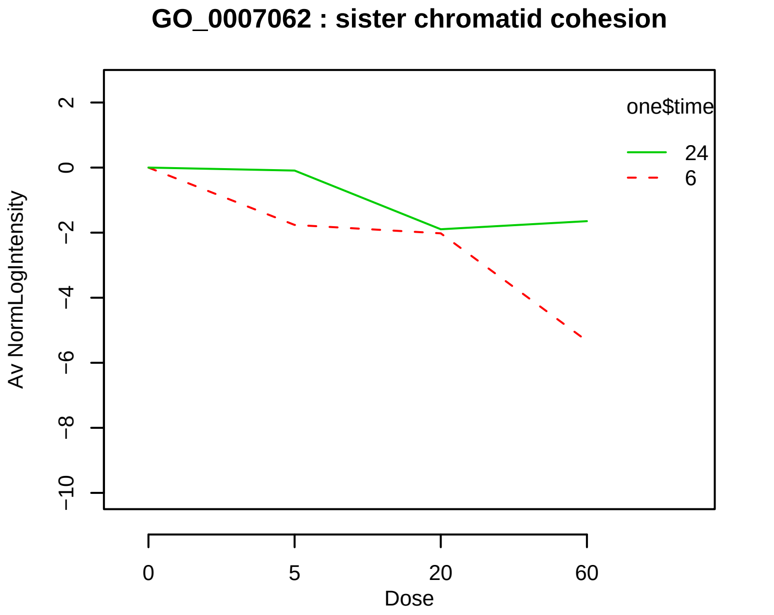
GO_0007062 : sister chromatid cohesion
Av NormLogIntensity
2
0
−2
−4
−6
−8
−10
one$time
24
6
0
5
20
60
Dose

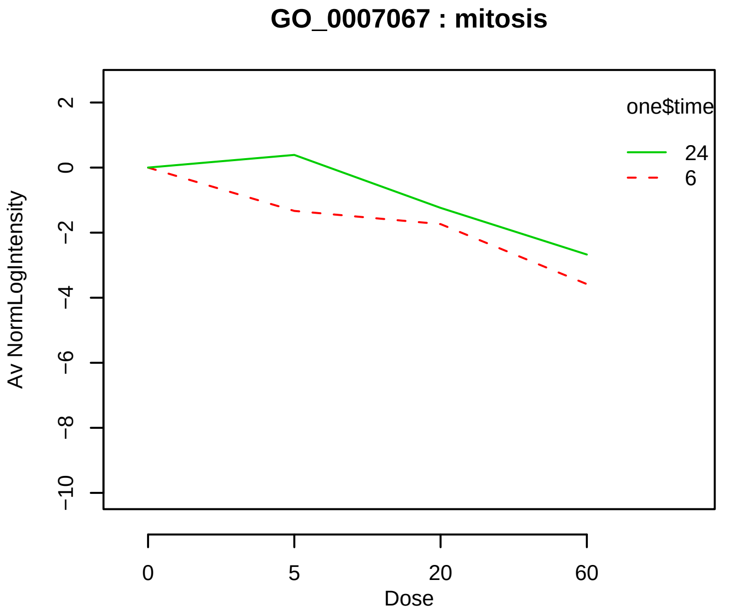
GO_0007067 : mitosis
Av NormLogIntensity
2
0
−2
−4
−6
−8
−10
one$time
24
6
0
5
20
60
Dose

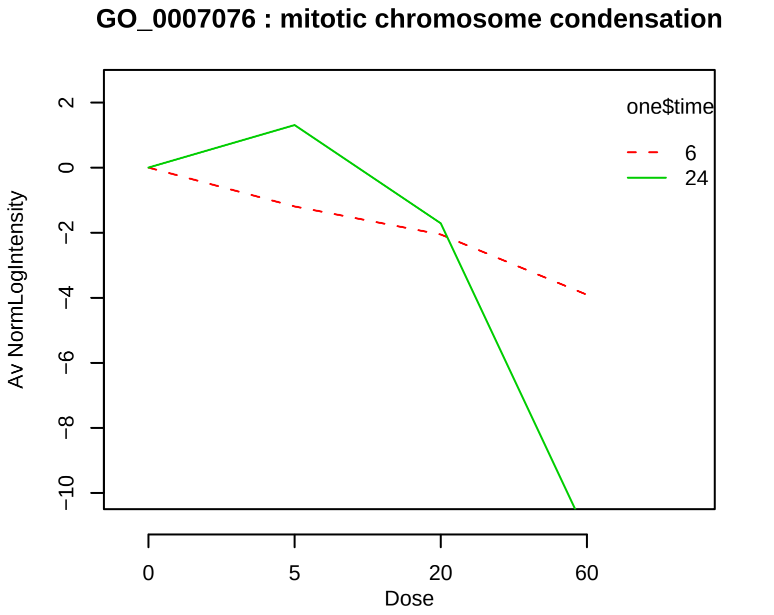
GO_0007076 : mitotic chromosome condensation
Av NormLogIntensity
2
0
−2
−4
−6
−8
−10
one$time
6
24
0
5
20
60
Dose

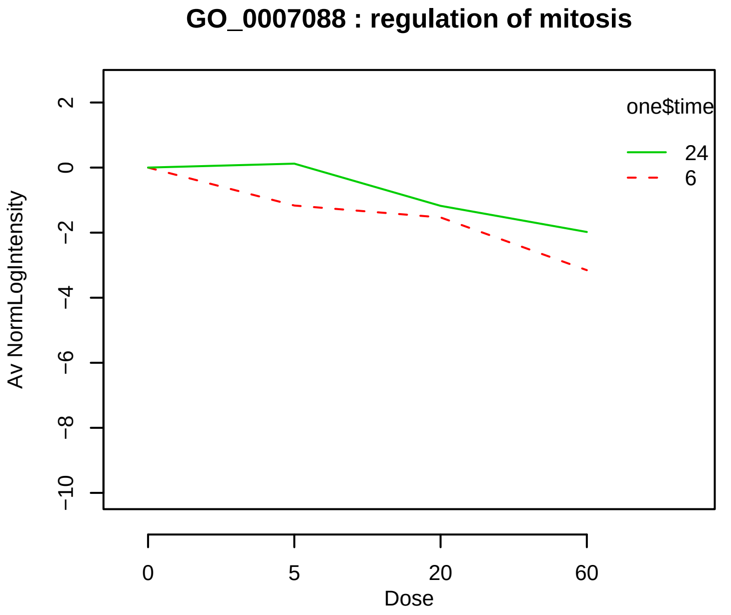
GO_0007088 : regulation of mitosis
Av NormLogIntensity
2
0
−2
−4
−6
−8
−10
one$time
24
6
0
5
20
60
Dose

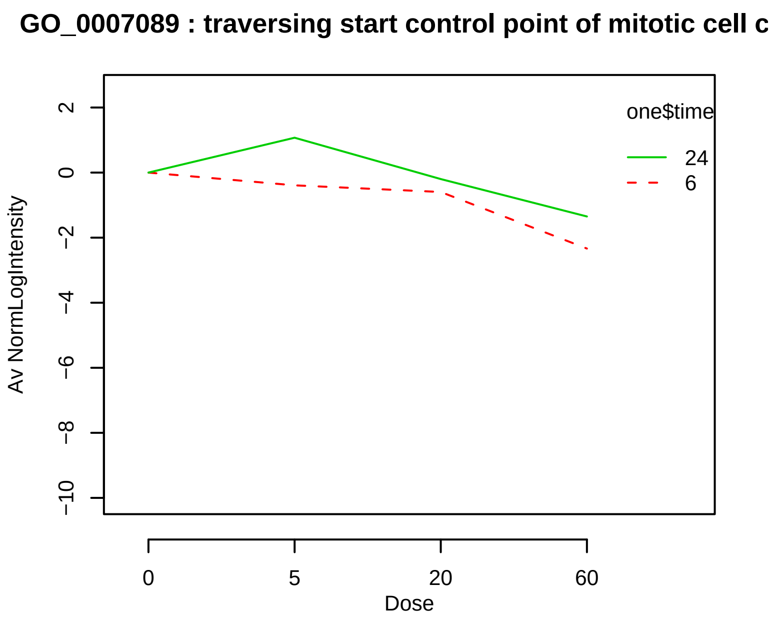
GO_0007089 : traversing start control point of mitotic cell c
Av NormLogIntensity
Dose
one$time
24
6

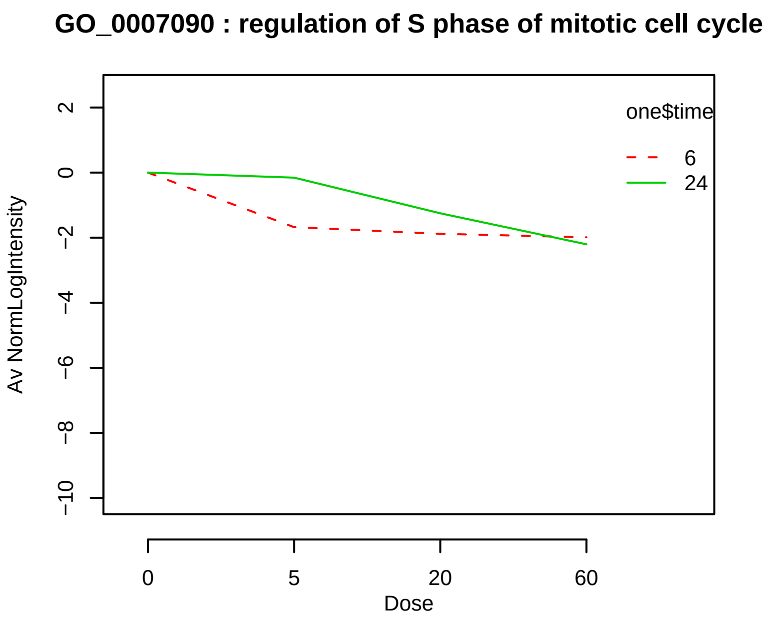
GO_0007090 : regulation of S phase of mitotic cell cycle
Av NormLogIntensity
Dose
one$time
6
24

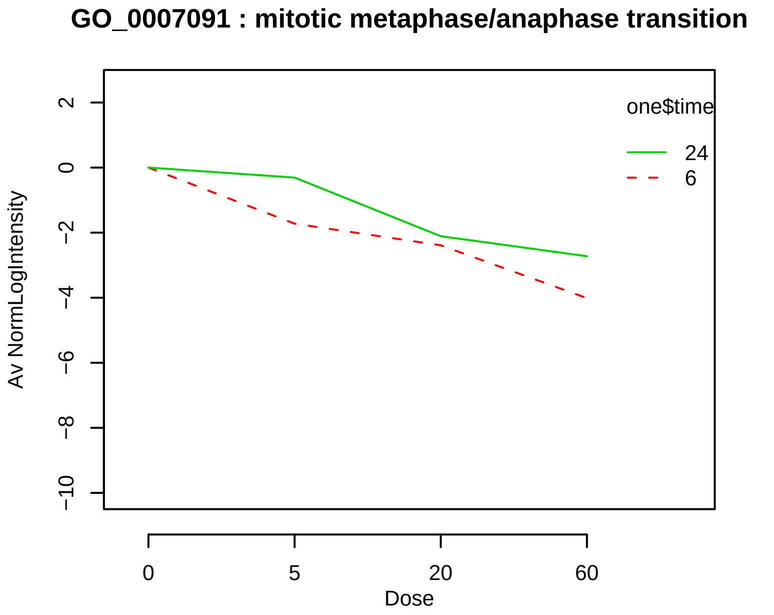
GO_0007091 : mitotic metaphase/anaphase transition
Av NormLogIntensity
Dose
one$time
24
6

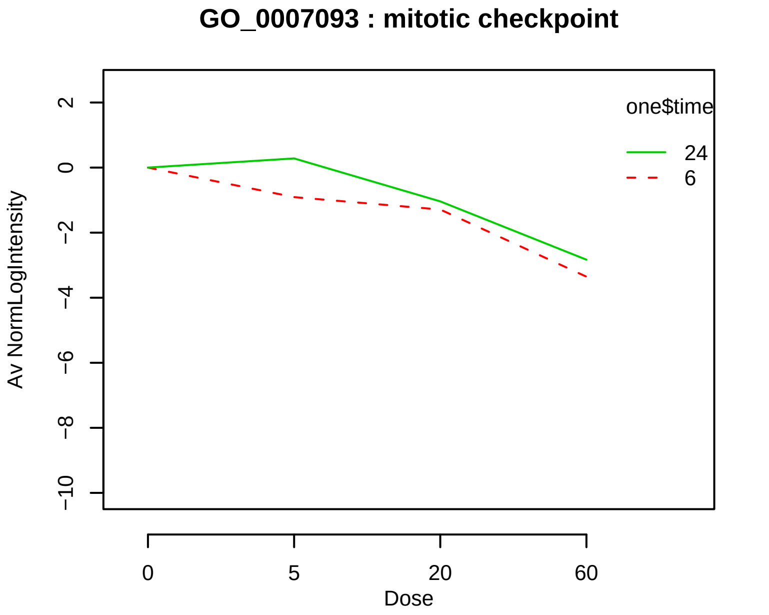
GO_0007093 : mitotic checkpoint
Av NormLogIntensity
Dose
one$time
24
6

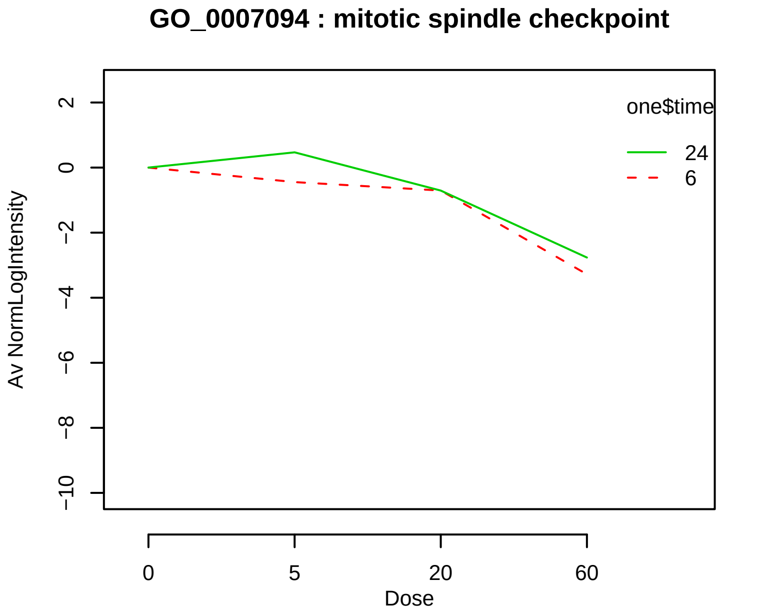
GO_0007094 : mitotic spindle checkpoint
Av NormLogIntensity
Dose
one$time
24
6

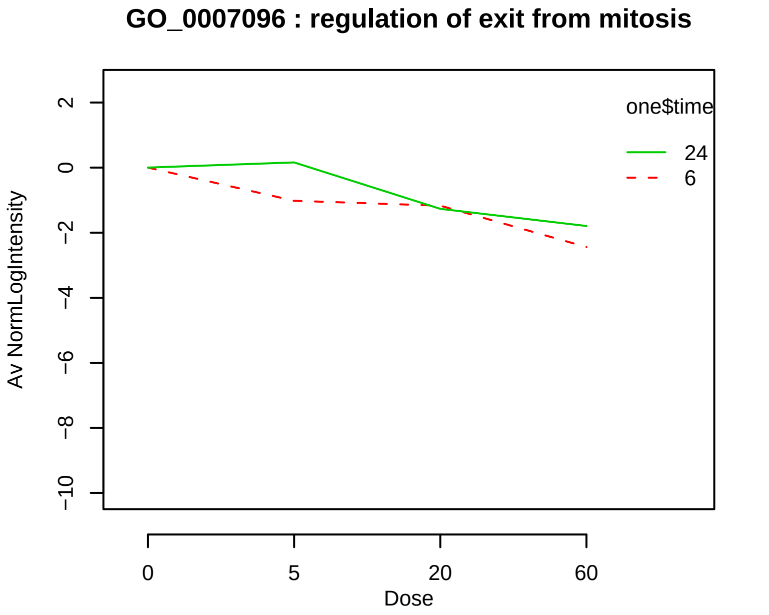
GO_0007096 : regulation of exit from mitosis
Av NormLogIntensity
Dose
one$time
24
6

GO_0007098 : centrosome cycle

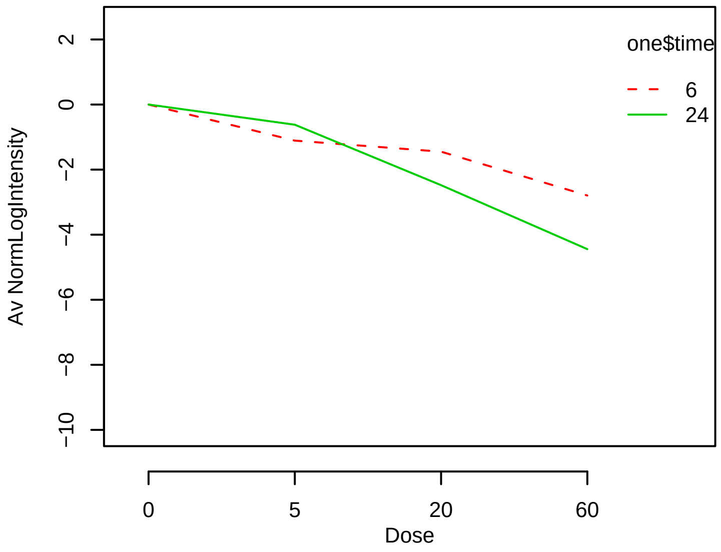

GO_0007126 : meiosis

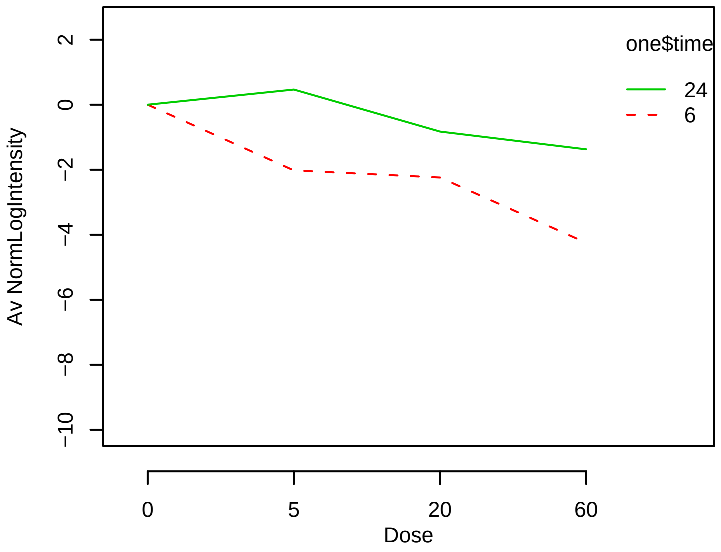

GO_0007127 : meiosis I

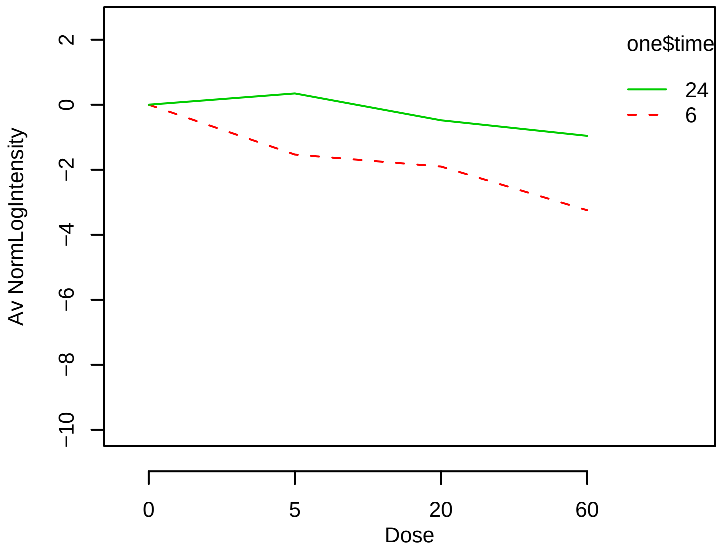

GO_0007128 : meiotic prophase I

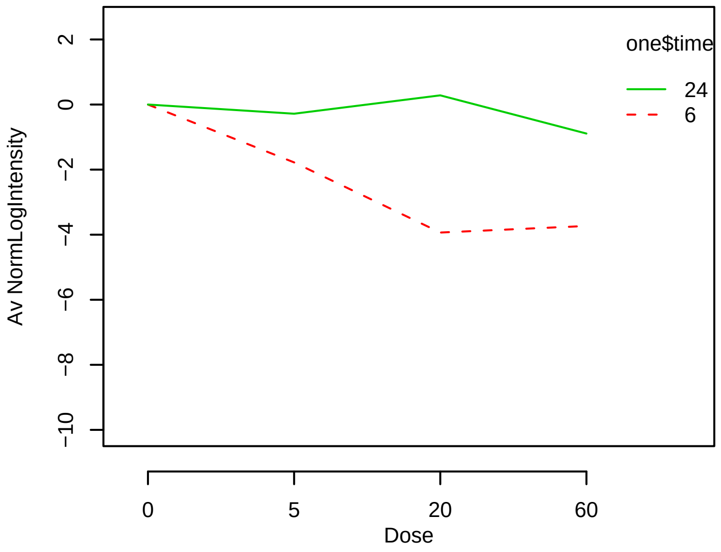

GO_0007129 : synapsis

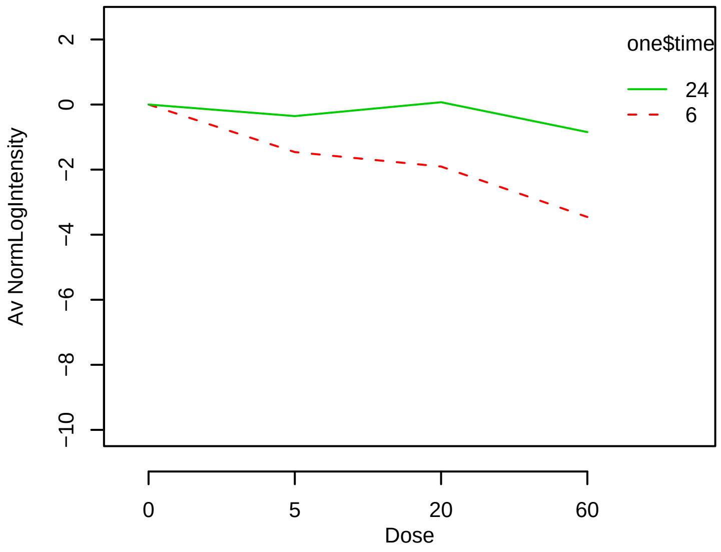

GO_0007130 : synaptonemal complex formation

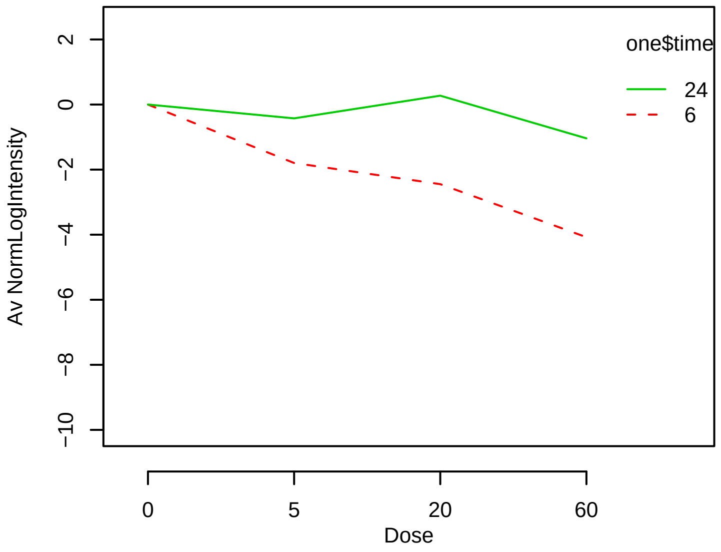

GO_0007131 : meiotic recombination

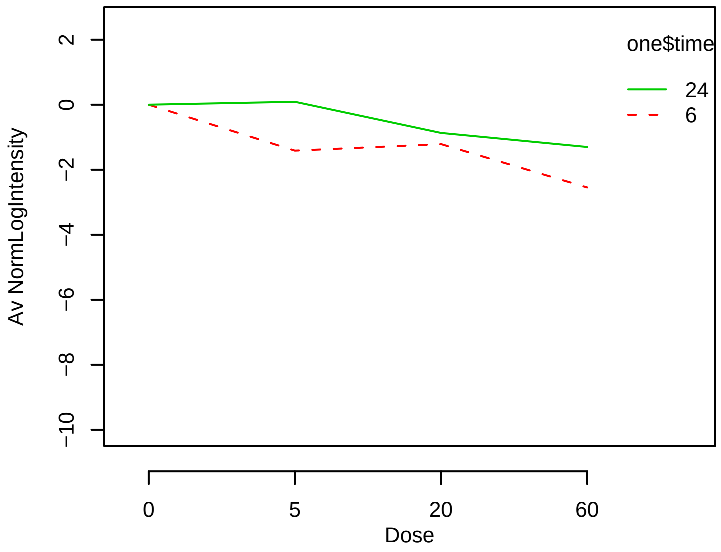

GO_0007140 : male meiosis

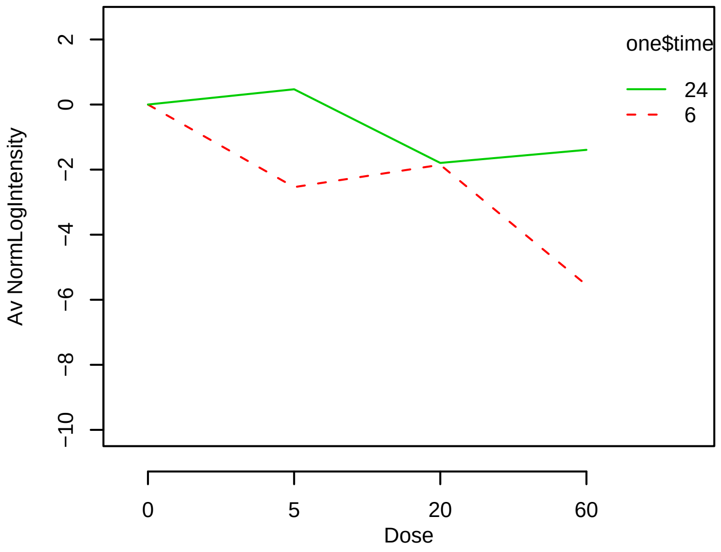

GO_0007156 : homophilic cell adhesion

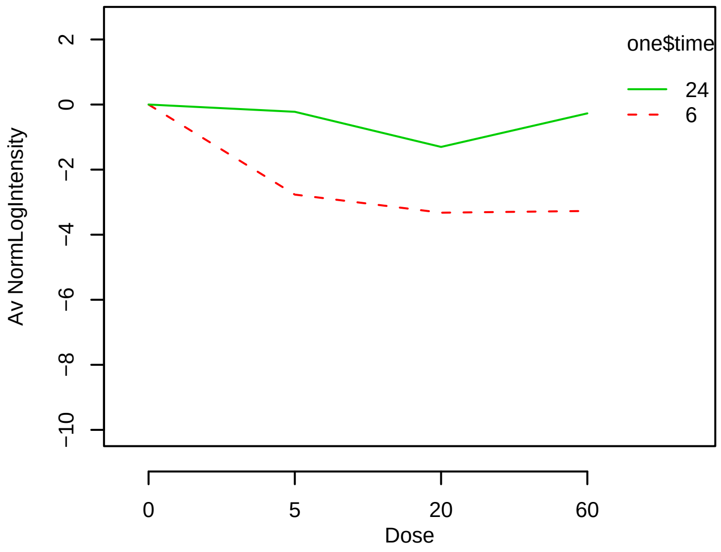

GO_0007157 : heterophilic cell adhesion

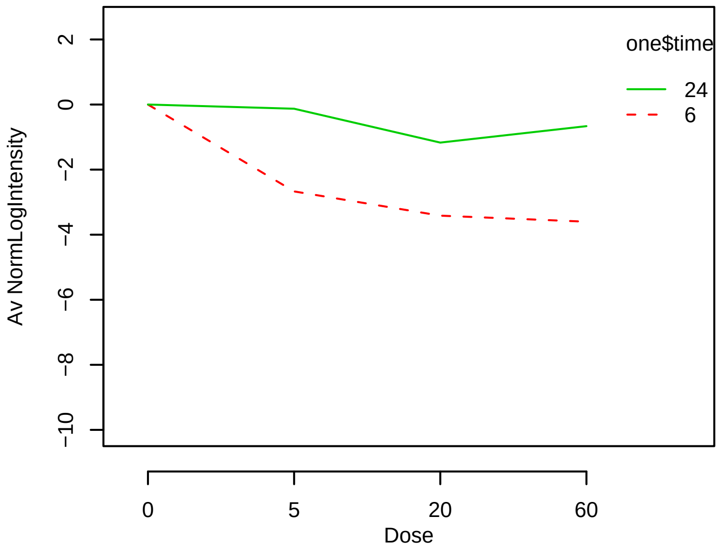

GO_0007158 : neuron adhesion

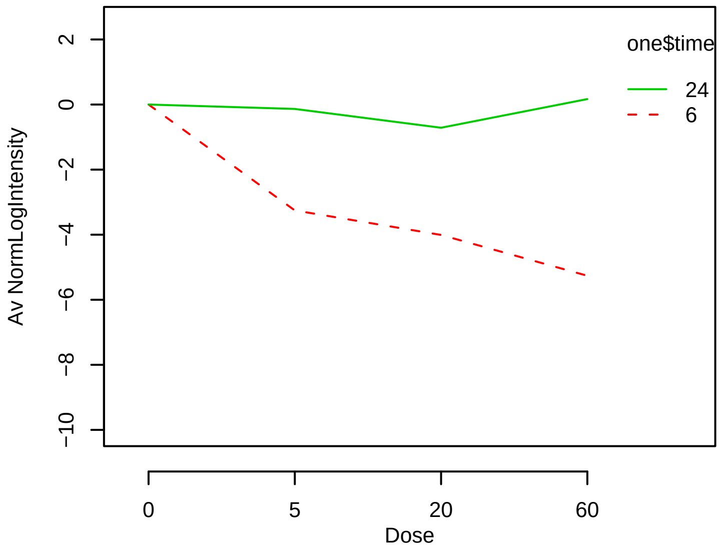

GO_0007159 : leukocyte adhesion

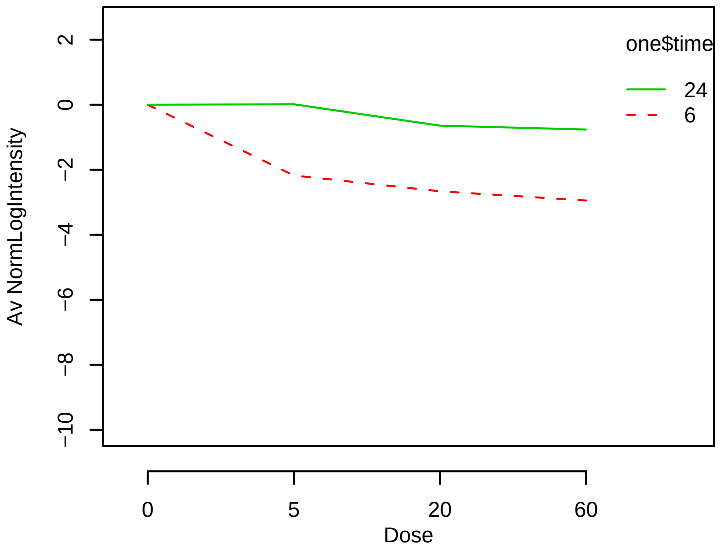

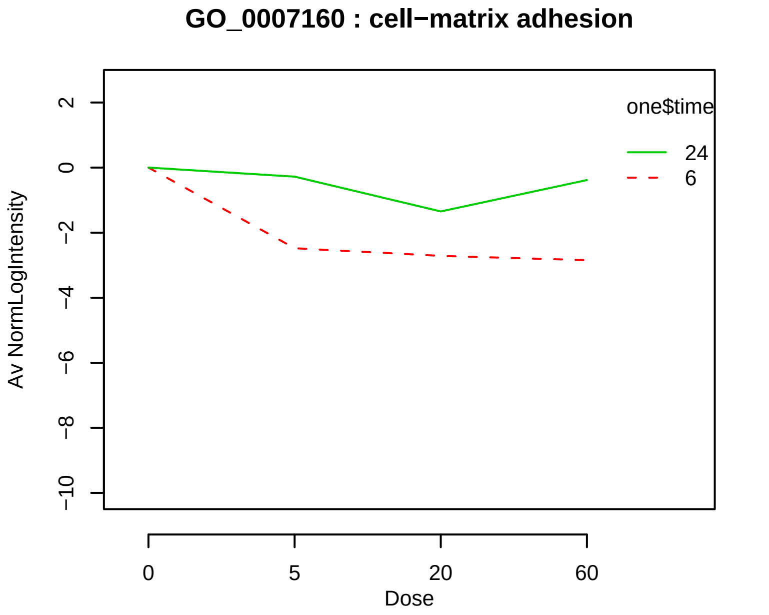
GO_0007160 : cell−matrix adhesion
one$time
24
6
Av NormLogIntensity
Dose
0
5
20
60

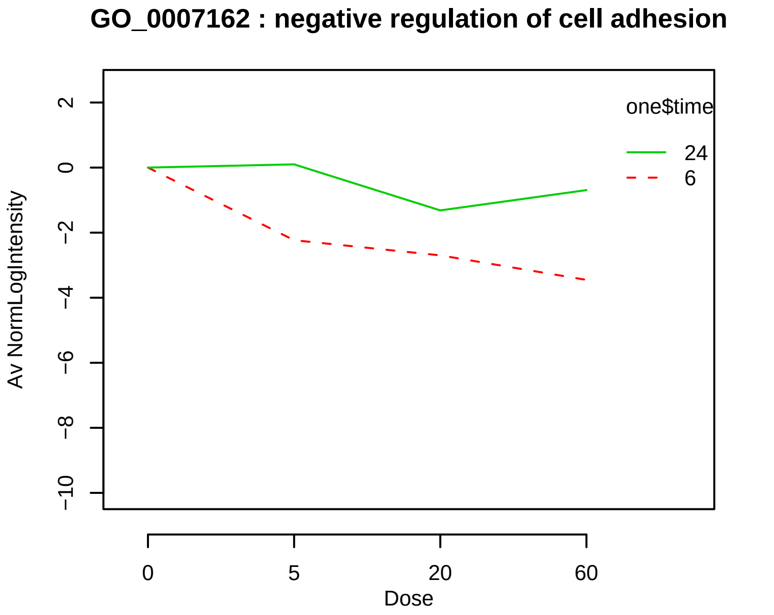
GO_0007162 : negative regulation of cell adhesion
one$time
24
6
Av NormLogIntensity
Dose
0
5
20
60

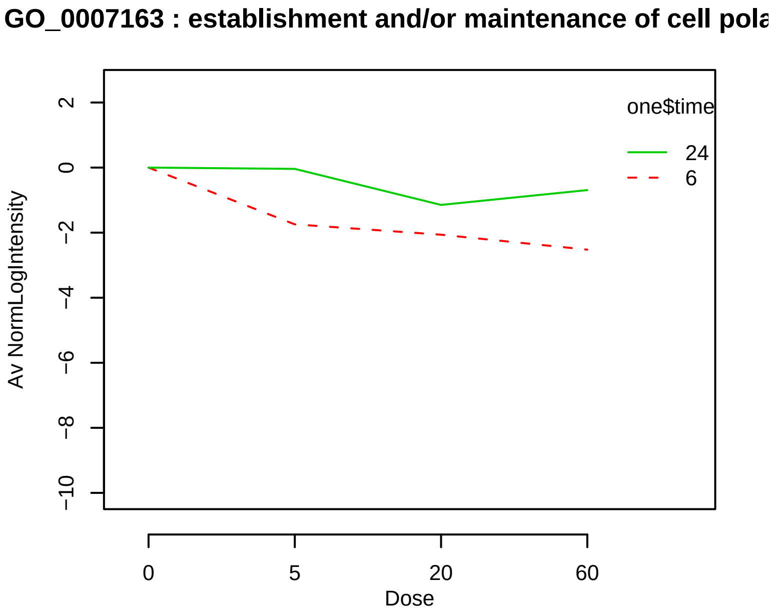
GO_0007163 : establishment and/or maintenance of cell pola
one$time
24
6
Av NormLogIntensity
Dose
0
5
20
60

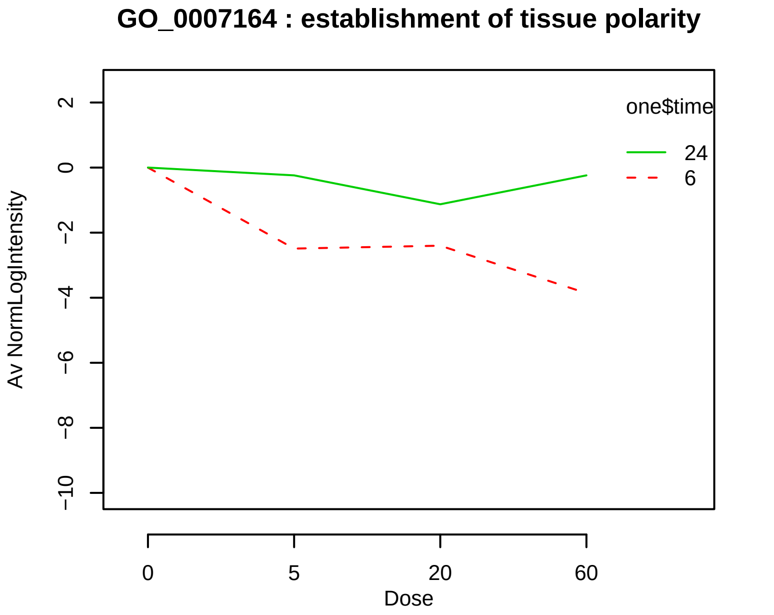
GO_0007164 : establishment of tissue polarity
one$time
24
6
Av NormLogIntensity
Dose
0
5
20
60

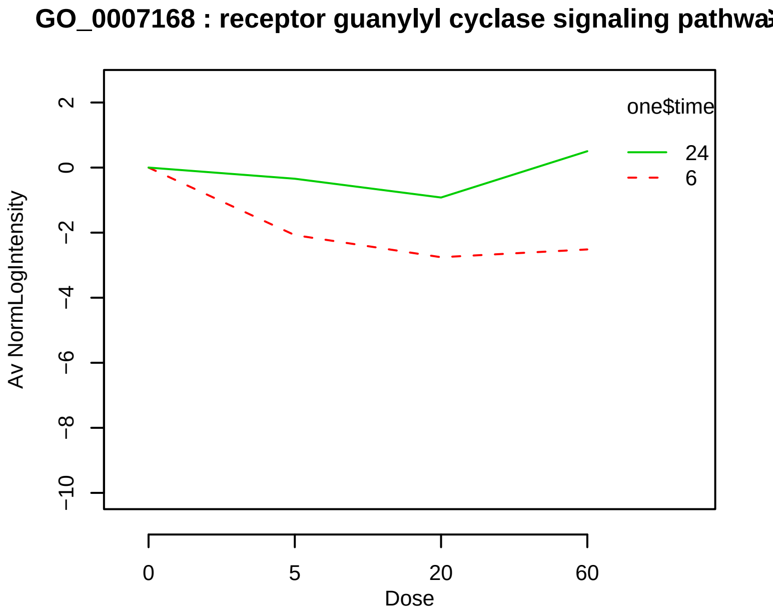
GO_0007168 : receptor guanylyl cyclase signaling pathwa
one$time
24
6
Av NormLogIntensity
Dose
0
5
20
60

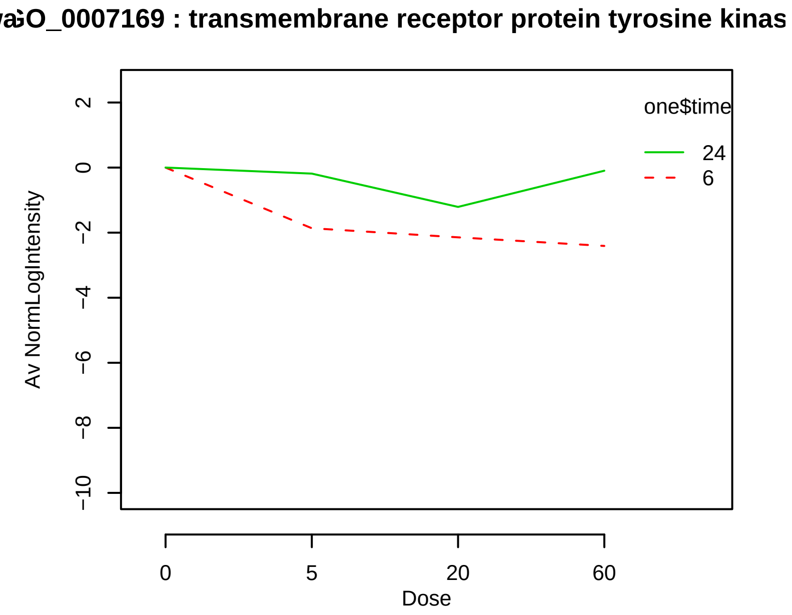
GO_0007169 : transmembrane receptor protein tyrosine kinas
one$time
24
6
Av NormLogIntensity
Dose
0
5
20
60

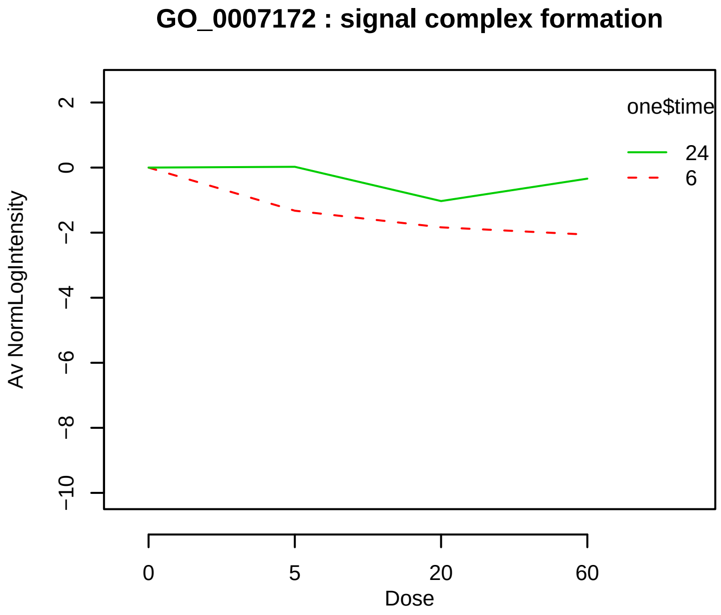
GO_0007172 : signal complex formation
one$time
24
6
Av NormLogIntensity
Dose

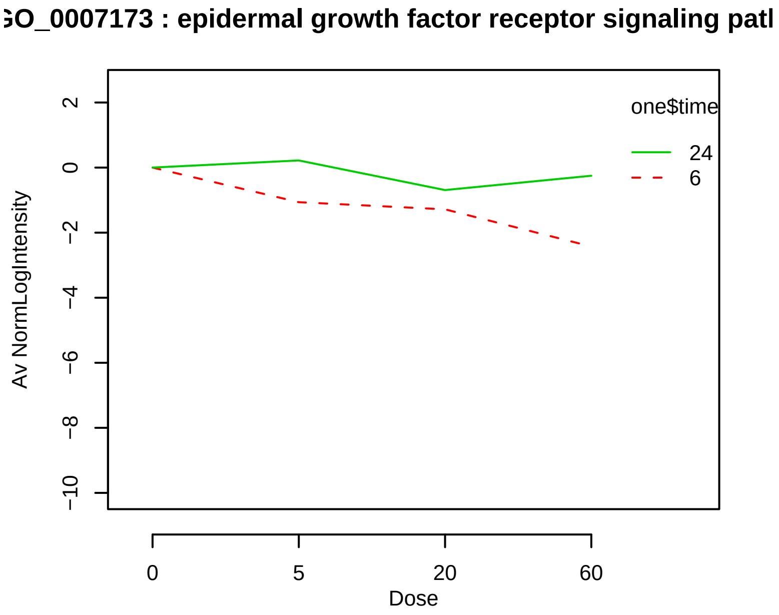
GO_0007173 : epidermal growth factor receptor signaling patl
one$time
24
6
Av NormLogIntensity
Dose

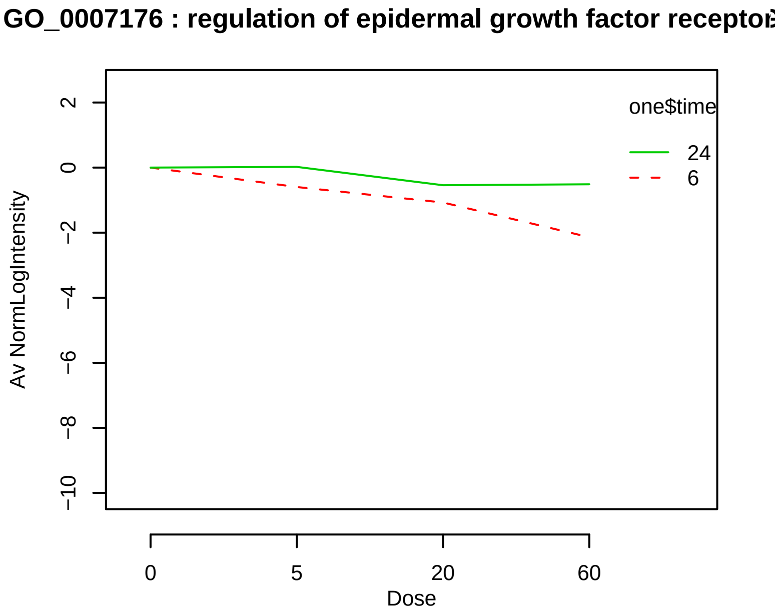
GO_0007176 : regulation of epidermal growth factor recepto
one$time
24
6
Av NormLogIntensity
Dose

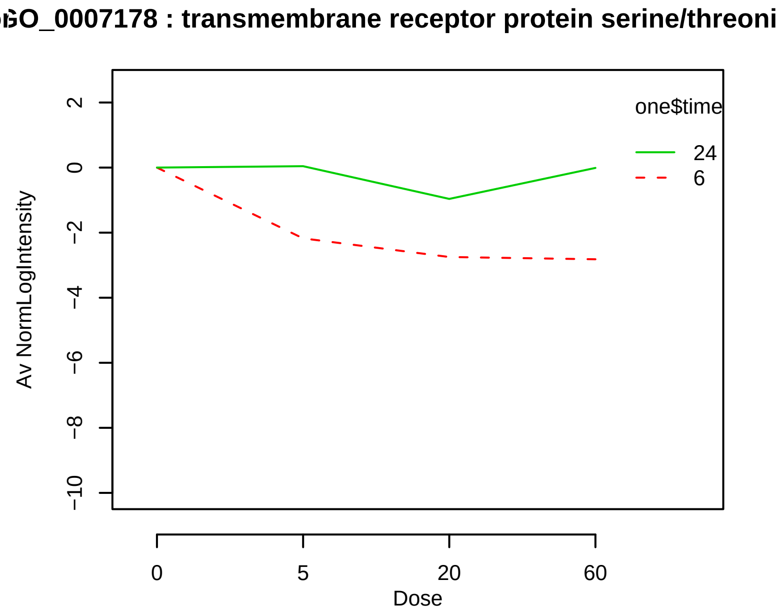
GO_0007178 : transmembrane receptor protein serine/threoni
one$time
24
6
Av NormLogIntensity
Dose

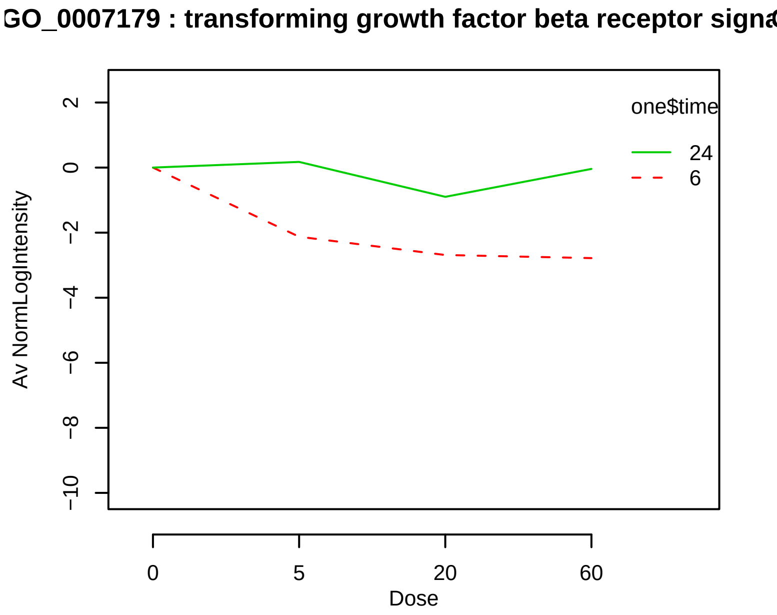
GO_0007179 : transforming growth factor beta receptor signa
one$time
24
6
Av NormLogIntensity
Dose

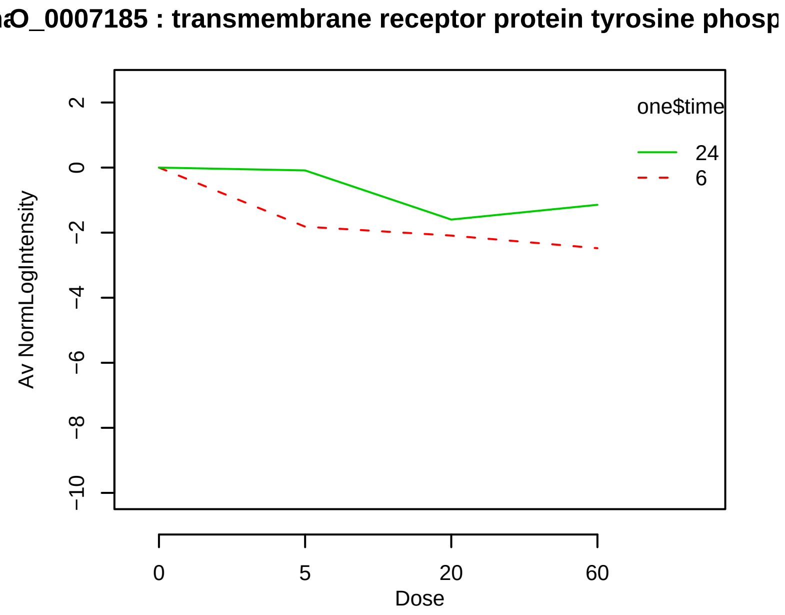
O_0007185 : transmembrane receptor protein tyrosine phosp
one$time
24
6
Av NormLogIntensity
Dose

GO_0007187 : G−protein signaling\, coupled to cyclic nucleo

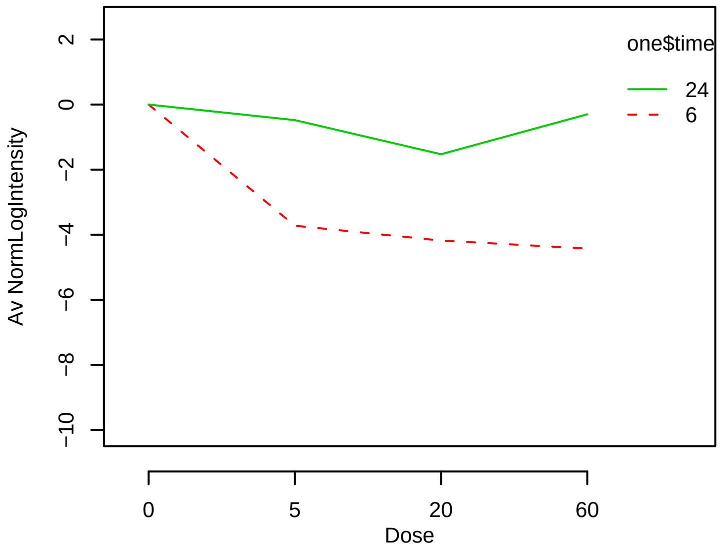

GO_0007188 : G−protein signaling\, coupled to cAMP nucleot

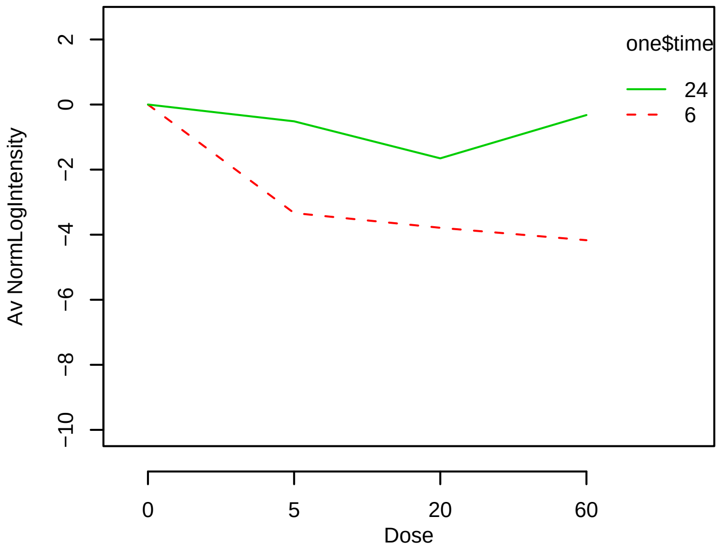

GO_0007189 : G−protein signaling\, adenylate cyclase activa

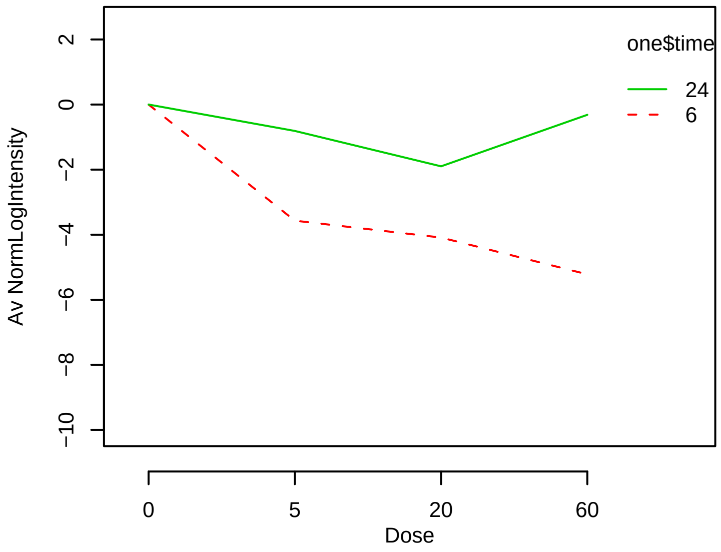

GO_0007190 : adenylate cyclase activation

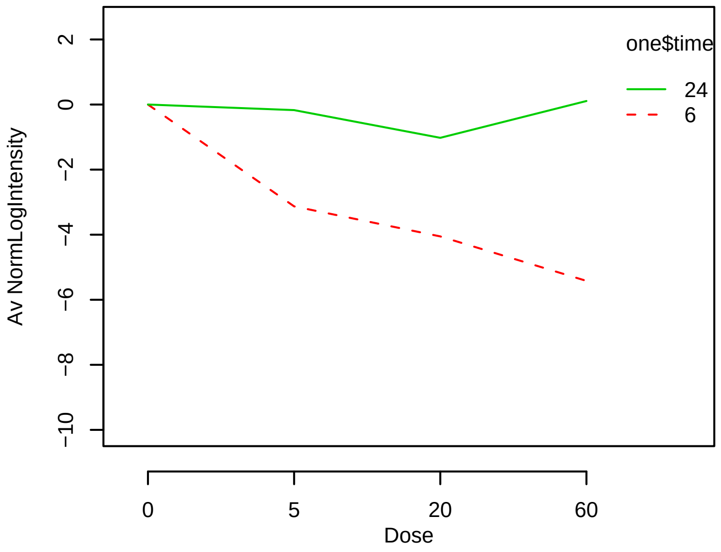

GO_0007193 : G−protein signaling\, adenylate cyclase inhibi

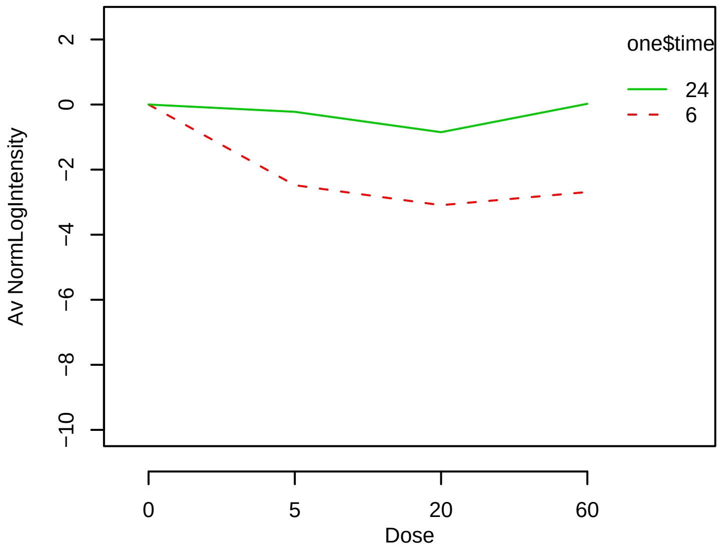

GO_0007194 : negative regulation of adenylate cyclase activ

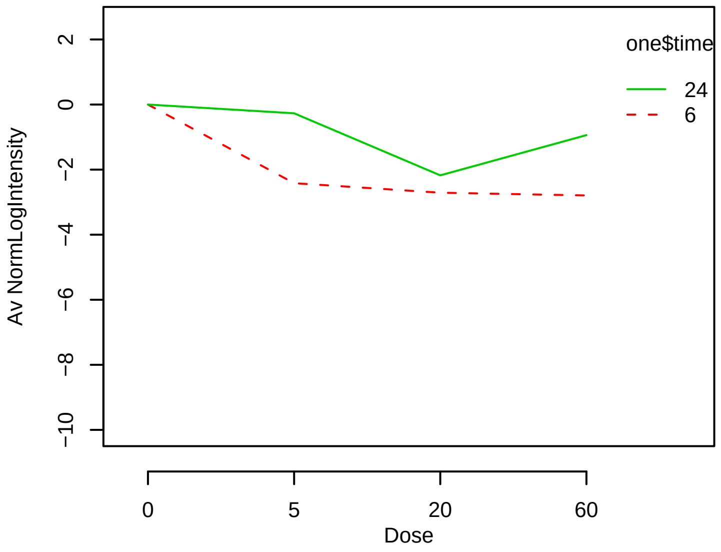

GO_0007200 : G−protein signaling\, coupled to IP3 second me

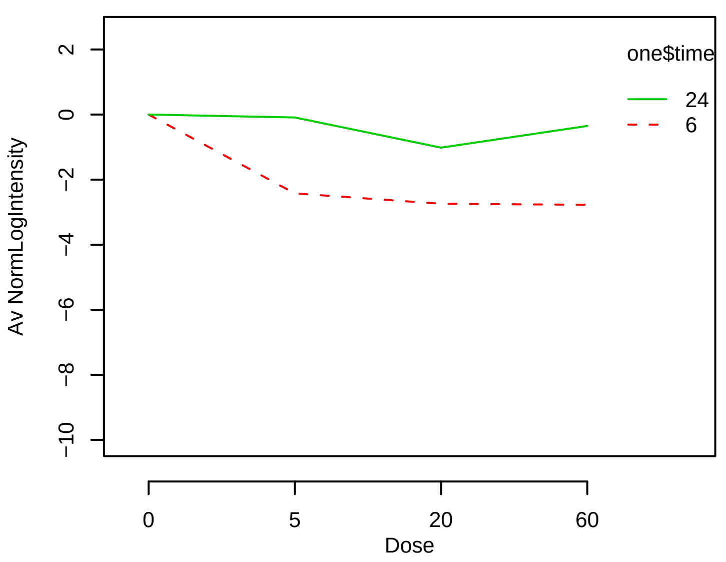

GO_0007202 : phospholipase C activation

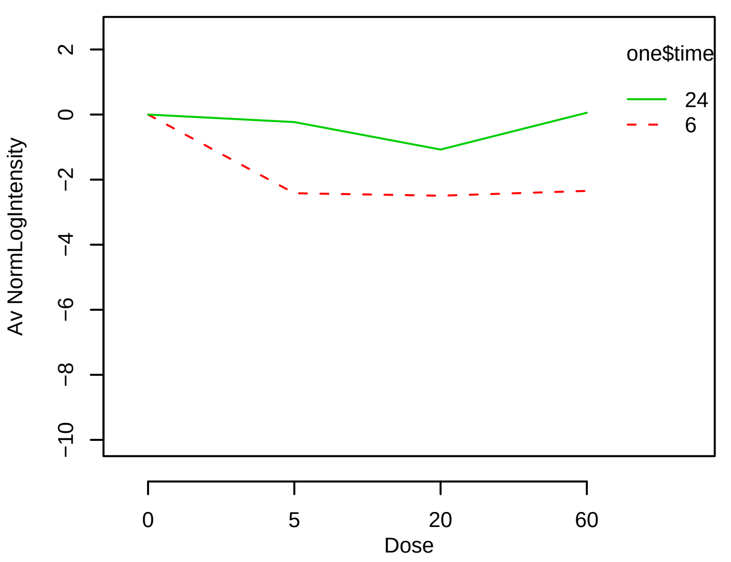

GO_0007204 : elevation of cytosolic calcium ion concentrati

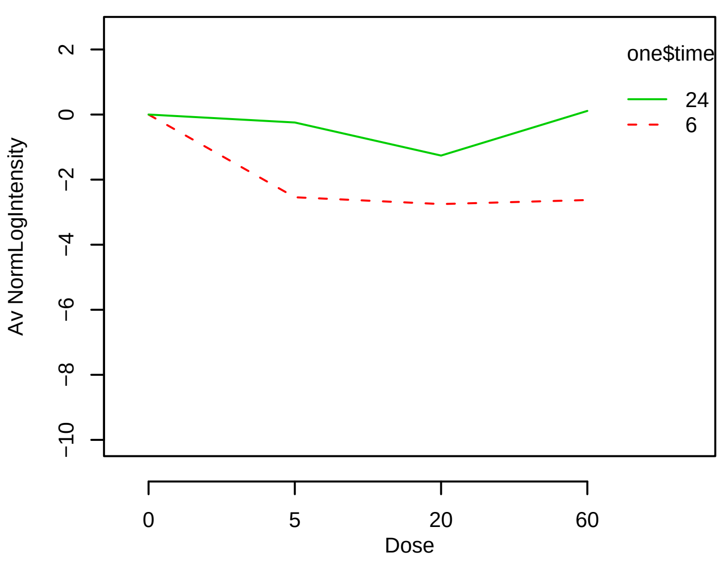

GO_0007205 : protein kinase C activation

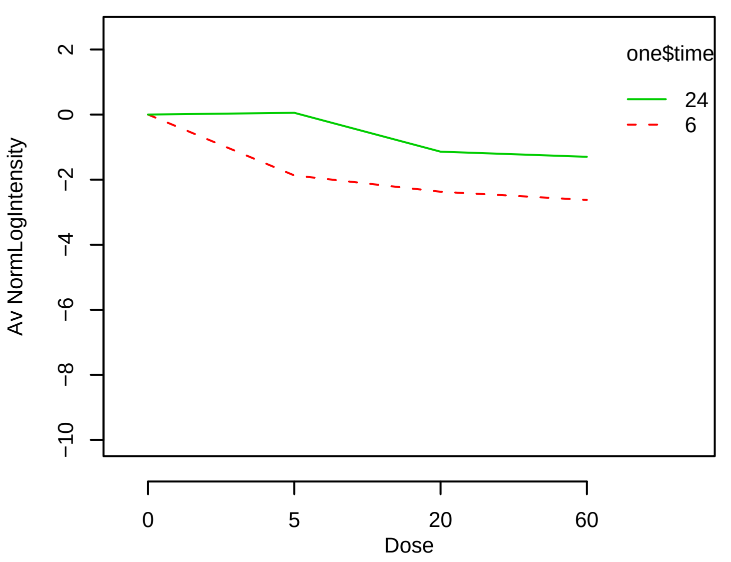

GO_0007212 : dopamine receptor signaling pathway

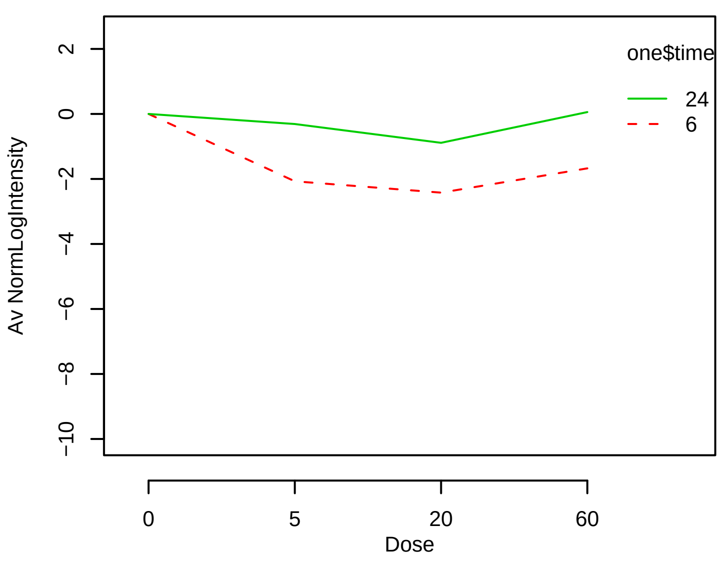

GO_0007213 : acetylcholine receptor signaling\, muscarinic p

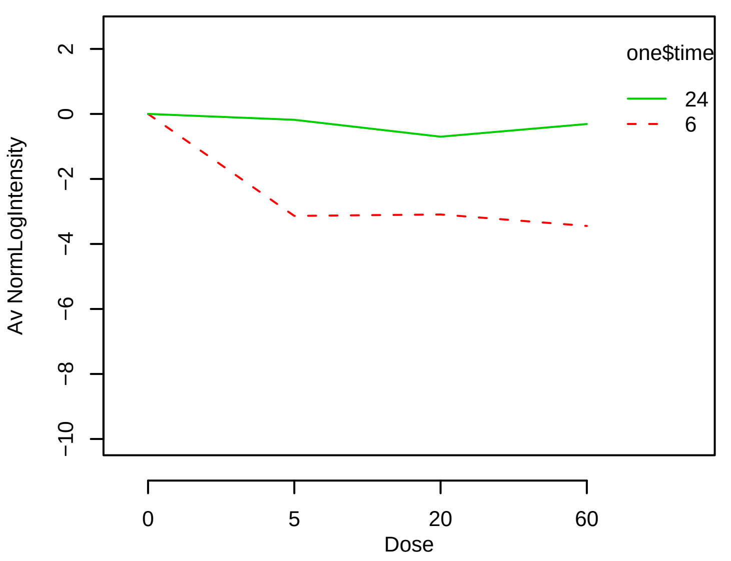

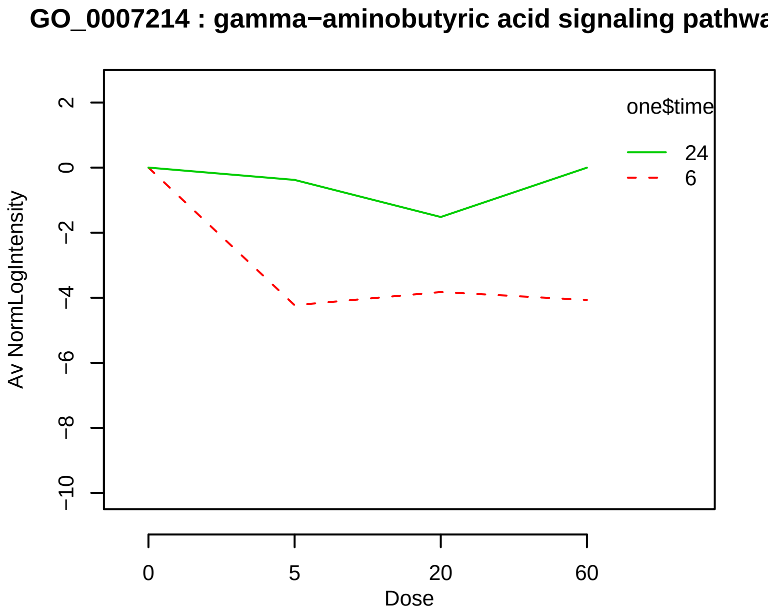
GO_0007214 : gamma−aminobutyric acid signaling pathwa
Av NormLogIntensity
Dose
one$time
24
6

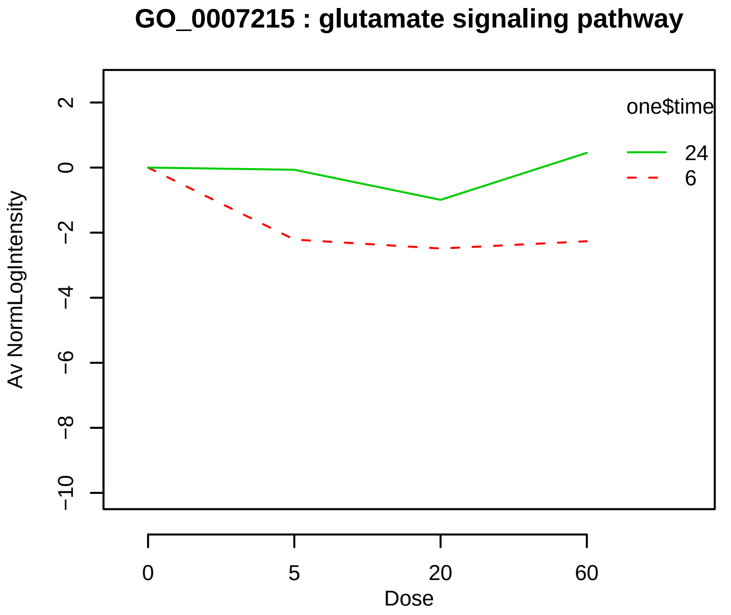
GO_0007215 : glutamate signaling pathway
Av NormLogIntensity
Dose
one$time
24
6

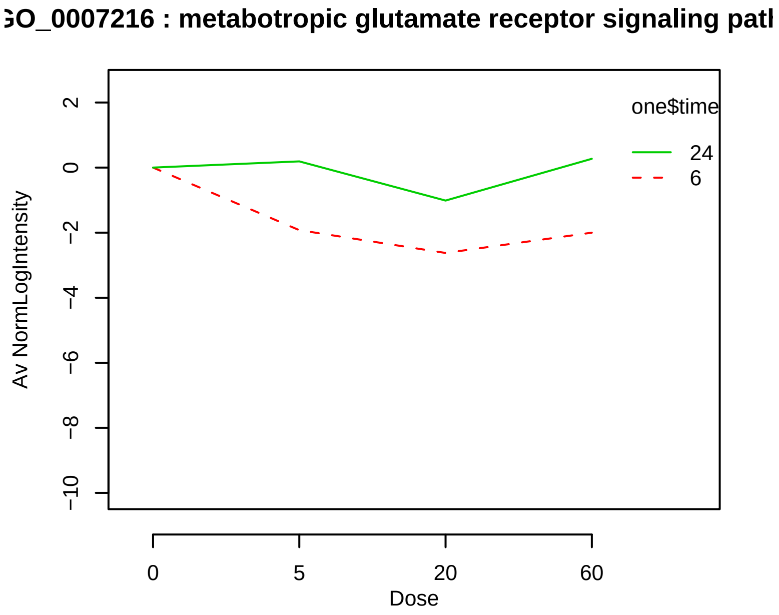
GO_0007216 : metabotropic glutamate receptor signaling patl
Av NormLogIntensity
Dose
one$time
24
6

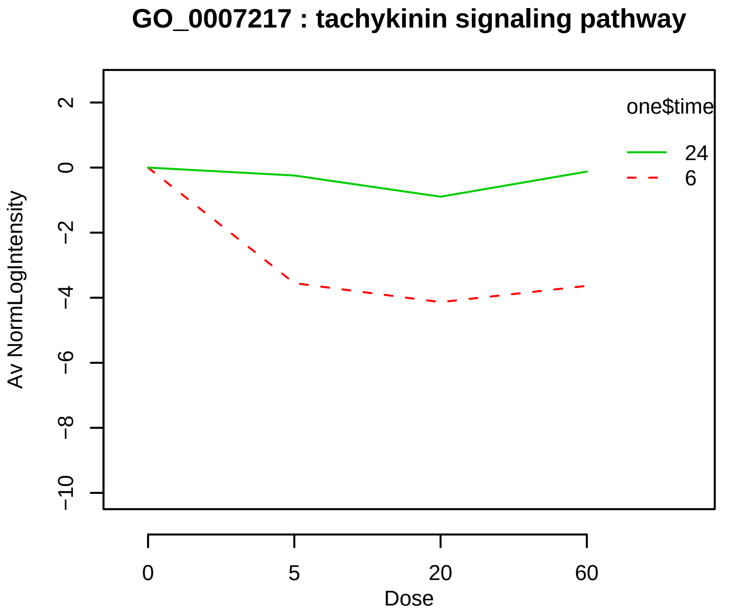
GO_0007217 : tachykinin signaling pathway
Av NormLogIntensity
Dose
one$time
24
6

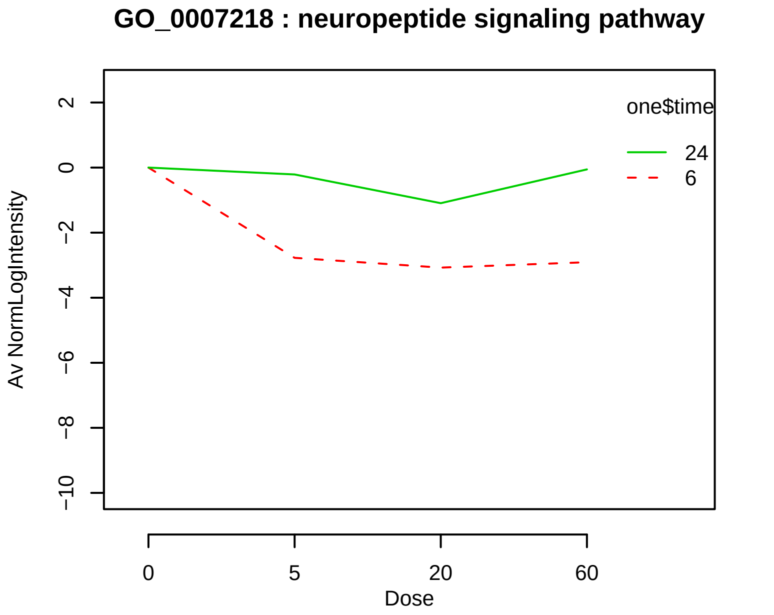
GO_0007218 : neuropeptide signaling pathway
Av NormLogIntensity
Dose
one$time
24
6

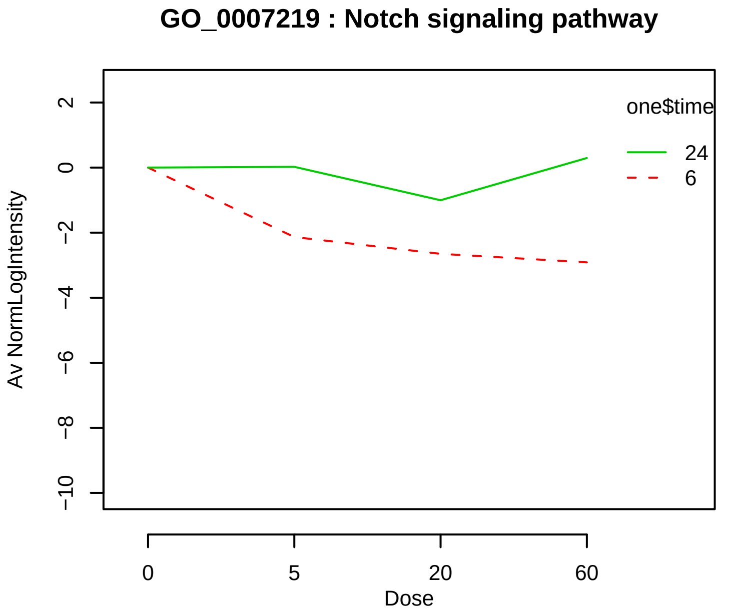
GO_0007219 : Notch signaling pathway
Av NormLogIntensity
Dose
one$time
24
6

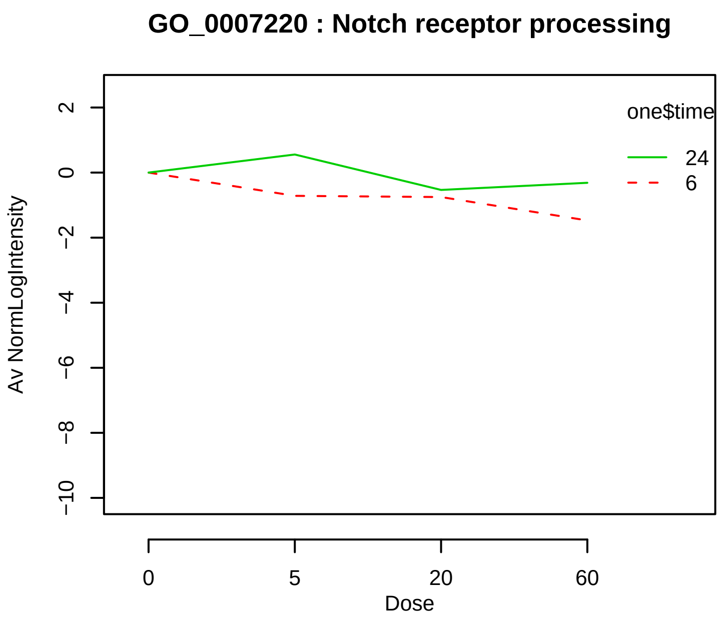
GO_0007220 : Notch receptor processing
one$time
24
6
Av NormLogIntensity
2
0
−2
−4
−6
−8
−10
0
5
20
60
Dose

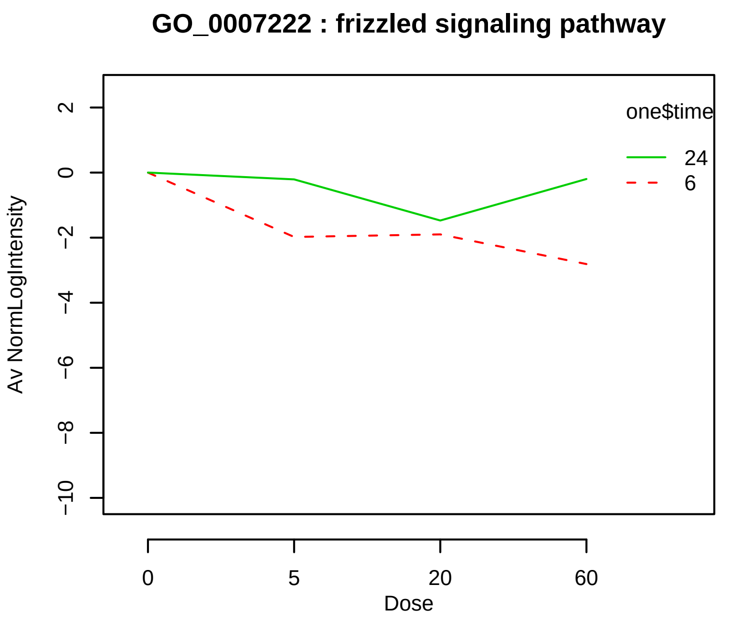
GO_0007222 : frizzled signaling pathway
one$time
24
6
Av NormLogIntensity
2
0
−2
−4
−6
−8
−10
0
5
20
60
Dose

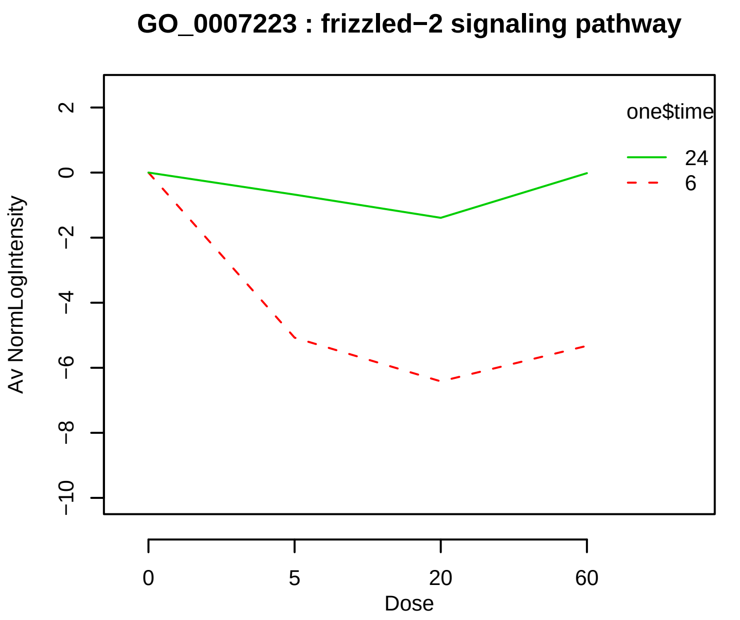
GO_0007223 : frizzled−2 signaling pathway
one$time
24
6
Av NormLogIntensity
2
0
−2
−4
−6
−8
−10
0
5
20
60
Dose

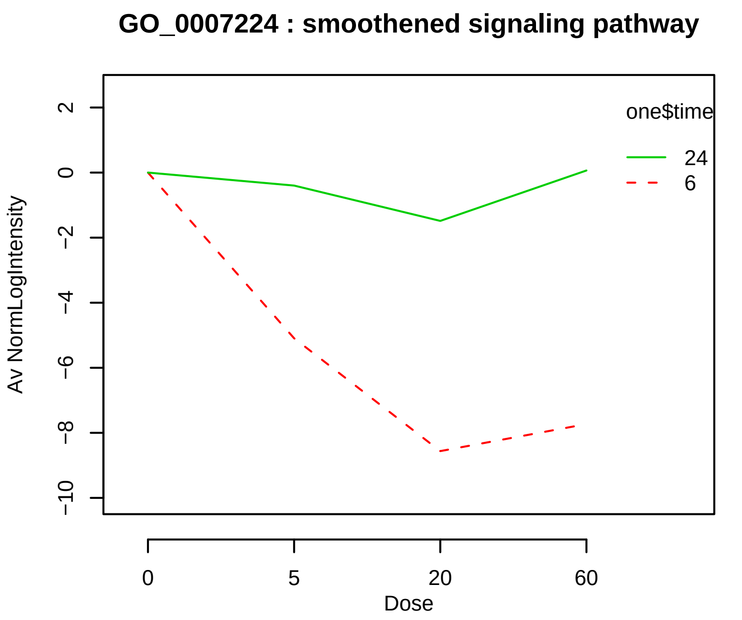
GO_0007224 : smoothened signaling pathway
one$time
24
6
Av NormLogIntensity
2
0
−2
−4
−6
−8
−10
0
5
20
60
Dose

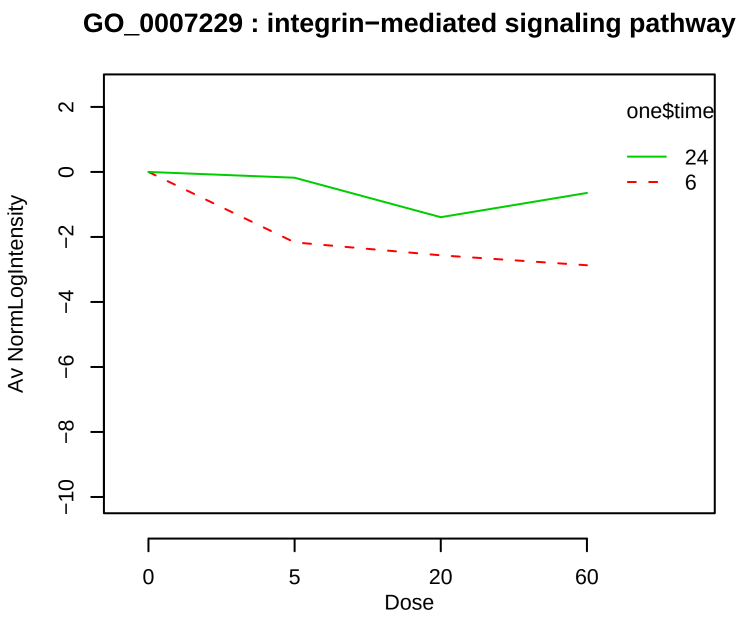
GO_0007229 : integrin−mediated signaling pathway
one$time
24
6
Av NormLogIntensity
2
0
−2
−4
−6
−8
−10
0
5
20
60
Dose

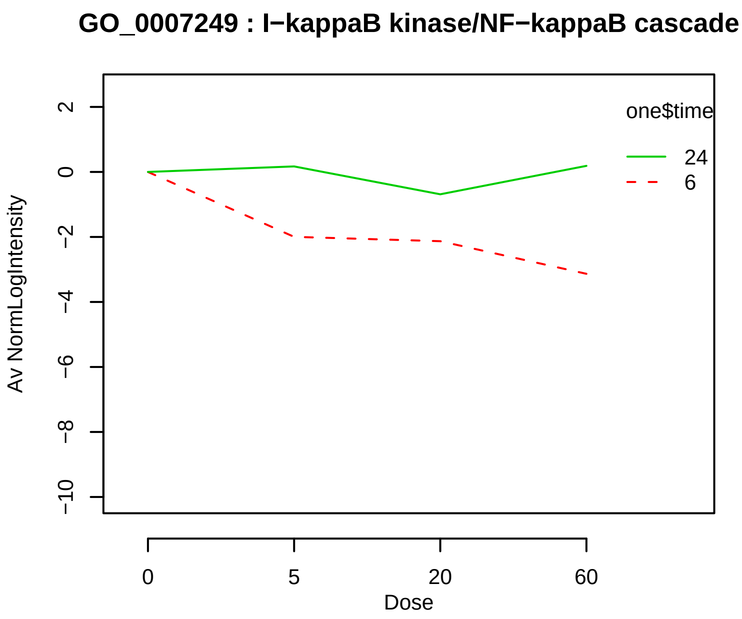
GO_0007249 : I−kappaB kinase/NF−kappaB cascade
one$time
24
6
Av NormLogIntensity
2
0
−2
−4
−6
−8
−10
0
5
20
60
Dose

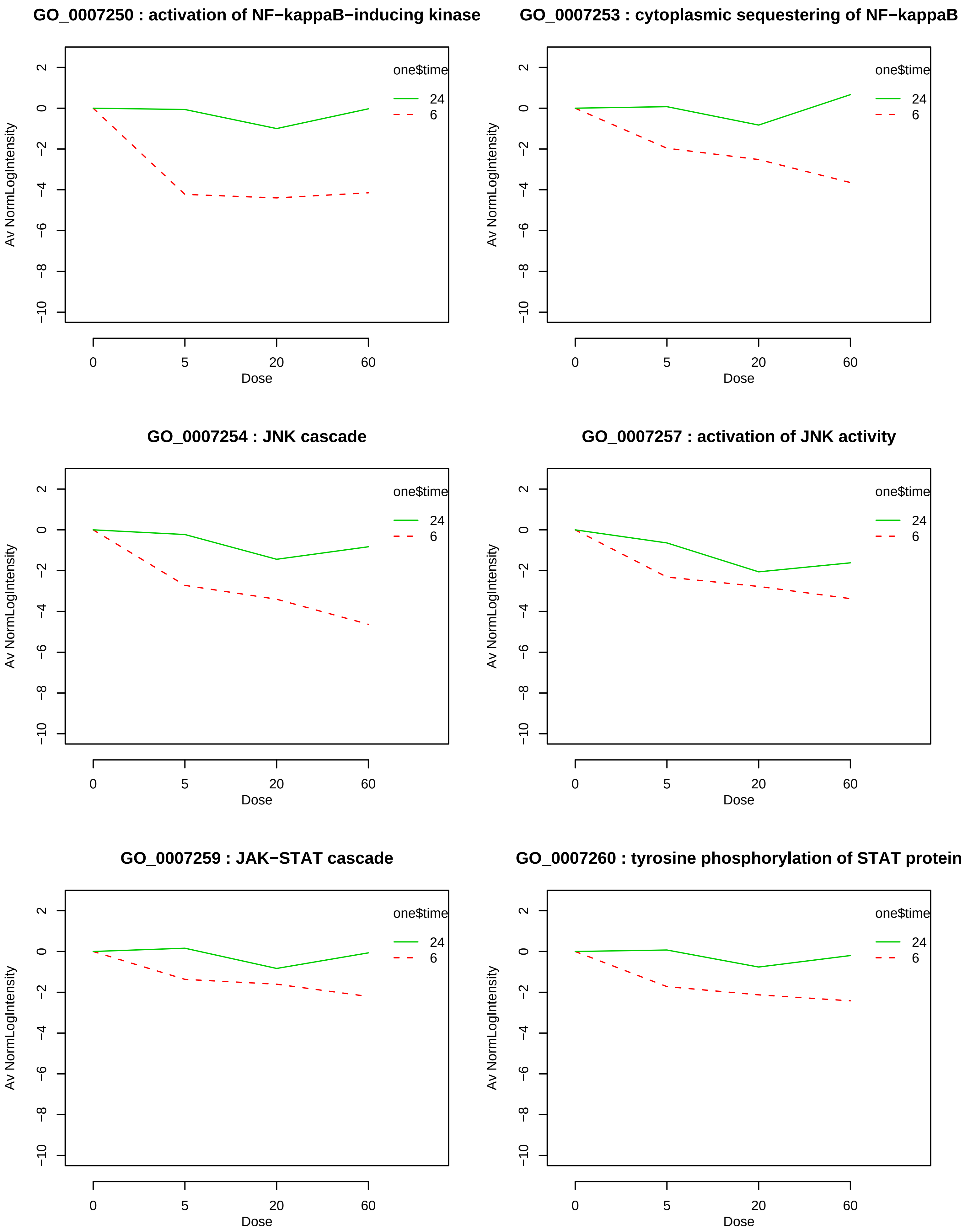
GO_0007250 : activation of NF−kappaB−inducing kinase
GO_0007253 : cytoplasmic sequestering of NF−kappaB
GO_0007254 : JNK cascade
GO_0007257 : activation of JNK activity
GO_0007259 : JAK−STAT cascade
GO_0007260 : tyrosine phosphorylation of STAT protein
Av NormLogIntensity
Dose
one$time
24
6
0
5
20
60
2
0
−2
−4
−6
−8
−10

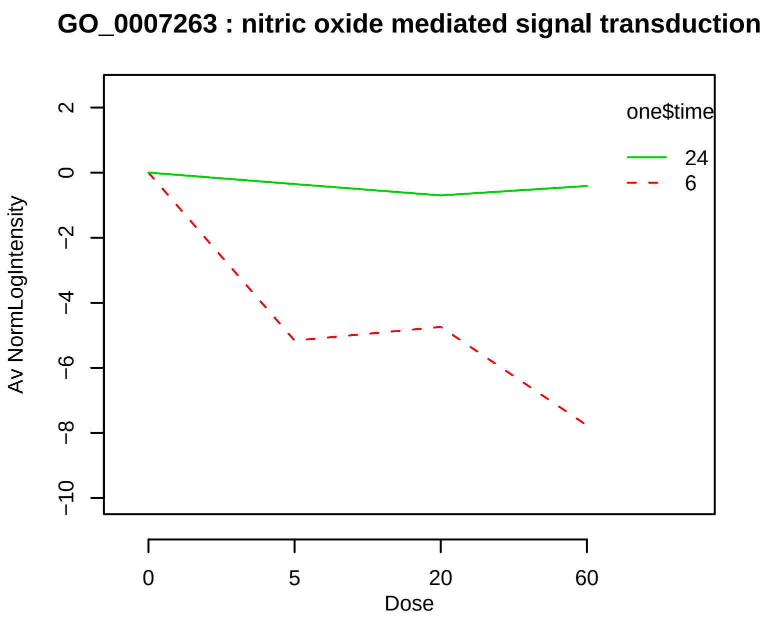
GO_0007263 : nitric oxide mediated signal transduction
Av NormLogIntensity
2
0
−2
−4
−6
−8
−10
one$time
24
6
0
5
20
60
Dose

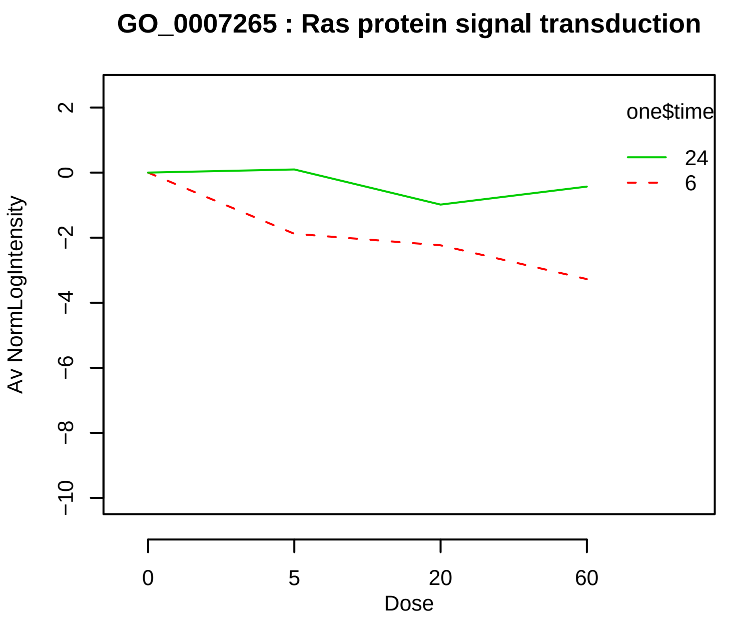
GO_0007265 : Ras protein signal transduction
Av NormLogIntensity
2
0
−2
−4
−6
−8
−10
one$time
24
6
0
5
20
60
Dose

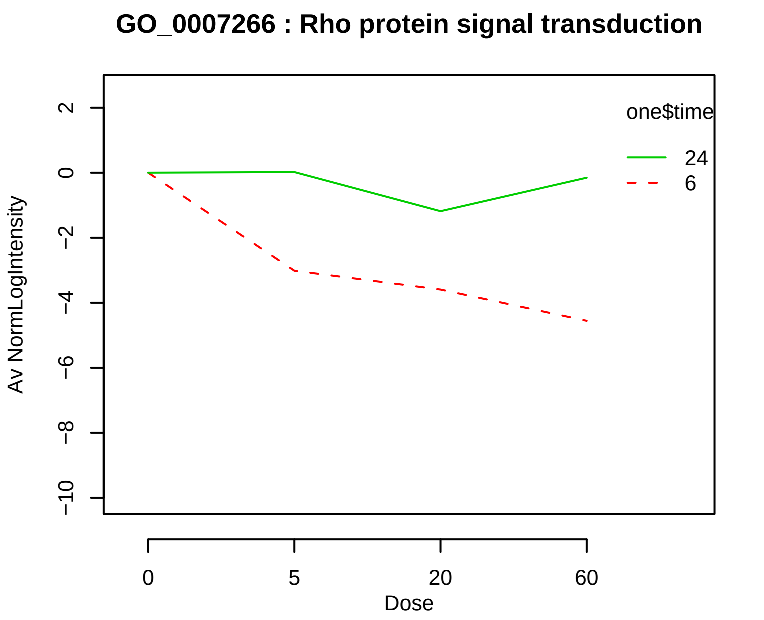
GO_0007266 : Rho protein signal transduction
Av NormLogIntensity
2
0
−2
−4
−6
−8
−10
one$time
24
6
0
5
20
60
Dose

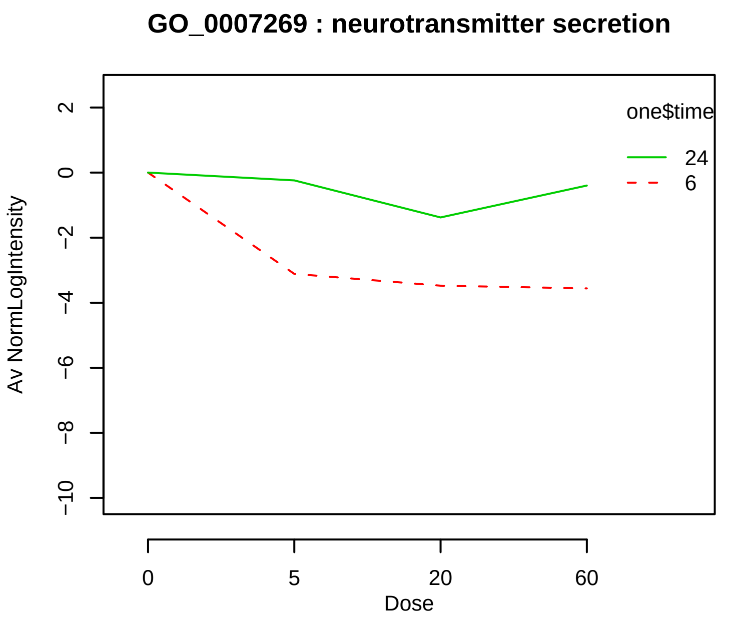
GO_0007269 : neurotransmitter secretion
Av NormLogIntensity
2
0
−2
−4
−6
−8
−10
one$time
24
6
0
5
20
60
Dose

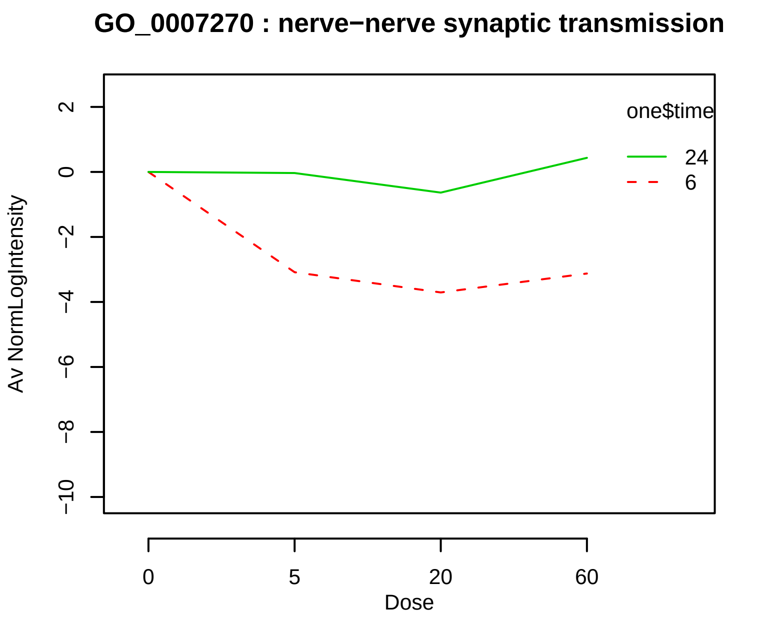
GO_0007270 : nerve−nerve synaptic transmission
Av NormLogIntensity
2
0
−2
−4
−6
−8
−10
one$time
24
6
0
5
20
60
Dose

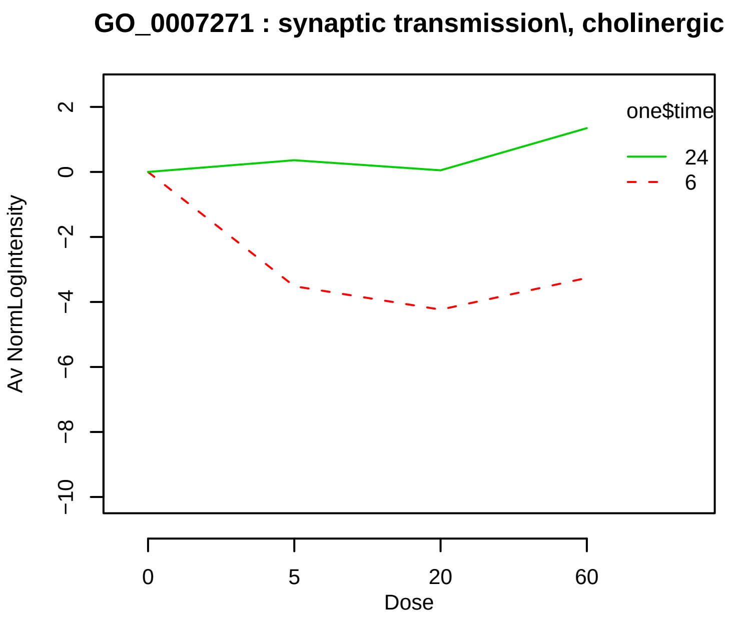
GO_0007271 : synaptic transmission\, cholinergic
Av NormLogIntensity
2
0
−2
−4
−6
−8
−10
one$time
24
6
0
5
20
60
Dose

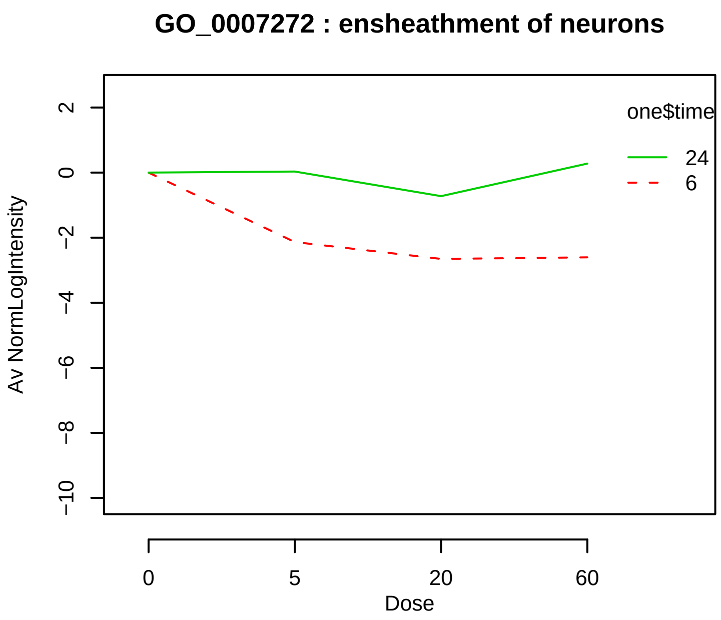
GO_0007272 : ensheathment of neurons
Av NormLogIntensity
Dose
one$time
24
6

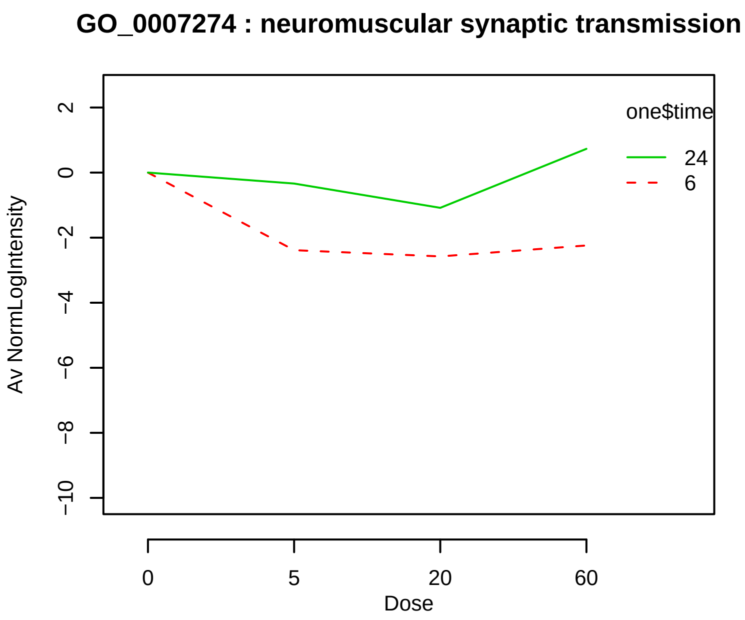
GO_0007274 : neuromuscular synaptic transmission
Av NormLogIntensity
Dose
one$time
24
6

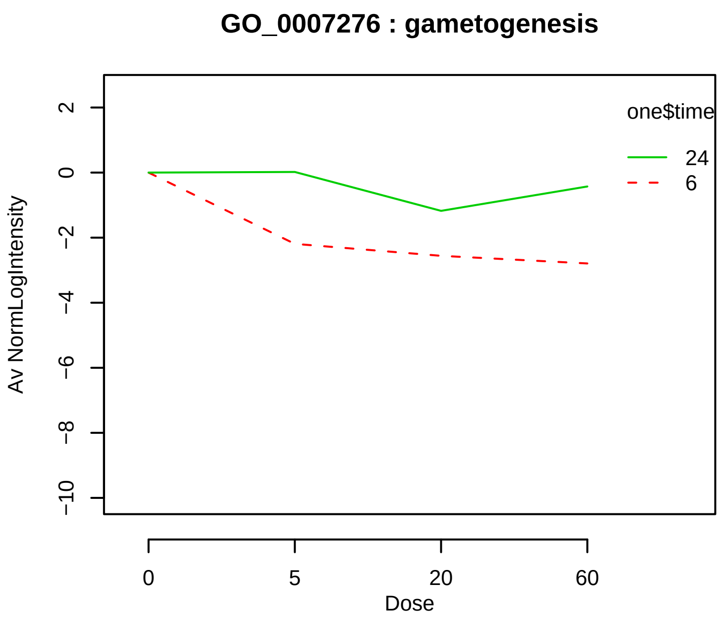
GO_0007276 : gametogenesis
Av NormLogIntensity
Dose
one$time
24
6

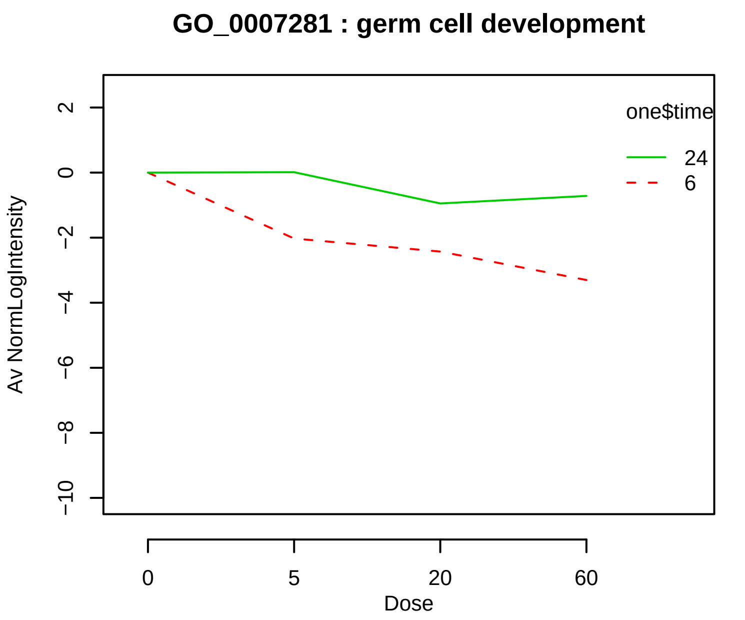
GO_0007281 : germ cell development
Av NormLogIntensity
Dose
one$time
24
6

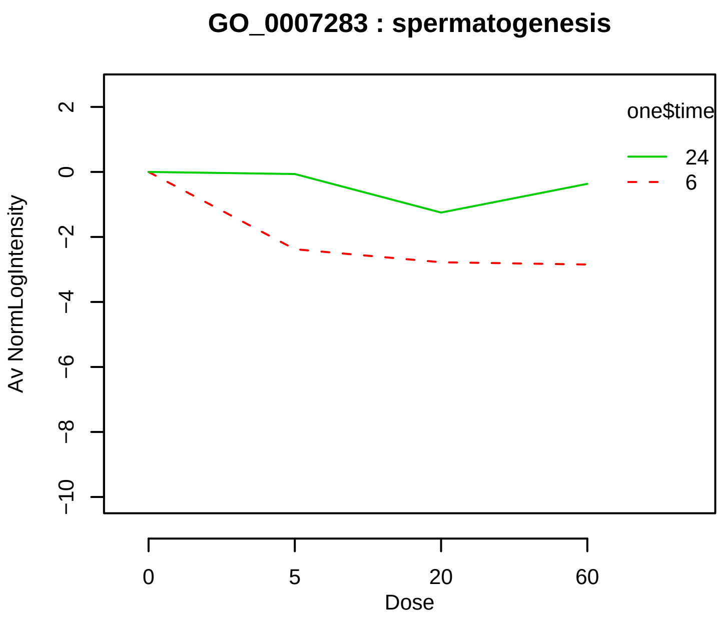
GO_0007283 : spermatogenesis
Av NormLogIntensity
Dose
one$time
24
6

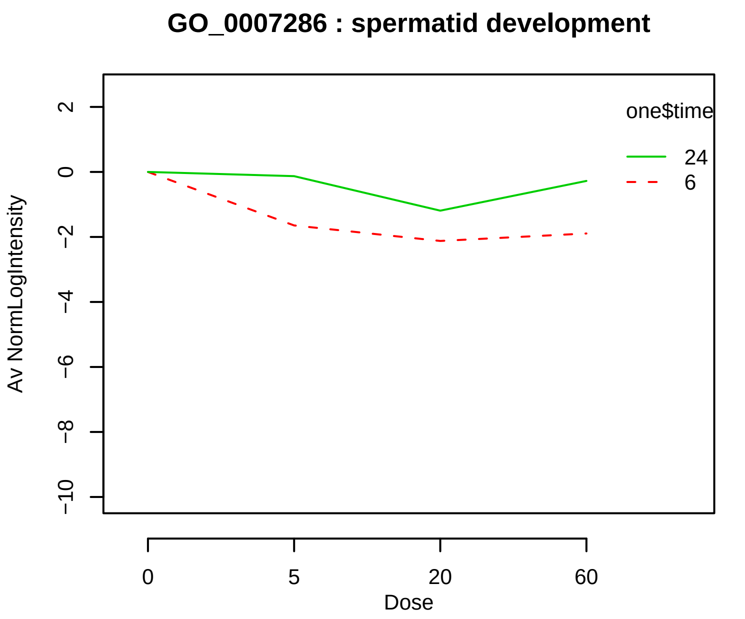
GO_0007286 : spermatid development
Av NormLogIntensity
Dose
one$time
24
6

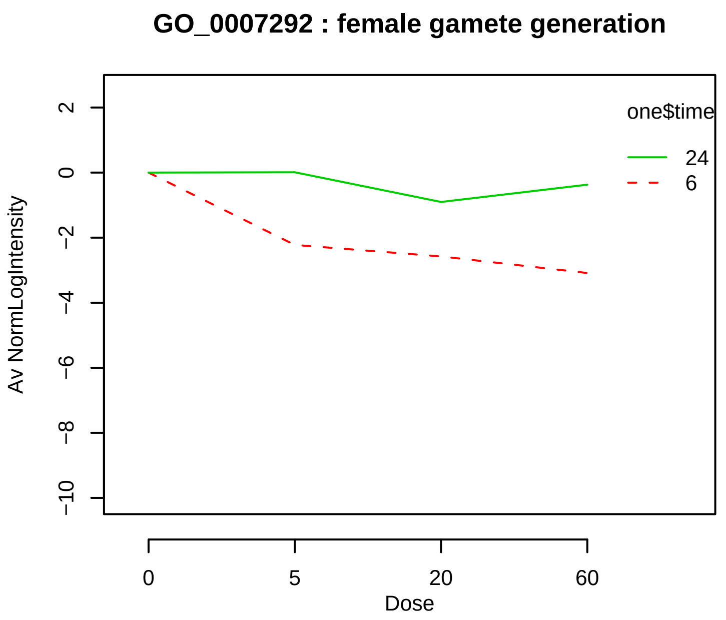
GO_0007292 : female gamete generation
one$time
24
6
Av NormLogIntensity
Dose

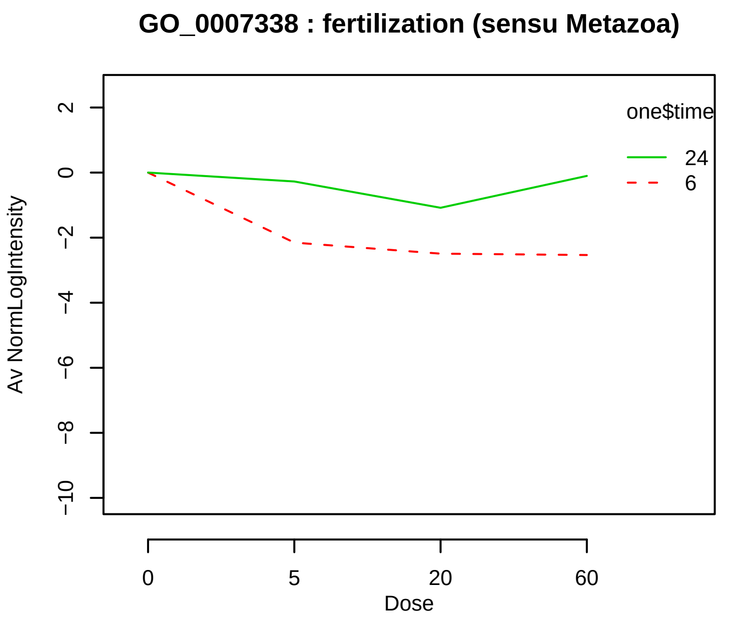
GO_0007338 : fertilization (sensu Metazoa)
one$time
24
6
Av NormLogIntensity
Dose

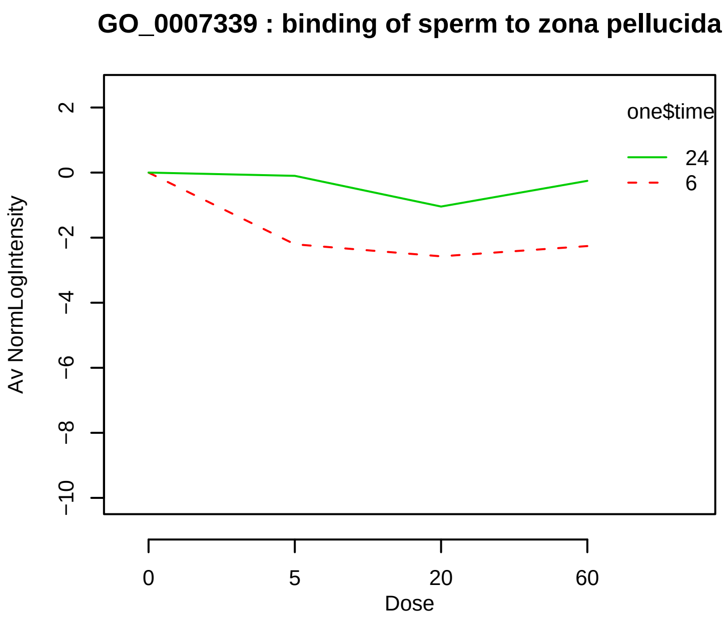
GO_0007339 : binding of sperm to zona pellucida
one$time
24
6
Av NormLogIntensity
Dose

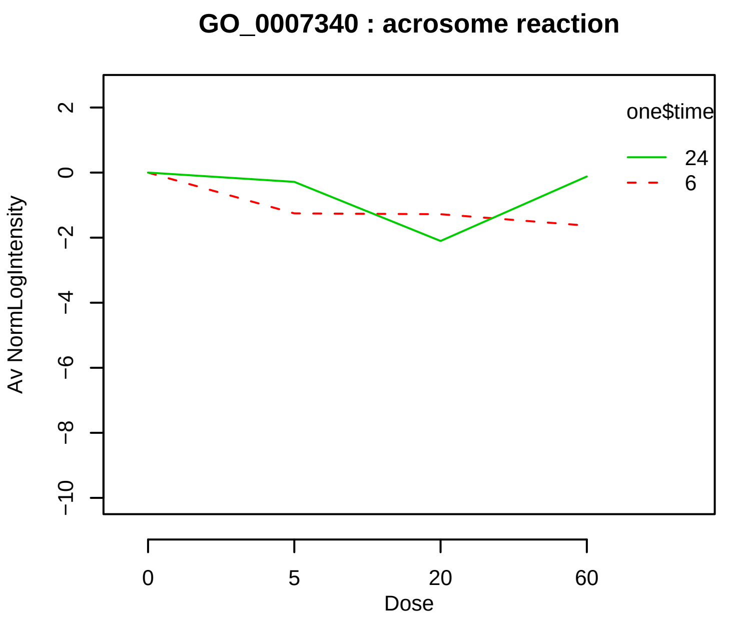
GO_0007340 : acrosome reaction
one$time
24
6
Av NormLogIntensity
Dose

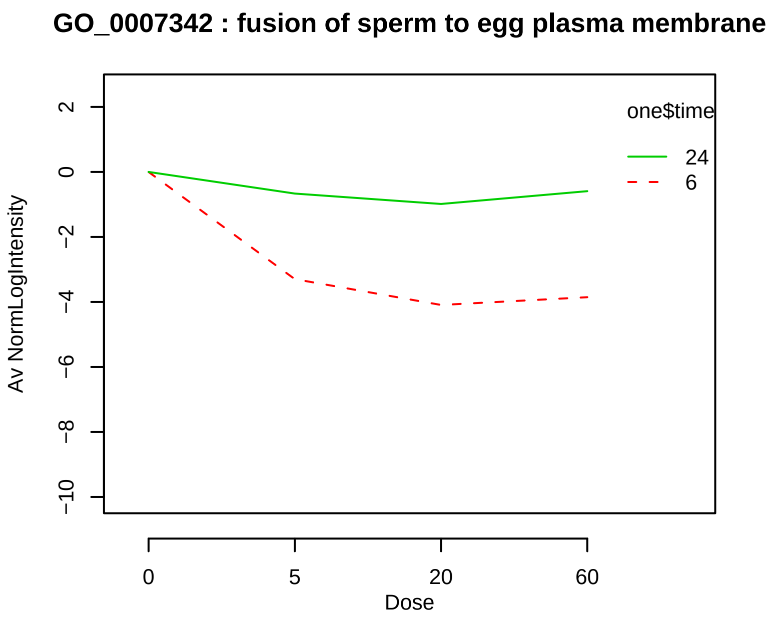
GO_0007342 : fusion of sperm to egg plasma membrane
one$time
24
6
Av NormLogIntensity
Dose

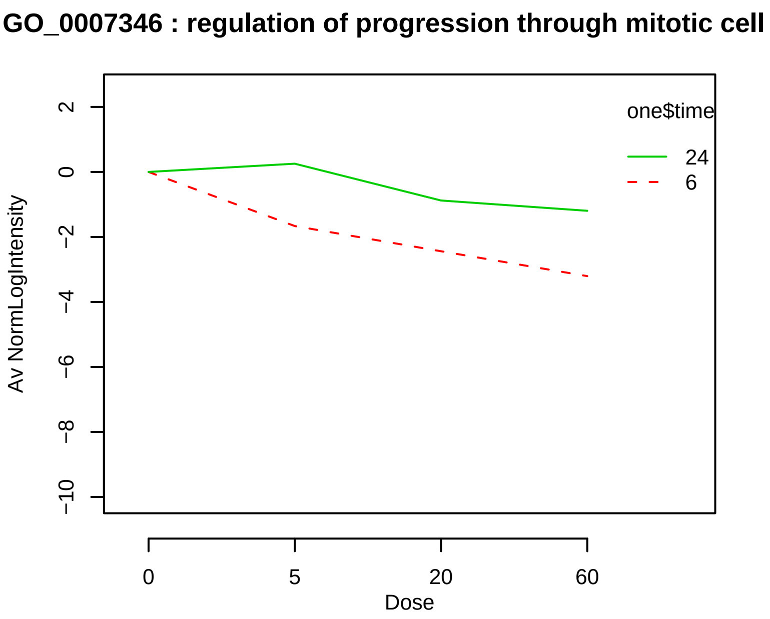
GO_0007346 : regulation of progression through mitotic cell
one$time
24
6
Av NormLogIntensity
Dose

GO_0007350 : blastoderm segmentation

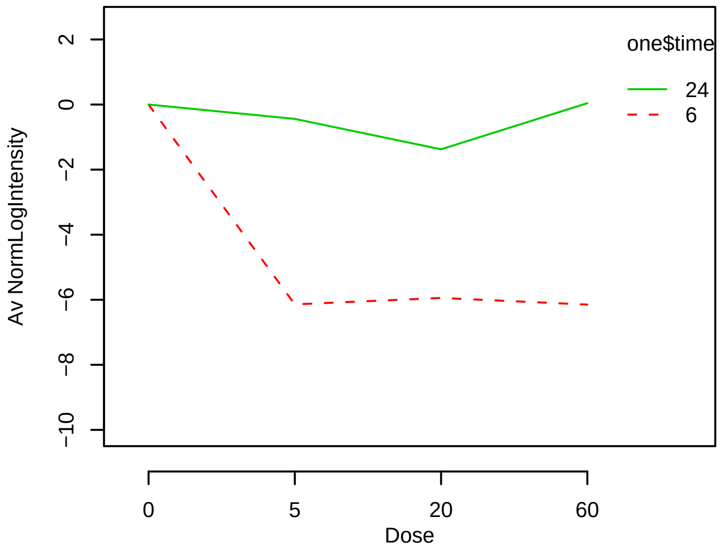

GO_0007351 : regional subdivision

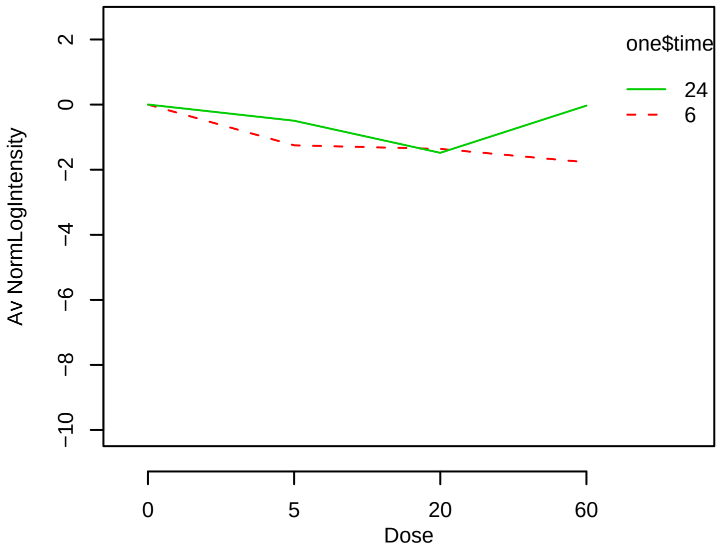

GO_0007368 : determination of left/right symmetry

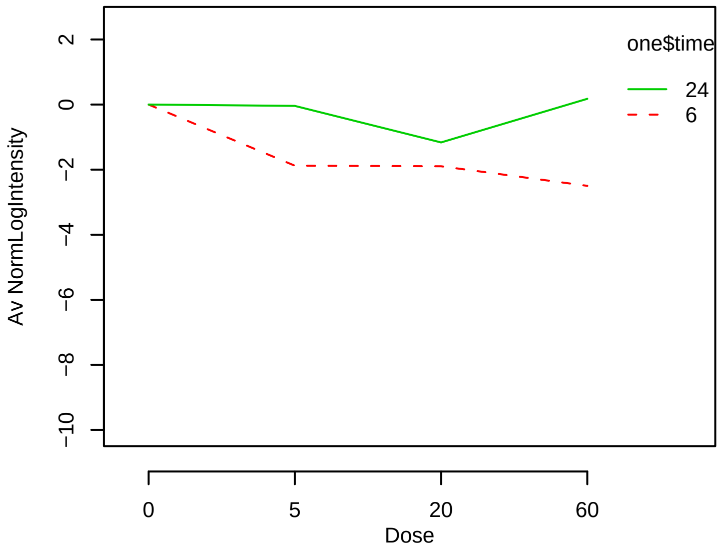

GO_0007369 : gastrulation

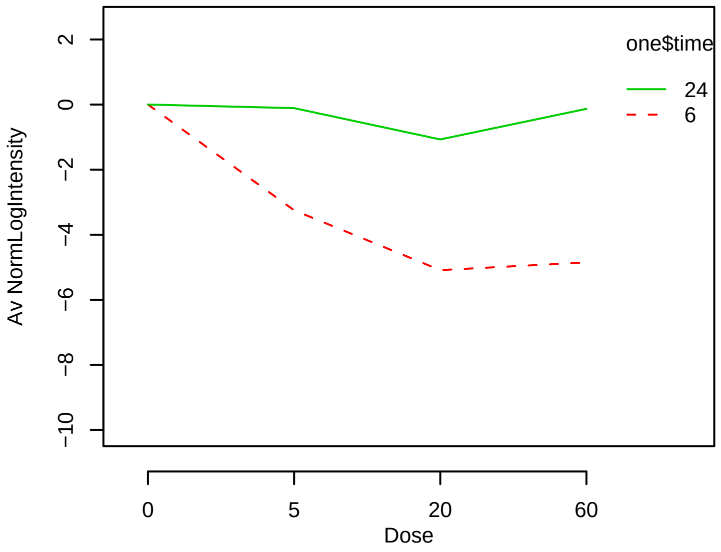

GO_0007379 : segment specification

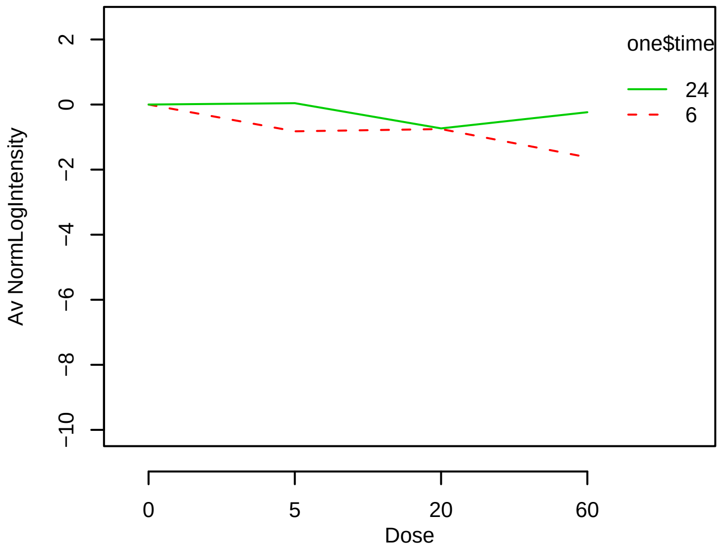

GO_0007386 : compartment specification

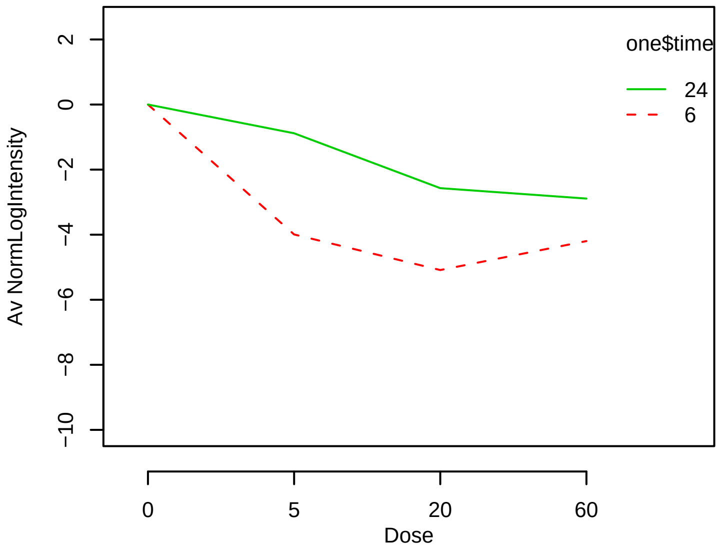

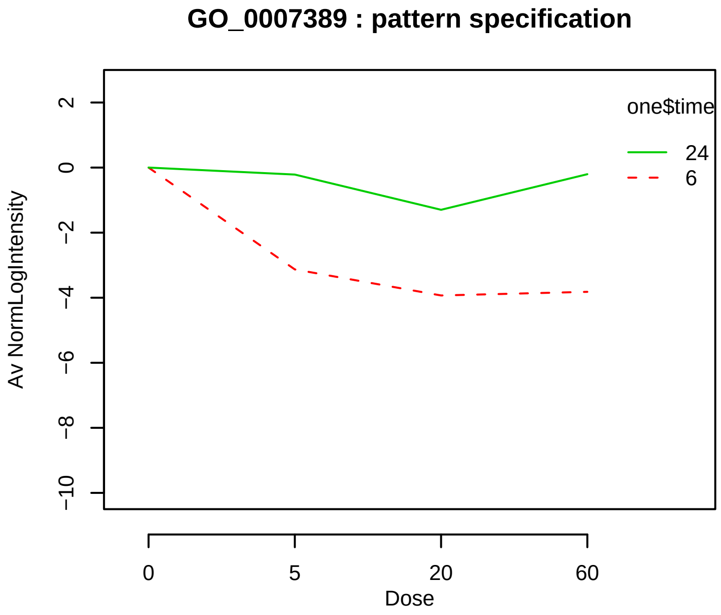
GO_0007389 : pattern specification
Av NormLogIntensity
2 0 −2 −4 −6 −8 −10
one$time
24
6
0 5 20 60
Dose

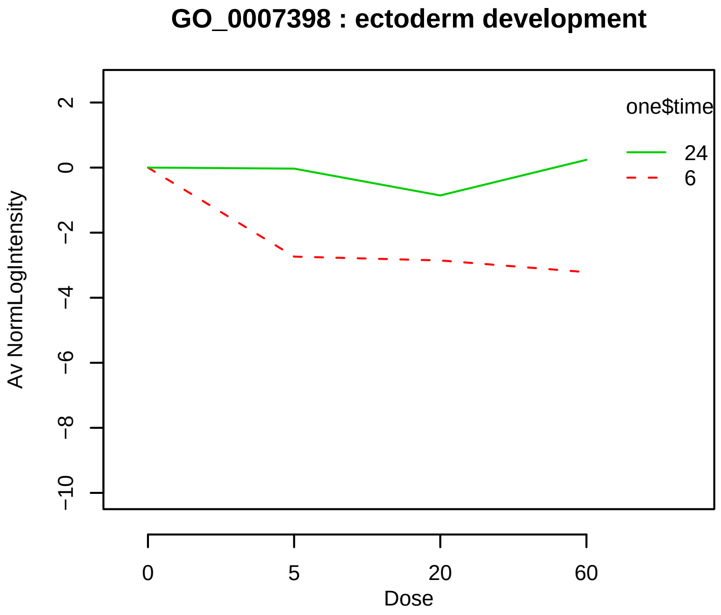
GO_0007398 : ectoderm development
Av NormLogIntensity
2 0 −2 −4 −6 −8 −10
one$time
24
6
0 5 20 60
Dose

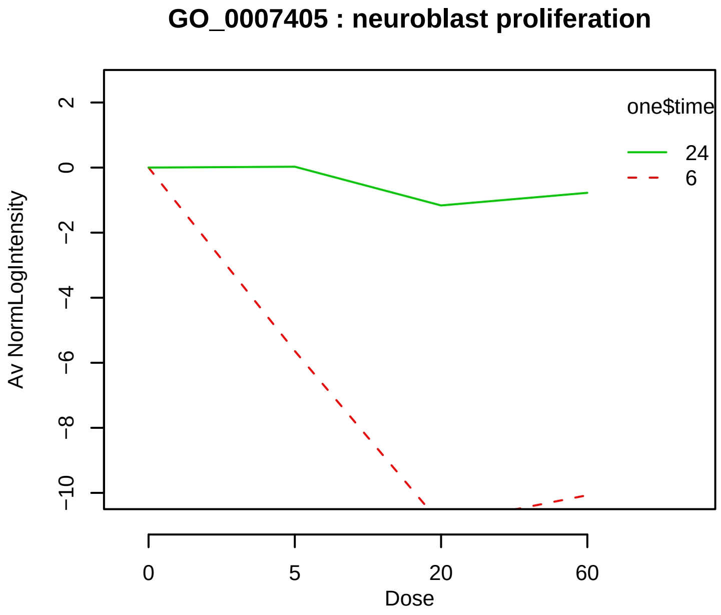
GO_0007405 : neuroblast proliferation
Av NormLogIntensity
2 0 −2 −4 −6 −8 −10
one$time
24
6
0 5 20 60
Dose

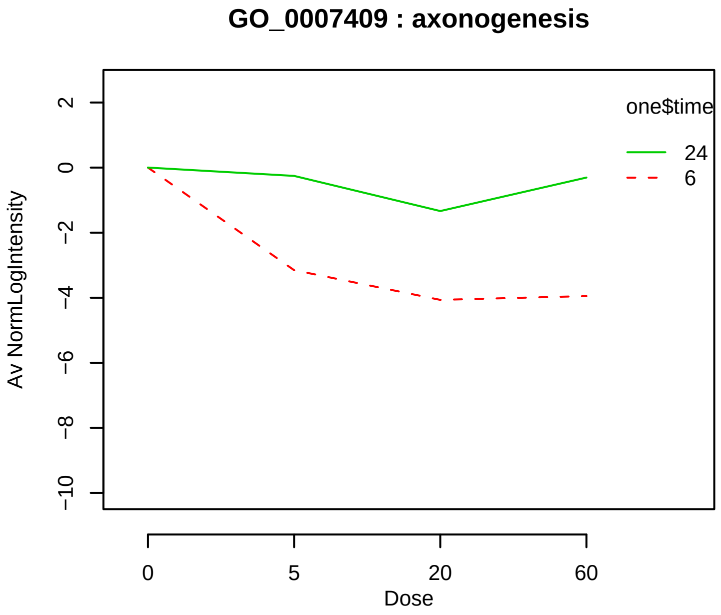
GO_0007409 : axonogenesis
Av NormLogIntensity
2 0 −2 −4 −6 −8 −10
one$time
24
6
0 5 20 60
Dose

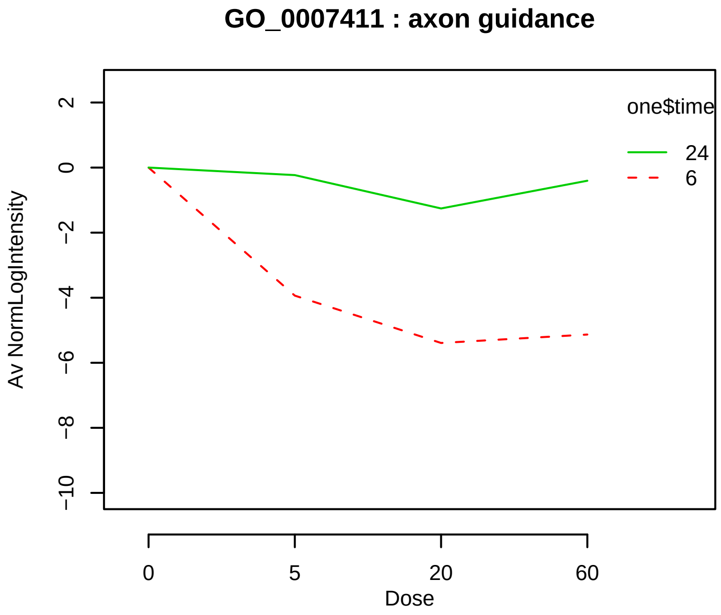
GO_0007411 : axon guidance
Av NormLogIntensity
2 0 −2 −4 −6 −8 −10
one$time
24
6
0 5 20 60
Dose

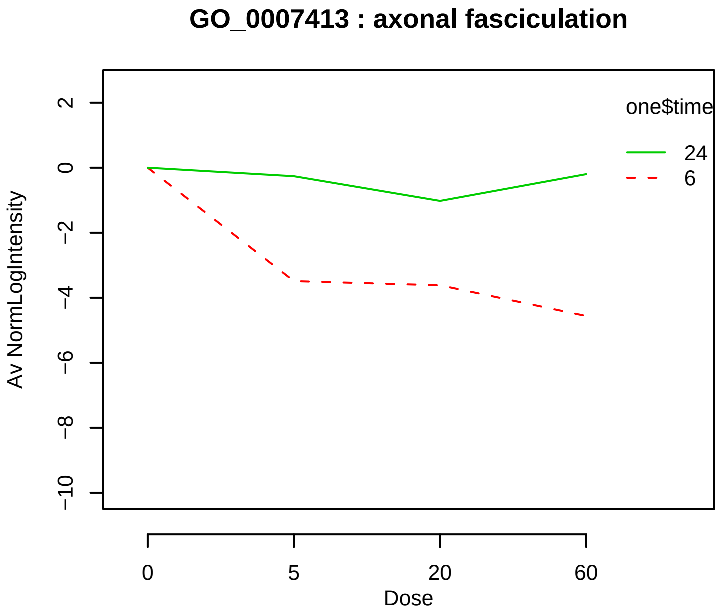
GO_0007413 : axonal fasciculation
Av NormLogIntensity
2 0 −2 −4 −6 −8 −10
one$time
24
6
0 5 20 60
Dose

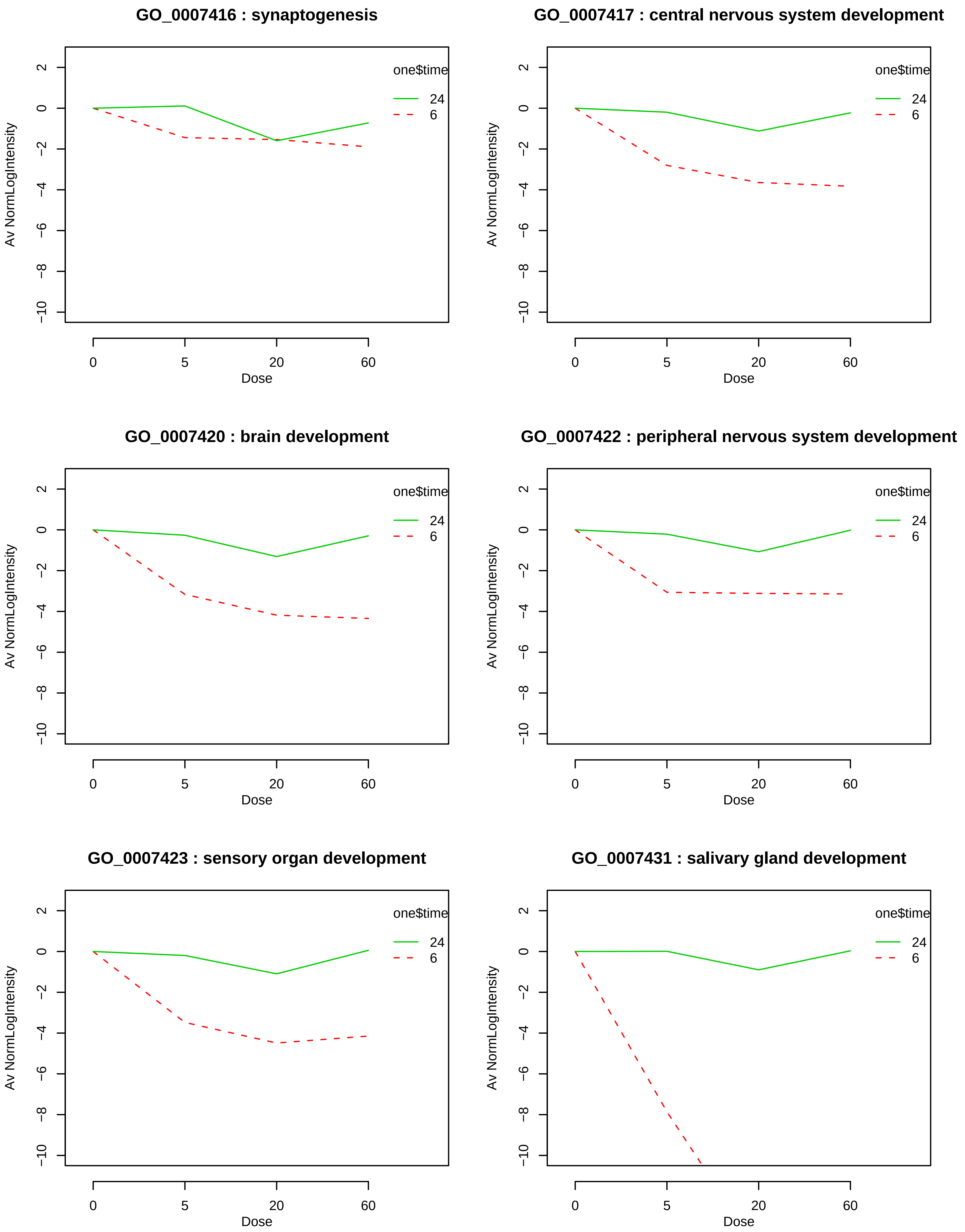

GO_0007416 : synaptogenesis
GO_0007417 : central nervous system development
GO_0007420 : brain development
GO_0007422 : peripheral nervous system development
GO_0007423 : sensory organ development
GO_0007431 : salivary gland development
one$time
24
6
Av NormLogIntensity
Dose
0
5
20
60
2
0
−2
−4
−6
−8
−10

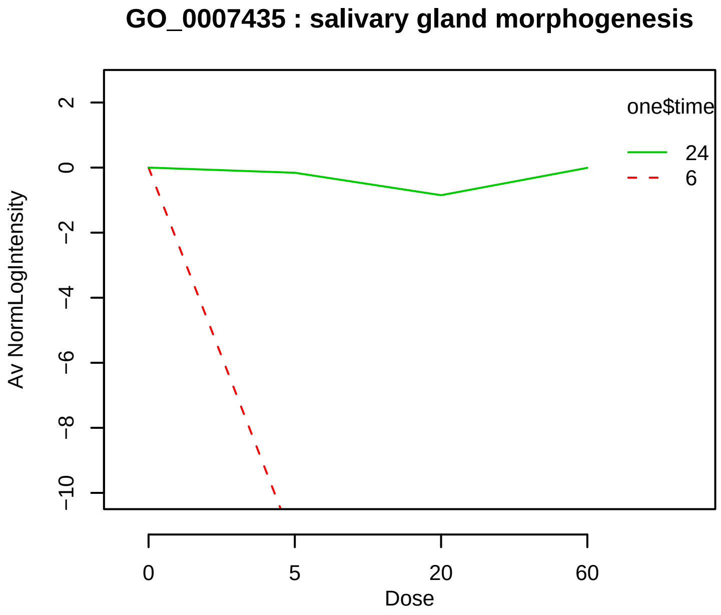
GO_0007435 : salivary gland morphogenesis
Av NormLogIntensity
2
0
−2
−4
−6
−8
−10
one$time
24
6
0
5
20
60
Dose

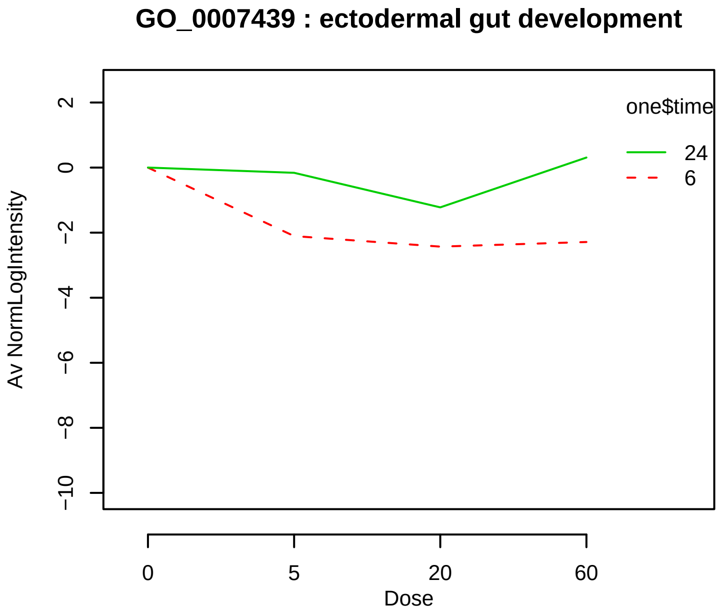
GO_0007439 : ectodermal gut development
Av NormLogIntensity
2
0
−2
−4
−6
−8
−10
one$time
24
6
0
5
20
60
Dose

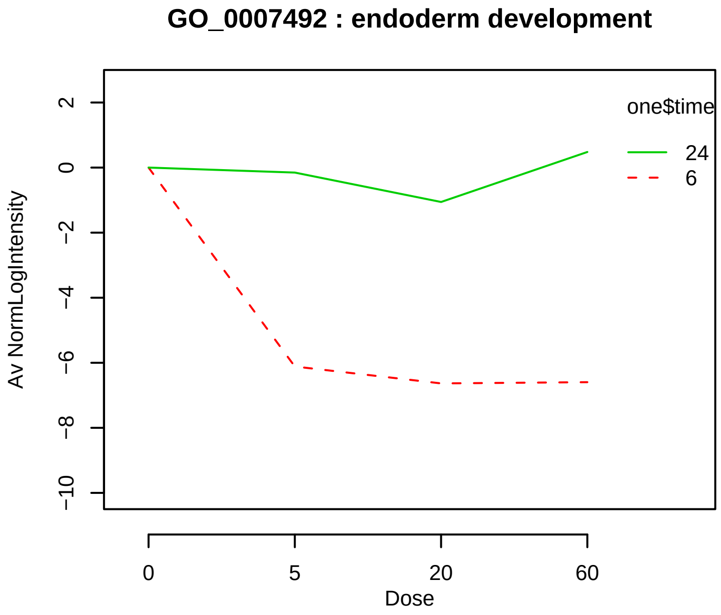
GO_0007492 : endoderm development
Av NormLogIntensity
2
0
−2
−4
−6
−8
−10
one$time
24
6
0
5
20
60
Dose

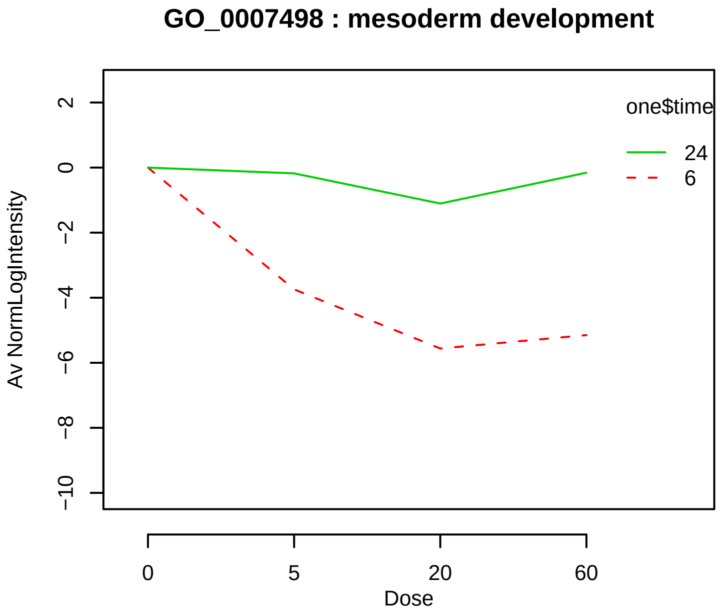
GO_0007498 : mesoderm development
Av NormLogIntensity
2
0
−2
−4
−6
−8
−10
one$time
24
6
0
5
20
60
Dose

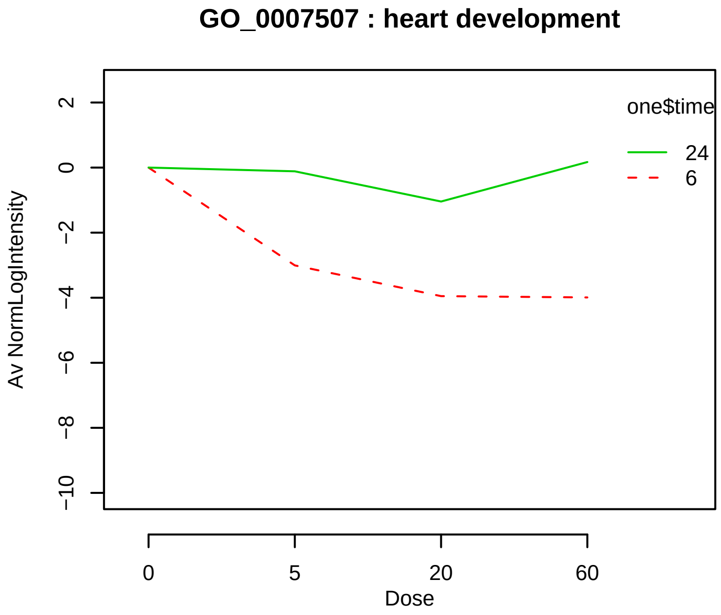
GO_0007507 : heart development
Av NormLogIntensity
2
0
−2
−4
−6
−8
−10
one$time
24
6
0
5
20
60
Dose

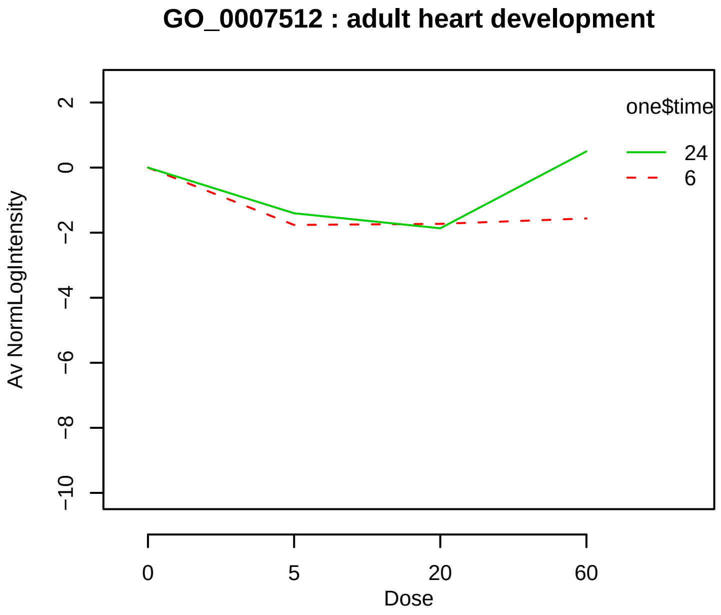
GO_0007512 : adult heart development
Av NormLogIntensity
2
0
−2
−4
−6
−8
−10
one$time
24
6
0
5
20
60
Dose

GO_0007516 : hemocyte development (sensu Arthropoda

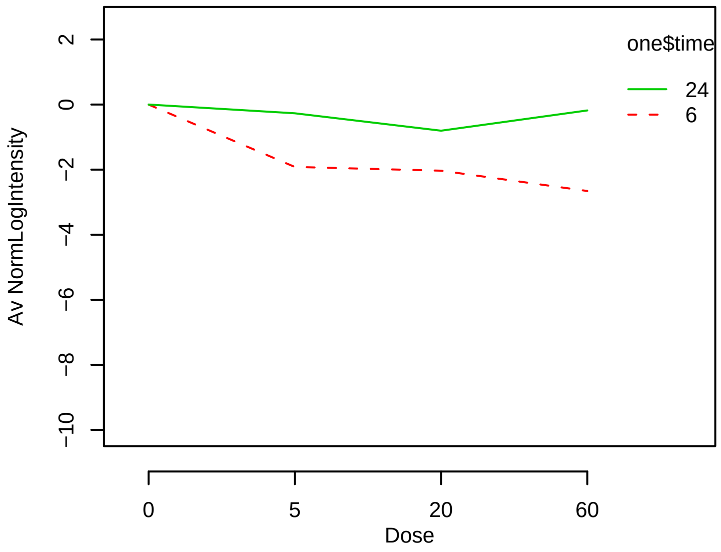

GO_0007517 : muscle development

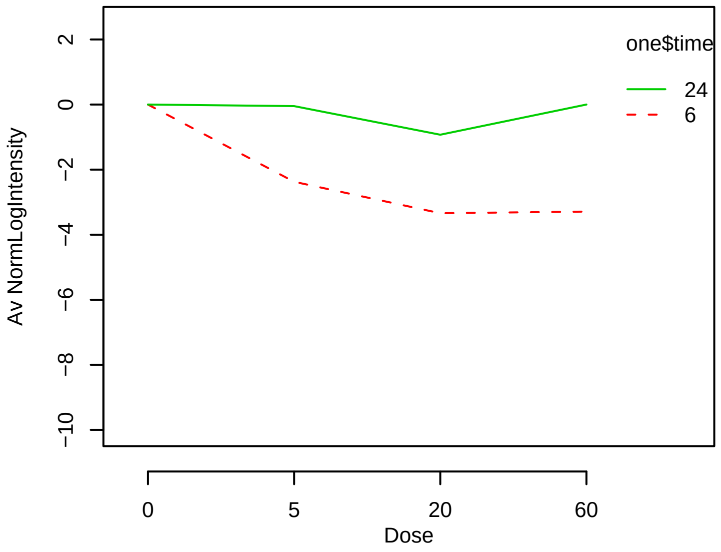

GO_0007519 : striated muscle development

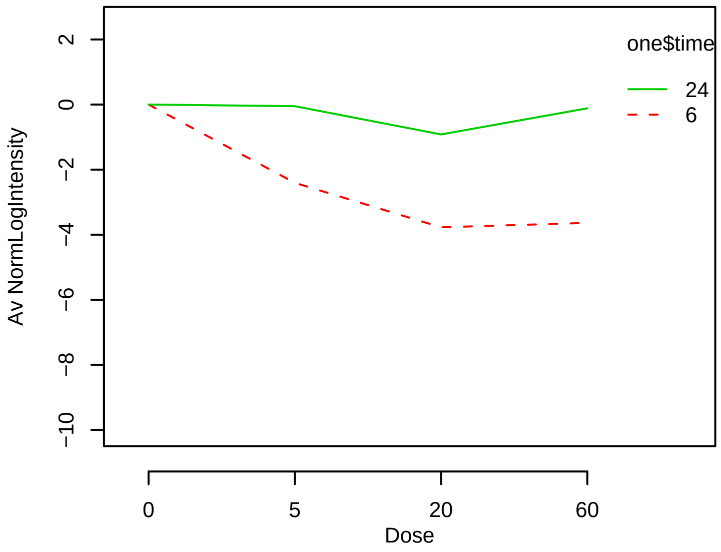

GO_0007520 : myoblast fusion

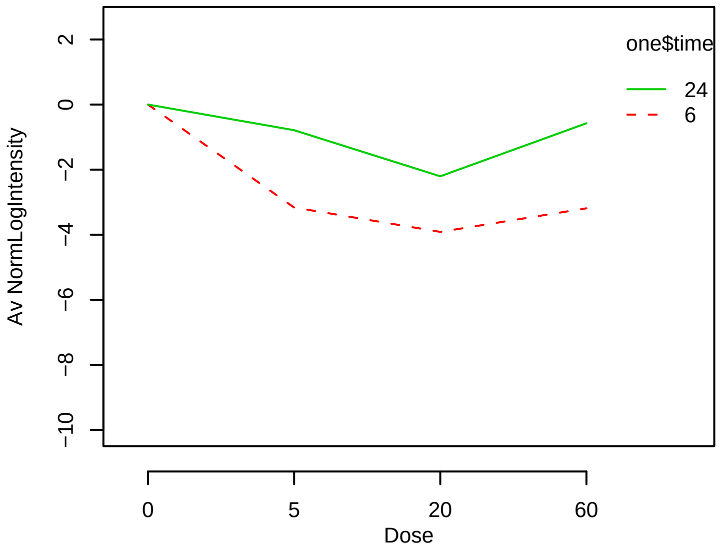

GO_0007528 : neuromuscular junction development

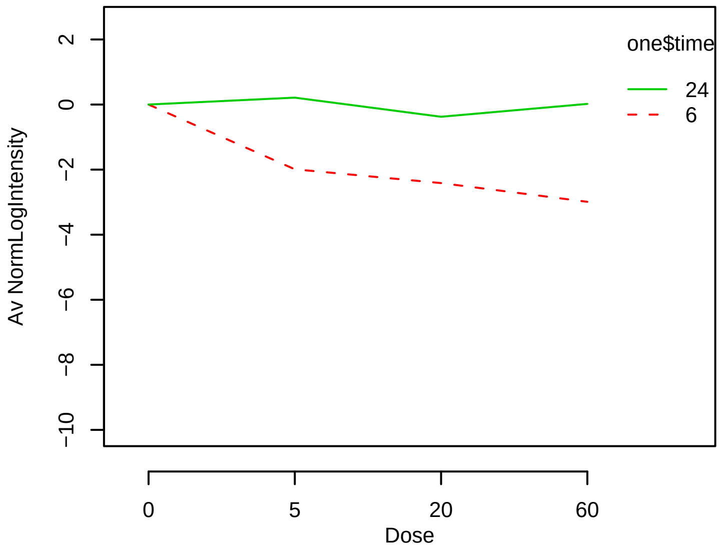

GO_0007530 : sex determination

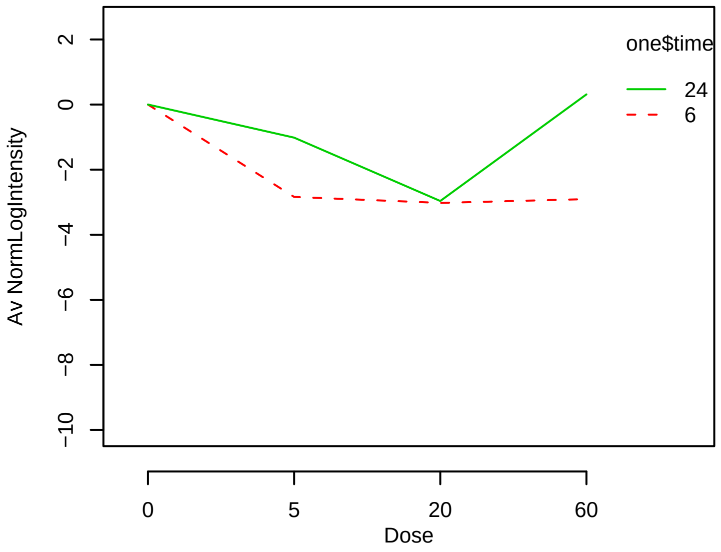

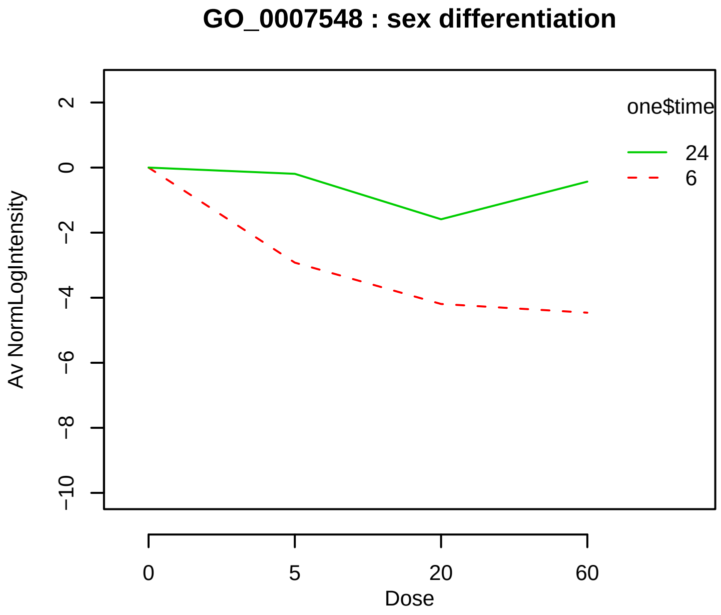
GO_0007548 : sex differentiation
one$time
24
6
Av NormLogIntensity
2
0
−2
−4
−6
−8
−10
0
5
20
60
Dose

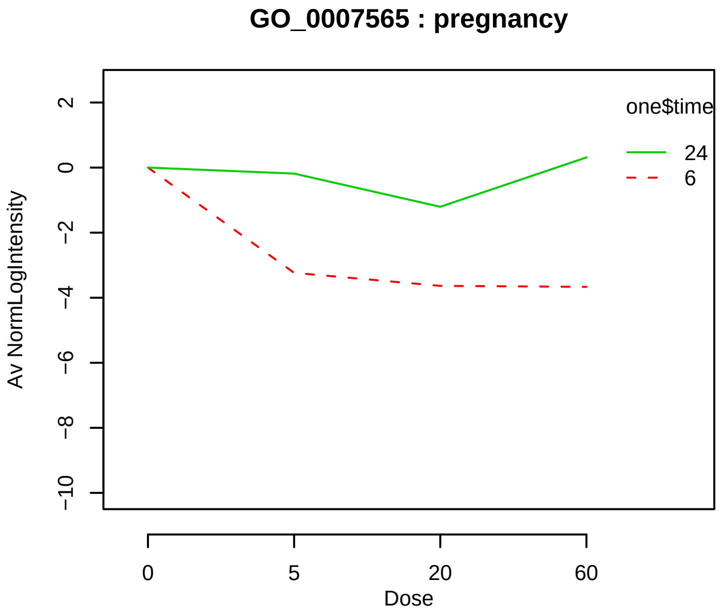
GO_0007565 : pregnancy
one$time
24
6
Av NormLogIntensity
2
0
−2
−4
−6
−8
−10
0
5
20
60
Dose

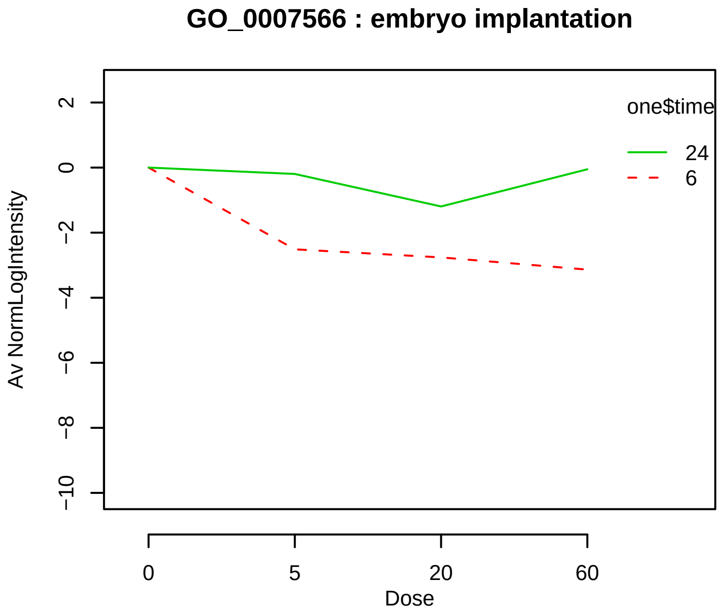
GO_0007566 : embryo implantation
one$time
24
6
Av NormLogIntensity
2
0
−2
−4
−6
−8
−10
0
5
20
60
Dose

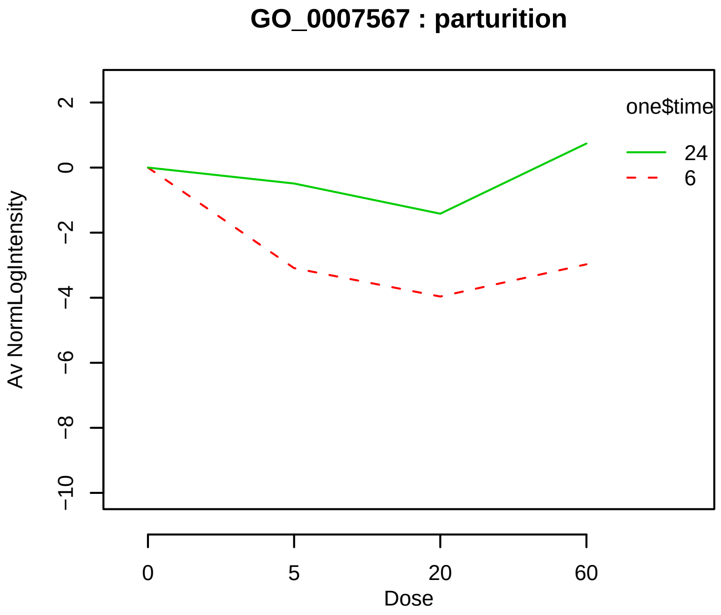
GO_0007567 : parturition
one$time
24
6
Av NormLogIntensity
2
0
−2
−4
−6
−8
−10
0
5
20
60
Dose

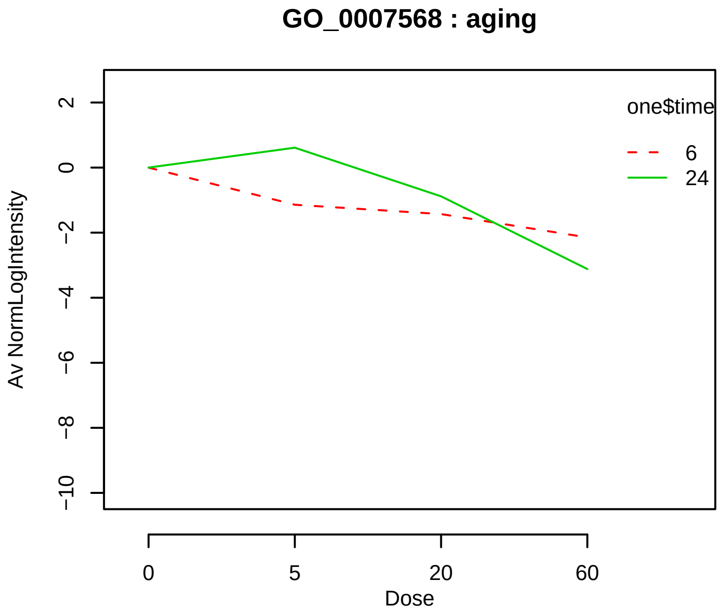
GO_0007568 : aging
one$time
6
24
Av NormLogIntensity
2
0
−2
−4
−6
−8
−10
0
5
20
60
Dose

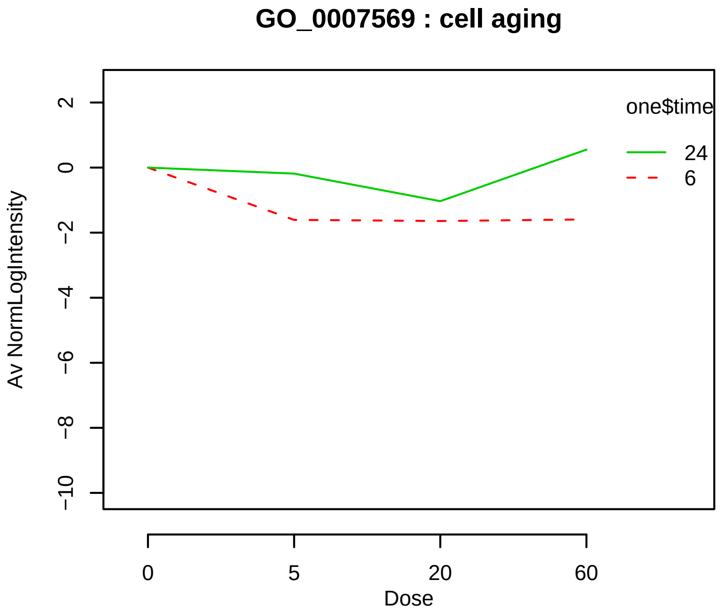
GO_0007569 : cell aging
one$time
24
6
Av NormLogIntensity
2
0
−2
−4
−6
−8
−10
0
5
20
60
Dose

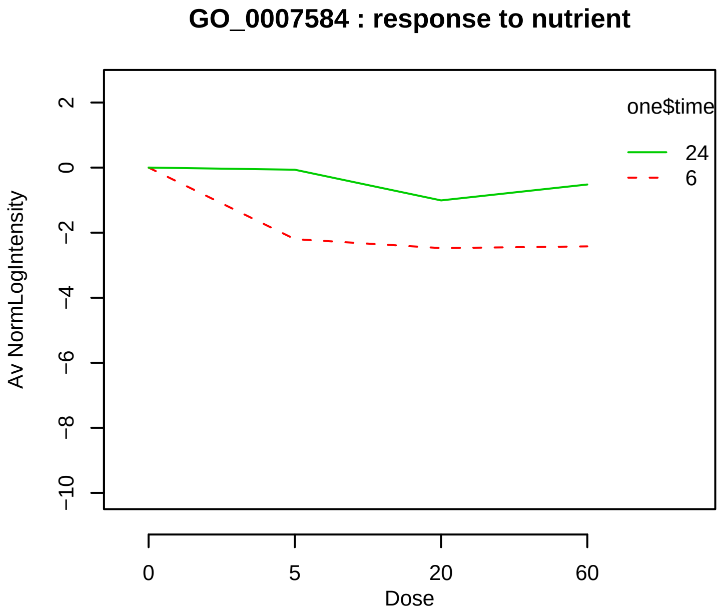
GO_0007584 : response to nutrient
Av NormLogIntensity
2
0
−2
−4
−6
−8
−10
one$time
24
6
0
5
20
60
Dose

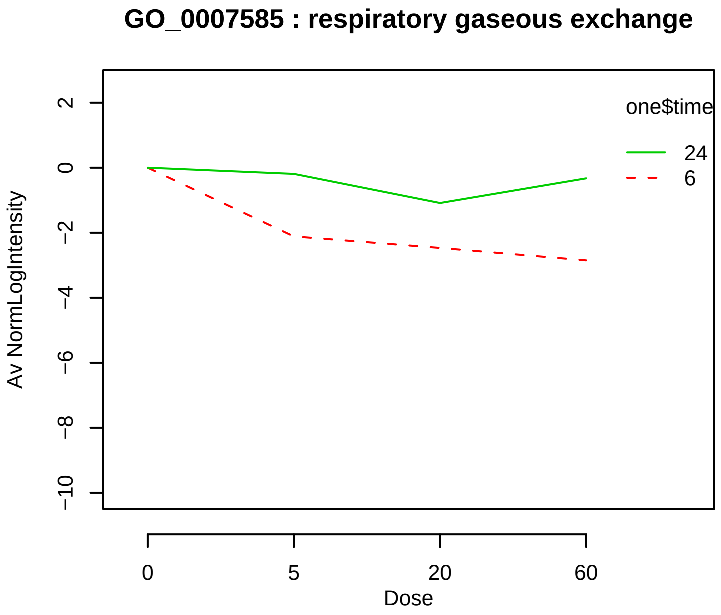
GO_0007585 : respiratory gaseous exchange
Av NormLogIntensity
2
0
−2
−4
−6
−8
−10
one$time
24
6
0
5
20
60
Dose

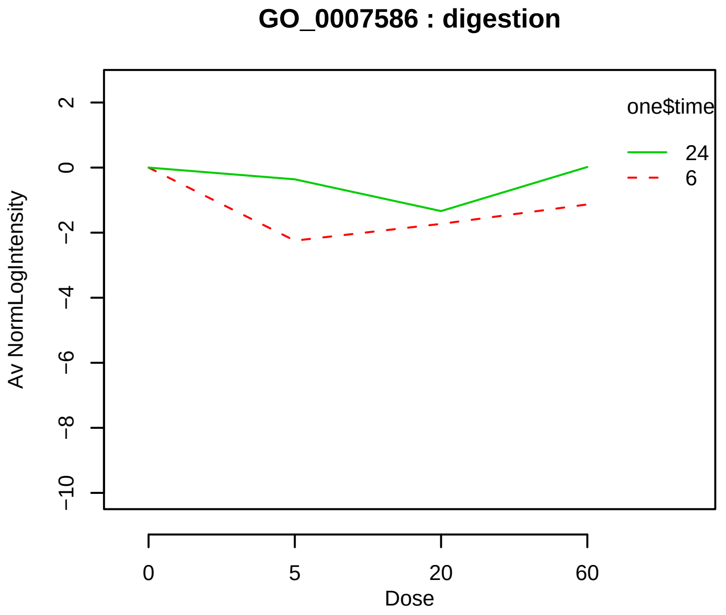
GO_0007586 : digestion
Av NormLogIntensity
2
0
−2
−4
−6
−8
−10
one$time
24
6
0
5
20
60
Dose

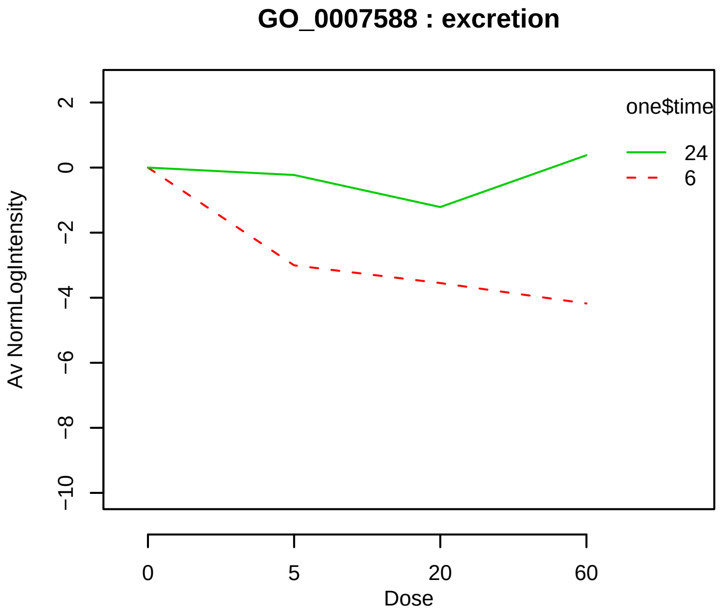
GO_0007588 : excretion
Av NormLogIntensity
2
0
−2
−4
−6
−8
−10
one$time
24
6
0
5
20
60
Dose

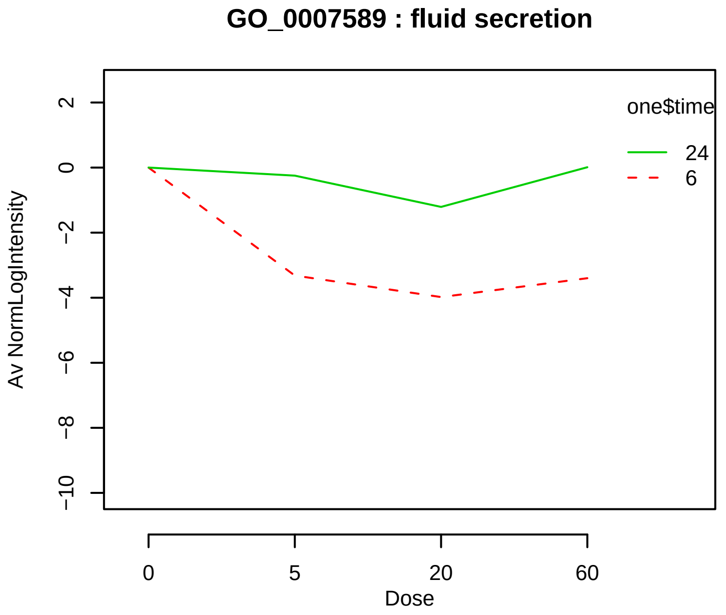
GO_0007589 : fluid secretion
Av NormLogIntensity
2
0
−2
−4
−6
−8
−10
one$time
24
6
0
5
20
60
Dose

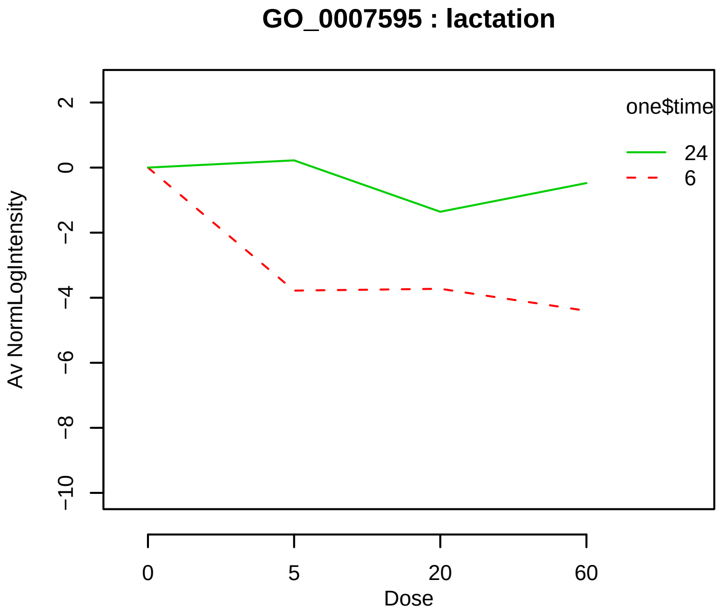
GO_0007595 : lactation
Av NormLogIntensity
2
0
−2
−4
−6
−8
−10
one$time
24
6
0
5
20
60
Dose

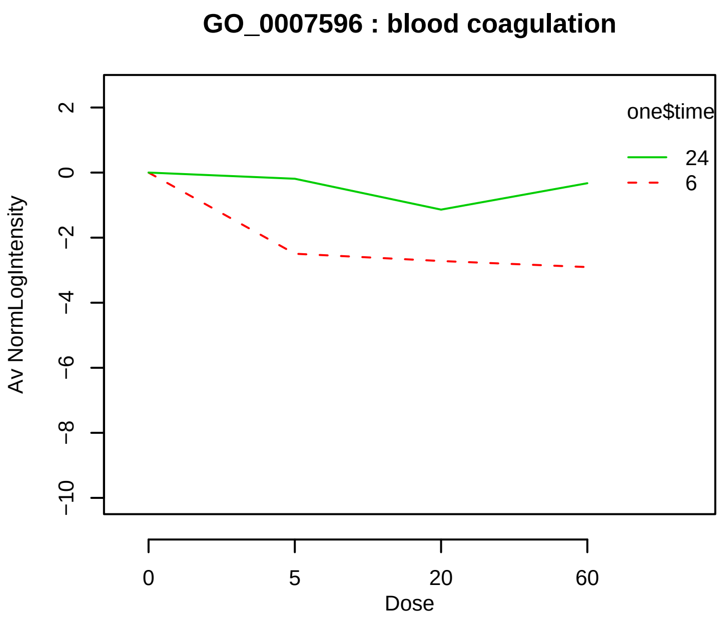
GO_0007596 : blood coagulation
Av NormLogIntensity
2
0
−2
−4
−6
−8
−10
0
5
20
60
Dose
one$time
24
6

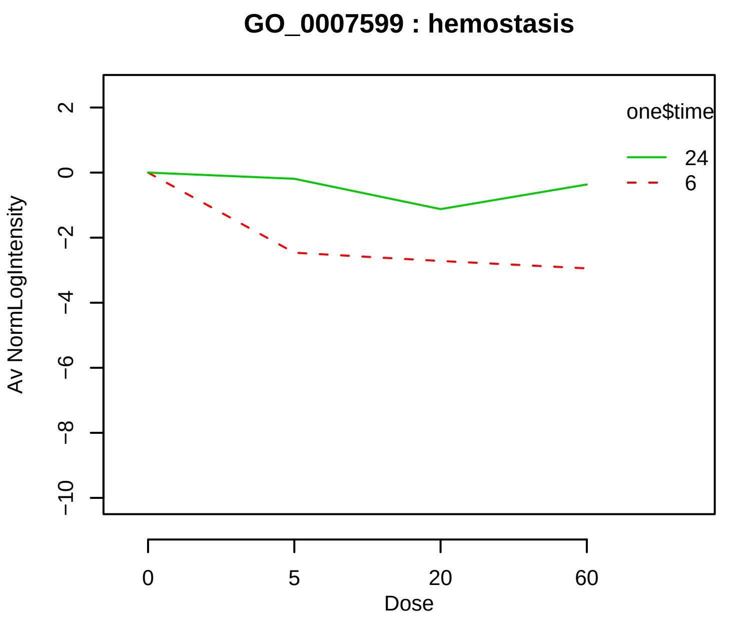
GO_0007599 : hemostasis
Av NormLogIntensity
2
0
−2
−4
−6
−8
−10
0
5
20
60
Dose
one$time
24
6

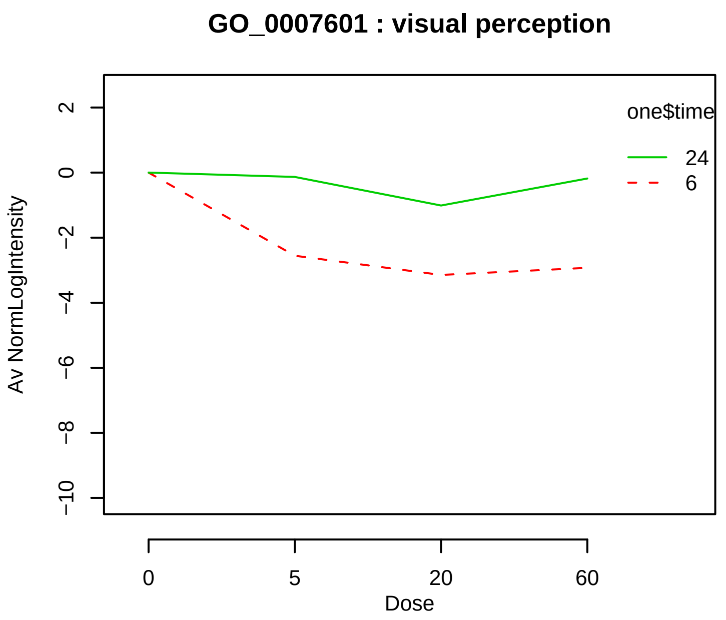
GO_0007601 : visual perception
Av NormLogIntensity
2
0
−2
−4
−6
−8
−10
0
5
20
60
Dose
one$time
24
6

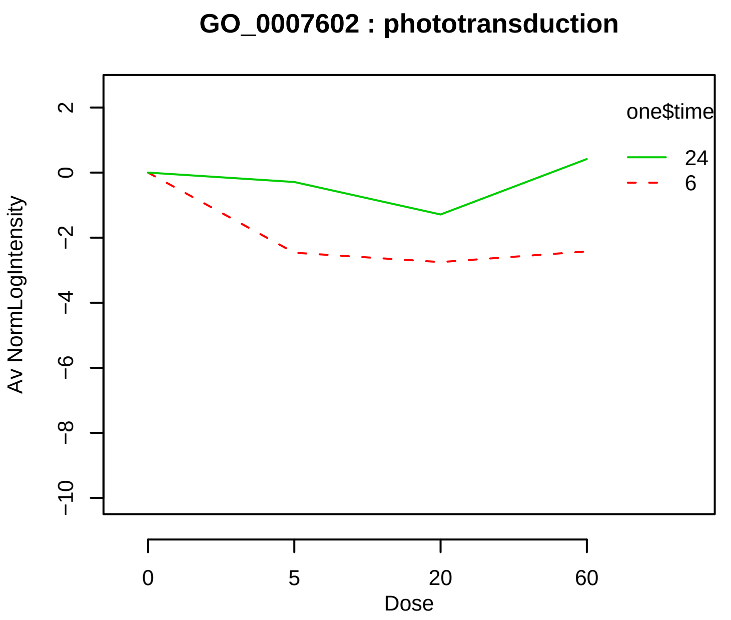
GO_0007602 : phototransduction
Av NormLogIntensity
2
0
−2
−4
−6
−8
−10
0
5
20
60
Dose
one$time
24
6

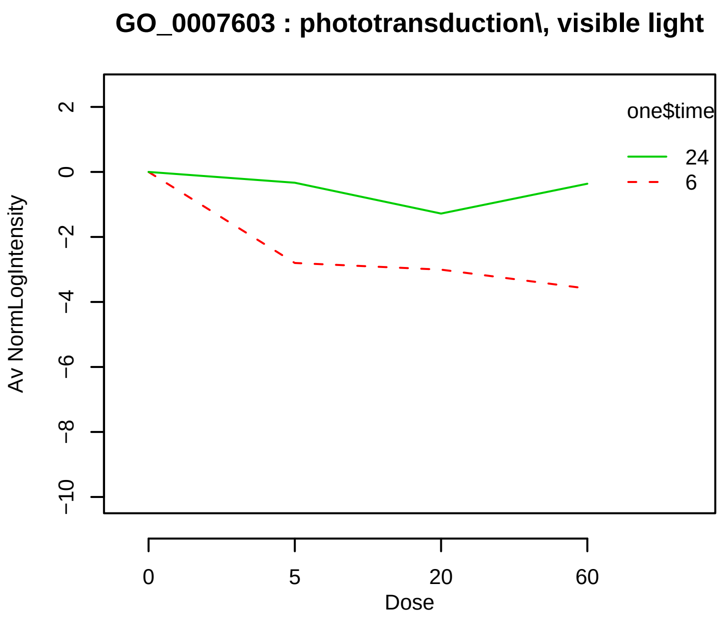
GO_0007603 : phototransduction\, visible light
Av NormLogIntensity
2
0
−2
−4
−6
−8
−10
0
5
20
60
Dose
one$time
24
6

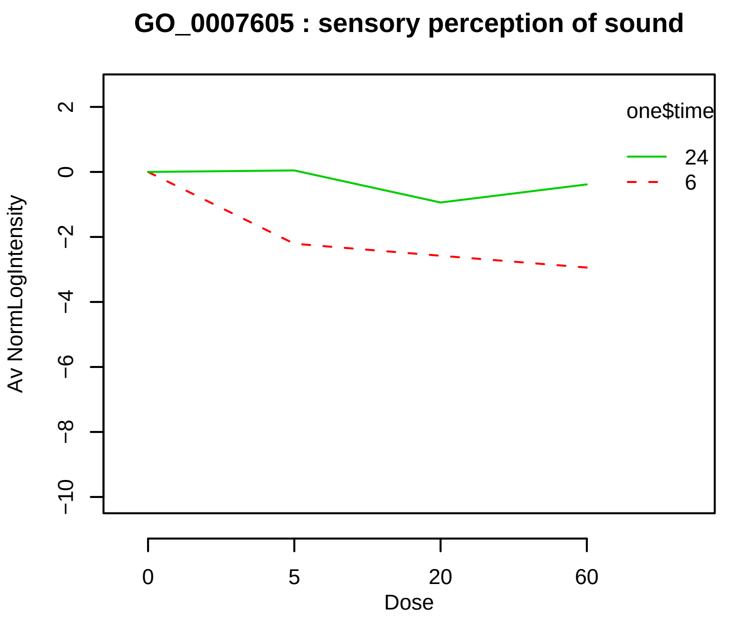
GO_0007605 : sensory perception of sound
Av NormLogIntensity
2
0
−2
−4
−6
−8
−10
0
5
20
60
Dose
one$time
24
6

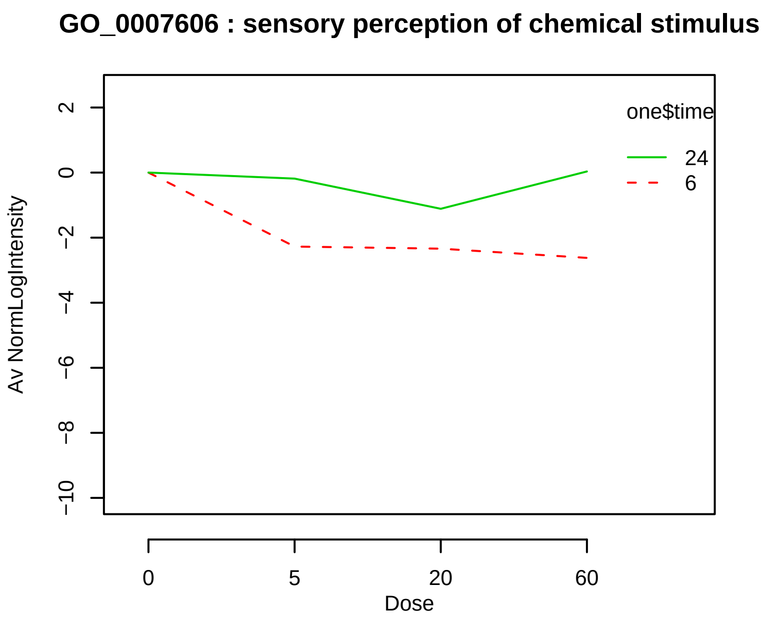
GO_0007606 : sensory perception of chemical stimulus
Av NormLogIntensity
Dose
one$time
24
6
0
5
20
60
2
0
−2
−4
−6
−8
−10

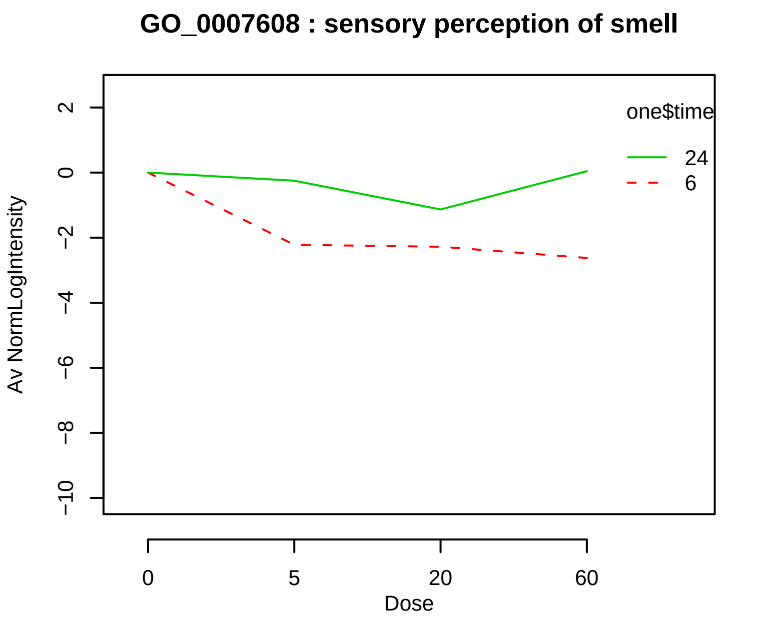
GO_0007608 : sensory perception of smell
Av NormLogIntensity
Dose
one$time
24
6
0
5
20
60
2
0
−2
−4
−6
−8
−10

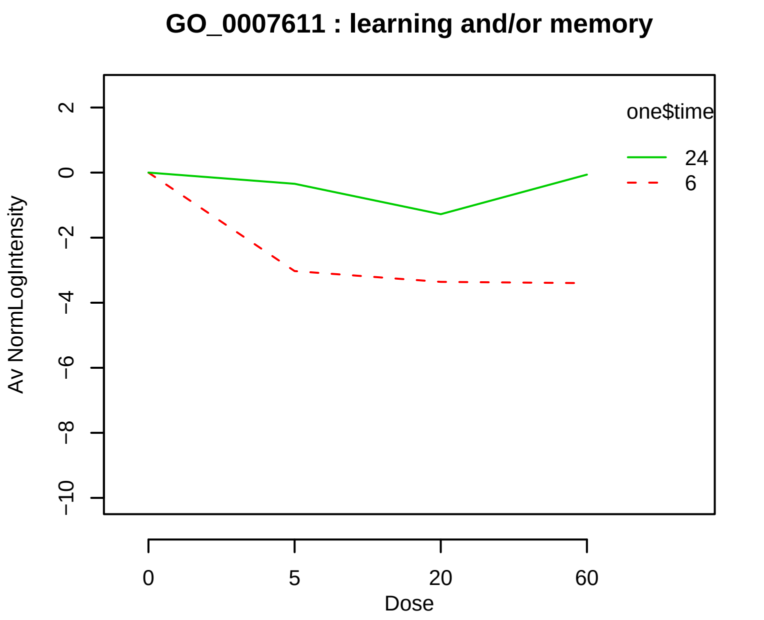
GO_0007611 : learning and/or memory
Av NormLogIntensity
Dose
one$time
24
6
0
5
20
60
2
0
−2
−4
−6
−8
−10

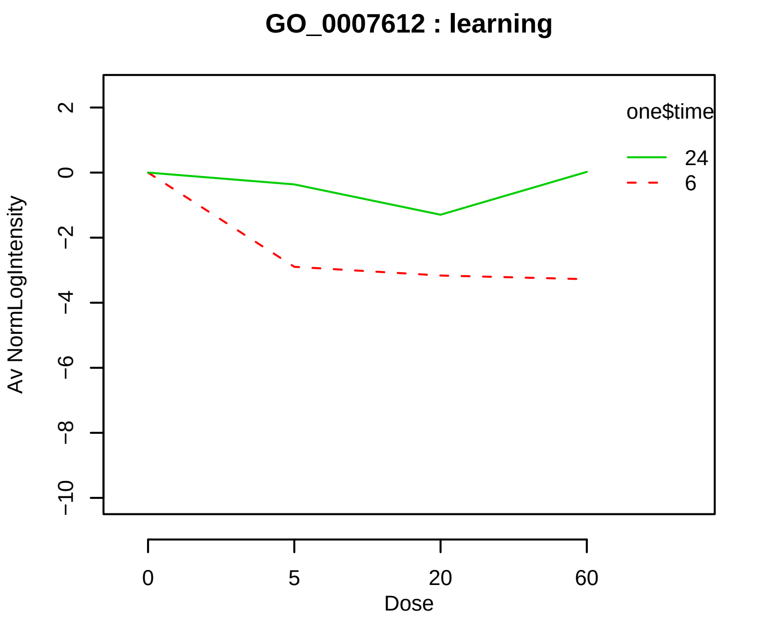
GO_0007612 : learning
Av NormLogIntensity
Dose
one$time
24
6
0
5
20
60
2
0
−2
−4
−6
−8
−10

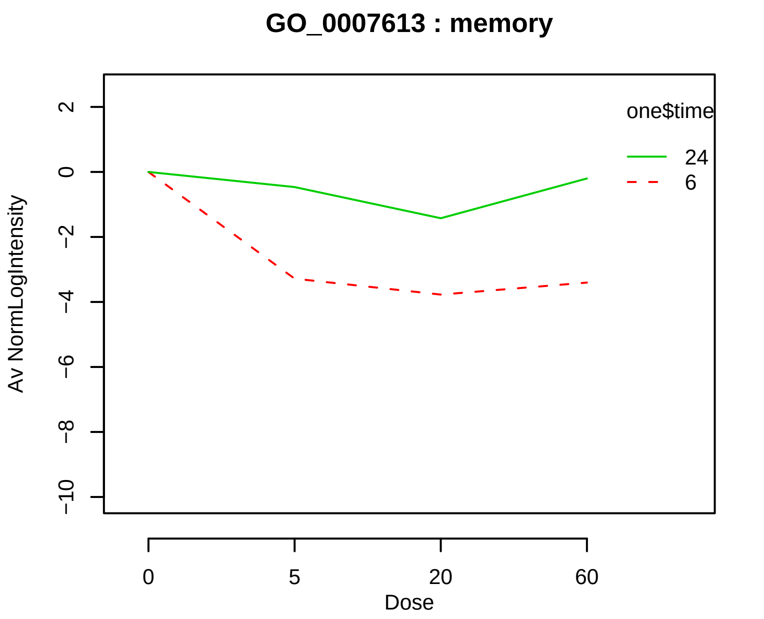
GO_0007613 : memory
Av NormLogIntensity
Dose
one$time
24
6
0
5
20
60
2
0
−2
−4
−6
−8
−10

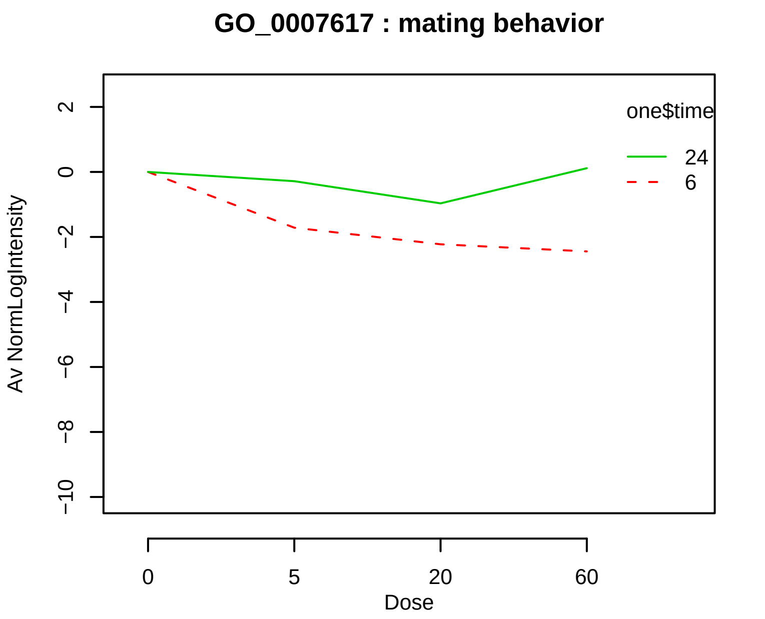
GO_0007617 : mating behavior
Av NormLogIntensity
Dose
one$time
24
6
0
5
20
60
2
0
−2
−4
−6
−8
−10

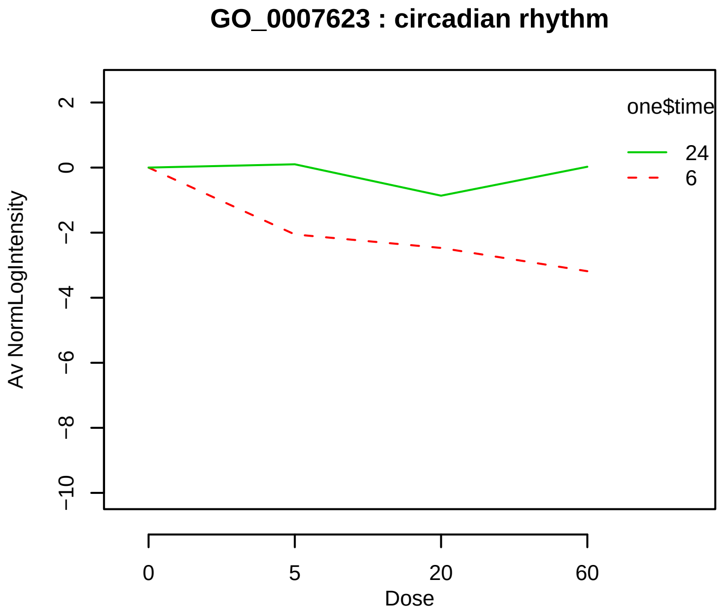
GO_0007623 : circadian rhythm
Av NormLogIntensity
Dose
one$time
24
6
2
0
−2
−4
−6
−8
−10
0
5
20
60

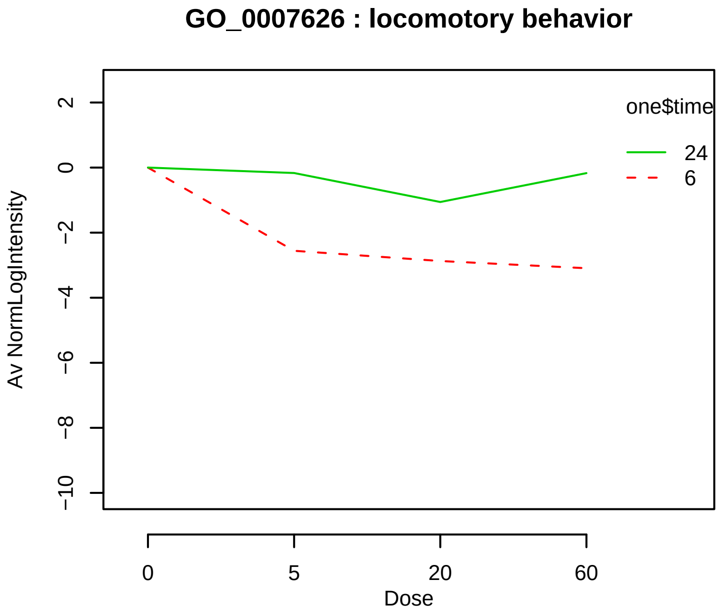
GO_0007626 : locomotory behavior
Av NormLogIntensity
Dose
one$time
24
6
2
0
−2
−4
−6
−8
−10
0
5
20
60

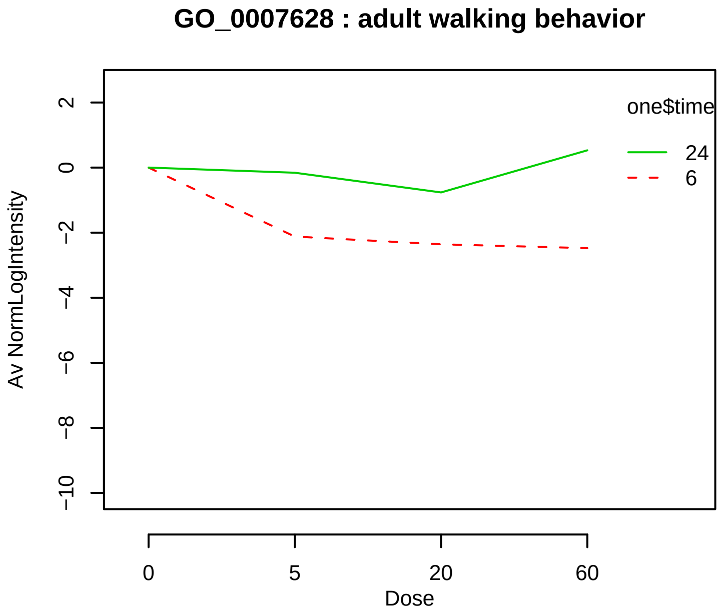
GO_0007628 : adult walking behavior
Av NormLogIntensity
Dose
one$time
24
6
2
0
−2
−4
−6
−8
−10
0
5
20
60

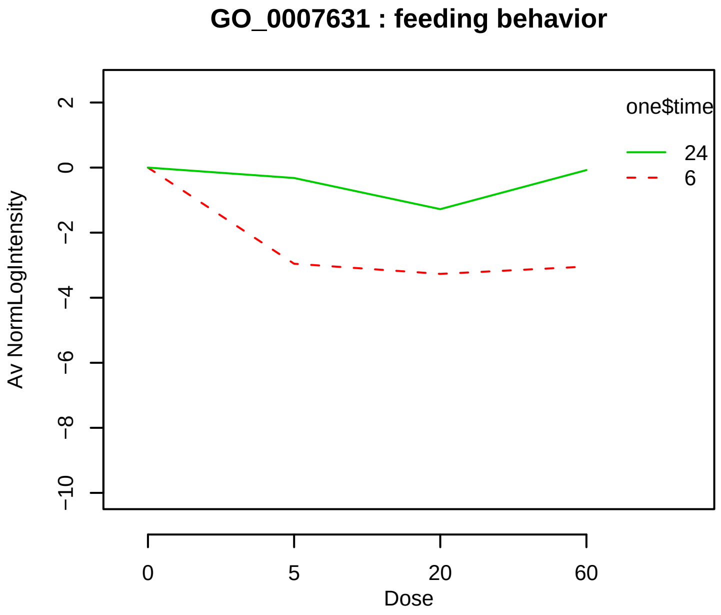
GO_0007631 : feeding behavior
Av NormLogIntensity
Dose
one$time
24
6
2
0
−2
−4
−6
−8
−10
0
5
20
60

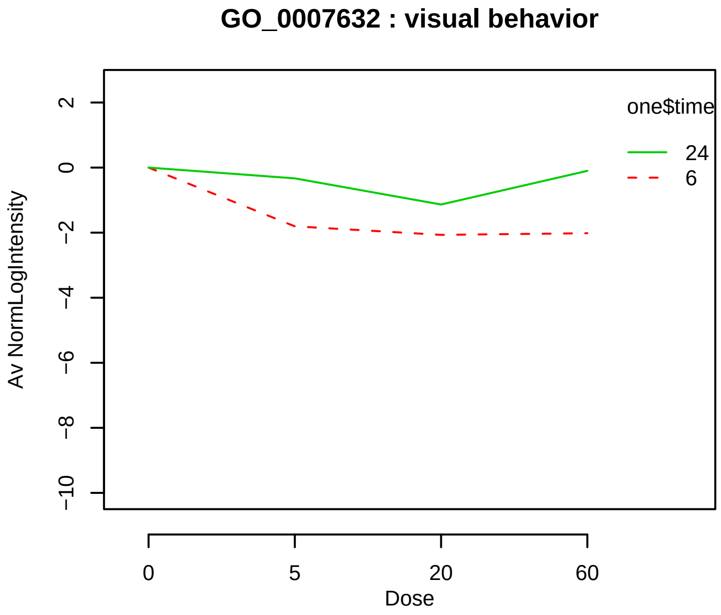
GO_0007632 : visual behavior
Av NormLogIntensity
Dose
one$time
24
6
2
0
−2
−4
−6
−8
−10
0
5
20
60

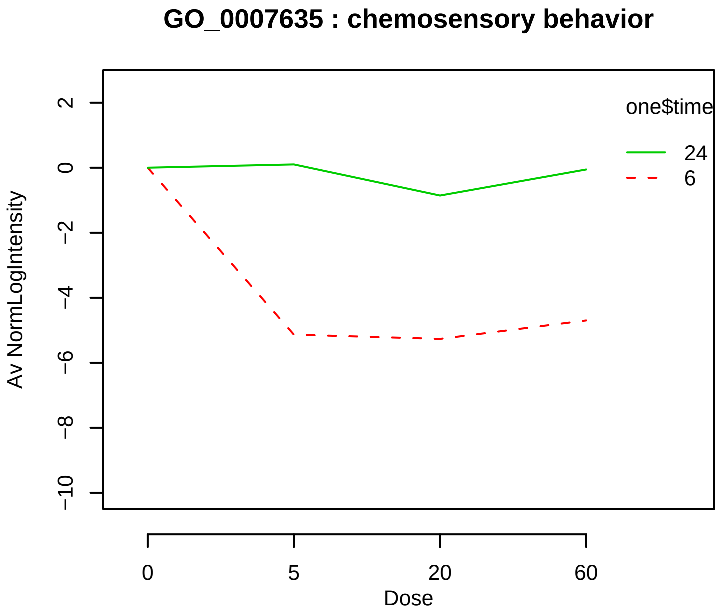
GO_0007635 : chemosensory behavior
Av NormLogIntensity
Dose
one$time
24
6
2
0
−2
−4
−6
−8
−10
0
5
20
60

GO_0007638 : mechanosensory behavior

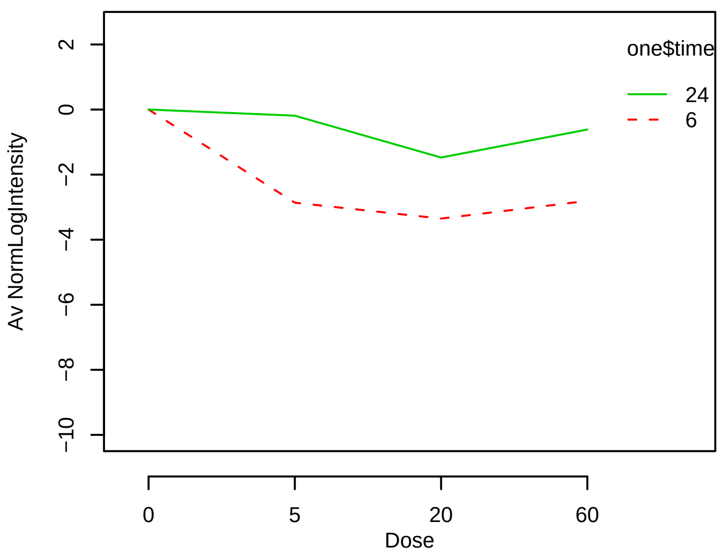

GO_0008015 : circulation

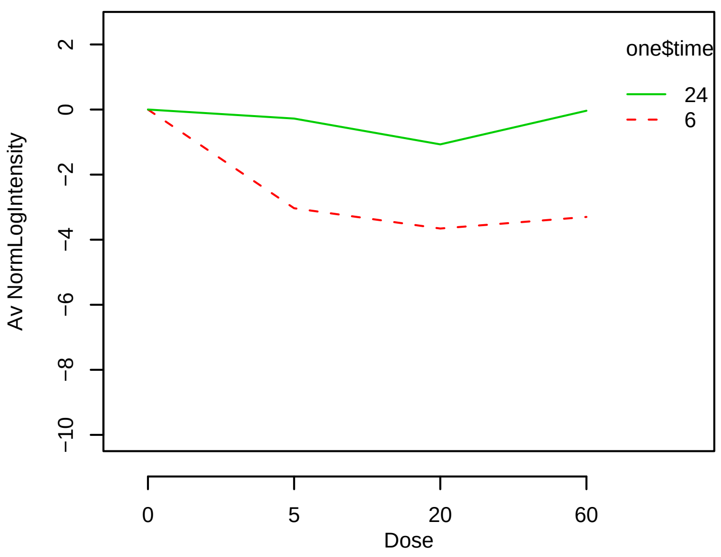

GO_0008016 : regulation of heart contraction

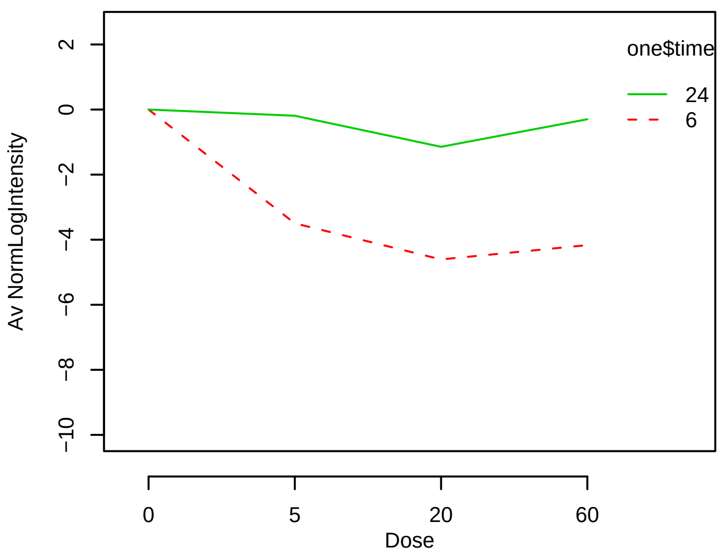

GO_0008033 : tRNA processing

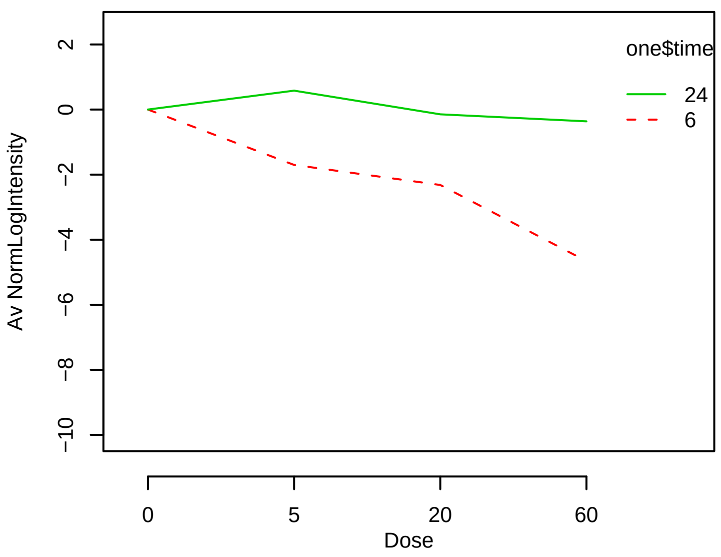

GO_0008037 : cell recognition

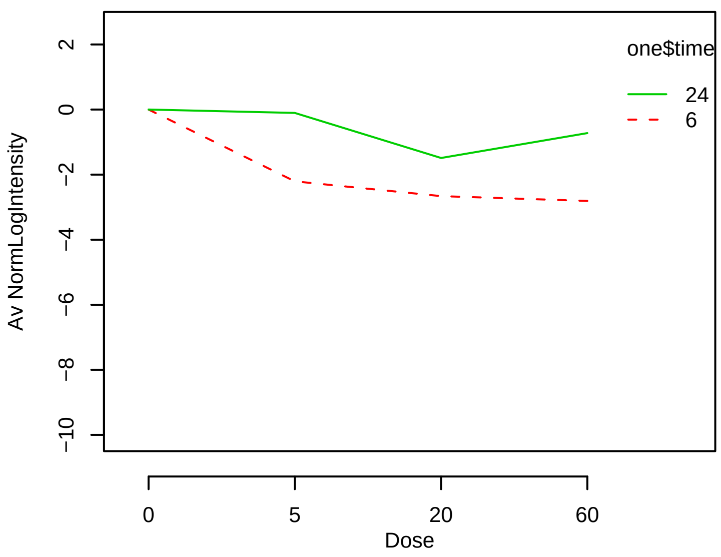

GO_0008038 : neuron recognition

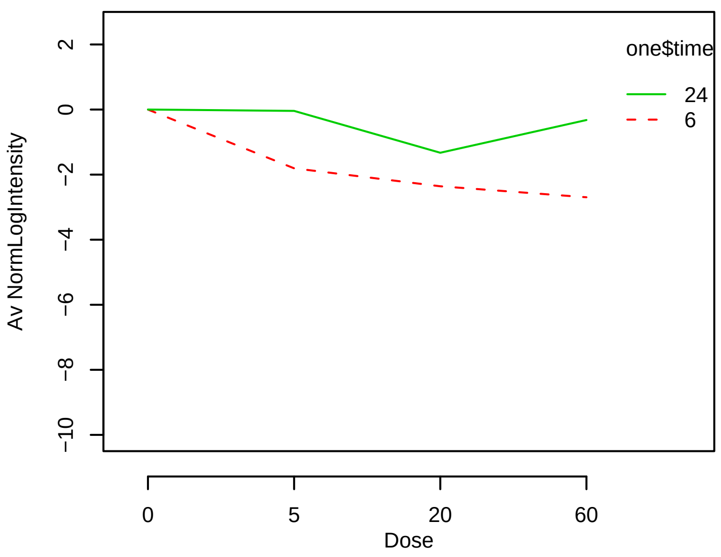

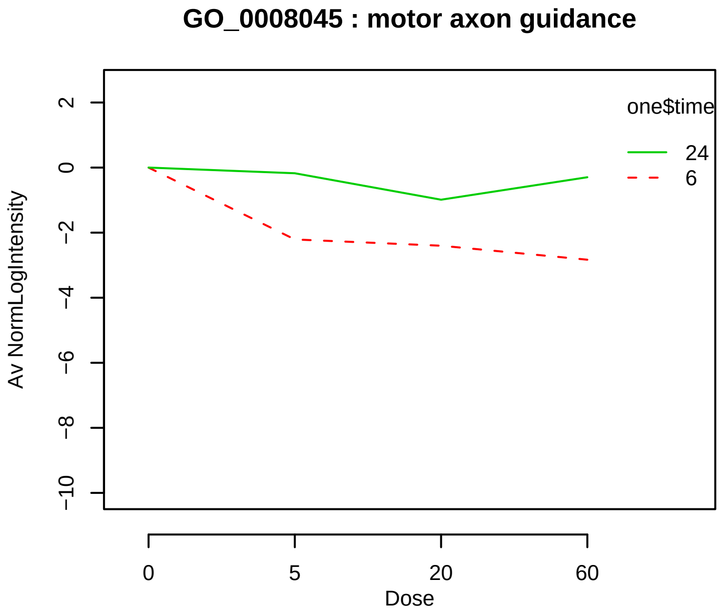
GO_0008045 : motor axon guidance
Av NormLogIntensity
Dose
one$time
24
6
2
0
−2
−4
−6
−8
−10
0
5
20
60

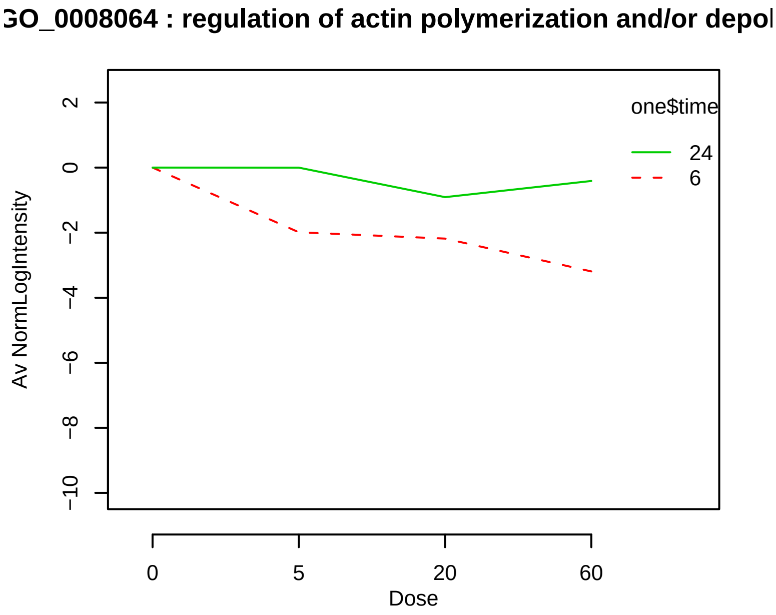
GO_0008064 : regulation of actin polymerization and/or depol
Av NormLogIntensity
Dose
one$time
24
6
2
0
−2
−4
−6
−8
−10
0
5
20
60

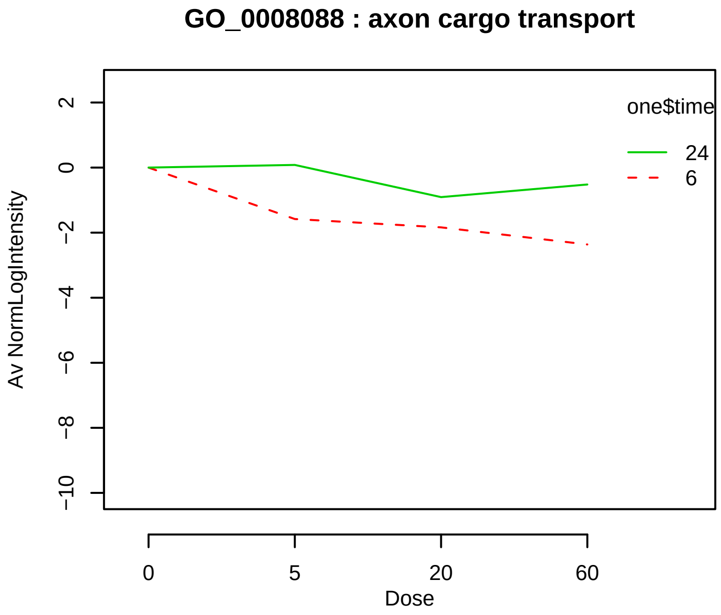
GO_0008088 : axon cargo transport
Av NormLogIntensity
Dose
one$time
24
6
2
0
−2
−4
−6
−8
−10
0
5
20
60

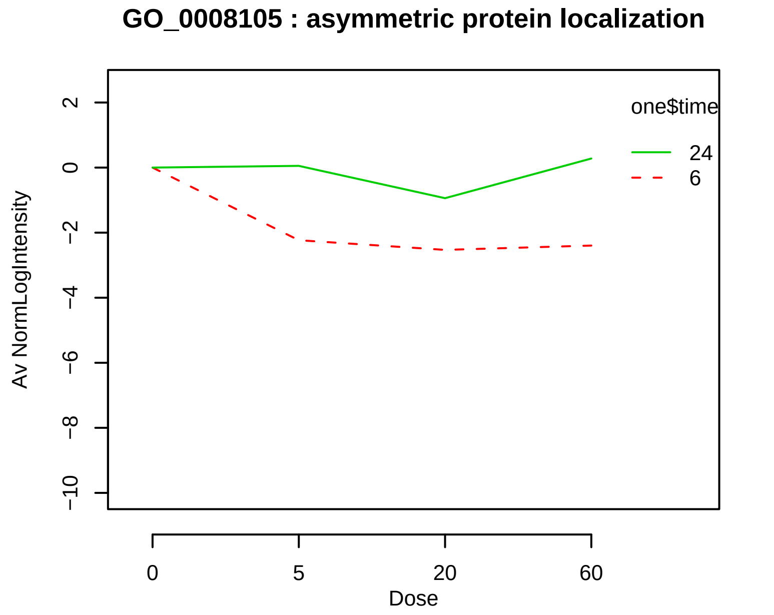
GO_0008105 : asymmetric protein localization
Av NormLogIntensity
Dose
one$time
24
6
2
0
−2
−4
−6
−8
−10
0
5
20
60

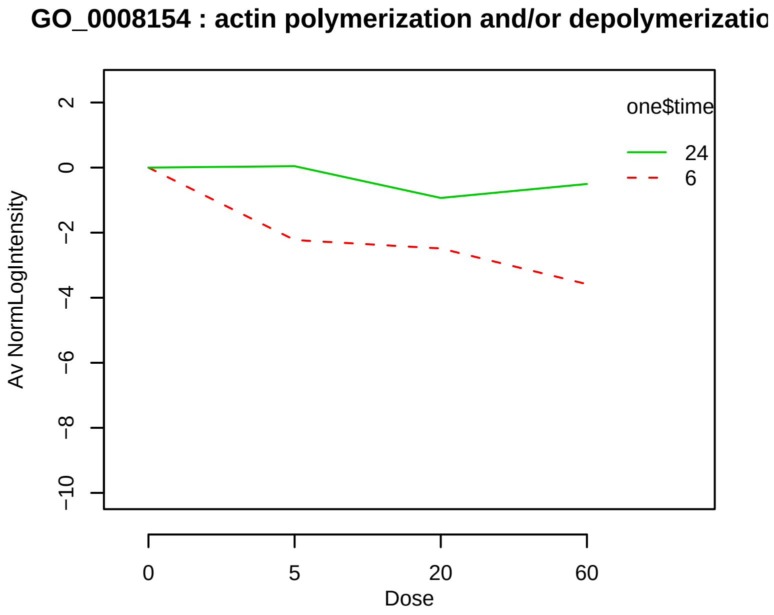
GO_0008154 : actin polymerization and/or depolymerizatio
Av NormLogIntensity
Dose
one$time
24
6
2
0
−2
−4
−6
−8
−10
0
5
20
60

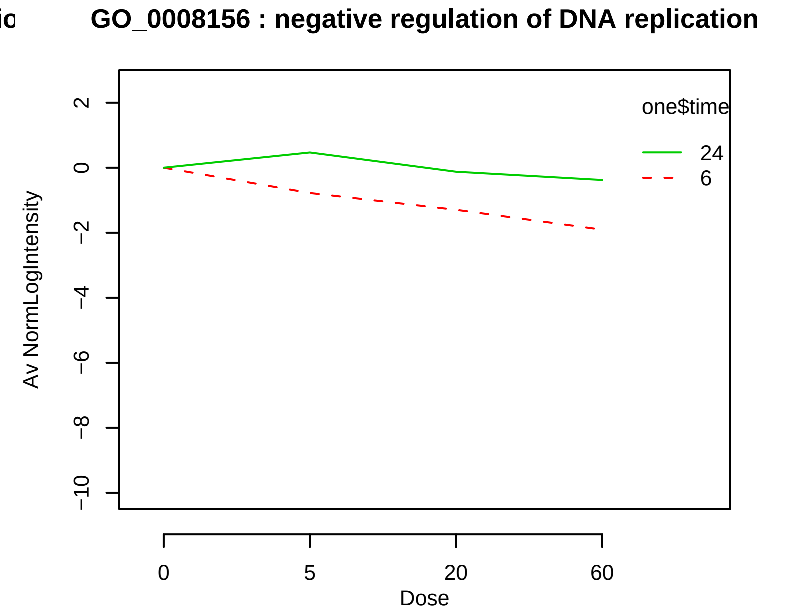
GO_0008156 : negative regulation of DNA replication
Av NormLogIntensity
Dose
one$time
24
6
2
0
−2
−4
−6
−8
−10
0
5
20
60

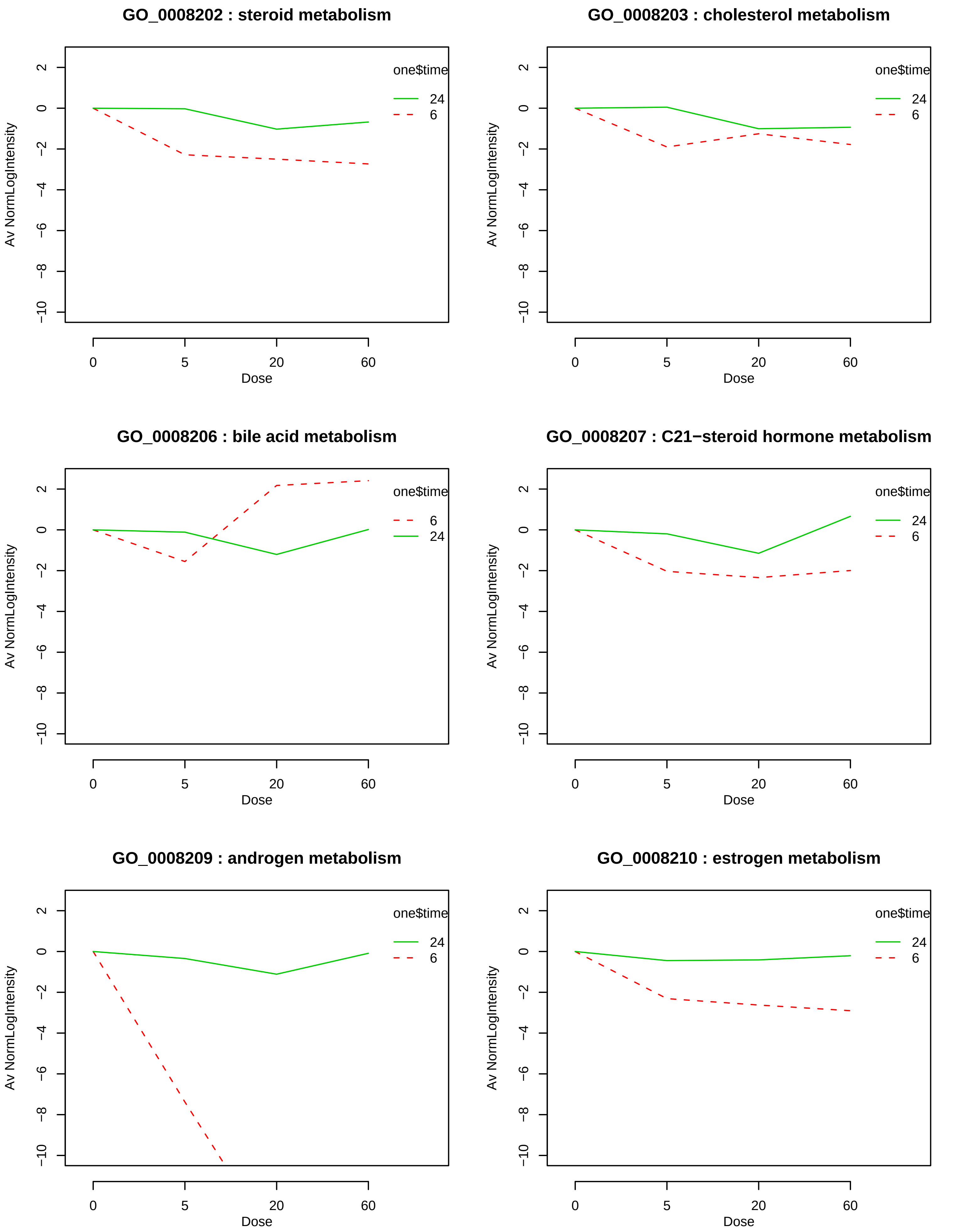
GO_0008202 : steroid metabolism
GO_0008203 : cholesterol metabolism
GO_0008206 : bile acid metabolism
GO_0008207 : C21−steroid hormone metabolism
GO_0008209 : androgen metabolism
GO_0008210 : estrogen metabolism
Av NormLogIntensity
Dose
0
5
20
60
2
0
−2
−4
−6
−8
−10
one$time
24
6

GO_0008213 : protein amino acid alkylation

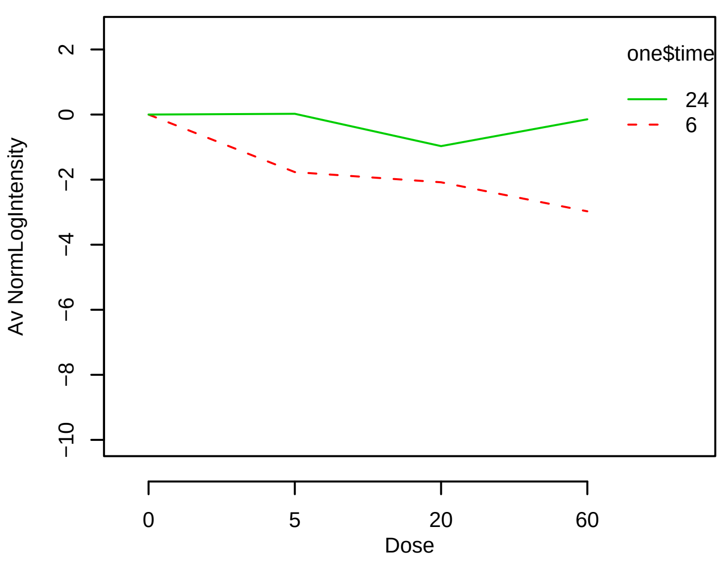

GO_0008216 : spermidine metabolism

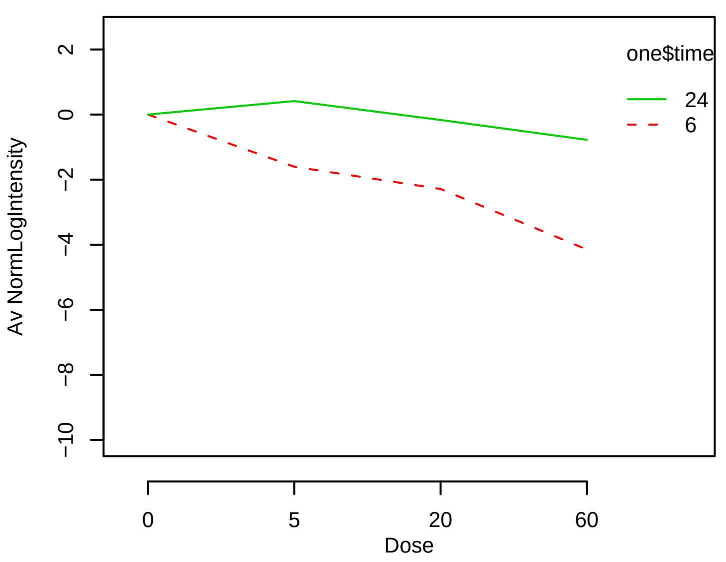

GO_0008217 : blood pressure regulation

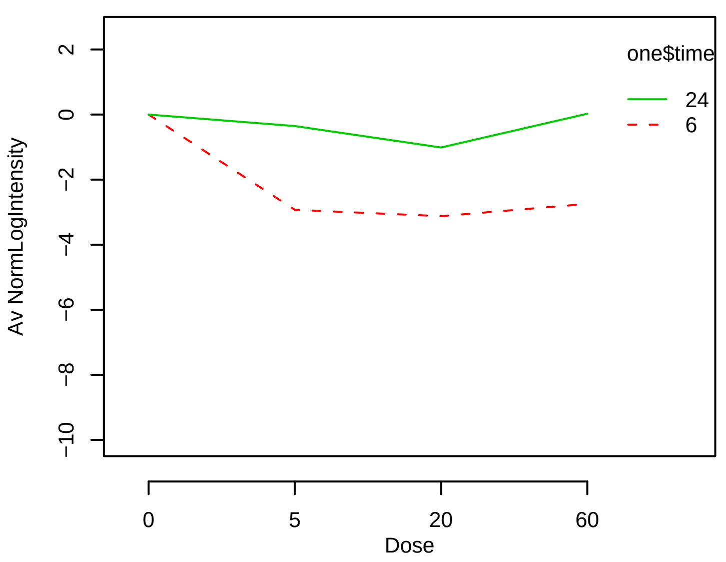

GO_0008272 : sulfate transport

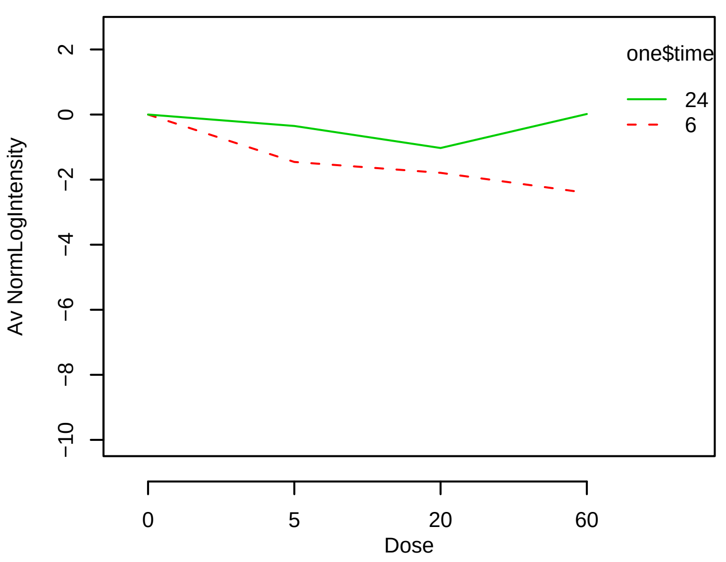

GO_0008277 : regulation of G−protein coupled receptor prote

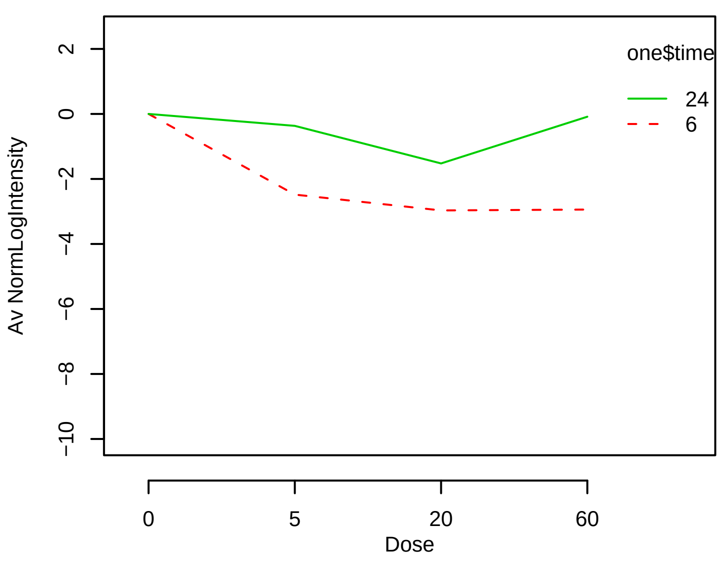

GO_0008284 : positive regulation of cell proliferation

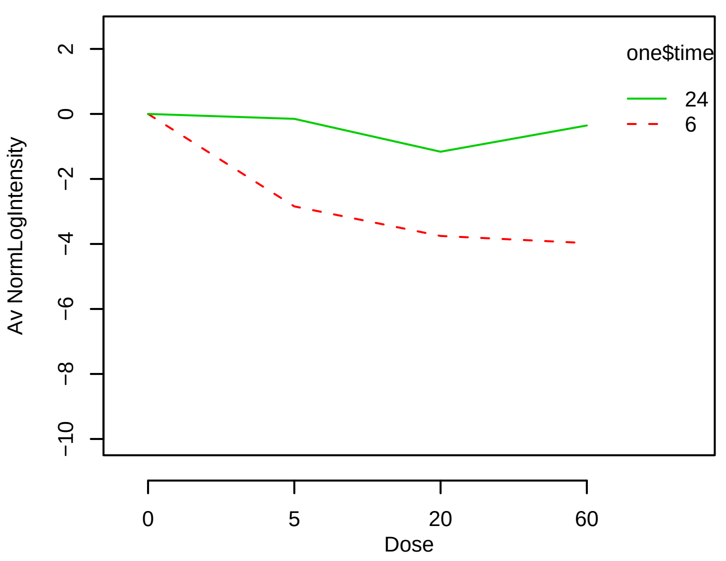

GO_0008285 : negative regulation of cell proliferation

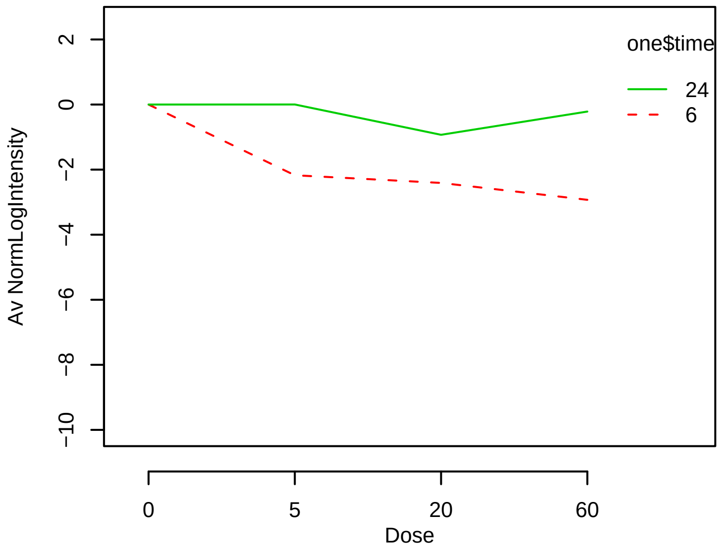

GO_0008286 : insulin receptor signaling pathway

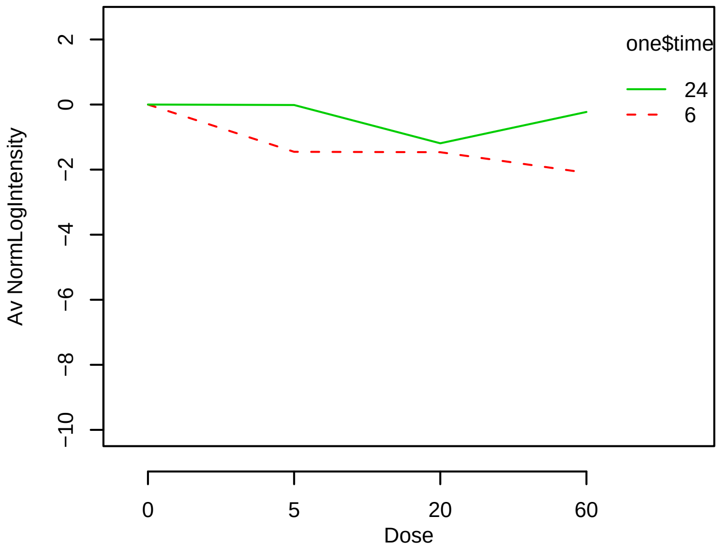

GO_0008299 : isoprenoid biosynthesis

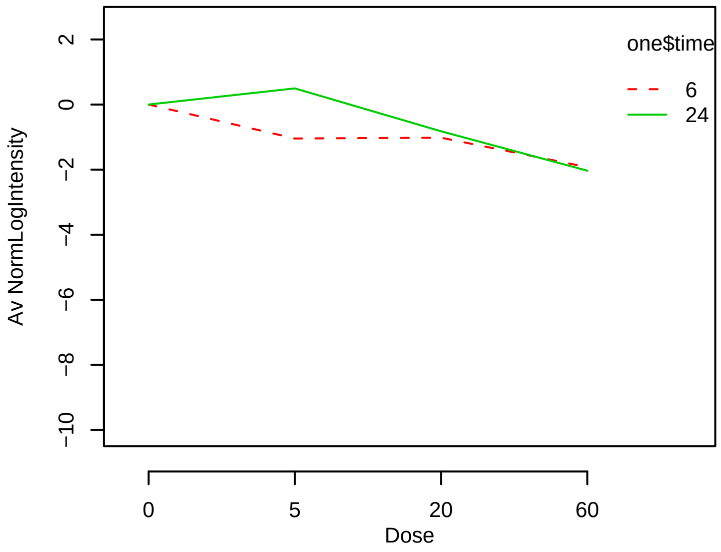

GO_0008306 : associative learning

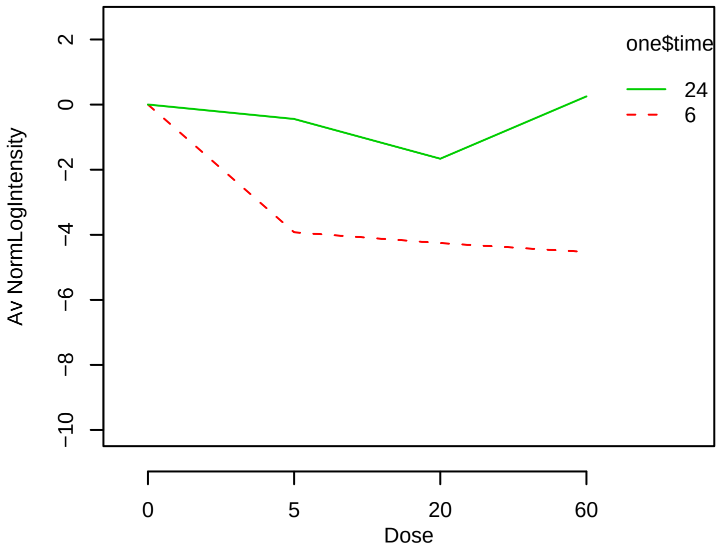

GO_0008333 : endosome to lysosome transport

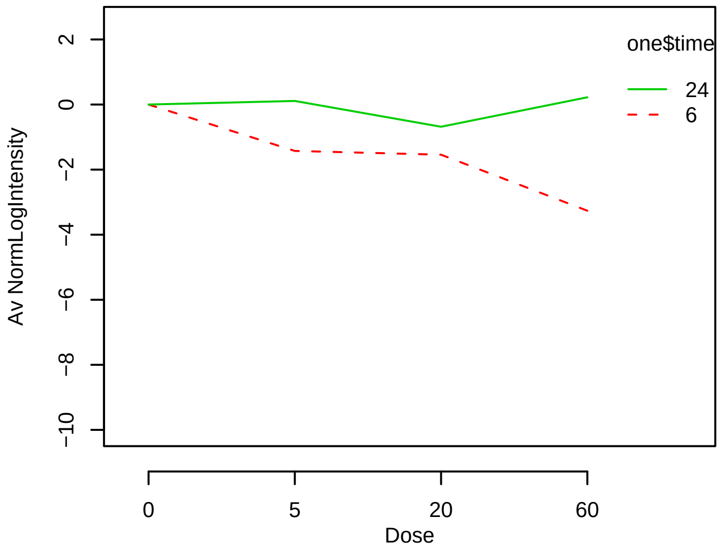

GO_0008344 : adult locomotory behavior

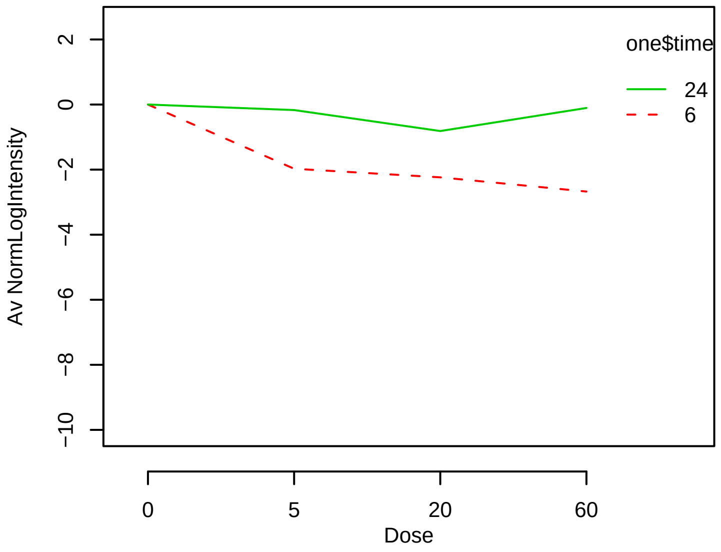

GO_0008354 : germ cell migration

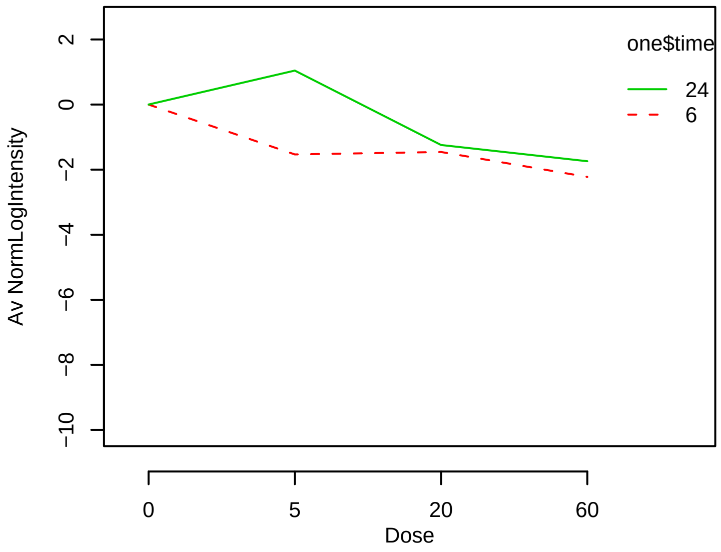

GO_0008360 : regulation of cell shape

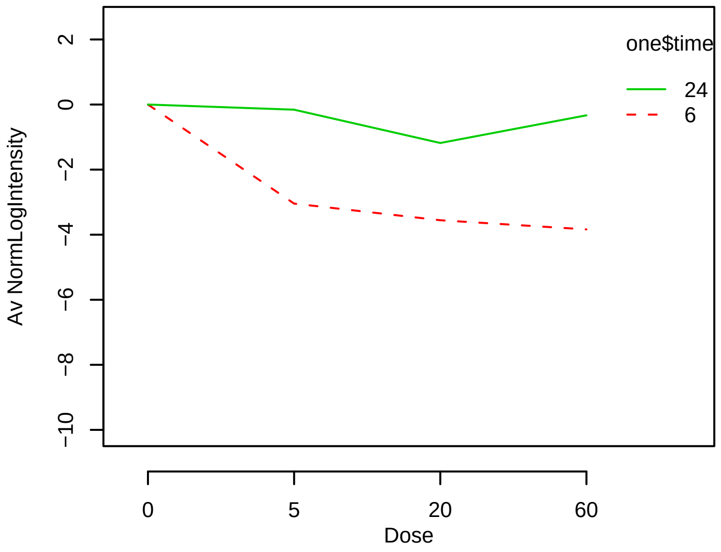

GO_0008361 : regulation of cell size

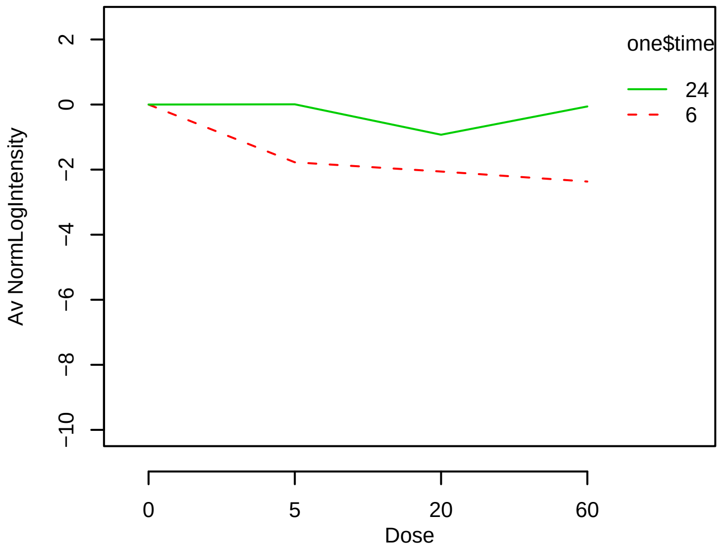

GO_0008366 : axon ensheathment

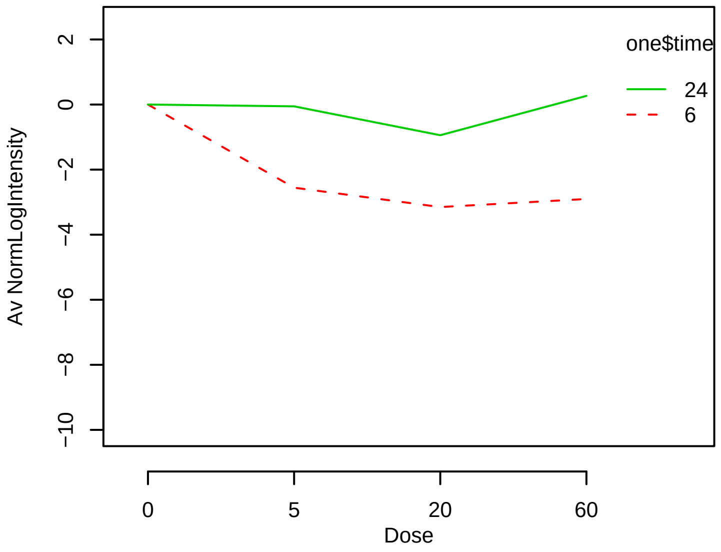

GO_0008380 : RNA splicing

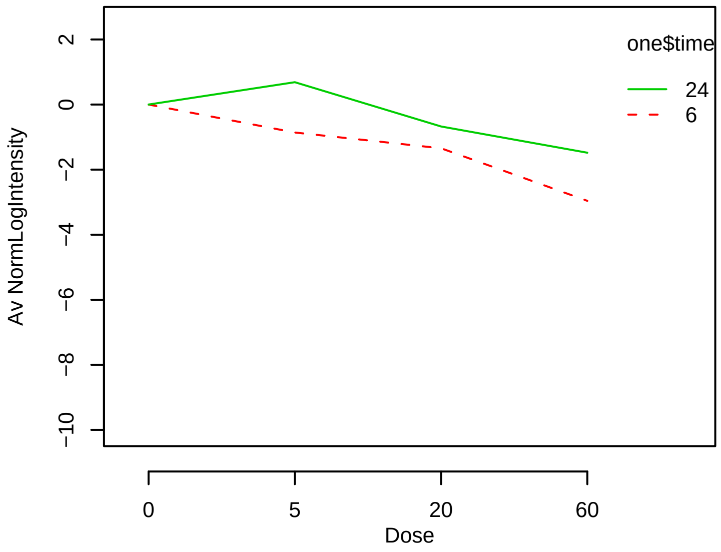

GO_0008406 : gonad development

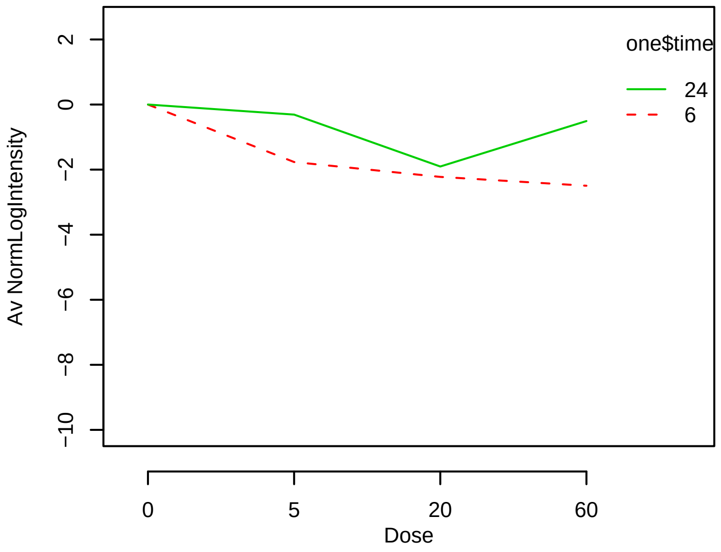

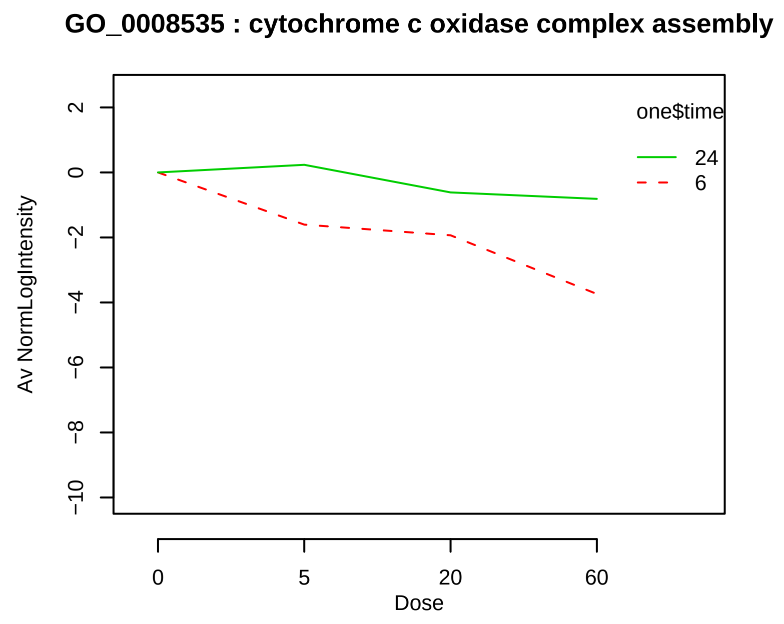
GO_0008535 : cytochrome c oxidase complex assembly
one$time
24
6
Av NormLogIntensity
2
0
−2
−4
−6
−8
−10
0
5
20
60
Dose

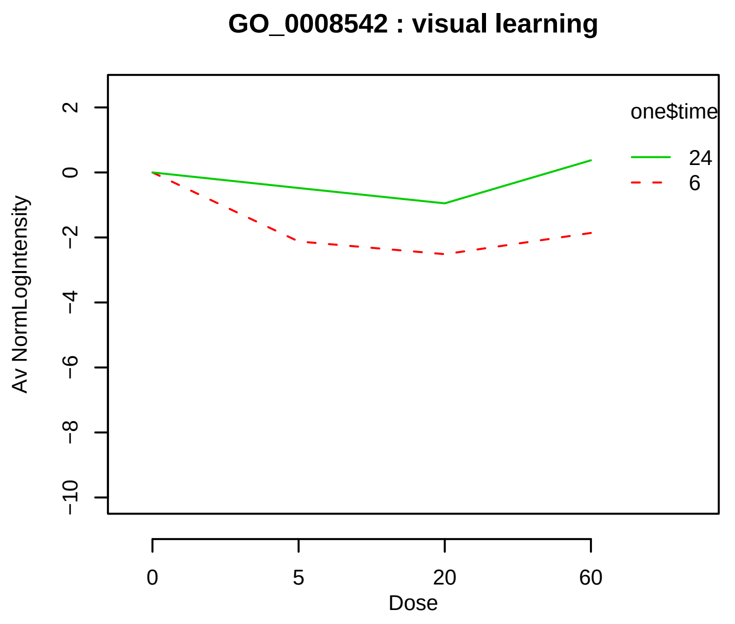
GO_0008542 : visual learning
one$time
24
6
Av NormLogIntensity
2
0
−2
−4
−6
−8
−10
0
5
20
60
Dose

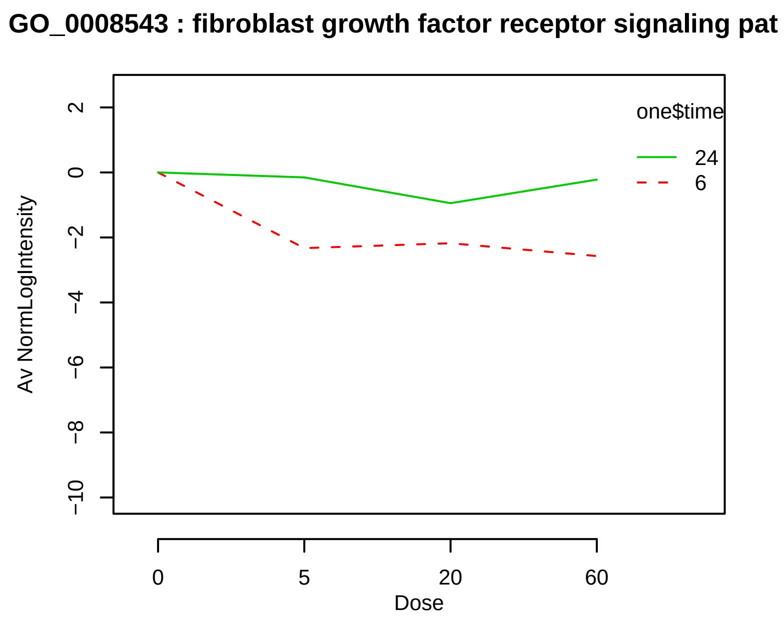
GO_0008543 : fibroblast growth factor receptor signaling pat
one$time
24
6
Av NormLogIntensity
2
0
−2
−4
−6
−8
−10
0
5
20
60
Dose

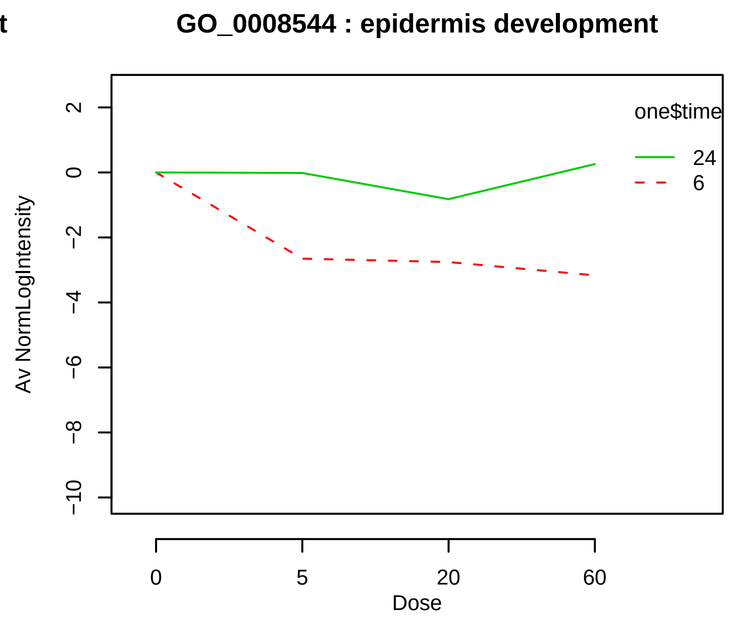
GO_0008544 : epidermis development
one$time
24
6
Av NormLogIntensity
2
0
−2
−4
−6
−8
−10
0
5
20
60
Dose

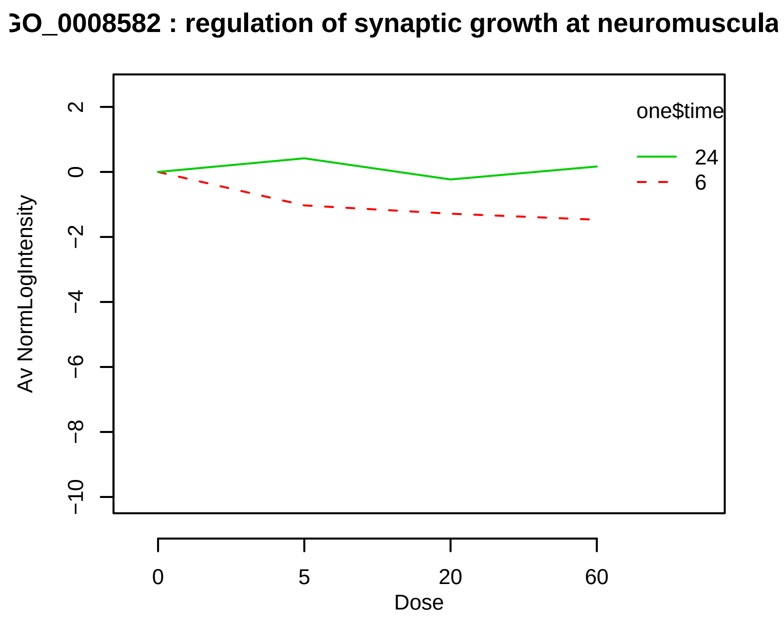
GO_0008582 : regulation of synaptic growth at neuromuscula
one$time
24
6
Av NormLogIntensity
2
0
−2
−4
−6
−8
−10
0
5
20
60
Dose

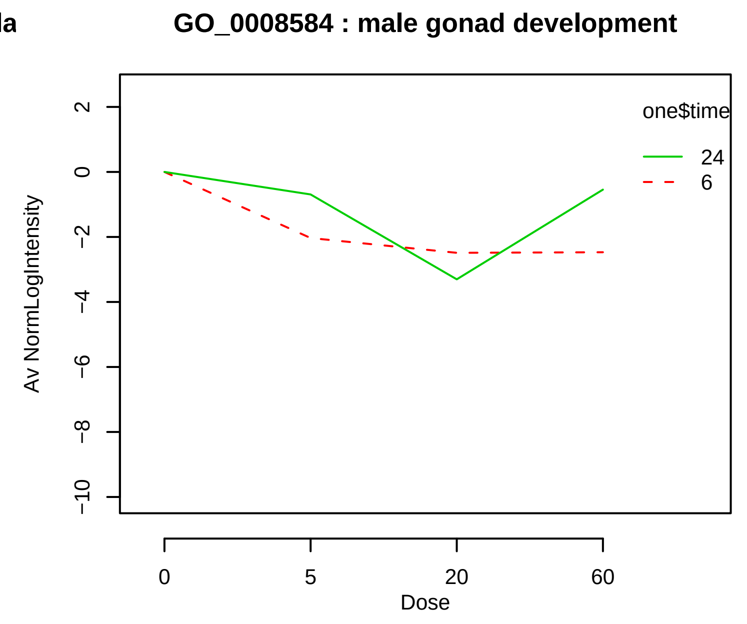
GO_0008584 : male gonad development
one$time
24
6
Av NormLogIntensity
2
0
−2
−4
−6
−8
−10
0
5
20
60
Dose

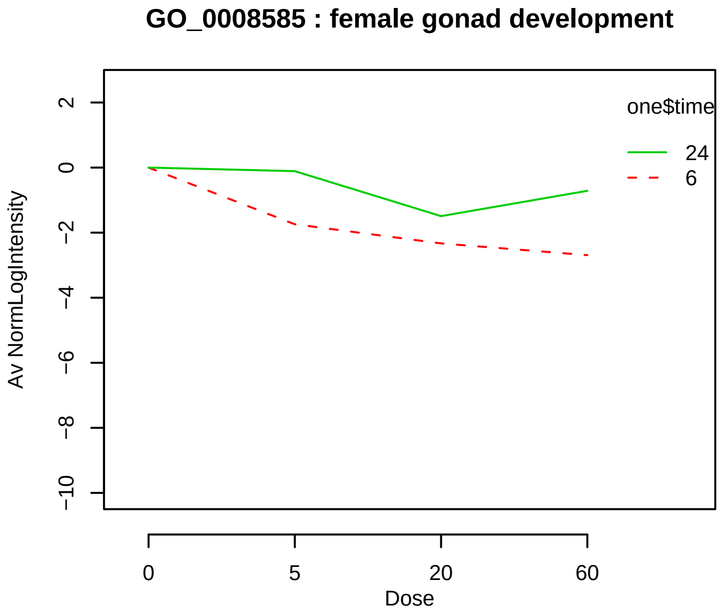
GO_0008585 : female gonad development
one$time
24
6
Av NormLogIntensity
2
0
−2
−4
−6
−8
−10
0
5
20
60
Dose

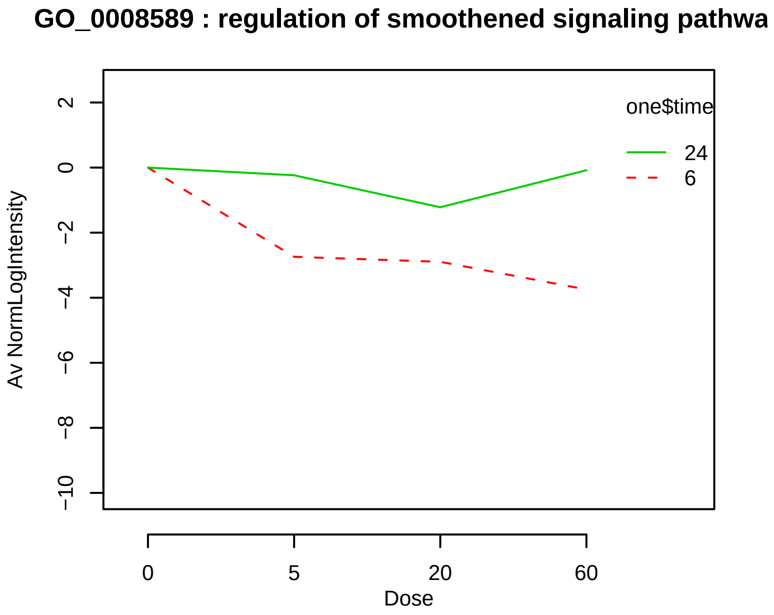
GO_0008589 : regulation of smoothened signaling pathwa
one$time
24
6
Av NormLogIntensity
2
0
−2
−4
−6
−8
−10
0
5
20
60
Dose

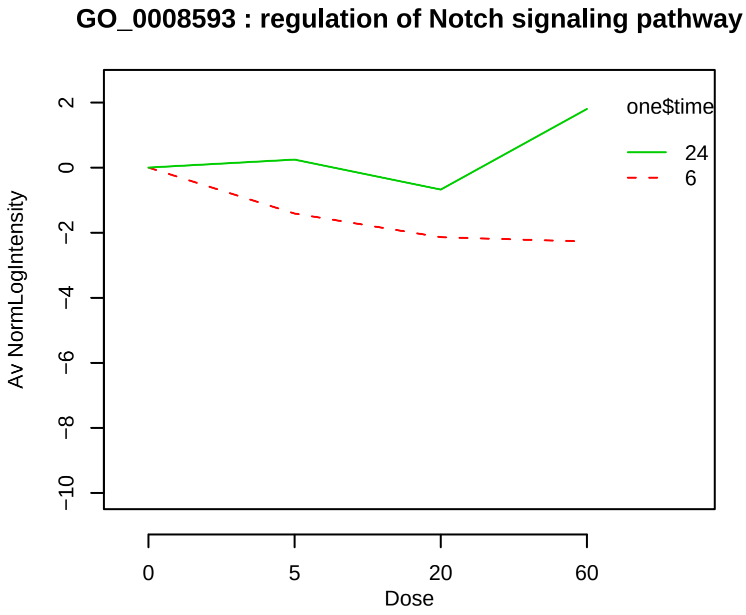
GO_0008593 : regulation of Notch signaling pathway
one$time
24
6
Av NormLogIntensity
2
0
−2
−4
−6
−8
−10
0
5
20
60
Dose

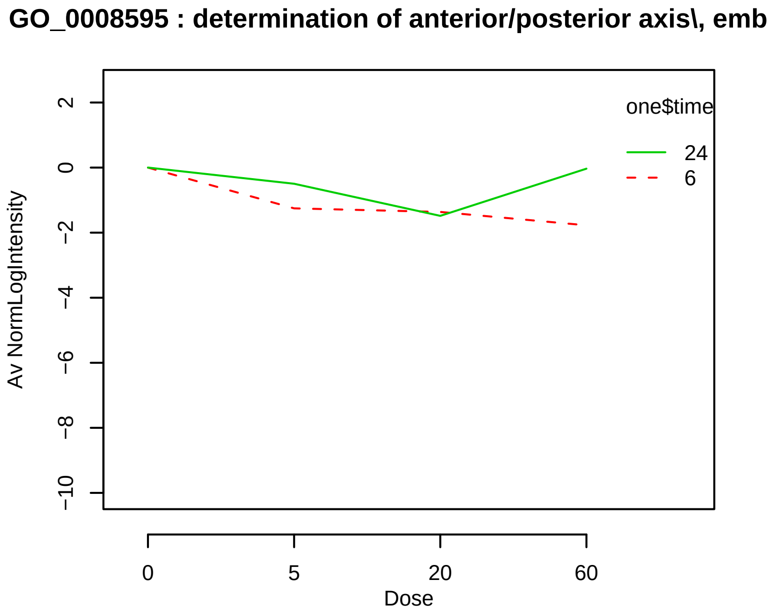
GO_0008595 : determination of anterior/posterior axis\, emb
one$time
24
6
Av NormLogIntensity
2
0
−2
−4
−6
−8
−10
0
5
20
60
Dose

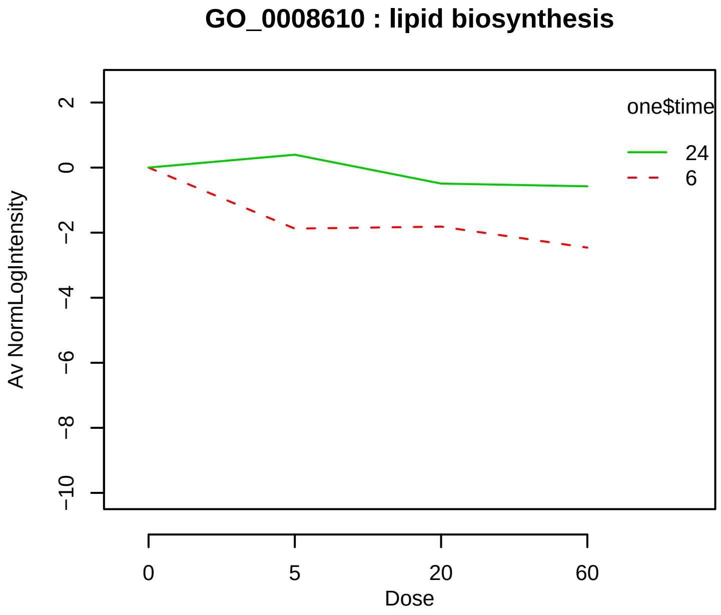
GO_0008610 : lipid biosynthesis
one$time
24
6
Av NormLogIntensity
2
0
−2
−4
−6
−8
−10
0
5
20
60
Dose

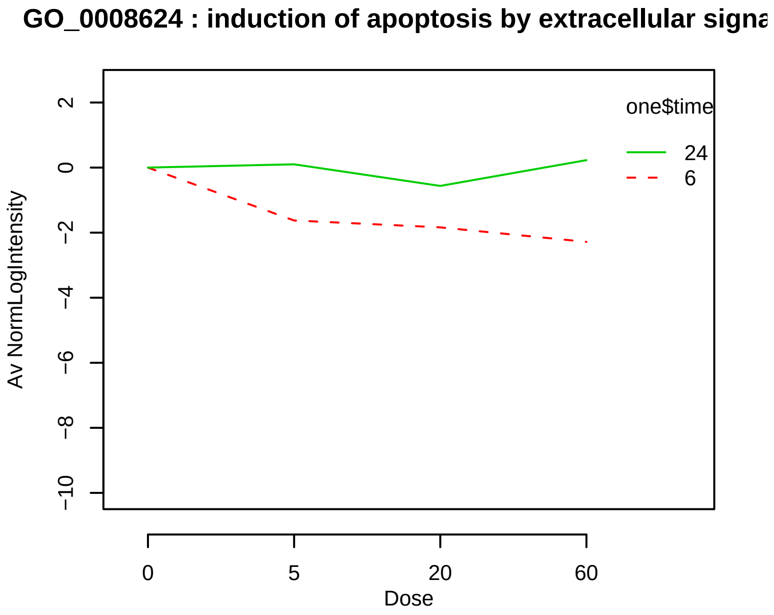
GO_0008624 : induction of apoptosis by extracellular signa
one$time
24
6
Av NormLogIntensity
2
0
−2
−4
−6
−8
−10
0
5
20
60
Dose

GO_0008625 : induction of apoptosis via death domain recep

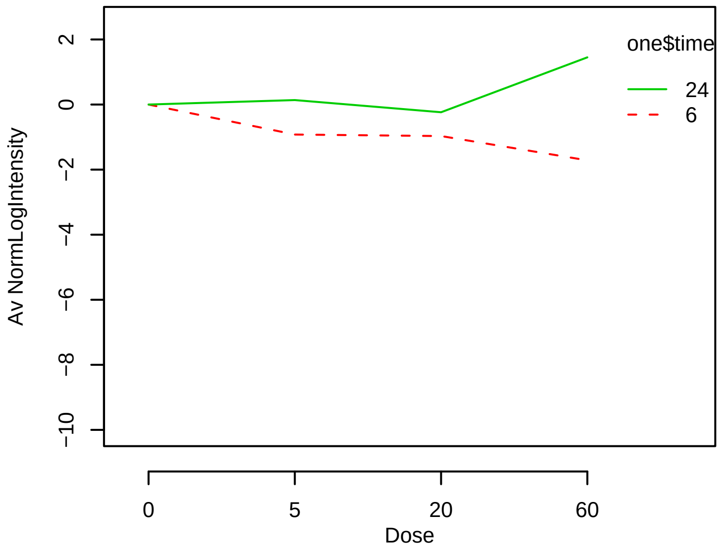

GO_0008629 : induction of apoptosis by intracellular signa

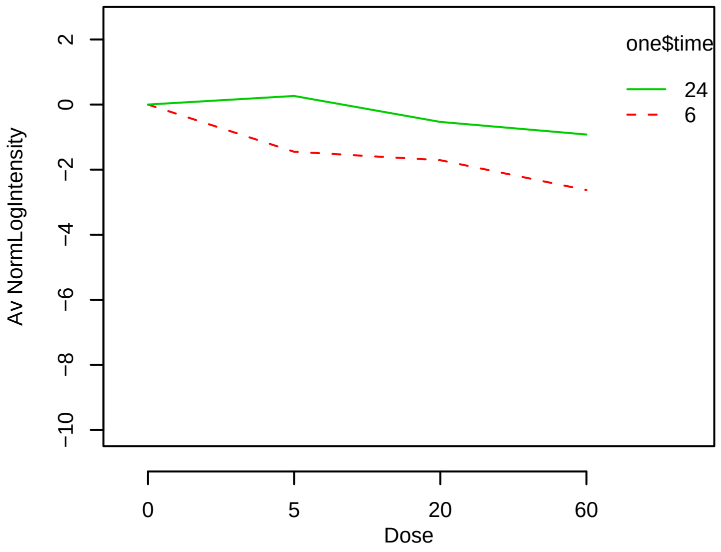

GO_0008630 : DNA damage response\, signal transduction re

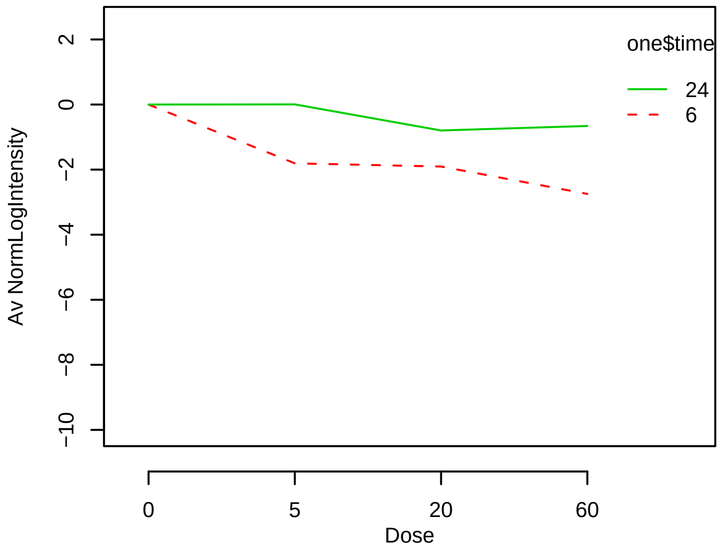

GO_0008631 : induction of apoptosis by oxidative stress

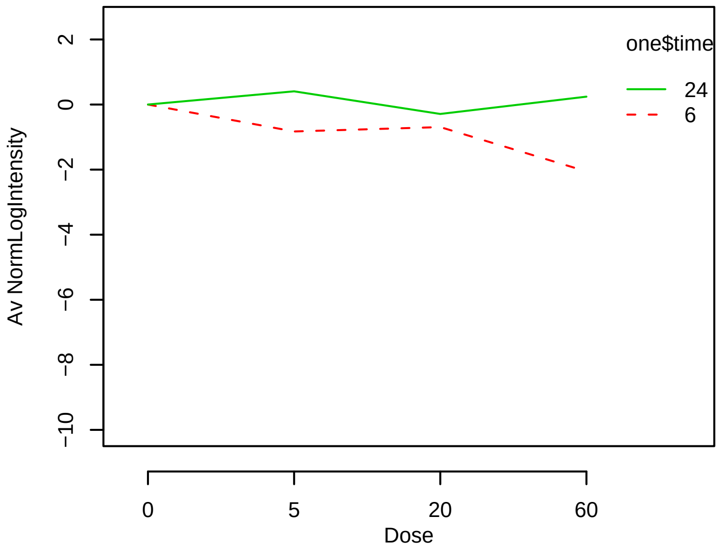

GO_0008632 : apoptotic program

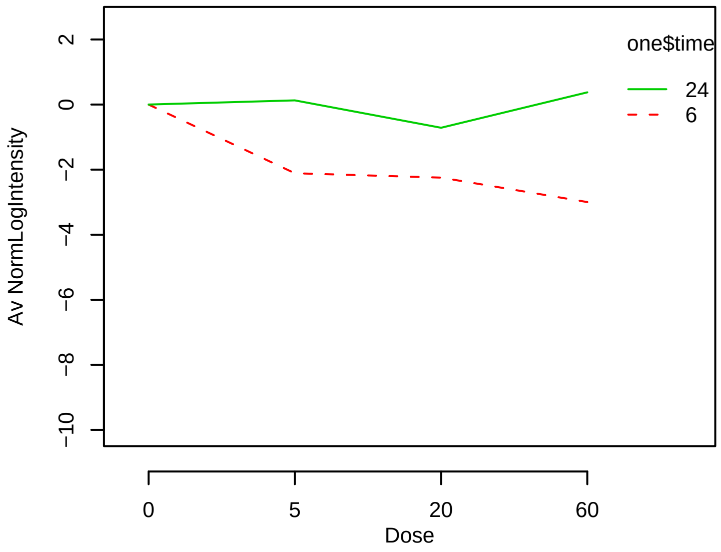

GO_0008634 : negative regulation of survival gene product a

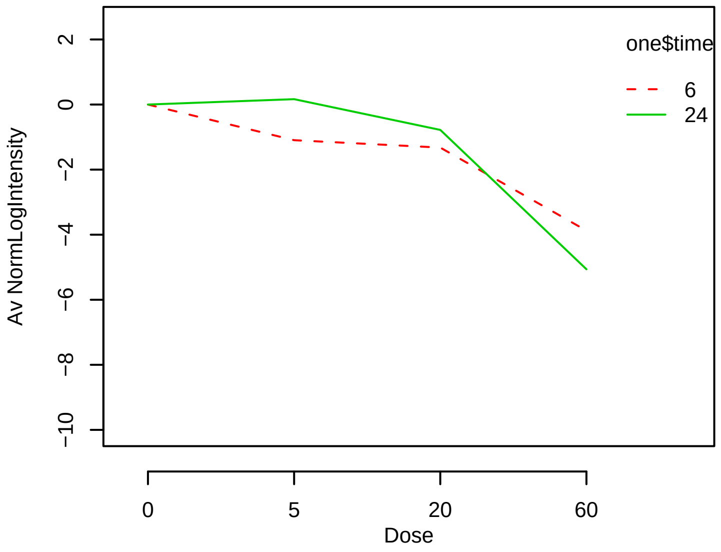

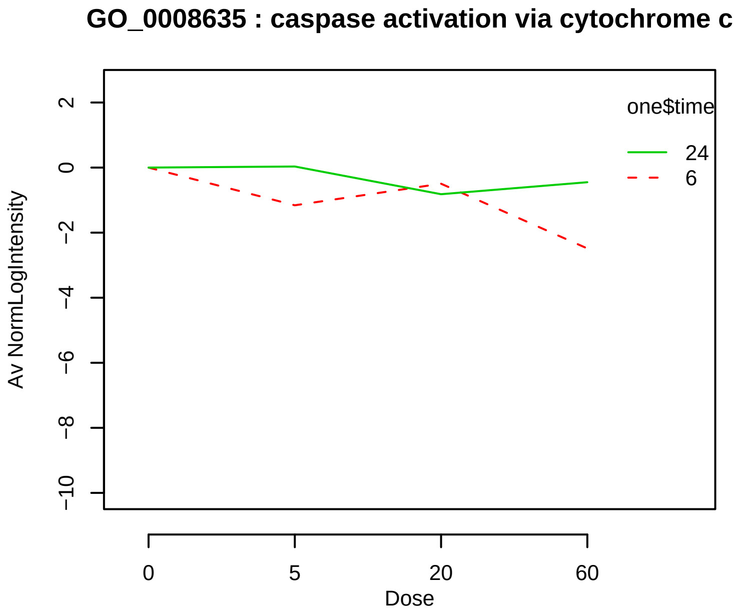
GO_0008635 : caspase activation via cytochrome c
one$time
24
6
Av NormLogIntensity
Dose

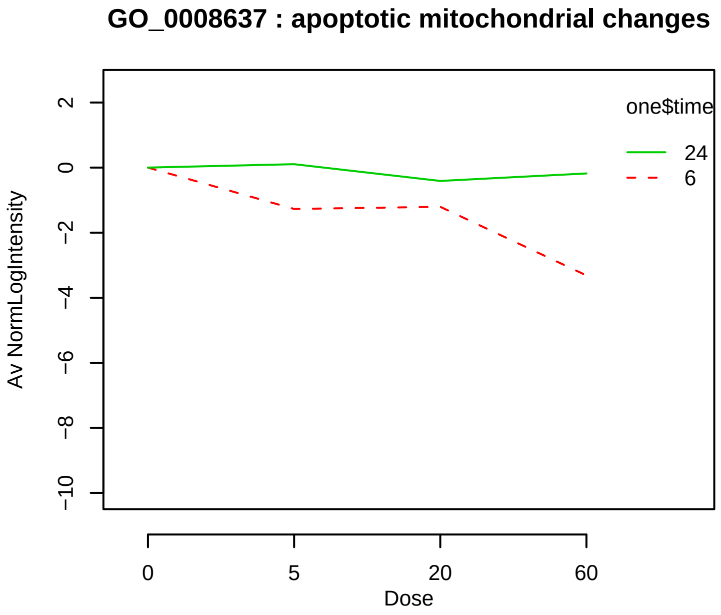
GO_0008637 : apoptotic mitochondrial changes
one$time
24
6
Av NormLogIntensity
Dose

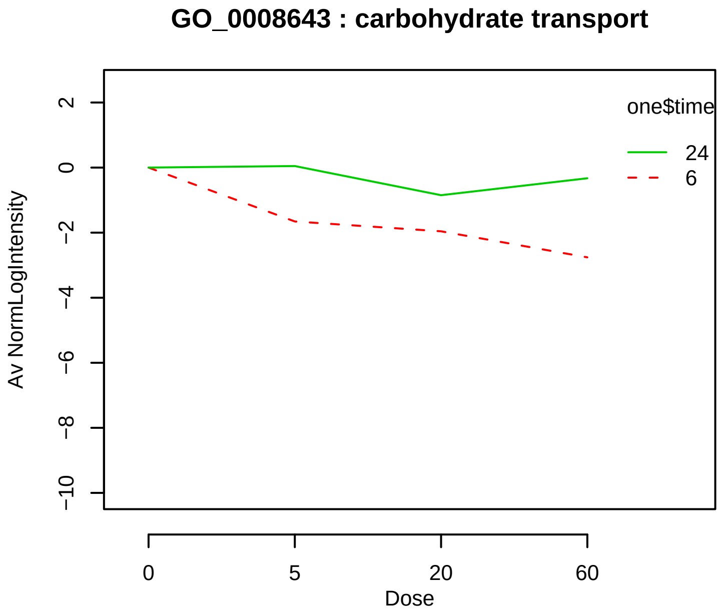
GO_0008643 : carbohydrate transport
one$time
24
6
Av NormLogIntensity
Dose

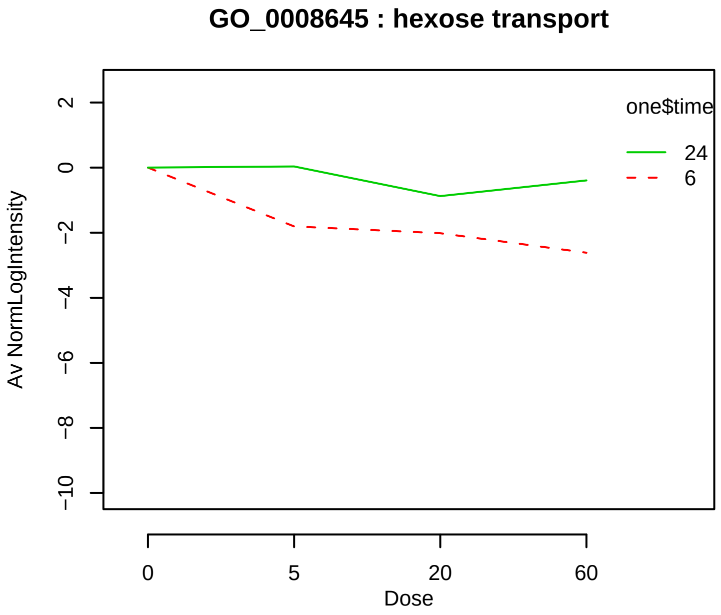
GO_0008645 : hexose transport
one$time
24
6
Av NormLogIntensity
Dose

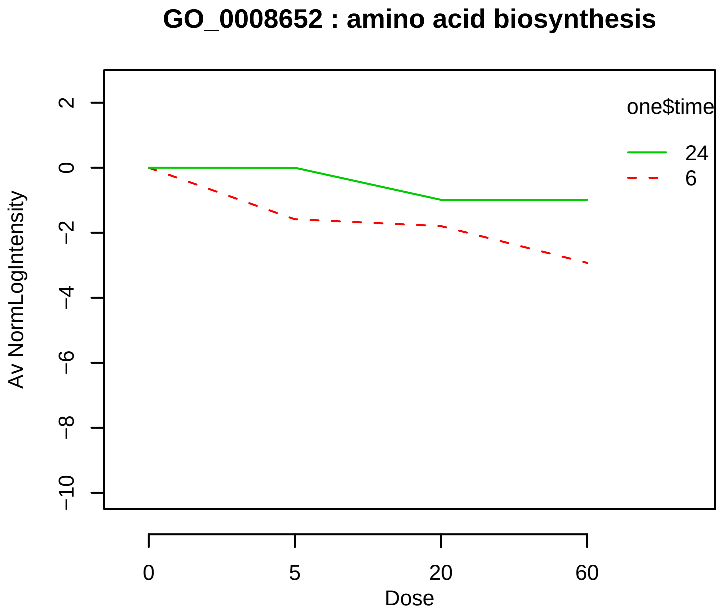
GO_0008652 : amino acid biosynthesis
one$time
24
6
Av NormLogIntensity
Dose

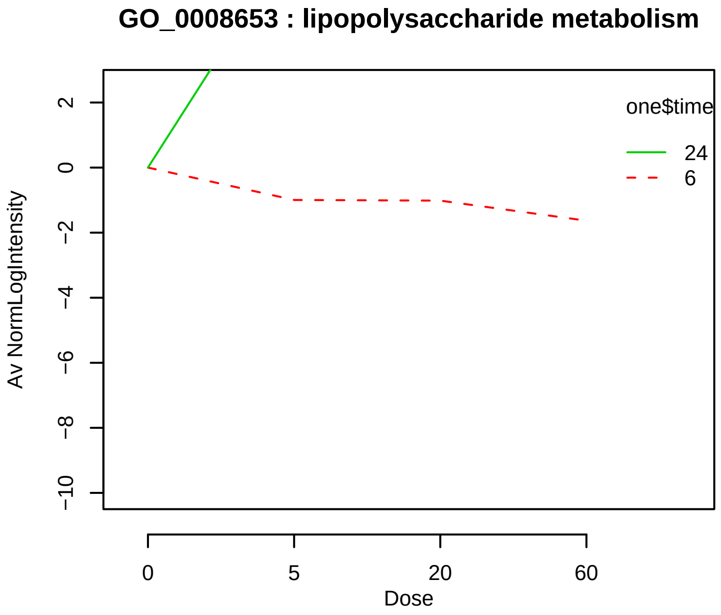
GO_0008653 : lipopolysaccharide metabolism
one$time
24
6
Av NormLogIntensity
Dose

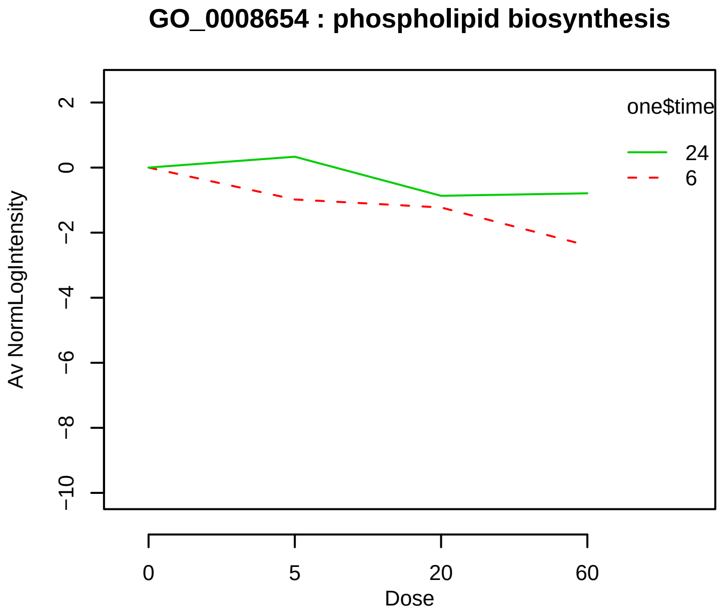
GO_0008654 : phospholipid biosynthesis
one$time
24
6
Av NormLogIntensity
Dose
0
5
20
60

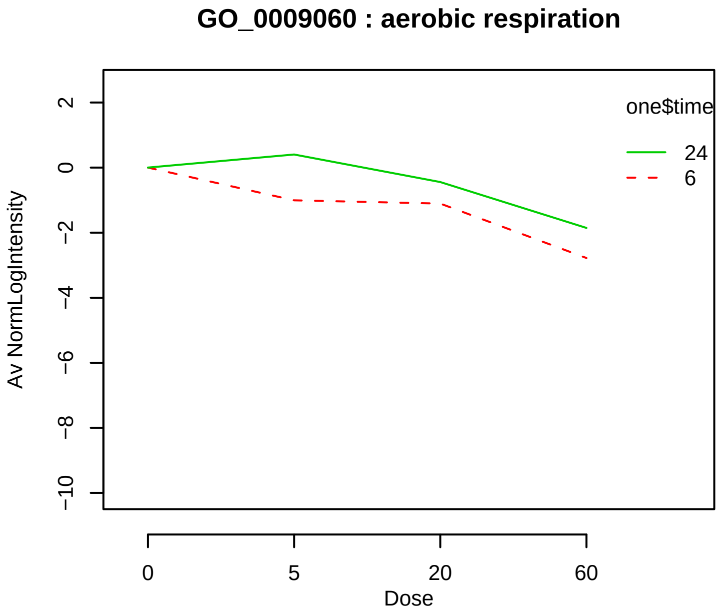
GO_0009060 : aerobic respiration
one$time
24
6
Av NormLogIntensity
Dose
0
5
20
60

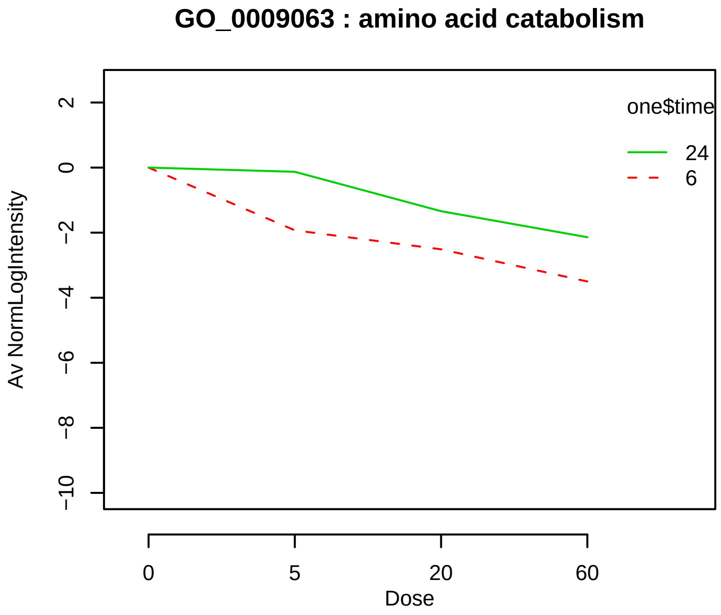
GO_0009063 : amino acid catabolism
one$time
24
6
Av NormLogIntensity
Dose
0
5
20
60

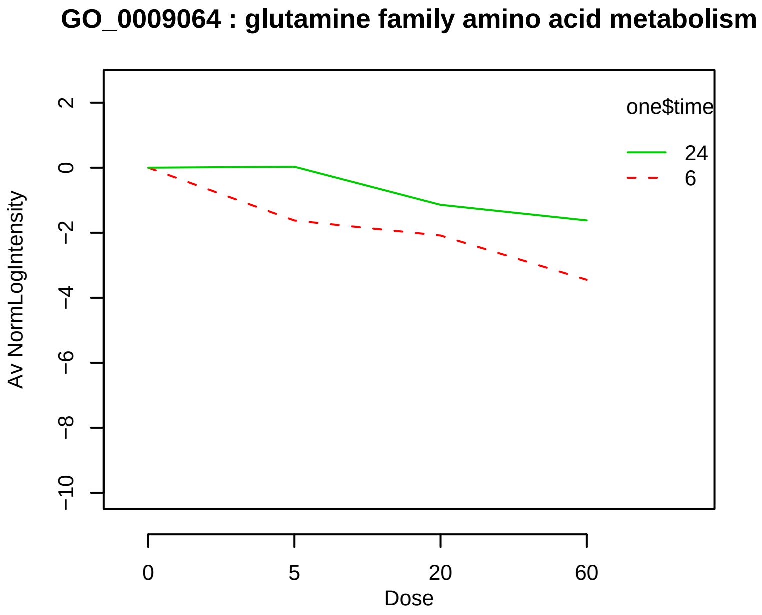
GO_0009064 : glutamine family amino acid metabolism
one$time
24
6
Av NormLogIntensity
Dose
0
5
20
60

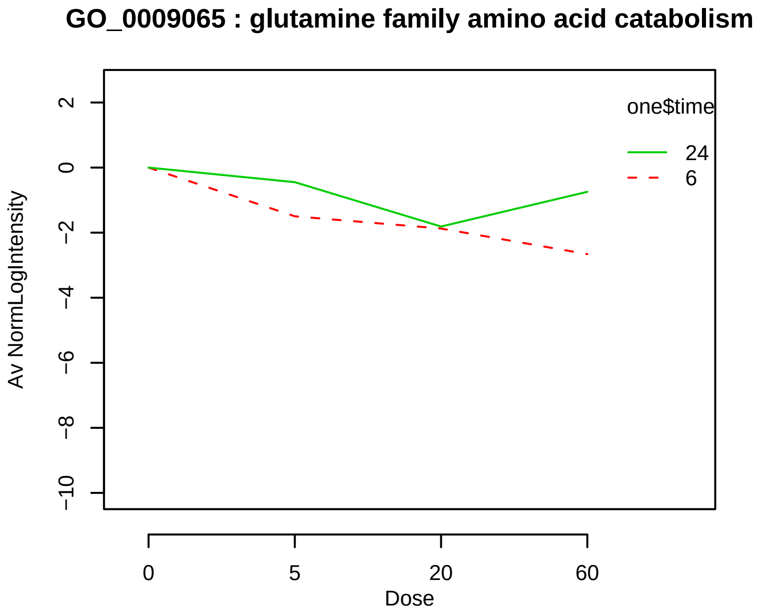
GO_0009065 : glutamine family amino acid catabolism
one$time
24
6
Av NormLogIntensity
Dose
0
5
20
60

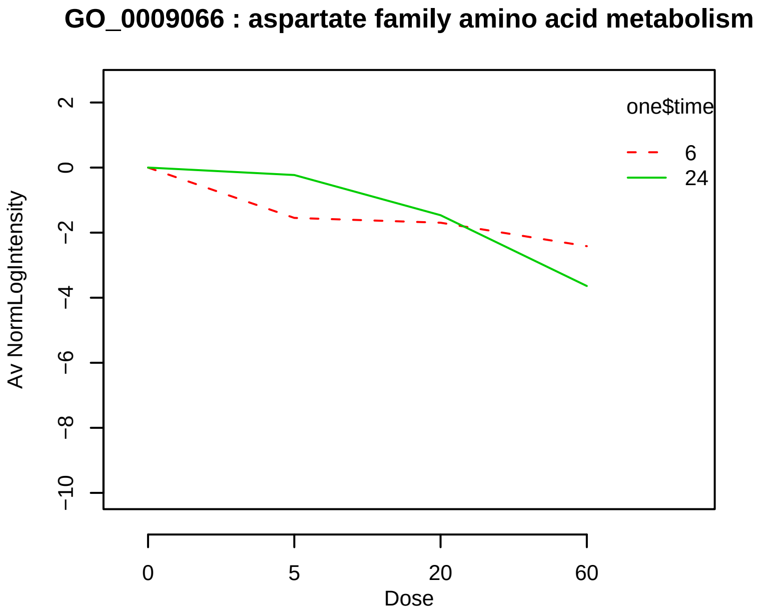
GO_0009066 : aspartate family amino acid metabolism
one$time
6
24
Av NormLogIntensity
Dose
0
5
20
60

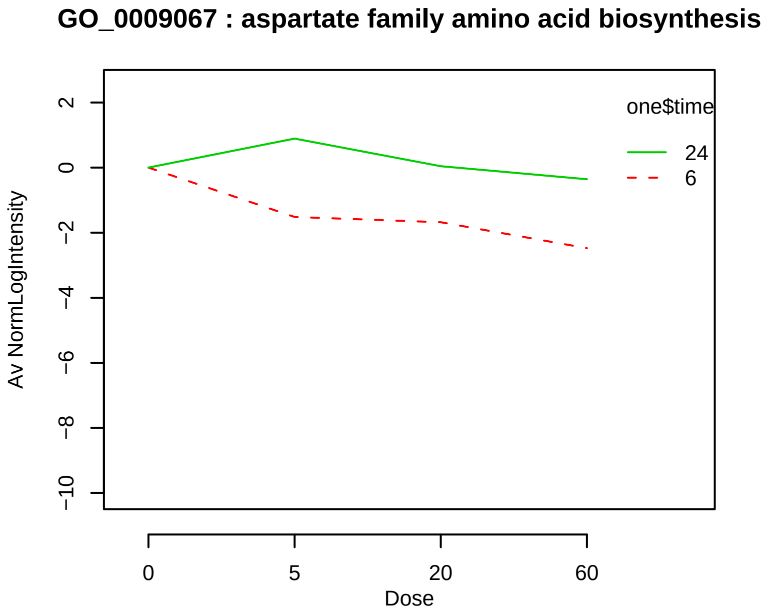
GO_0009067 : aspartate family amino acid biosynthesis
one$time
24
6
Av NormLogIntensity
2
0
−2
−4
−6
−8
−10
0
5
20
60
Dose

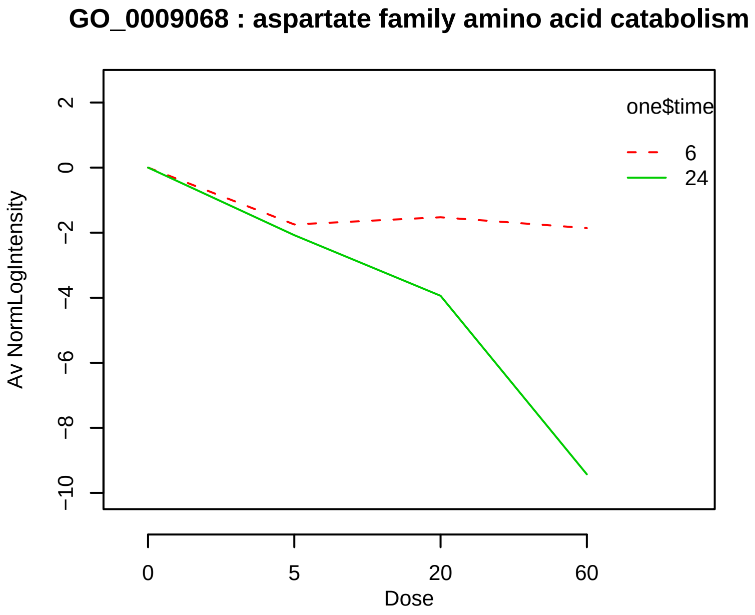
GO_0009068 : aspartate family amino acid catabolism
one$time
6
24
Av NormLogIntensity
2
0
−2
−4
−6
−8
−10
0
5
20
60
Dose

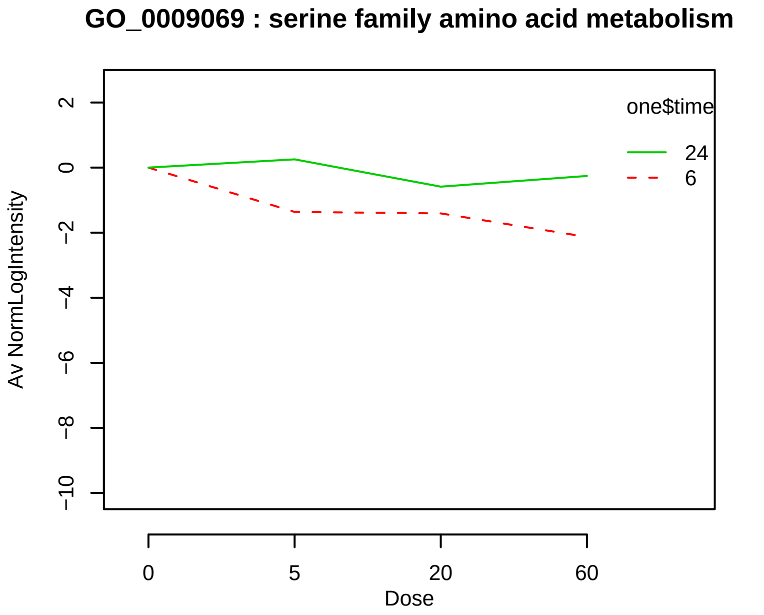
GO_0009069 : serine family amino acid metabolism
one$time
24
6
Av NormLogIntensity
2
0
−2
−4
−6
−8
−10
0
5
20
60
Dose

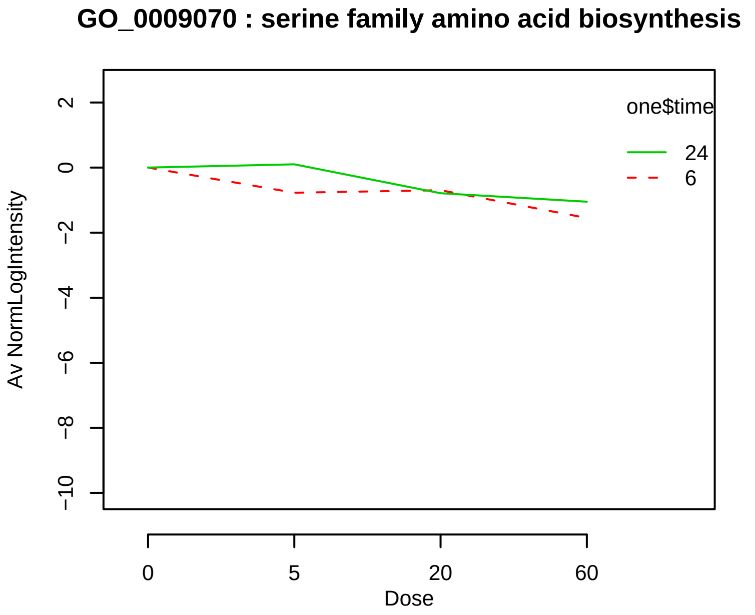
GO_0009070 : serine family amino acid biosynthesis
one$time
24
6
Av NormLogIntensity
2
0
−2
−4
−6
−8
−10
0
5
20
60
Dose

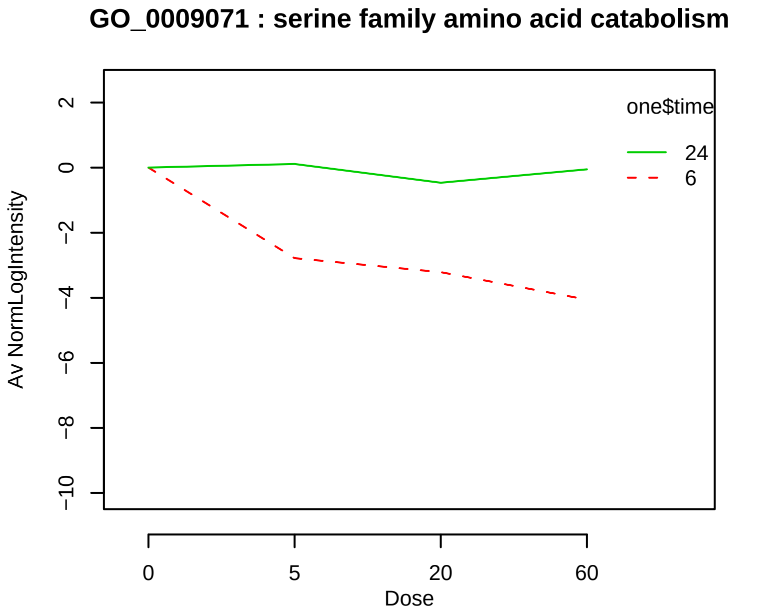
GO_0009071 : serine family amino acid catabolism
one$time
24
6
Av NormLogIntensity
2
0
−2
−4
−6
−8
−10
0
5
20
60
Dose

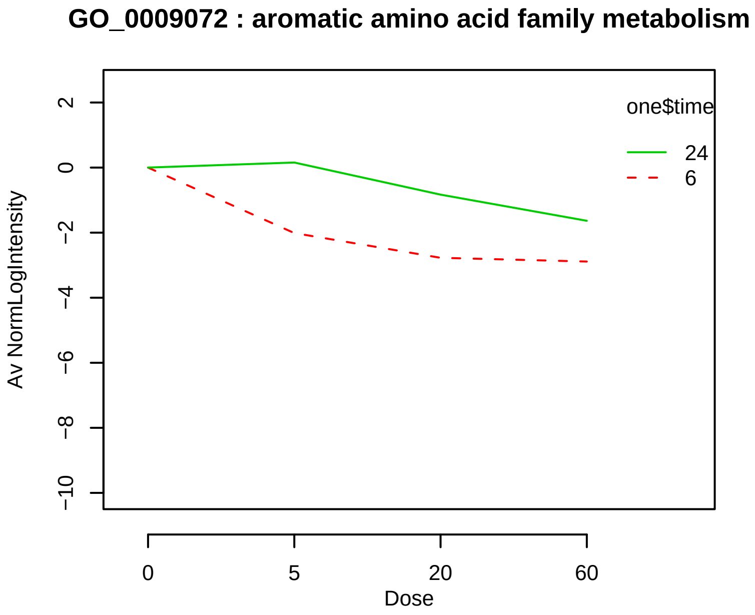
GO_0009072 : aromatic amino acid family metabolism
one$time
24
6
Av NormLogIntensity
2
0
−2
−4
−6
−8
−10
0
5
20
60
Dose

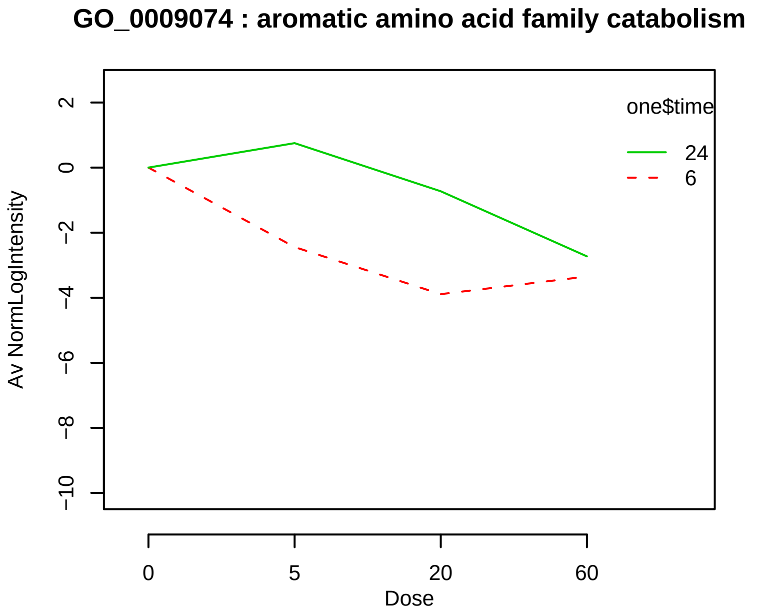
GO_0009074 : aromatic amino acid family catabolism
one$time
24
6
Av NormLogIntensity
2
0
−2
−4
−6
−8
−10
0
5
20
60
Dose

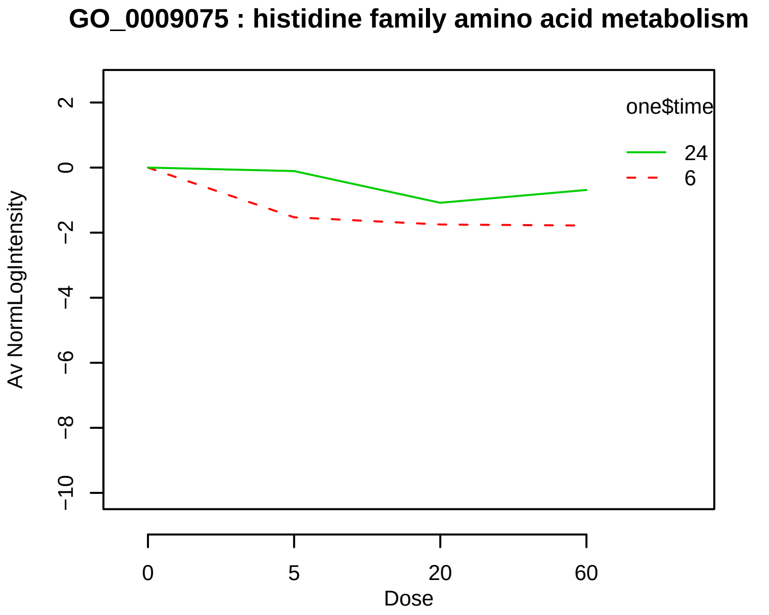
GO_0009075 : histidine family amino acid metabolism
one$time
24
6
Av NormLogIntensity
2
0
−2
−4
−6
−8
−10
0
5
20
60
Dose

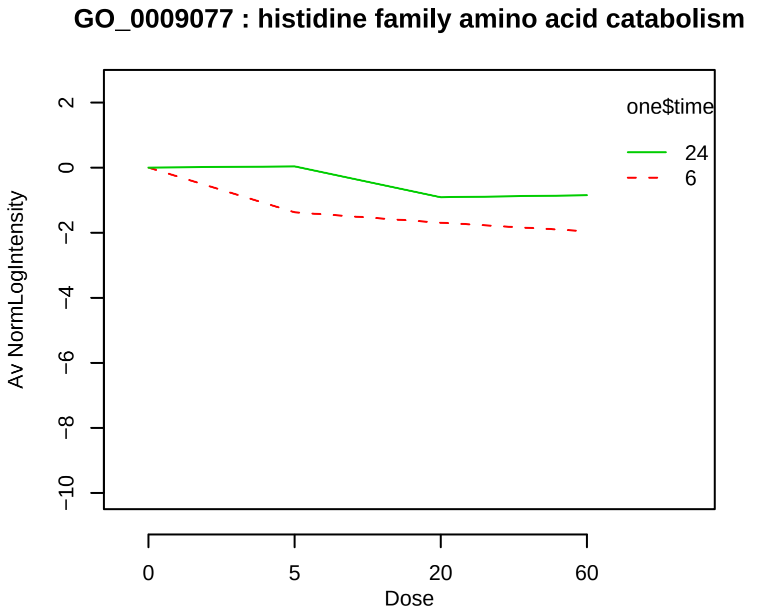
GO_0009077 : histidine family amino acid catabolism
one$time
24
6
Av NormLogIntensity
2
0
−2
−4
−6
−8
−10
0
5
20
60
Dose

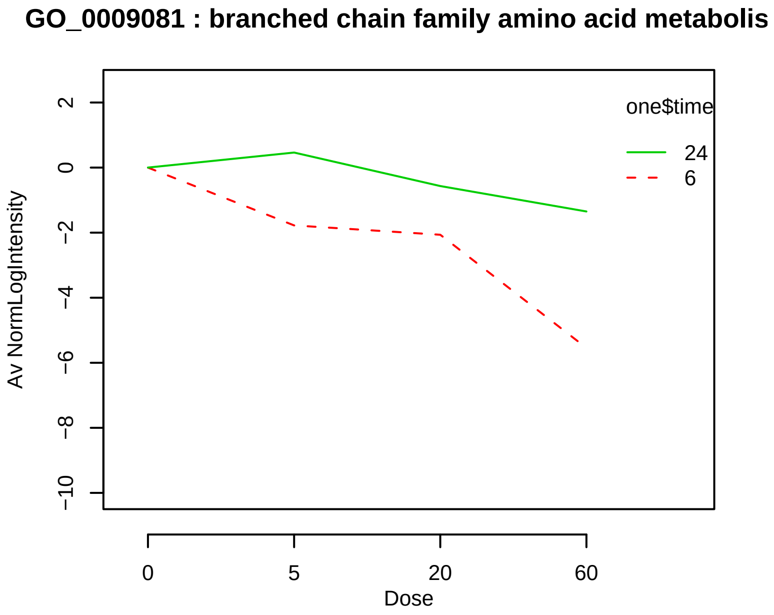
GO_0009081 : branched chain family amino acid metabolis
one$time
24
6
Av NormLogIntensity
2
0
−2
−4
−6
−8
−10
0
5
20
60
Dose

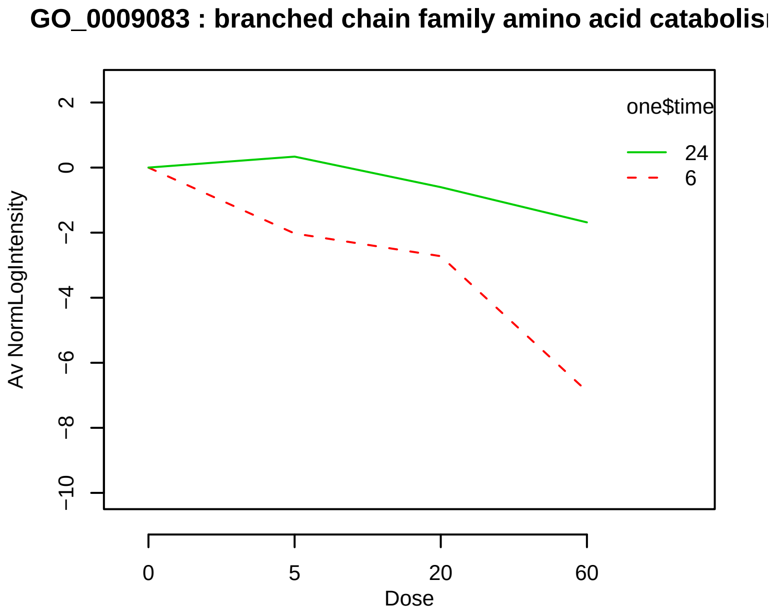
GO_0009083 : branched chain family amino acid catabolis
one$time
24
6
Av NormLogIntensity
2
0
−2
−4
−6
−8
−10
0
5
20
60
Dose

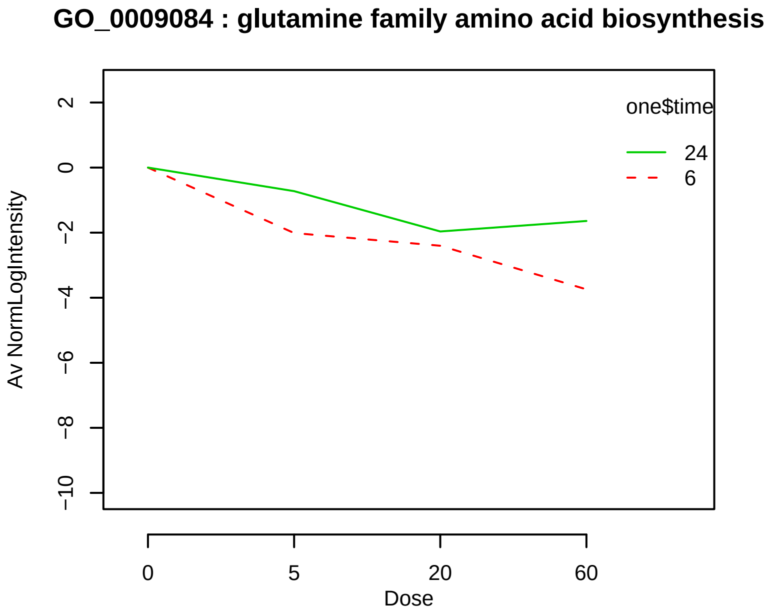
GO_0009084 : glutamine family amino acid biosynthesis
one$time
24
6
Av NormLogIntensity
2
0
−2
−4
−6
−8
−10
0
5
20
60
Dose

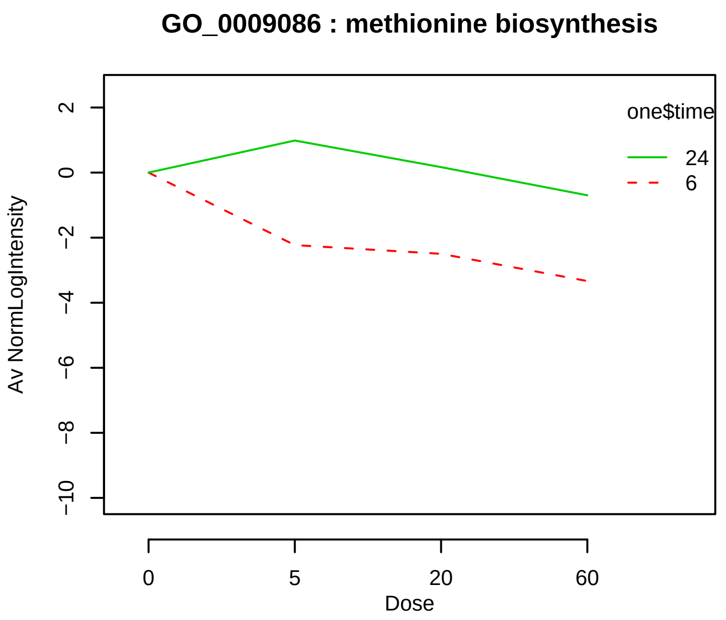

GO_0009086 : methionine biosynthesis
one$time
24
6
Av NormLogIntensity
Dose

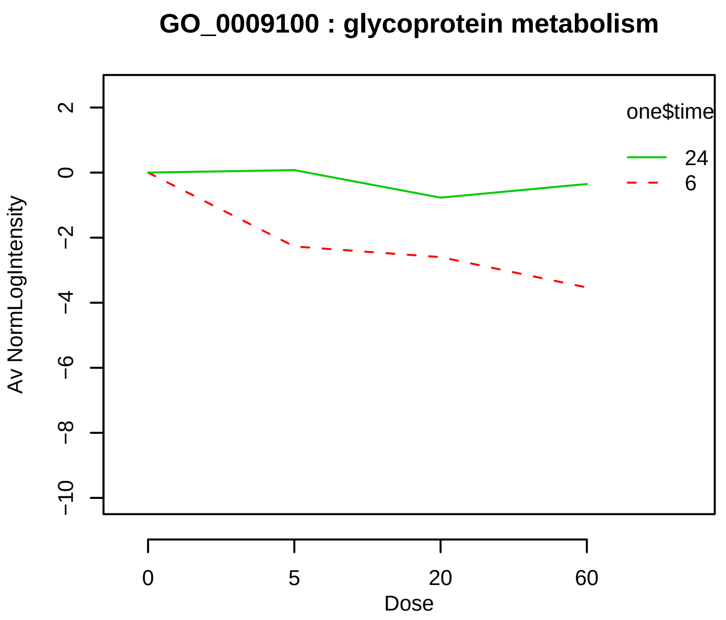

GO_0009100 : glycoprotein metabolism
one$time
24
6
Av NormLogIntensity
Dose

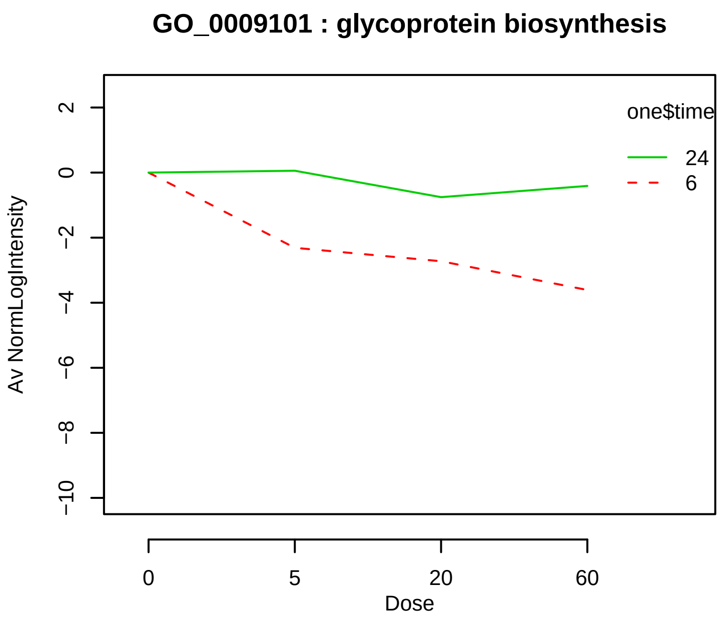

GO_0009101 : glycoprotein biosynthesis
one$time
24
6
Av NormLogIntensity
Dose

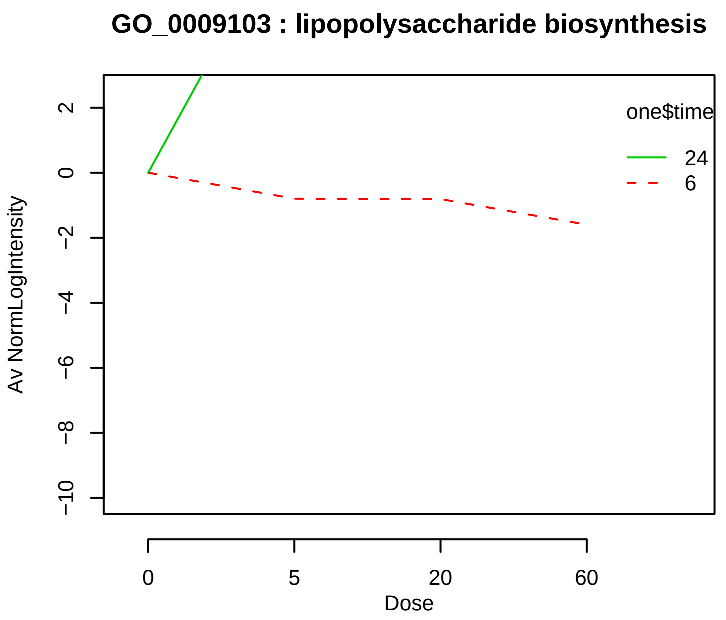

GO_0009103 : lipopolysaccharide biosynthesis
one$time
24
6
Av NormLogIntensity
Dose

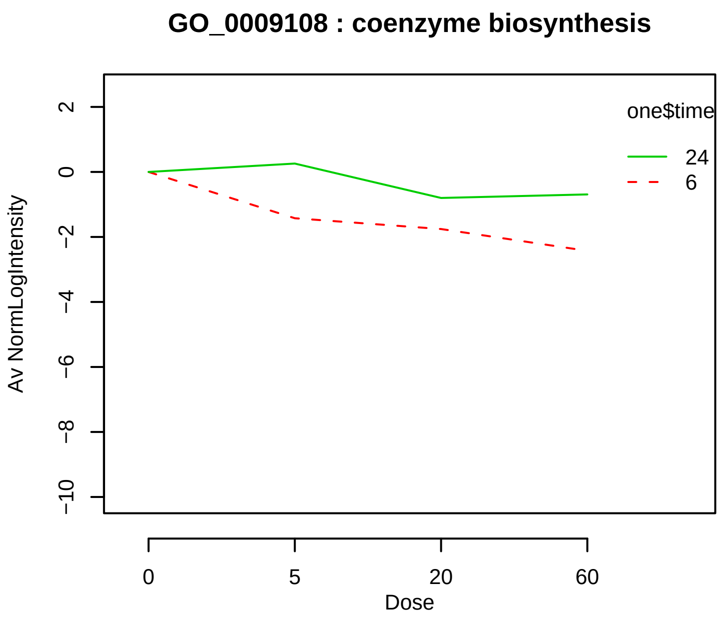

GO_0009108 : coenzyme biosynthesis
one$time
24
6
Av NormLogIntensity
Dose

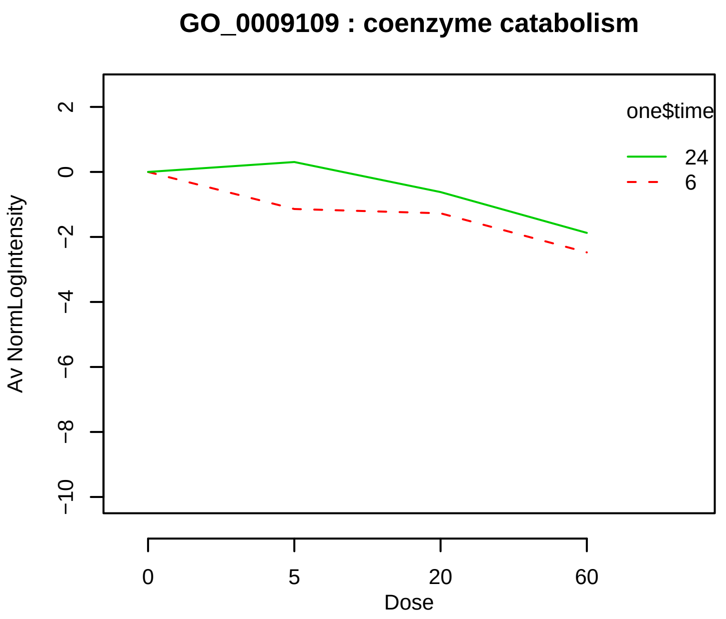

GO_0009109 : coenzyme catabolism
one$time
24
6
Av NormLogIntensity
Dose

GO_0009110 : vitamin biosynthesis

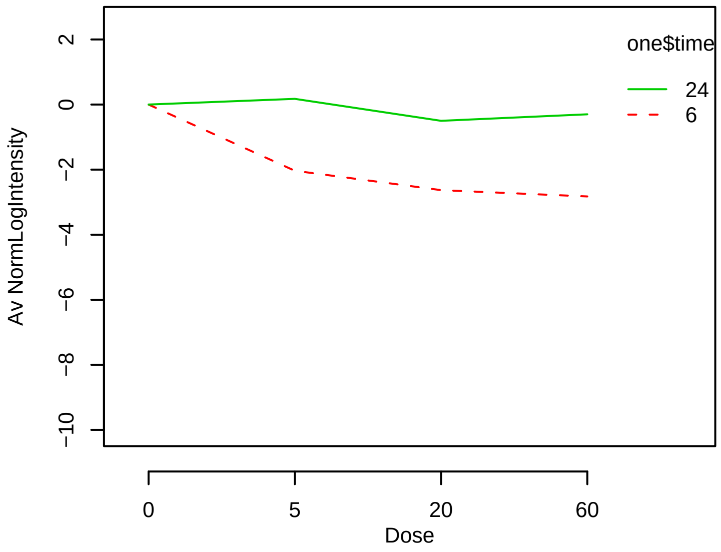

GO_0009112 : nucleobase metabolism

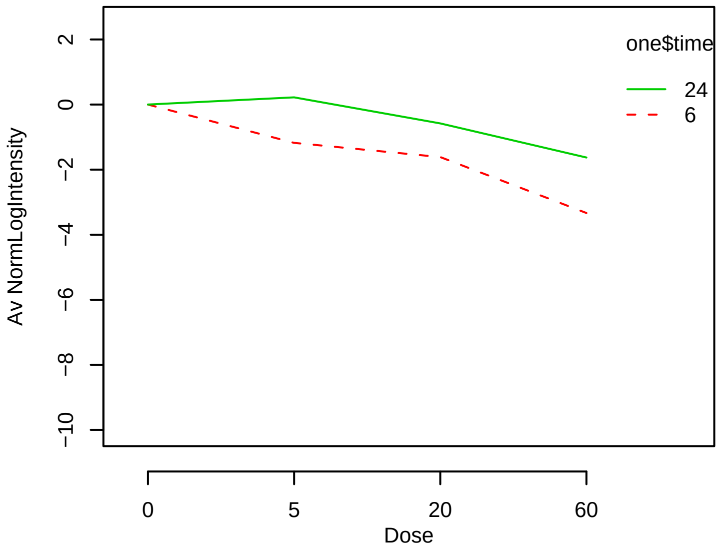

GO_0009116 : nucleoside metabolism

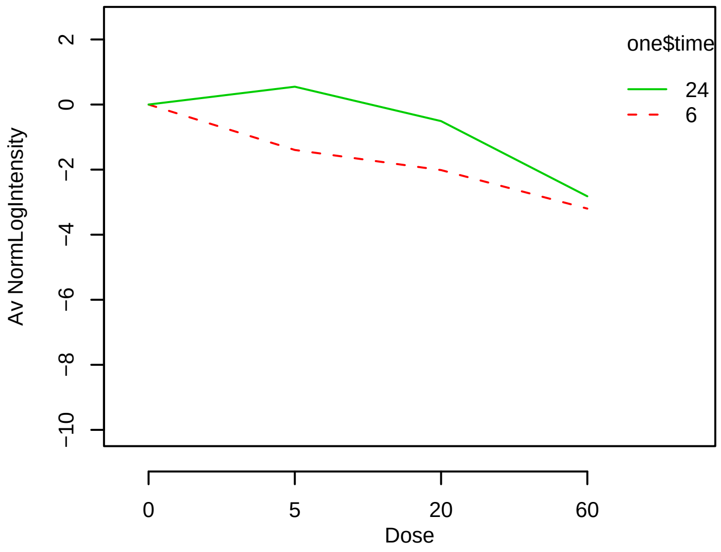

GO_0009117 : nucleotide metabolism

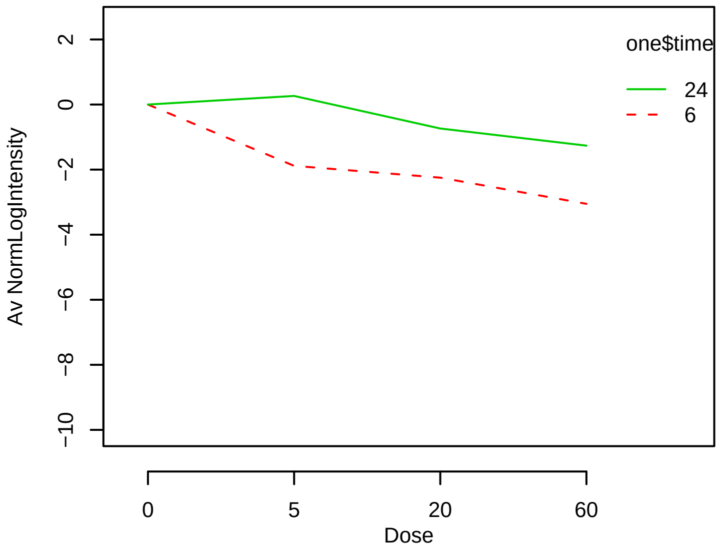

GO_0009119 : ribonucleoside metabolism

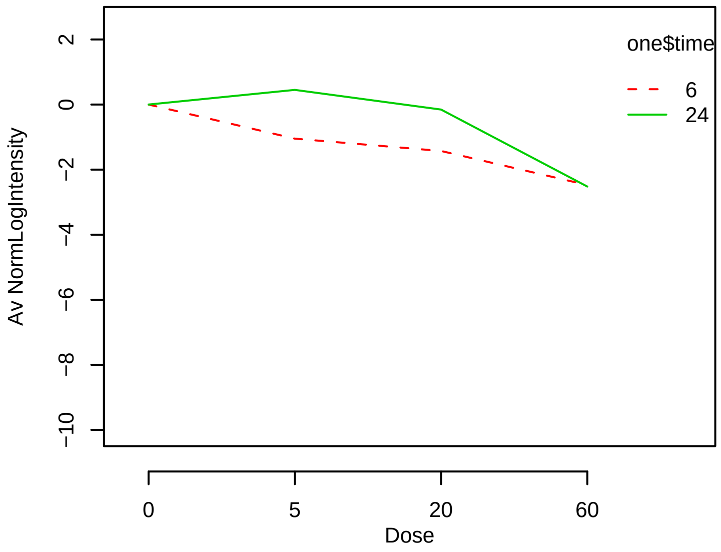

GO_0009120 : deoxyribonucleoside metabolism

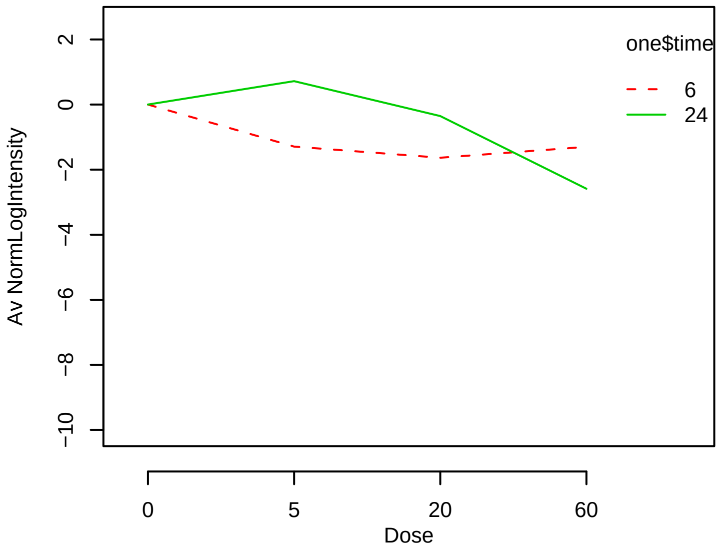

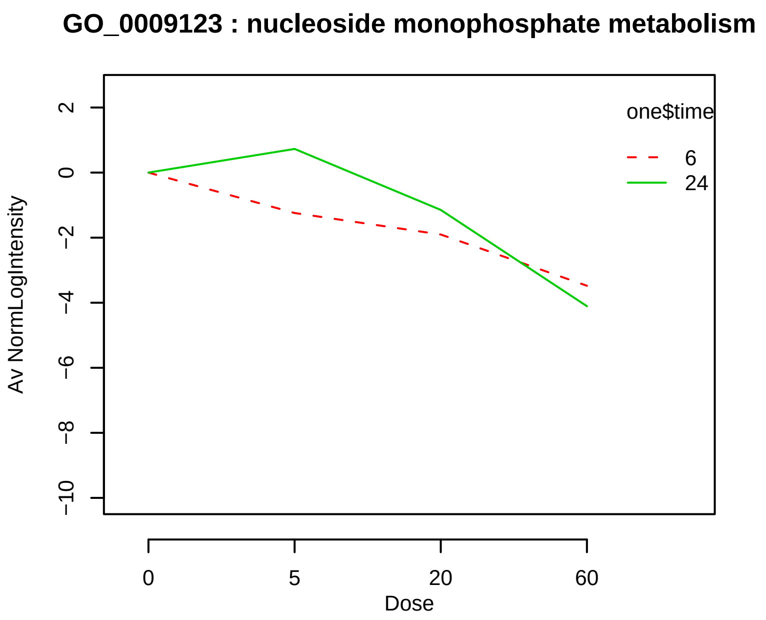
GO_0009123 : nucleoside monophosphate metabolism
Av NormLogIntensity
2
0
−2
−4
−6
−8
−10
one$time
6
24
0
5
20
60
Dose

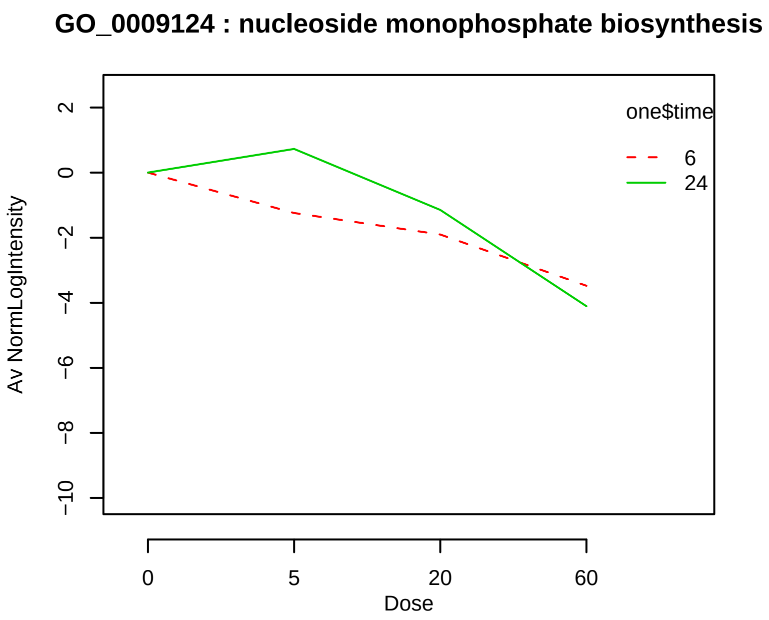
GO_0009124 : nucleoside monophosphate biosynthesis
Av NormLogIntensity
2
0
−2
−4
−6
−8
−10
one$time
6
24
0
5
20
60
Dose

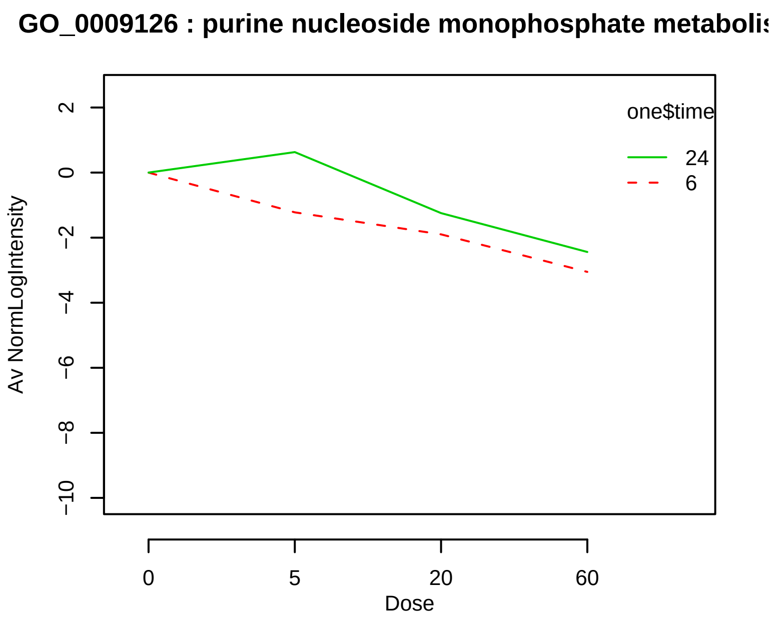
GO_0009126 : purine nucleoside monophosphate metabolis
Av NormLogIntensity
2
0
−2
−4
−6
−8
−10
one$time
24
6
0
5
20
60
Dose

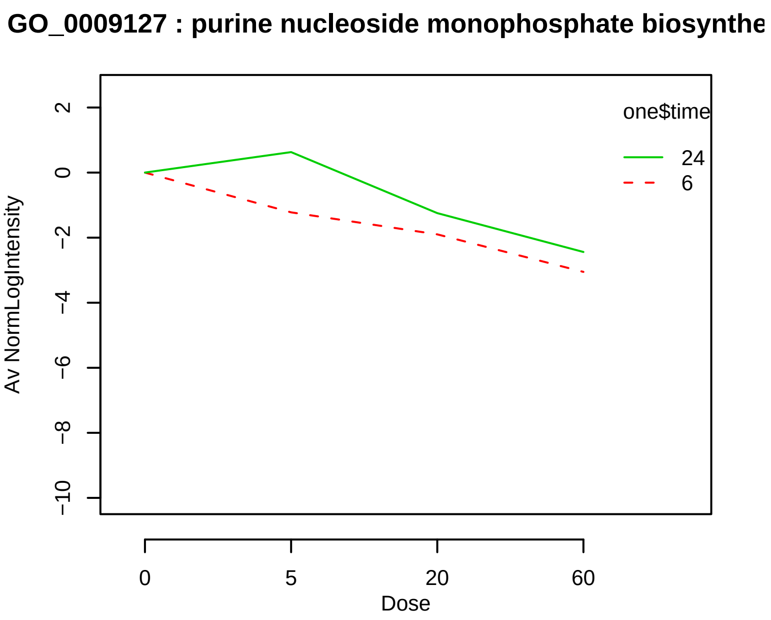
GO_0009127 : purine nucleoside monophosphate biosynthe
Av NormLogIntensity
2
0
−2
−4
−6
−8
−10
one$time
24
6
0
5
20
60
Dose

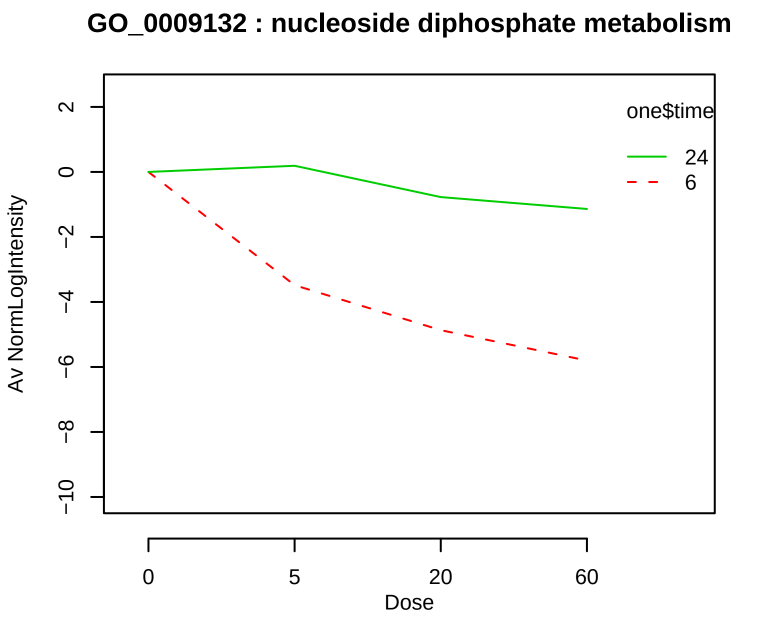
GO_0009132 : nucleoside diphosphate metabolism
Av NormLogIntensity
2
0
−2
−4
−6
−8
−10
one$time
24
6
0
5
20
60
Dose

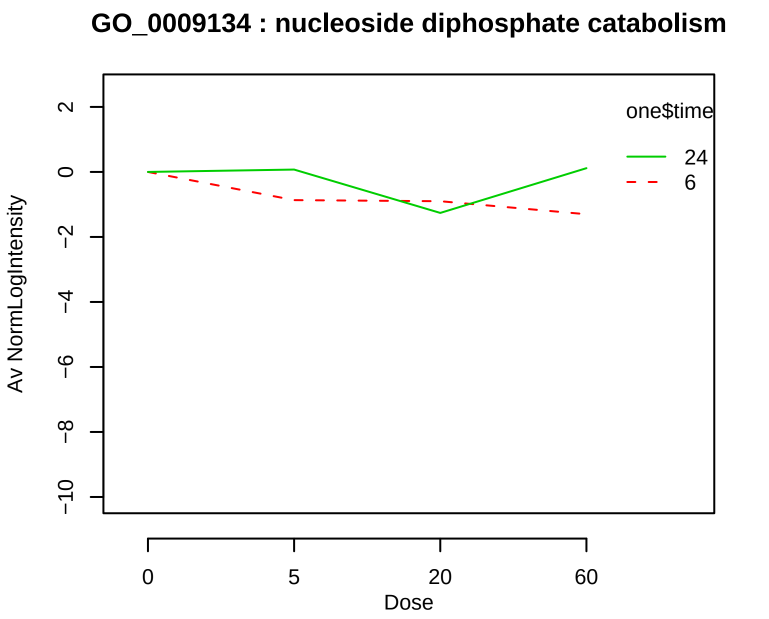
GO_0009134 : nucleoside diphosphate catabolism
Av NormLogIntensity
2
0
−2
−4
−6
−8
−10
one$time
24
6
0
5
20
60
Dose

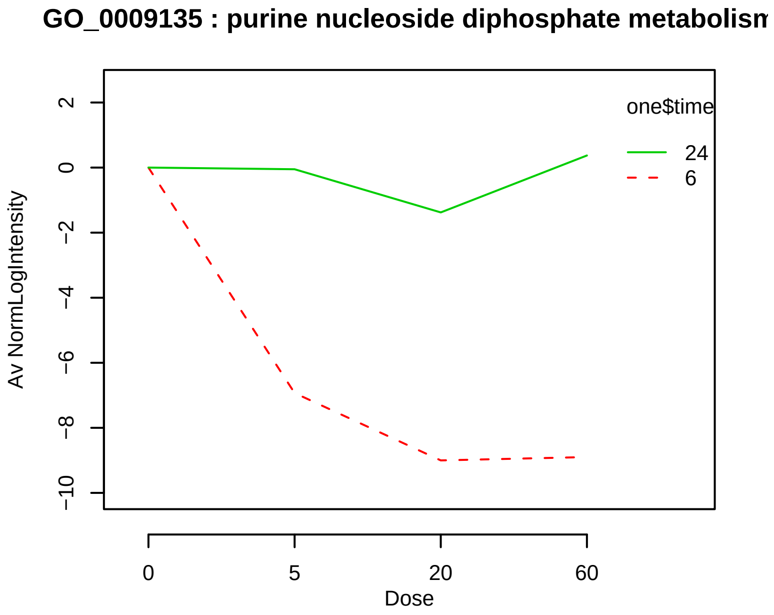

GO_0009135 : purine nucleoside diphosphate metabolism
Av NormLogIntensity
2
0
−2
−4
−6
−8
−10
one$time
24
6
0
5
20
60
Dose

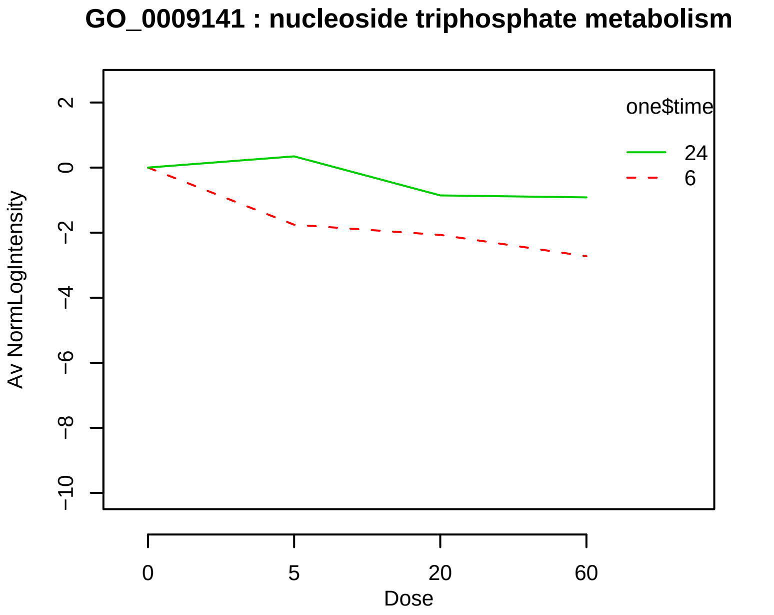

GO_0009141 : nucleoside triphosphate metabolism
Av NormLogIntensity
2
0
−2
−4
−6
−8
−10
one$time
24
6
0
5
20
60
Dose

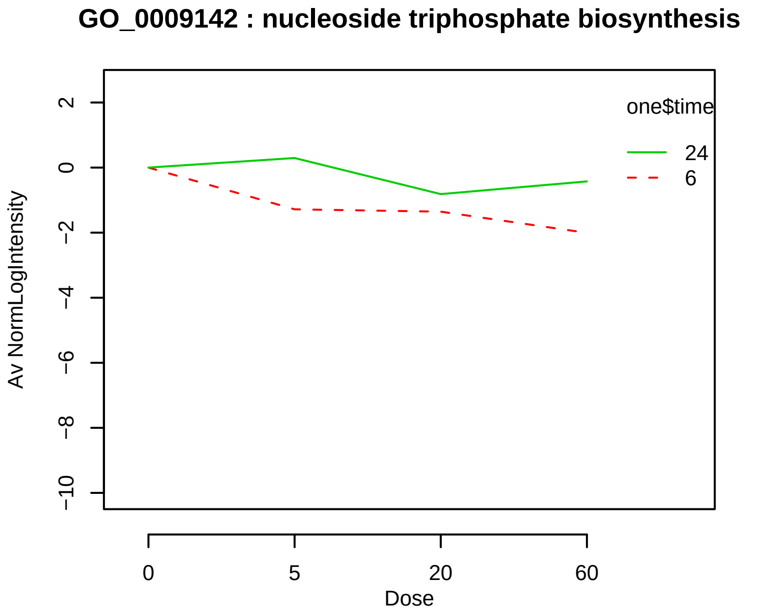

GO_0009142 : nucleoside triphosphate biosynthesis
Av NormLogIntensity
2
0
−2
−4
−6
−8
−10
one$time
24
6
0
5
20
60
Dose

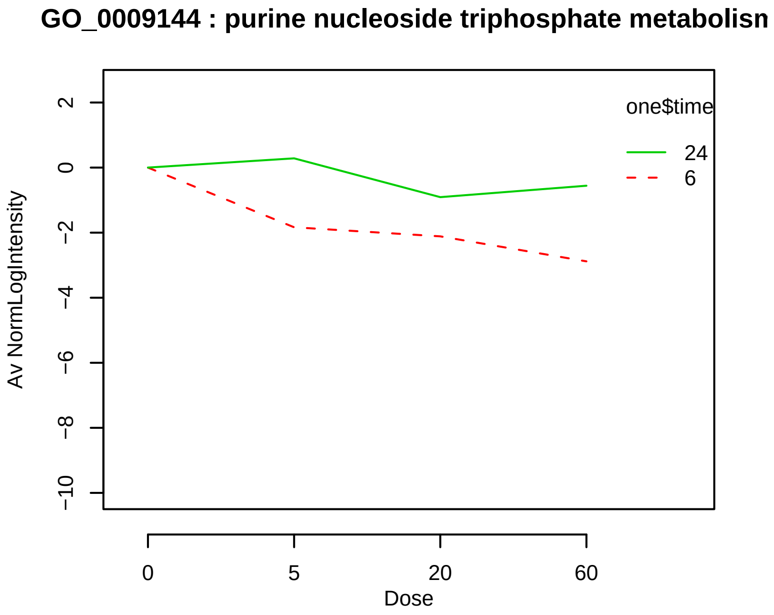

GO_0009144 : purine nucleoside triphosphate metabolism
Av NormLogIntensity
2
0
−2
−4
−6
−8
−10
one$time
24
6
0
5
20
60
Dose

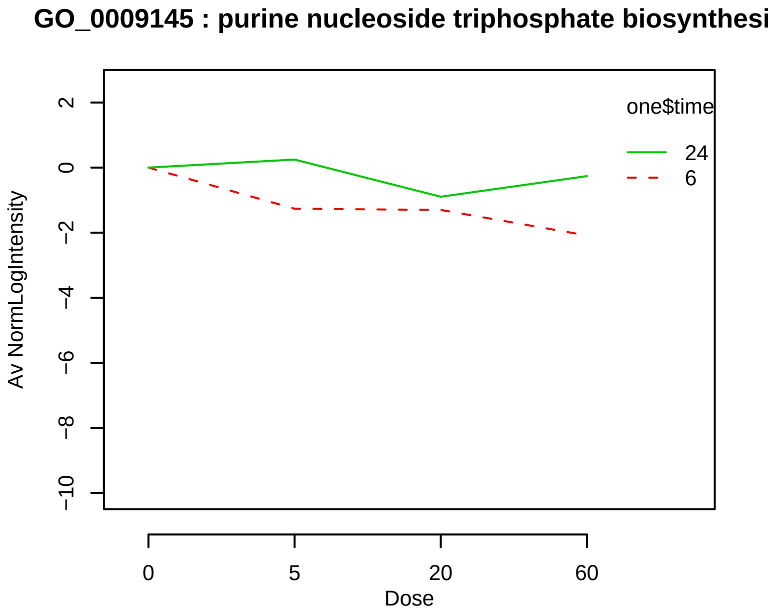

GO_0009145 : purine nucleoside triphosphate biosynthesi
Av NormLogIntensity
2
0
−2
−4
−6
−8
−10
one$time
24
6
0
5
20
60
Dose

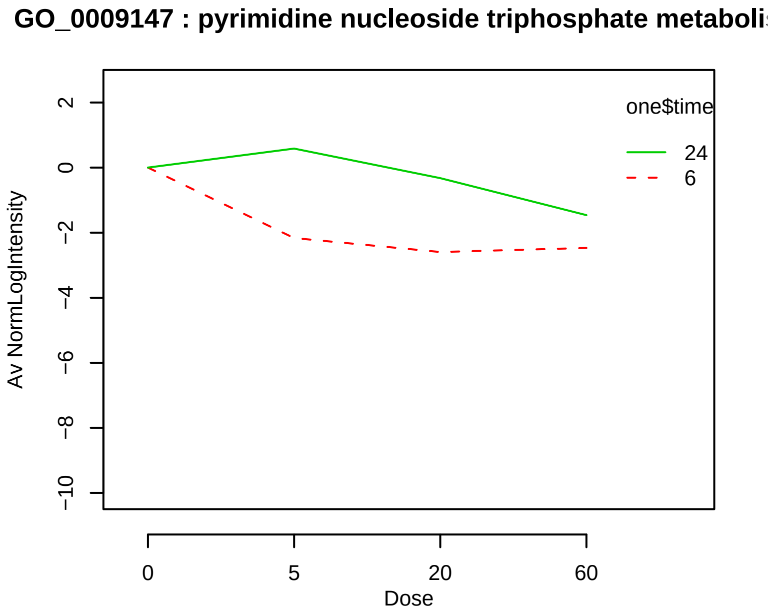

GO_0009147 : pyrimidine nucleoside triphosphate metaboli
Av NormLogIntensity
2
0
−2
−4
−6
−8
−10
one$time
24
6
0
5
20
60
Dose

GO_0009150 : purine ribonucleotide metabolism

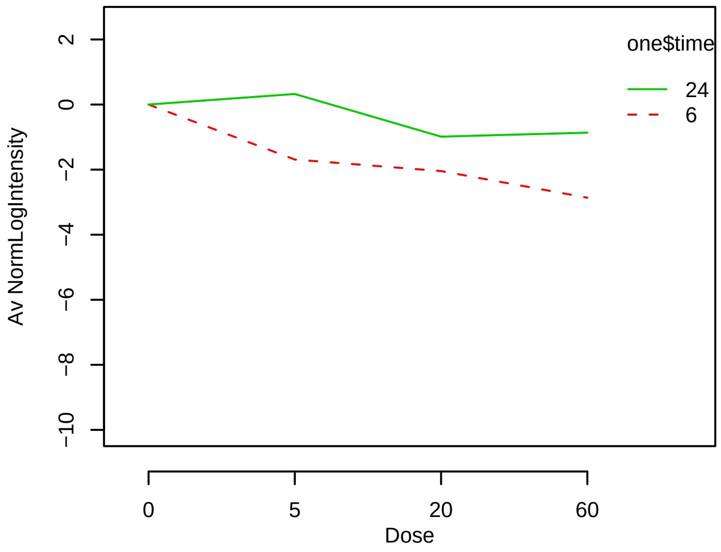

GO_0009152 : purine ribonucleotide biosynthesis

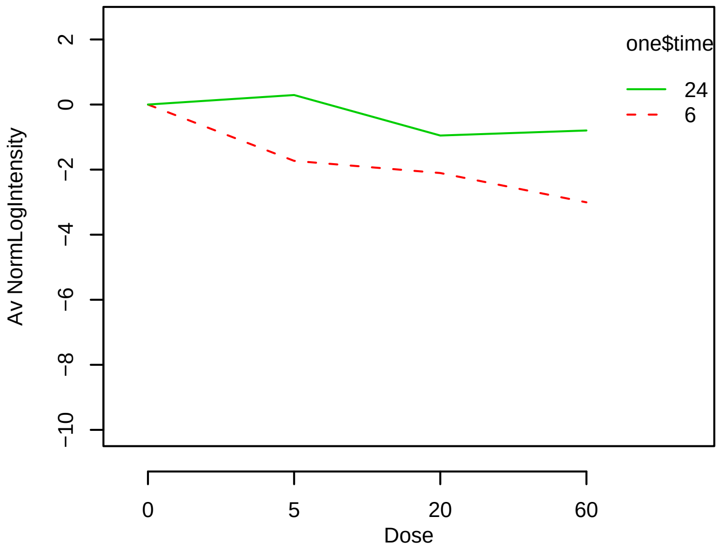

GO_0009154 : purine ribonucleotide catabolism

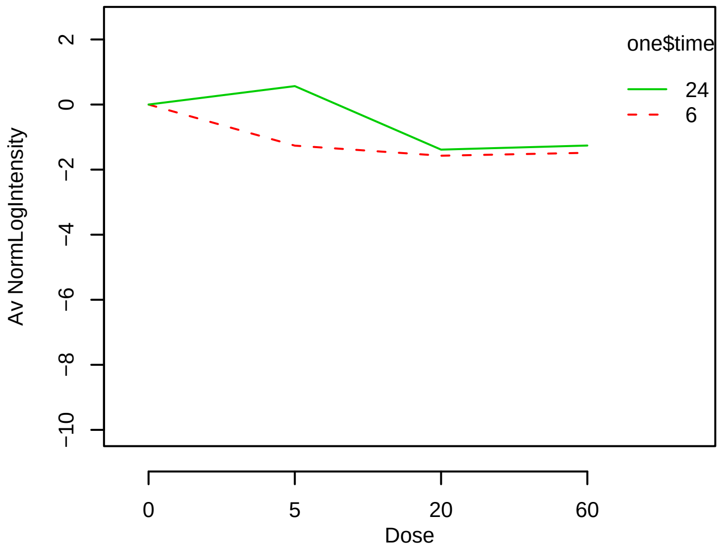

GO_0009156 : ribonucleoside monophosphate biosynthes

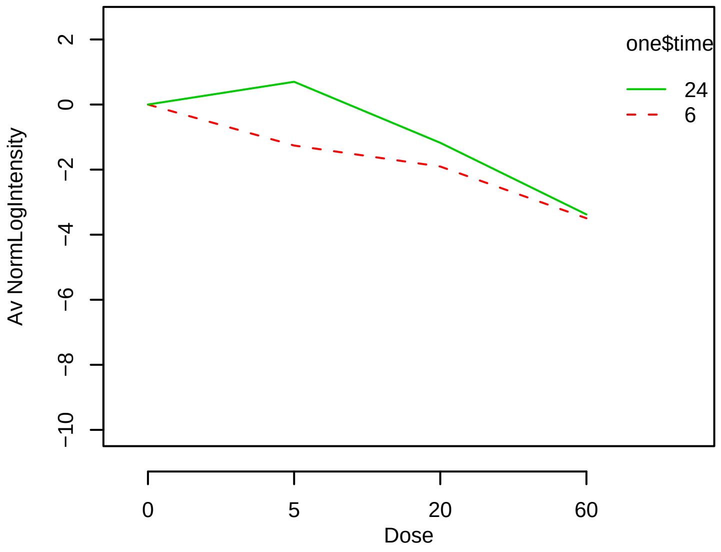

GO_0009161 : ribonucleoside monophosphate metabolisr

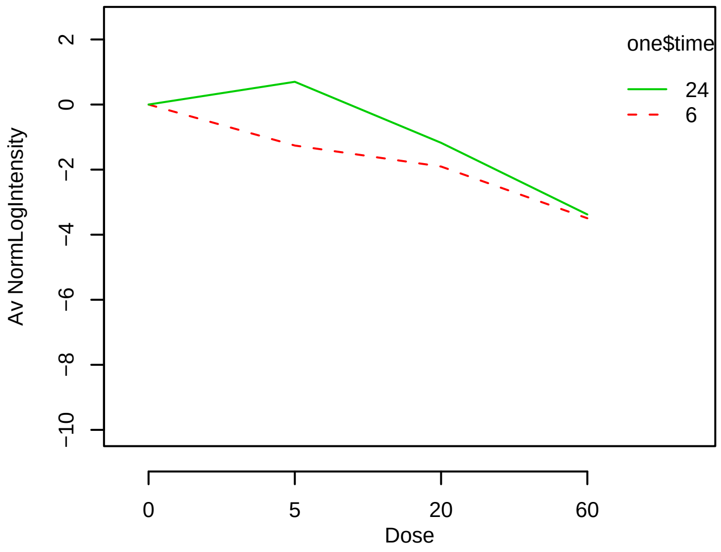

GO_0009165 : nucleotide biosynthesis

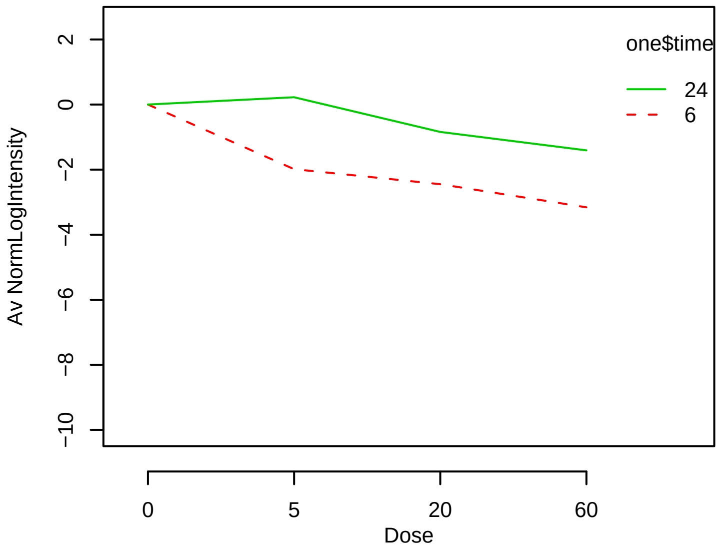

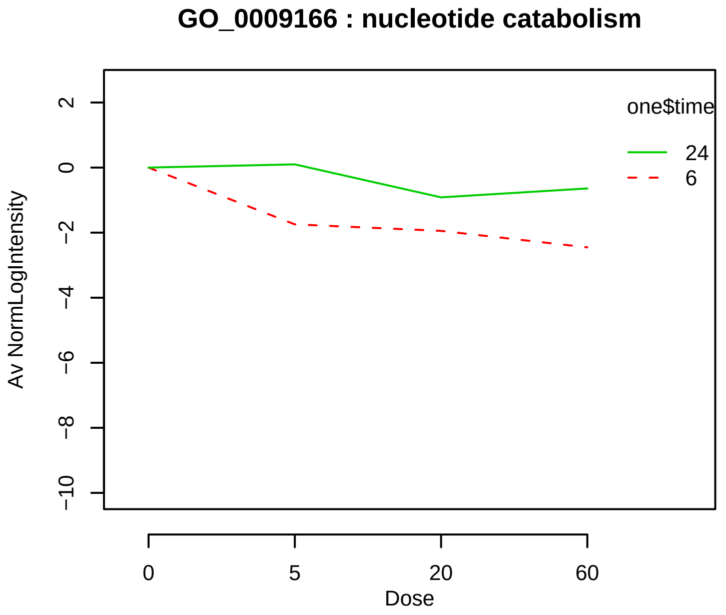
GO_0009166 : nucleotide catabolism
Av NormLogIntensity
Dose
one$time
24
6

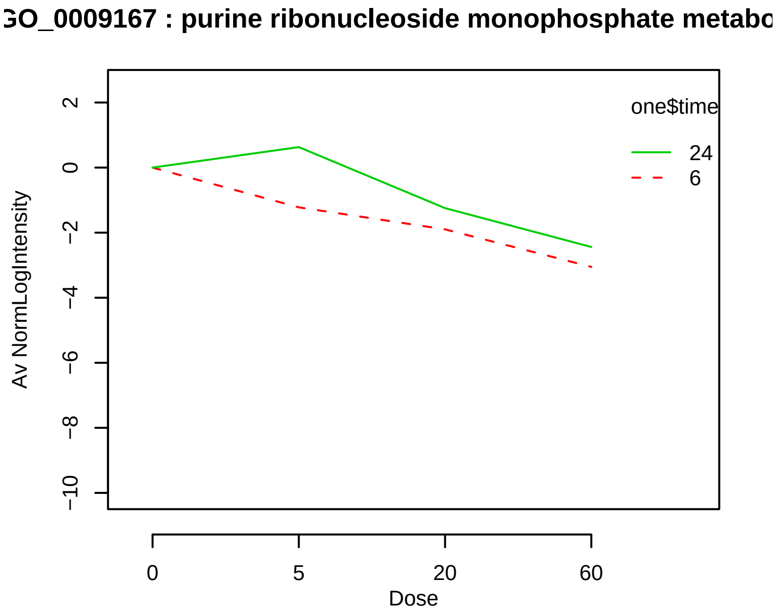
O_0009167 : purine ribonucleoside monophosphate metabo
Av NormLogIntensity
Dose
one$time
24
6

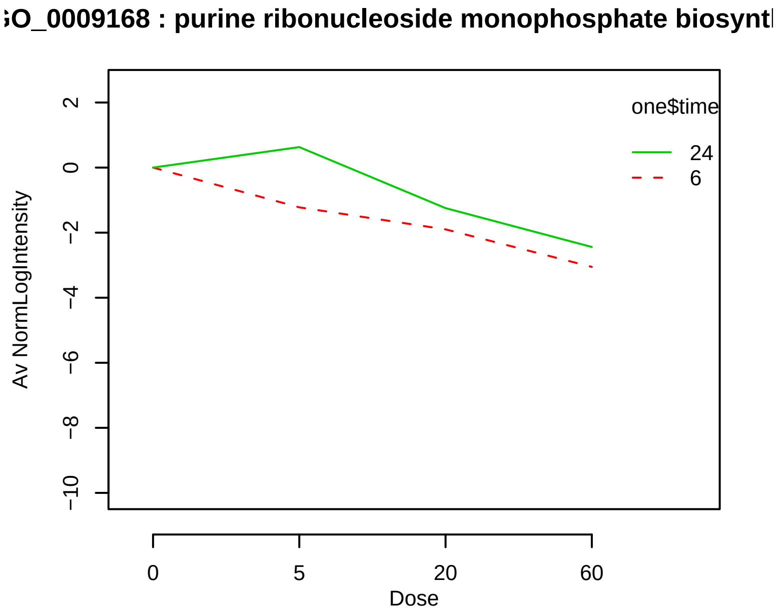
O_0009168 : purine ribonucleoside monophosphate biosynt
Av NormLogIntensity
Dose
one$time
24
6

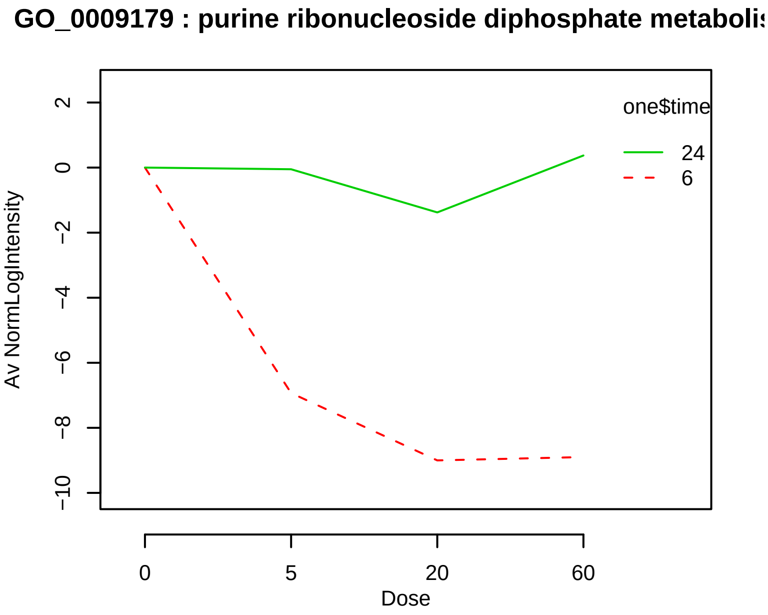
GO_0009179 : purine ribonucleoside diphosphate metaboli
Av NormLogIntensity
Dose
one$time
24
6

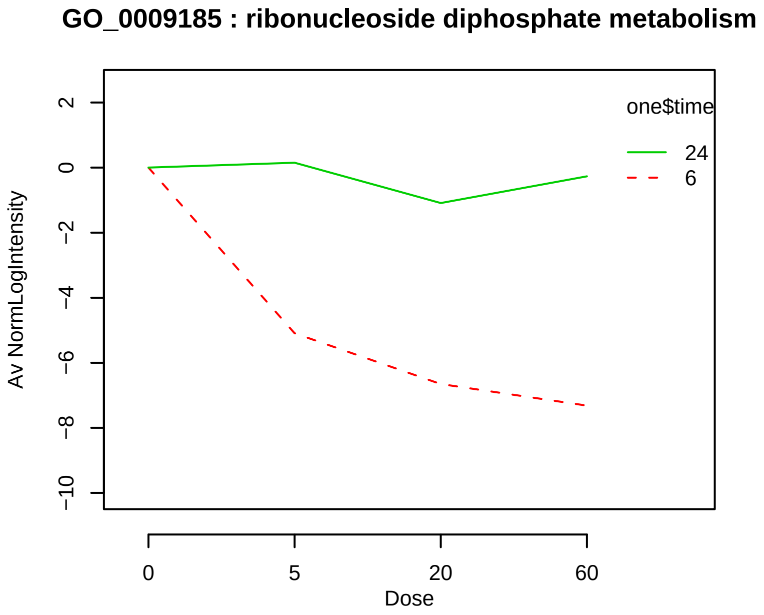
GO_0009185 : ribonucleoside diphosphate metabolism
Av NormLogIntensity
Dose
one$time
24
6

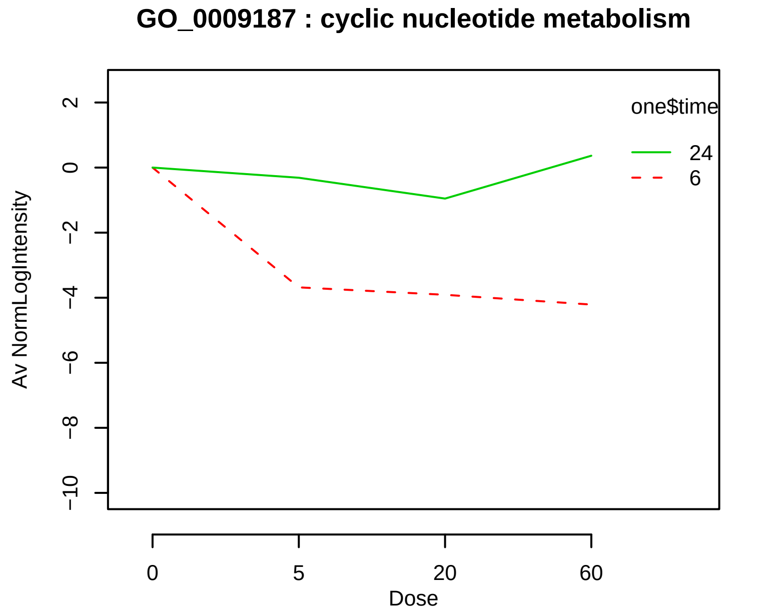
GO_0009187 : cyclic nucleotide metabolism
Av NormLogIntensity
Dose
one$time
24
6

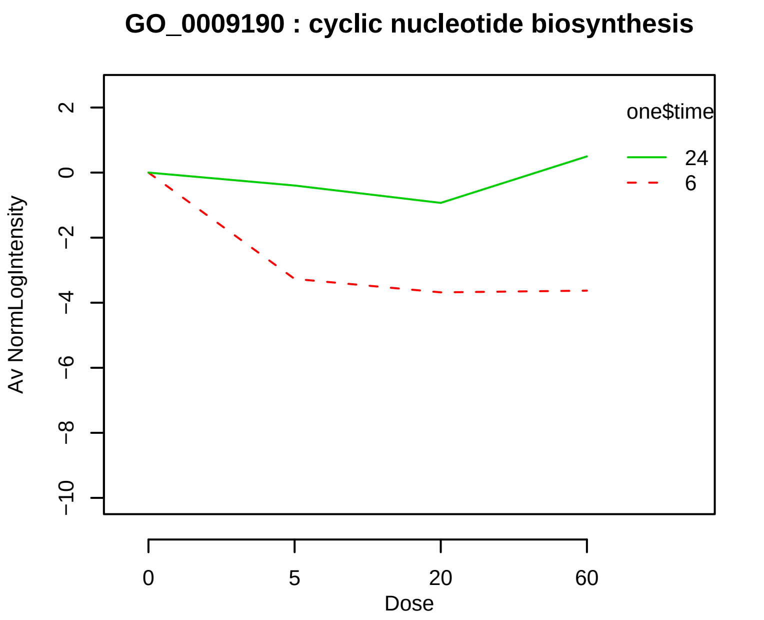
GO_0009190 : cyclic nucleotide biosynthesis
Av NormLogIntensity
2
0
−2
−4
−6
−8
−10
0
5
20
60
Dose
one$time
24
6

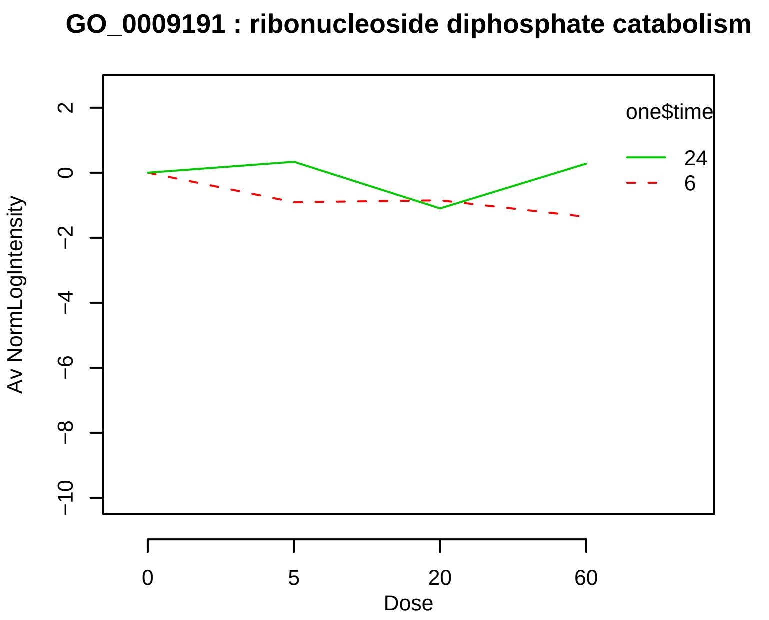
GO_0009191 : ribonucleoside diphosphate catabolism
Av NormLogIntensity
2
0
−2
−4
−6
−8
−10
0
5
20
60
Dose
one$time
24
6

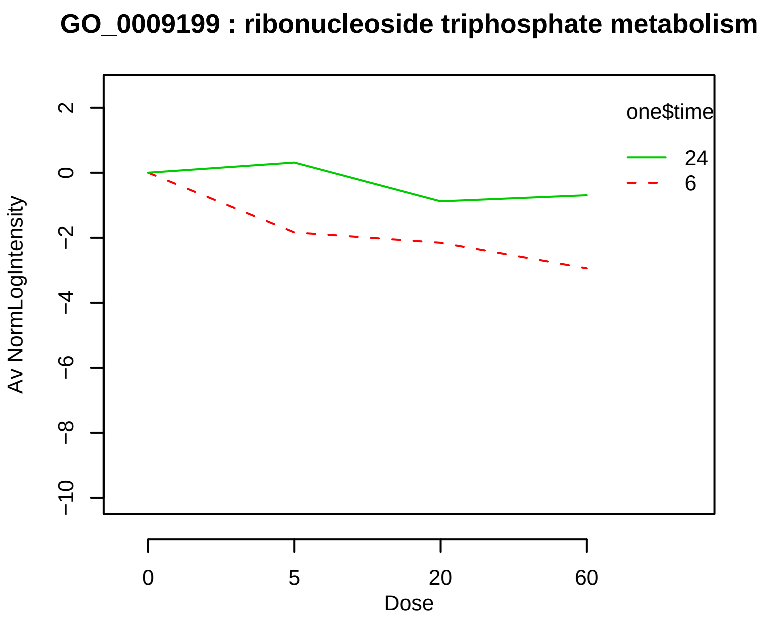
GO_0009199 : ribonucleoside triphosphate metabolism
Av NormLogIntensity
2
0
−2
−4
−6
−8
−10
0
5
20
60
Dose
one$time
24
6

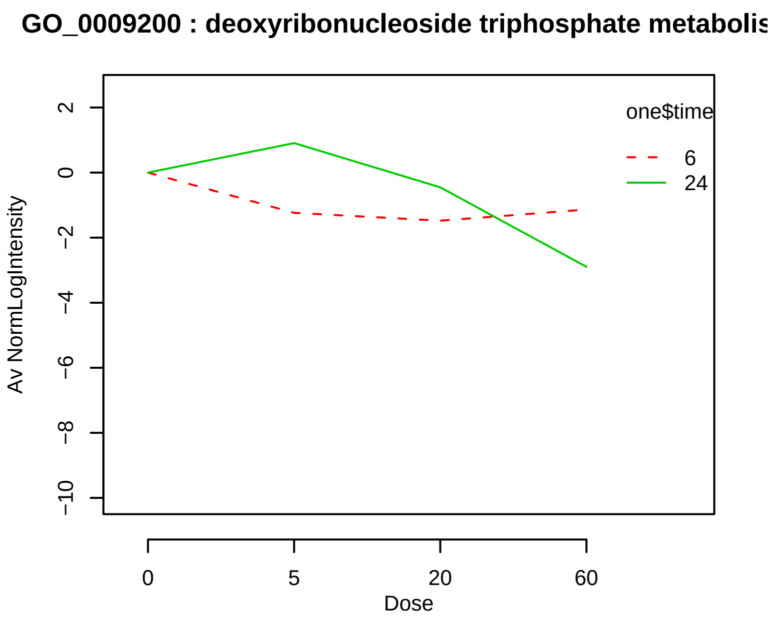
GO_0009200 : deoxyribonucleoside triphosphate metabolis
Av NormLogIntensity
2
0
−2
−4
−6
−8
−10
0
5
20
60
Dose
one$time
6
24

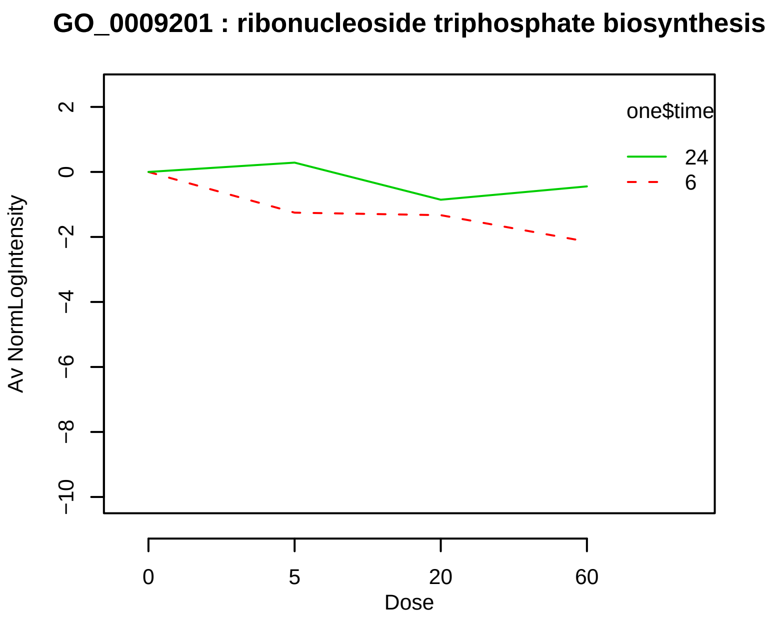
GO_0009201 : ribonucleoside triphosphate biosynthesis
Av NormLogIntensity
2
0
−2
−4
−6
−8
−10
0
5
20
60
Dose
one$time
24
6

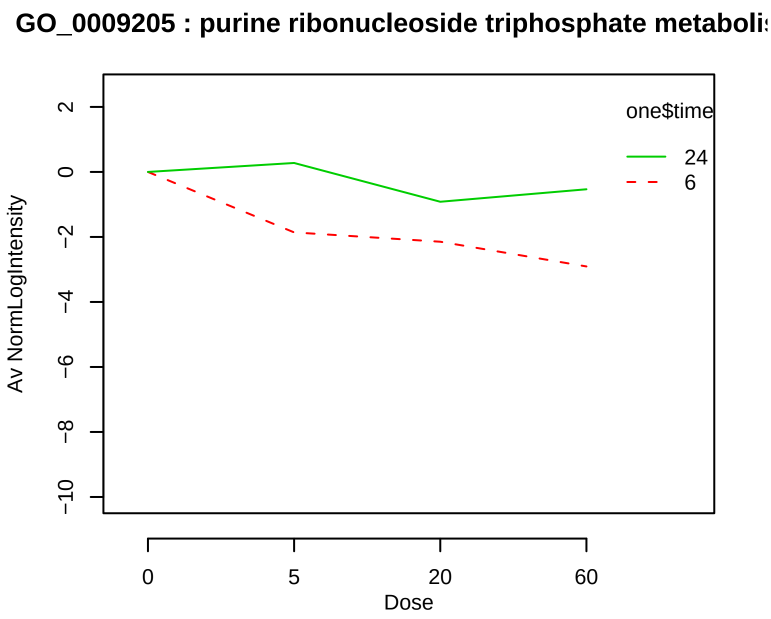
GO_0009205 : purine ribonucleoside triphosphate metabolis
Av NormLogIntensity
2
0
−2
−4
−6
−8
−10
0
5
20
60
Dose
one$time
24
6

GO_0009206 : purine ribonucleoside triphosphate biosynthe

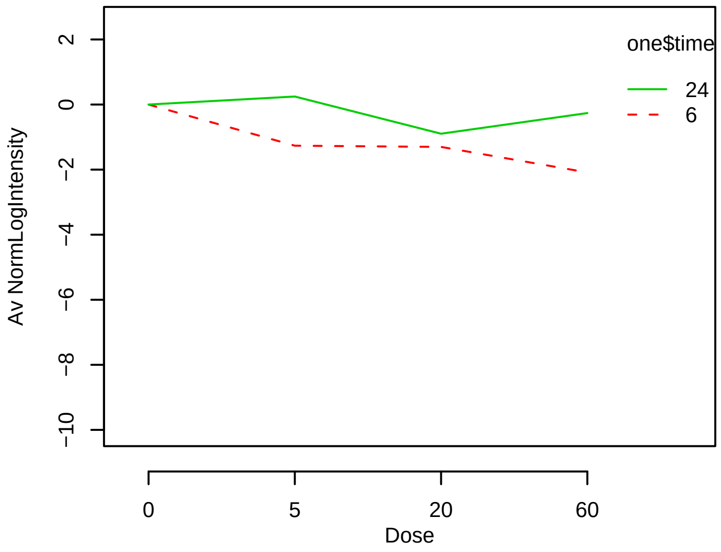

GO_0009208 : pyrimidine ribonucleoside triphosphate metabo

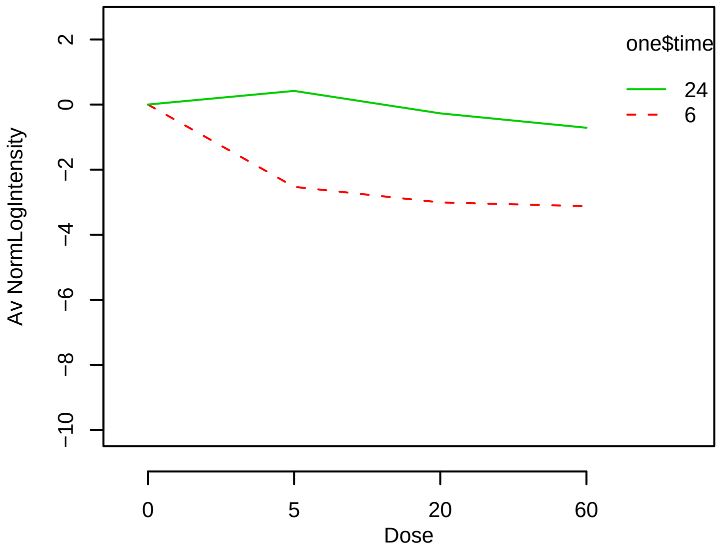

GO_0009209 : pyrimidine ribonucleoside triphosphate biosyn

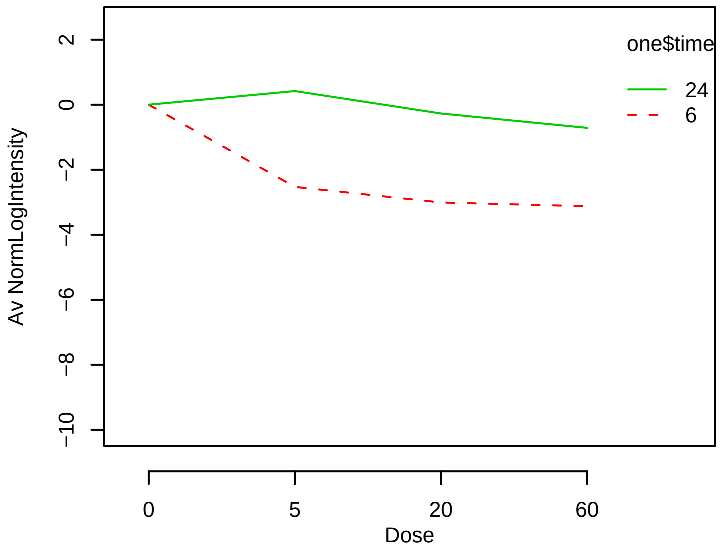

O_0009211 : pyrimidine deoxyribonucleoside triphosphate m

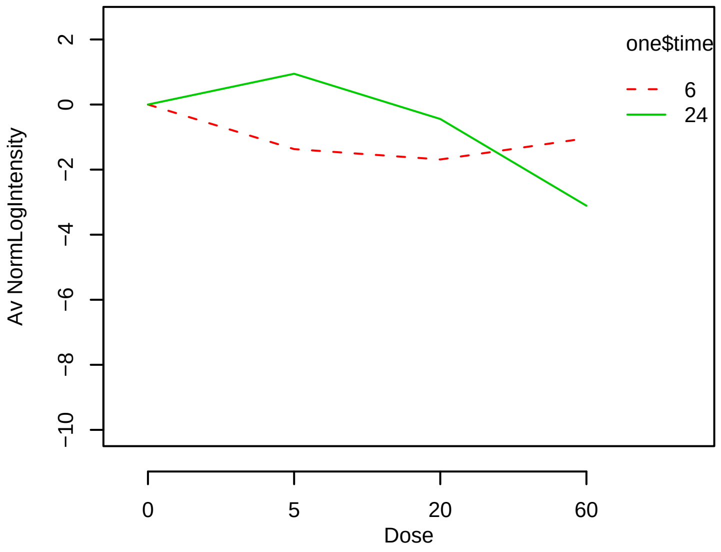

GO_0009218 : pyrimidine ribonucleotide metabolism

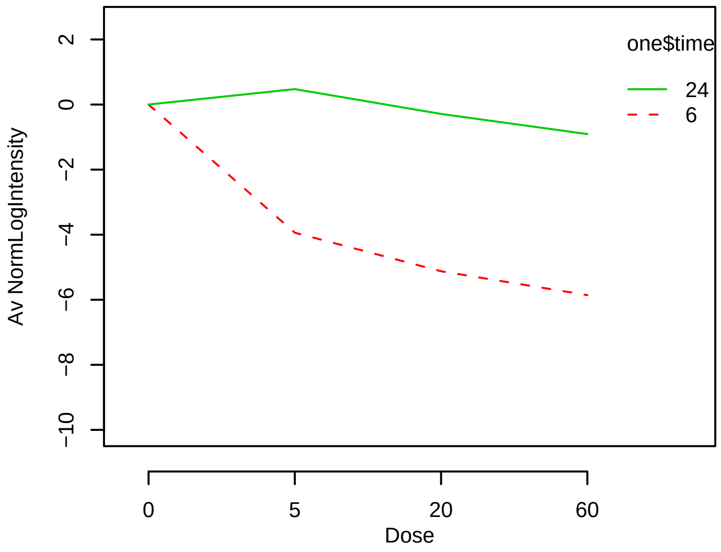

GO_0009219 : pyrimidine deoxyribonucleotide metabolism

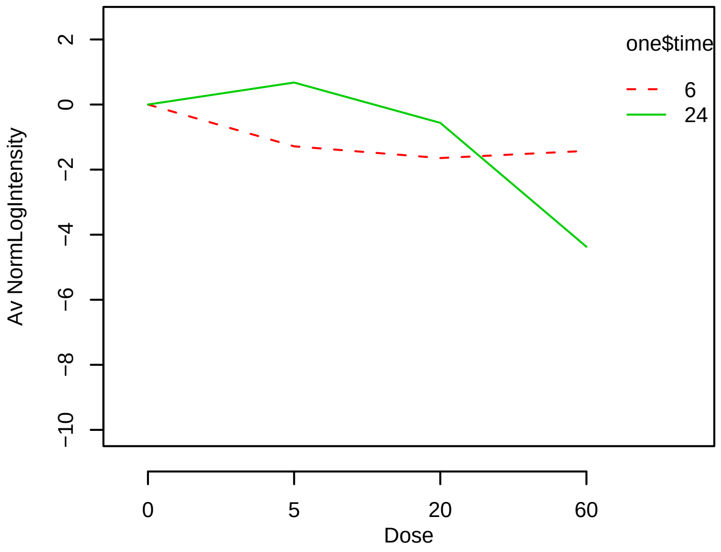

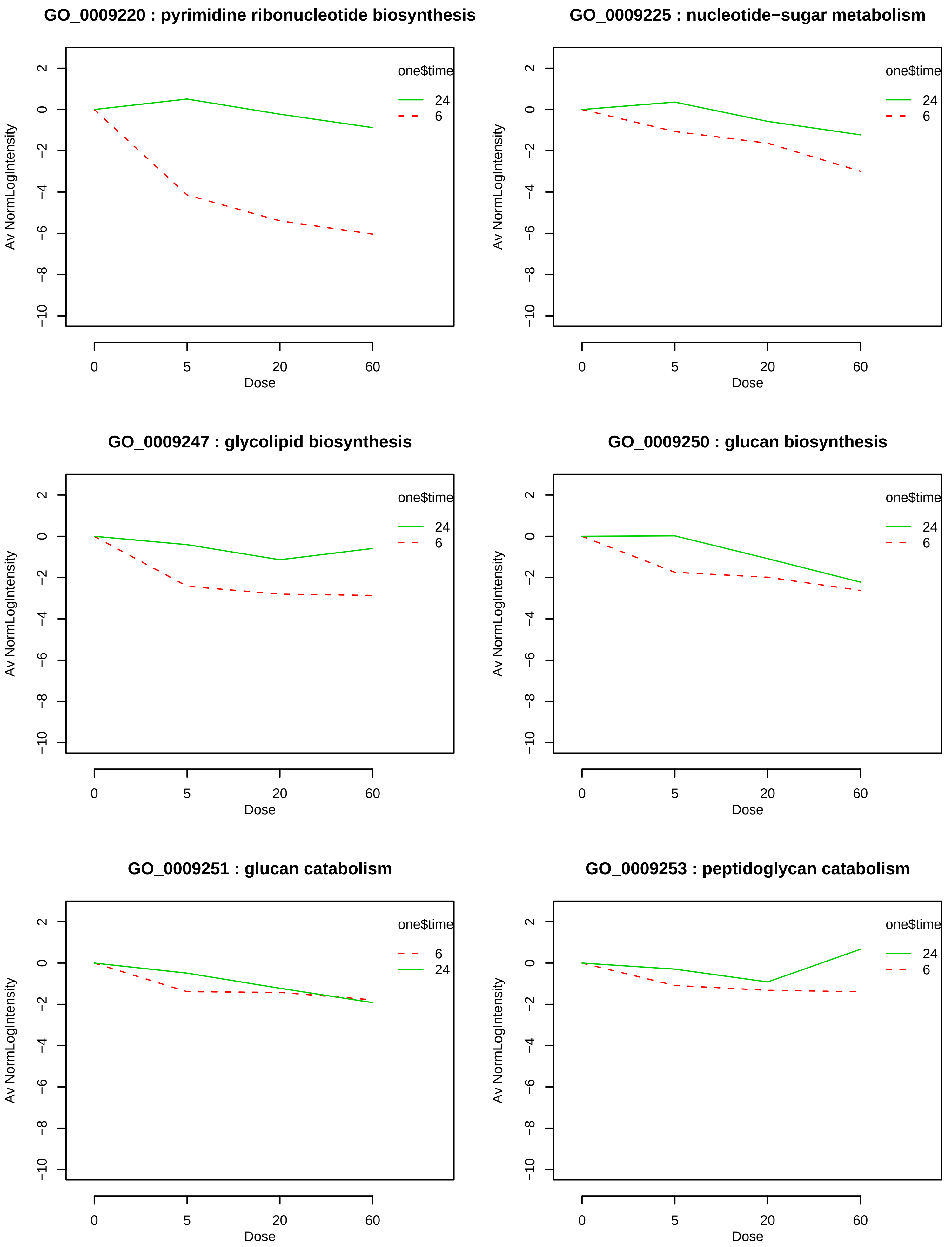
GO_0009220 : pyrimidine ribonucleotide biosynthesis
GO_0009225 : nucleotide−sugar metabolism
GO_0009247 : glycolipid biosynthesis
GO_0009250 : glucan biosynthesis
GO_0009251 : glucan catabolism
GO_0009253 : peptidoglycan catabolism
Av NormLogIntensity
Dose
0
5
20
60
2
0
−2
−4
−6
−8
−10
one$time
24
6

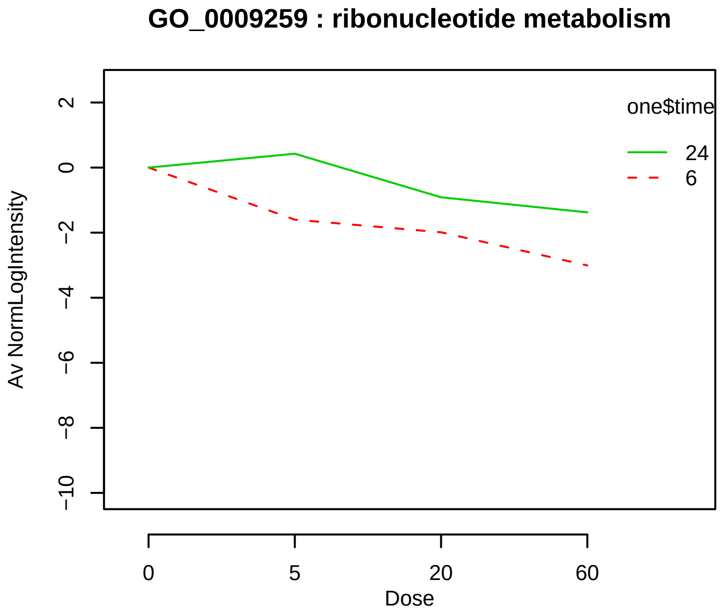
GO_0009259 : ribonucleotide metabolism
Av NormLogIntensity
2
0
−2
−4
−6
−8
−10
one$time
24
6
0
5
20
60
Dose

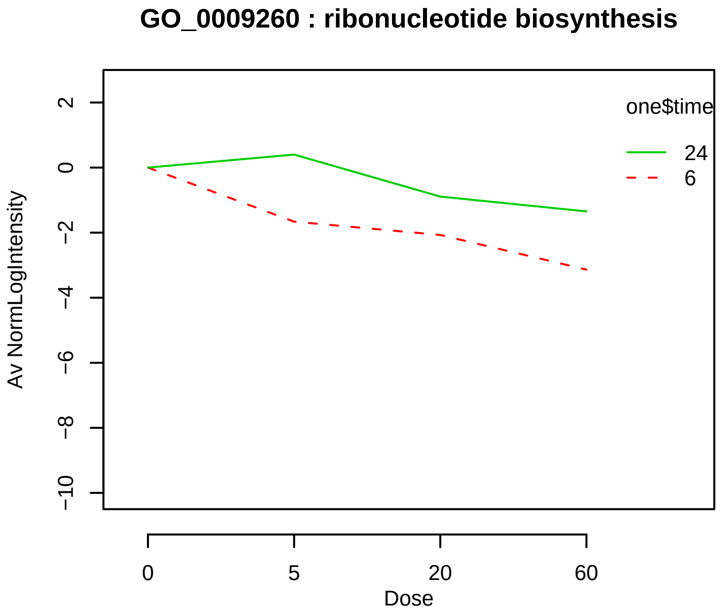
GO_0009260 : ribonucleotide biosynthesis
Av NormLogIntensity
2
0
−2
−4
−6
−8
−10
one$time
24
6
0
5
20
60
Dose

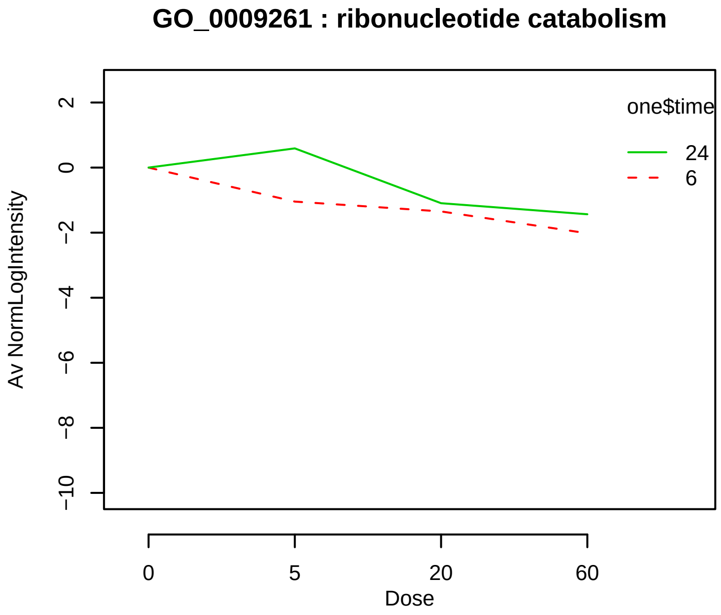
GO_0009261 : ribonucleotide catabolism
Av NormLogIntensity
2
0
−2
−4
−6
−8
−10
one$time
24
6
0
5
20
60
Dose

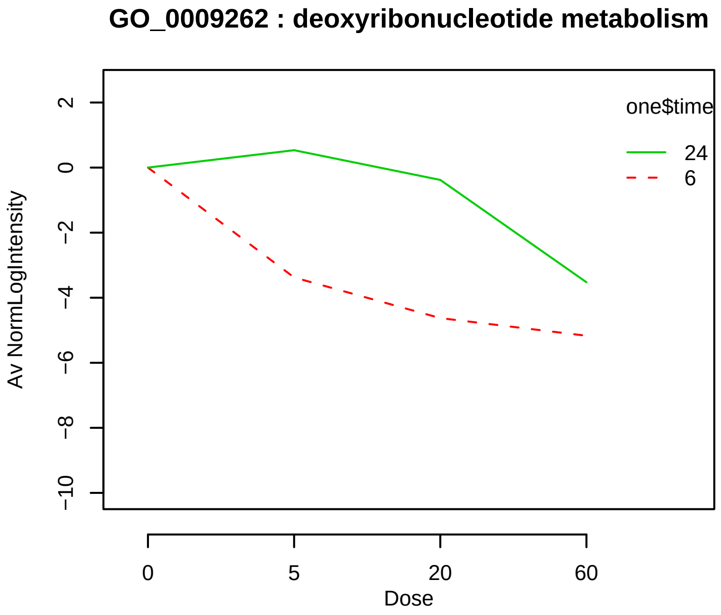
GO_0009262 : deoxyribonucleotide metabolism
Av NormLogIntensity
2
0
−2
−4
−6
−8
−10
one$time
24
6
0
5
20
60
Dose

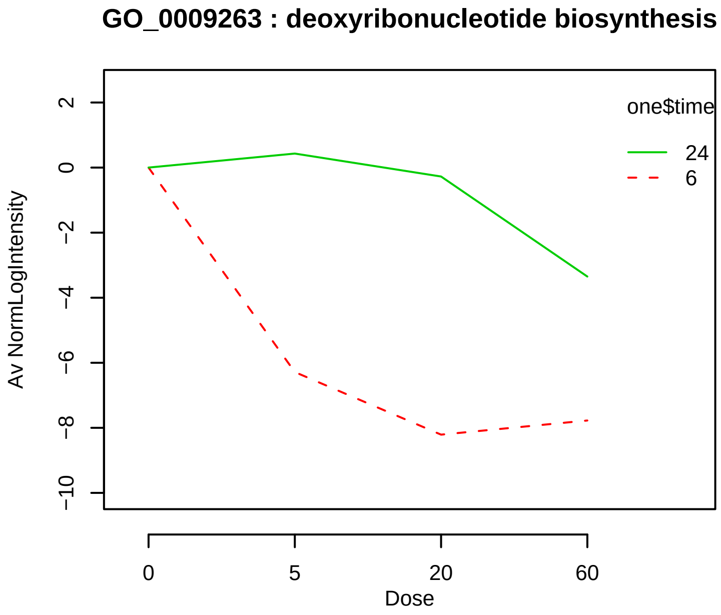
GO_0009263 : deoxyribonucleotide biosynthesis
Av NormLogIntensity
2
0
−2
−4
−6
−8
−10
one$time
24
6
0
5
20
60
Dose

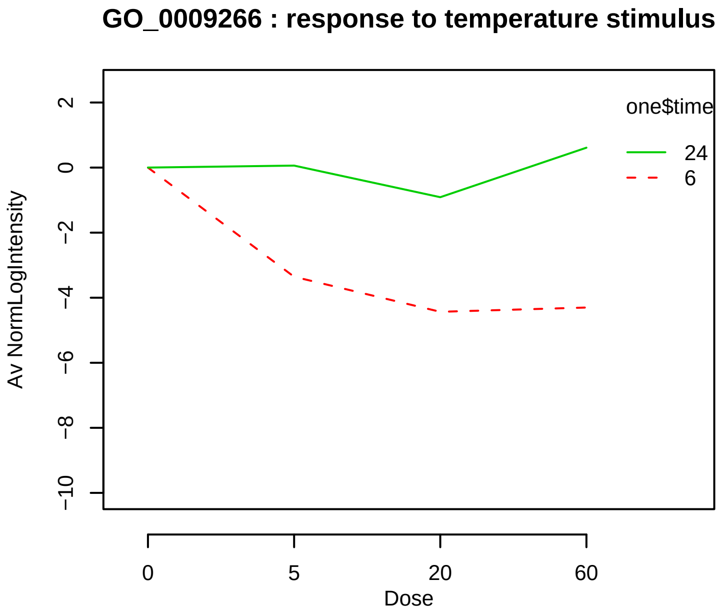
GO_0009266 : response to temperature stimulus
Av NormLogIntensity
2
0
−2
−4
−6
−8
−10
one$time
24
6
0
5
20
60
Dose

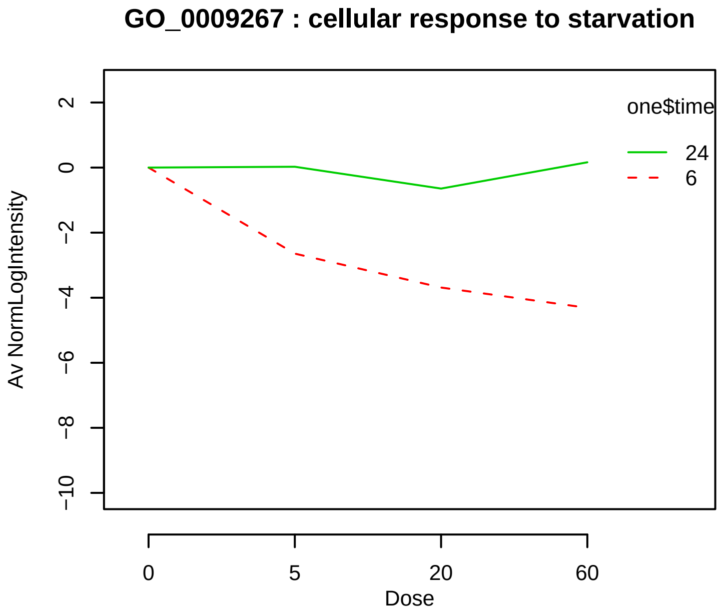
GO_0009267 : cellular response to starvation
Av NormLogIntensity
Dose
one$time
24
6
2
0
−2
−4
−6
−8
−10
0
5
20
60

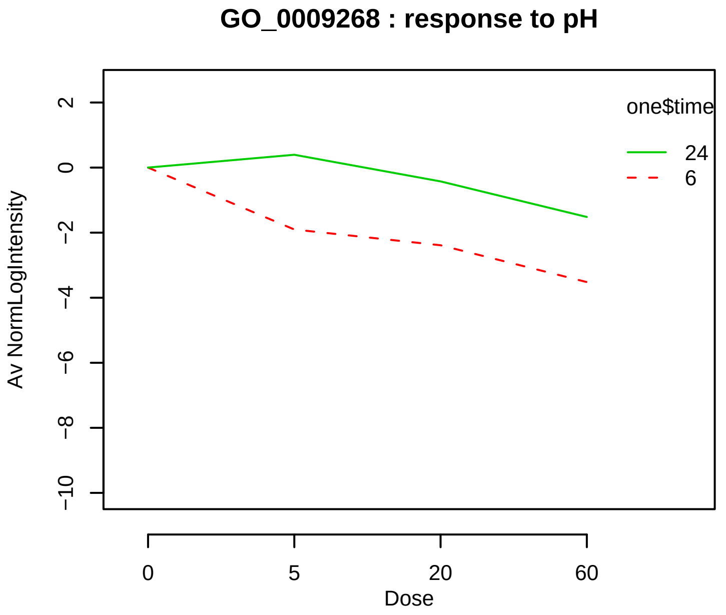
GO_0009268 : response to pH
Av NormLogIntensity
Dose
one$time
24
6
2
0
−2
−4
−6
−8
−10
0
5
20
60

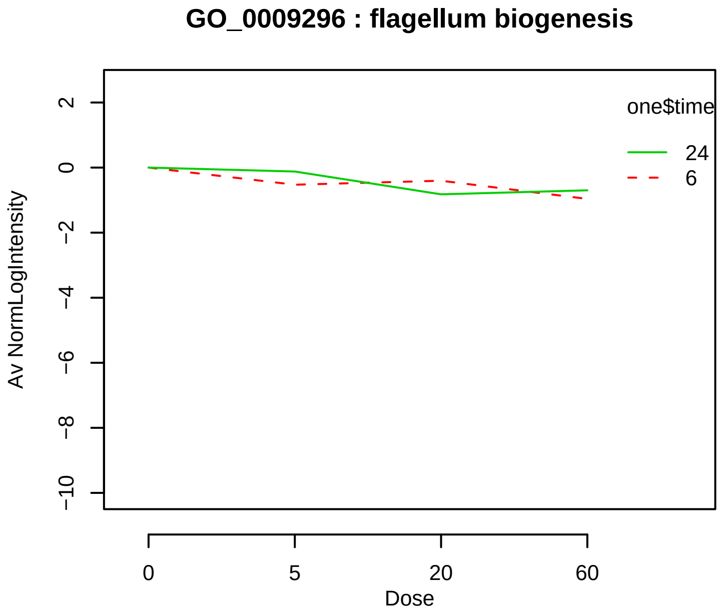
GO_0009296 : flagellum biogenesis
Av NormLogIntensity
Dose
one$time
24
6
2
0
−2
−4
−6
−8
−10
0
5
20
60

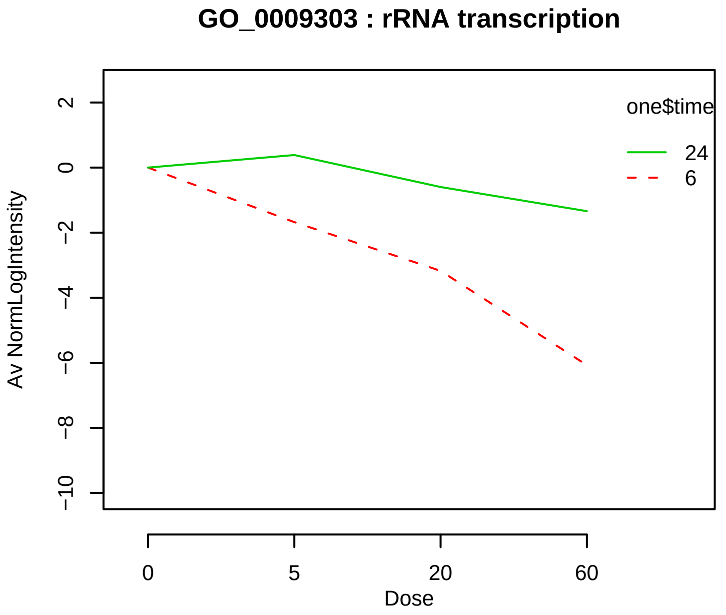
GO_0009303 : rRNA transcription
Av NormLogIntensity
Dose
one$time
24
6
2
0
−2
−4
−6
−8
−10
0
5
20
60

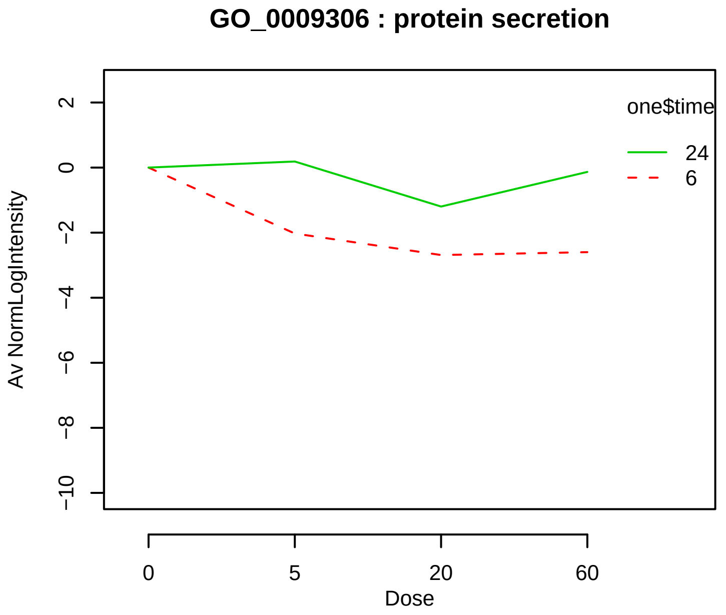
GO_0009306 : protein secretion
Av NormLogIntensity
Dose
one$time
24
6
2
0
−2
−4
−6
−8
−10
0
5
20
60

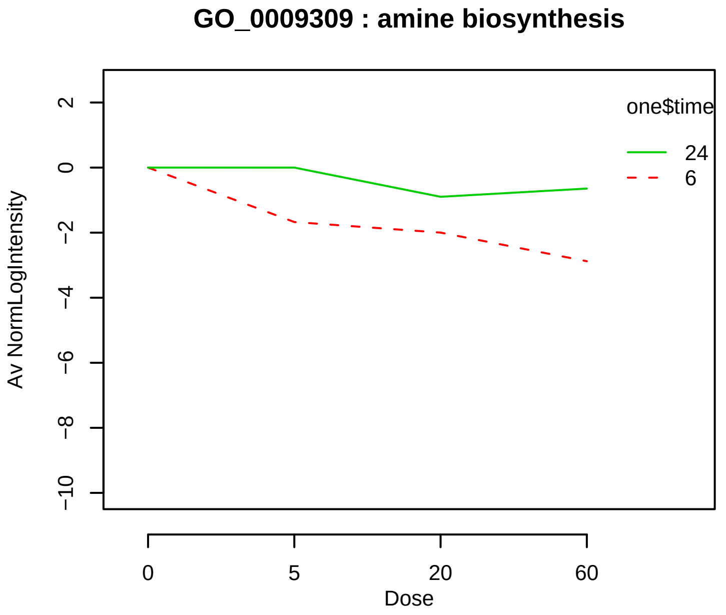
GO_0009309 : amine biosynthesis
Av NormLogIntensity
Dose
one$time
24
6
2
0
−2
−4
−6
−8
−10
0
5
20
60

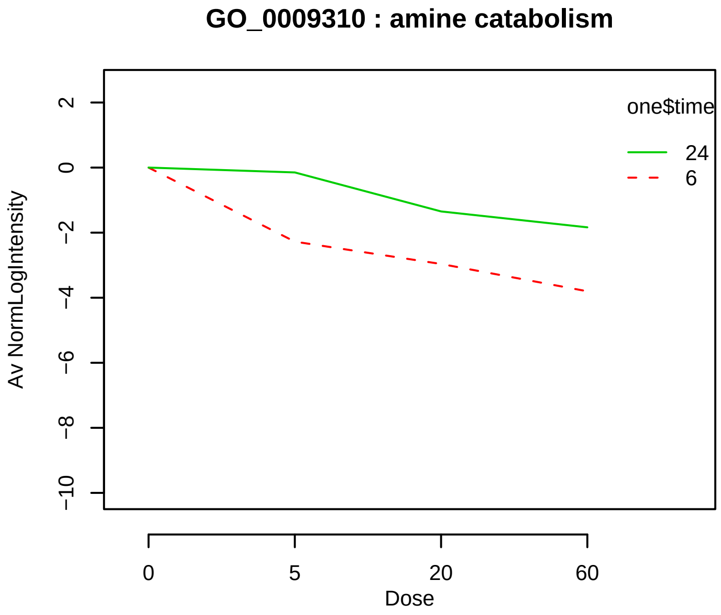
GO_0009310 : amine catabolism
Av NormLogIntensity
Dose
one$time
24
6
2
0
−2
−4
−6
−8
−10
0
5
20
60

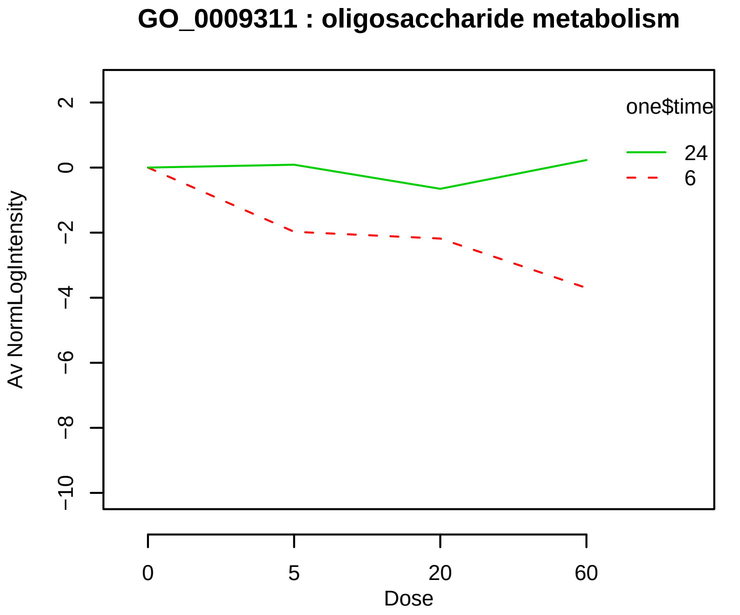
GO_0009311 : oligosaccharide metabolism
Av NormLogIntensity
Dose
one$time
24
6
2
0
−2
−4
−6
−8
−10
0
5
20
60

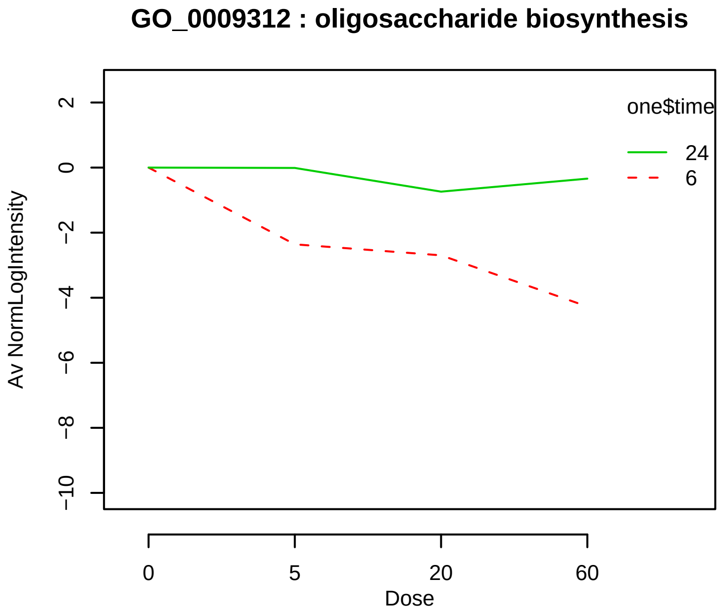
GO_0009312 : oligosaccharide biosynthesis
Av NormLogIntensity
Dose
one$time
24
6
2
0
−2
−4
−6
−8
−10
0
5
20
60

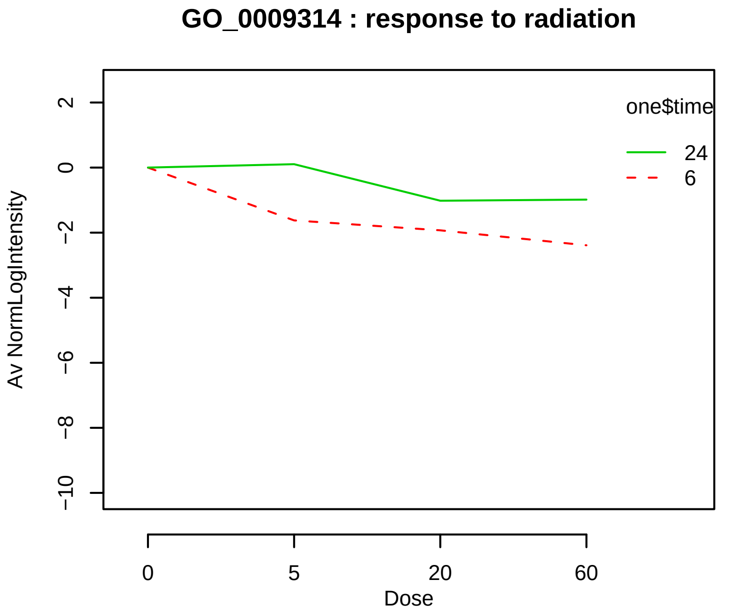
GO_0009314 : response to radiation
Av NormLogIntensity
Dose
one$time
24
6
2
0
−2
−4
−6
−8
−10
0
5
20
60

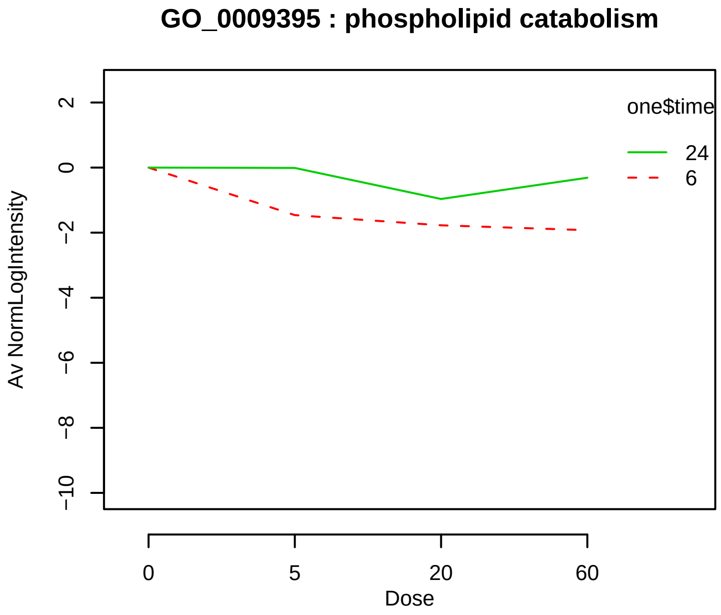
GO_0009395 : phospholipid catabolism
Av NormLogIntensity
Dose
one$time
24
6
2
0
−2
−4
−6
−8
−10
0
5
20
60

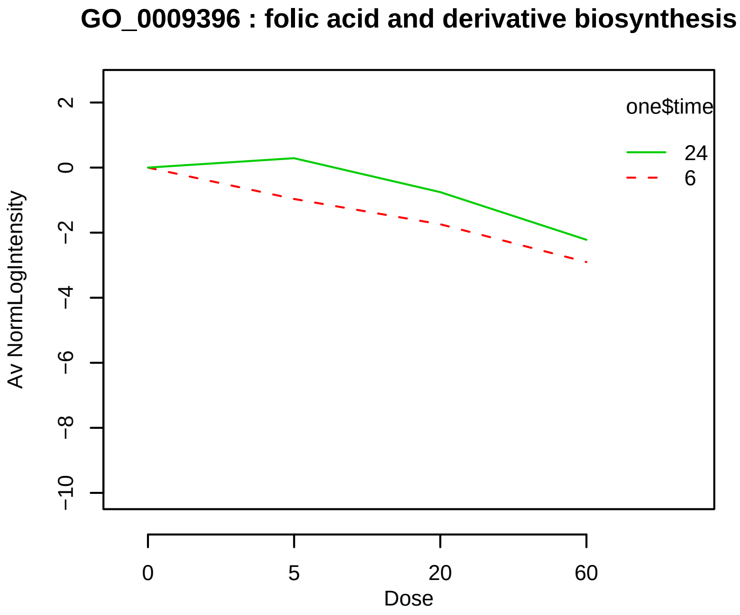
GO_0009396 : folic acid and derivative biosynthesis
Av NormLogIntensity
Dose
one$time
24
6
2
0
−2
−4
−6
−8
−10
0
5
20
60

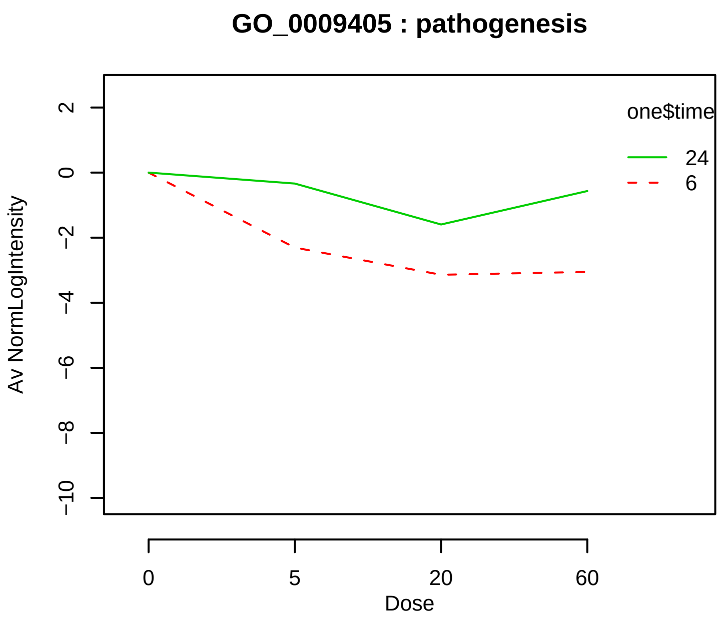
GO_0009405 : pathogenesis
Av NormLogIntensity
2
0
−2
−4
−6
−8
−10
0
5
20
60
Dose
one$time
24
6

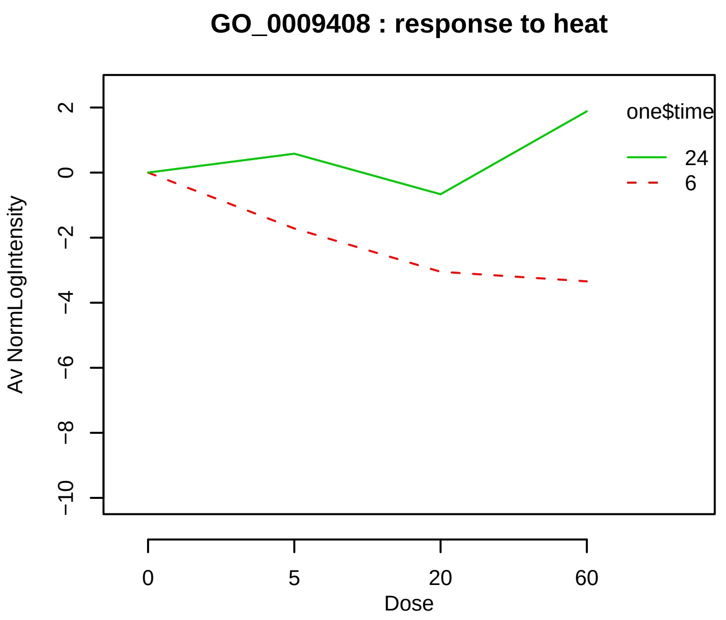
GO_0009408 : response to heat
Av NormLogIntensity
2
0
−2
−4
−6
−8
−10
0
5
20
60
Dose
one$time
24
6

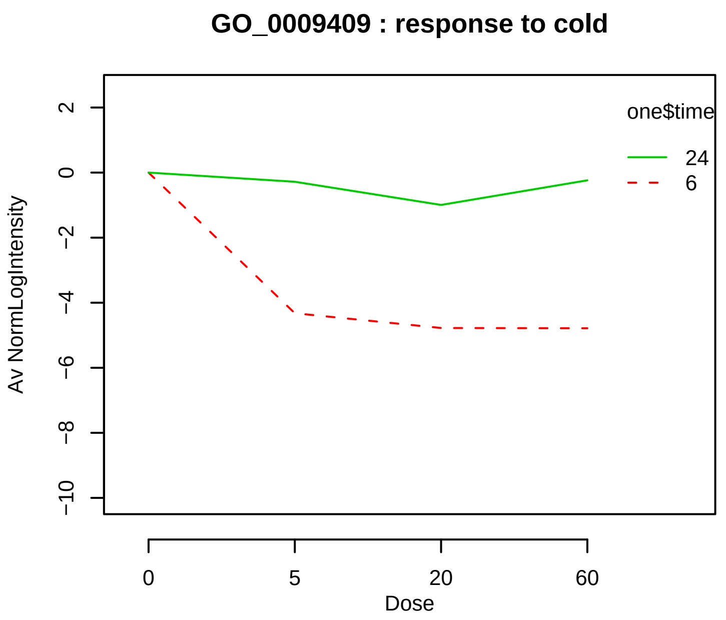
GO_0009409 : response to cold
Av NormLogIntensity
2
0
−2
−4
−6
−8
−10
0
5
20
60
Dose
one$time
24
6

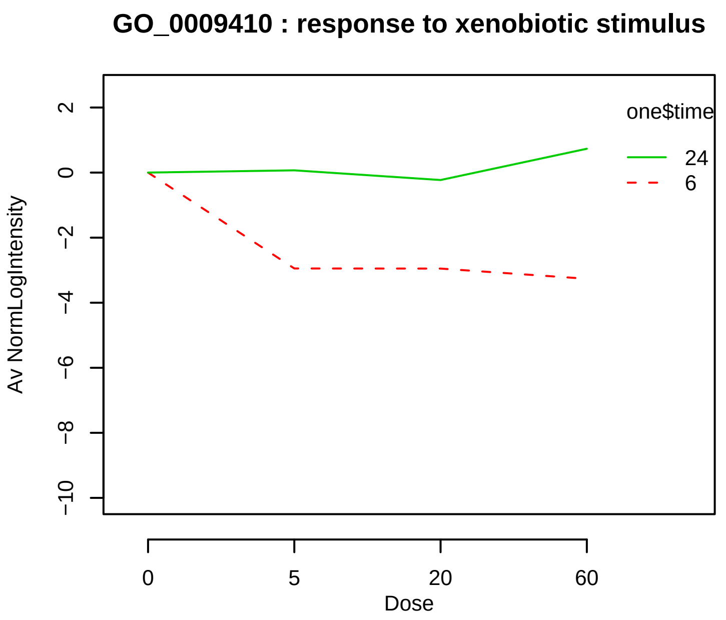
GO_0009410 : response to xenobiotic stimulus
Av NormLogIntensity
2
0
−2
−4
−6
−8
−10
0
5
20
60
Dose
one$time
24
6

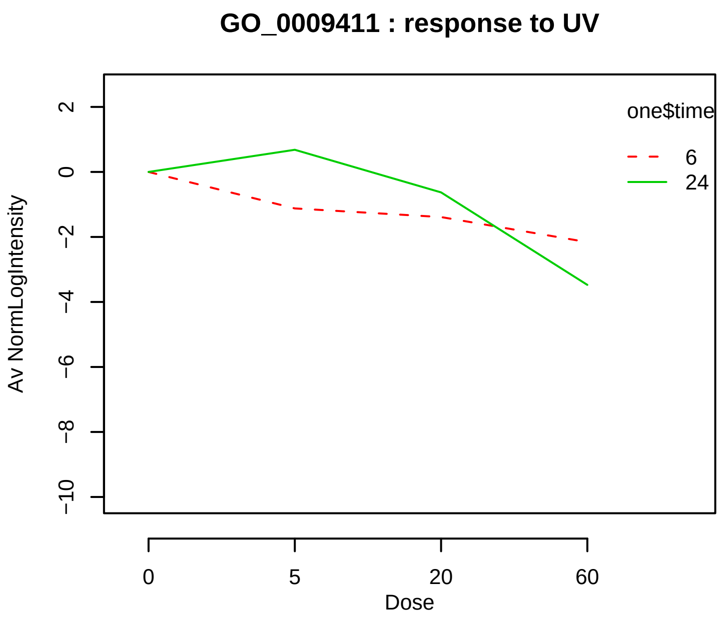
GO_0009411 : response to UV
Av NormLogIntensity
2
0
−2
−4
−6
−8
−10
0
5
20
60
Dose
one$time
6
24

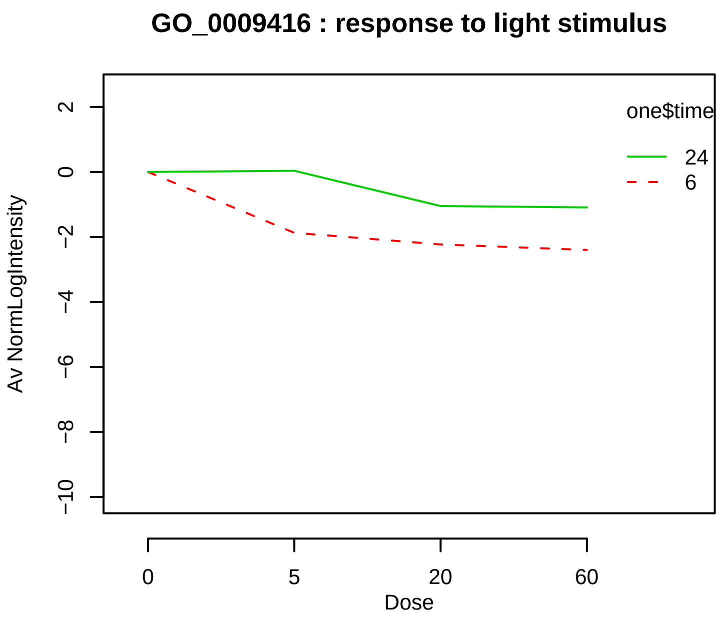
GO_0009416 : response to light stimulus
Av NormLogIntensity
2
0
−2
−4
−6
−8
−10
0
5
20
60
Dose
one$time
24
6

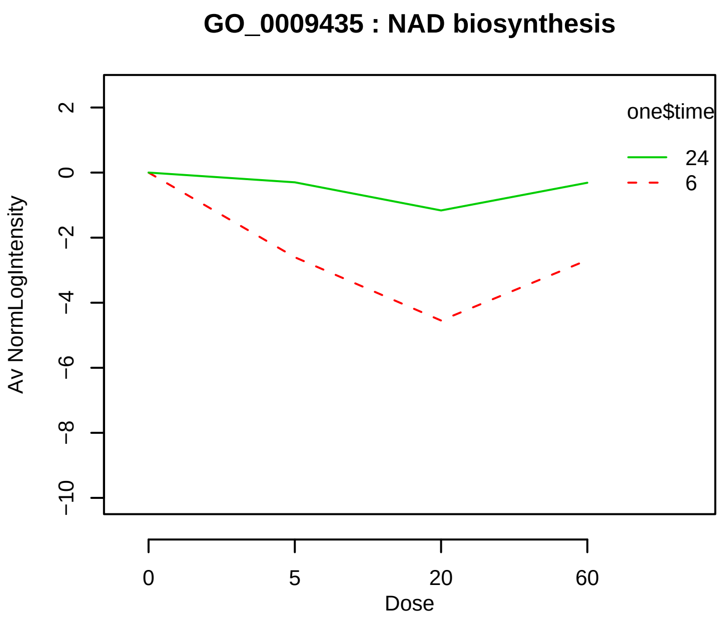
GO_0009435 : NAD biosynthesis
Av NormLogIntensity
2
0
−2
−4
−6
−8
−10
one$time
24
6
0
5
20
60
Dose

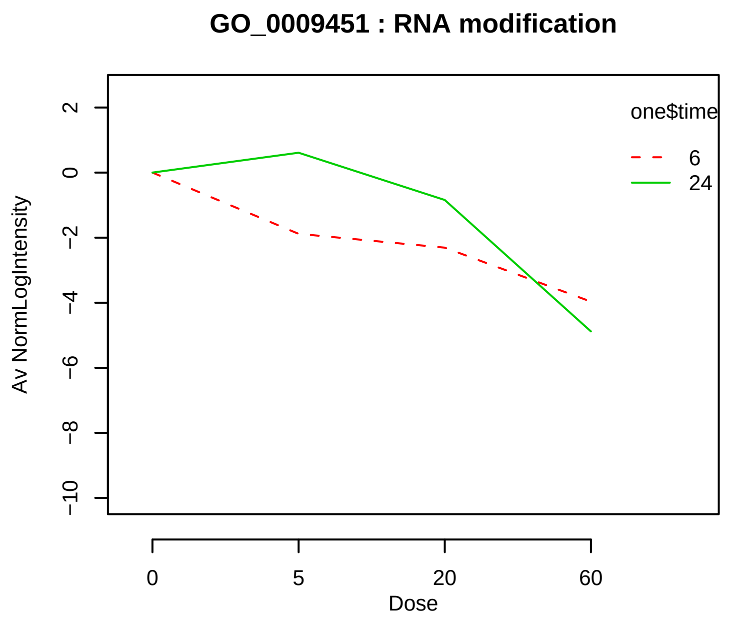
GO_0009451 : RNA modification
Av NormLogIntensity
2
0
−2
−4
−6
−8
−10
one$time
6
24
0
5
20
60
Dose

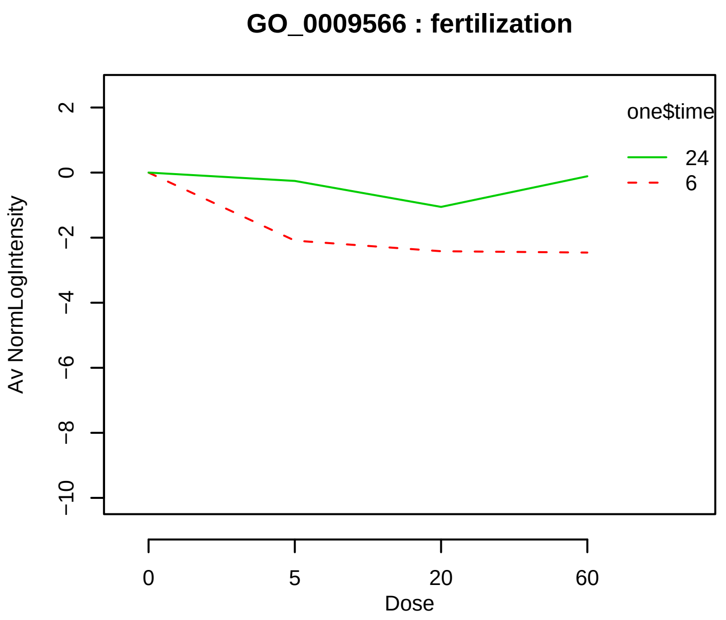
GO_0009566 : fertilization
Av NormLogIntensity
2
0
−2
−4
−6
−8
−10
one$time
24
6
0
5
20
60
Dose

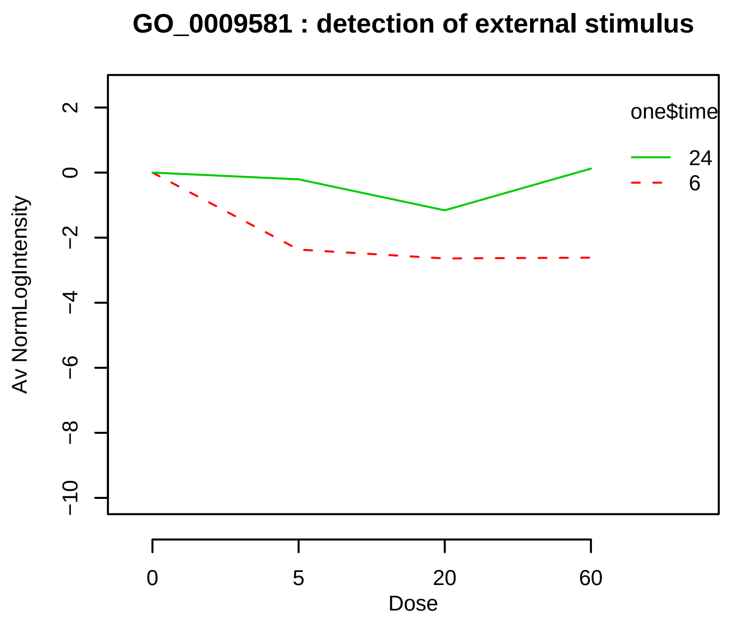
GO_0009581 : detection of external stimulus
Av NormLogIntensity
2
0
−2
−4
−6
−8
−10
one$time
24
6
0
5
20
60
Dose

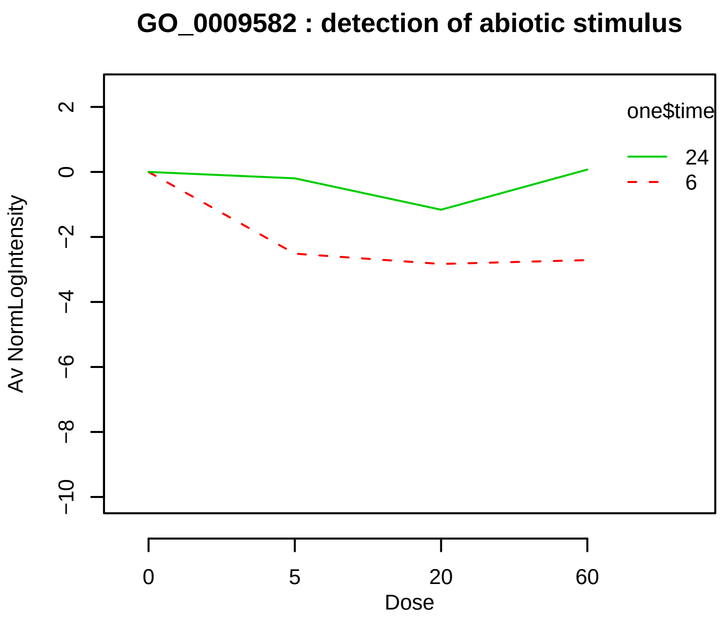
GO_0009582 : detection of abiotic stimulus
Av NormLogIntensity
2
0
−2
−4
−6
−8
−10
one$time
24
6
0
5
20
60
Dose

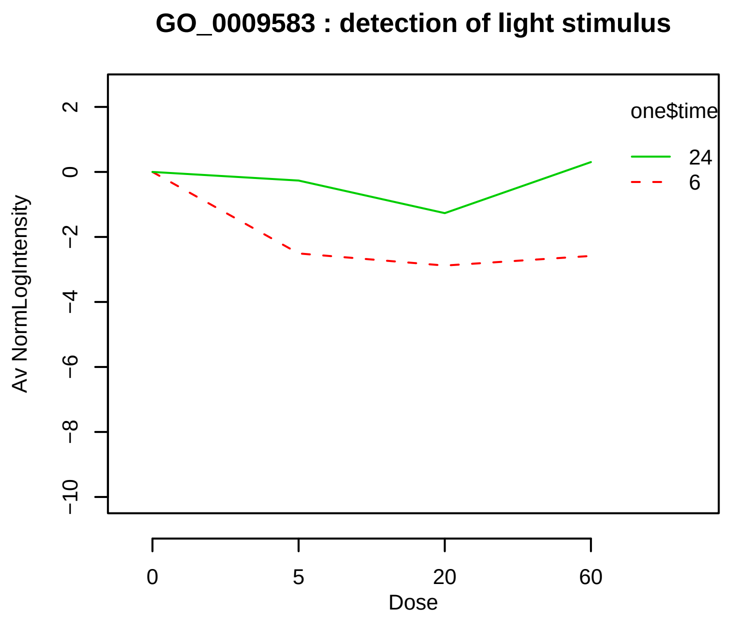
GO_0009583 : detection of light stimulus
Av NormLogIntensity
2
0
−2
−4
−6
−8
−10
one$time
24
6
0
5
20
60
Dose

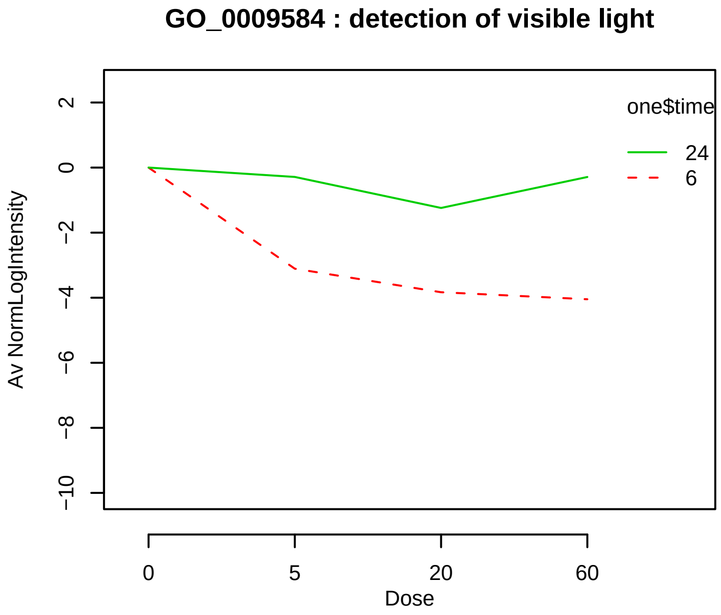
GO_0009584 : detection of visible light
Av NormLogIntensity
2
0
−2
−4
−6
−8
−10
one$time
24
6
0
5
20
60
Dose

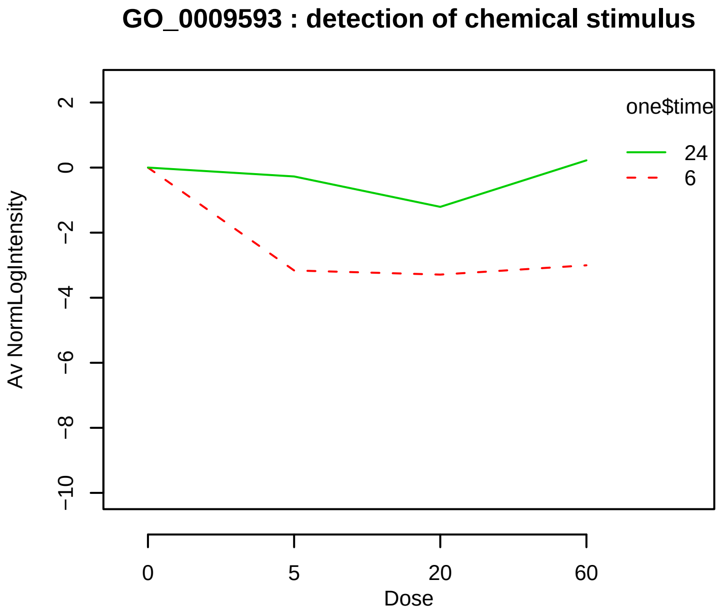
GO_0009593 : detection of chemical stimulus
Av NormLogIntensity
2
0
−2
−4
−6
−8
−10
one$time
24
6
0
5
20
60
Dose

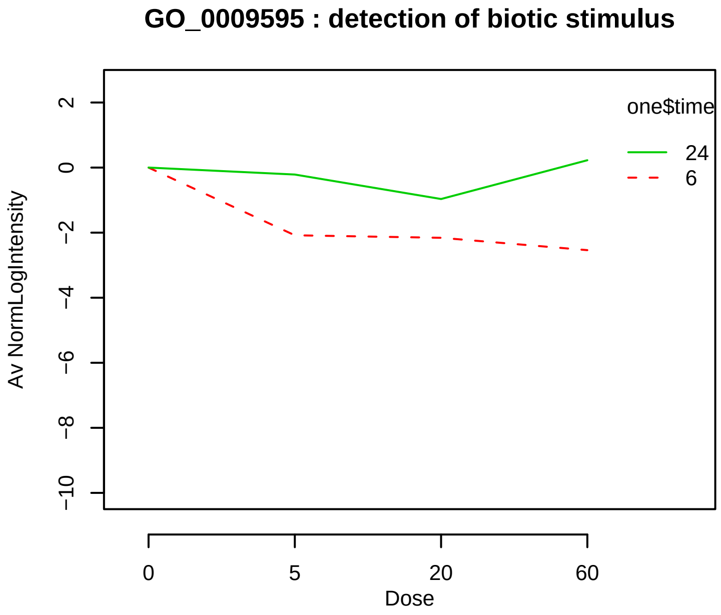
GO_0009595 : detection of biotic stimulus
Av NormLogIntensity
2
0
−2
−4
−6
−8
−10
one$time
24
6
0
5
20
60
Dose

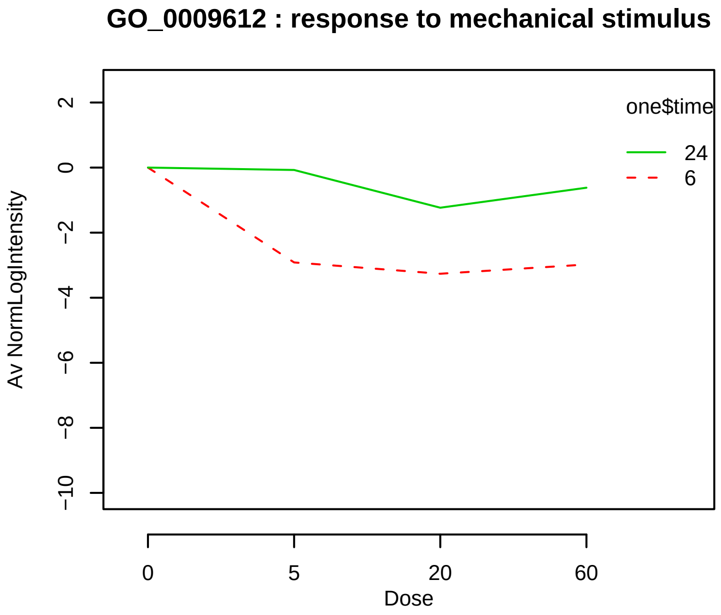
GO_0009612 : response to mechanical stimulus
Av NormLogIntensity
2
0
−2
−4
−6
−8
−10
one$time
24
6
0
5
20
60
Dose

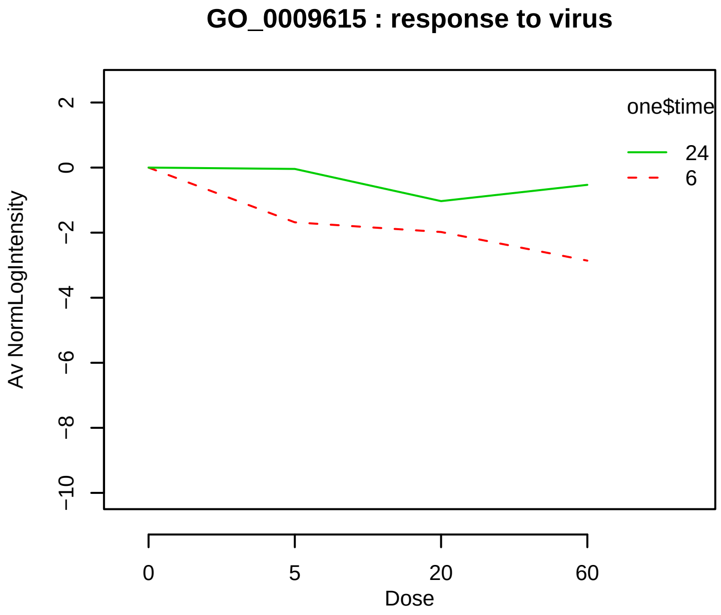
GO_0009615 : response to virus
Av NormLogIntensity
2
0
−2
−4
−6
−8
−10
one$time
24
6
0
5
20
60
Dose

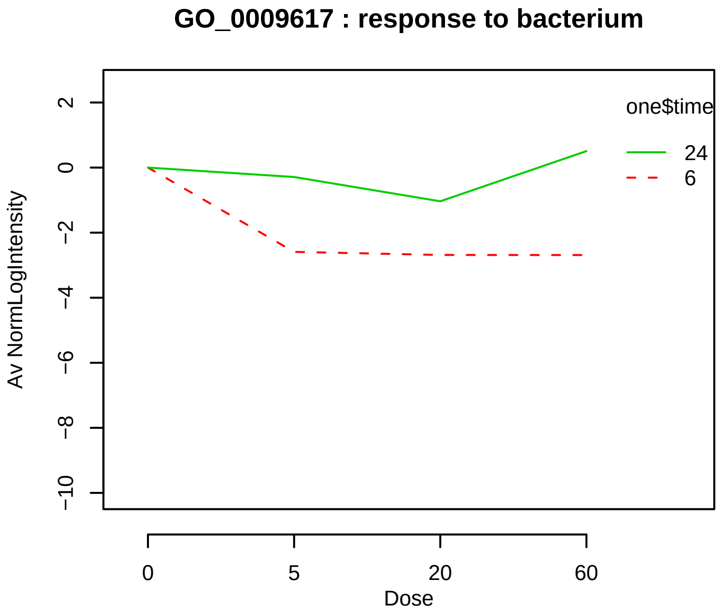
GO_0009617 : response to bacterium
Av NormLogIntensity
2
0
−2
−4
−6
−8
−10
one$time
24
6
0
5
20
60
Dose

GO_0009620 : response to fungus

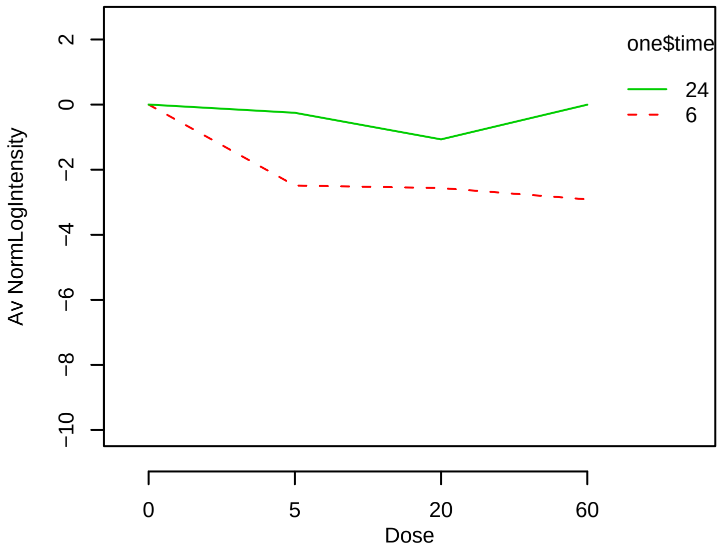

GO_0009636 : response to toxin

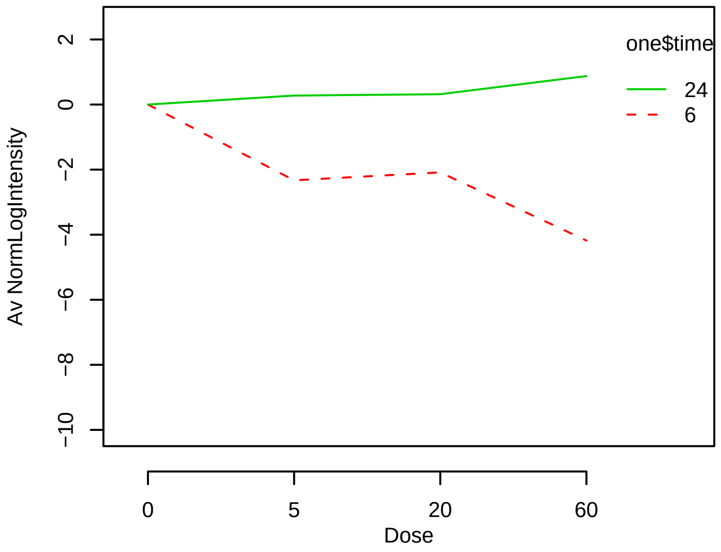

GO_0009725 : response to hormone stimulus

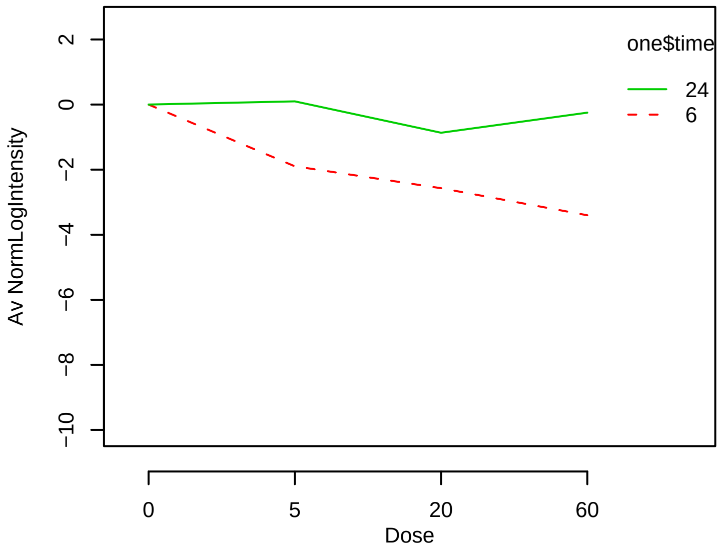

GO_0009743 : response to carbohydrate stimulus

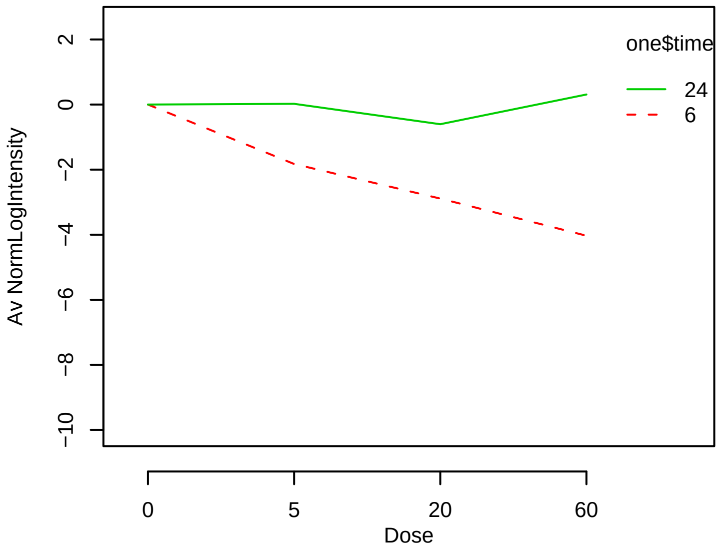

GO_0009746 : response to hexose stimulus

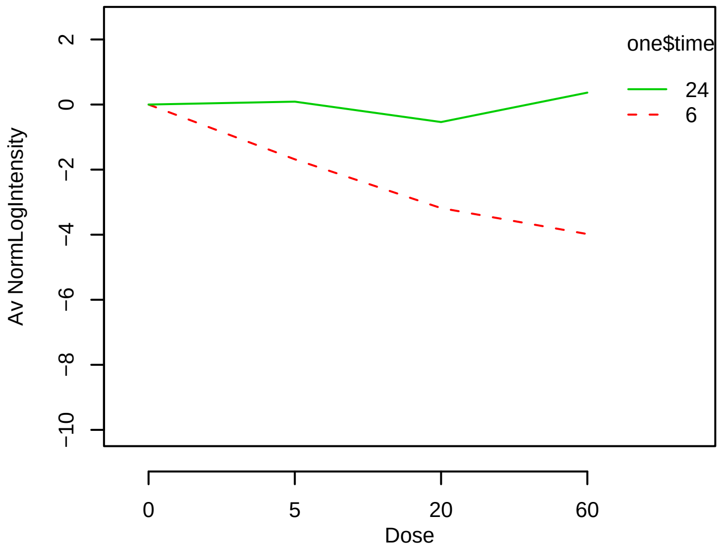

GO_0009749 : response to glucose stimulus

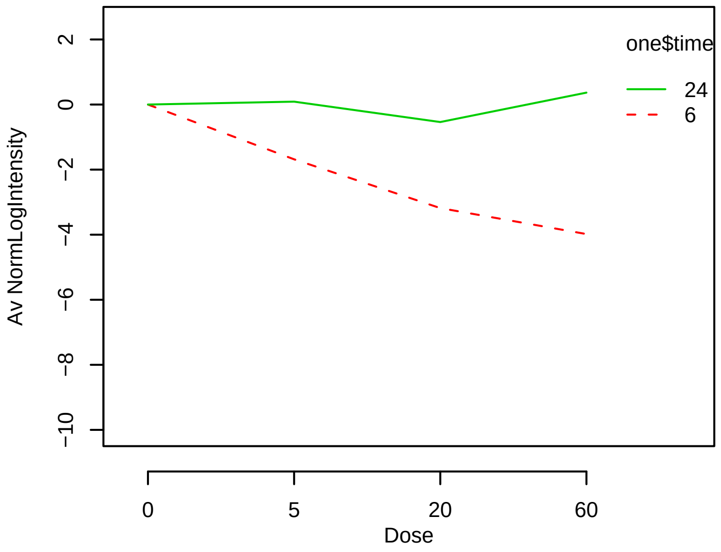

GO_0009755 : hormone−mediated signaling

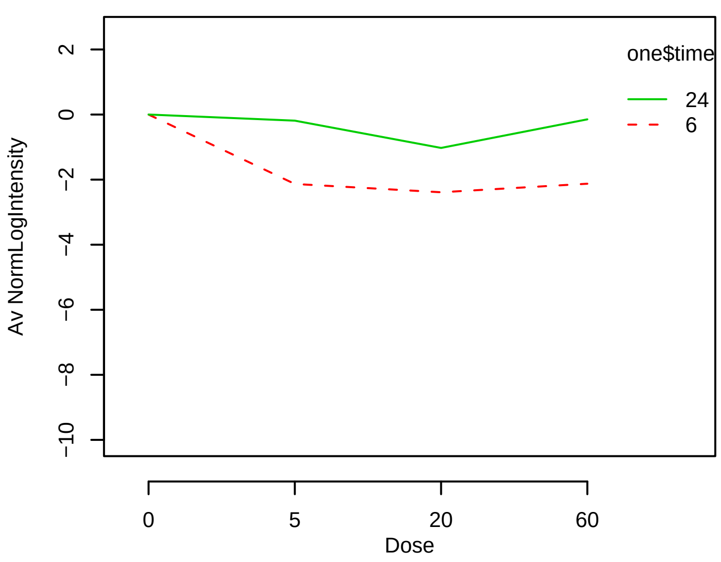

GO_0009791 : post−embryonic development

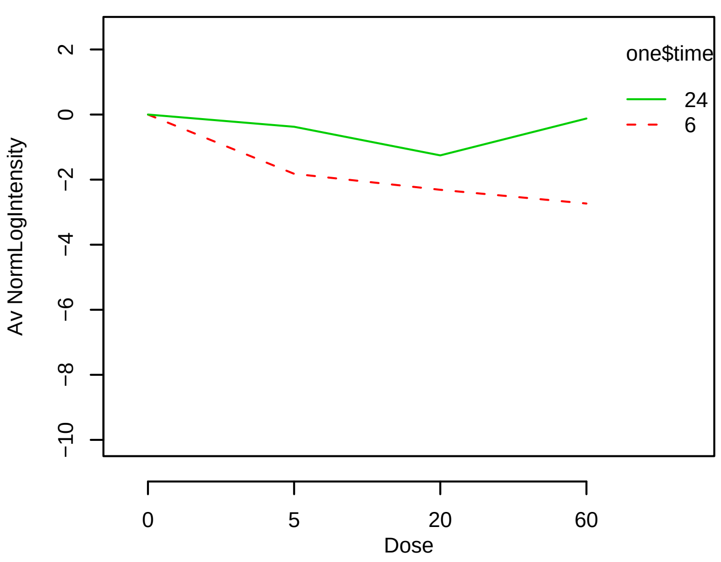

GO_0009792 : embryonic development (sensu Metazoa)

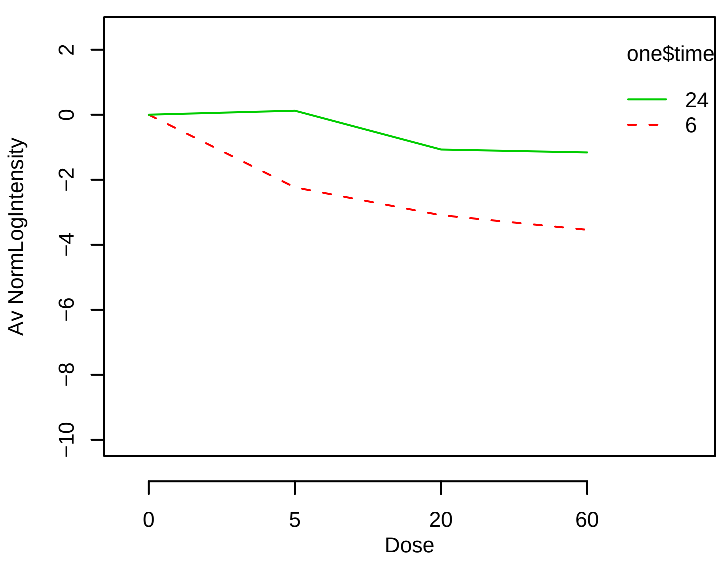

GO_0009798 : axis specification

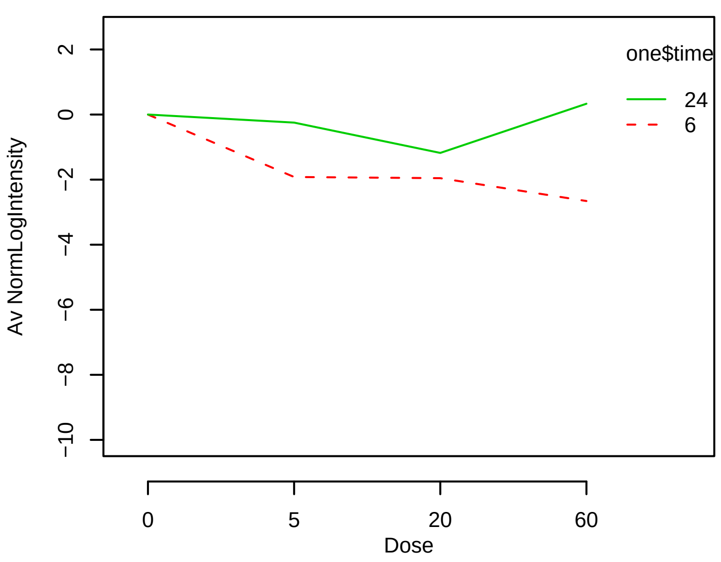

GO_0009799 : determination of symmetry

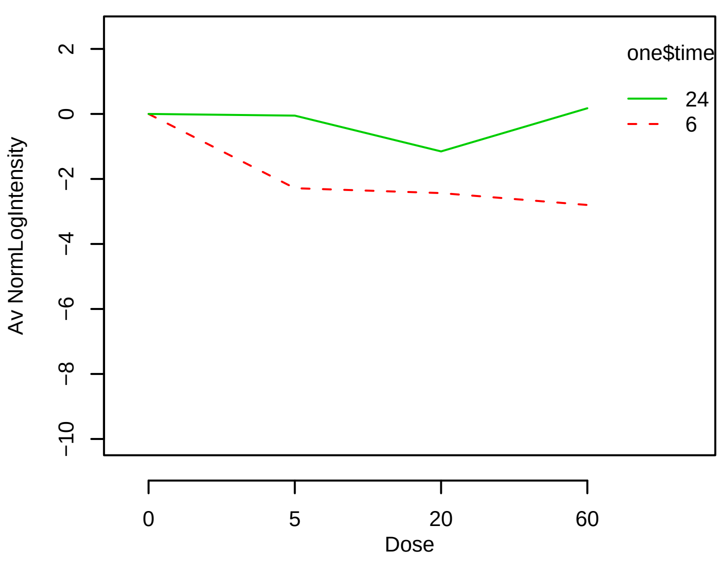

GO_0009855 : determination of bilateral symmetry

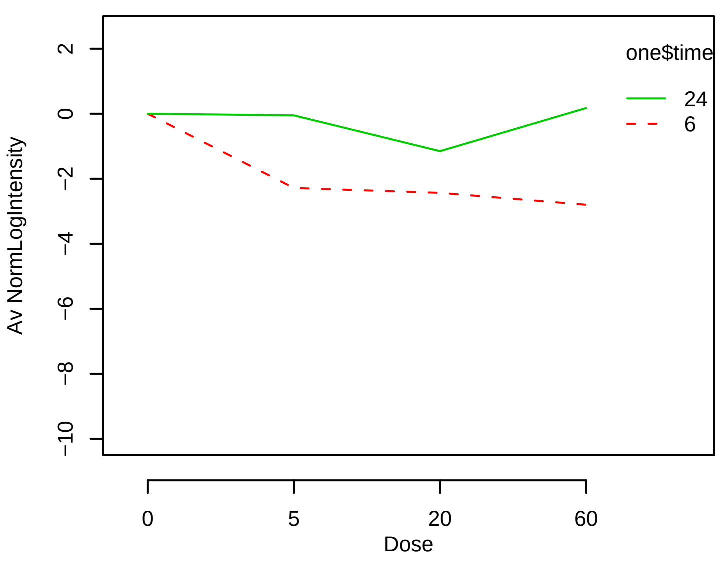

GO_0009880 : embryonic pattern specification

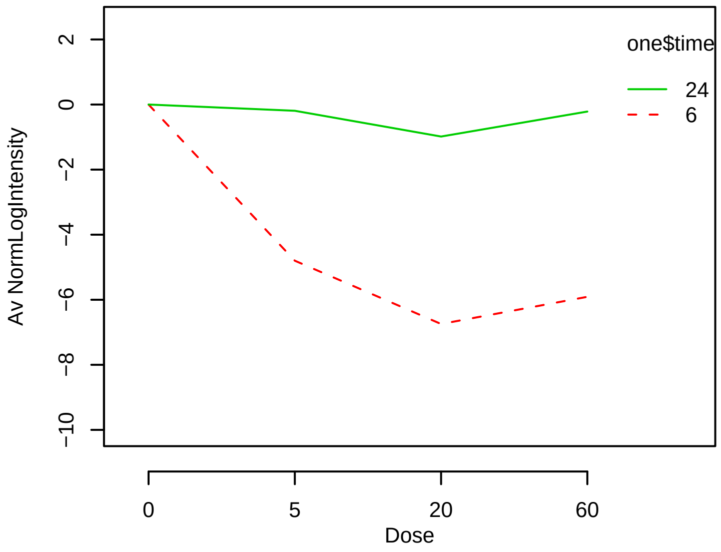

GO_0009889 : regulation of biosynthesis

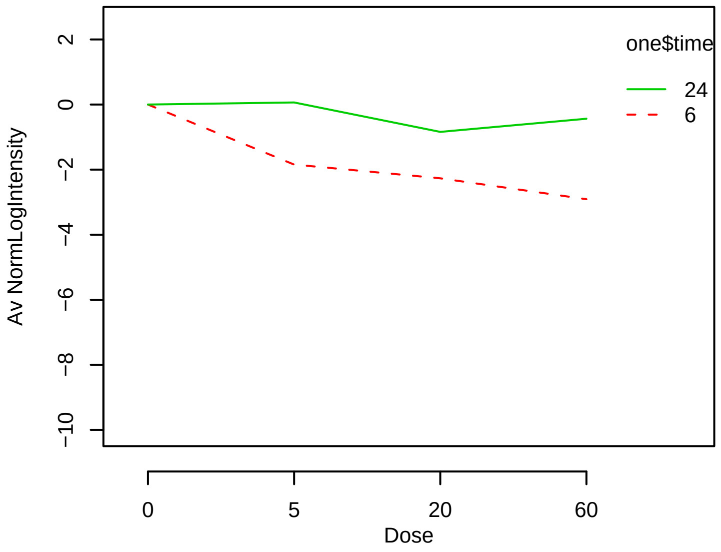

GO_0009890 : negative regulation of biosynthesis

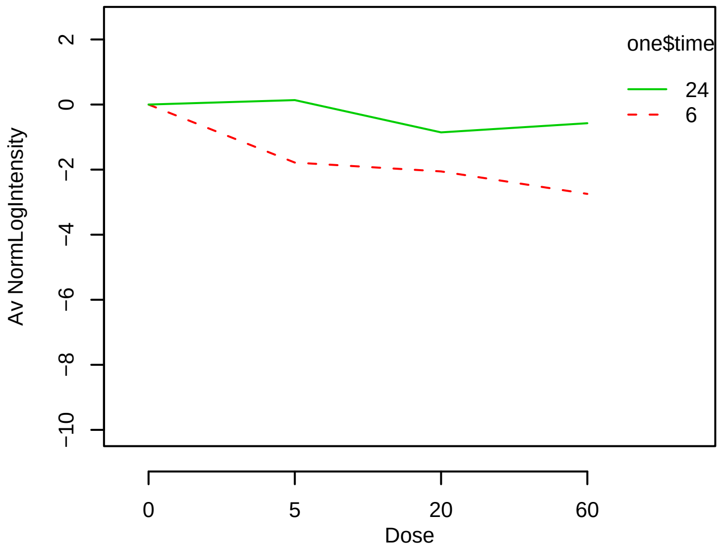

GO_0009891 : positive regulation of biosynthesis

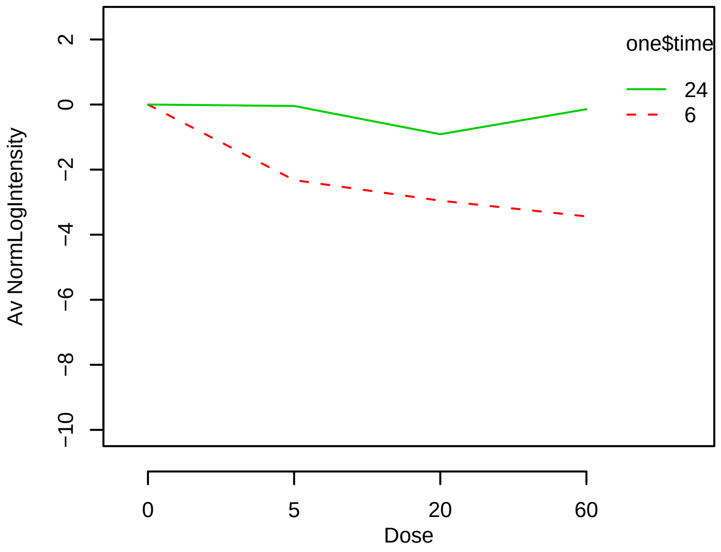

GO_0009894 : regulation of catabolism

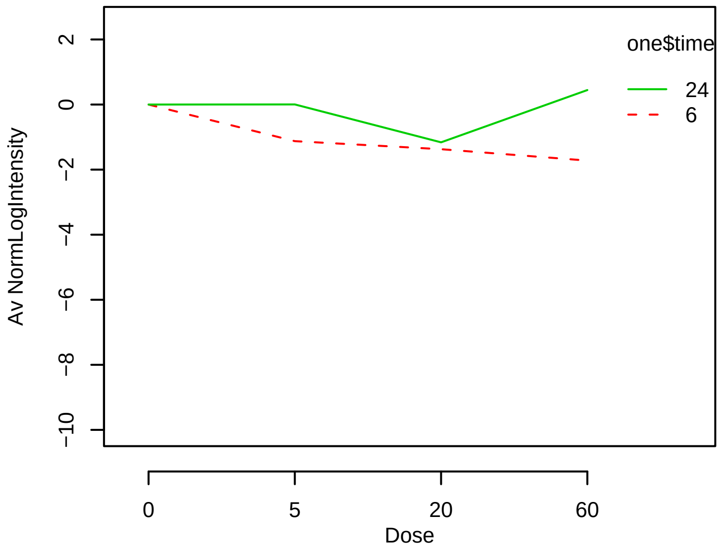

GO_0009895 : negative regulation of catabolism

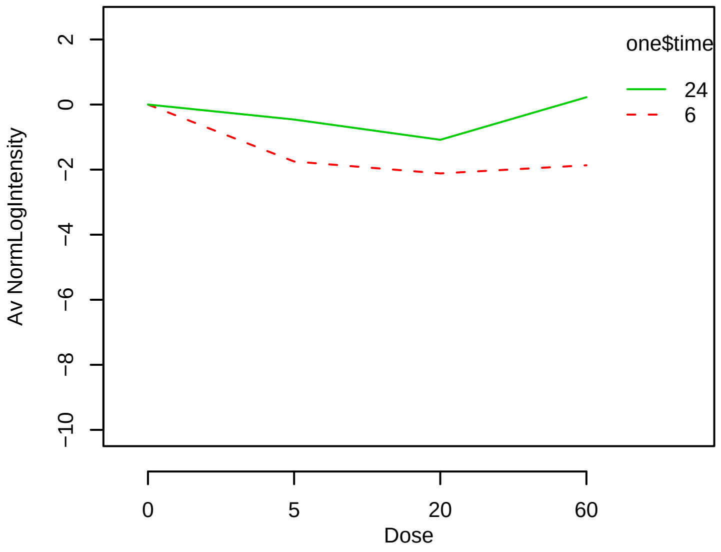

GO_0009912 : auditory receptor cell fate commitment

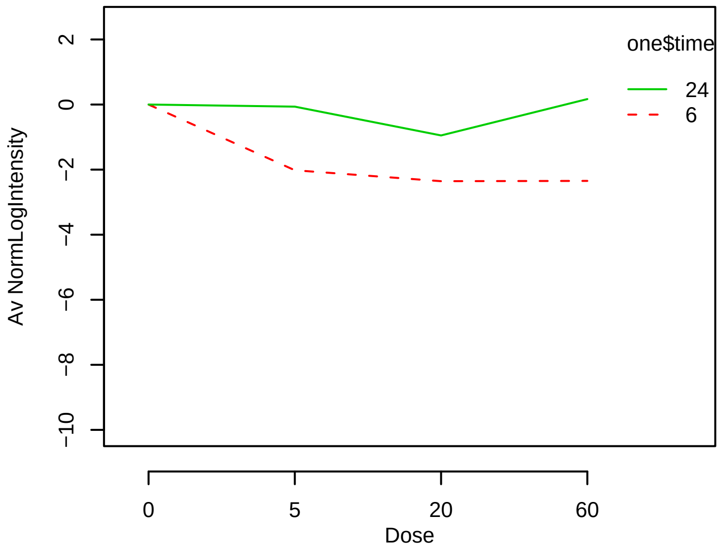

GO_0009913 : epidermal cell differentiation

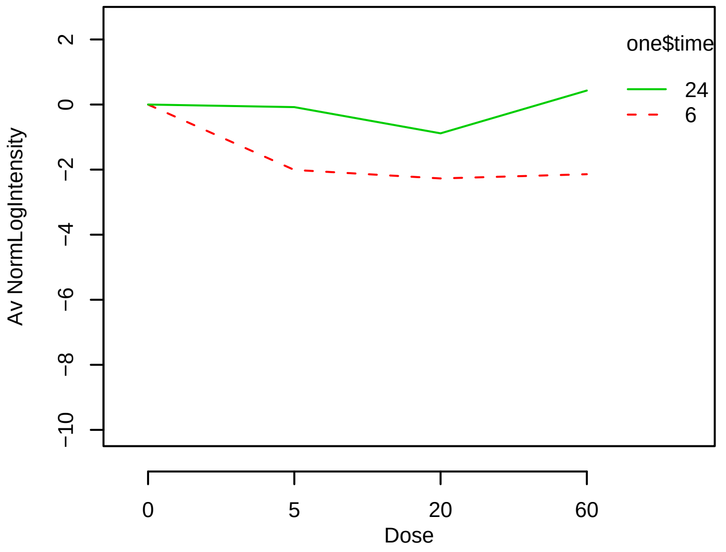

GO_0009948 : anterior/posterior axis specification

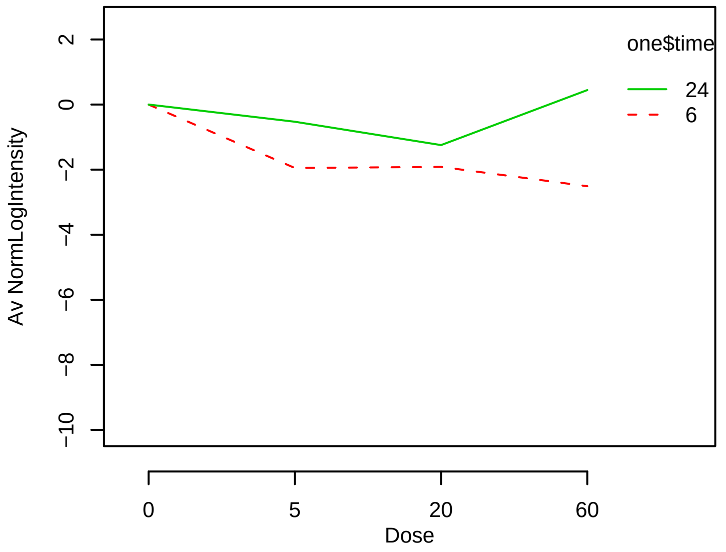

GO_0009952 : anterior/posterior pattern formation

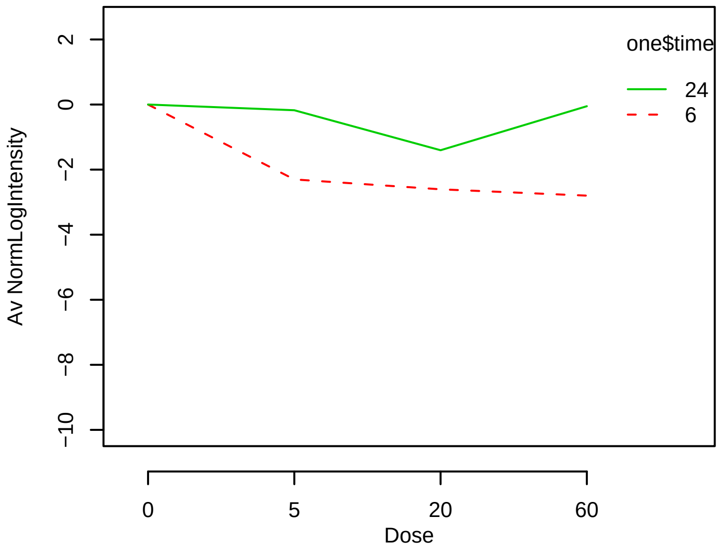

GO_0009953 : dorsal/ventral pattern formation

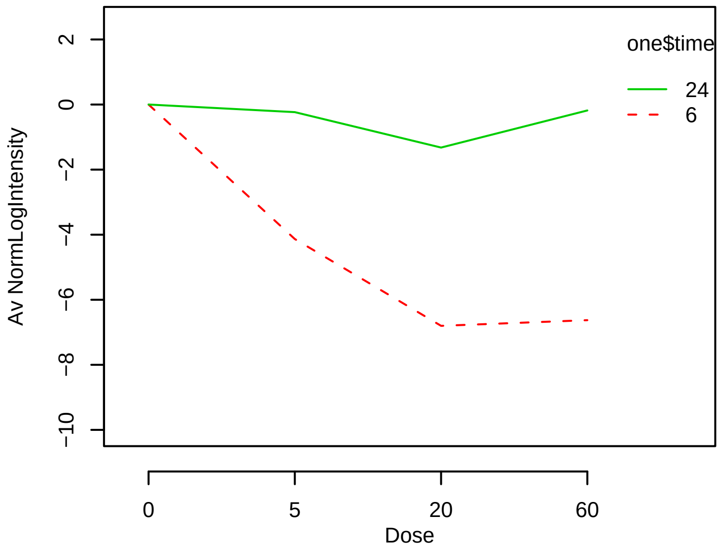

GO_0009954 : proximal/distal pattern formation

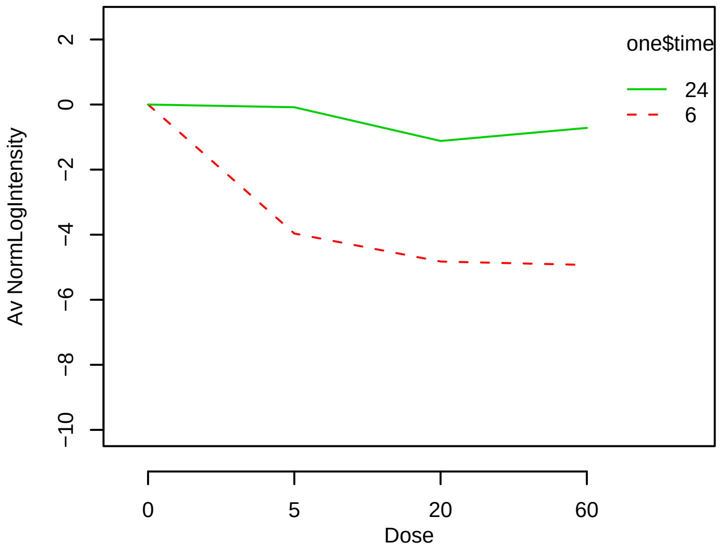

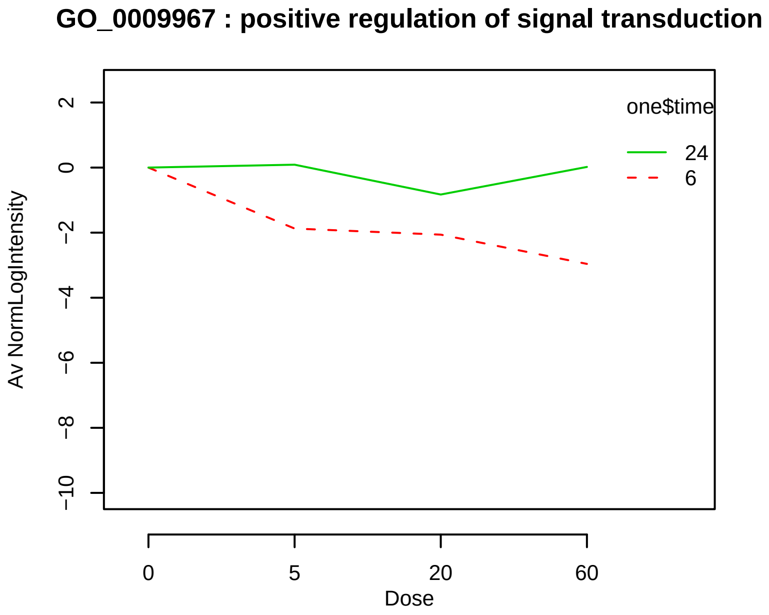
GO_0009967 : positive regulation of signal transduction
one$time
24
6
Av NormLogIntensity
2
0
−2
−4
−6
−8
−10
0
5
20
60
Dose

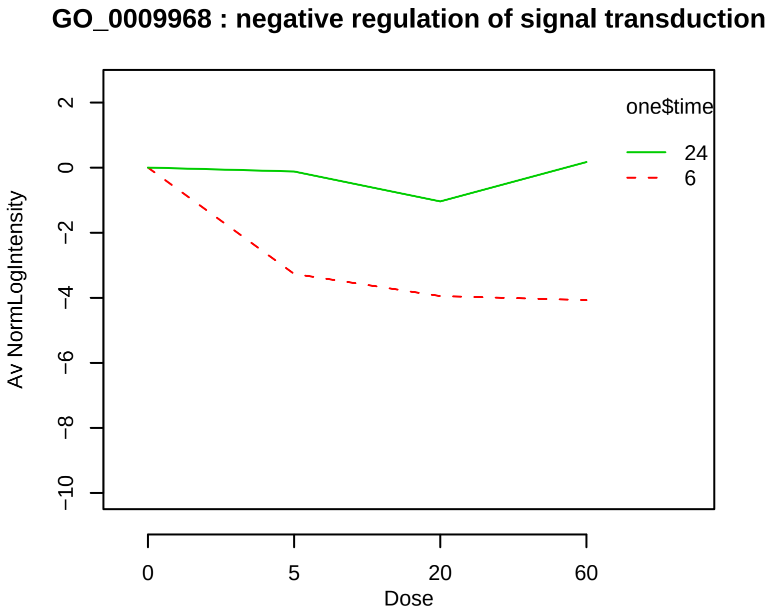
GO_0009968 : negative regulation of signal transduction
one$time
24
6
Av NormLogIntensity
2
0
−2
−4
−6
−8
−10
0
5
20
60
Dose

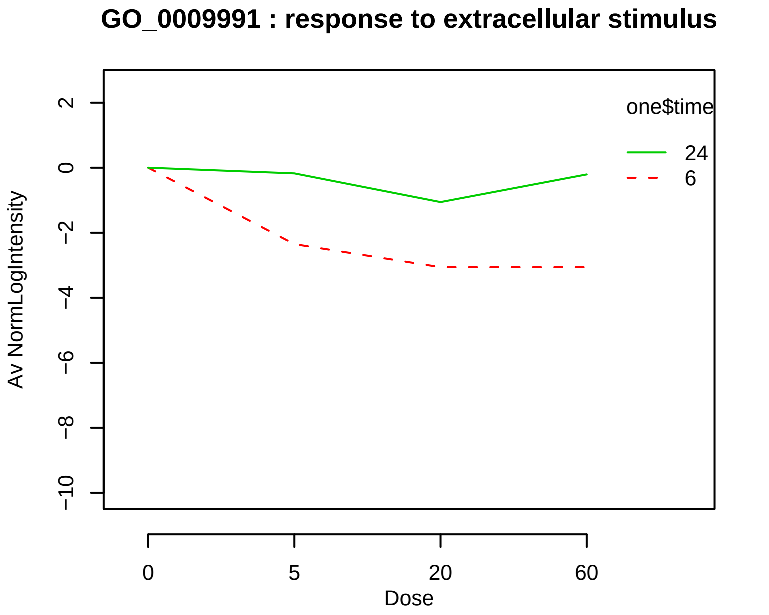
GO_0009991 : response to extracellular stimulus
one$time
24
6
Av NormLogIntensity
2
0
−2
−4
−6
−8
−10
0
5
20
60
Dose

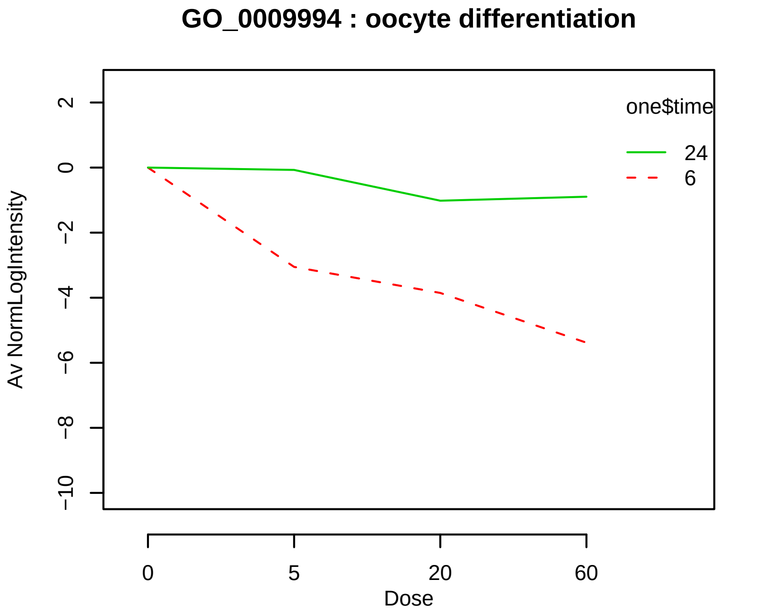
GO_0009994 : oocyte differentiation
one$time
24
6
Av NormLogIntensity
2
0
−2
−4
−6
−8
−10
0
5
20
60
Dose

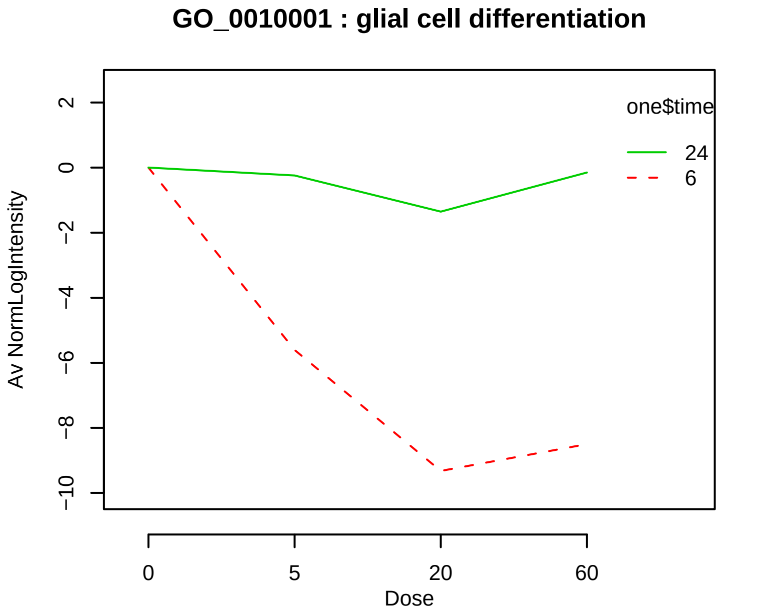
GO_0010001 : glial cell differentiation
one$time
24
6
Av NormLogIntensity
2
0
−2
−4
−6
−8
−10
0
5
20
60
Dose

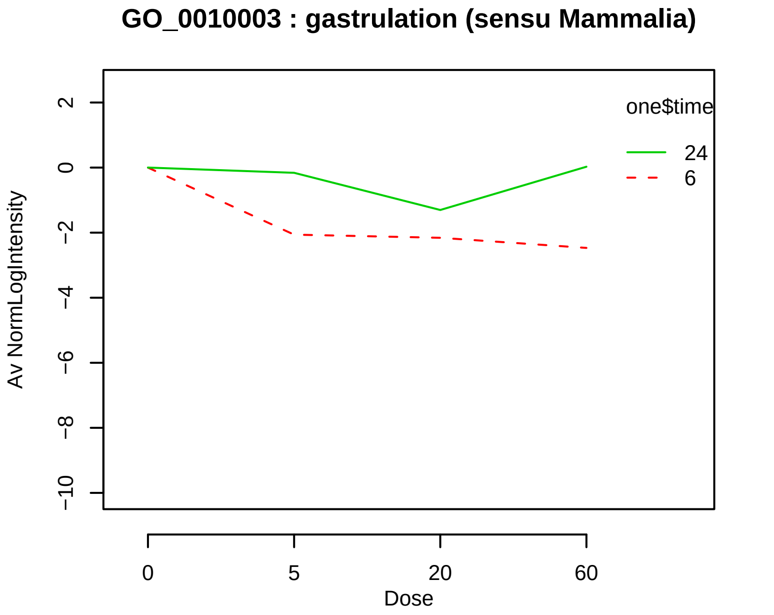
GO_0010003 : gastrulation (sensu Mammalia)
one$time
24
6
Av NormLogIntensity
2
0
−2
−4
−6
−8
−10
0
5
20
60
Dose

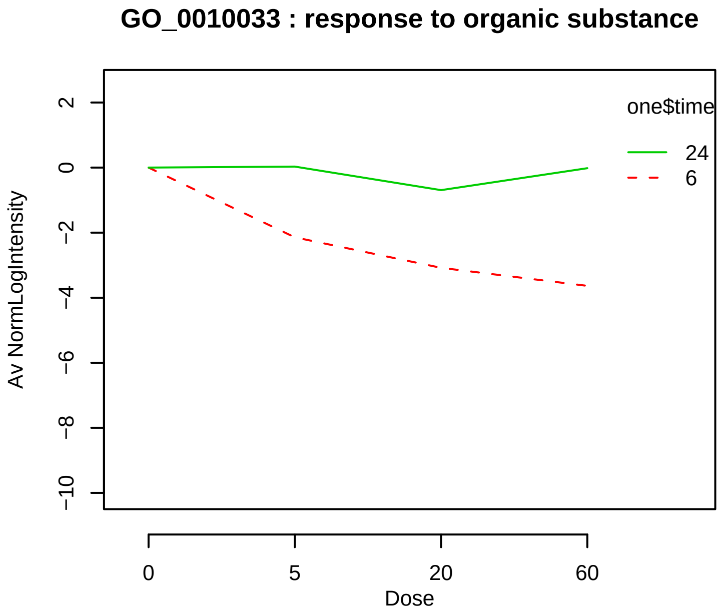
GO_0010033 : response to organic substance
one$time
24
6
Av NormLogIntensity
Dose
2
0
−2
−4
−6
−8
−10
0
5
20
60

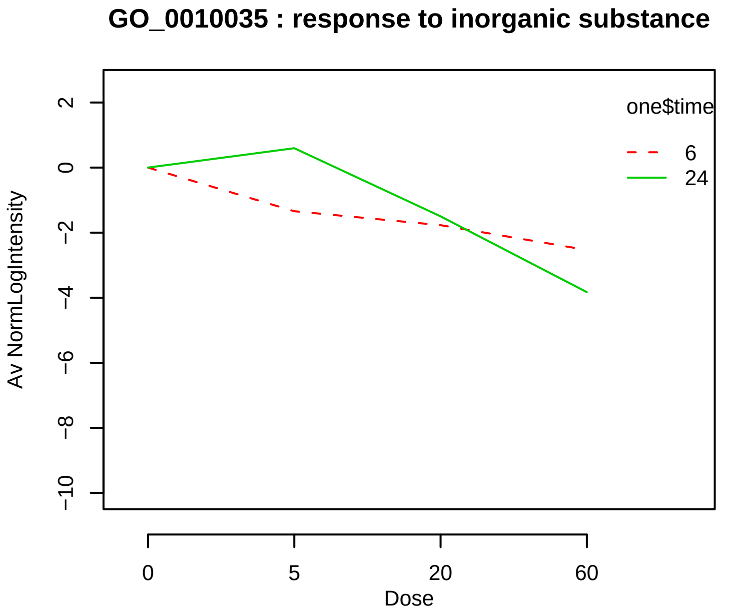
GO_0010035 : response to inorganic substance
one$time
6
24
Av NormLogIntensity
Dose
2
0
−2
−4
−6
−8
−10
0
5
20
60

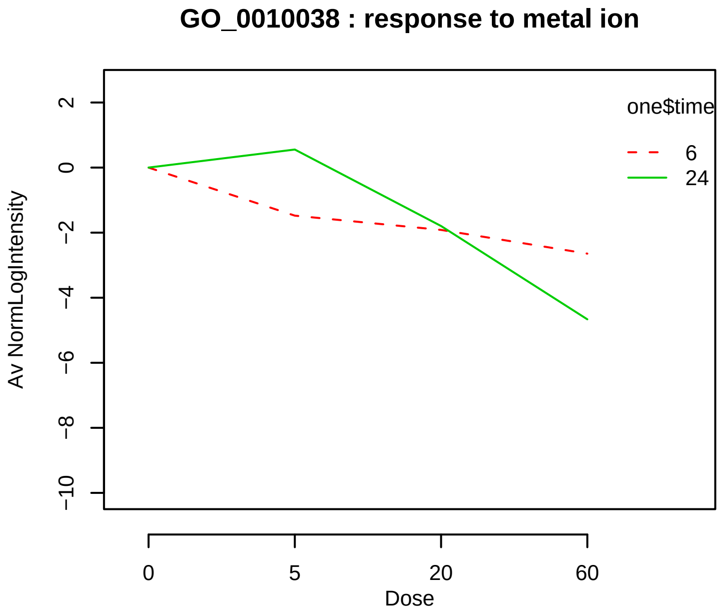
GO_0010038 : response to metal ion
one$time
6
24
Av NormLogIntensity
Dose
2
0
−2
−4
−6
−8
−10
0
5
20
60

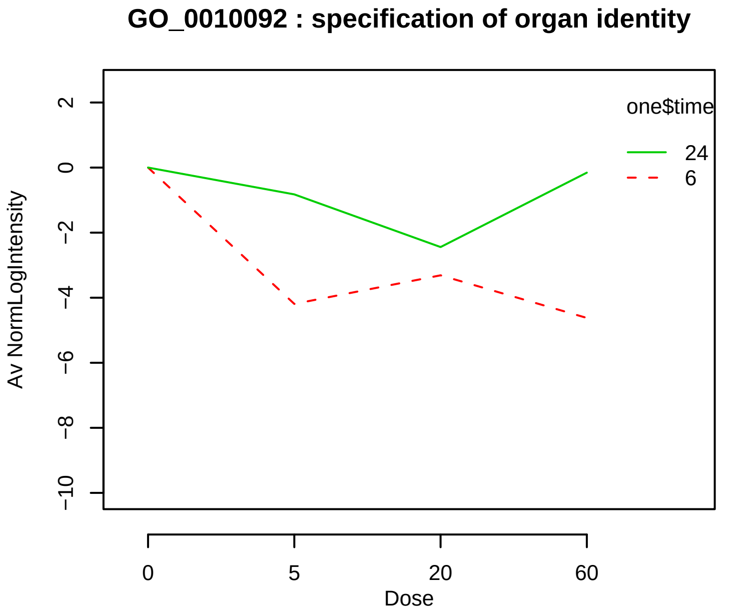
GO_0010092 : specification of organ identity
one$time
24
6
Av NormLogIntensity
Dose
2
0
−2
−4
−6
−8
−10
0
5
20
60

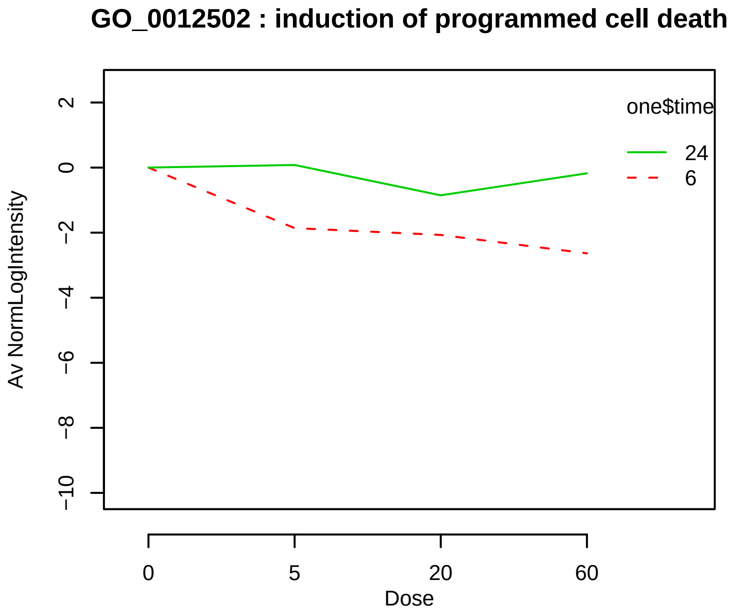
GO_0012502 : induction of programmed cell death
one$time
24
6
Av NormLogIntensity
Dose
2
0
−2
−4
−6
−8
−10
0
5
20
60

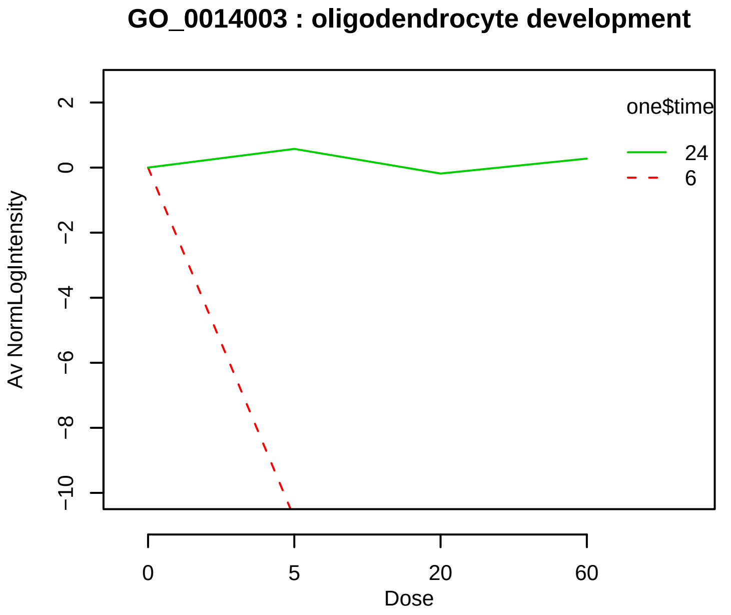
GO_0014003 : oligodendrocyte development
one$time
24
6
Av NormLogIntensity
Dose
2
0
−2
−4
−6
−8
−10
0
5
20
60

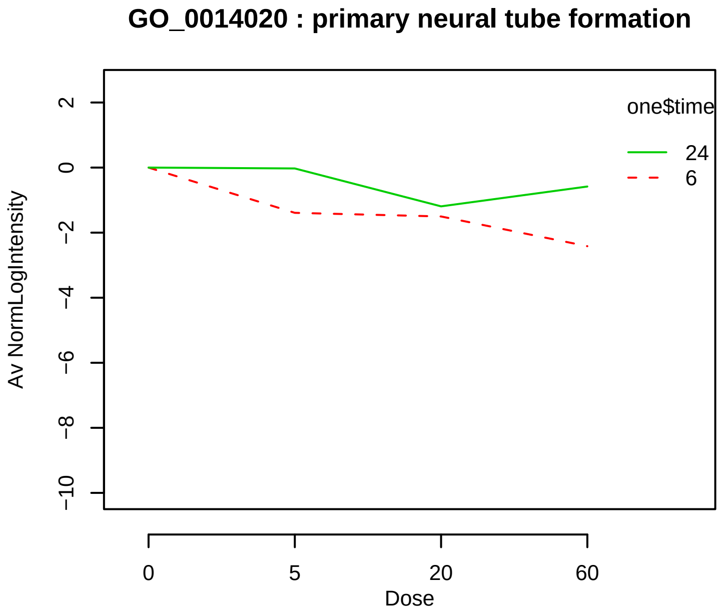
GO_0014020 : primary neural tube formation
one$time
24
6
Av NormLogIntensity
Dose
0
5
20
60

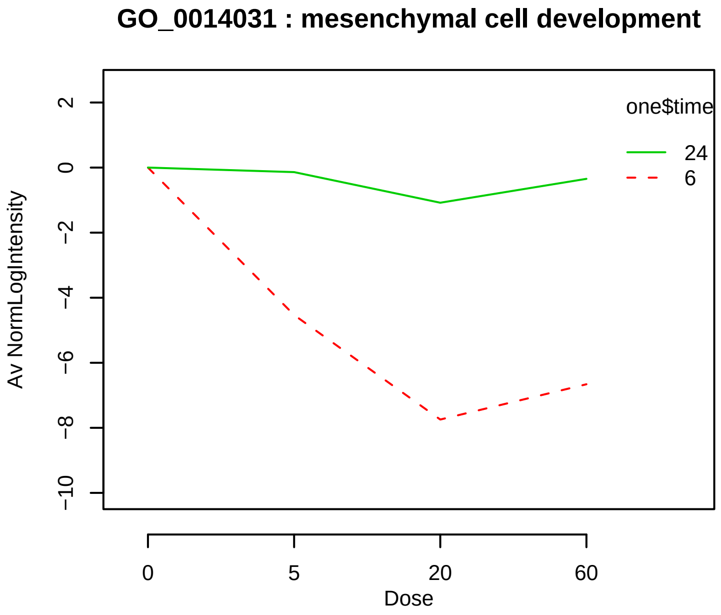
GO_0014031 : mesenchymal cell development
one$time
24
6
Av NormLogIntensity
Dose
0
5
20
60

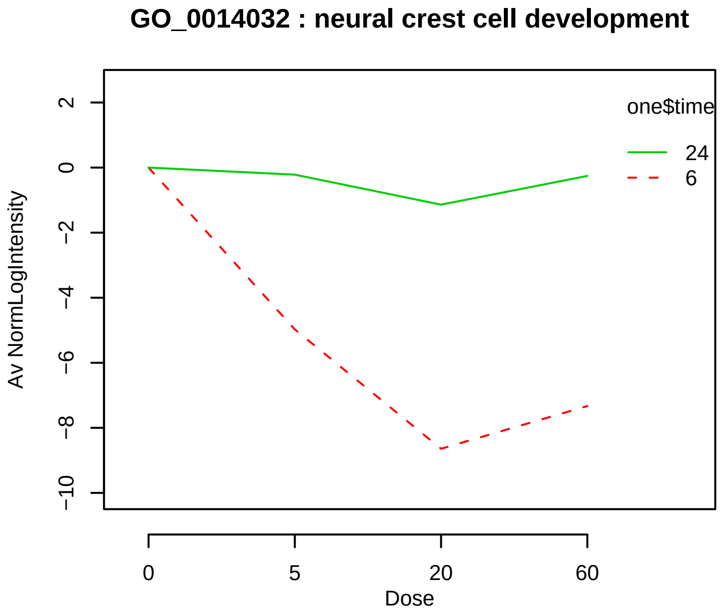
GO_0014032 : neural crest cell development
one$time
24
6
Av NormLogIntensity
Dose
0
5
20
60

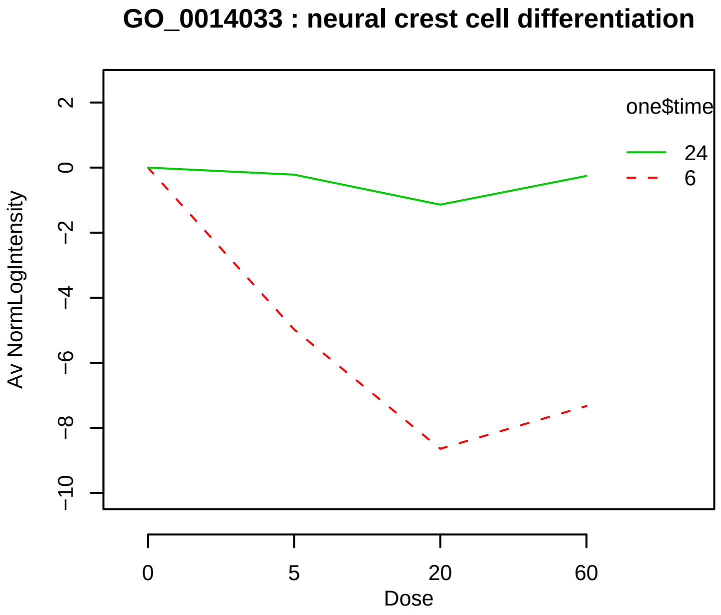
GO_0014033 : neural crest cell differentiation
one$time
24
6
Av NormLogIntensity
Dose
0
5
20
60

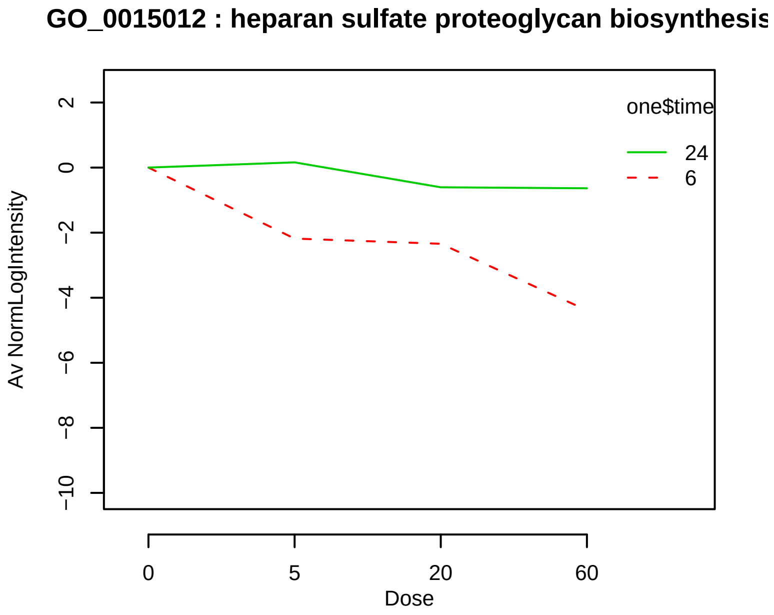
GO_0015012 : heparan sulfate proteoglycan biosynthesis
one$time
24
6
Av NormLogIntensity
Dose
0
5
20
60

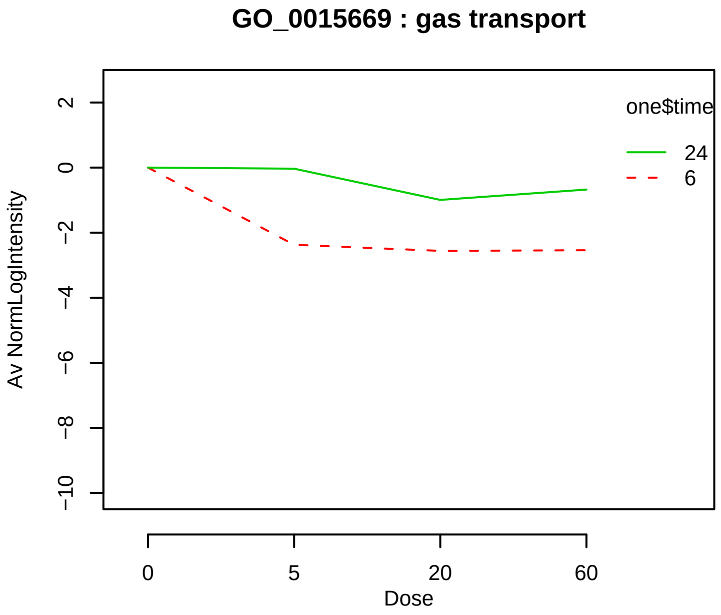
GO_0015669 : gas transport
one$time
24
6
Av NormLogIntensity
Dose
0
5
20
60

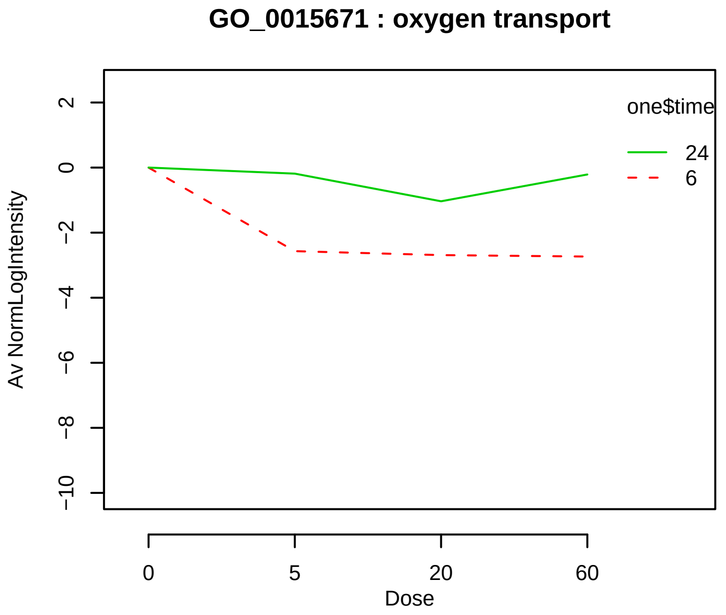
GO_0015671 : oxygen transport
Av NormLogIntensity
2
0
−2
−4
−6
−8
−10
one$time
24
6
0
5
20
60
Dose

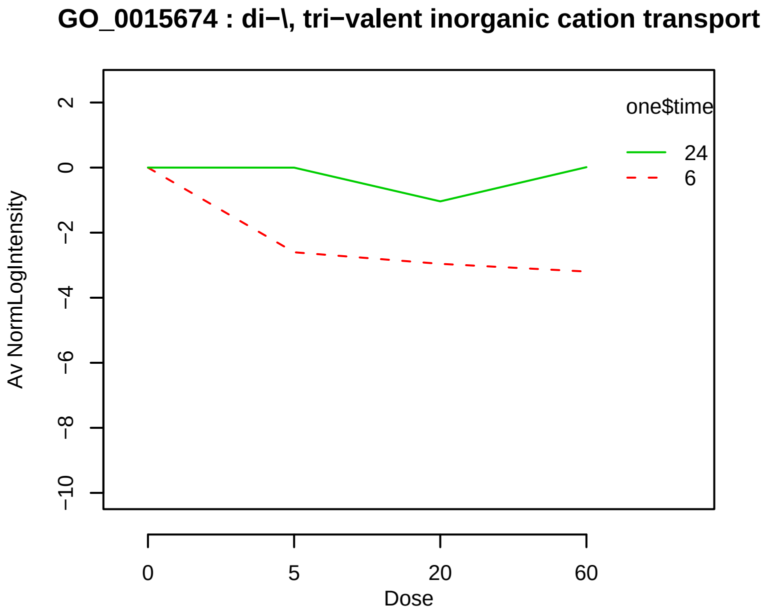
GO_0015674 : di−\, tri−valent inorganic cation transport
Av NormLogIntensity
2
0
−2
−4
−6
−8
−10
one$time
24
6
0
5
20
60
Dose

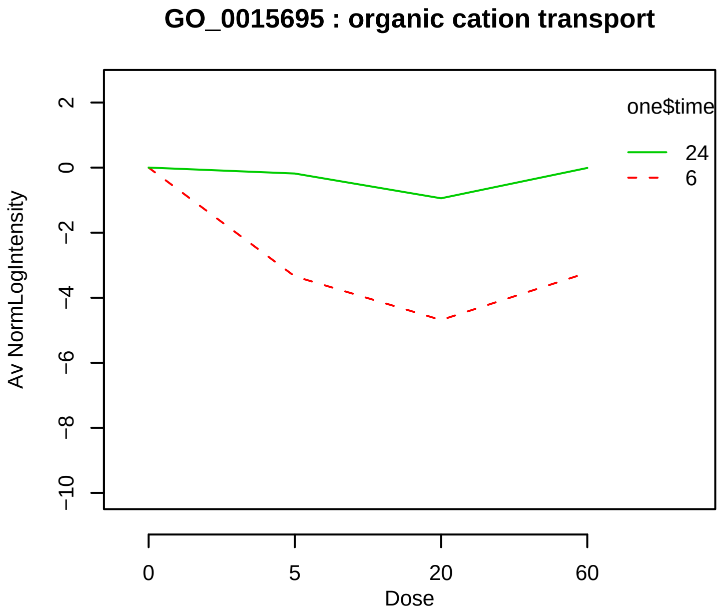
GO_0015695 : organic cation transport
Av NormLogIntensity
2
0
−2
−4
−6
−8
−10
one$time
24
6
0
5
20
60
Dose

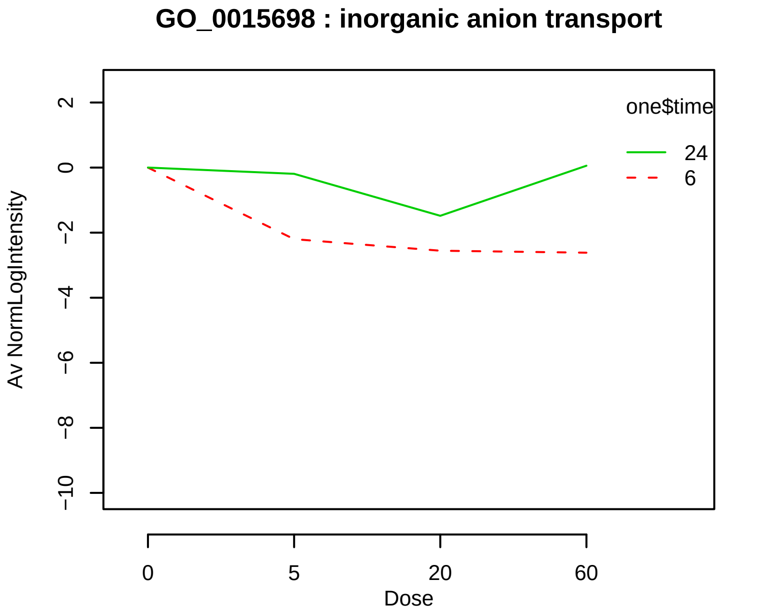
GO_0015698 : inorganic anion transport
Av NormLogIntensity
2
0
−2
−4
−6
−8
−10
one$time
24
6
0
5
20
60
Dose

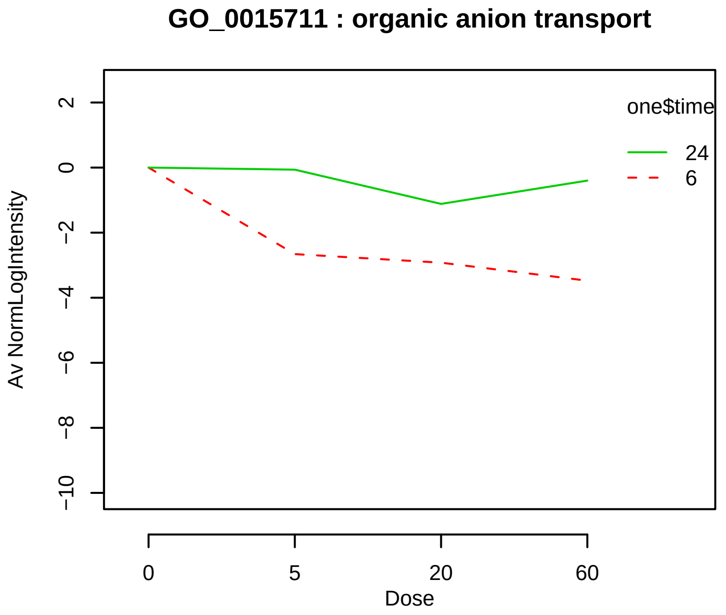
GO_0015711 : organic anion transport
Av NormLogIntensity
2
0
−2
−4
−6
−8
−10
one$time
24
6
0
5
20
60
Dose

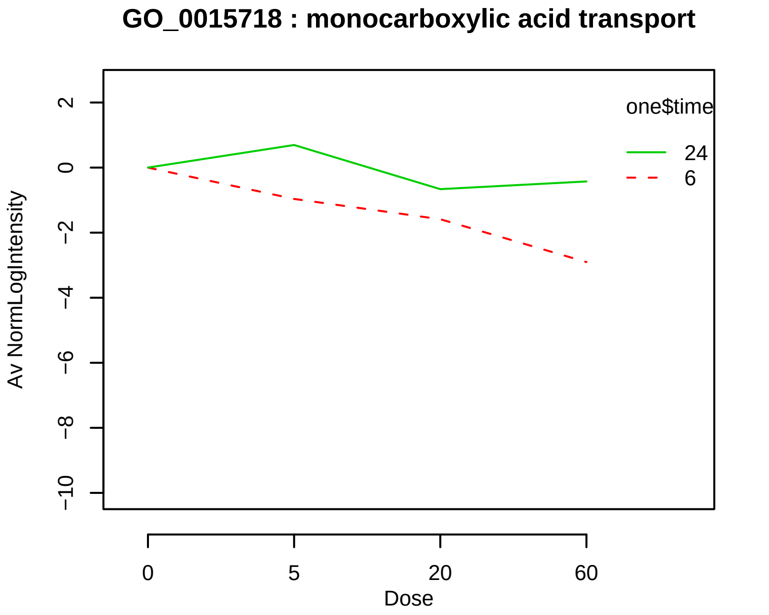
GO_0015718 : monocarboxylic acid transport
Av NormLogIntensity
2
0
−2
−4
−6
−8
−10
one$time
24
6
0
5
20
60
Dose

GO_0015749 : monosaccharide transport

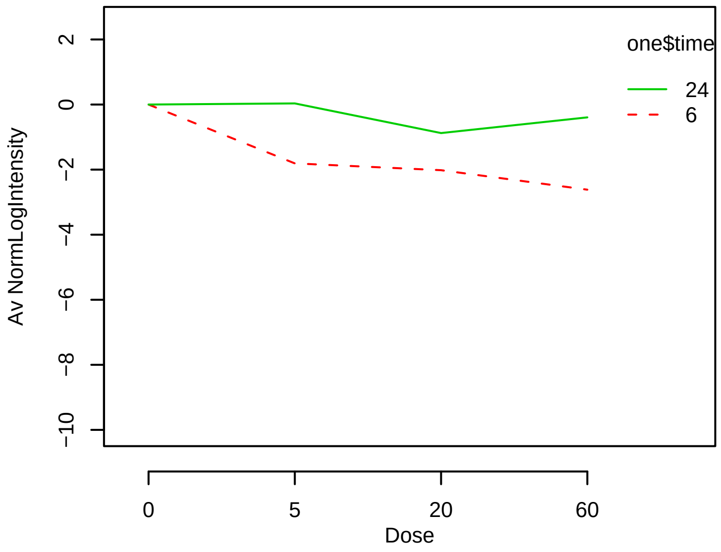

GO_0015758 : glucose transport

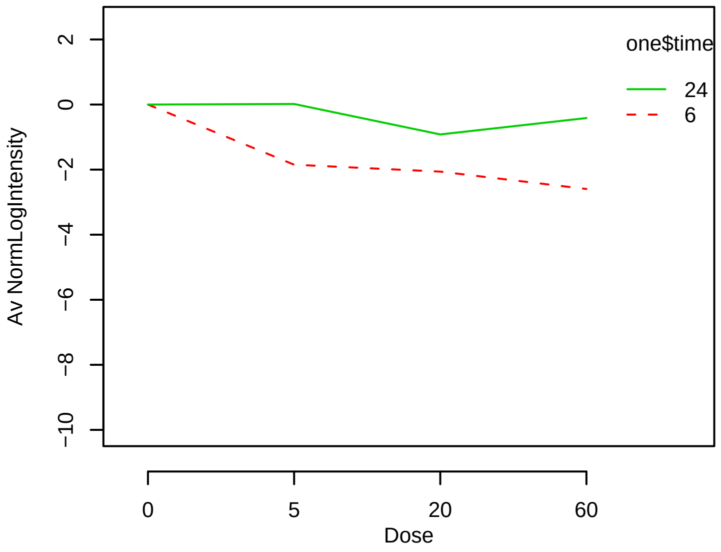

GO_0015780 : nucleotide−sugar transport

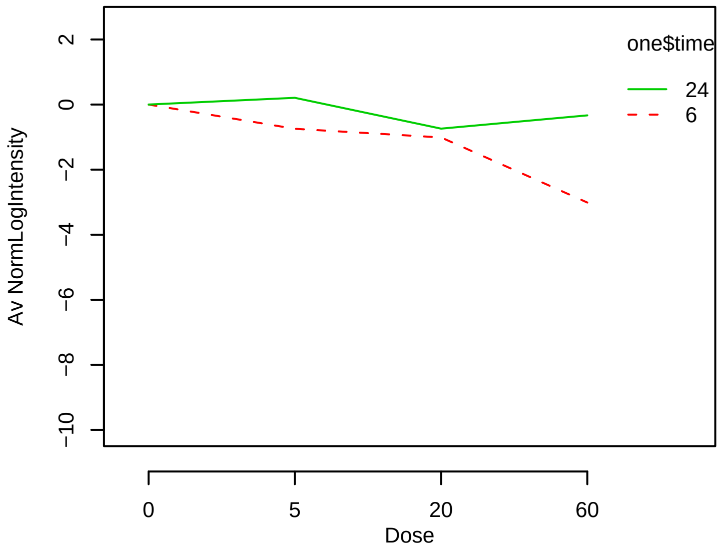

GO_0015781 : pyrimidine nucleotide−sugar transport

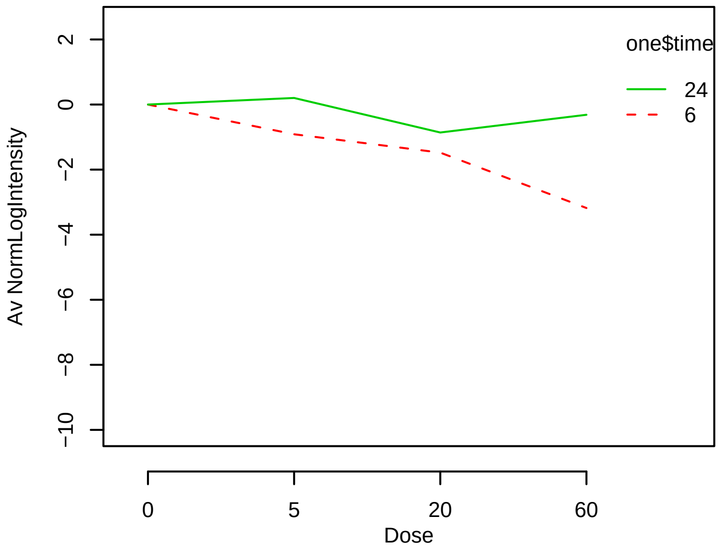

GO_0015800 : acidic amino acid transport

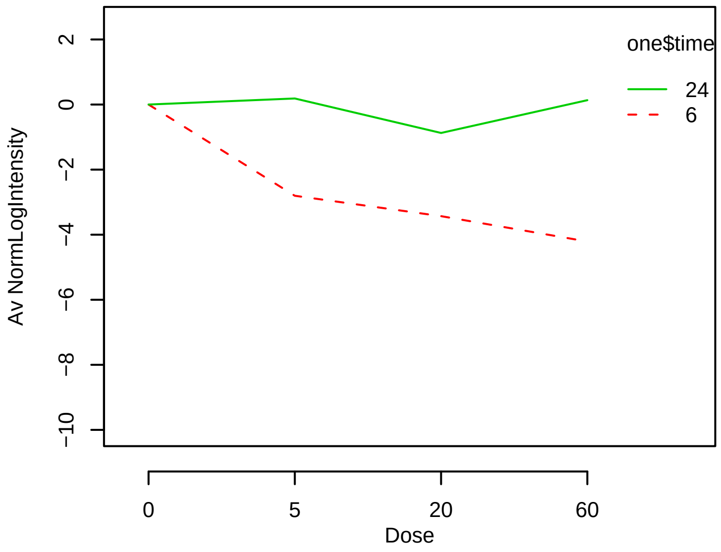

GO_0015802 : basic amino acid transport

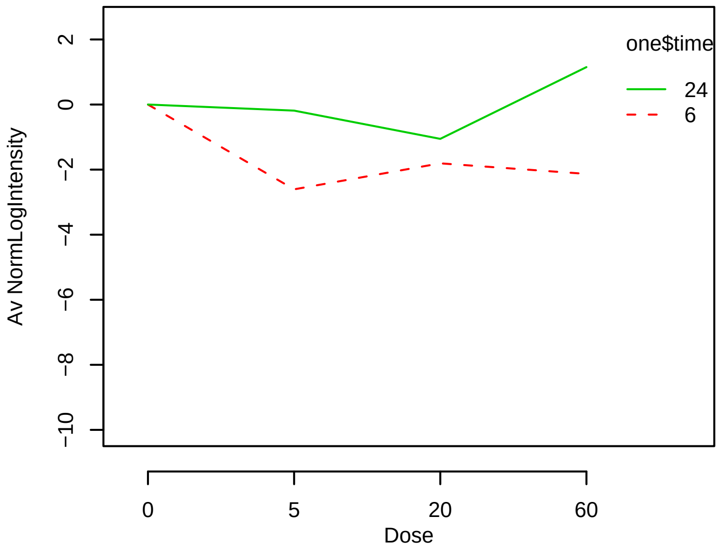

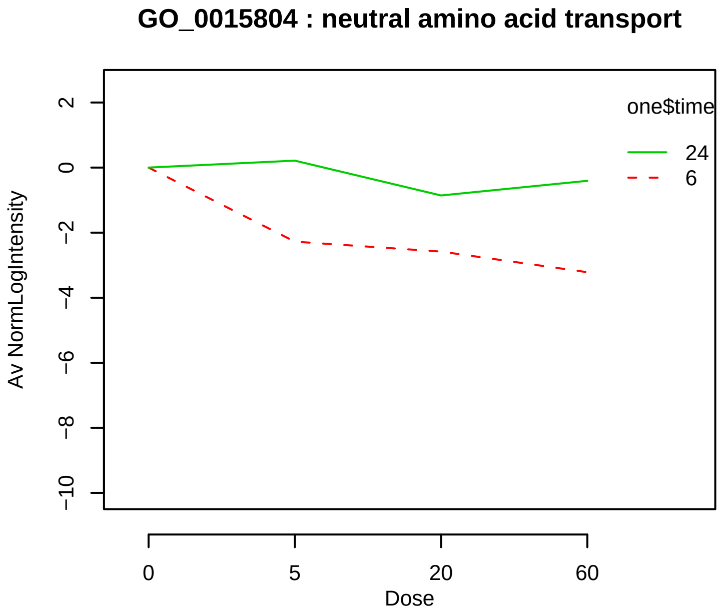
GO_0015804 : neutral amino acid transport
one$time
24
6
Av NormLogIntensity
Dose
0
5
20
60

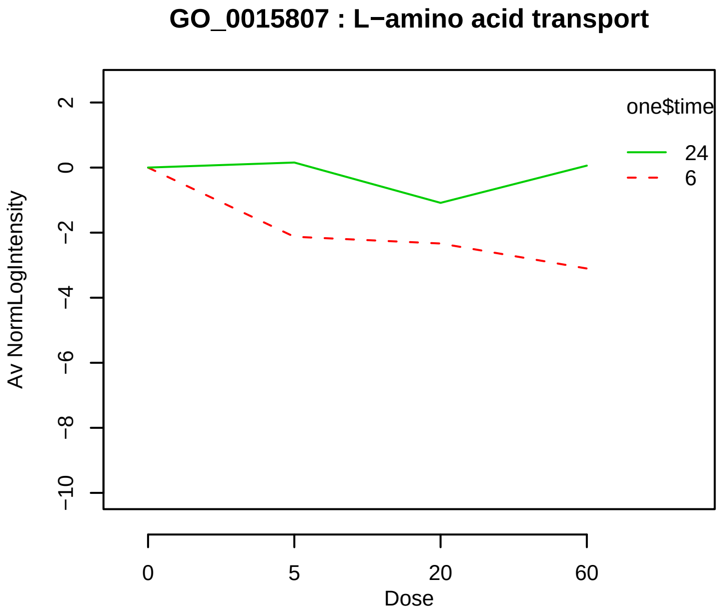
GO_0015807 : L−amino acid transport
one$time
24
6
Av NormLogIntensity
Dose
0
5
20
60

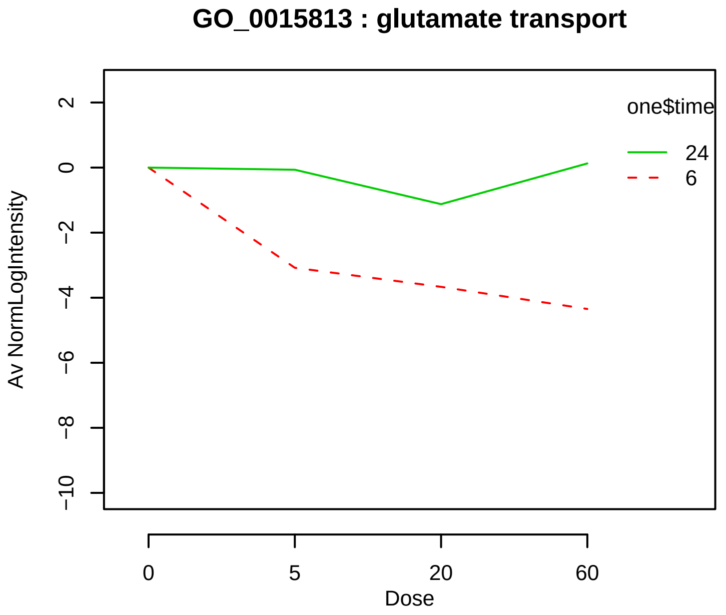
GO_0015813 : glutamate transport
one$time
24
6
Av NormLogIntensity
Dose
0
5
20
60

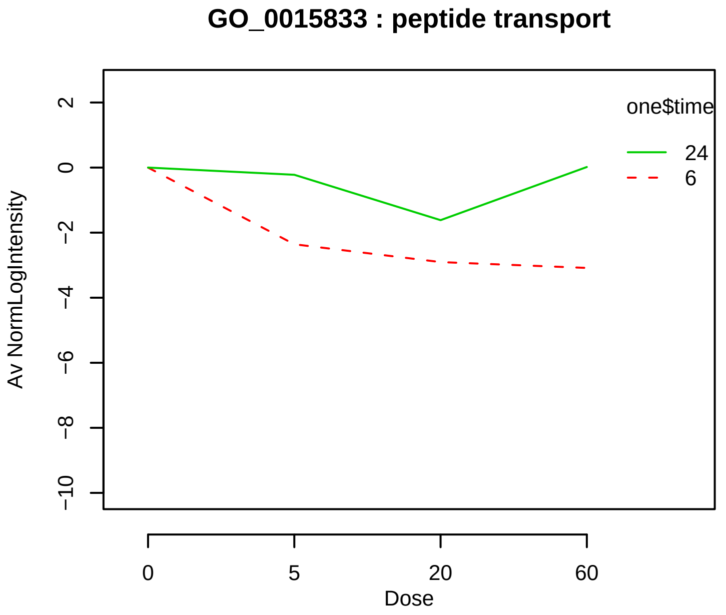
GO_0015833 : peptide transport
one$time
24
6
Av NormLogIntensity
Dose
0
5
20
60

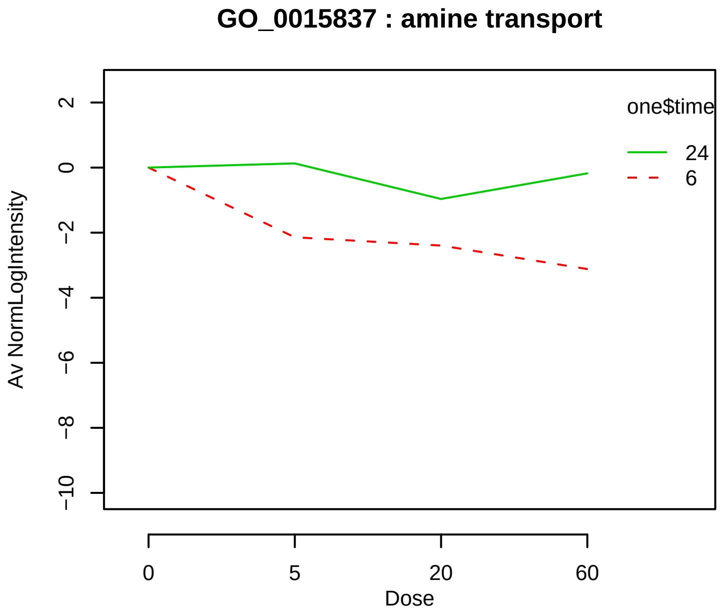
GO_0015837 : amine transport
one$time
24
6
Av NormLogIntensity
Dose
0
5
20
60

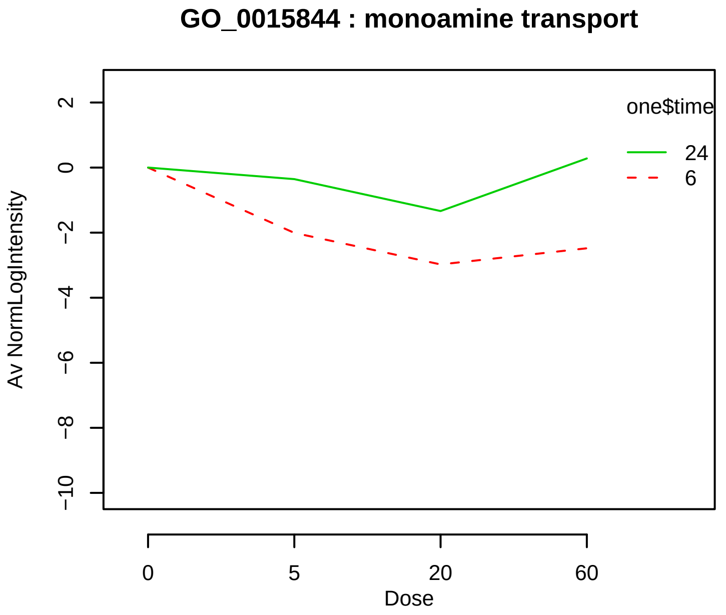
GO_0015844 : monoamine transport
one$time
24
6
Av NormLogIntensity
Dose
0
5
20
60

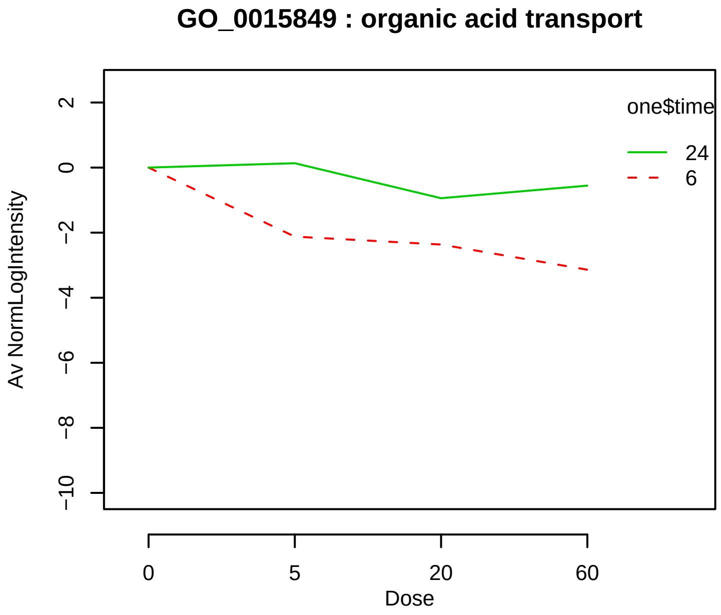

GO_0015849 : organic acid transport
one$time
24
6
Av NormLogIntensity
Dose
0
5
20
60

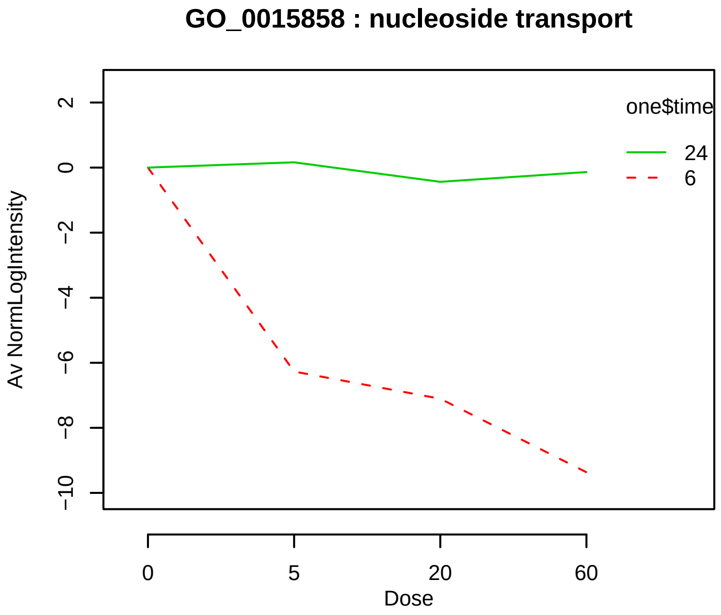

GO_0015858 : nucleoside transport
one$time
24
6
Av NormLogIntensity
Dose
0
5
20
60

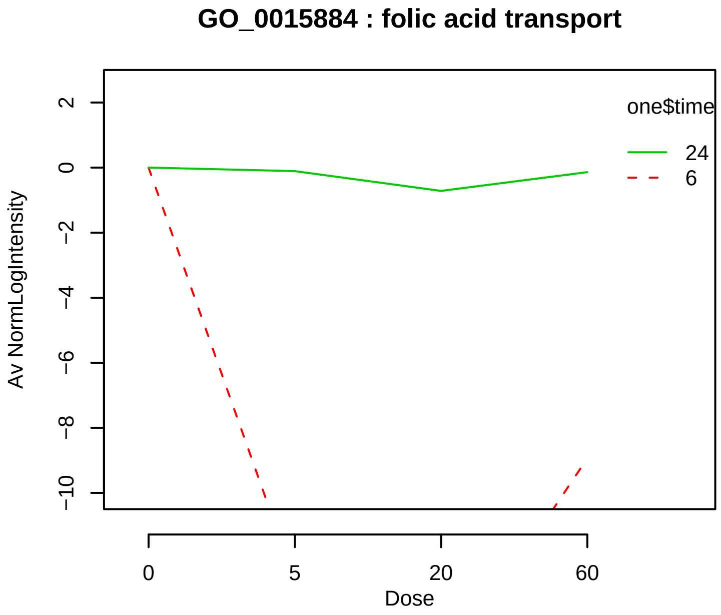

GO_0015884 : folic acid transport
one$time
24
6
Av NormLogIntensity
Dose
0
5
20
60

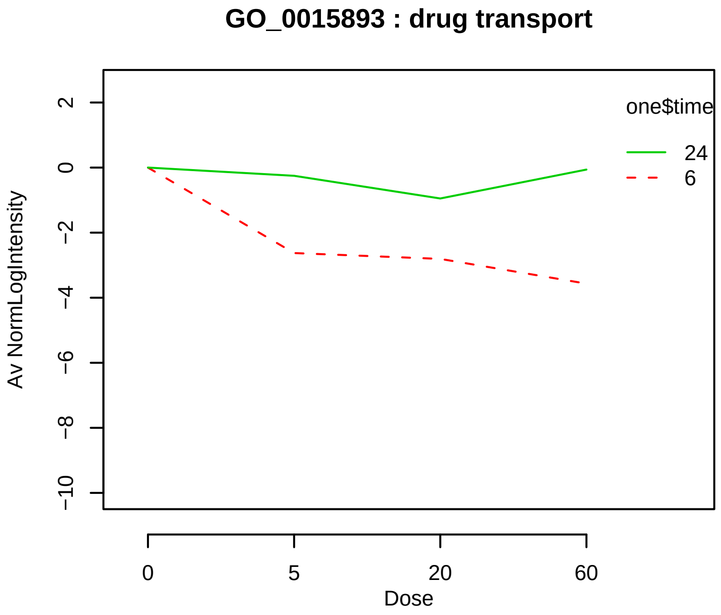

GO_0015893 : drug transport
one$time
24
6
Av NormLogIntensity
Dose
0
5
20
60

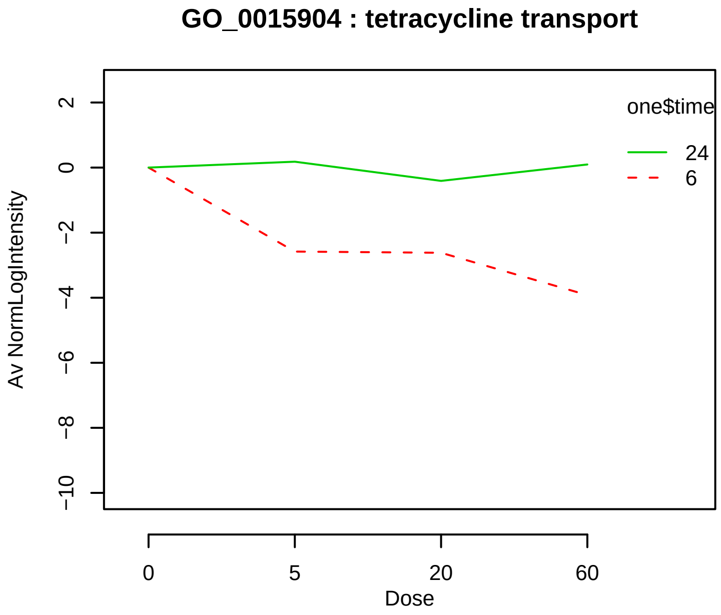

GO_0015904 : tetracycline transport
one$time
24
6
Av NormLogIntensity
Dose
0
5
20
60

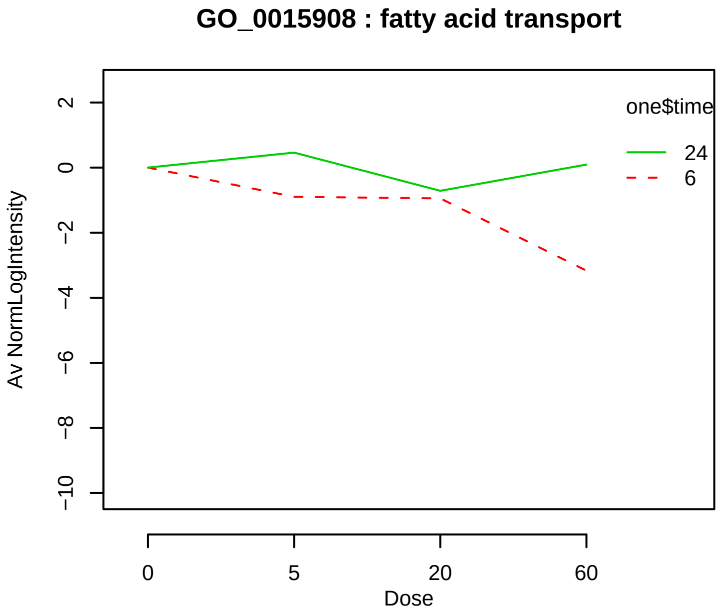

GO_0015908 : fatty acid transport
one$time
24
6
Av NormLogIntensity
Dose
0
5
20
60

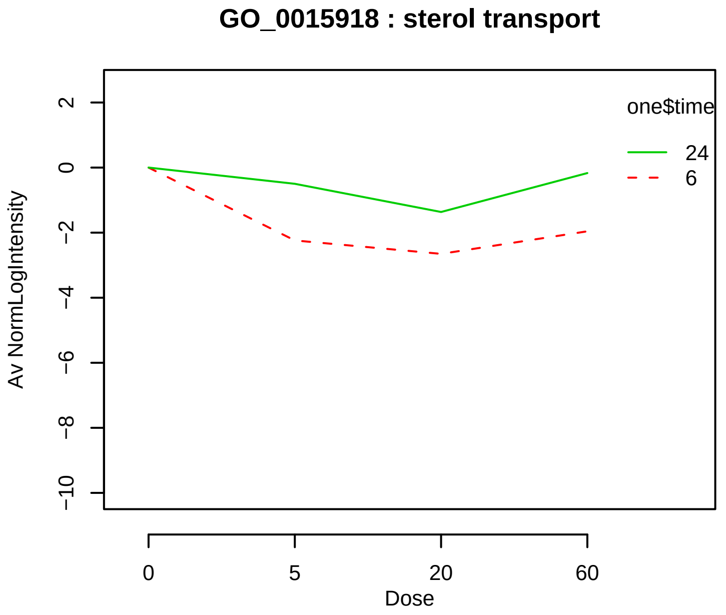
GO_0015918 : sterol transport
one$time
24
6
Av NormLogIntensity
2
0
−2
−4
−6
−8
−10
0
5
20
60
Dose

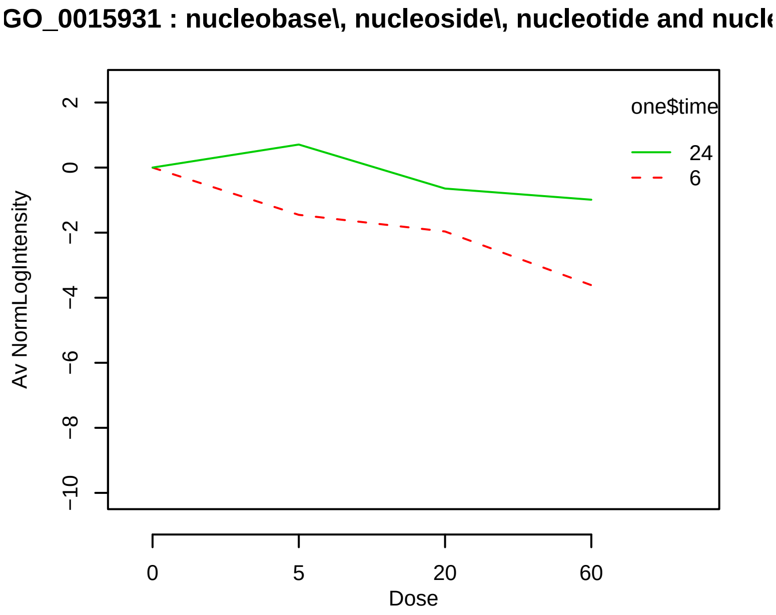
GO_0015931 : nucleobase\, nucleoside\, nucleotide and nucle
one$time
24
6
Av NormLogIntensity
2
0
−2
−4
−6
−8
−10
0
5
20
60
Dose

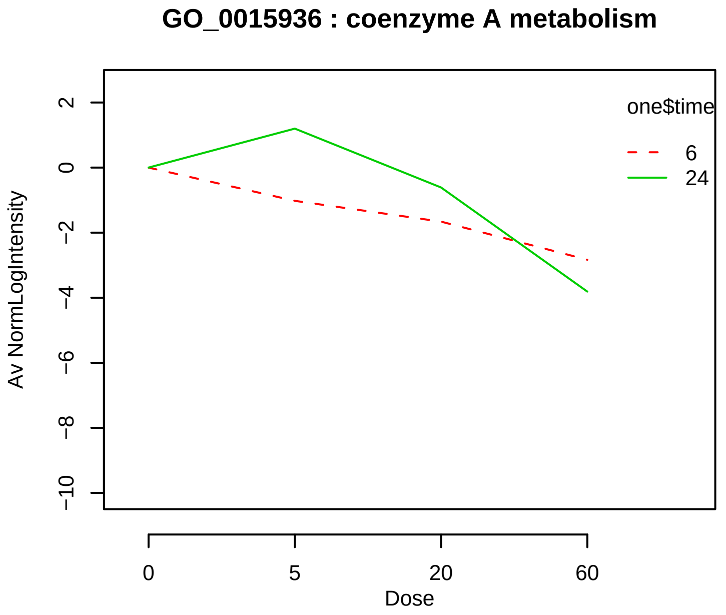
GO_0015936 : coenzyme A metabolism
one$time
6
24
Av NormLogIntensity
2
0
−2
−4
−6
−8
−10
0
5
20
60
Dose

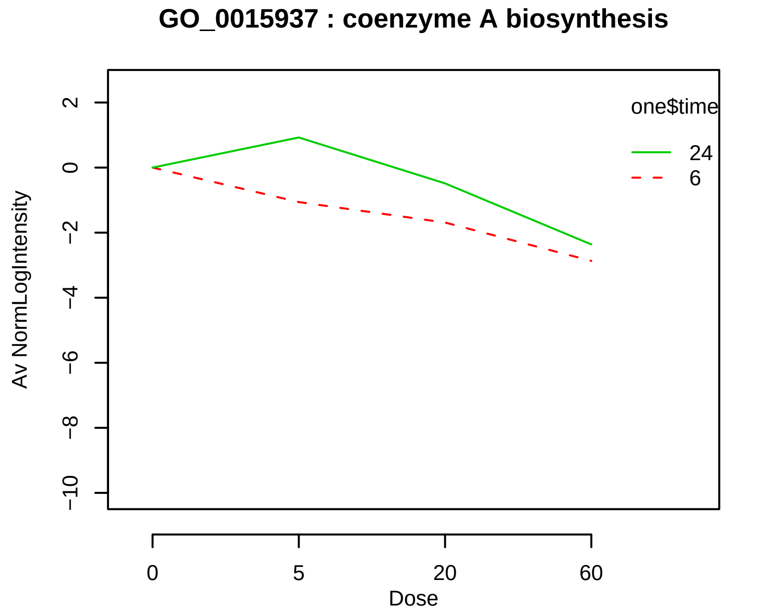
GO_0015937 : coenzyme A biosynthesis
one$time
24
6
Av NormLogIntensity
2
0
−2
−4
−6
−8
−10
0
5
20
60
Dose

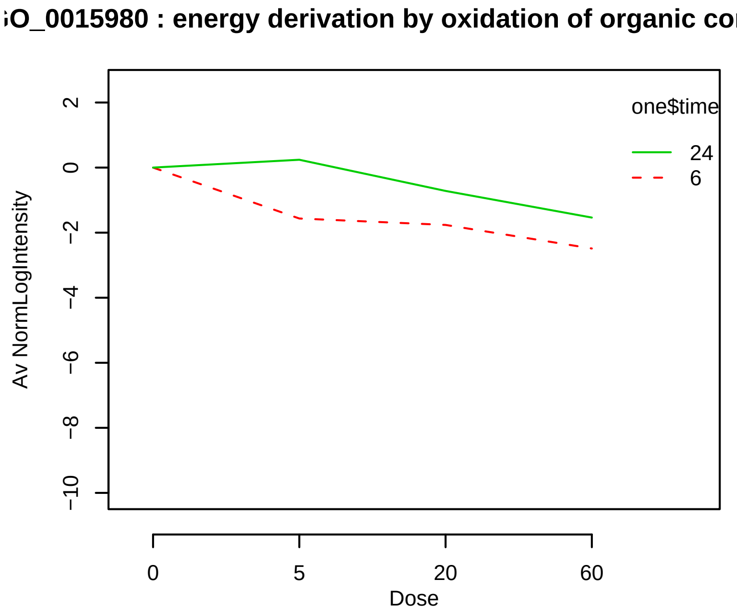
O_0015980 : energy derivation by oxidation of organic comp
one$time
24
6
Av NormLogIntensity
2
0
−2
−4
−6
−8
−10
0
5
20
60
Dose

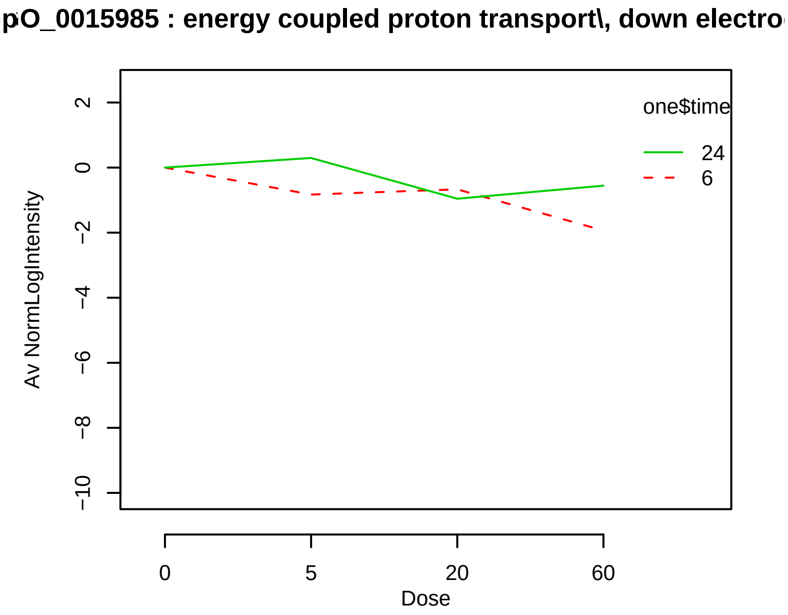
O_0015985 : energy coupled proton transport\, down electro
one$time
24
6
Av NormLogIntensity
2
0
−2
−4
−6
−8
−10
0
5
20
60
Dose

GO_0015986 : ATP synthesis coupled proton transport

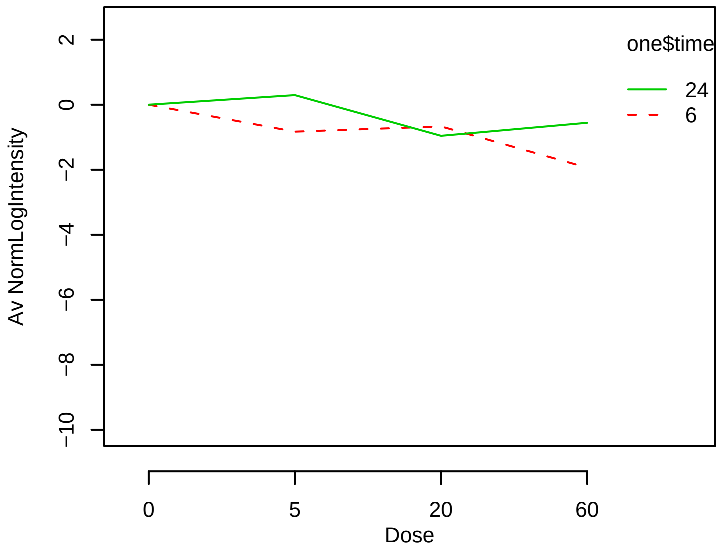

GO_0015988 : energy coupled proton transport\, against elec

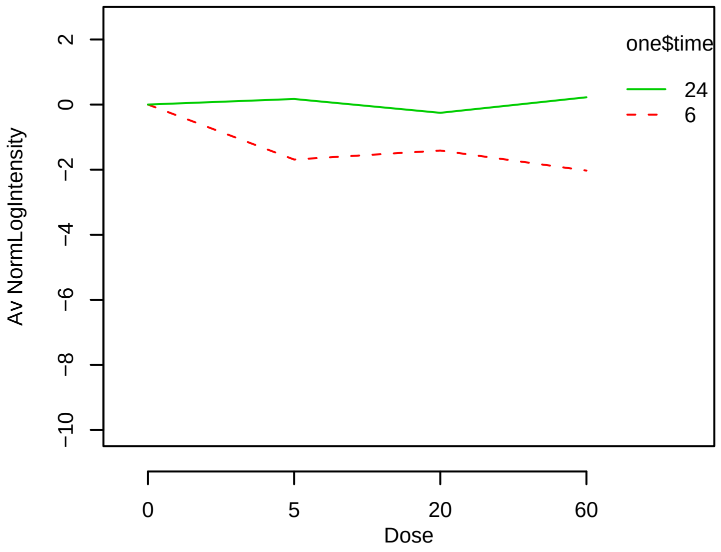

GO_0015991 : ATP hydrolysis coupled proton transport

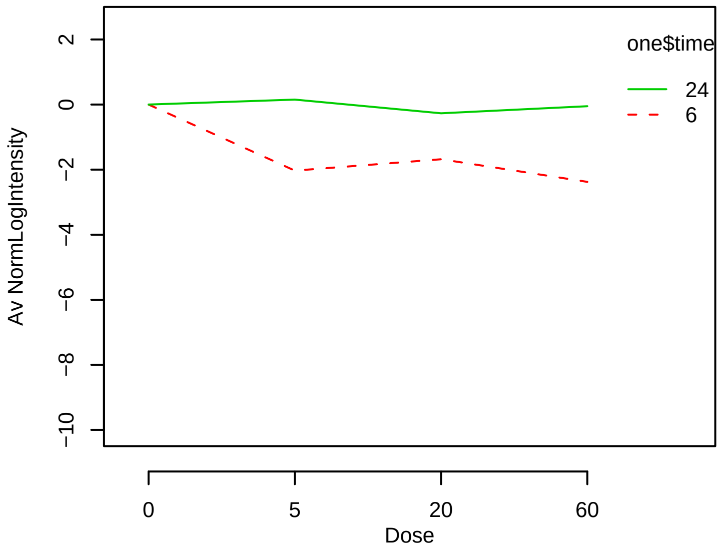

GO_0015992 : proton transport

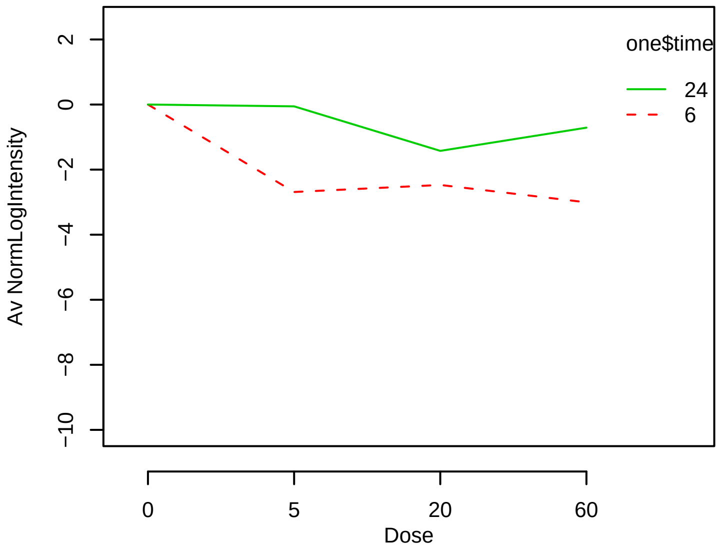

GO_0016032 : viral life cycle

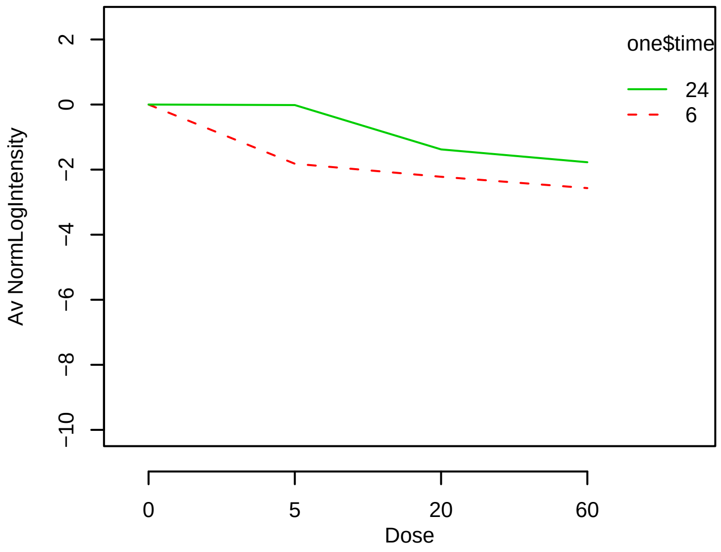

GO_0016042 : lipid catabolism

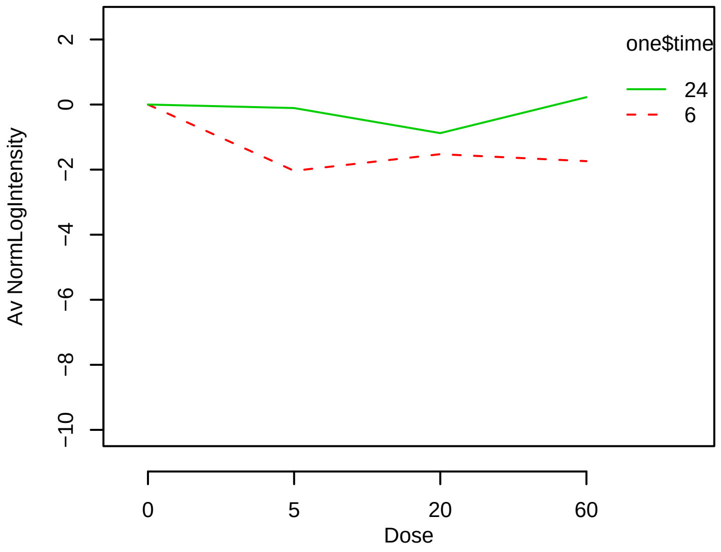

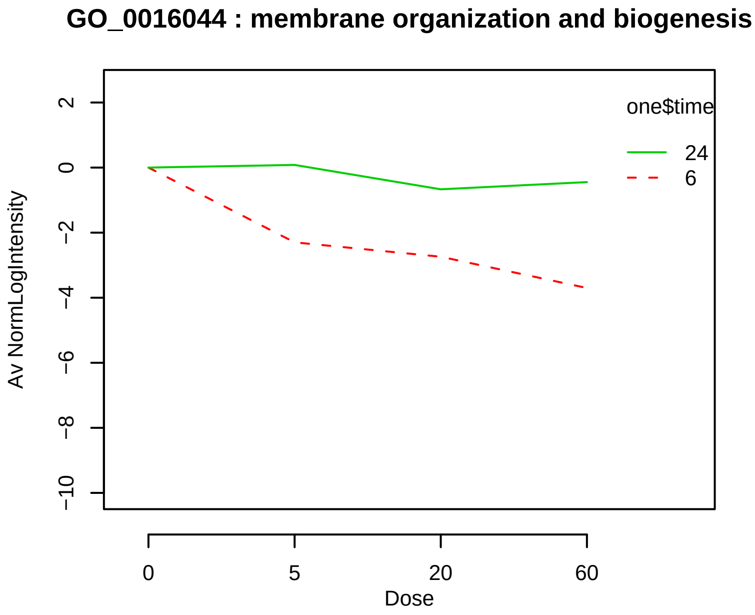
GO_0016044 : membrane organization and biogenesis
Av NormLogIntensity
2
0
−2
−4
−6
−8
−10
one$time
24
6
0
5
20
60
Dose

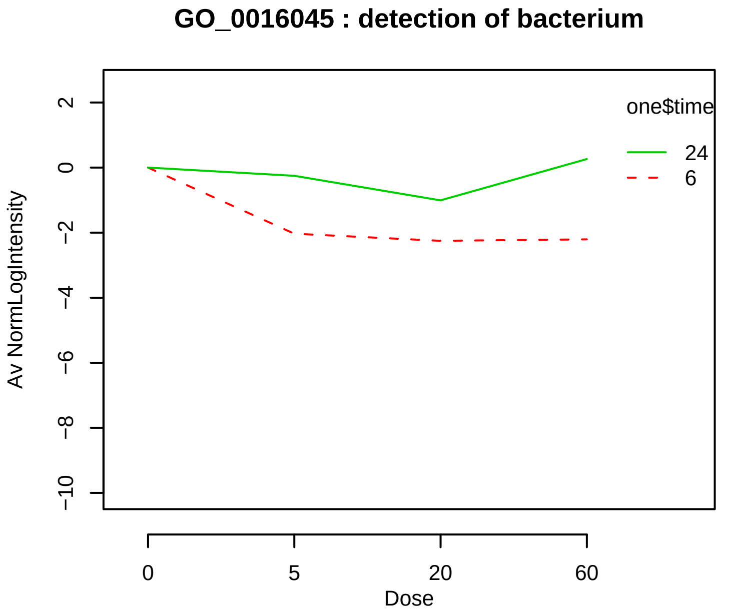
GO_0016045 : detection of bacterium
Av NormLogIntensity
2
0
−2
−4
−6
−8
−10
one$time
24
6
0
5
20
60
Dose

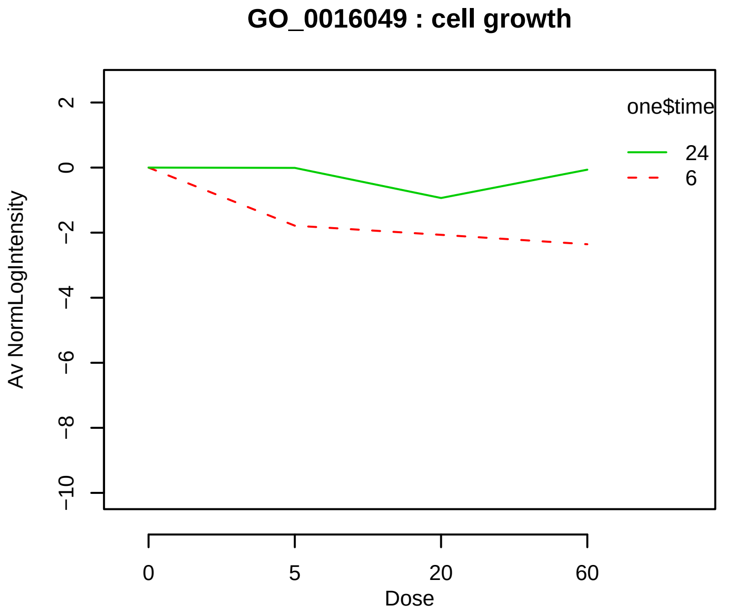
GO_0016049 : cell growth
Av NormLogIntensity
2
0
−2
−4
−6
−8
−10
one$time
24
6
0
5
20
60
Dose

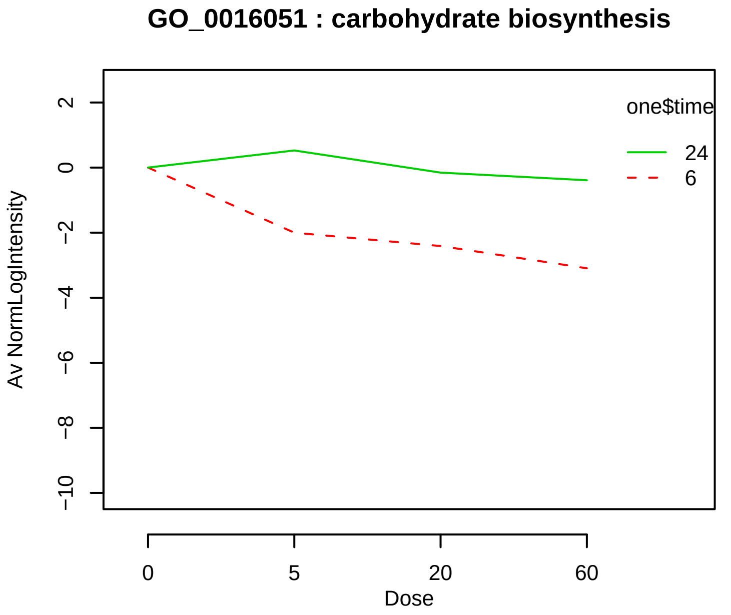
GO_0016051 : carbohydrate biosynthesis
Av NormLogIntensity
2
0
−2
−4
−6
−8
−10
one$time
24
6
0
5
20
60
Dose

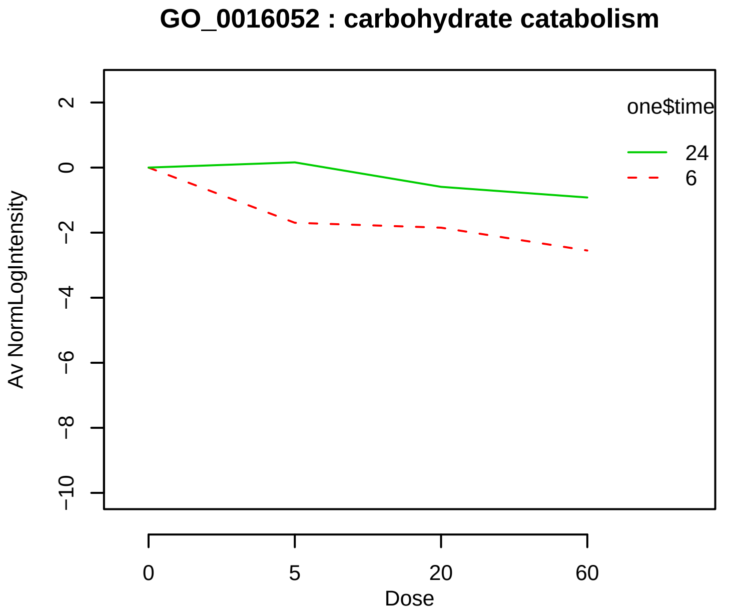
GO_0016052 : carbohydrate catabolism
Av NormLogIntensity
2
0
−2
−4
−6
−8
−10
one$time
24
6
0
5
20
60
Dose

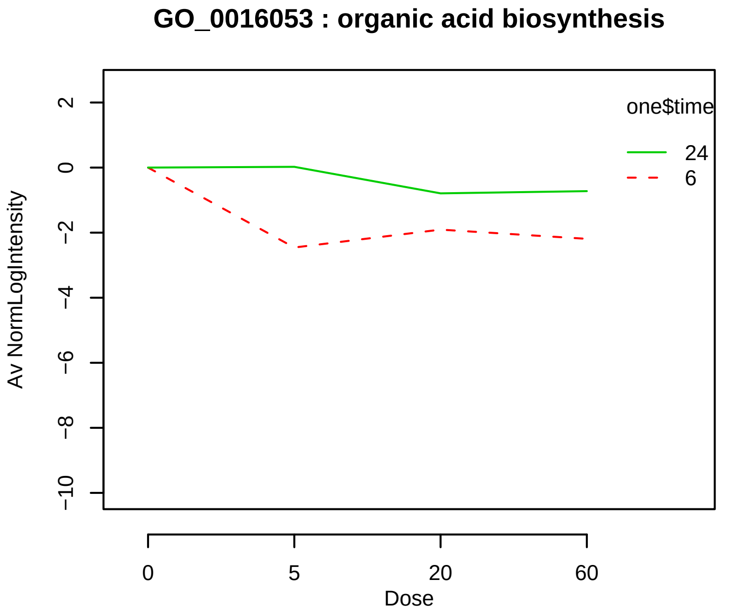
GO_0016053 : organic acid biosynthesis
Av NormLogIntensity
2
0
−2
−4
−6
−8
−10
one$time
24
6
0
5
20
60
Dose

GO_0016054 : organic acid catabolism

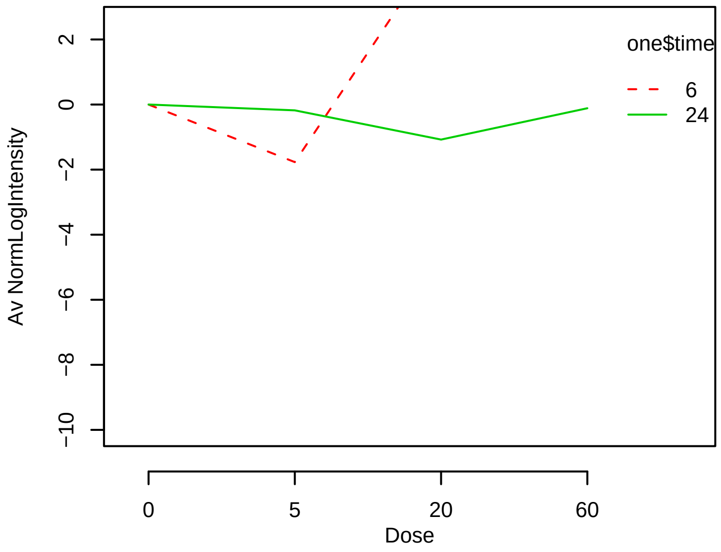

GO_0016055 : Wnt receptor signaling pathway

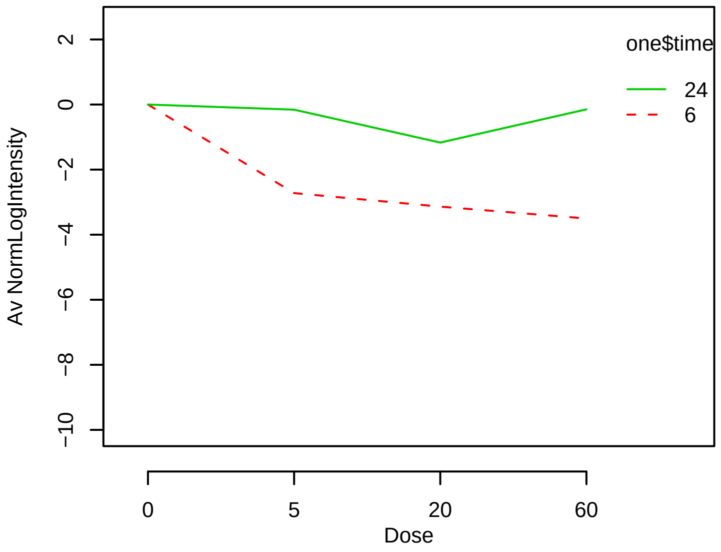

GO_0016064 : immunoglobulin mediated immune respons

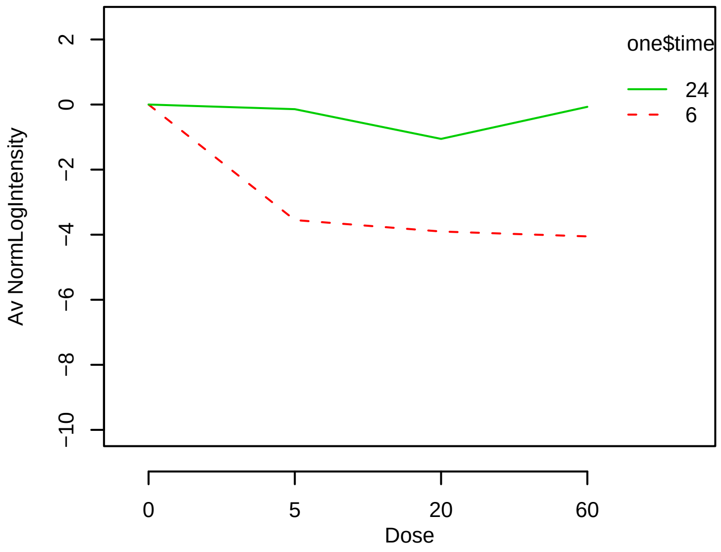

GO_0016071 : mRNA metabolism

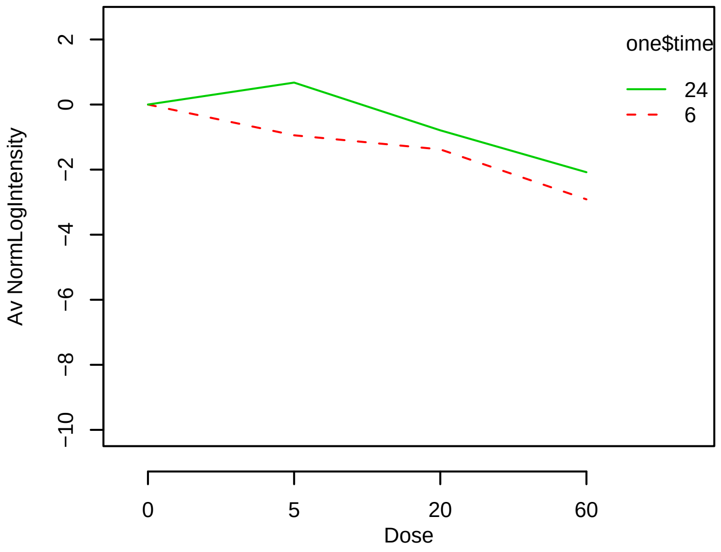

GO_0016072 : rRNA metabolism

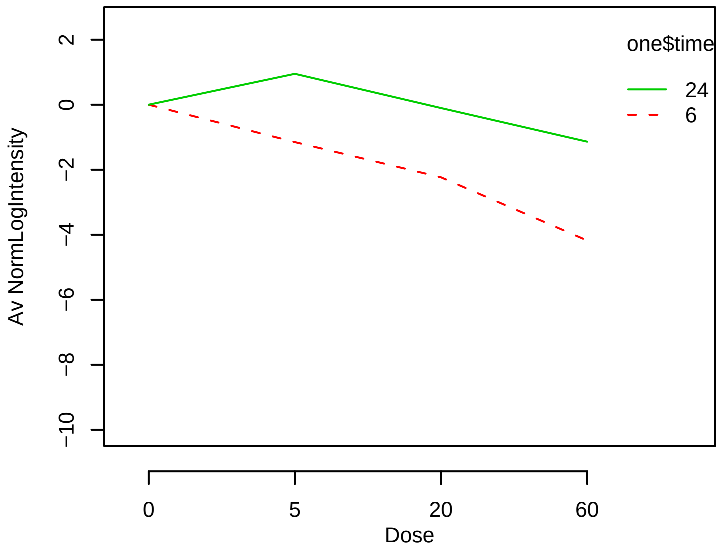

GO_0016073 : snRNA metabolism

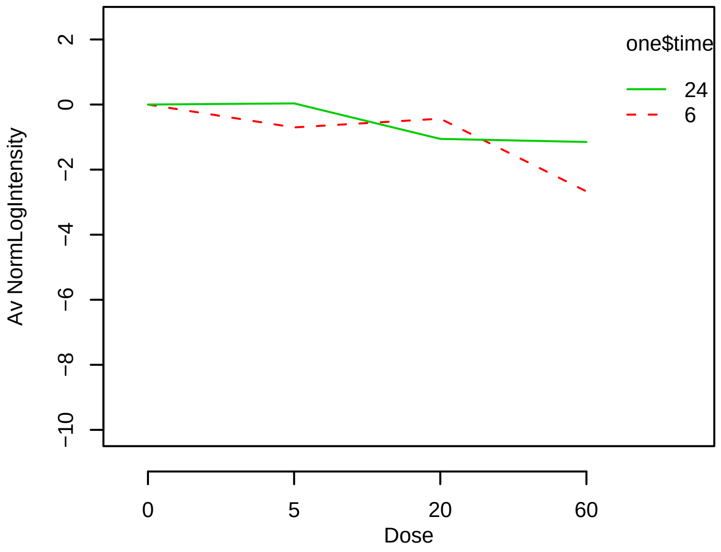

GO_0016079 : synaptic vesicle exocytosis

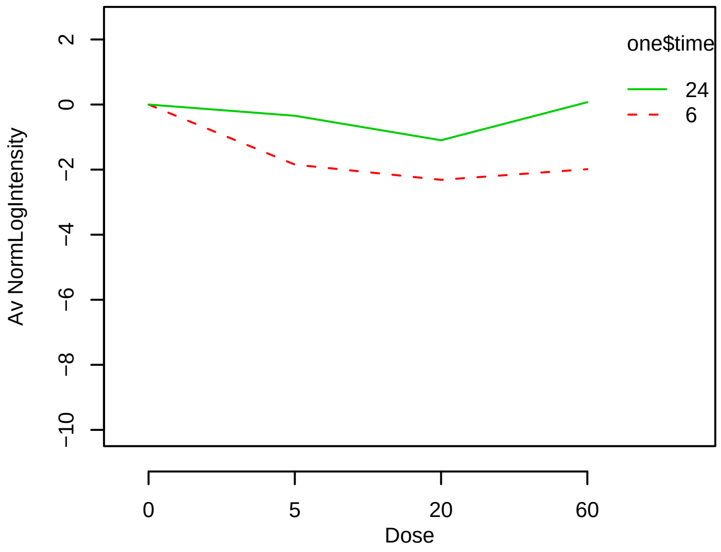

GO_0016096 : polyisoprenoid metabolism

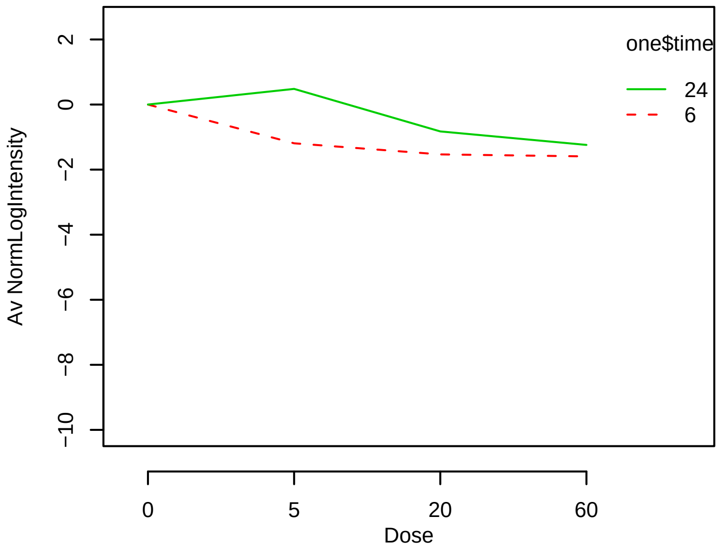

GO_0016108 : tetraterpenoid metabolism

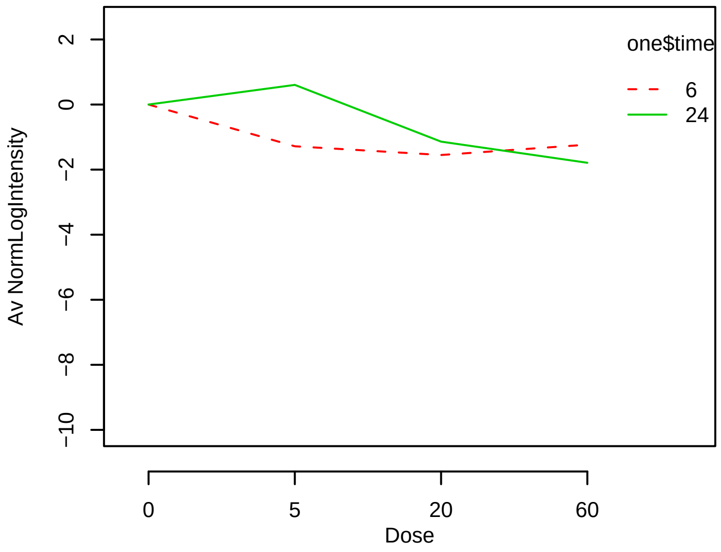

GO_0016116 : carotenoid metabolism

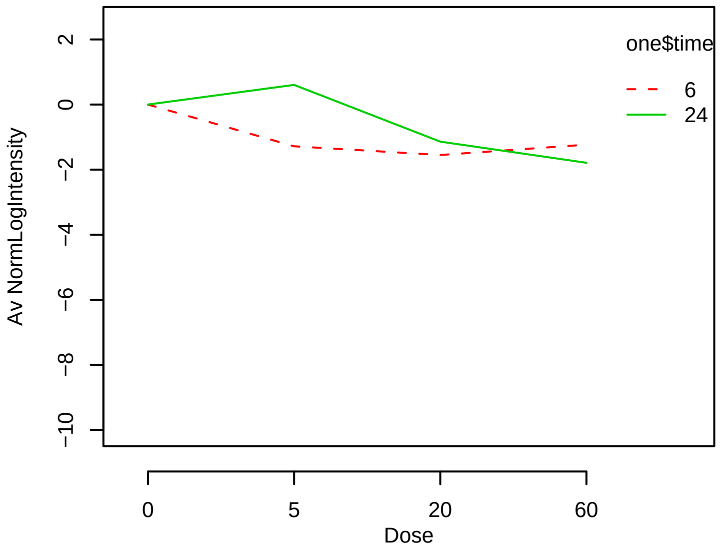

GO_0016125 : sterol metabolism

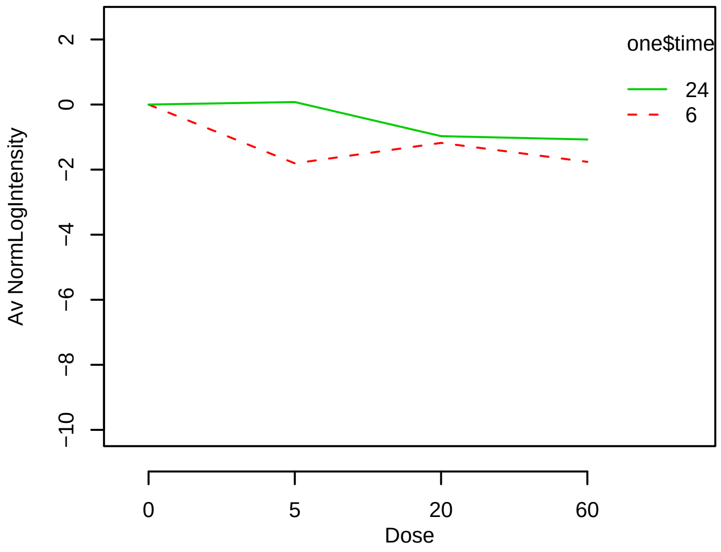

GO_0016126 : sterol biosynthesis

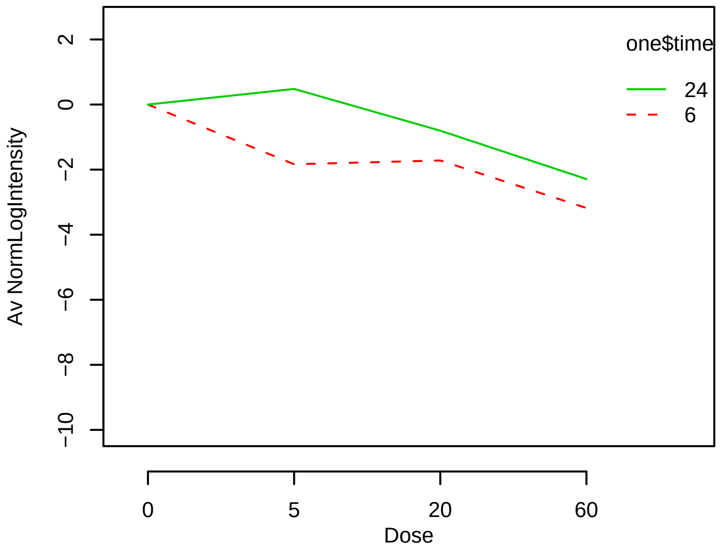

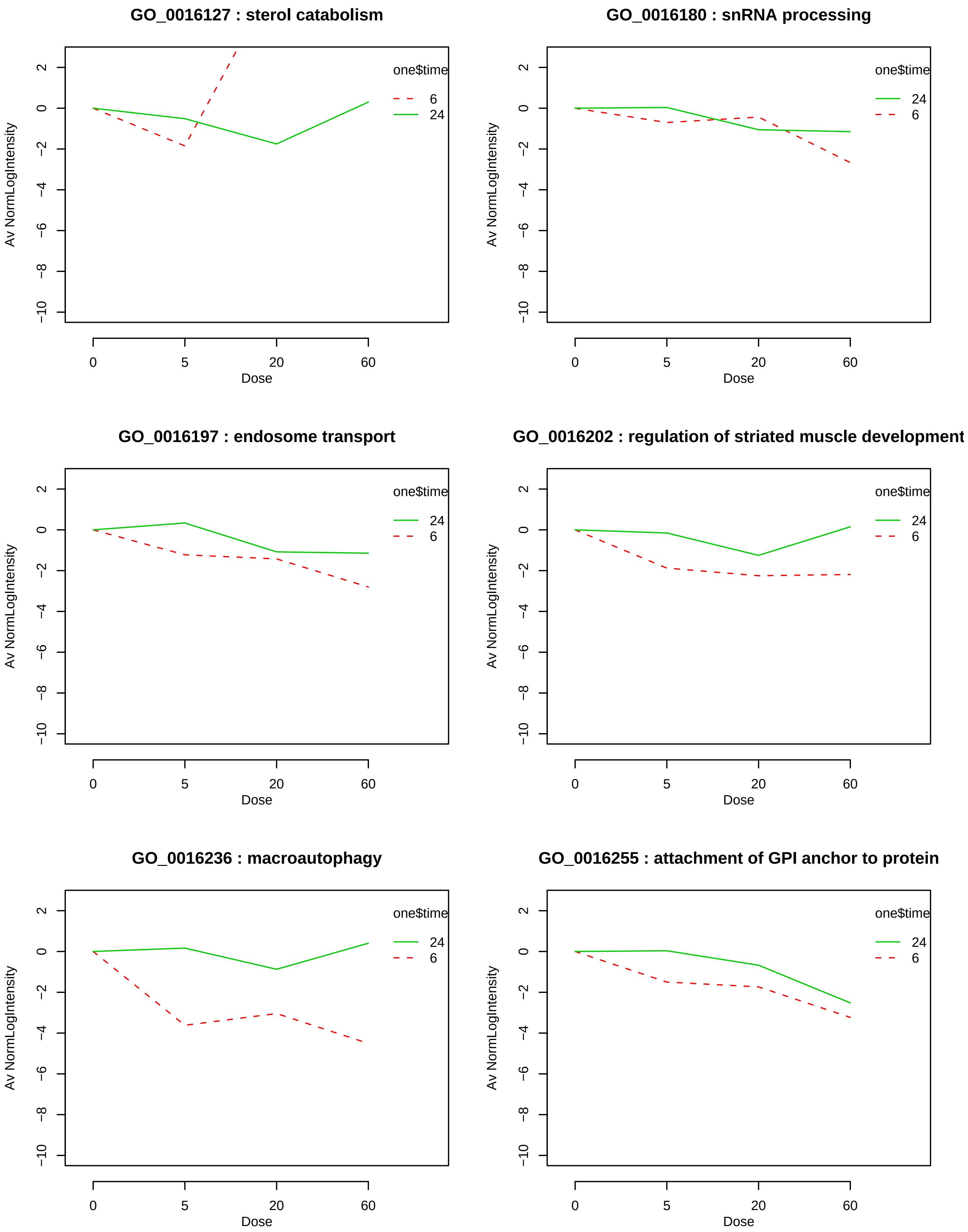

GO_0016127 : sterol catabolism
GO_0016180 : snRNA processing
GO_0016197 : endosome transport
GO_0016202 : regulation of striated muscle development
GO_0016236 : macroautophagy
GO_0016255 : attachment of GPI anchor to protein
one$time
6
24
Av NormLogIntensity
Dose
0
5
20
60
2
0
−2
−4
−6
−8
−10

GO_0016311 : dephosphorylation

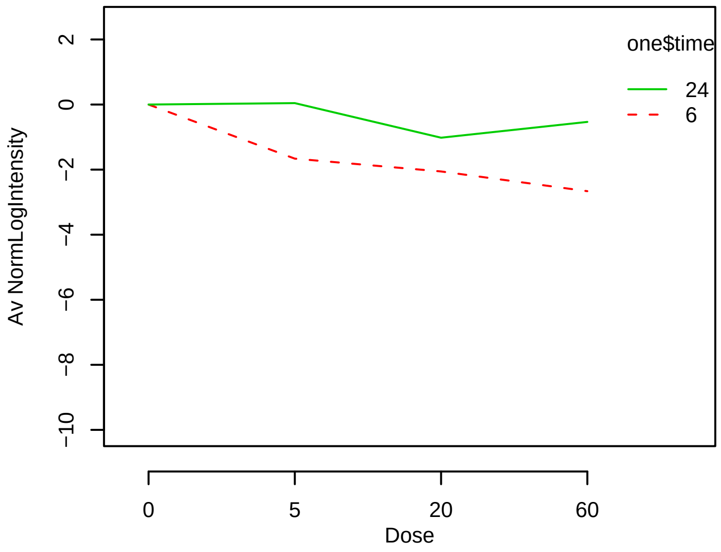

GO_0016322 : neuron remodeling

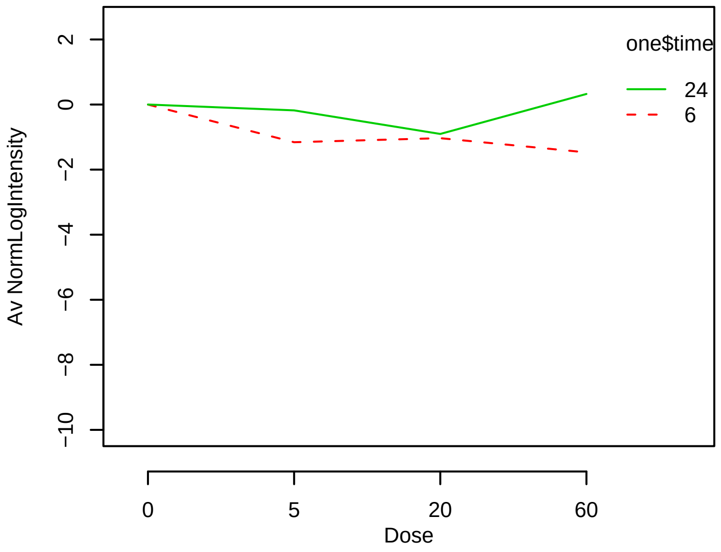

GO_0016331 : morphogenesis of embryonic epithelium

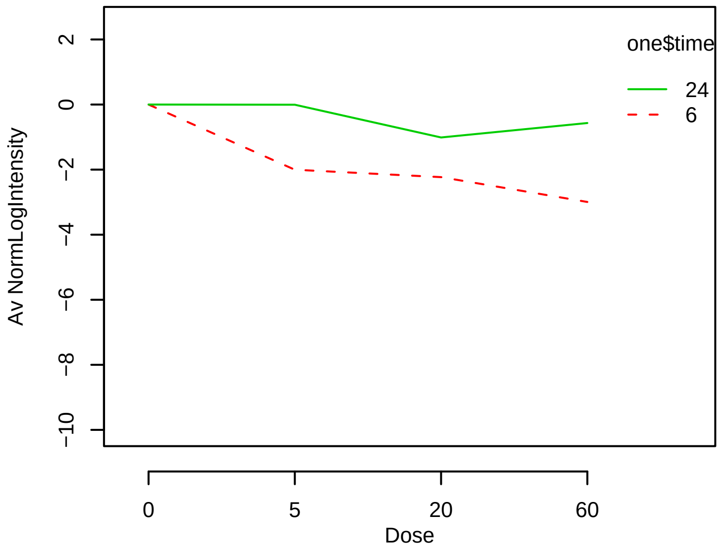

GO_0016337 : cell−cell adhesion

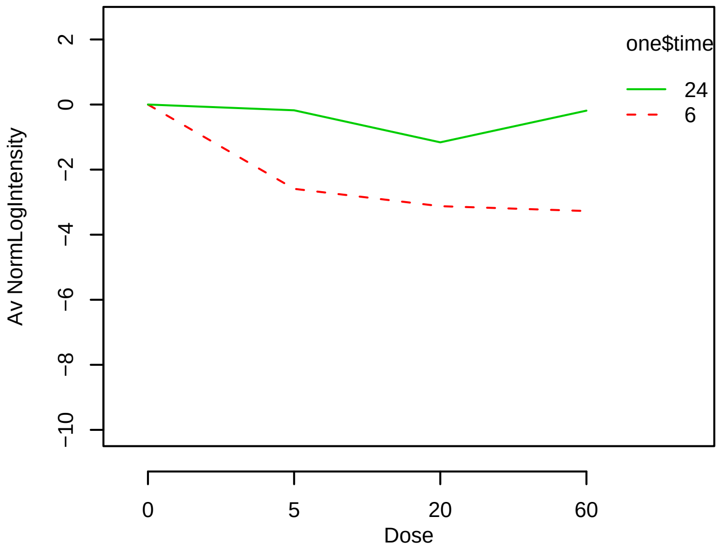

GO_0016338 : calcium−independent cell−cell adhesion

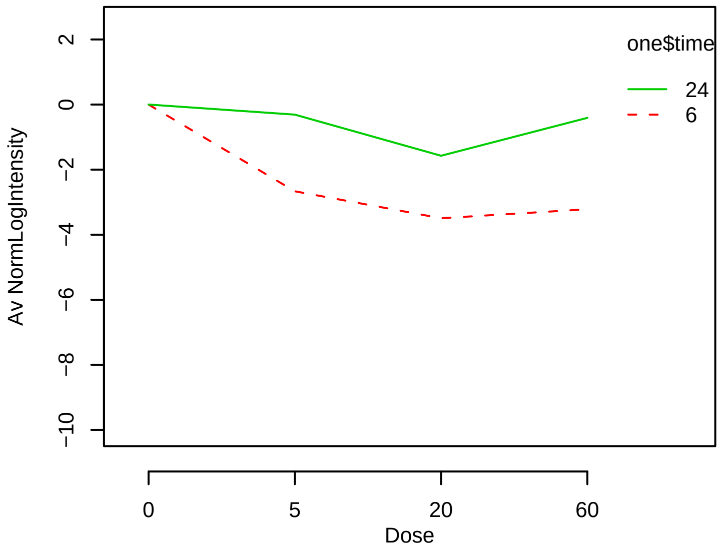

GO_0016339 : calcium−dependent cell−cell adhesion

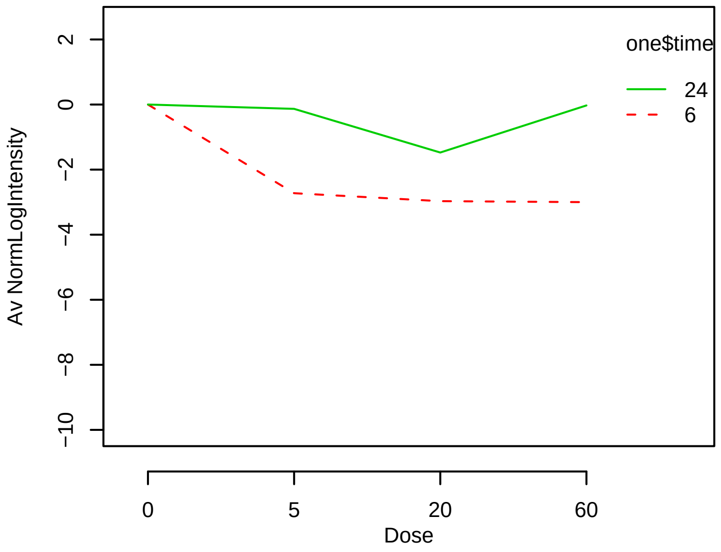

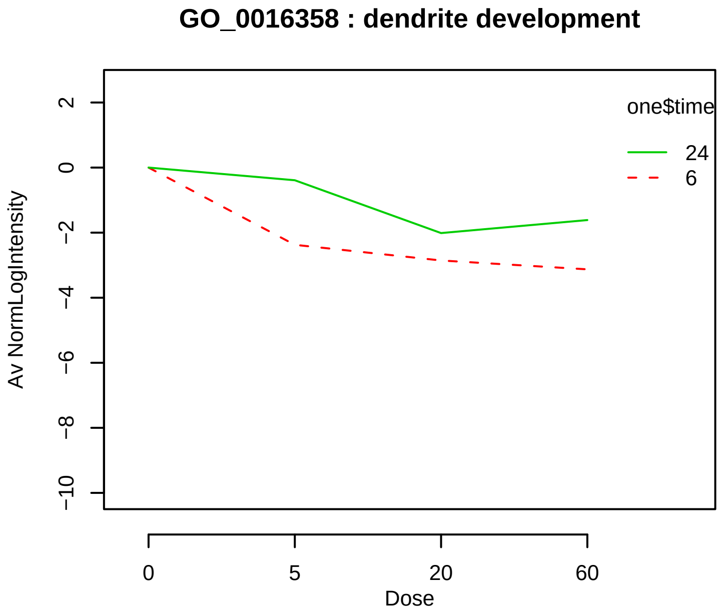
GO_0016358 : dendrite development
one$time
24
6
Av NormLogIntensity
2
0
−2
−4
−6
−8
−10
0
5
20
60
Dose

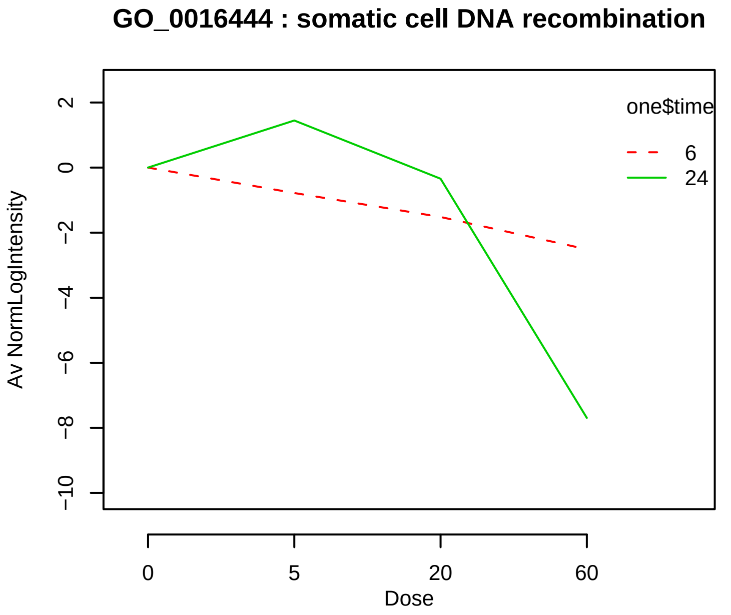
GO_0016444 : somatic cell DNA recombination
one$time
6
24
Av NormLogIntensity
2
0
−2
−4
−6
−8
−10
0
5
20
60
Dose

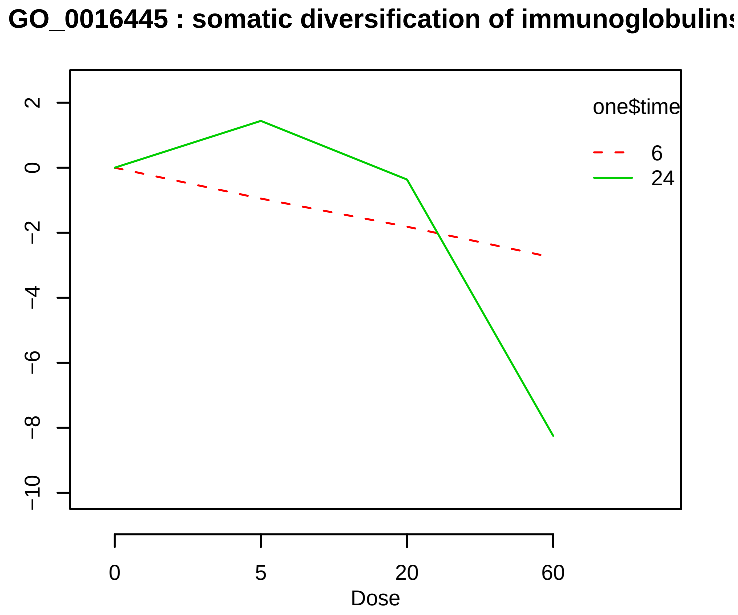
GO_0016445 : somatic diversification of immunoglobulins
one$time
6
24
Av NormLogIntensity
2
0
−2
−4
−6
−8
−10
0
5
20
60
Dose

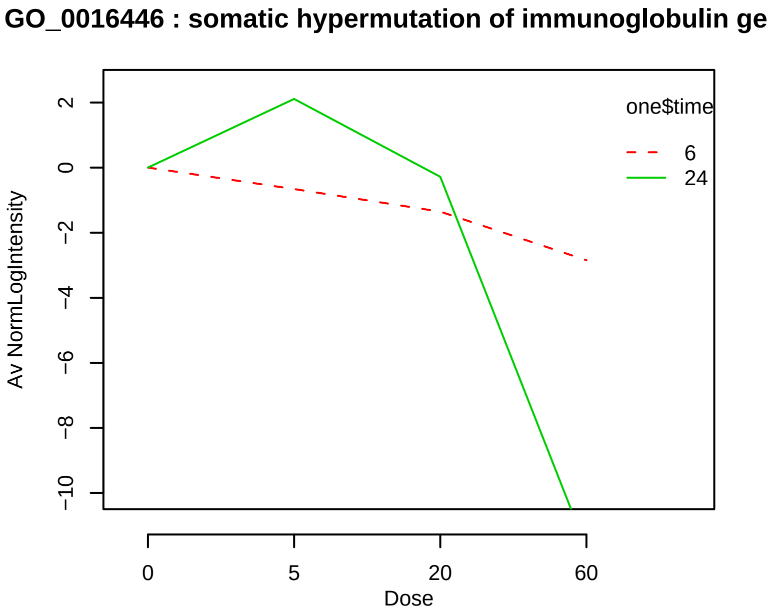
GO_0016446 : somatic hypermutation of immunoglobulin ge
one$time
6
24
Av NormLogIntensity
2
0
−2
−4
−6
−8
−10
0
5
20
60
Dose

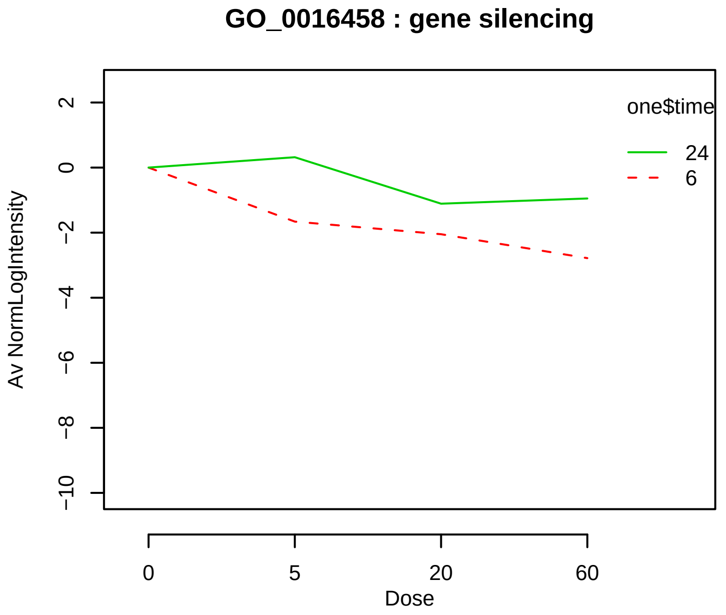
GO_0016458 : gene silencing
one$time
24
6
Av NormLogIntensity
2
0
−2
−4
−6
−8
−10
0
5
20
60
Dose

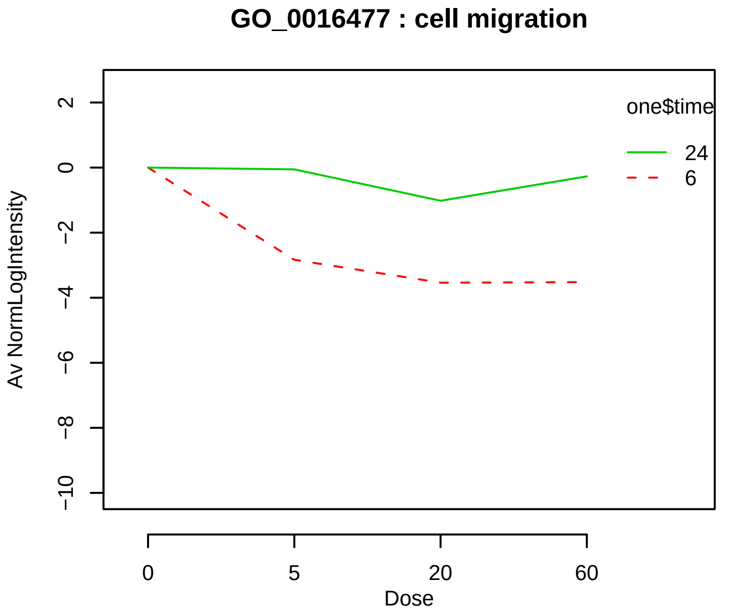
GO_0016477 : cell migration
one$time
24
6
Av NormLogIntensity
2
0
−2
−4
−6
−8
−10
0
5
20
60
Dose

GO_0016478 : negative regulation of translation

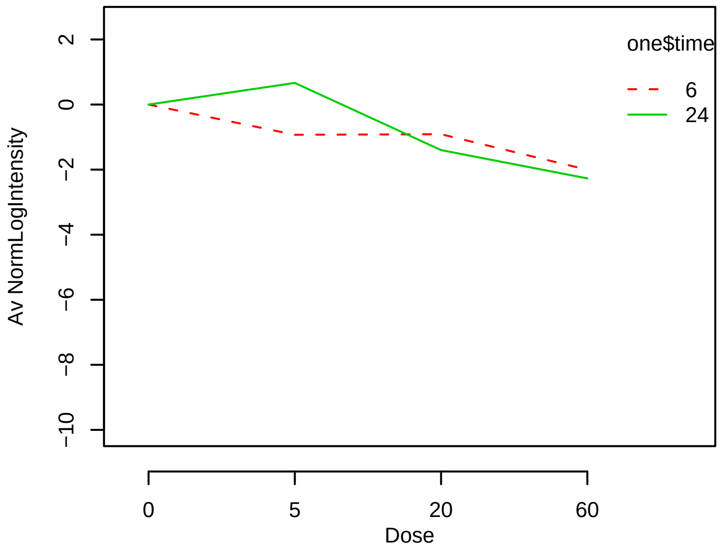

GO_0016485 : protein processing

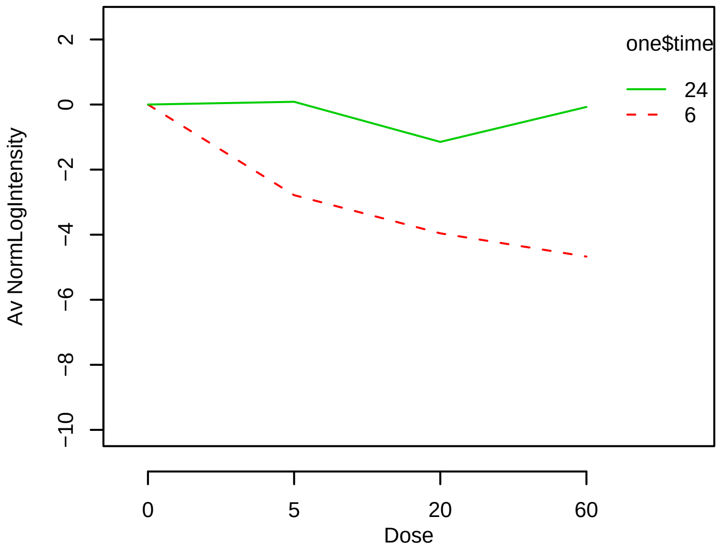

GO_0016486 : peptide hormone processing

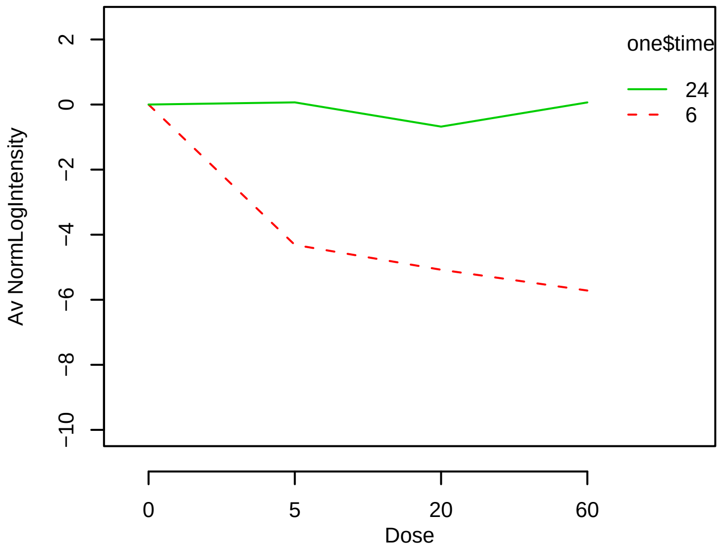

GO_0016525 : negative regulation of angiogenesis

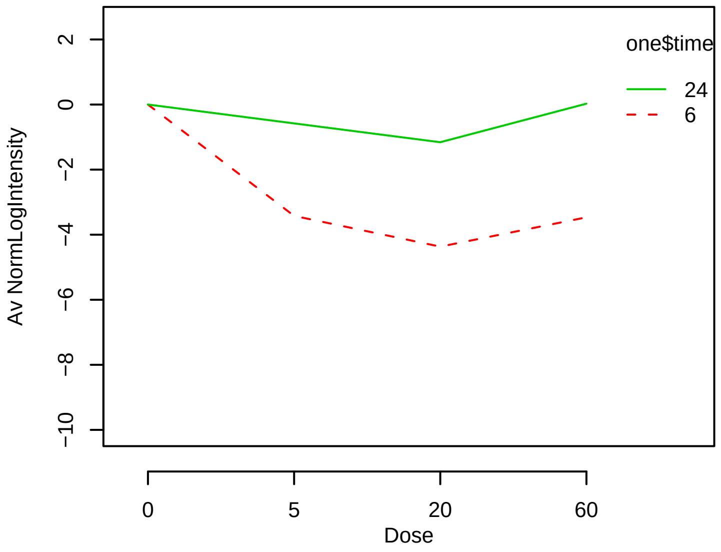

GO_0016540 : protein autoprocessing

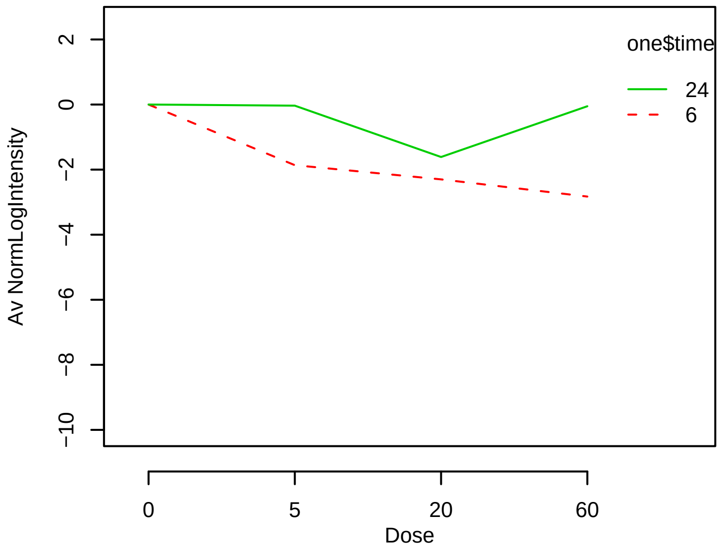

GO_0016547 : RNA editing

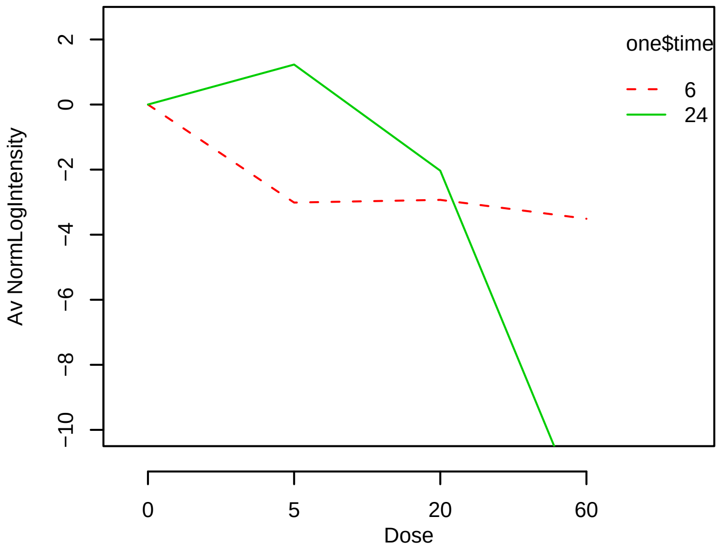

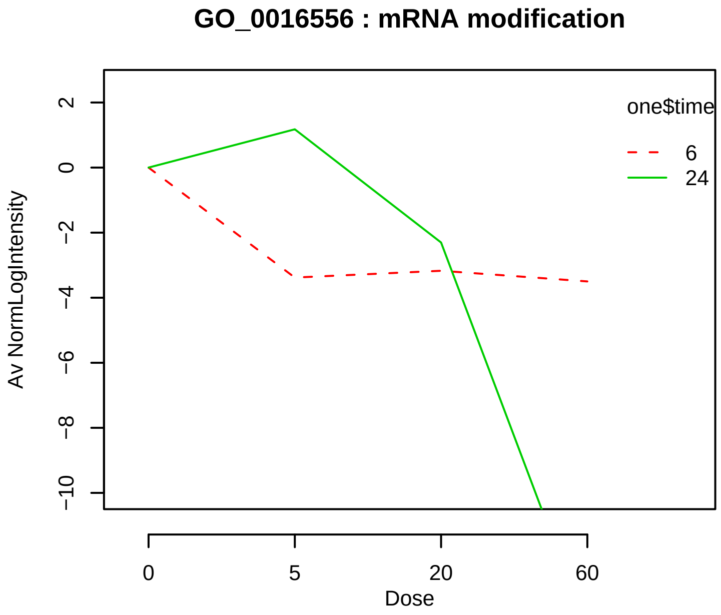
GO_0016556 : mRNA modification
one$time
6
24
Av NormLogIntensity
2
0
−2
−4
−6
−8
−10
0
5
20
60
Dose

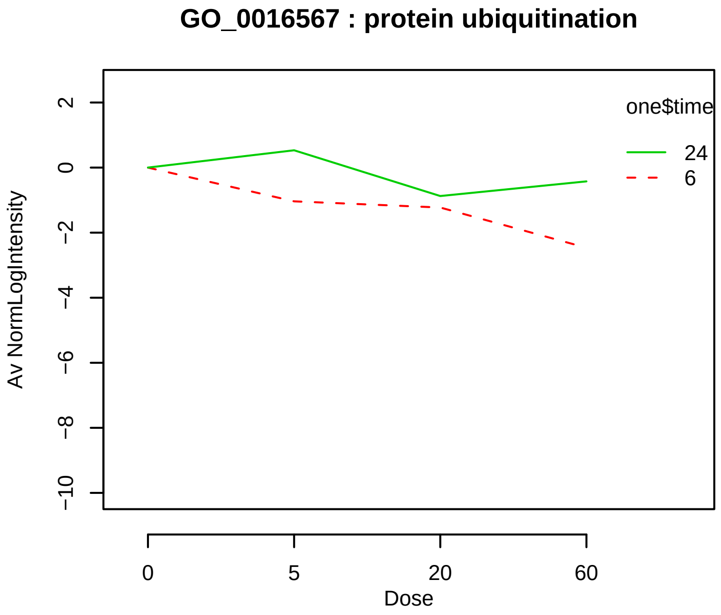
GO_0016567 : protein ubiquitination
one$time
24
6
Av NormLogIntensity
2
0
−2
−4
−6
−8
−10
0
5
20
60
Dose

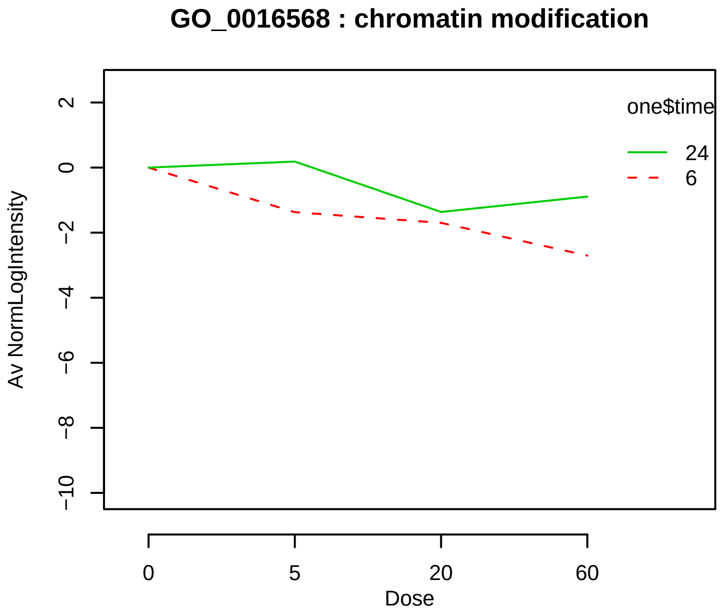
GO_0016568 : chromatin modification
one$time
24
6
Av NormLogIntensity
2
0
−2
−4
−6
−8
−10
0
5
20
60
Dose

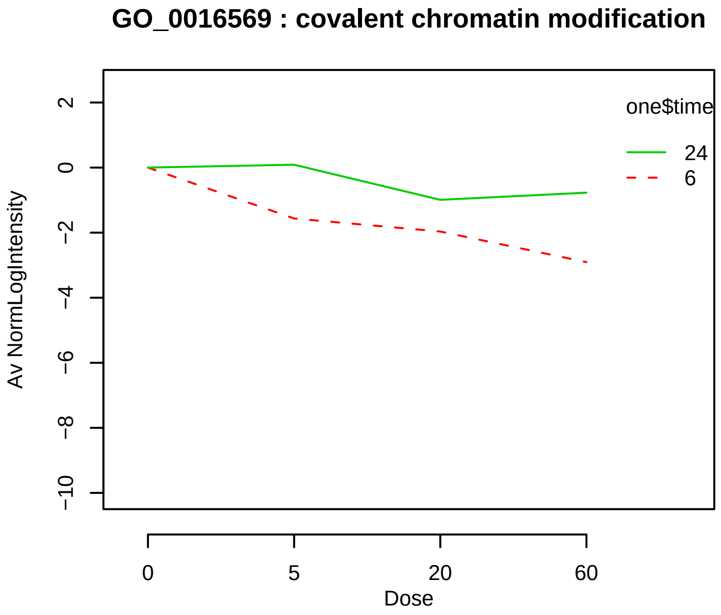
GO_0016569 : covalent chromatin modification
one$time
24
6
Av NormLogIntensity
2
0
−2
−4
−6
−8
−10
0
5
20
60
Dose

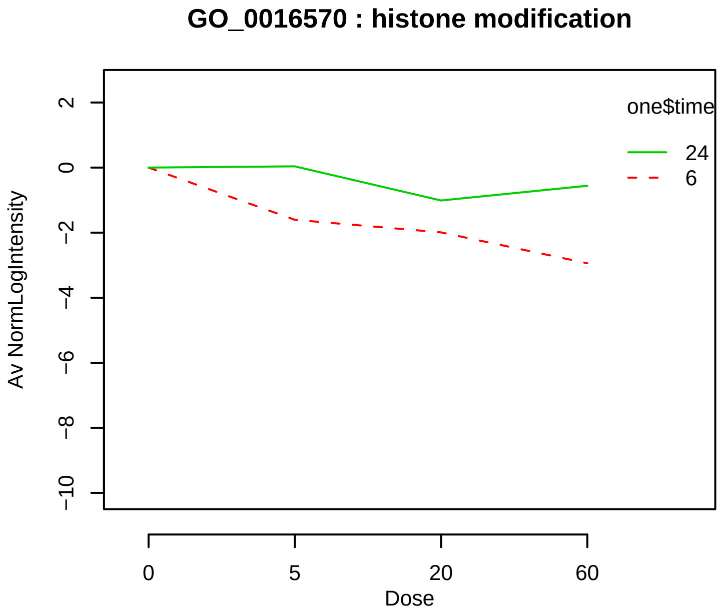
GO_0016570 : histone modification
one$time
24
6
Av NormLogIntensity
2
0
−2
−4
−6
−8
−10
0
5
20
60
Dose

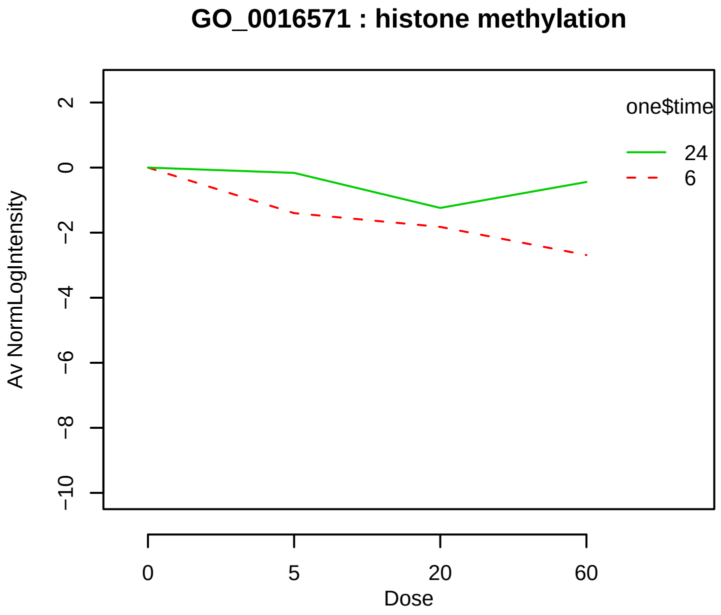
GO_0016571 : histone methylation
one$time
24
6
Av NormLogIntensity
2
0
−2
−4
−6
−8
−10
0
5
20
60
Dose

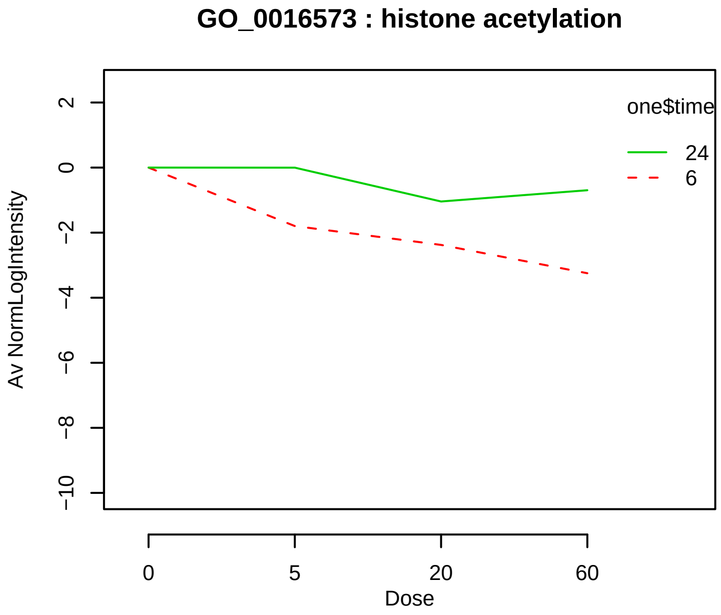
GO_0016573 : histone acetylation
one$time
24
6
Av NormLogIntensity
Dose
2
0
−2
−4
−6
−8
−10
0
5
20
60

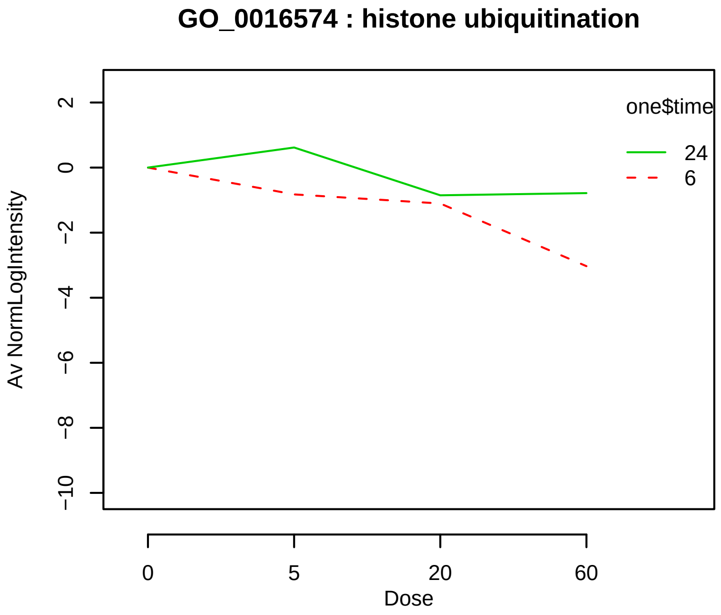
GO_0016574 : histone ubiquitination
one$time
24
6
Av NormLogIntensity
Dose
2
0
−2
−4
−6
−8
−10
0
5
20
60

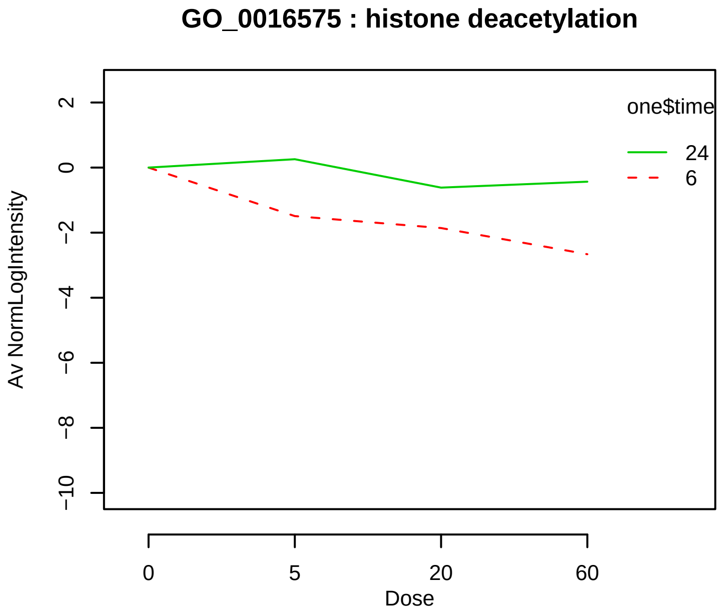
GO_0016575 : histone deacetylation
one$time
24
6
Av NormLogIntensity
Dose
2
0
−2
−4
−6
−8
−10
0
5
20
60

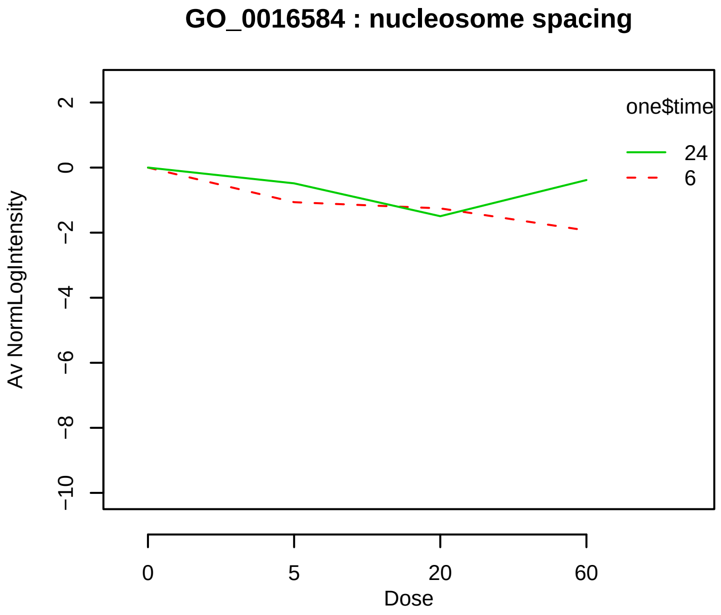
GO_0016584 : nucleosome spacing
one$time
24
6
Av NormLogIntensity
Dose
2
0
−2
−4
−6
−8
−10
0
5
20
60

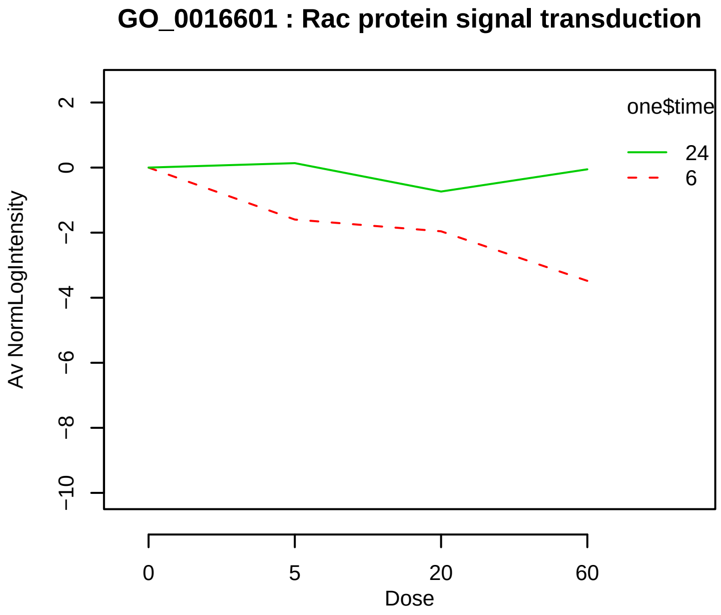
GO_0016601 : Rac protein signal transduction
one$time
24
6
Av NormLogIntensity
Dose
2
0
−2
−4
−6
−8
−10
0
5
20
60

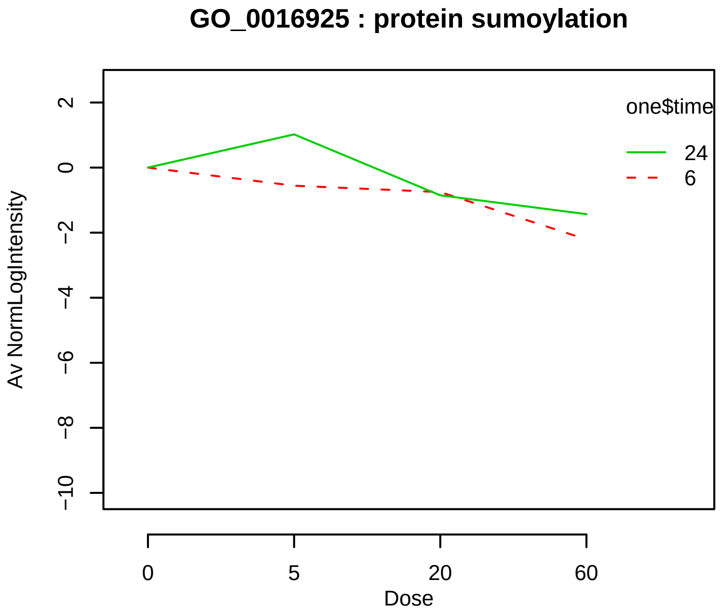
GO_0016925 : protein sumoylation
one$time
24
6
Av NormLogIntensity
Dose
2
0
−2
−4
−6
−8
−10
0
5
20
60

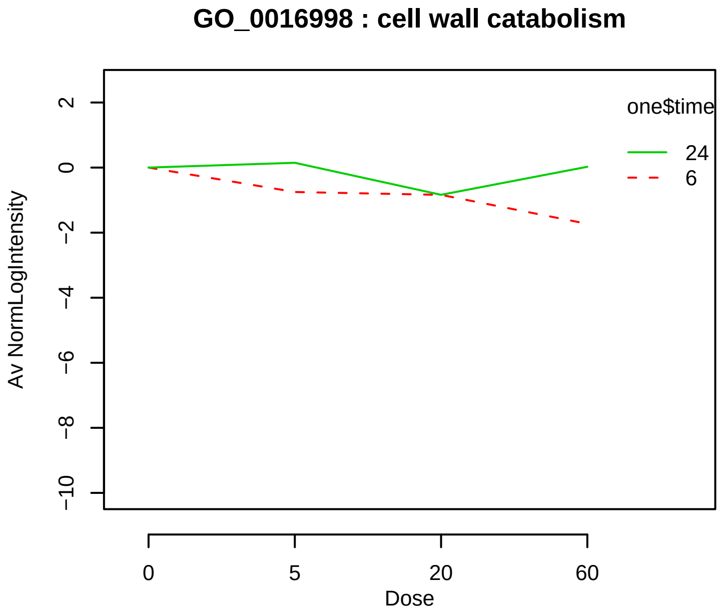
GO_0016998 : cell wall catabolism
one$time
24
6
Av NormLogIntensity
Dose

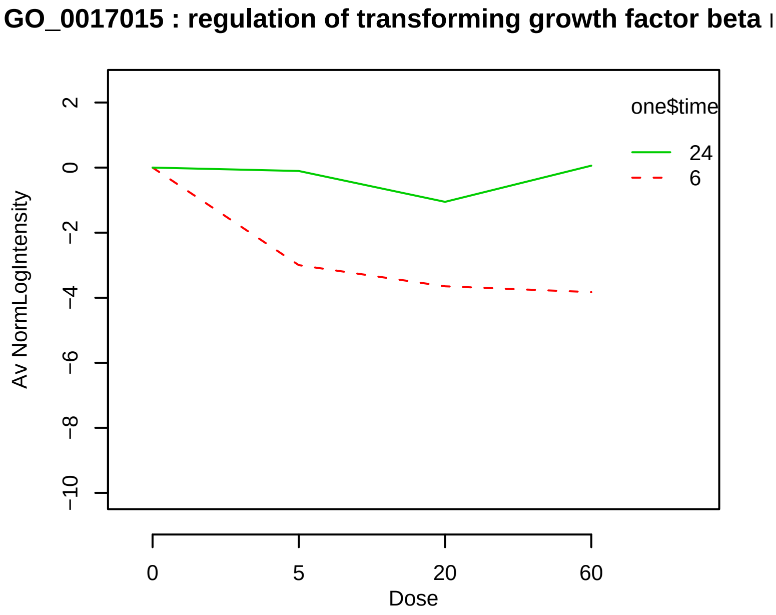
GO_0017015 : regulation of transforming growth factor beta r
one$time
24
6
Av NormLogIntensity
Dose

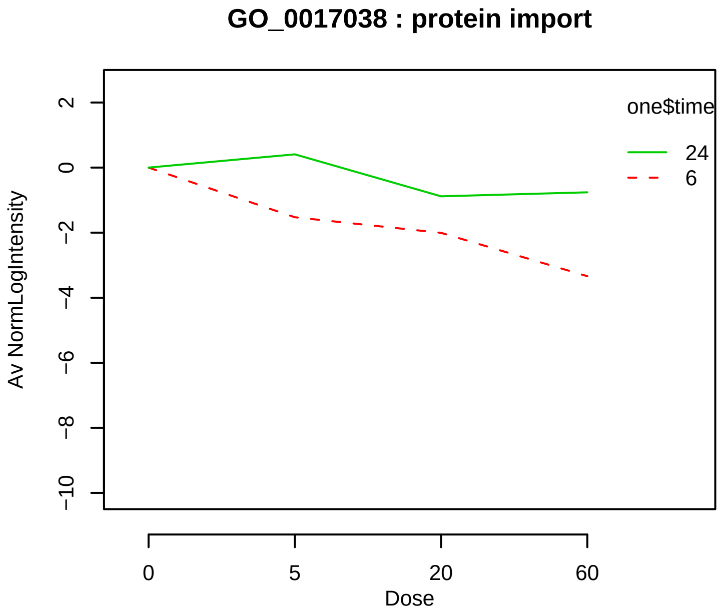
GO_0017038 : protein import
one$time
24
6
Av NormLogIntensity
Dose

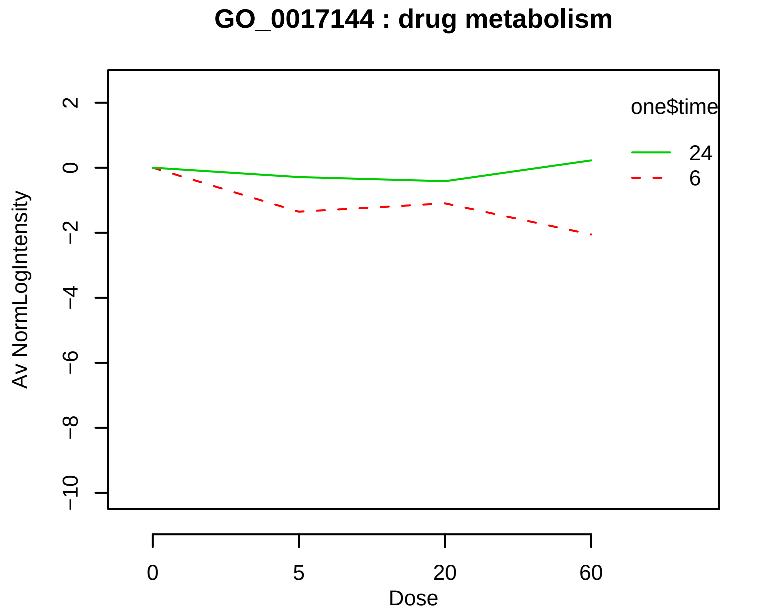
GO_0017144 : drug metabolism
one$time
24
6
Av NormLogIntensity
Dose

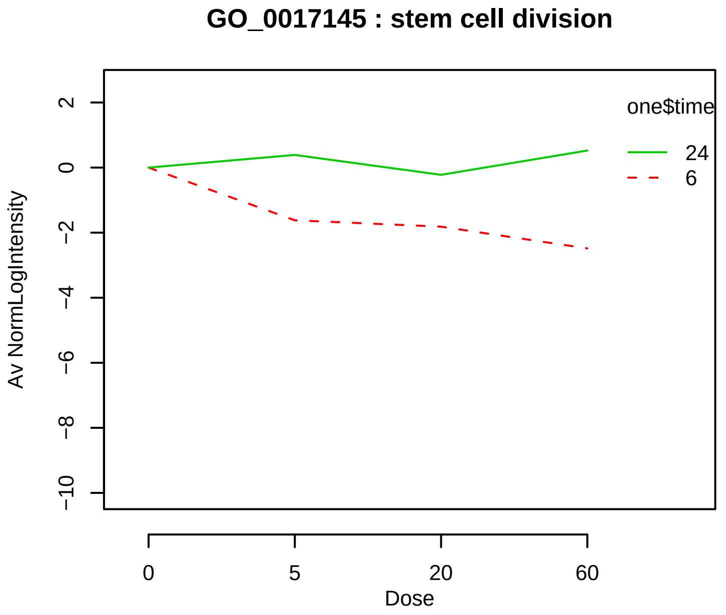
GO_0017145 : stem cell division
one$time
24
6
Av NormLogIntensity
Dose

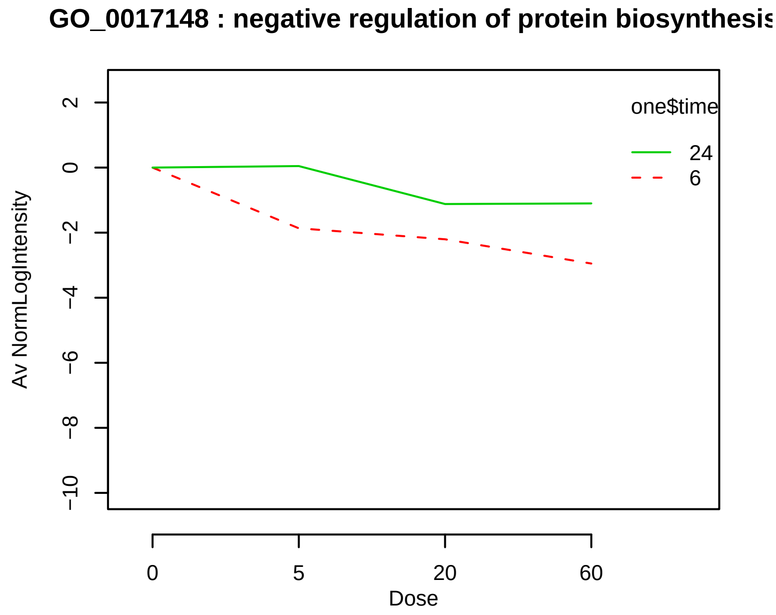
GO_0017148 : negative regulation of protein biosynthesis
one$time
24
6
Av NormLogIntensity
Dose

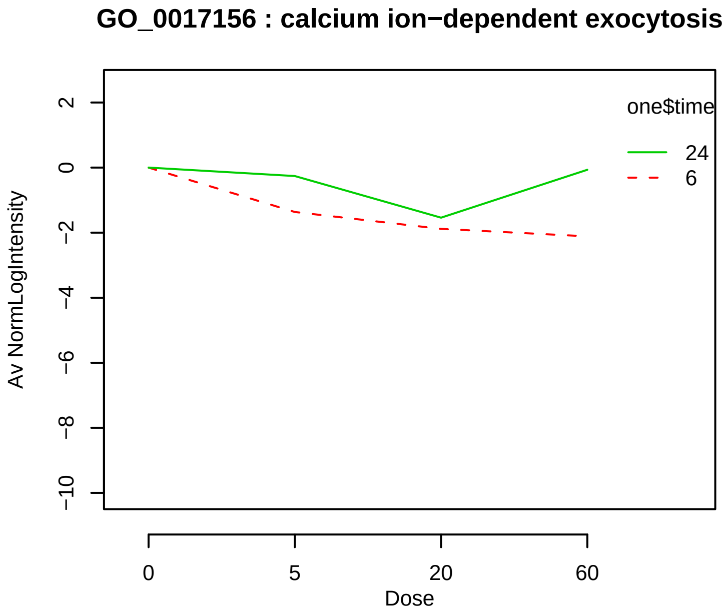
GO_0017156 : calcium ion−dependent exocytosis
one$time
24
6
Av NormLogIntensity
2
0
−2
−4
−6
−8
−10
0
5
20
60
Dose

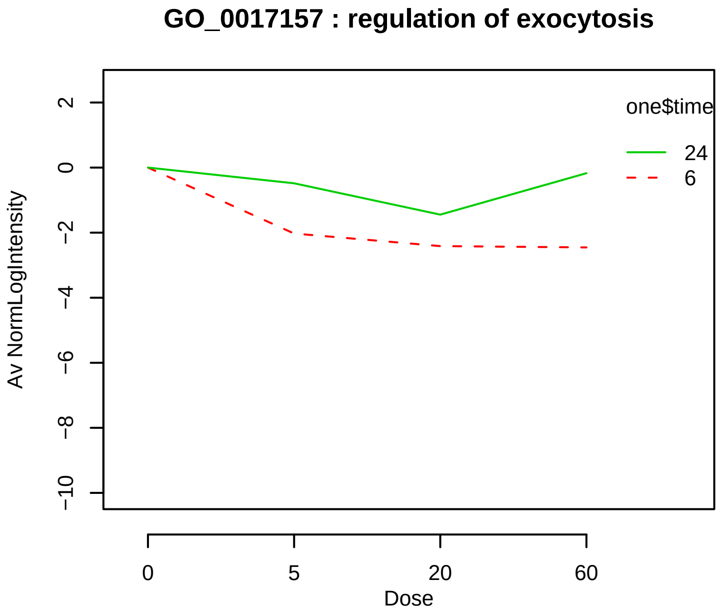
GO_0017157 : regulation of exocytosis
one$time
24
6
Av NormLogIntensity
2
0
−2
−4
−6
−8
−10
0
5
20
60
Dose

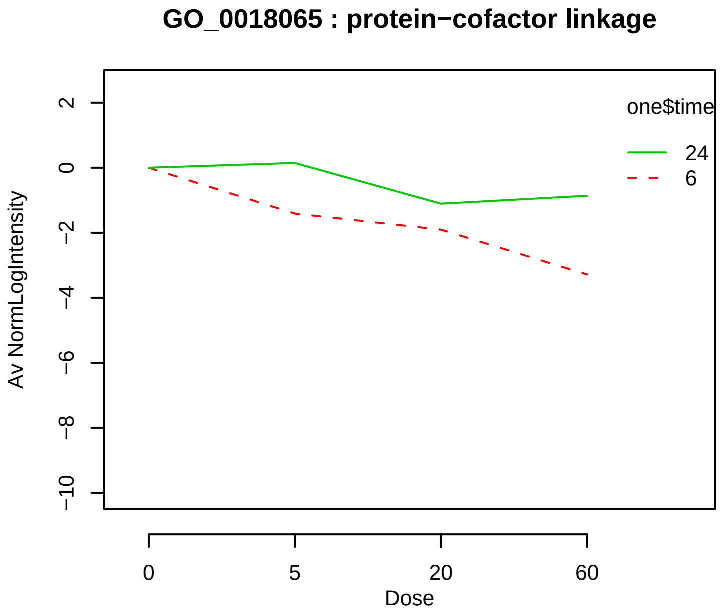
GO_0018065 : protein−cofactor linkage
one$time
24
6
Av NormLogIntensity
2
0
−2
−4
−6
−8
−10
0
5
20
60
Dose

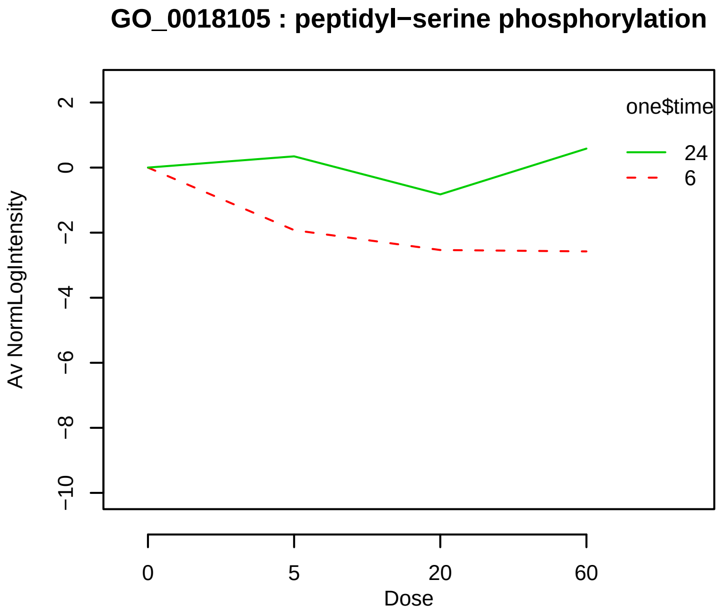
GO_0018105 : peptidyl−serine phosphorylation
one$time
24
6
Av NormLogIntensity
2
0
−2
−4
−6
−8
−10
0
5
20
60
Dose

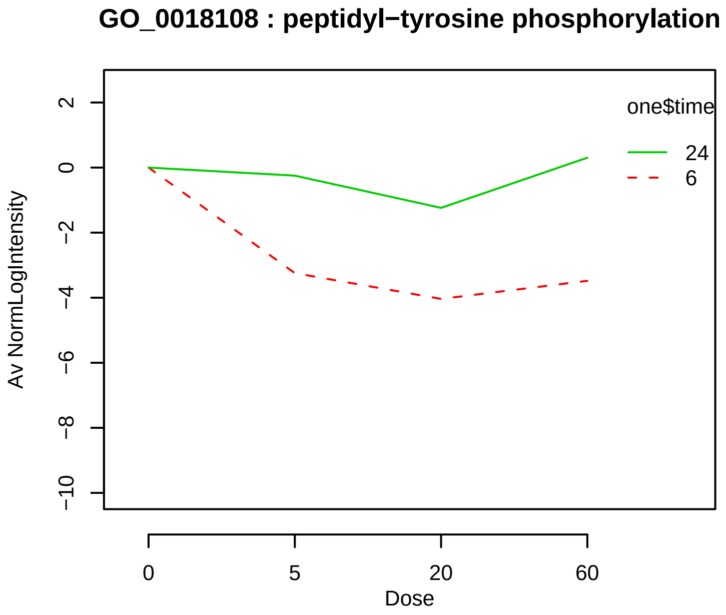
GO_0018108 : peptidyl−tyrosine phosphorylation
one$time
24
6
Av NormLogIntensity
2
0
−2
−4
−6
−8
−10
0
5
20
60
Dose

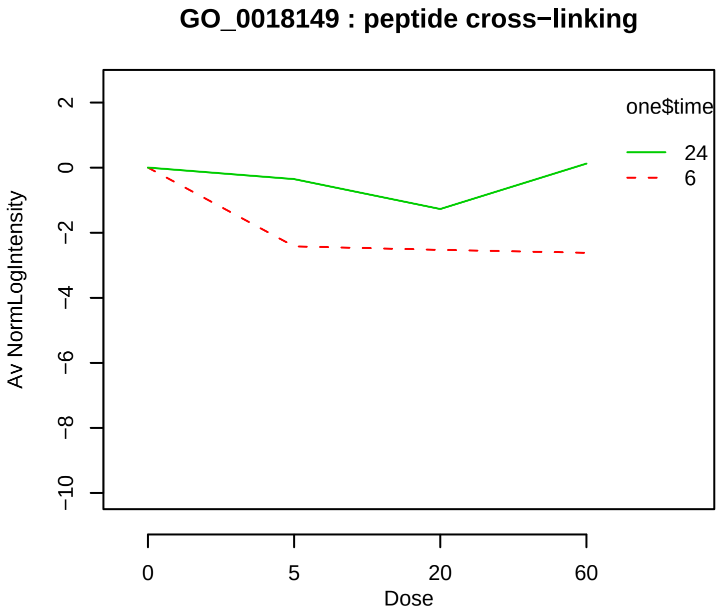
GO_0018149 : peptide cross−linking
one$time
24
6
Av NormLogIntensity
2
0
−2
−4
−6
−8
−10
0
5
20
60
Dose

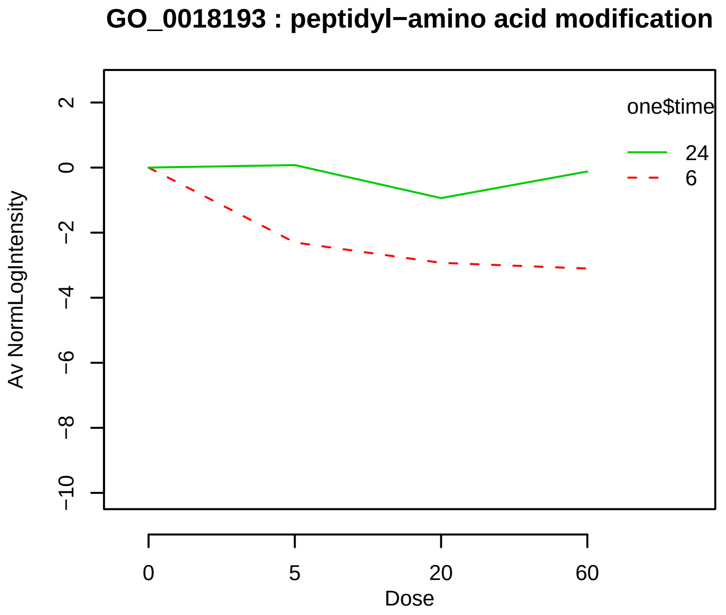
GO_0018193 : peptidyl−amino acid modification
one$time
24
6
Av NormLogIntensity
2
0
−2
−4
−6
−8
−10
0
5
20
60
Dose

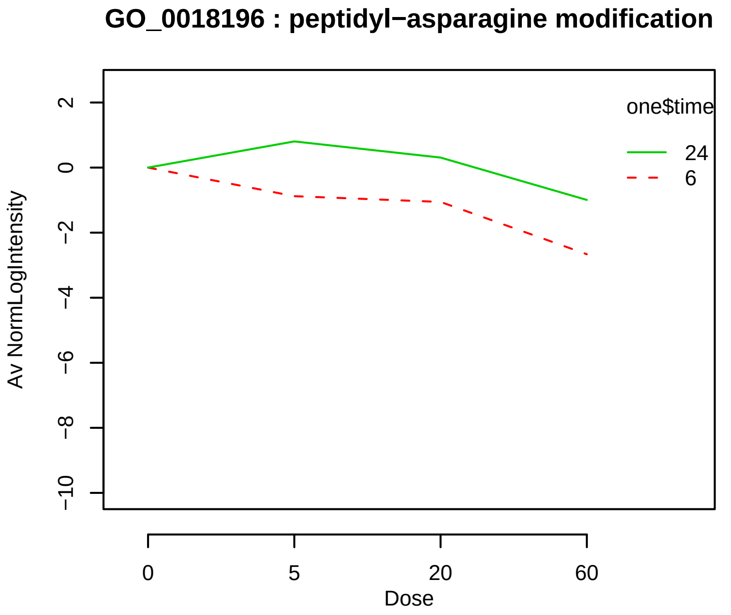
GO_0018196 : peptidyl−asparagine modification
one$time
24
6
Av NormLogIntensity
2
0
−2
−4
−6
−8
−10
0
5
20
60
Dose

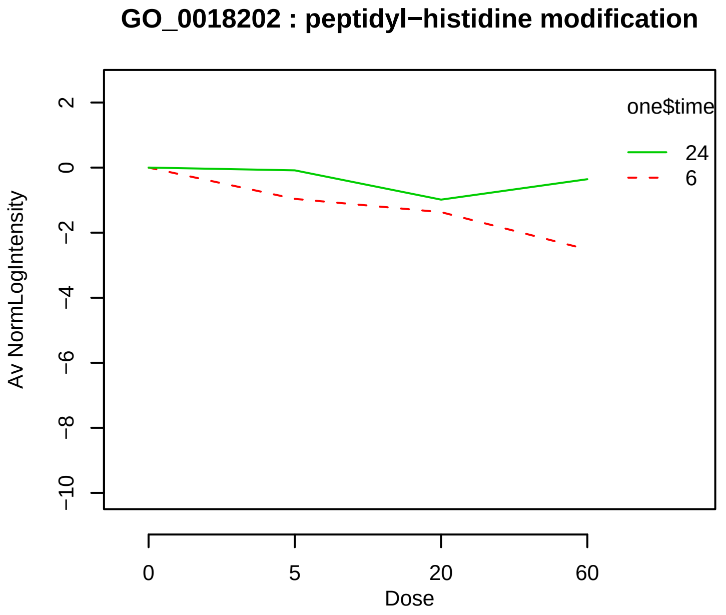
GO_0018202 : peptidyl−histidine modification
one$time
24
6
Av NormLogIntensity
2
0
−2
−4
−6
−8
−10
0
5
20
60
Dose

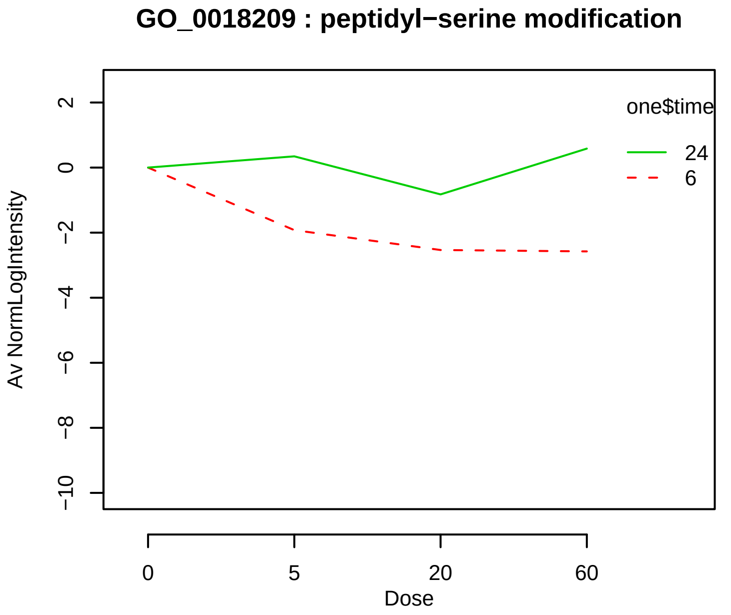
GO_0018209 : peptidyl−serine modification
one$time
24
6
Av NormLogIntensity
2
0
−2
−4
−6
−8
−10
0
5
20
60
Dose

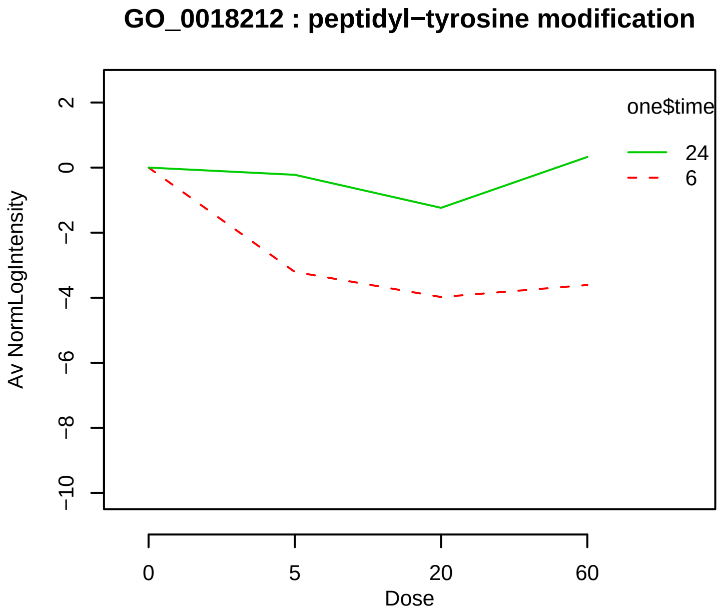
GO_0018212 : peptidyl−tyrosine modification
one$time
24
6
Av NormLogIntensity
2
0
−2
−4
−6
−8
−10
0
5
20
60
Dose

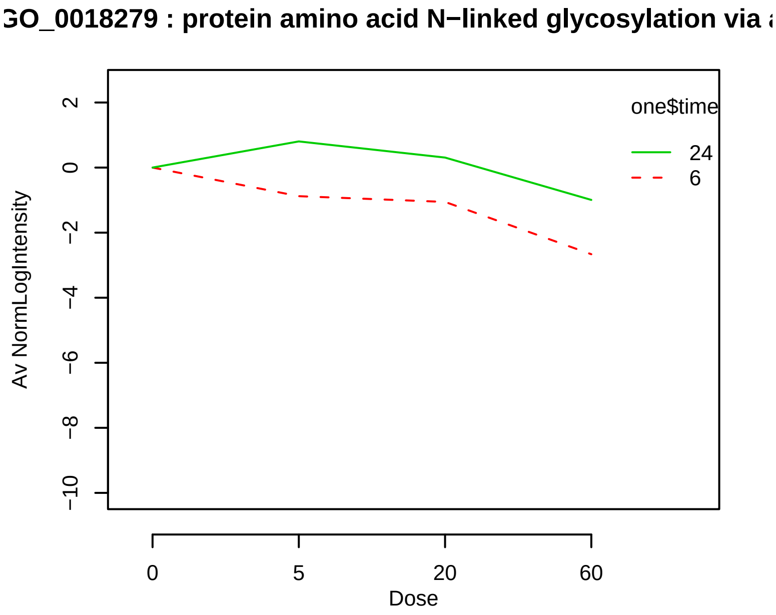
GO_0018279 : protein amino acid N−linked glycosylation via
one$time
24
6
Av NormLogIntensity
2
0
−2
−4
−6
−8
−10
0
5
20
60
Dose

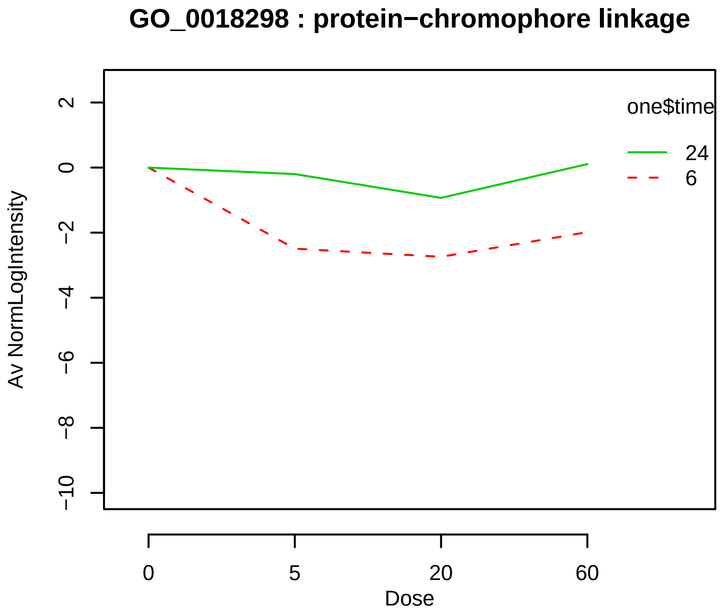
GO_0018298 : protein−chromophore linkage
one$time
24
6
Av NormLogIntensity
2
0
−2
−4
−6
−8
−10
0
5
20
60
Dose

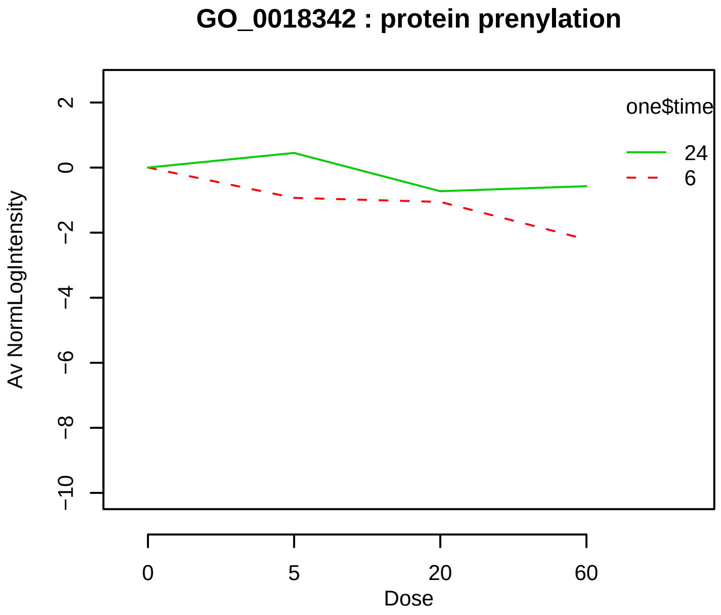
GO_0018342 : protein prenylation
one$time
24
6
Av NormLogIntensity
2
0
−2
−4
−6
−8
−10
0
5
20
60
Dose

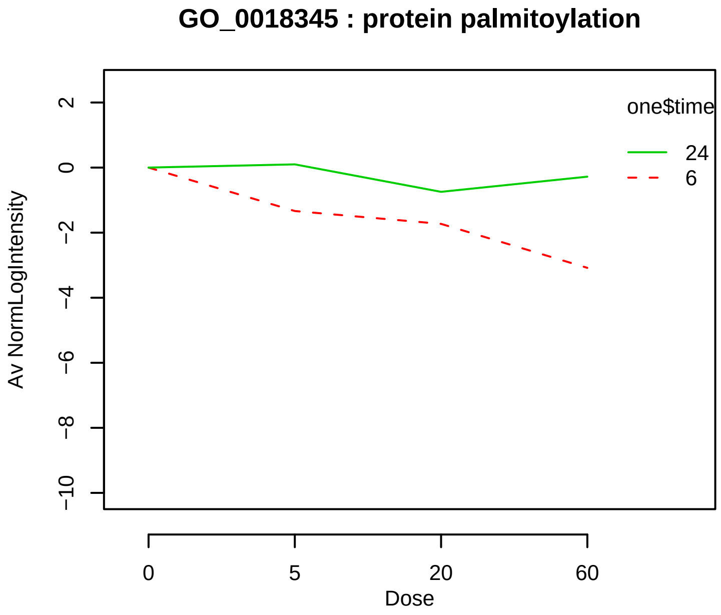
GO_0018345 : protein palmitoylation
one$time
24
6
Av NormLogIntensity
2
0
−2
−4
−6
−8
−10
0
5
20
60
Dose

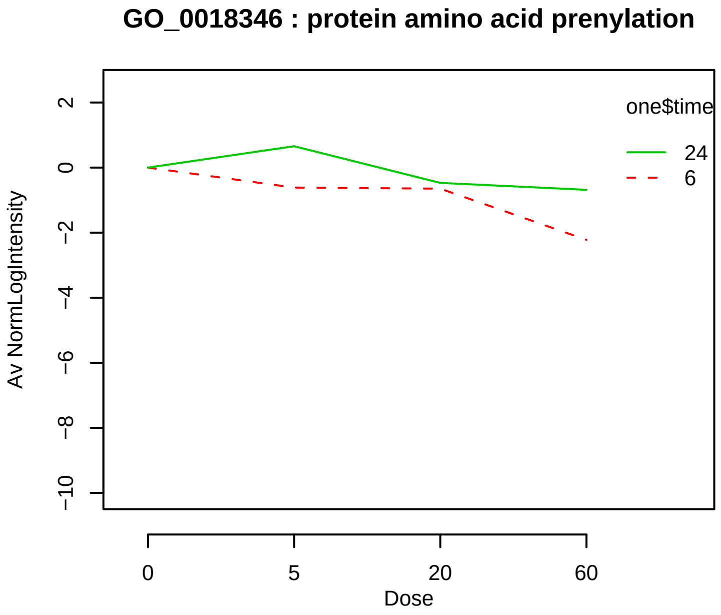
GO_0018346 : protein amino acid prenylation
one$time
24
6
Av NormLogIntensity
2
0
−2
−4
−6
−8
−10
0
5
20
60
Dose

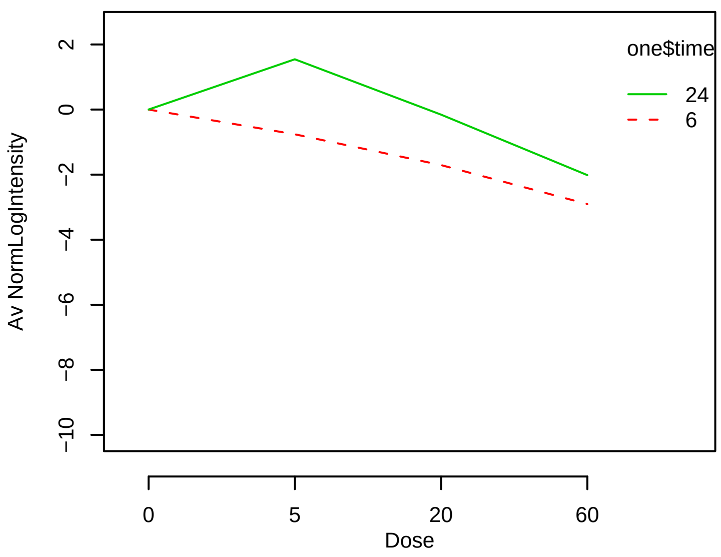
one$time
24
6
Av NormLogIntensity
2
0
−2
−4
−6
−8
−10
0
5
20
60
Dose

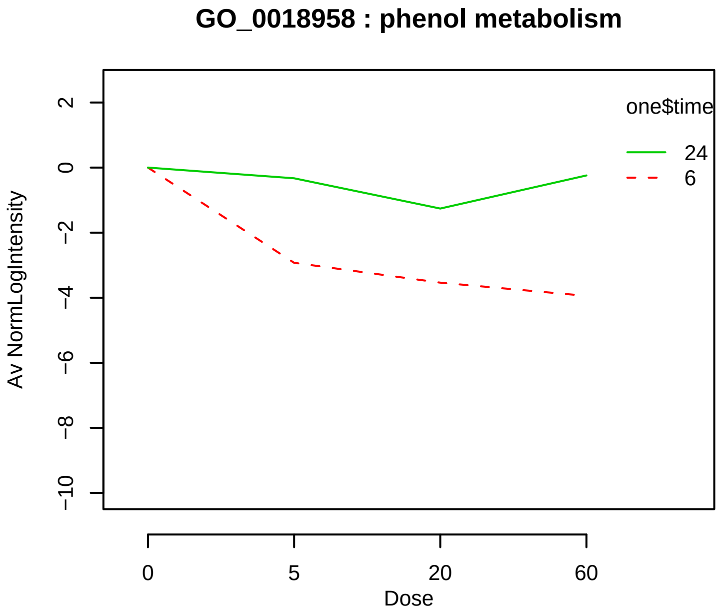
GO_0018958 : phenol metabolism
one$time
24
6
Av NormLogIntensity
2
0
−2
−4
−6
−8
−10
0
5
20
60
Dose

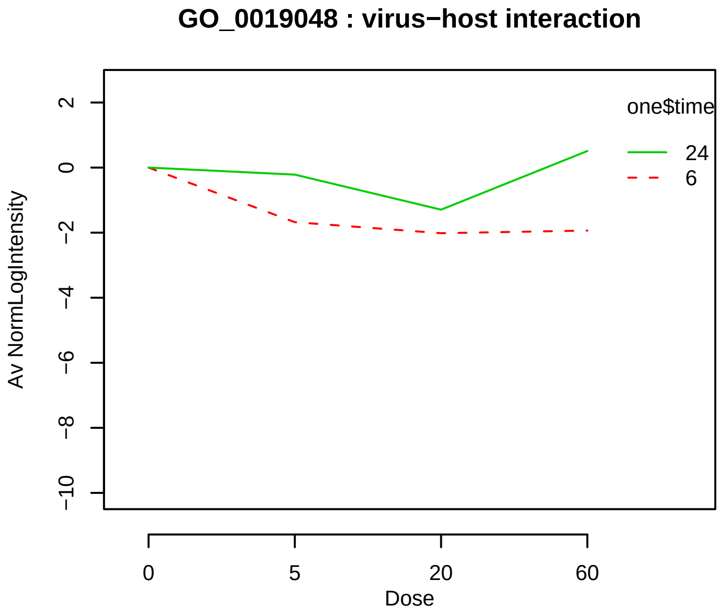
GO_0019048 : virus−host interaction
Av NormLogIntensity
2
0
−2
−4
−6
−8
−10
one$time
24
6
0
5
20
60
Dose

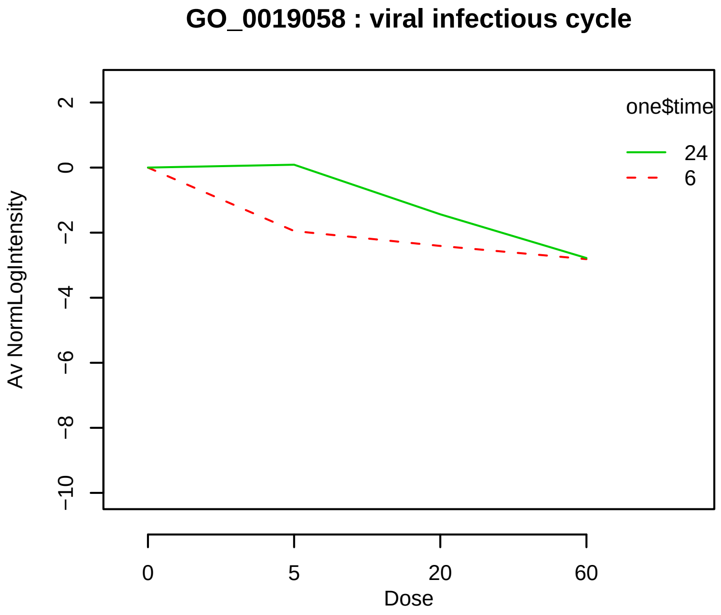
GO_0019058 : viral infectious cycle
Av NormLogIntensity
2
0
−2
−4
−6
−8
−10
one$time
24
6
0
5
20
60
Dose

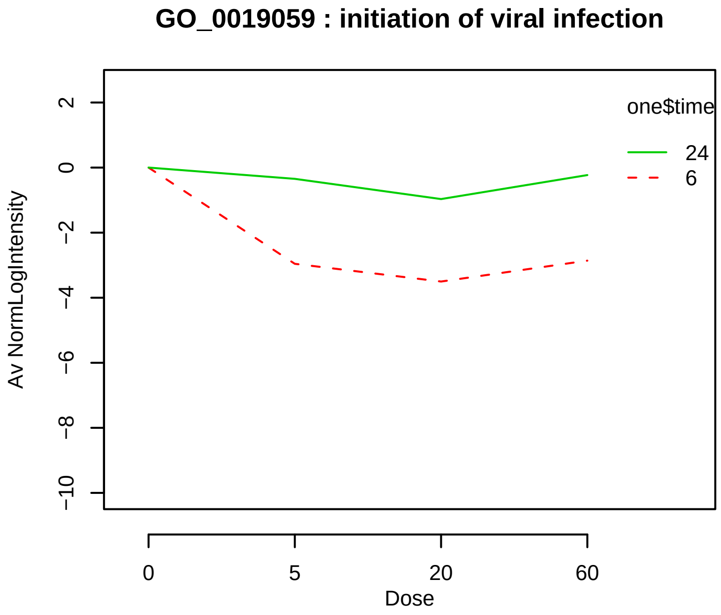
GO_0019059 : initiation of viral infection
Av NormLogIntensity
2
0
−2
−4
−6
−8
−10
one$time
24
6
0
5
20
60
Dose

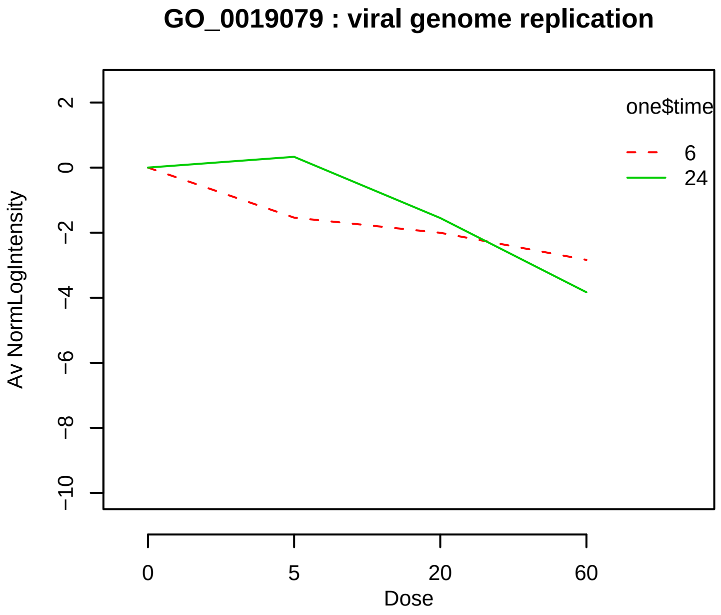
GO_0019079 : viral genome replication
Av NormLogIntensity
2
0
−2
−4
−6
−8
−10
one$time
6
24
0
5
20
60
Dose

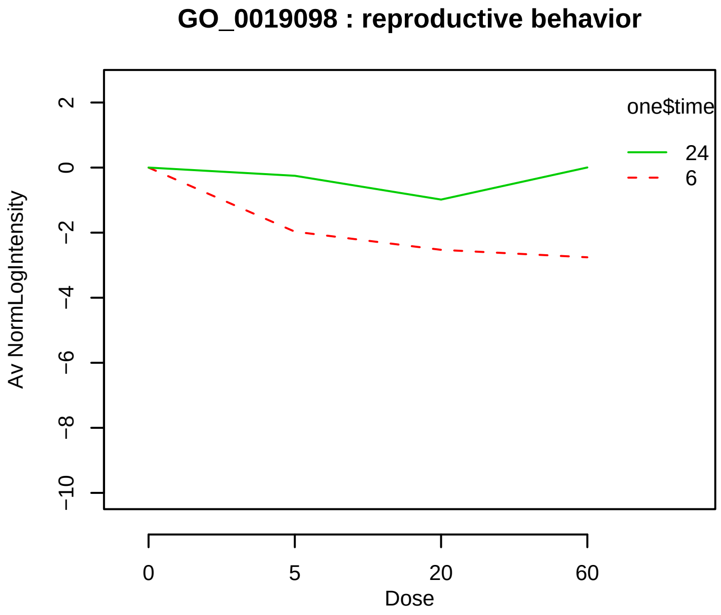
GO_0019098 : reproductive behavior
Av NormLogIntensity
2
0
−2
−4
−6
−8
−10
one$time
24
6
0
5
20
60
Dose

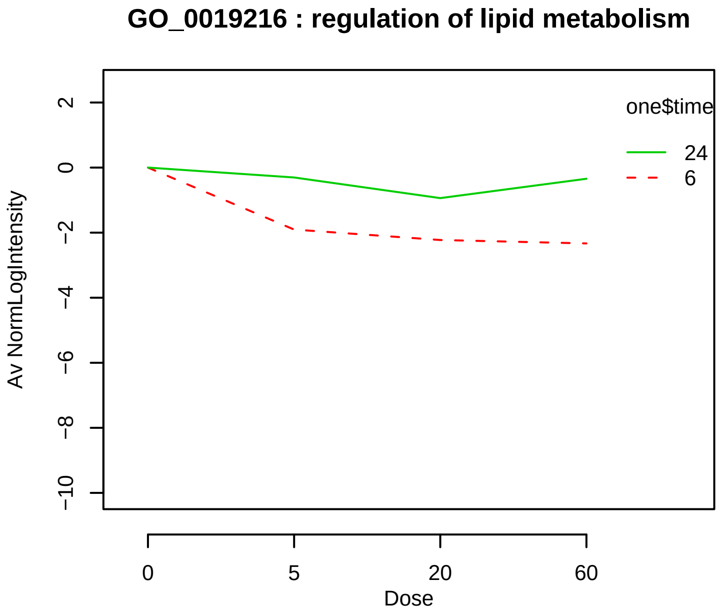
GO_0019216 : regulation of lipid metabolism
Av NormLogIntensity
2
0
−2
−4
−6
−8
−10
one$time
24
6
0
5
20
60
Dose

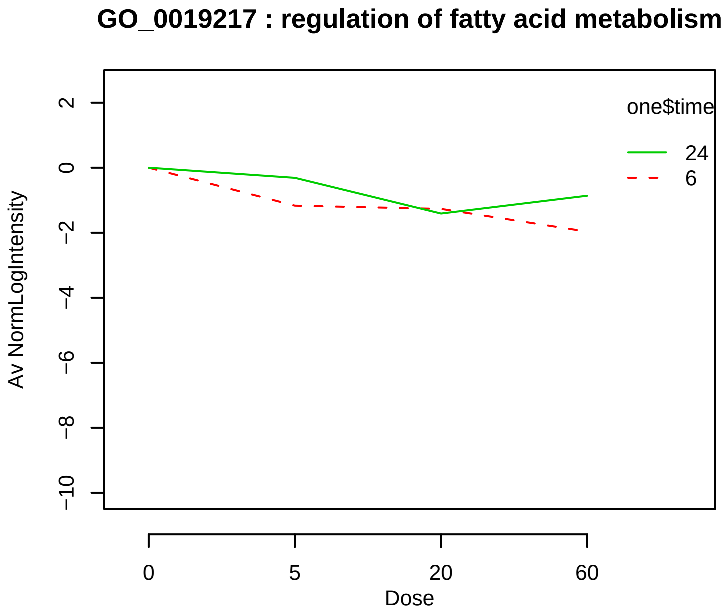
GO_0019217 : regulation of fatty acid metabolism
Av NormLogIntensity
Dose
one$time
24
6

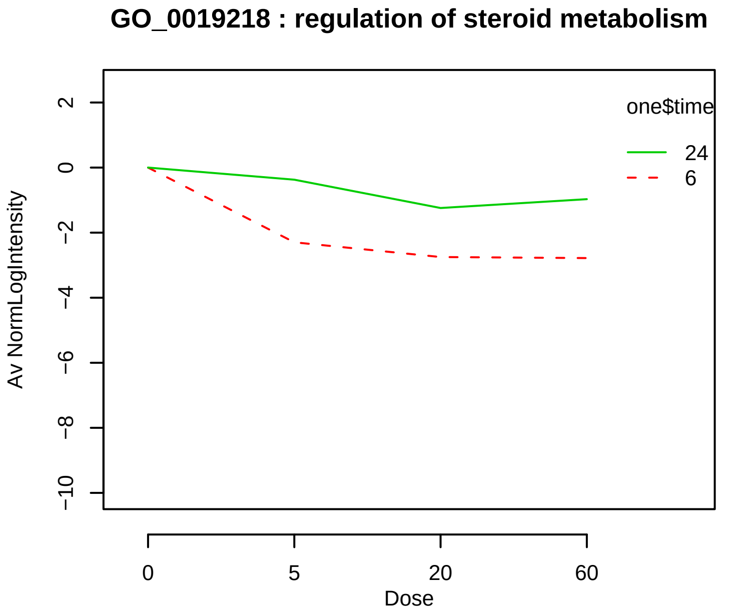
GO_0019218 : regulation of steroid metabolism
Av NormLogIntensity
Dose
one$time
24
6

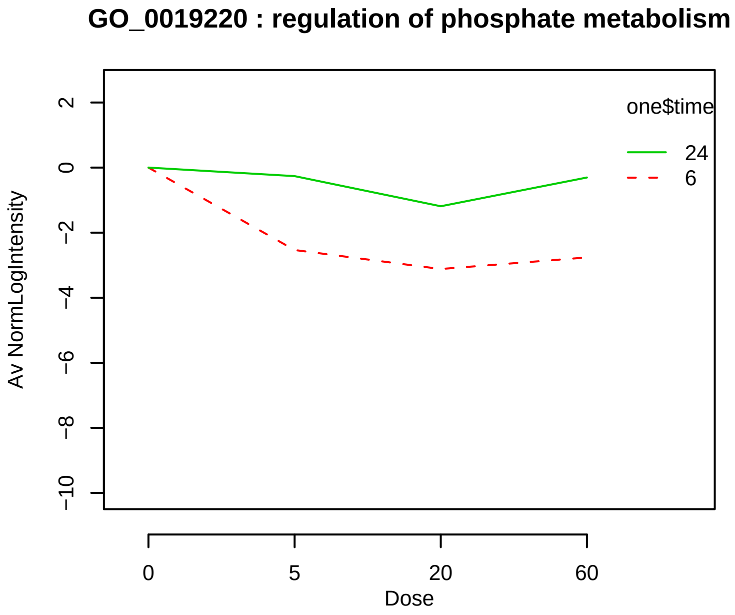
GO_0019220 : regulation of phosphate metabolism
Av NormLogIntensity
Dose
one$time
24
6

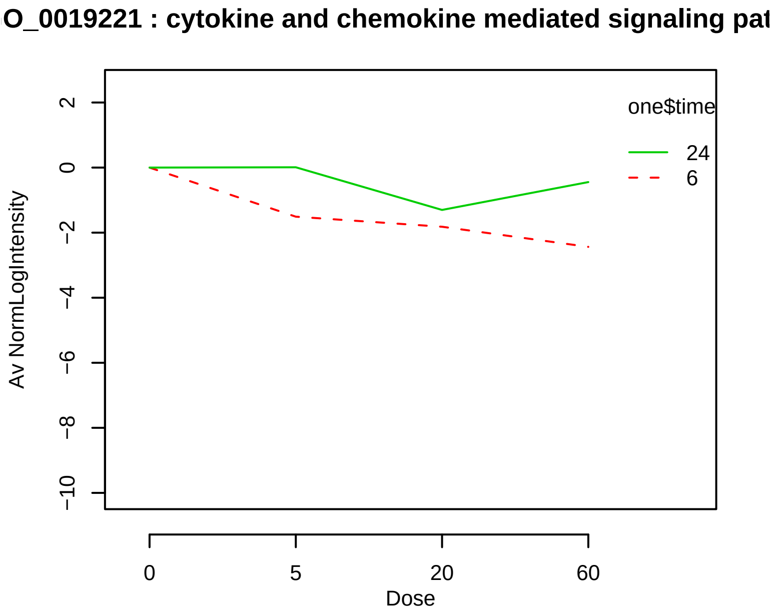
O_0019221 : cytokine and chemokine mediated signaling pat
Av NormLogIntensity
Dose
one$time
24
6

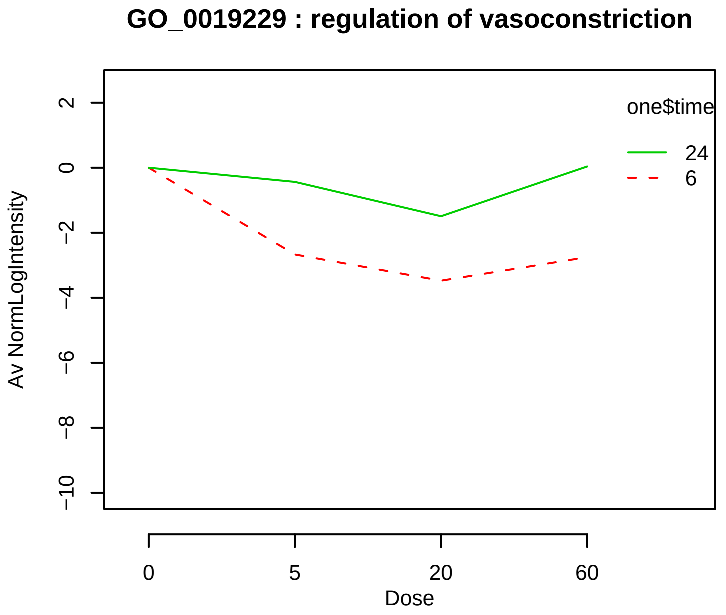
GO_0019229 : regulation of vasoconstriction
Av NormLogIntensity
Dose
one$time
24
6

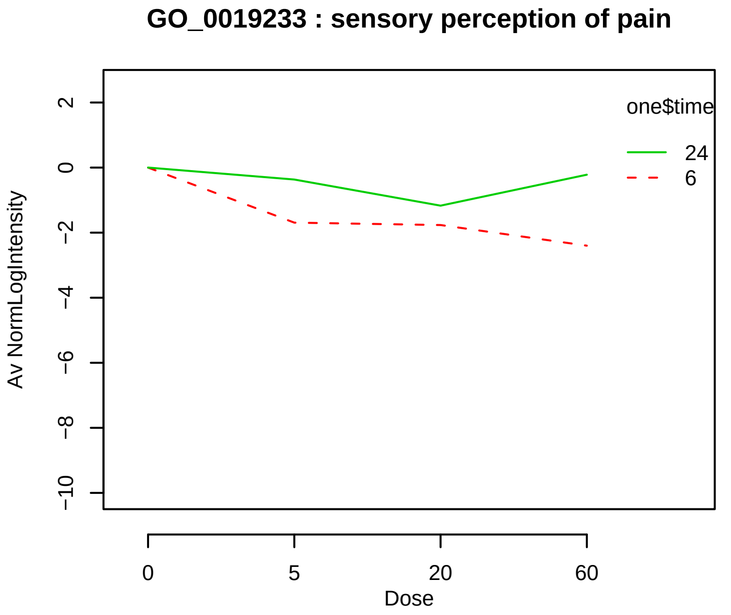
GO_0019233 : sensory perception of pain
Av NormLogIntensity
Dose
one$time
24
6

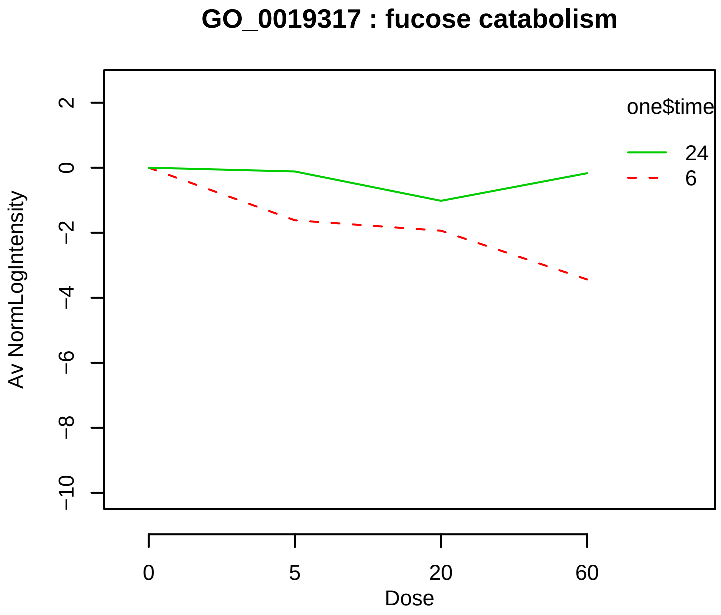
GO_0019317 : fucose catabolism
one$time
24
6
Av NormLogIntensity
2
0
−2
−4
−6
−8
−10
0
5
20
60
Dose

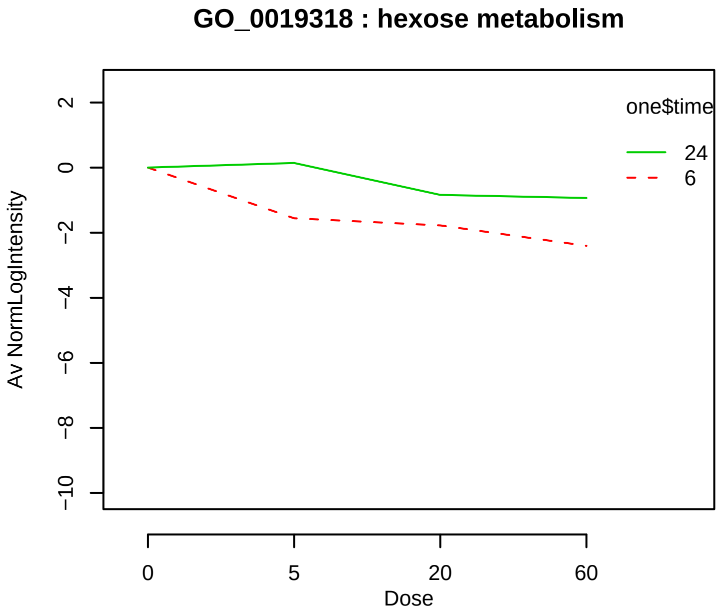
GO_0019318 : hexose metabolism
one$time
24
6
Av NormLogIntensity
2
0
−2
−4
−6
−8
−10
0
5
20
60
Dose

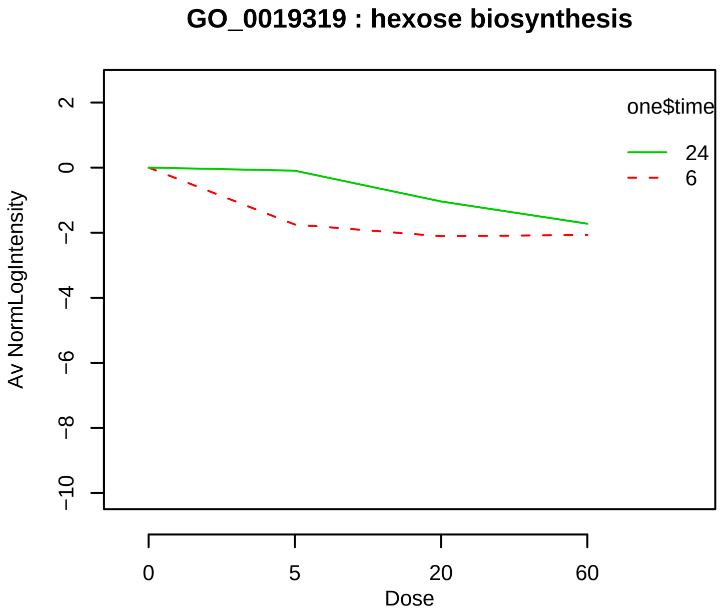
GO_0019319 : hexose biosynthesis
one$time
24
6
Av NormLogIntensity
2
0
−2
−4
−6
−8
−10
0
5
20
60
Dose

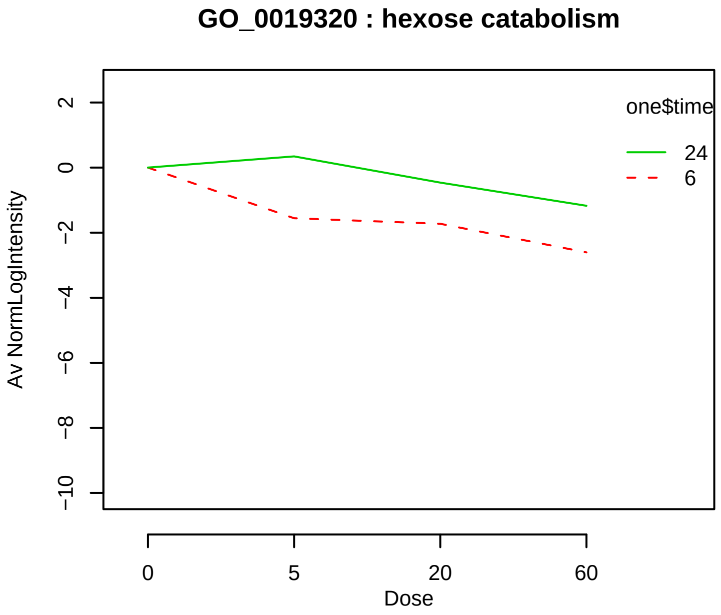
GO_0019320 : hexose catabolism
one$time
24
6
Av NormLogIntensity
2
0
−2
−4
−6
−8
−10
0
5
20
60
Dose

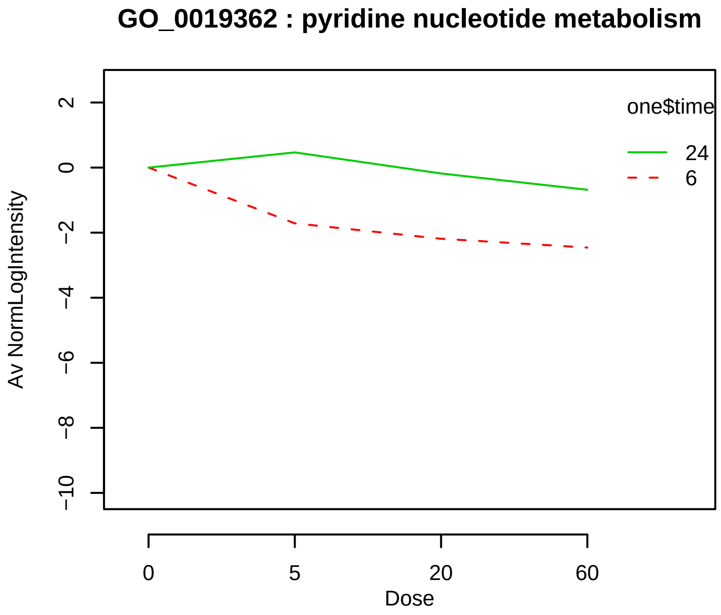
GO_0019362 : pyridine nucleotide metabolism
one$time
24
6
Av NormLogIntensity
2
0
−2
−4
−6
−8
−10
0
5
20
60
Dose

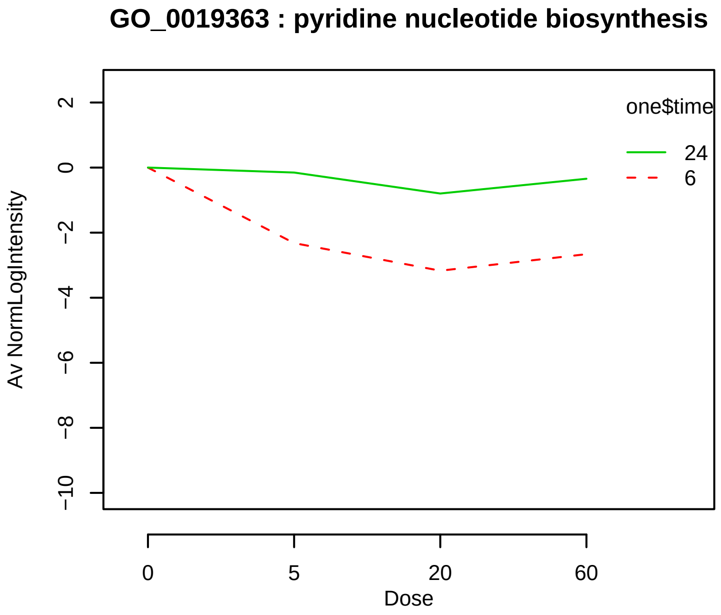
GO_0019363 : pyridine nucleotide biosynthesis
one$time
24
6
Av NormLogIntensity
2
0
−2
−4
−6
−8
−10
0
5
20
60
Dose

GO_0019369 : arachidonic acid metabolism

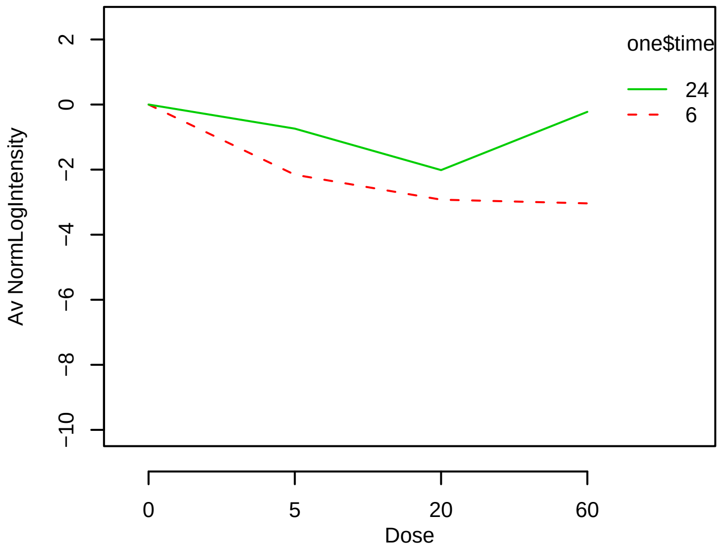

GO_0019370 : leukotriene biosynthesis

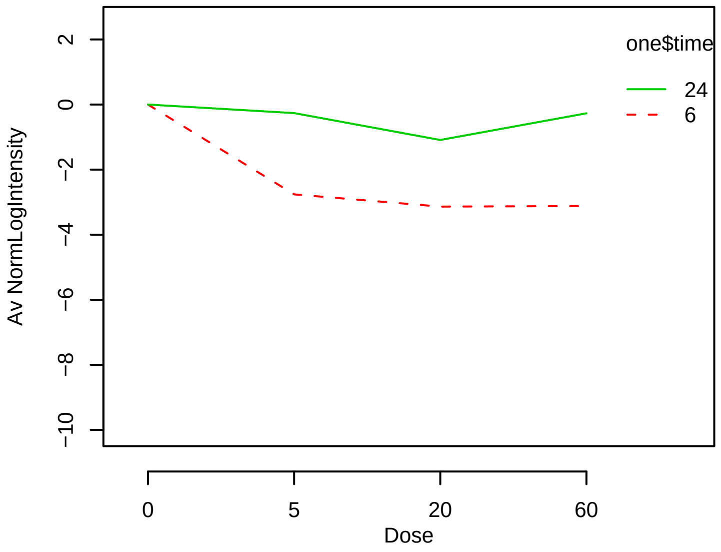

GO_0019377 : glycolipid catabolism

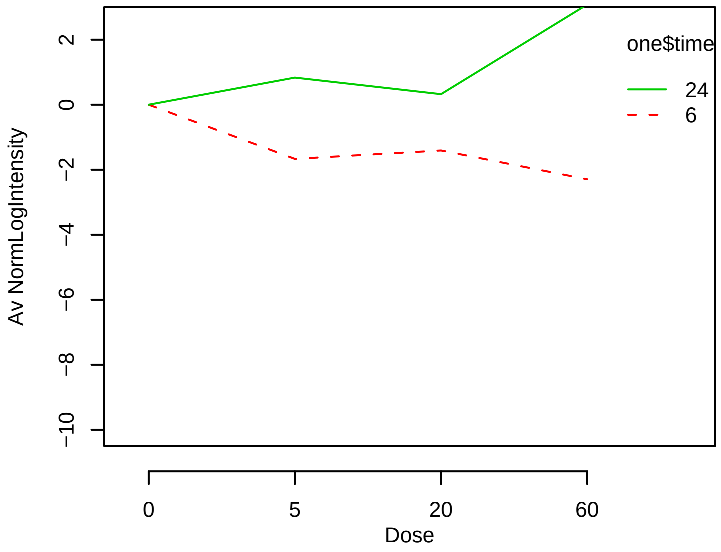

GO_0019395 : fatty acid oxidation

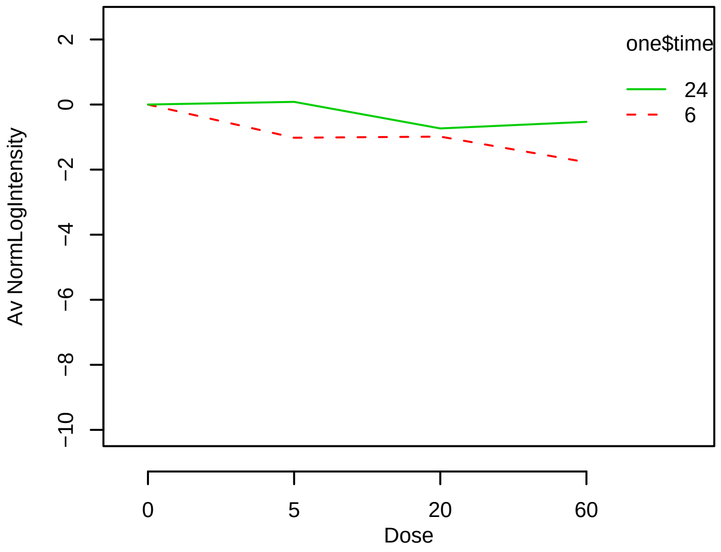

GO_0019438 : aromatic compound biosynthesis

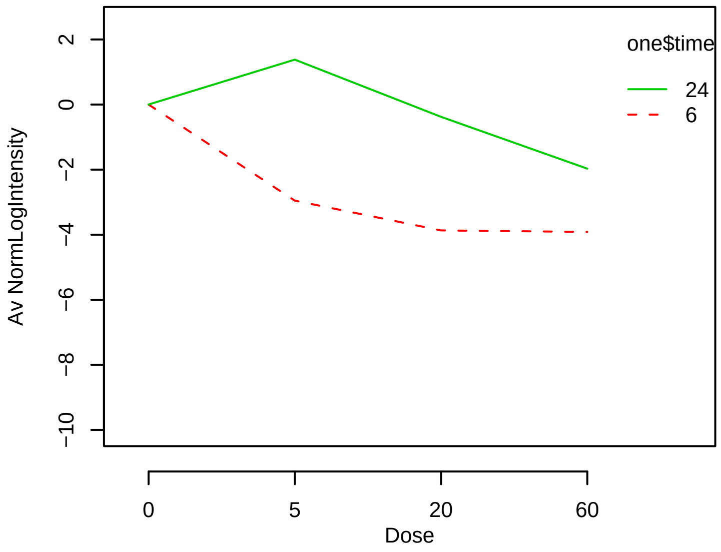

GO_0019439 : aromatic compound catabolism

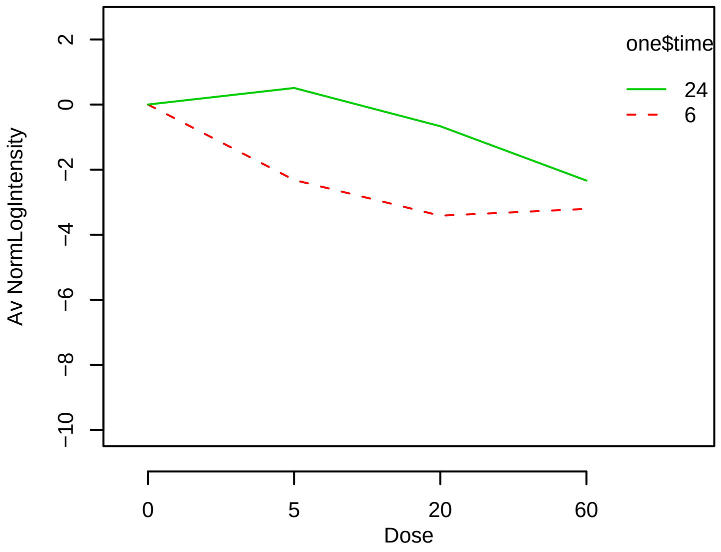

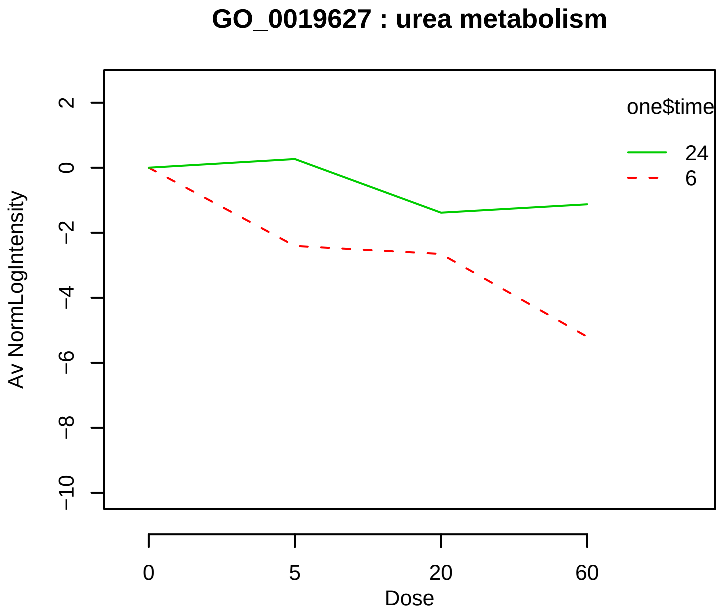
GO_0019627 : urea metabolism
one$time
24
6
Av NormLogIntensity
Dose

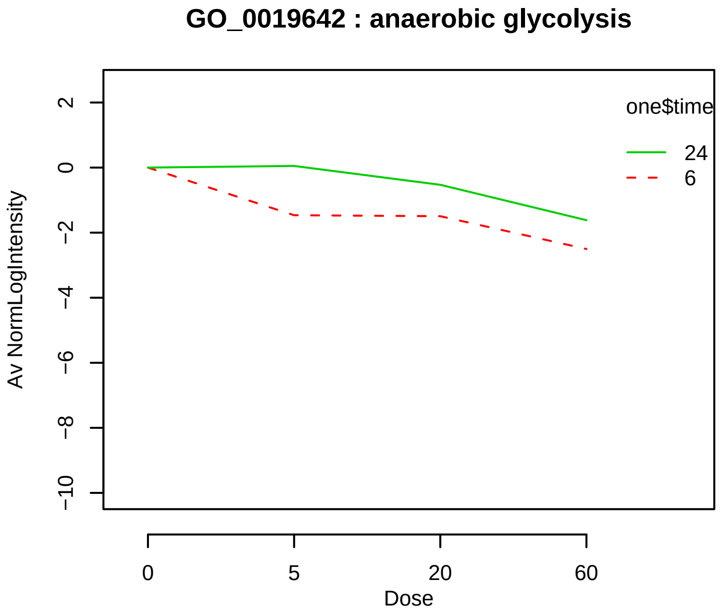
GO_0019642 : anaerobic glycolysis
one$time
24
6
Av NormLogIntensity
Dose

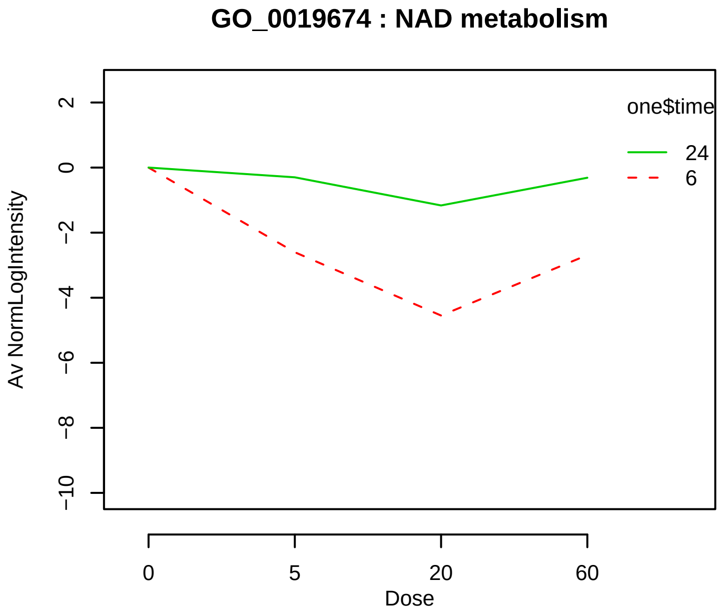
GO_0019674 : NAD metabolism
one$time
24
6
Av NormLogIntensity
Dose

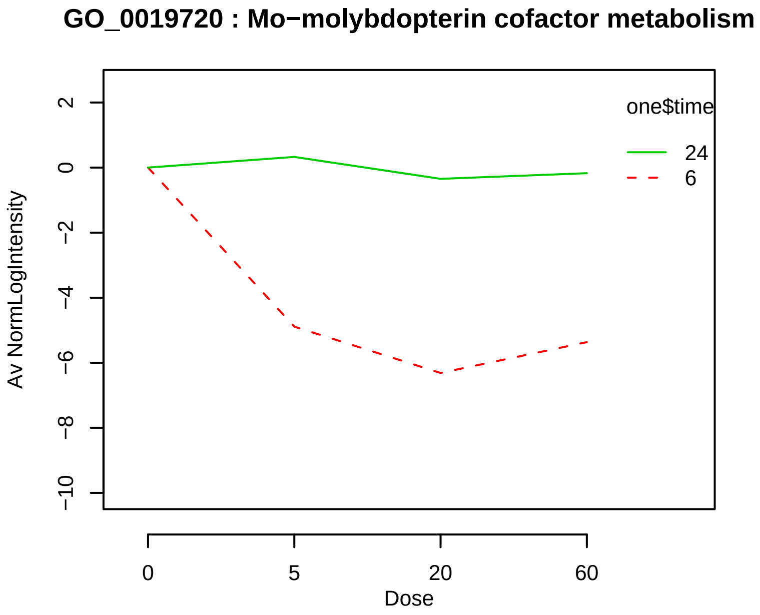
GO_0019720 : Mo−molybdopterin cofactor metabolism
one$time
24
6
Av NormLogIntensity
Dose

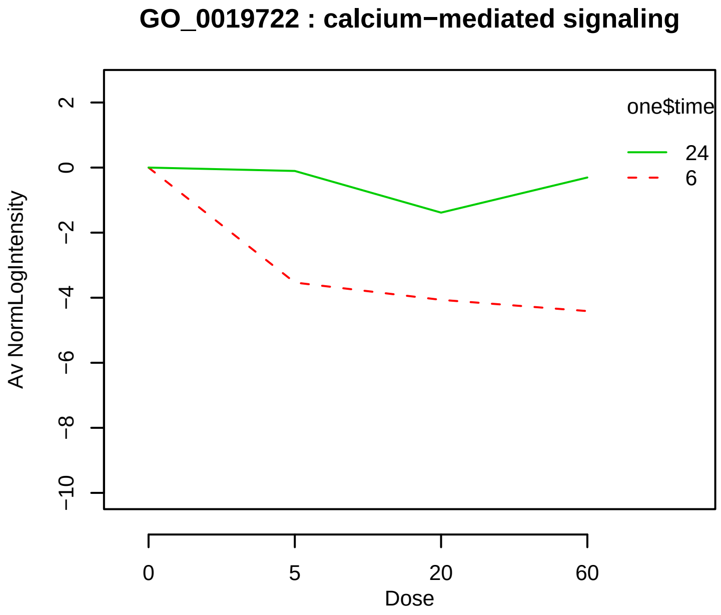
GO_0019722 : calcium−mediated signaling
one$time
24
6
Av NormLogIntensity
Dose

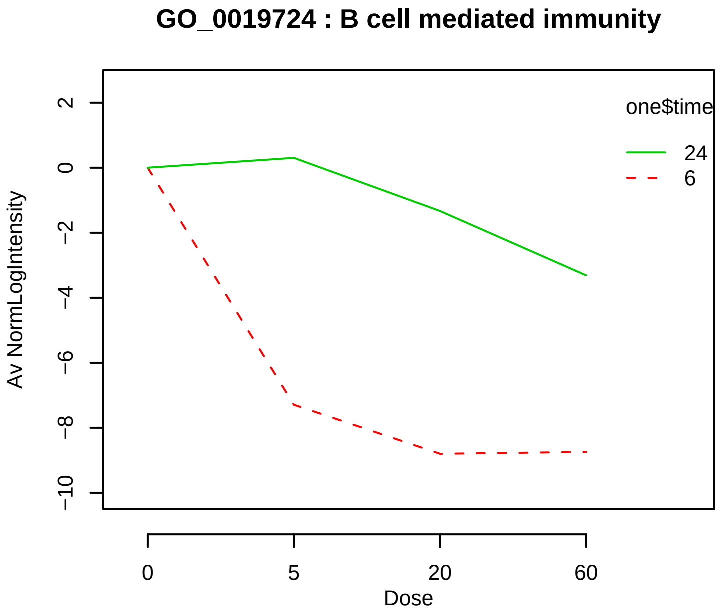
GO_0019724 : B cell mediated immunity
one$time
24
6
Av NormLogIntensity
Dose

GO_0019725 : cell homeostasis

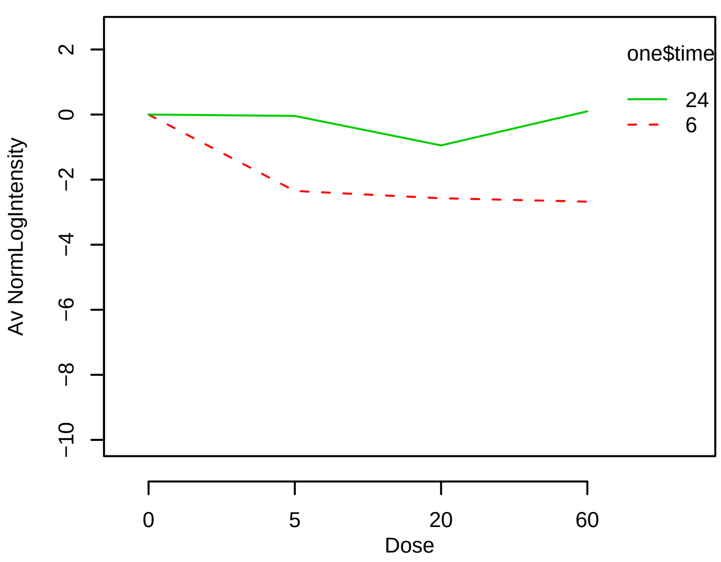

GO_0019730 : antimicrobial humoral response

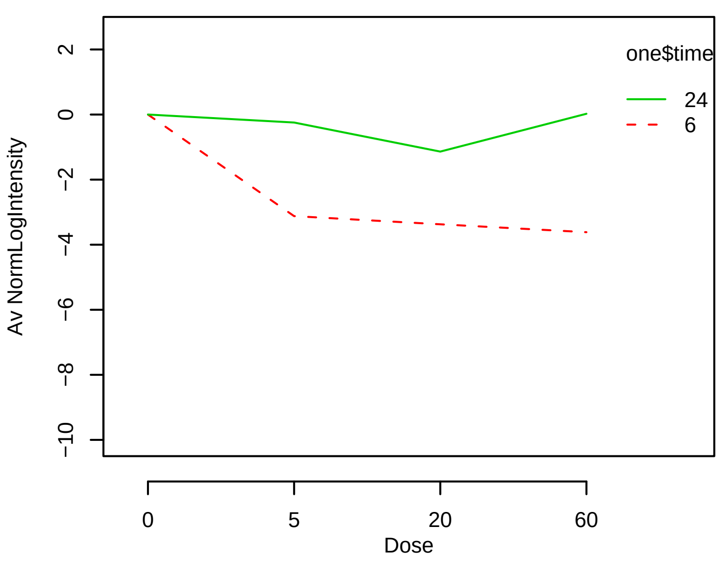

GO_0019731 : antibacterial humoral response

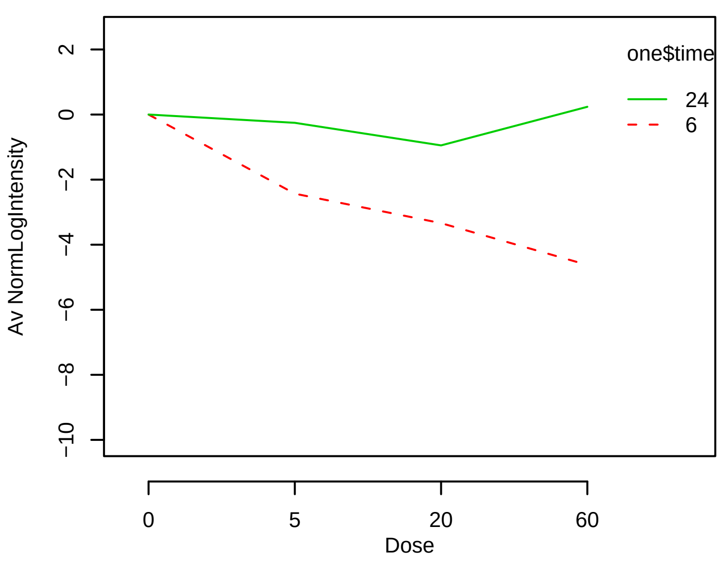

GO_0019735 : antimicrobial humoral response (sensu Verteb

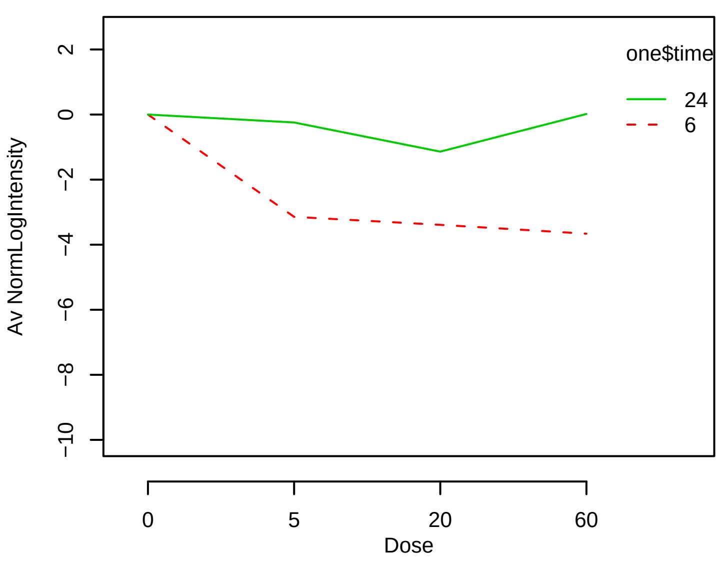

GO_0019748 : secondary metabolism

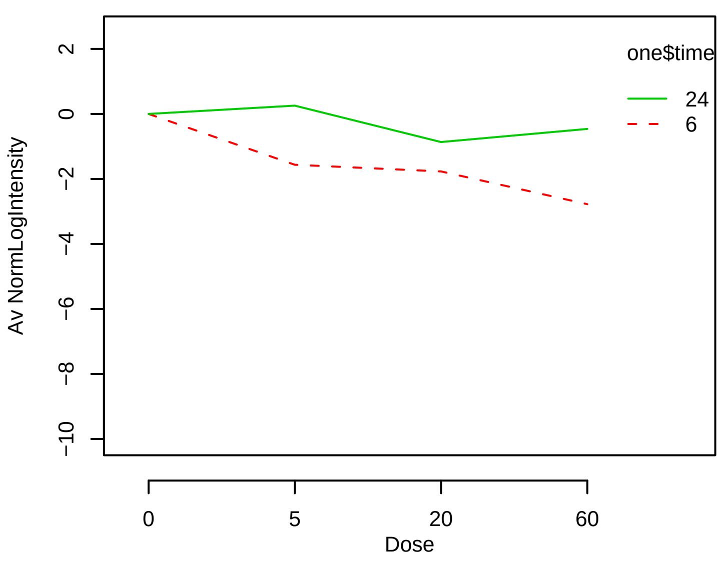

GO_0019751 : polyol metabolism

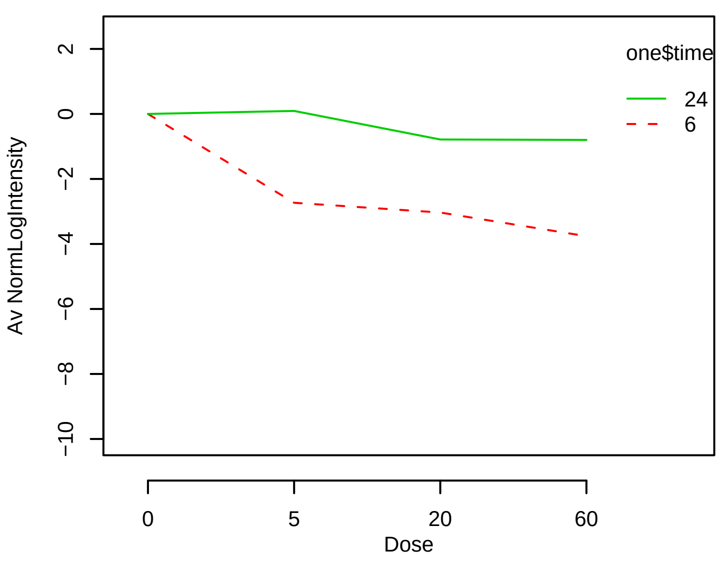

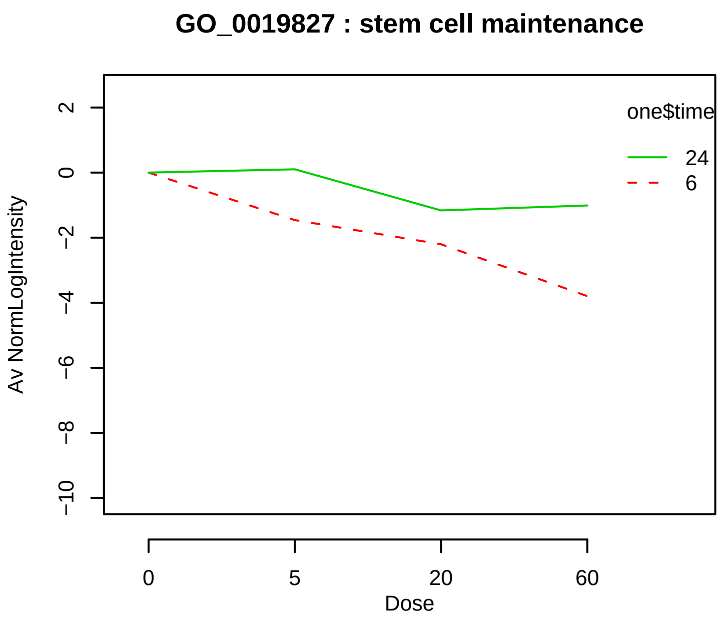
GO_0019827 : stem cell maintenance
one$time
24
6
Av NormLogIntensity
2
0
−2
−4
−6
−8
−10
0
5
20
60
Dose

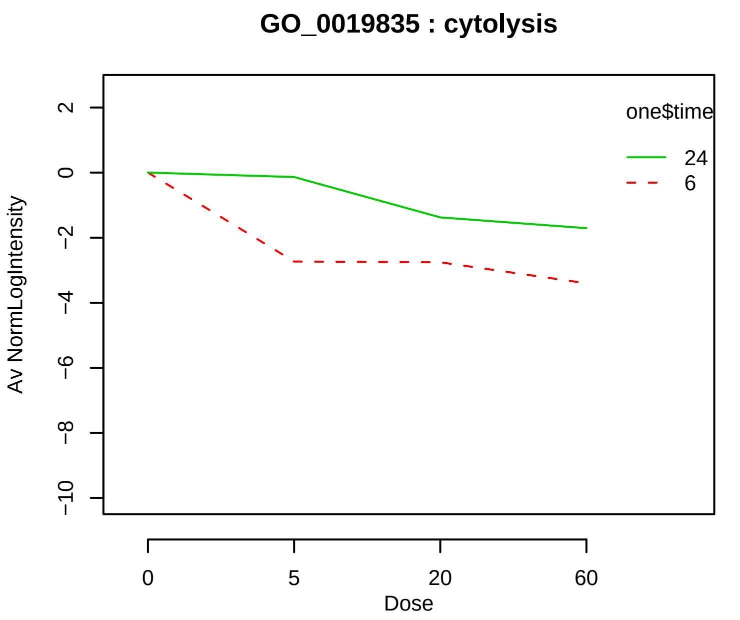
GO_0019835 : cytolysis
one$time
24
6
Av NormLogIntensity
2
0
−2
−4
−6
−8
−10
0
5
20
60
Dose

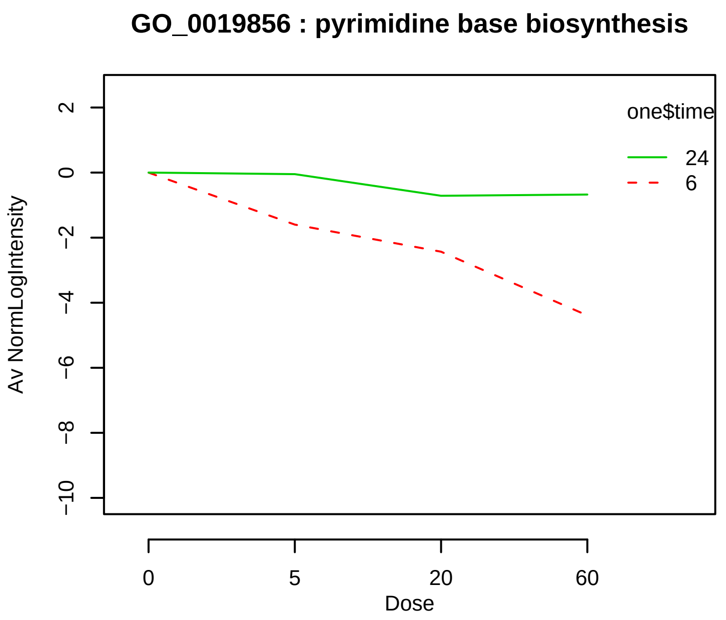
GO_0019856 : pyrimidine base biosynthesis
one$time
24
6
Av NormLogIntensity
2
0
−2
−4
−6
−8
−10
0
5
20
60
Dose

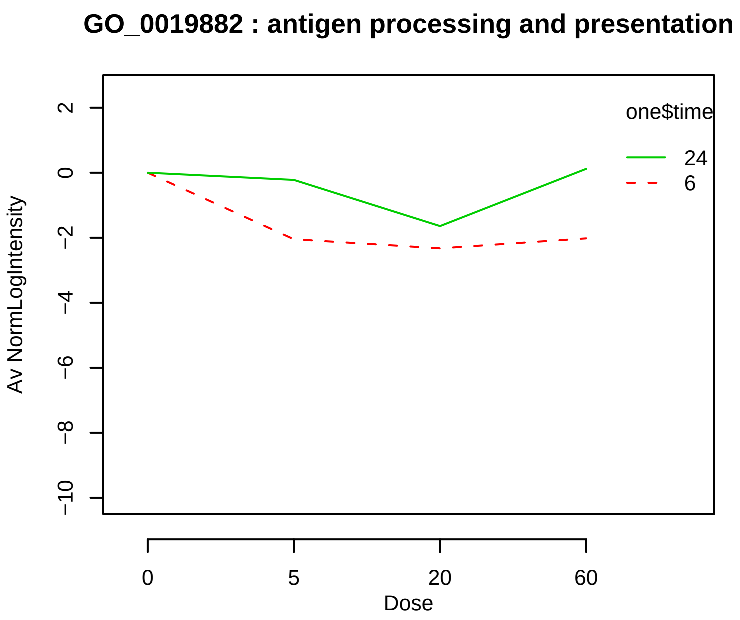
GO_0019882 : antigen processing and presentation
one$time
24
6
Av NormLogIntensity
2
0
−2
−4
−6
−8
−10
0
5
20
60
Dose

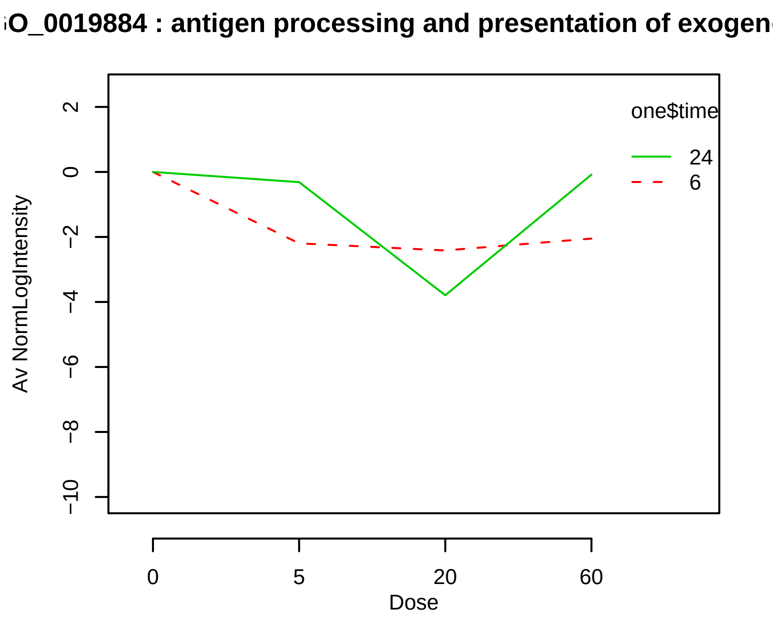
O_0019884 : antigen processing and presentation of exogen
one$time
24
6
Av NormLogIntensity
2
0
−2
−4
−6
−8
−10
0
5
20
60
Dose

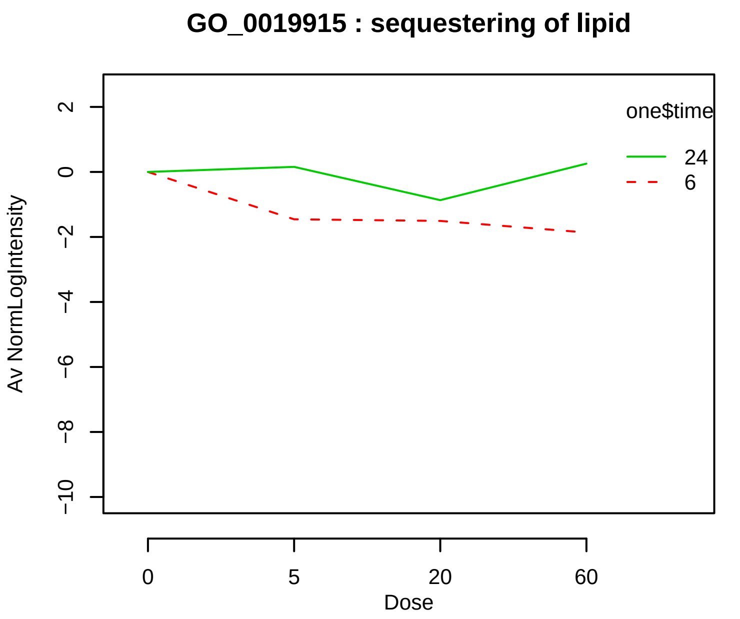
GO_0019915 : sequestering of lipid
one$time
24
6
Av NormLogIntensity
2
0
−2
−4
−6
−8
−10
0
5
20
60
Dose

GO_0019932 : second−messenger−mediated signaling

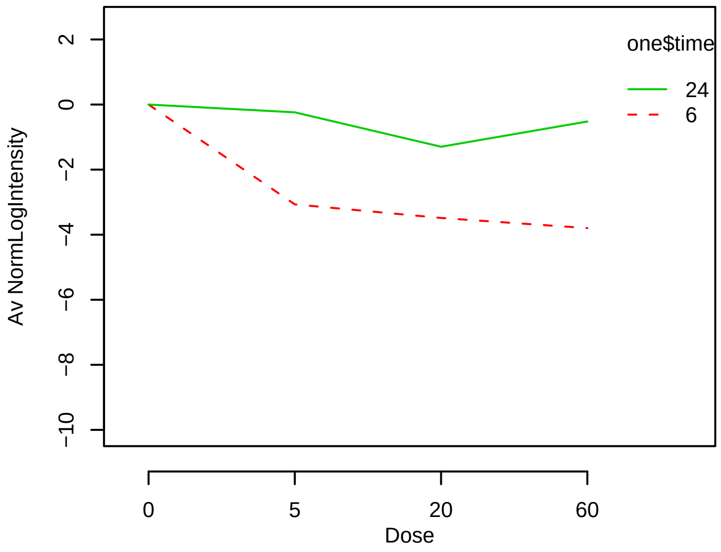

GO_0019933 : cAMP−mediated signaling

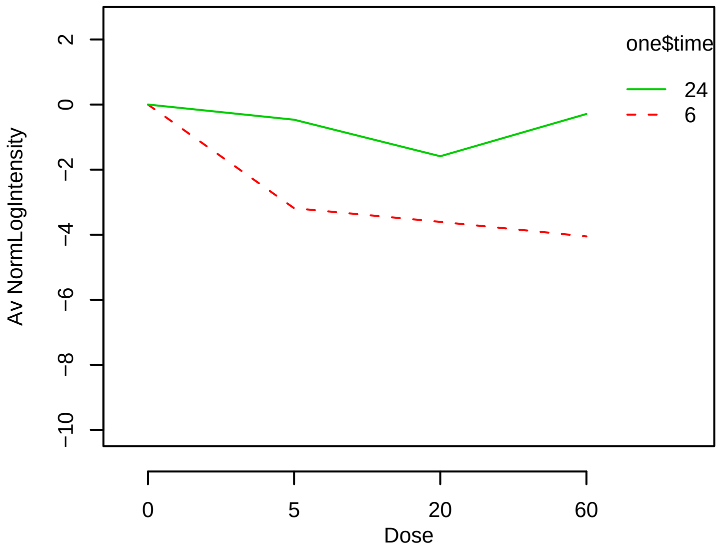

GO_0019935 : cyclic−nucleotide−mediated signaling

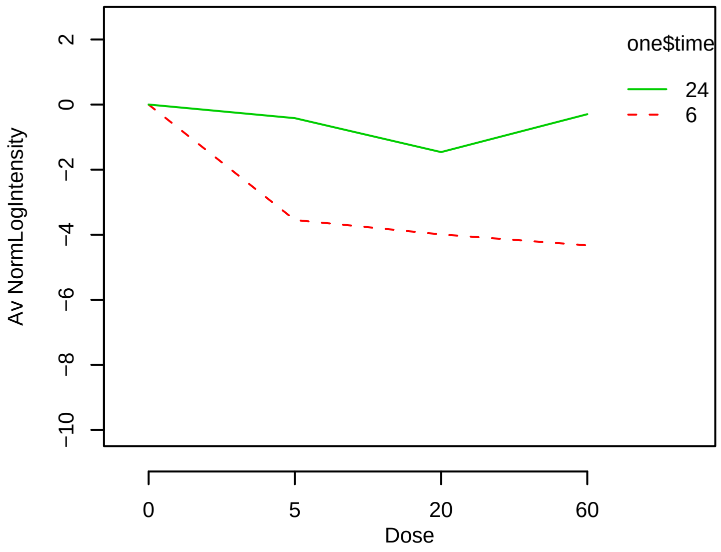

GO_0019941 : modification−dependent protein catabolism

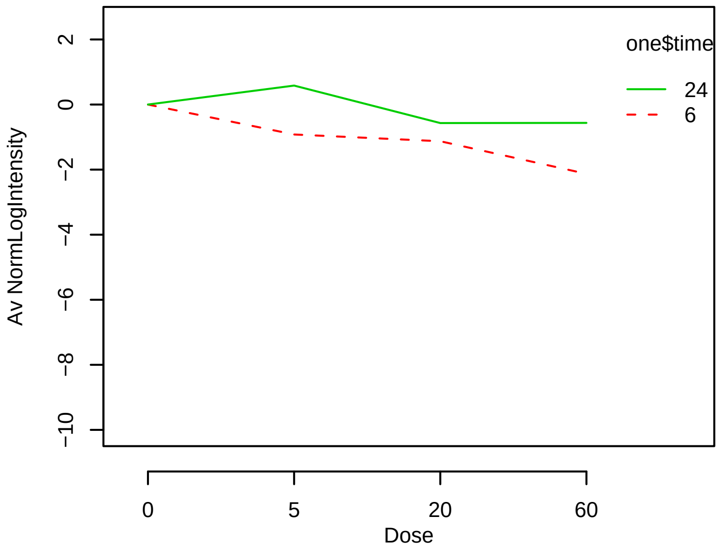

GO_0020027 : hemoglobin metabolism

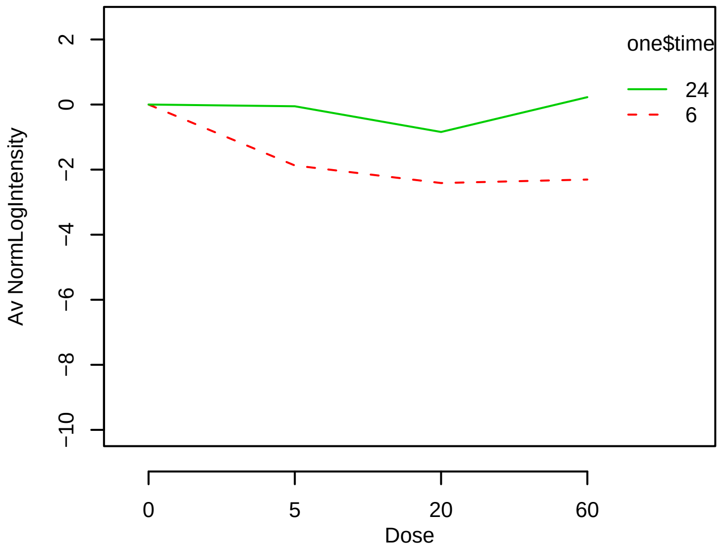

GO_0021782 : glial cell development

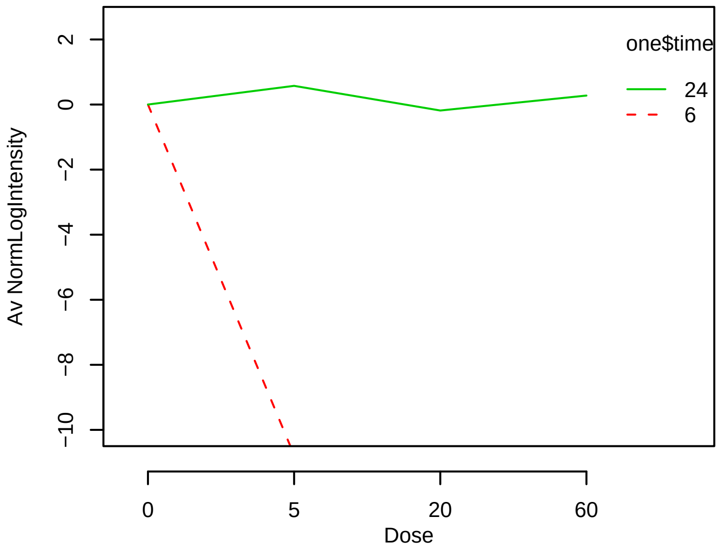

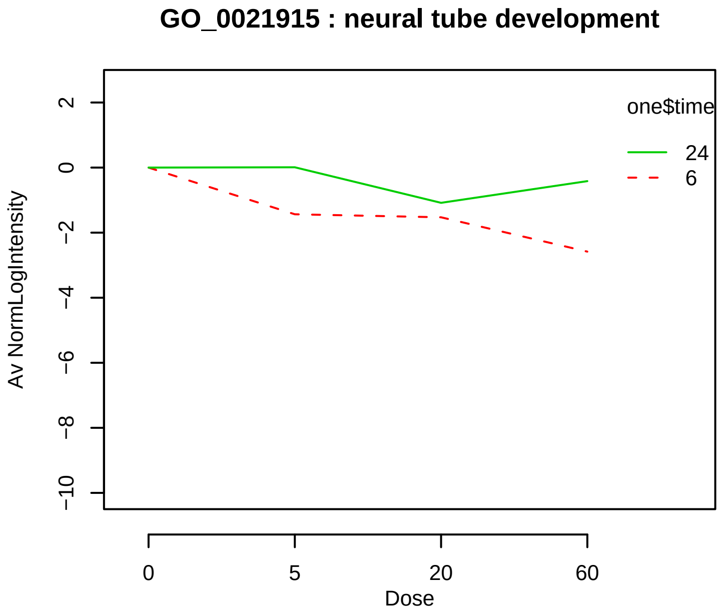
GO_0021915 : neural tube development
Av NormLogIntensity
2
0
−2
−4
−6
−8
−10
0
5
20
60
Dose
one$time
24
6

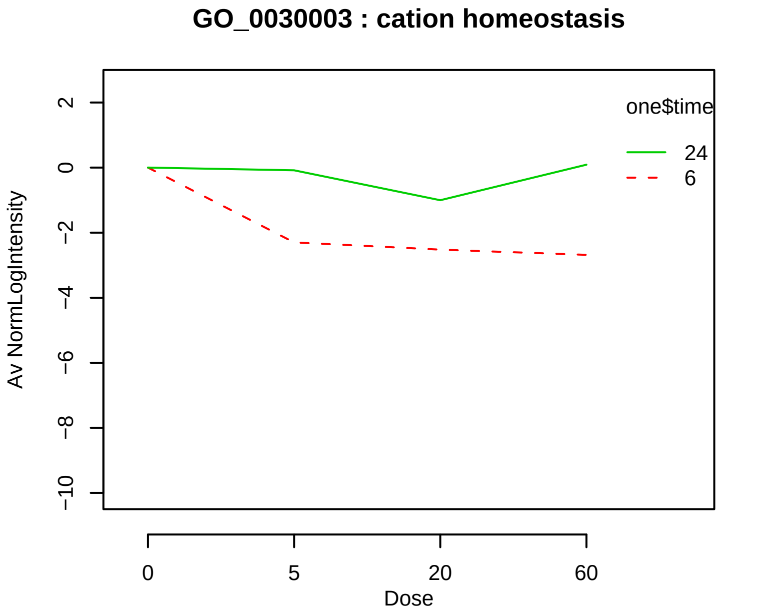
GO_0030003 : cation homeostasis
Av NormLogIntensity
2
0
−2
−4
−6
−8
−10
0
5
20
60
Dose
one$time
24
6

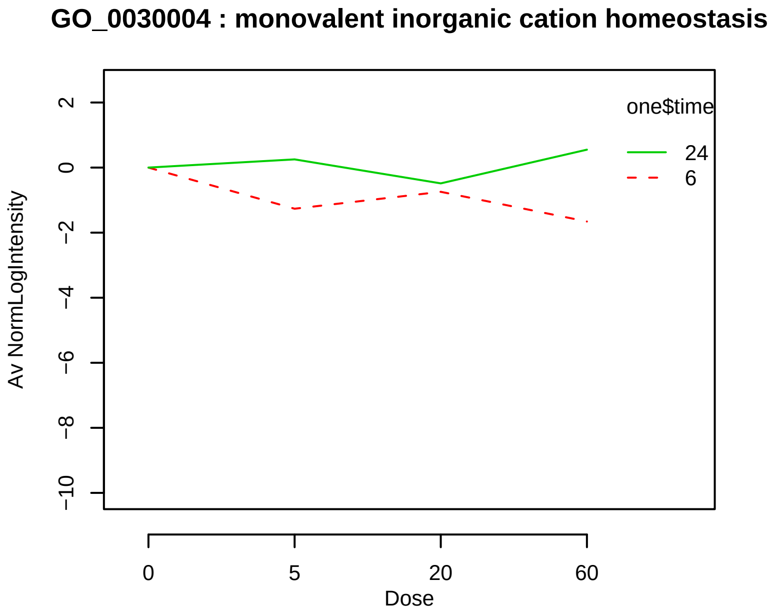
GO_0030004 : monovalent inorganic cation homeostasis
Av NormLogIntensity
2
0
−2
−4
−6
−8
−10
0
5
20
60
Dose
one$time
24
6

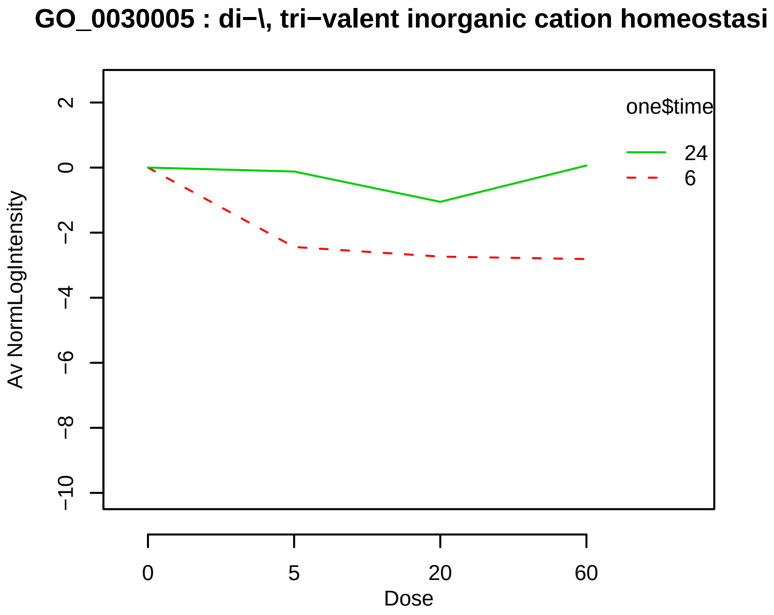
GO_0030005 : di−\, tri−valent inorganic cation homeostasi
Av NormLogIntensity
2
0
−2
−4
−6
−8
−10
0
5
20
60
Dose
one$time
24
6

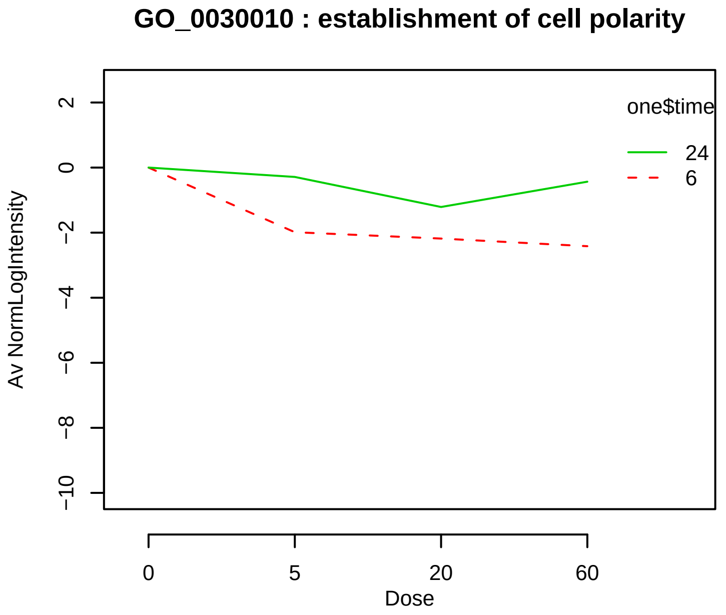
GO_0030010 : establishment of cell polarity
Av NormLogIntensity
2
0
−2
−4
−6
−8
−10
0
5
20
60
Dose
one$time
24
6

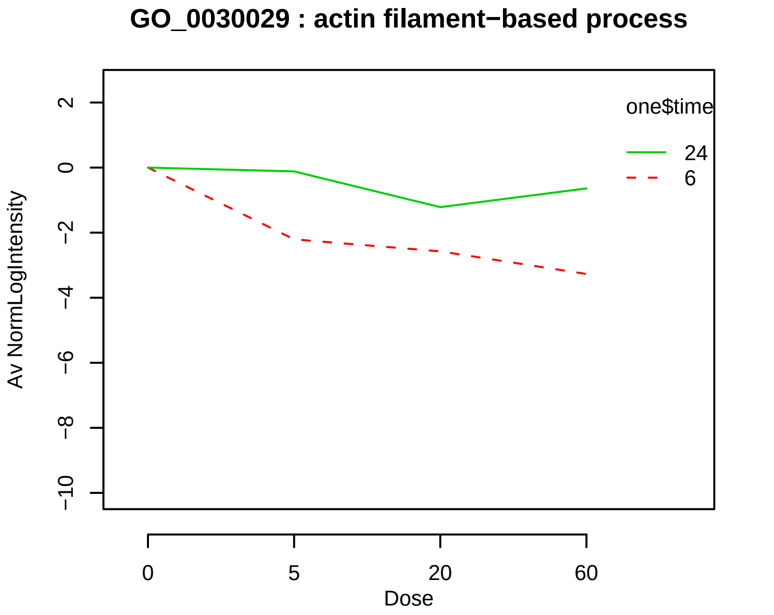
GO_0030029 : actin filament−based process
Av NormLogIntensity
2
0
−2
−4
−6
−8
−10
0
5
20
60
Dose
one$time
24
6

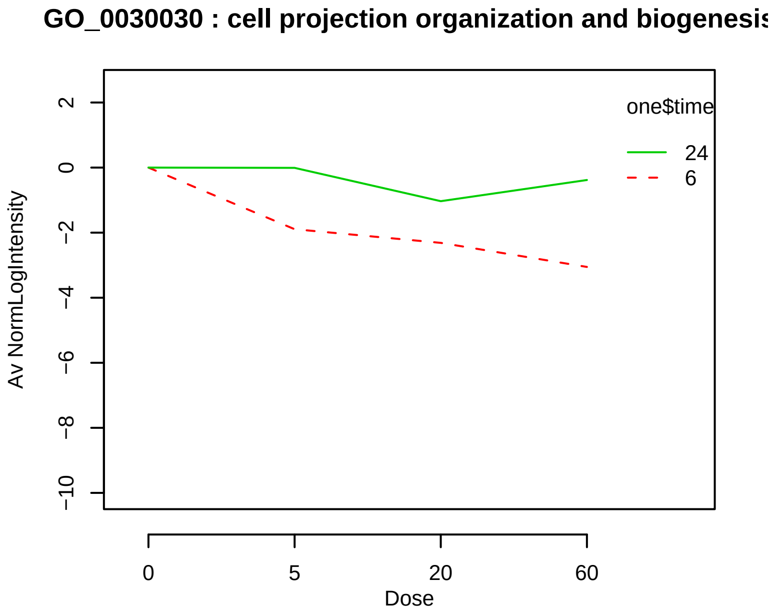
GO_0030030 : cell projection organization and biogenesis
one$time
24
6
Av NormLogIntensity
Dose

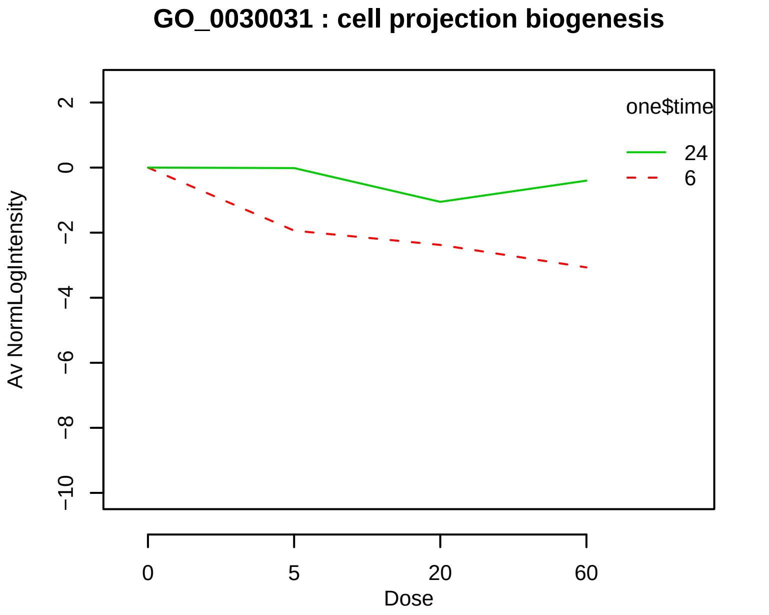
GO_0030031 : cell projection biogenesis
one$time
24
6
Av NormLogIntensity
Dose

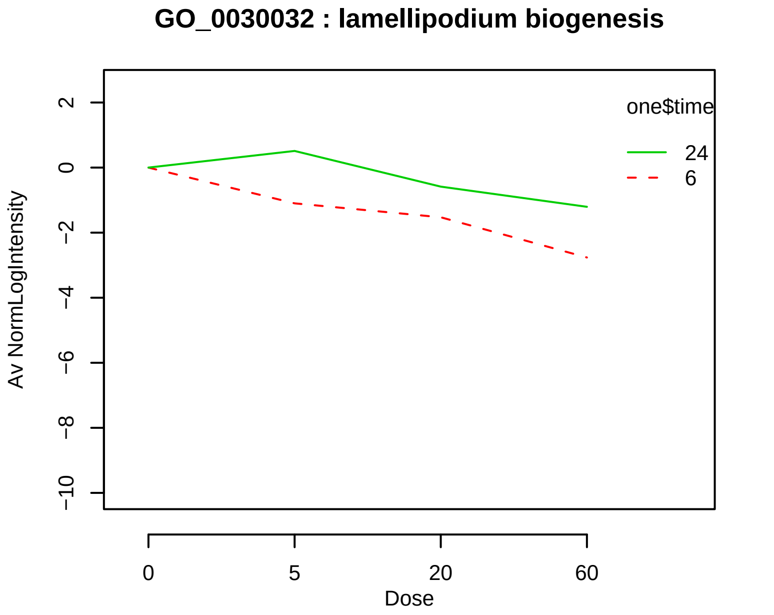
GO_0030032 : lamellipodium biogenesis
one$time
24
6
Av NormLogIntensity
Dose

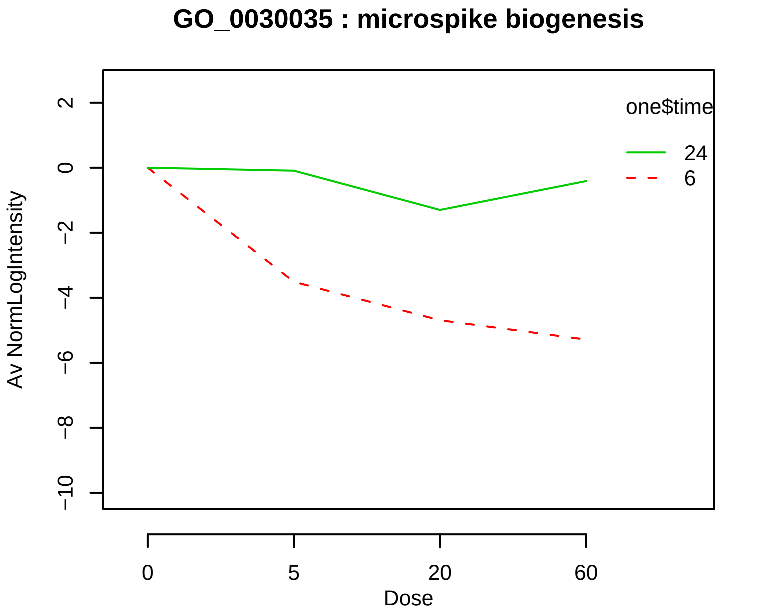
GO_0030035 : microspike biogenesis
one$time
24
6
Av NormLogIntensity
Dose

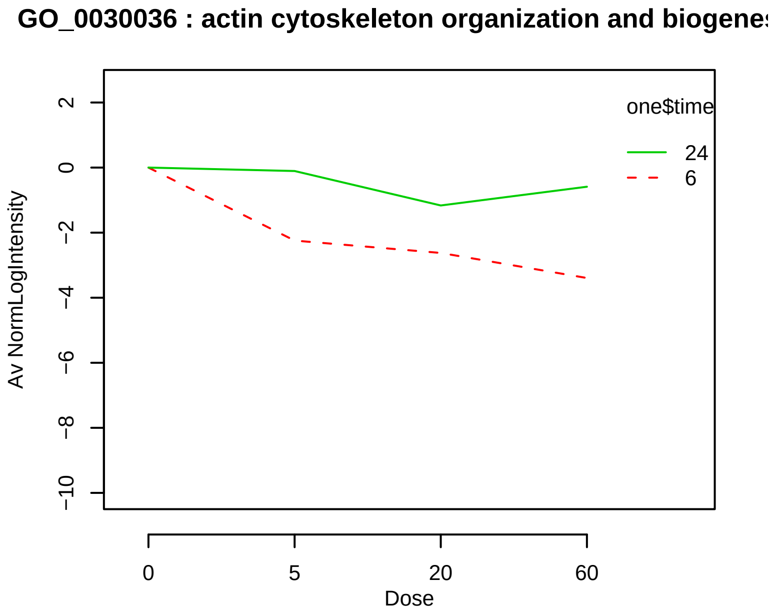
GO_0030036 : actin cytoskeleton organization and biogenes
one$time
24
6
Av NormLogIntensity
Dose

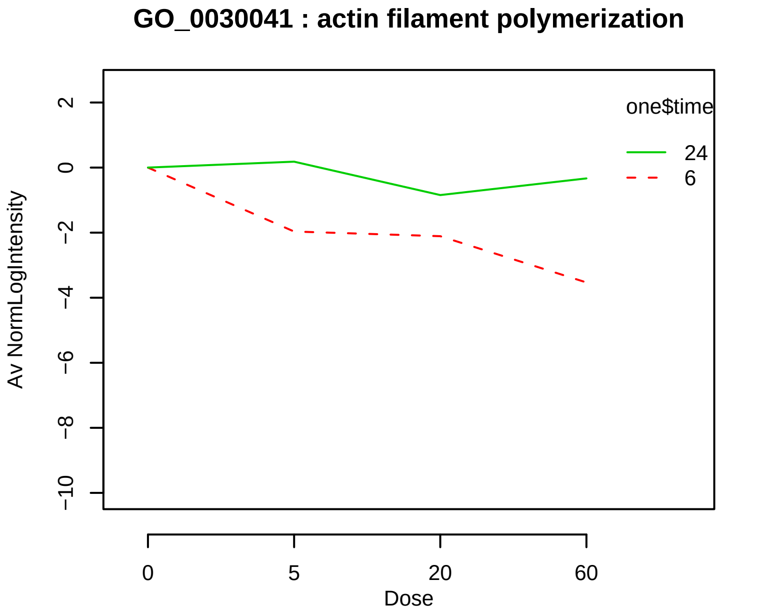
GO_0030041 : actin filament polymerization
one$time
24
6
Av NormLogIntensity
Dose

GO_0030042 : actin filament depolymerization

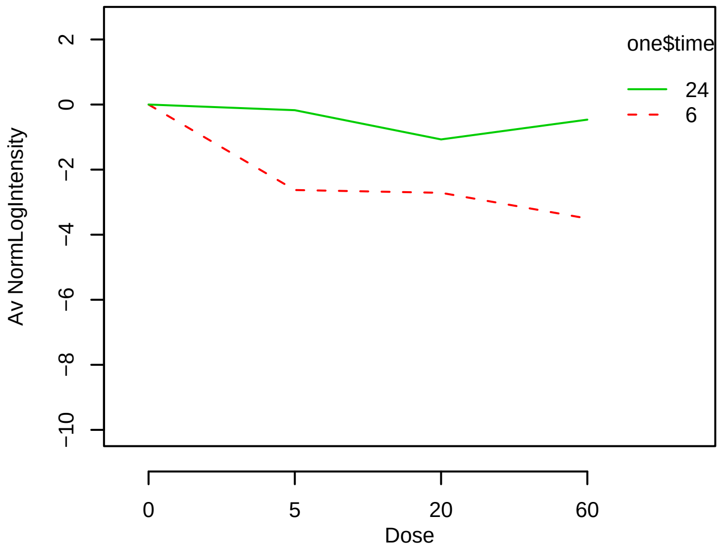

GO_0030048 : actin filament−based movement

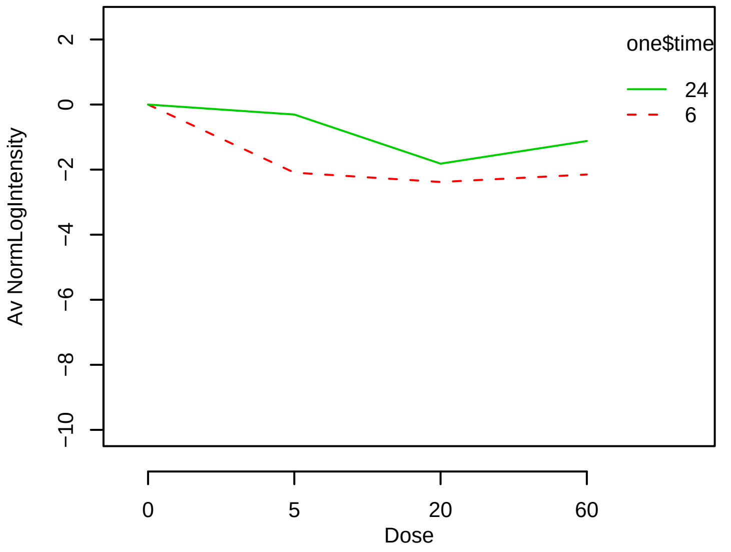

GO_0030071 : regulation of mitotic metaphase/anaphase tran

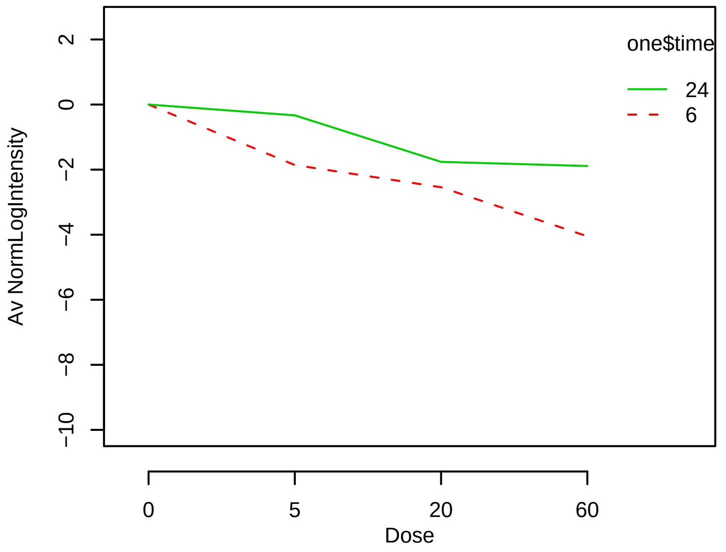

GO_0030072 : peptide hormone secretion

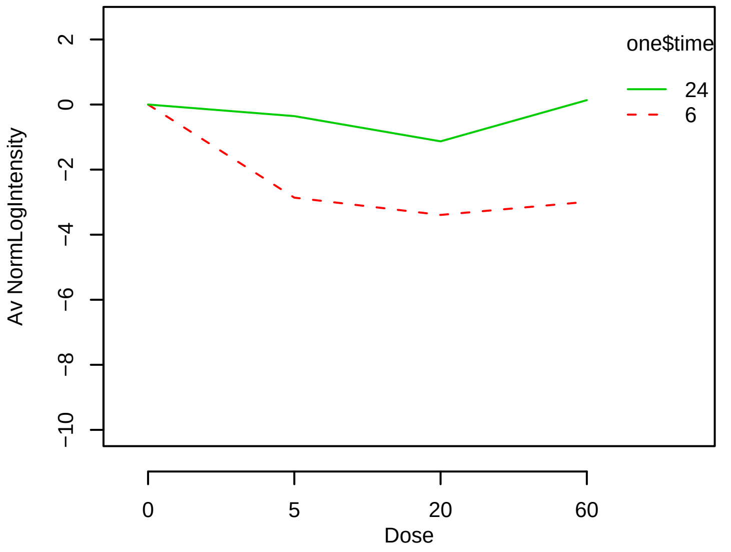

GO_0030073 : insulin secretion

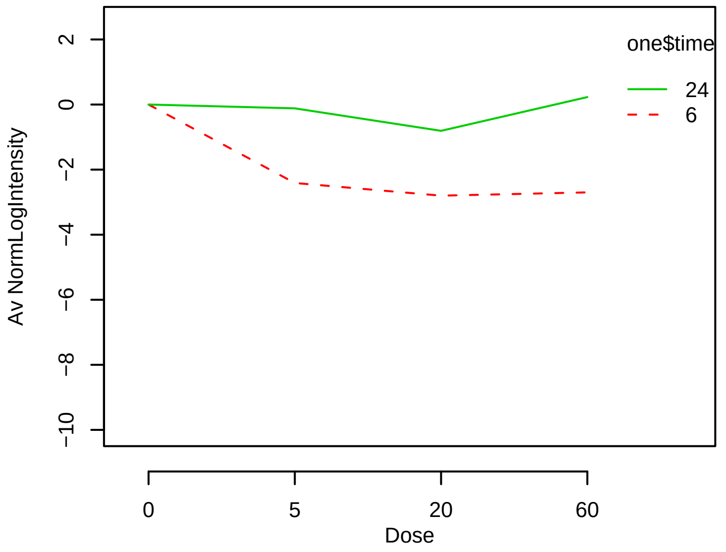

GO_0030097 : hemopoiesis

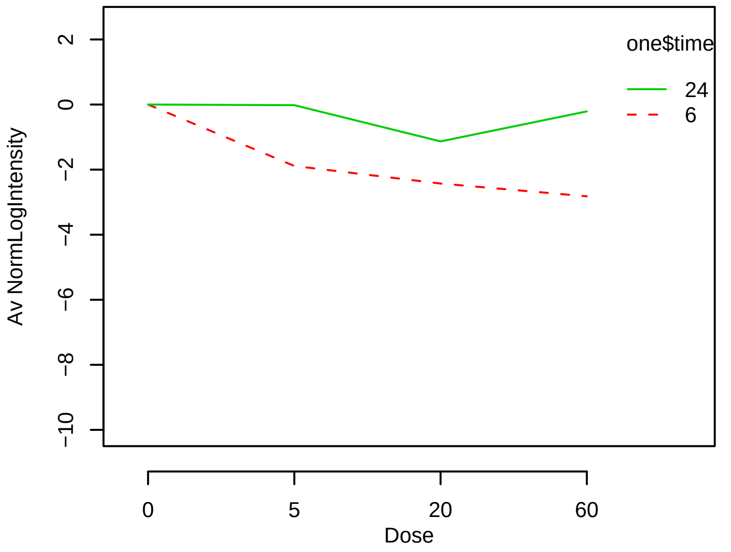

GO_0030098 : lymphocyte differentiation

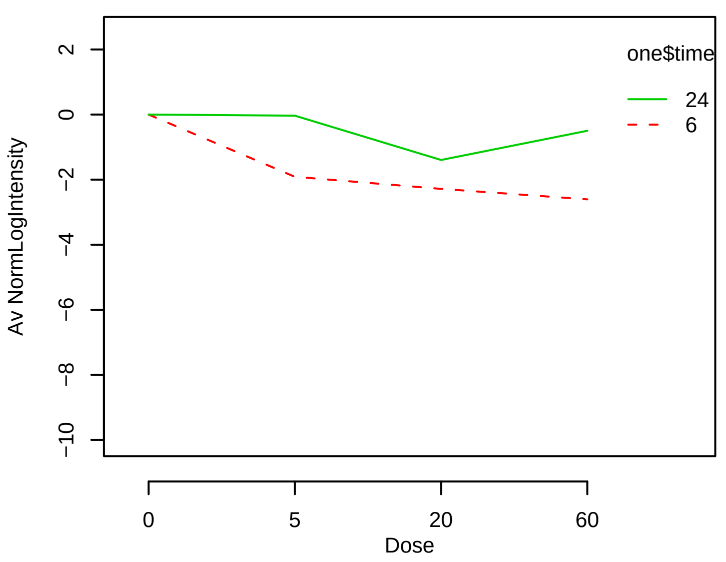

GO_0030099 : myeloid cell differentiation

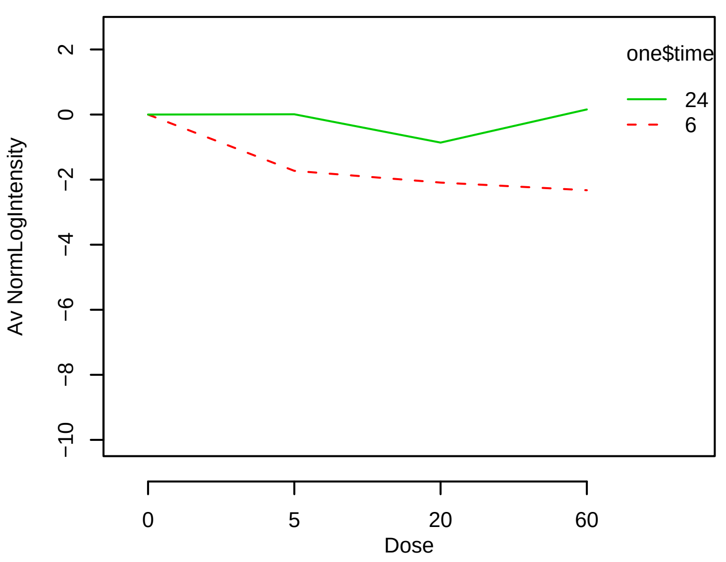

GO_0030100 : regulation of endocytosis

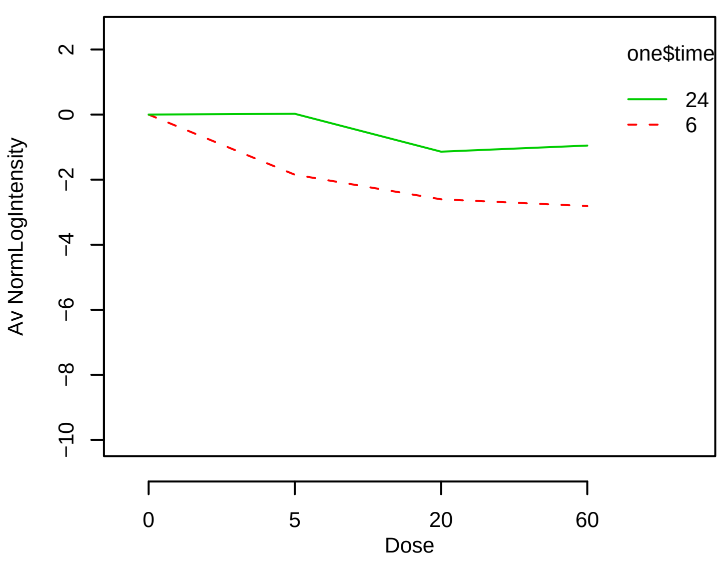

GO_0030101 : natural killer cell activation

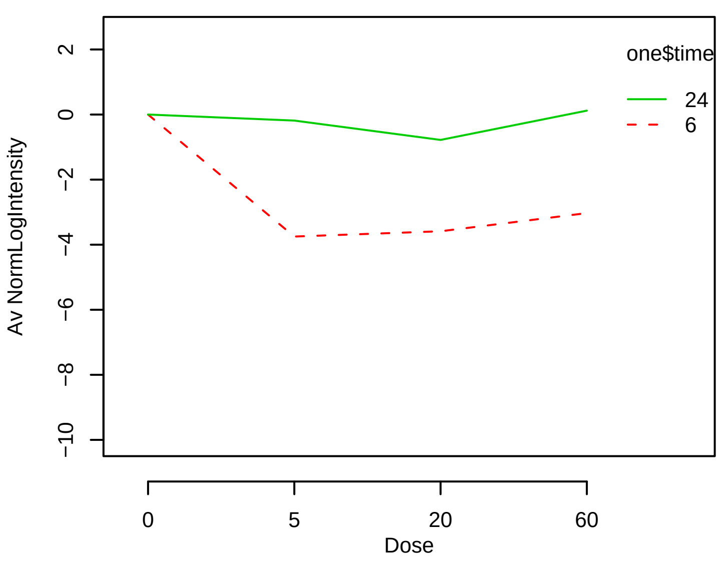

GO_0030111 : regulation of Wnt receptor signaling pathwa

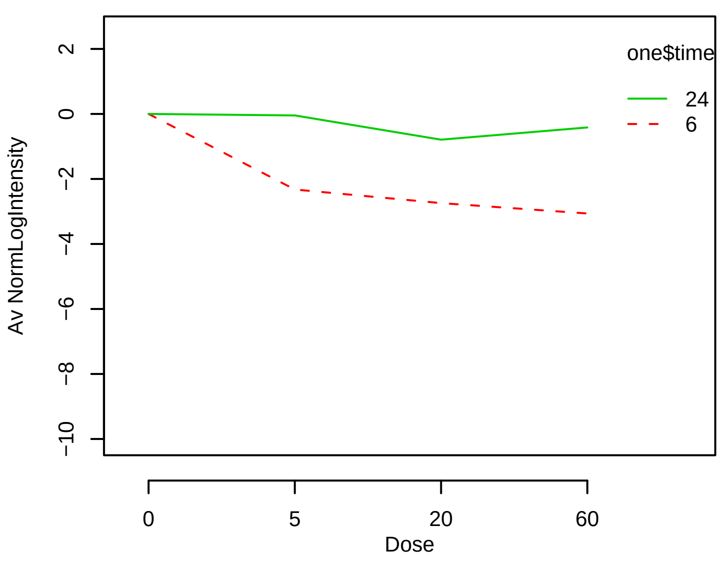

GO_0030146 : diuresis

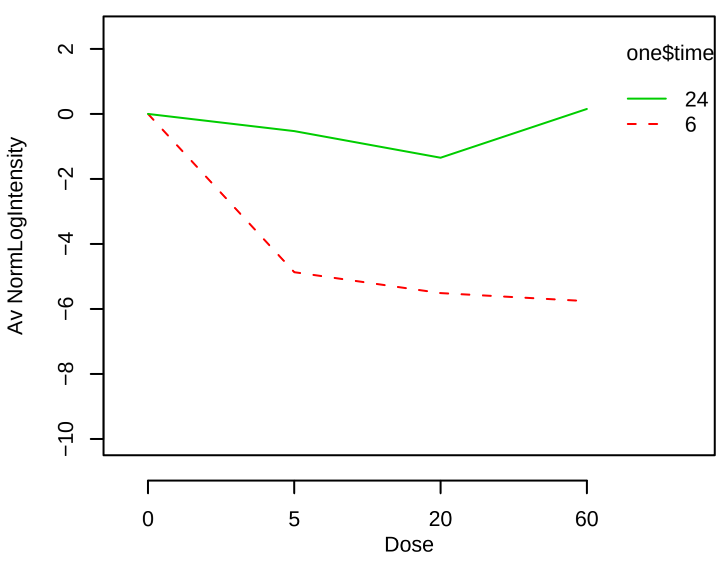

GO_0030148 : sphingolipid biosynthesis

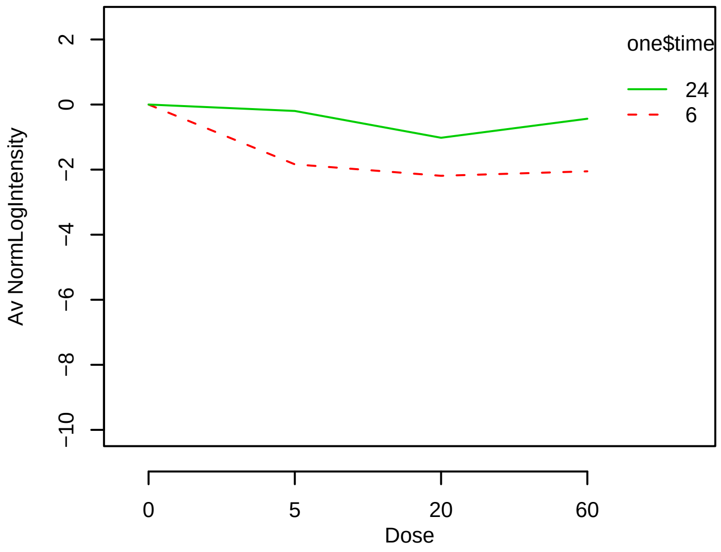

GO_0030149 : sphingolipid catabolism

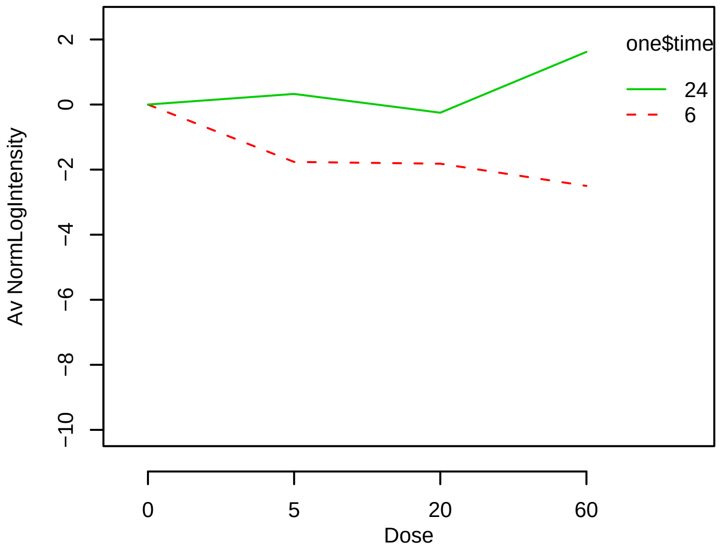

GO_0030155 : regulation of cell adhesion

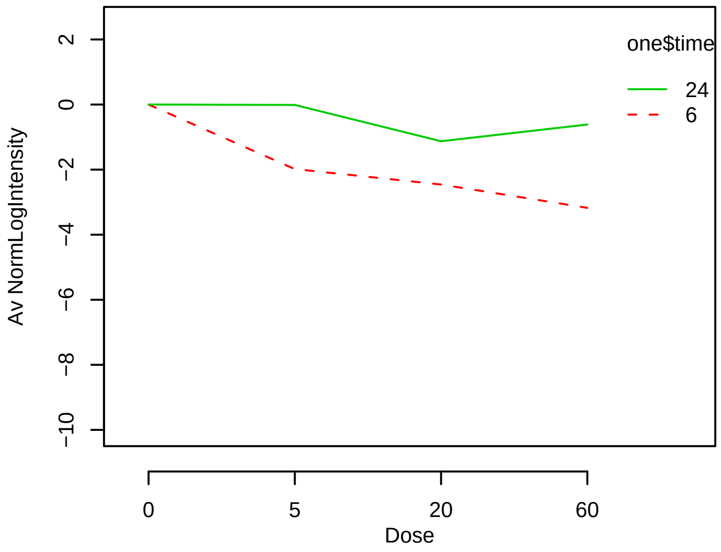

GO_0030162 : regulation of proteolysis

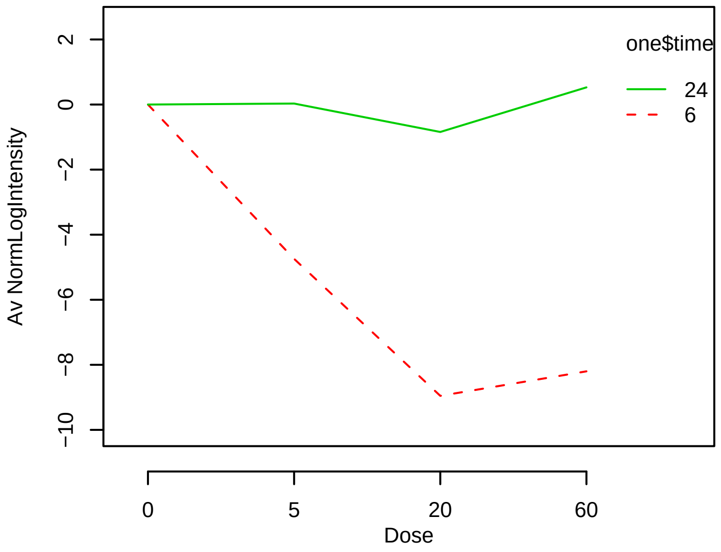

GO_0030163 : protein catabolism

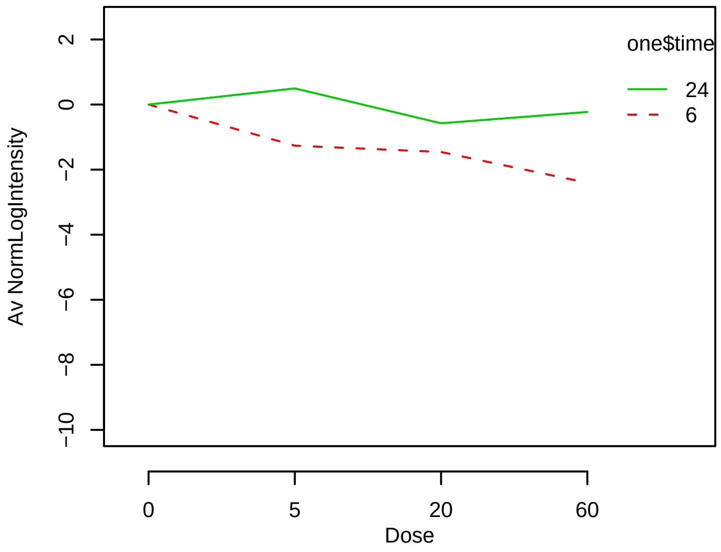

GO_0030166 : proteoglycan biosynthesis

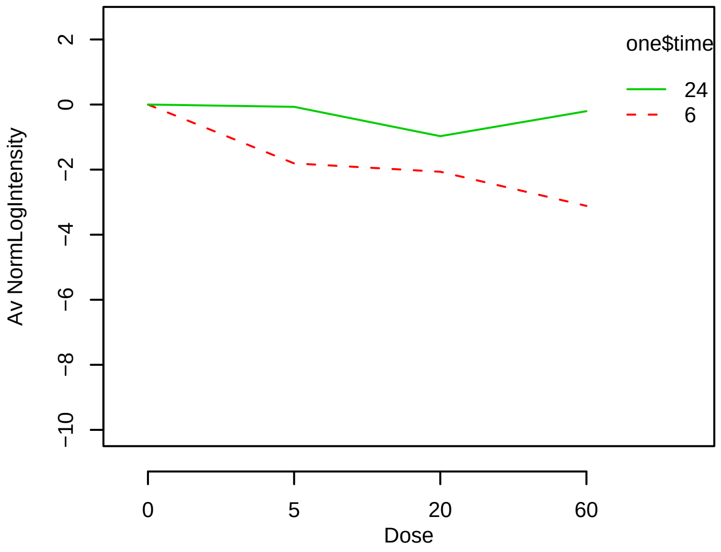

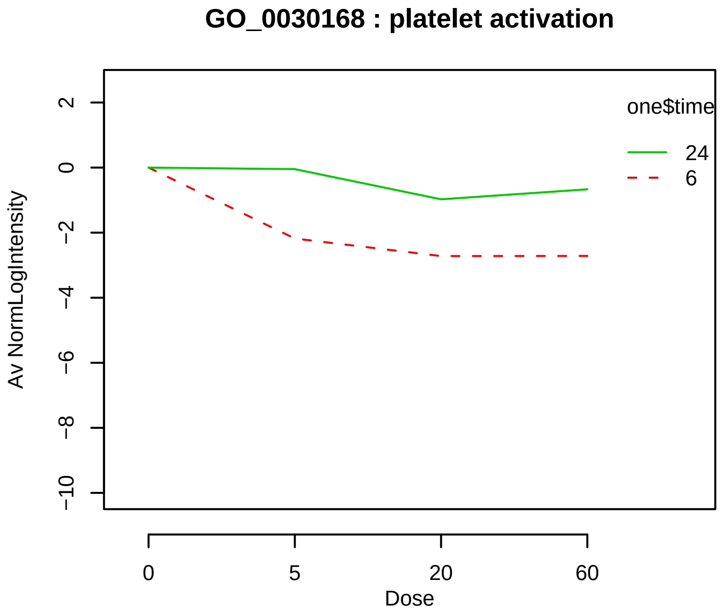
GO_0030168 : platelet activation
one$time
24
6
Av NormLogIntensity
Dose
0
5
20
60
2
0
−2
−4
−6
−8
−10

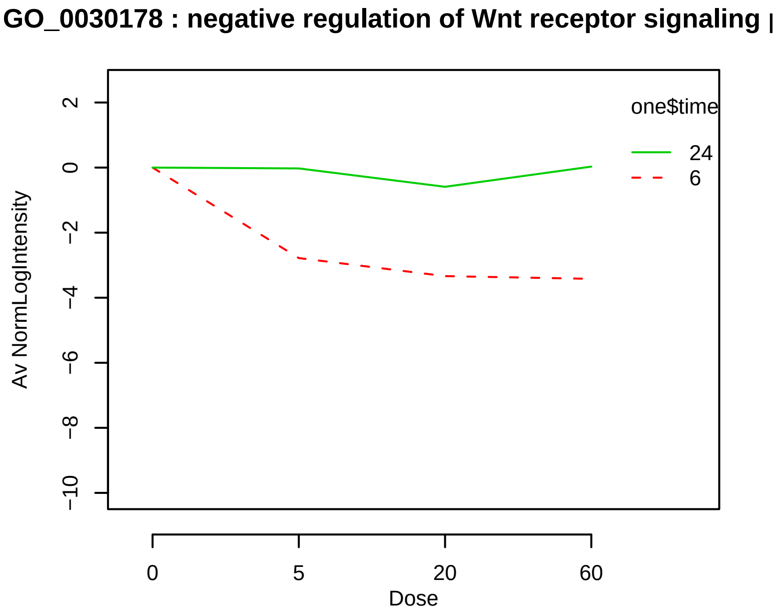
GO_0030178 : negative regulation of Wnt receptor signaling
one$time
24
6
Av NormLogIntensity
Dose
0
5
20
60
2
0
−2
−4
−6
−8
−10

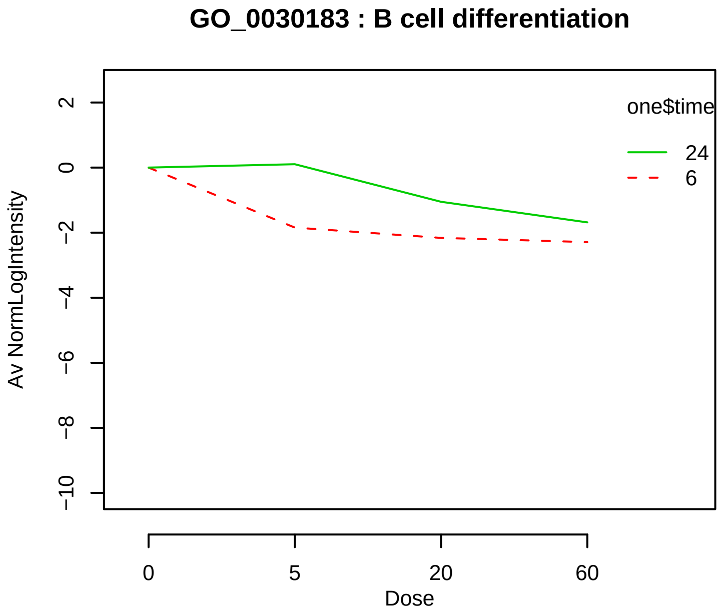
GO_0030183 : B cell differentiation
one$time
24
6
Av NormLogIntensity
Dose
0
5
20
60
2
0
−2
−4
−6
−8
−10

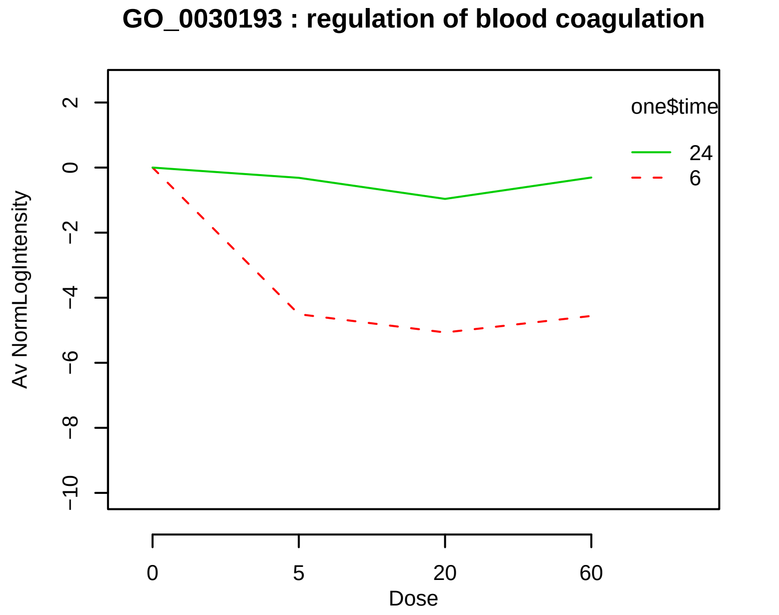
GO_0030193 : regulation of blood coagulation
one$time
24
6
Av NormLogIntensity
Dose
0
5
20
60
2
0
−2
−4
−6
−8
−10

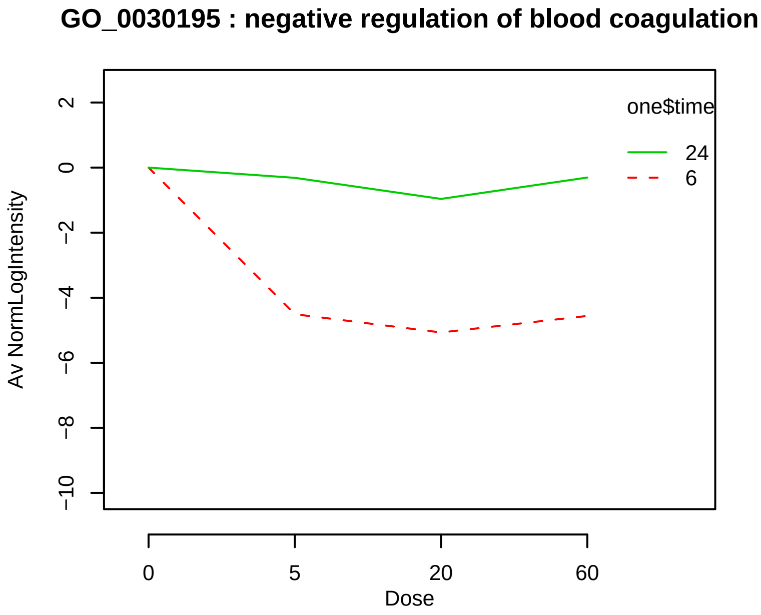
GO_0030195 : negative regulation of blood coagulation
one$time
24
6
Av NormLogIntensity
Dose
0
5
20
60
2
0
−2
−4
−6
−8
−10

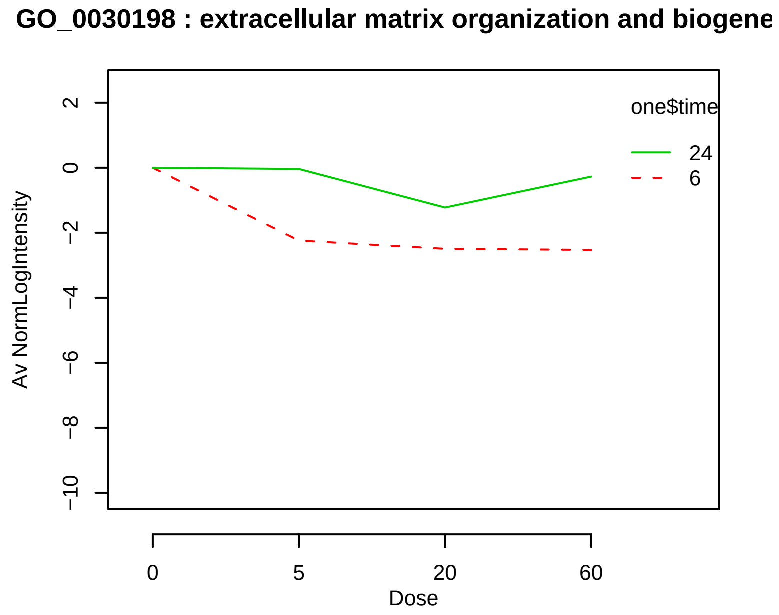
GO_0030198 : extracellular matrix organization and biogene
one$time
24
6
Av NormLogIntensity
Dose
0
5
20
60
2
0
−2
−4
−6
−8
−10

GO_0030199 : collagen fibril organization

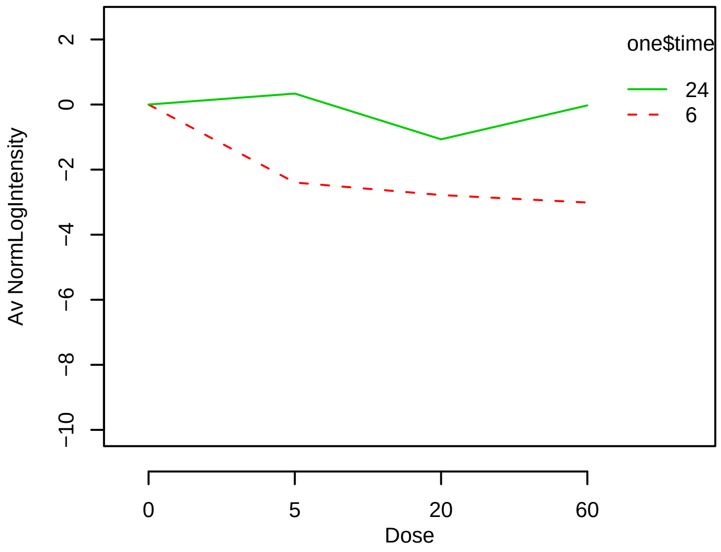

GO_0030201 : heparan sulfate proteoglycan metabolism

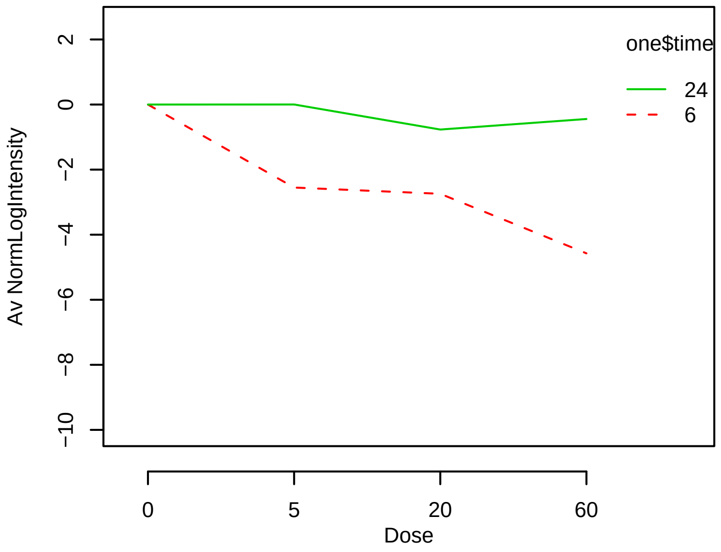

GO_0030203 : glycosaminoglycan metabolism

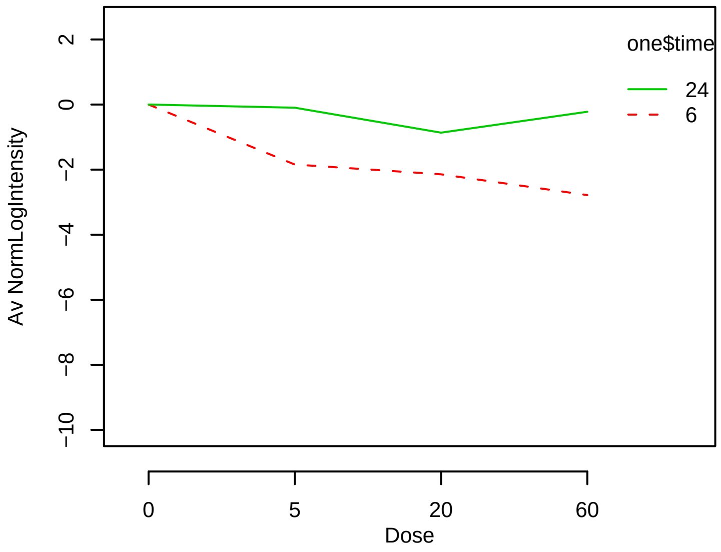

GO_0030204 : chondroitin sulfate metabolism

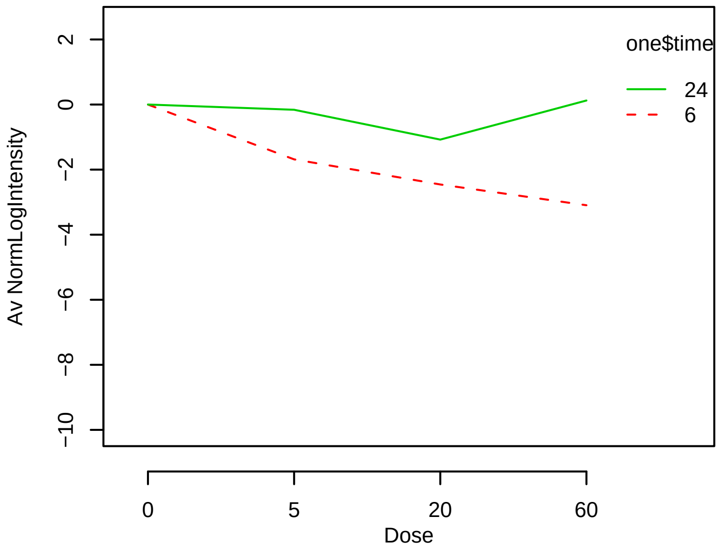

GO_0030206 : chondroitin sulfate biosynthesis

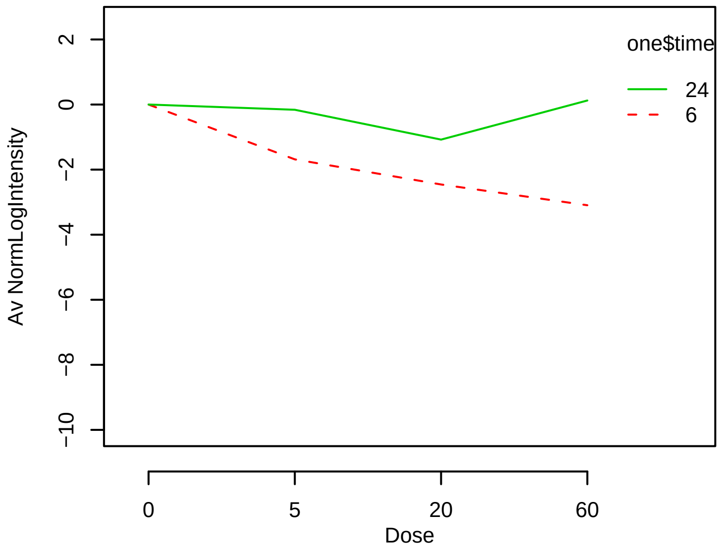

GO_0030212 : hyaluronan metabolism

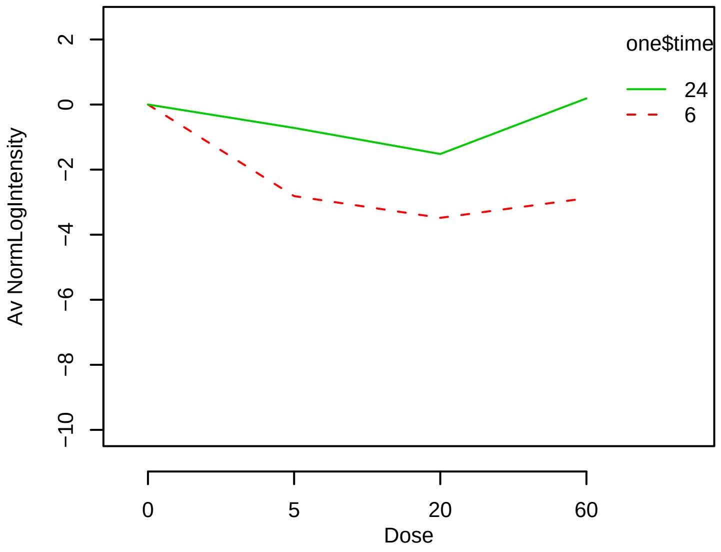

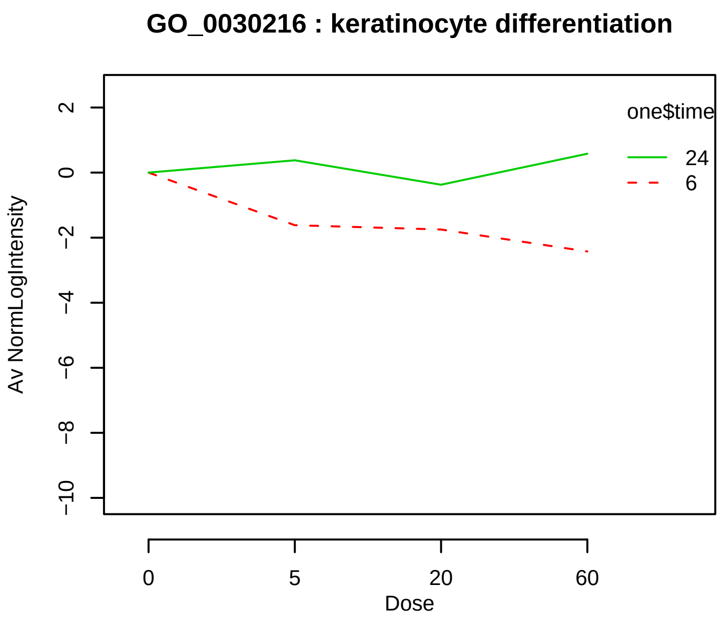
GO_0030216 : keratinocyte differentiation
one$time
24
6
Av NormLogIntensity
Dose

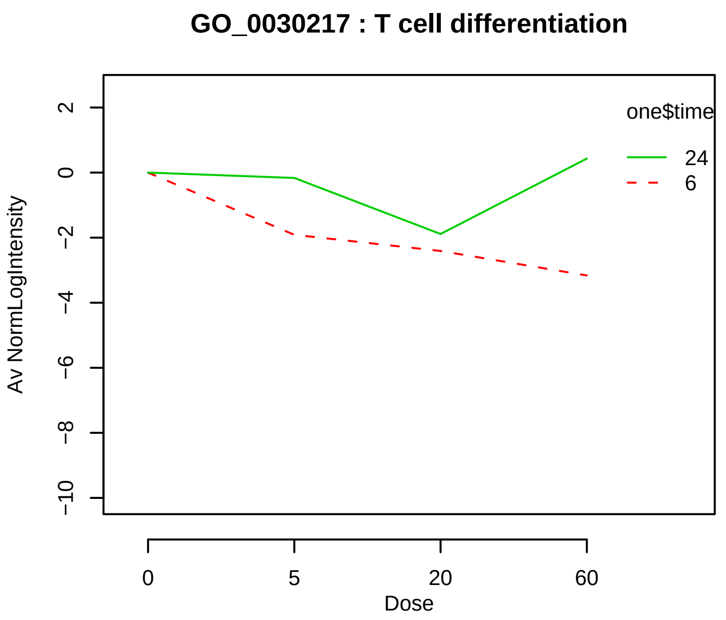
GO_0030217 : T cell differentiation
one$time
24
6
Av NormLogIntensity
Dose

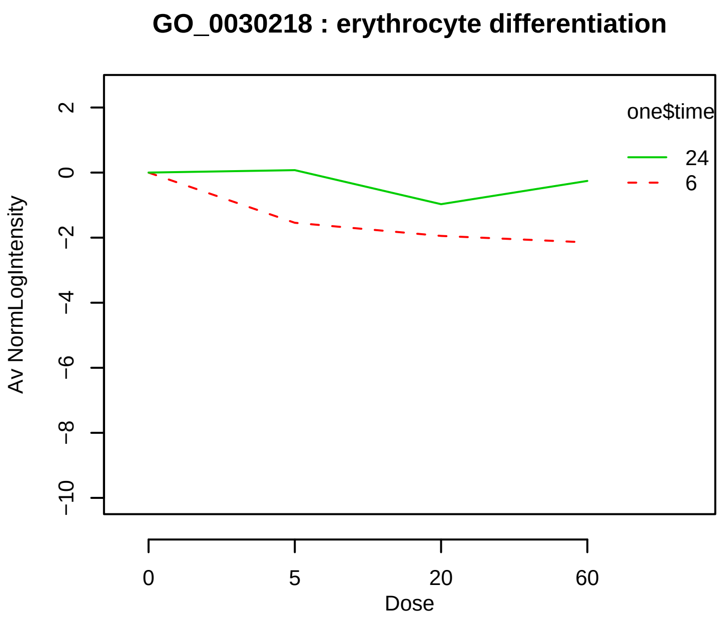
GO_0030218 : erythrocyte differentiation
one$time
24
6
Av NormLogIntensity
Dose

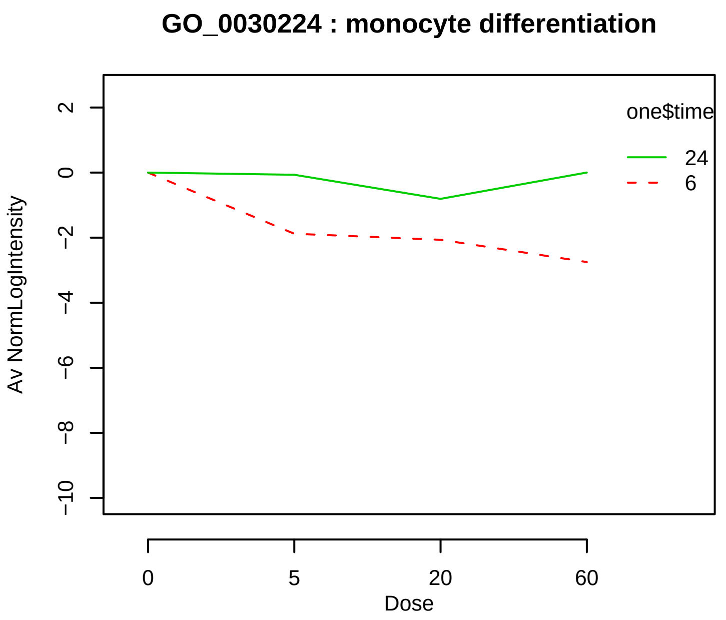
GO_0030224 : monocyte differentiation
one$time
24
6
Av NormLogIntensity
Dose

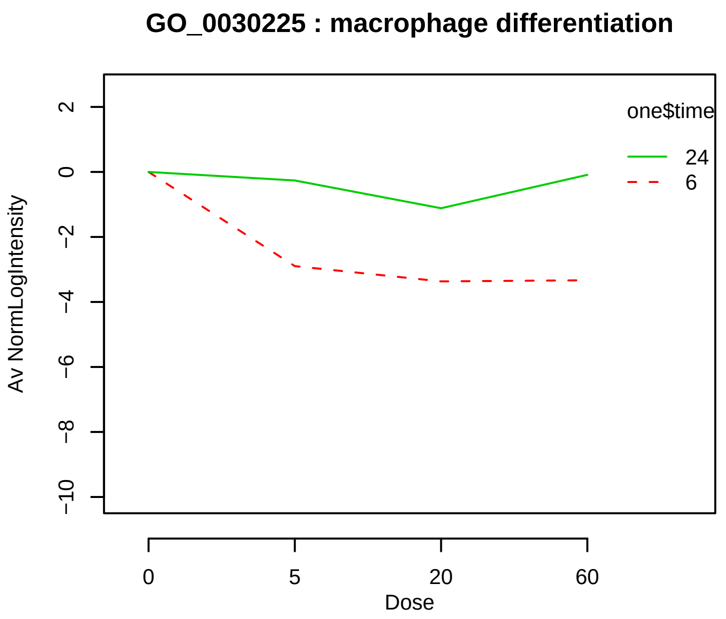
GO_0030225 : macrophage differentiation
one$time
24
6
Av NormLogIntensity
Dose

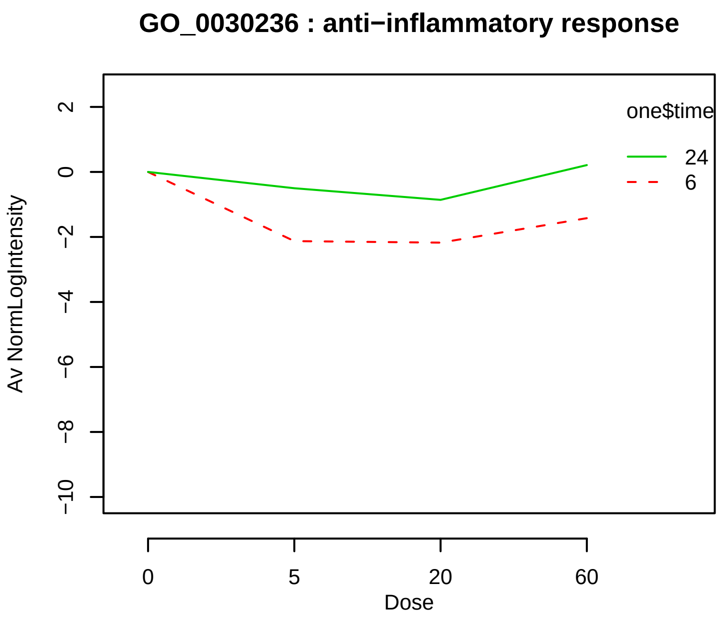
GO_0030236 : anti−inflammatory response
one$time
24
6
Av NormLogIntensity
Dose

GO_0030238 : male sex determination

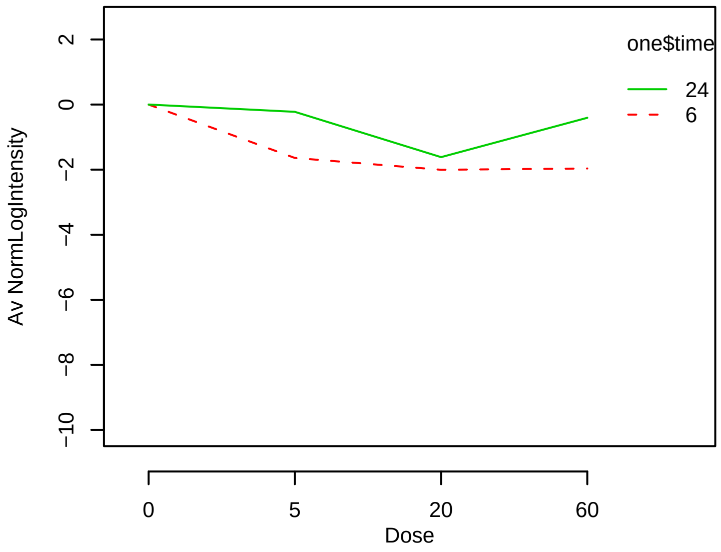

GO_0030239 : myofibril assembly

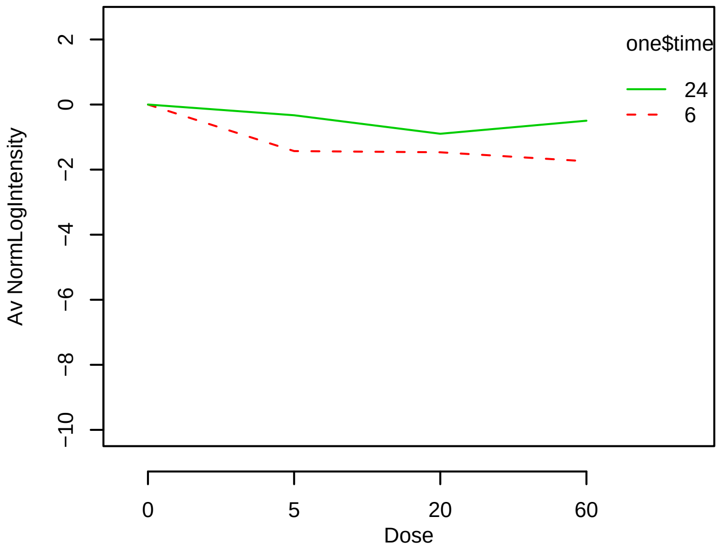

GO_0030258 : lipid modification

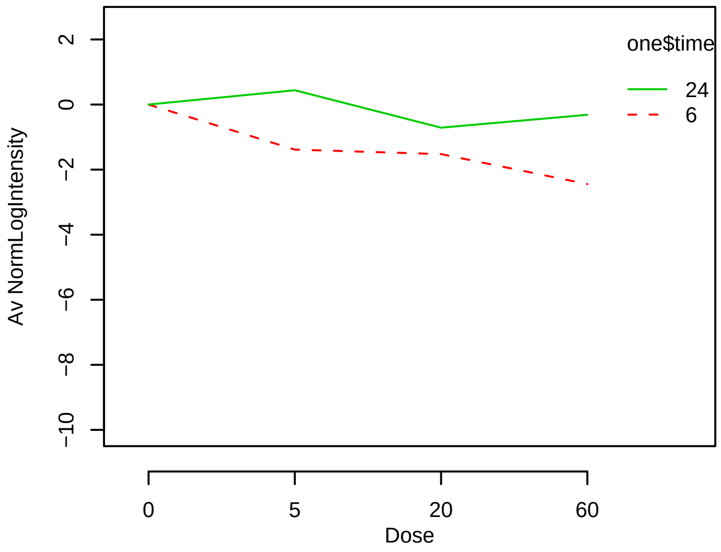

GO_0030260 : entry into host cell

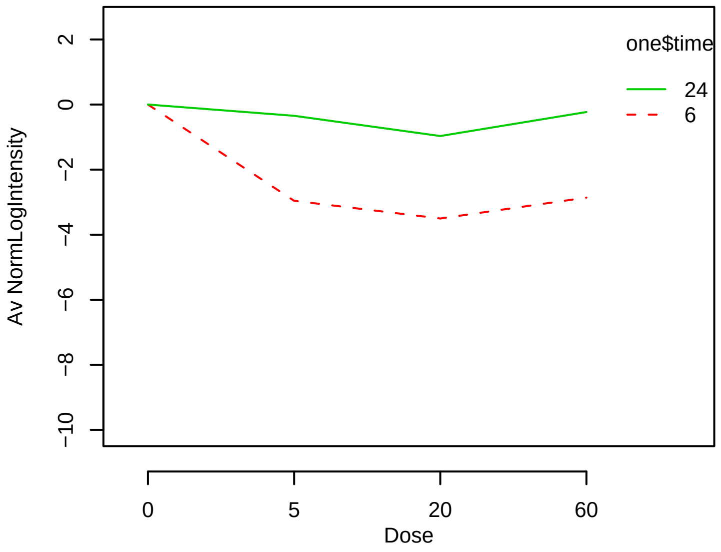

GO_0030261 : chromosome condensation

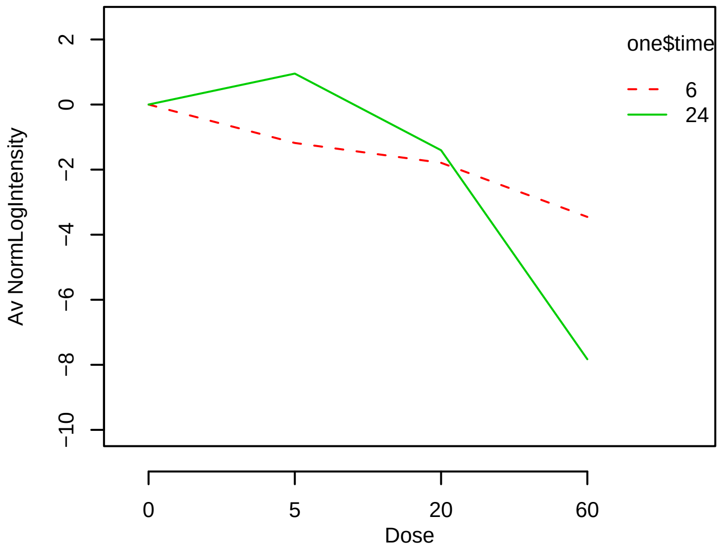

GO_0030262 : apoptotic nuclear changes

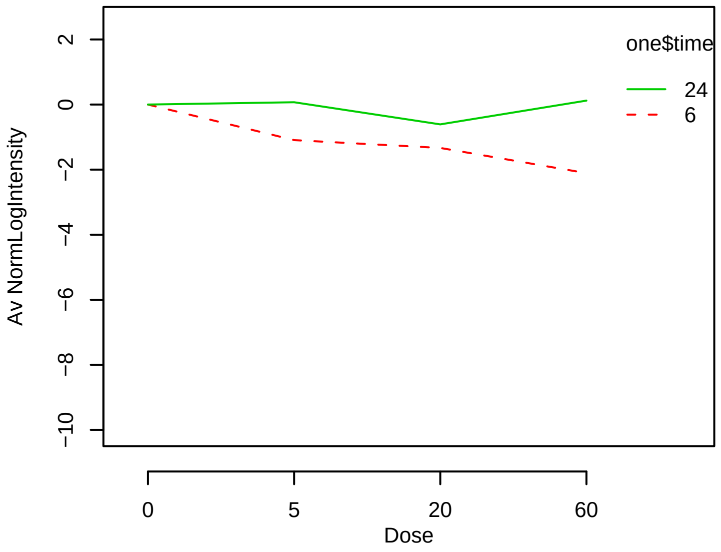

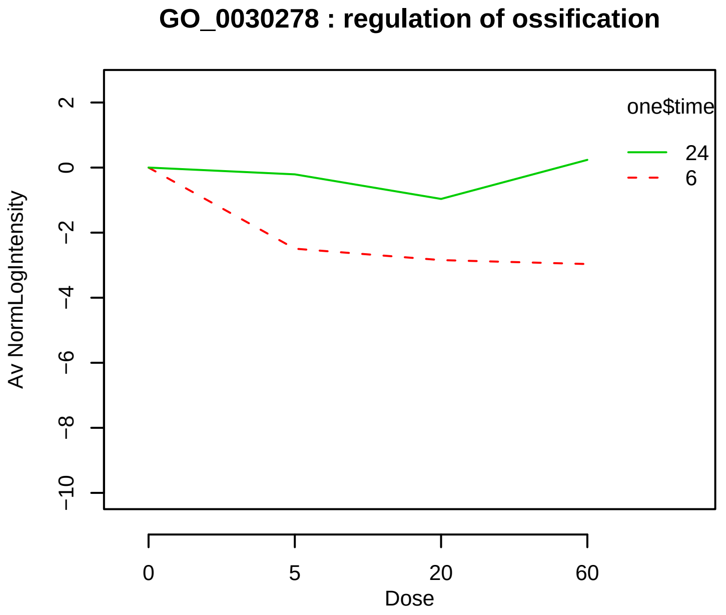
GO_0030278 : regulation of ossification
one$time
24
6
Av NormLogIntensity
Dose
0
5
20
60
2
0
−2
−4
−6
−8
−10

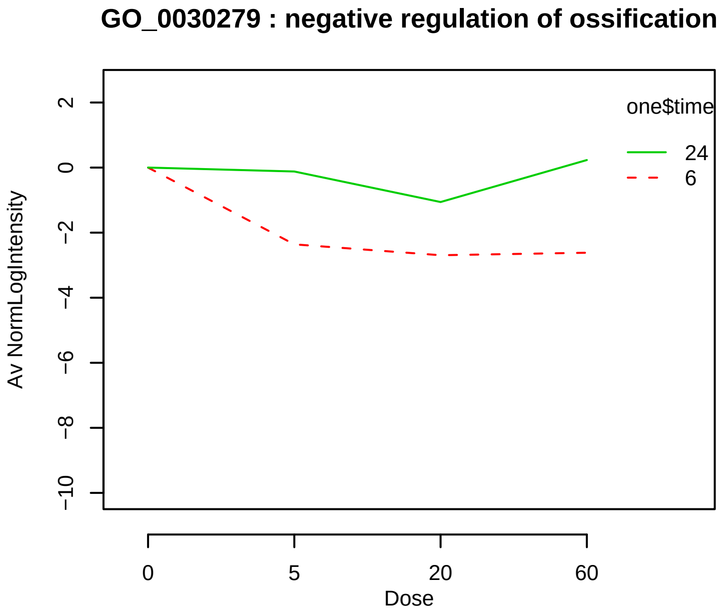
GO_0030279 : negative regulation of ossification
one$time
24
6
Av NormLogIntensity
Dose
0
5
20
60
2
0
−2
−4
−6
−8
−10

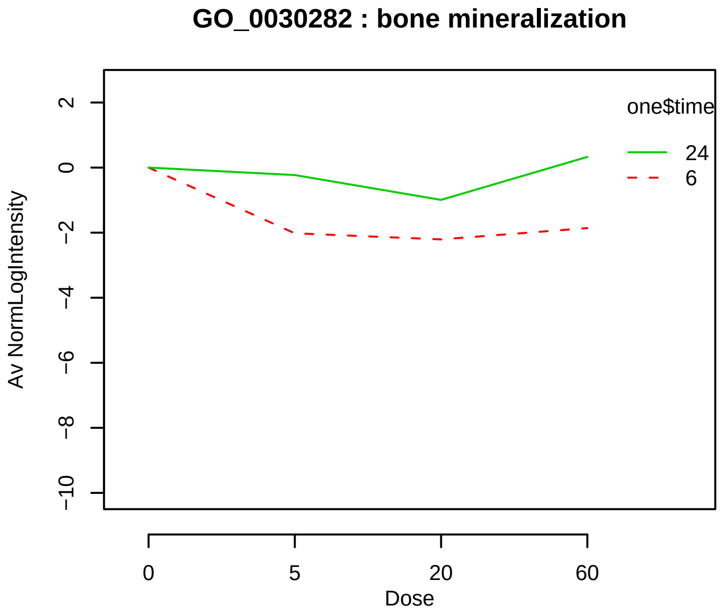
GO_0030282 : bone mineralization
one$time
24
6
Av NormLogIntensity
Dose
0
5
20
60
2
0
−2
−4
−6
−8
−10

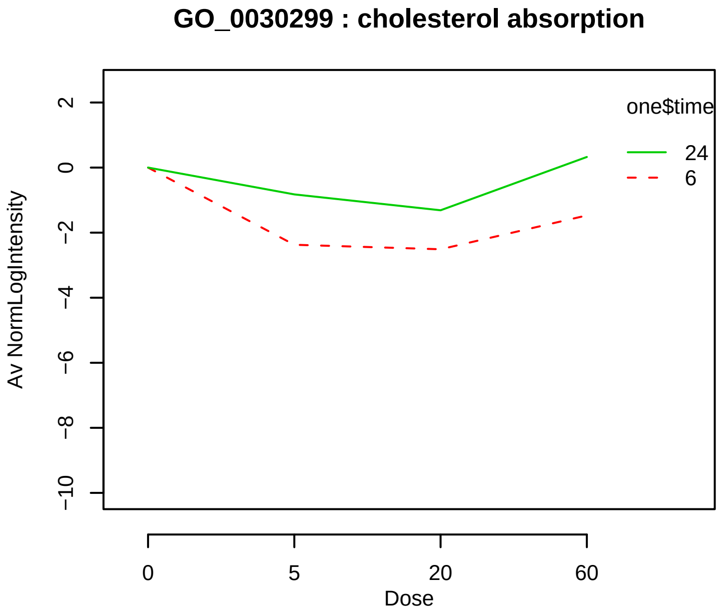
GO_0030299 : cholesterol absorption
one$time
24
6
Av NormLogIntensity
Dose
0
5
20
60
2
0
−2
−4
−6
−8
−10

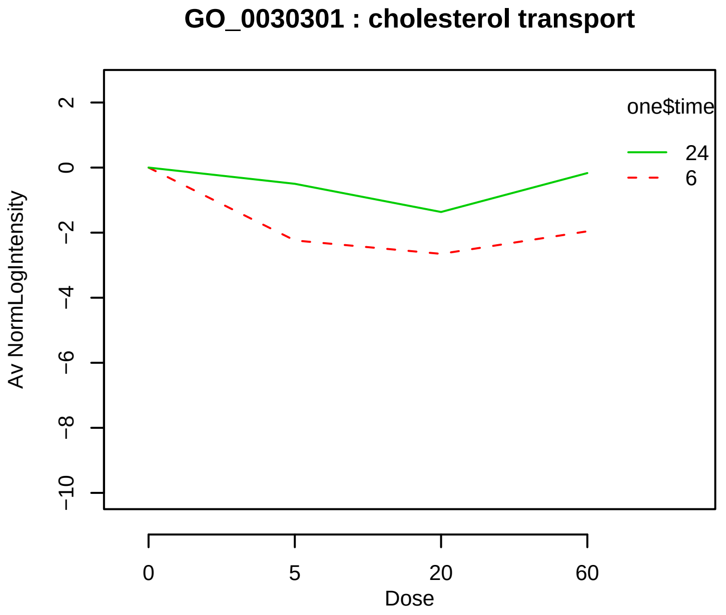
GO_0030301 : cholesterol transport
one$time
24
6
Av NormLogIntensity
Dose
0
5
20
60
2
0
−2
−4
−6
−8
−10

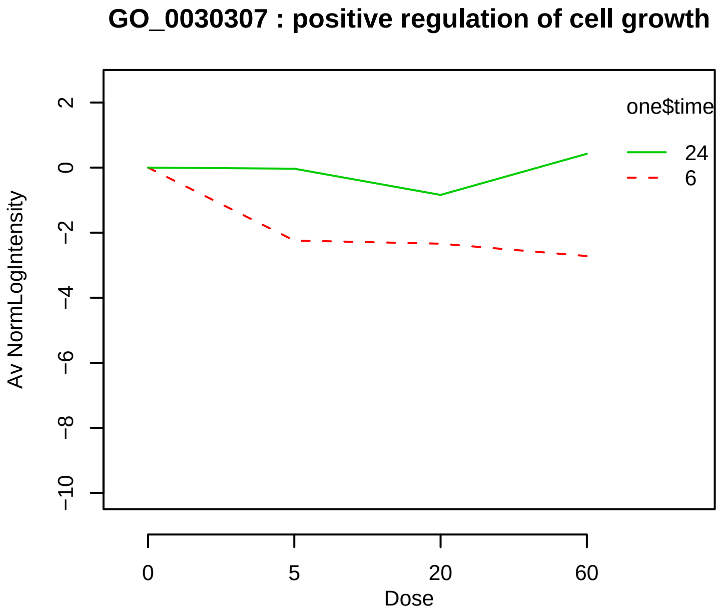
GO_0030307 : positive regulation of cell growth
one$time
24
6
Av NormLogIntensity
Dose
0
5
20
60
2
0
−2
−4
−6
−8
−10

GO_0030308 : negative regulation of cell growth

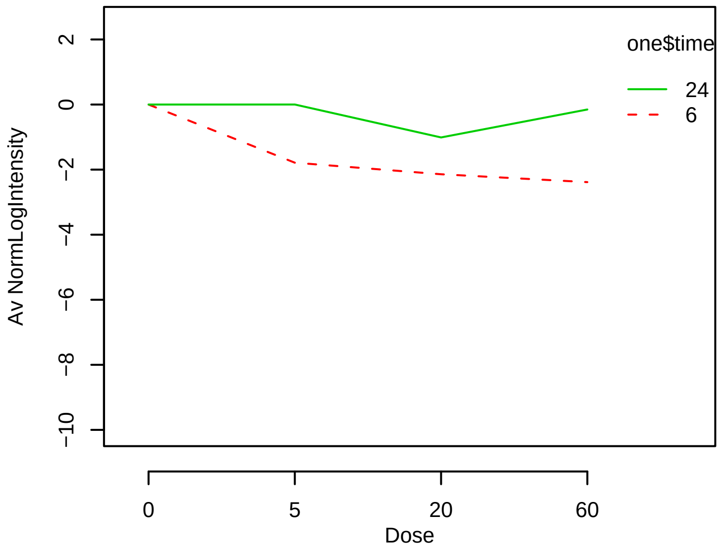

GO_0030316 : osteoclast differentiation

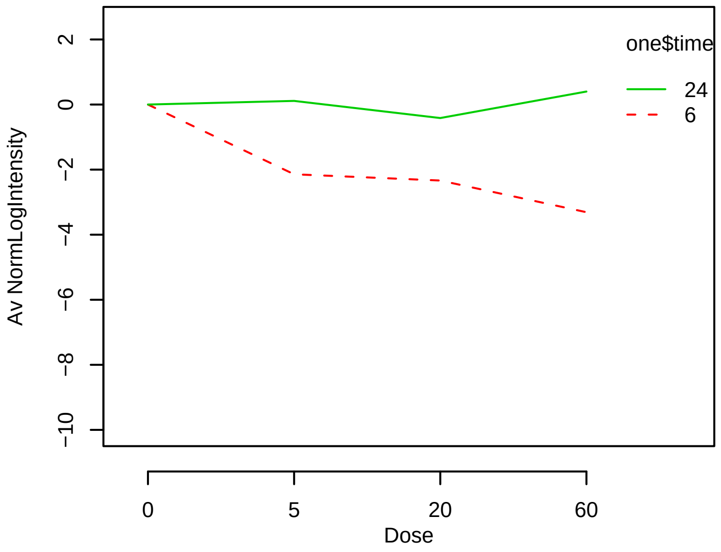

GO_0030317 : sperm motility

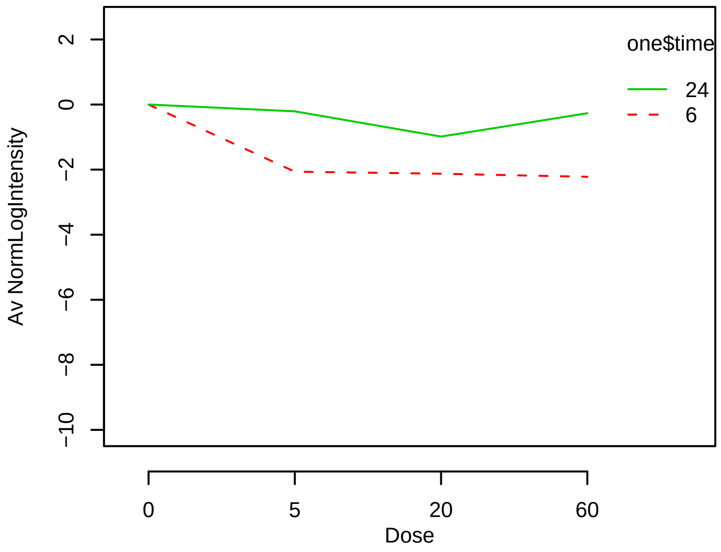

GO_0030318 : melanocyte differentiation

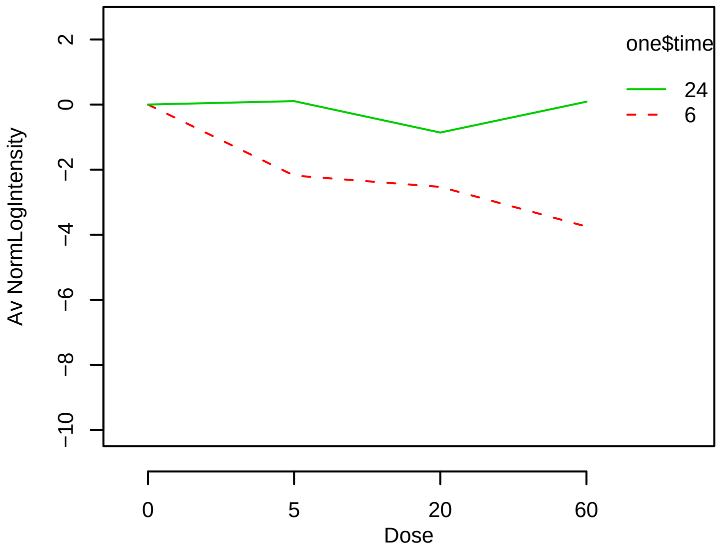

GO_0030323 : respiratory tube development

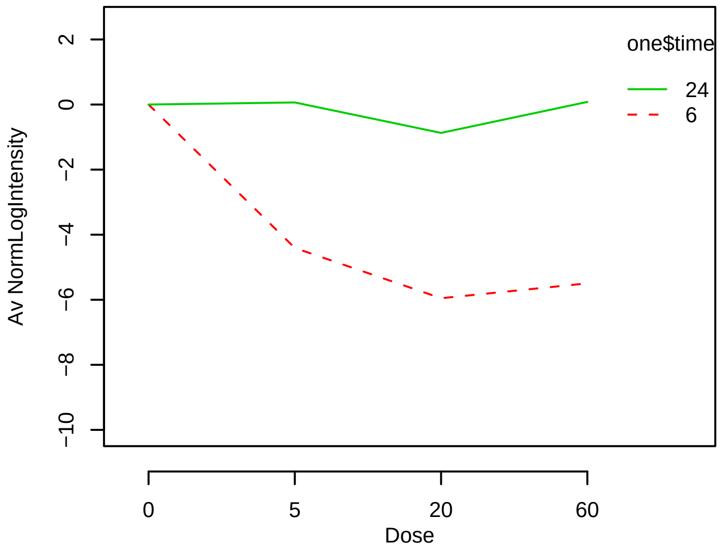

GO_0030324 : lung development

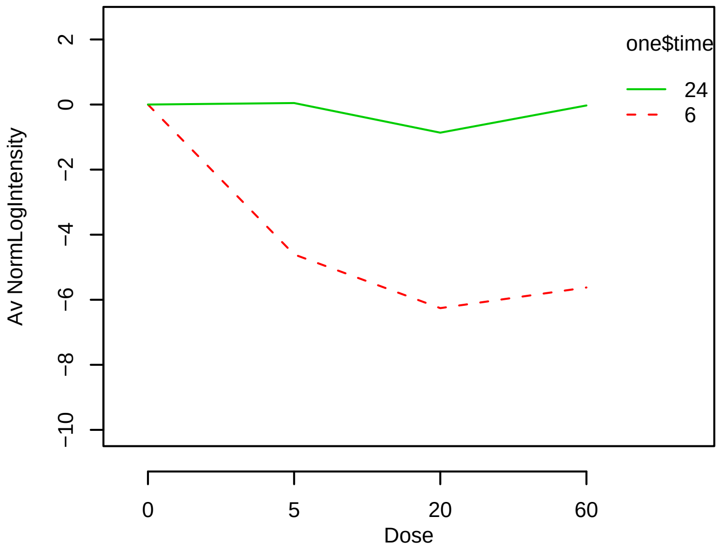

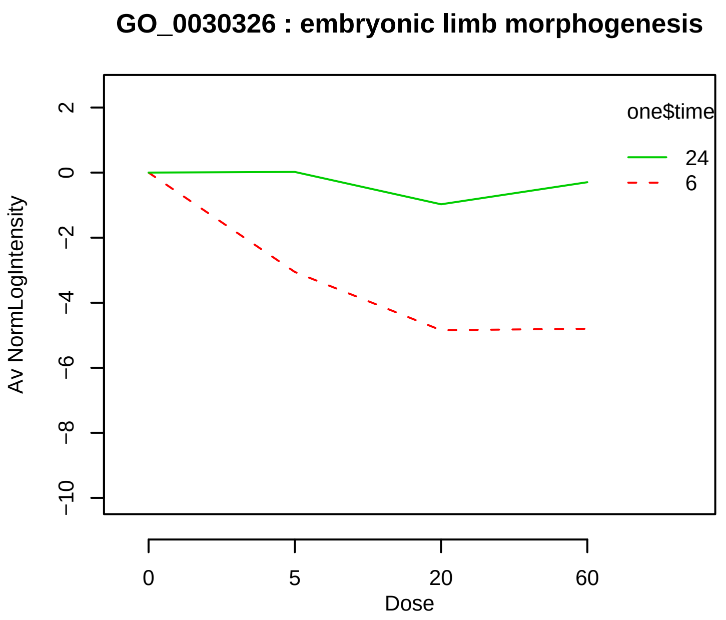
GO_0030326 : embryonic limb morphogenesis
one$time
24
6
Av NormLogIntensity
2
0
−2
−4
−6
−8
−10
0
5
20
60
Dose

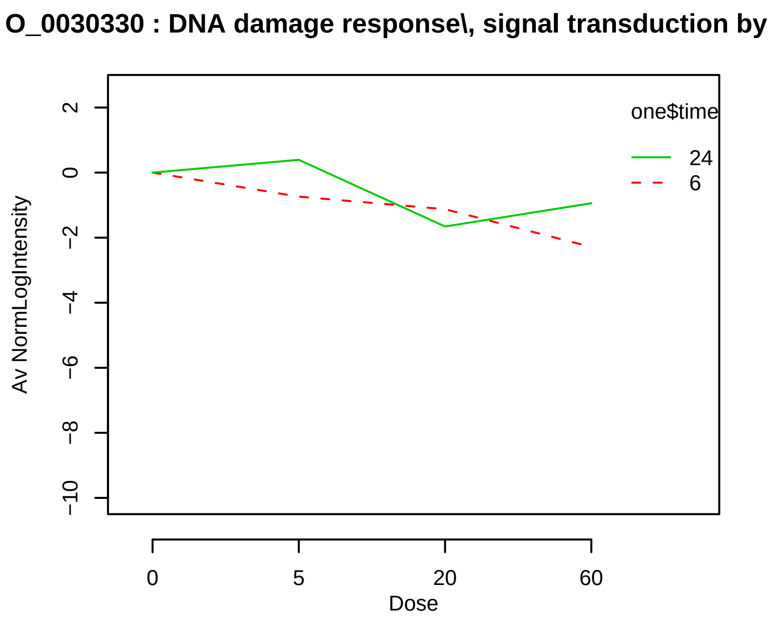
O_0030330 : DNA damage response\, signal transduction by
one$time
24
6
Av NormLogIntensity
2
0
−2
−4
−6
−8
−10
0
5
20
60
Dose

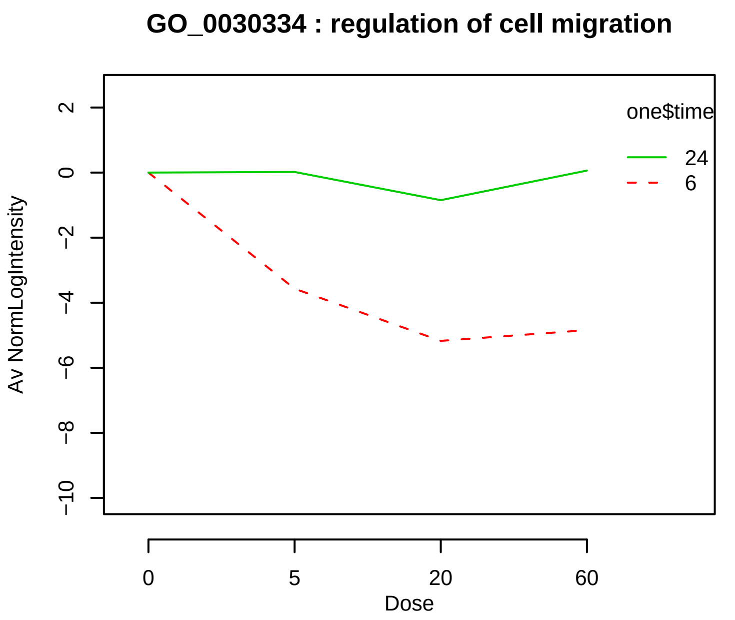
GO_0030334 : regulation of cell migration
one$time
24
6
Av NormLogIntensity
2
0
−2
−4
−6
−8
−10
0
5
20
60
Dose

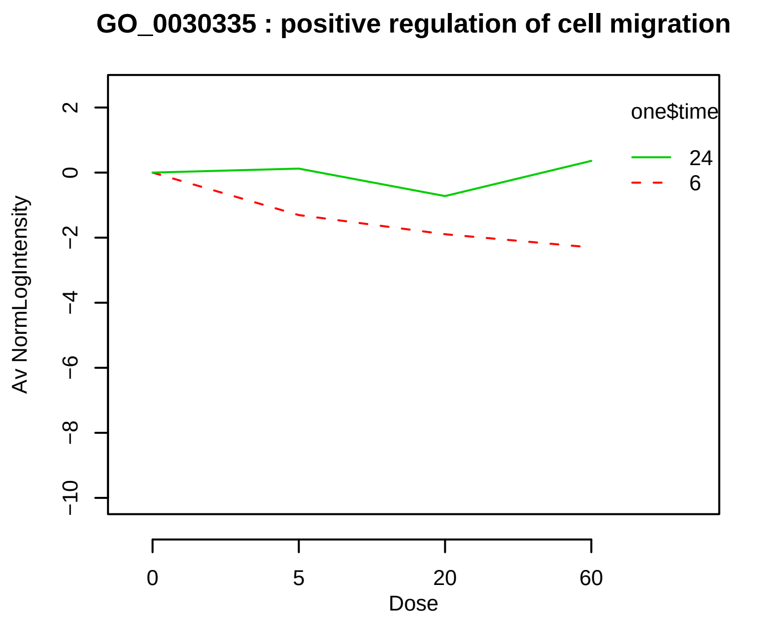
GO_0030335 : positive regulation of cell migration
one$time
24
6
Av NormLogIntensity
2
0
−2
−4
−6
−8
−10
0
5
20
60
Dose

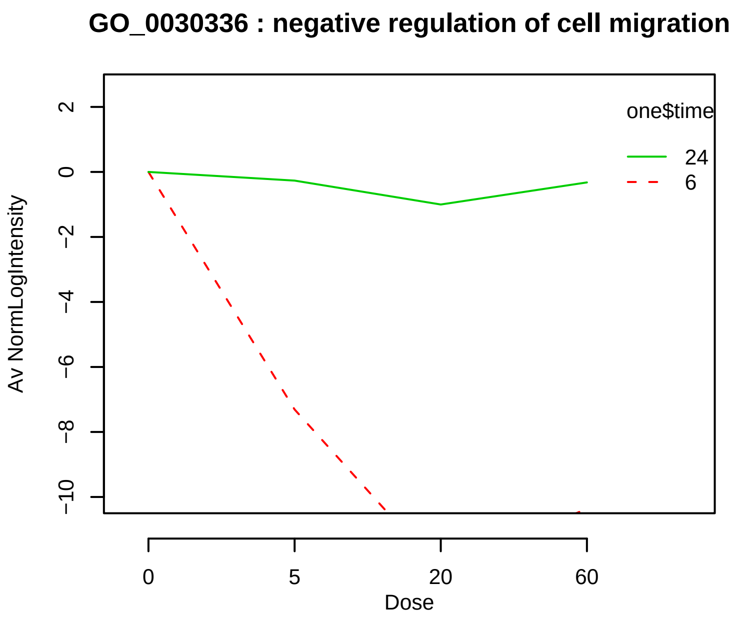
GO_0030336 : negative regulation of cell migration
one$time
24
6
Av NormLogIntensity
2
0
−2
−4
−6
−8
−10
0
5
20
60
Dose

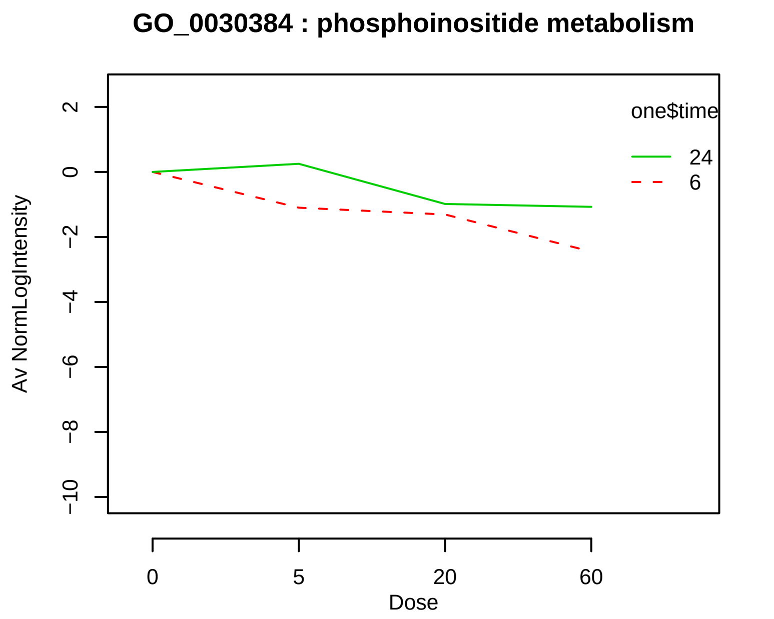
GO_0030384 : phosphoinositide metabolism
one$time
24
6
Av NormLogIntensity
2
0
−2
−4
−6
−8
−10
0
5
20
60
Dose

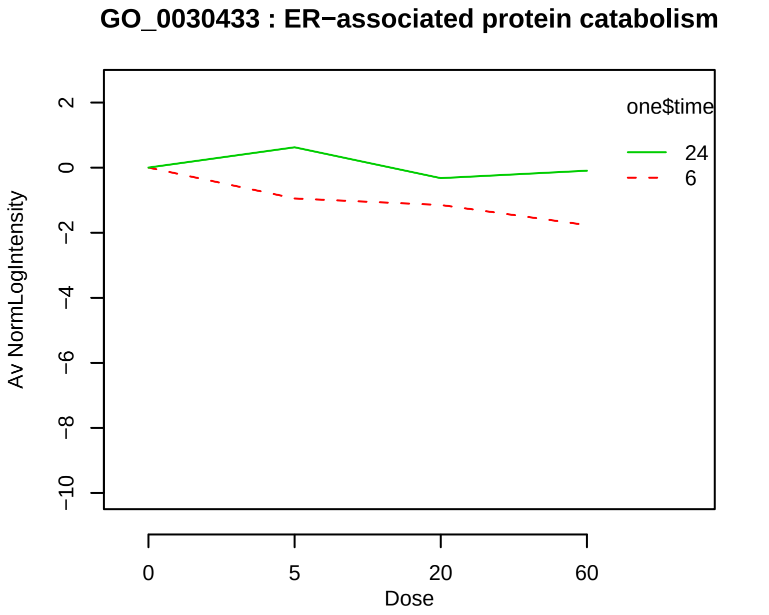
GO_0030433 : ER−associated protein catabolism
one$time
24
6
Av NormLogIntensity
Dose

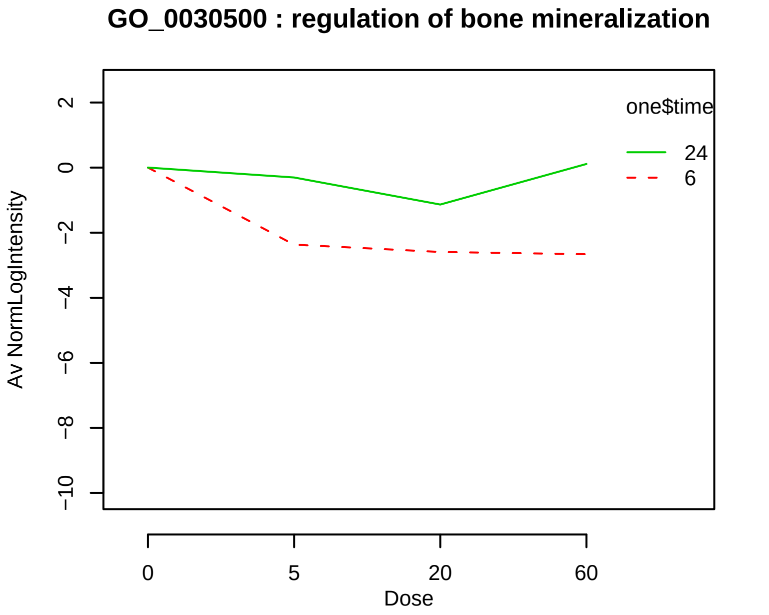
GO_0030500 : regulation of bone mineralization
one$time
24
6
Av NormLogIntensity
Dose

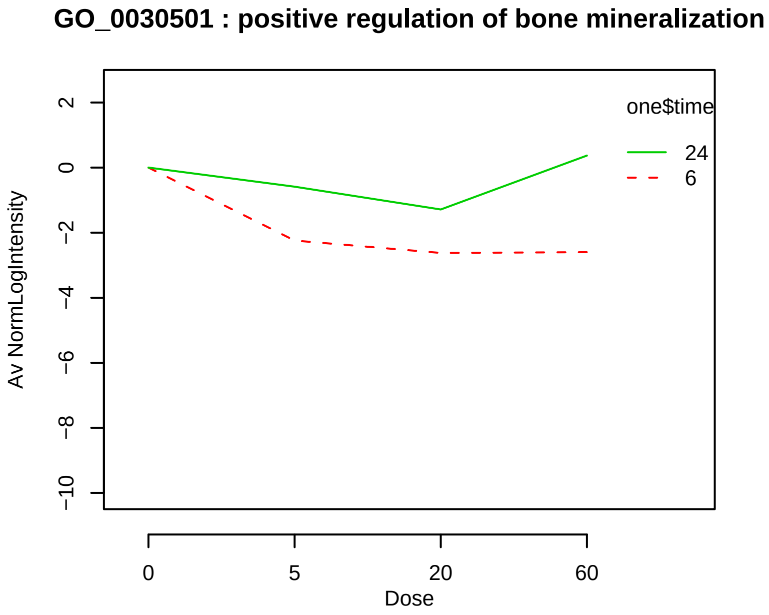
GO_0030501 : positive regulation of bone mineralization
one$time
24
6
Av NormLogIntensity
Dose

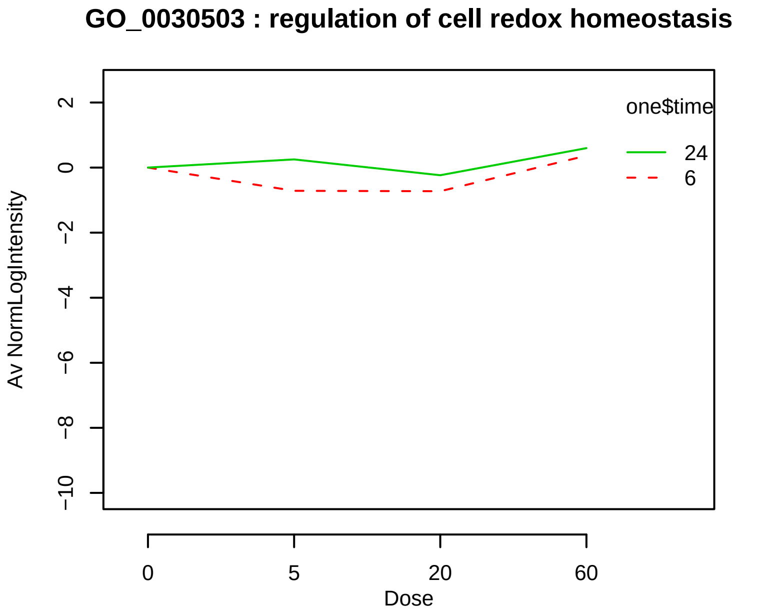
GO_0030503 : regulation of cell redox homeostasis
one$time
24
6
Av NormLogIntensity
Dose

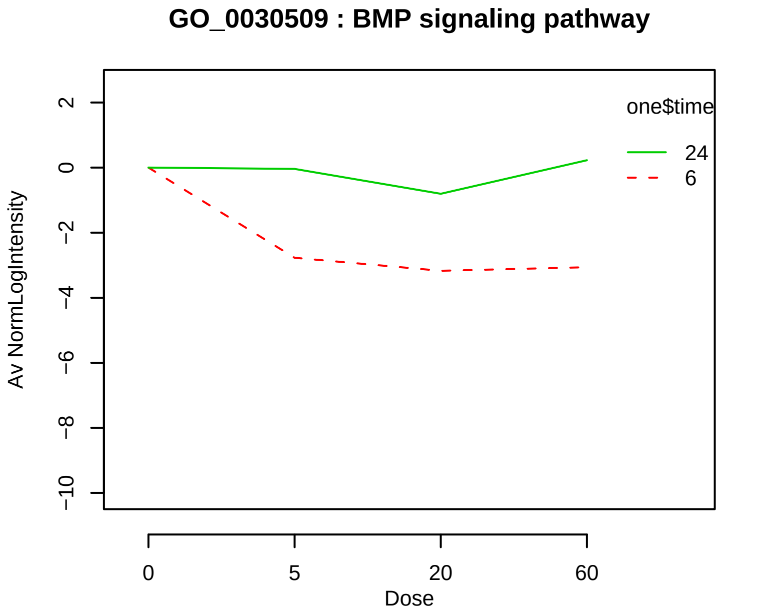
GO_0030509 : BMP signaling pathway
one$time
24
6
Av NormLogIntensity
Dose

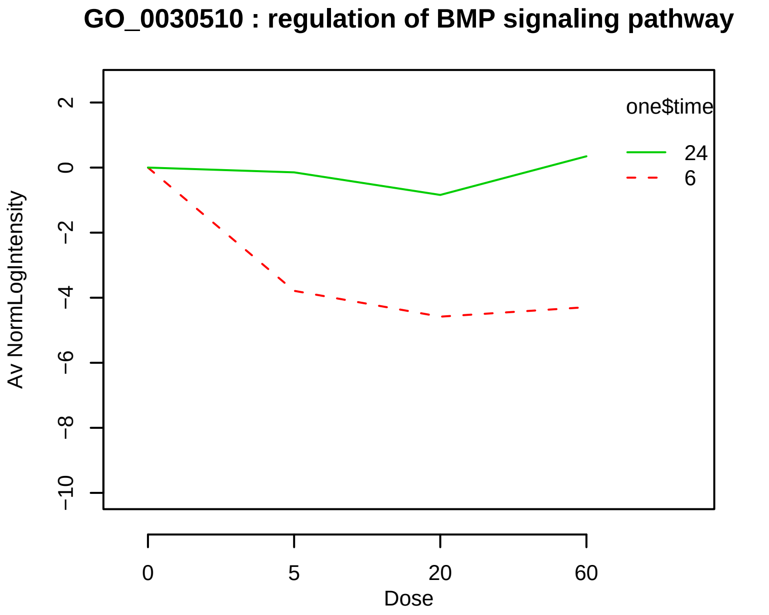
GO_0030510 : regulation of BMP signaling pathway
one$time
24
6
Av NormLogIntensity
Dose

GO_0030512 : negative regulation of transforming growth fa

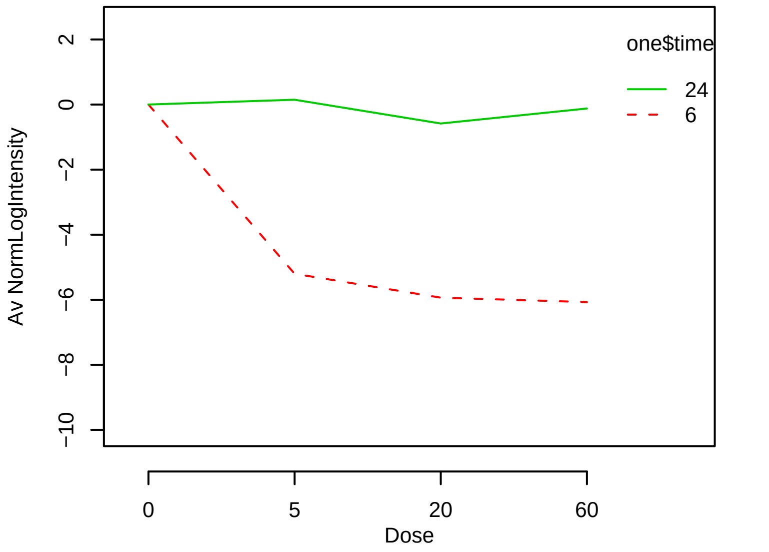

GO_0030514 : negative regulation of BMP signaling pathwa

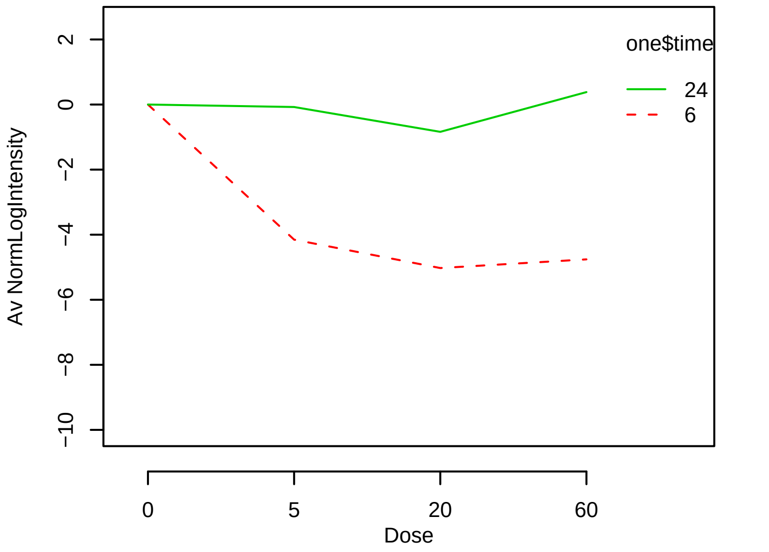

GO_0030516 : regulation of axon extension

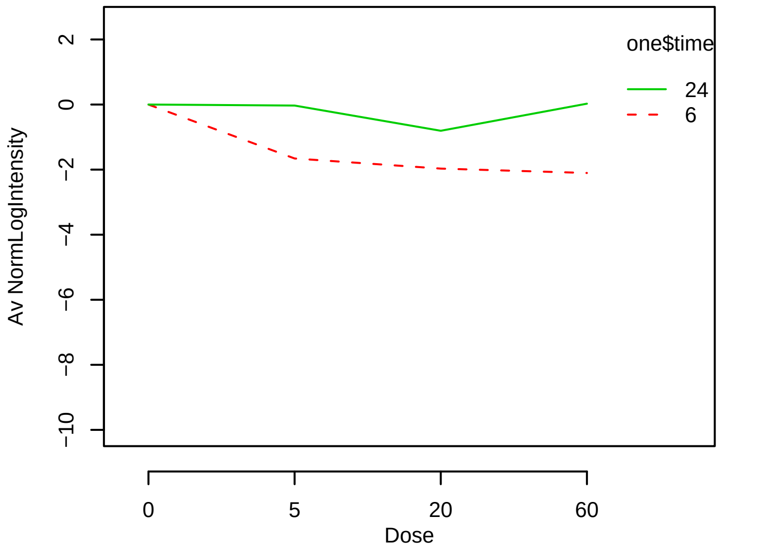

GO_0030517 : negative regulation of axon extension

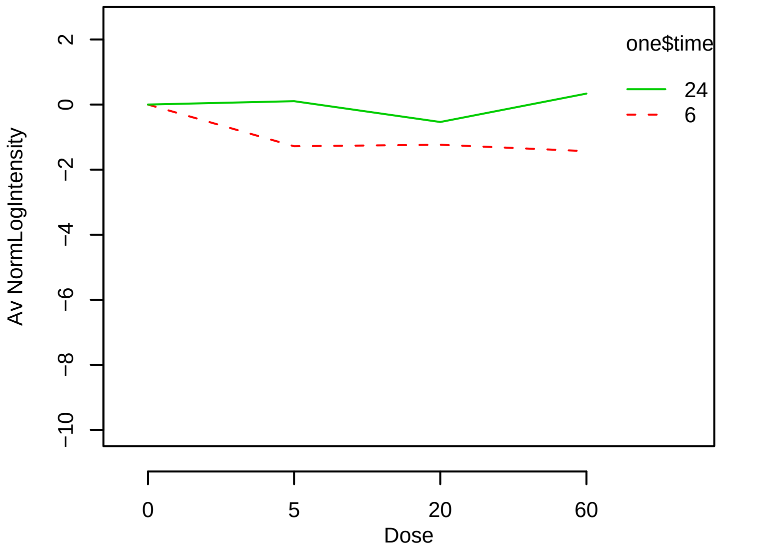

GO_0030518 : steroid hormone receptor signaling pathwa

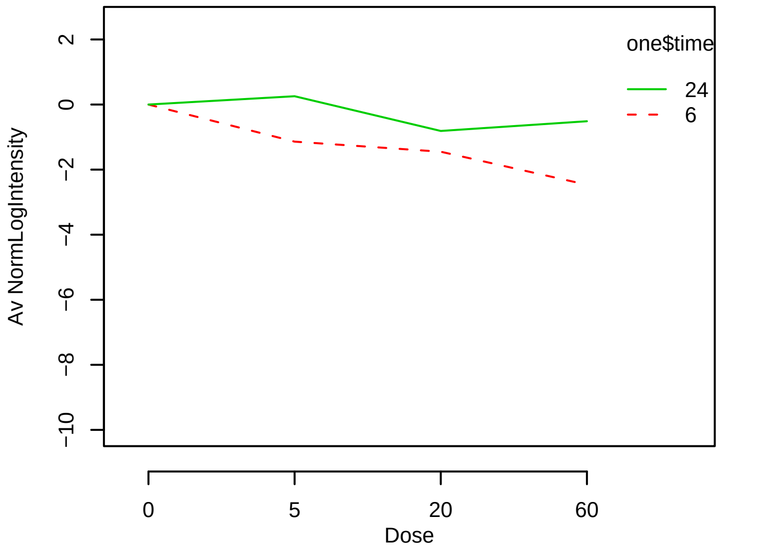

GO_0030520 : estrogen receptor signaling pathway

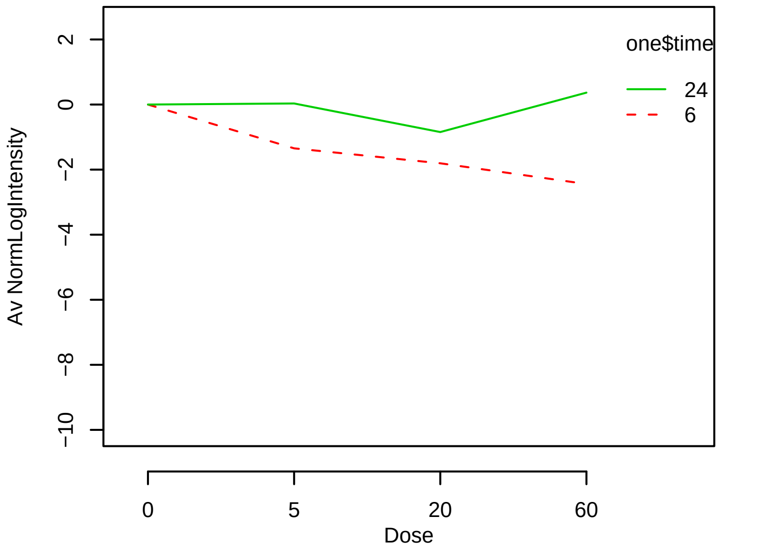

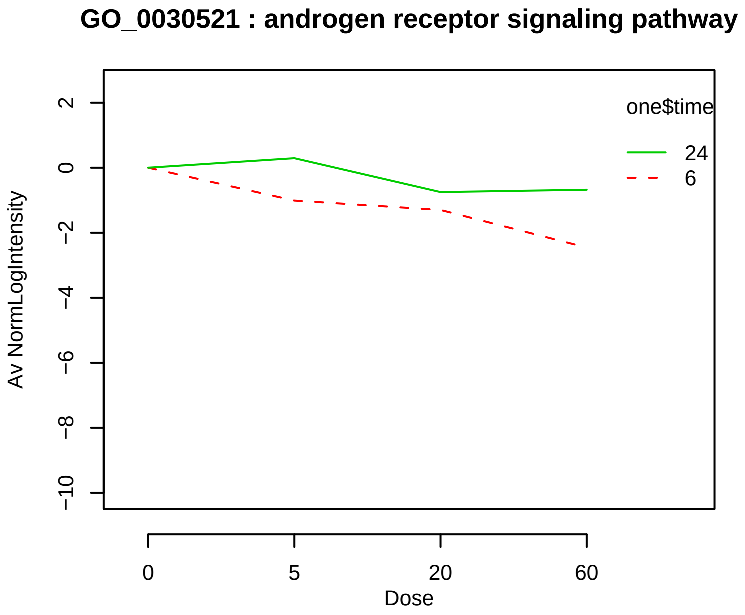
GO_0030521 : androgen receptor signaling pathway
Av NormLogIntensity
Dose
one$time
24
6

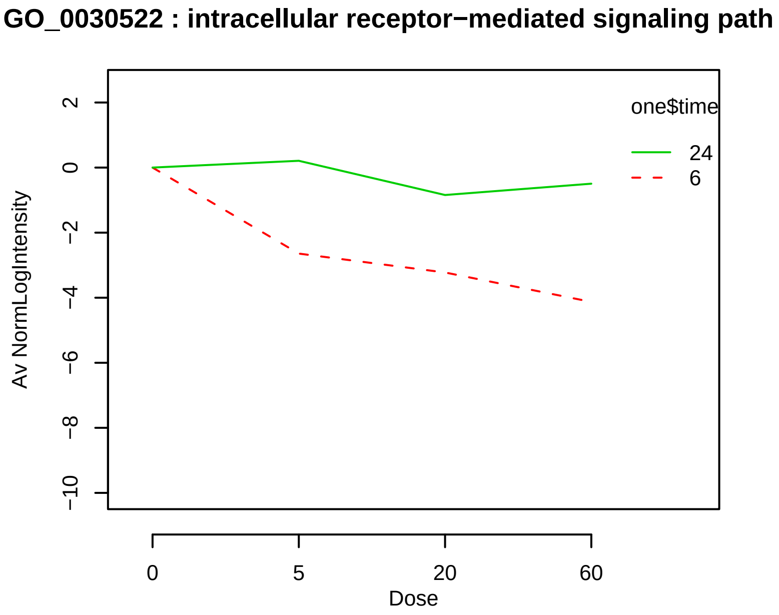
GO_0030522 : intracellular receptor−mediated signaling path
Av NormLogIntensity
Dose
one$time
24
6

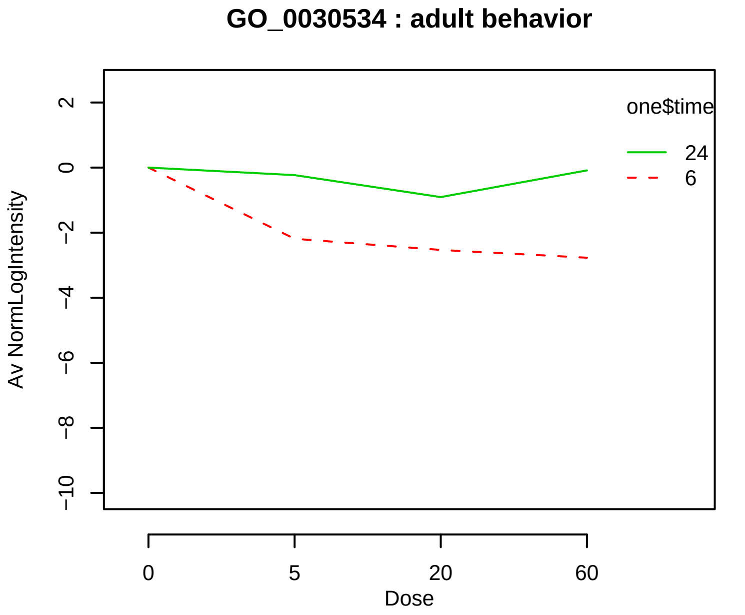
GO_0030534 : adult behavior
Av NormLogIntensity
Dose
one$time
24
6

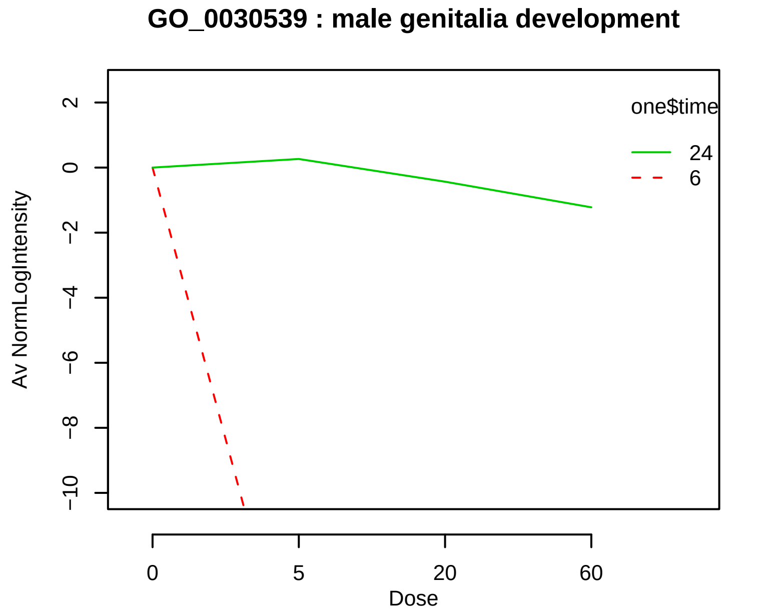
GO_0030539 : male genitalia development
Av NormLogIntensity
Dose
one$time
24
6

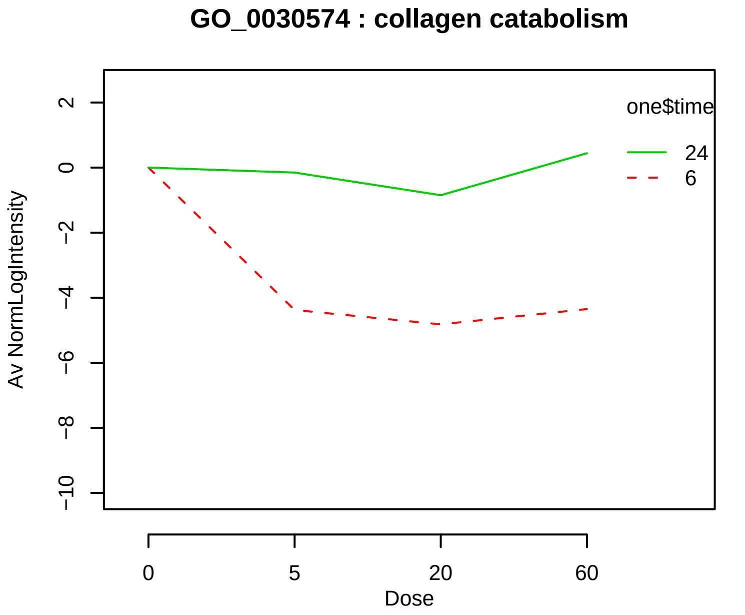
GO_0030574 : collagen catabolism
Av NormLogIntensity
Dose
one$time
24
6

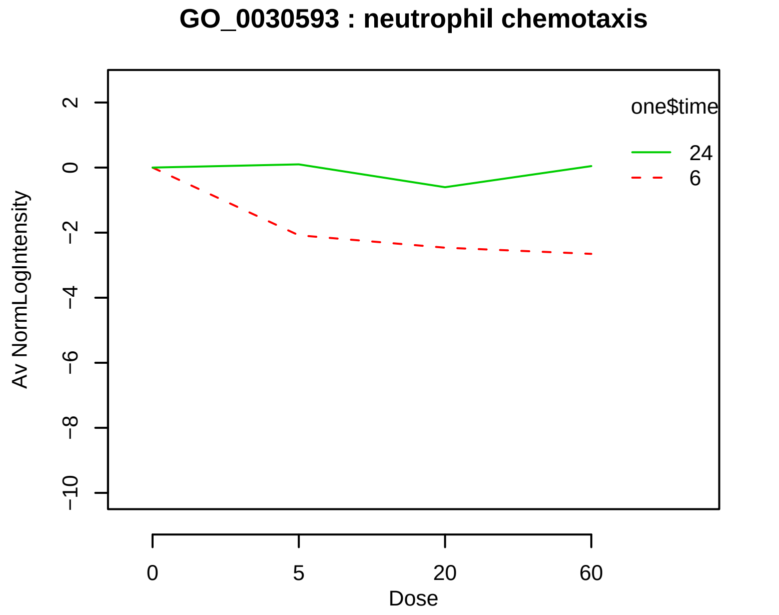
GO_0030593 : neutrophil chemotaxis
Av NormLogIntensity
Dose
one$time
24
6

GO_0030595 : leukocyte chemotaxis

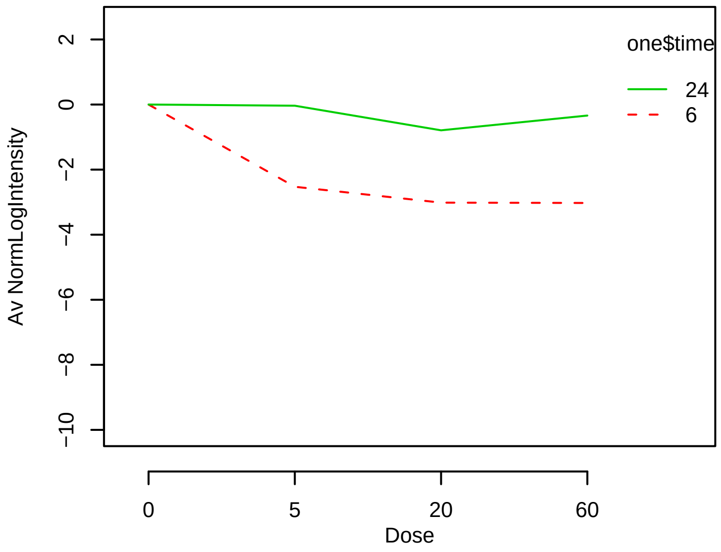

GO_0030641 : hydrogen ion homeostasis

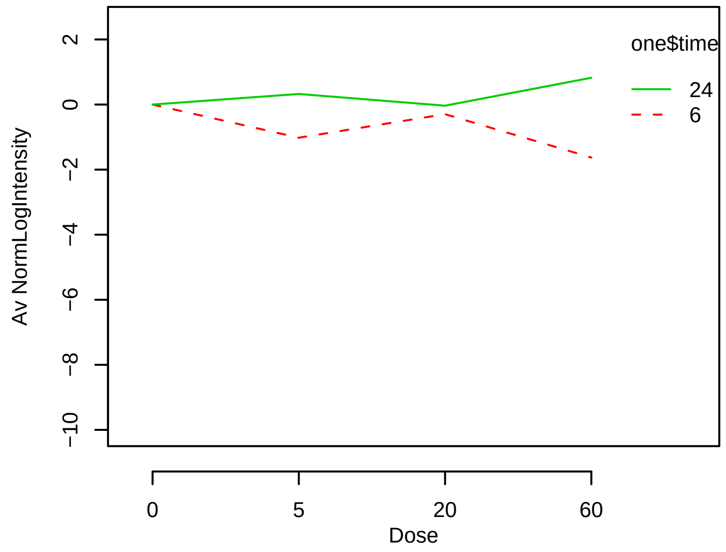

GO_0030705 : cytoskeleton−dependent intracellular transp

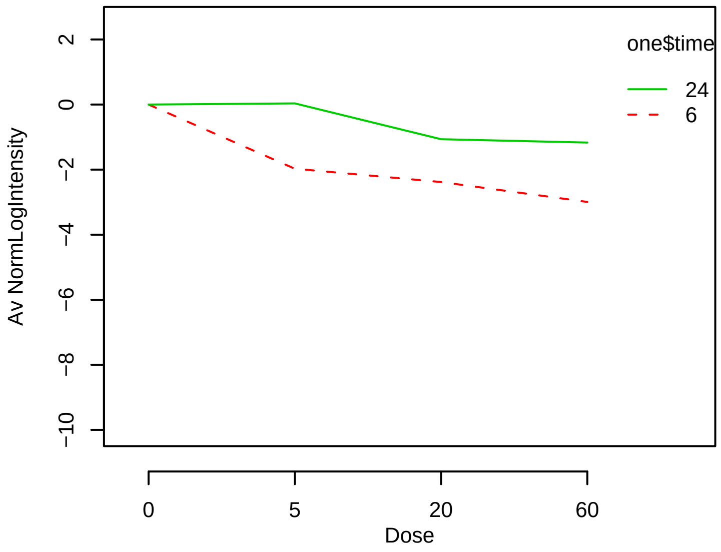

GO_0030728 : ovulation

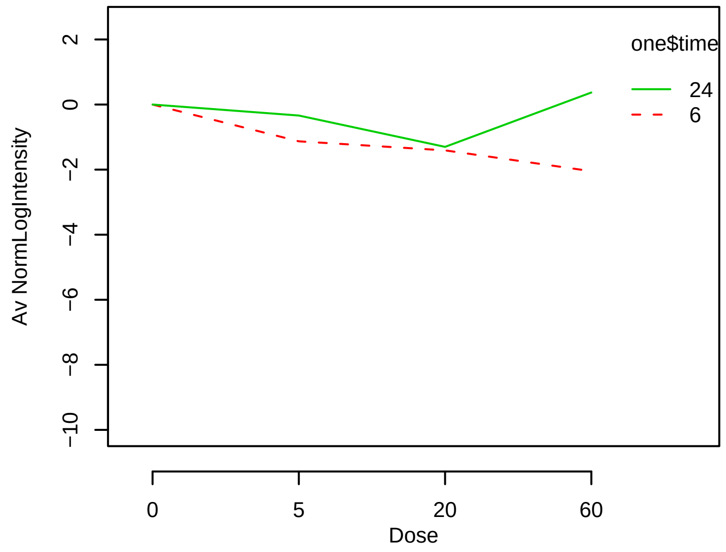

GO_0030799 : regulation of cyclic nucleotide metabolism

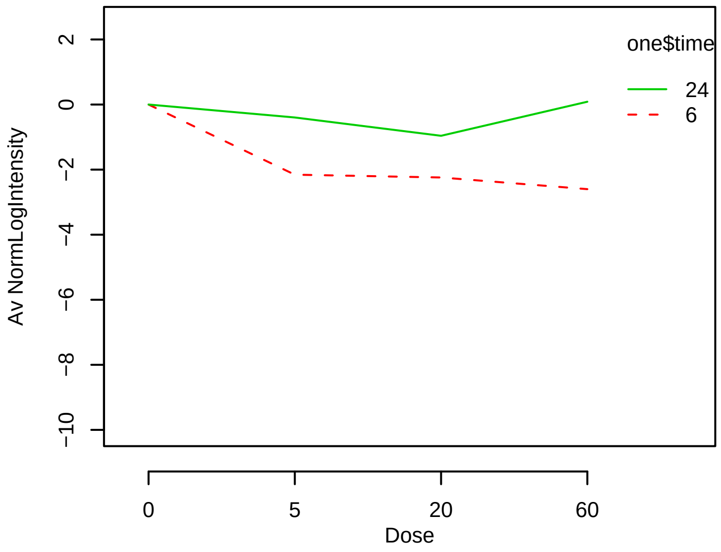

GO_0030802 : regulation of cyclic nucleotide biosynthesi

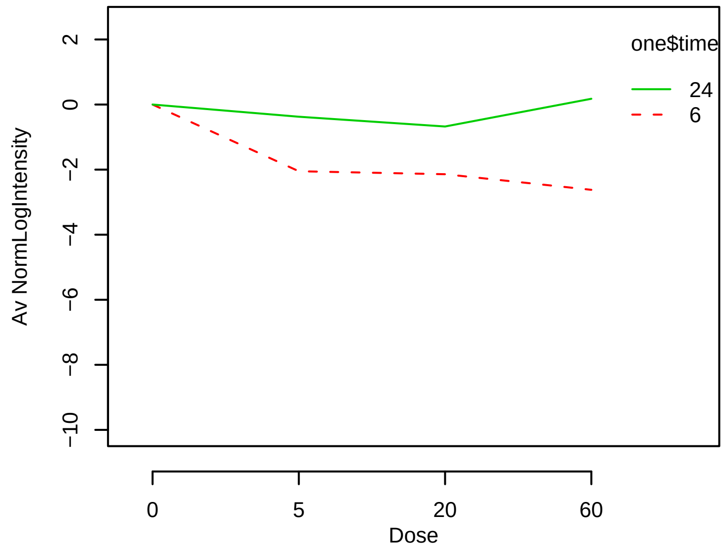

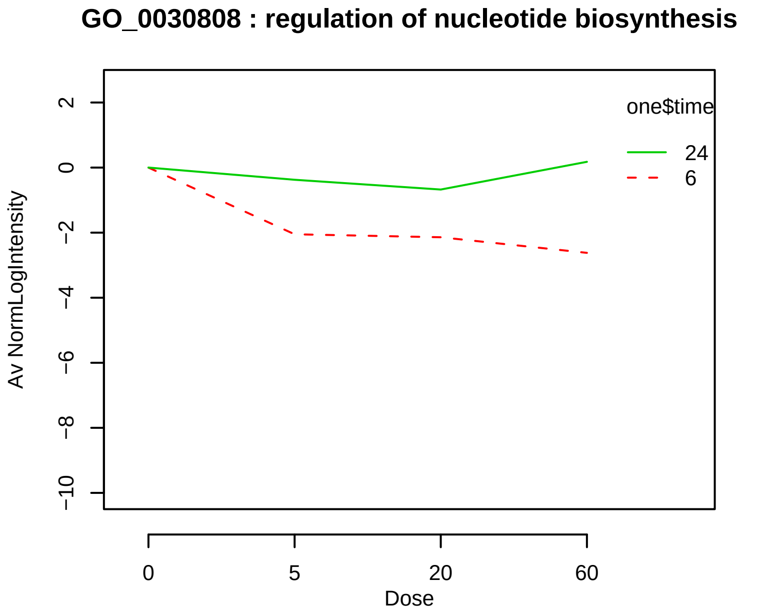
GO_0030808 : regulation of nucleotide biosynthesis
one$time
24
6
Av NormLogIntensity
2
0
−2
−4
−6
−8
−10
0
5
20
60
Dose

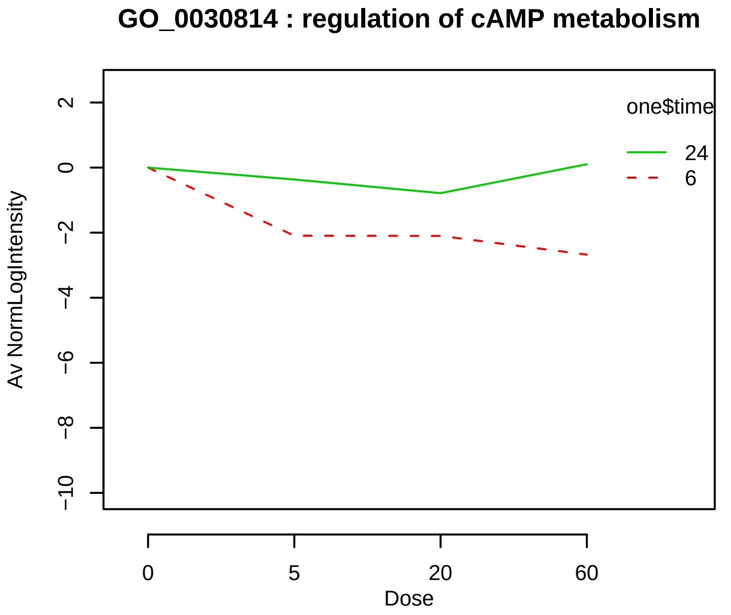
GO_0030814 : regulation of cAMP metabolism
one$time
24
6
Av NormLogIntensity
2
0
−2
−4
−6
−8
−10
0
5
20
60
Dose

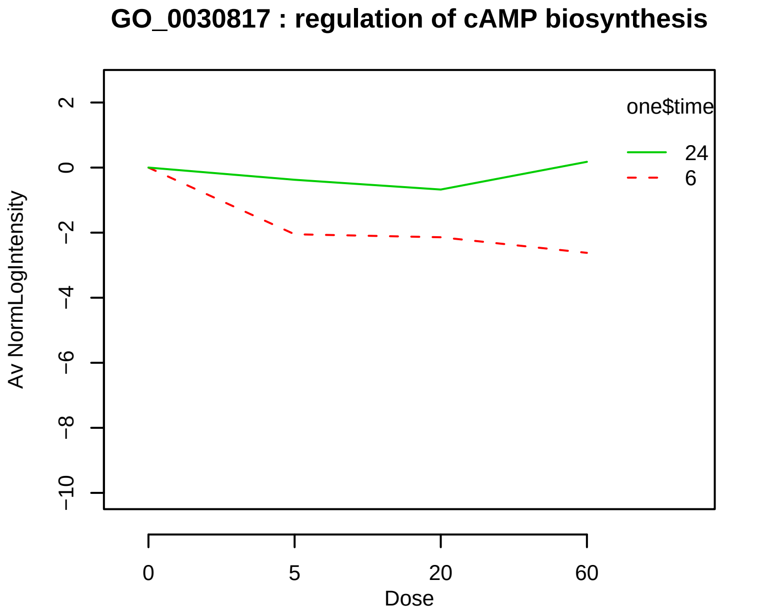
GO_0030817 : regulation of cAMP biosynthesis
one$time
24
6
Av NormLogIntensity
2
0
−2
−4
−6
−8
−10
0
5
20
60
Dose

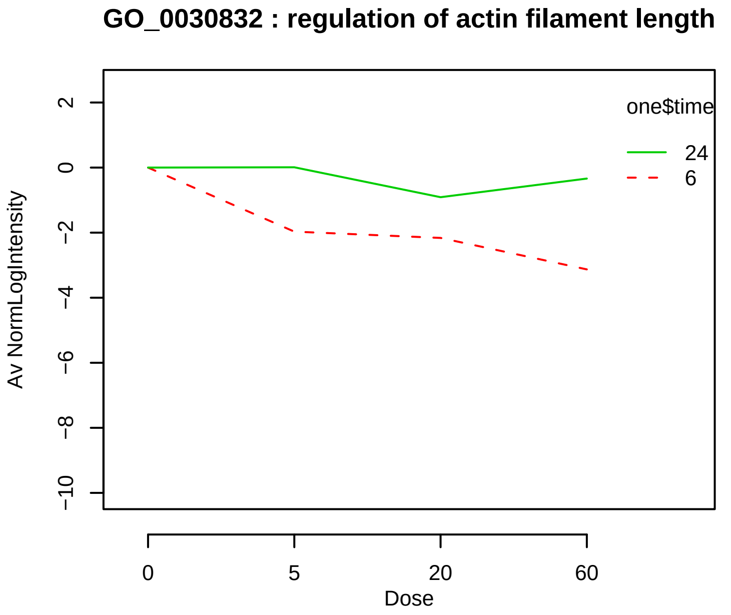
GO_0030832 : regulation of actin filament length
one$time
24
6
Av NormLogIntensity
2
0
−2
−4
−6
−8
−10
0
5
20
60
Dose

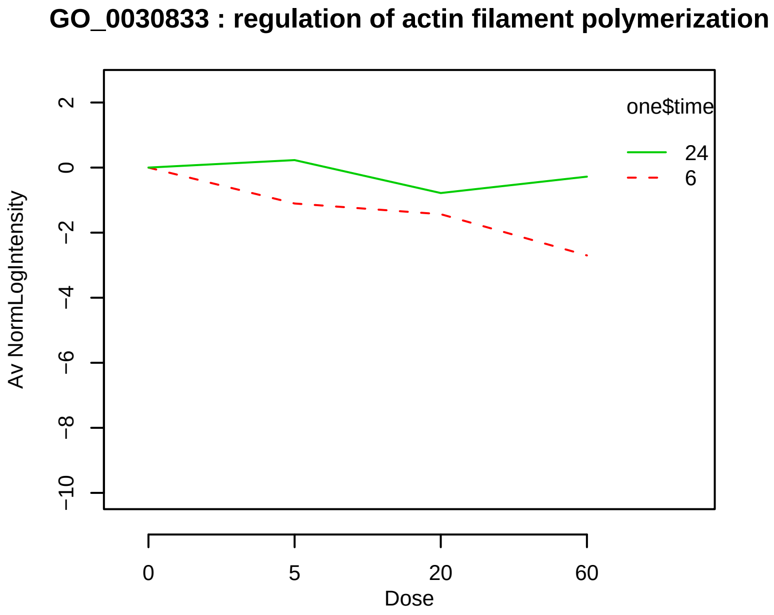
GO_0030833 : regulation of actin filament polymerization
one$time
24
6
Av NormLogIntensity
2
0
−2
−4
−6
−8
−10
0
5
20
60
Dose

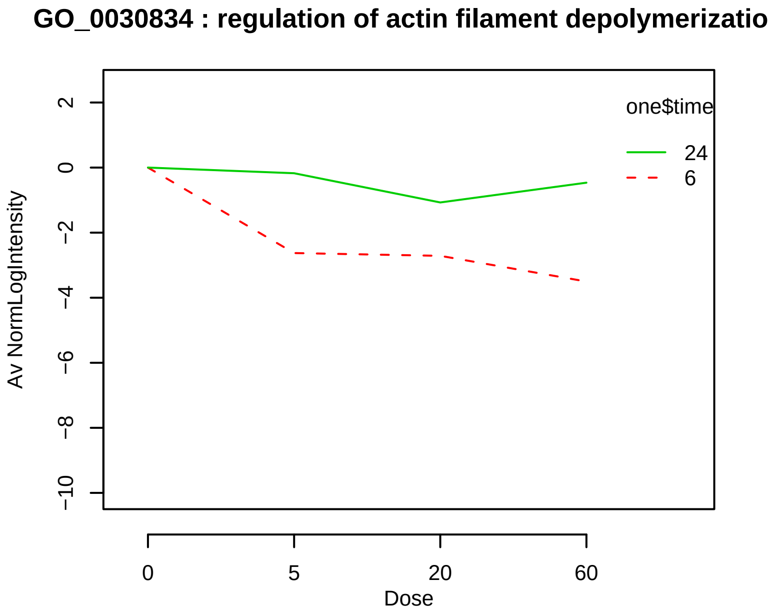
GO_0030834 : regulation of actin filament depolymerizatio
one$time
24
6
Av NormLogIntensity
2
0
−2
−4
−6
−8
−10
0
5
20
60
Dose

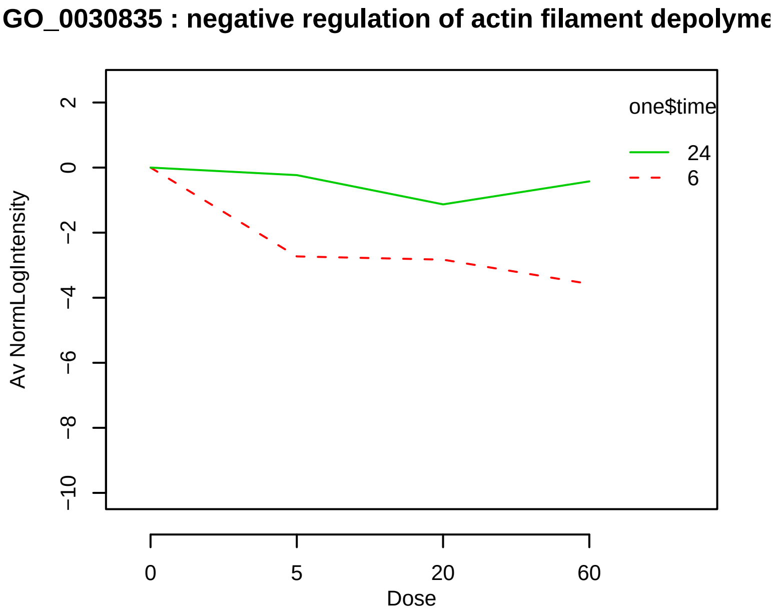
GO_0030835 : negative regulation of actin filament depolyme
Av NormLogIntensity
Dose
one$time
24
6
2
0
−2
−4
−6
−8
−10
0
5
20
60

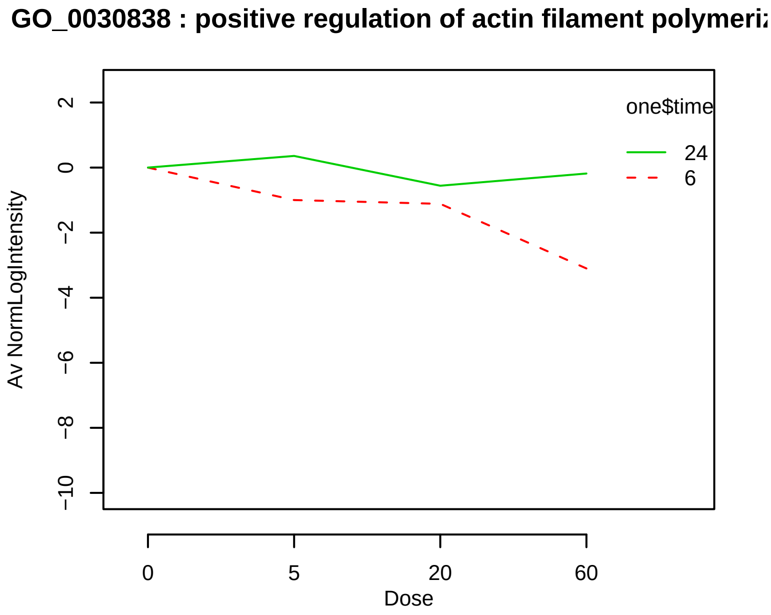
GO_0030838 : positive regulation of actin filament polymeriz
Av NormLogIntensity
Dose
one$time
24
6
2
0
−2
−4
−6
−8
−10
0
5
20
60

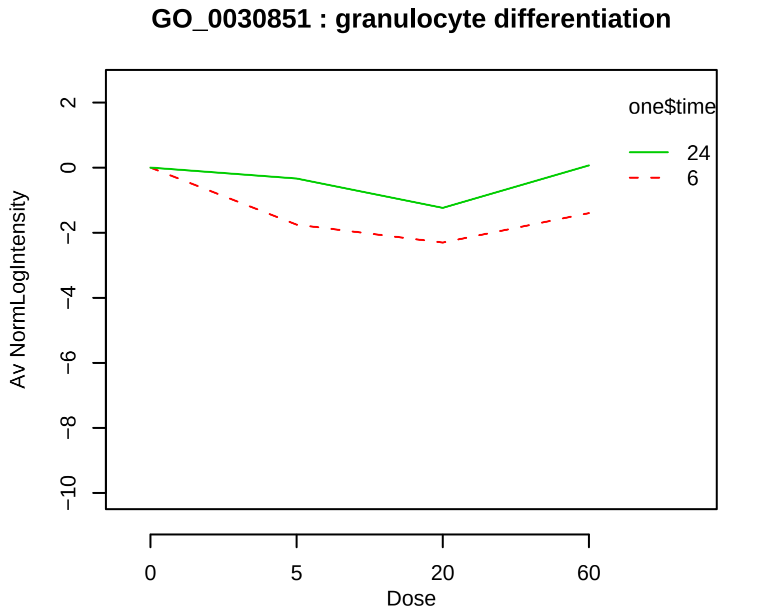
GO_0030851 : granulocyte differentiation
Av NormLogIntensity
Dose
one$time
24
6
2
0
−2
−4
−6
−8
−10
0
5
20
60

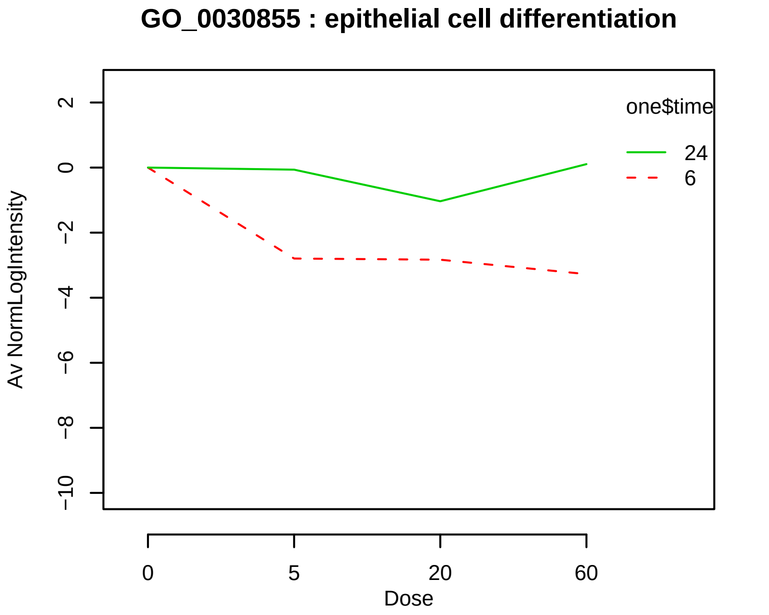
GO_0030855 : epithelial cell differentiation
Av NormLogIntensity
Dose
one$time
24
6
2
0
−2
−4
−6
−8
−10
0
5
20
60

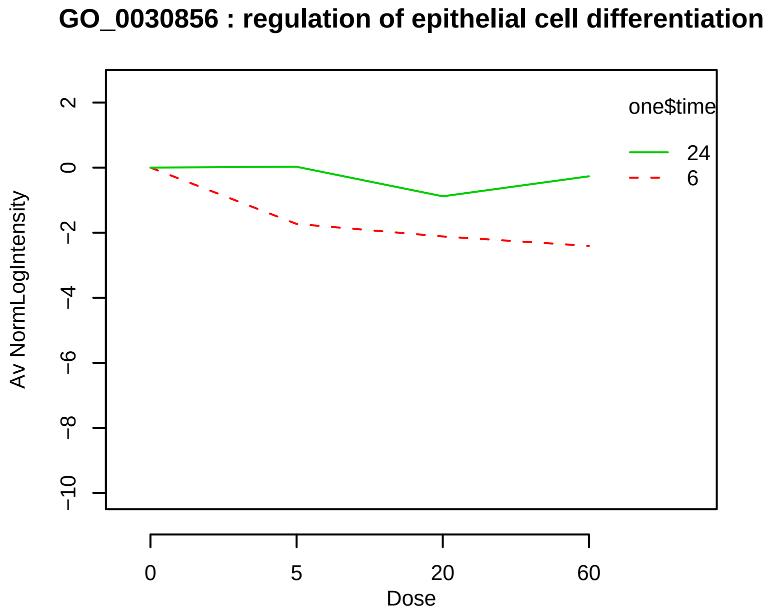
GO_0030856 : regulation of epithelial cell differentiation
Av NormLogIntensity
Dose
one$time
24
6
2
0
−2
−4
−6
−8
−10
0
5
20
60

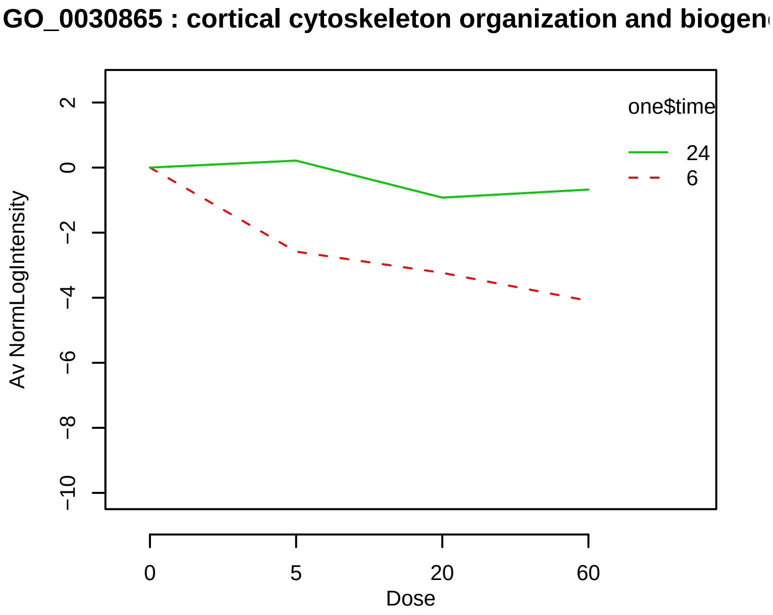
GO_0030865 : cortical cytoskeleton organization and biogen
Av NormLogIntensity
Dose
one$time
24
6
2
0
−2
−4
−6
−8
−10
0
5
20
60

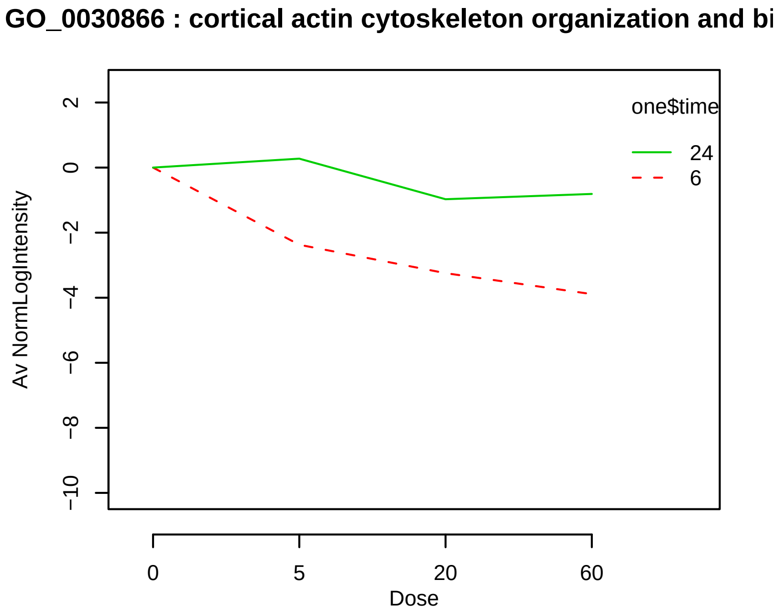
GO_0030866 : cortical actin cytoskeleton organization and bi
one$time
24
6
Av NormLogIntensity
Dose
0
5
20
60

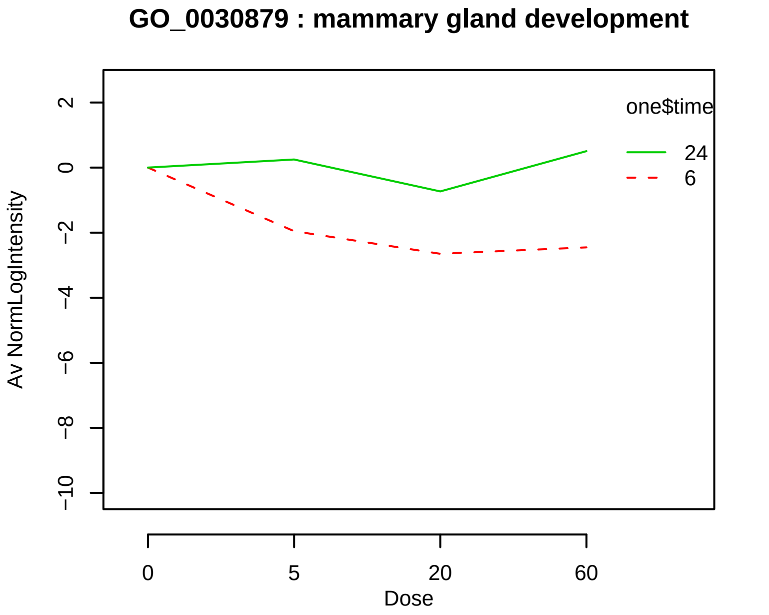
GO_0030879 : mammary gland development
one$time
24
6
Av NormLogIntensity
Dose
0
5
20
60

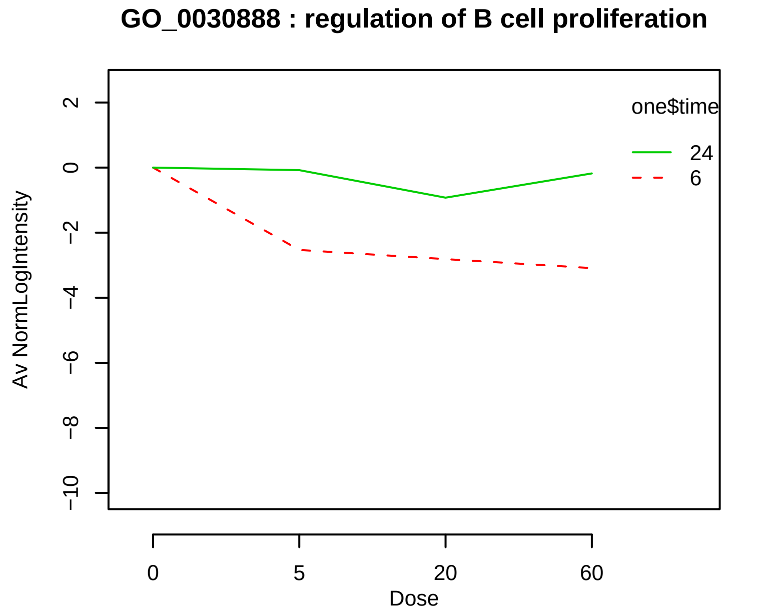
GO_0030888 : regulation of B cell proliferation
one$time
24
6
Av NormLogIntensity
Dose
0
5
20
60

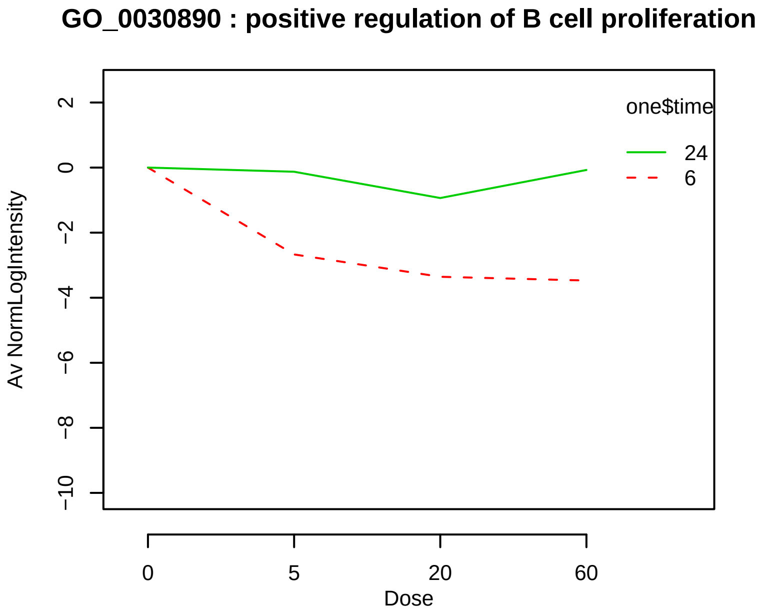
GO_0030890 : positive regulation of B cell proliferation
one$time
24
6
Av NormLogIntensity
Dose
0
5
20
60

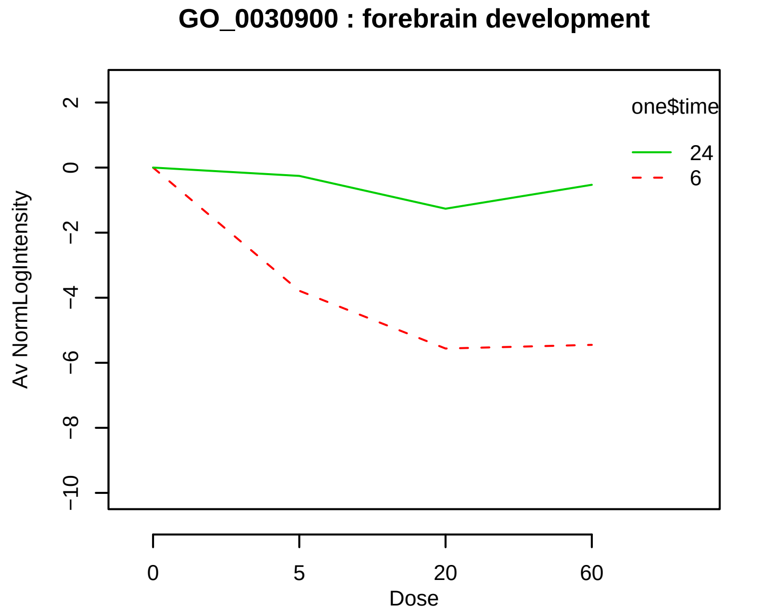
GO_0030900 : forebrain development
one$time
24
6
Av NormLogIntensity
Dose
0
5
20
60

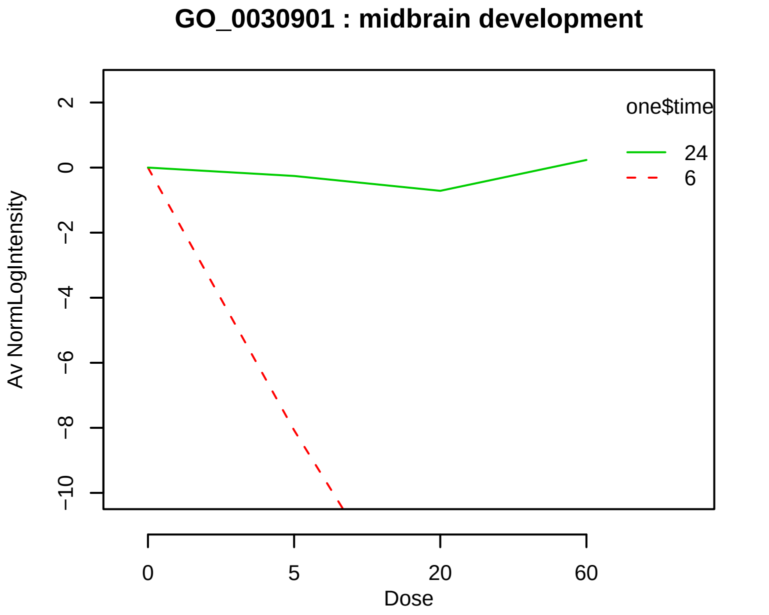
GO_0030901 : midbrain development
one$time
24
6
Av NormLogIntensity
Dose
0
5
20
60

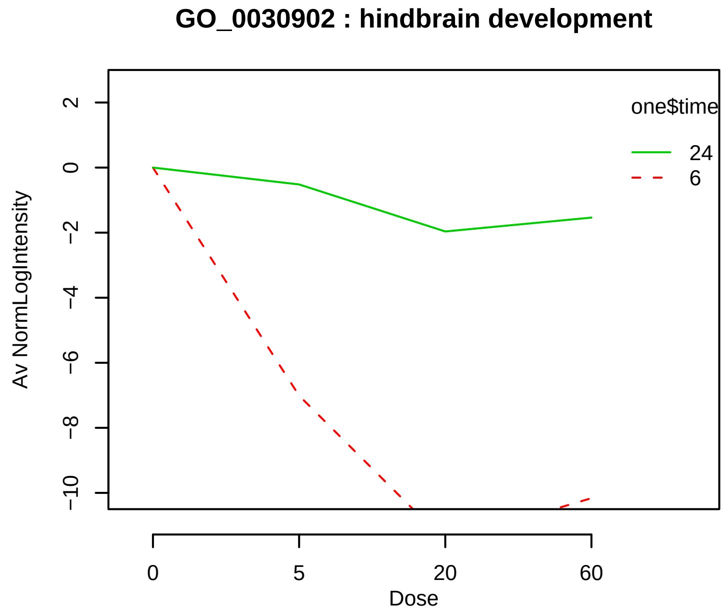
GO_0030902 : hindbrain development
Av NormLogIntensity
Dose
one$time
24
6

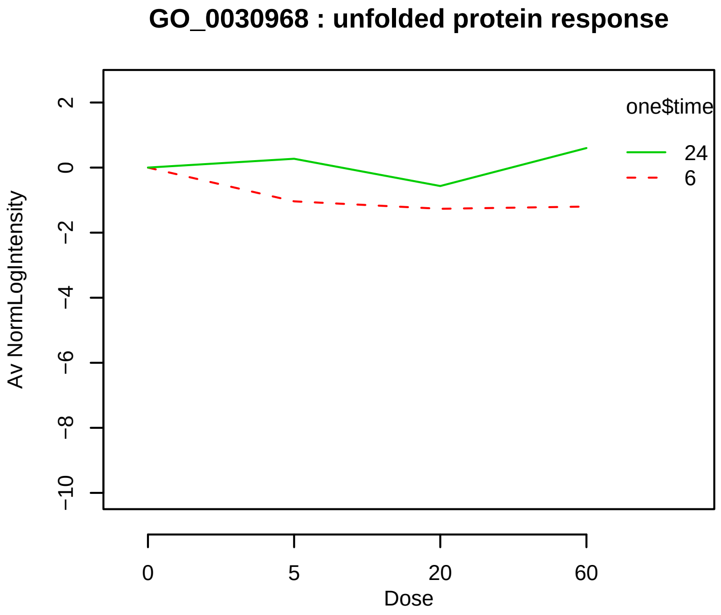
GO_0030968 : unfolded protein response
Av NormLogIntensity
Dose
one$time
24
6

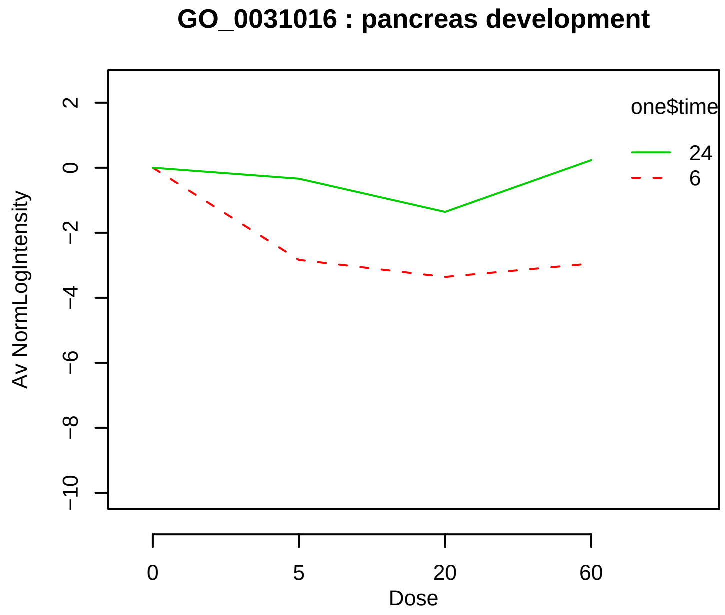
GO_0031016 : pancreas development
Av NormLogIntensity
Dose
one$time
24
6

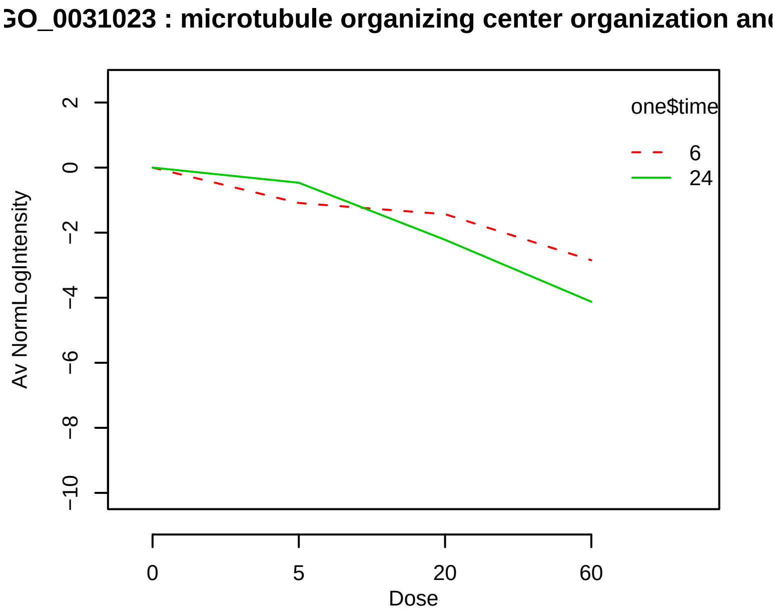
GO_0031023 : microtubule organizing center organization and
Av NormLogIntensity
Dose
one$time
6
24

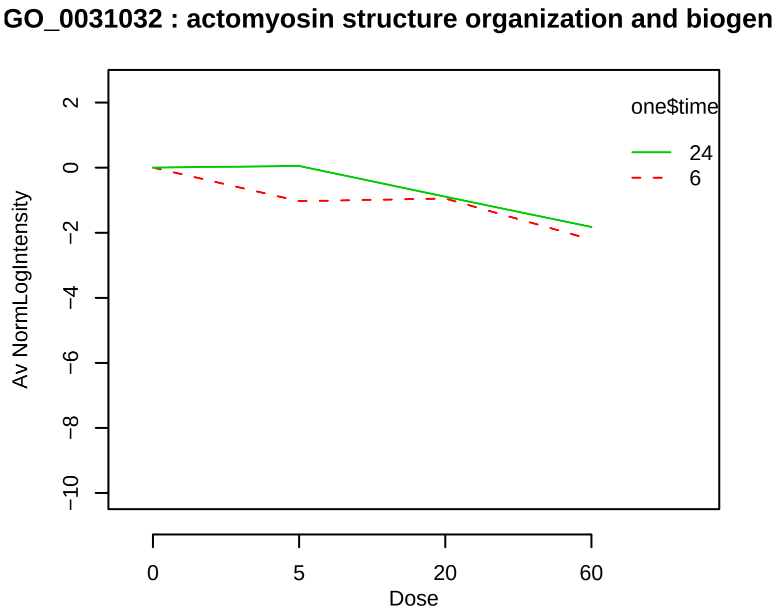
GO_0031032 : actomyosin structure organization and biogen
Av NormLogIntensity
Dose
one$time
24
6

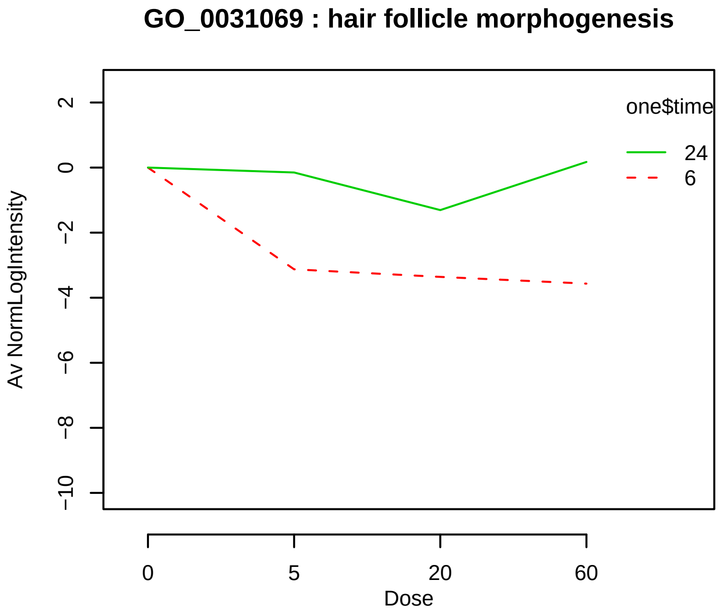
GO_0031069 : hair follicle morphogenesis
Av NormLogIntensity
Dose
one$time
24
6

GO_0031098 : stress−activated protein kinase signaling path

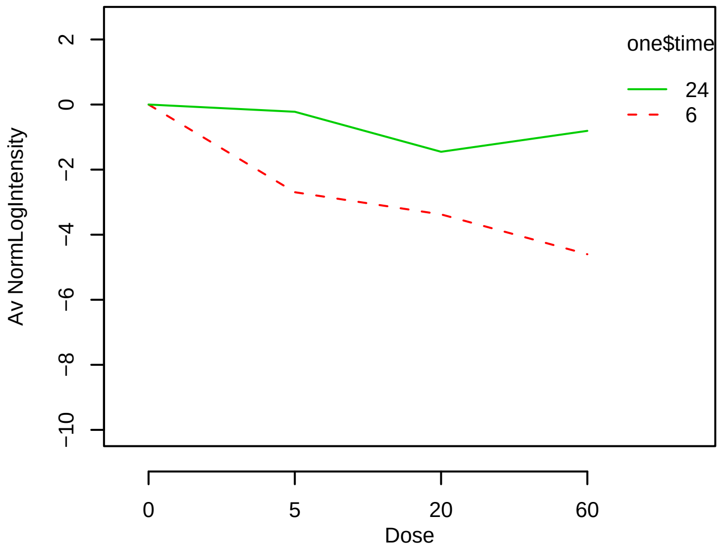

GO_0031099 : regeneration

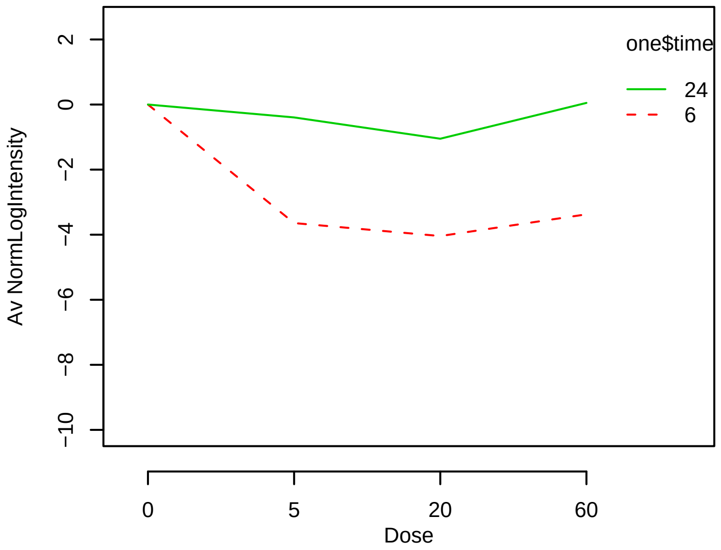

GO_0031109 : microtubule polymerization or depolymerizat

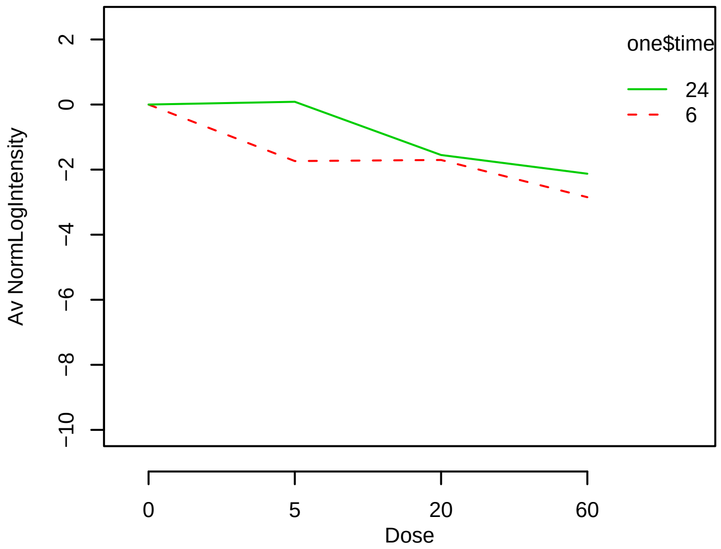

GO_0031110 : regulation of microtubule polymerization or de

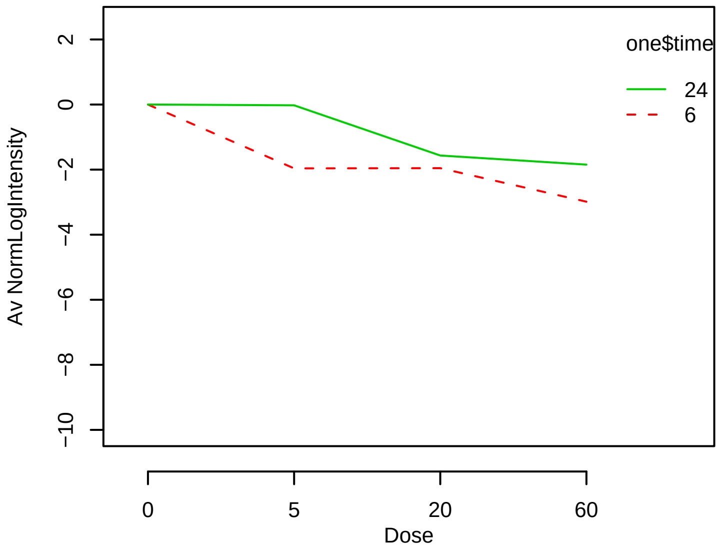

GO_0031111 : negative regulation of microtubule polymeriza

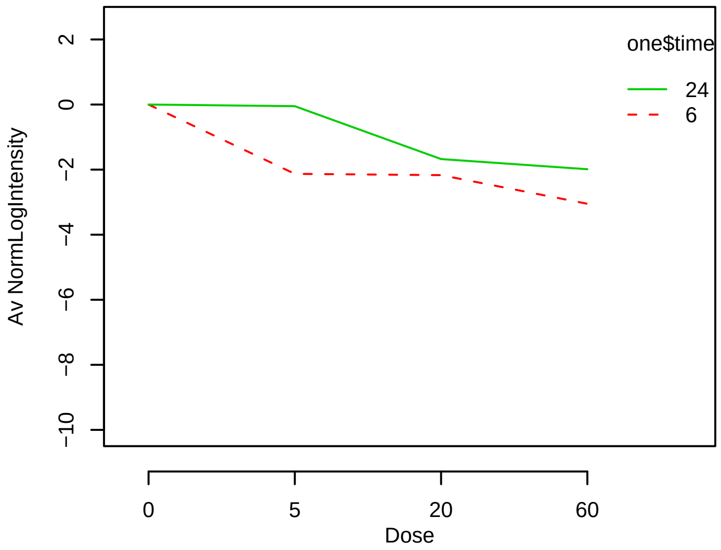

GO_0031114 : regulation of microtubule depolymerizatior

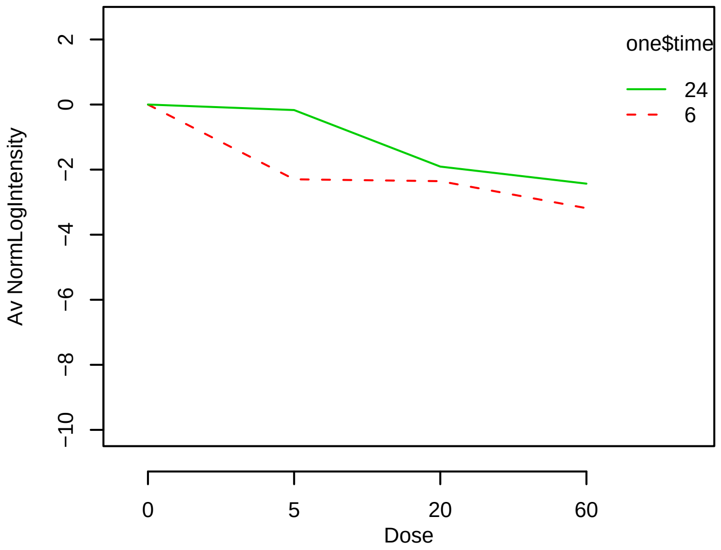

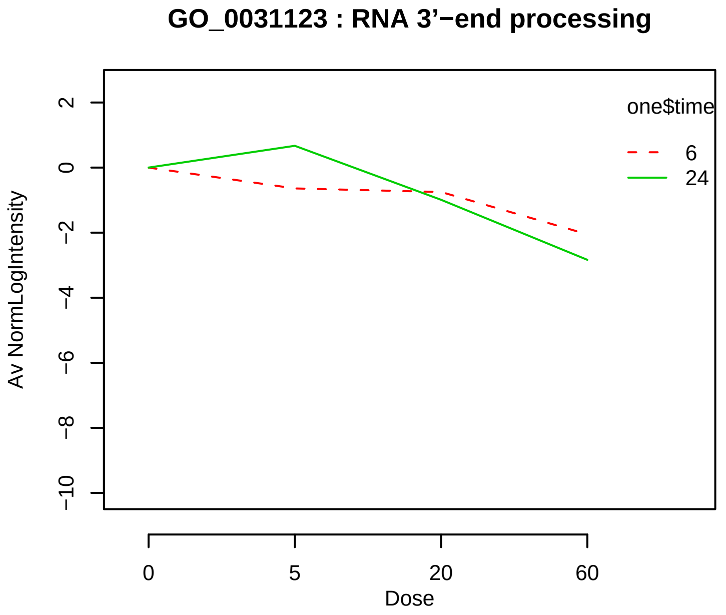
GO_0031123 : RNA 3'−end processing
one$time
6
24
Av NormLogIntensity
Dose
0
5
20
60

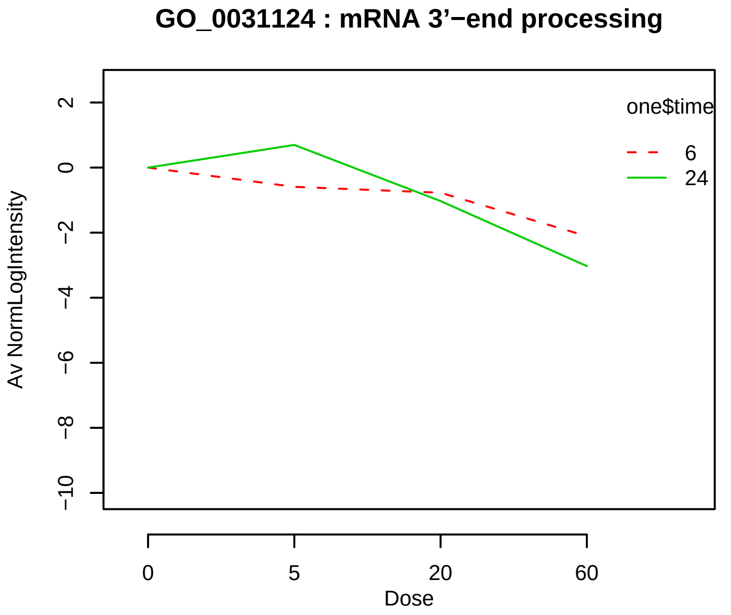
GO_0031124 : mRNA 3'−end processing
one$time
6
24
Av NormLogIntensity
Dose

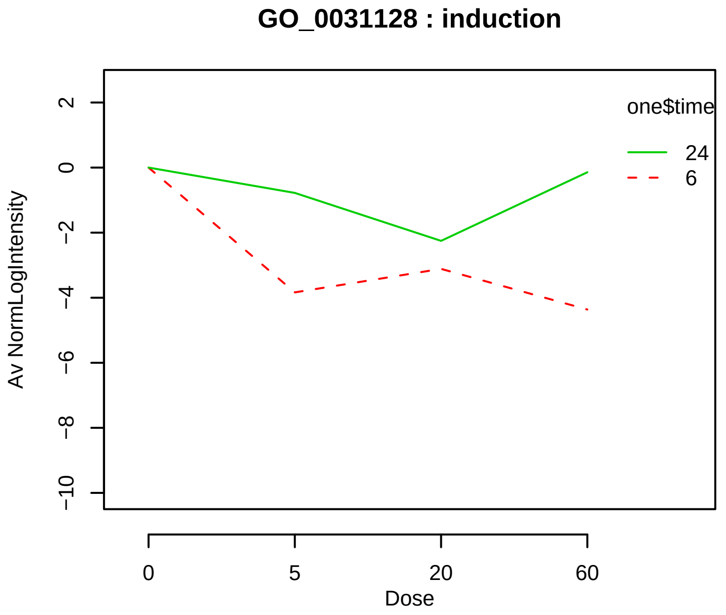
GO_0031128 : induction
one$time
24
6
Av NormLogIntensity
Dose

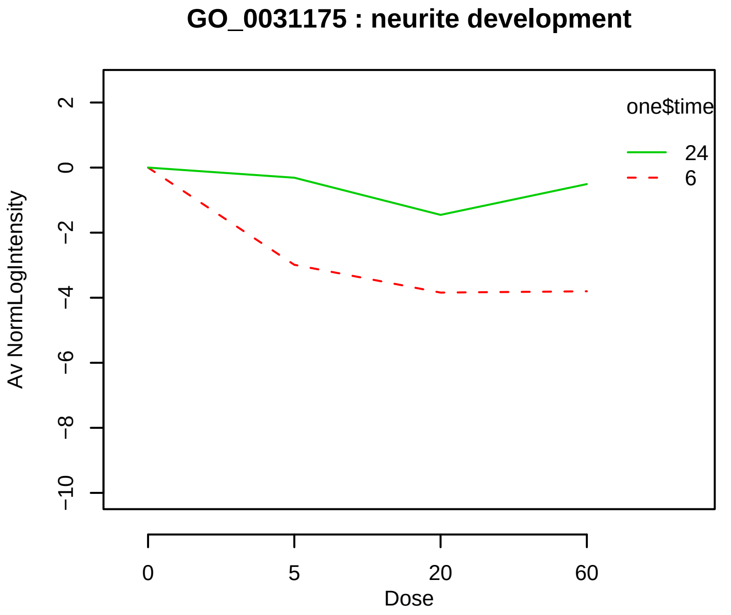
GO_0031175 : neurite development
one$time
24
6
Av NormLogIntensity
Dose

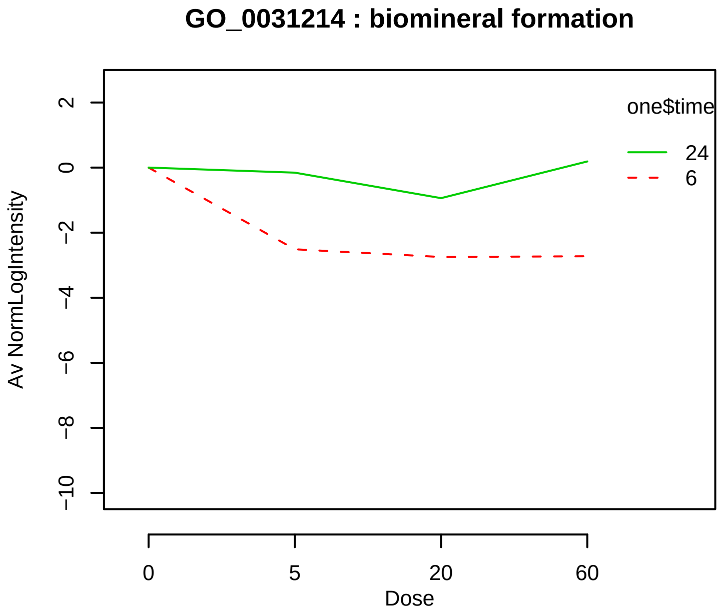
GO_0031214 : biomineral formation
one$time
24
6
Av NormLogIntensity
Dose

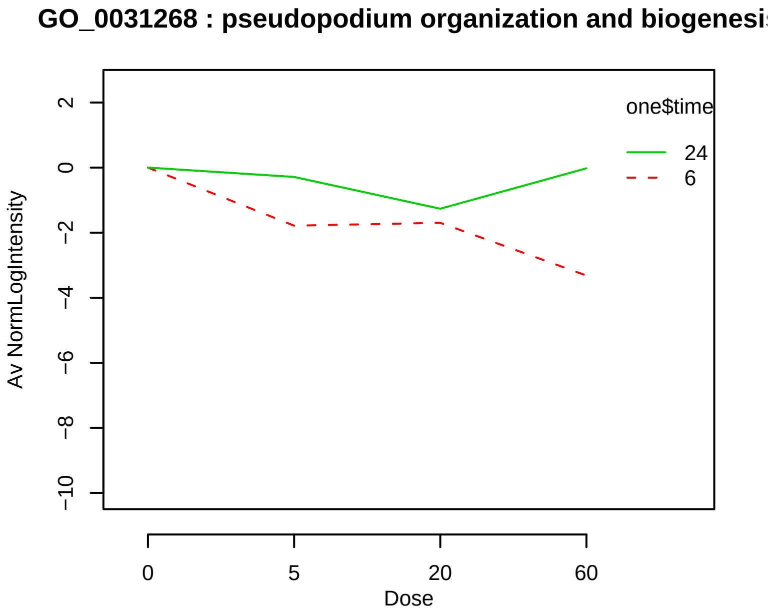
GO_0031268 : pseudopodium organization and biogenesi
one$time
24
6
Av NormLogIntensity
Dose

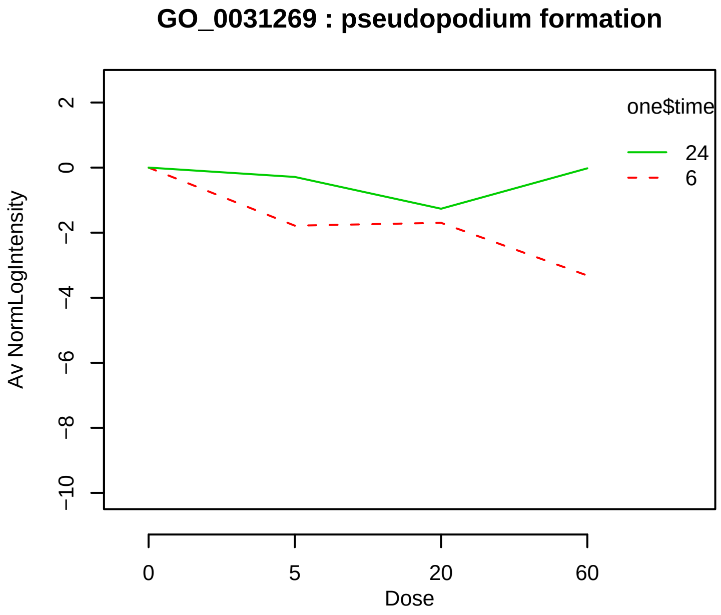
GO_0031269 : pseudopodium formation
Av NormLogIntensity
Dose
one$time
24
6

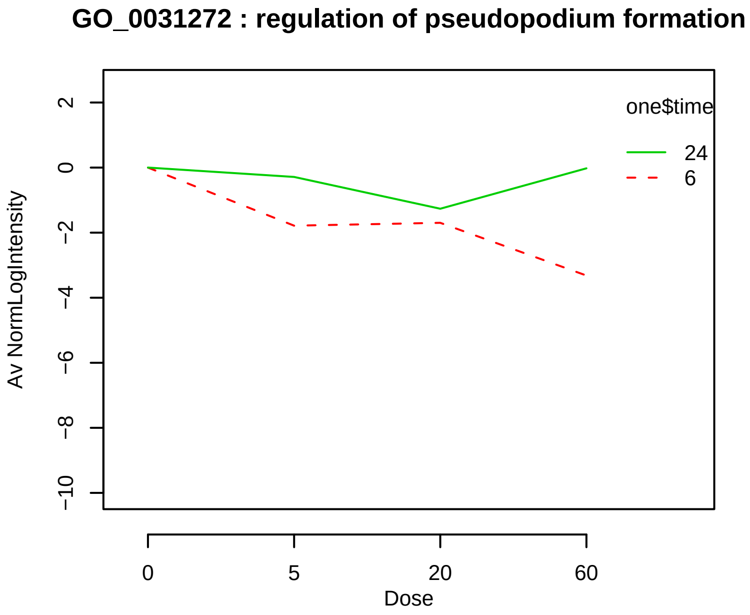
GO_0031272 : regulation of pseudopodium formation
Av NormLogIntensity
Dose
one$time
24
6

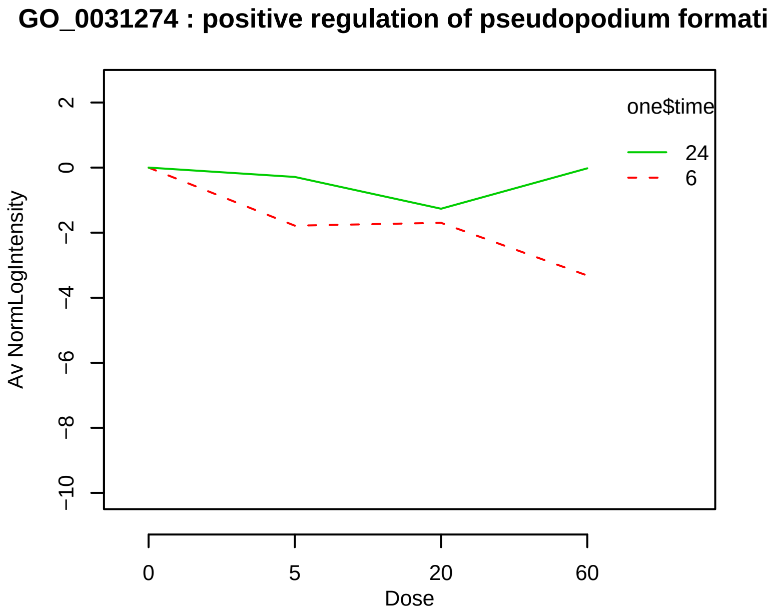
GO_0031274 : positive regulation of pseudopodium formati
Av NormLogIntensity
Dose
one$time
24
6

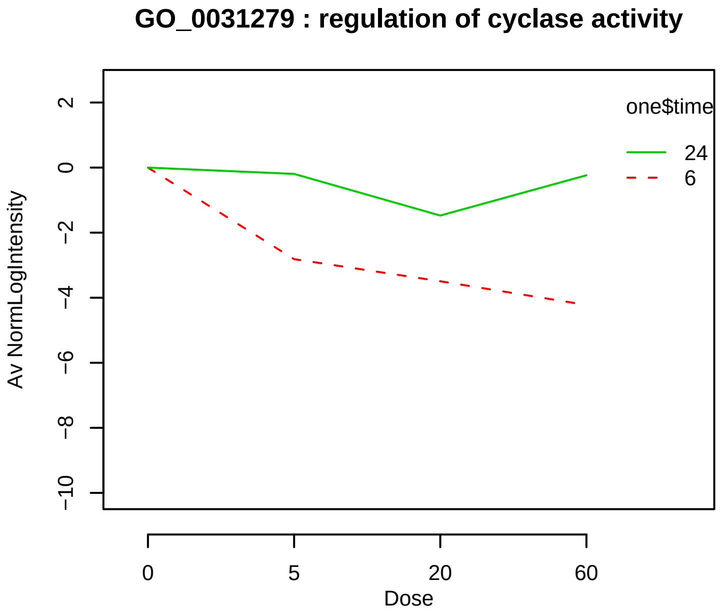
GO_0031279 : regulation of cyclase activity
Av NormLogIntensity
Dose
one$time
24
6

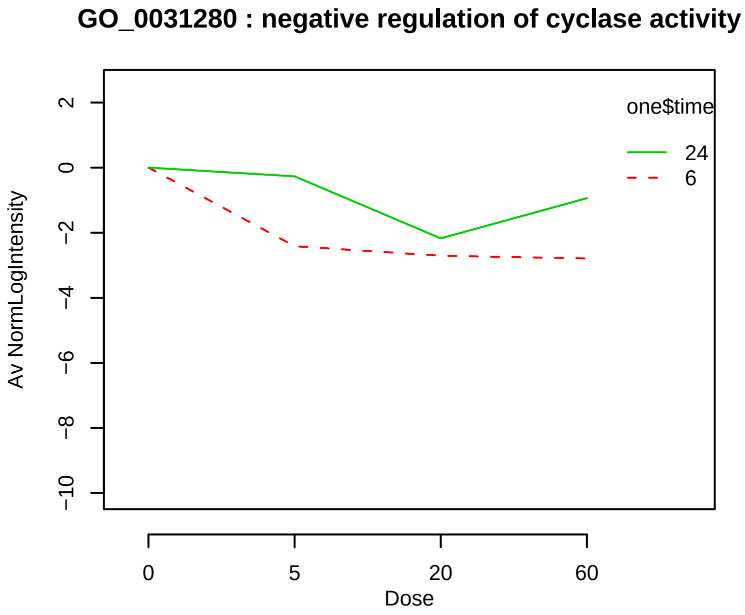
GO_0031280 : negative regulation of cyclase activity
Av NormLogIntensity
Dose
one$time
24
6

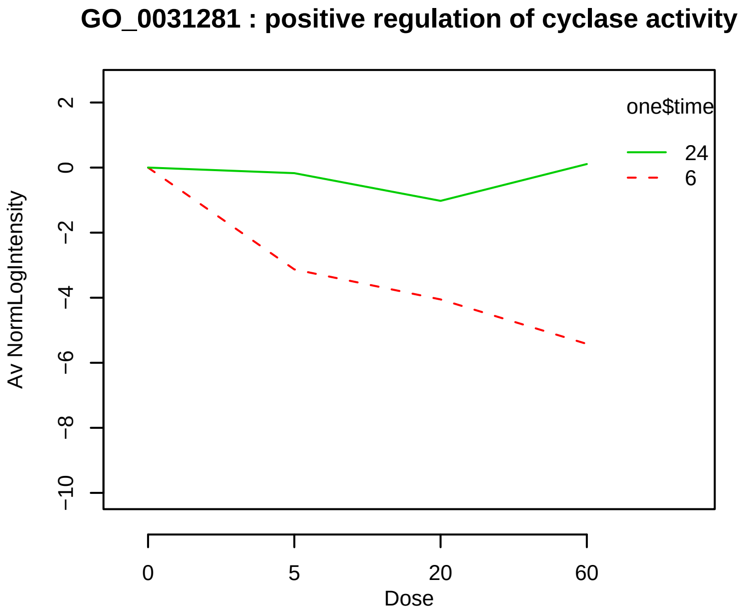
GO_0031281 : positive regulation of cyclase activity
Av NormLogIntensity
Dose
one$time
24
6

GO_0031326 : regulation of cellular biosynthesis

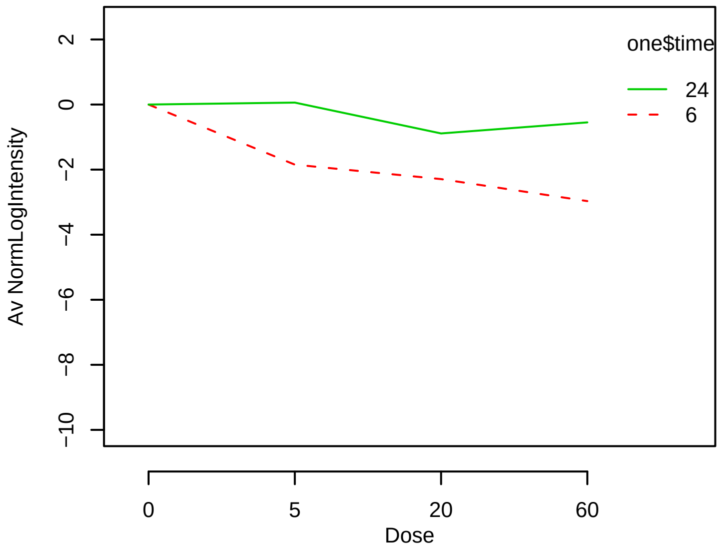

GO_0031327 : negative regulation of cellular biosynthesis

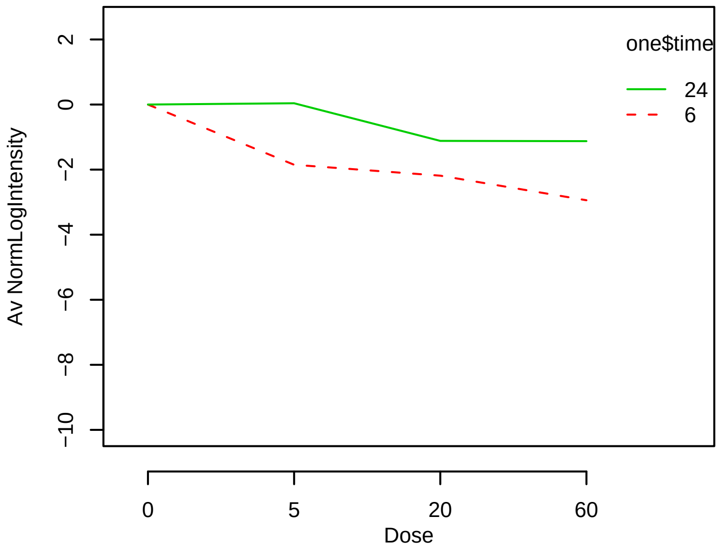

GO_0031328 : positive regulation of cellular biosynthesis

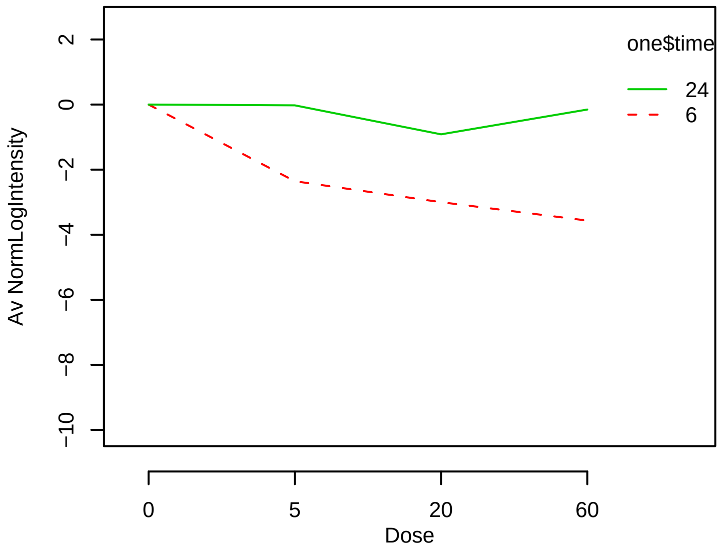

GO_0031344 : regulation of cell projection organization and

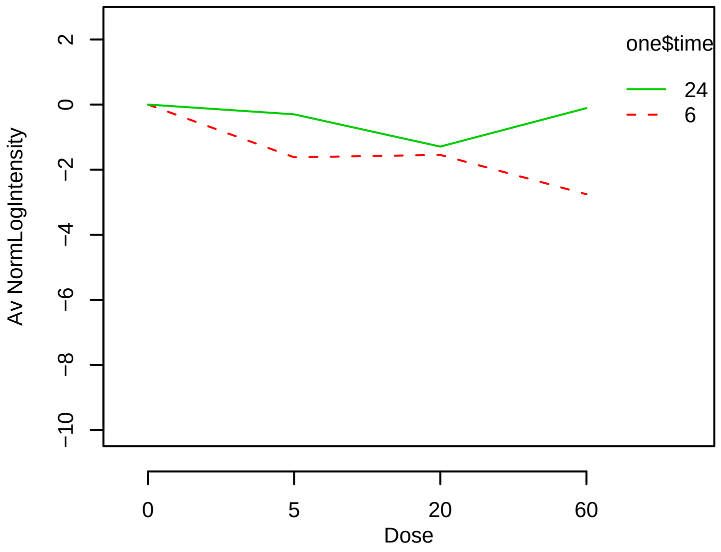

GO_0031346 : positive regulation of cell projection organiza

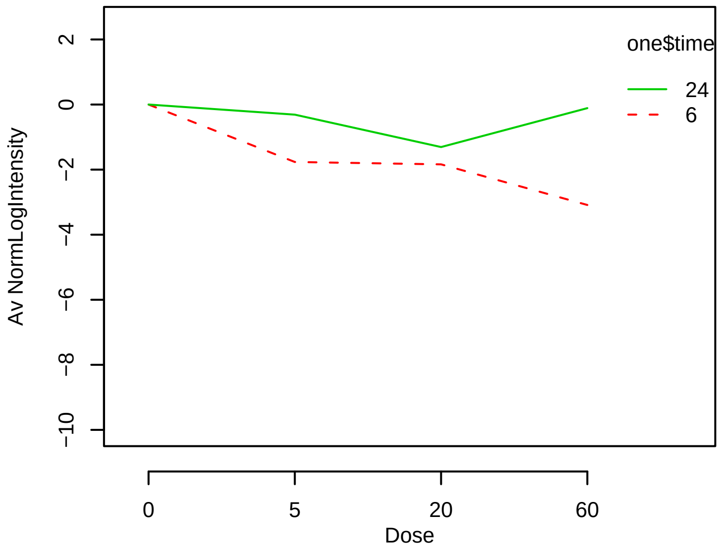

GO_0031365 : N−terminal protein amino acid modificatior

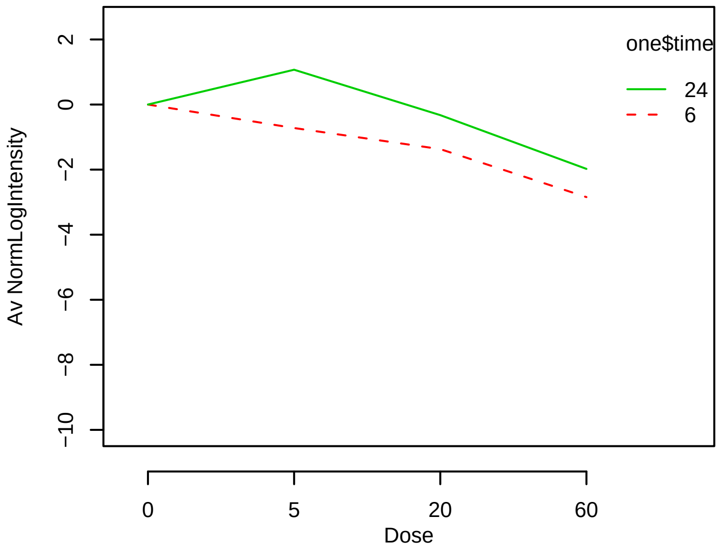

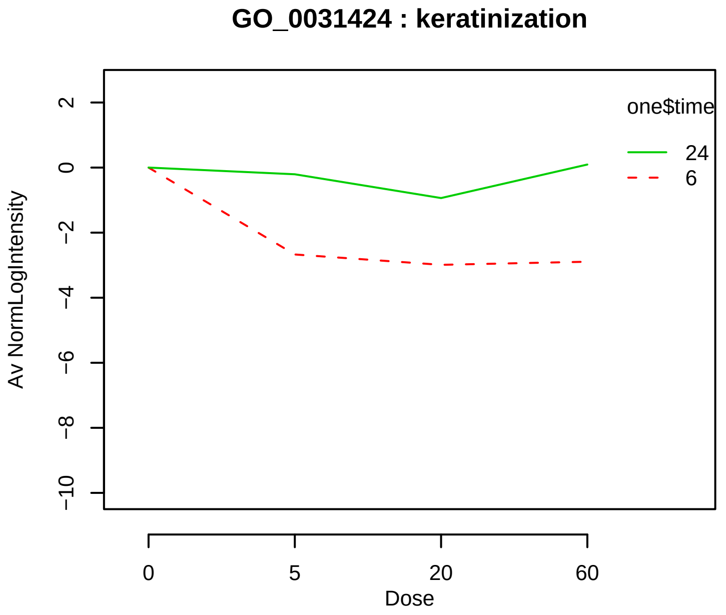
GO_0031424 : keratinization
one$time
24
6
Av NormLogIntensity
Dose
0
5
20
60
2
0
−2
−4
−6
−8
−10

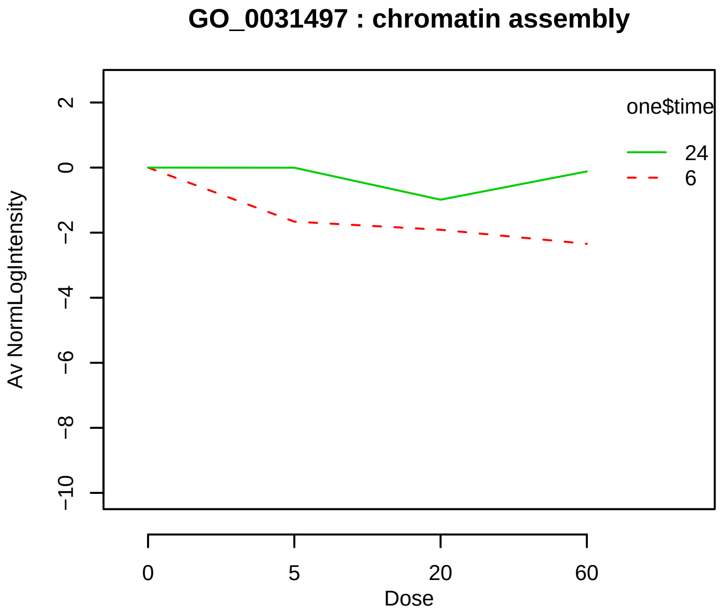
GO_0031497 : chromatin assembly
one$time
24
6
Av NormLogIntensity
Dose
0
5
20
60
2
0
−2
−4
−6
−8
−10

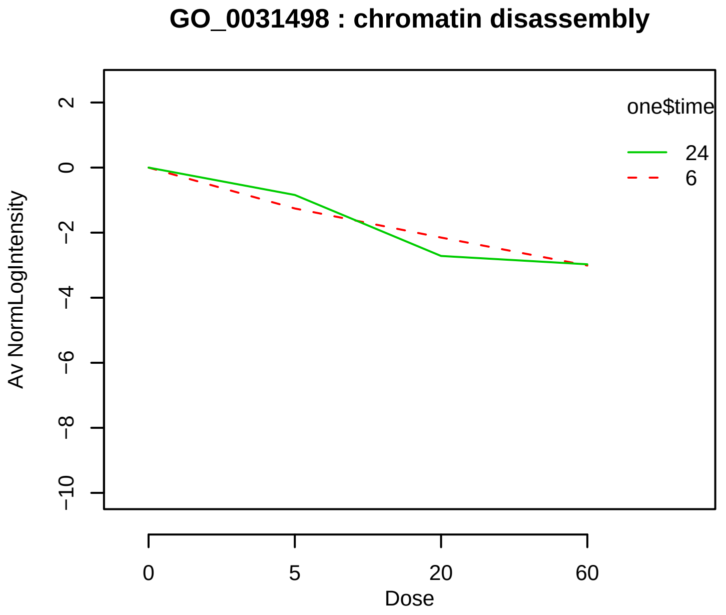
GO_0031498 : chromatin disassembly
one$time
24
6
Av NormLogIntensity
Dose
0
5
20
60
2
0
−2
−4
−6
−8
−10

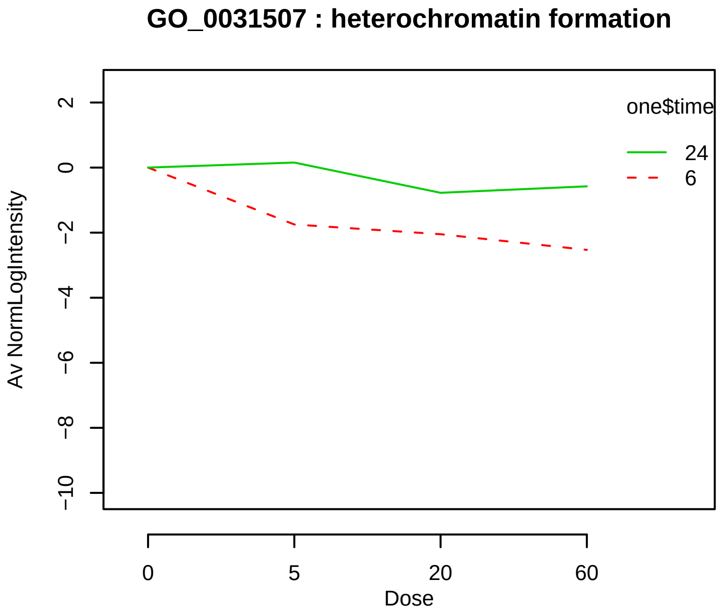
GO_0031507 : heterochromatin formation
one$time
24
6
Av NormLogIntensity
Dose
0
5
20
60
2
0
−2
−4
−6
−8
−10

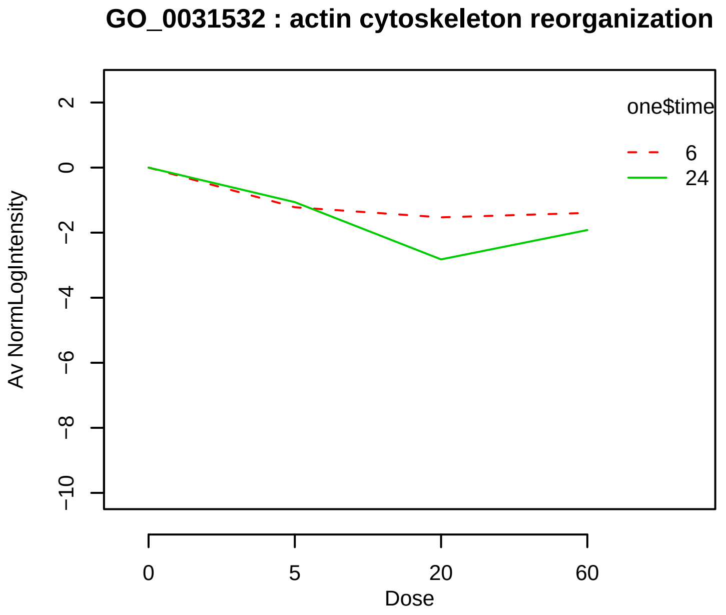
GO_0031532 : actin cytoskeleton reorganization
one$time
6
24
Av NormLogIntensity
Dose
0
5
20
60
2
0
−2
−4
−6
−8
−10

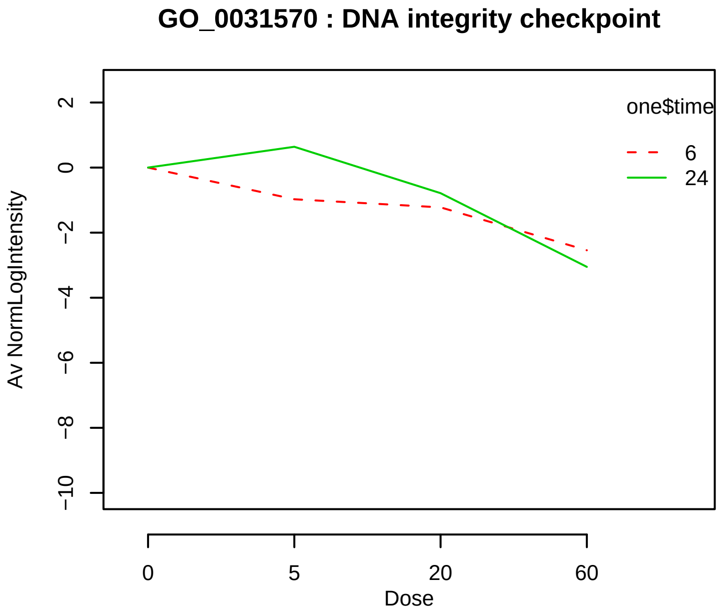
GO_0031570 : DNA integrity checkpoint
one$time
6
24
Av NormLogIntensity
Dose
0
5
20
60
2
0
−2
−4
−6
−8
−10

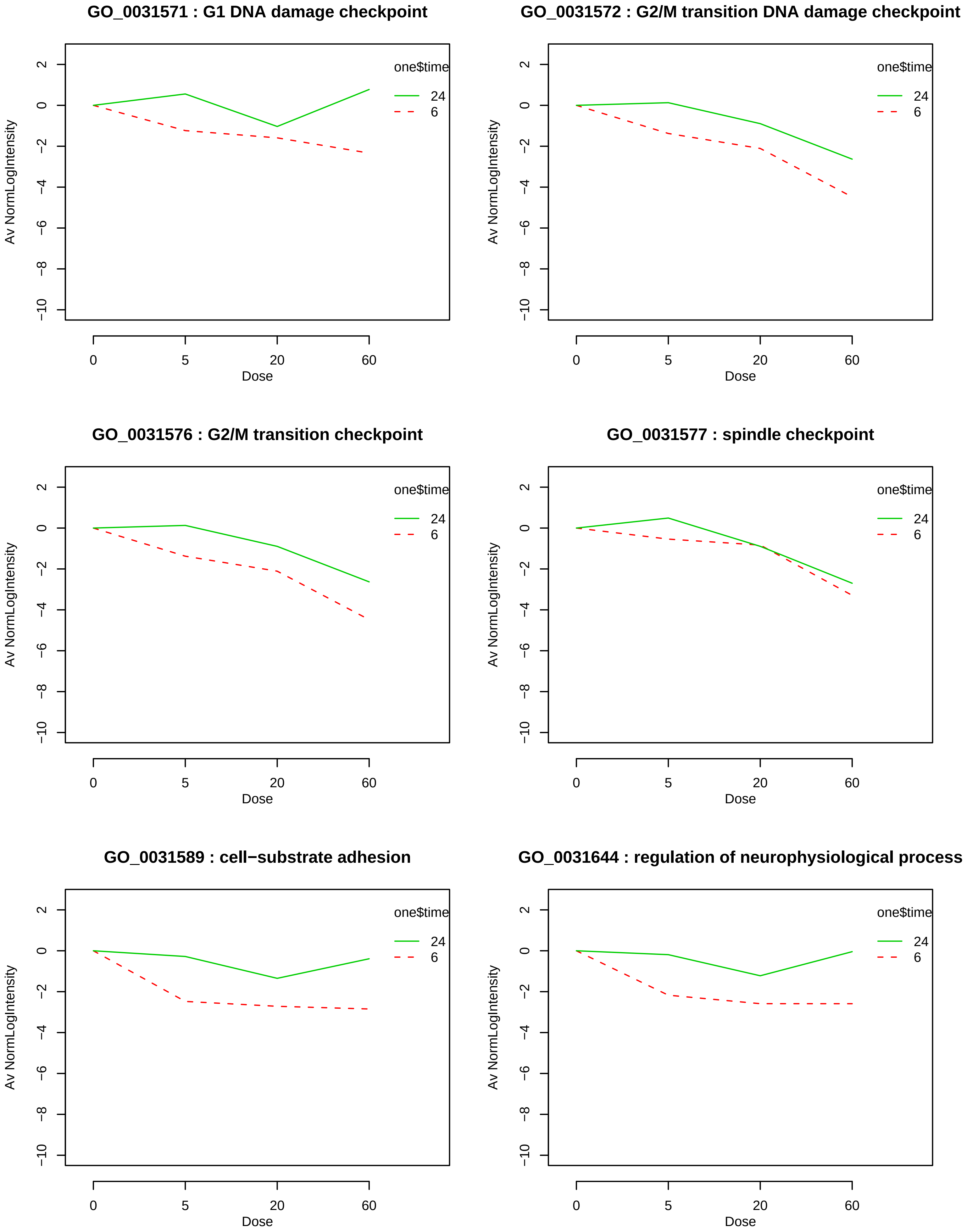
GO_0031571 : G1 DNA damage checkpoint
GO_0031572 : G2/M transition DNA damage checkpoint
GO_0031576 : G2/M transition checkpoint
GO_0031577 : spindle checkpoint
GO_0031589 : cell−substrate adhesion
GO_0031644 : regulation of neurophysiological process
Av NormLogIntensity
Dose
one$time
24
6
0
5
20
60
2
0
−2
−4
−6
−8
−10

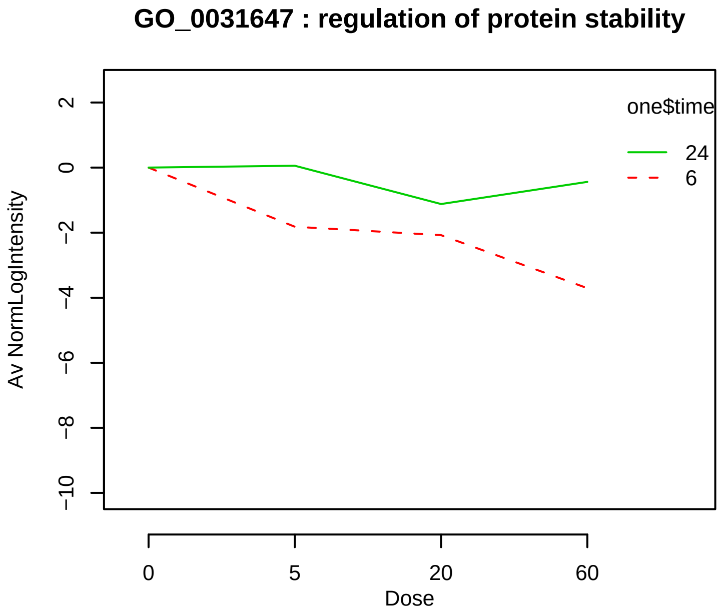
GO_0031647 : regulation of protein stability
Av NormLogIntensity
2
0
−2
−4
−6
−8
−10
0
5
20
60
Dose
one$time
24
6

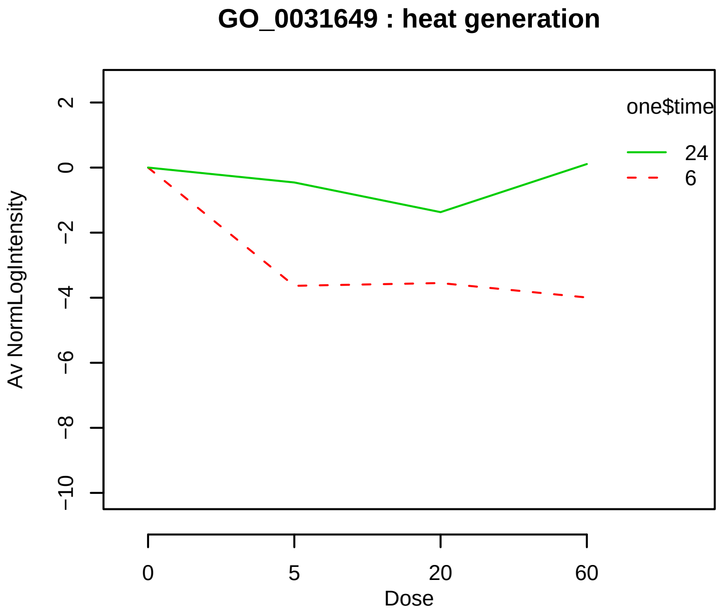
GO_0031649 : heat generation
Av NormLogIntensity
2
0
−2
−4
−6
−8
−10
0
5
20
60
Dose
one$time
24
6

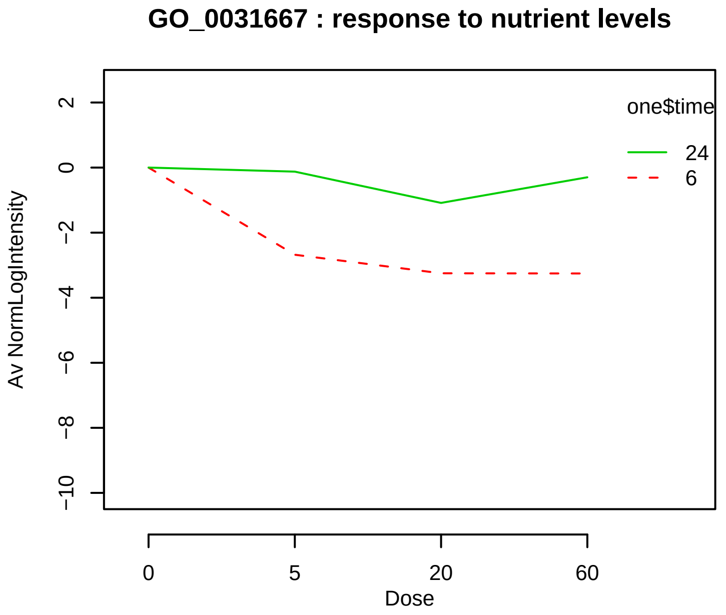
GO_0031667 : response to nutrient levels
Av NormLogIntensity
2
0
−2
−4
−6
−8
−10
0
5
20
60
Dose
one$time
24
6

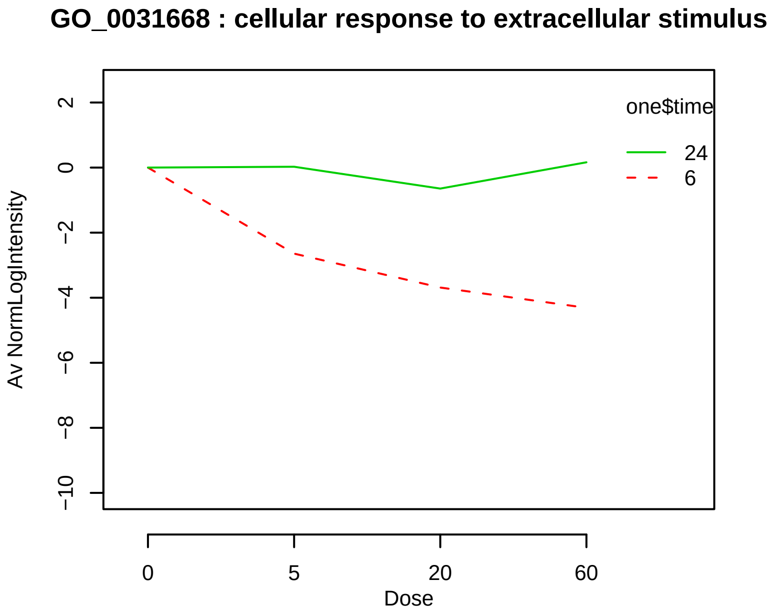
GO_0031668 : cellular response to extracellular stimulus
Av NormLogIntensity
2
0
−2
−4
−6
−8
−10
0
5
20
60
Dose
one$time
24
6

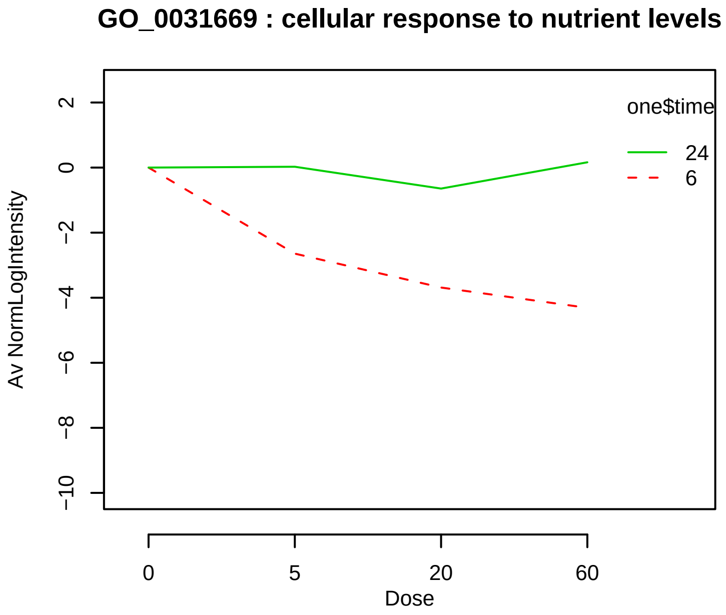
GO_0031669 : cellular response to nutrient levels
Av NormLogIntensity
2
0
−2
−4
−6
−8
−10
0
5
20
60
Dose
one$time
24
6

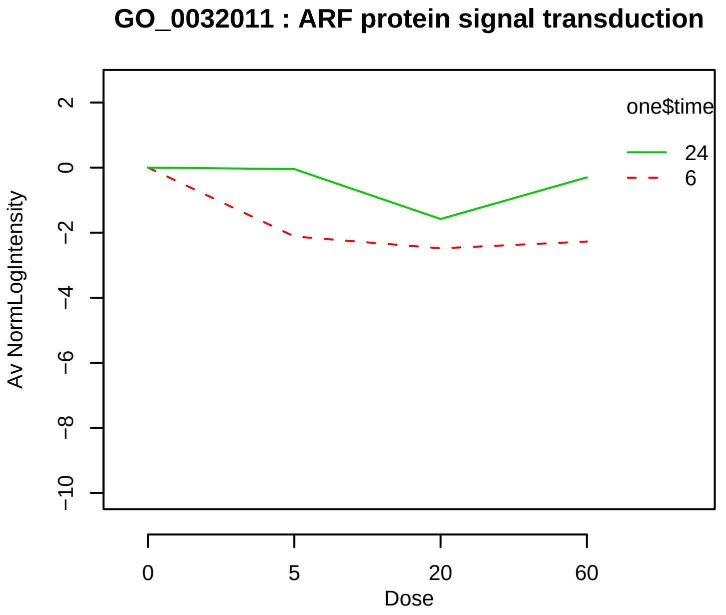
GO_0032011 : ARF protein signal transduction
Av NormLogIntensity
2
0
−2
−4
−6
−8
−10
0
5
20
60
Dose
one$time
24
6

GO_0032012 : regulation of ARF protein signal transductio

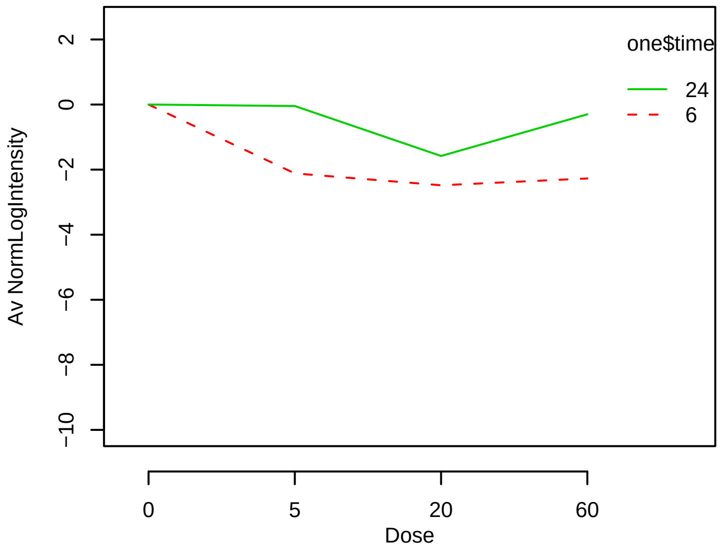

GO_0032147 : activation of protein kinase activity

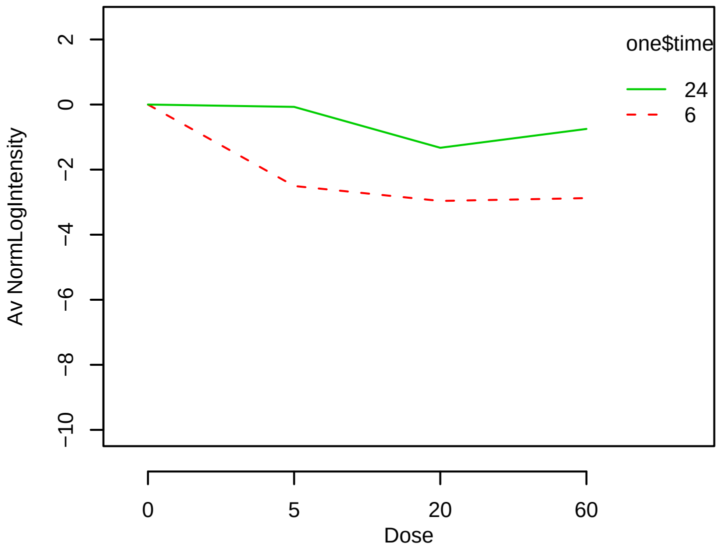

GO_0032200 : telomere organization and biogenesis

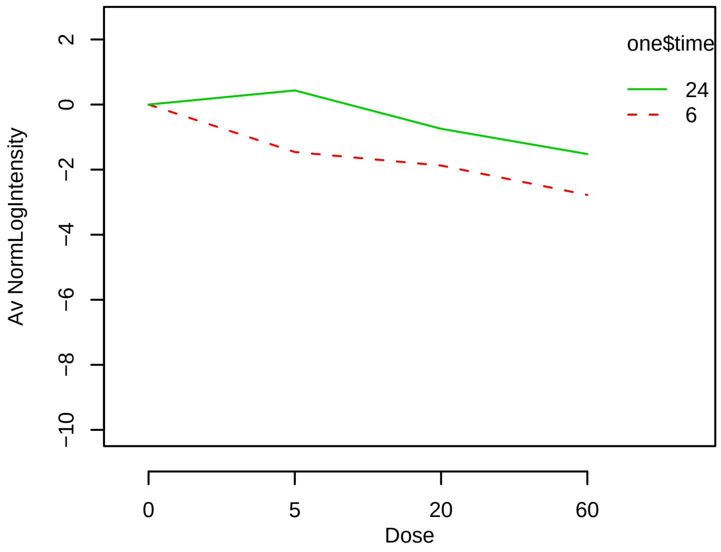

GO_0035023 : regulation of Rho protein signal transductio

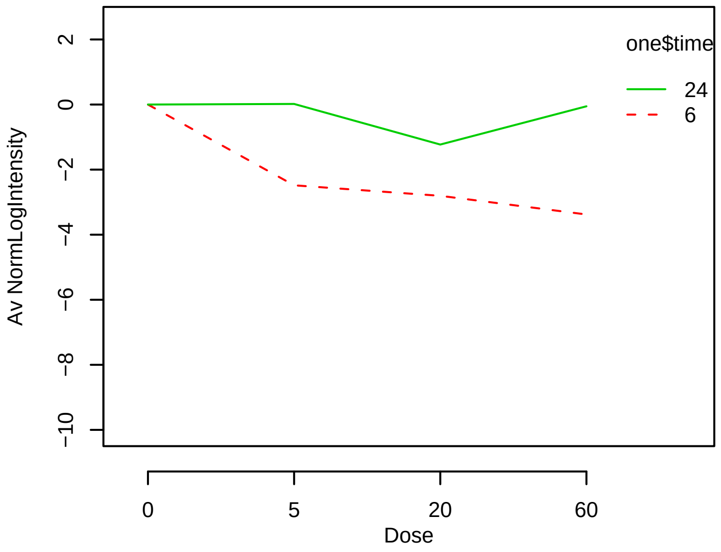

GO_0035036 : sperm−egg recognition

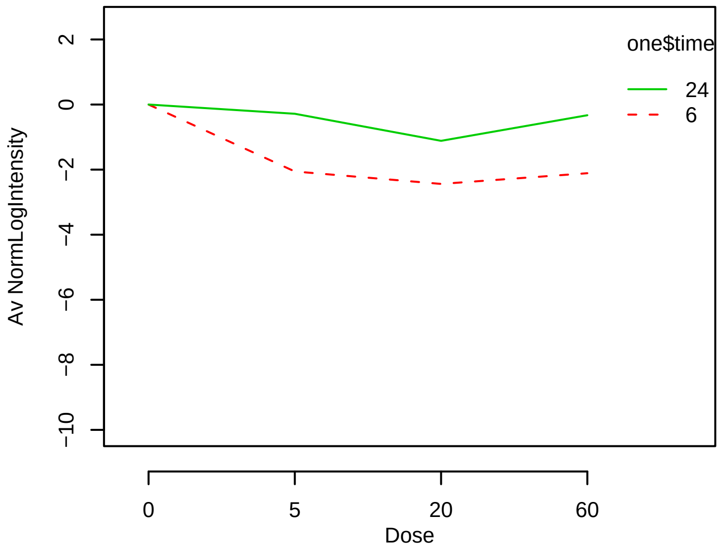

GO_0035050 : embryonic heart tube development

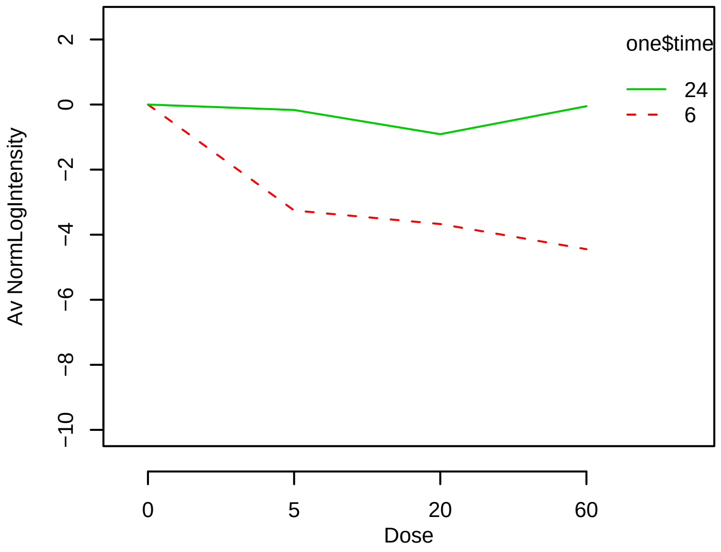

GO_0035051 : cardiac cell differentiation

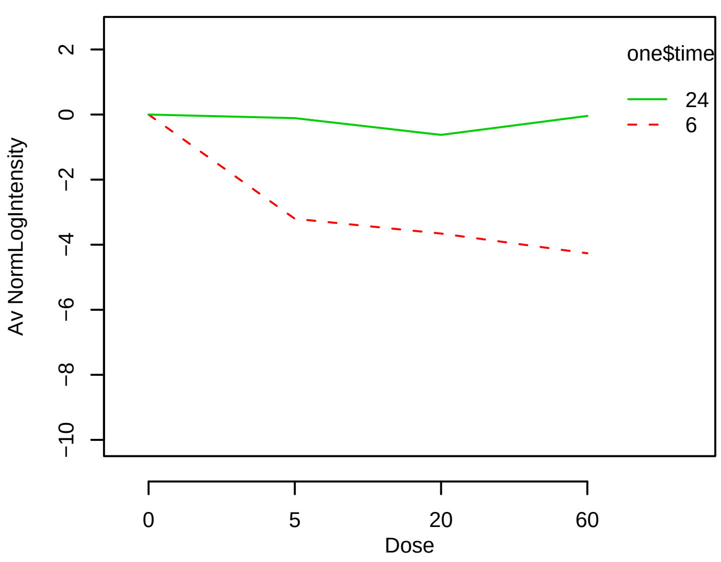

GO_0035058 : sensory cilium biogenesis

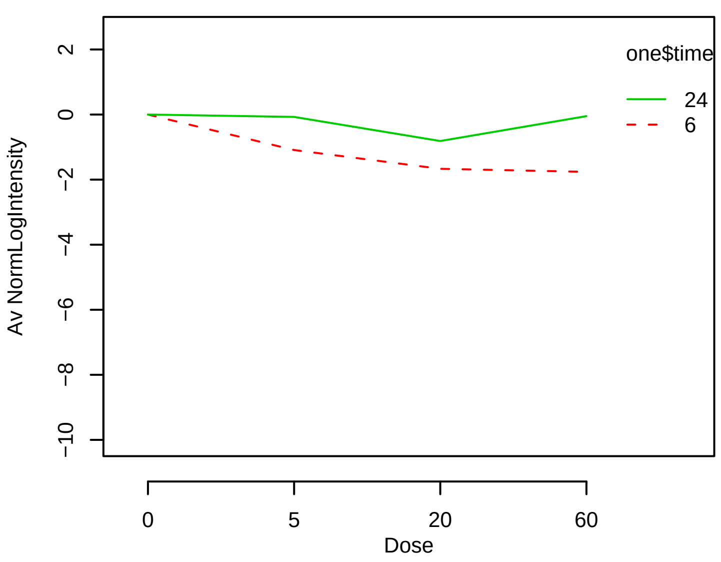

GO_0035088 : establishment and/or maintenance of apical/ba

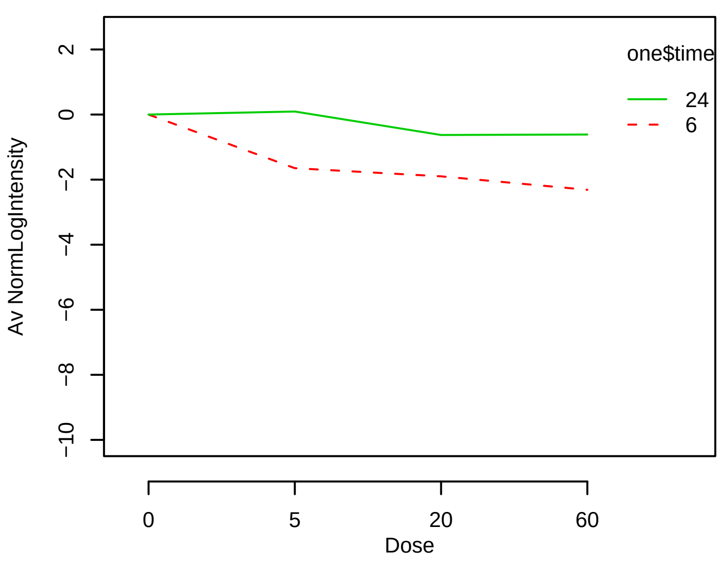

GO_0035107 : appendage morphogenesis

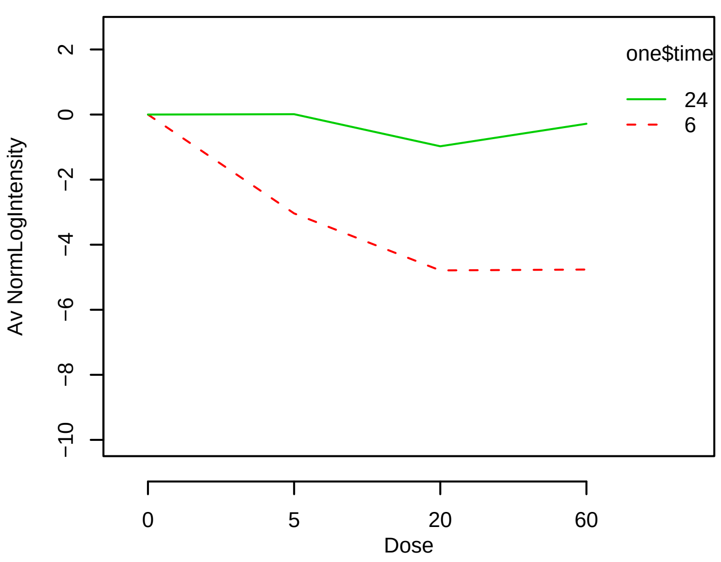

GO_0035108 : limb morphogenesis

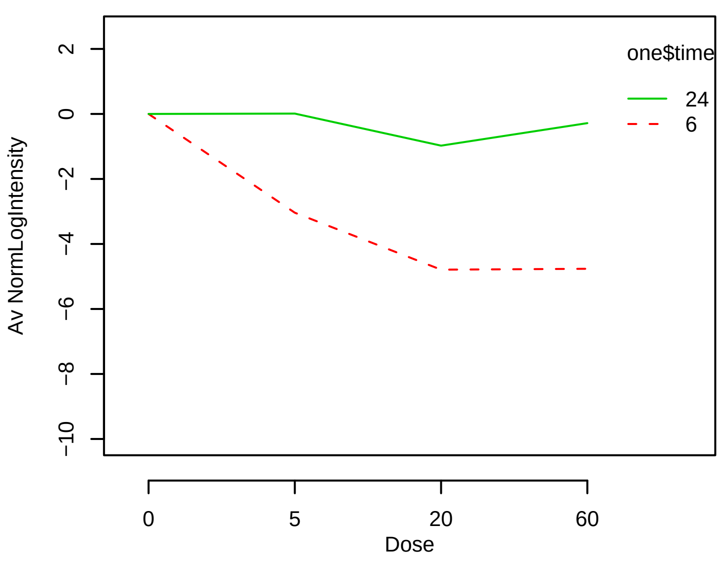

GO_0035113 : embryonic appendage morphogenesis

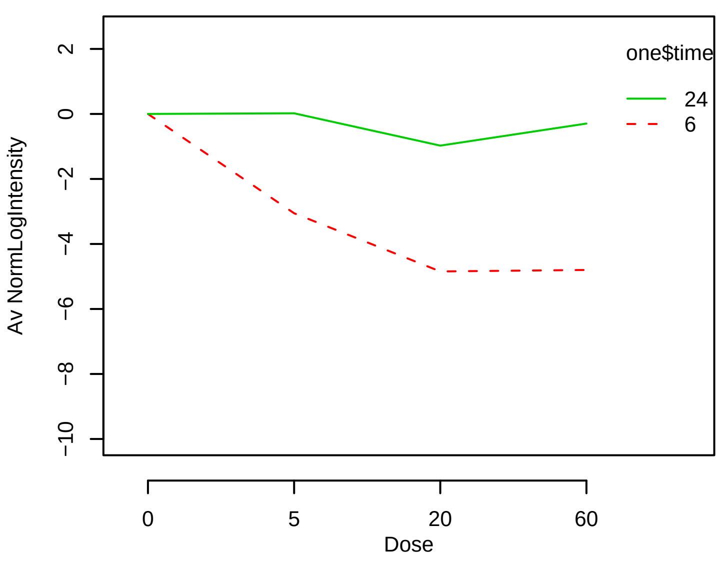

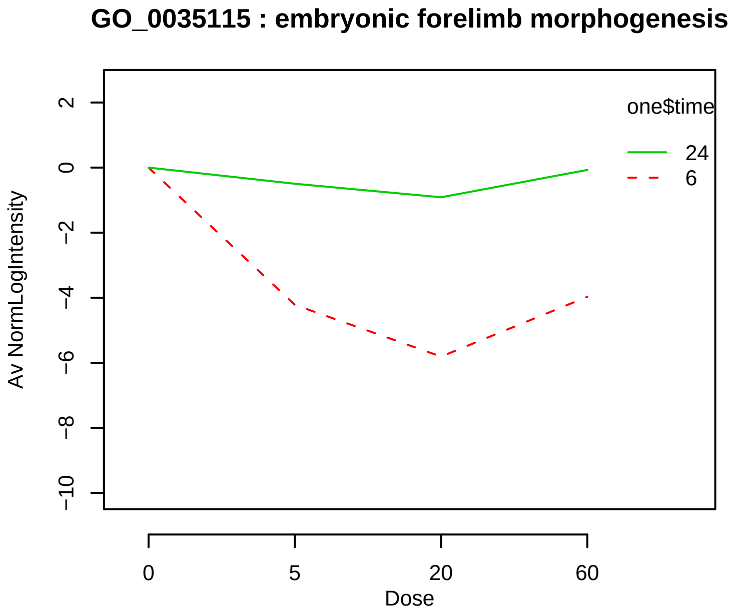
GO_0035115 : embryonic forelimb morphogenesis
one$time
24
6
Av NormLogIntensity
2
0
−2
−4
−6
−8
−10
0
5
20
60
Dose

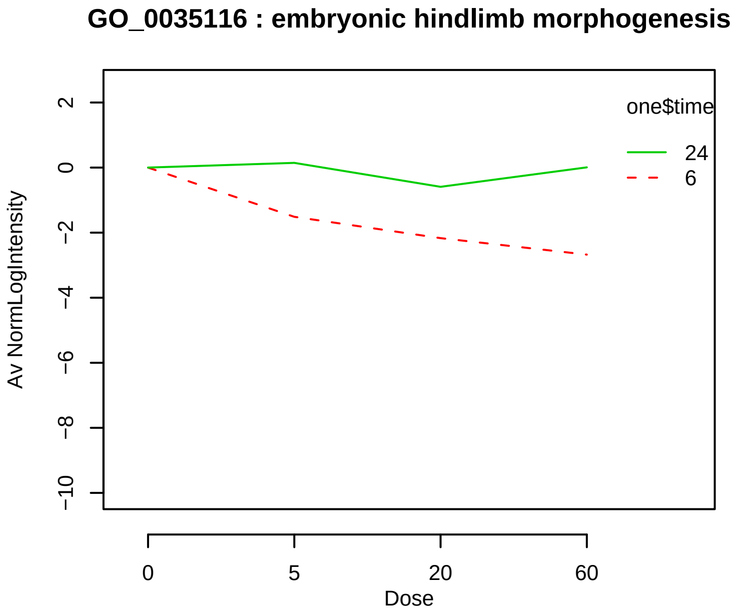
GO_0035116 : embryonic hindlimb morphogenesis
one$time
24
6
Av NormLogIntensity
2
0
−2
−4
−6
−8
−10
0
5
20
60
Dose

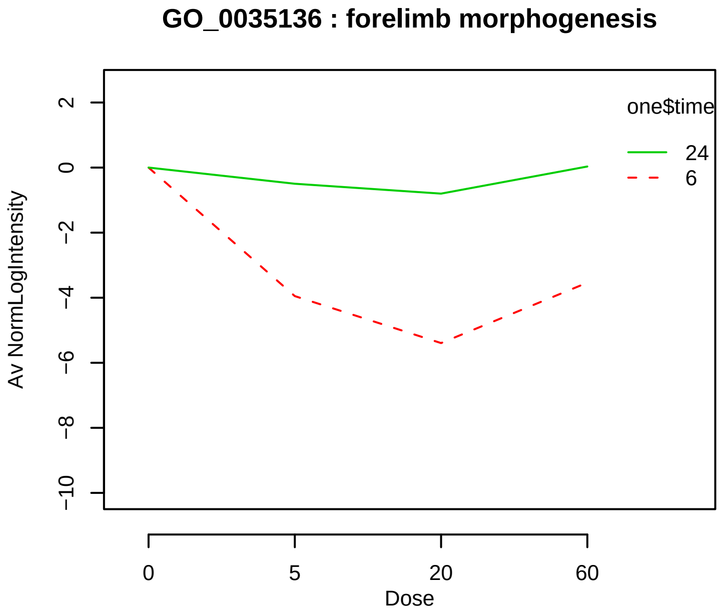
GO_0035136 : forelimb morphogenesis
one$time
24
6
Av NormLogIntensity
2
0
−2
−4
−6
−8
−10
0
5
20
60
Dose

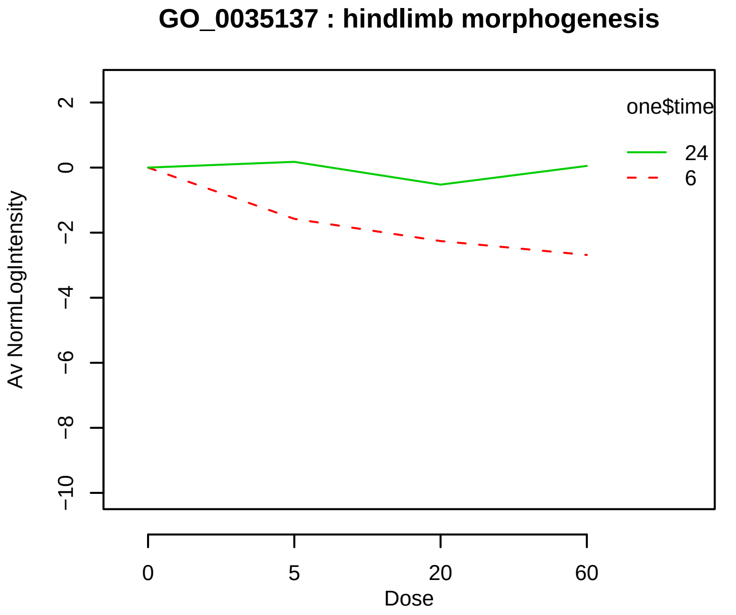
GO_0035137 : hindlimb morphogenesis
one$time
24
6
Av NormLogIntensity
2
0
−2
−4
−6
−8
−10
0
5
20
60
Dose

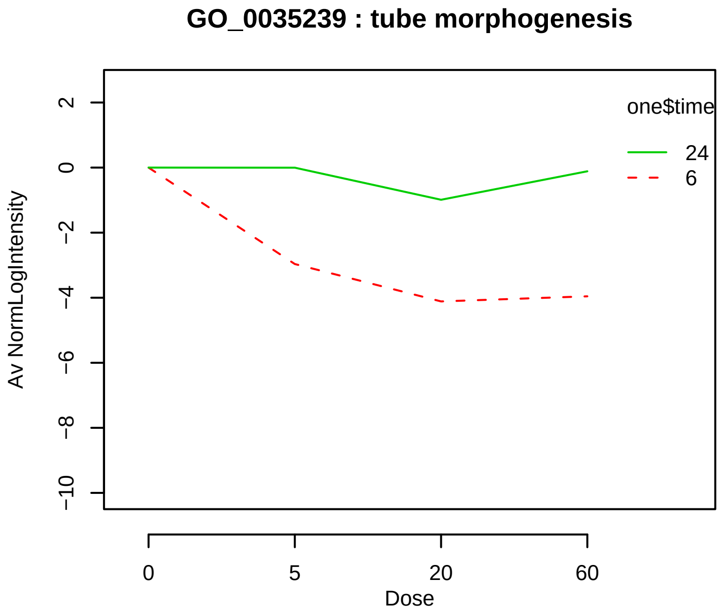
GO_0035239 : tube morphogenesis
one$time
24
6
Av NormLogIntensity
2
0
−2
−4
−6
−8
−10
0
5
20
60
Dose

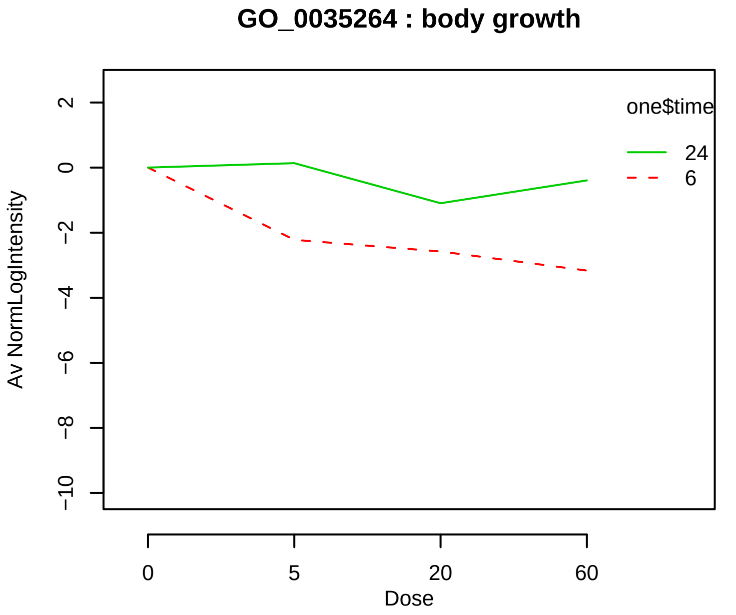
GO_0035264 : body growth
one$time
24
6
Av NormLogIntensity
2
0
−2
−4
−6
−8
−10
0
5
20
60
Dose

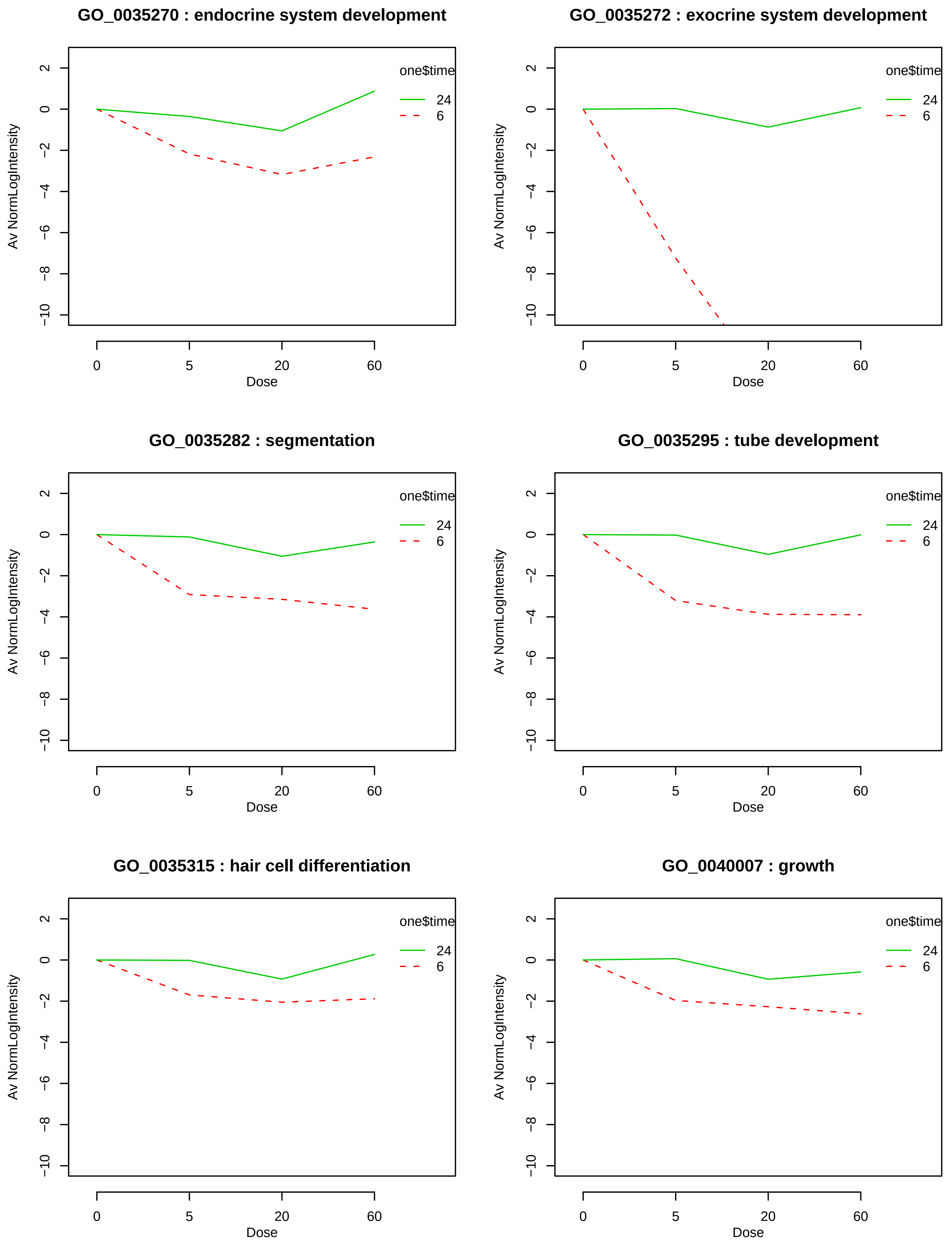
GO_0035270 : endocrine system development
GO_0035272 : exocrine system development
GO_0035282 : segmentation
GO_0035295 : tube development
GO_0035315 : hair cell differentiation
GO_0040007 : growth
Av NormLogIntensity
Dose
one$time
24
6
0
5
20
60
2
0
−2
−4
−6
−8
−10

GO_0040008 : regulation of growth

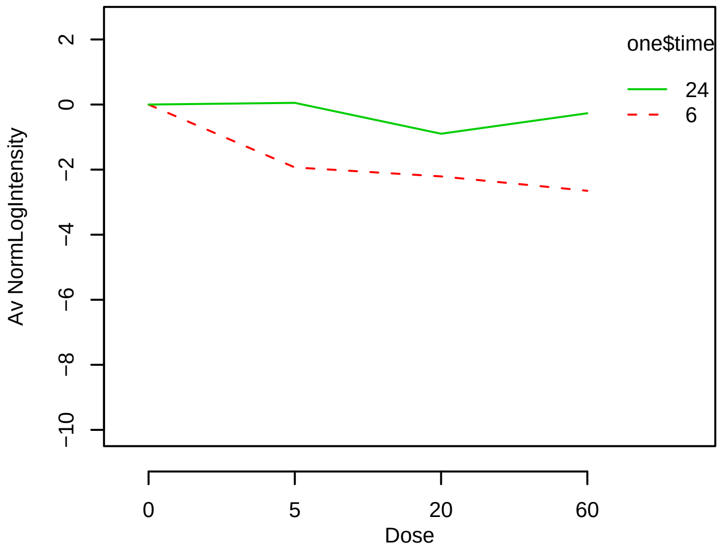

GO_0040012 : regulation of locomotion

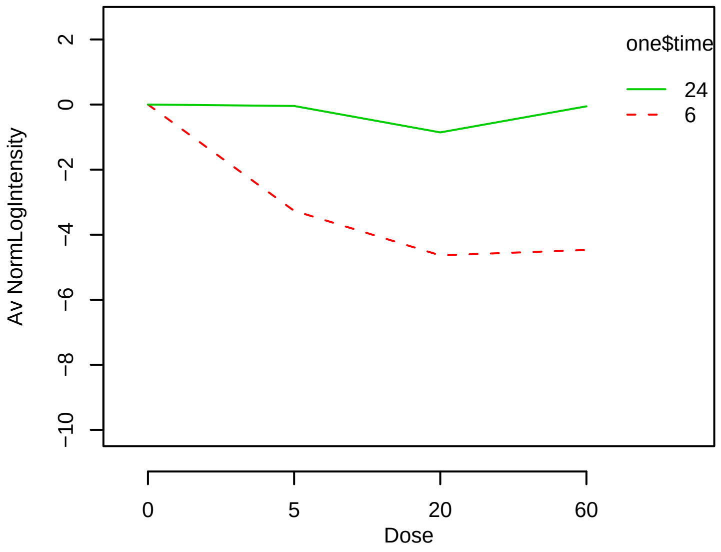

GO_0040013 : negative regulation of locomotion

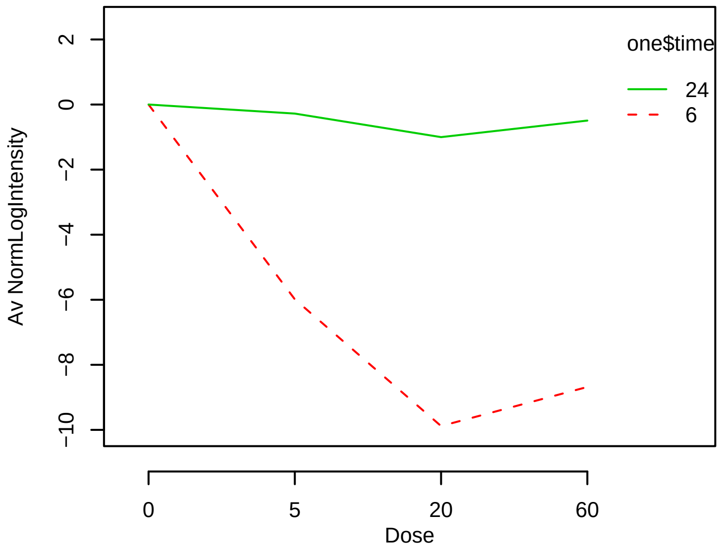

GO_0040014 : regulation of body size

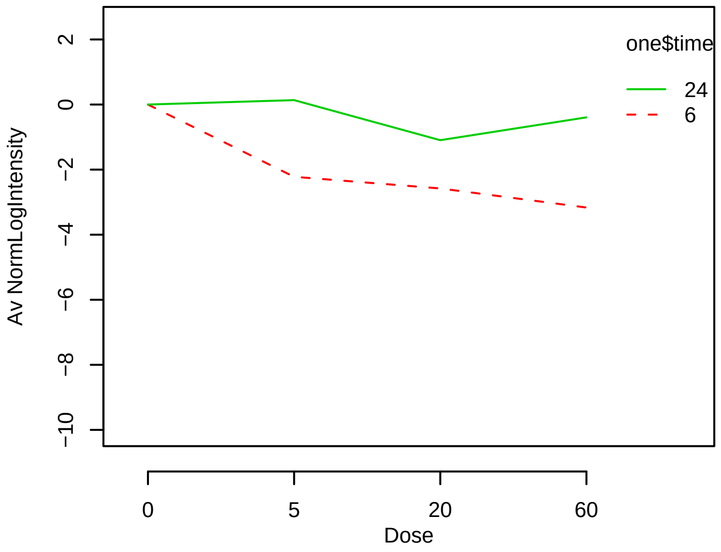

GO_0040015 : negative regulation of body size

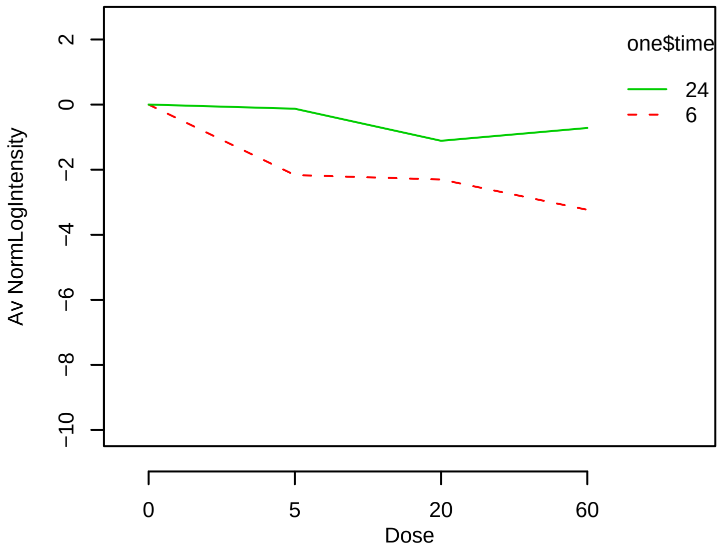

GO_0040017 : positive regulation of locomotion

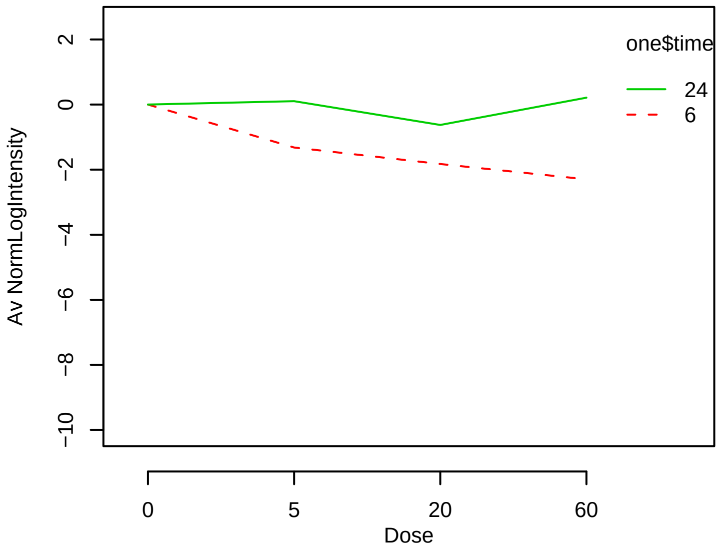

GO_0040018 : positive regulation of body size

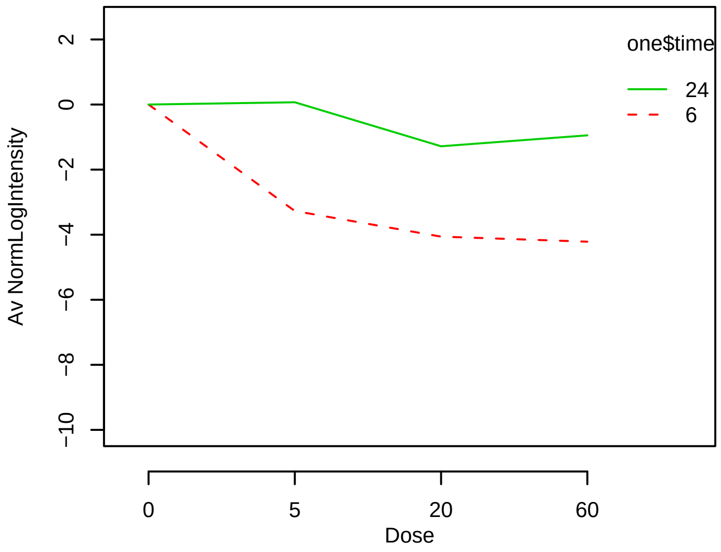

GO_0040023 : establishment of nucleus localization

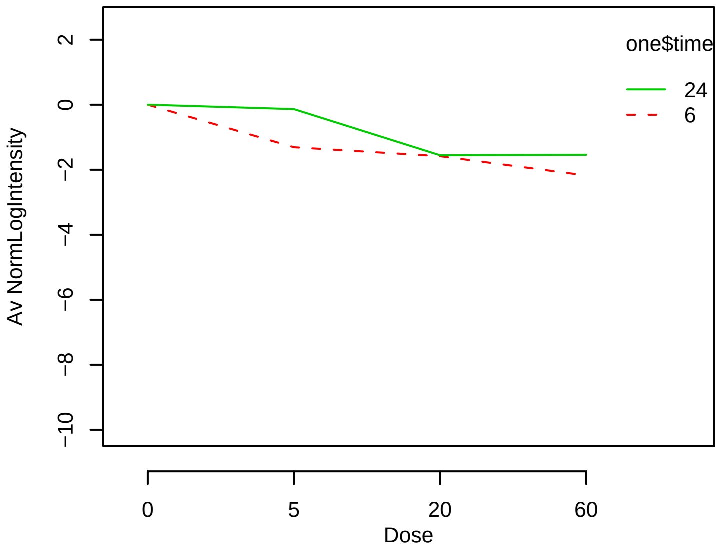

GO_0040029 : regulation of gene expression\, epigenetic

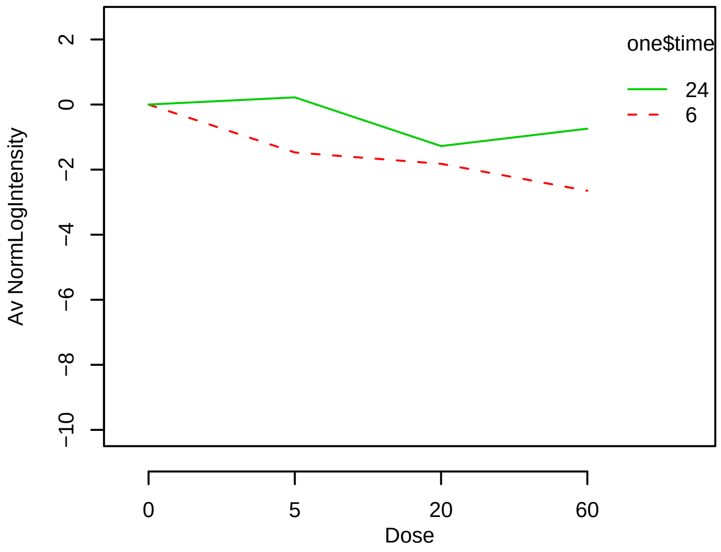

GO_0042033 : chemokine biosynthesis

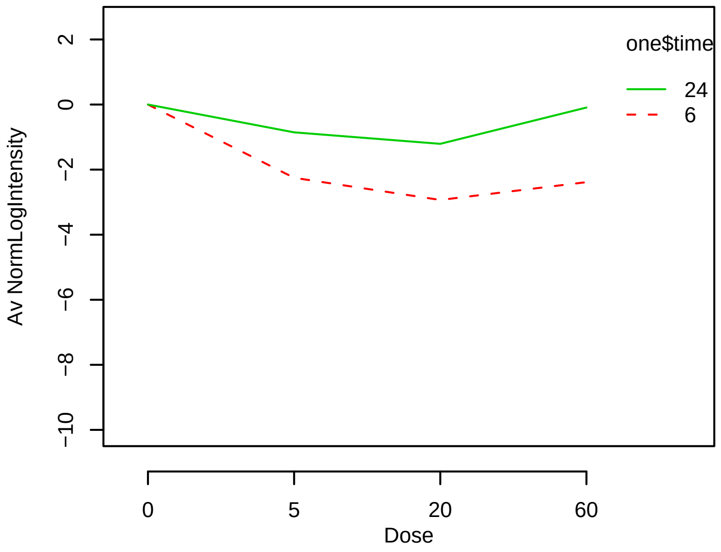

GO_0042035 : regulation of cytokine biosynthesis

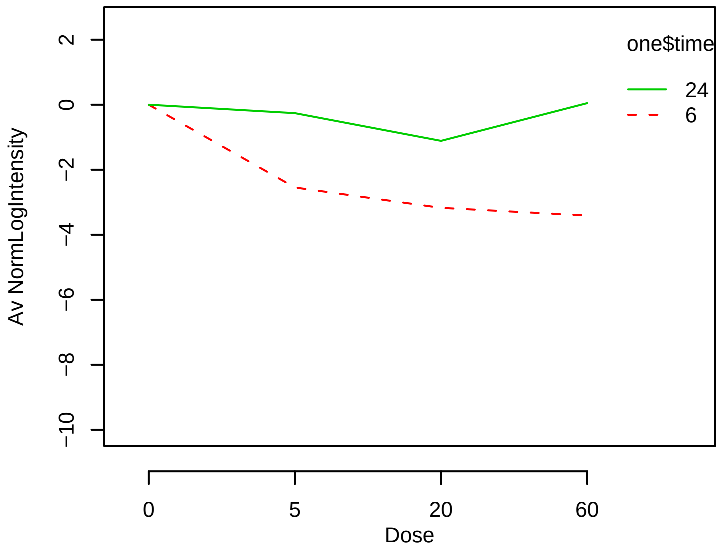

GO_0042036 : negative regulation of cytokine biosynthesi

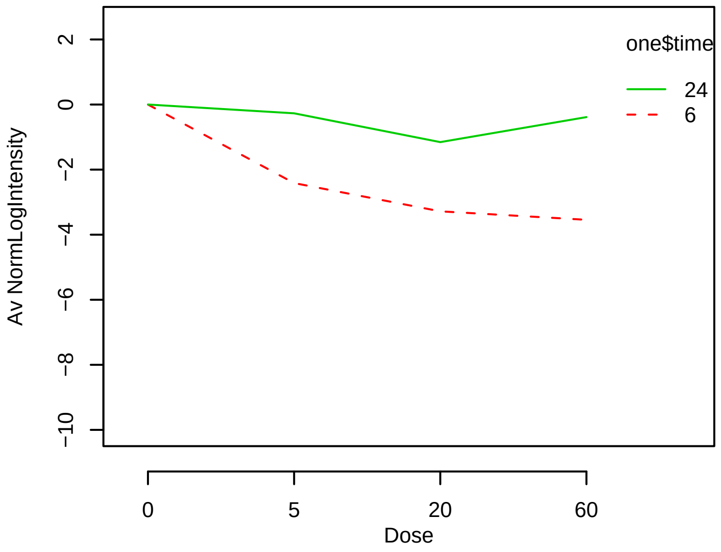

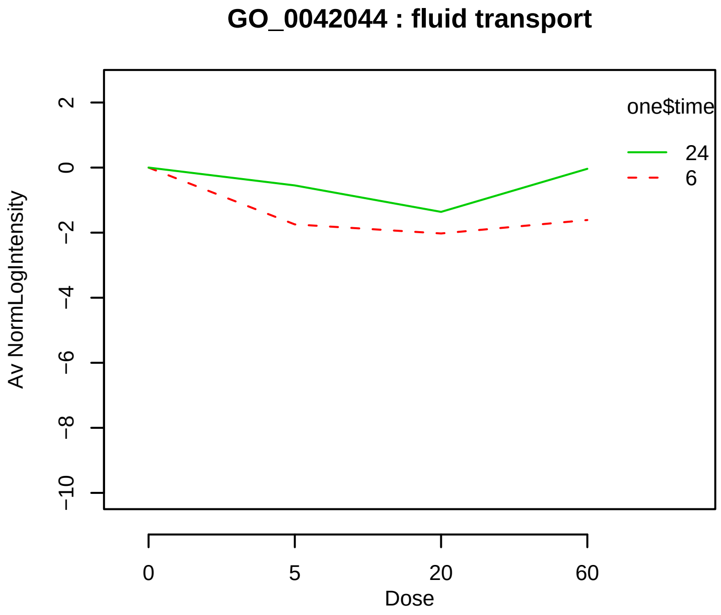
GO_0042044 : fluid transport
Av NormLogIntensity
Dose
one$time
24
6
2
0
−2
−4
−6
−8
−10
0
5
20
60

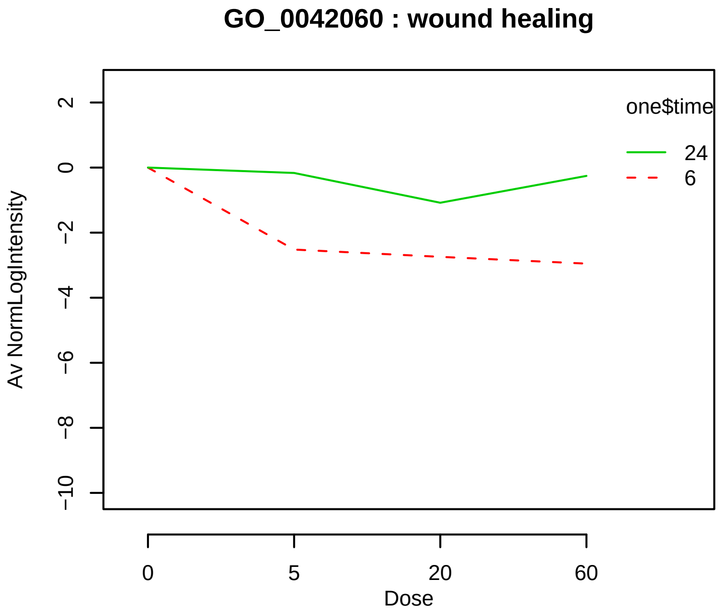
GO_0042060 : wound healing
Av NormLogIntensity
Dose
one$time
24
6
2
0
−2
−4
−6
−8
−10
0
5
20
60

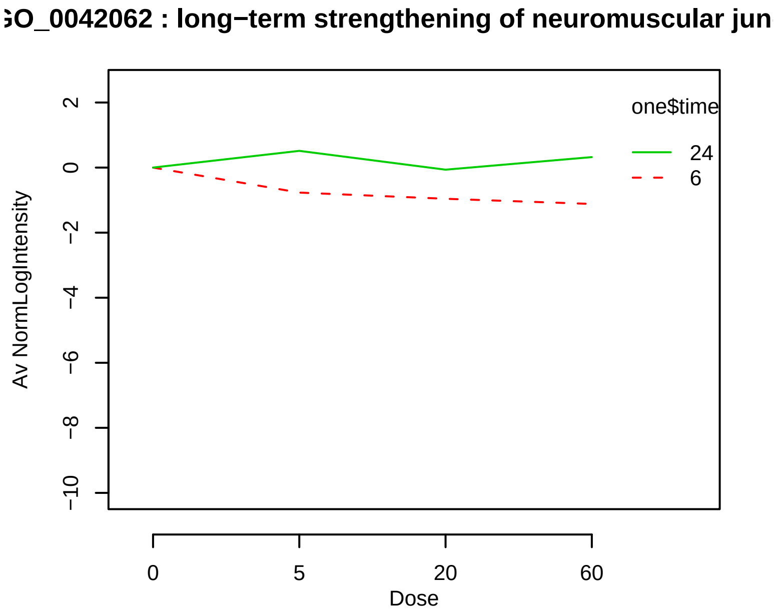
GO_0042062 : long−term strengthening of neuromuscular jun
Av NormLogIntensity
Dose
one$time
24
6
2
0
−2
−4
−6
−8
−10
0
5
20
60

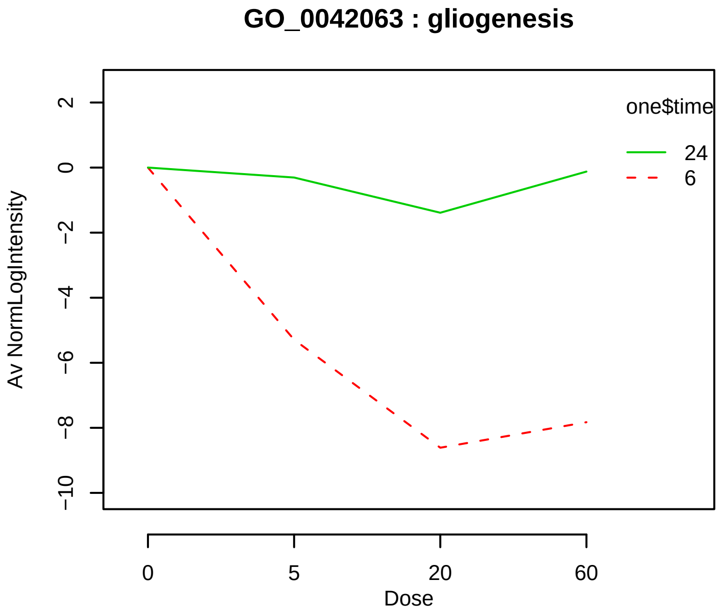
GO_0042063 : gliogenesis
Av NormLogIntensity
Dose
one$time
24
6
2
0
−2
−4
−6
−8
−10
0
5
20
60

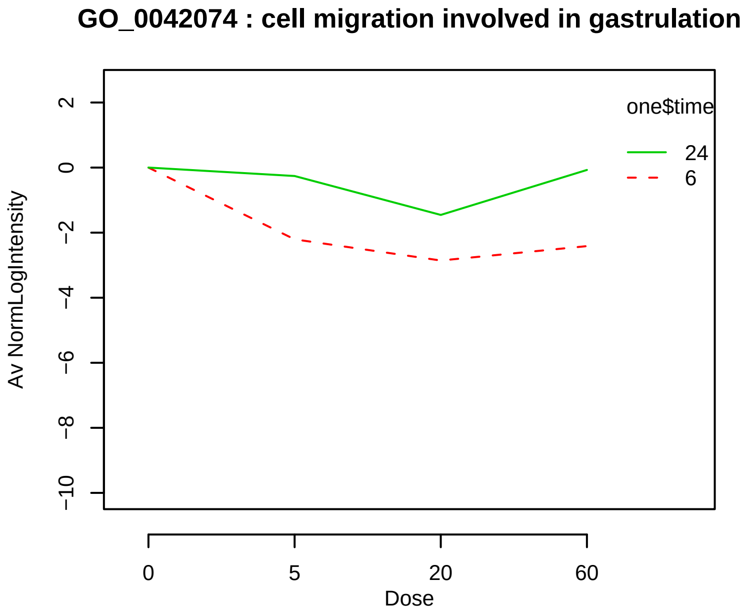
GO_0042074 : cell migration involved in gastrulation
Av NormLogIntensity
Dose
one$time
24
6
2
0
−2
−4
−6
−8
−10
0
5
20
60

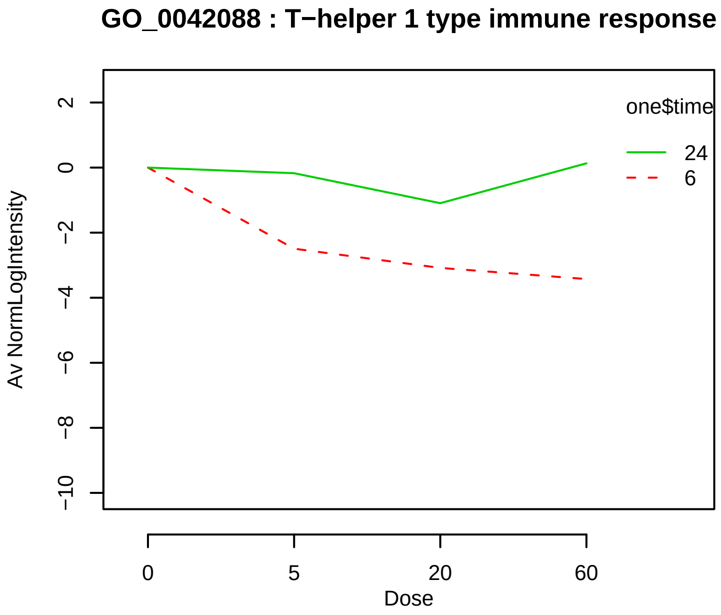
GO_0042088 : T−helper 1 type immune response
Av NormLogIntensity
Dose
one$time
24
6
2
0
−2
−4
−6
−8
−10
0
5
20
60

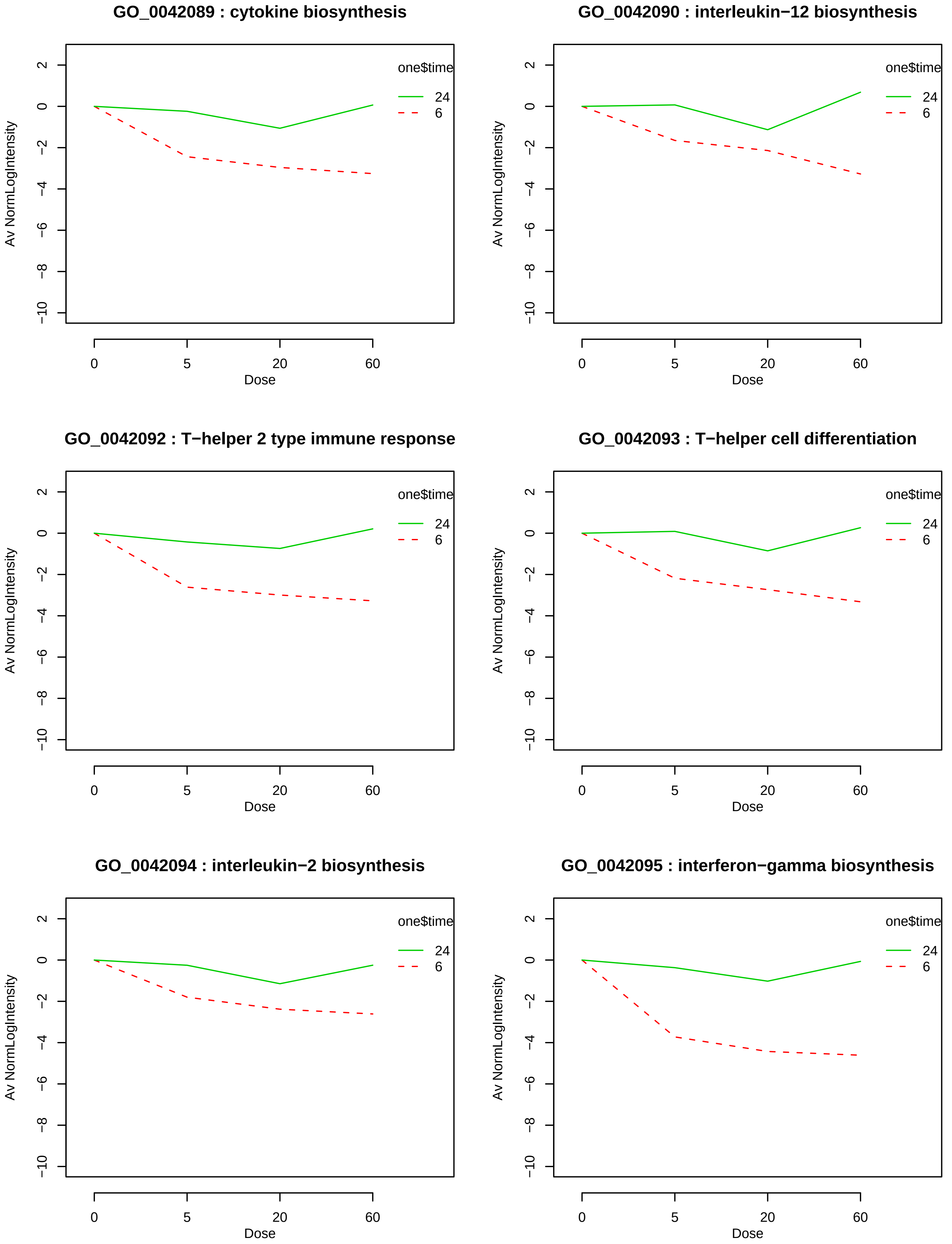
GO_0042089 : cytokine biosynthesis
GO_0042090 : interleukin−12 biosynthesis
GO_0042092 : T−helper 2 type immune response
GO_0042093 : T−helper cell differentiation
GO_0042094 : interleukin−2 biosynthesis
GO_0042095 : interferon−gamma biosynthesis
Av NormLogIntensity
Dose
one$time
24
6
0
5
20
60
2
0
−2
−4
−6
−8
−10

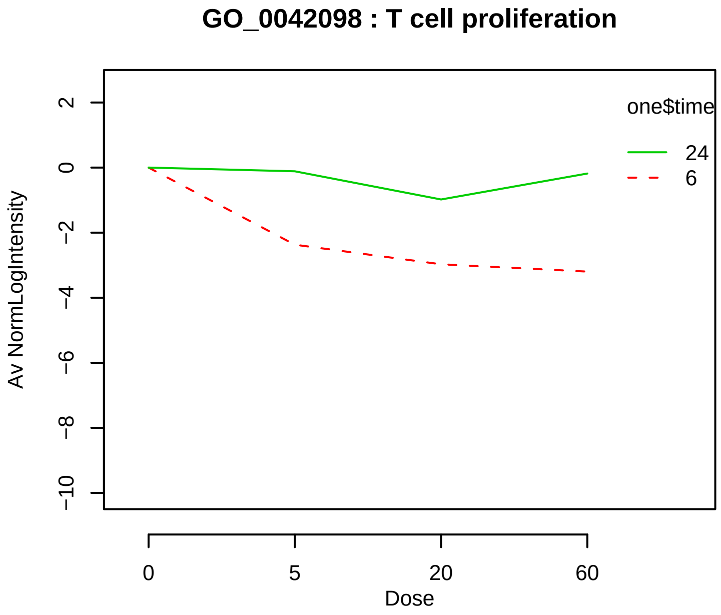
GO_0042098 : T cell proliferation
Av NormLogIntensity
Dose
one$time
24
6

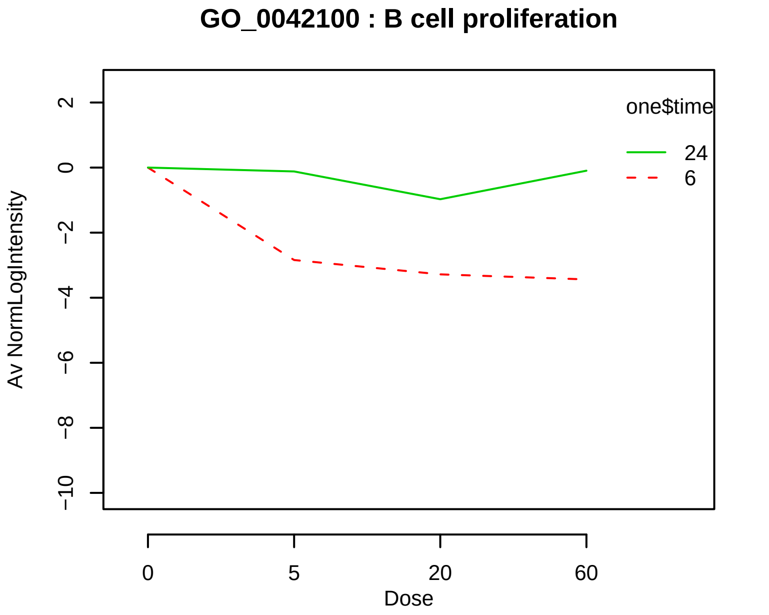
GO_0042100 : B cell proliferation
Av NormLogIntensity
Dose
one$time
24
6

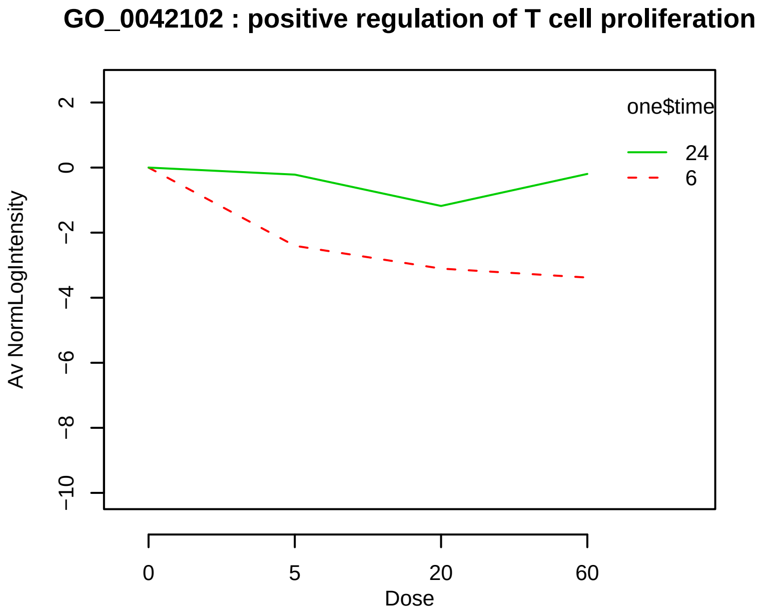
GO_0042102 : positive regulation of T cell proliferation
Av NormLogIntensity
Dose
one$time
24
6

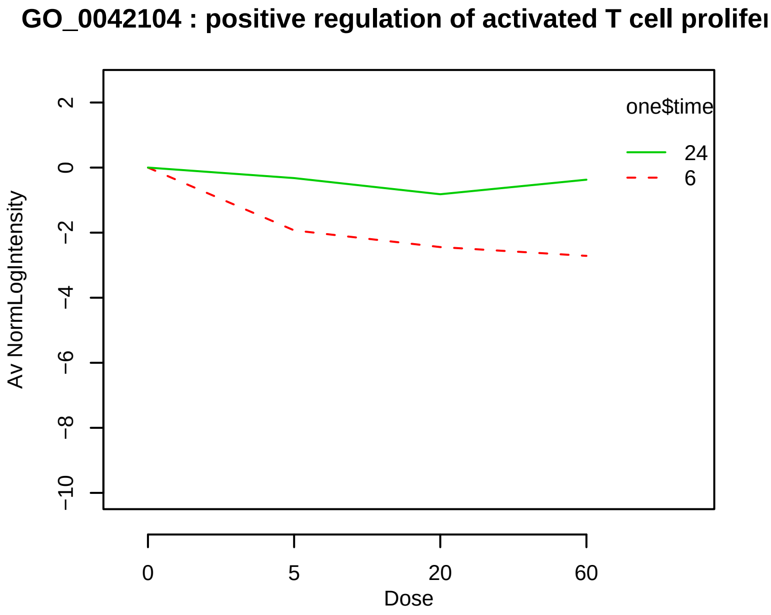
GO_0042104 : positive regulation of activated T cell prolifer
Av NormLogIntensity
Dose
one$time
24
6

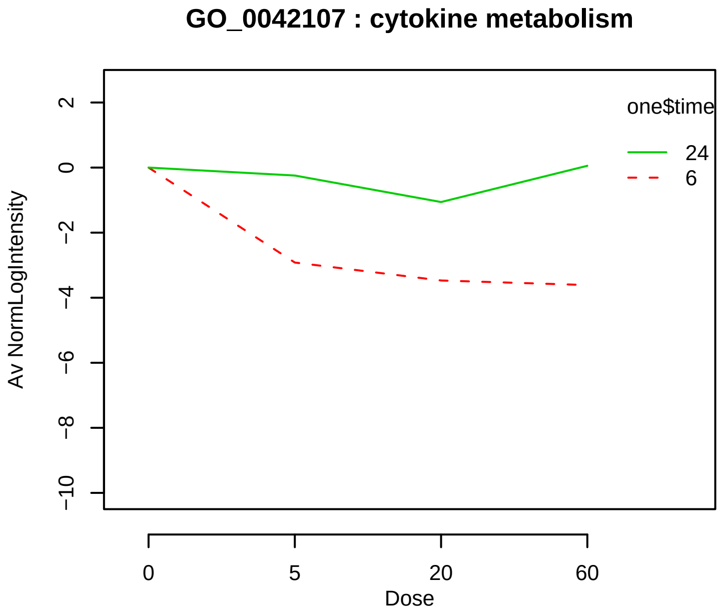
GO_0042107 : cytokine metabolism
Av NormLogIntensity
Dose
one$time
24
6

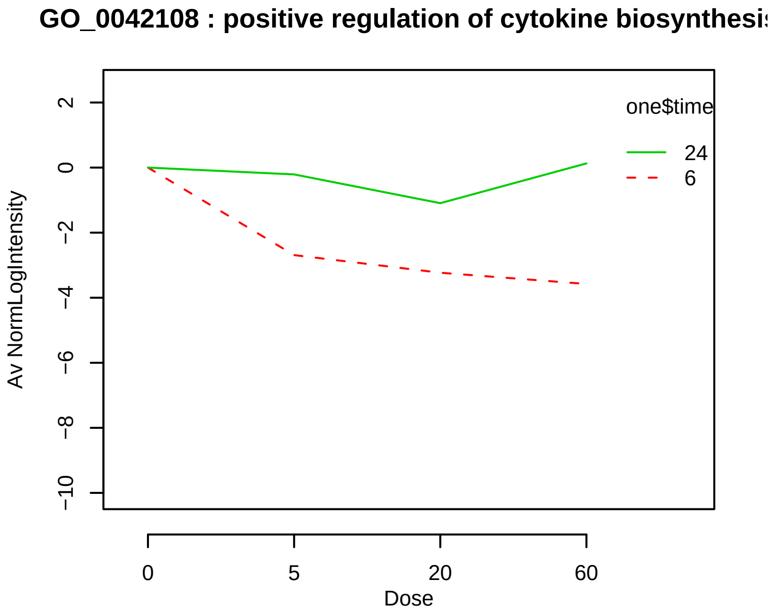
GO_0042108 : positive regulation of cytokine biosynthesis
Av NormLogIntensity
Dose
one$time
24
6

GO_0042110 : T cell activation

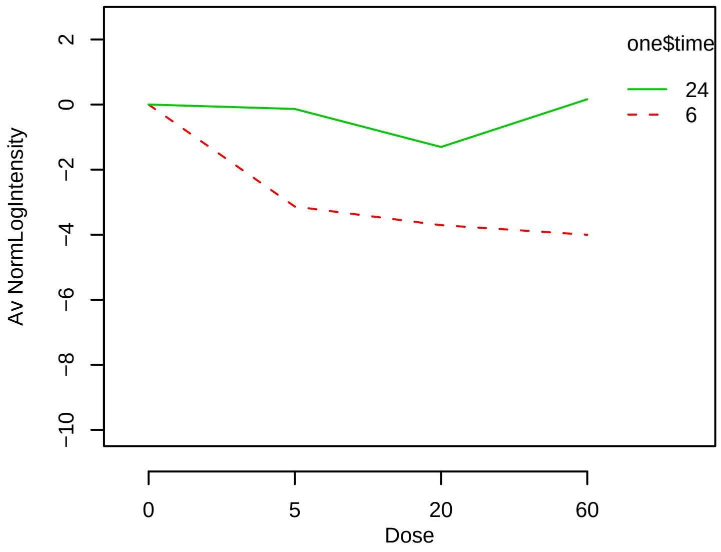

GO_0042113 : B cell activation

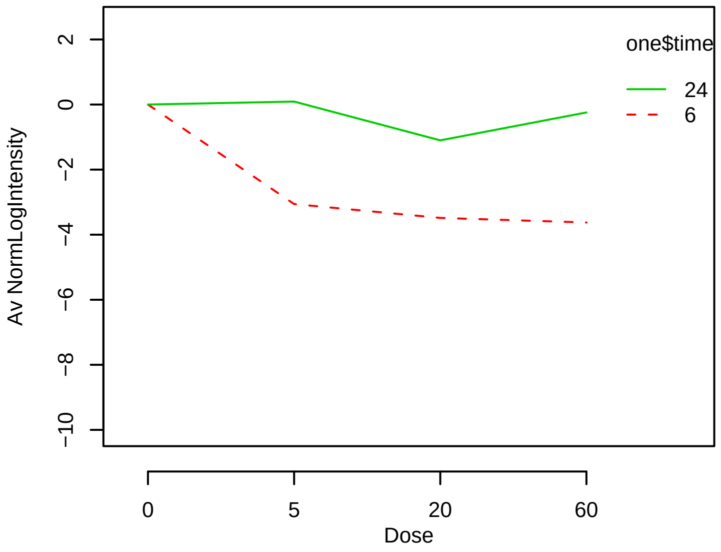

GO_0042116 : macrophage activation

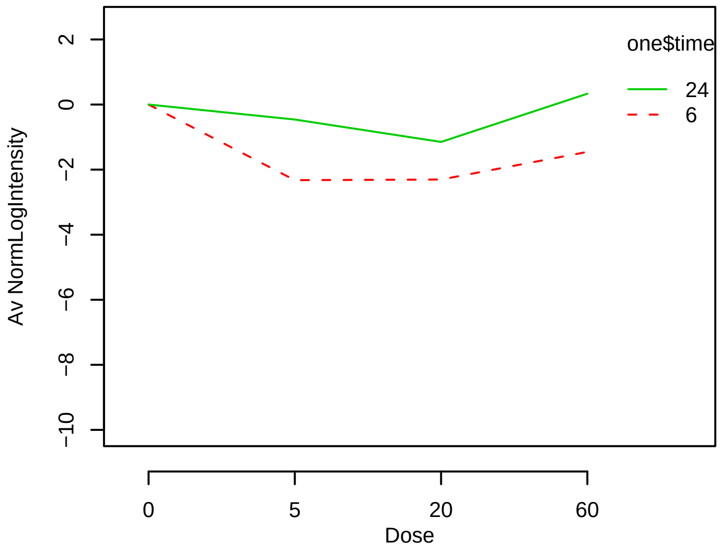

GO_0042119 : neutrophil activation

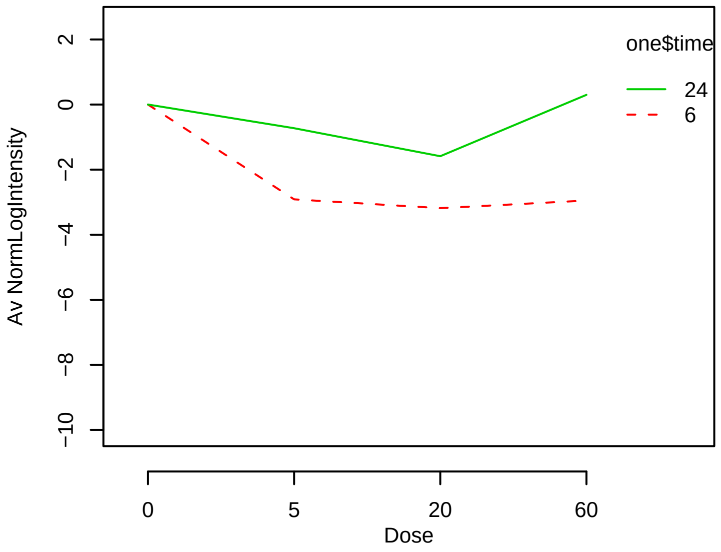

GO_0042129 : regulation of T cell proliferation

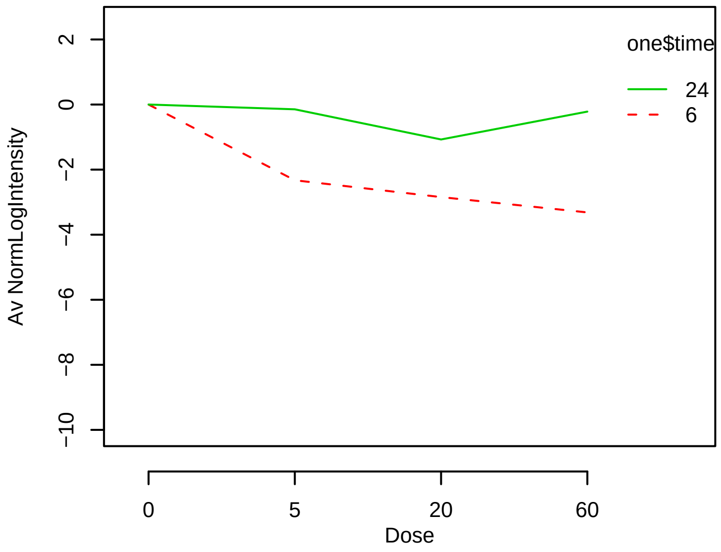

GO_0042130 : negative regulation of T cell proliferation

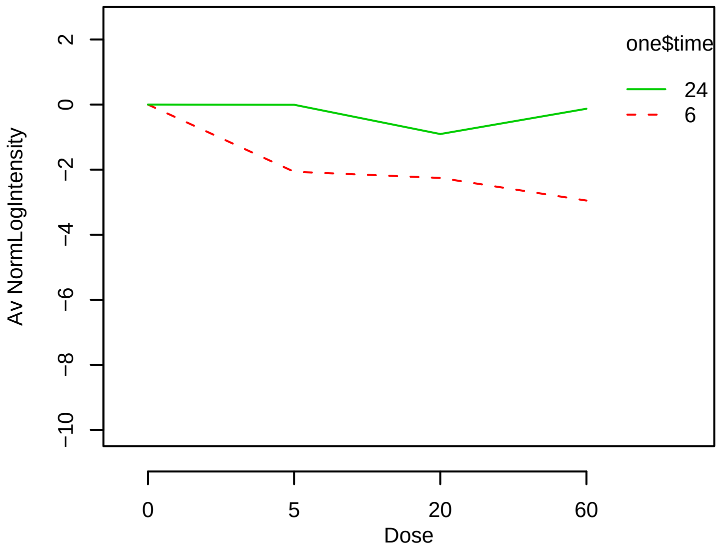

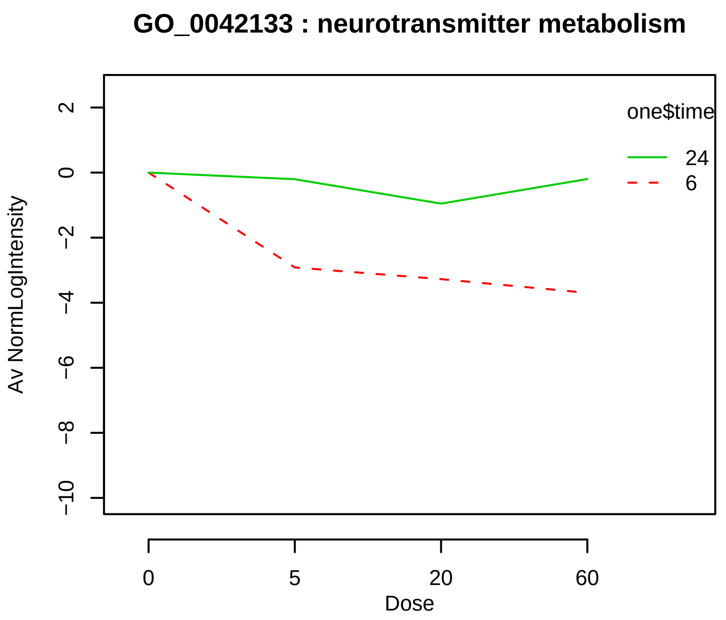
GO_0042133 : neurotransmitter metabolism
Av NormLogIntensity
Dose
one$time
24
6

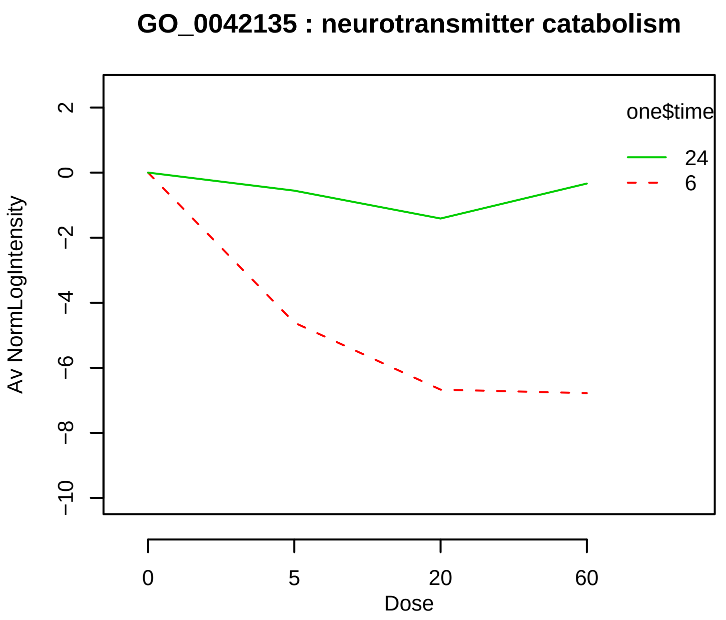
GO_0042135 : neurotransmitter catabolism
Av NormLogIntensity
Dose
one$time
24
6

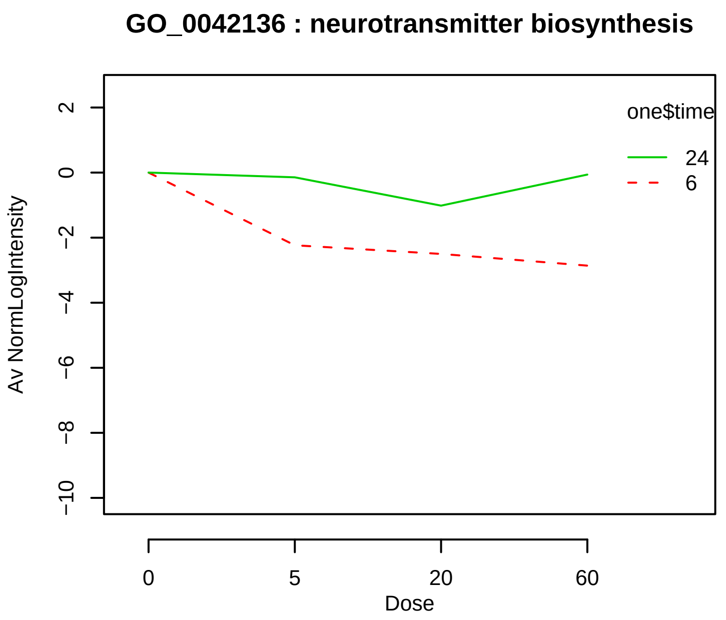
GO_0042136 : neurotransmitter biosynthesis
Av NormLogIntensity
Dose
one$time
24
6

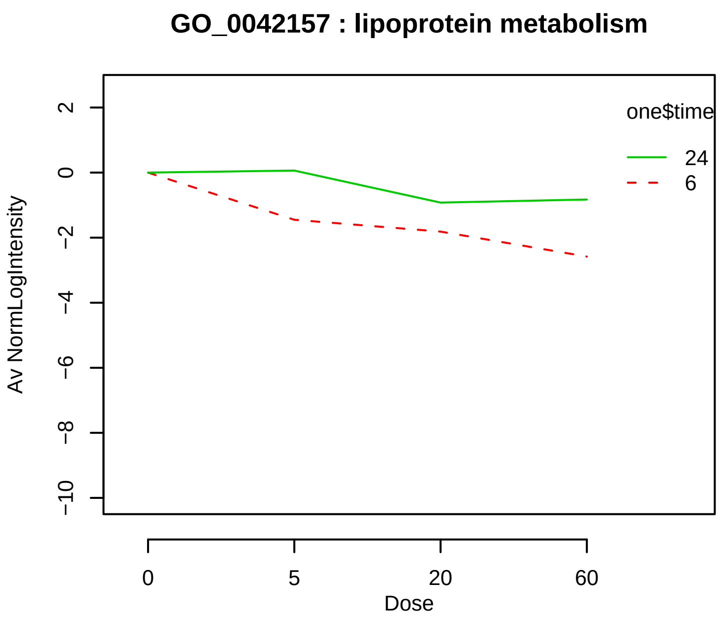
GO_0042157 : lipoprotein metabolism
Av NormLogIntensity
Dose
one$time
24
6

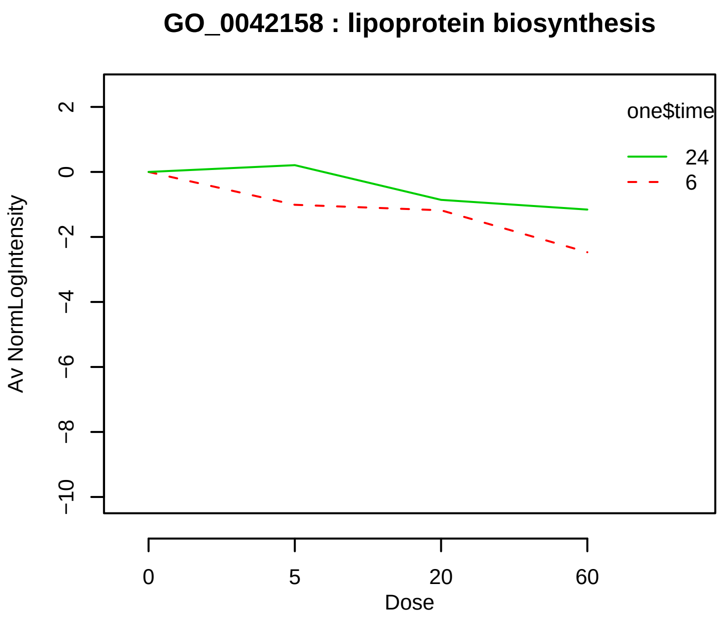
GO_0042158 : lipoprotein biosynthesis
Av NormLogIntensity
Dose
one$time
24
6

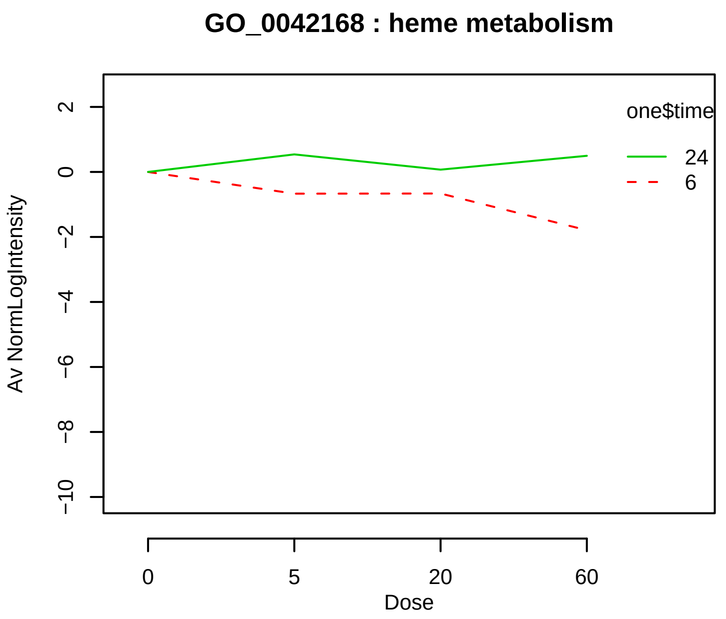
GO_0042168 : heme metabolism
Av NormLogIntensity
Dose
one$time
24
6

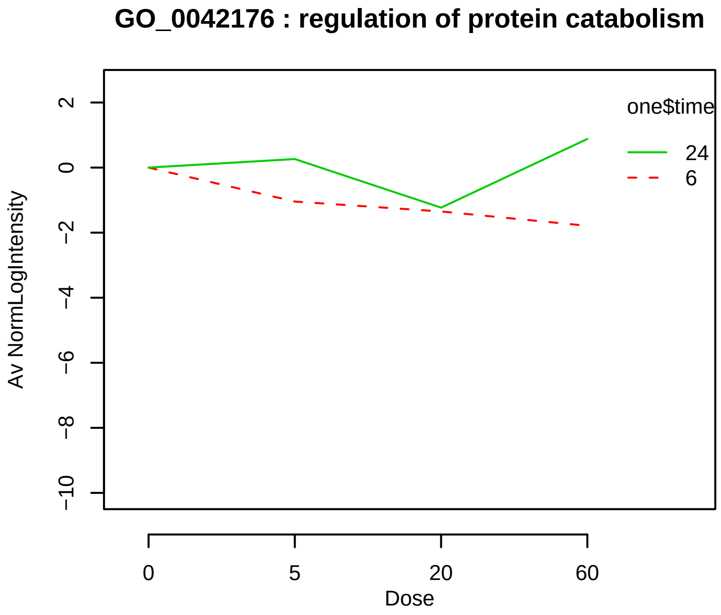

GO_0042176 : regulation of protein catabolism
Av NormLogIntensity
Dose
one$time
24
6

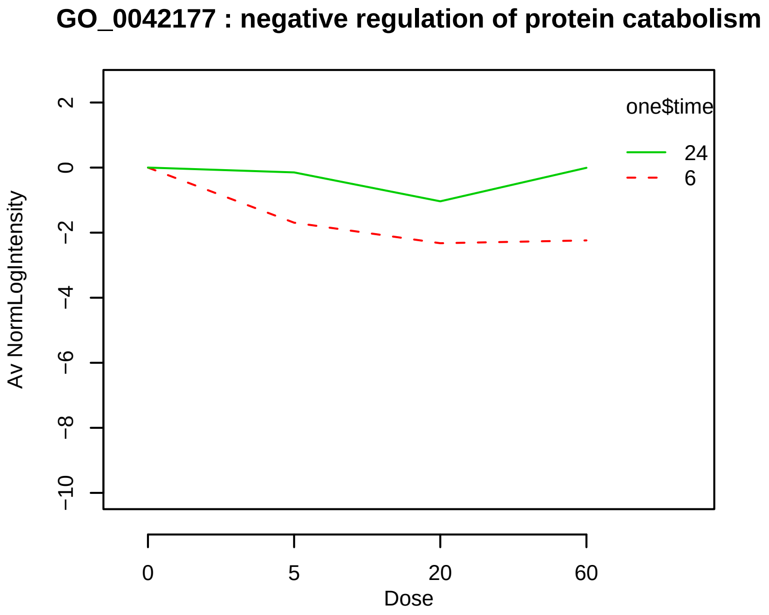

GO_0042177 : negative regulation of protein catabolism
Av NormLogIntensity
Dose
one$time
24
6

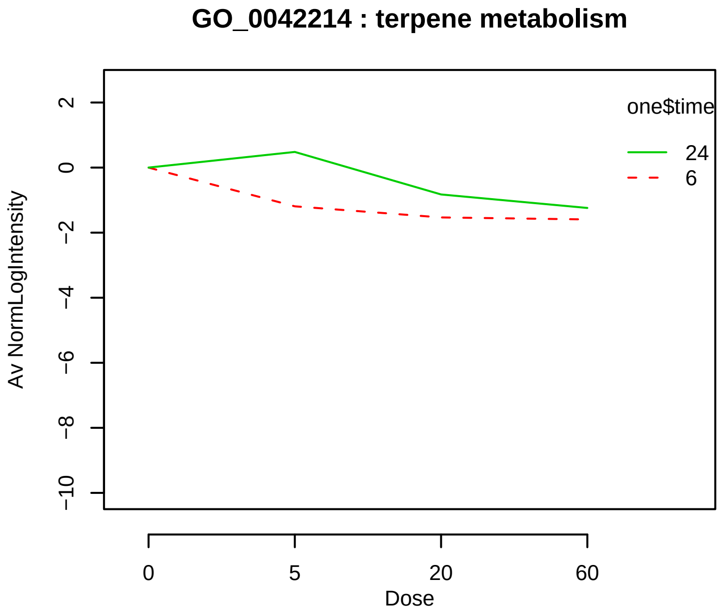

GO_0042214 : terpene metabolism
Av NormLogIntensity
Dose
one$time
24
6

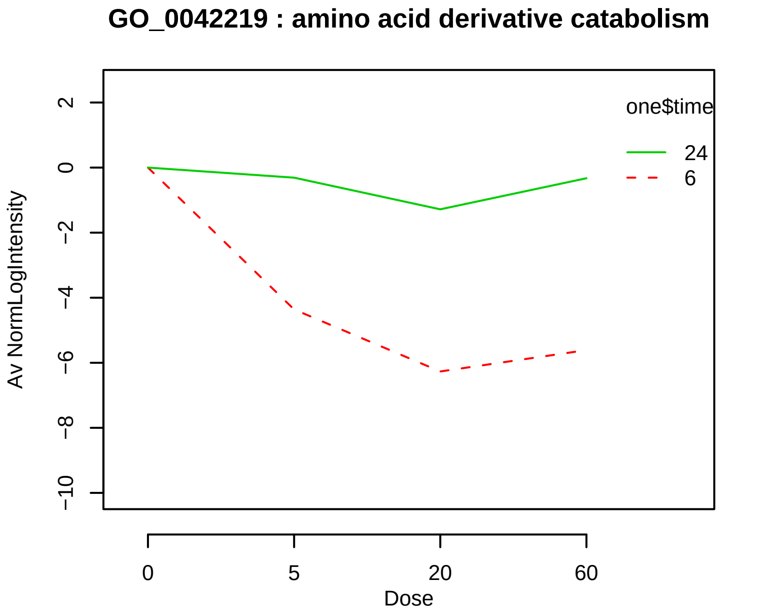

GO_0042219 : amino acid derivative catabolism
Av NormLogIntensity
Dose
one$time
24
6

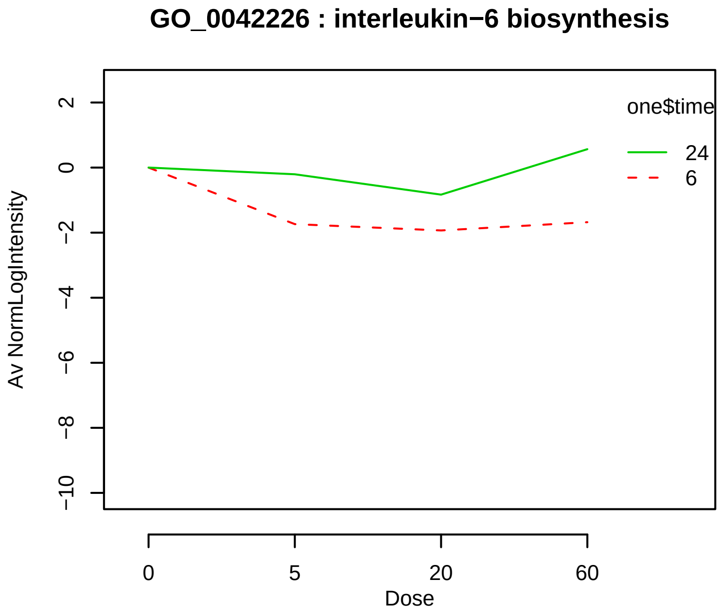

GO_0042226 : interleukin−6 biosynthesis
Av NormLogIntensity
Dose
one$time
24
6

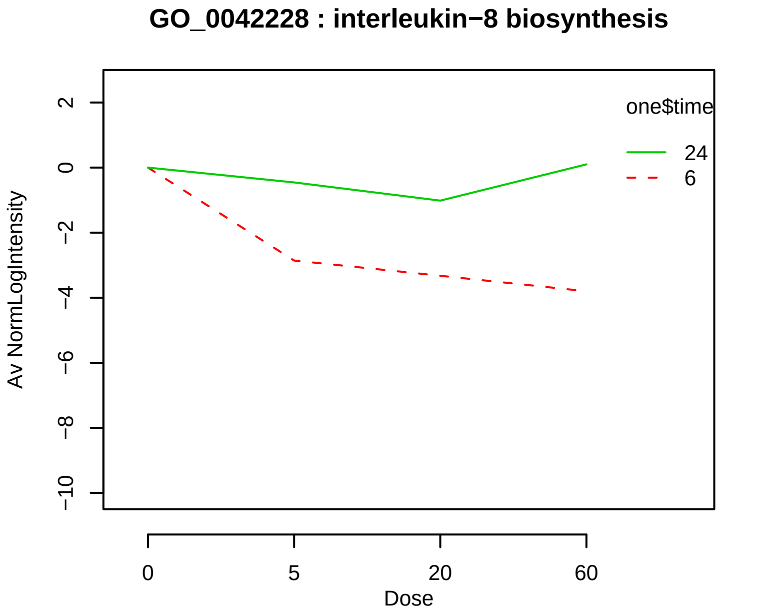

GO_0042228 : interleukin−8 biosynthesis
Av NormLogIntensity
Dose
one$time
24
6

GO_0042246 : tissue regeneration

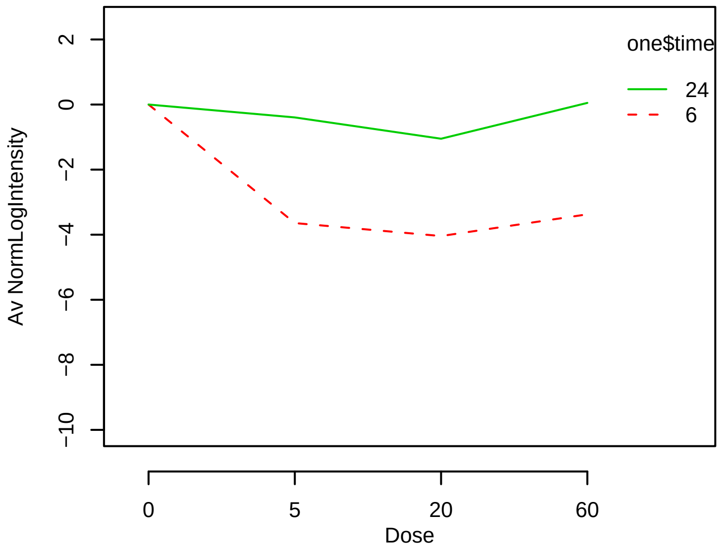

O_0042253 : granulocyte macrophage colony−stimulating fa

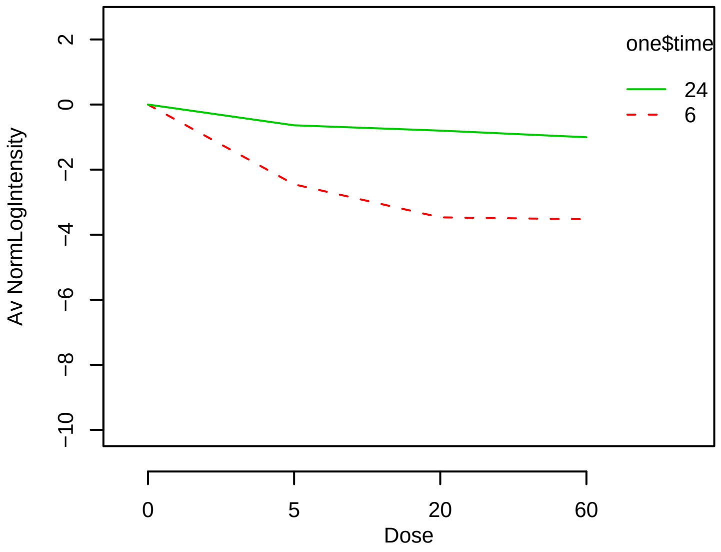

GO_0042254 : ribosome biogenesis and assembly

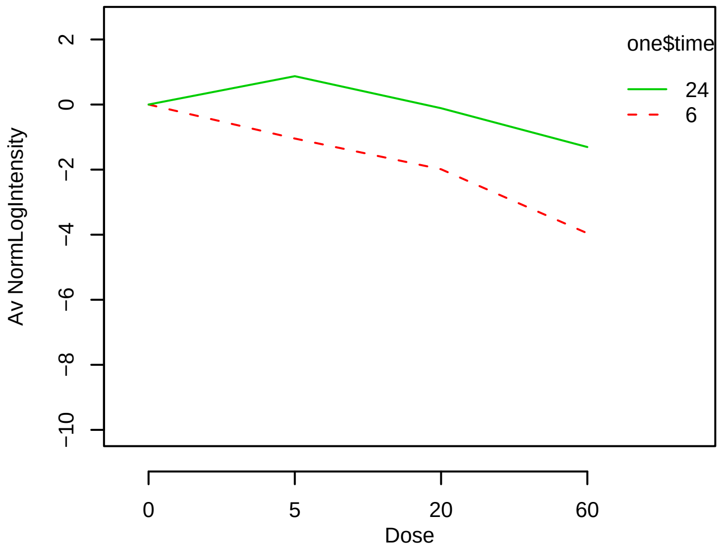

GO_0042267 : natural killer cell mediated cytotoxicity

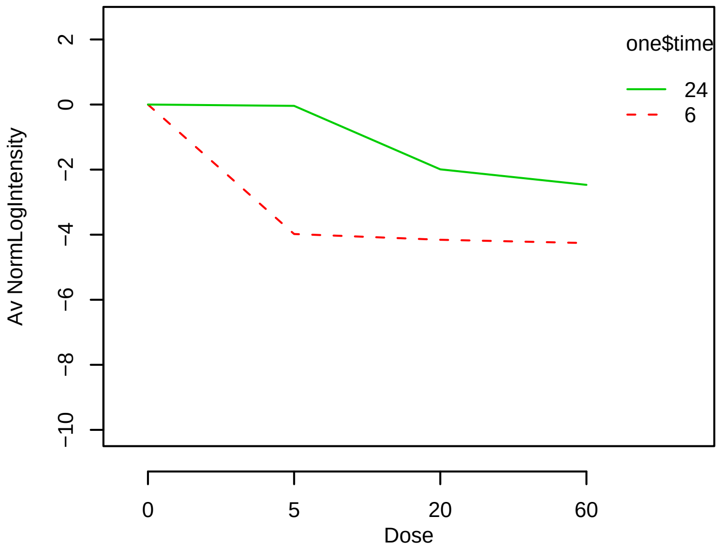

GO_0042278 : purine nucleoside metabolism

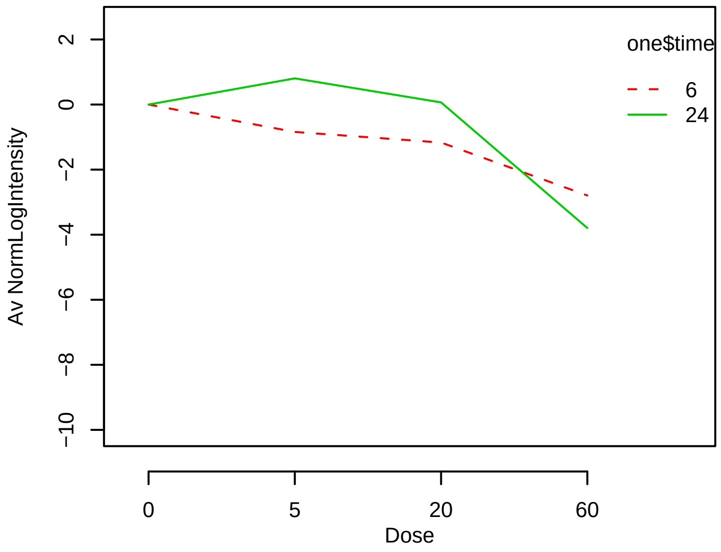

GO_0042303 : molting cycle

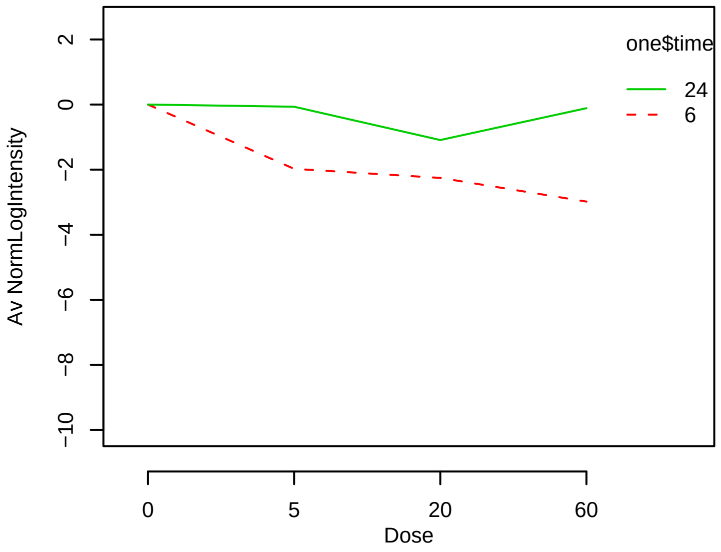

GO_0042306 : regulation of protein import into nucleus

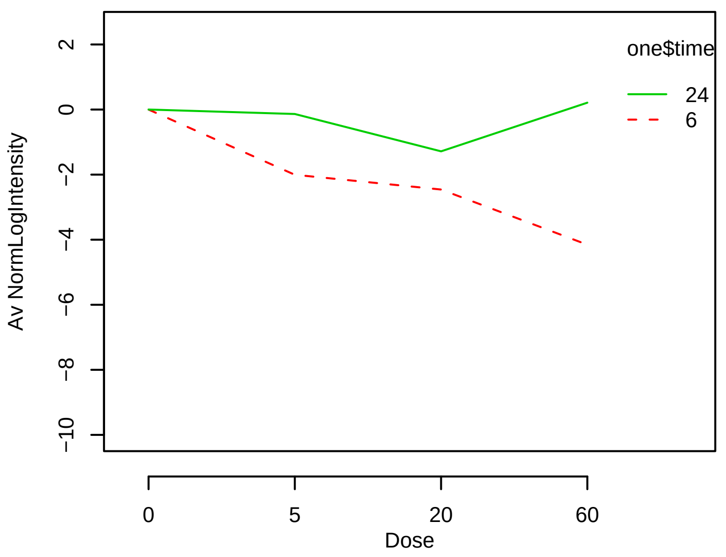

GO_0042307 : positive regulation of protein import into nucl

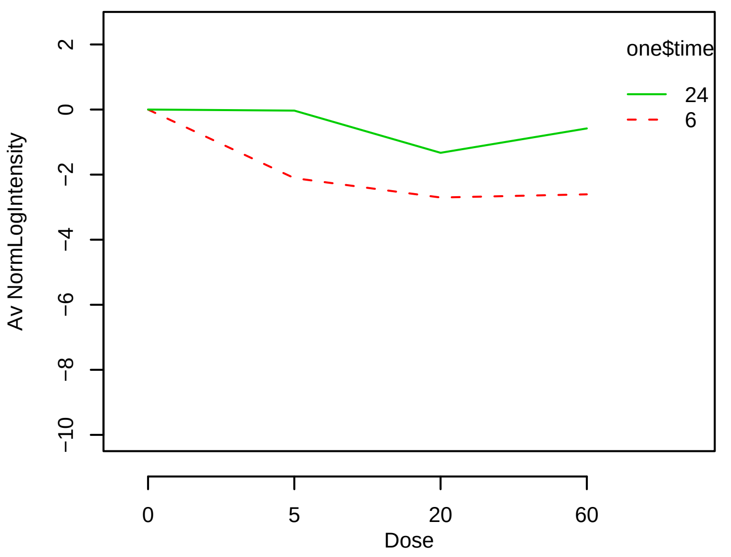

GO_0042308 : negative regulation of protein import into nucl

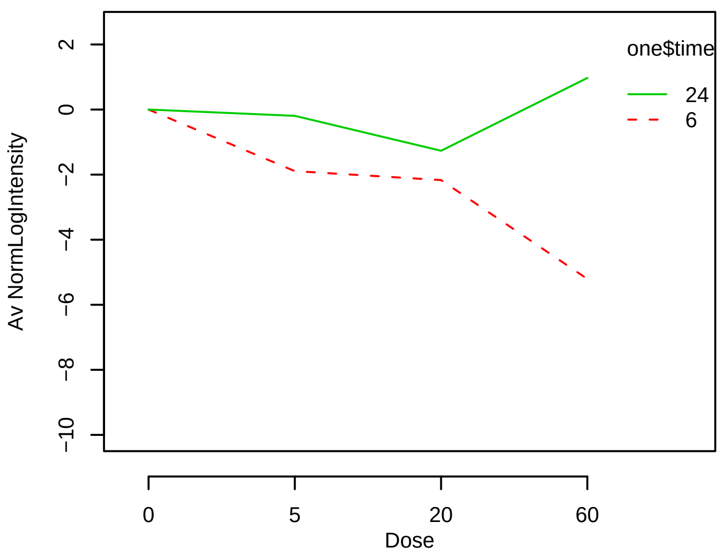

GO_0042310 : vasoconstriction

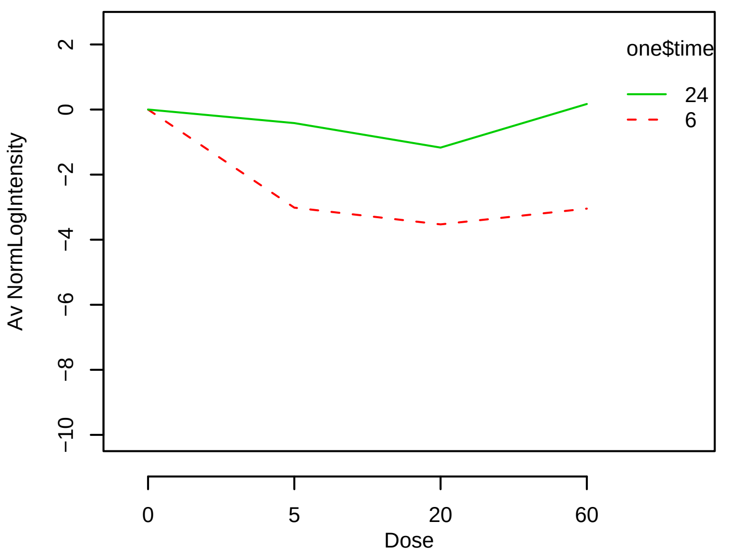

GO_0042311 : vasodilation

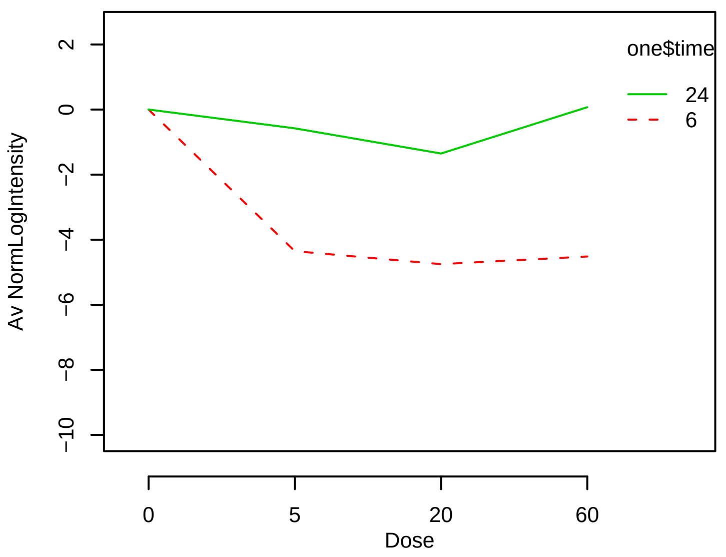

GO_0042312 : regulation of vasodilation

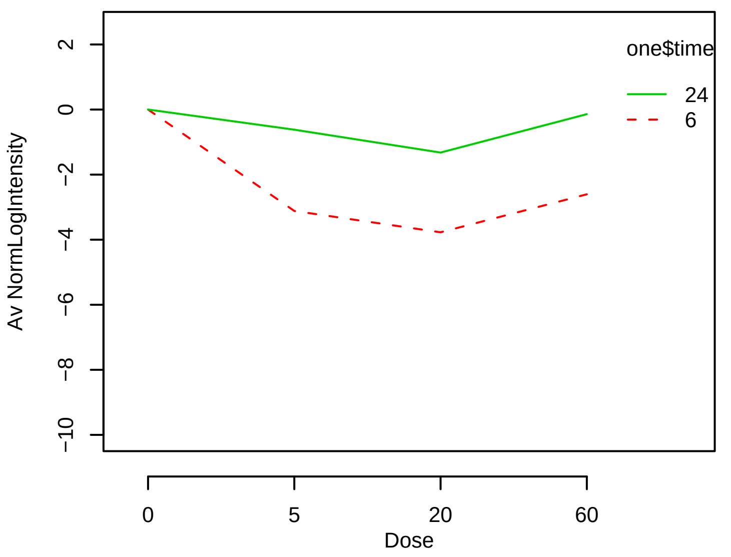

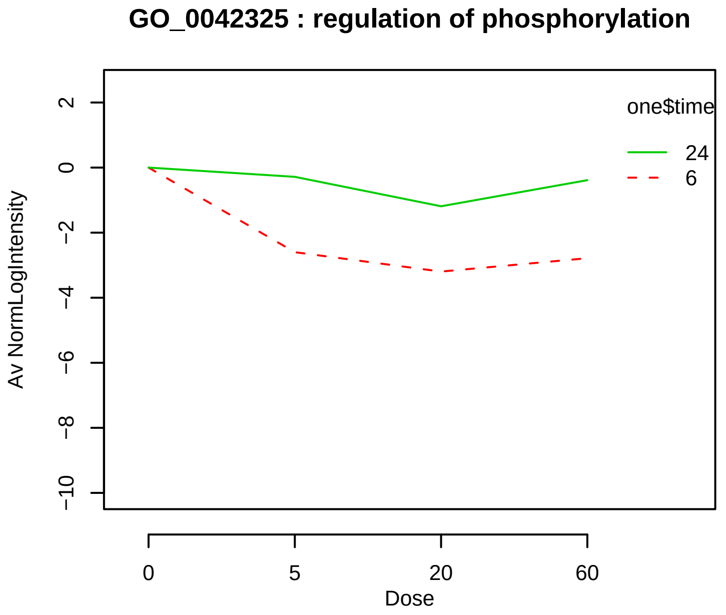
GO_0042325 : regulation of phosphorylation
Av NormLogIntensity
Dose
one$time
24
6
2
0
−2
−4
−6
−8
−10
0
5
20
60

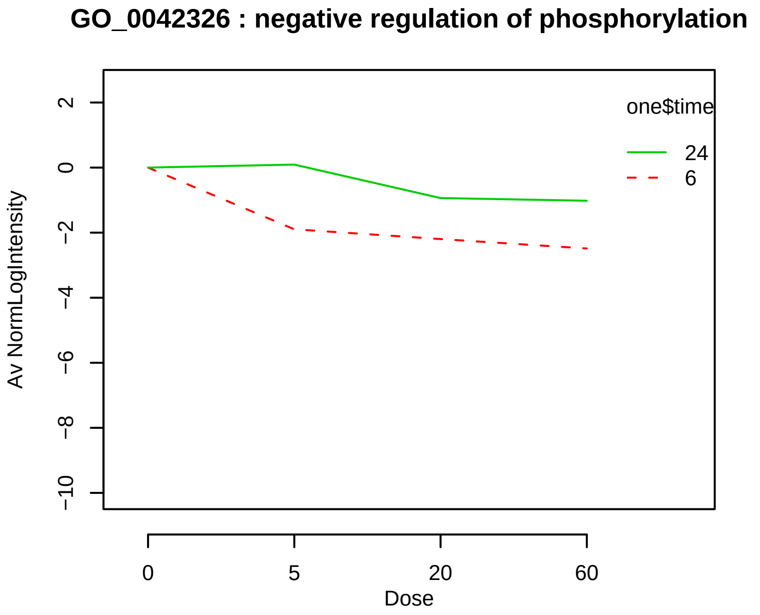
GO_0042326 : negative regulation of phosphorylation
Av NormLogIntensity
Dose
one$time
24
6
2
0
−2
−4
−6
−8
−10
0
5
20
60

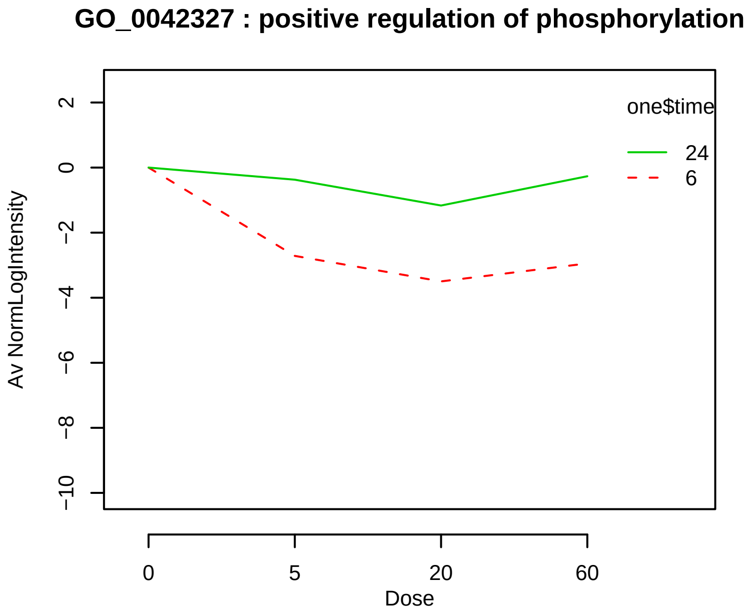
GO_0042327 : positive regulation of phosphorylation
Av NormLogIntensity
Dose
one$time
24
6
2
0
−2
−4
−6
−8
−10
0
5
20
60

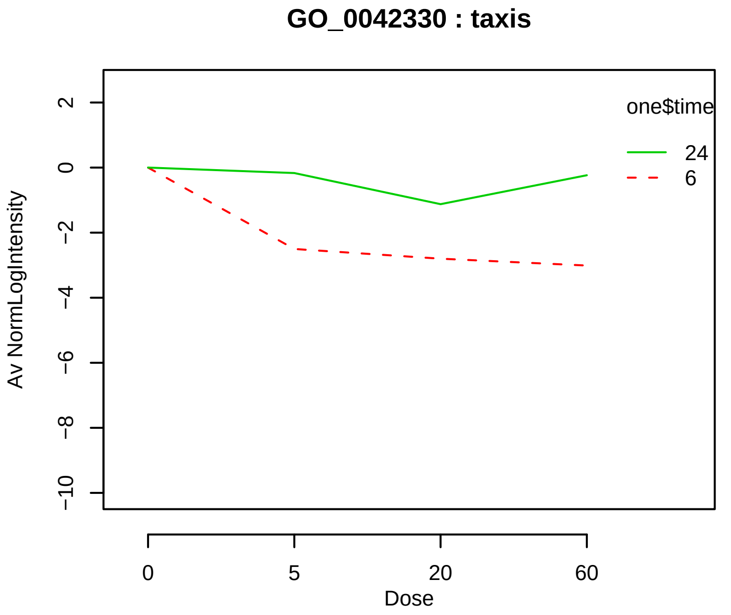
GO_0042330 : taxis
Av NormLogIntensity
Dose
one$time
24
6
2
0
−2
−4
−6
−8
−10
0
5
20
60

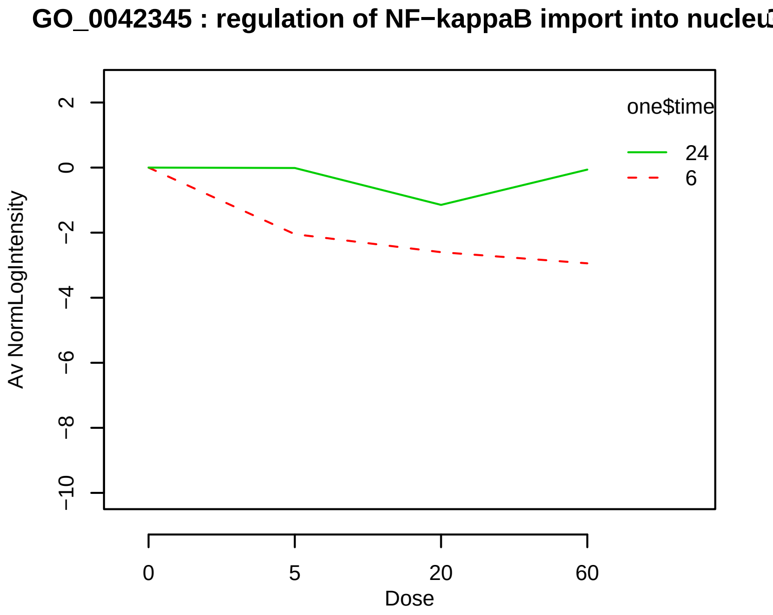
GO_0042345 : regulation of NF−kappaB import into nucleu
Av NormLogIntensity
Dose
one$time
24
6
2
0
−2
−4
−6
−8
−10
0
5
20
60

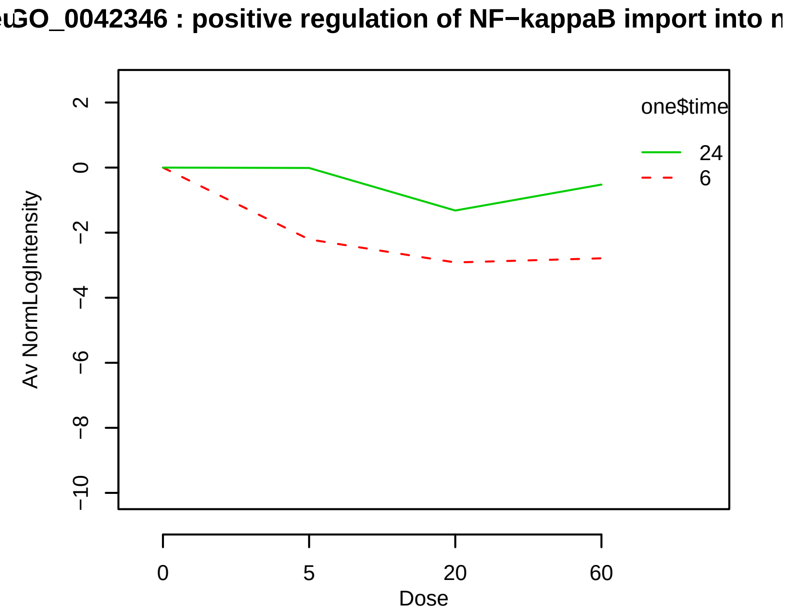
GO_0042346 : positive regulation of NF−kappaB import into n
Av NormLogIntensity
Dose
one$time
24
6
2
0
−2
−4
−6
−8
−10
0
5
20
60

GO_0042347 : negative regulation of NF−kappaB import into r

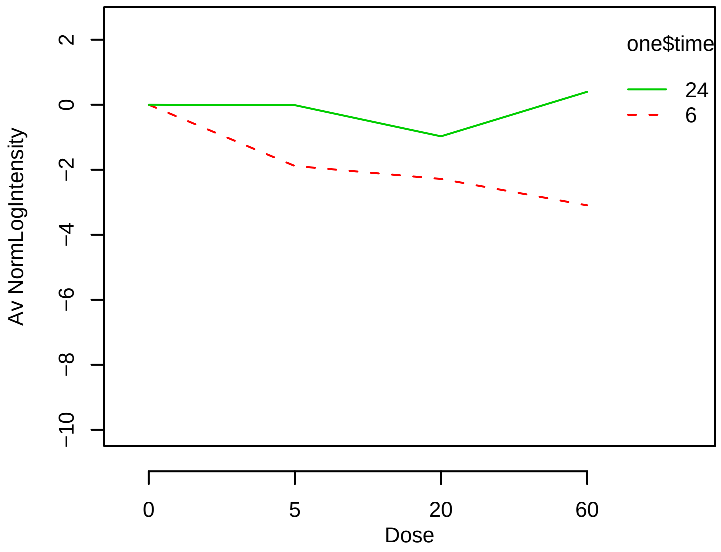

GO_0042348 : NF−kappaB import into nucleus

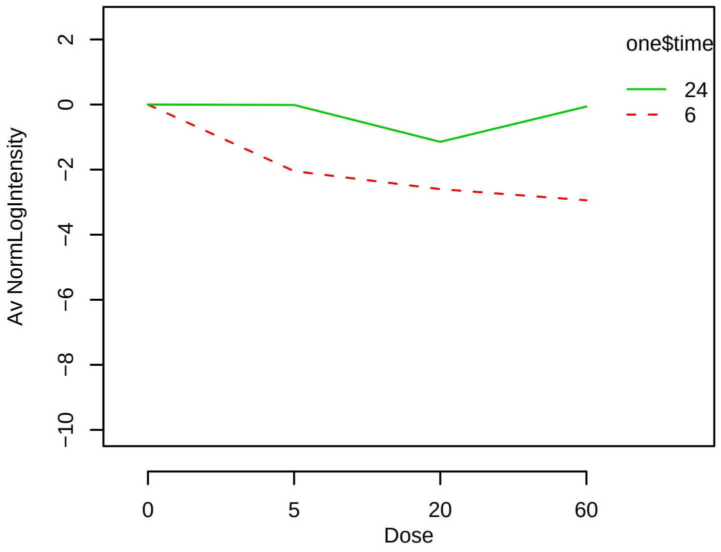

GO_0042354 : L−fucose metabolism

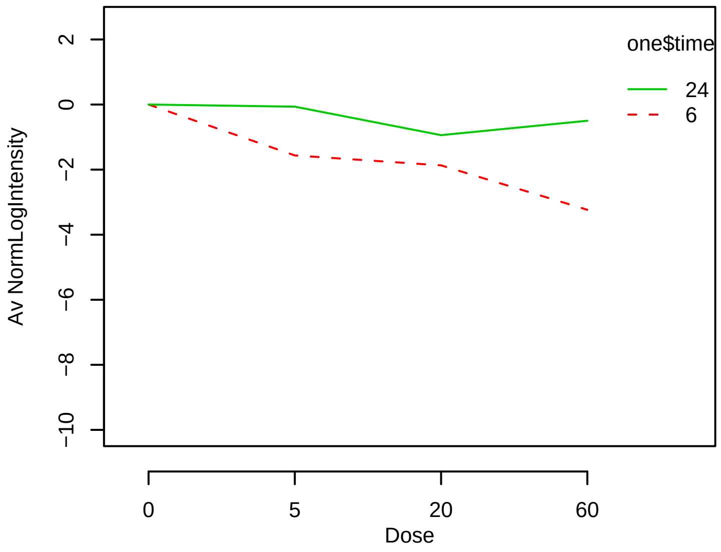

GO_0042355 : L−fucose catabolism

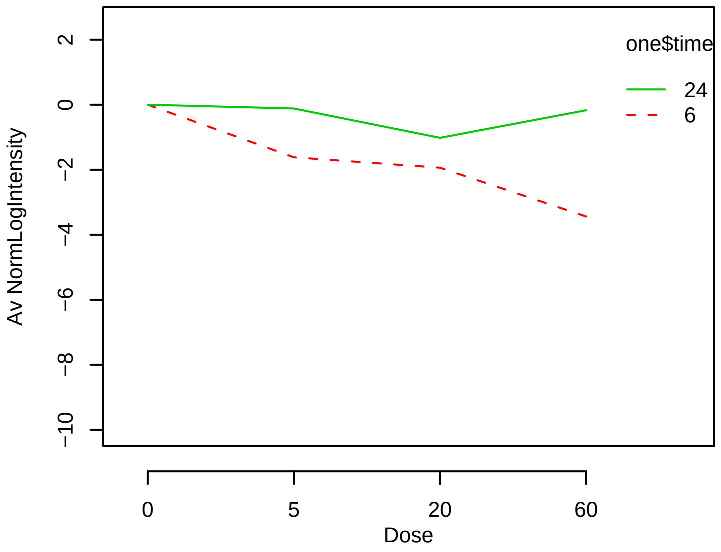

GO_0042364 : water−soluble vitamin biosynthesis

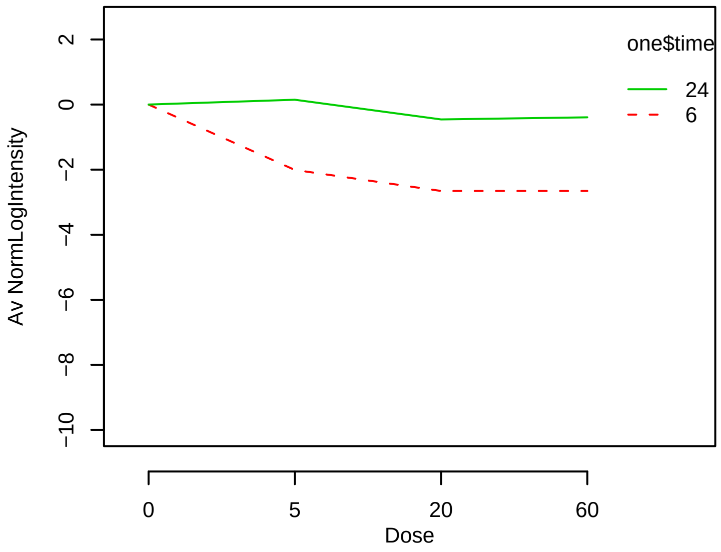

GO_0042375 : quinone cofactor metabolism

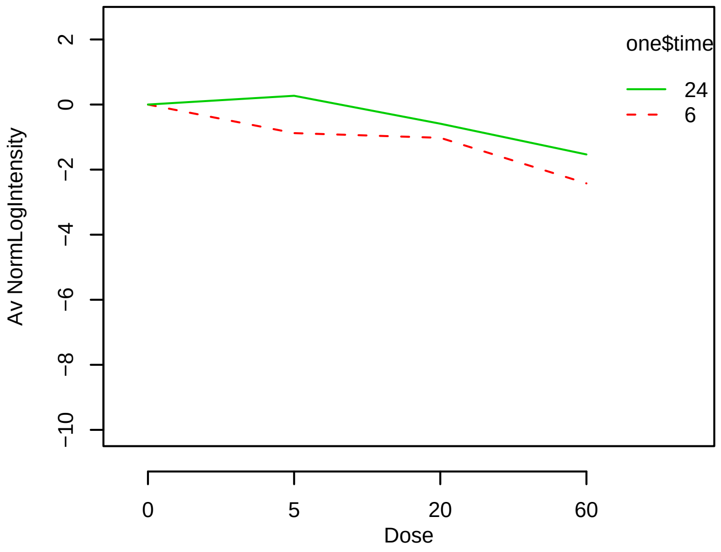

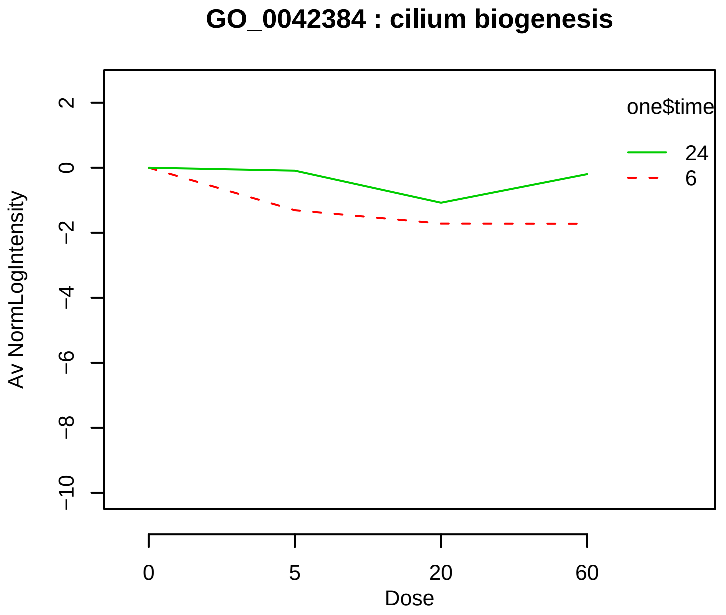
GO_0042384 : cilium biogenesis
one$time
24
6
Av NormLogIntensity
Dose
0
5
20
60

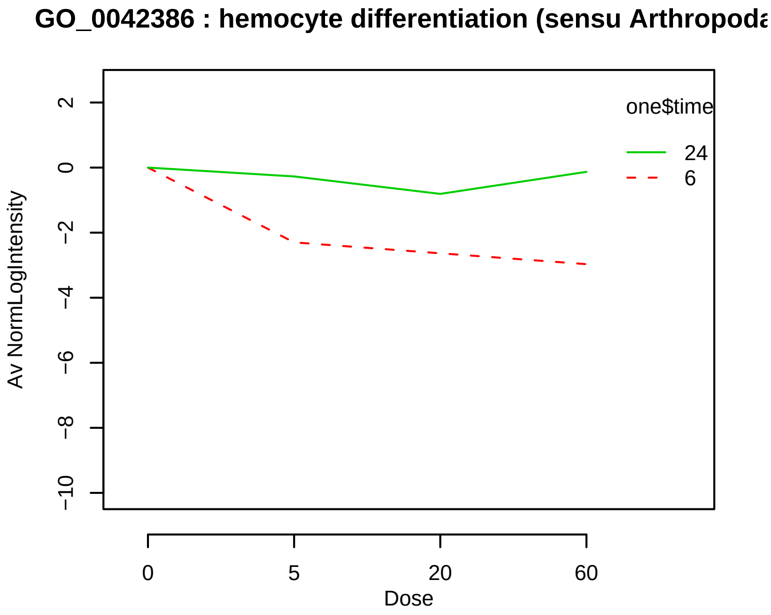
GO_0042386 : hemocyte differentiation (sensu Arthropoda
one$time
24
6
Av NormLogIntensity
Dose

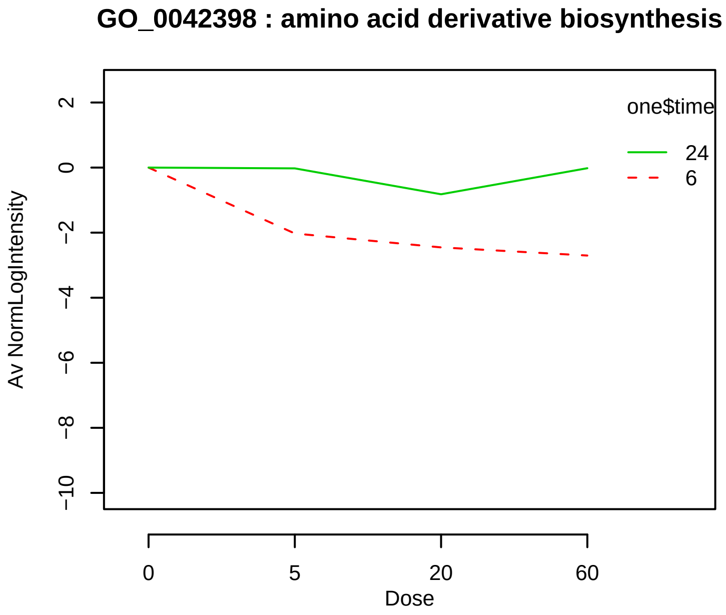
GO_0042398 : amino acid derivative biosynthesis
one$time
24
6
Av NormLogIntensity
Dose

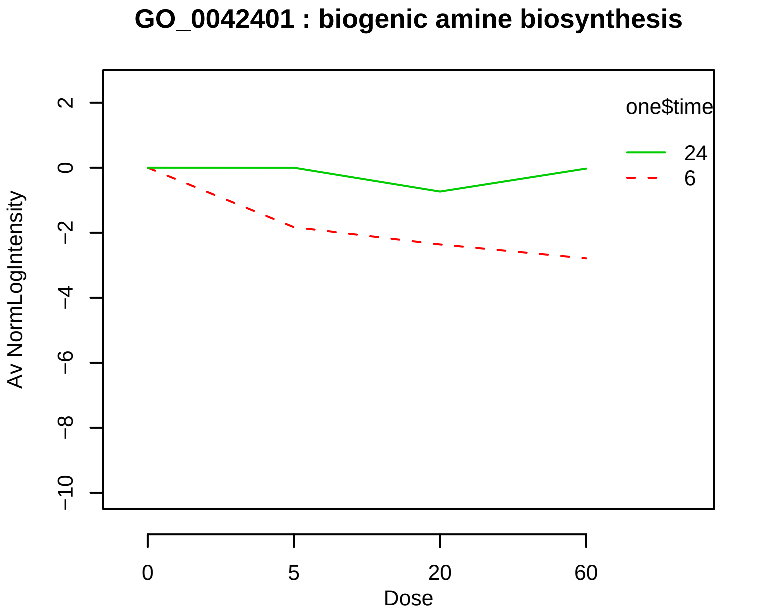
GO_0042401 : biogenic amine biosynthesis
one$time
24
6
Av NormLogIntensity
Dose

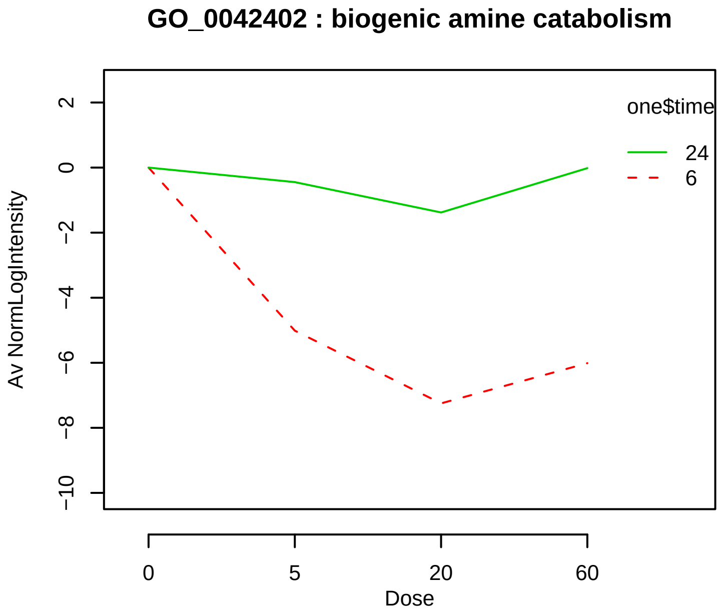
GO_0042402 : biogenic amine catabolism
one$time
24
6
Av NormLogIntensity
Dose

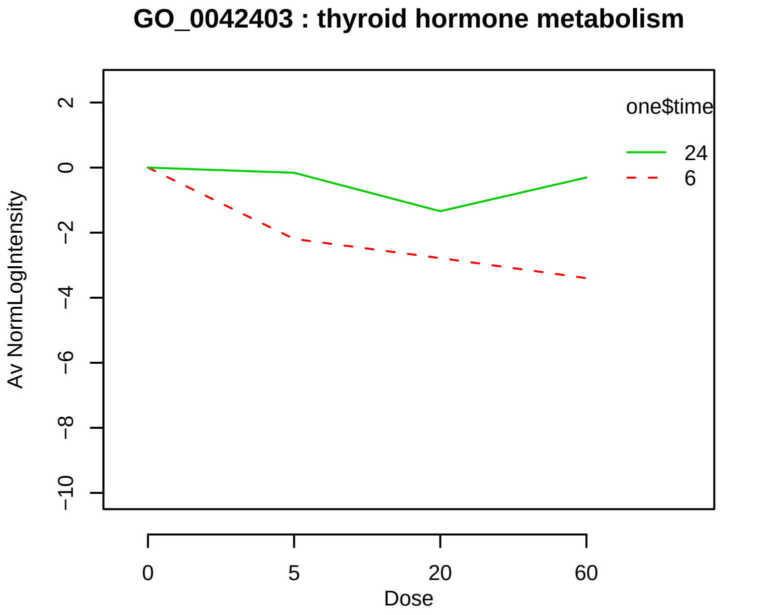
GO_0042403 : thyroid hormone metabolism
one$time
24
6
Av NormLogIntensity
Dose

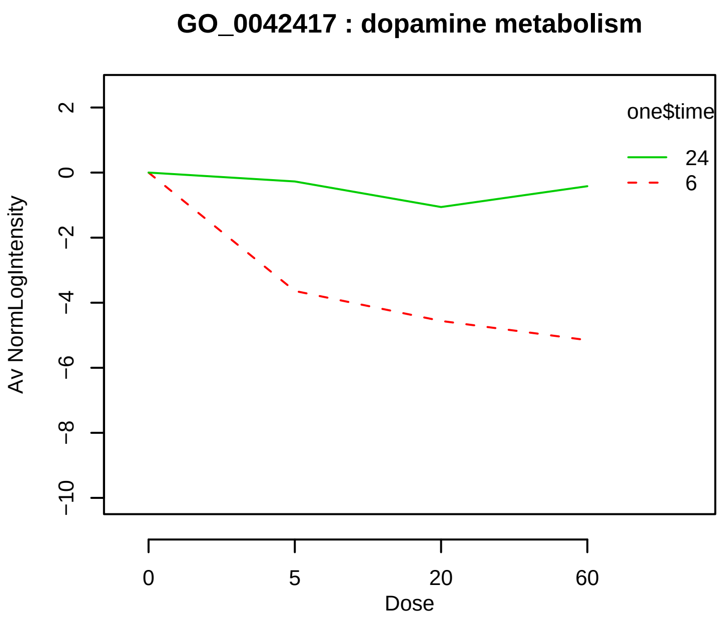
GO_0042417 : dopamine metabolism
Av NormLogIntensity
2
0
−2
−4
−6
−8
−10
one$time
24
6
0
5
20
60
Dose

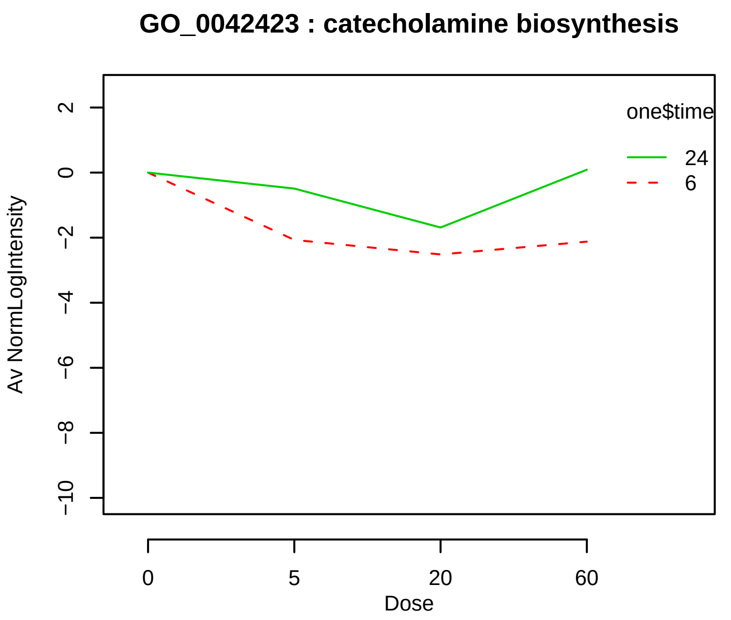
GO_0042423 : catecholamine biosynthesis
Av NormLogIntensity
2
0
−2
−4
−6
−8
−10
one$time
24
6
0
5
20
60
Dose

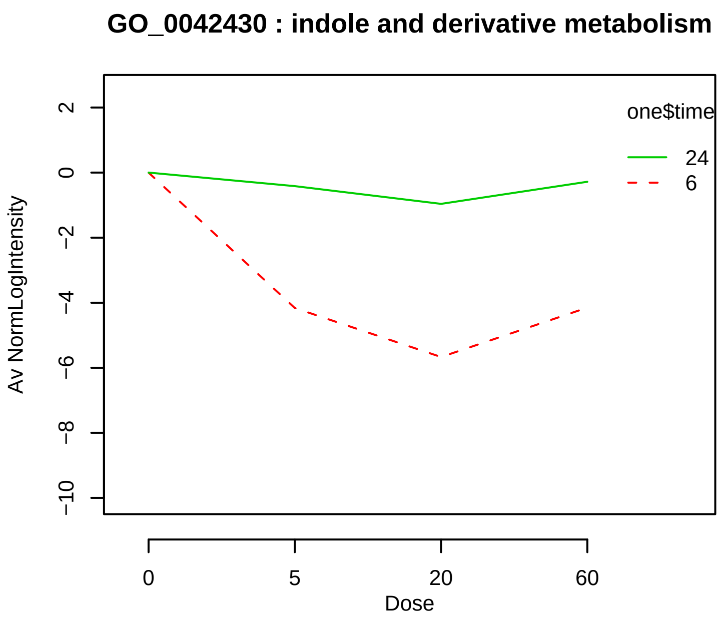
GO_0042430 : indole and derivative metabolism
Av NormLogIntensity
2
0
−2
−4
−6
−8
−10
one$time
24
6
0
5
20
60
Dose

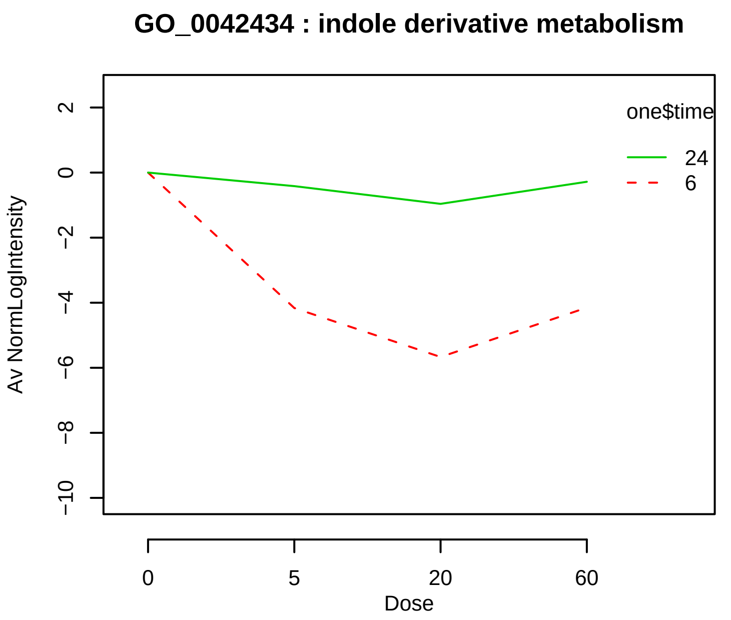
GO_0042434 : indole derivative metabolism
Av NormLogIntensity
2
0
−2
−4
−6
−8
−10
one$time
24
6
0
5
20
60
Dose

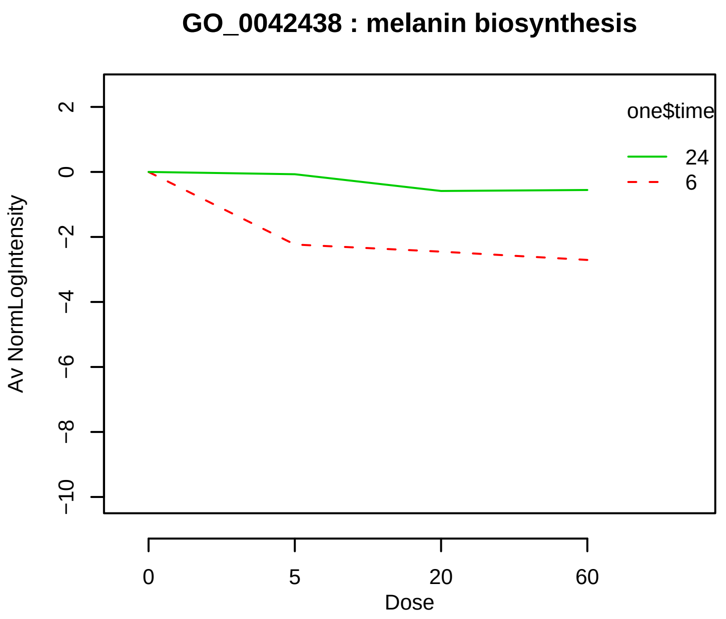
GO_0042438 : melanin biosynthesis
Av NormLogIntensity
2
0
−2
−4
−6
−8
−10
one$time
24
6
0
5
20
60
Dose

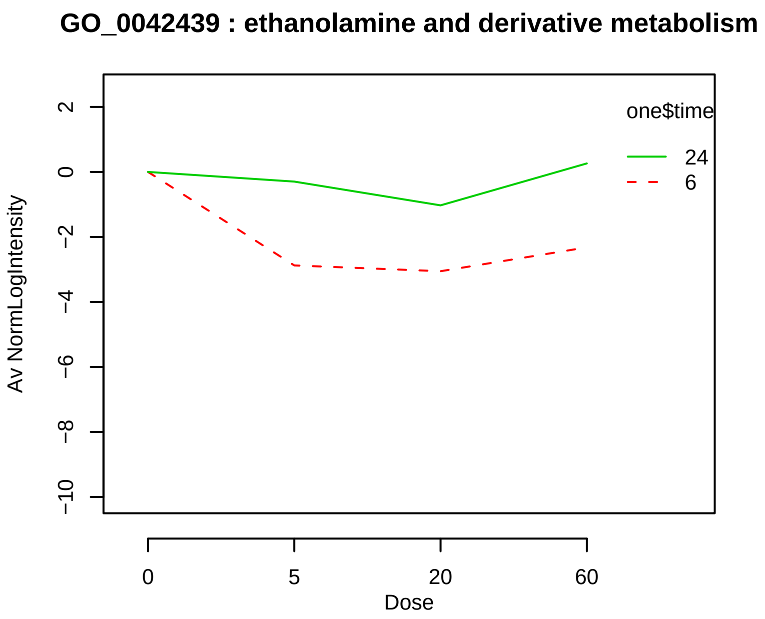
GO_0042439 : ethanolamine and derivative metabolism
Av NormLogIntensity
2
0
−2
−4
−6
−8
−10
one$time
24
6
0
5
20
60
Dose

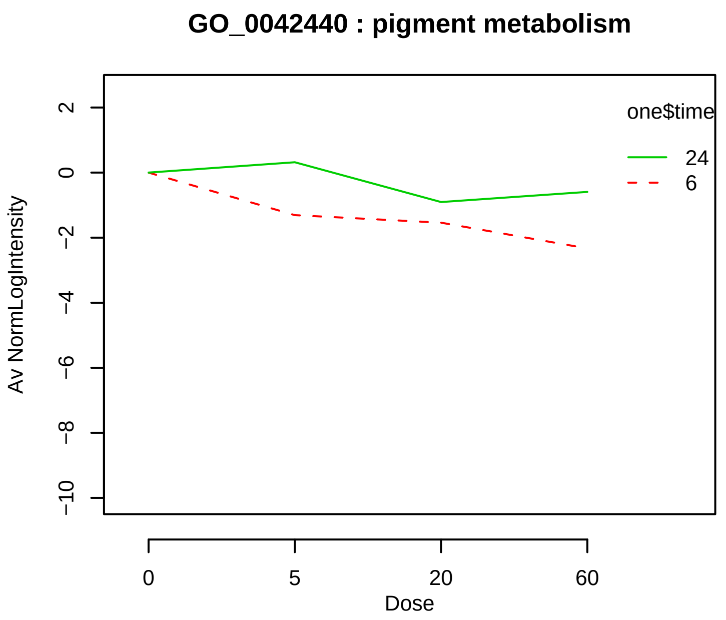
GO_0042440 : pigment metabolism
Av NormLogIntensity
2
0
−2
−4
−6
−8
−10
one$time
24
6
0
5
20
60
Dose

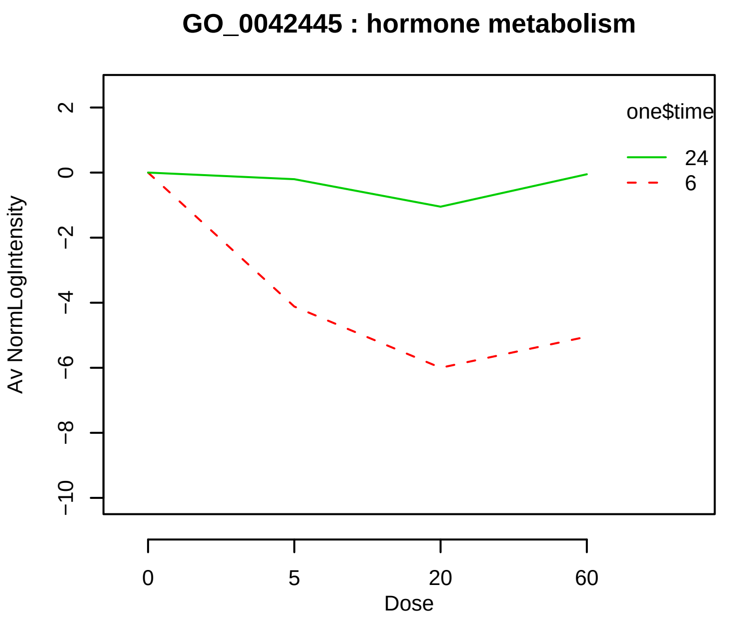
GO_0042445 : hormone metabolism
Av NormLogIntensity
2
0
−2
−4
−6
−8
−10
one$time
24
6
0
5
20
60
Dose

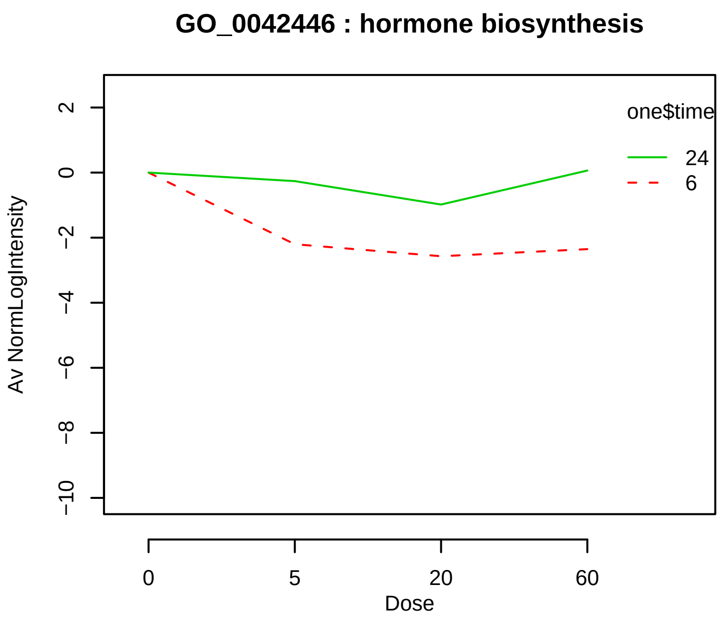
GO_0042446 : hormone biosynthesis
Av NormLogIntensity
2
0
−2
−4
−6
−8
−10
one$time
24
6
0
5
20
60
Dose

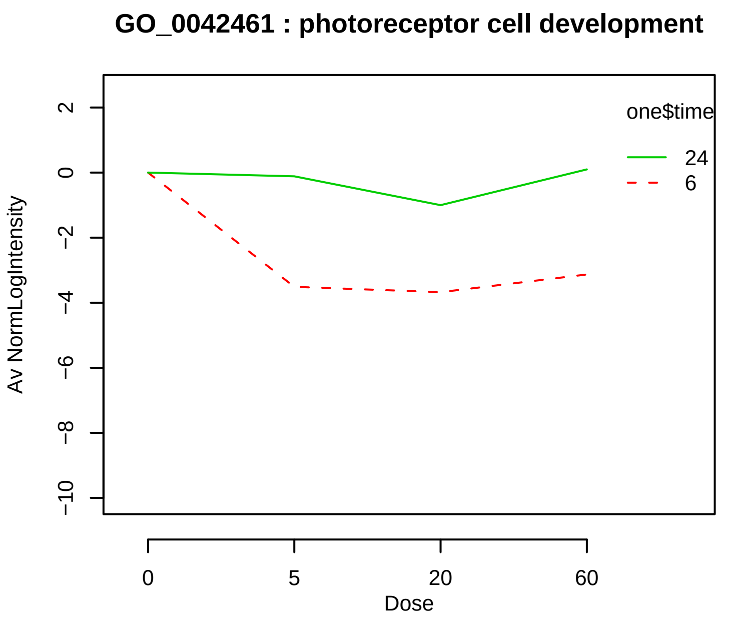
GO_0042461 : photoreceptor cell development
Av NormLogIntensity
2
0
−2
−4
−6
−8
−10
one$time
24
6
0
5
20
60
Dose

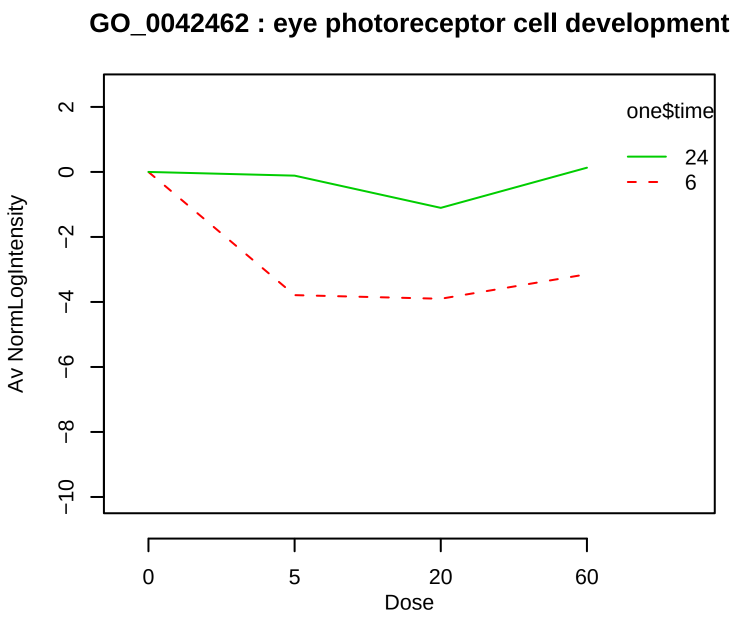
GO_0042462 : eye photoreceptor cell development
Av NormLogIntensity
2
0
−2
−4
−6
−8
−10
one$time
24
6
0
5
20
60
Dose

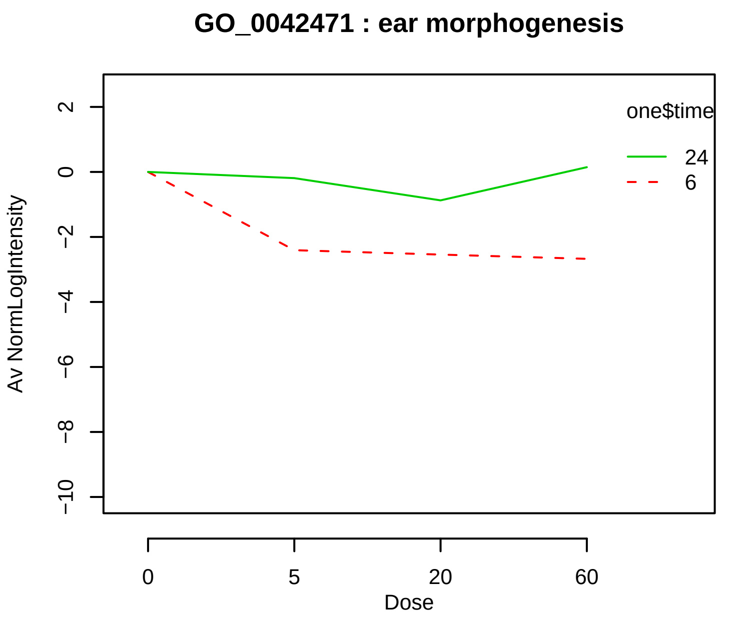
GO_0042471 : ear morphogenesis
Av NormLogIntensity
2
0
−2
−4
−6
−8
−10
one$time
24
6
0
5
20
60
Dose

GO_0042472 : inner ear morphogenesis

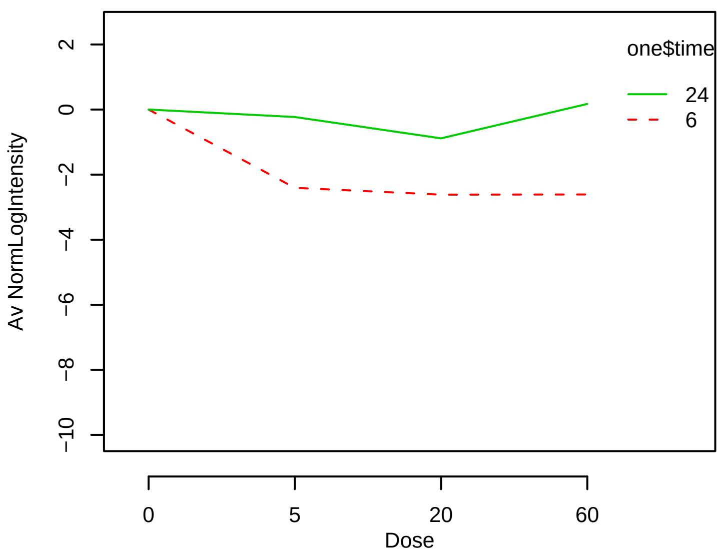

GO_0042475 : odontogenesis (sensu Vertebrata)

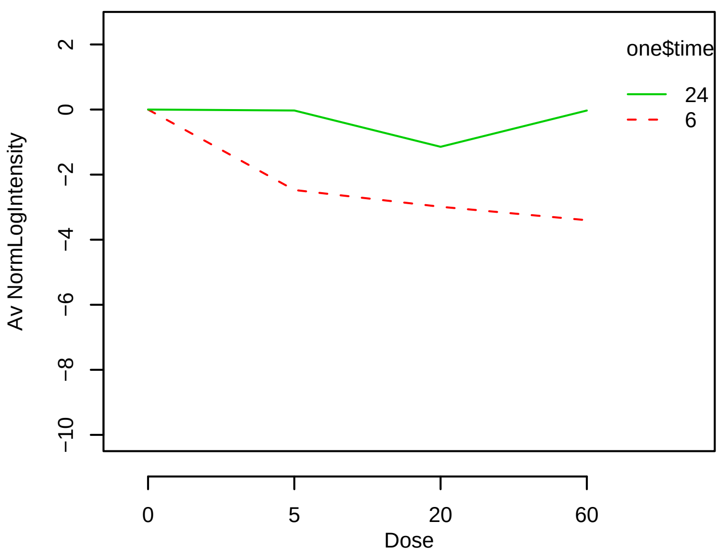

GO_0042476 : odontogenesis

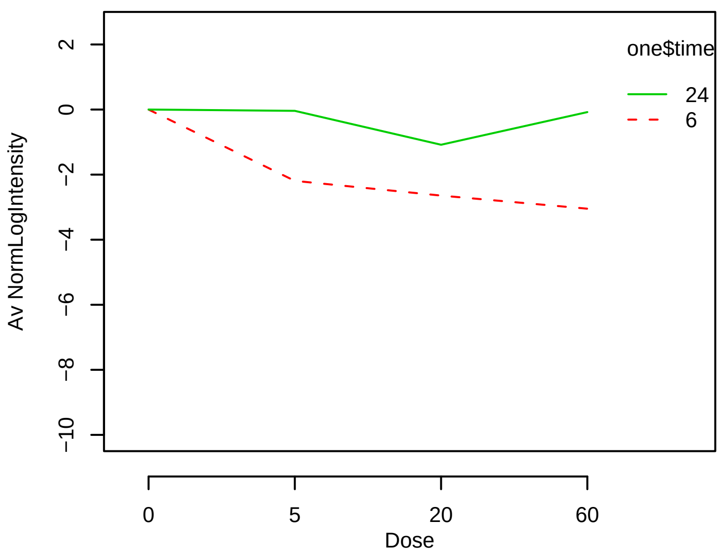

GO_0042490 : mechanoreceptor differentiation

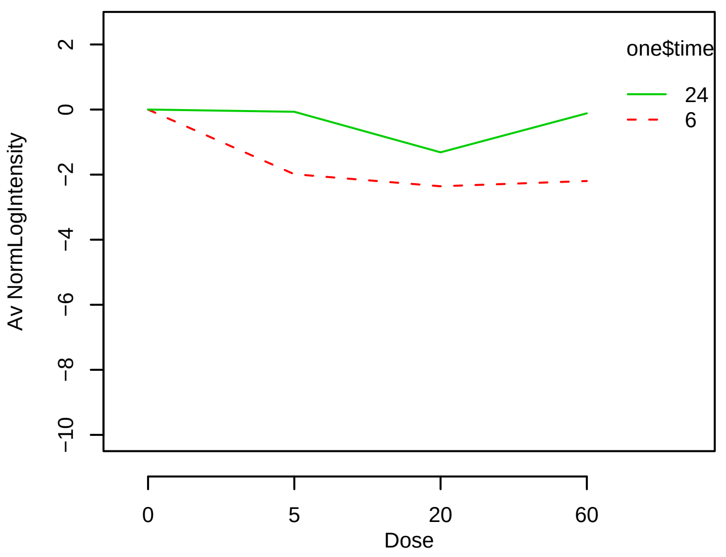

GO_0042491 : auditory receptor cell differentiation

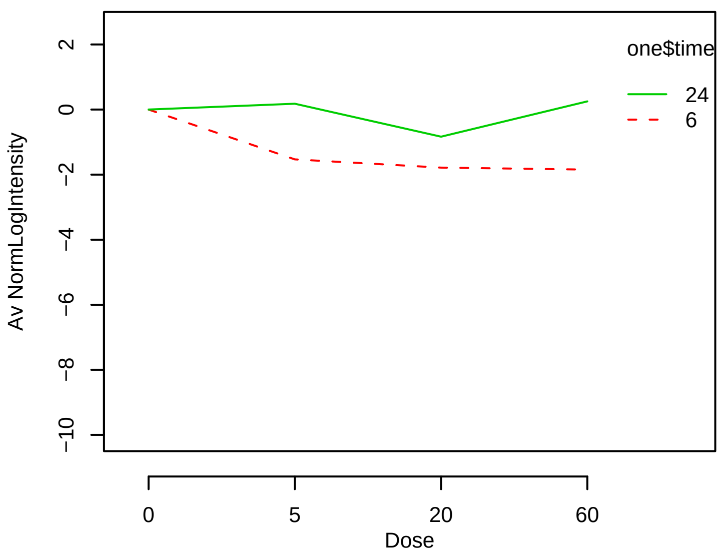

GO_0042493 : response to drug

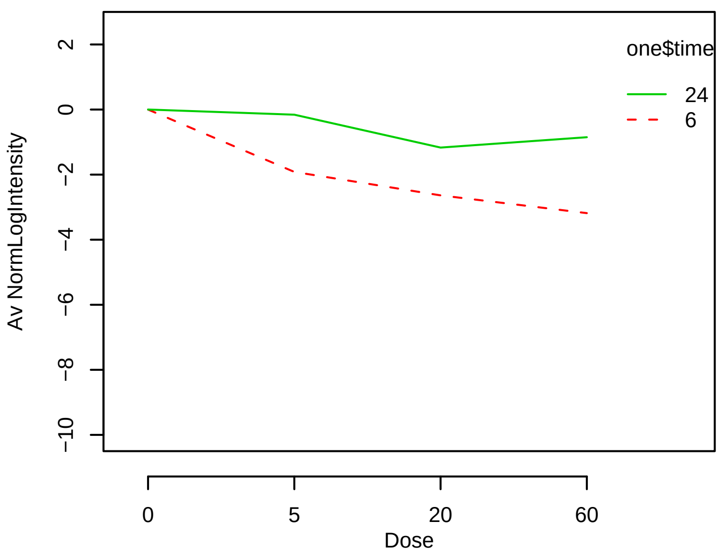

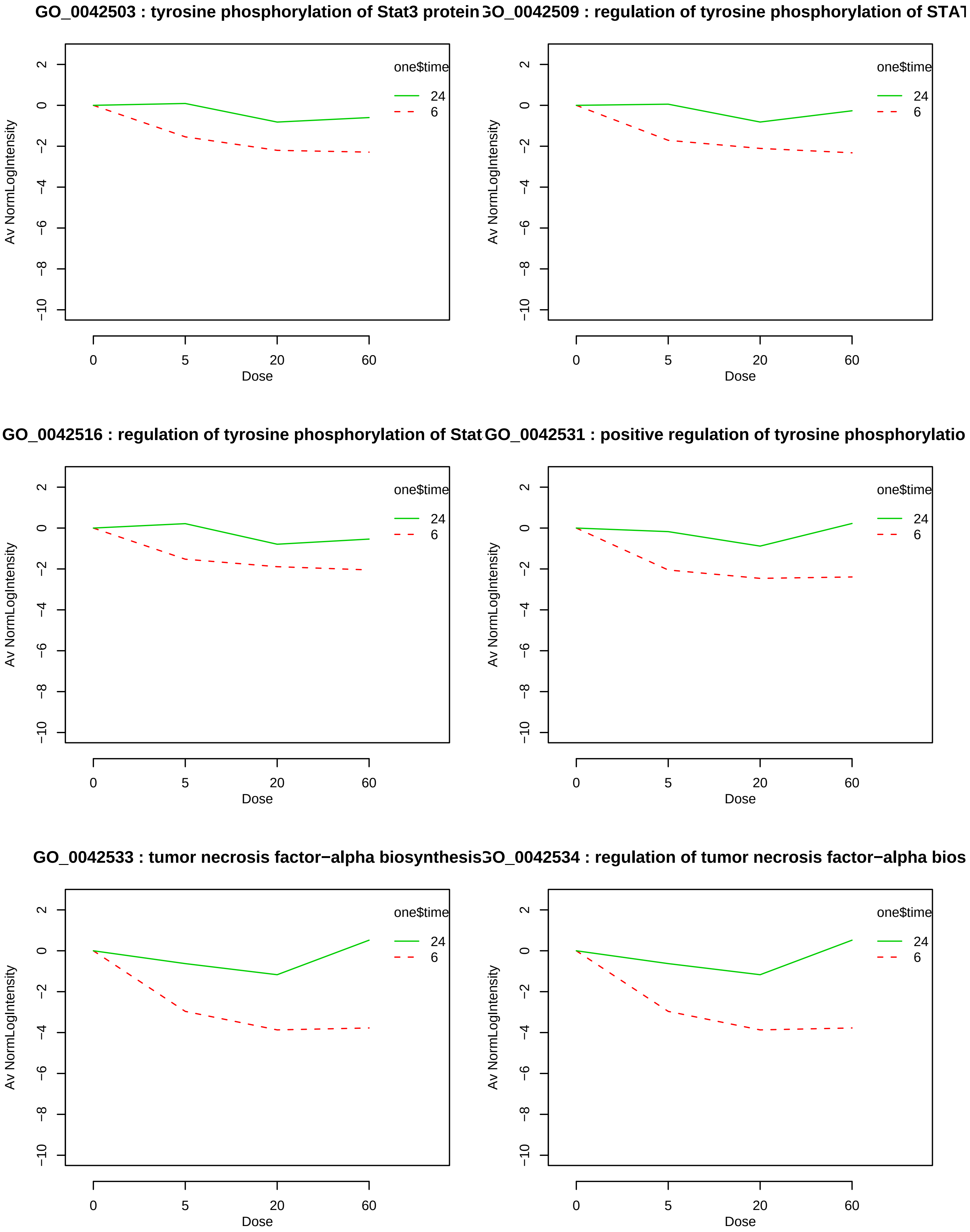
GO_0042503 : tyrosine phosphorylation of Stat3 protein
GO_0042509 : regulation of tyrosine phosphorylation of STAT
GO_0042516 : regulation of tyrosine phosphorylation of Stat
GO_0042531 : positive regulation of tyrosine phosphorylatio
GO_0042533 : tumor necrosis factor−alpha biosynthesis
GO_0042534 : regulation of tumor necrosis factor−alpha bios
Av NormLogIntensity
Dose
one$time
24
6
0
5
20
60
2
0
−2
−4
−6
−8
−10

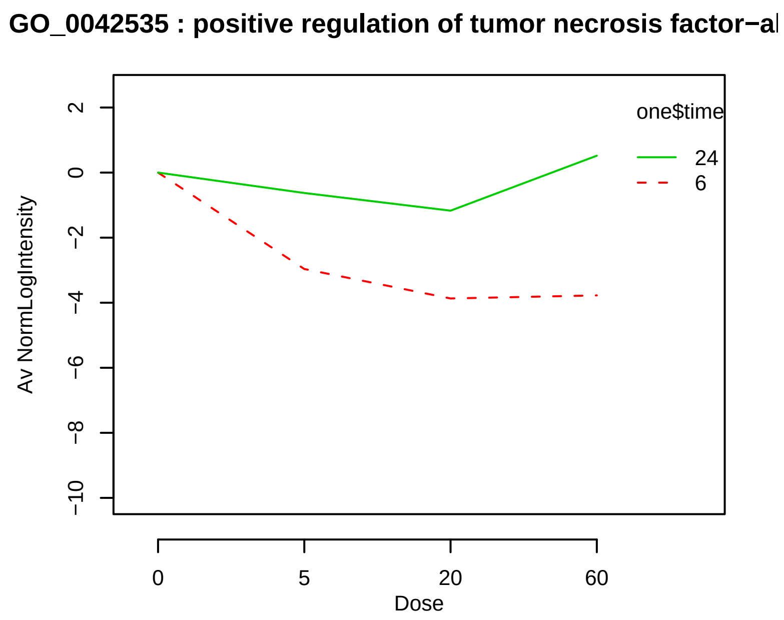
GO_0042535 : positive regulation of tumor necrosis factor−al
one$time
24
6
Av NormLogIntensity
Dose
0
5
20
60

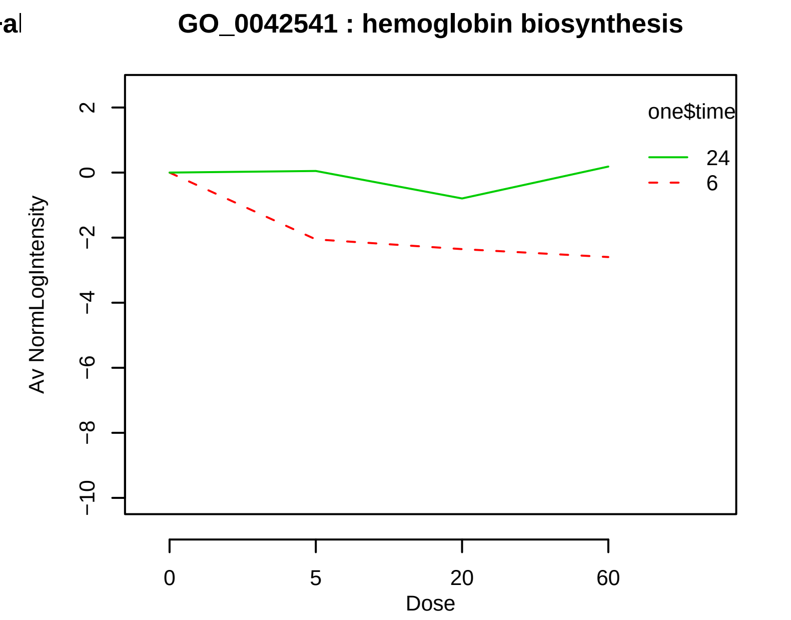
GO_0042541 : hemoglobin biosynthesis
one$time
24
6
Av NormLogIntensity
Dose

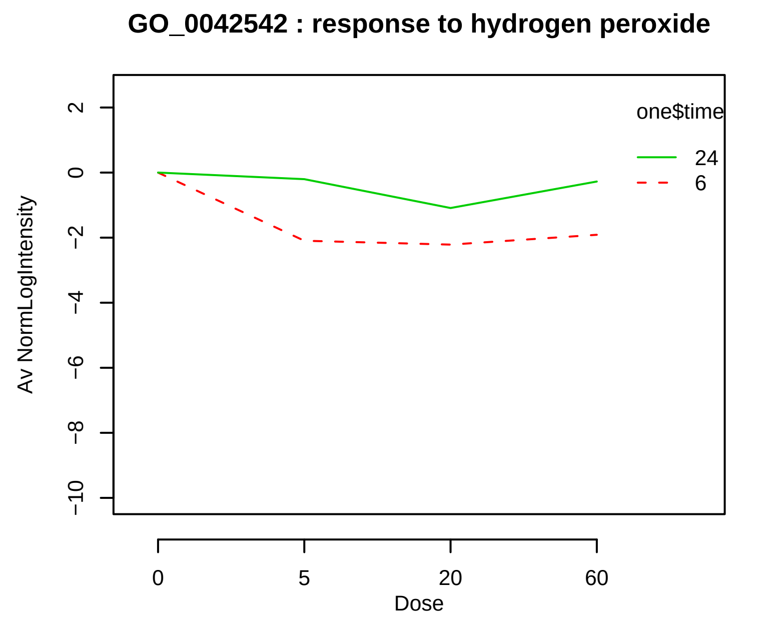
GO_0042542 : response to hydrogen peroxide
one$time
24
6
Av NormLogIntensity
Dose

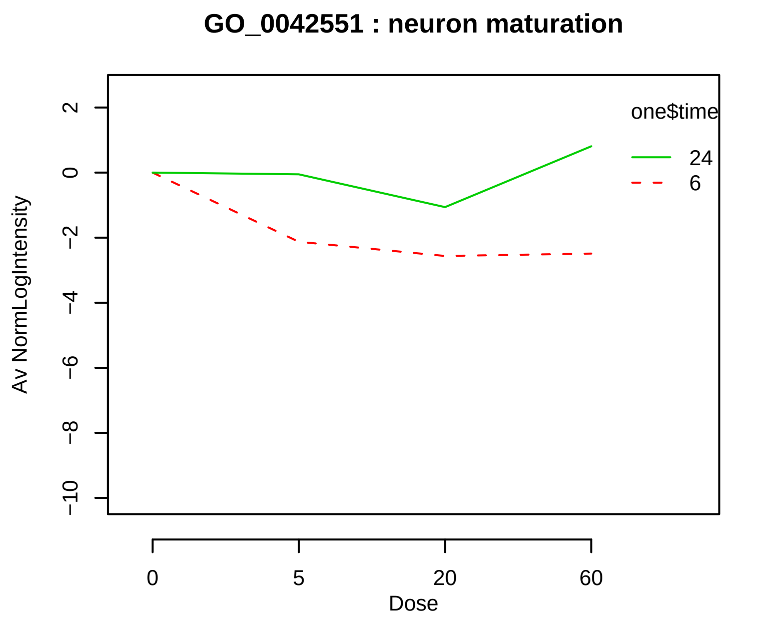
GO_0042551 : neuron maturation
one$time
24
6
Av NormLogIntensity
Dose

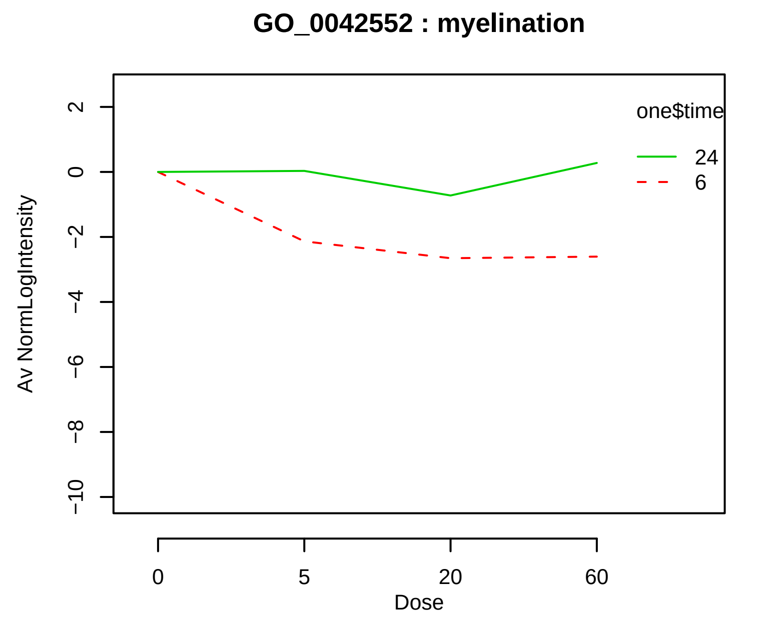
GO_0042552 : myelination
one$time
24
6
Av NormLogIntensity
Dose

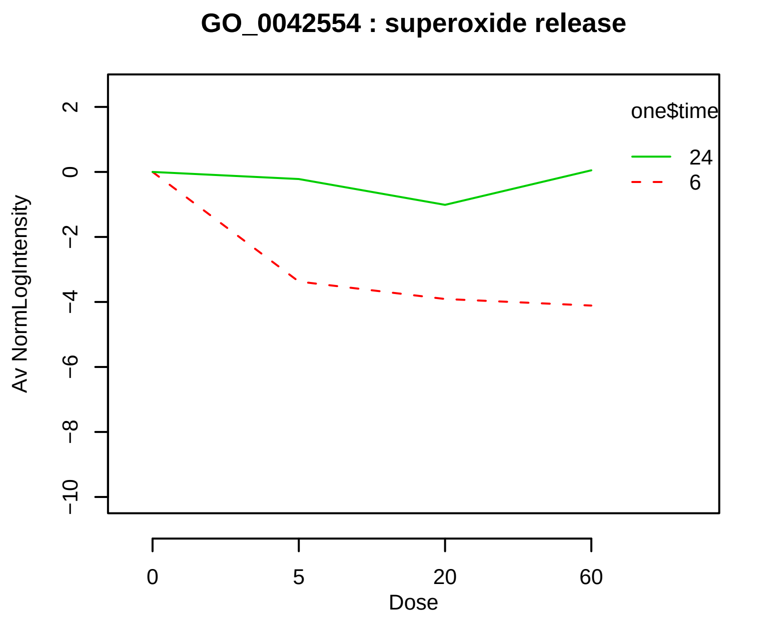
GO_0042554 : superoxide release
one$time
24
6
Av NormLogIntensity
Dose

GO_0042558 : pteridine and derivative metabolism

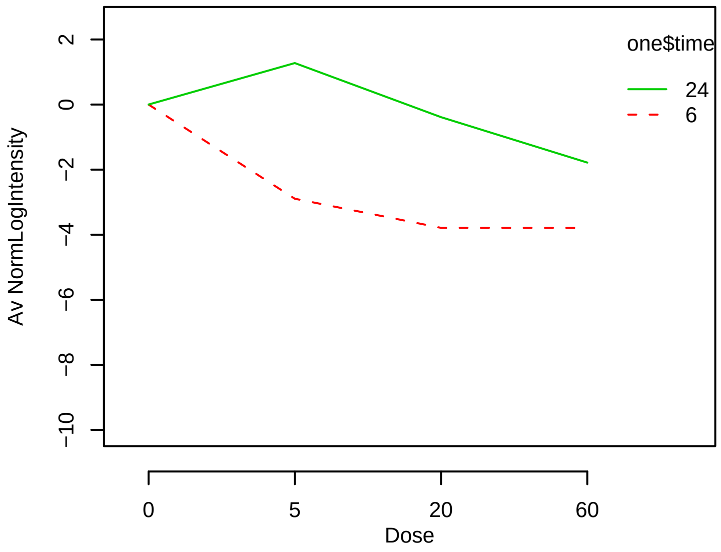

GO_0042559 : pteridine and derivative biosynthesis

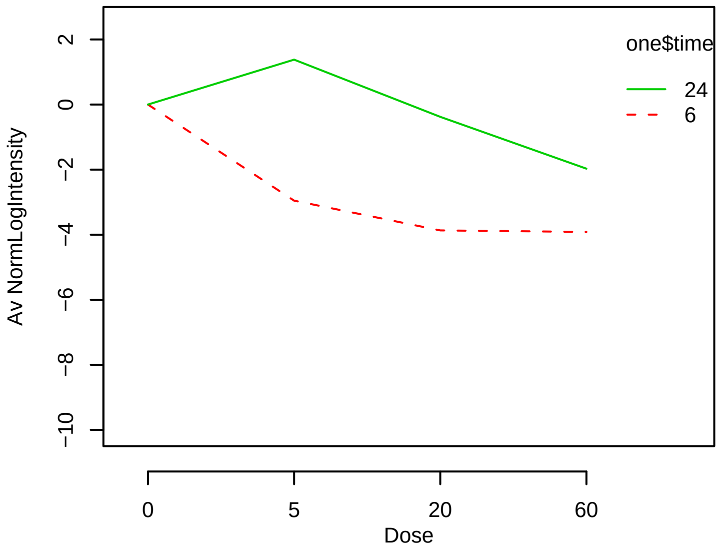

GO_0042572 : retinol metabolism

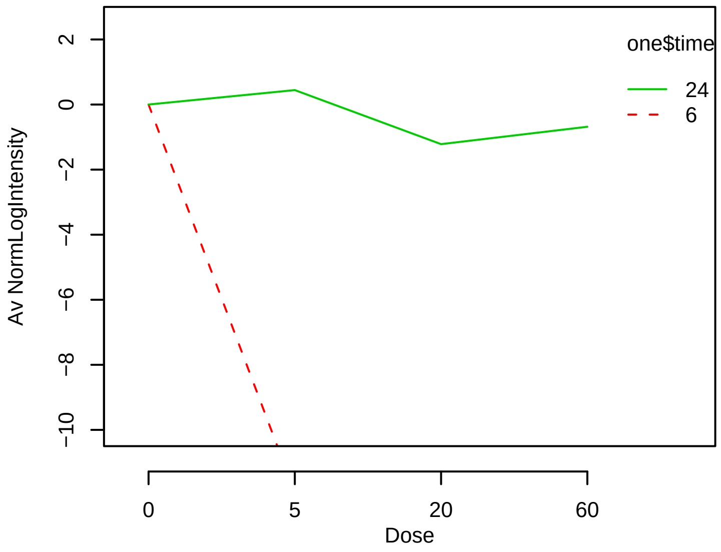

GO_0042573 : retinoic acid metabolism

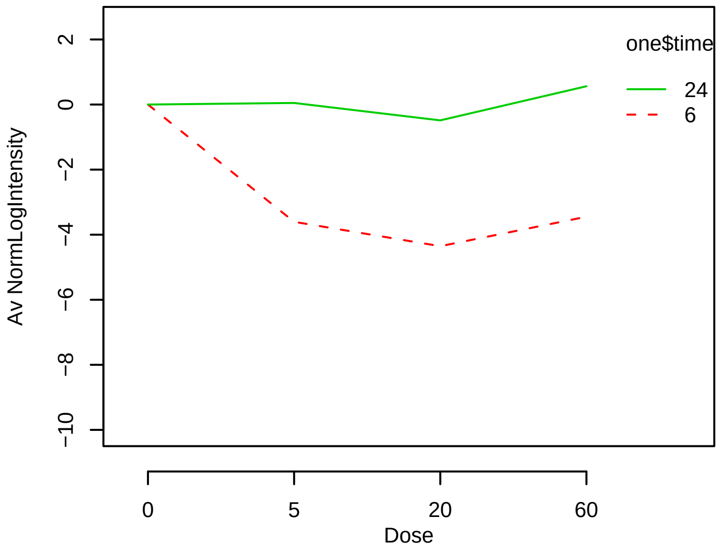

GO_0042593 : glucose homeostasis

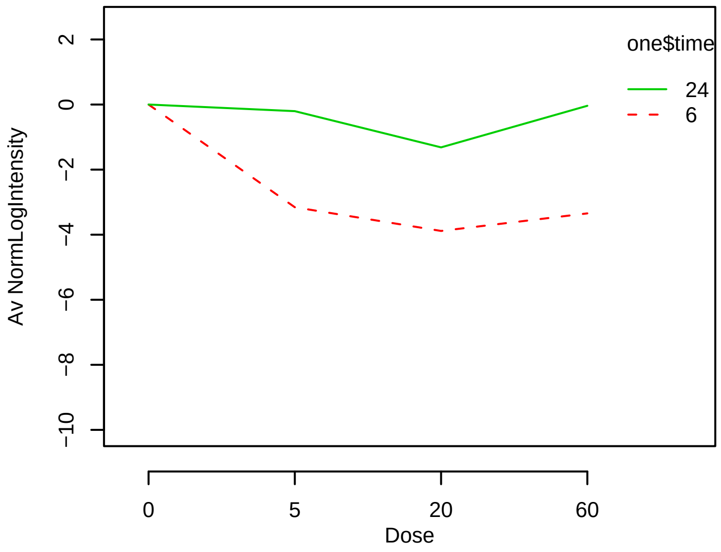

GO_0042594 : response to starvation

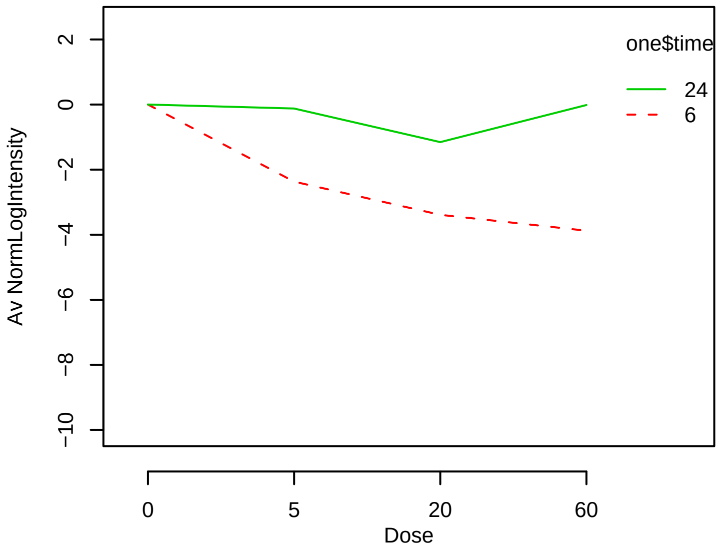

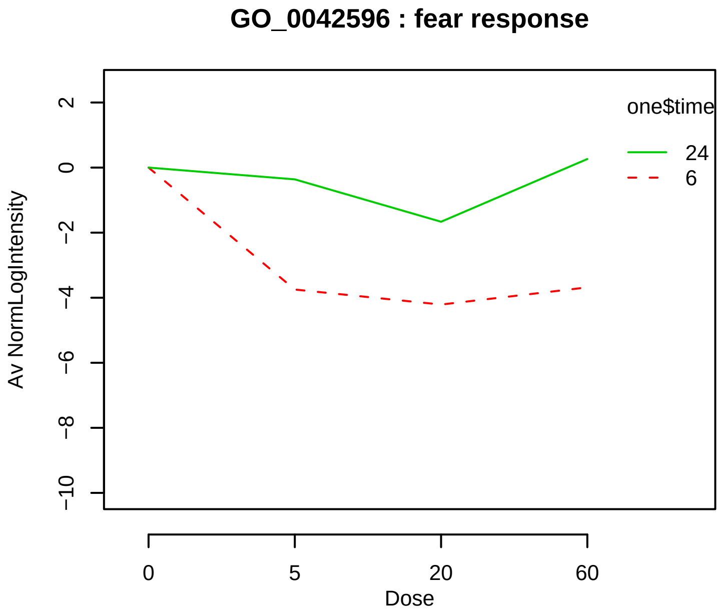

GO_0042596 : fear response
one$time
24
6
Av NormLogIntensity
Dose
0
5
20
60
2
0
−2
−4
−6
−8
−10

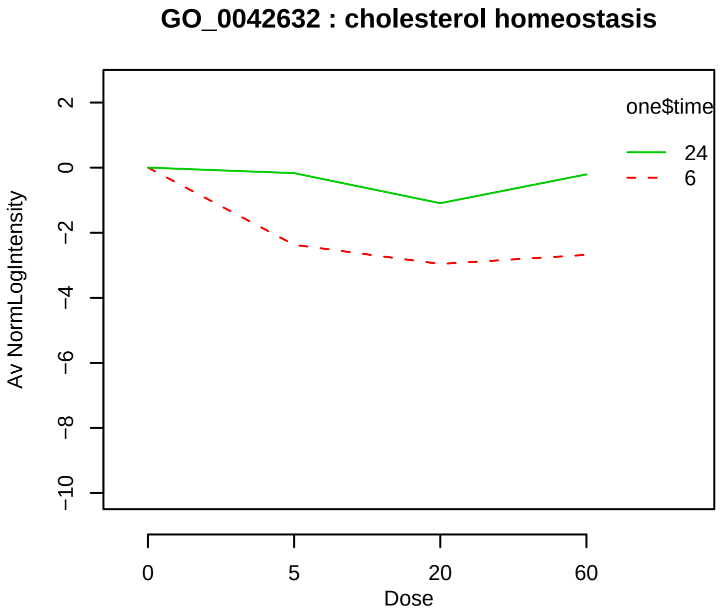

GO_0042632 : cholesterol homeostasis
one$time
24
6
Av NormLogIntensity
Dose
0
5
20
60
2
0
−2
−4
−6
−8
−10

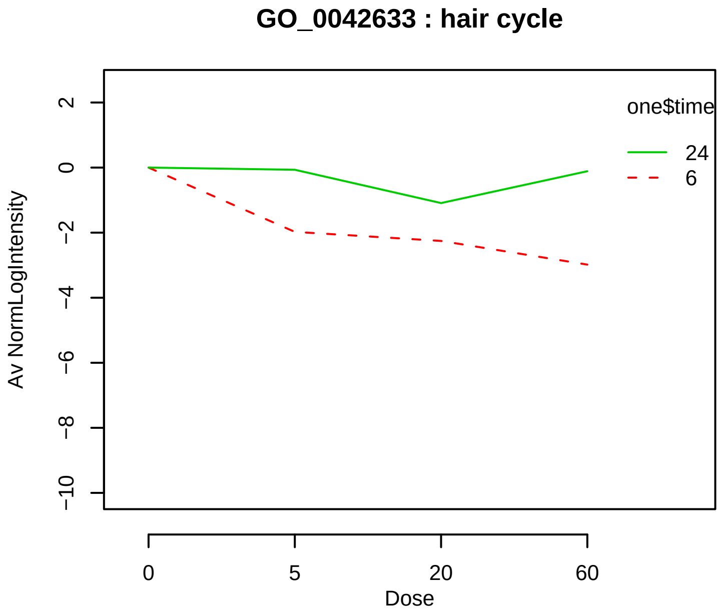

GO_0042633 : hair cycle
one$time
24
6
Av NormLogIntensity
Dose
0
5
20
60
2
0
−2
−4
−6
−8
−10

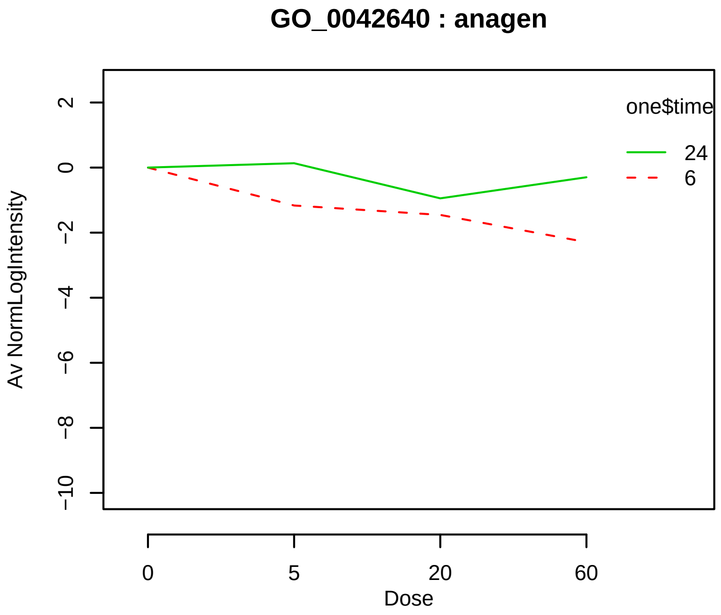

GO_0042640 : anagen
one$time
24
6
Av NormLogIntensity
Dose
0
5
20
60
2
0
−2
−4
−6
−8
−10

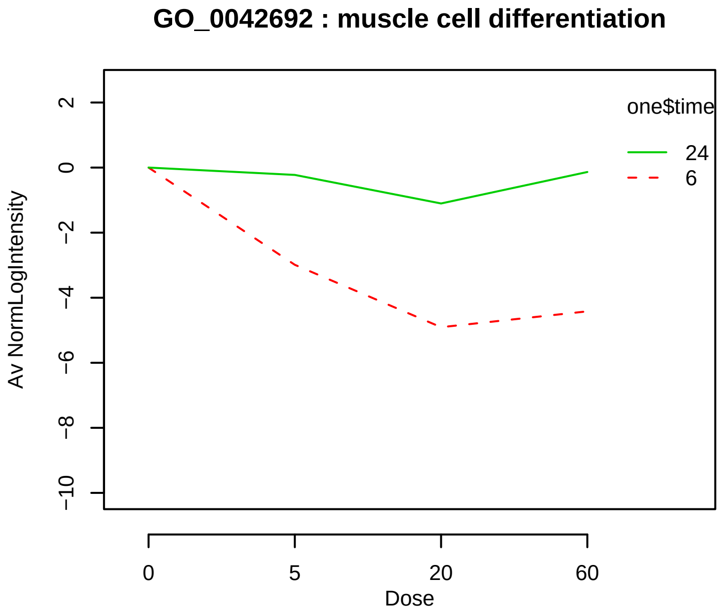

GO_0042692 : muscle cell differentiation
one$time
24
6
Av NormLogIntensity
Dose
0
5
20
60
2
0
−2
−4
−6
−8
−10

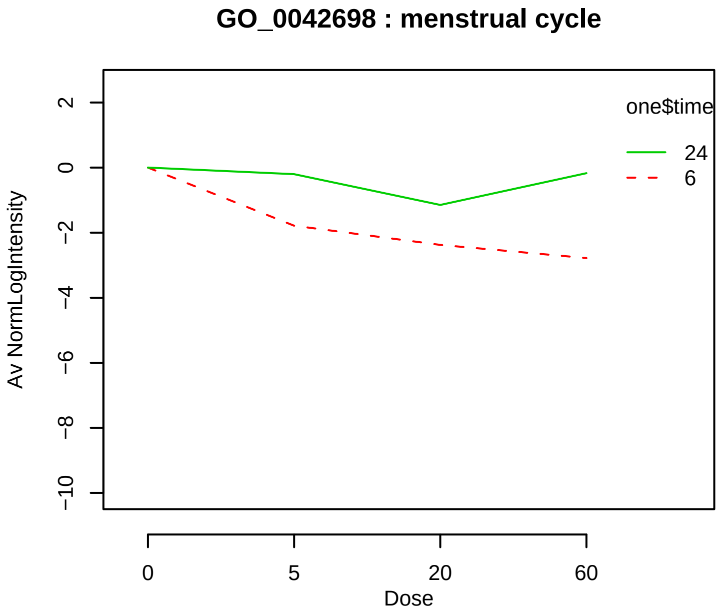

GO_0042698 : menstrual cycle
one$time
24
6
Av NormLogIntensity
Dose
0
5
20
60
2
0
−2
−4
−6
−8
−10

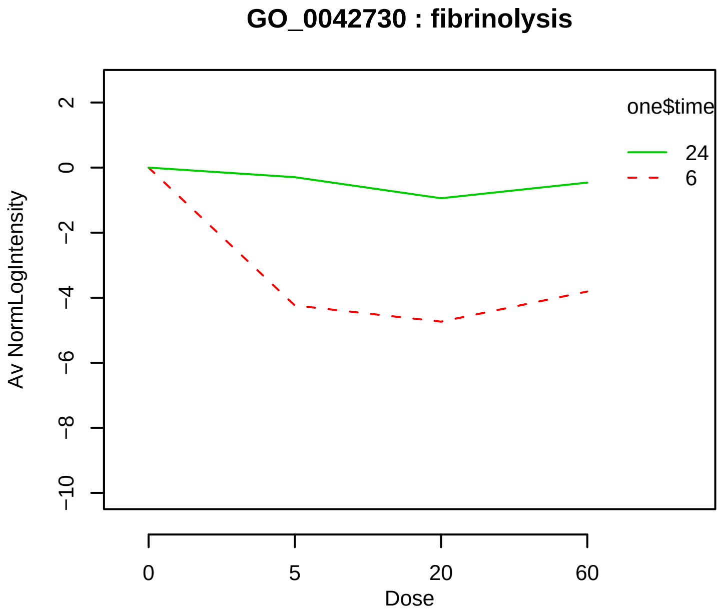
GO_0042730 : fibrinolysis
Av NormLogIntensity
Dose
one$time
24
6
2
0
−2
−4
−6
−8
−10
0
5
20
60

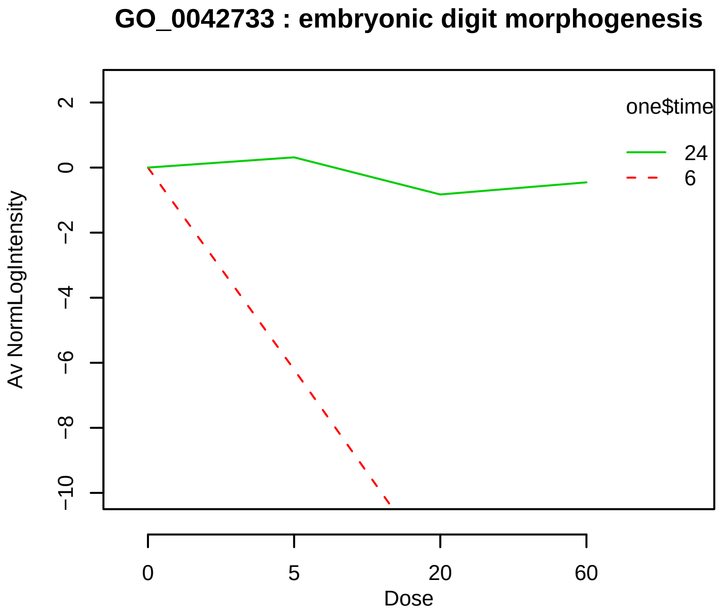
GO_0042733 : embryonic digit morphogenesis
Av NormLogIntensity
Dose
one$time
24
6
2
0
−2
−4
−6
−8
−10
0
5
20
60

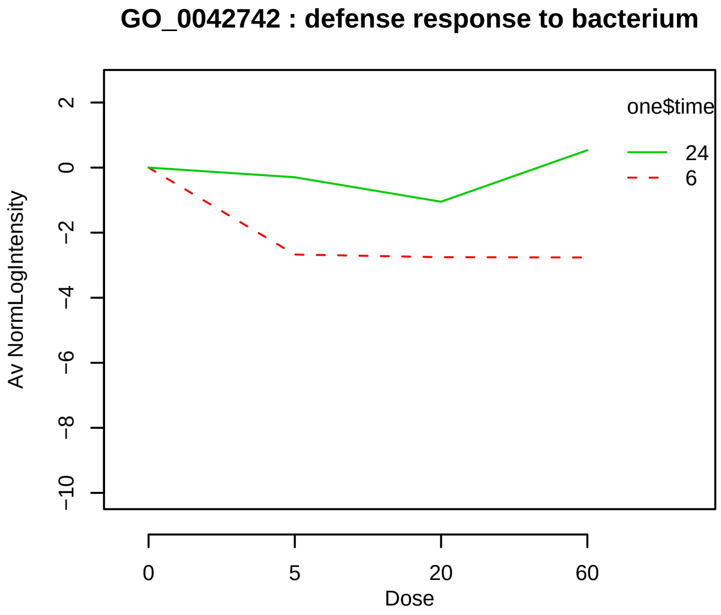
GO_0042742 : defense response to bacterium
Av NormLogIntensity
Dose
one$time
24
6
2
0
−2
−4
−6
−8
−10
0
5
20
60

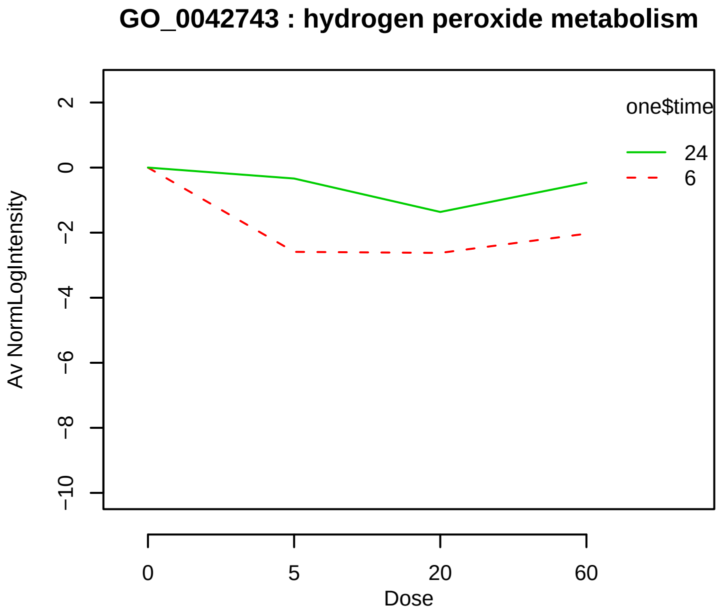
GO_0042743 : hydrogen peroxide metabolism
Av NormLogIntensity
Dose
one$time
24
6
2
0
−2
−4
−6
−8
−10
0
5
20
60

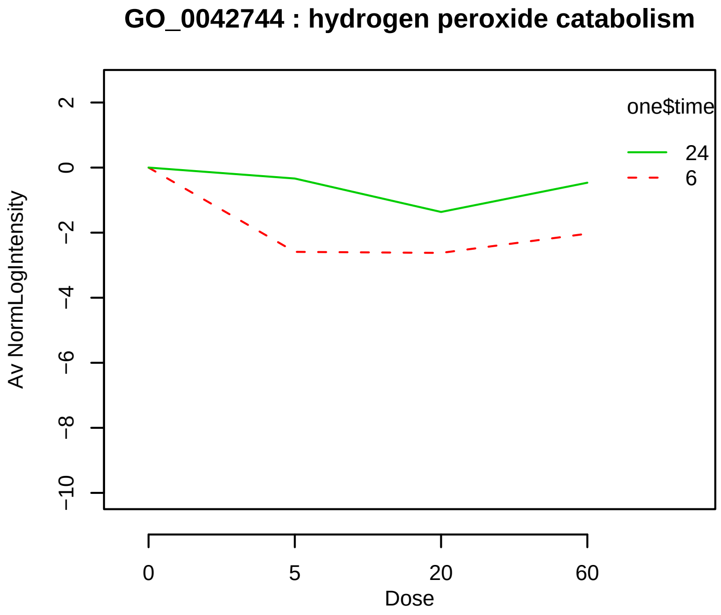
GO_0042744 : hydrogen peroxide catabolism
Av NormLogIntensity
Dose
one$time
24
6
2
0
−2
−4
−6
−8
−10
0
5
20
60

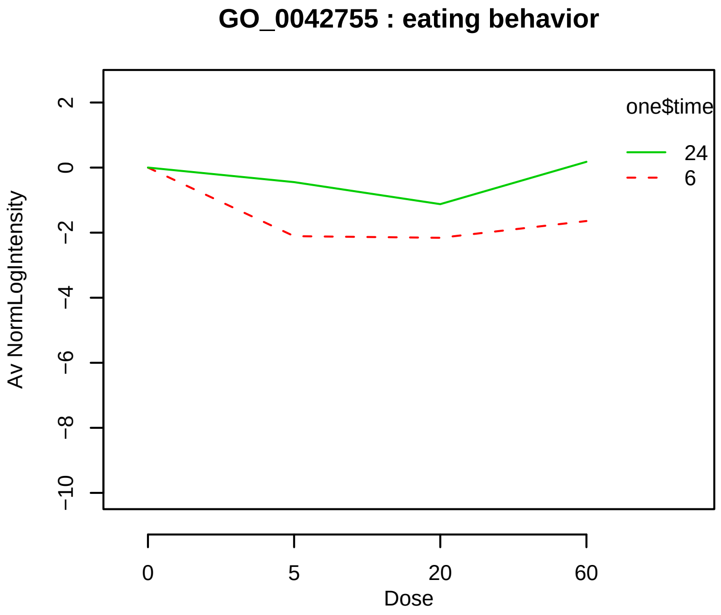
GO_0042755 : eating behavior
Av NormLogIntensity
Dose
one$time
24
6
2
0
−2
−4
−6
−8
−10
0
5
20
60

GO_0042769 : DNA damage response\, detection of DNA dam

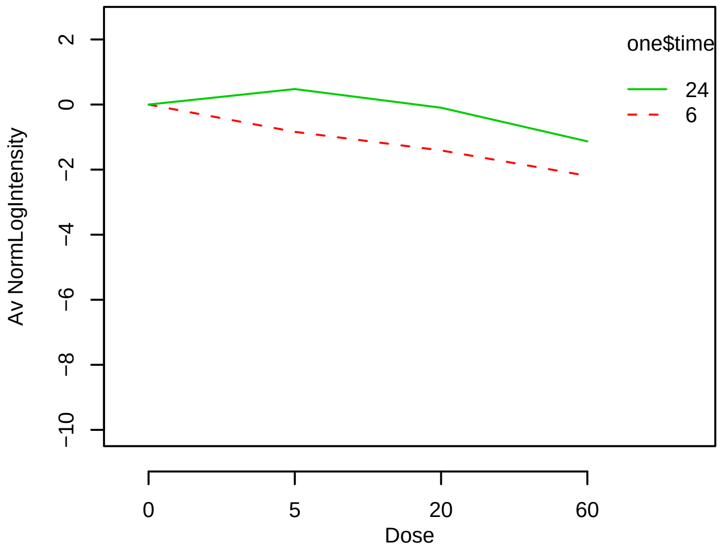

GO_0042770 : DNA damage response\, signal transductio

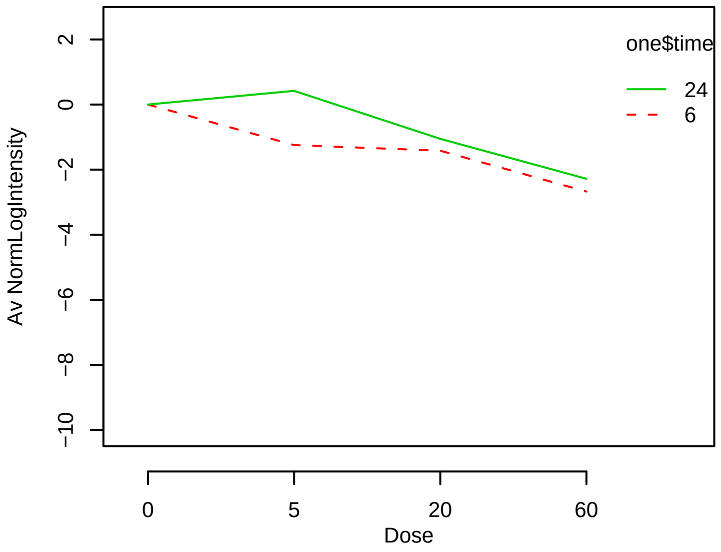

O_0042771 : DNA damage response\, signal transduction by

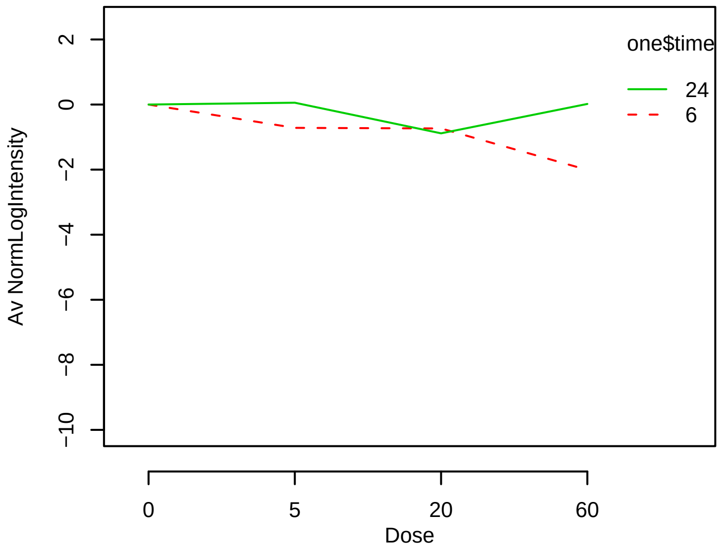

GO_0042773 : ATP synthesis coupled electron transport

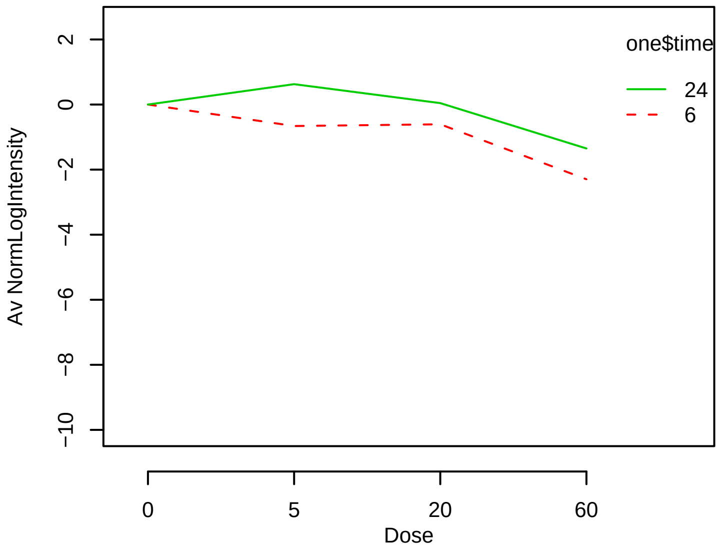

O_0042775 : ATP synthesis coupled electron transport (sens

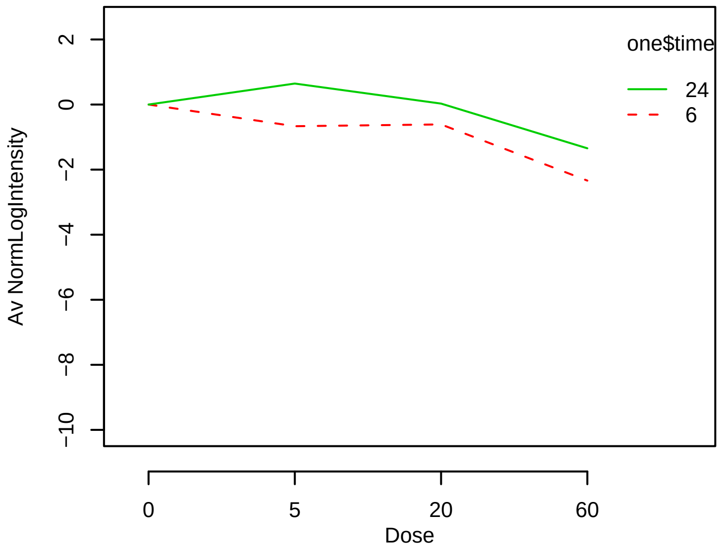

GO_0042787 : protein ubiquitination during ubiquitin−depend

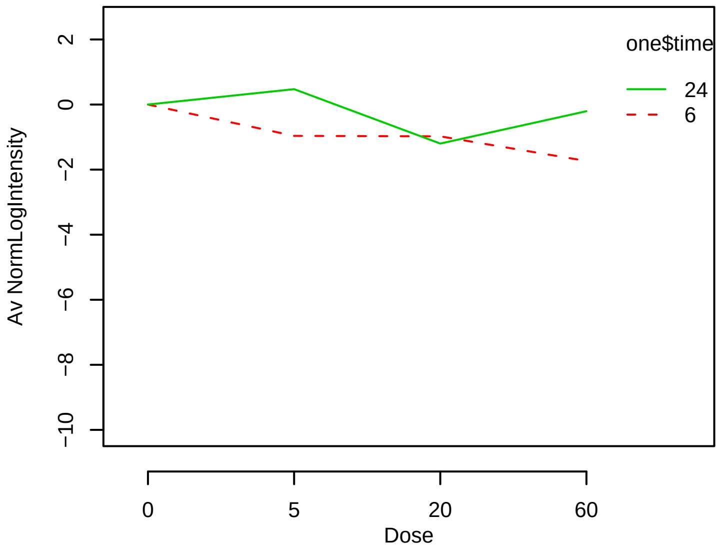

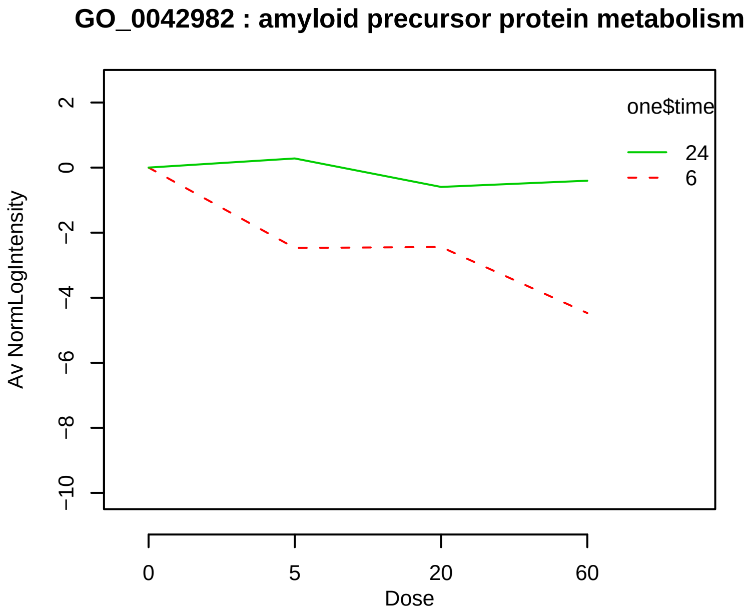
GO_0042982 : amyloid precursor protein metabolism
one$time
24
6
Av NormLogIntensity
2
0
−2
−4
−6
−8
−10
0
5
20
60
Dose

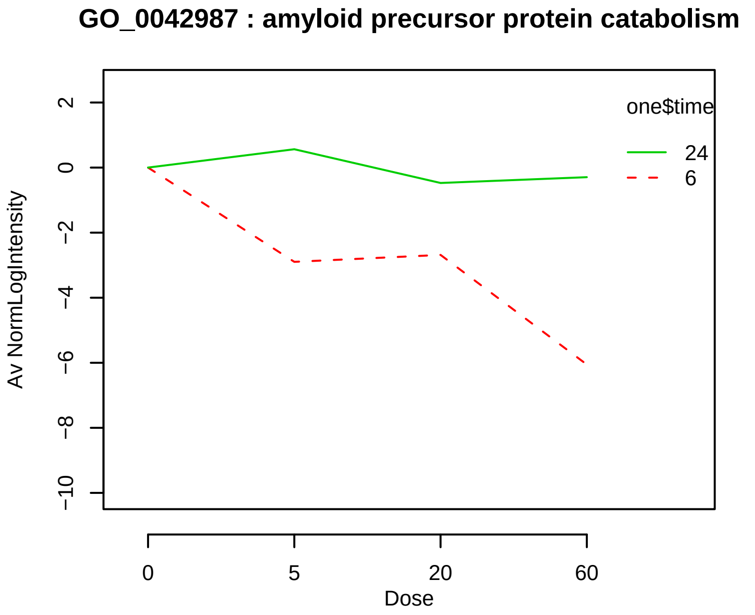
GO_0042987 : amyloid precursor protein catabolism
one$time
24
6
Av NormLogIntensity
2
0
−2
−4
−6
−8
−10
0
5
20
60
Dose

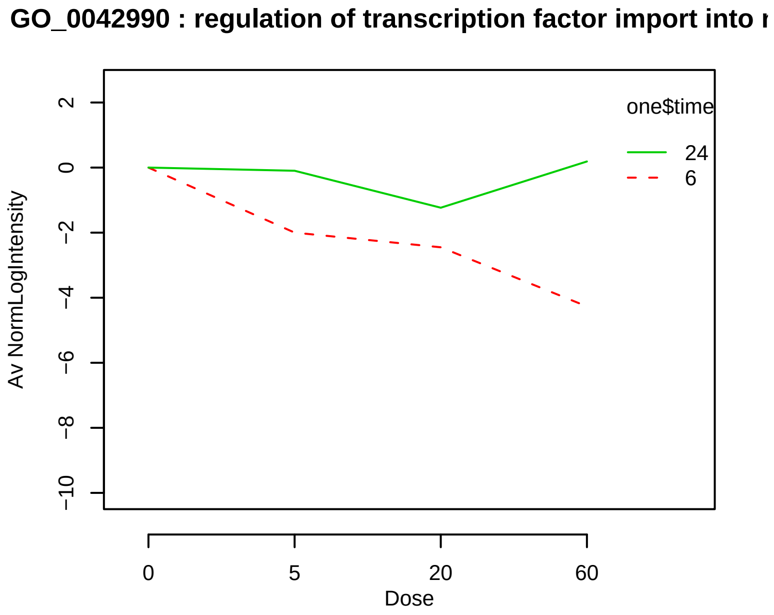
GO_0042990 : regulation of transcription factor import into r
one$time
24
6
Av NormLogIntensity
2
0
−2
−4
−6
−8
−10
0
5
20
60
Dose

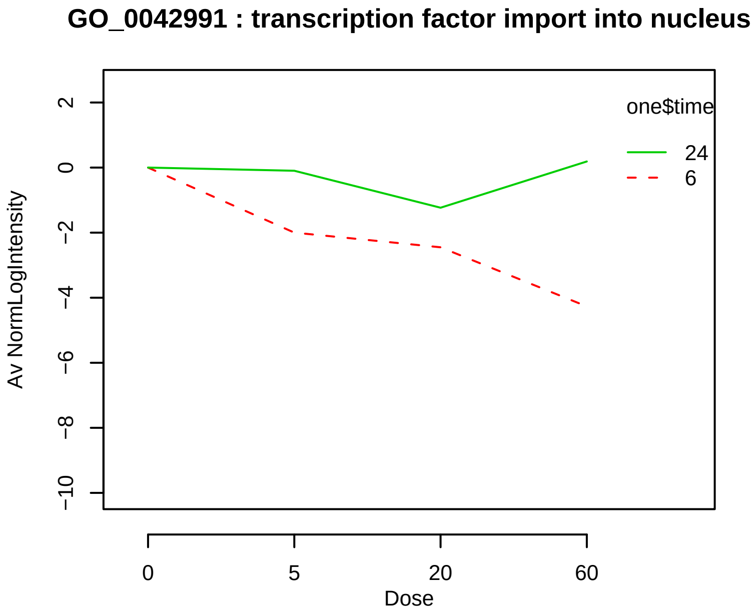
GO_0042991 : transcription factor import into nucleus
one$time
24
6
Av NormLogIntensity
2
0
−2
−4
−6
−8
−10
0
5
20
60
Dose

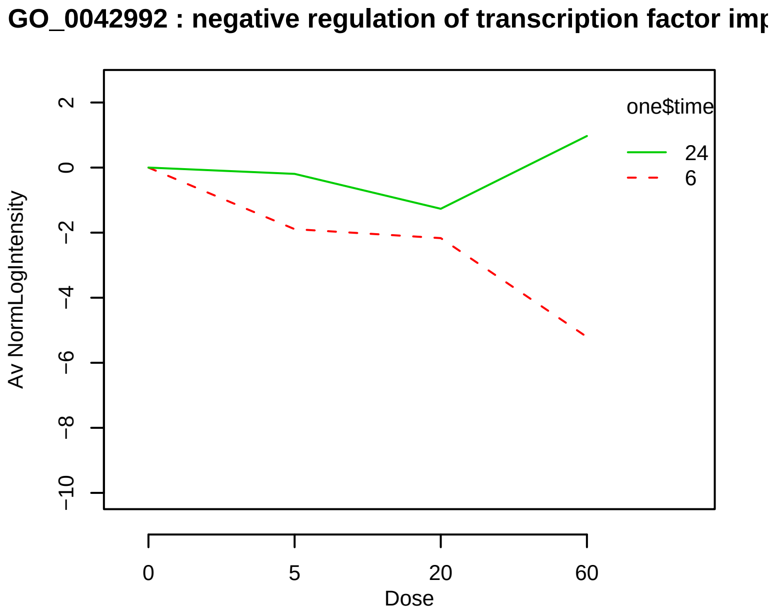
GO_0042992 : negative regulation of transcription factor imp
one$time
24
6
Av NormLogIntensity
2
0
−2
−4
−6
−8
−10
0
5
20
60
Dose

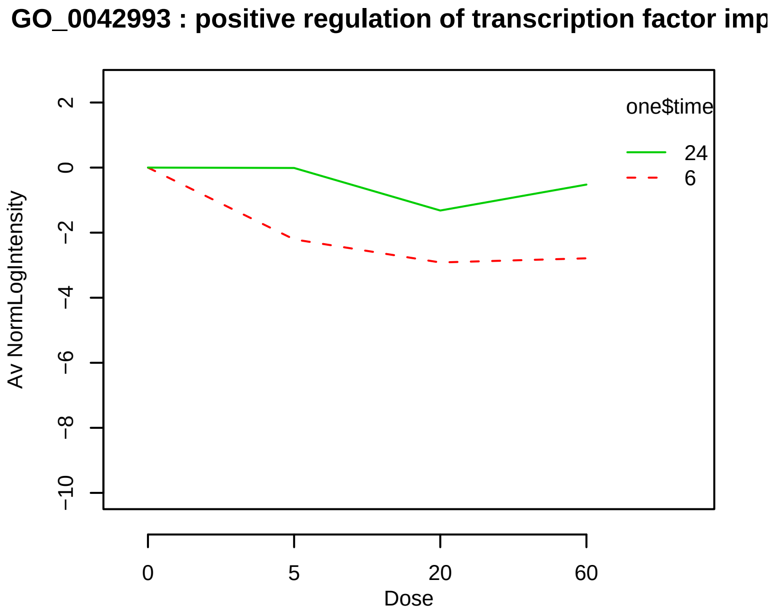
GO_0042993 : positive regulation of transcription factor imp
one$time
24
6
Av NormLogIntensity
2
0
−2
−4
−6
−8
−10
0
5
20
60
Dose

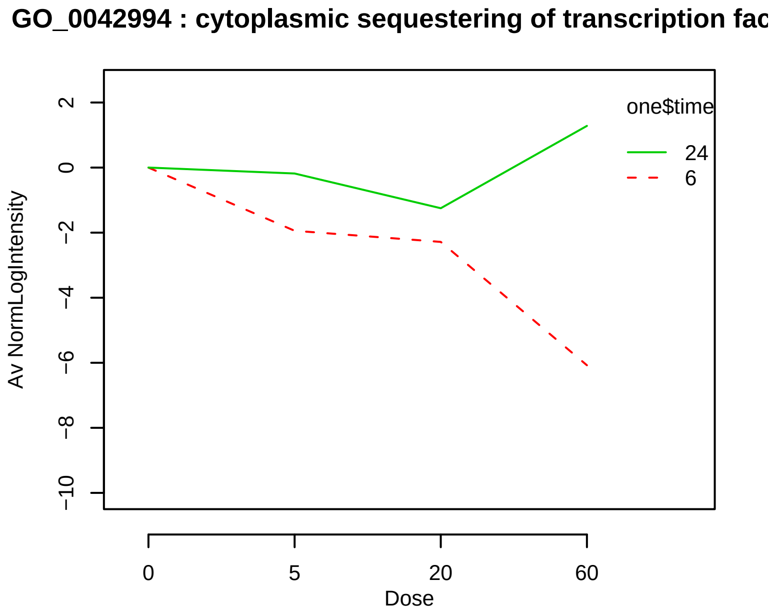
GO_0042994 : cytoplasmic sequestering of transcription fac
Av NormLogIntensity
Dose
one$time
24
6
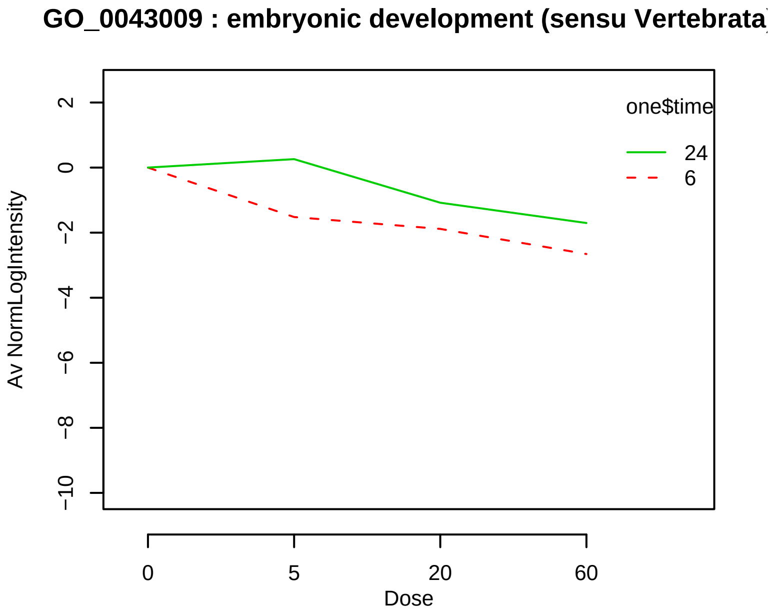
GO_0043009 : embryonic development (sensu Vertebrata
Av NormLogIntensity
Dose
one$time
24
6
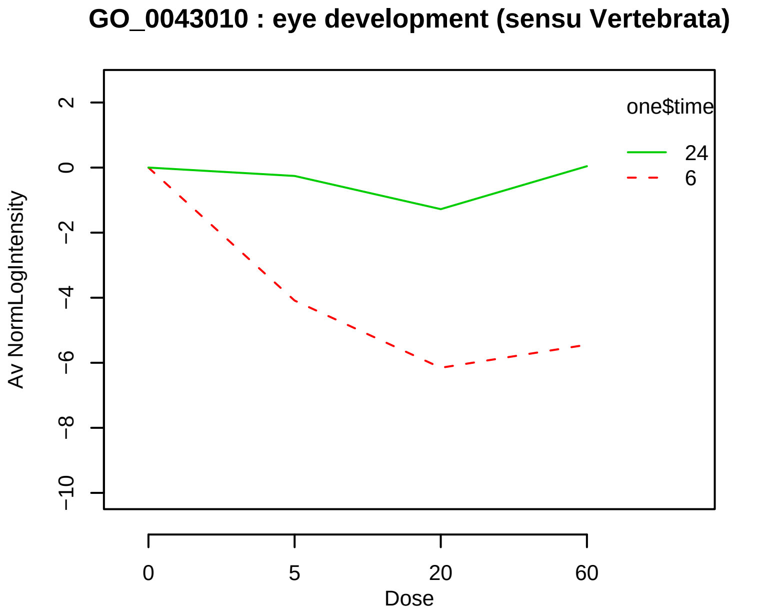
GO_0043010 : eye development (sensu Vertebrata)
Av NormLogIntensity
Dose
one$time
24
6
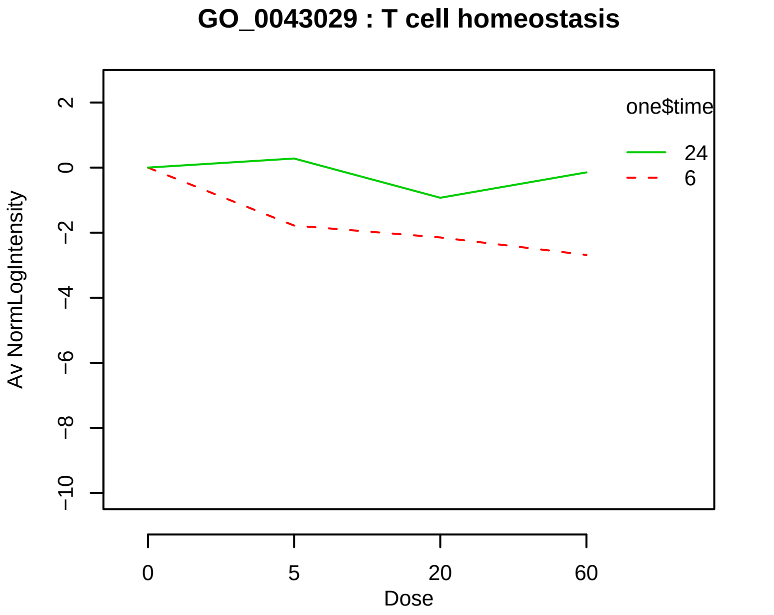
GO_0043029 : T cell homeostasis
Av NormLogIntensity
Dose
one$time
24
6
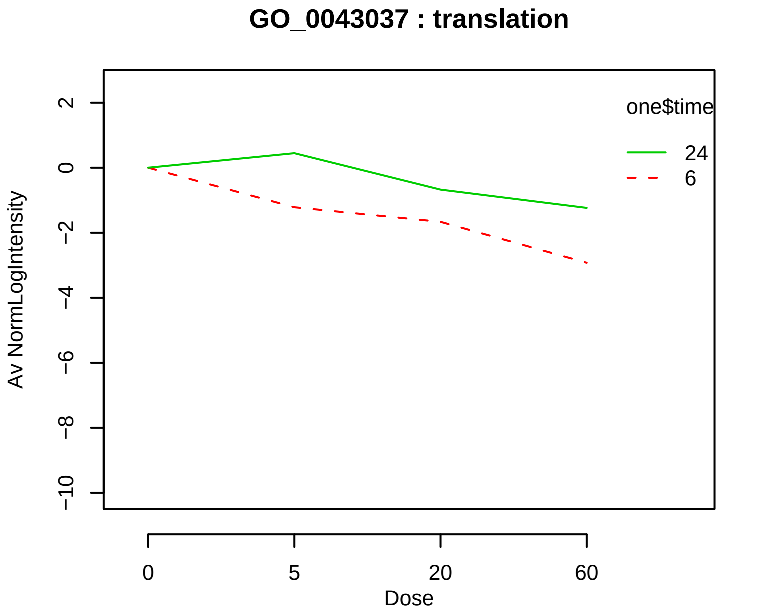
GO_0043037 : translation
Av NormLogIntensity
Dose
one$time
24
6
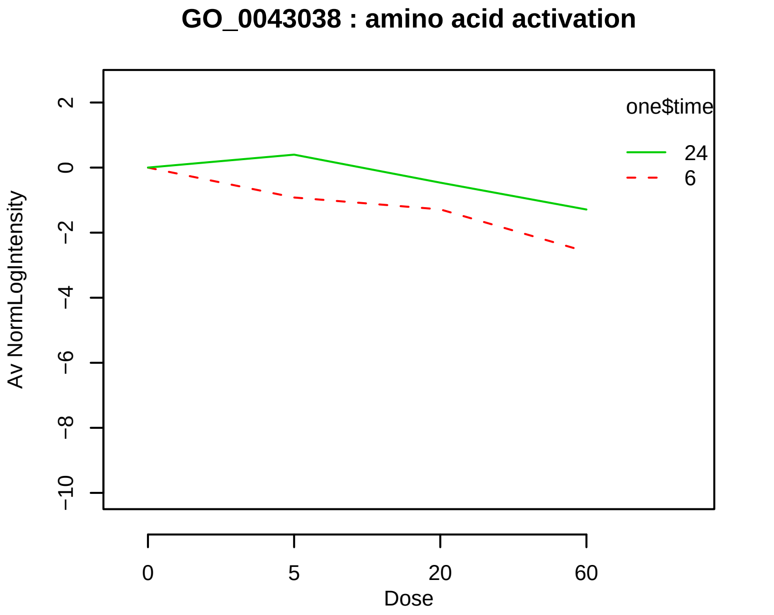
GO_0043038 : amino acid activation
Av NormLogIntensity
Dose
one$time
24
6

GO_0043039 : tRNA aminoacylation

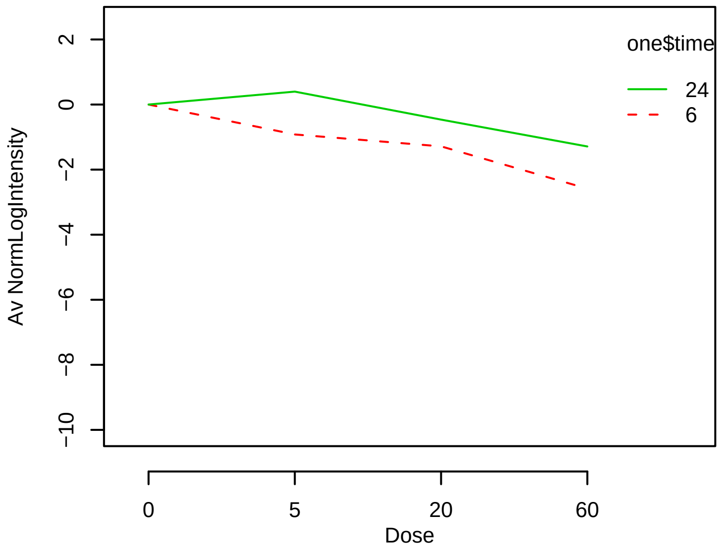

GO_0043043 : peptide biosynthesis

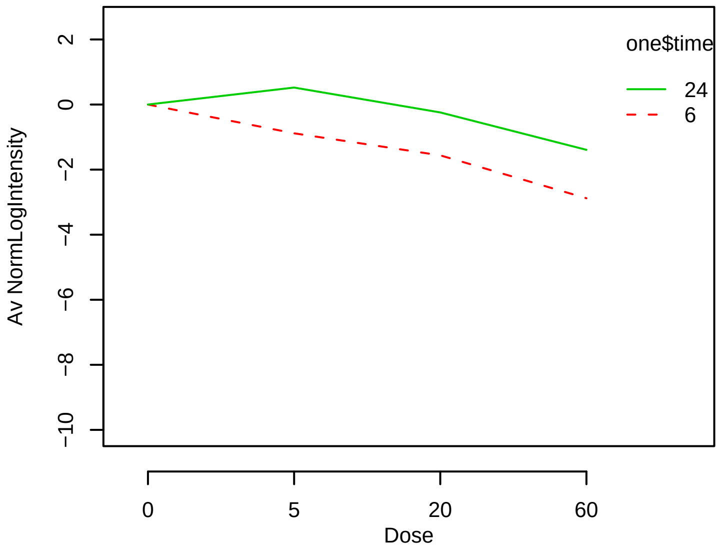

GO_0043062 : extracellular structure organization and bioge

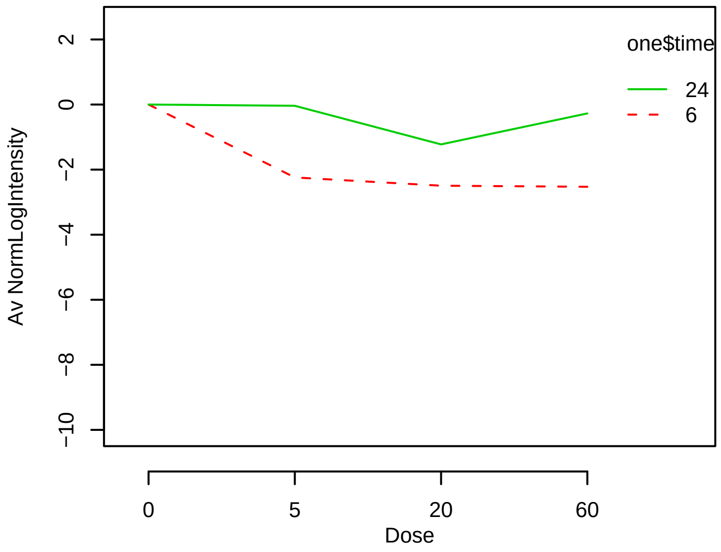

GO_0043064 : flagellum organization and biogenesis

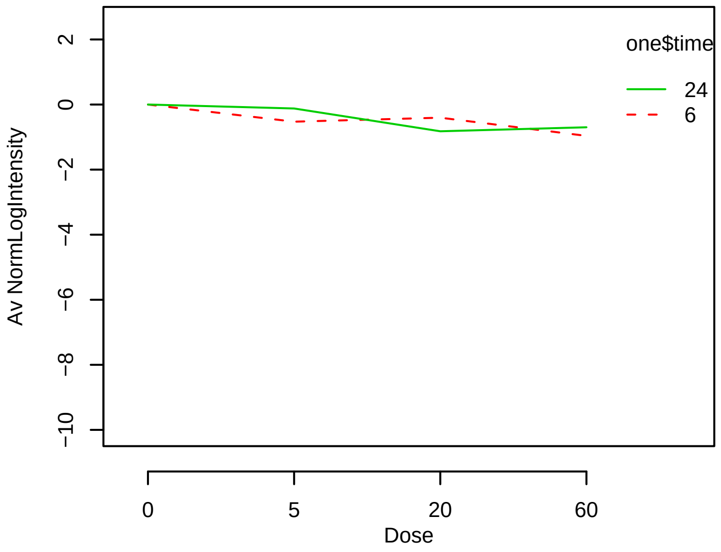

GO_0043065 : positive regulation of apoptosis

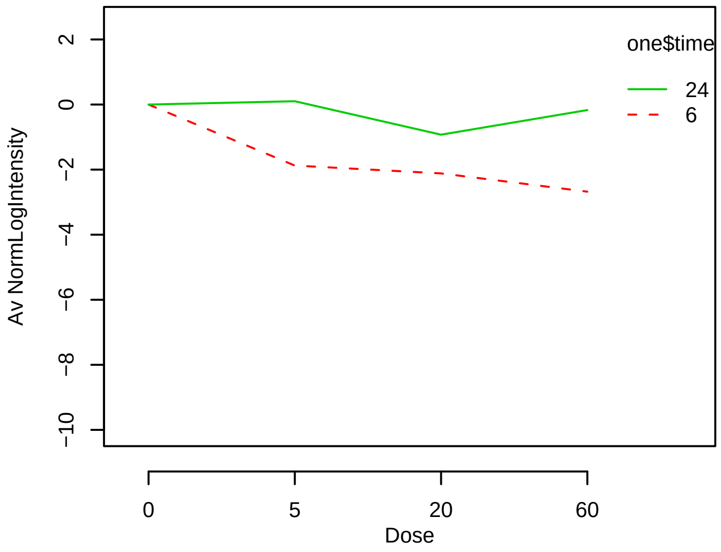

GO_0043066 : negative regulation of apoptosis

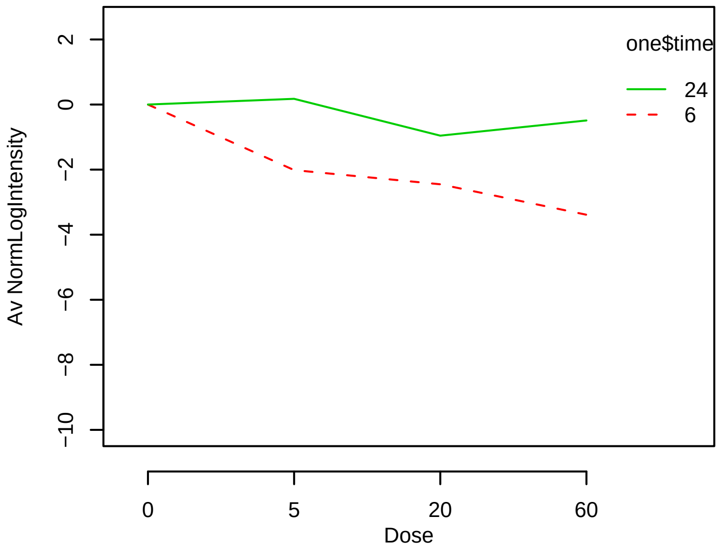

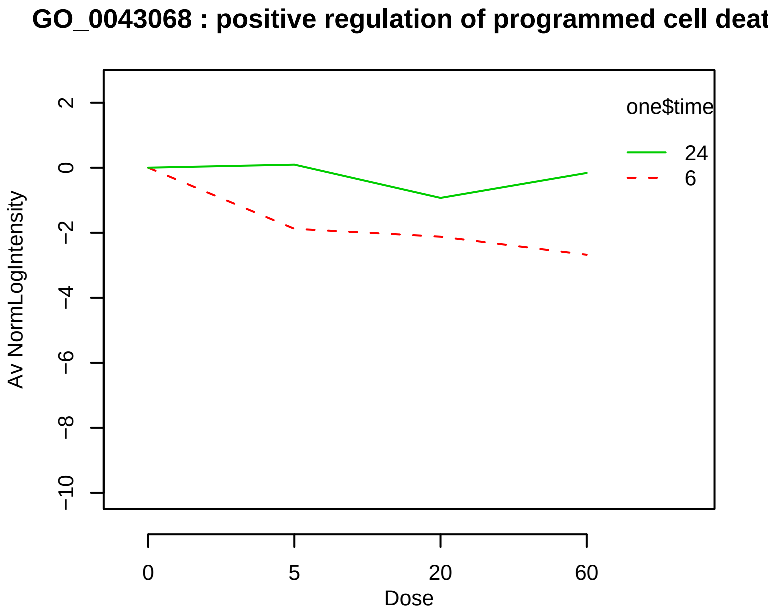
GO_0043068 : positive regulation of programmed cell deat
Av NormLogIntensity
2
0
−2
−4
−6
−8
−10
one$time
24
6
0
5
20
60
Dose

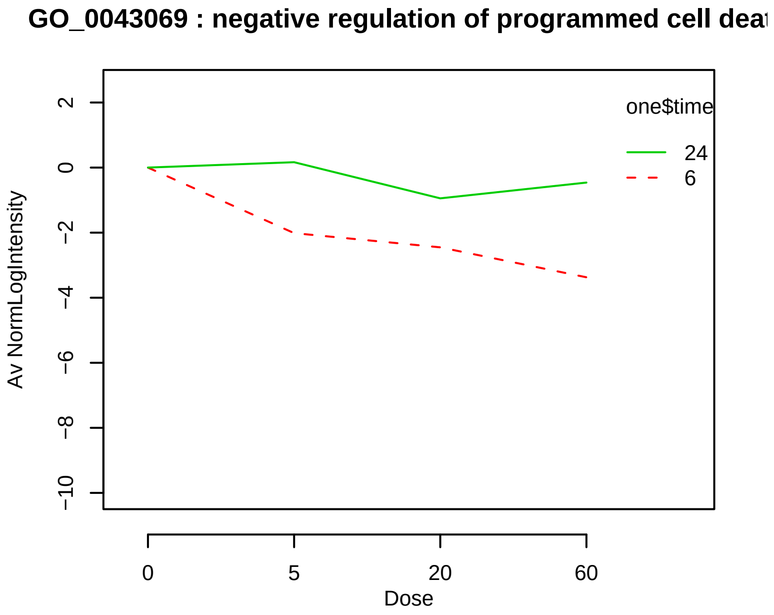
GO_0043069 : negative regulation of programmed cell deat
Av NormLogIntensity
2
0
−2
−4
−6
−8
−10
one$time
24
6
0
5
20
60
Dose

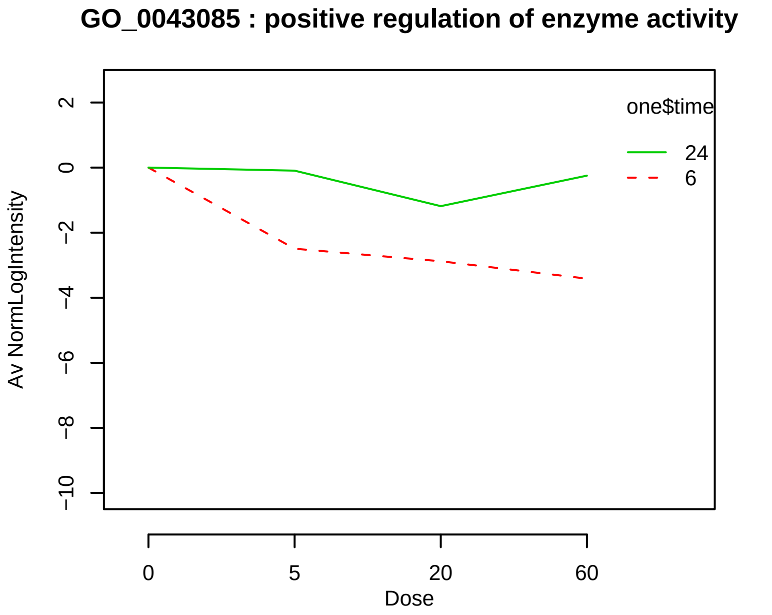
GO_0043085 : positive regulation of enzyme activity
Av NormLogIntensity
2
0
−2
−4
−6
−8
−10
one$time
24
6
0
5
20
60
Dose

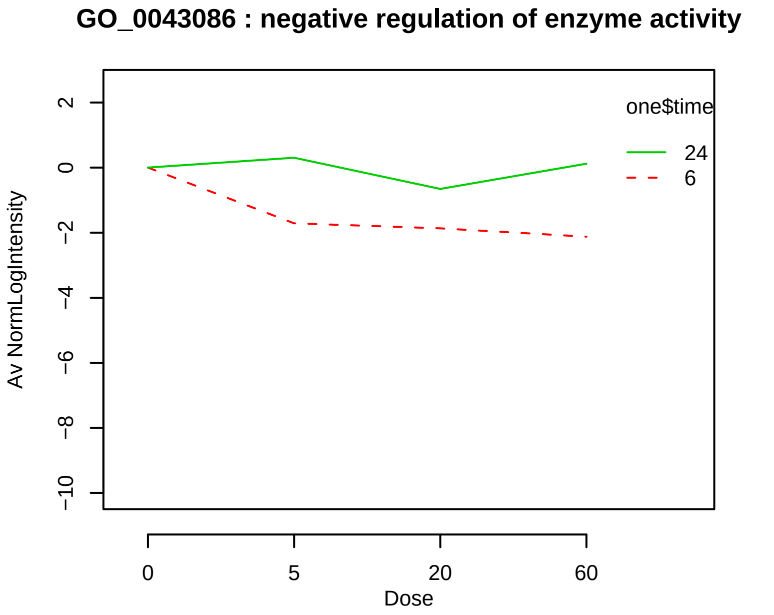
GO_0043086 : negative regulation of enzyme activity
Av NormLogIntensity
2
0
−2
−4
−6
−8
−10
one$time
24
6
0
5
20
60
Dose

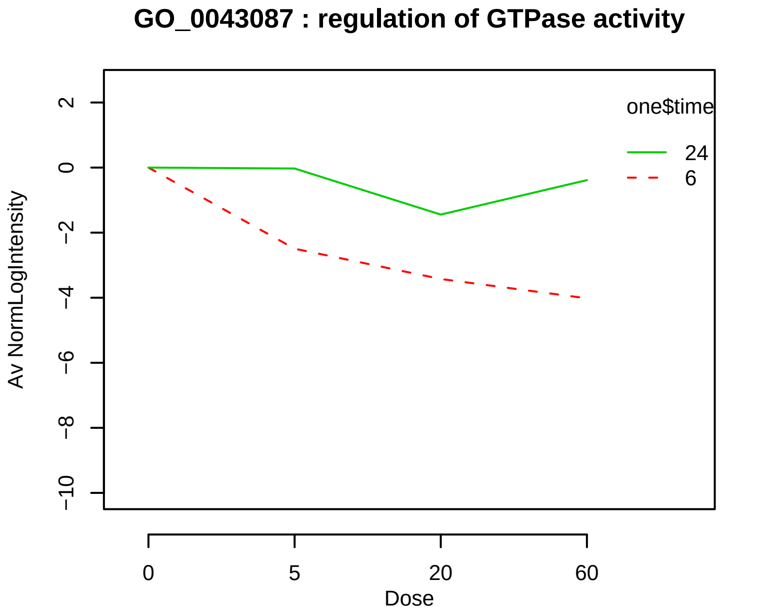
GO_0043087 : regulation of GTPase activity
Av NormLogIntensity
2
0
−2
−4
−6
−8
−10
one$time
24
6
0
5
20
60
Dose

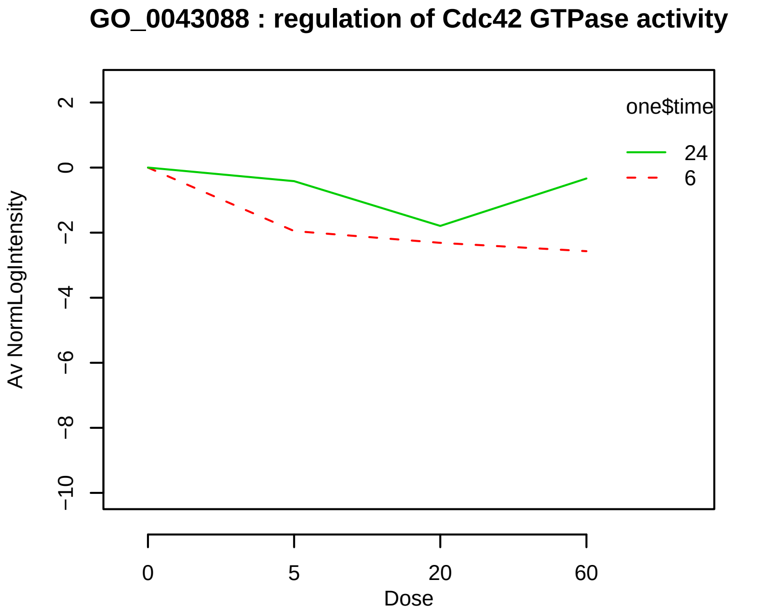
GO_0043088 : regulation of Cdc42 GTPase activity
Av NormLogIntensity
2
0
−2
−4
−6
−8
−10
one$time
24
6
0
5
20
60
Dose

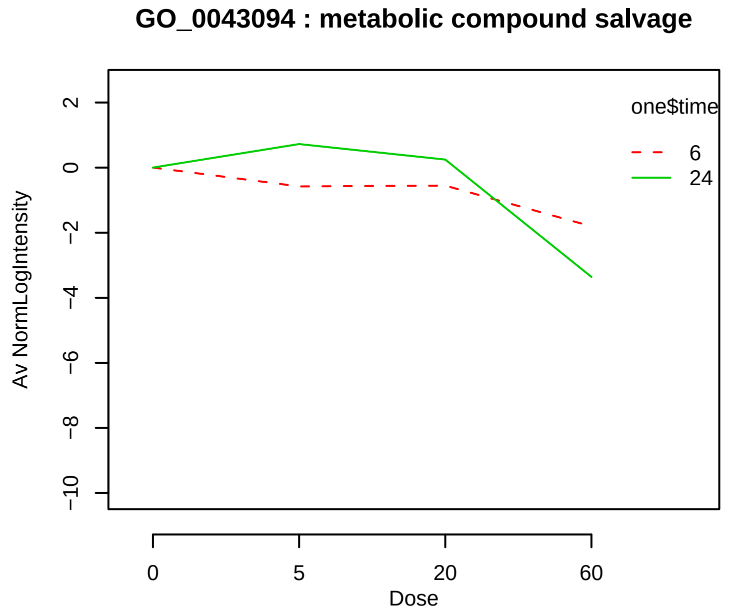
GO_0043094 : metabolic compound salvage
one$time
6
24
Av NormLogIntensity
2
0
−2
−4
−6
−8
−10
0
5
20
60
Dose

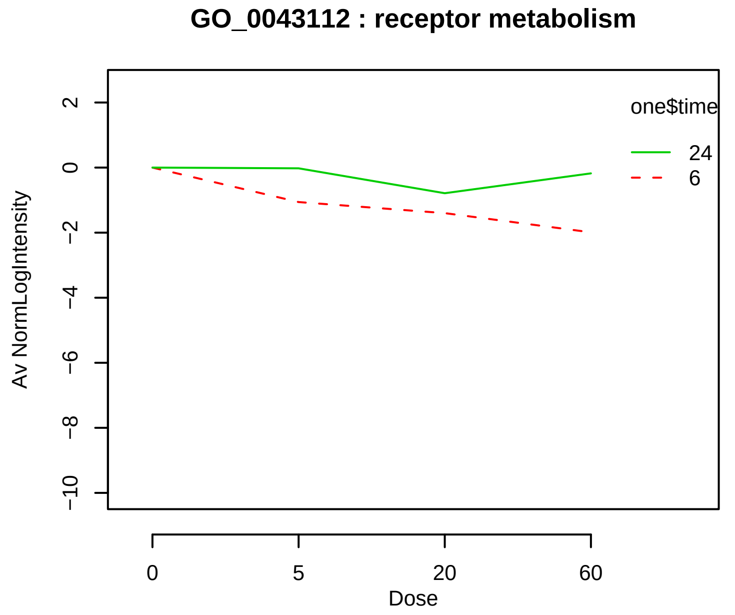
GO_0043112 : receptor metabolism
one$time
24
6
Av NormLogIntensity
2
0
−2
−4
−6
−8
−10
0
5
20
60
Dose

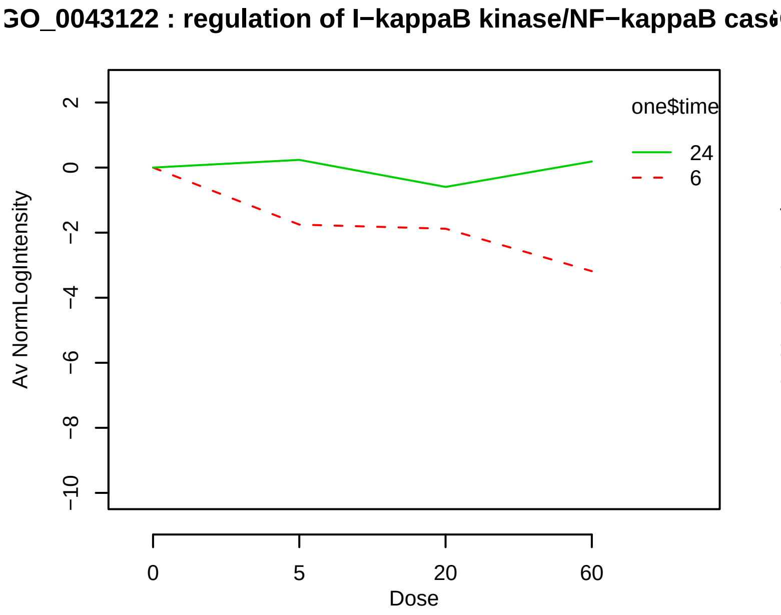
GO_0043122 : regulation of I−kappaB kinase/NF−kappaB cas
one$time
24
6
Av NormLogIntensity
2
0
−2
−4
−6
−8
−10
0
5
20
60
Dose

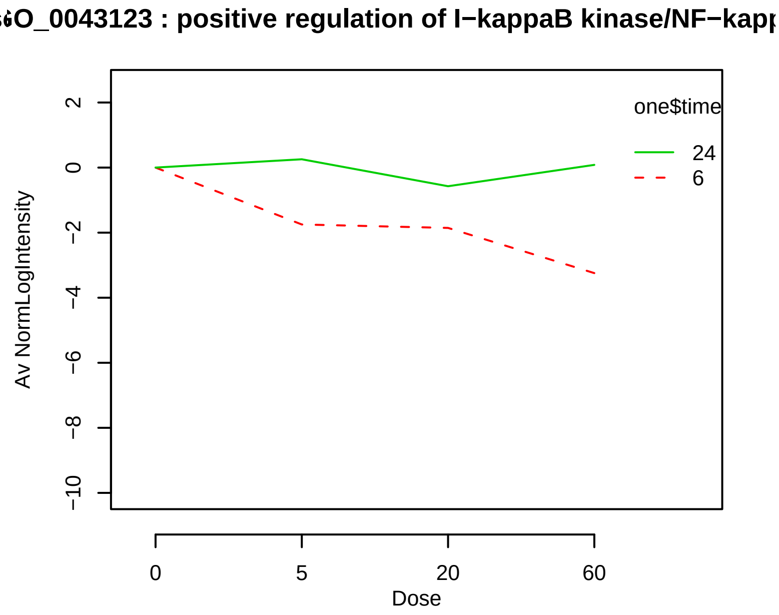
O_0043123 : positive regulation of I−kappaB kinase/NF−kapp
one$time
24
6
Av NormLogIntensity
2
0
−2
−4
−6
−8
−10
0
5
20
60
Dose

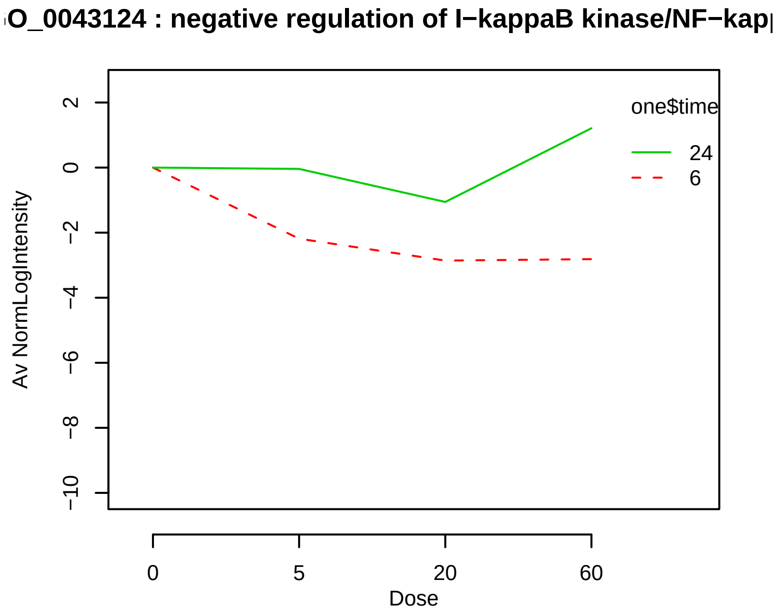
O_0043124 : negative regulation of I−kappaB kinase/NF−kap
one$time
24
6
Av NormLogIntensity
2
0
−2
−4
−6
−8
−10
0
5
20
60
Dose

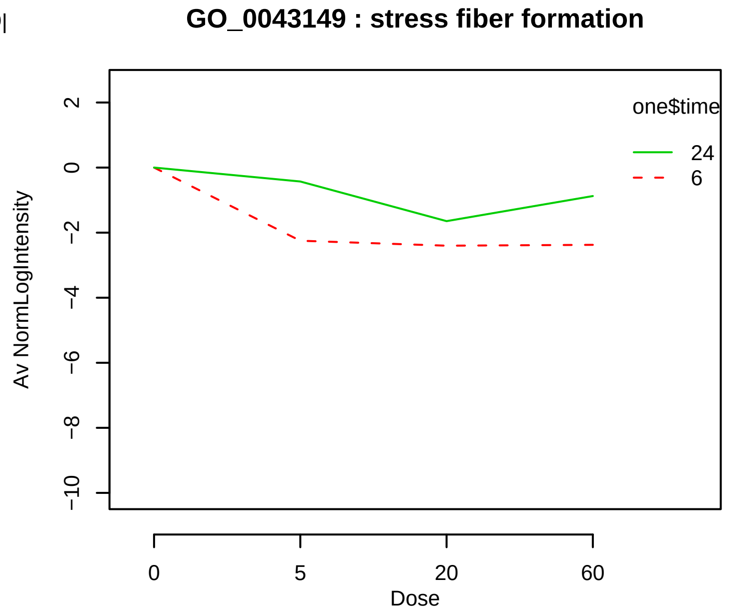
GO_0043149 : stress fiber formation
one$time
24
6
Av NormLogIntensity
2
0
−2
−4
−6
−8
−10
0
5
20
60
Dose

GO_0043154 : negative regulation of caspase activity

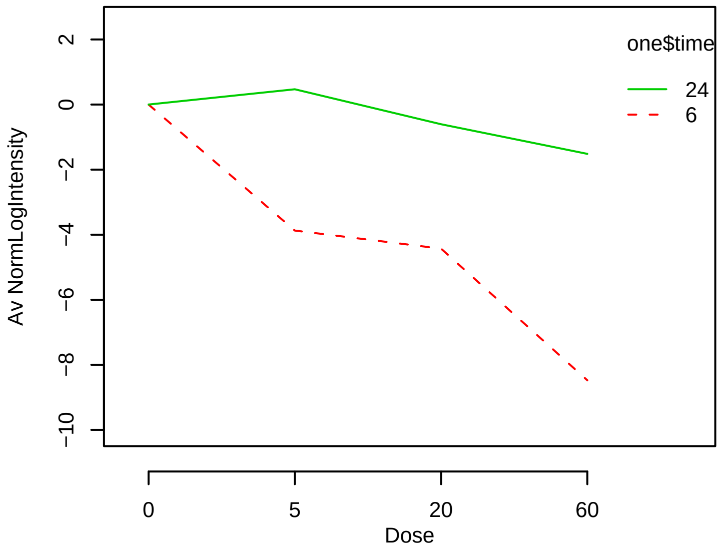

O_0043161 : proteasomal ubiquitin−dependent protein catab

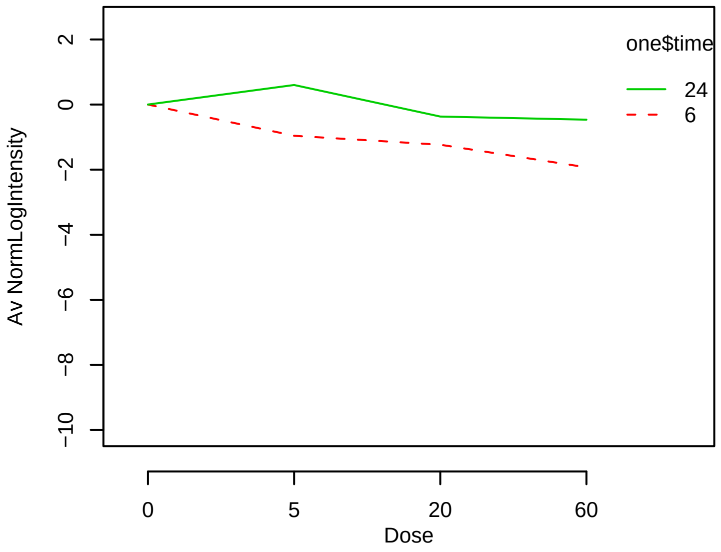

GO_0043193 : positive regulation of gene−specific transcript

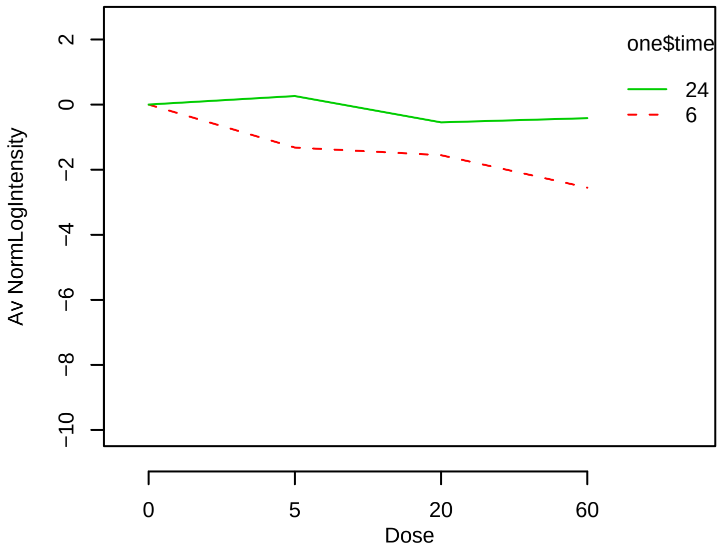

GO_0043241 : protein complex disassembly

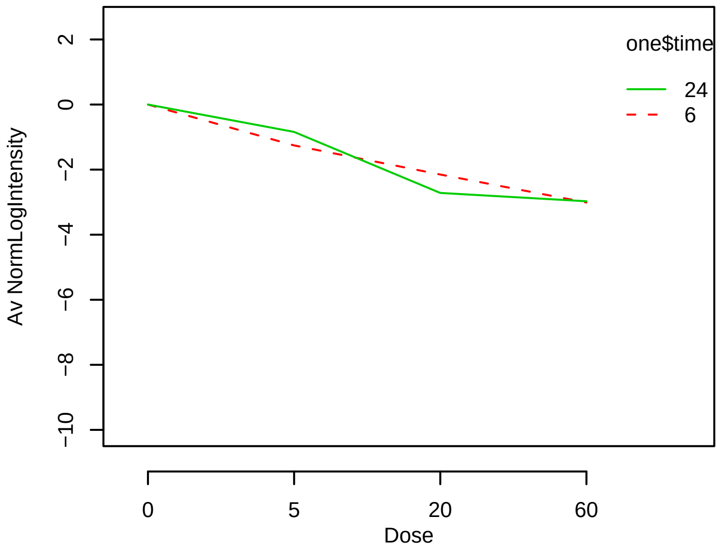

GO_0043254 : regulation of protein complex assembly

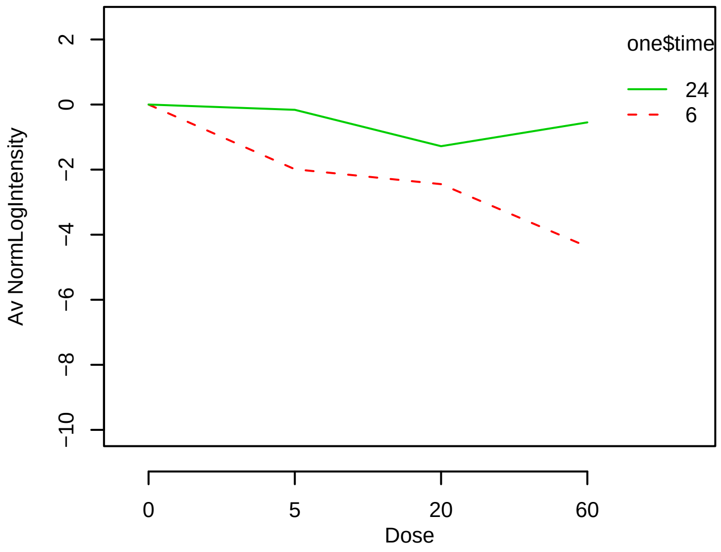

GO_0043255 : regulation of carbohydrate biosynthesis

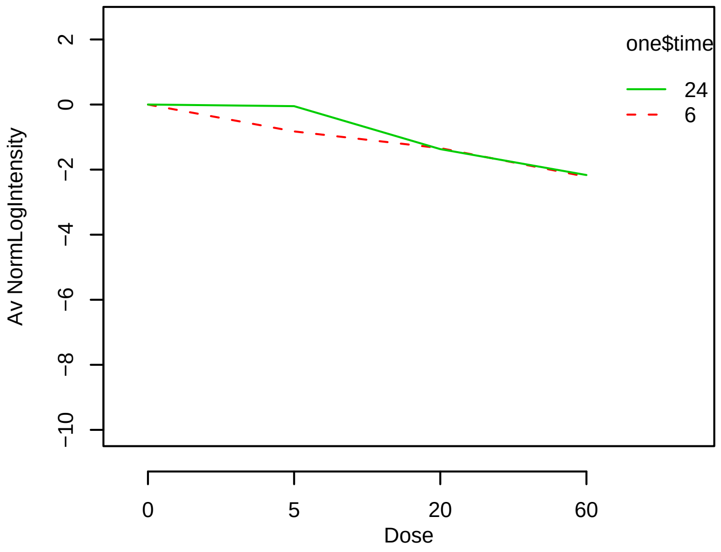

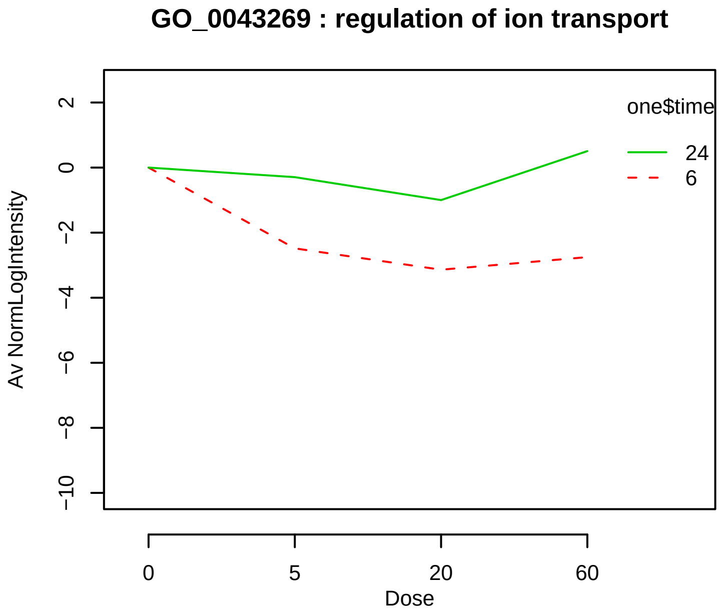
GO_0043269 : regulation of ion transport
one$time
24
6
Av NormLogIntensity
2
0
−2
−4
−6
−8
−10
0
5
20
60
Dose

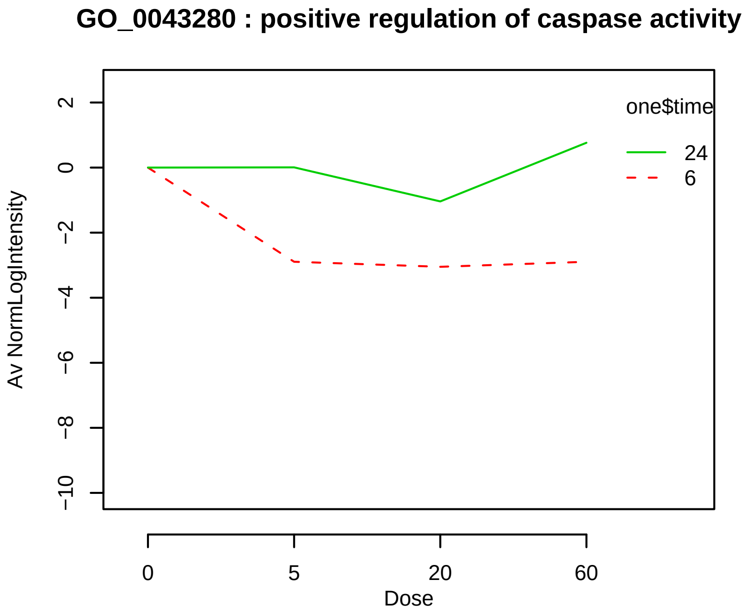
GO_0043280 : positive regulation of caspase activity
one$time
24
6
Av NormLogIntensity
2
0
−2
−4
−6
−8
−10
0
5
20
60
Dose

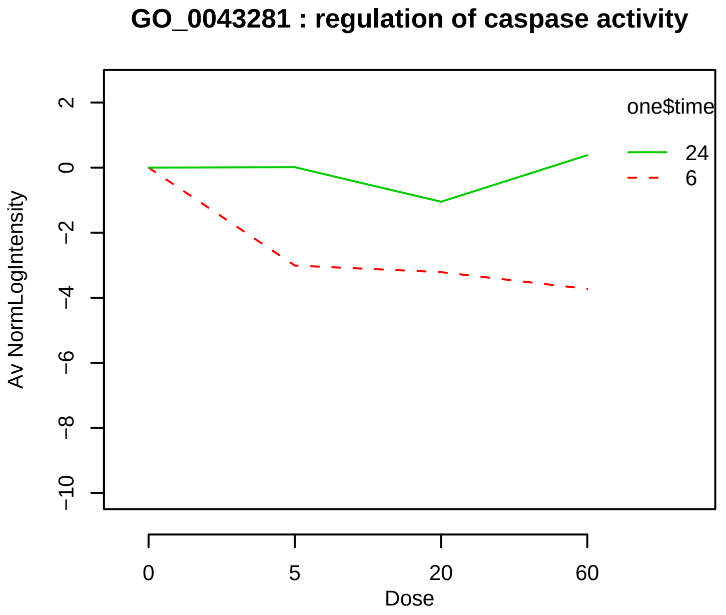
GO_0043281 : regulation of caspase activity
one$time
24
6
Av NormLogIntensity
2
0
−2
−4
−6
−8
−10
0
5
20
60
Dose

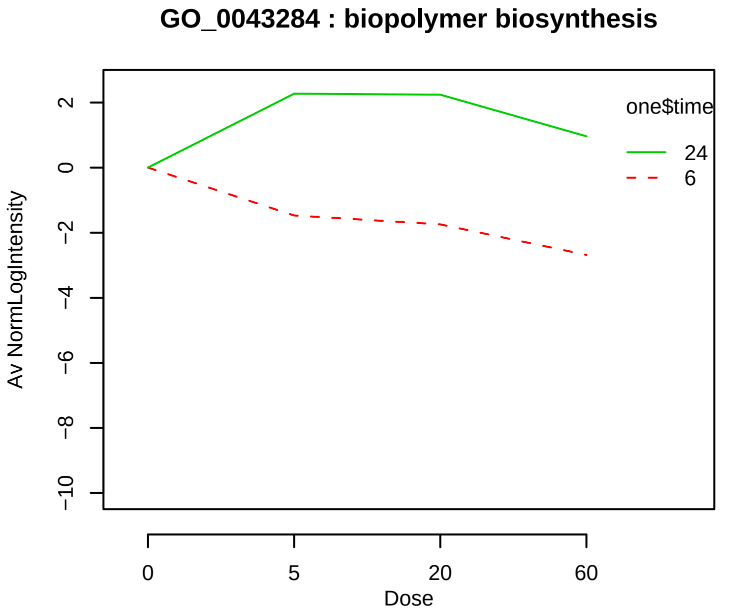
GO_0043284 : biopolymer biosynthesis
one$time
24
6
Av NormLogIntensity
2
0
−2
−4
−6
−8
−10
0
5
20
60
Dose

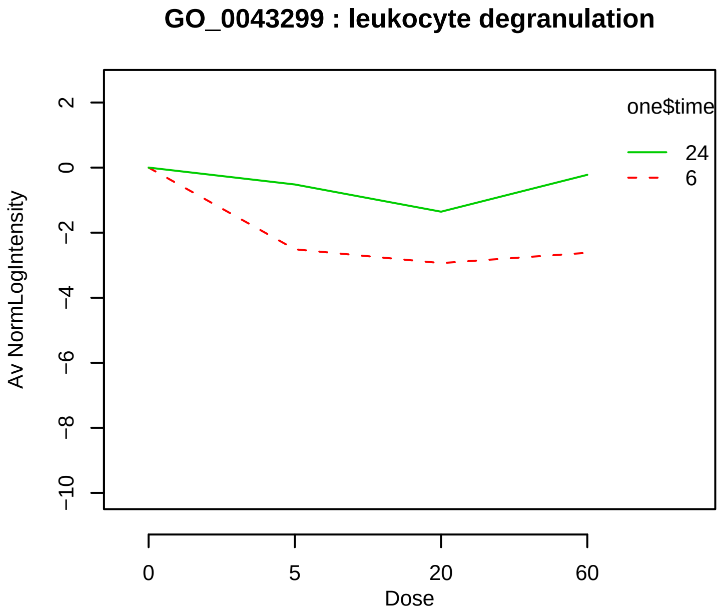
GO_0043299 : leukocyte degranulation
one$time
24
6
Av NormLogIntensity
2
0
−2
−4
−6
−8
−10
0
5
20
60
Dose

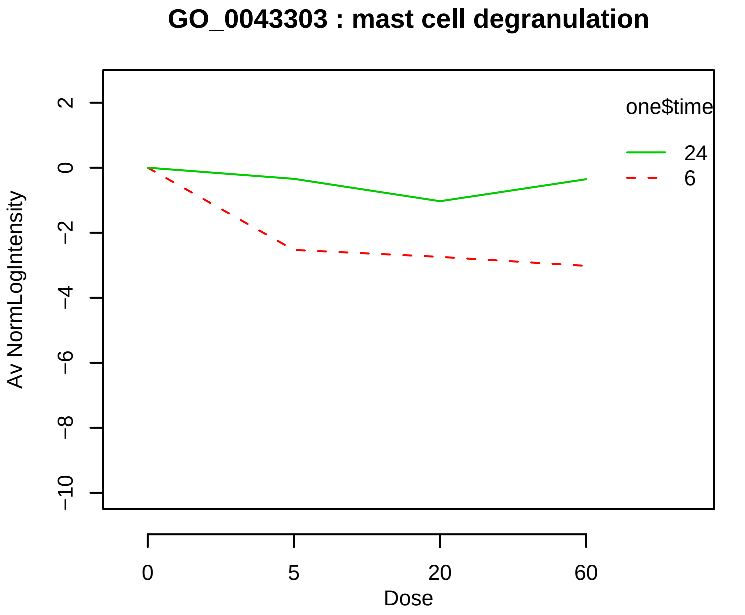
GO_0043303 : mast cell degranulation
one$time
24
6
Av NormLogIntensity
2
0
−2
−4
−6
−8
−10
0
5
20
60
Dose

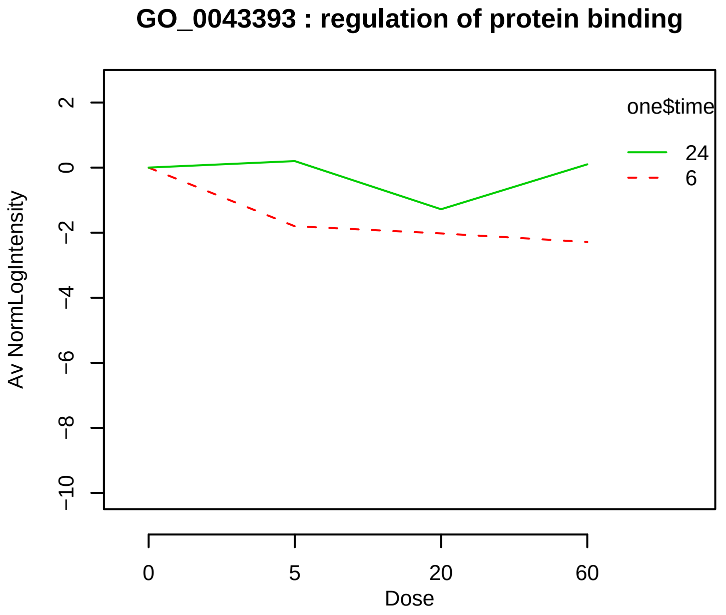
GO_0043393 : regulation of protein binding
Av NormLogIntensity
2
0
−2
−4
−6
−8
−10
one$time
24
6
0
5
20
60
Dose

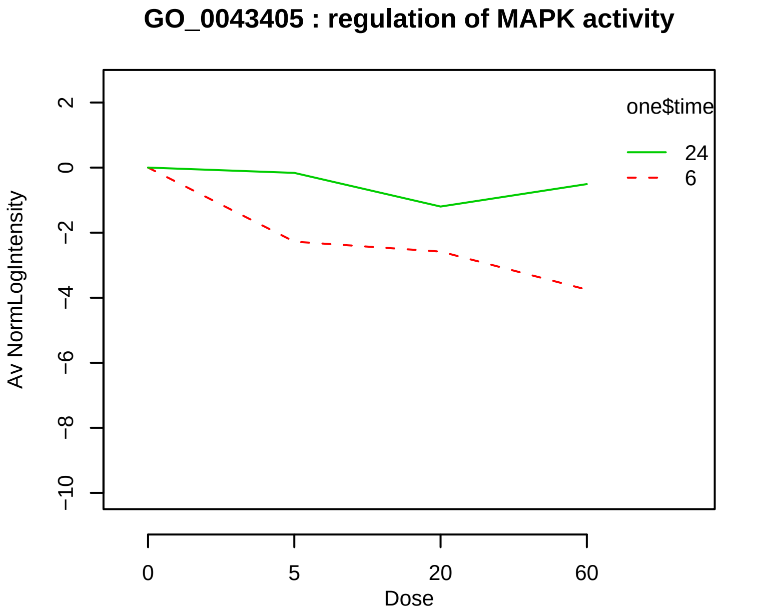
GO_0043405 : regulation of MAPK activity
Av NormLogIntensity
2
0
−2
−4
−6
−8
−10
one$time
24
6
0
5
20
60
Dose

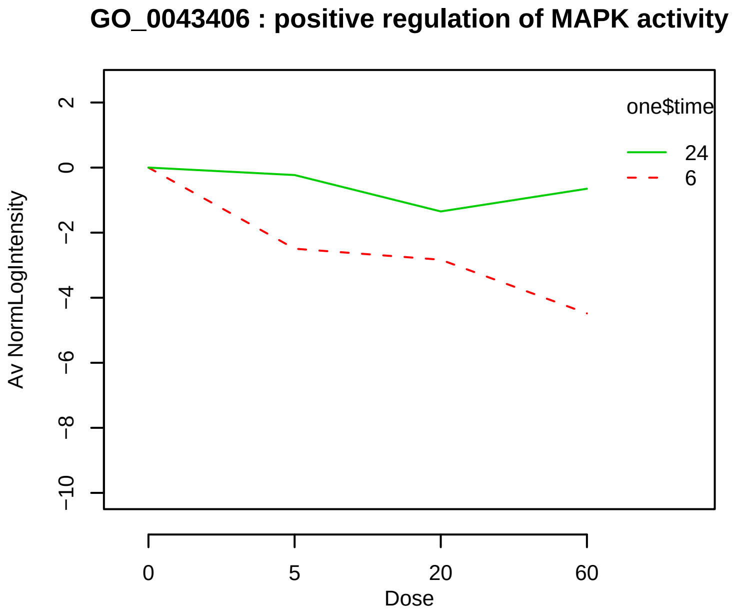
GO_0043406 : positive regulation of MAPK activity
Av NormLogIntensity
2
0
−2
−4
−6
−8
−10
one$time
24
6
0
5
20
60
Dose

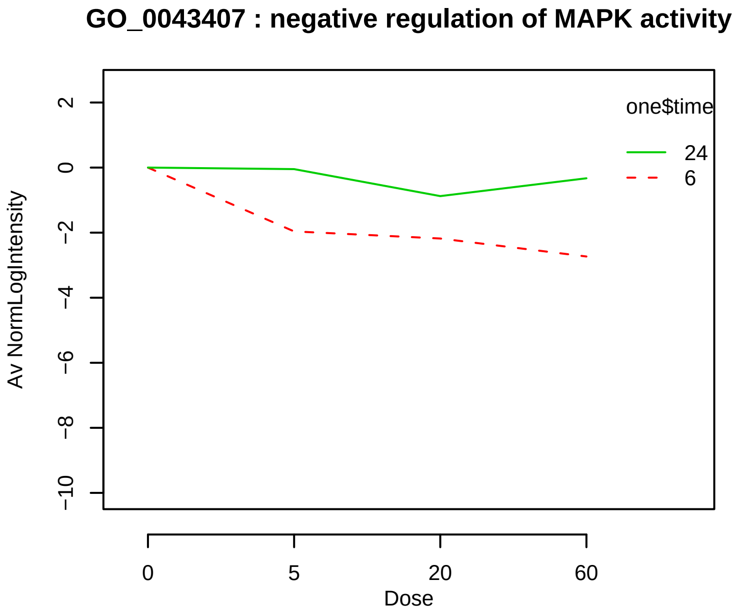
GO_0043407 : negative regulation of MAPK activity
Av NormLogIntensity
2
0
−2
−4
−6
−8
−10
one$time
24
6
0
5
20
60
Dose

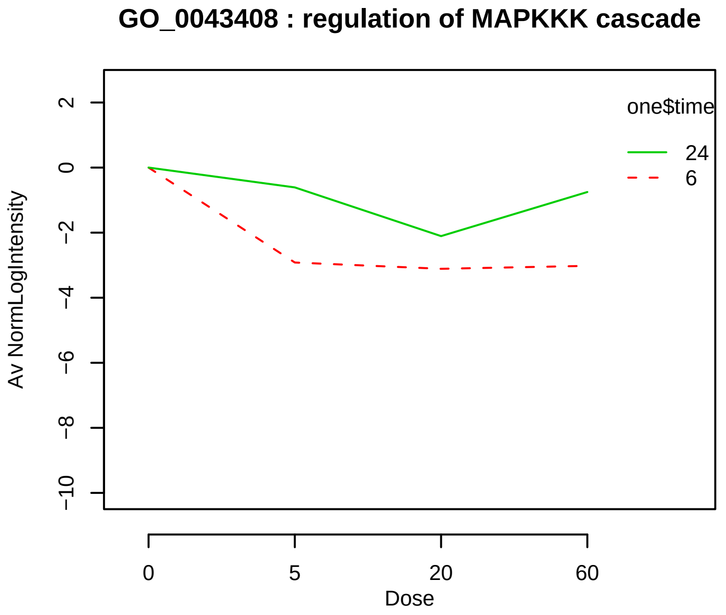
GO_0043408 : regulation of MAPKKK cascade
Av NormLogIntensity
2
0
−2
−4
−6
−8
−10
one$time
24
6
0
5
20
60
Dose

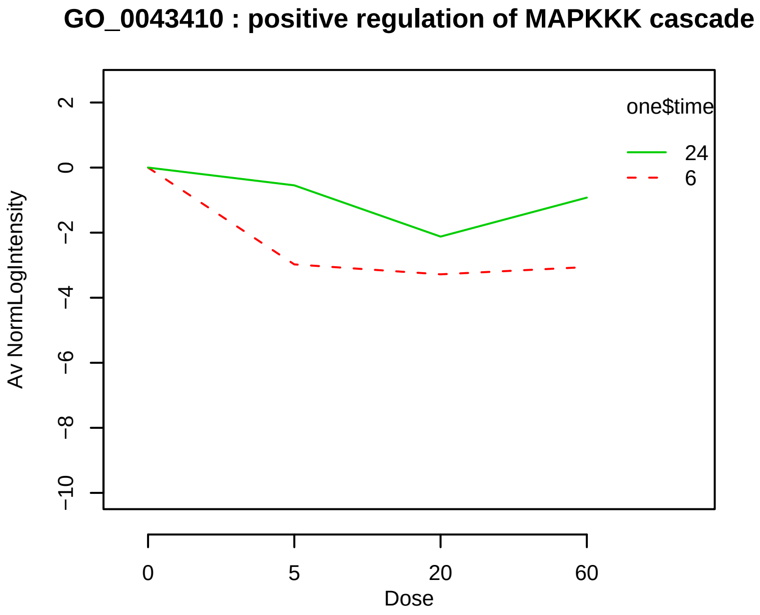
GO_0043410 : positive regulation of MAPKKK cascade
Av NormLogIntensity
2
0
−2
−4
−6
−8
−10
one$time
24
6
0
5
20
60
Dose

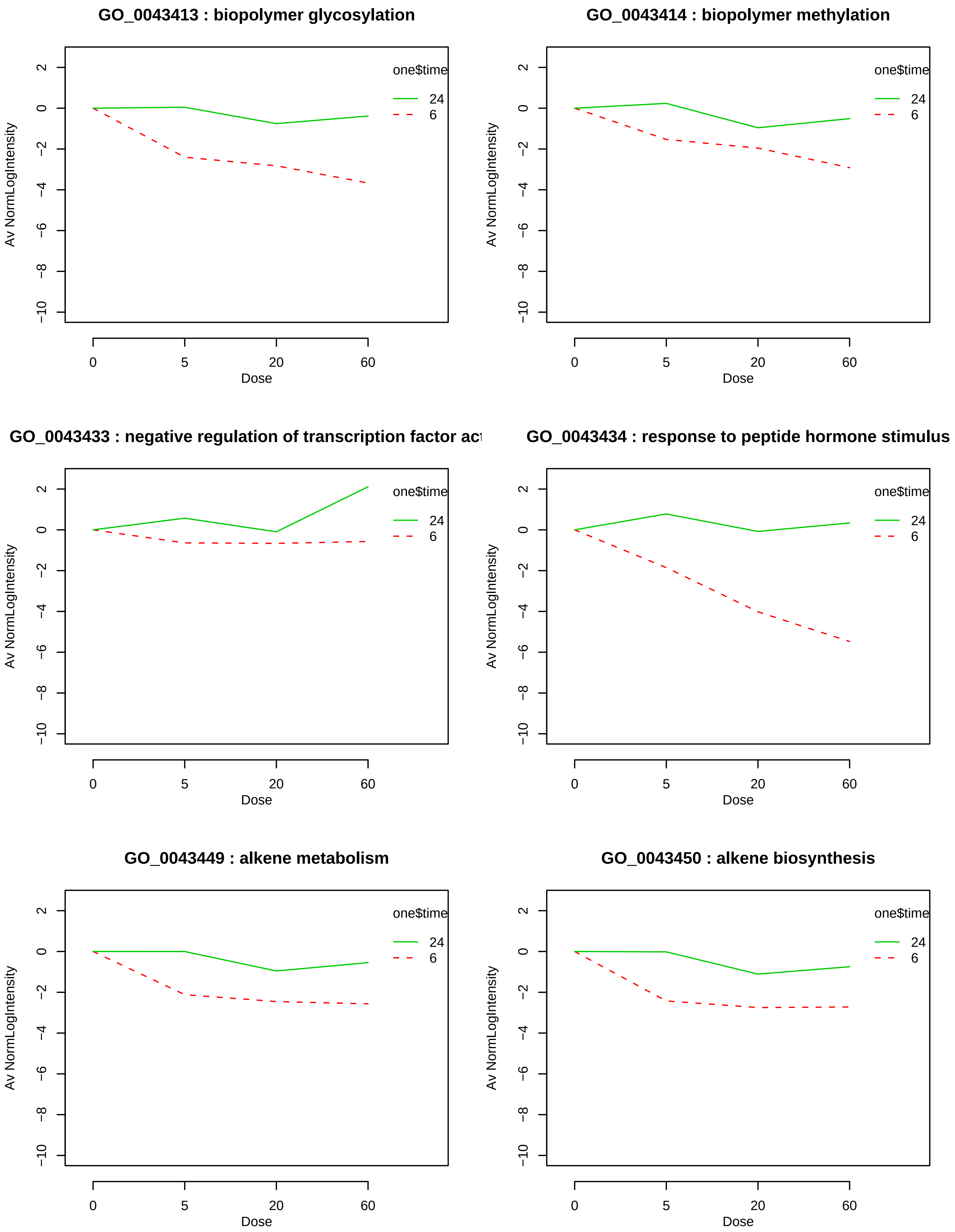
GO_0043413 : biopolymer glycosylation
GO_0043414 : biopolymer methylation
GO_0043433 : negative regulation of transcription factor act
GO_0043434 : response to peptide hormone stimulus
GO_0043449 : alkene metabolism
GO_0043450 : alkene biosynthesis
Av NormLogIntensity
Dose
one$time
24
6
0
5
20
60
2
0
−2
−4
−6
−8
−10

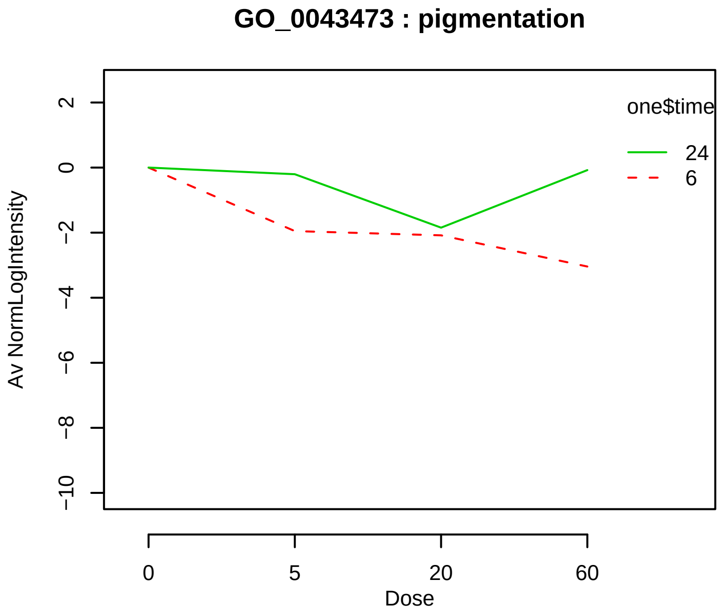
GO_0043473 : pigmentation
one$time
24
6
Av NormLogIntensity
2
0
−2
−4
−6
−8
−10
0
5
20
60
Dose

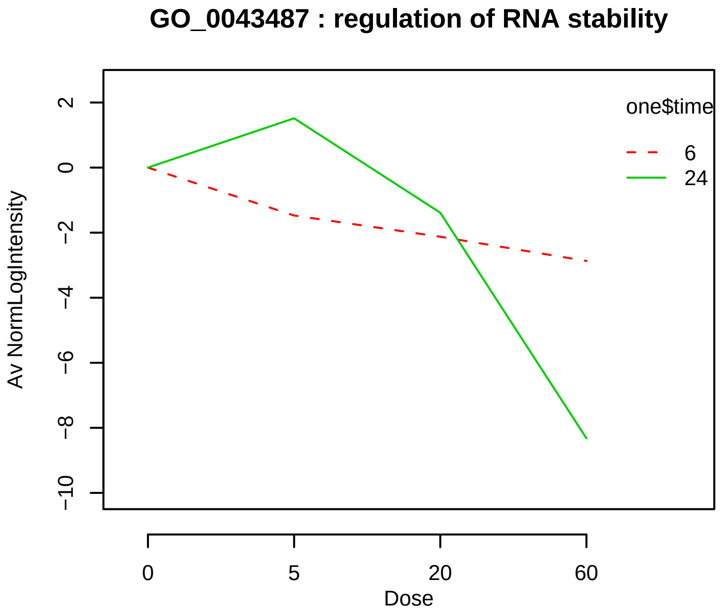
GO_0043487 : regulation of RNA stability
one$time
6
24
Av NormLogIntensity
2
0
−2
−4
−6
−8
−10
0
5
20
60
Dose

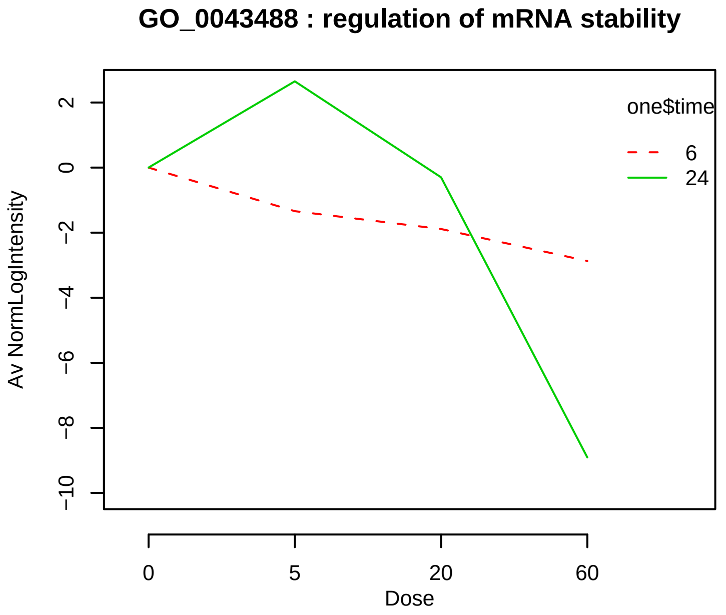
GO_0043488 : regulation of mRNA stability
one$time
6
24
Av NormLogIntensity
2
0
−2
−4
−6
−8
−10
0
5
20
60
Dose

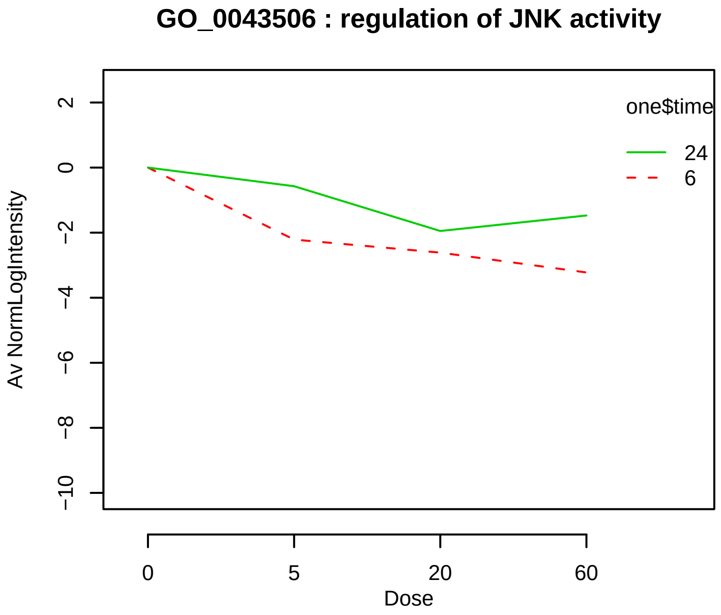
GO_0043506 : regulation of JNK activity
one$time
24
6
Av NormLogIntensity
2
0
−2
−4
−6
−8
−10
0
5
20
60
Dose

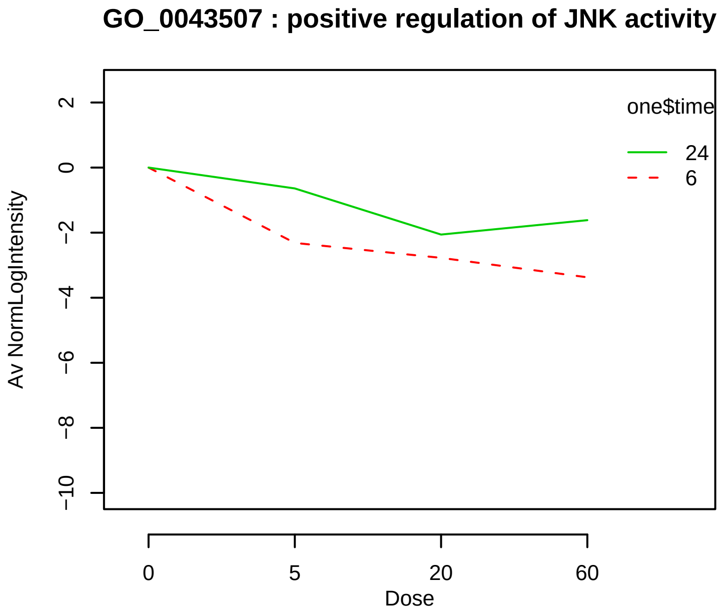
GO_0043507 : positive regulation of JNK activity
one$time
24
6
Av NormLogIntensity
2
0
−2
−4
−6
−8
−10
0
5
20
60
Dose

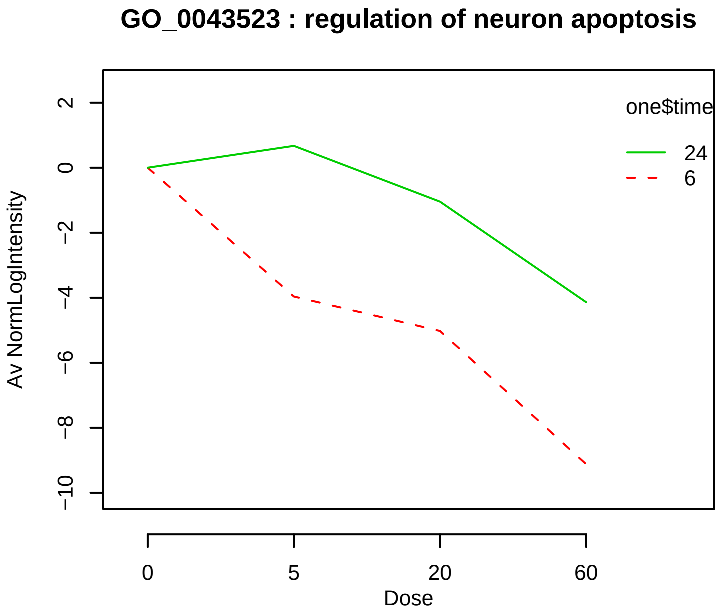
GO_0043523 : regulation of neuron apoptosis
one$time
24
6
Av NormLogIntensity
2
0
−2
−4
−6
−8
−10
0
5
20
60
Dose

GO_0043524 : negative regulation of neuron apoptosis

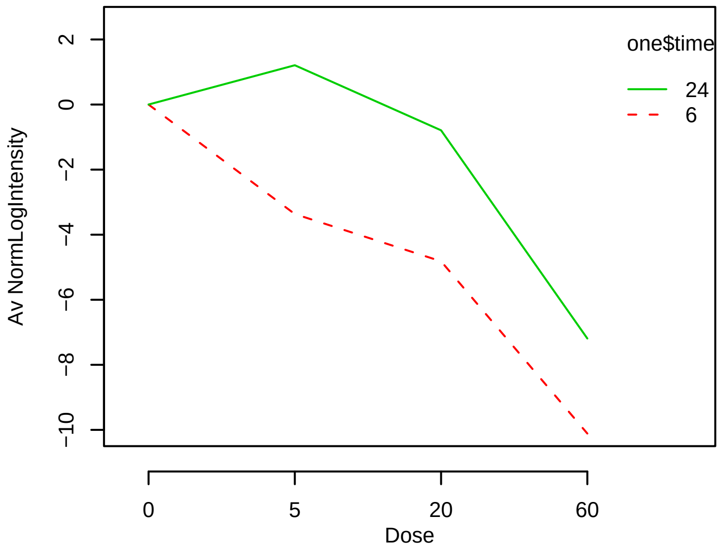

GO_0043542 : endothelial cell migration

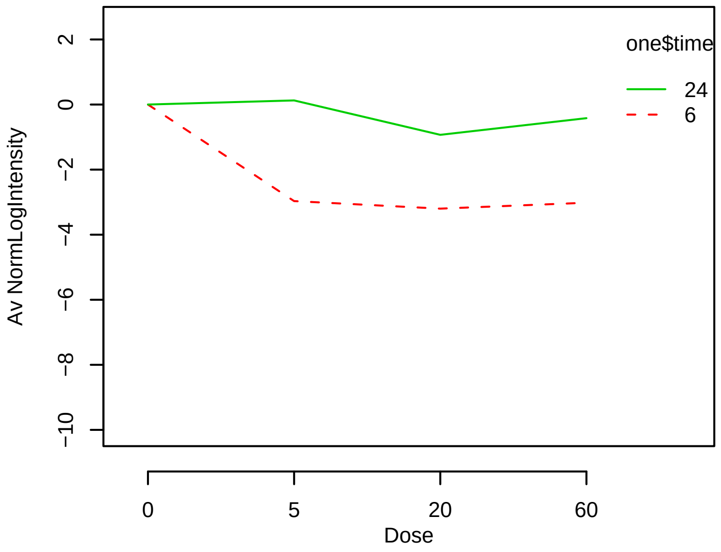

GO_0043543 : protein amino acid acylation

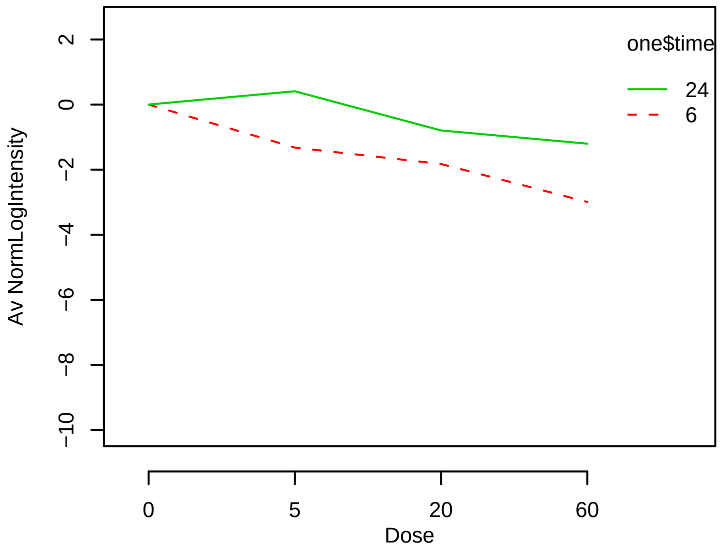

GO_0043545 : molybdopterin cofactor metabolism

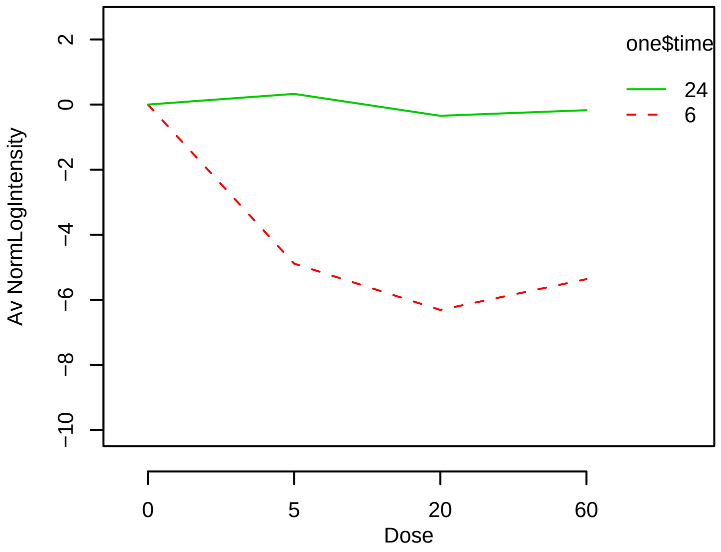

GO_0043549 : regulation of kinase activity

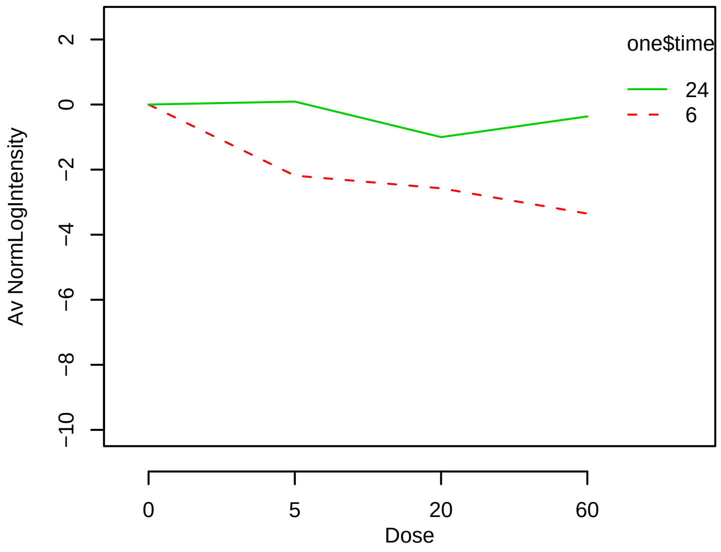

GO_0043550 : regulation of lipid kinase activity

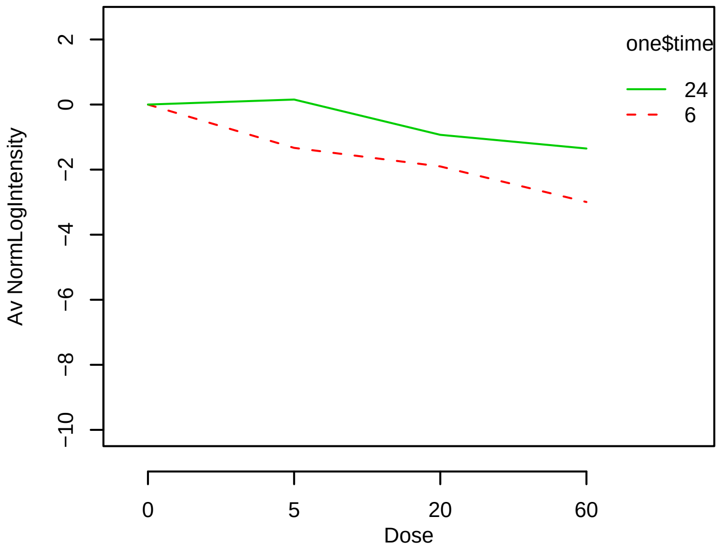

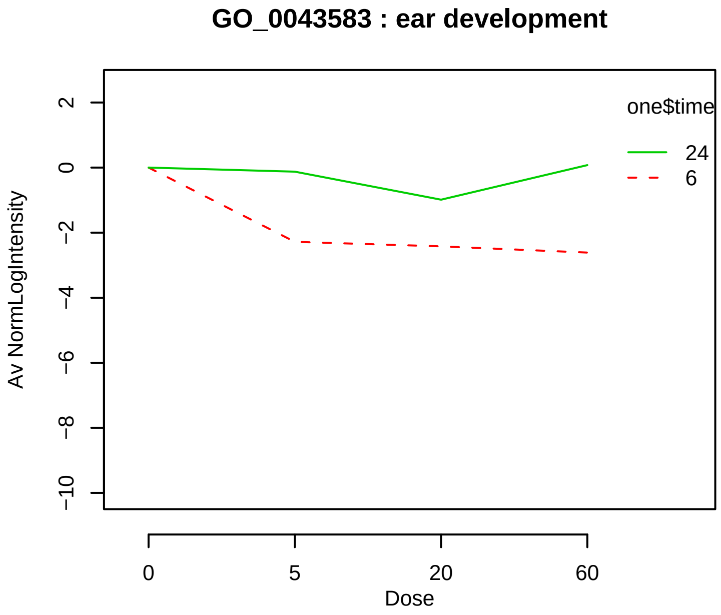
GO_0043583 : ear development
Av NormLogIntensity
Dose
one$time
24
6

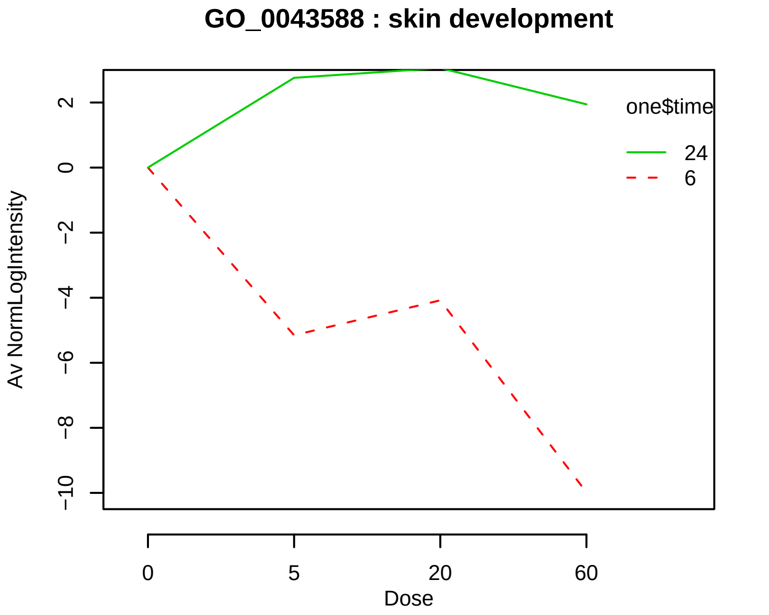
GO_0043588 : skin development
Av NormLogIntensity
Dose
one$time
24
6

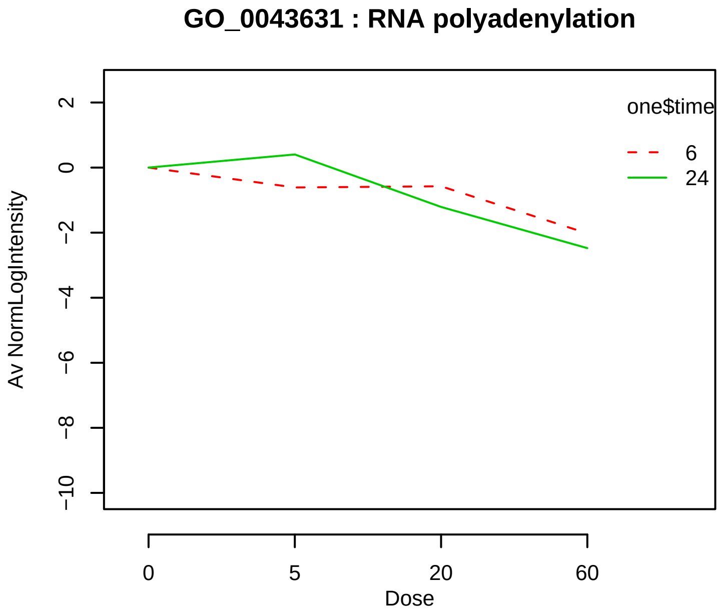
GO_0043631 : RNA polyadenylation
Av NormLogIntensity
Dose
one$time
6
24

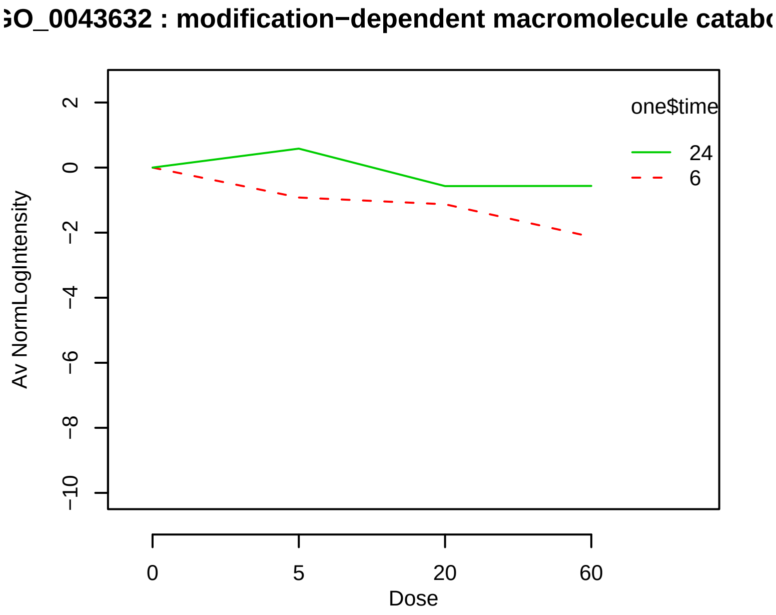
GO_0043632 : modification−dependent macromolecule catabo
Av NormLogIntensity
Dose
one$time
24
6

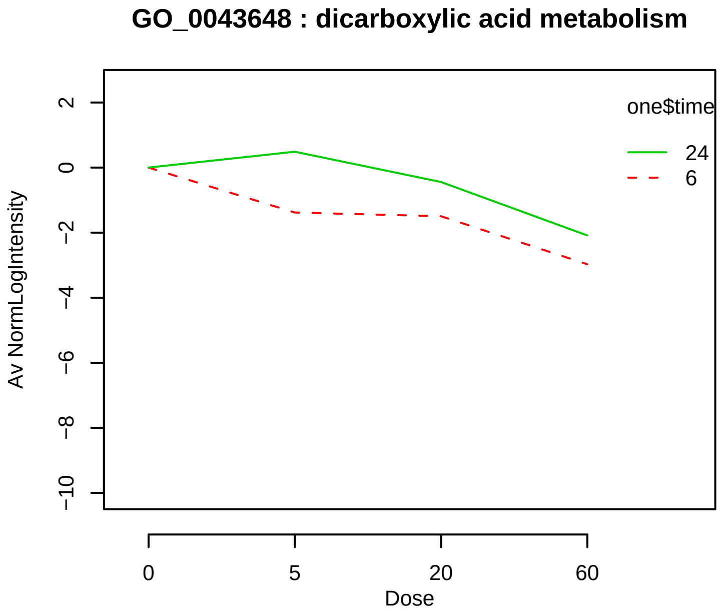
GO_0043648 : dicarboxylic acid metabolism
Av NormLogIntensity
Dose
one$time
24
6

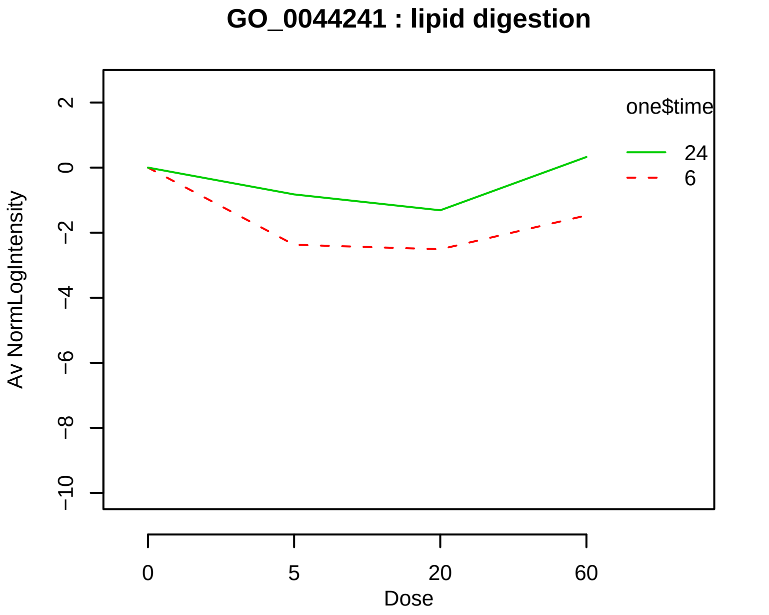
GO_0044241 : lipid digestion
Av NormLogIntensity
Dose
one$time
24
6

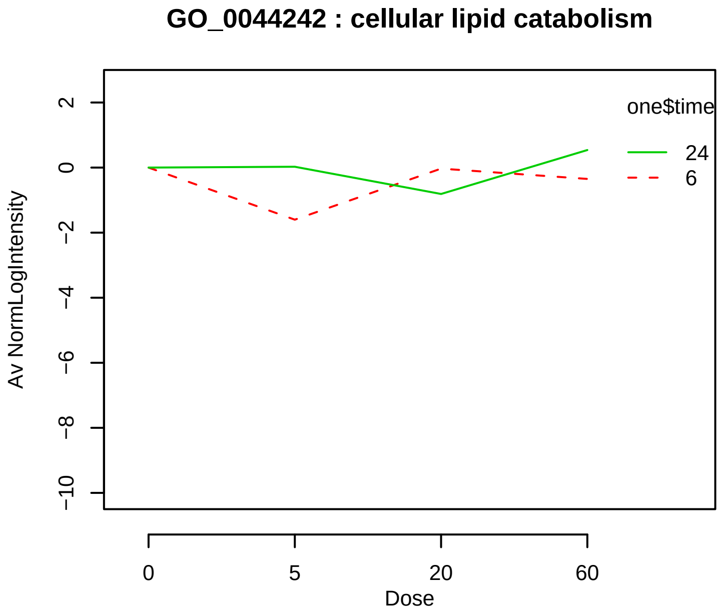
GO_0044242 : cellular lipid catabolism
one$time
24
6
Av NormLogIntensity
Dose
0
5
20
60
2
0
−2
−4
−6
−8
−10

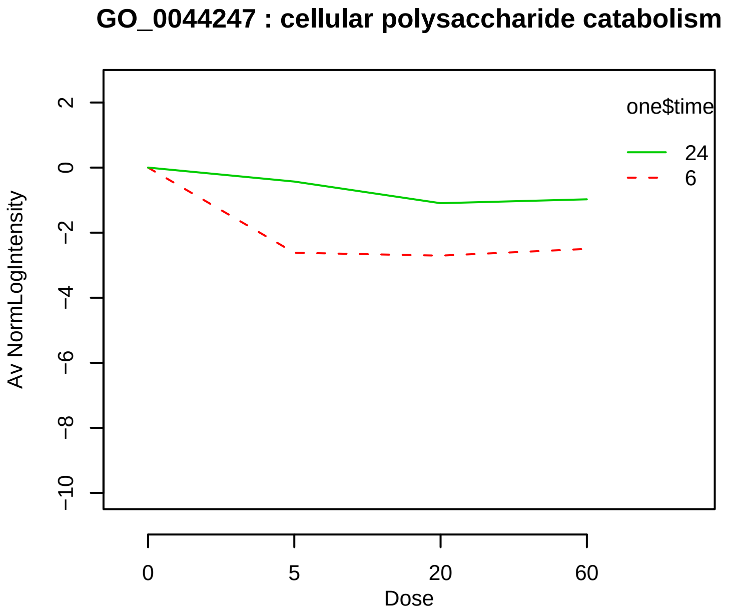
GO_0044247 : cellular polysaccharide catabolism
one$time
24
6
Av NormLogIntensity
Dose
0
5
20
60
2
0
−2
−4
−6
−8
−10

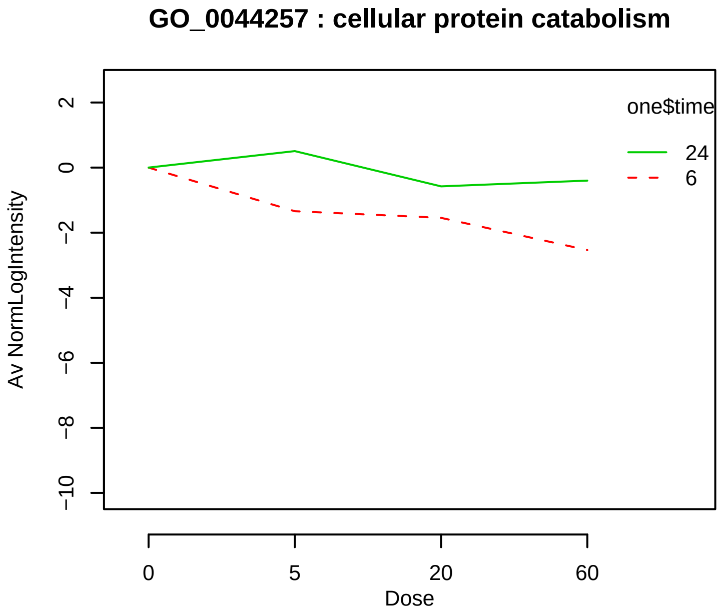
GO_0044257 : cellular protein catabolism
one$time
24
6
Av NormLogIntensity
Dose
0
5
20
60
2
0
−2
−4
−6
−8
−10

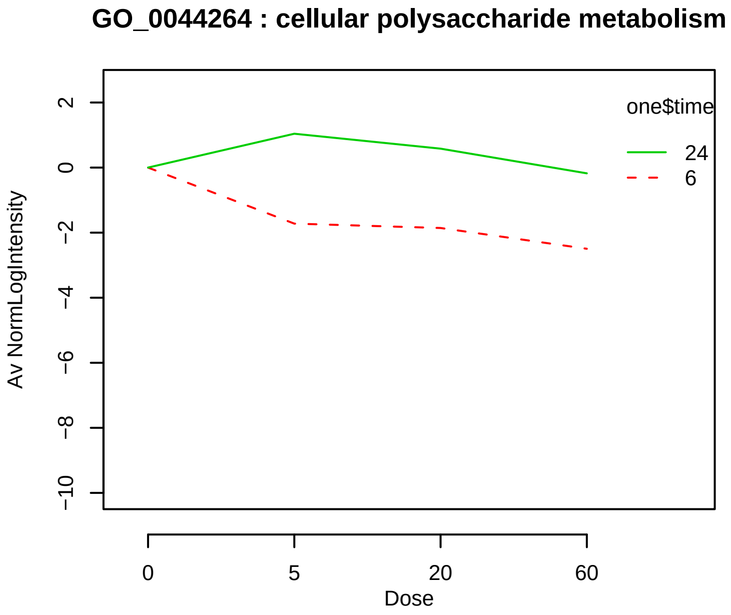
GO_0044264 : cellular polysaccharide metabolism
one$time
24
6
Av NormLogIntensity
Dose
0
5
20
60
2
0
−2
−4
−6
−8
−10

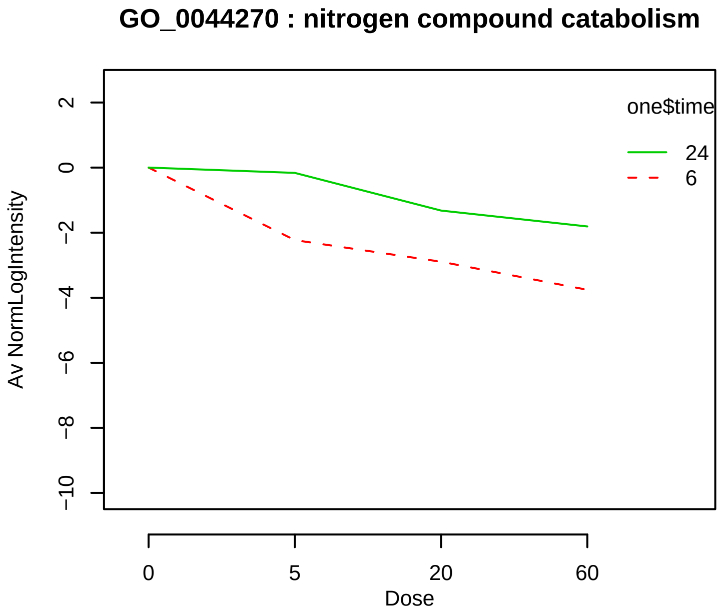
GO_0044270 : nitrogen compound catabolism
one$time
24
6
Av NormLogIntensity
Dose
0
5
20
60
2
0
−2
−4
−6
−8
−10

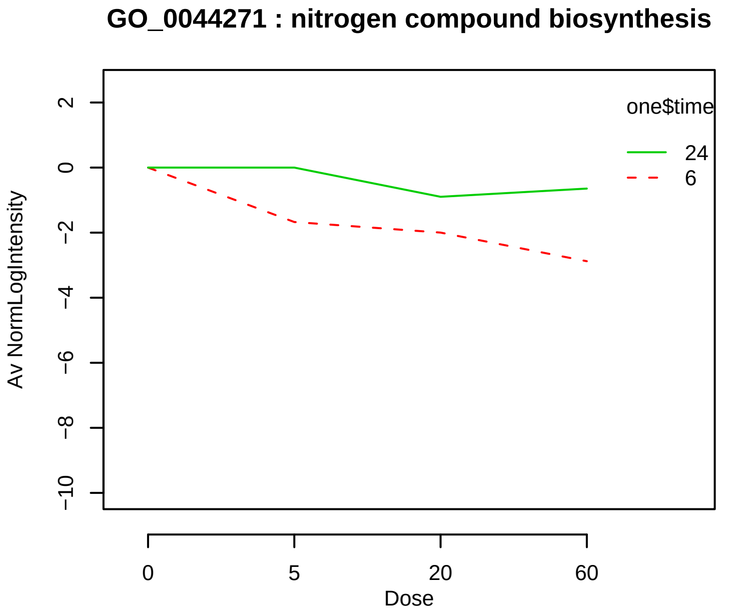
GO_0044271 : nitrogen compound biosynthesis
one$time
24
6
Av NormLogIntensity
Dose
0
5
20
60
2
0
−2
−4
−6
−8
−10

GO_0044272 : sulfur compound biosynthesis

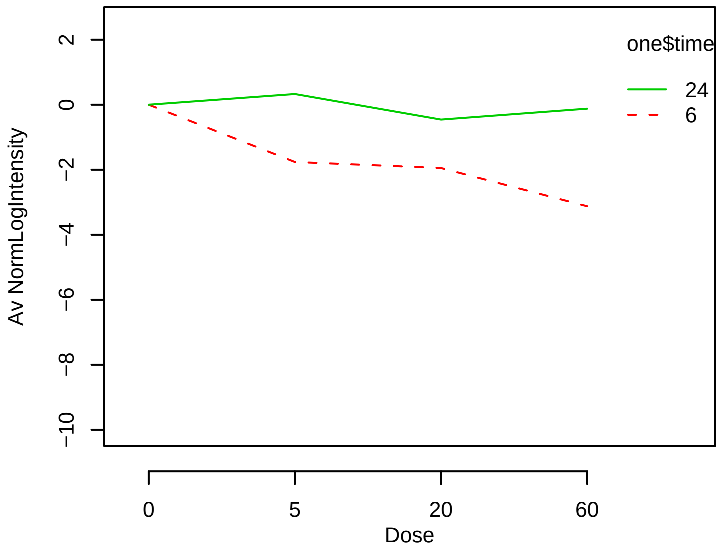

GO_0044275 : cellular carbohydrate catabolism

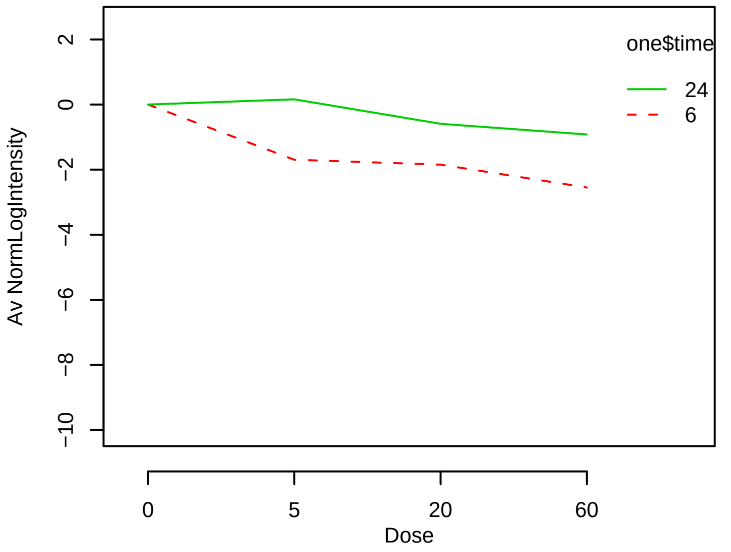

O_0044403 : symbiosis\, encompassing mutualism through p

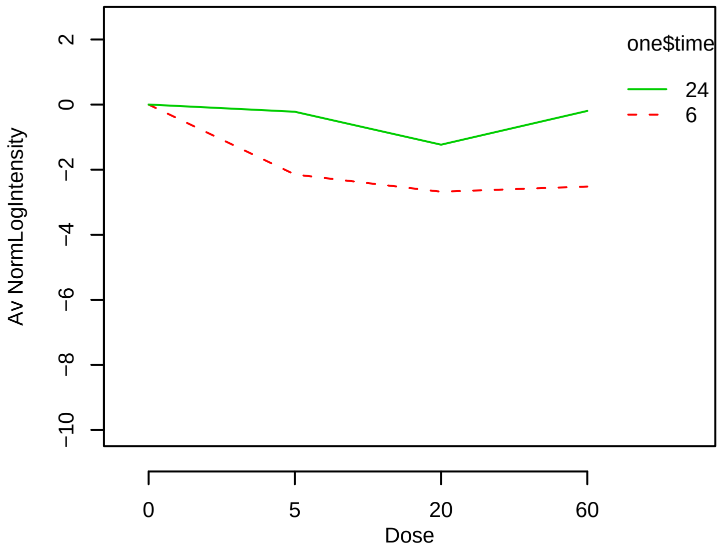

GO_0044409 : entry into host

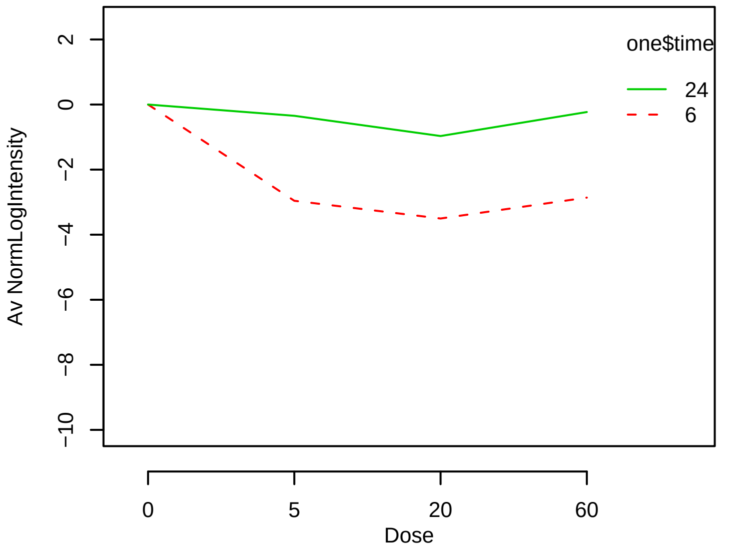

GO_0044419 : interspecies interaction between organisms

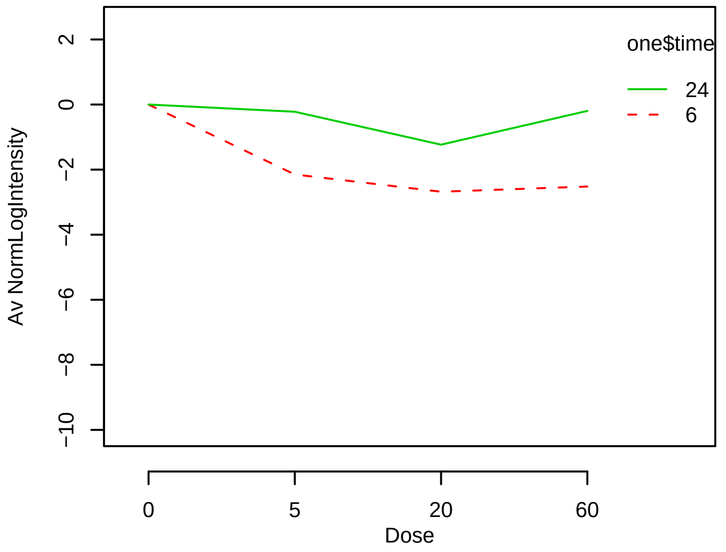

O_0045005 : maintenance of fidelity during DNA−dependent I

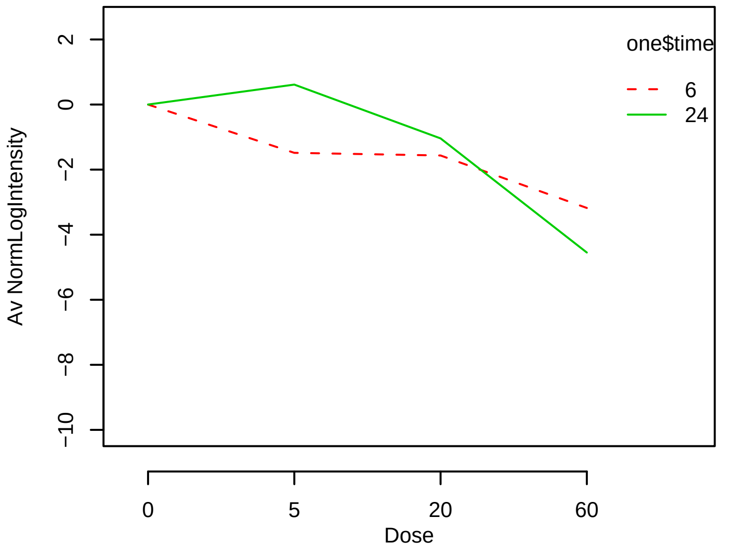

GO_0045017 : glycerolipid biosynthesis

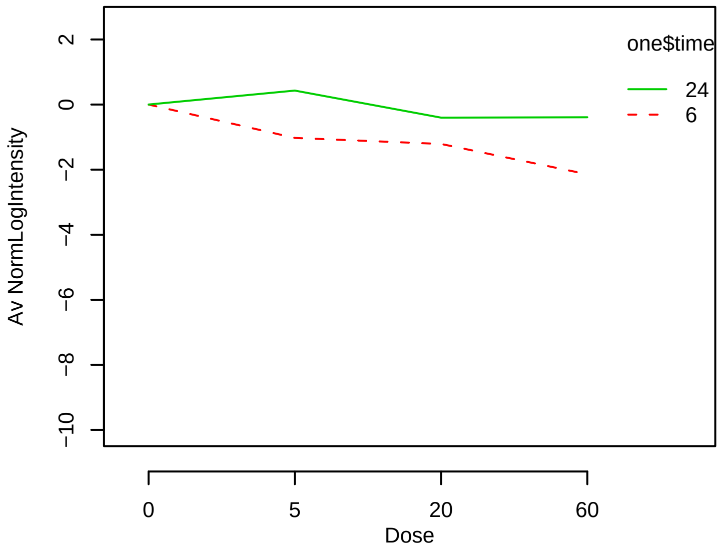

GO_0045026 : plasma membrane fusion

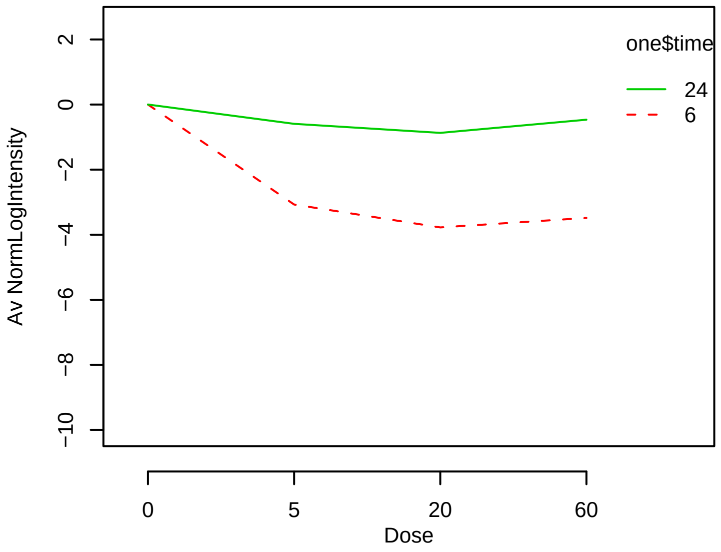

GO_0045039 : protein import into mitochondrial inner membı

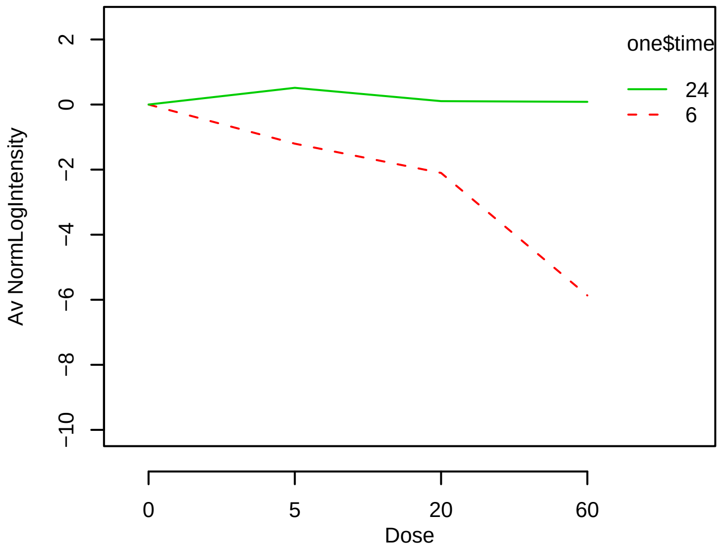

GO_0045045 : secretory pathway

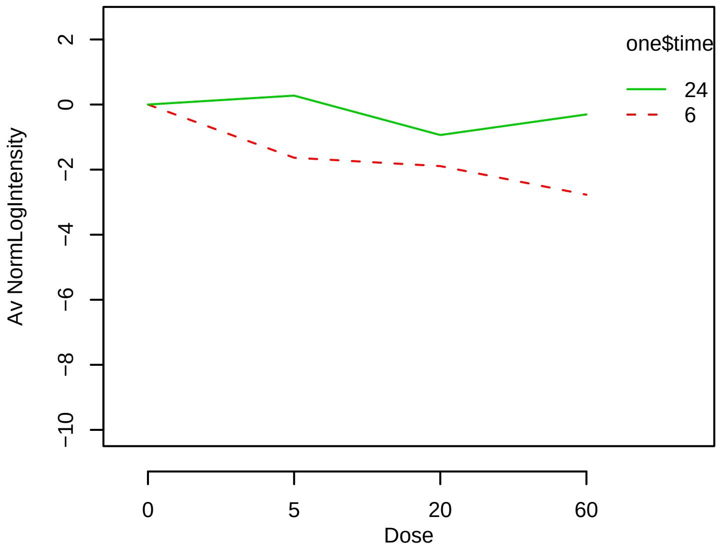

GO_0045047 : protein targeting to ER

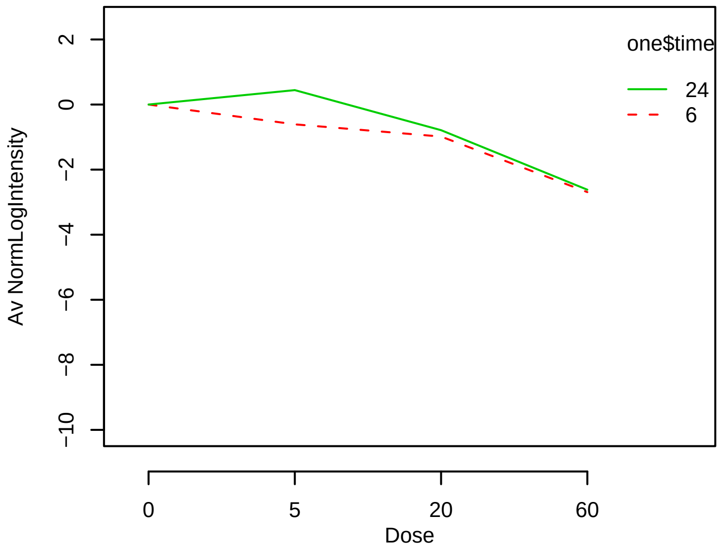

GO_0045055 : regulated secretory pathway

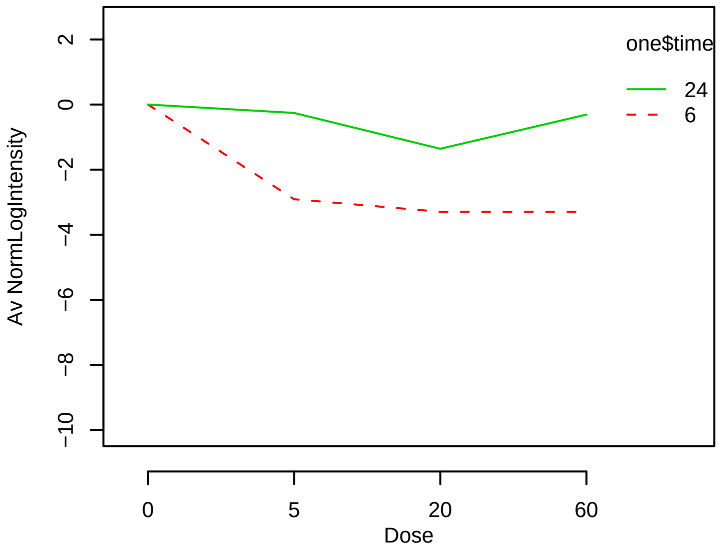

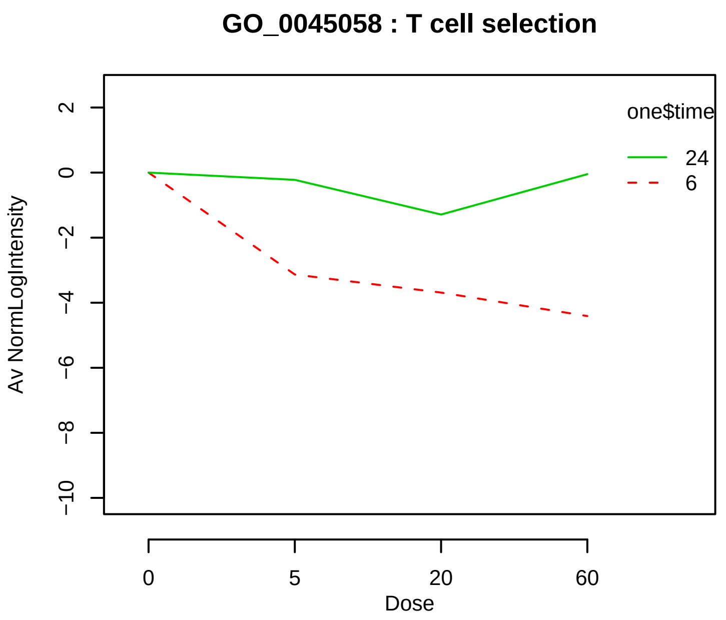
GO_0045058 : T cell selection
one$time
24
6
Av NormLogIntensity
2
0
−2
−4
−6
−8
−10
0
5
20
60
Dose

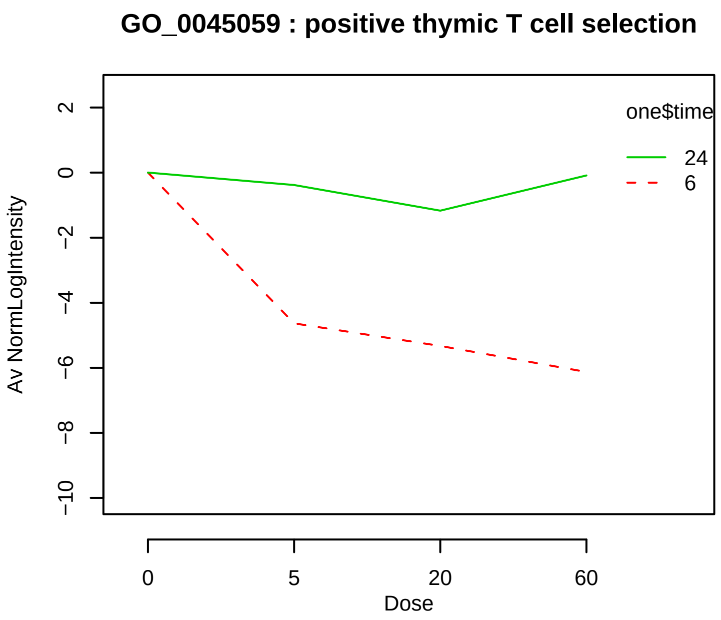
GO_0045059 : positive thymic T cell selection
one$time
24
6
Av NormLogIntensity
2
0
−2
−4
−6
−8
−10
0
5
20
60
Dose

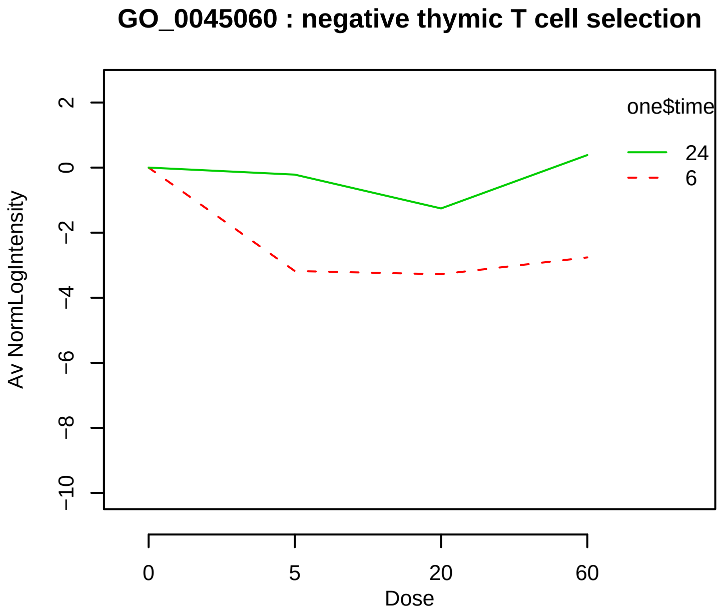
GO_0045060 : negative thymic T cell selection
one$time
24
6
Av NormLogIntensity
2
0
−2
−4
−6
−8
−10
0
5
20
60
Dose

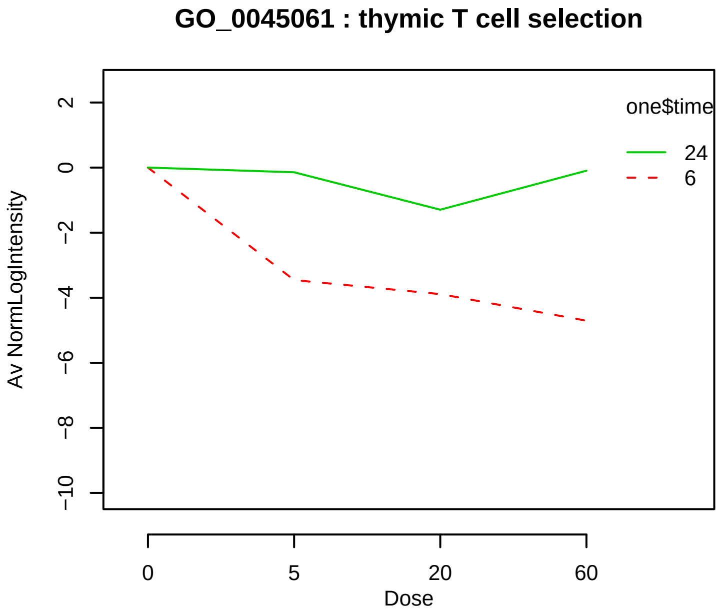
GO_0045061 : thymic T cell selection
one$time
24
6
Av NormLogIntensity
2
0
−2
−4
−6
−8
−10
0
5
20
60
Dose

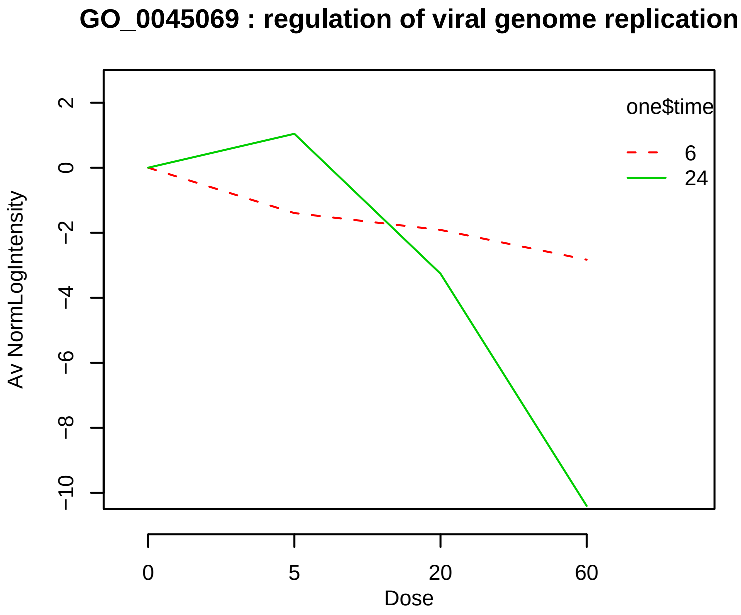
GO_0045069 : regulation of viral genome replication
one$time
6
24
Av NormLogIntensity
2
0
−2
−4
−6
−8
−10
0
5
20
60
Dose

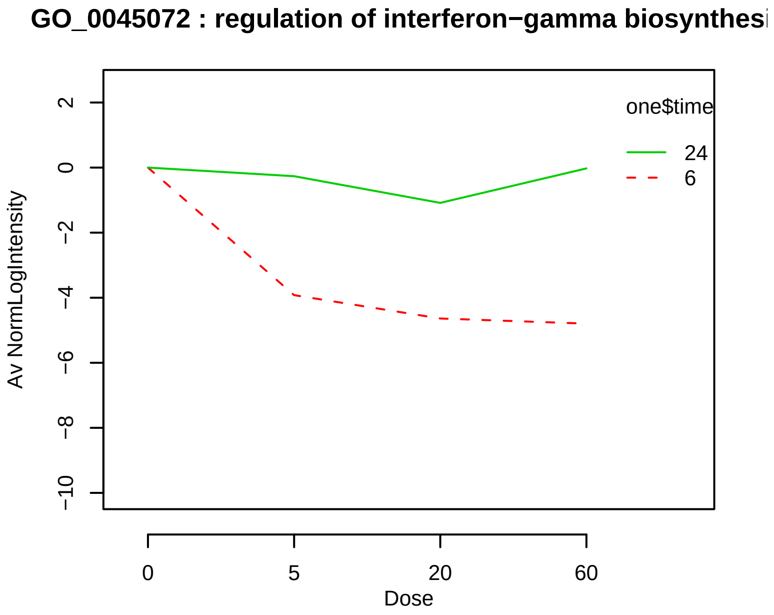
GO_0045072 : regulation of interferon−gamma biosynthesi
one$time
24
6
Av NormLogIntensity
2
0
−2
−4
−6
−8
−10
0
5
20
60
Dose

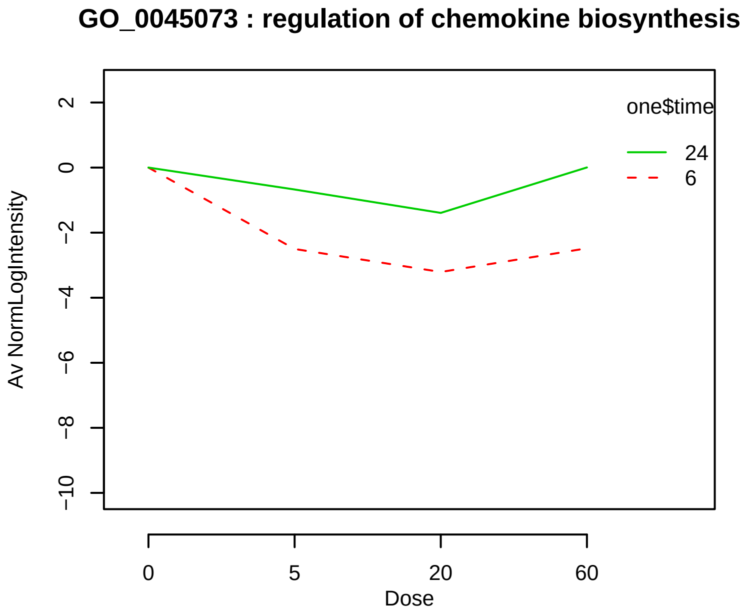
GO_0045073 : regulation of chemokine biosynthesis
one$time
24
6
Av NormLogIntensity
Dose

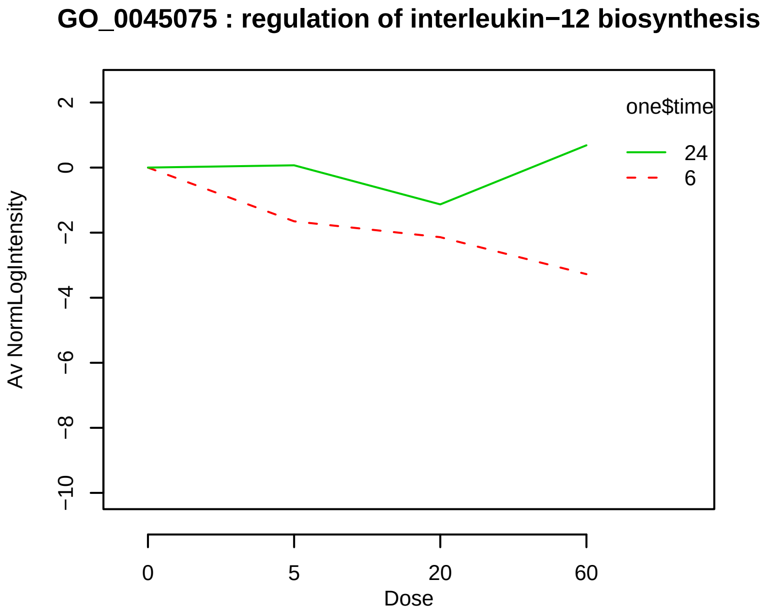
GO_0045075 : regulation of interleukin−12 biosynthesis
one$time
24
6
Av NormLogIntensity
Dose

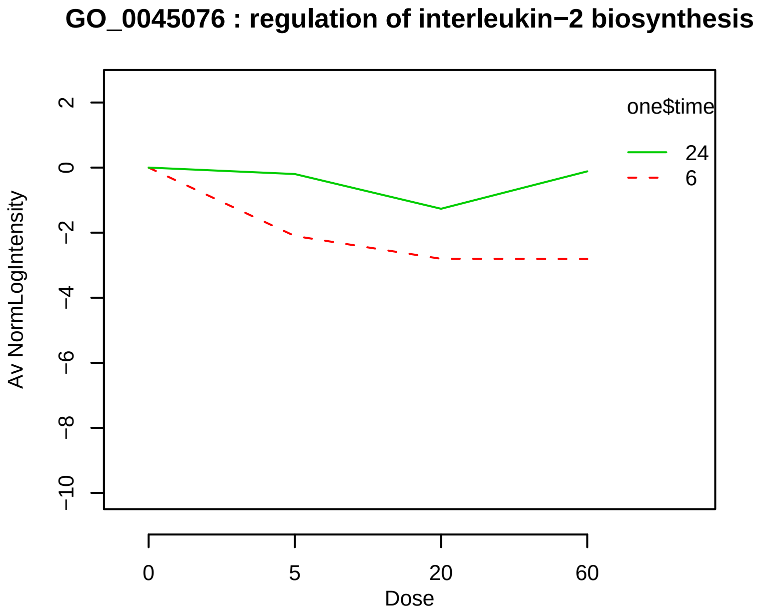
GO_0045076 : regulation of interleukin−2 biosynthesis
one$time
24
6
Av NormLogIntensity
Dose

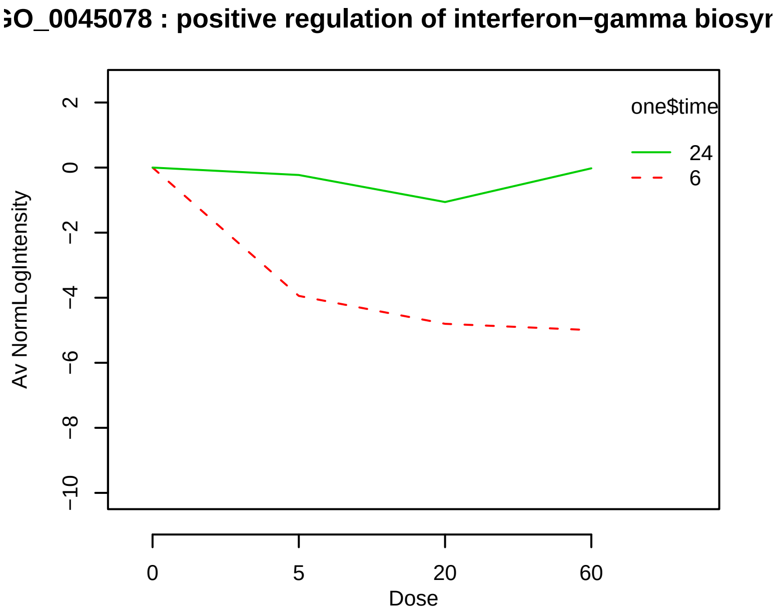
GO_0045078 : positive regulation of interferon−gamma biosyn
one$time
24
6
Av NormLogIntensity
Dose

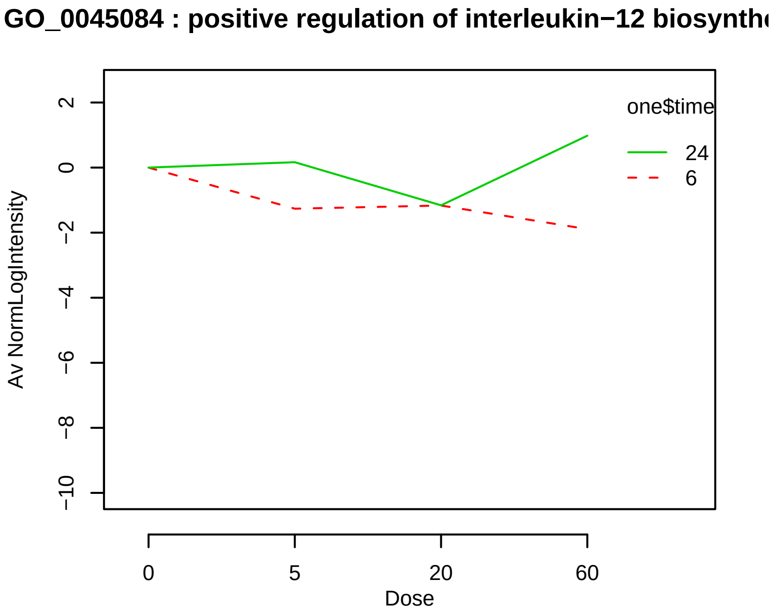
GO_0045084 : positive regulation of interleukin−12 biosynth
one$time
24
6
Av NormLogIntensity
Dose

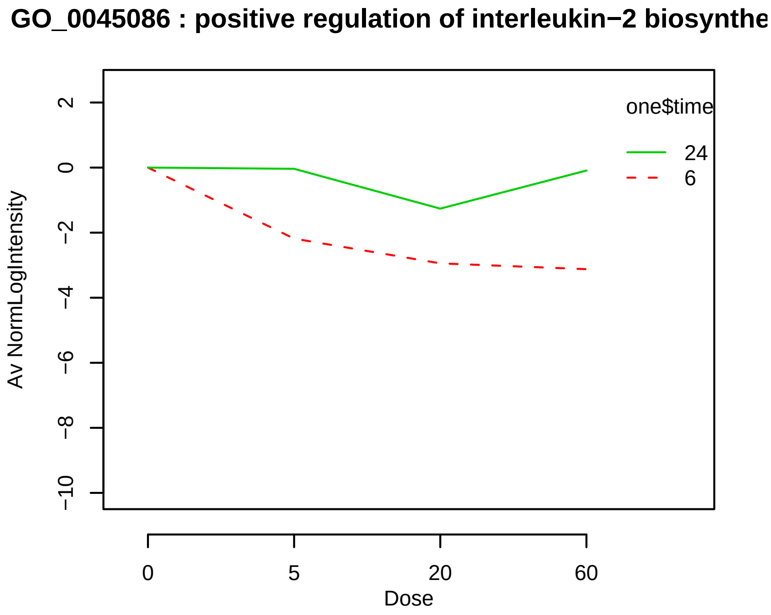
GO_0045086 : positive regulation of interleukin−2 biosynthe
one$time
24
6
Av NormLogIntensity
Dose

GO_0045087 : innate immune response

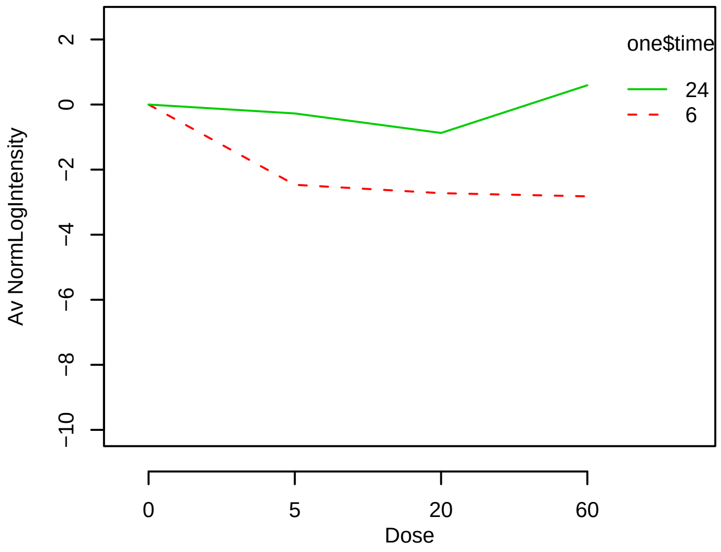

GO_0045088 : regulation of innate immune response

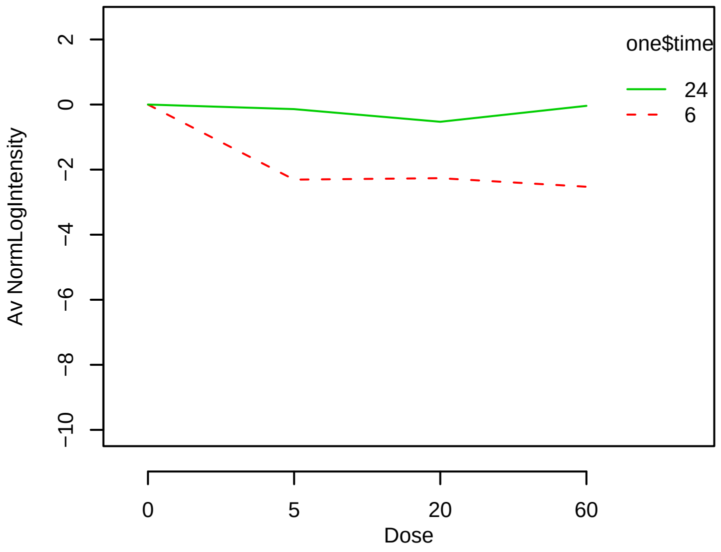

GO_0045089 : positive regulation of innate immune respon

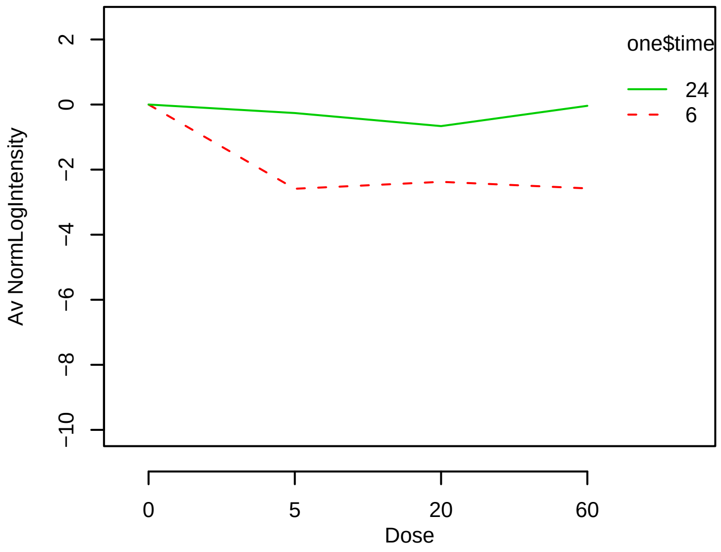

GO_0045103 : intermediate filament−based process

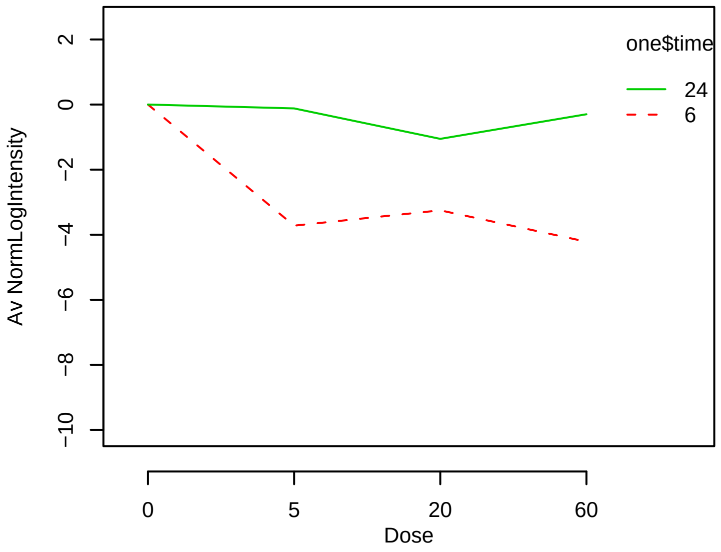

GO_0045104 : intermediate filament cytoskeleton organizatio

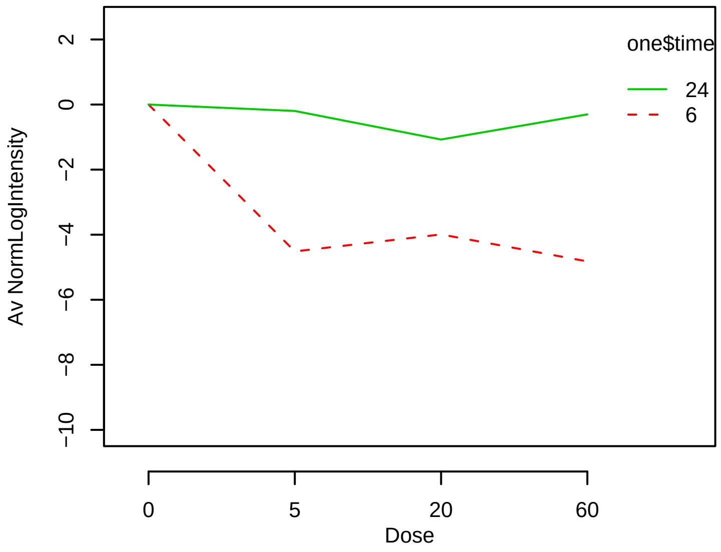

GO_0045137 : development of primary sexual characteristi

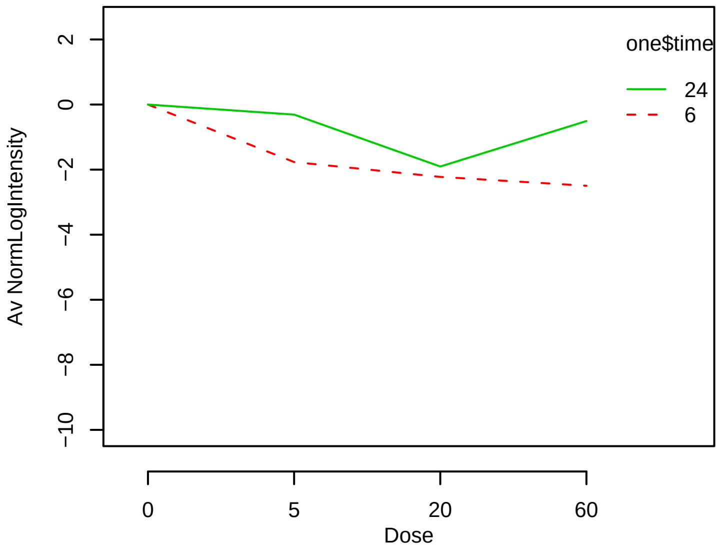

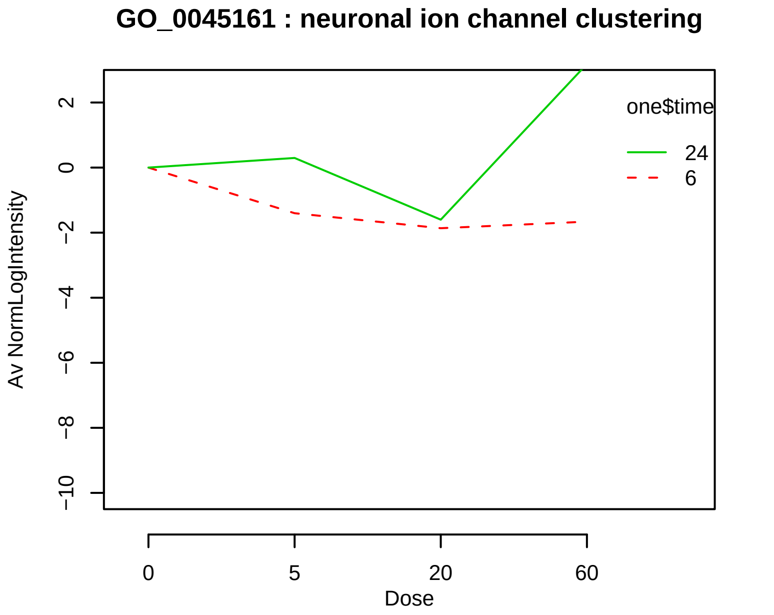

GO_0045161 : neuronal ion channel clustering
Av NormLogIntensity
2
0
−2
−4
−6
−8
−10
one$time
24
6
0
5
20
60
Dose

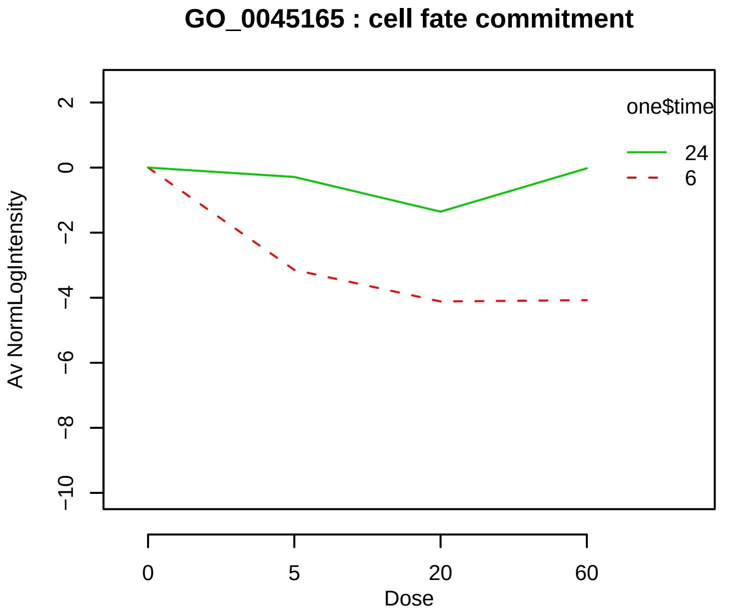

GO_0045165 : cell fate commitment
Av NormLogIntensity
2
0
−2
−4
−6
−8
−10
one$time
24
6
0
5
20
60
Dose

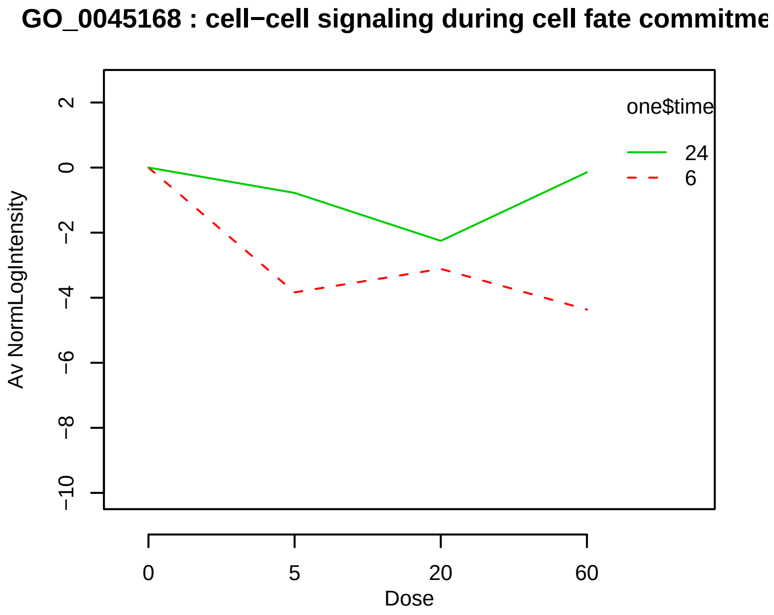

GO_0045168 : cell−cell signaling during cell fate commitme
Av NormLogIntensity
2
0
−2
−4
−6
−8
−10
one$time
24
6
0
5
20
60
Dose

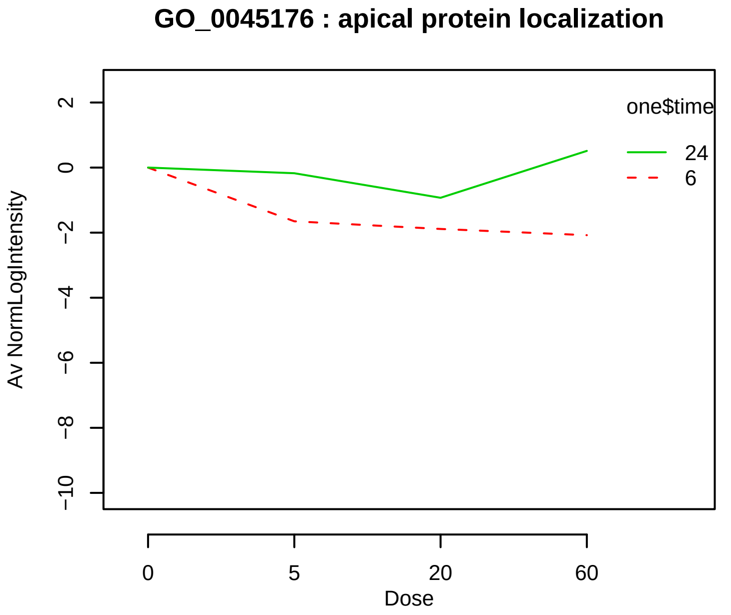

GO_0045176 : apical protein localization
Av NormLogIntensity
2
0
−2
−4
−6
−8
−10
one$time
24
6
0
5
20
60
Dose

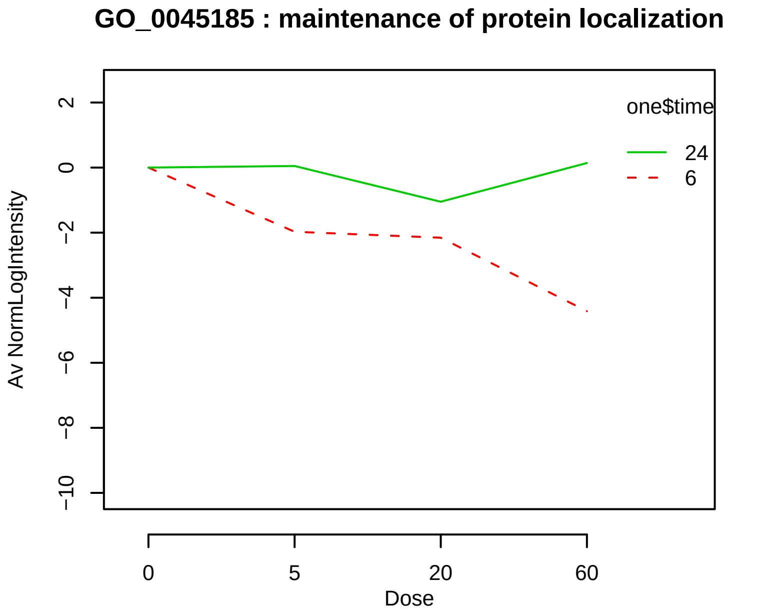

GO_0045185 : maintenance of protein localization
Av NormLogIntensity
2
0
−2
−4
−6
−8
−10
one$time
24
6
0
5
20
60
Dose

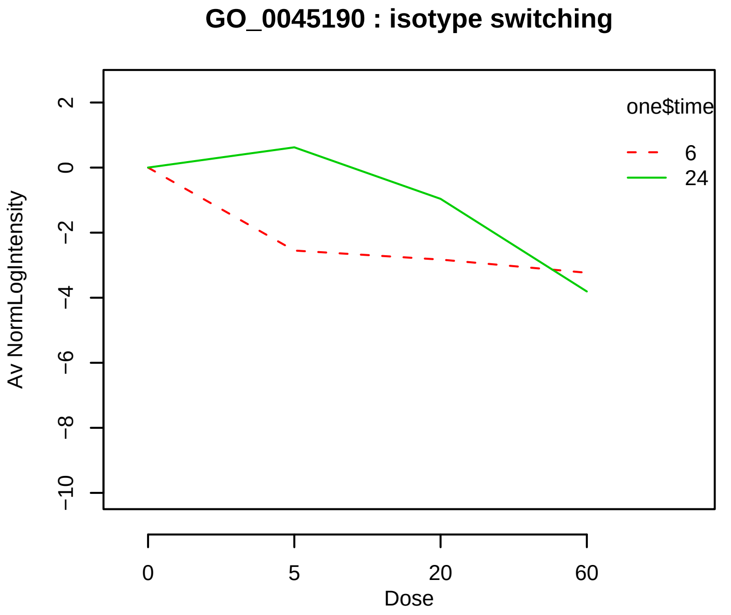

GO_0045190 : isotype switching
Av NormLogIntensity
2
0
−2
−4
−6
−8
−10
one$time
6
24
0
5
20
60
Dose

GO_0045191 : regulation of isotype switching

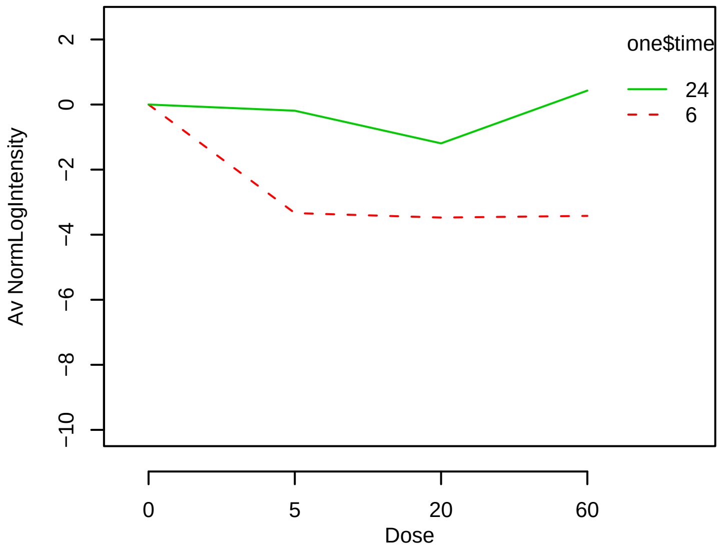

GO_0045197 : establishment and/or maintenance of epithelia

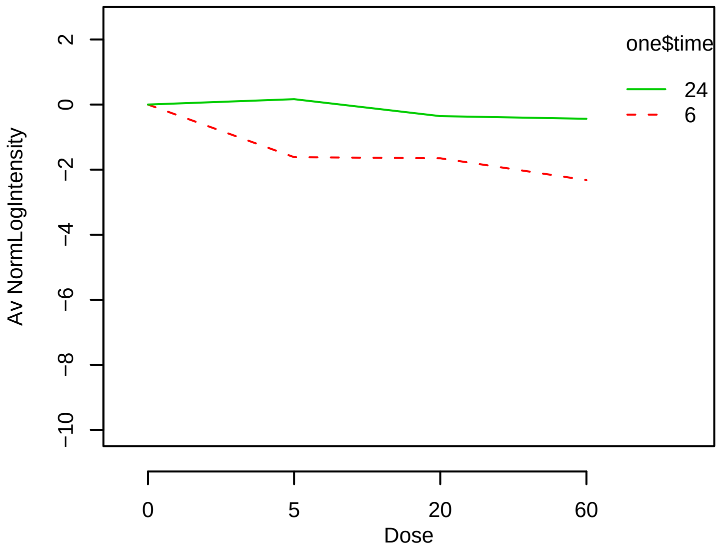

GO_0045216 : intercellular junction assembly and maintenai

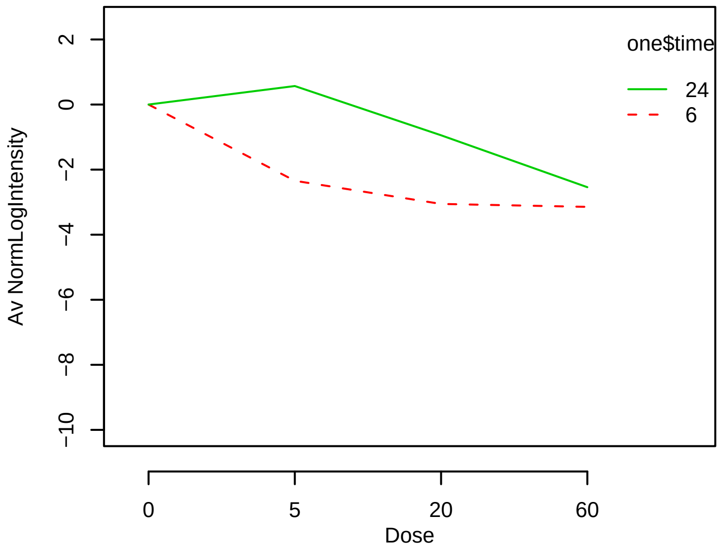

GO_0045321 : leukocyte activation

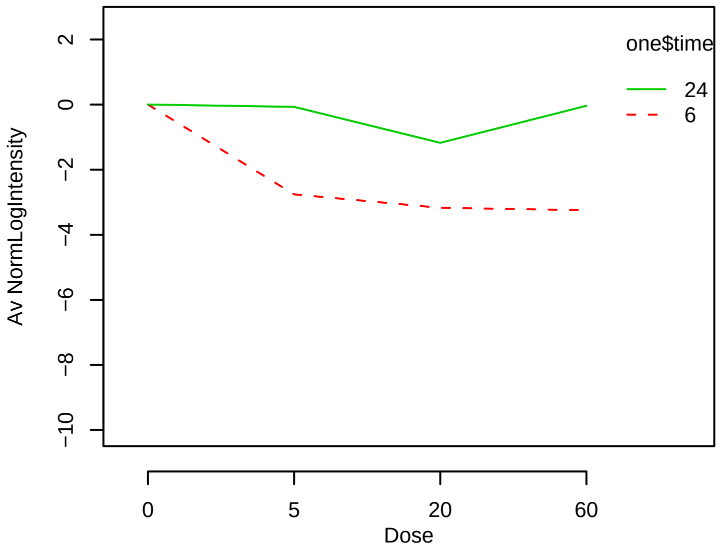

GO_0045333 : cellular respiration

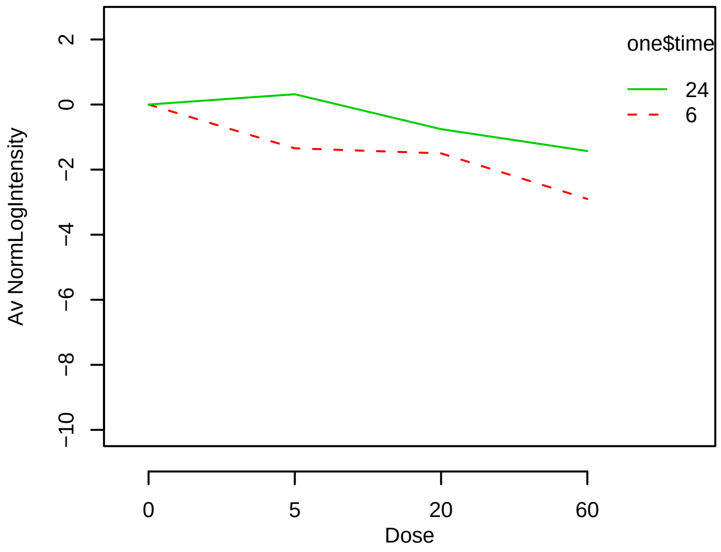

GO_0045342 : MHC class II biosynthesis

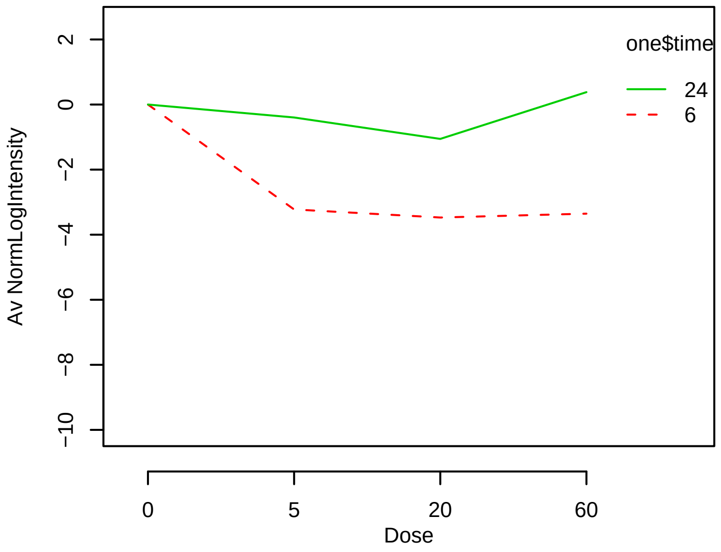

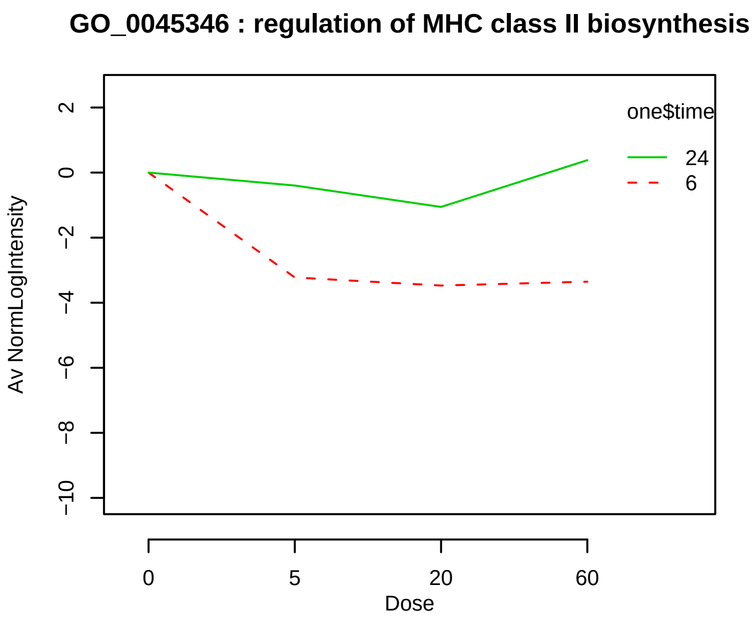
GO_0045346 : regulation of MHC class II biosynthesis
one$time
24
6
Av NormLogIntensity
2
0
−2
−4
−6
−8
−10
0
5
20
60
Dose

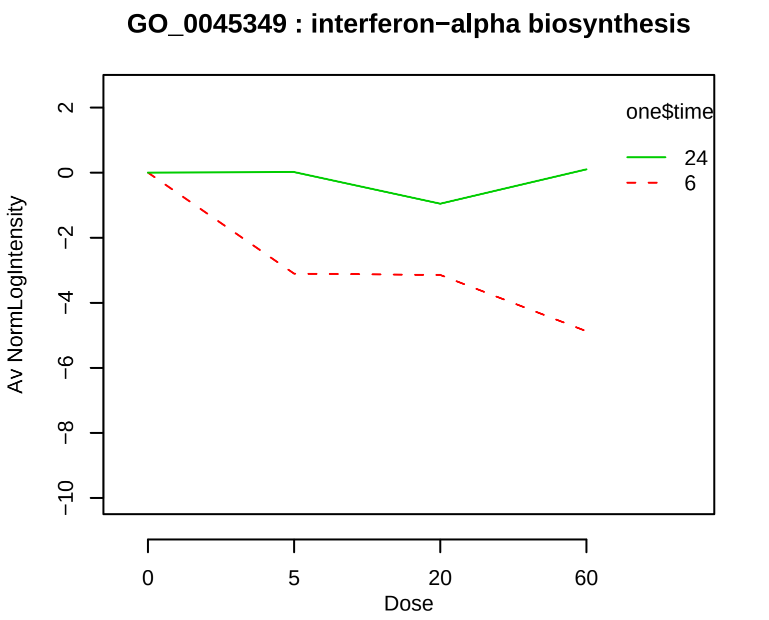
GO_0045349 : interferon−alpha biosynthesis
one$time
24
6
Av NormLogIntensity
2
0
−2
−4
−6
−8
−10
0
5
20
60
Dose

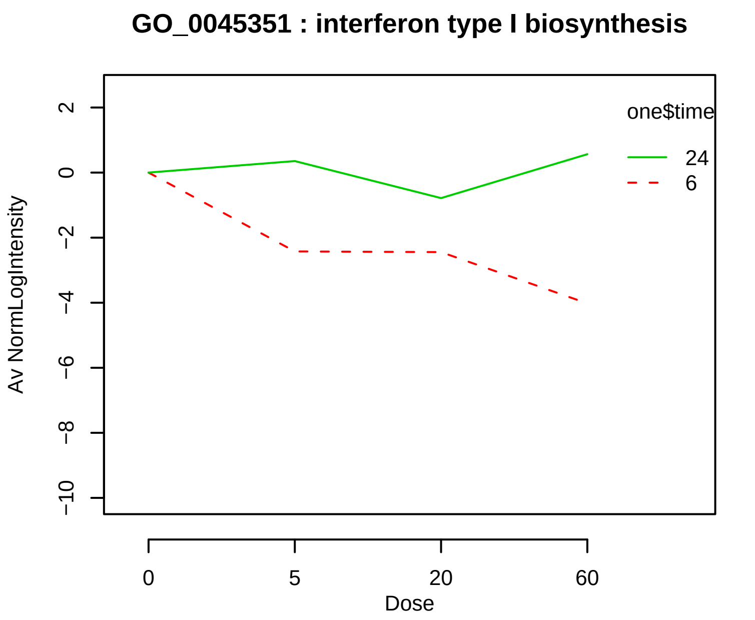
GO_0045351 : interferon type I biosynthesis
one$time
24
6
Av NormLogIntensity
2
0
−2
−4
−6
−8
−10
0
5
20
60
Dose

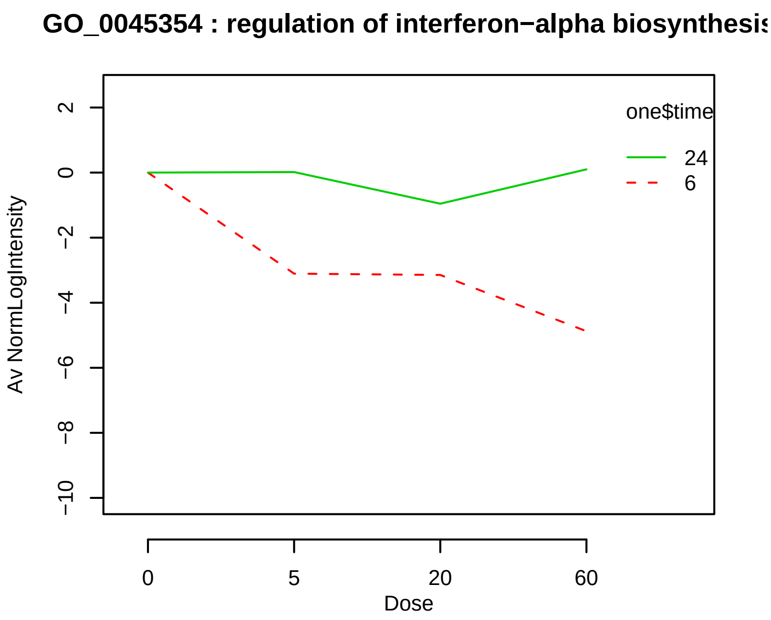
GO_0045354 : regulation of interferon−alpha biosynthesis
one$time
24
6
Av NormLogIntensity
2
0
−2
−4
−6
−8
−10
0
5
20
60
Dose

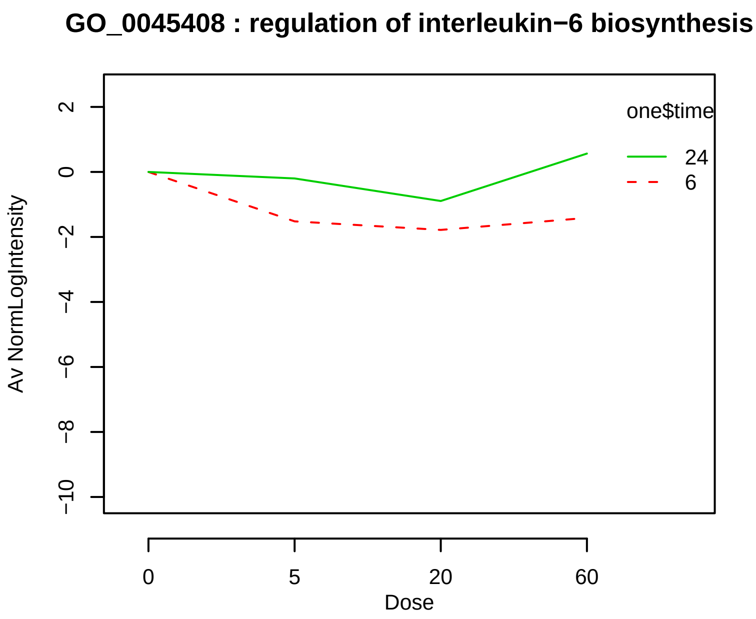
GO_0045408 : regulation of interleukin−6 biosynthesis
one$time
24
6
Av NormLogIntensity
2
0
−2
−4
−6
−8
−10
0
5
20
60
Dose

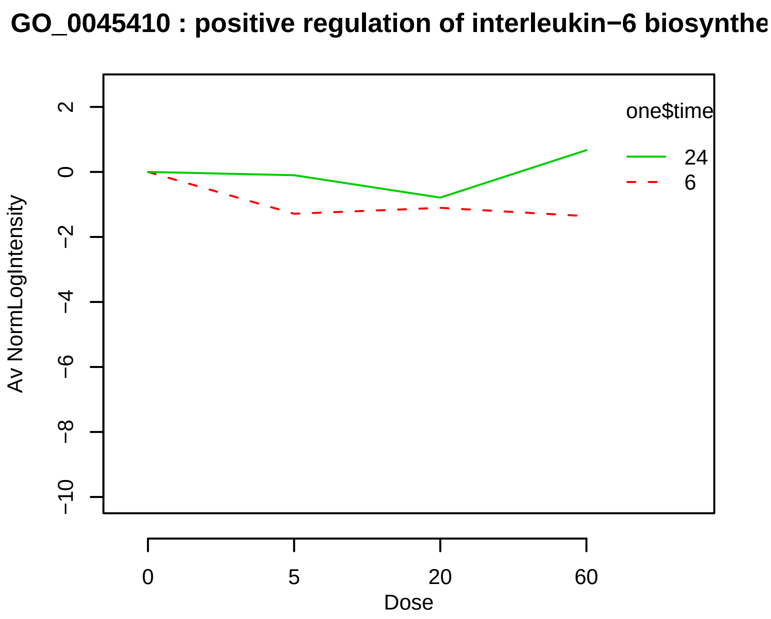
GO_0045410 : positive regulation of interleukin−6 biosynthe
one$time
24
6
Av NormLogIntensity
2
0
−2
−4
−6
−8
−10
0
5
20
60
Dose

GO_0045414 : regulation of interleukin−8 biosynthesis

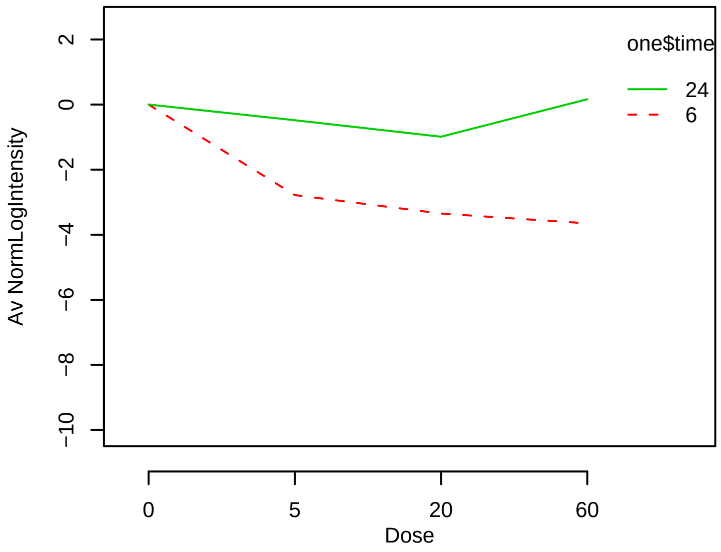

GO_0045416 : positive regulation of interleukin−8 biosynthe

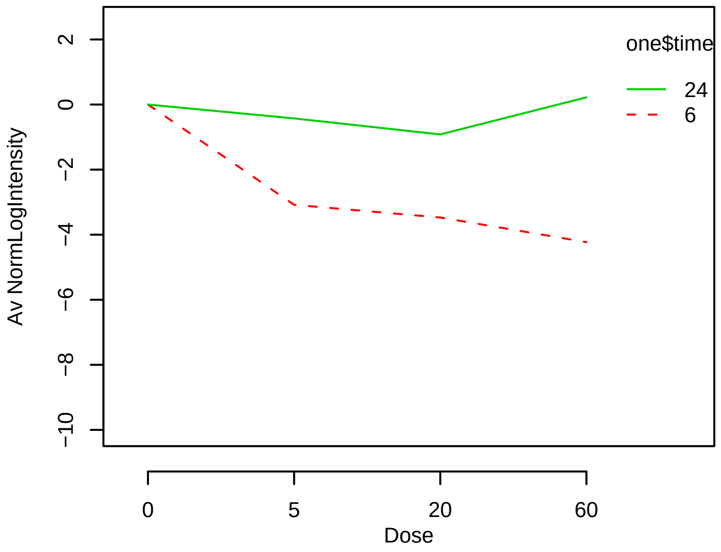

GO_0045426 : quinone cofactor biosynthesis

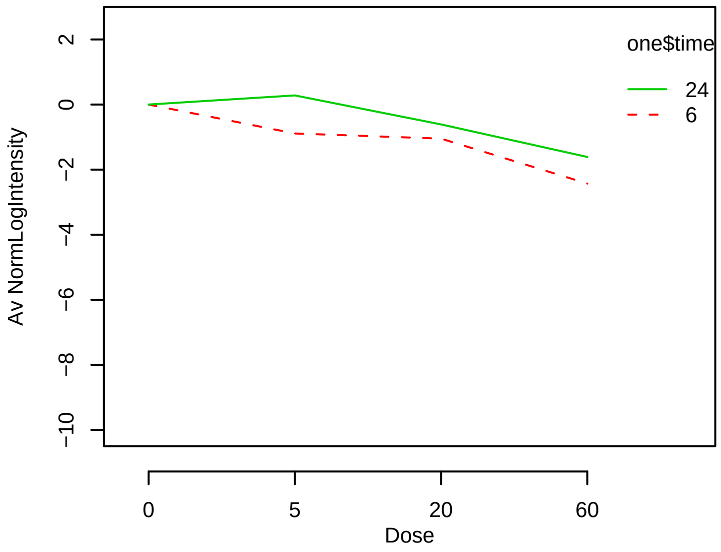

GO_0045428 : regulation of nitric oxide biosynthesis

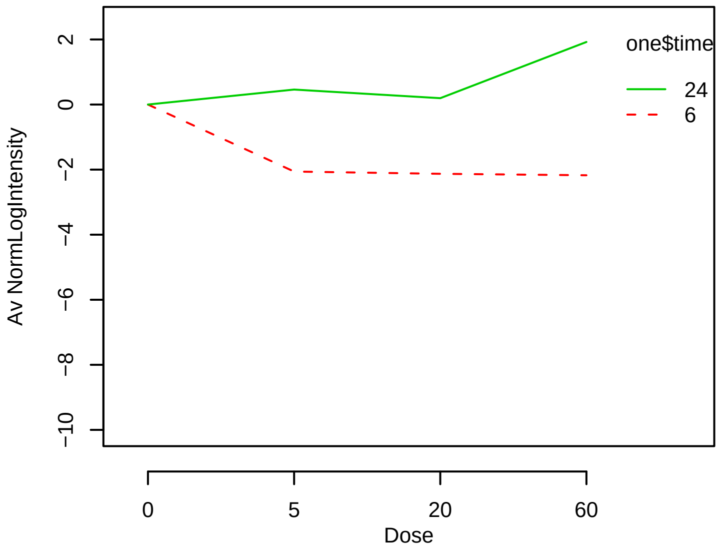

GO_0045444 : fat cell differentiation

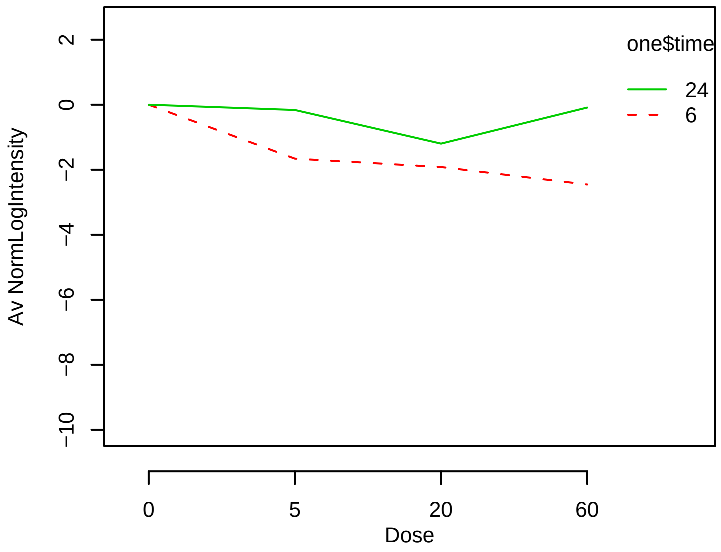

GO_0045445 : myoblast differentiation

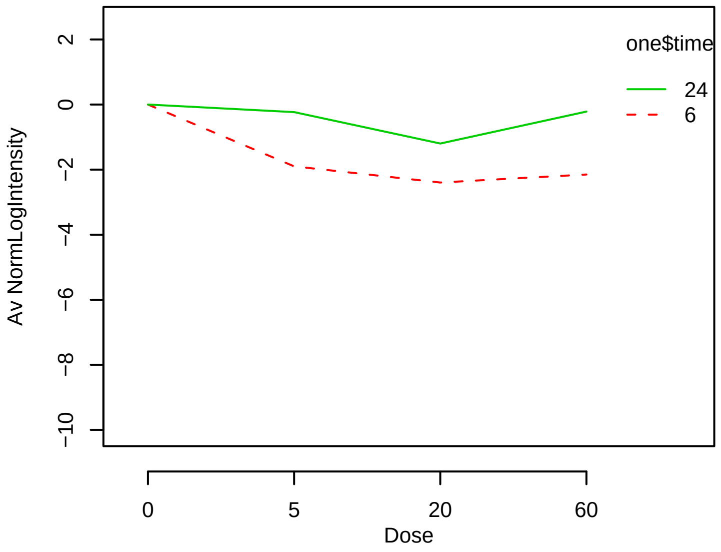

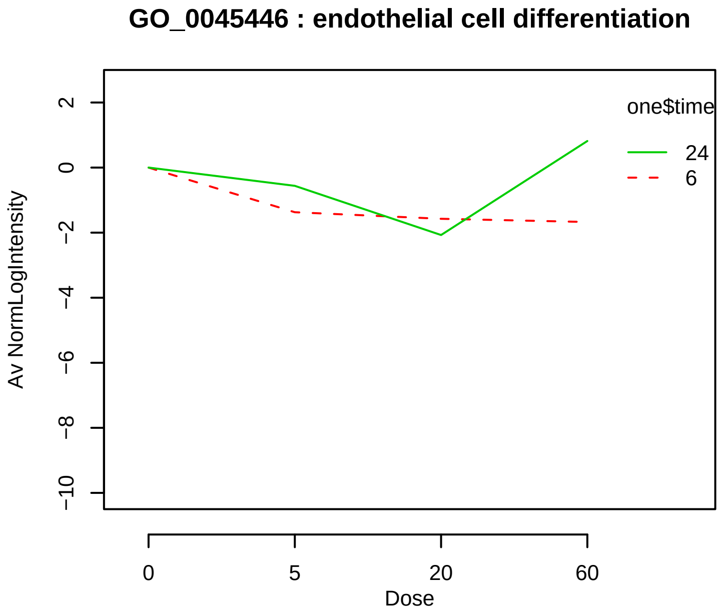

GO_0045446 : endothelial cell differentiation
one$time
24
6
Av NormLogIntensity
2
0
−2
−4
−6
−8
−10
0
5
20
60
Dose

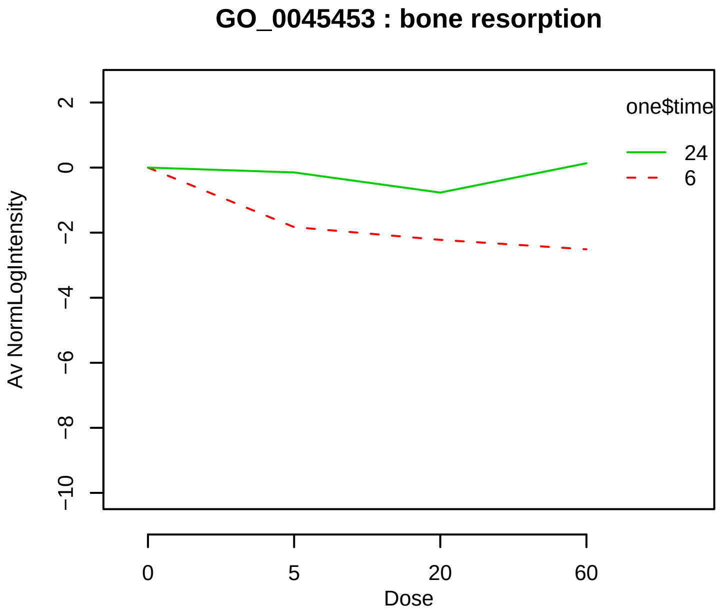

GO_0045453 : bone resorption
one$time
24
6
Av NormLogIntensity
2
0
−2
−4
−6
−8
−10
0
5
20
60
Dose

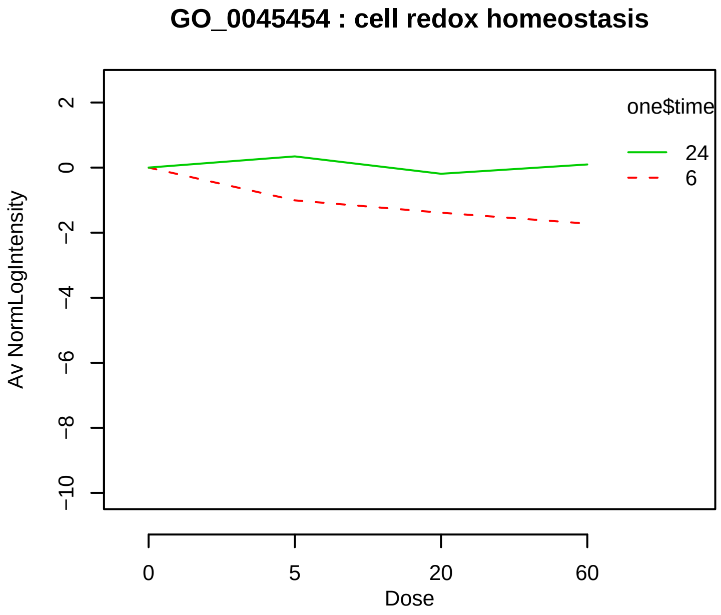

GO_0045454 : cell redox homeostasis
one$time
24
6
Av NormLogIntensity
2
0
−2
−4
−6
−8
−10
0
5
20
60
Dose

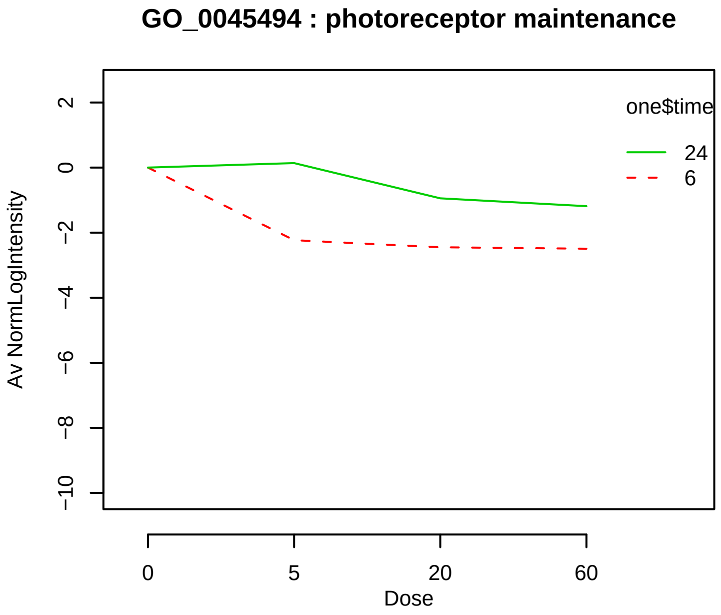

GO_0045494 : photoreceptor maintenance
one$time
24
6
Av NormLogIntensity
2
0
−2
−4
−6
−8
−10
0
5
20
60
Dose

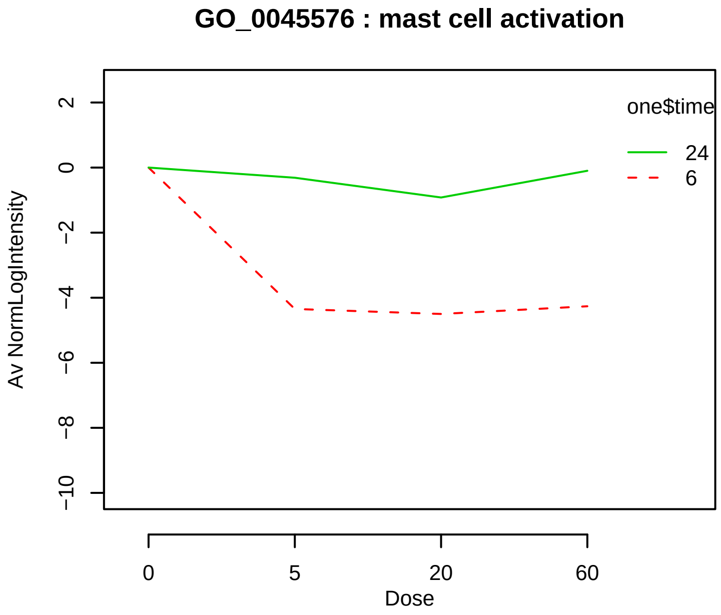

GO_0045576 : mast cell activation
one$time
24
6
Av NormLogIntensity
2
0
−2
−4
−6
−8
−10
0
5
20
60
Dose

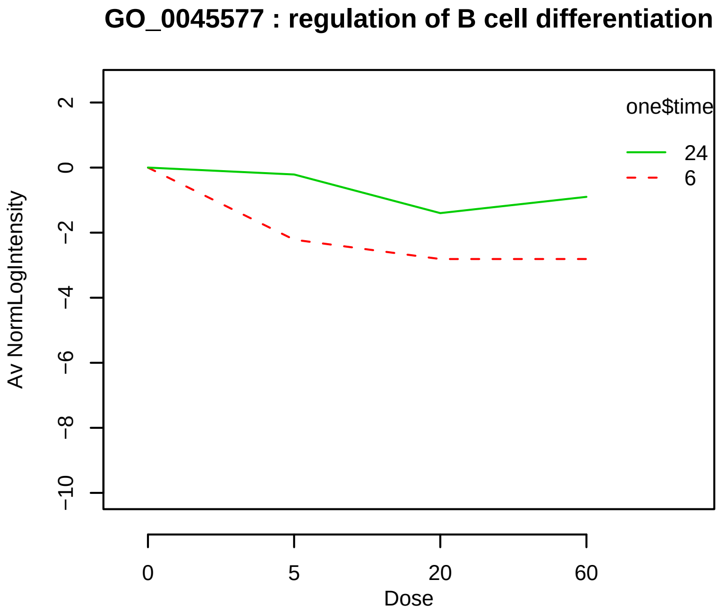

GO_0045577 : regulation of B cell differentiation
one$time
24
6
Av NormLogIntensity
2
0
−2
−4
−6
−8
−10
0
5
20
60
Dose

GO_0045579 : positive regulation of B cell differentiation

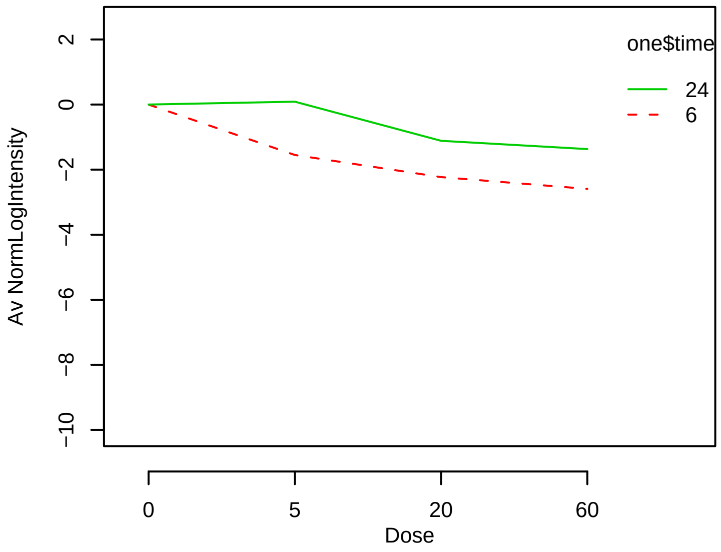

GO_0045580 : regulation of T cell differentiation

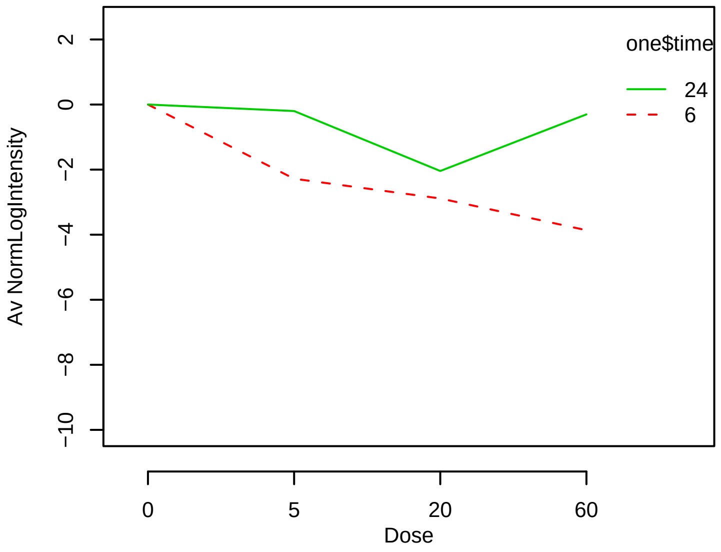

GO_0045582 : positive regulation of T cell differentiation

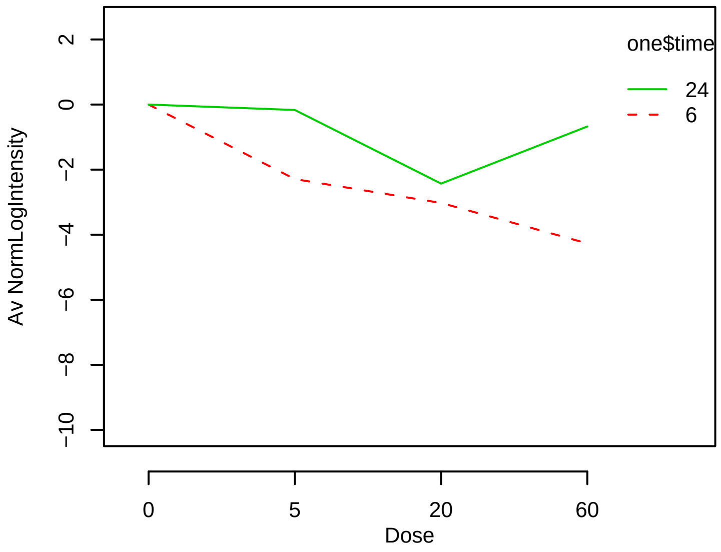

GO_0045595 : regulation of cell differentiation

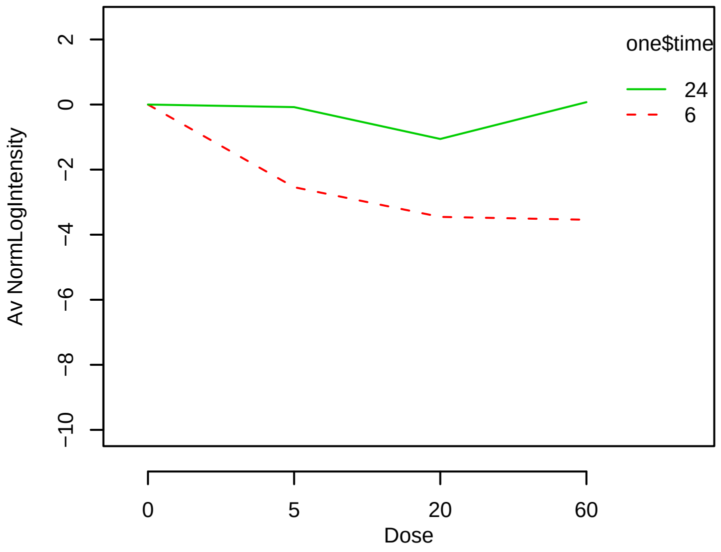

GO_0045596 : negative regulation of cell differentiation

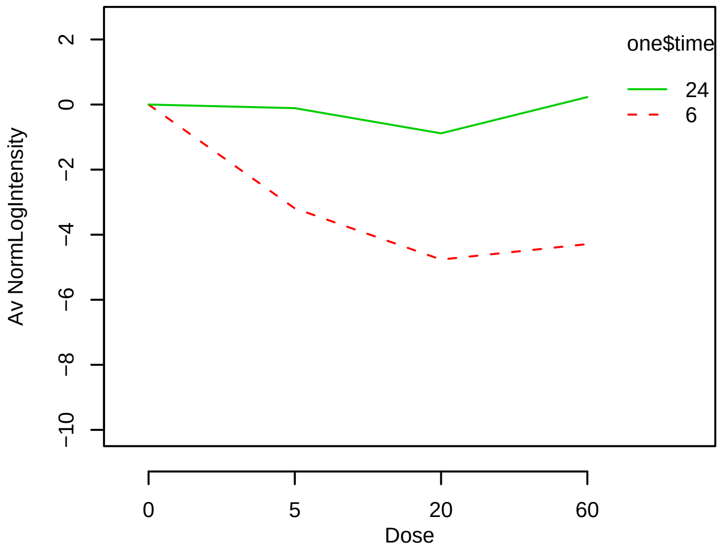

GO_0045597 : positive regulation of cell differentiation

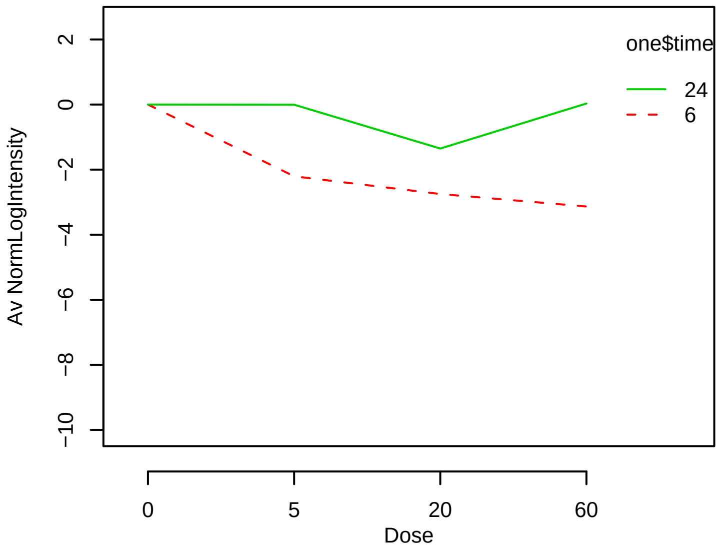

GO_0045598 : regulation of fat cell differentiation

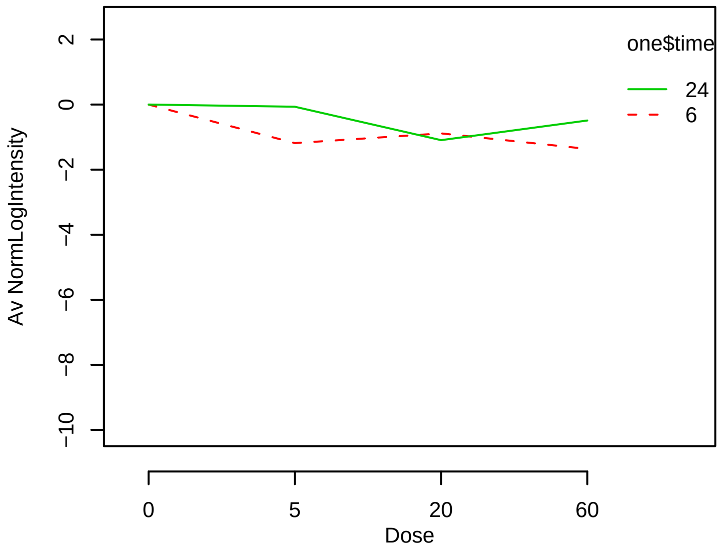

GO_0045604 : regulation of epidermal cell differentiation

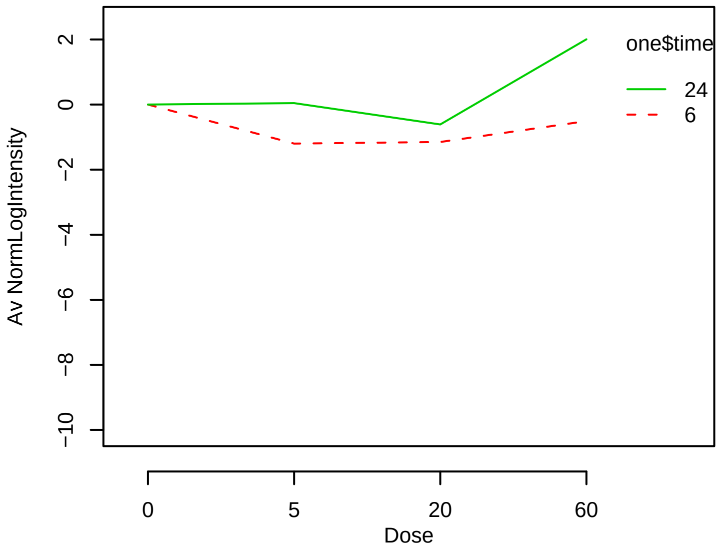

GO_0045619 : regulation of lymphocyte differentiation

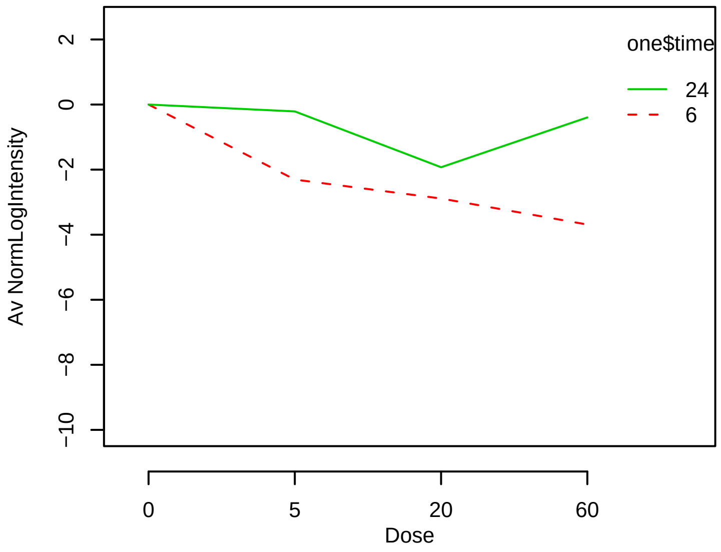

GO_0045620 : negative regulation of lymphocyte differentiat

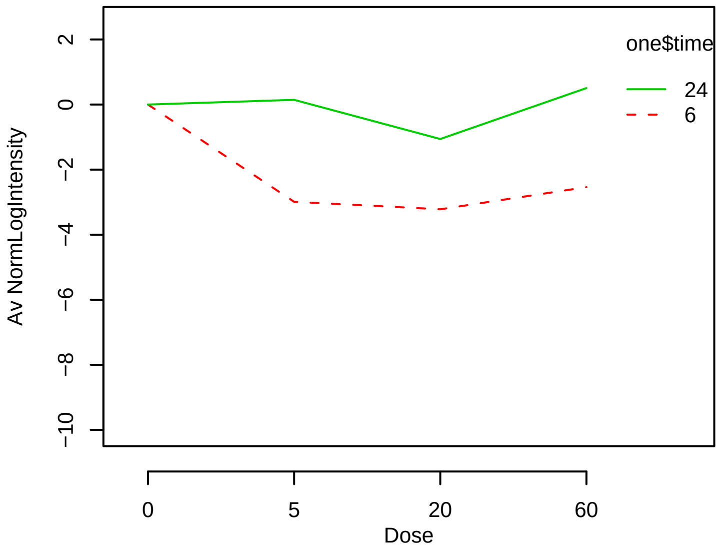

GO_0045621 : positive regulation of lymphocyte differentiat

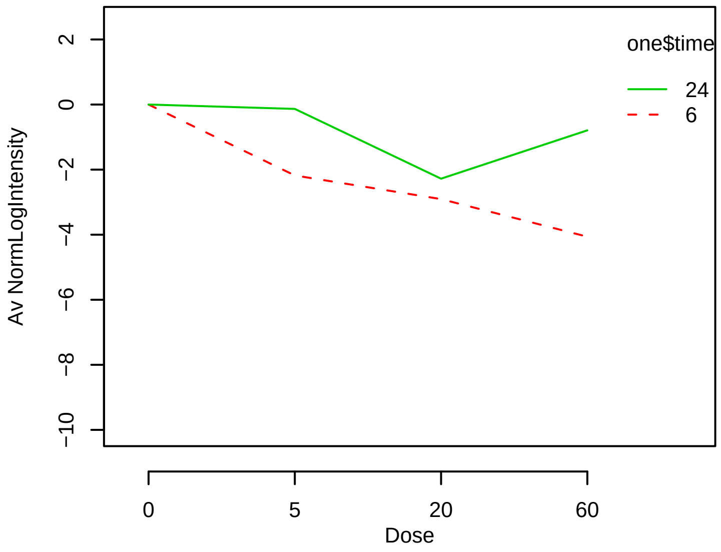

GO_0045622 : regulation of T−helper cell differentiation

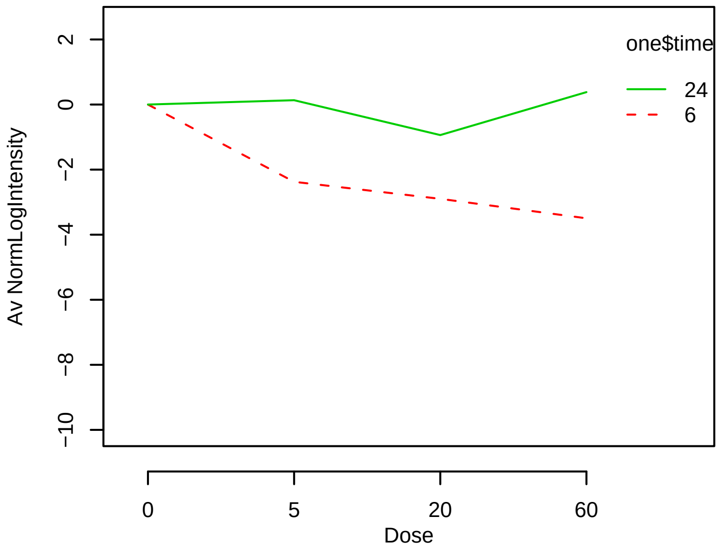

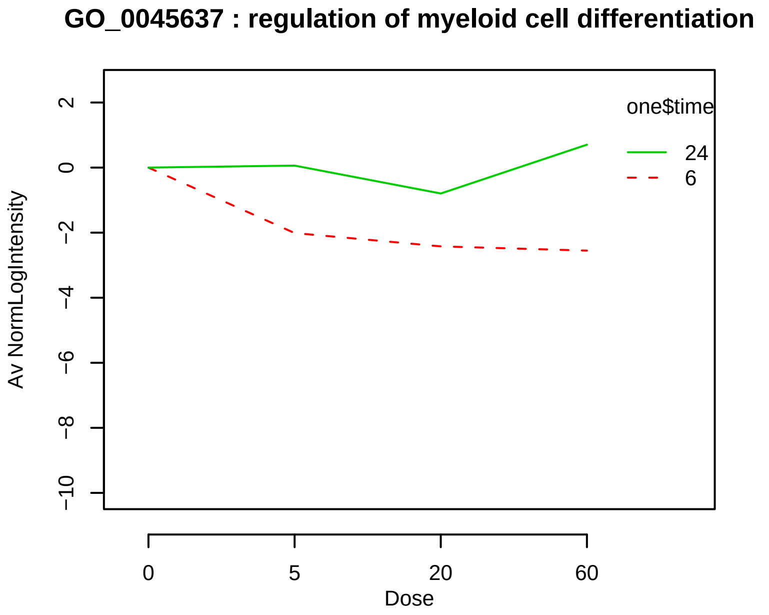
GO_0045637 : regulation of myeloid cell differentiation
Av NormLogIntensity
2
0
−2
−4
−6
−8
−10
one$time
24
6
0
5
20
60
Dose

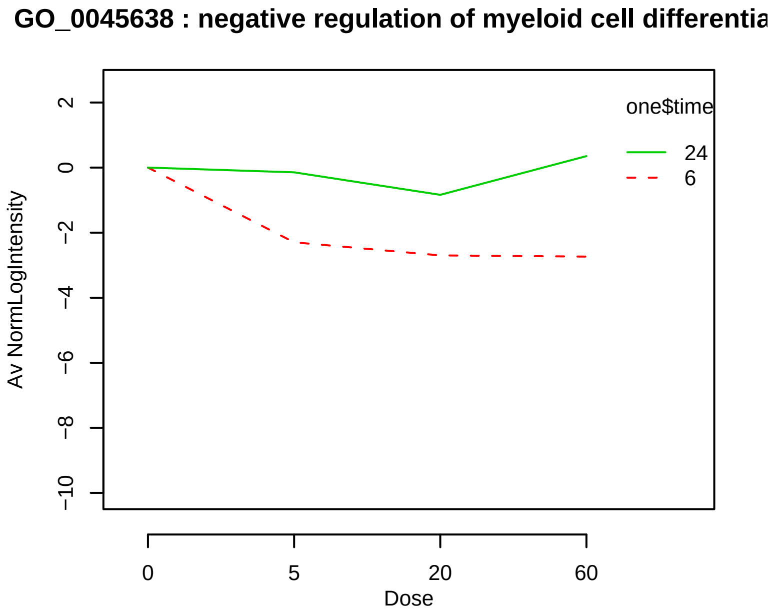
GO_0045638 : negative regulation of myeloid cell differentia
Av NormLogIntensity
2
0
−2
−4
−6
−8
−10
one$time
24
6
0
5
20
60
Dose

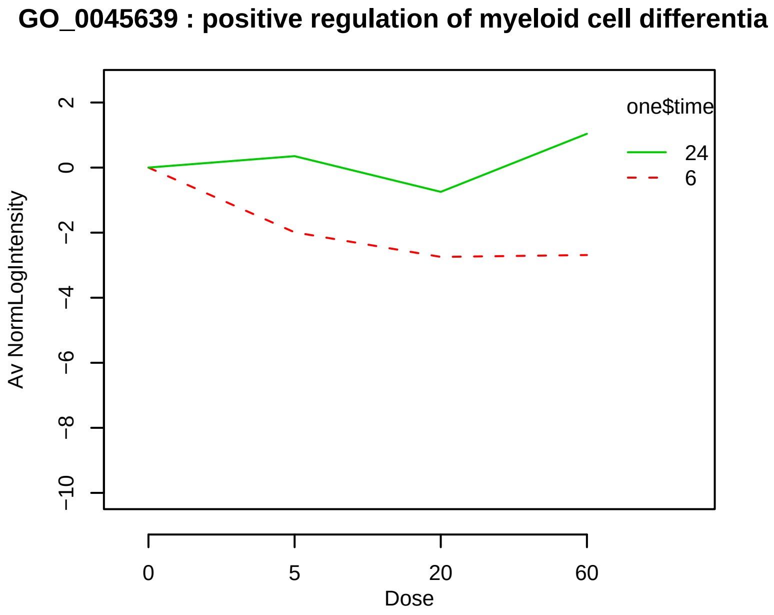
GO_0045639 : positive regulation of myeloid cell differentia
Av NormLogIntensity
2
0
−2
−4
−6
−8
−10
one$time
24
6
0
5
20
60
Dose

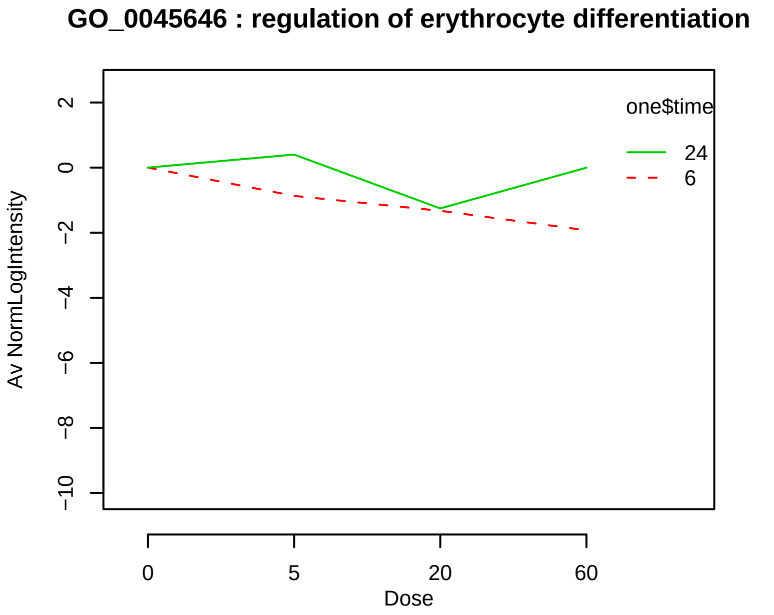
GO_0045646 : regulation of erythrocyte differentiation
Av NormLogIntensity
2
0
−2
−4
−6
−8
−10
one$time
24
6
0
5
20
60
Dose

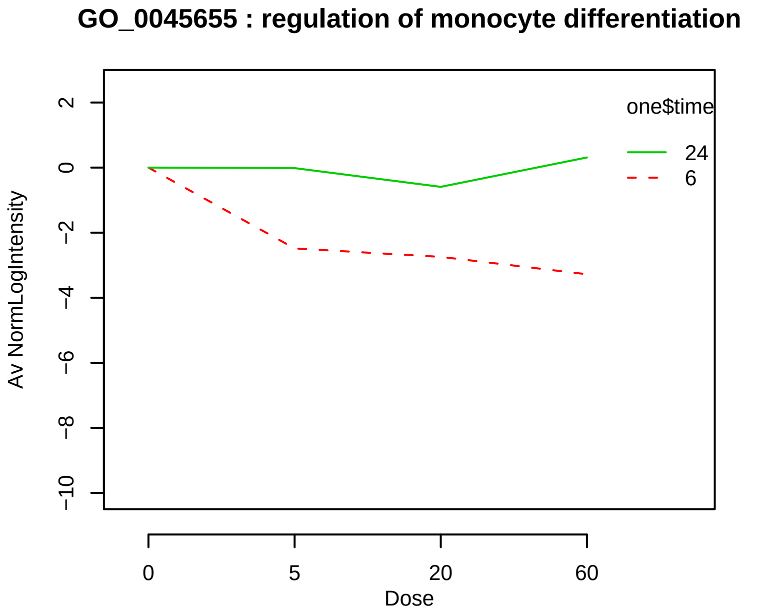
GO_0045655 : regulation of monocyte differentiation
Av NormLogIntensity
2
0
−2
−4
−6
−8
−10
one$time
24
6
0
5
20
60
Dose

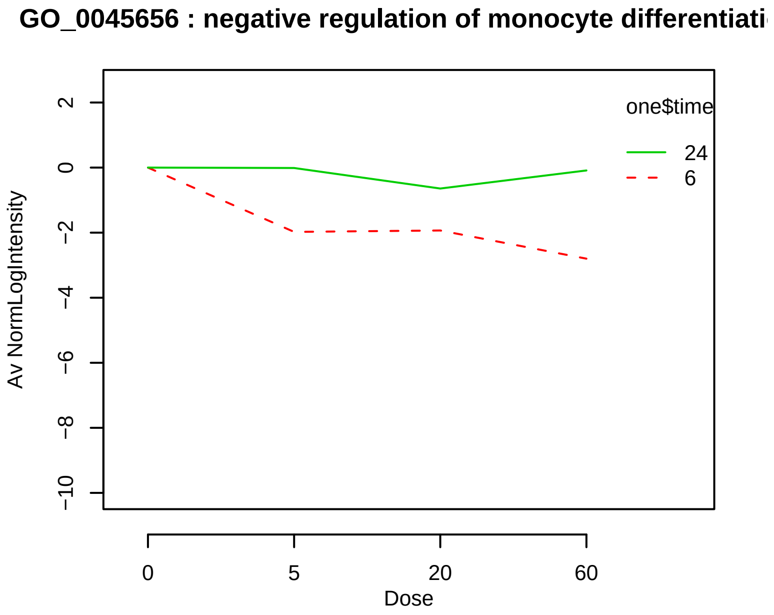
GO_0045656 : negative regulation of monocyte differentiati
Av NormLogIntensity
2
0
−2
−4
−6
−8
−10
one$time
24
6
0
5
20
60
Dose

GO_0045661 : regulation of myoblast differentiation

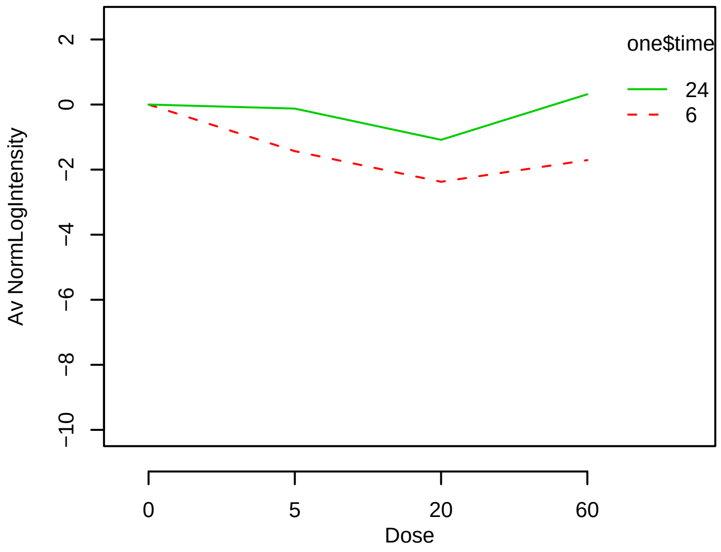

GO_0045664 : regulation of neuron differentiation

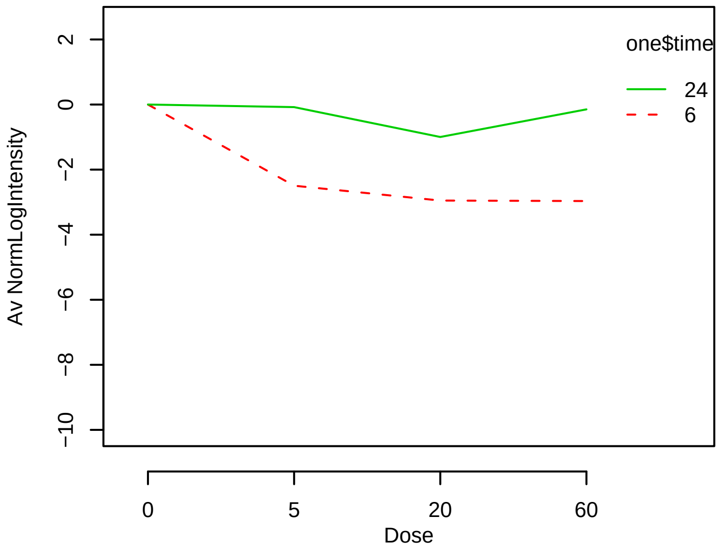

GO_0045665 : negative regulation of neuron differentiatio

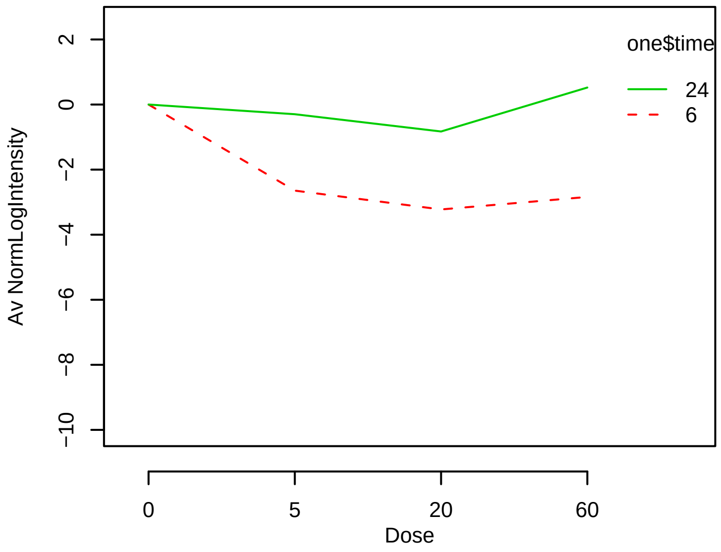

GO_0045667 : regulation of osteoblast differentiation

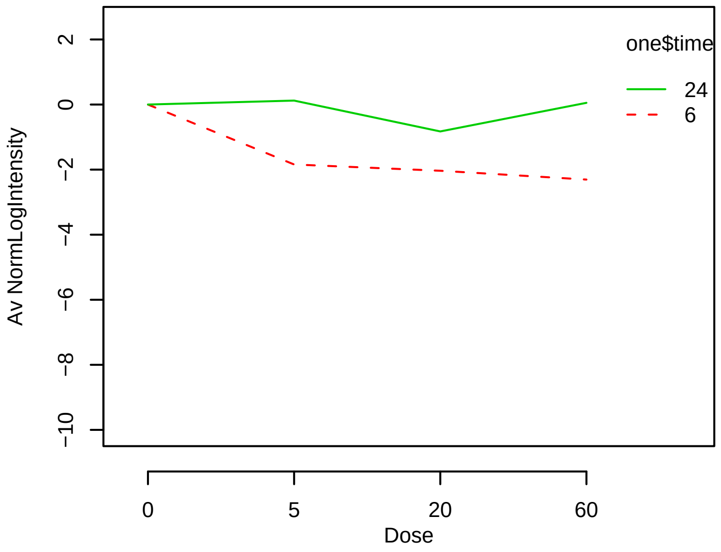

GO_0045670 : regulation of osteoclast differentiation

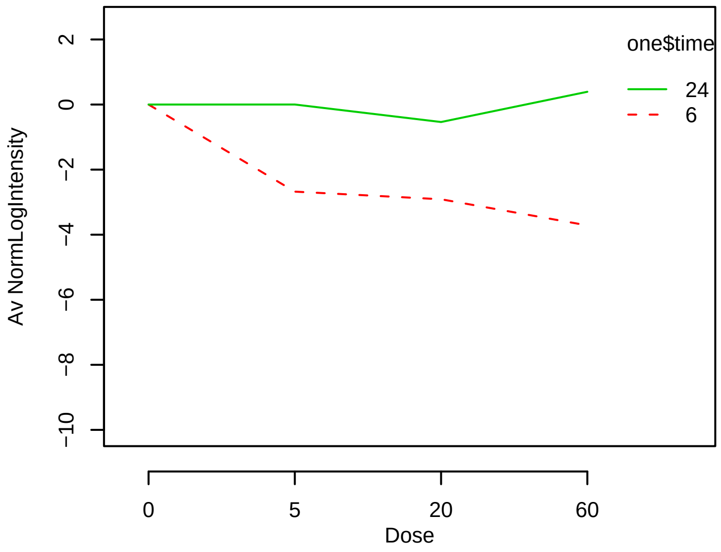

GO_0045682 : regulation of epidermis development

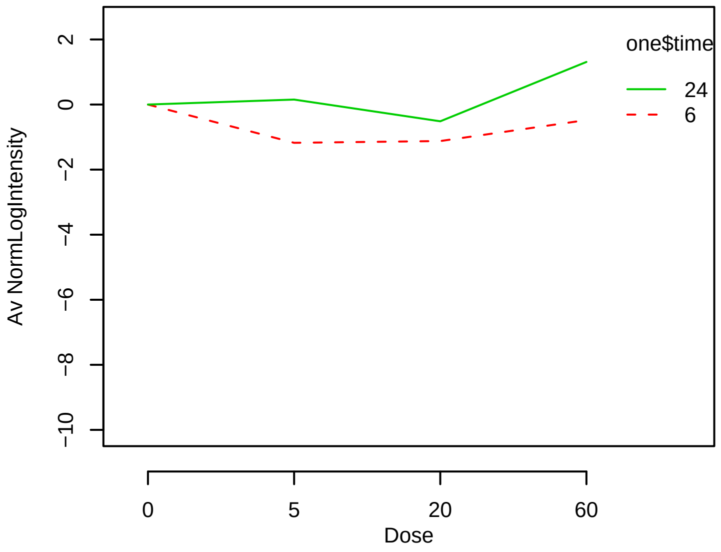

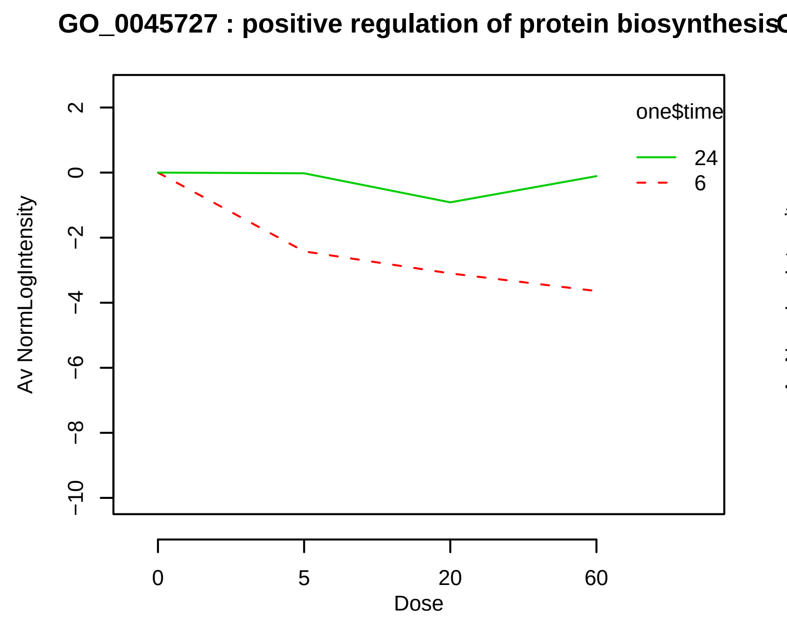
GO_0045727 : positive regulation of protein biosynthesis
one$time
24
6
Av NormLogIntensity
Dose
0
5
20
60
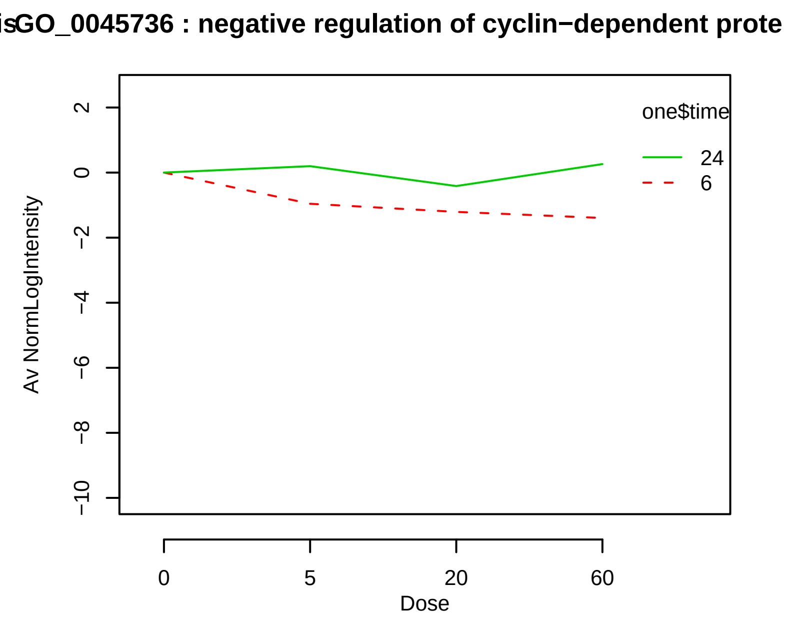
GO_0045736 : negative regulation of cyclin−dependent prote
one$time
24
6
Av NormLogIntensity
Dose
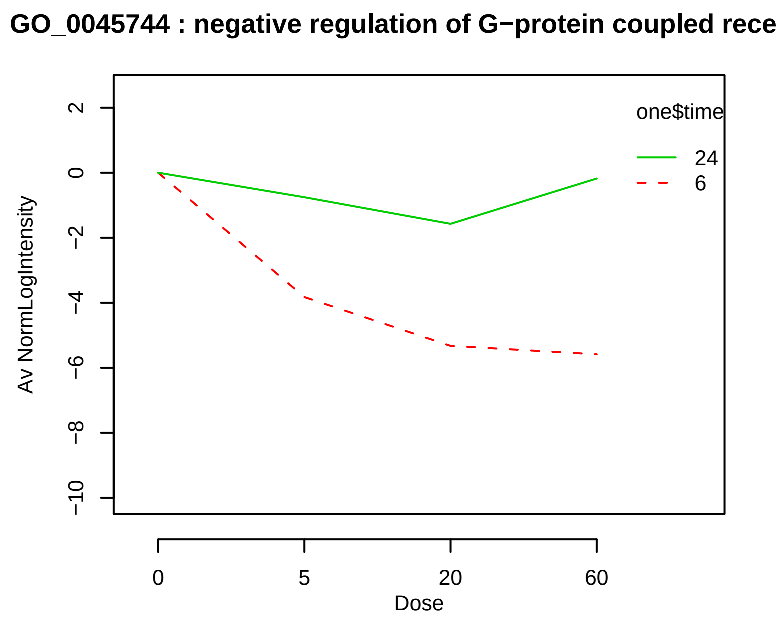
GO_0045744 : negative regulation of G−protein coupled rece
one$time
24
6
Av NormLogIntensity
Dose
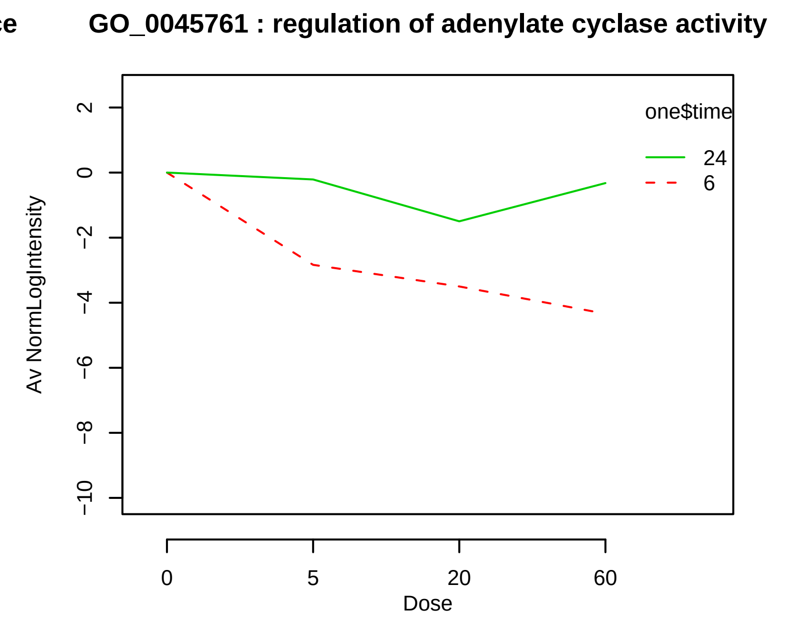
GO_0045761 : regulation of adenylate cyclase activity
one$time
24
6
Av NormLogIntensity
Dose
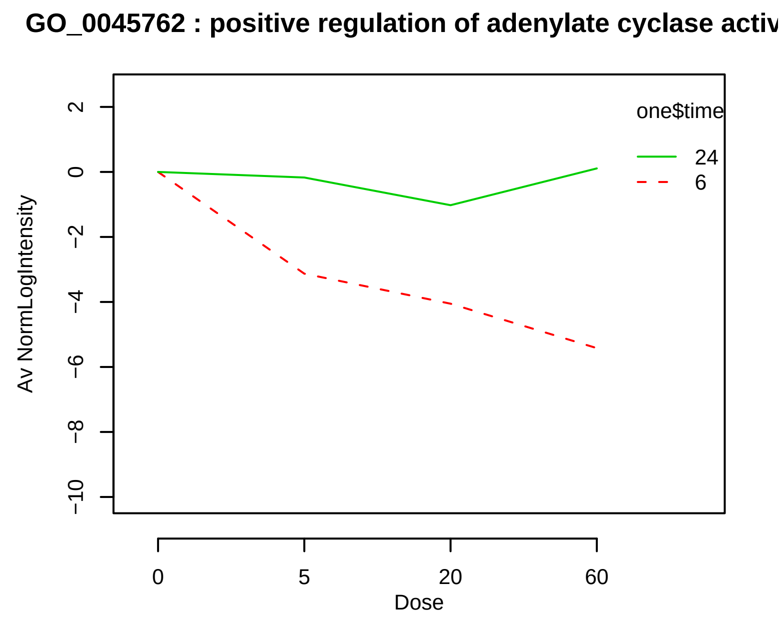
GO_0045762 : positive regulation of adenylate cyclase activ
one$time
24
6
Av NormLogIntensity
Dose
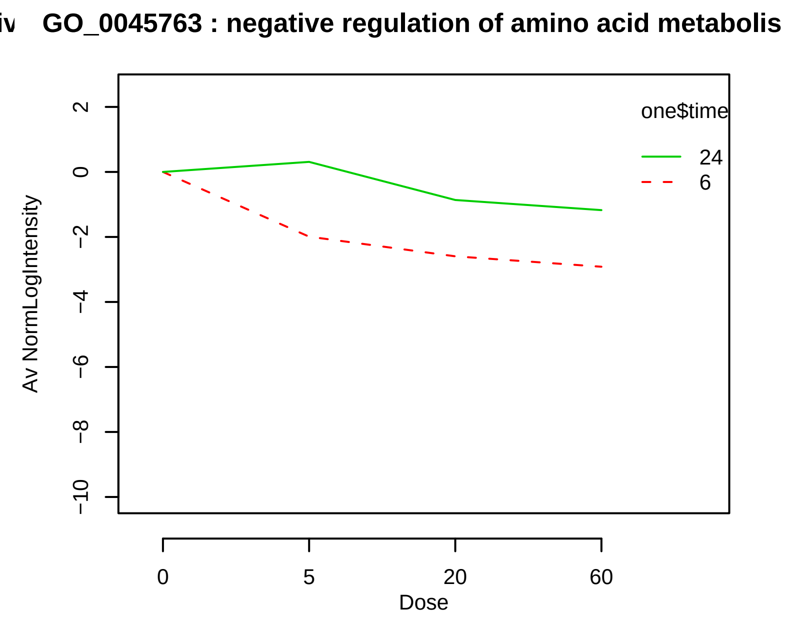
GO_0045763 : negative regulation of amino acid metabolis
one$time
24
6
Av NormLogIntensity
Dose

GO_0045764 : positive regulation of amino acid metabolisı

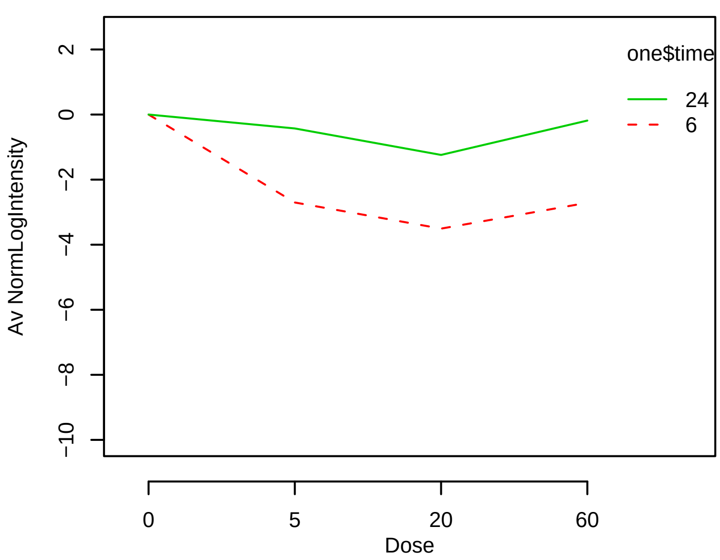

GO_0045765 : regulation of angiogenesis

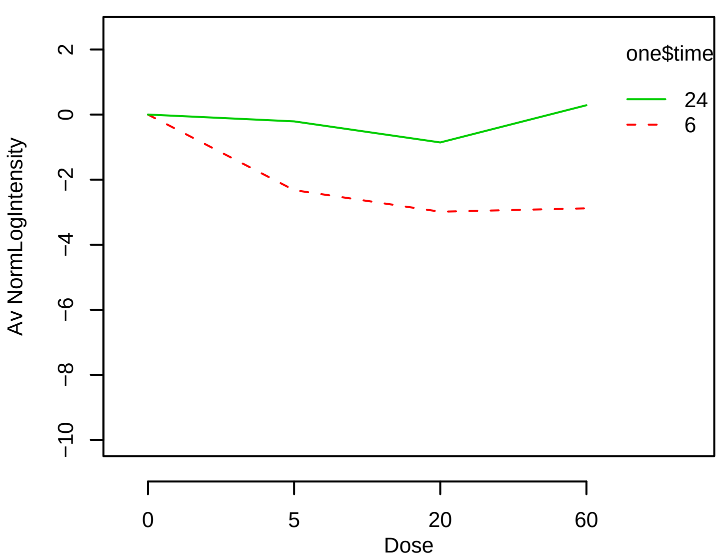

GO_0045766 : positive regulation of angiogenesis

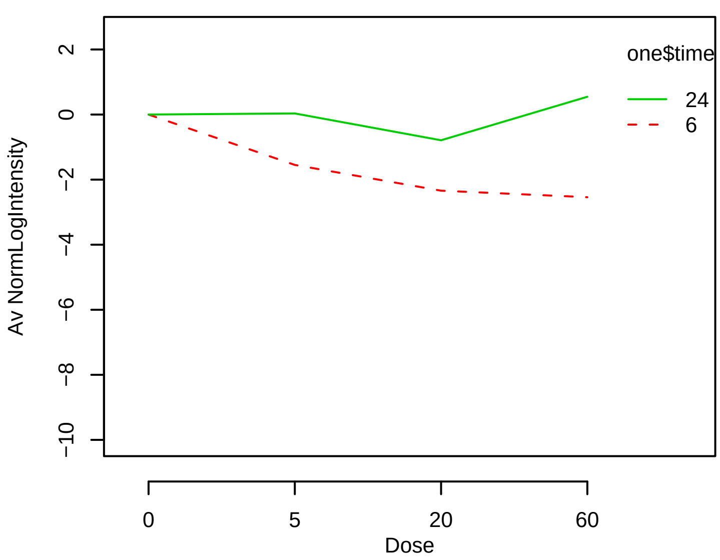

GO_0045767 : regulation of anti−apoptosis

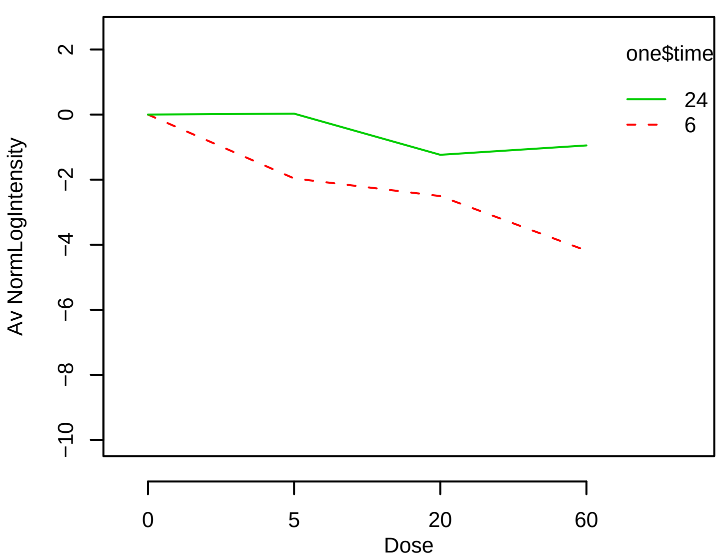

GO_0045768 : positive regulation of anti−apoptosis

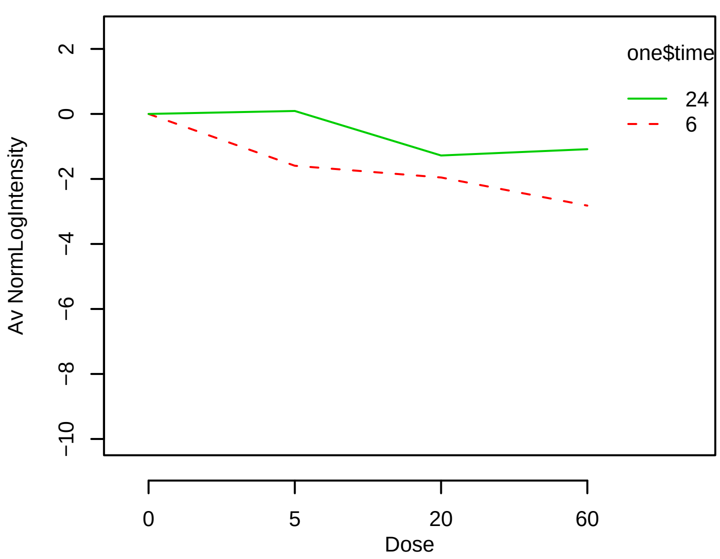

GO_0045773 : positive regulation of axon extension

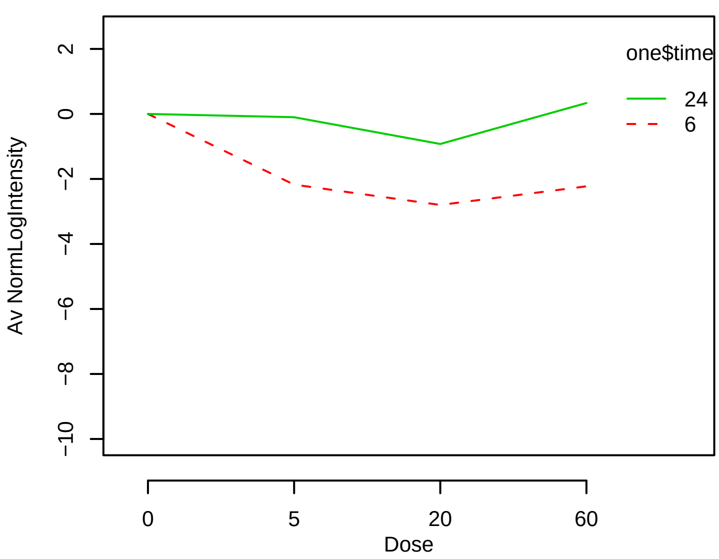

GO_0045778 : positive regulation of ossification

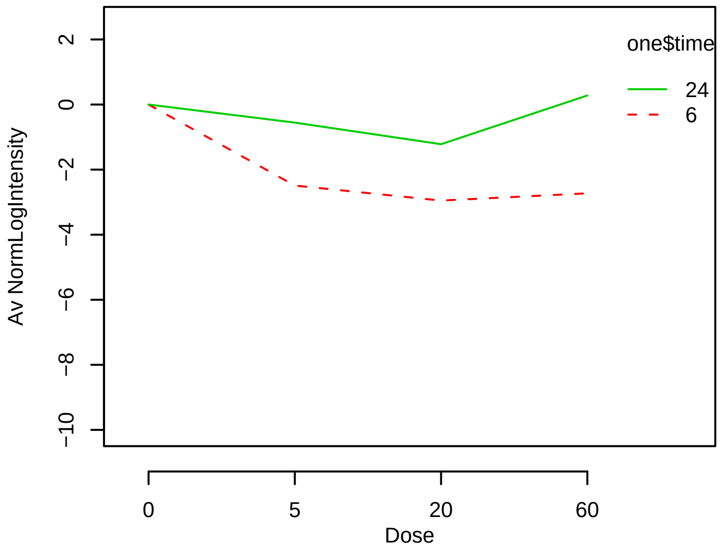

GO_0045785 : positive regulation of cell adhesion

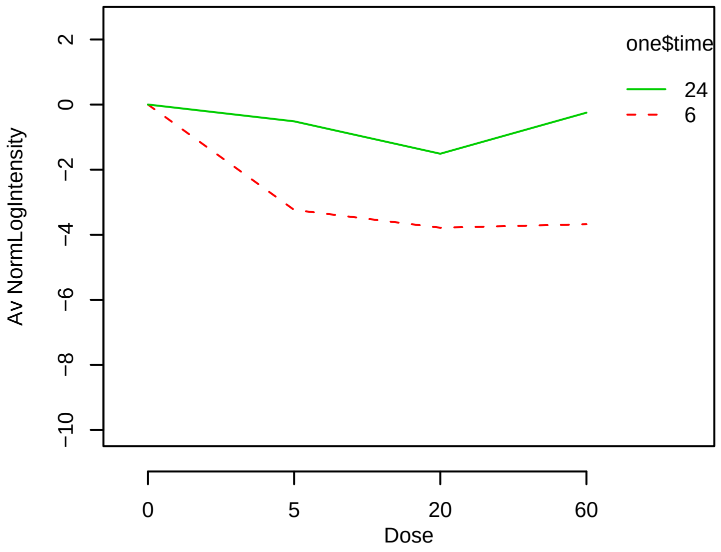

GO_0045786 : negative regulation of progression through ce

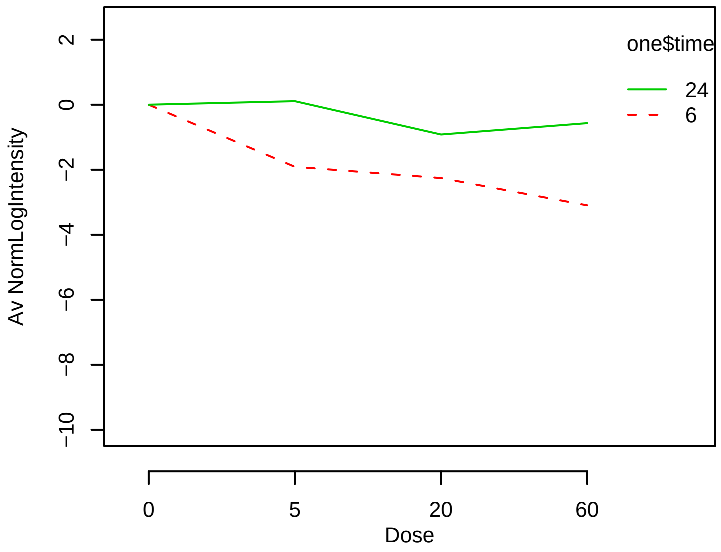

GO_0045787 : positive regulation of progression through cel

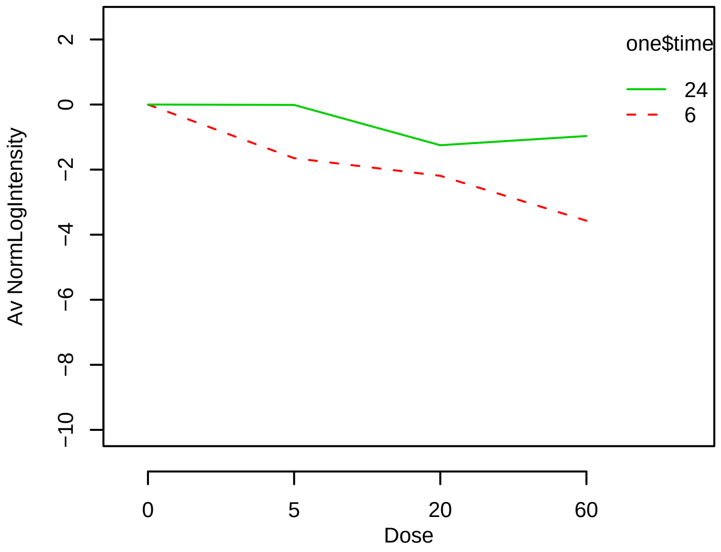

GO_0045792 : negative regulation of cell size

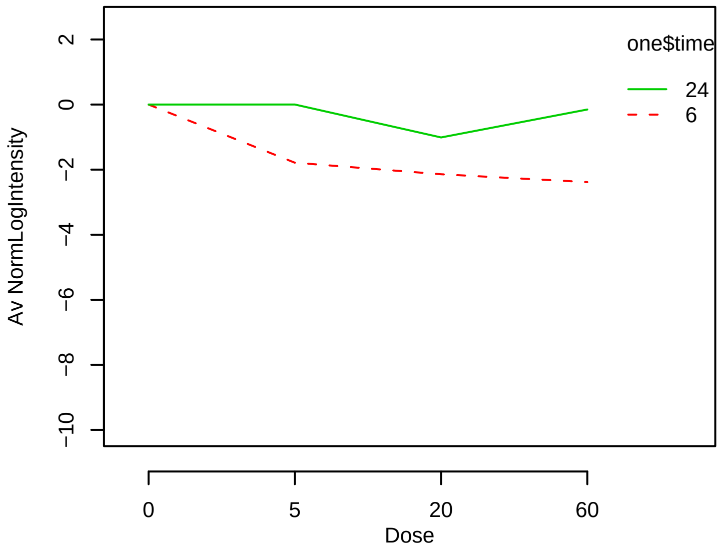

GO_0045793 : positive regulation of cell size

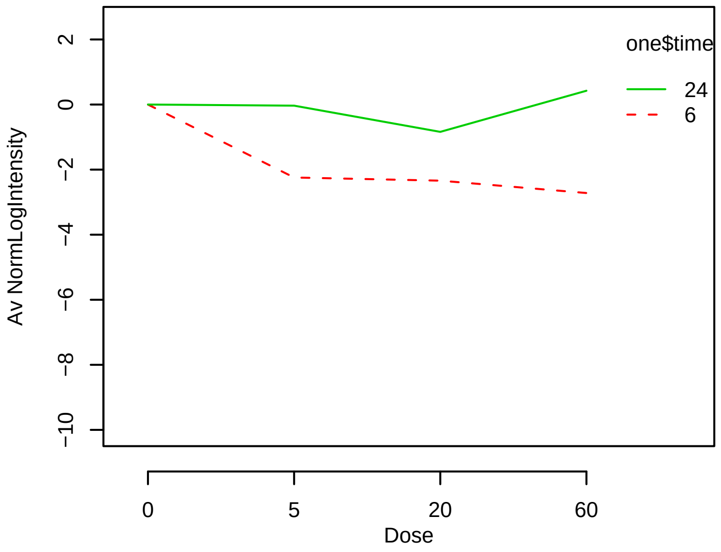

GO_0045806 : negative regulation of endocytosis

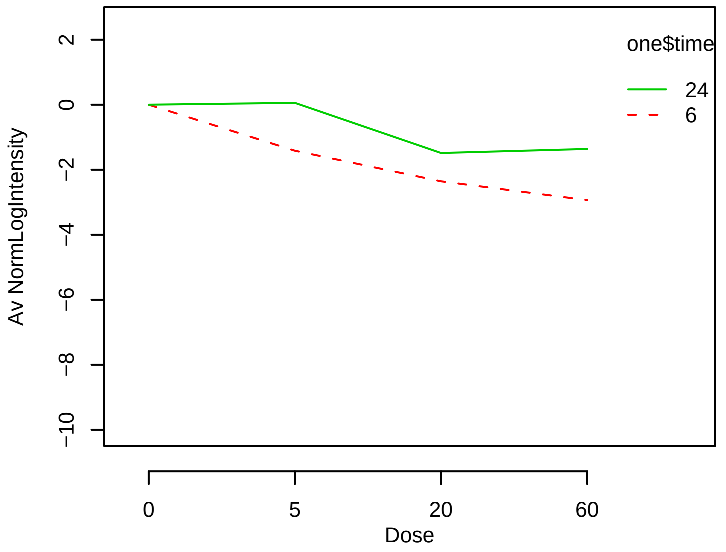

GO_0045807 : positive regulation of endocytosis

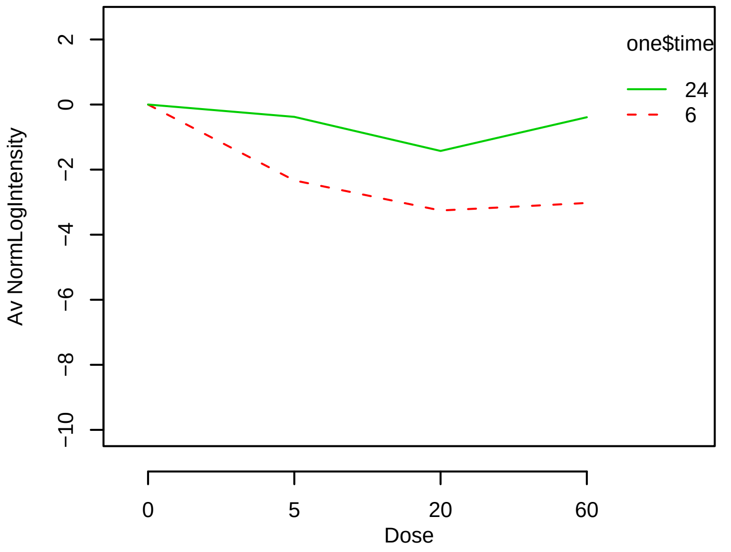

GO_0045814 : negative regulation of gene expression\, epige

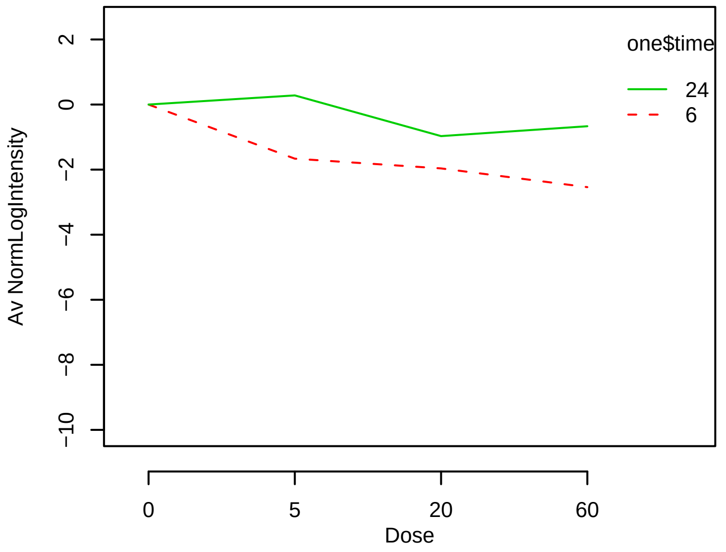

GO_0045815 : positive regulation of gene expression\, epige

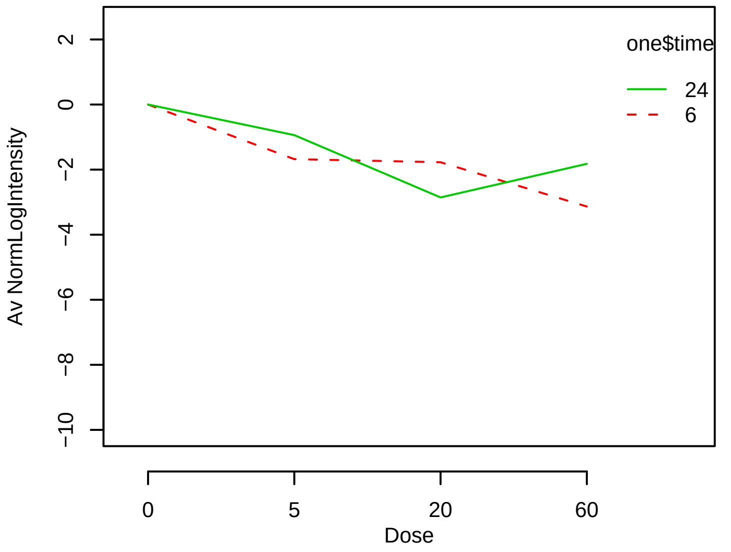

GO_0045823 : positive regulation of heart contraction

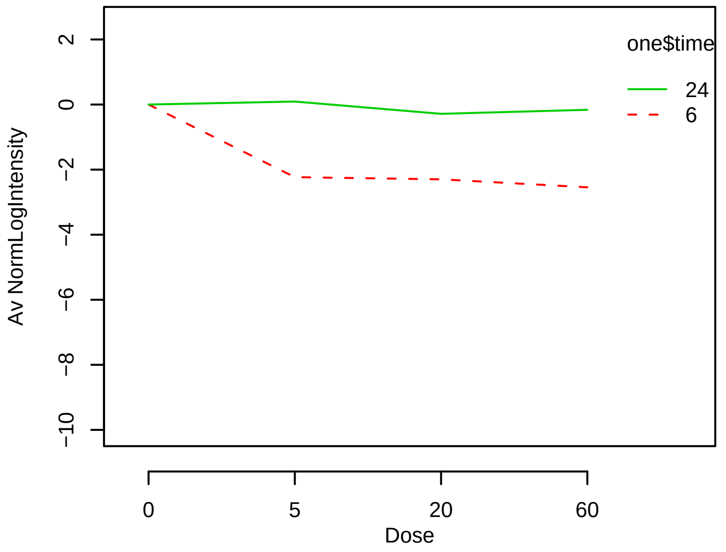

GO_0045830 : positive regulation of isotype switching

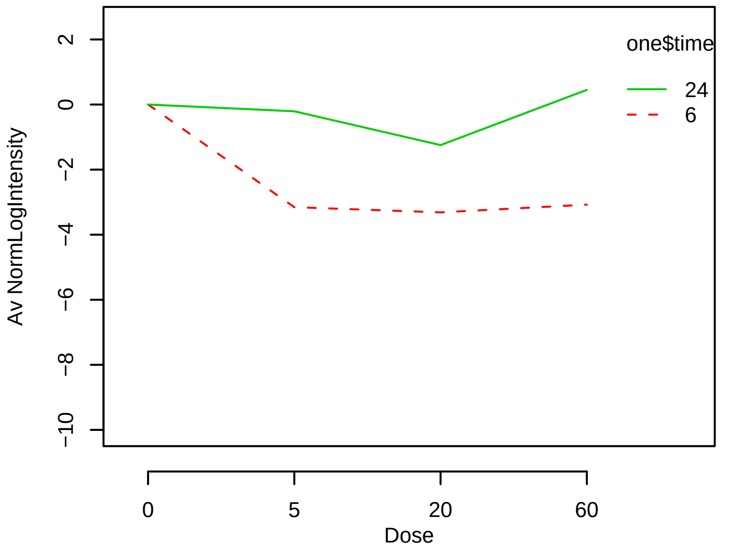

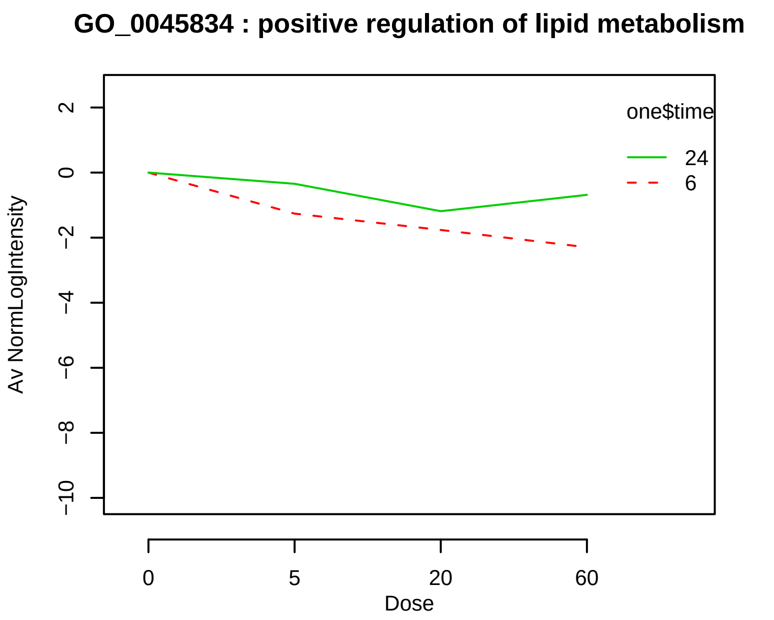
GO_0045834 : positive regulation of lipid metabolism
one$time
24
6
Av NormLogIntensity
2
0
−2
−4
−6
−8
−10
0
5
20
60
Dose

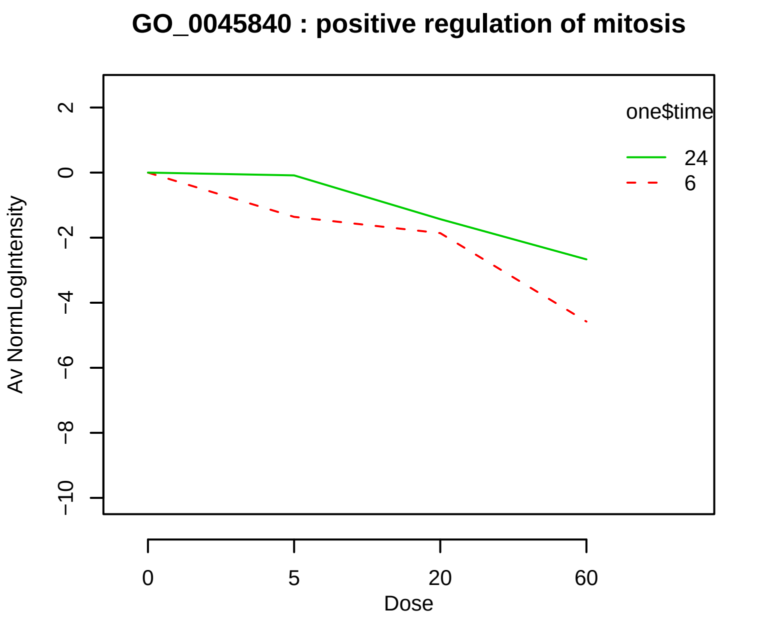
GO_0045840 : positive regulation of mitosis
one$time
24
6
Av NormLogIntensity
2
0
−2
−4
−6
−8
−10
0
5
20
60
Dose

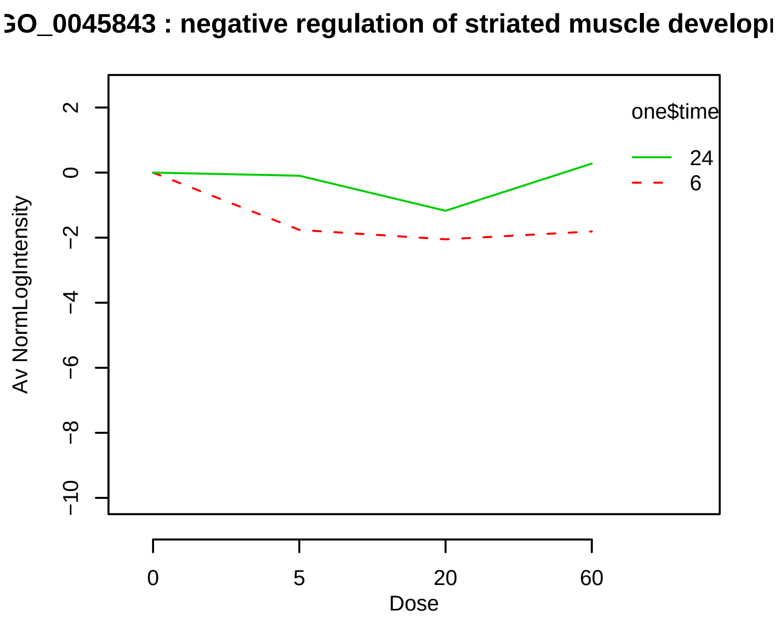
GO_0045843 : negative regulation of striated muscle develop
one$time
24
6
Av NormLogIntensity
2
0
−2
−4
−6
−8
−10
0
5
20
60
Dose

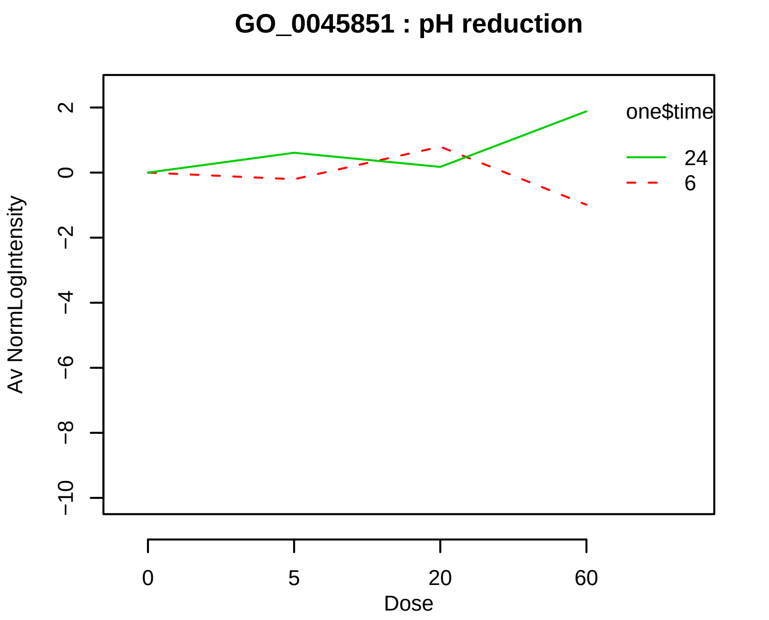
GO_0045851 : pH reduction
one$time
24
6
Av NormLogIntensity
2
0
−2
−4
−6
−8
−10
0
5
20
60
Dose

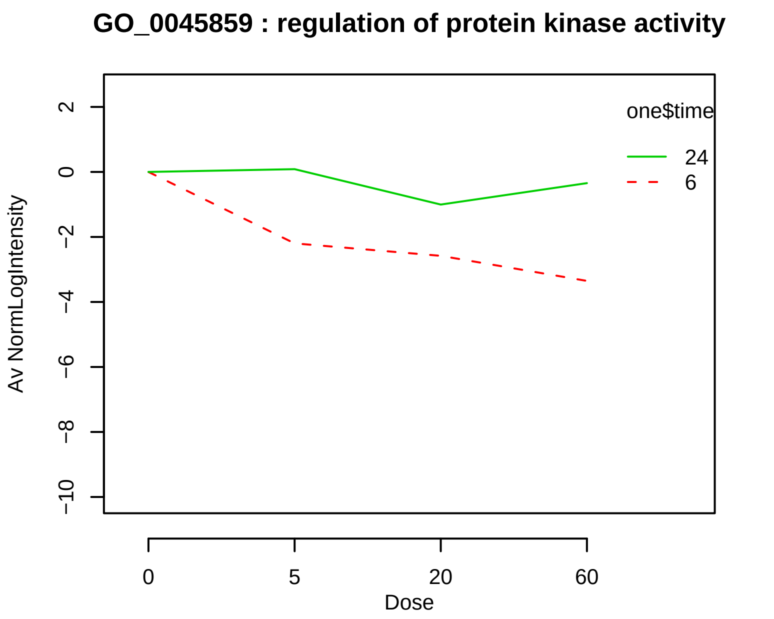
GO_0045859 : regulation of protein kinase activity
one$time
24
6
Av NormLogIntensity
2
0
−2
−4
−6
−8
−10
0
5
20
60
Dose

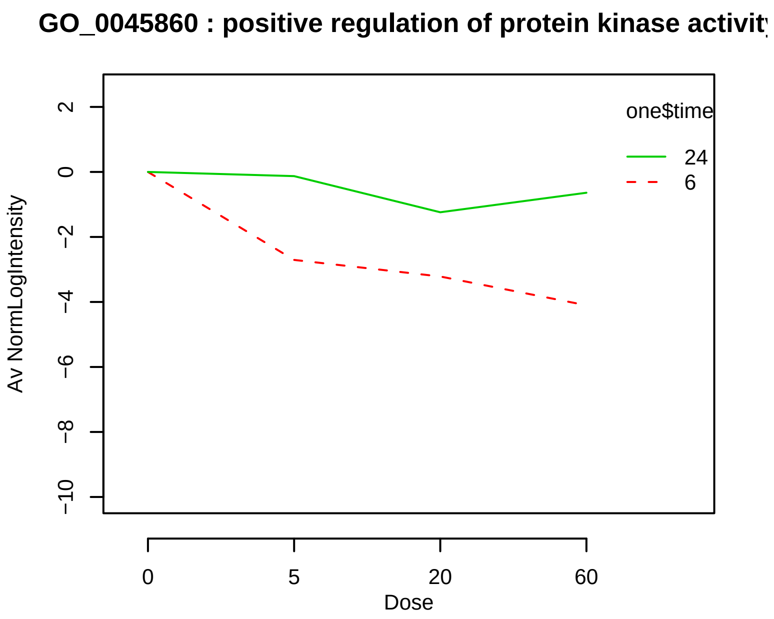
GO_0045860 : positive regulation of protein kinase activit
one$time
24
6
Av NormLogIntensity
2
0
−2
−4
−6
−8
−10
0
5
20
60
Dose

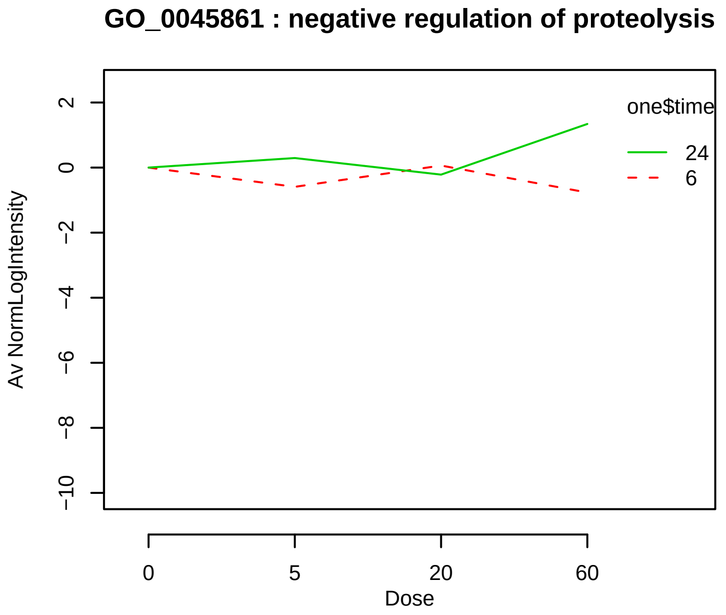
GO_0045861 : negative regulation of proteolysis
Av NormLogIntensity
2
0
−2
−4
−6
−8
−10
one$time
24
6
0
5
20
60
Dose

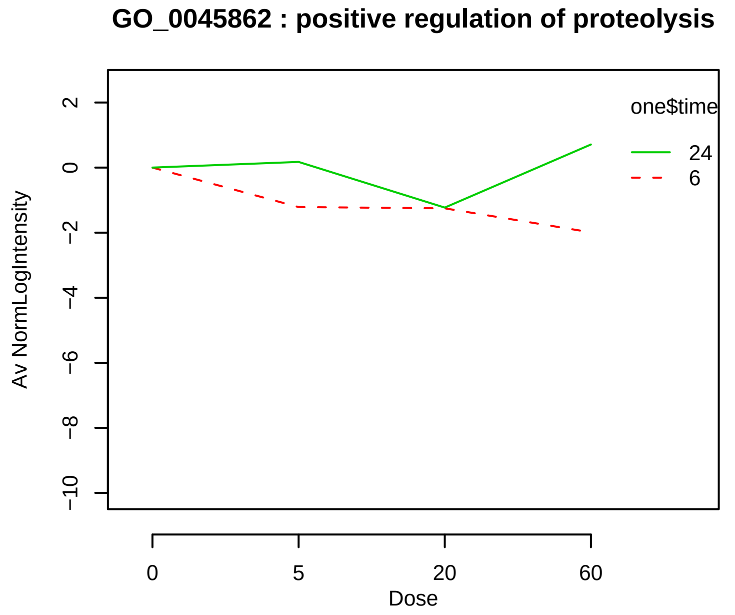
GO_0045862 : positive regulation of proteolysis
Av NormLogIntensity
2
0
−2
−4
−6
−8
−10
one$time
24
6
0
5
20
60
Dose

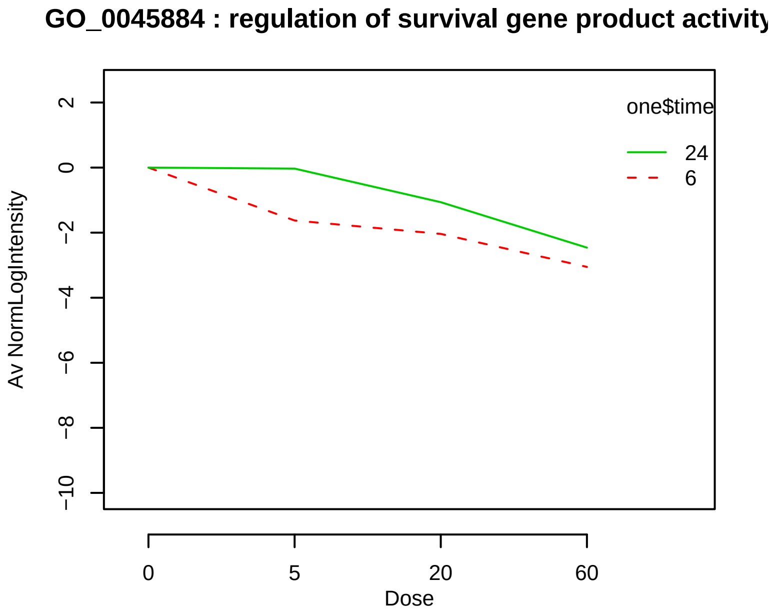
GO_0045884 : regulation of survival gene product activity
Av NormLogIntensity
2
0
−2
−4
−6
−8
−10
one$time
24
6
0
5
20
60
Dose

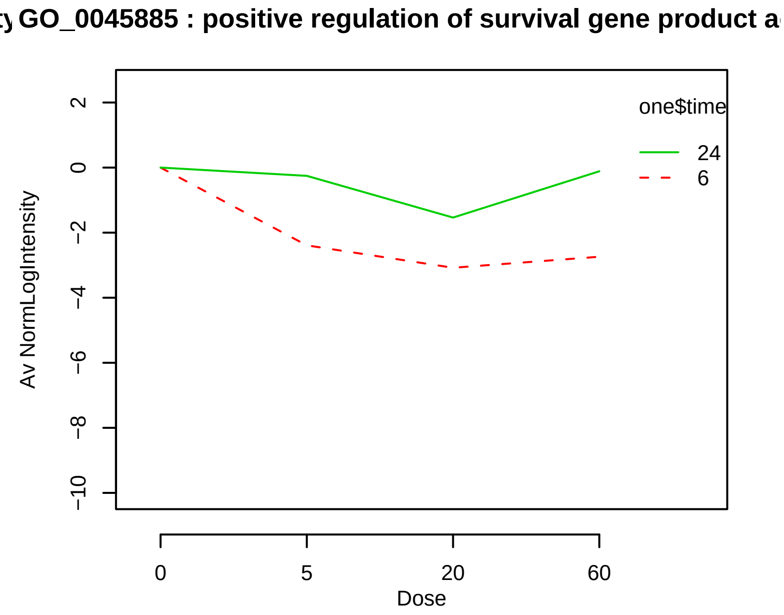
GO_0045885 : positive regulation of survival gene product a
Av NormLogIntensity
2
0
−2
−4
−6
−8
−10
one$time
24
6
0
5
20
60
Dose

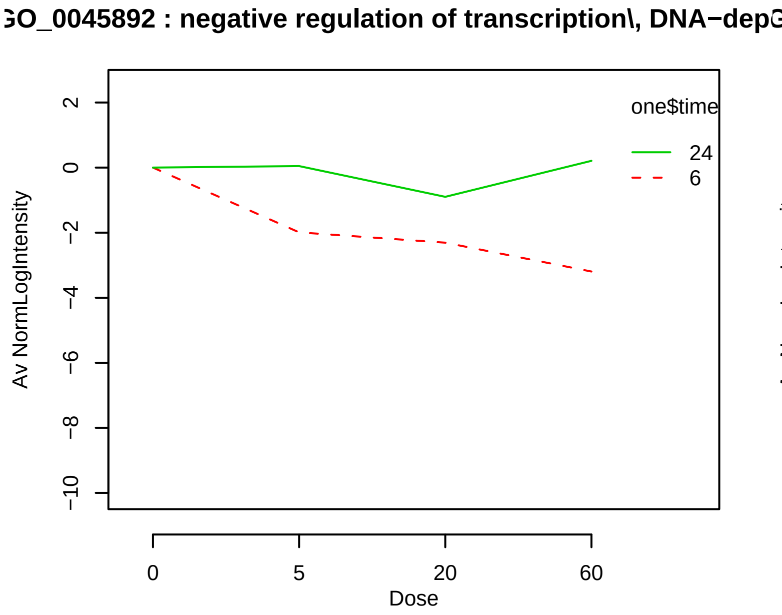
GO_0045892 : negative regulation of transcription\, DNA−dep
Av NormLogIntensity
2
0
−2
−4
−6
−8
−10
one$time
24
6
0
5
20
60
Dose

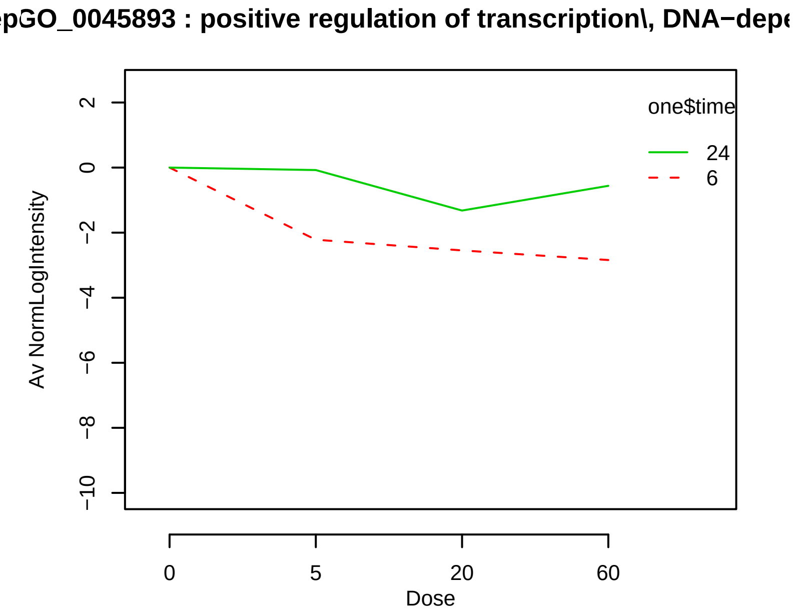
GO_0045893 : positive regulation of transcription\, DNA−depe
Av NormLogIntensity
2
0
−2
−4
−6
−8
−10
one$time
24
6
0
5
20
60
Dose

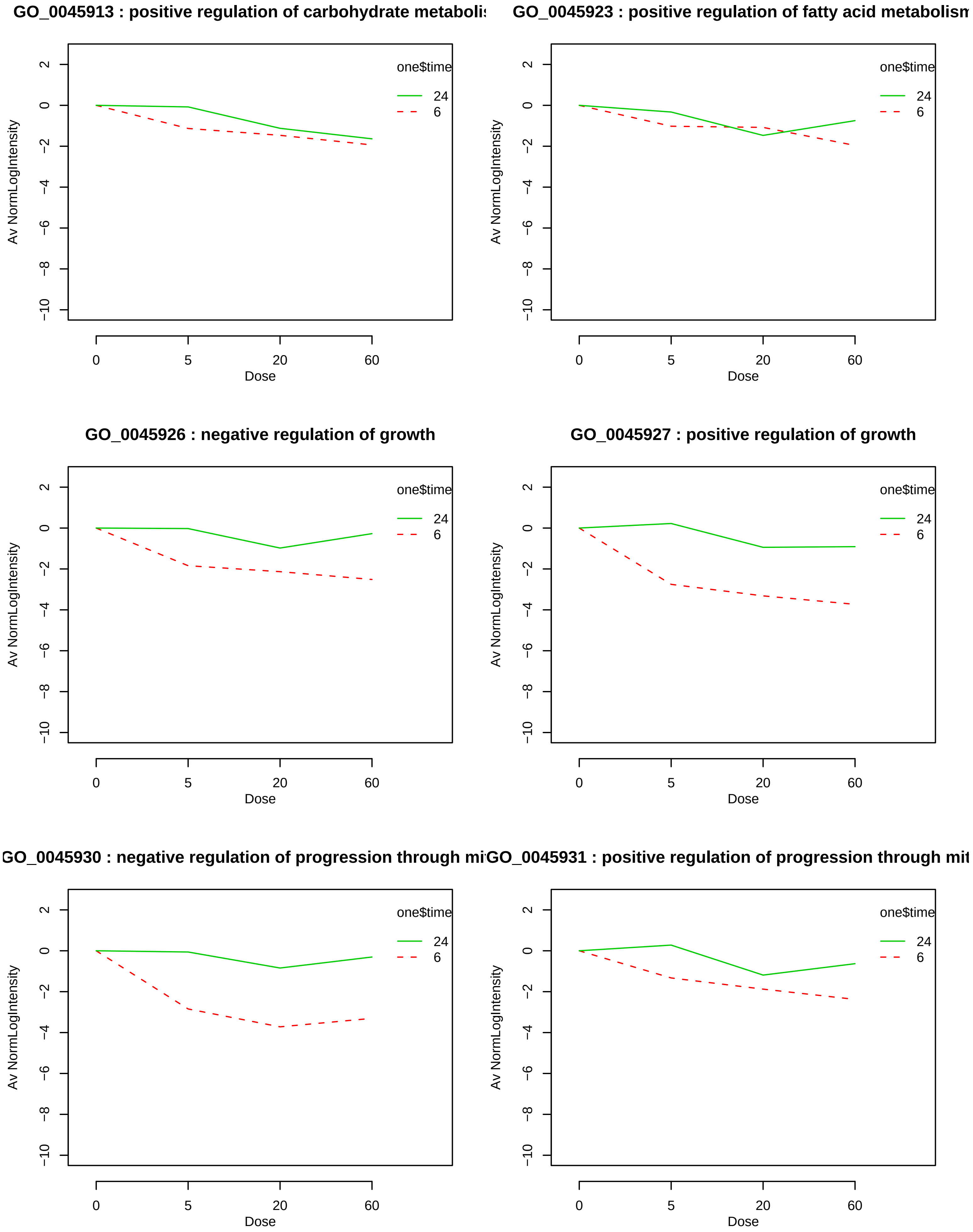
GO_0045913 : positive regulation of carbohydrate metaboli
GO_0045923 : positive regulation of fatty acid metabolism
GO_0045926 : negative regulation of growth
GO_0045927 : positive regulation of growth
GO_0045930 : negative regulation of progression through mi
GO_0045931 : positive regulation of progression through mit
Av NormLogIntensity
Dose
one$time
24
6
0
5
20
60
2
0
−2
−4
−6
−8
−10

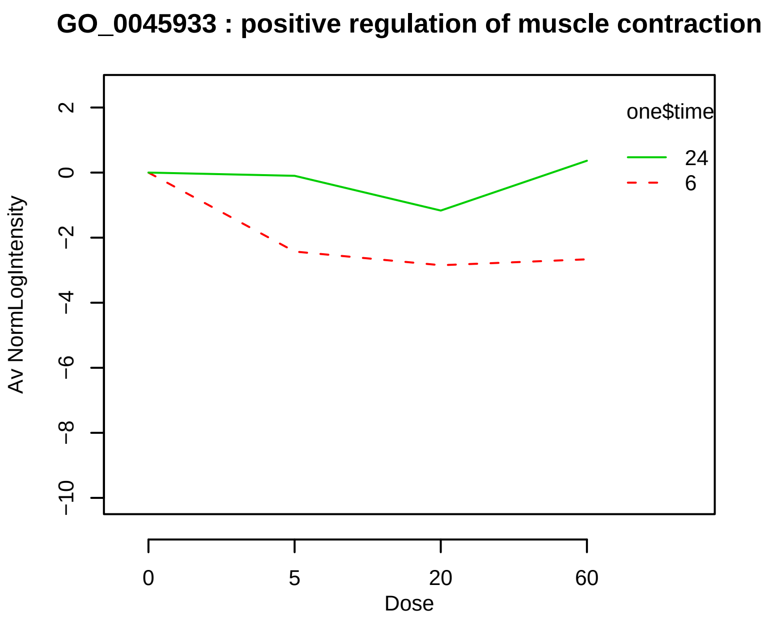
GO_0045933 : positive regulation of muscle contraction
one$time
24
6
Av NormLogIntensity
2
0
−2
−4
−6
−8
−10
0
5
20
60
Dose

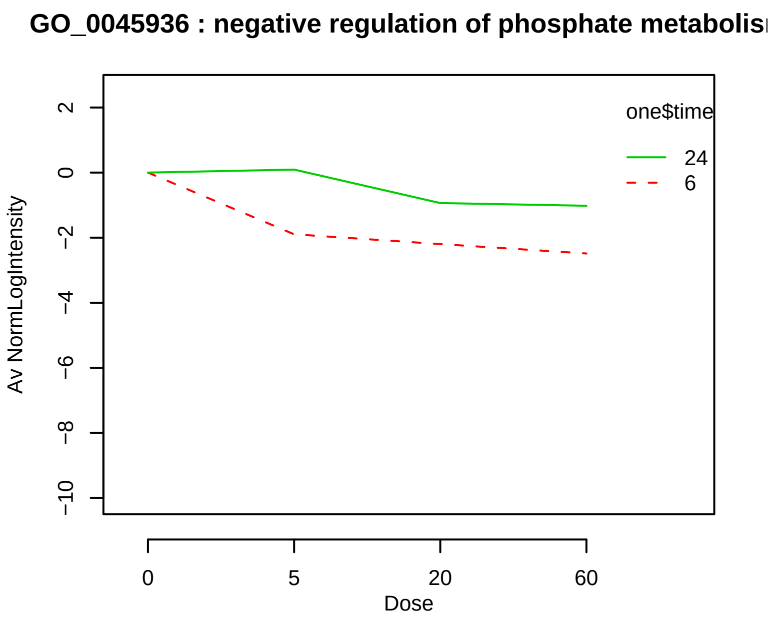
GO_0045936 : negative regulation of phosphate metabolis
one$time
24
6
Av NormLogIntensity
2
0
−2
−4
−6
−8
−10
0
5
20
60
Dose

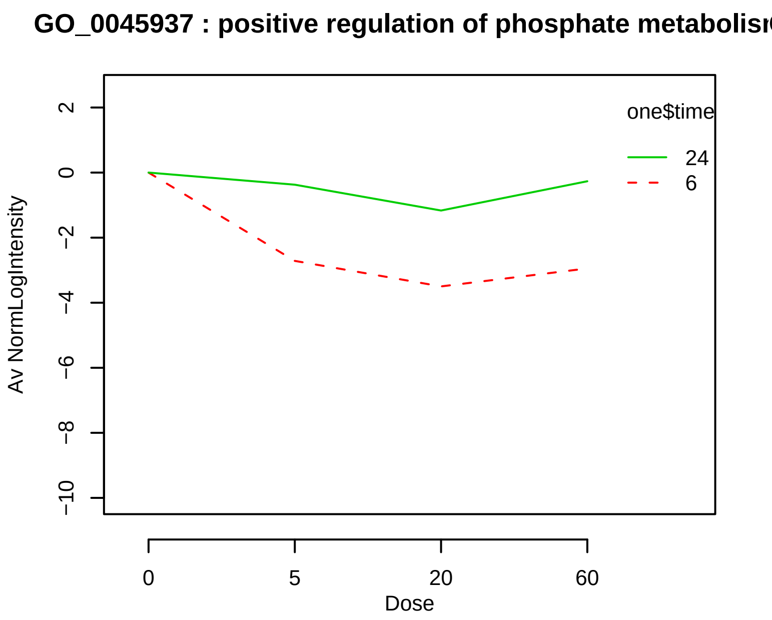
GO_0045937 : positive regulation of phosphate metabolis
one$time
24
6
Av NormLogIntensity
2
0
−2
−4
−6
−8
−10
0
5
20
60
Dose

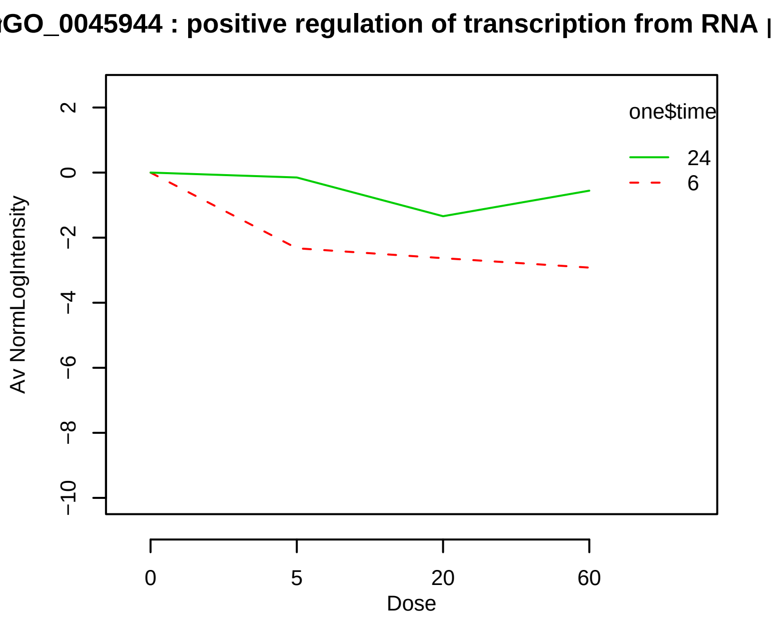
GO_0045944 : positive regulation of transcription from RNA
one$time
24
6
Av NormLogIntensity
2
0
−2
−4
−6
−8
−10
0
5
20
60
Dose

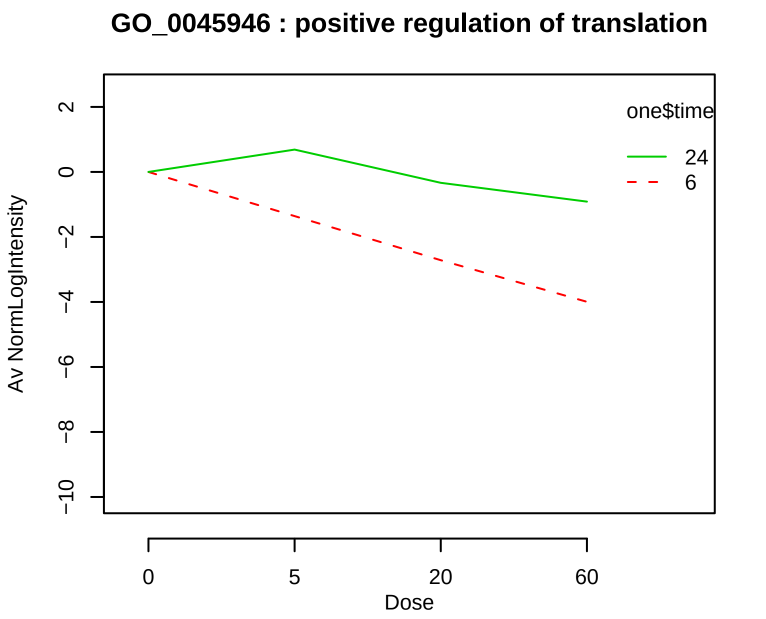
GO_0045946 : positive regulation of translation
one$time
24
6
Av NormLogIntensity
2
0
−2
−4
−6
−8
−10
0
5
20
60
Dose

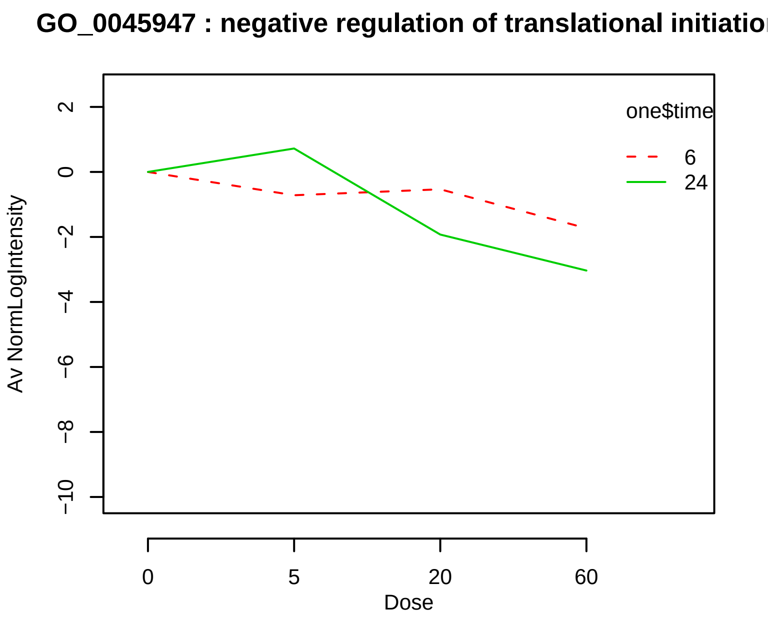
GO_0045947 : negative regulation of translational initiatio
one$time
6
24
Av NormLogIntensity
2
0
−2
−4
−6
−8
−10
0
5
20
60
Dose

GO_0045980 : negative regulation of nucleotide metabolis

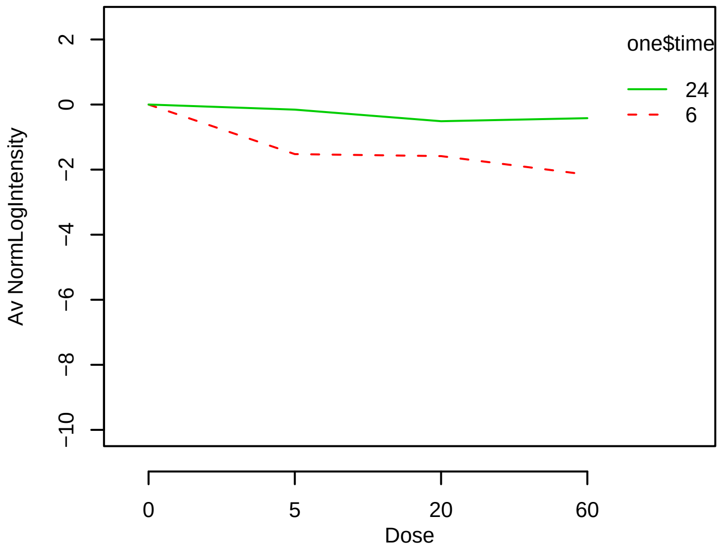

GO_0045987 : positive regulation of smooth muscle contract

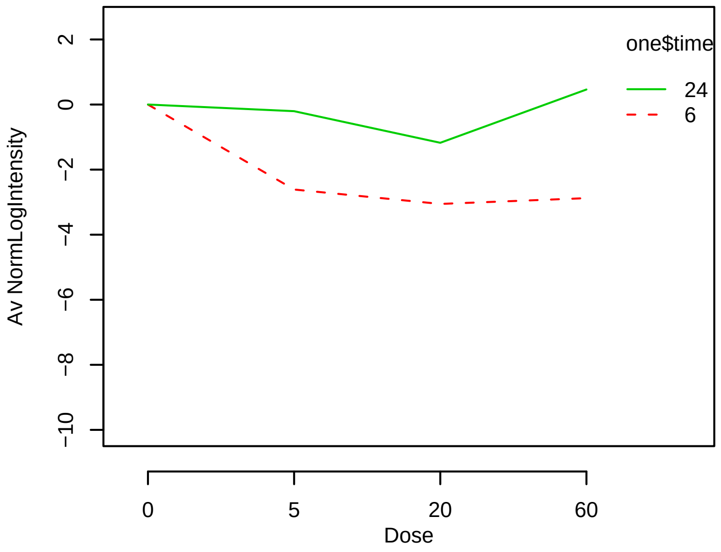

GO_0045995 : regulation of embryonic development

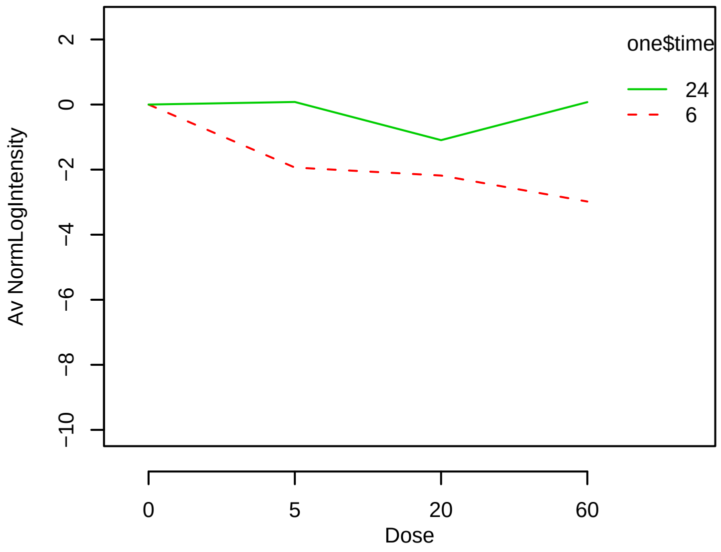

GO_0046006 : regulation of activated T cell proliferation

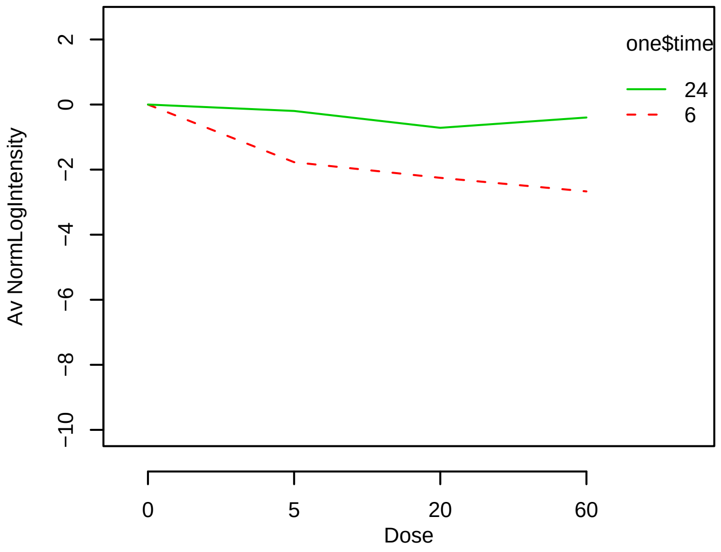

GO_0046034 : ATP metabolism

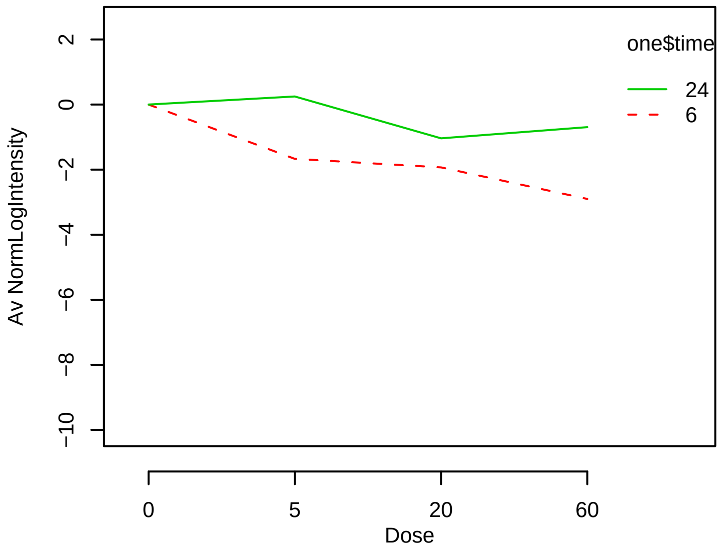

GO_0046036 : CTP metabolism

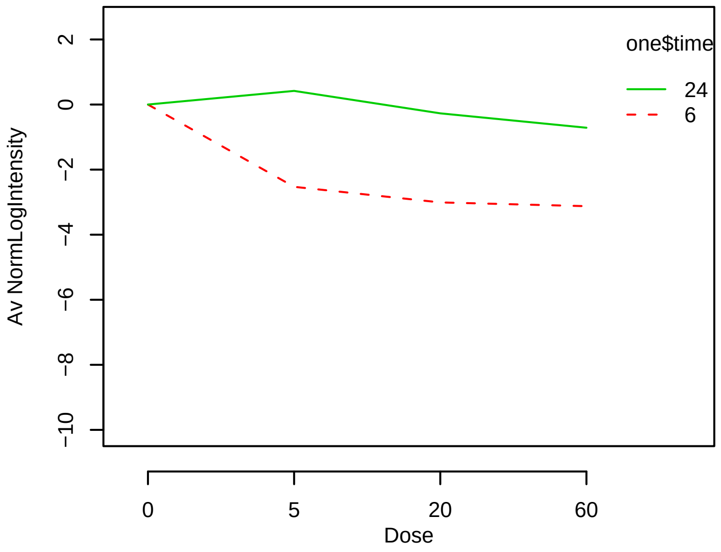

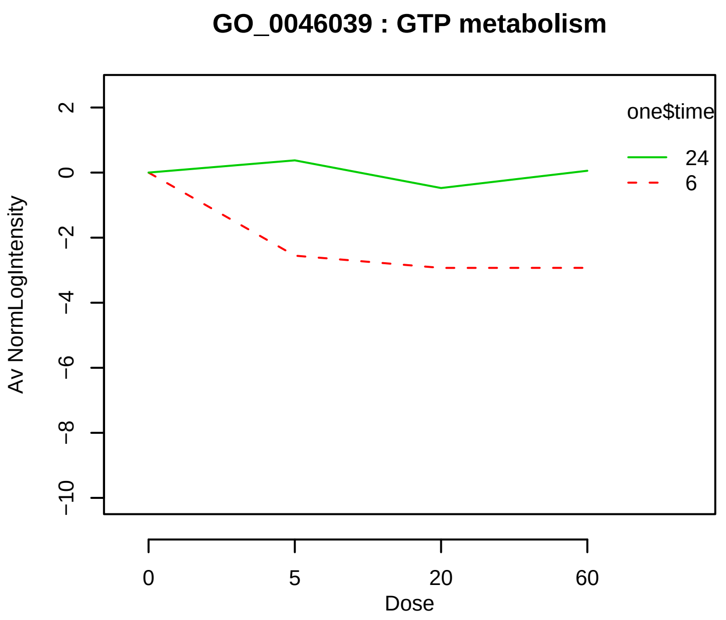
GO_0046039 : GTP metabolism
Av NormLogIntensity
2
0
−2
−4
−6
−8
−10
one$time
24
6
0
5
20
60
Dose

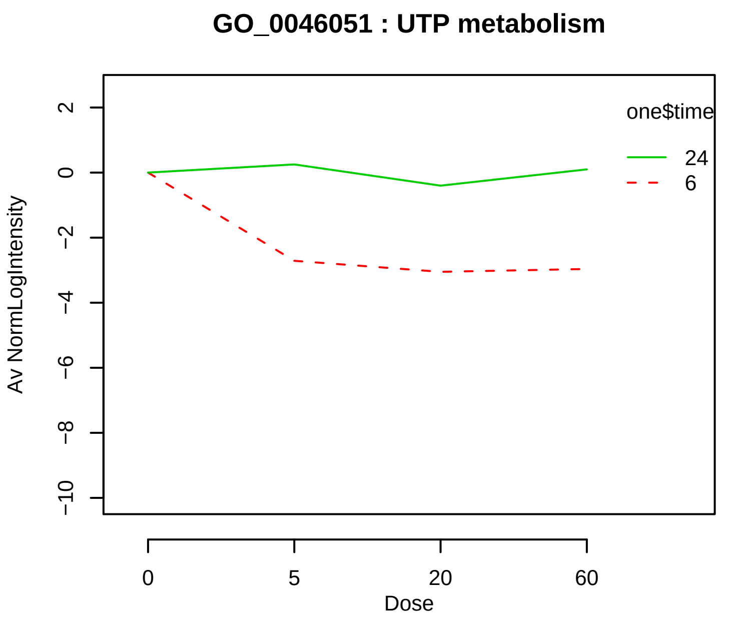
GO_0046051 : UTP metabolism
Av NormLogIntensity
2
0
−2
−4
−6
−8
−10
one$time
24
6
0
5
20
60
Dose

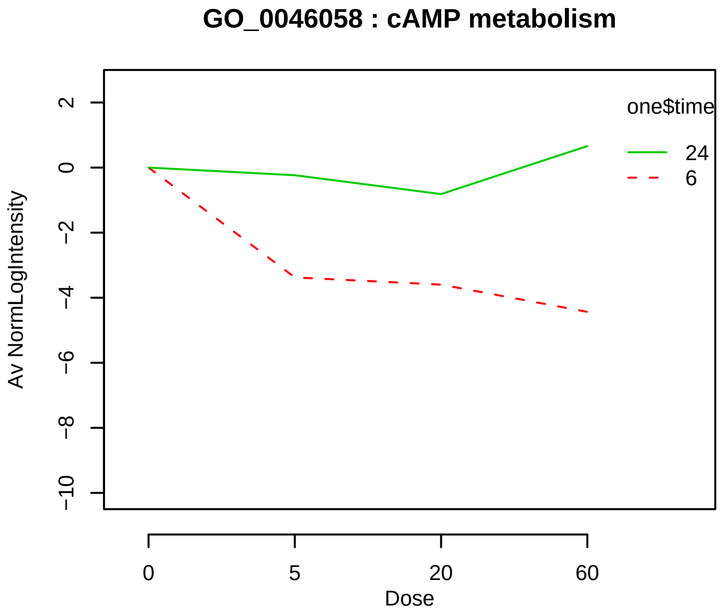
GO_0046058 : cAMP metabolism
Av NormLogIntensity
2
0
−2
−4
−6
−8
−10
one$time
24
6
0
5
20
60
Dose

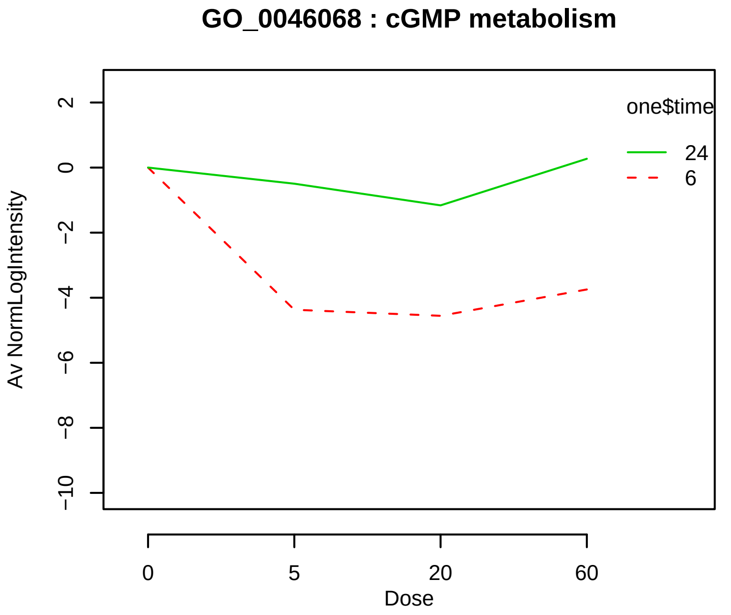
GO_0046068 : cGMP metabolism
Av NormLogIntensity
2
0
−2
−4
−6
−8
−10
one$time
24
6
0
5
20
60
Dose

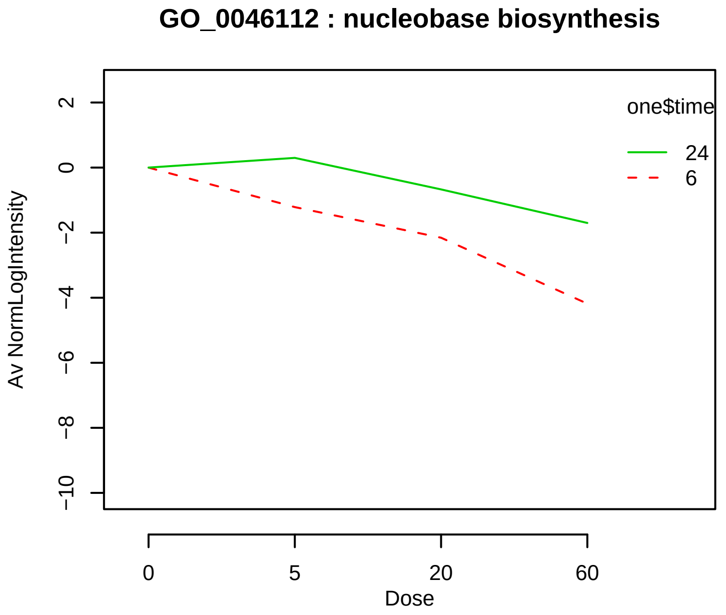
GO_0046112 : nucleobase biosynthesis
Av NormLogIntensity
2
0
−2
−4
−6
−8
−10
one$time
24
6
0
5
20
60
Dose

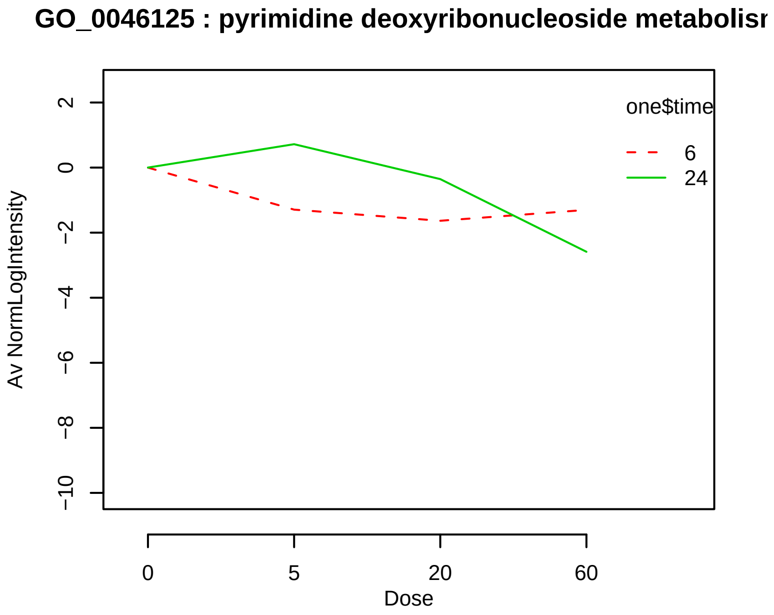
GO_0046125 : pyrimidine deoxyribonucleoside metabolisr
Av NormLogIntensity
2
0
−2
−4
−6
−8
−10
one$time
6
24
0
5
20
60
Dose

GO_0046128 : purine ribonucleoside metabolism

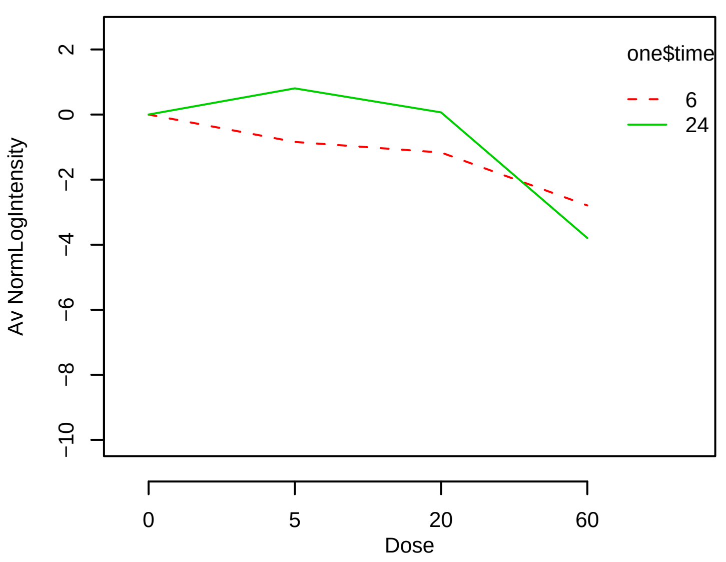

GO_0046146 : tetrahydrobiopterin metabolism

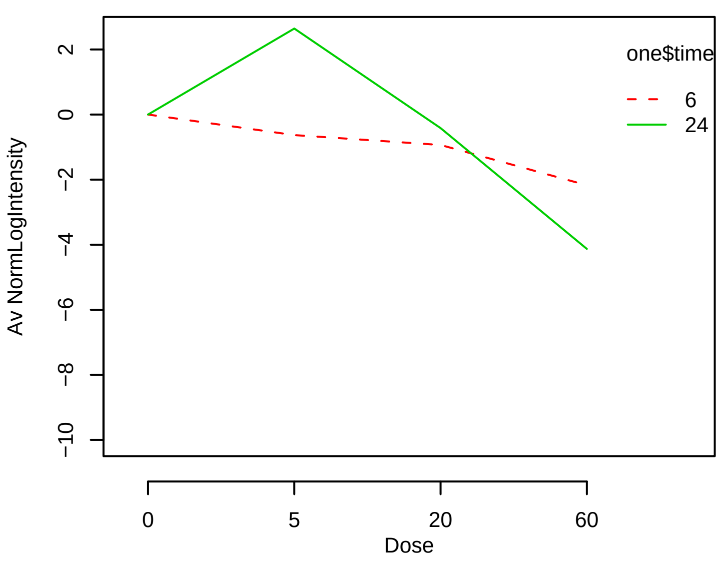

GO_0046148 : pigment biosynthesis

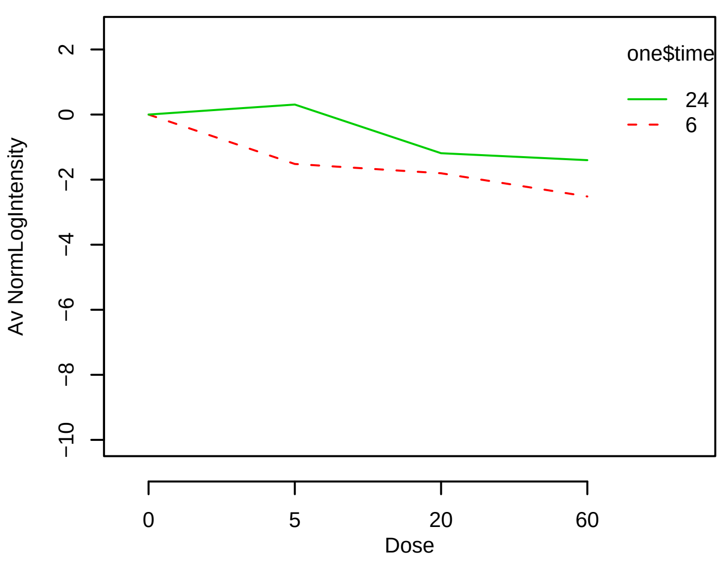

GO_0046164 : alcohol catabolism

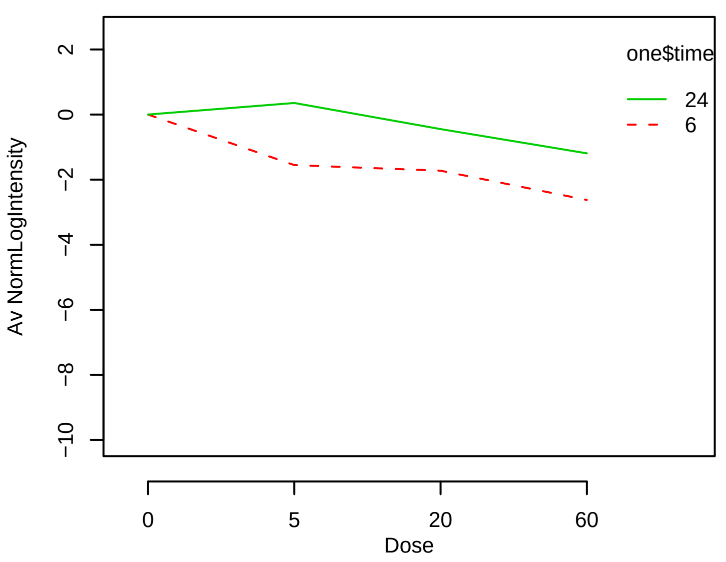

GO_0046165 : alcohol biosynthesis

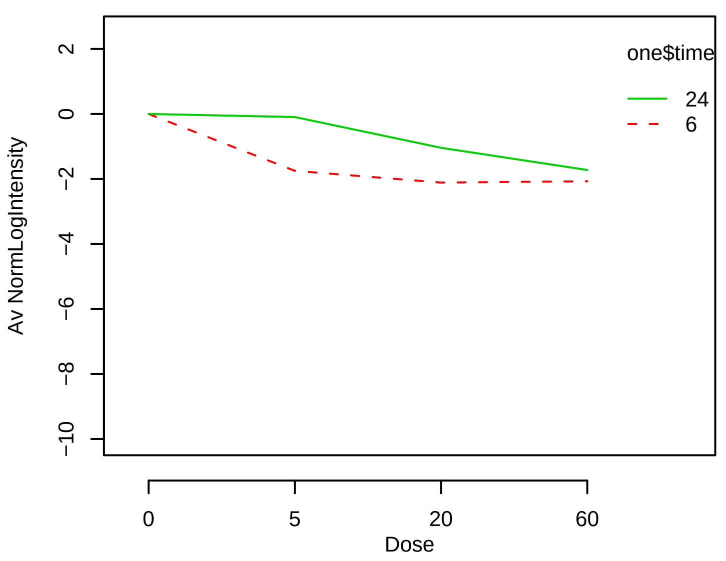

GO_0046209 : nitric oxide metabolism

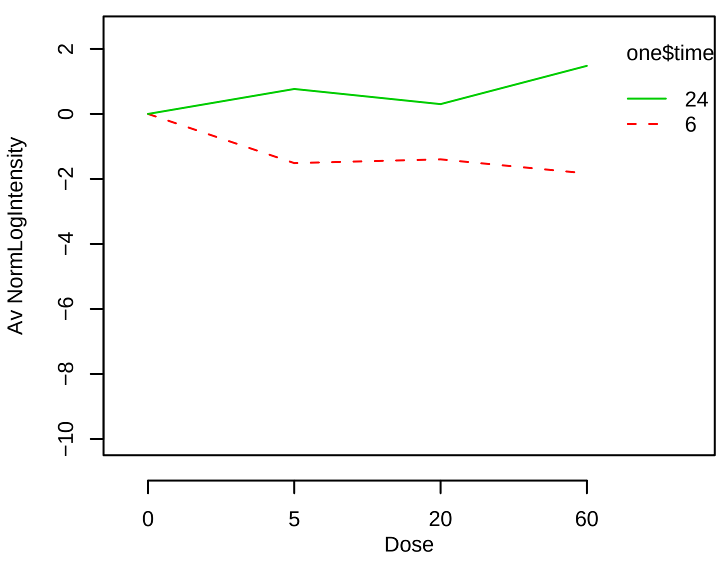

GO_0046323 : glucose import

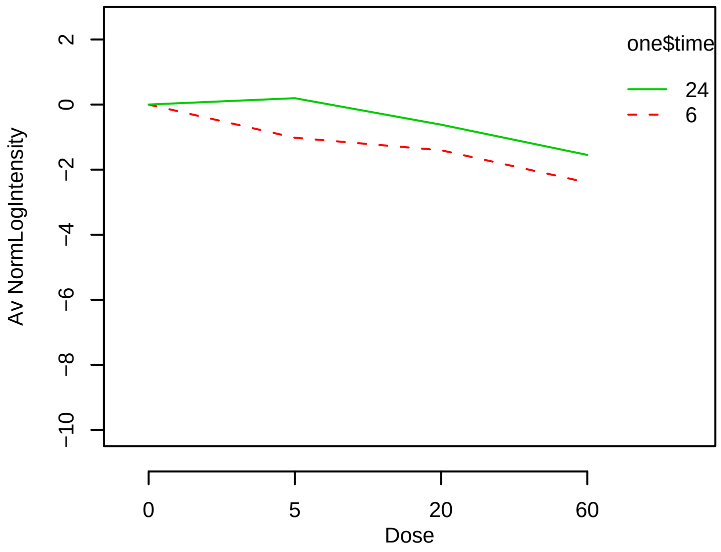

GO_0046324 : regulation of glucose import

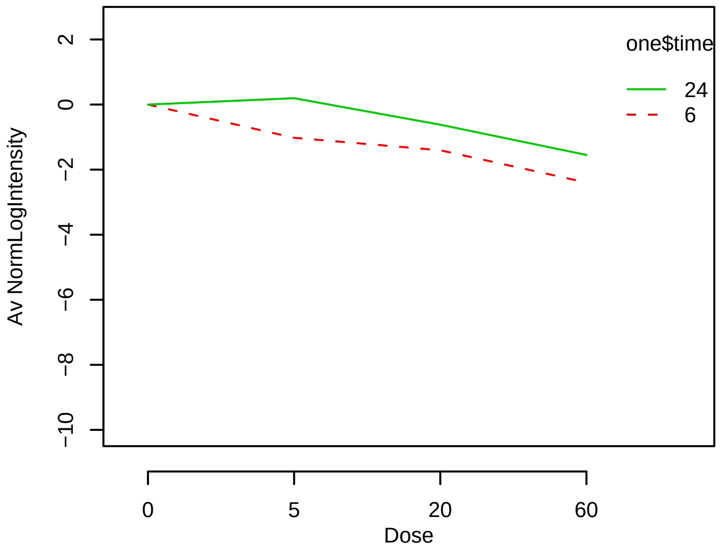

GO_0046328 : regulation of JNK cascade

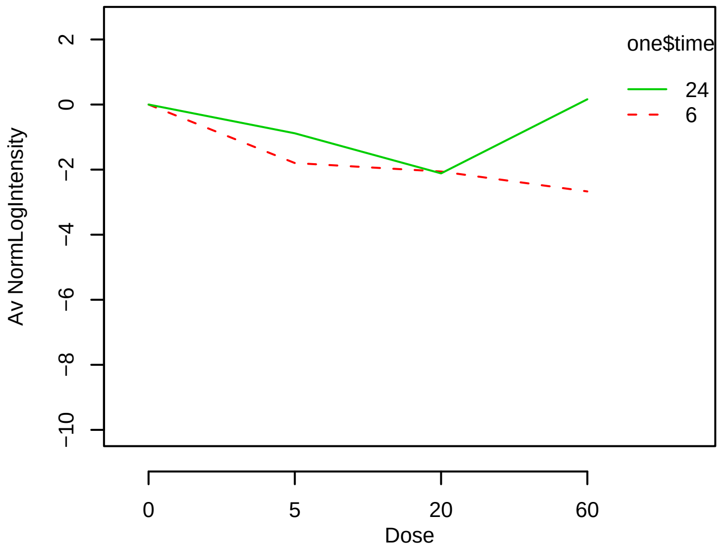

GO_0046330 : positive regulation of JNK cascade

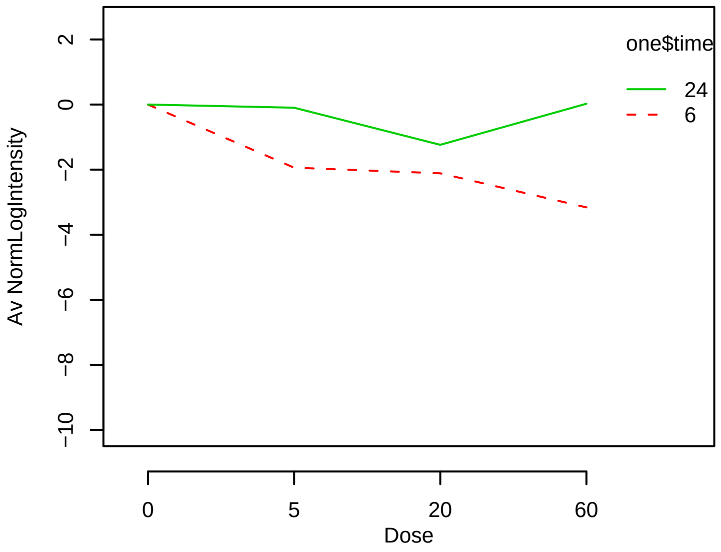

GO_0046339 : diacylglycerol metabolism

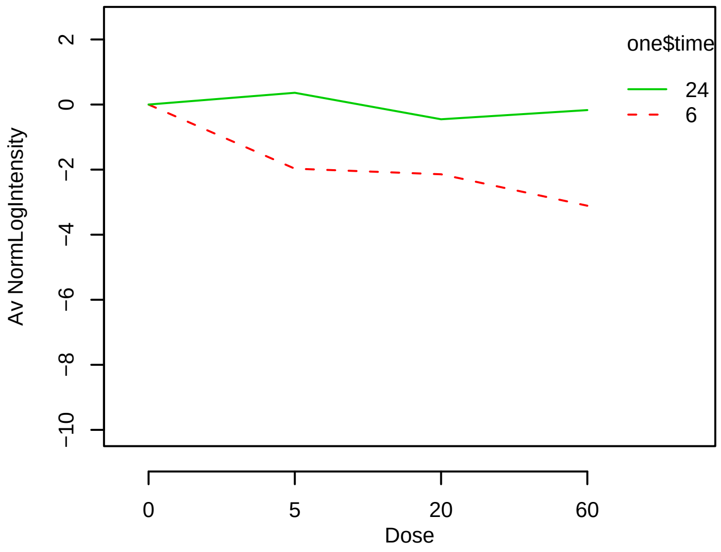

GO_0046348 : amino sugar catabolism

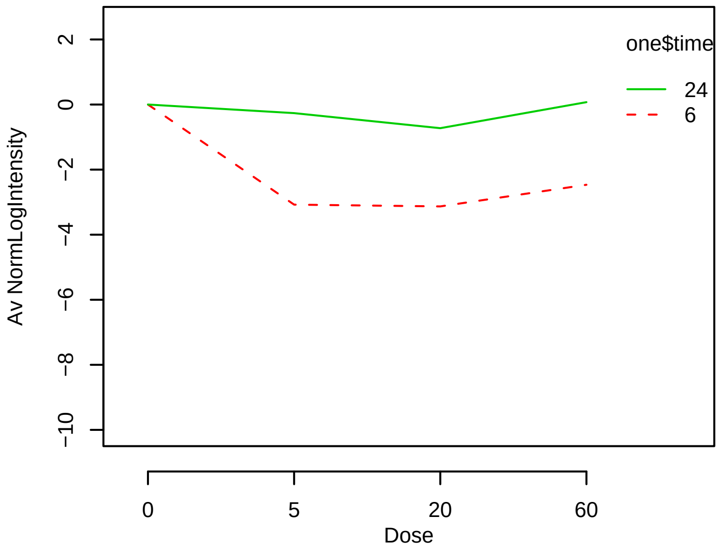

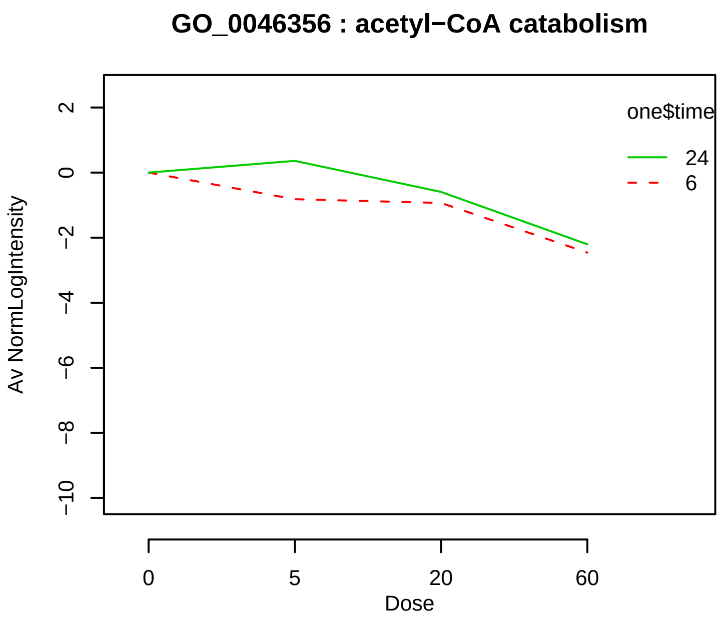
GO_0046356 : acetyl−CoA catabolism
Av NormLogIntensity
2
0
−2
−4
−6
−8
−10
one$time
24
6
0
5
20
60
Dose

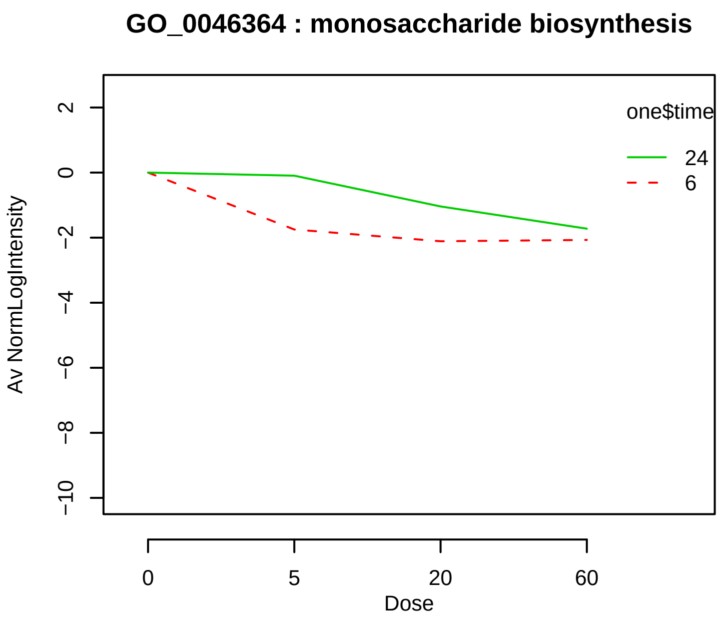
GO_0046364 : monosaccharide biosynthesis
Av NormLogIntensity
2
0
−2
−4
−6
−8
−10
one$time
24
6
0
5
20
60
Dose

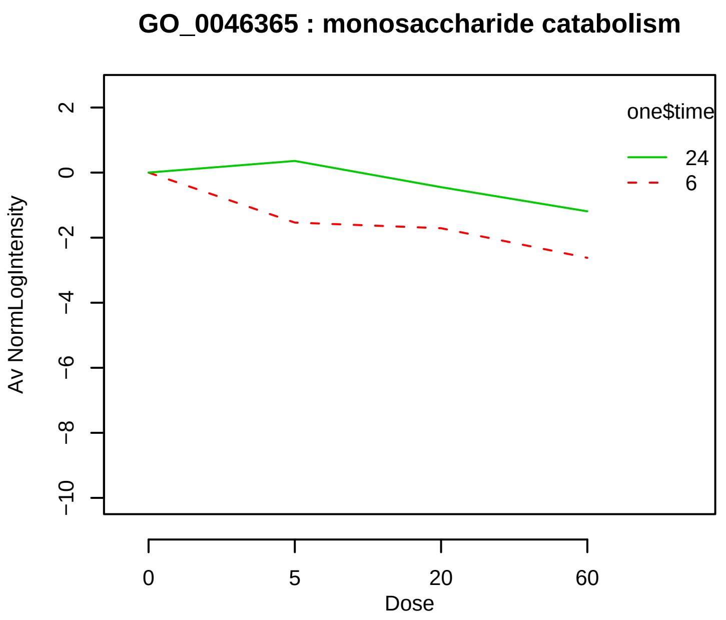
GO_0046365 : monosaccharide catabolism
Av NormLogIntensity
2
0
−2
−4
−6
−8
−10
one$time
24
6
0
5
20
60
Dose

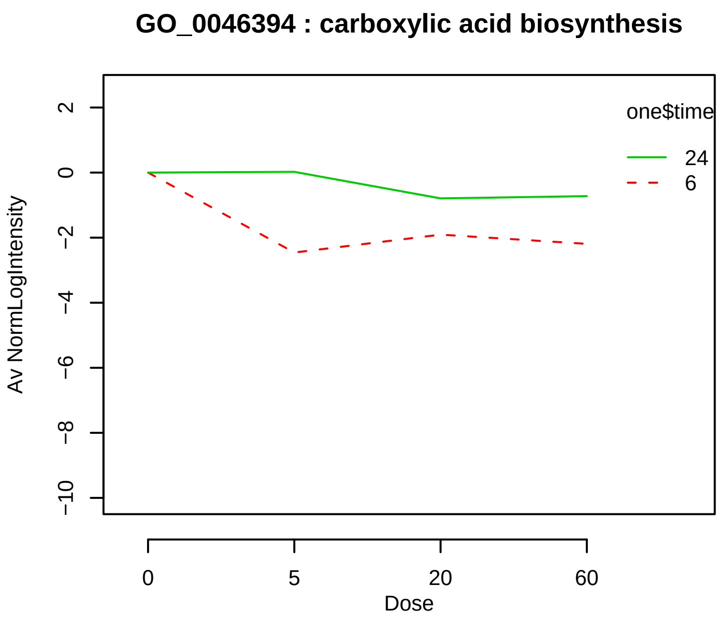
GO_0046394 : carboxylic acid biosynthesis
Av NormLogIntensity
2
0
−2
−4
−6
−8
−10
one$time
24
6
0
5
20
60
Dose

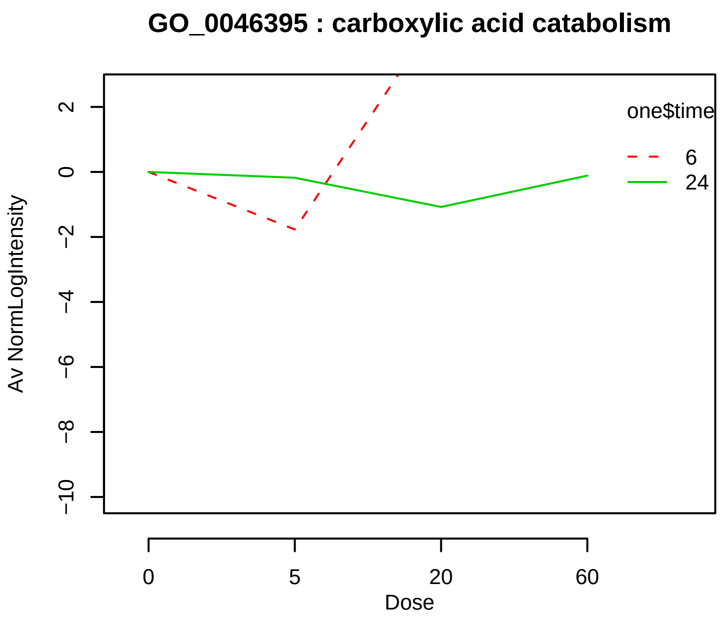
GO_0046395 : carboxylic acid catabolism
Av NormLogIntensity
2
0
−2
−4
−6
−8
−10
one$time
6
24
0
5
20
60
Dose

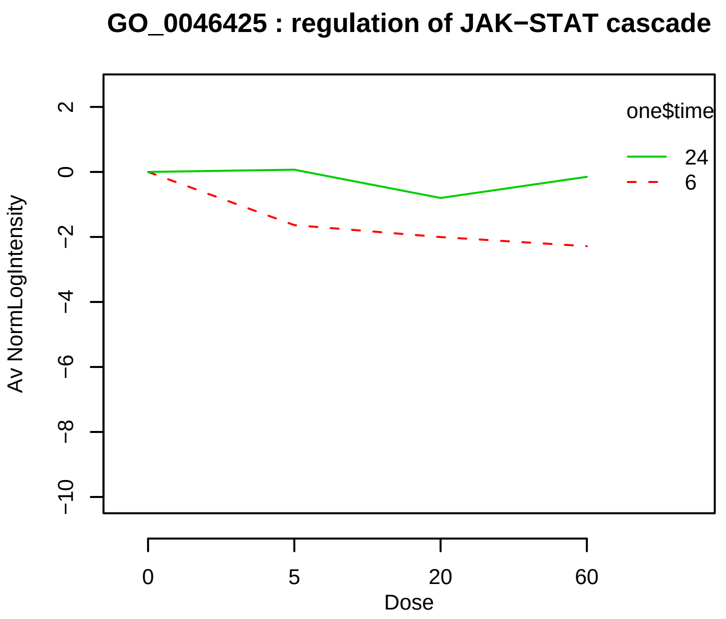
GO_0046425 : regulation of JAK−STAT cascade
Av NormLogIntensity
2
0
−2
−4
−6
−8
−10
one$time
24
6
0
5
20
60
Dose

GO_0046427 : positive regulation of JAK−STAT cascade

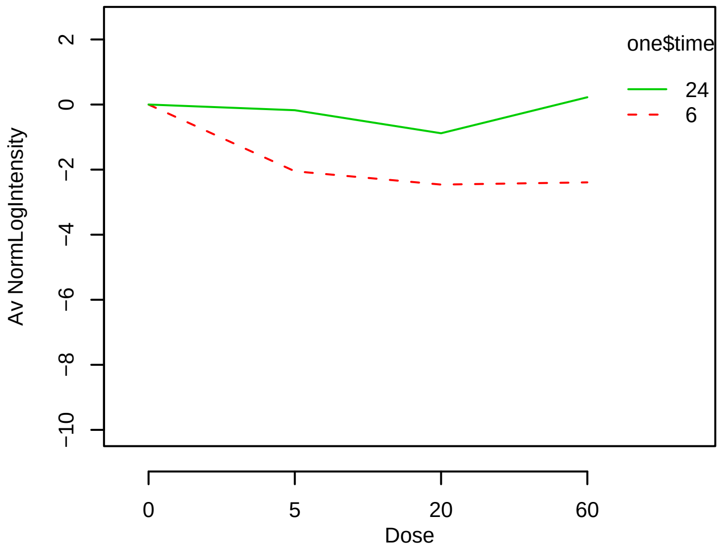

GO_0046456 : icosanoid biosynthesis

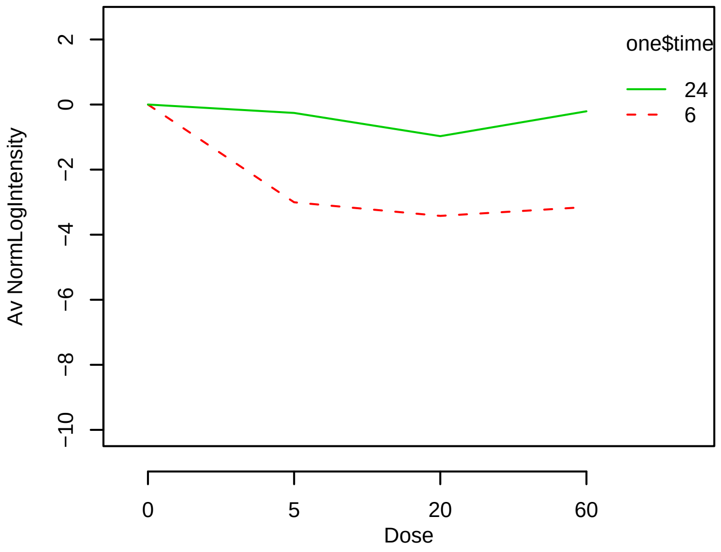

GO_0046457 : prostanoid biosynthesis

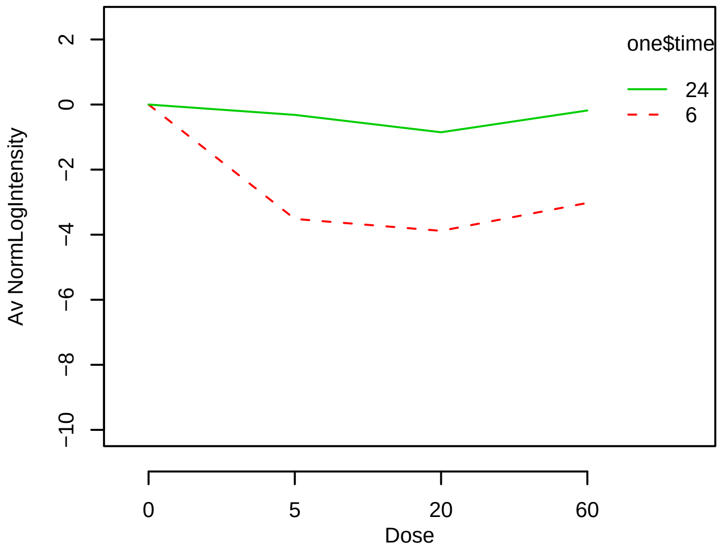

GO_0046460 : neutral lipid biosynthesis

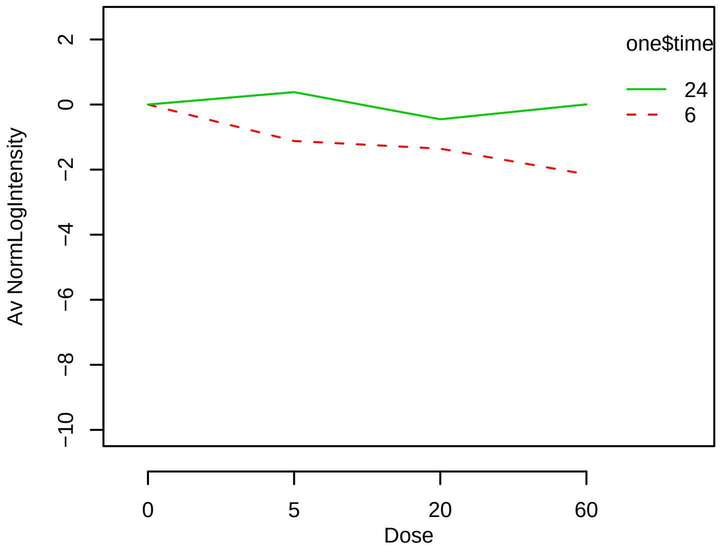

GO_0046463 : acylglycerol biosynthesis

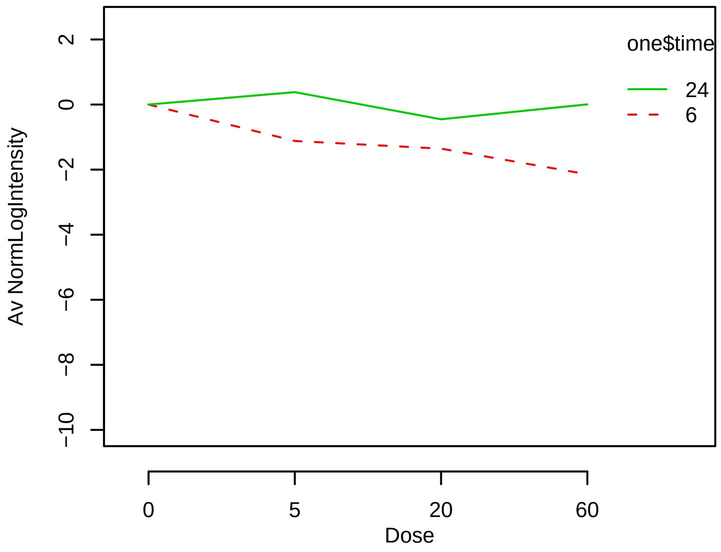

GO_0046466 : membrane lipid catabolism

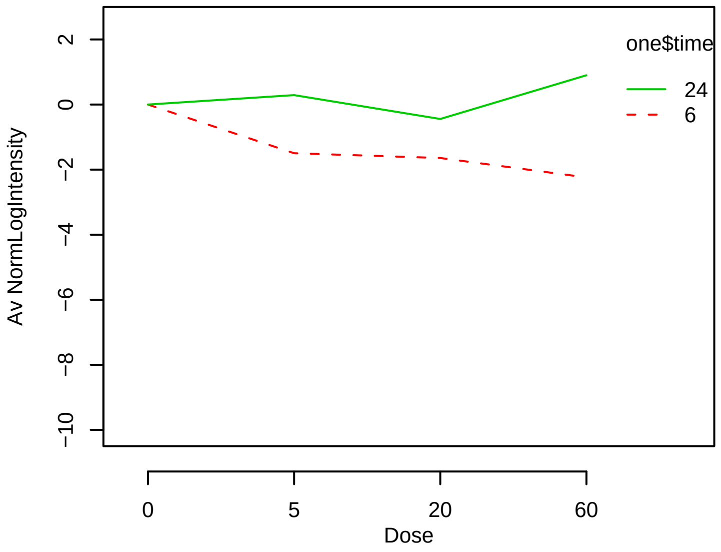

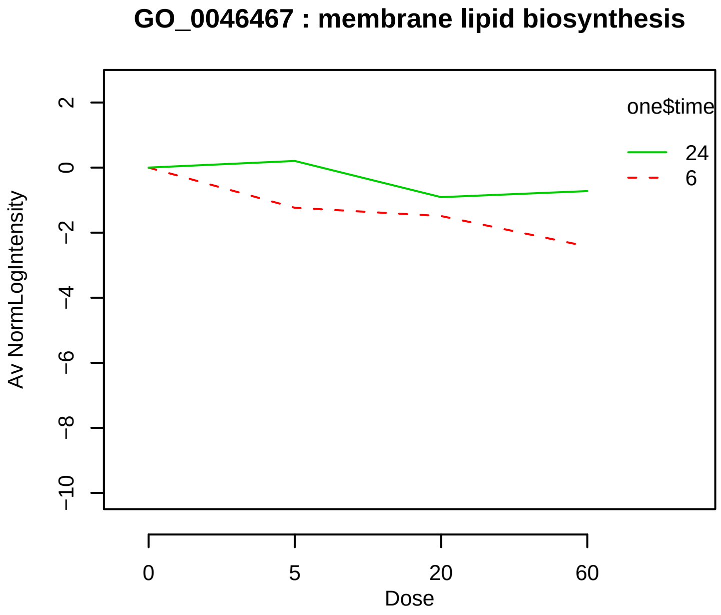
GO_0046467 : membrane lipid biosynthesis
Av NormLogIntensity
2
0
−2
−4
−6
−8
−10
one$time
24
6
0
5
20
60
Dose

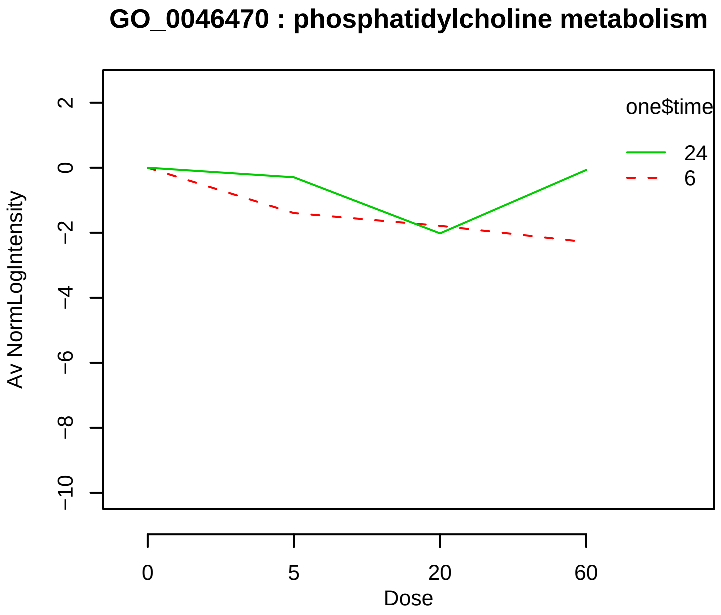
GO_0046470 : phosphatidylcholine metabolism
Av NormLogIntensity
2
0
−2
−4
−6
−8
−10
one$time
24
6
0
5
20
60
Dose

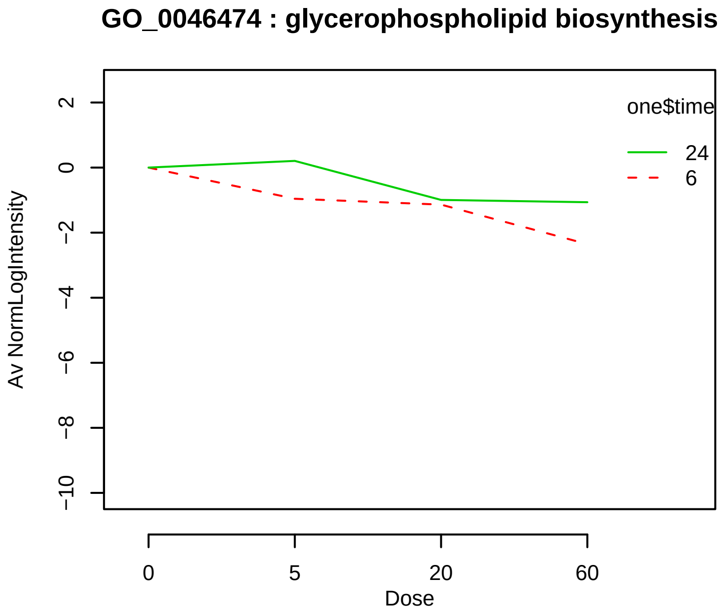
GO_0046474 : glycerophospholipid biosynthesis
Av NormLogIntensity
2
0
−2
−4
−6
−8
−10
one$time
24
6
0
5
20
60
Dose

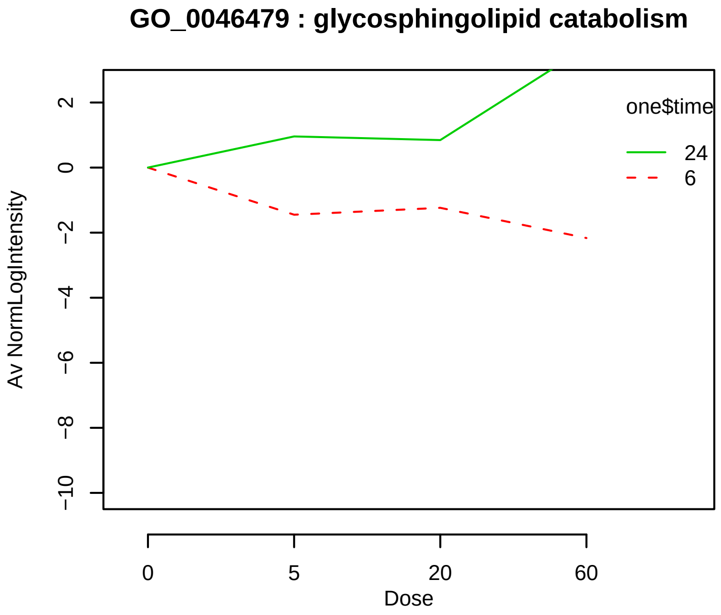
GO_0046479 : glycosphingolipid catabolism
Av NormLogIntensity
2
0
−2
−4
−6
−8
−10
one$time
24
6
0
5
20
60
Dose

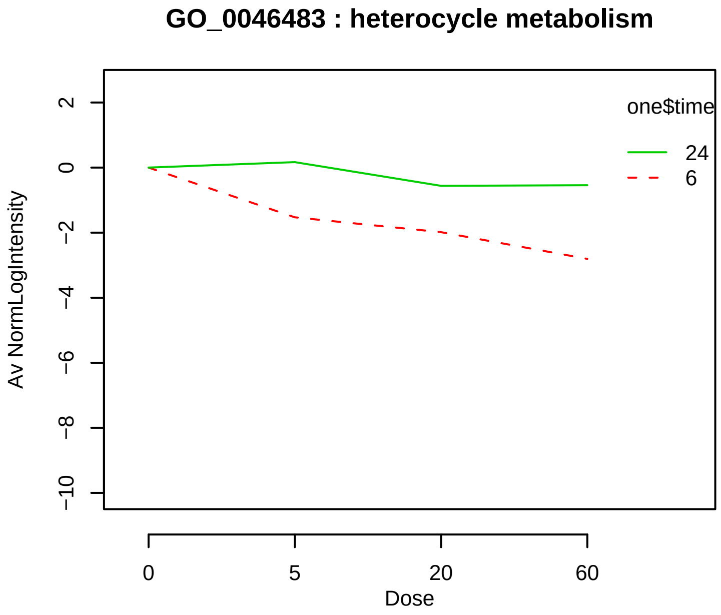
GO_0046483 : heterocycle metabolism
Av NormLogIntensity
2
0
−2
−4
−6
−8
−10
one$time
24
6
0
5
20
60
Dose

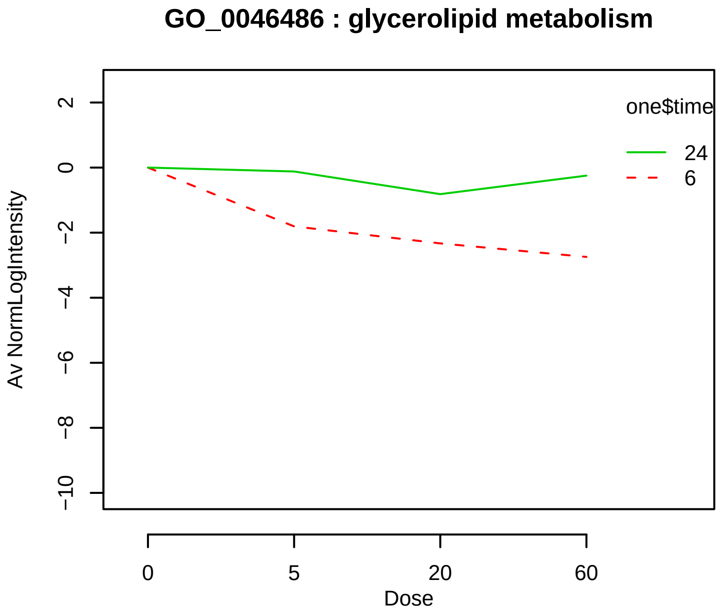
GO_0046486 : glycerolipid metabolism
Av NormLogIntensity
2
0
−2
−4
−6
−8
−10
one$time
24
6
0
5
20
60
Dose

GO_0046488 : phosphatidylinositol metabolism

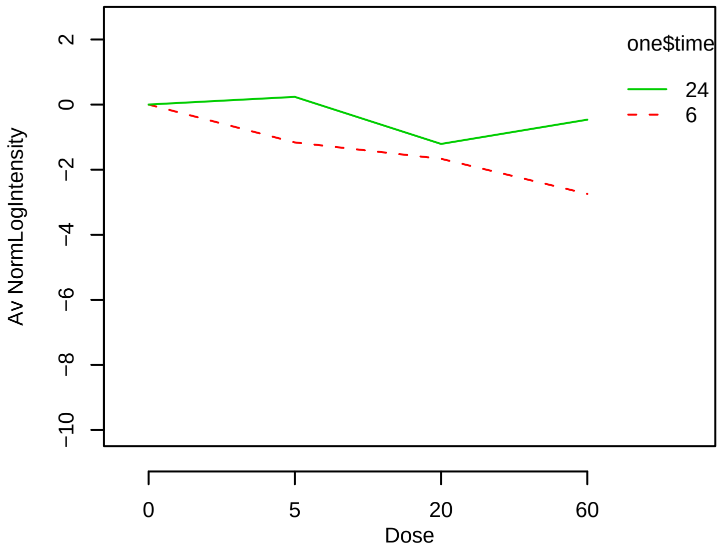

GO_0046489 : phosphoinositide biosynthesis

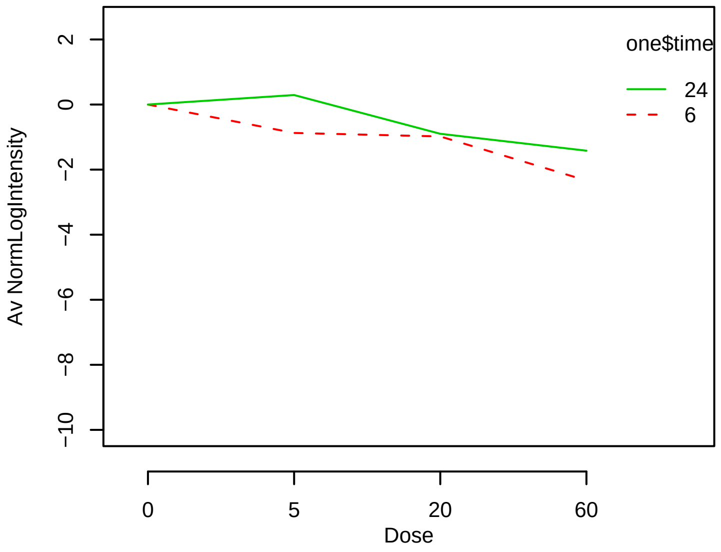

GO_0046504 : glycerol ether biosynthesis

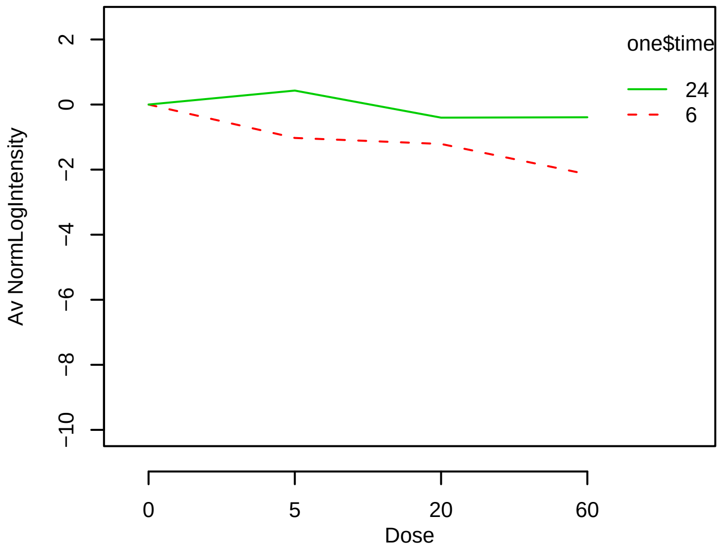

GO_0046513 : ceramide biosynthesis

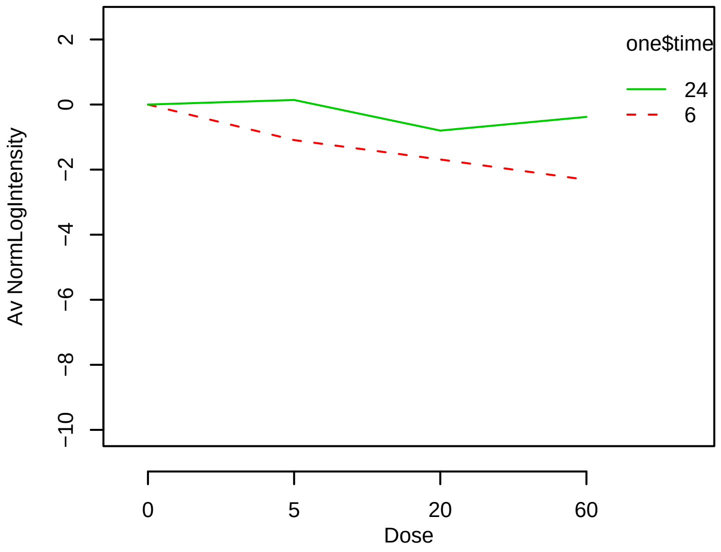

GO_0046519 : sphingoid metabolism

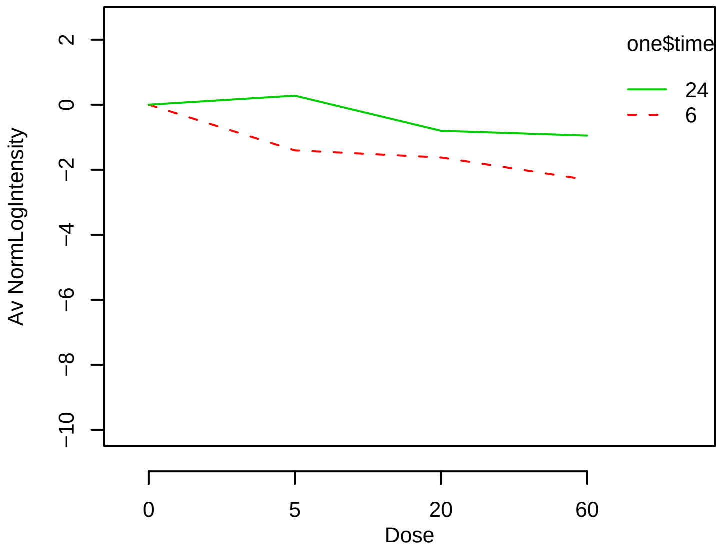

GO_0046520 : sphingoid biosynthesis

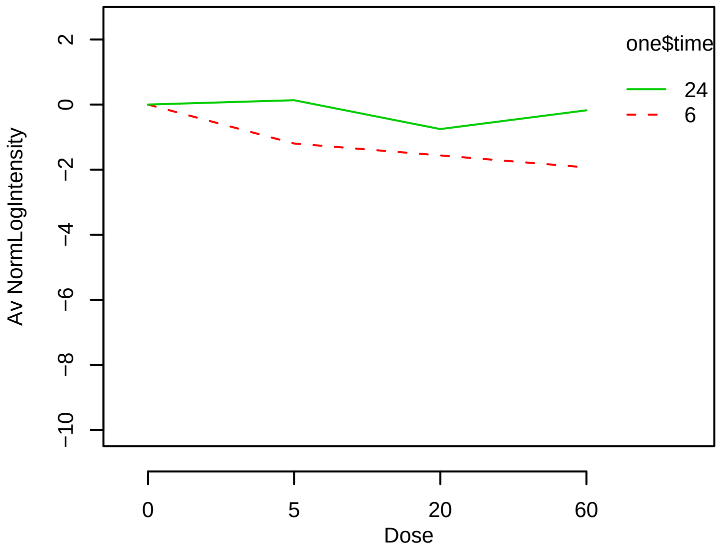

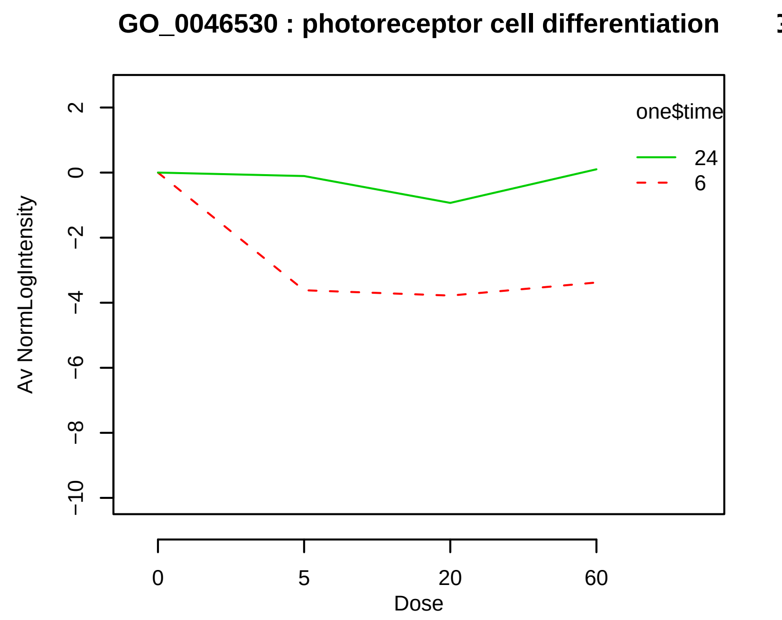
GO_0046530 : photoreceptor cell differentiation
one$time
24
6
Av NormLogIntensity
Dose

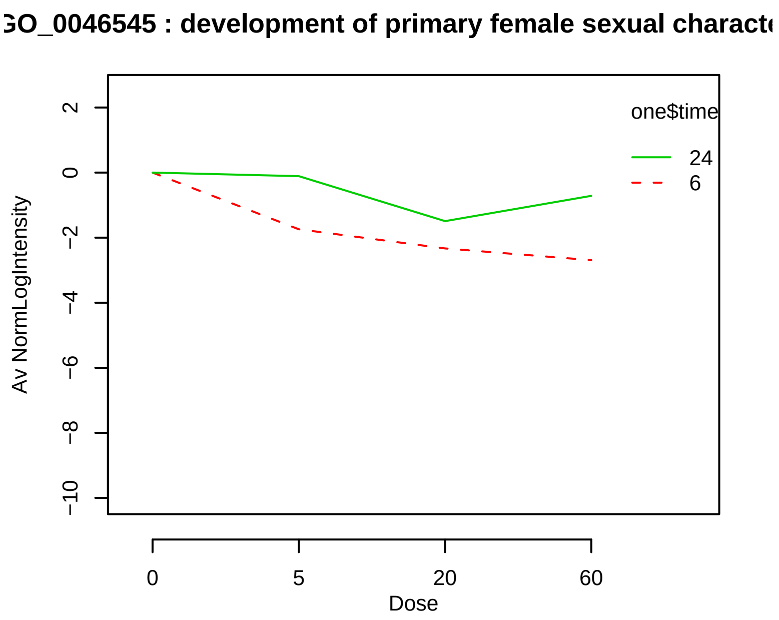
GO_0046545 : development of primary female sexual characte
one$time
24
6
Av NormLogIntensity
Dose

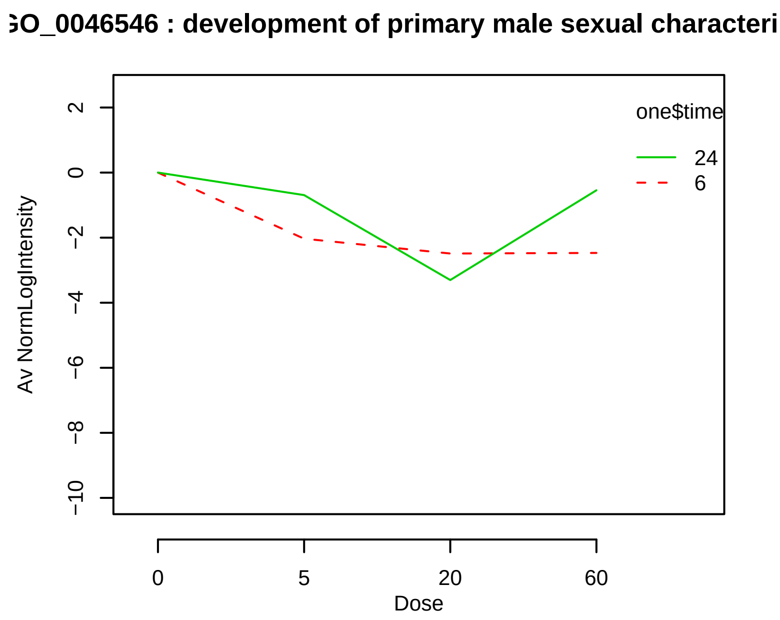
GO_0046546 : development of primary male sexual characteri
one$time
24
6
Av NormLogIntensity
Dose

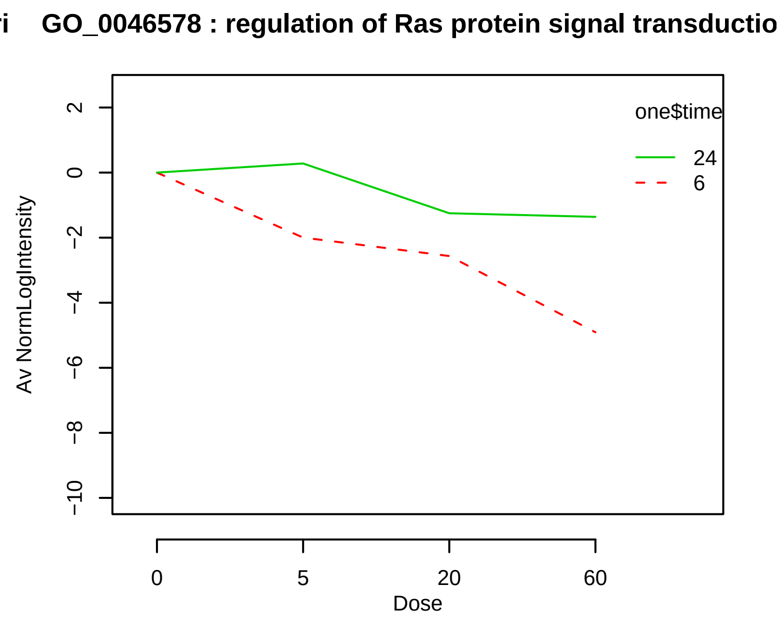
GO_0046578 : regulation of Ras protein signal transductio
one$time
24
6
Av NormLogIntensity
Dose

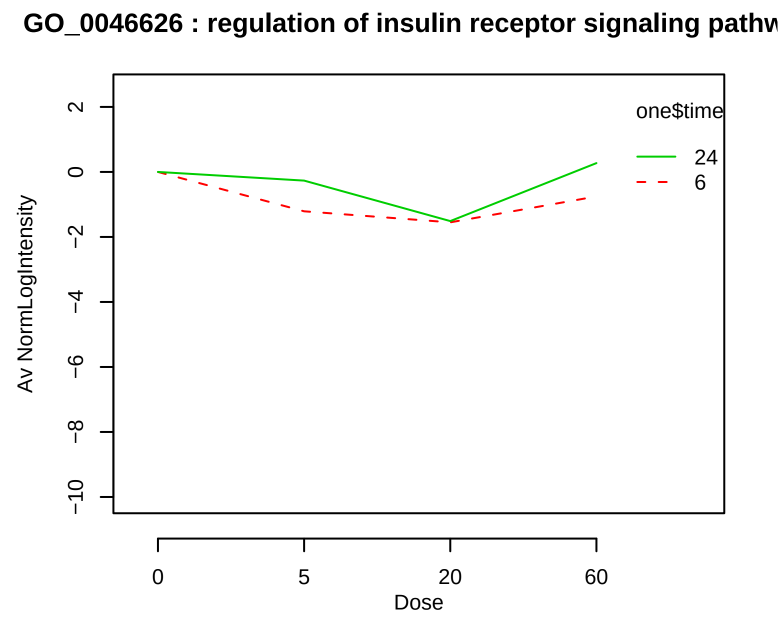
GO_0046626 : regulation of insulin receptor signaling pathw
one$time
24
6
Av NormLogIntensity
Dose

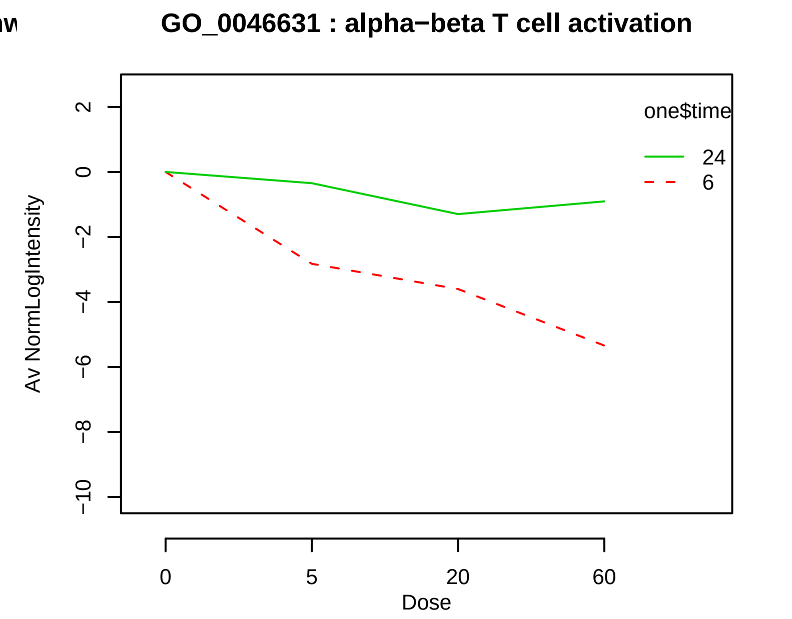
GO_0046631 : alpha−beta T cell activation
one$time
24
6
Av NormLogIntensity
Dose

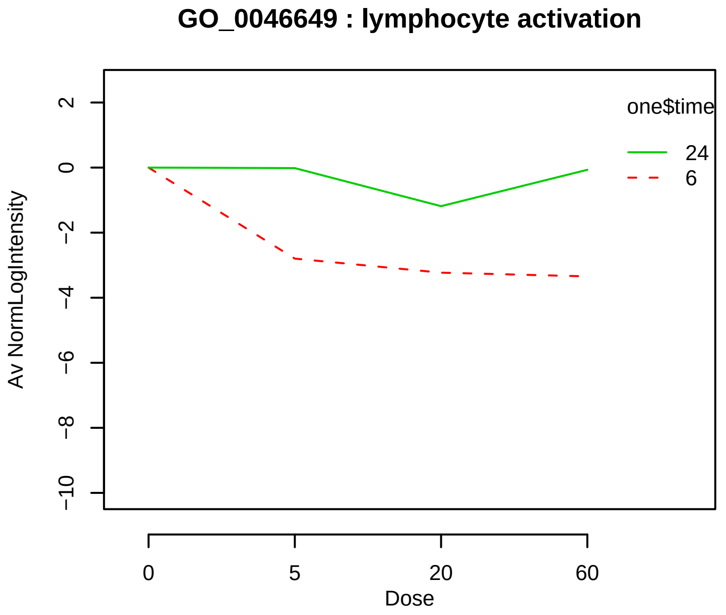
GO_0046649 : lymphocyte activation
one$time
24
6
Av NormLogIntensity
Dose
0
5
20
60

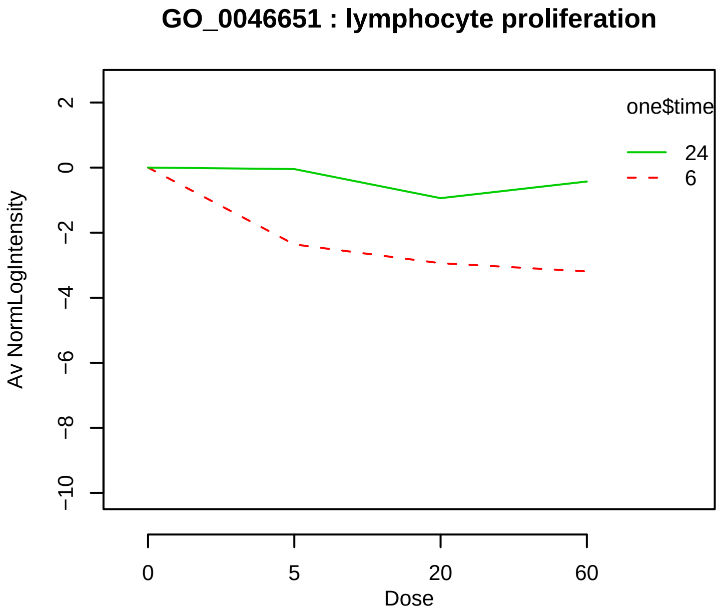
GO_0046651 : lymphocyte proliferation
one$time
24
6
Av NormLogIntensity
Dose
0
5
20
60

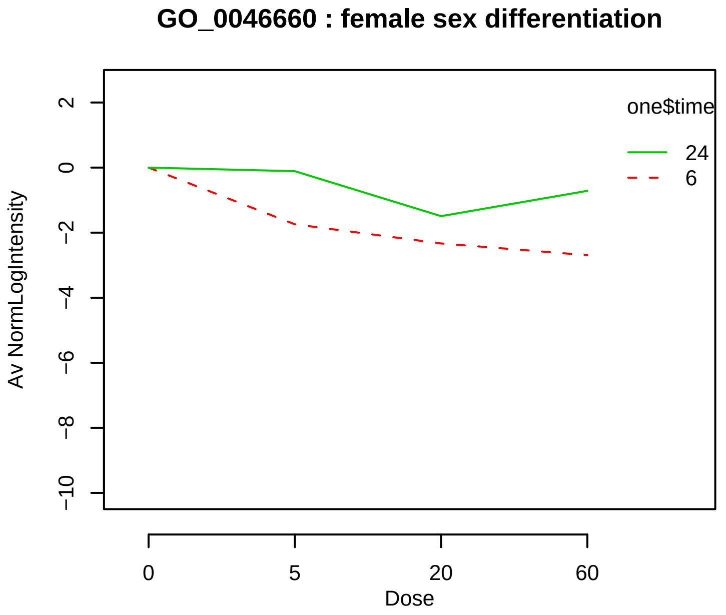
GO_0046660 : female sex differentiation
one$time
24
6
Av NormLogIntensity
Dose
0
5
20
60

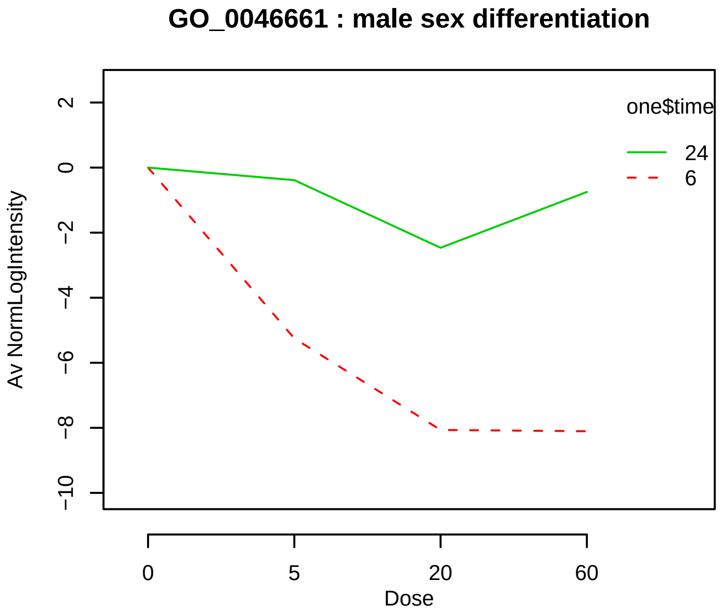
GO_0046661 : male sex differentiation
one$time
24
6
Av NormLogIntensity
Dose
0
5
20
60

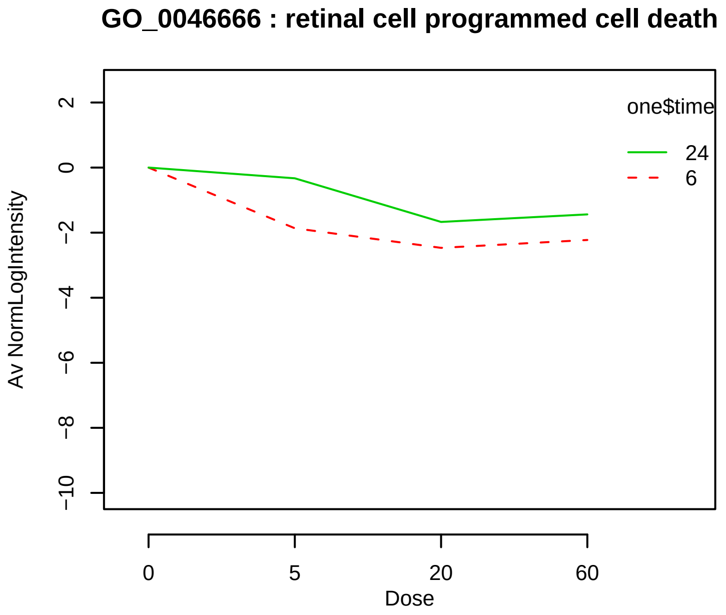
GO_0046666 : retinal cell programmed cell death
one$time
24
6
Av NormLogIntensity
Dose
0
5
20
60

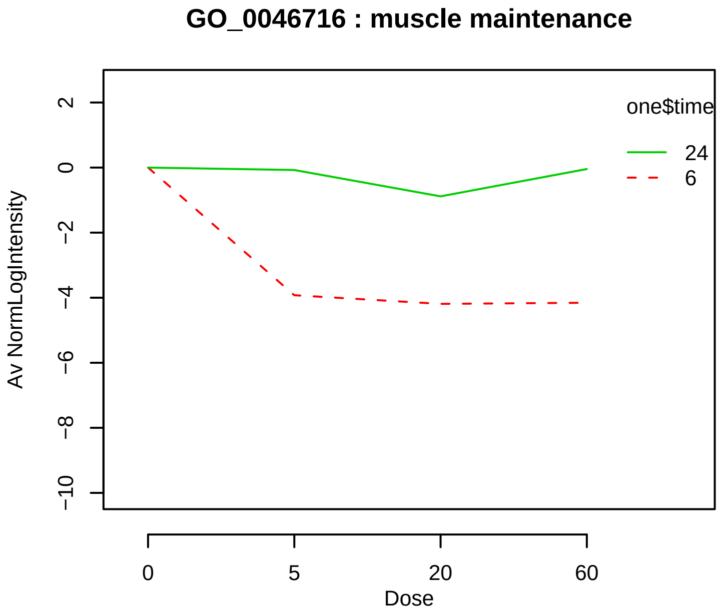
GO_0046716 : muscle maintenance
one$time
24
6
Av NormLogIntensity
Dose
0
5
20
60

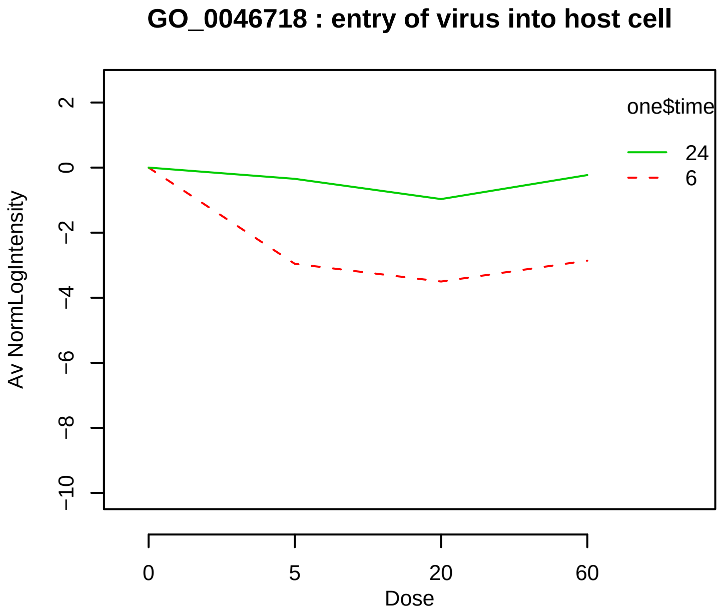
GO_0046718 : entry of virus into host cell
one$time
24
6
Av NormLogIntensity
Dose

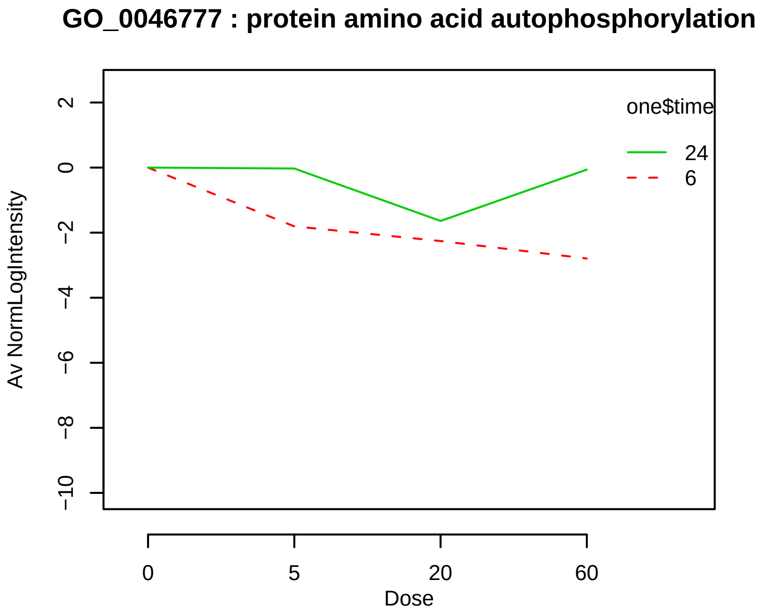
GO_0046777 : protein amino acid autophosphorylation
one$time
24
6
Av NormLogIntensity
Dose

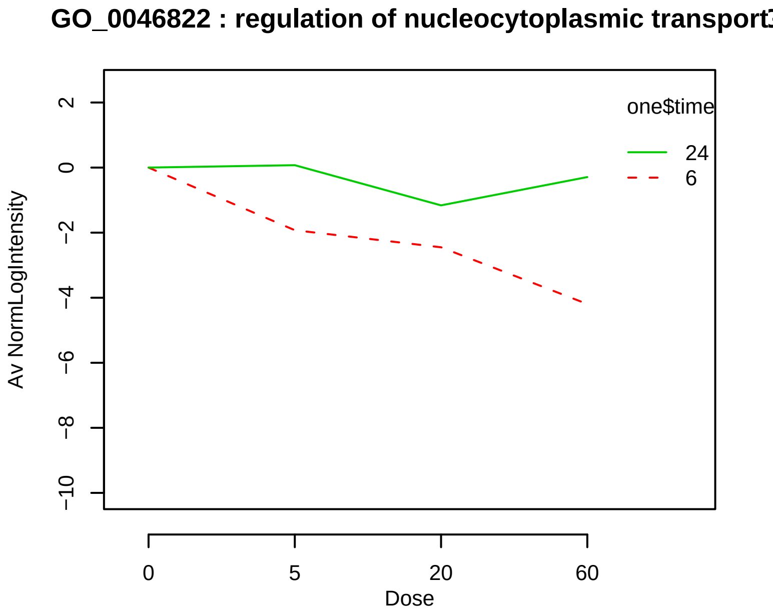
GO_0046822 : regulation of nucleocytoplasmic transport
one$time
24
6
Av NormLogIntensity
Dose

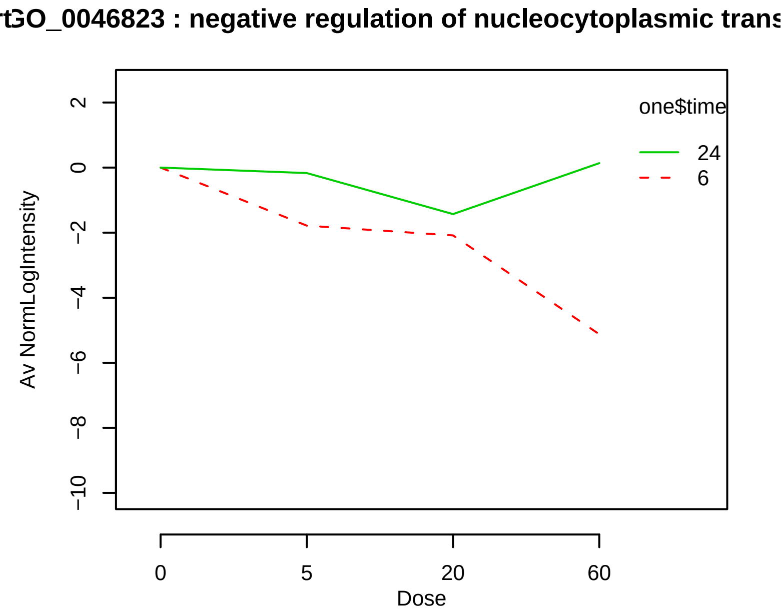
GO_0046823 : negative regulation of nucleocytoplasmic trans
one$time
24
6
Av NormLogIntensity
Dose

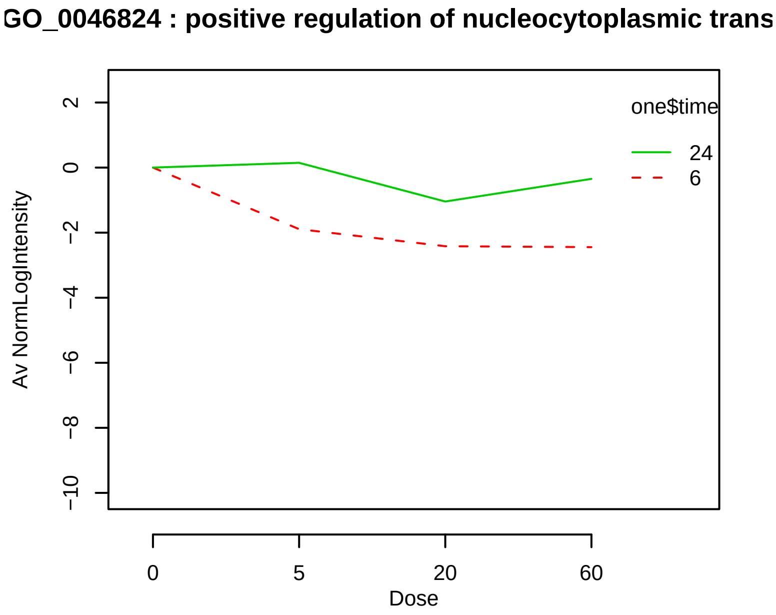
GO_0046824 : positive regulation of nucleocytoplasmic trans
one$time
24
6
Av NormLogIntensity
Dose

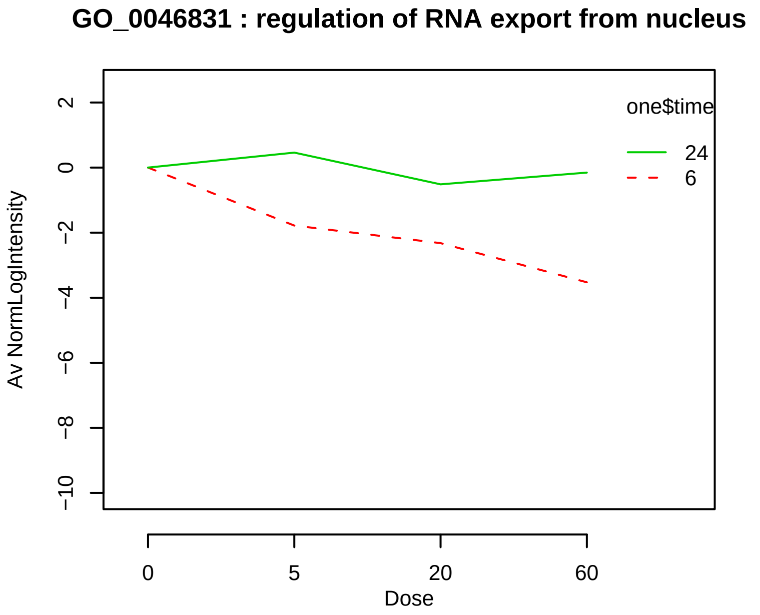
GO_0046831 : regulation of RNA export from nucleus
one$time
24
6
Av NormLogIntensity
Dose

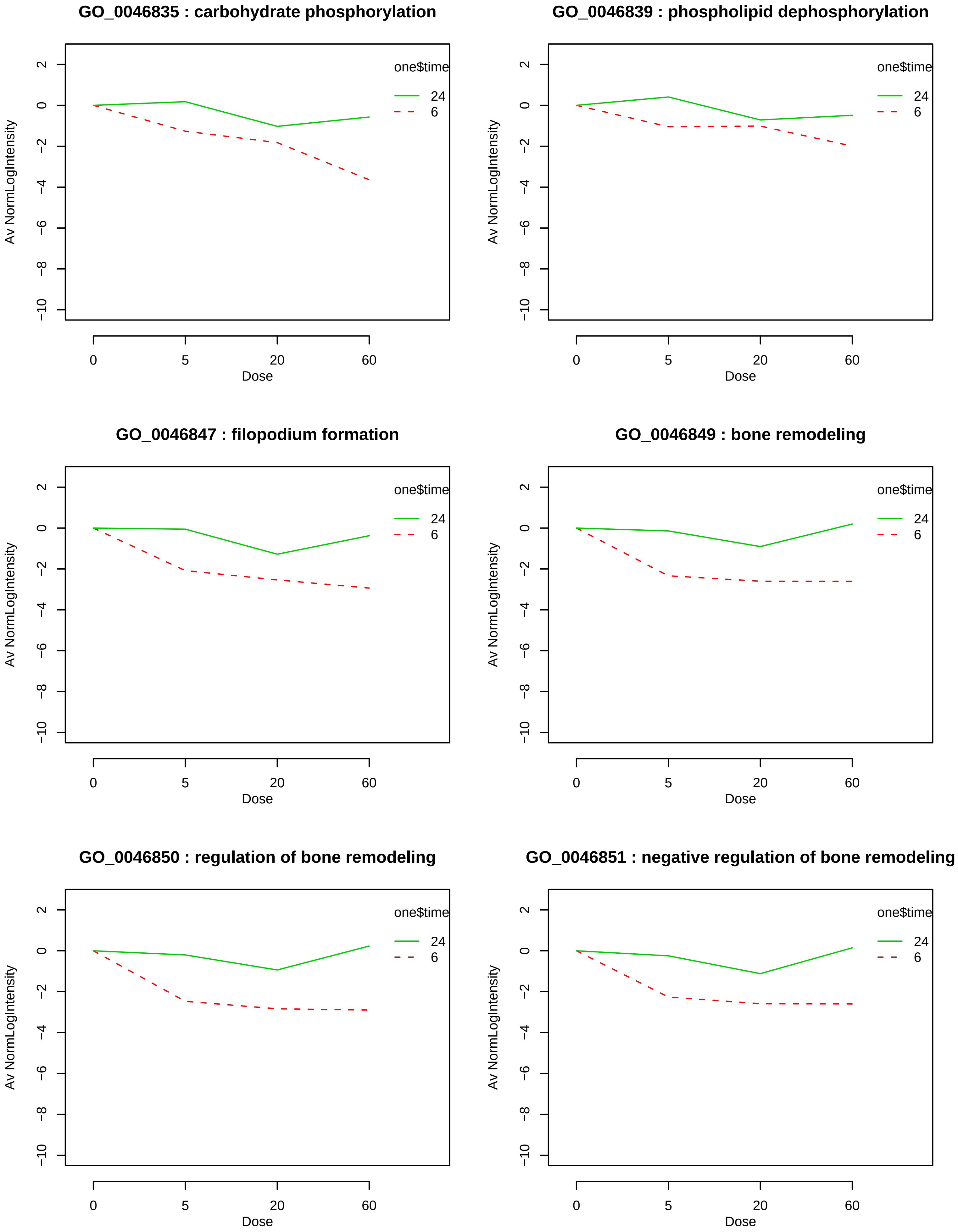
GO_0046835 : carbohydrate phosphorylation
Av NormLogIntensity
2
0
−2
−4
−6
−8
−10
one$time
24
6
0
5
20
60
Dose
GO_0046839 : phospholipid dephosphorylation
Av NormLogIntensity
one$time
24
6
Dose
GO_0046847 : filopodium formation
Av NormLogIntensity
one$time
24
6
Dose
GO_0046849 : bone remodeling
Av NormLogIntensity
one$time
24
6
Dose
GO_0046850 : regulation of bone remodeling
Av NormLogIntensity
one$time
24
6
Dose
GO_0046851 : negative regulation of bone remodeling
Av NormLogIntensity
one$time
24
6
Dose

GO_0046852 : positive regulation of bone remodeling

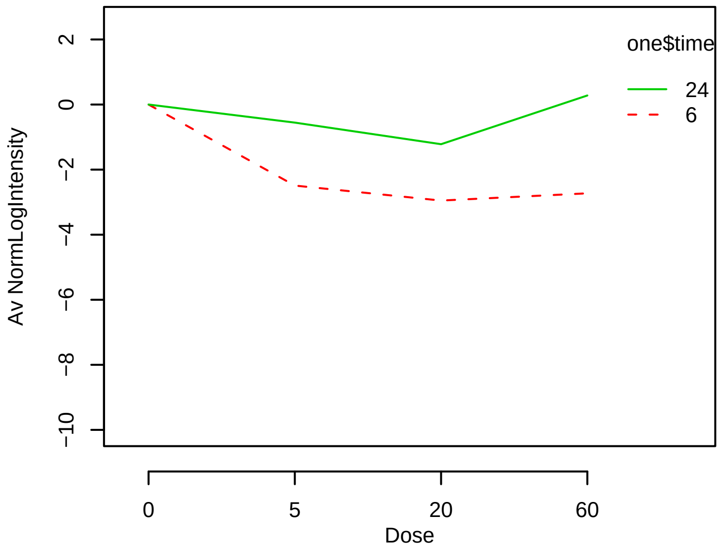

GO_0046879 : hormone secretion

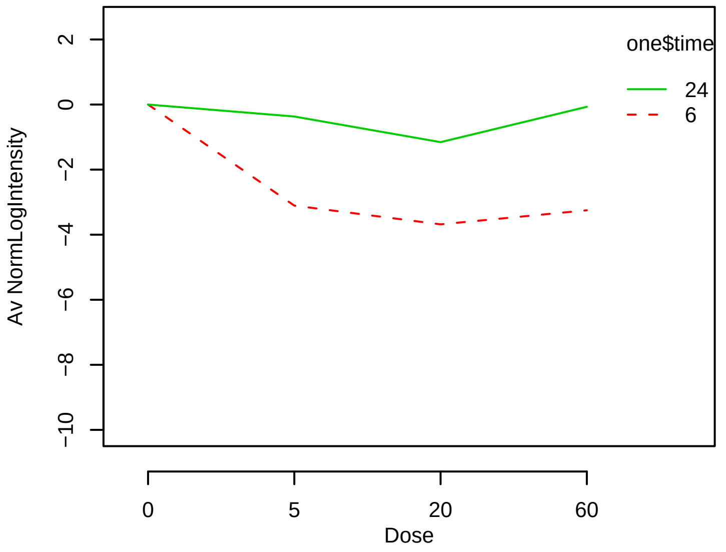

GO_0046883 : regulation of hormone secretion

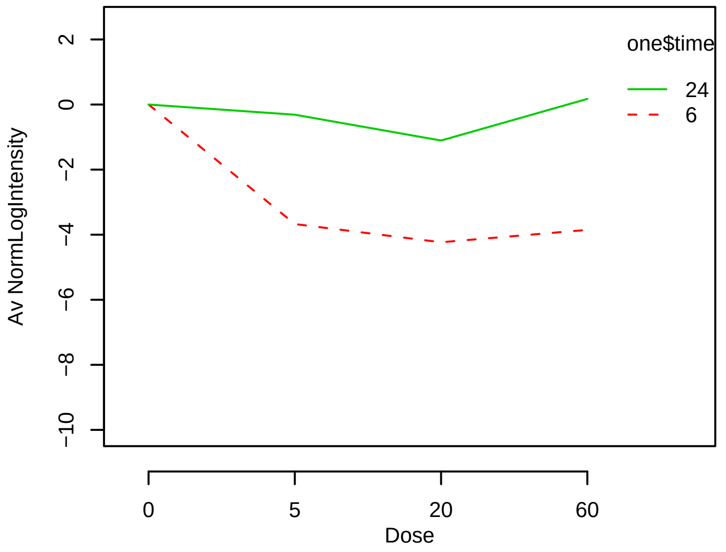

GO_0046888 : negative regulation of hormone secretion

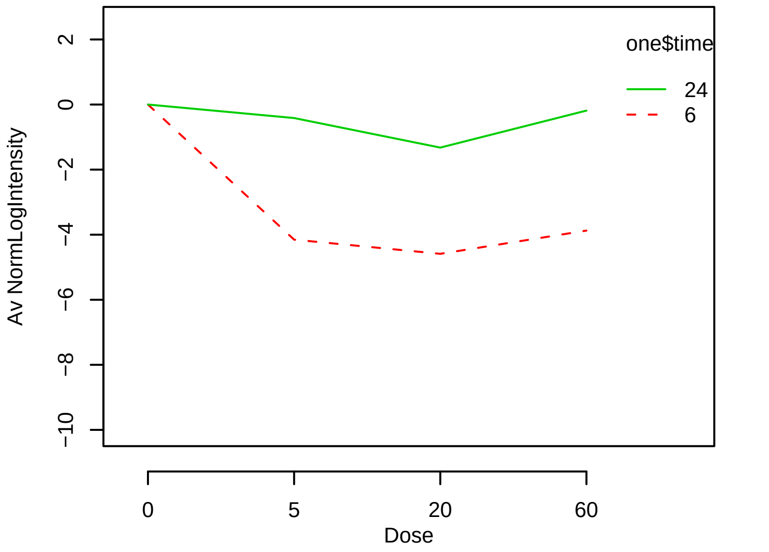

GO_0046890 : regulation of lipid biosynthesis

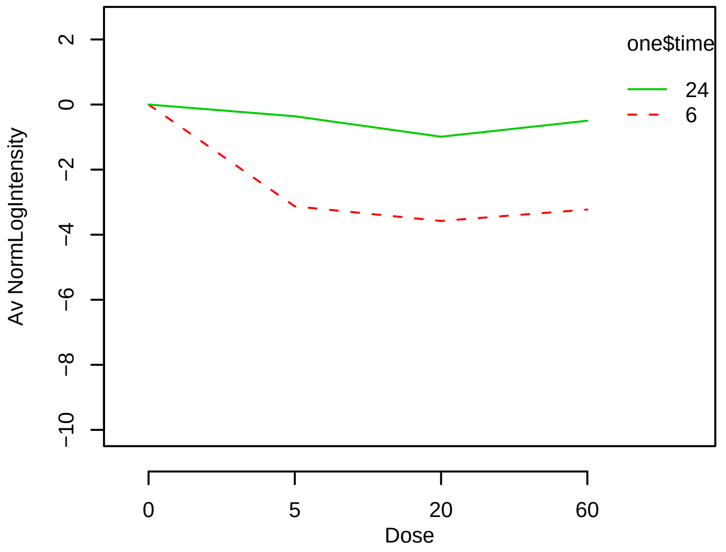

GO_0046916 : transition metal ion homeostasis

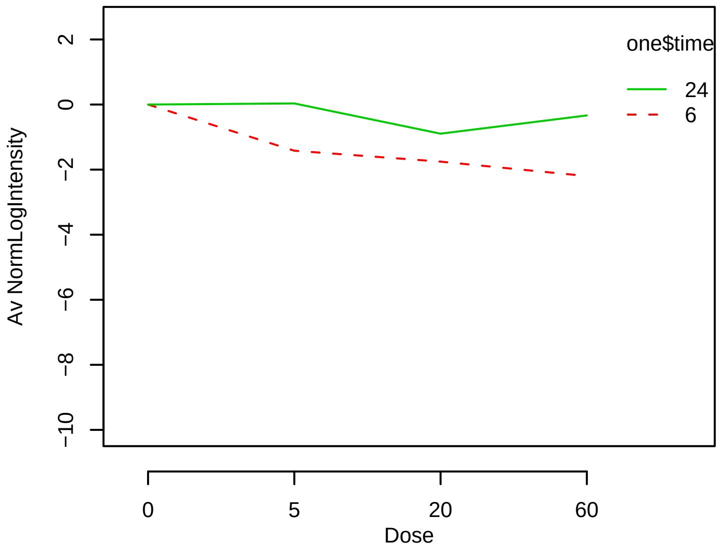

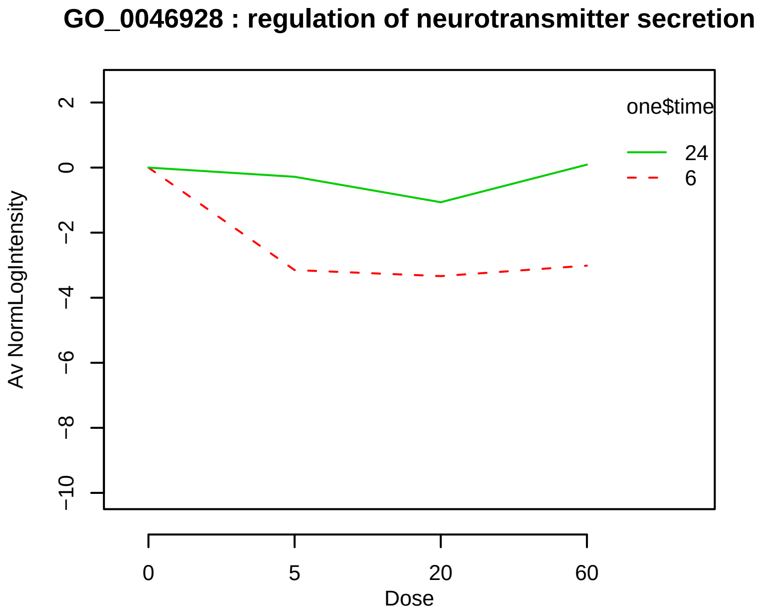
GO_0046928 : regulation of neurotransmitter secretion
one$time
24
6
Av NormLogIntensity
Dose

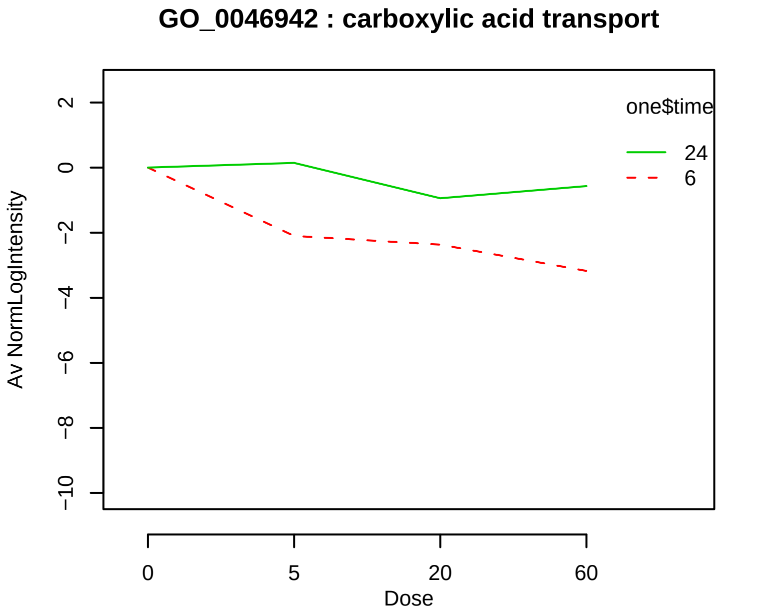
GO_0046942 : carboxylic acid transport
one$time
24
6
Av NormLogIntensity
Dose

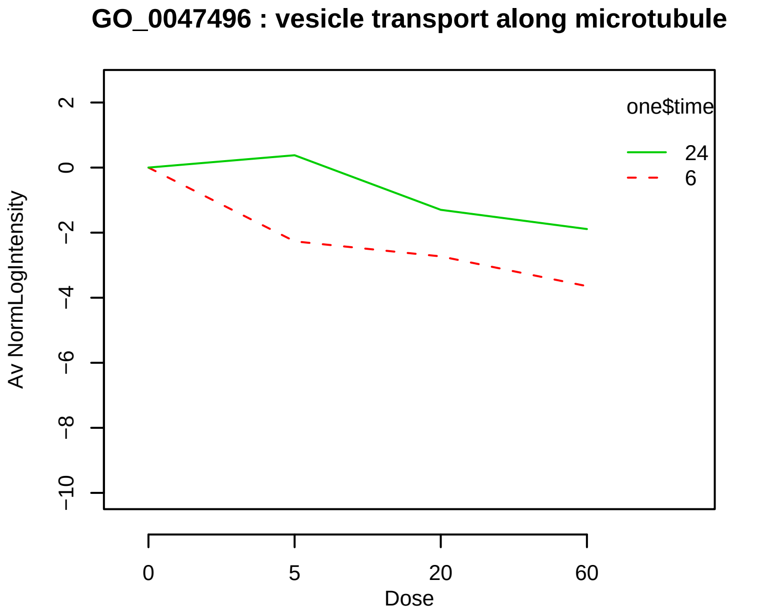
GO_0047496 : vesicle transport along microtubule
one$time
24
6
Av NormLogIntensity
Dose

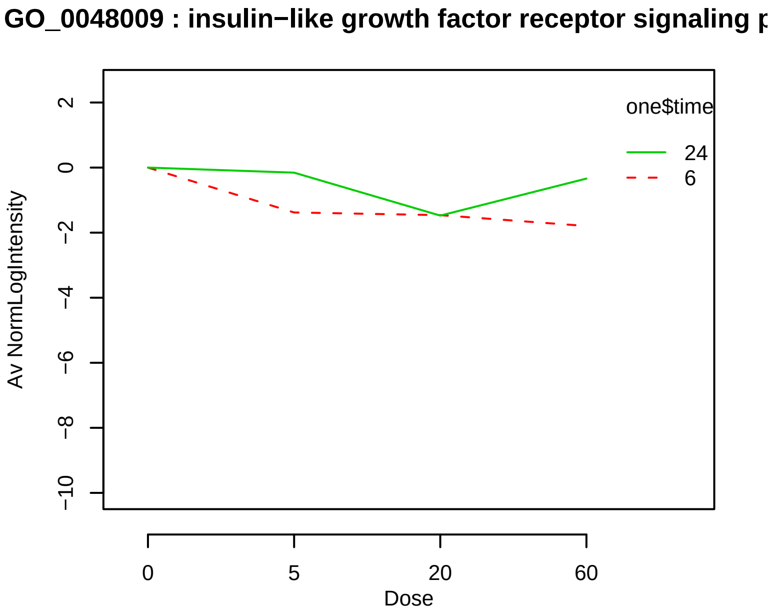
GO_0048009 : insulin−like growth factor receptor signaling p
one$time
24
6
Av NormLogIntensity
Dose

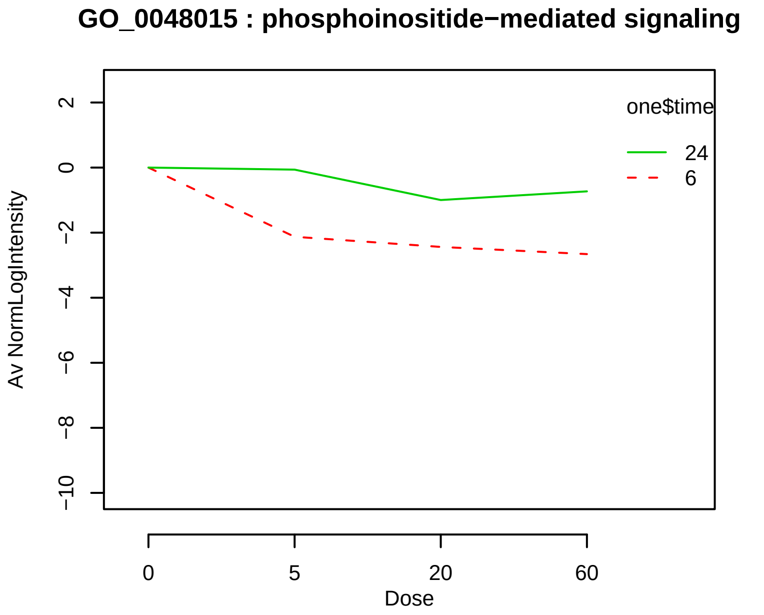
GO_0048015 : phosphoinositide−mediated signaling
one$time
24
6
Av NormLogIntensity
Dose

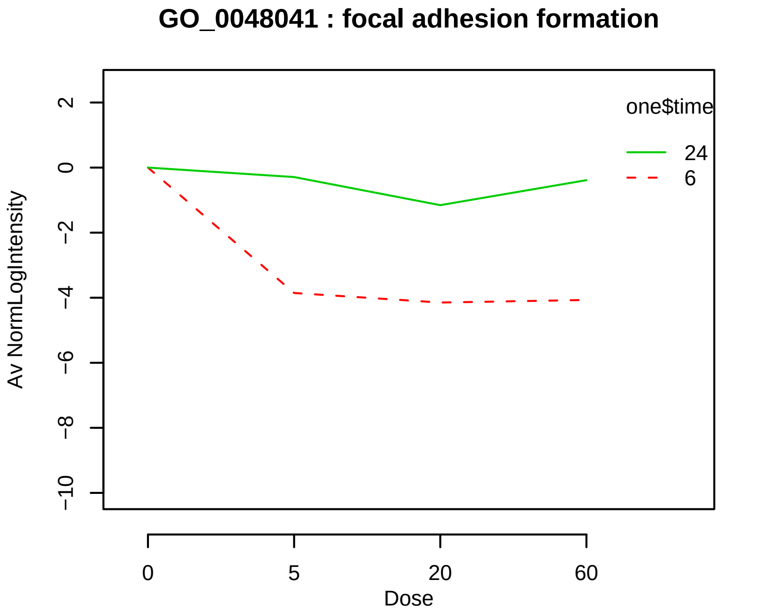
GO_0048041 : focal adhesion formation
one$time
24
6
Av NormLogIntensity
Dose

GO_0048048 : embryonic eye morphogenesis

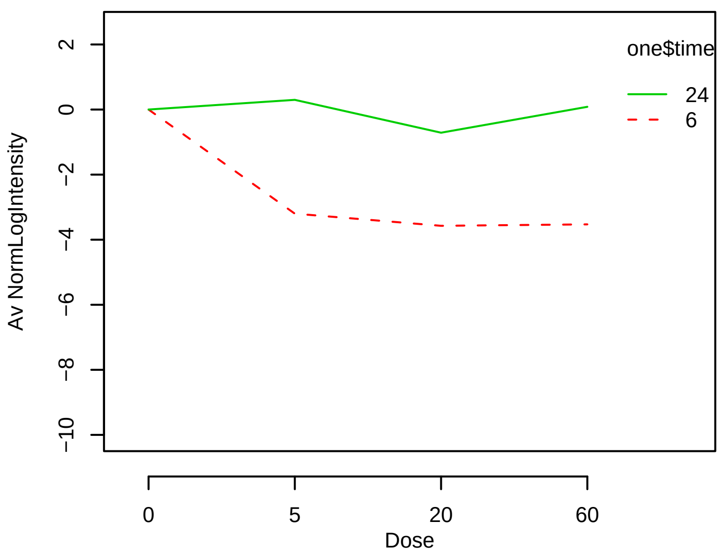

GO_0048066 : pigmentation during development

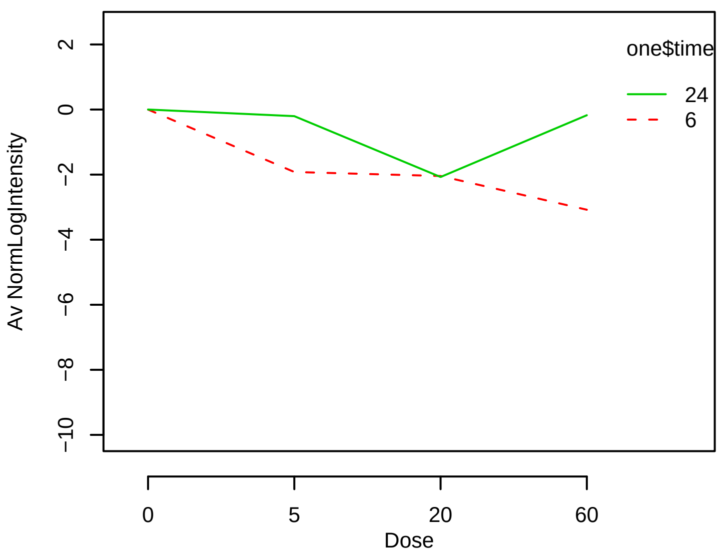

GO_0048069 : eye pigmentation

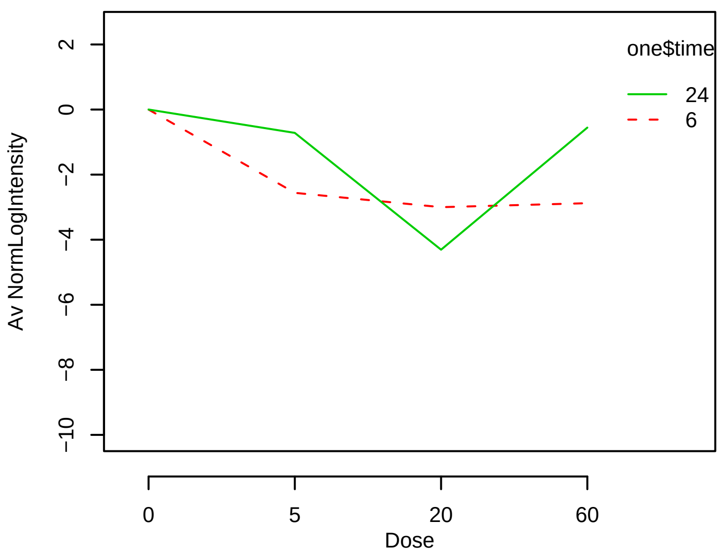

GO_0048144 : fibroblast proliferation

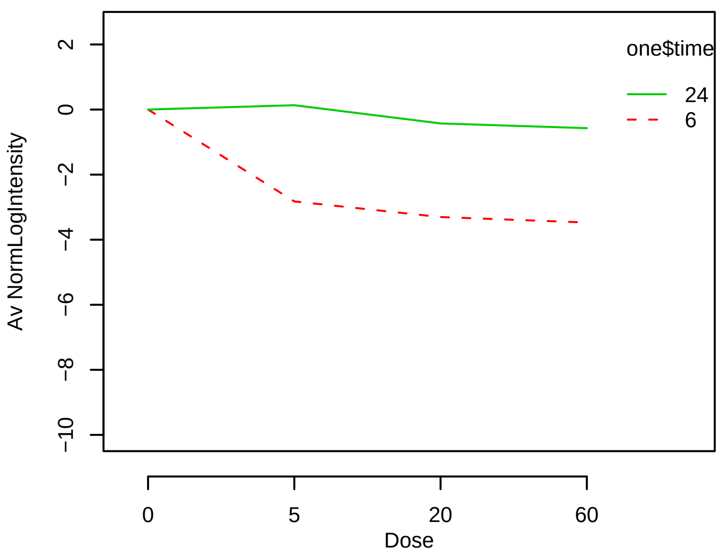

GO_0048145 : regulation of fibroblast proliferation

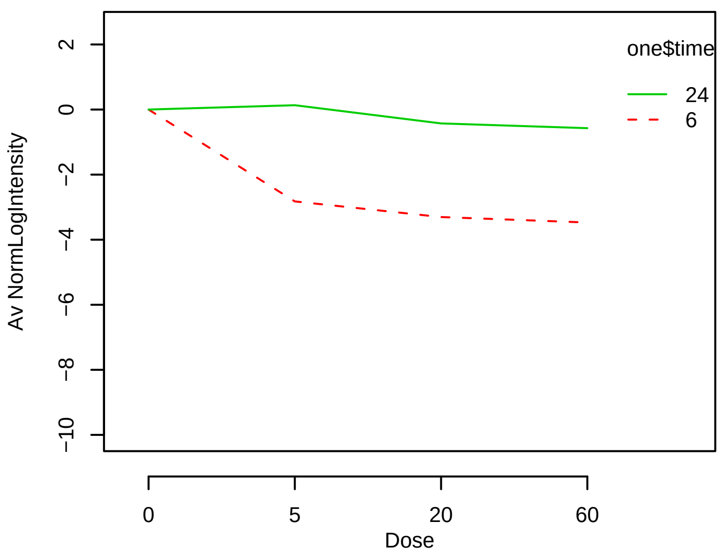

GO_0048157 : oogenesis (sensu Mammalia)

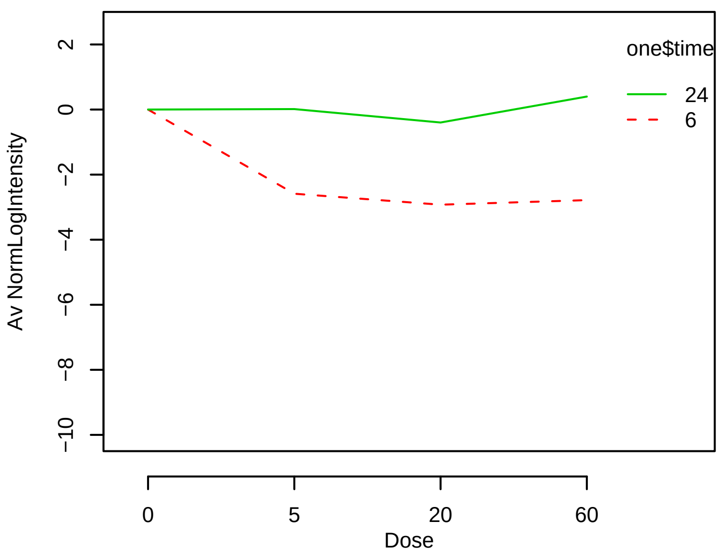

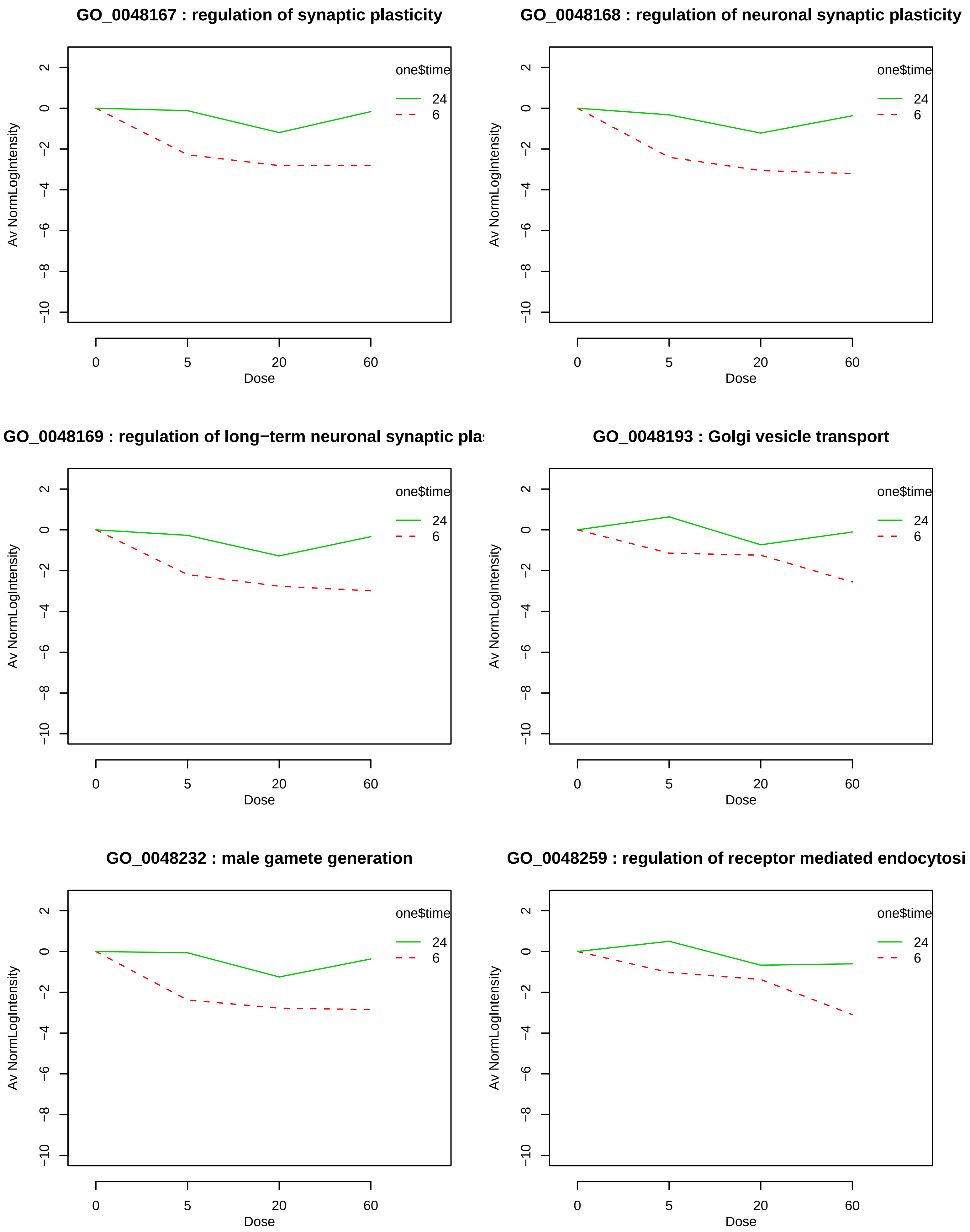
GO_0048167 : regulation of synaptic plasticity
Av NormLogIntensity
Dose
one$time
24
6
GO_0048168 : regulation of neuronal synaptic plasticity
Av NormLogIntensity
Dose
one$time
24
6
GO_0048169 : regulation of long−term neuronal synaptic pla
Av NormLogIntensity
Dose
one$time
24
6
GO_0048193 : Golgi vesicle transport
Av NormLogIntensity
Dose
one$time
24
6
GO_0048232 : male gamete generation
Av NormLogIntensity
Dose
one$time
24
6
GO_0048259 : regulation of receptor mediated endocytosi
Av NormLogIntensity
Dose
one$time
24
6

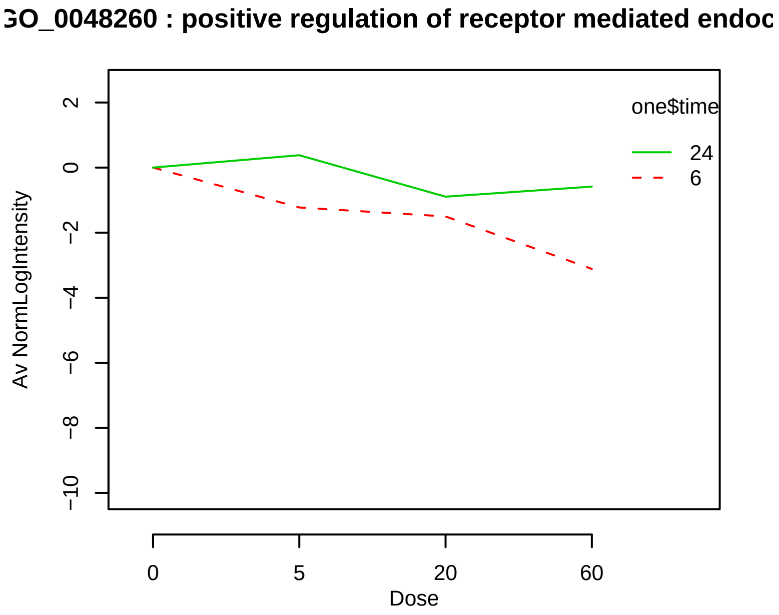
GO_0048260 : positive regulation of receptor mediated endoc
Av NormLogIntensity
Dose
one$time
24
6
2
0
−2
−4
−6
−8
−10
0
5
20
60

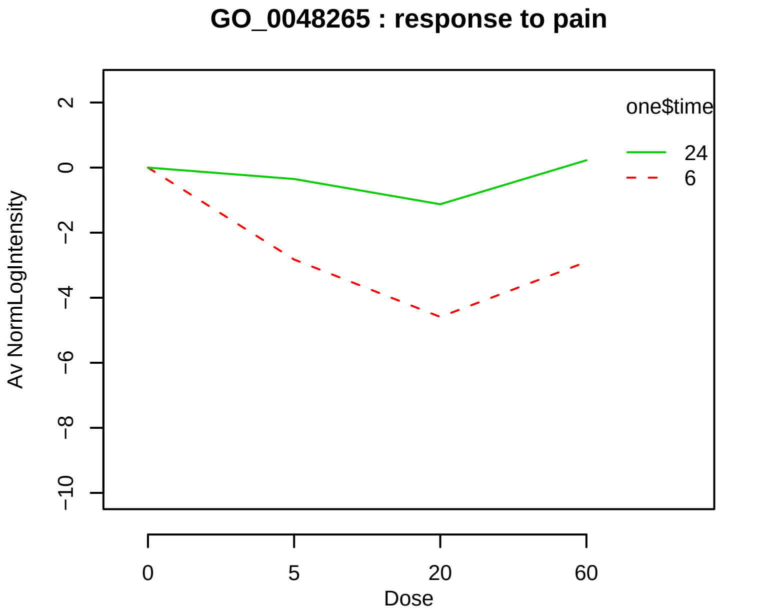
GO_0048265 : response to pain
Av NormLogIntensity
Dose
one$time
24
6
2
0
−2
−4
−6
−8
−10
0
5
20
60

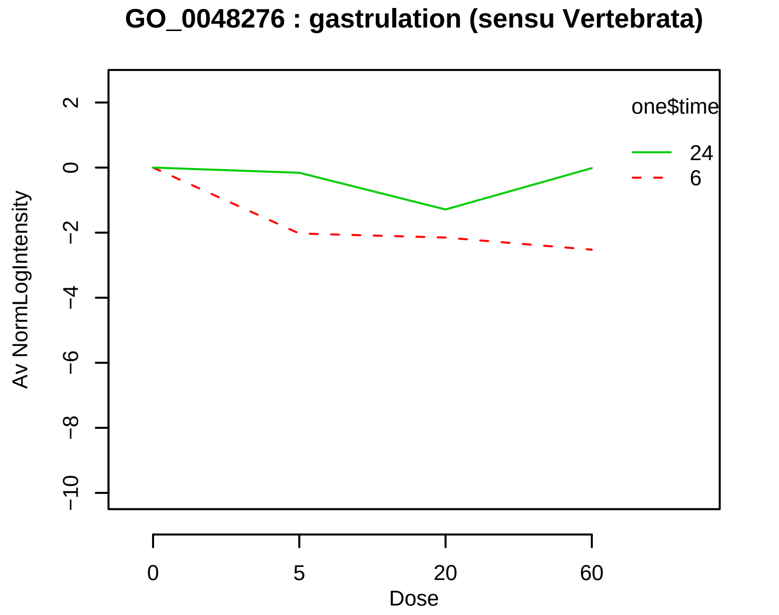
GO_0048276 : gastrulation (sensu Vertebrata)
Av NormLogIntensity
Dose
one$time
24
6
2
0
−2
−4
−6
−8
−10
0
5
20
60

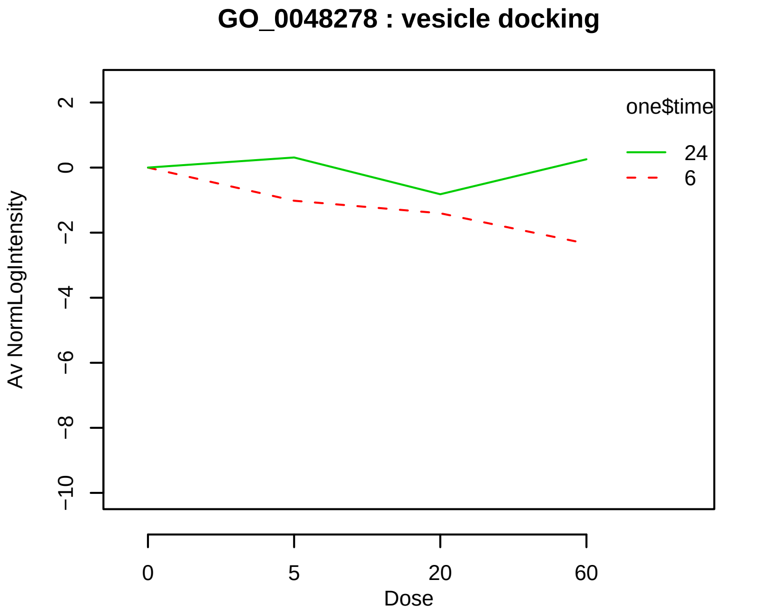
GO_0048278 : vesicle docking
Av NormLogIntensity
Dose
one$time
24
6
2
0
−2
−4
−6
−8
−10
0
5
20
60

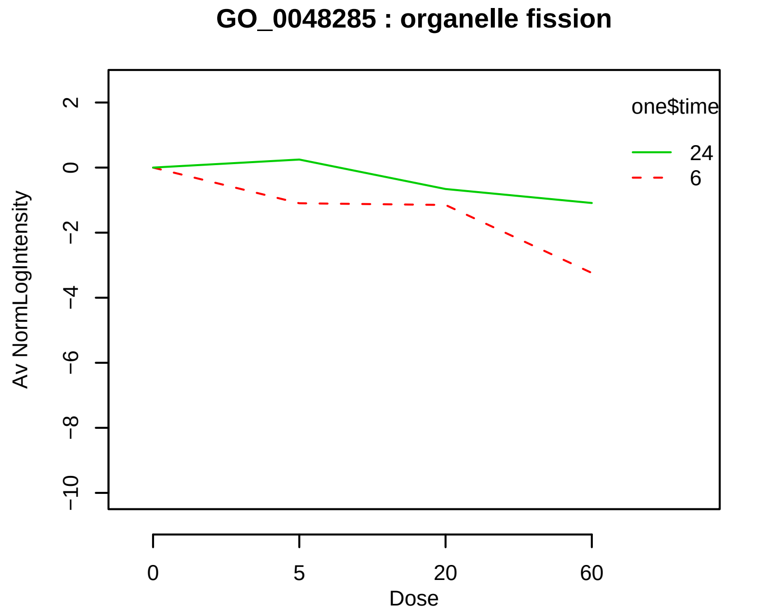
GO_0048285 : organelle fission
Av NormLogIntensity
Dose
one$time
24
6
2
0
−2
−4
−6
−8
−10
0
5
20
60

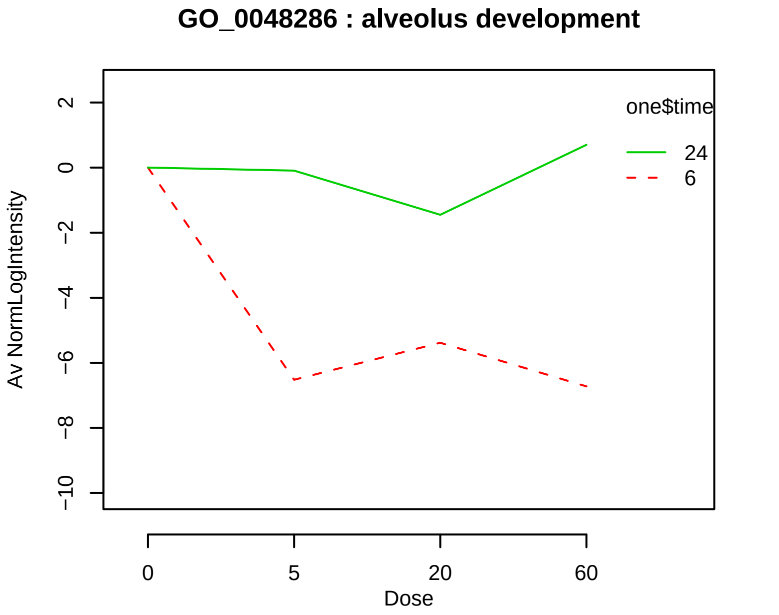
GO_0048286 : alveolus development
Av NormLogIntensity
Dose
one$time
24
6
2
0
−2
−4
−6
−8
−10
0
5
20
60

GO_0048291 : isotype switching to IgG isotypes

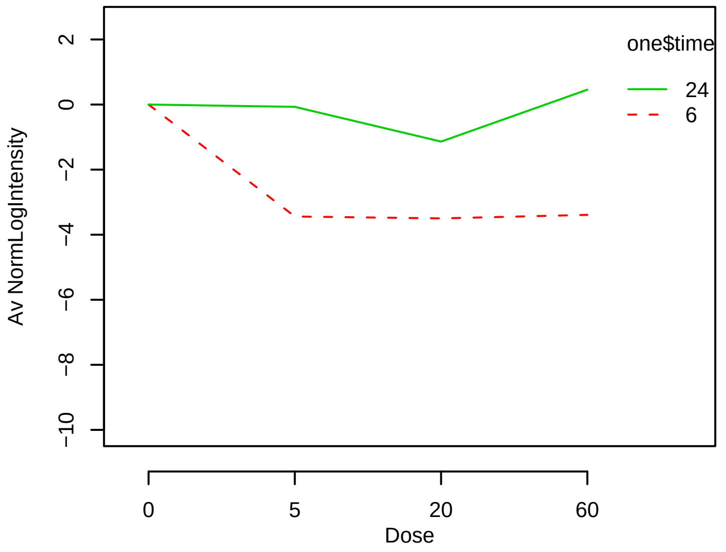

GO_0048302 : regulation of isotype switching to IgG isotyp

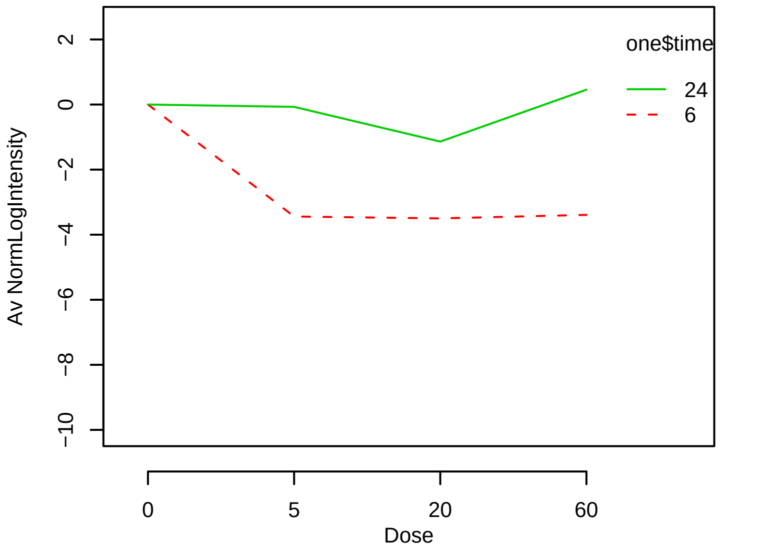

GO_0048304 : positive regulation of isotype switching to Ig(

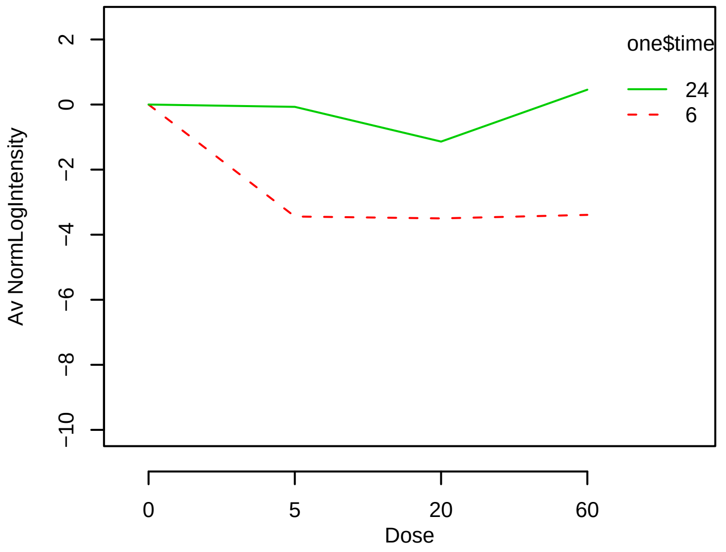

GO_0048305 : immunoglobulin secretion

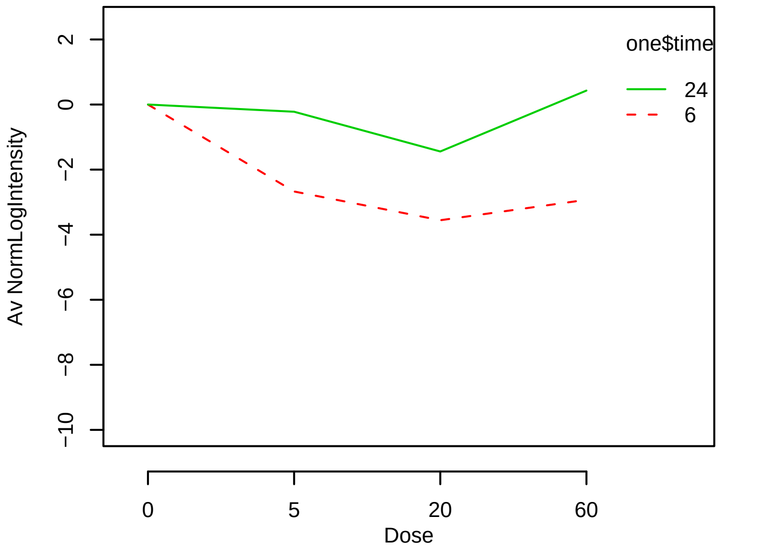

GO_0048332 : mesoderm morphogenesis

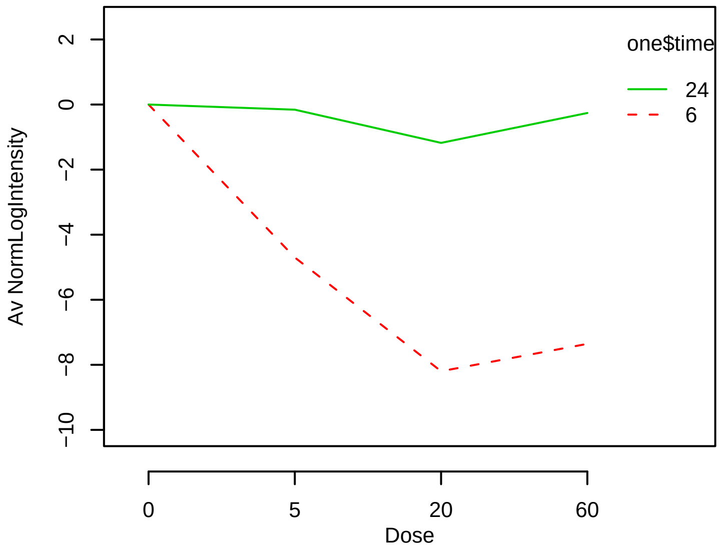

GO_0048339 : paraxial mesoderm development

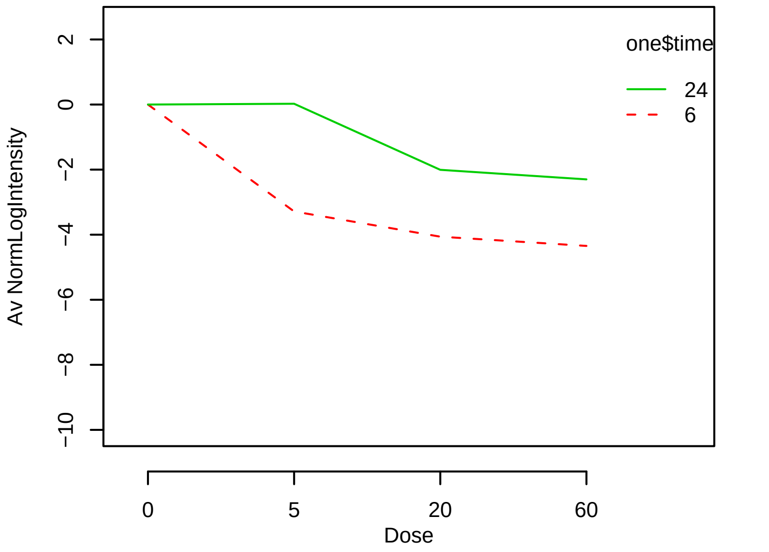

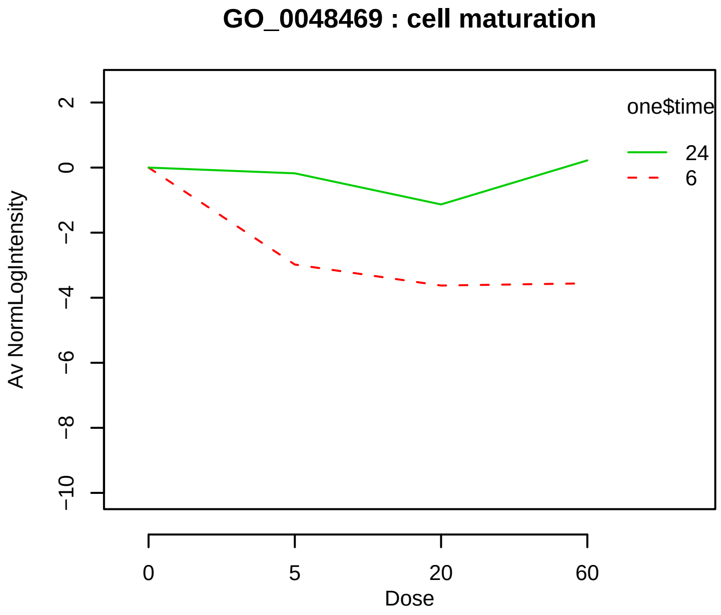
GO_0048469 : cell maturation
one$time
24
6
Av NormLogIntensity
Dose
0
5
20
60
2
0
−2
−4
−6
−8
−10

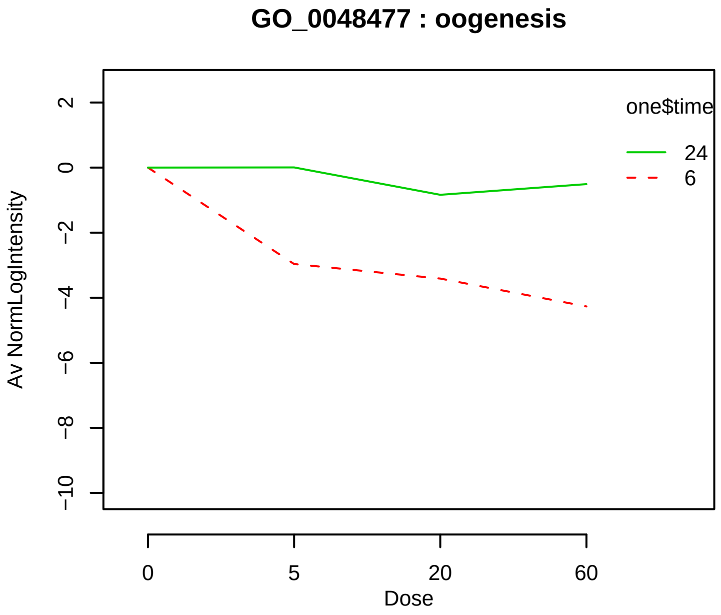
GO_0048477 : oogenesis
one$time
24
6
Av NormLogIntensity
Dose
0
5
20
60
2
0
−2
−4
−6
−8
−10

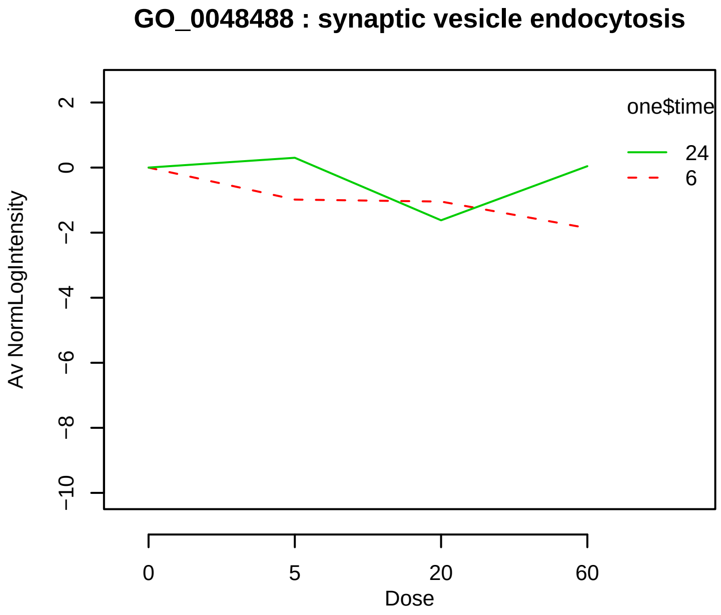
GO_0048488 : synaptic vesicle endocytosis
one$time
24
6
Av NormLogIntensity
Dose
0
5
20
60
2
0
−2
−4
−6
−8
−10

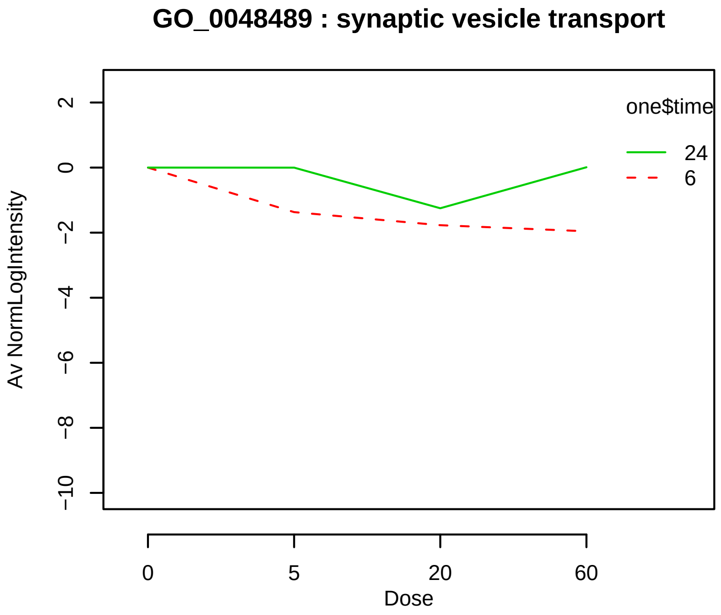
GO_0048489 : synaptic vesicle transport
one$time
24
6
Av NormLogIntensity
Dose
0
5
20
60
2
0
−2
−4
−6
−8
−10

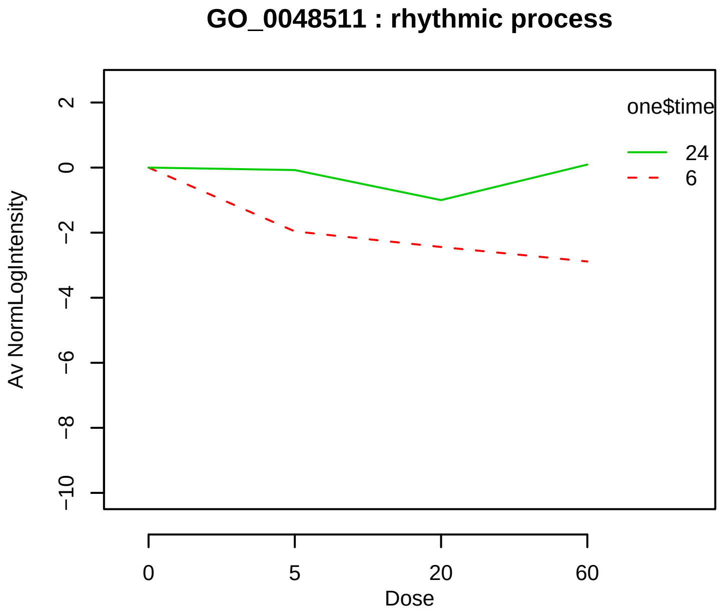
GO_0048511 : rhythmic process
one$time
24
6
Av NormLogIntensity
Dose
0
5
20
60
2
0
−2
−4
−6
−8
−10

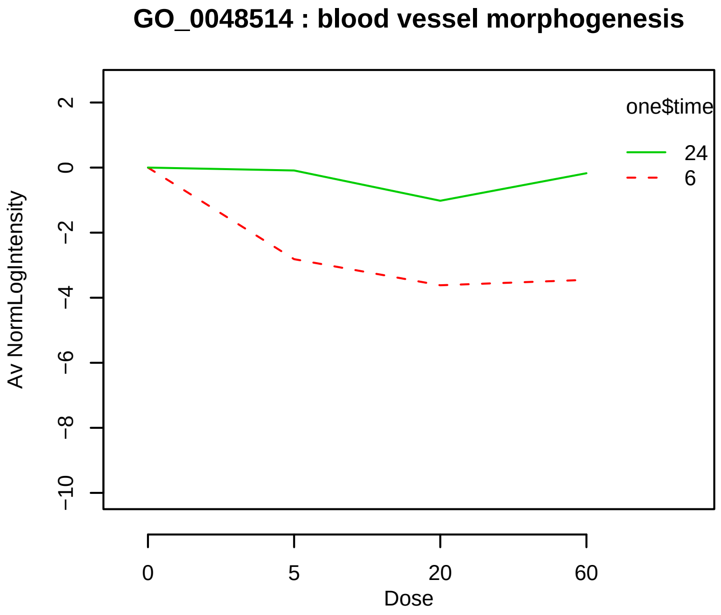
GO_0048514 : blood vessel morphogenesis
one$time
24
6
Av NormLogIntensity
Dose
0
5
20
60
2
0
−2
−4
−6
−8
−10

GO_0048515 : spermatid differentiation

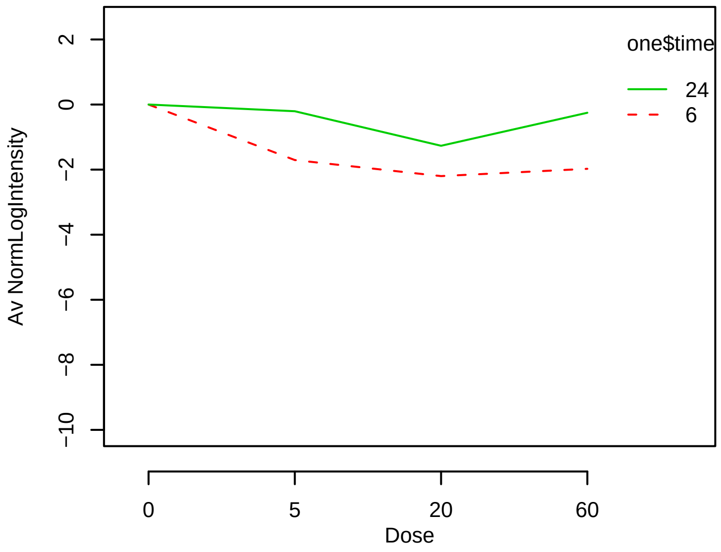

GO_0048534 : hemopoietic or lymphoid organ developmen

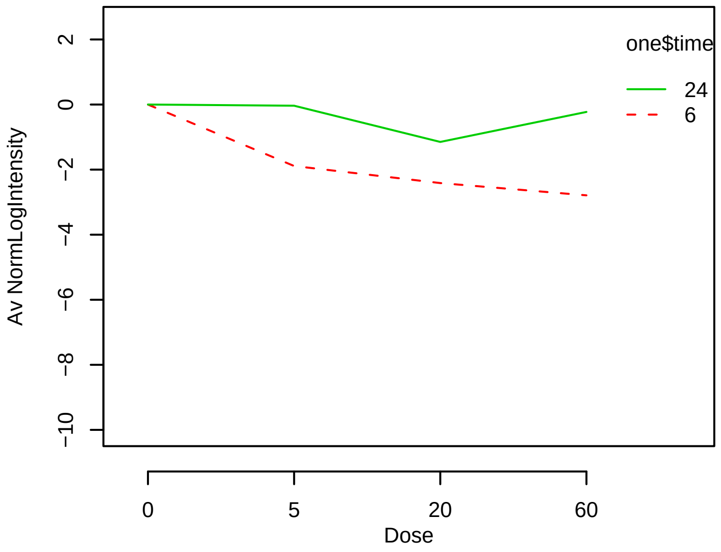

GO_0048535 : lymph node development

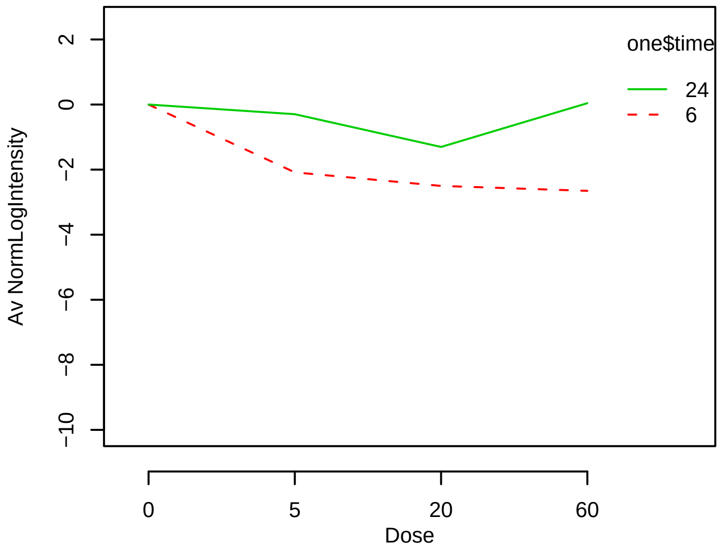

GO_0048546 : digestive tract morphogenesis

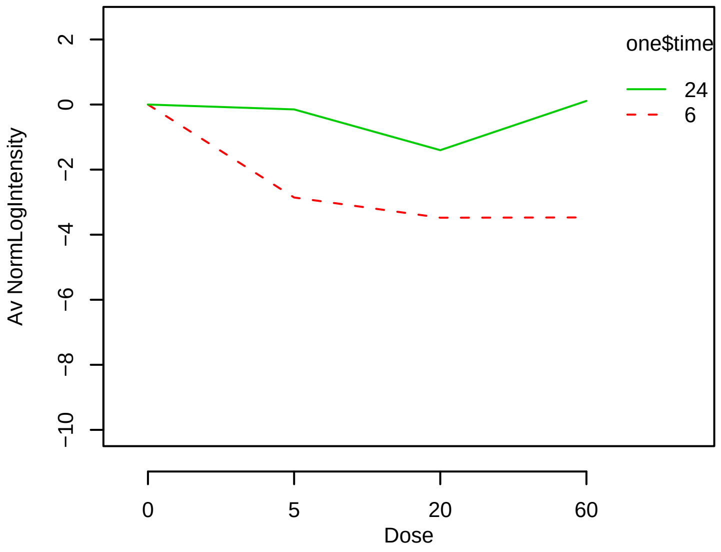

GO_0048547 : gut morphogenesis

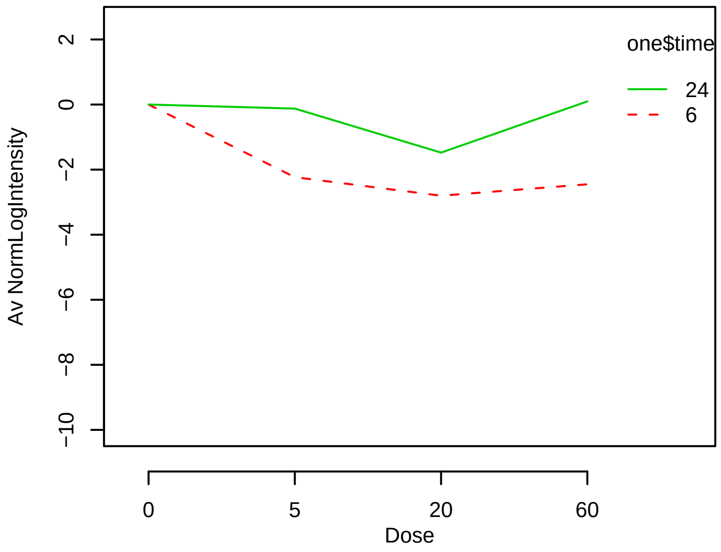

GO_0048557 : embryonic digestive tract morphogenesis

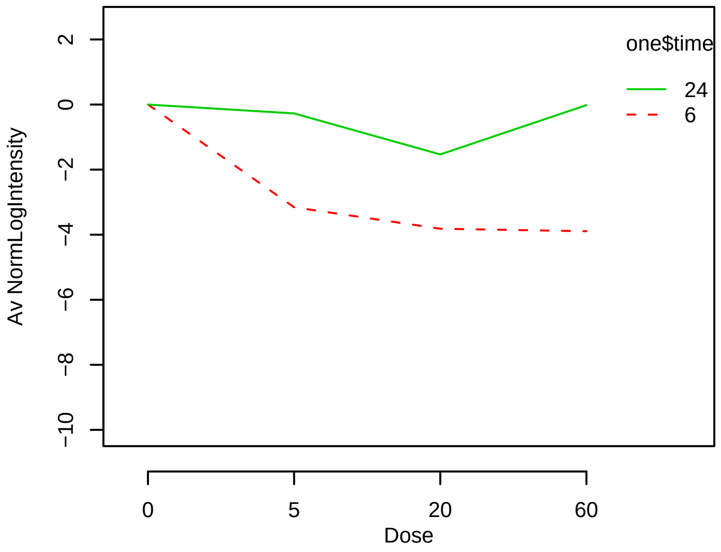

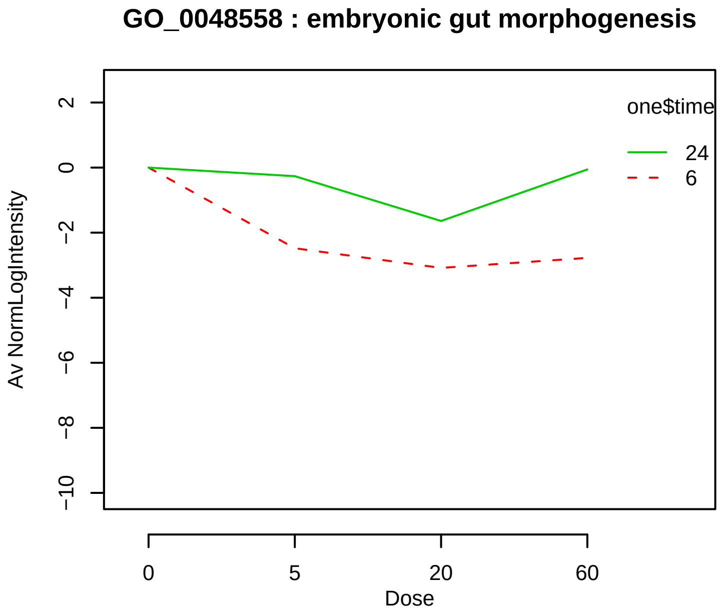
GO_0048558 : embryonic gut morphogenesis
Av NormLogIntensity
Dose
one$time
24
6

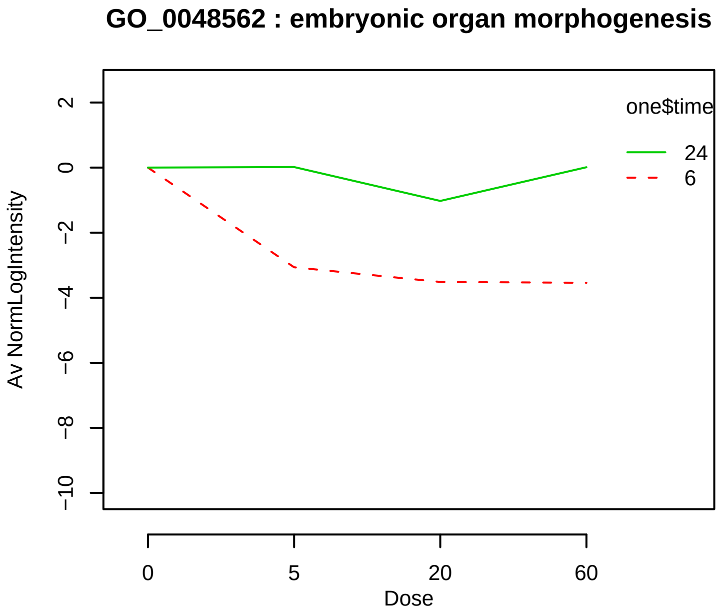
GO_0048562 : embryonic organ morphogenesis
Av NormLogIntensity
Dose
one$time
24
6

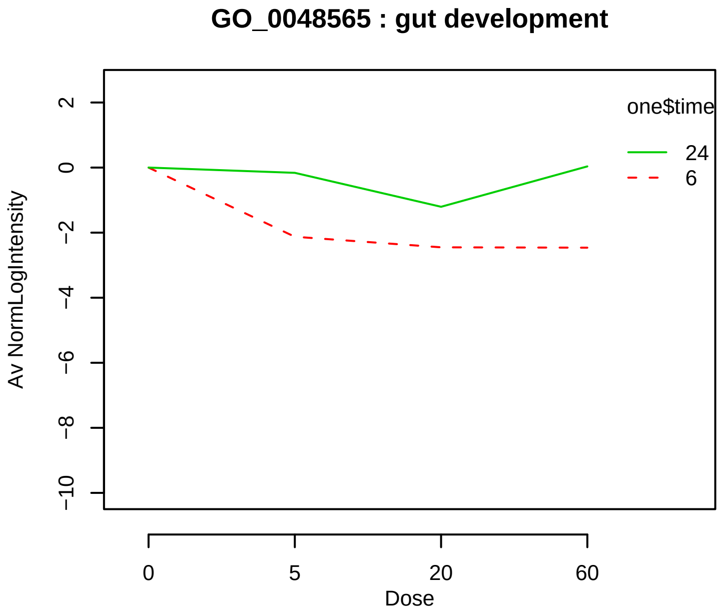
GO_0048565 : gut development
Av NormLogIntensity
Dose
one$time
24
6

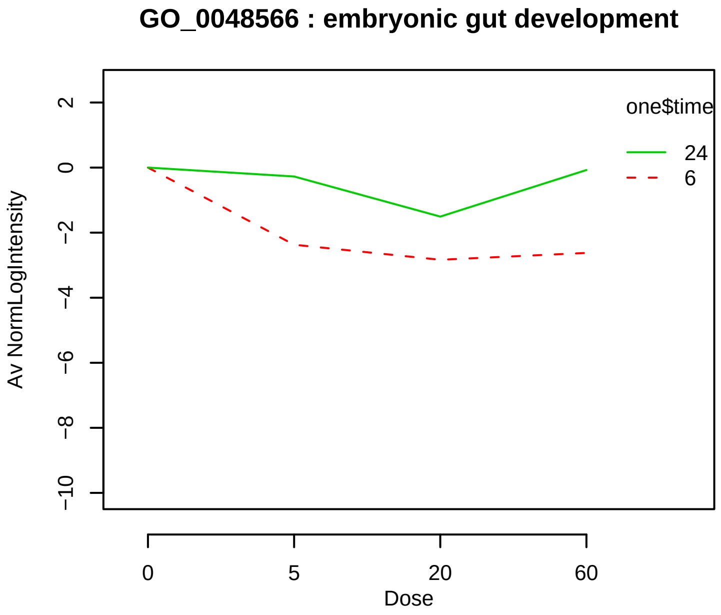
GO_0048566 : embryonic gut development
Av NormLogIntensity
Dose
one$time
24
6

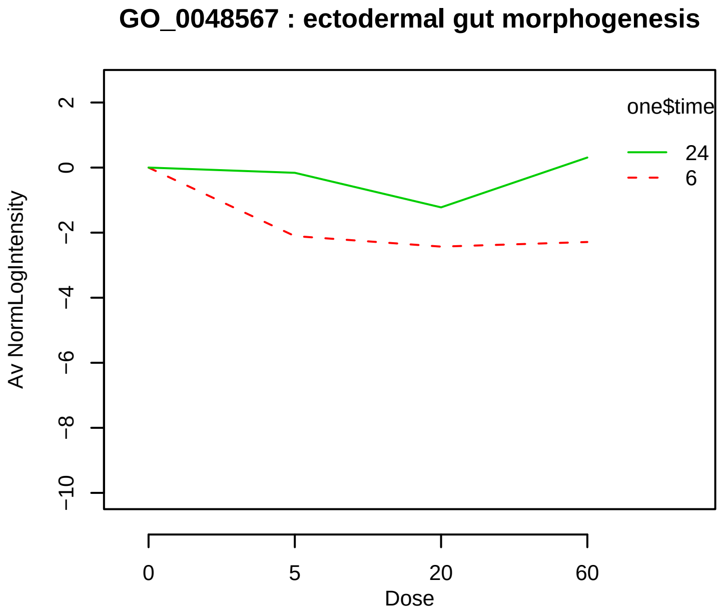
GO_0048567 : ectodermal gut morphogenesis
Av NormLogIntensity
Dose
one$time
24
6

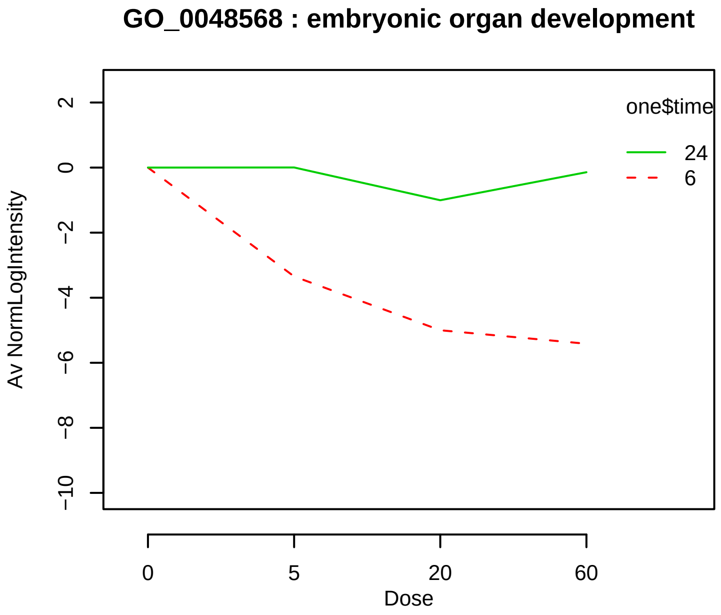
GO_0048568 : embryonic organ development
Av NormLogIntensity
Dose
one$time
24
6

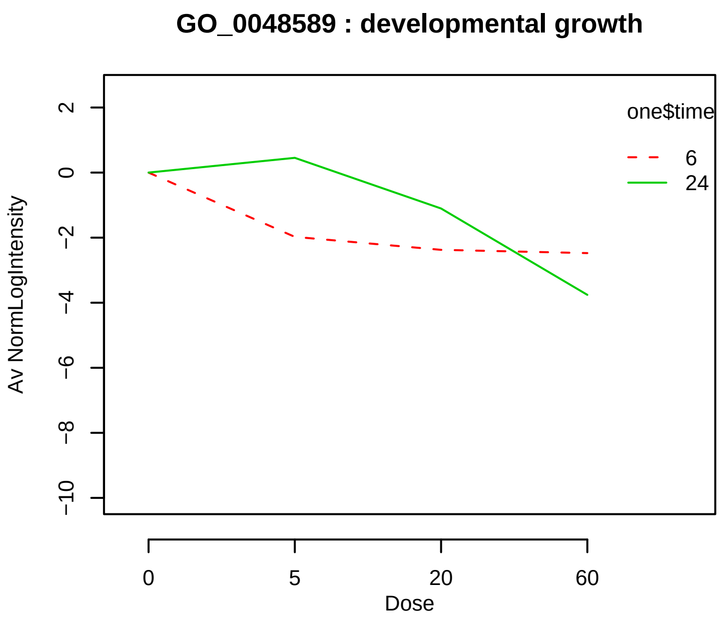
GO_0048589 : developmental growth
one$time
6
24
Av NormLogIntensity
2
0
−2
−4
−6
−8
−10
0
5
20
60
Dose

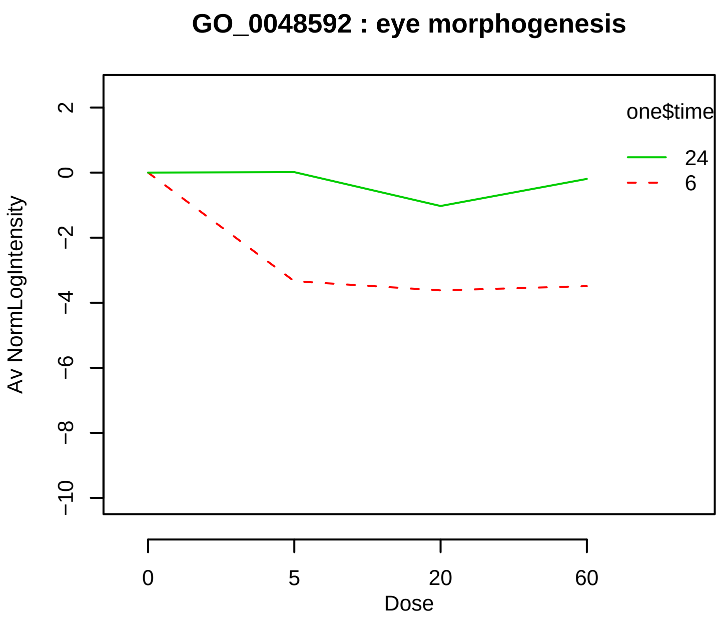
GO_0048592 : eye morphogenesis
one$time
24
6
Av NormLogIntensity
2
0
−2
−4
−6
−8
−10
0
5
20
60
Dose

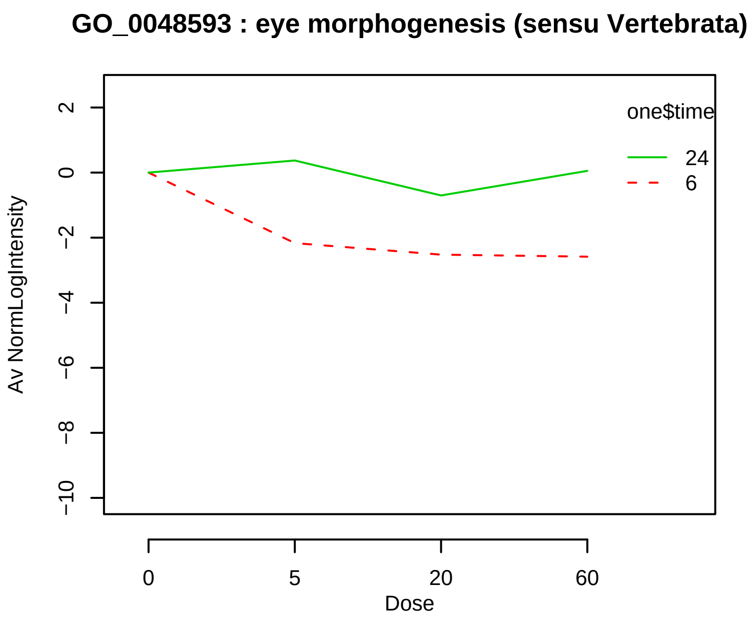
GO_0048593 : eye morphogenesis (sensu Vertebrata)
one$time
24
6
Av NormLogIntensity
2
0
−2
−4
−6
−8
−10
0
5
20
60
Dose

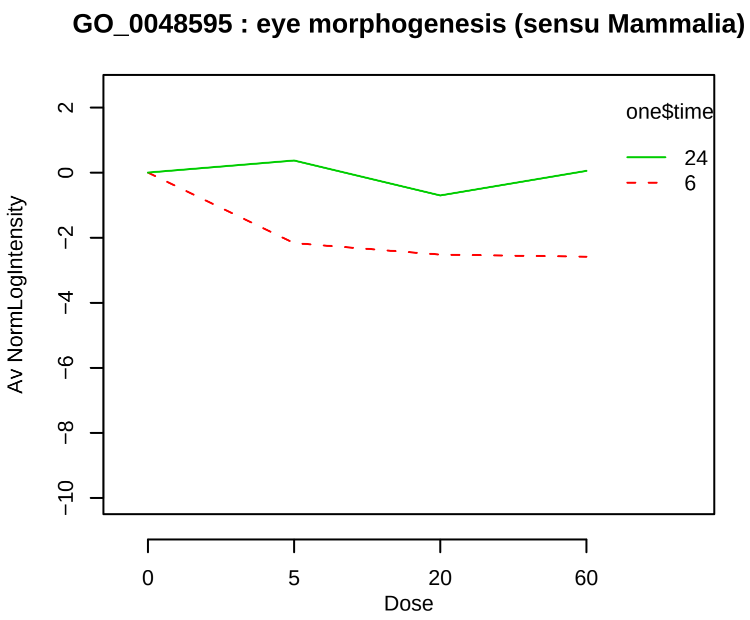
GO_0048595 : eye morphogenesis (sensu Mammalia)
one$time
24
6
Av NormLogIntensity
2
0
−2
−4
−6
−8
−10
0
5
20
60
Dose

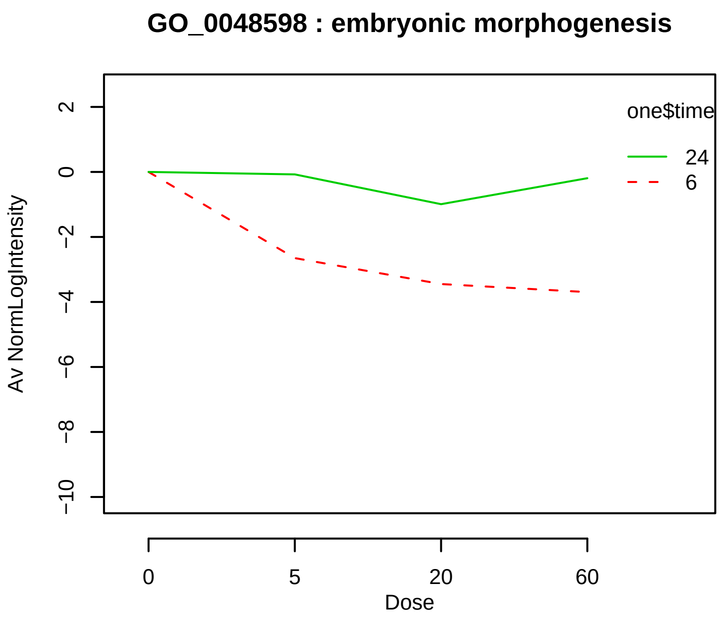
GO_0048598 : embryonic morphogenesis
one$time
24
6
Av NormLogIntensity
2
0
−2
−4
−6
−8
−10
0
5
20
60
Dose

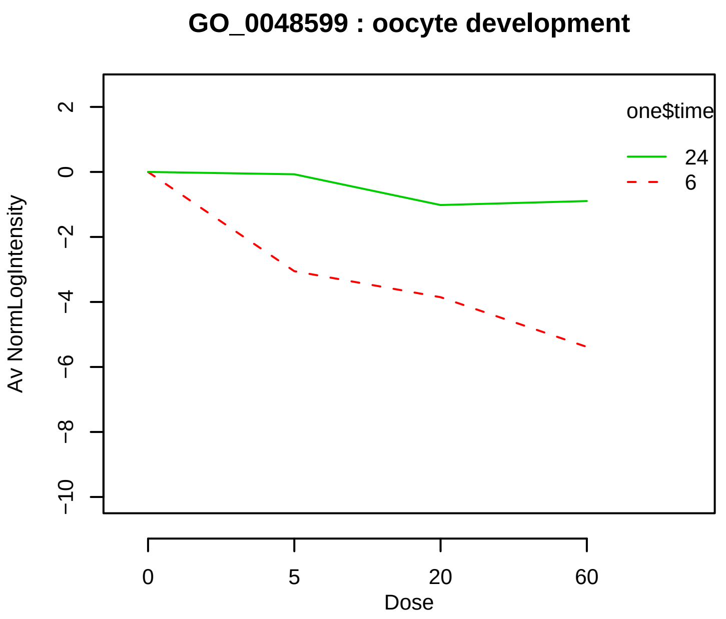
GO_0048599 : oocyte development
one$time
24
6
Av NormLogIntensity
2
0
−2
−4
−6
−8
−10
0
5
20
60
Dose

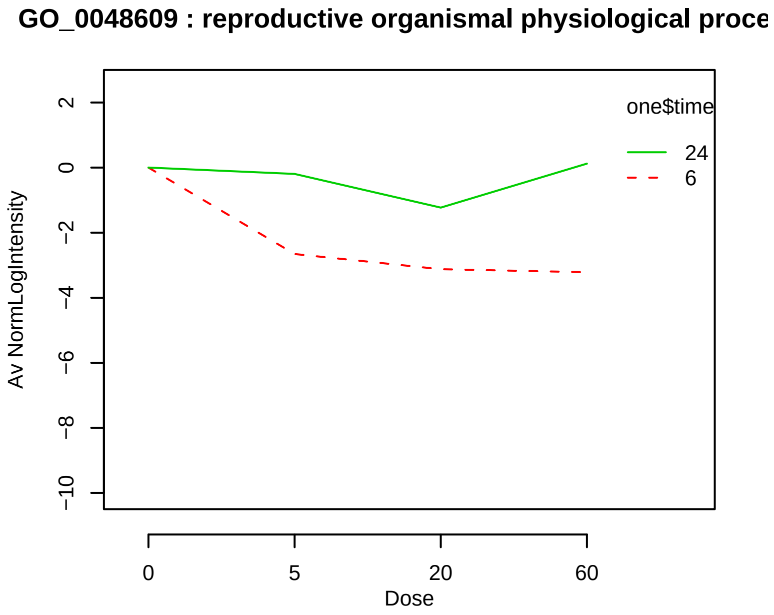
GO_0048609 : reproductive organismal physiological proce
one$time
24
6
Av NormLogIntensity
Dose
0
5
20
60

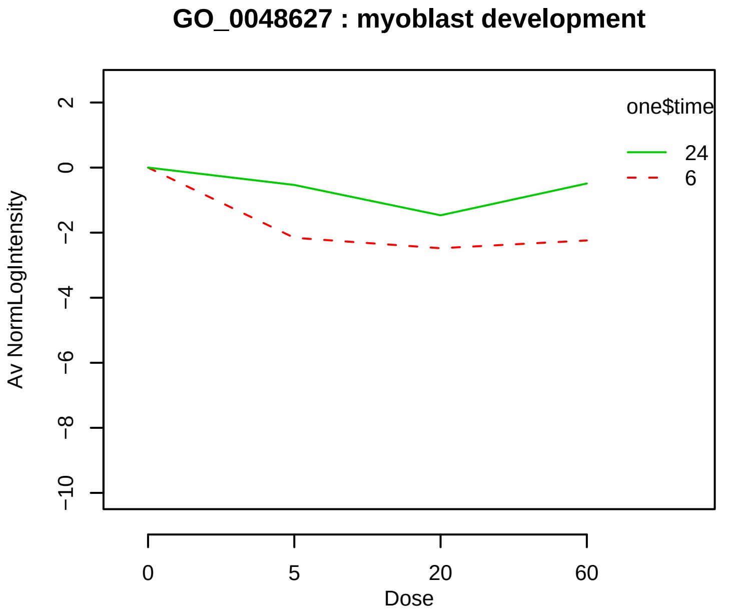
GO_0048627 : myoblast development
one$time
24
6
Av NormLogIntensity
Dose
0
5
20
60

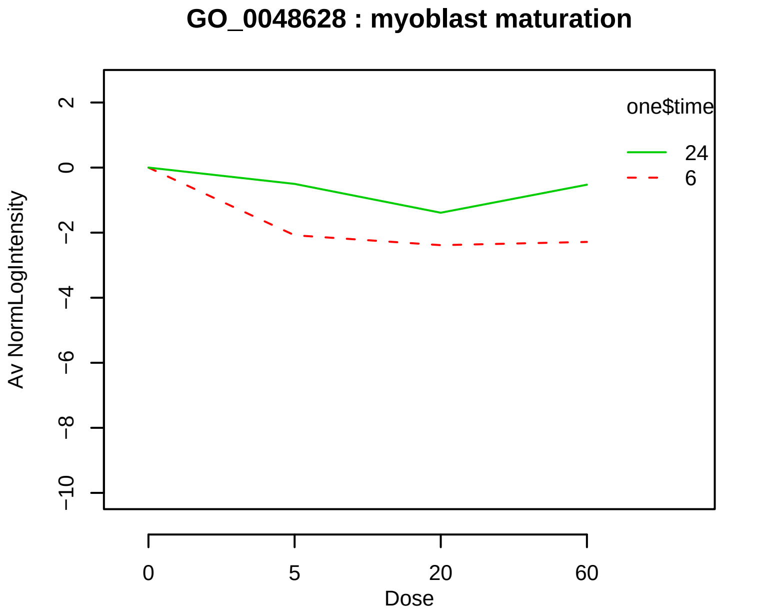
GO_0048628 : myoblast maturation
one$time
24
6
Av NormLogIntensity
Dose
0
5
20
60

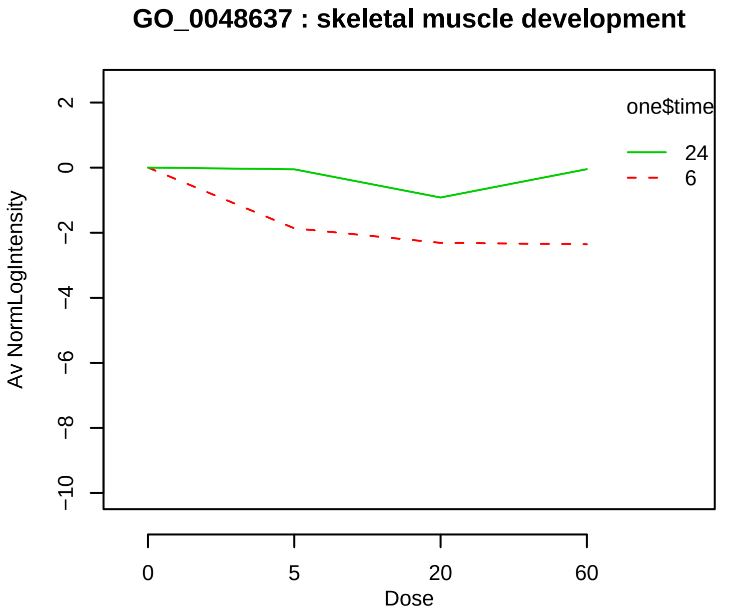
GO_0048637 : skeletal muscle development
one$time
24
6
Av NormLogIntensity
Dose
0
5
20
60

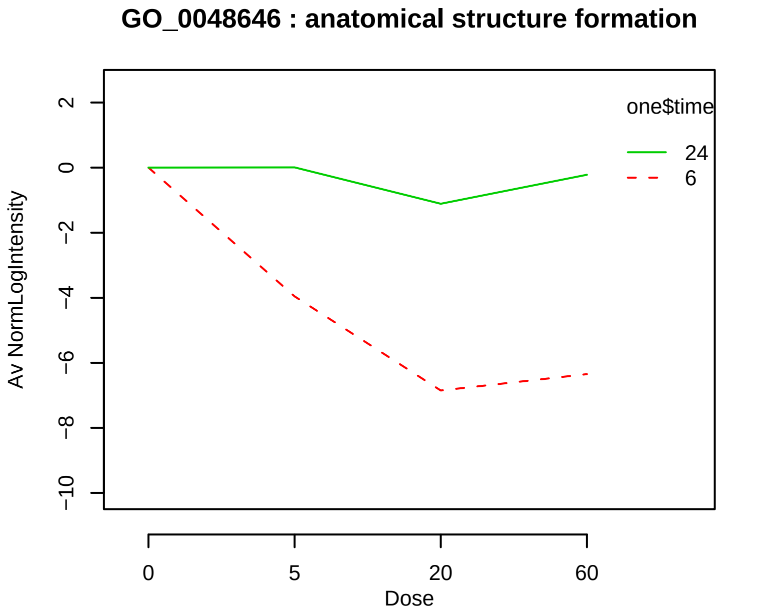
GO_0048646 : anatomical structure formation
one$time
24
6
Av NormLogIntensity
Dose
0
5
20
60

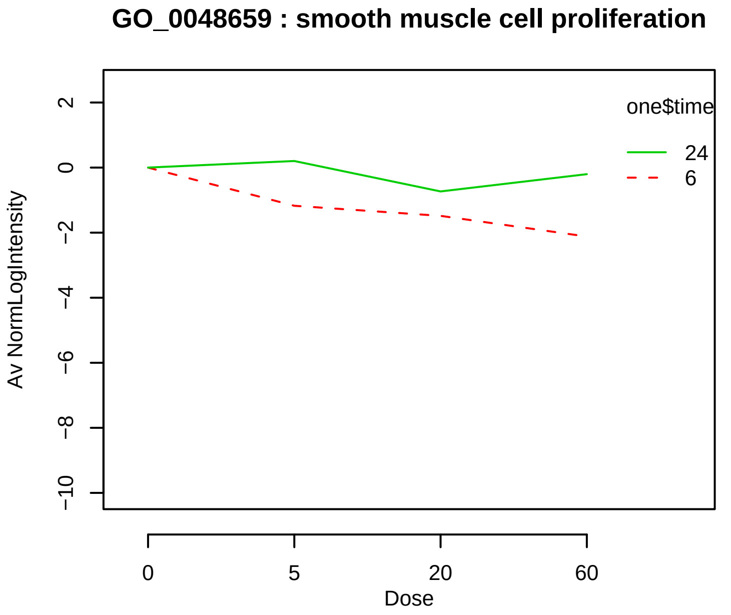
GO_0048659 : smooth muscle cell proliferation
one$time
24
6
Av NormLogIntensity
Dose
0
5
20
60

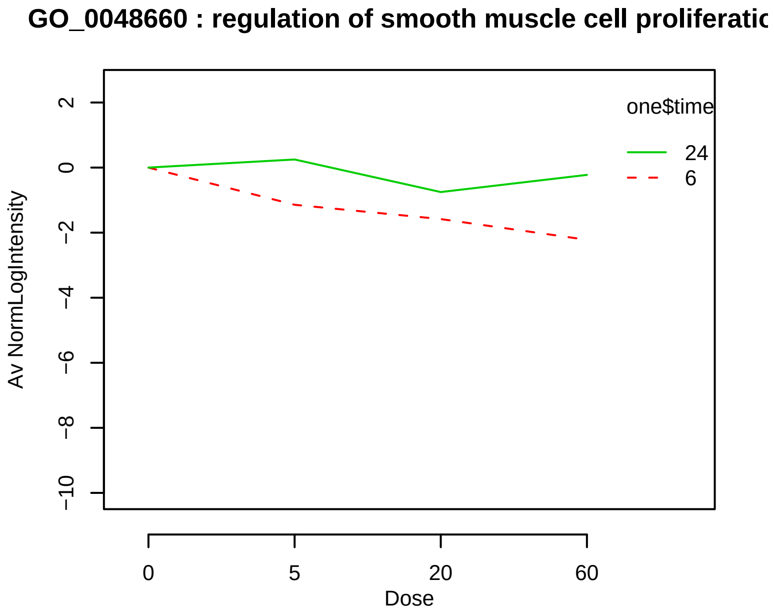
GO_0048660 : regulation of smooth muscle cell proliferatic
one$time
24
6
Av NormLogIntensity
Dose
0
5
20
60

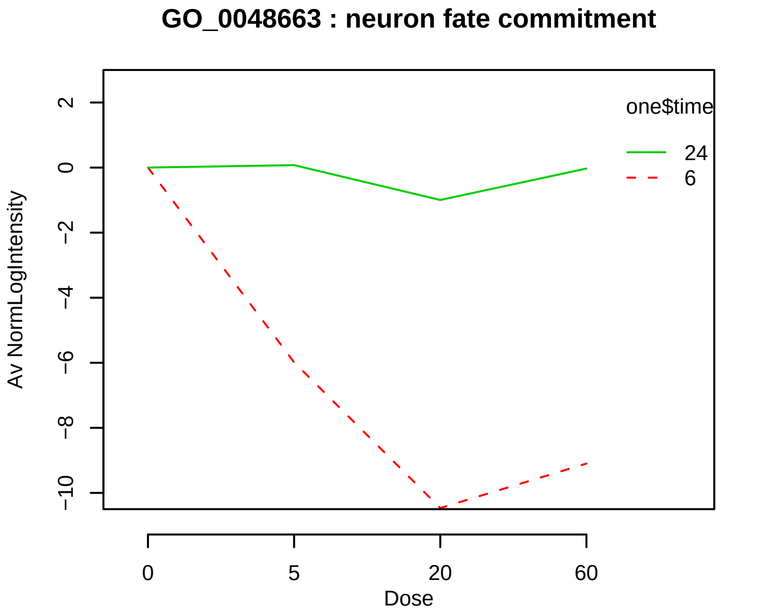
GO_0048663 : neuron fate commitment
one$time
24
6
Av NormLogIntensity
Dose
0
5
20
60

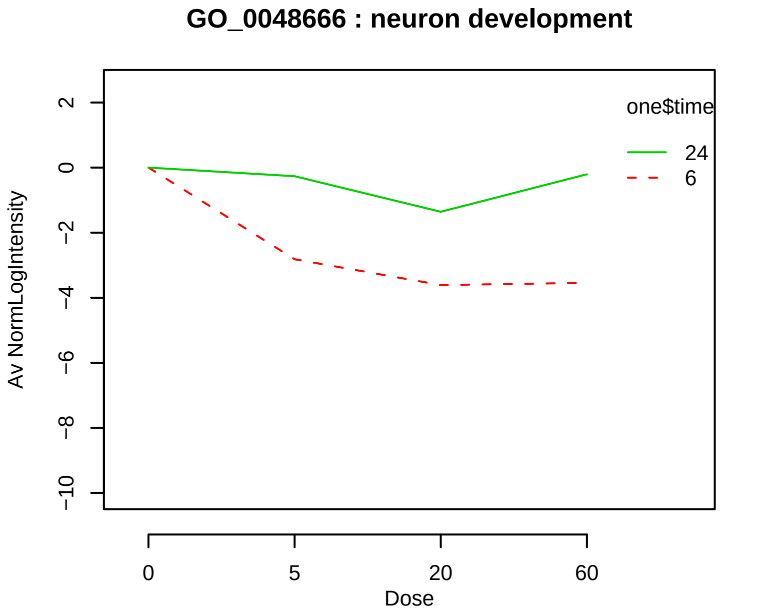
GO_0048666 : neuron development
one$time
24
6
Av NormLogIntensity
Dose
0
5
20
60

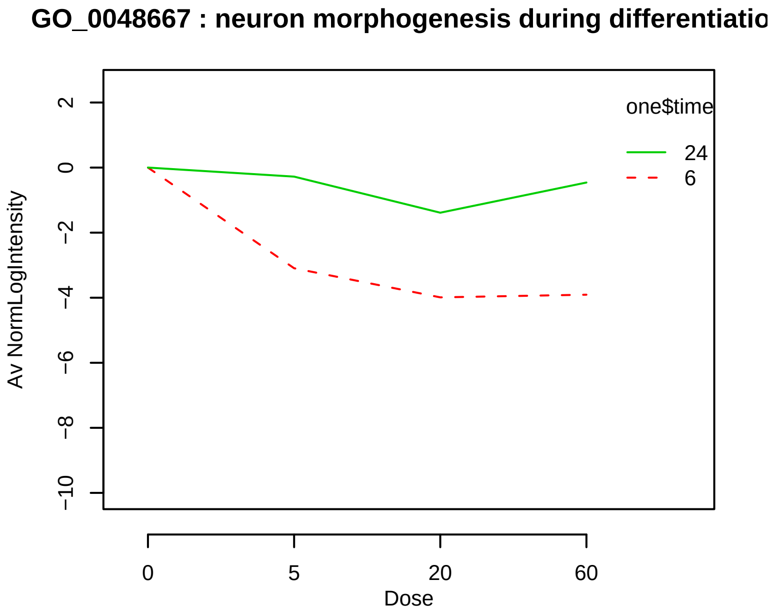
GO_0048667 : neuron morphogenesis during differentiatio
one$time
24
6
Av NormLogIntensity
Dose
0
5
20
60

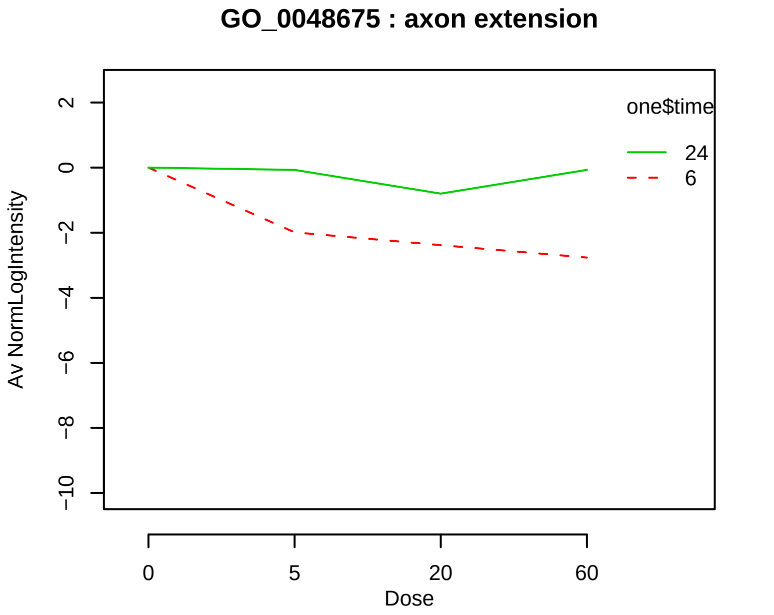
GO_0048675 : axon extension
one$time
24
6
Av NormLogIntensity
Dose
0
5
20
60

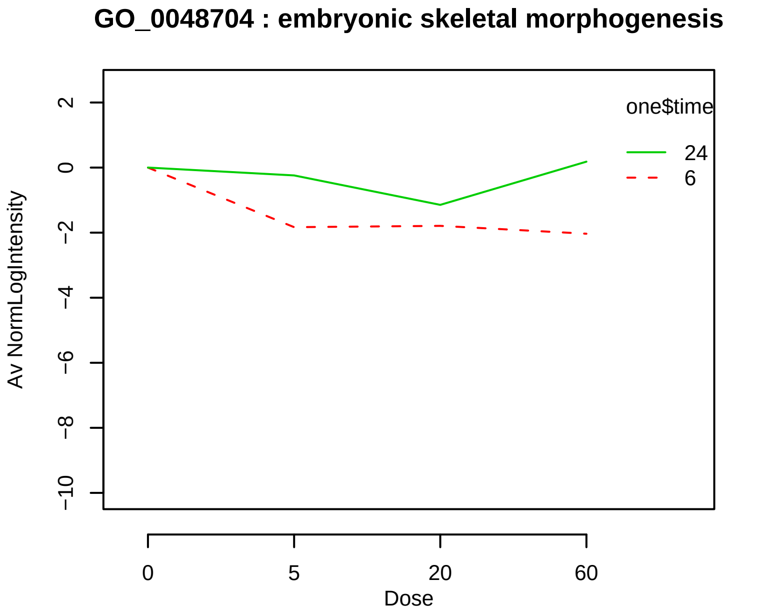
GO_0048704 : embryonic skeletal morphogenesis
one$time
24
6
Av NormLogIntensity
Dose
0
5
20
60

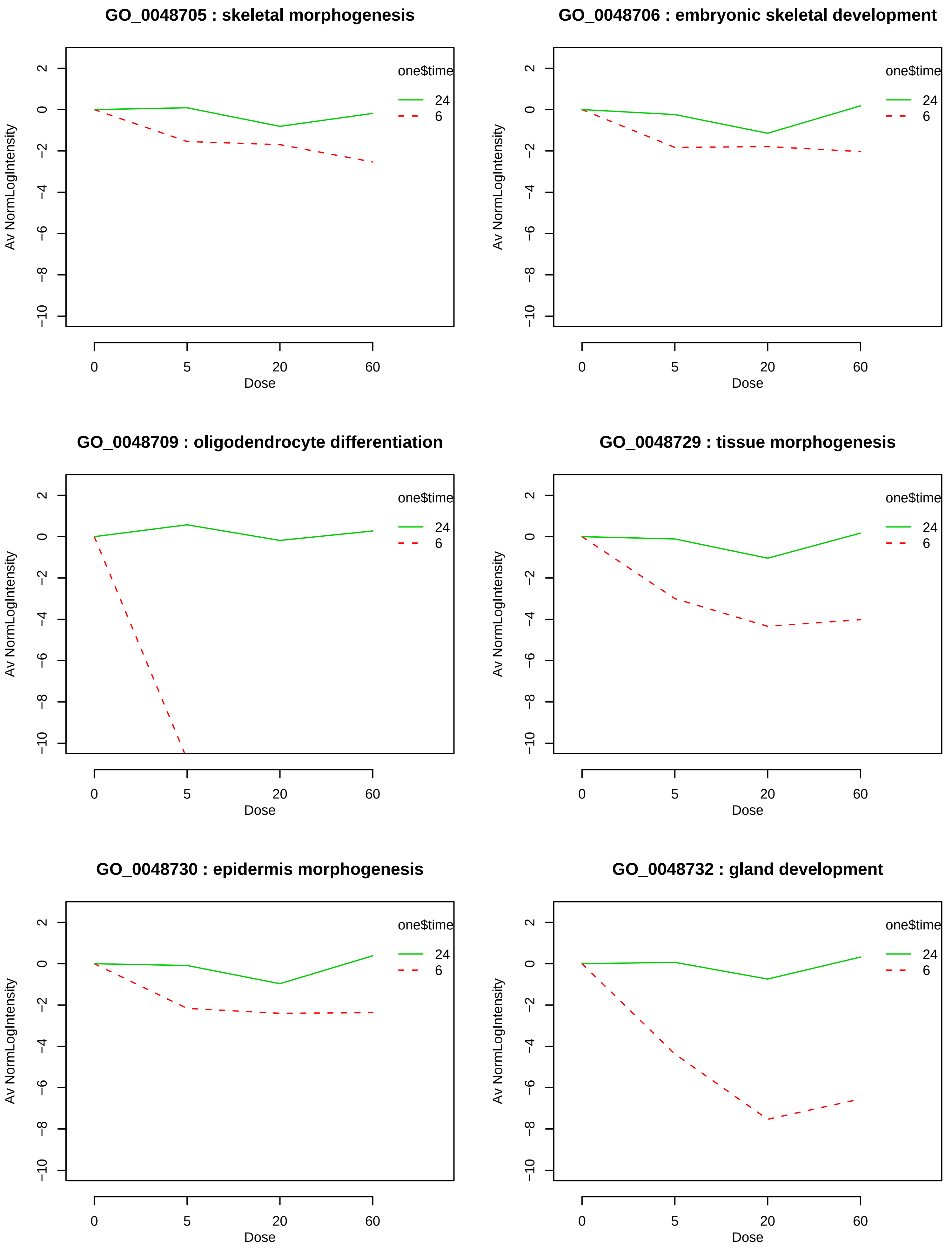
GO_0048705 : skeletal morphogenesis
GO_0048706 : embryonic skeletal development
GO_0048709 : oligodendrocyte differentiation
GO_0048729 : tissue morphogenesis
GO_0048730 : epidermis morphogenesis
GO_0048732 : gland development
Av NormLogIntensity
Dose
0
5
20
60
one$time
24
6
2
0
−2
−4
−6
−8
−10

GO_0048736 : appendage development

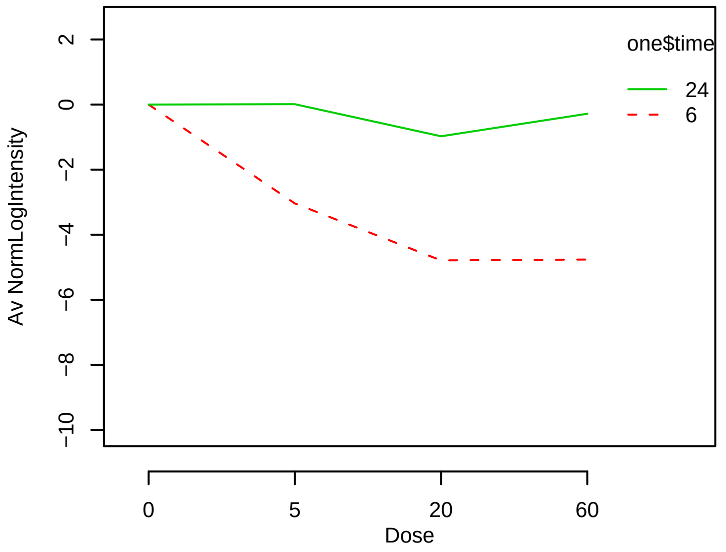

GO_0048738 : cardiac muscle development

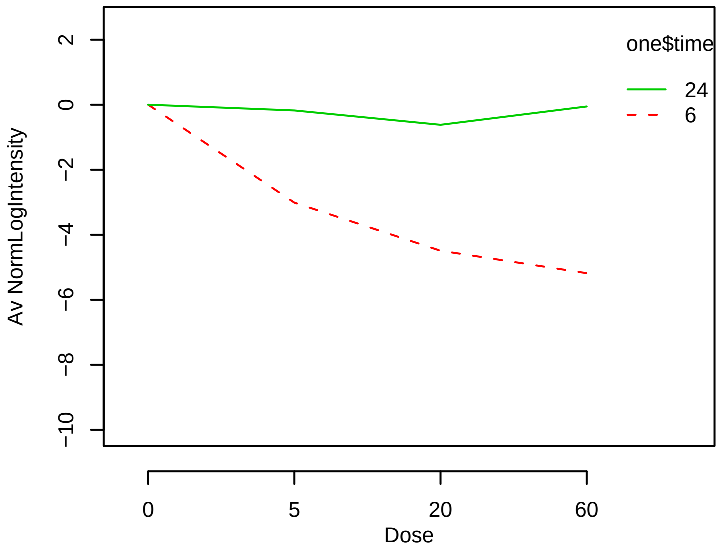

GO_0048741 : skeletal muscle fiber development

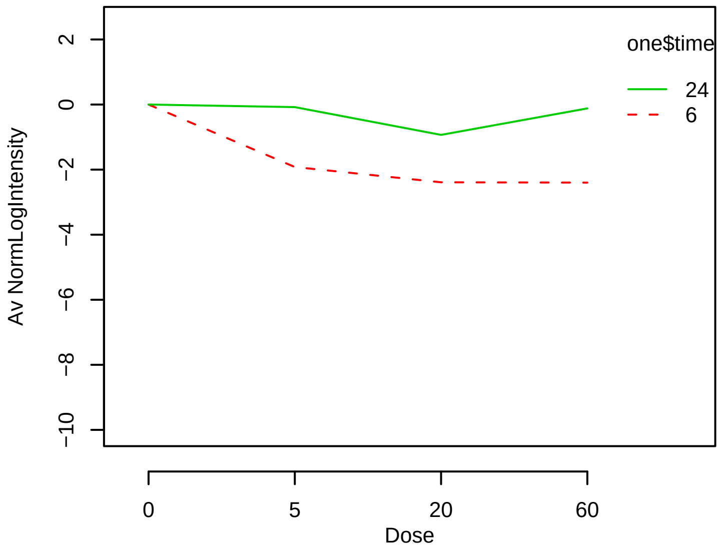

GO_0048747 : muscle fiber development

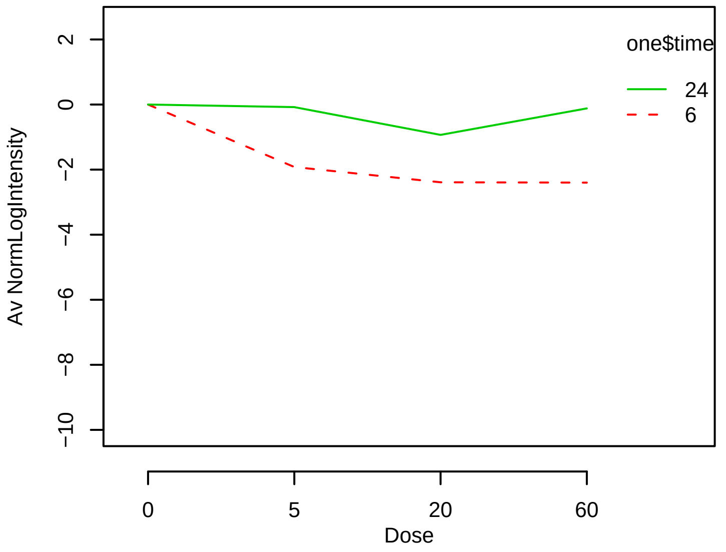

GO_0048754 : branching morphogenesis of a tube

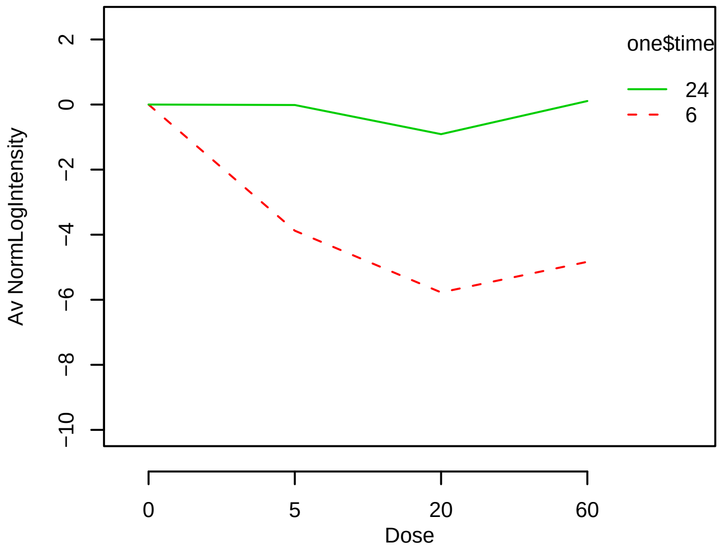

GO_0048762 : mesenchymal cell differentiation

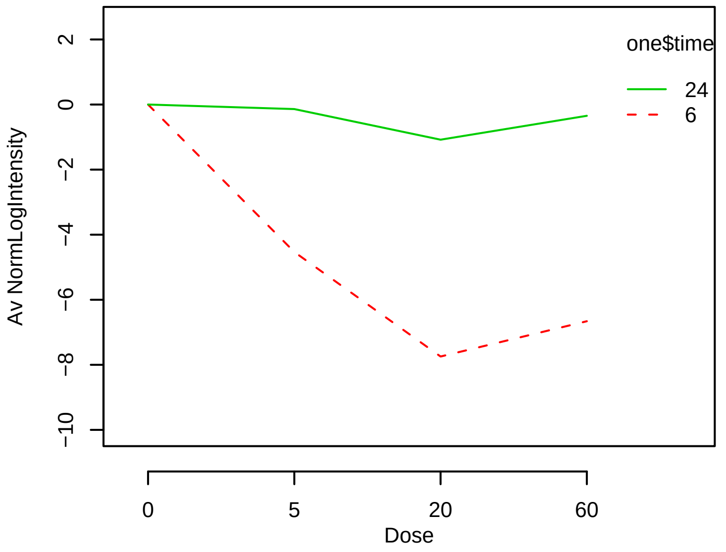

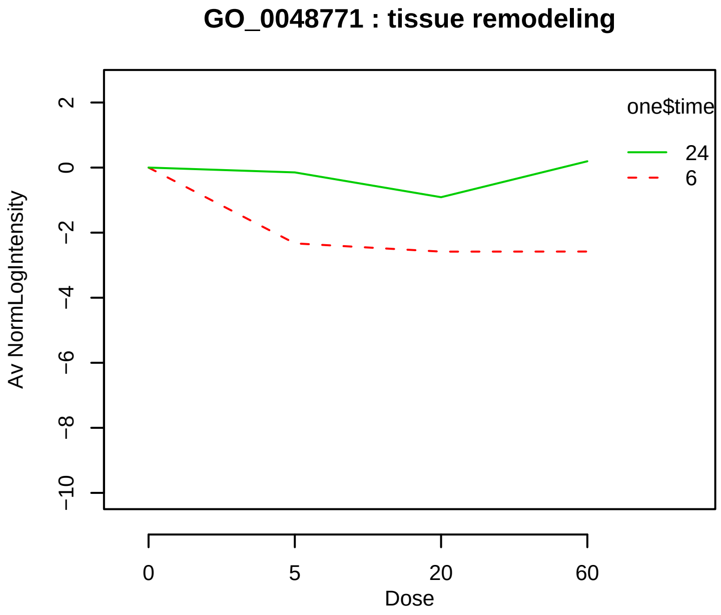
GO_0048771 : tissue remodeling
one$time
24
6
Av NormLogIntensity
Dose
0
5
20
60
2
0
−2
−4
−6
−8
−10

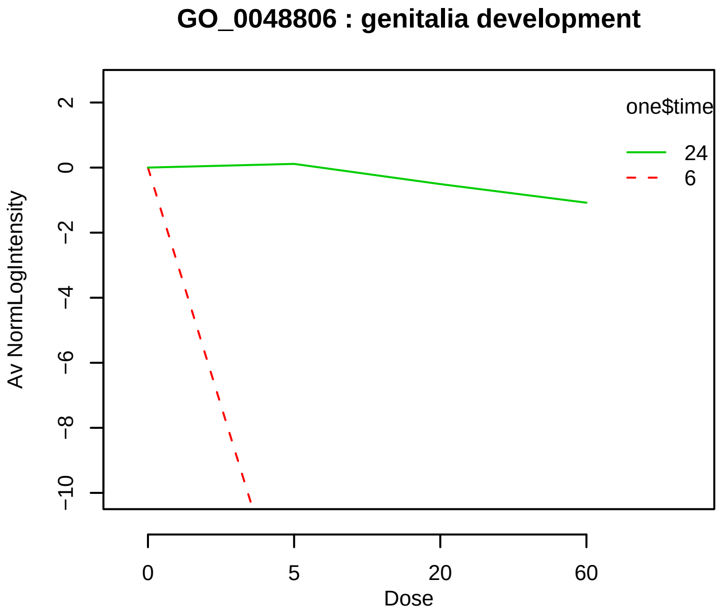
GO_0048806 : genitalia development
one$time
24
6
Av NormLogIntensity
Dose
0
5
20
60
2
0
−2
−4
−6
−8
−10

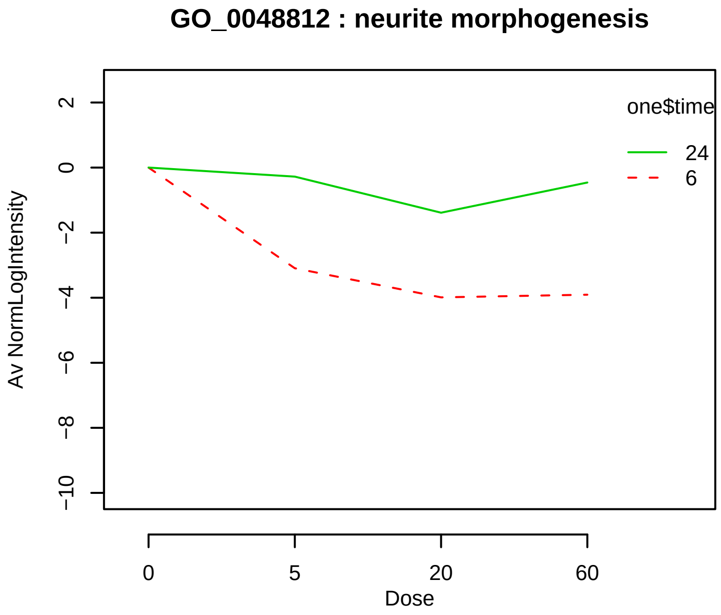
GO_0048812 : neurite morphogenesis
one$time
24
6
Av NormLogIntensity
Dose
0
5
20
60
2
0
−2
−4
−6
−8
−10

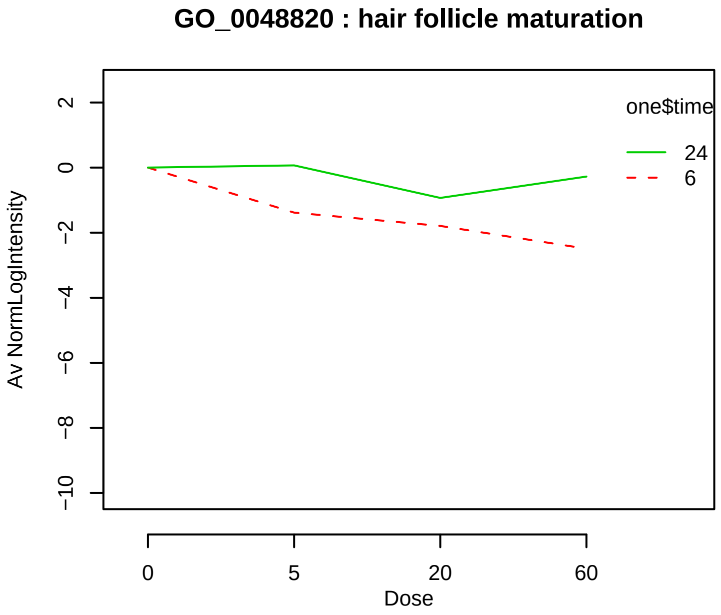
GO_0048820 : hair follicle maturation
one$time
24
6
Av NormLogIntensity
Dose
0
5
20
60
2
0
−2
−4
−6
−8
−10

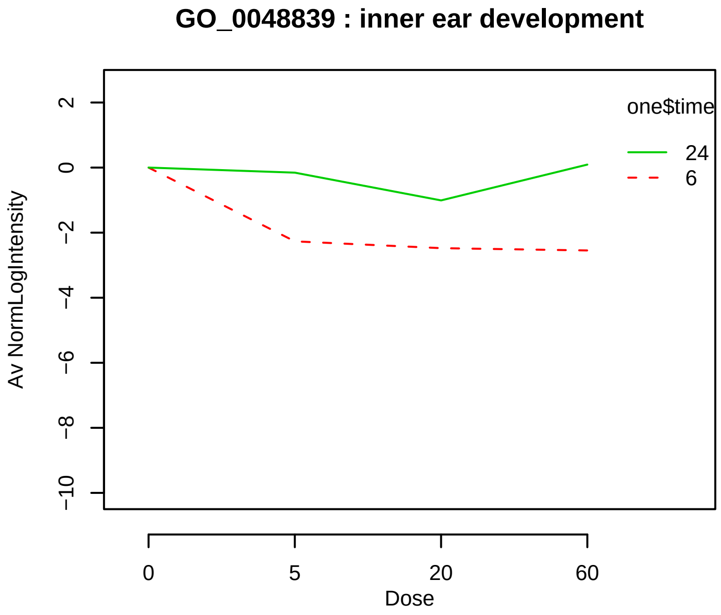
GO_0048839 : inner ear development
one$time
24
6
Av NormLogIntensity
Dose
0
5
20
60
2
0
−2
−4
−6
−8
−10

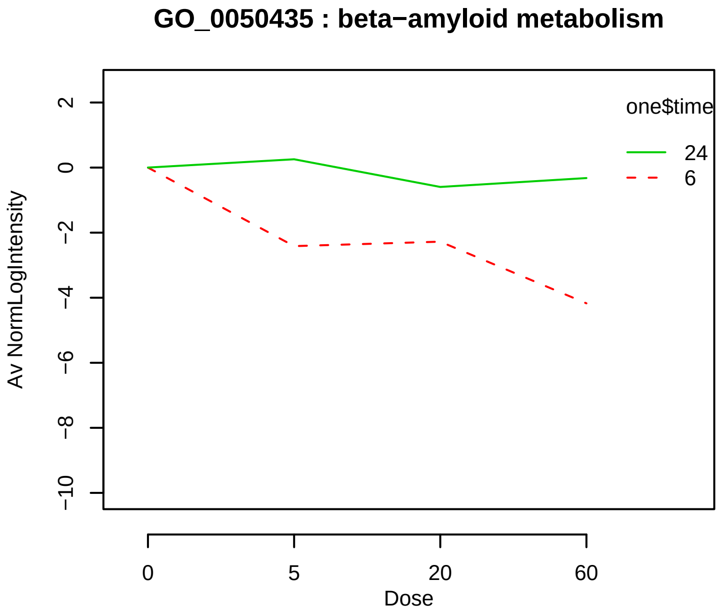
GO_0050435 : beta−amyloid metabolism
one$time
24
6
Av NormLogIntensity
Dose
0
5
20
60
2
0
−2
−4
−6
−8
−10

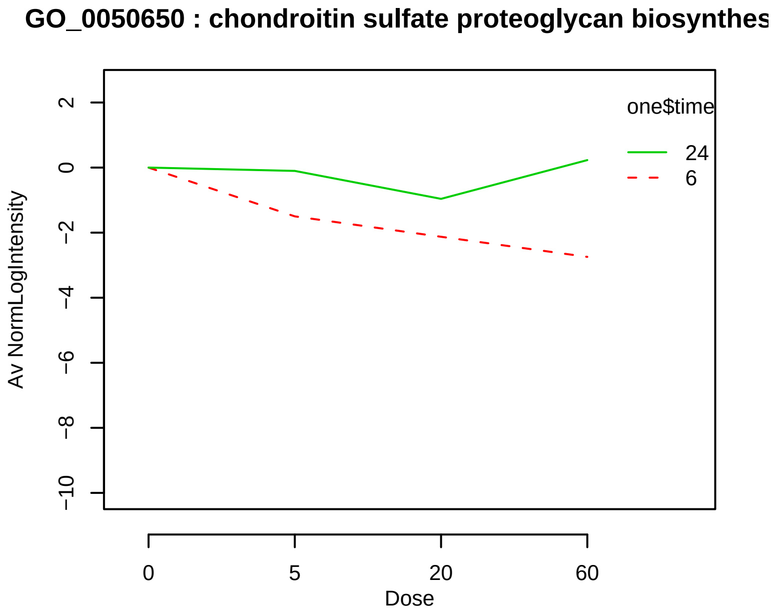
GO_0050650 : chondroitin sulfate proteoglycan biosynthes
Av NormLogIntensity
Dose
one$time
24
6

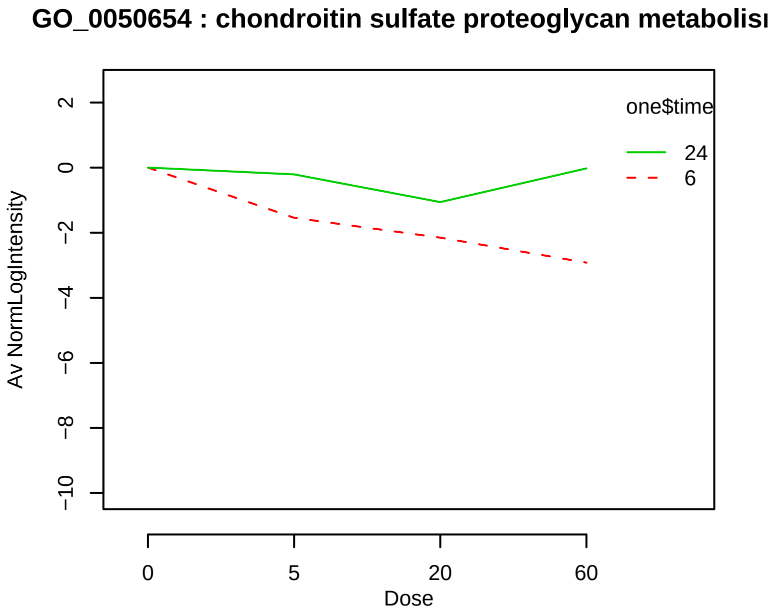
GO_0050654 : chondroitin sulfate proteoglycan metabolis
Av NormLogIntensity
Dose
one$time
24
6

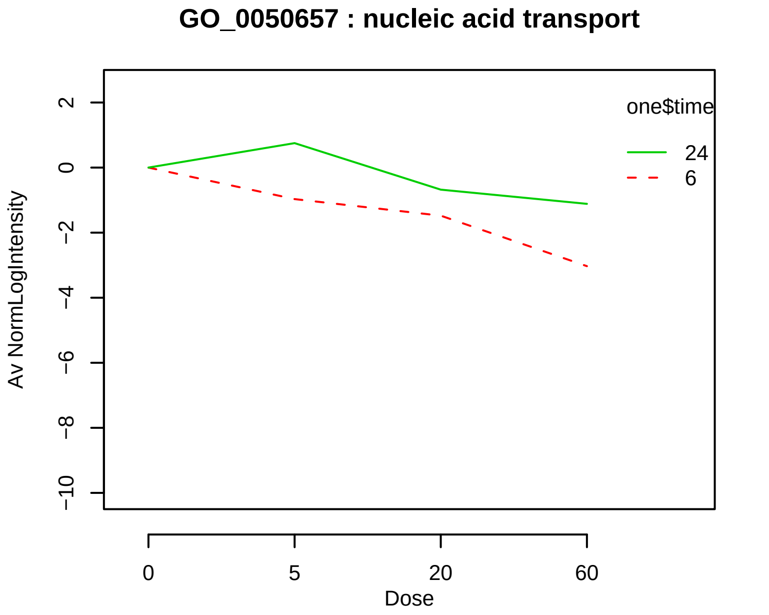
GO_0050657 : nucleic acid transport
Av NormLogIntensity
Dose
one$time
24
6

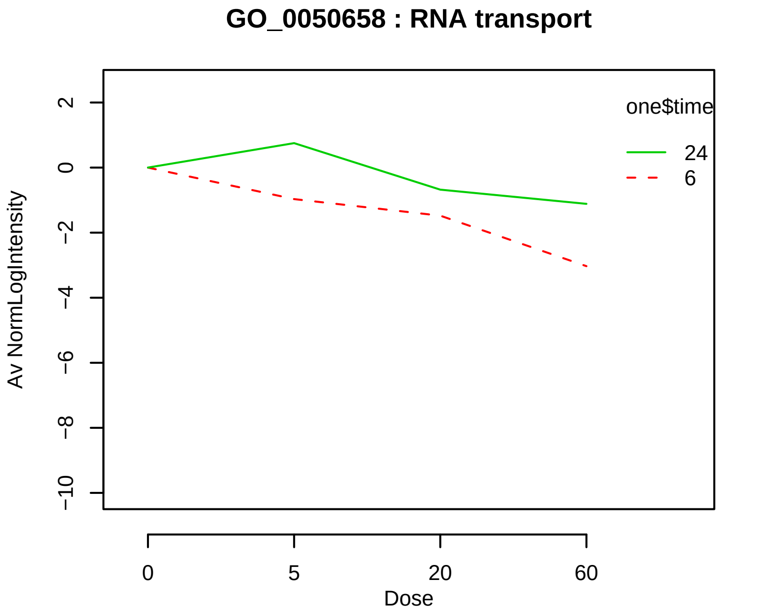
GO_0050658 : RNA transport
Av NormLogIntensity
Dose
one$time
24
6

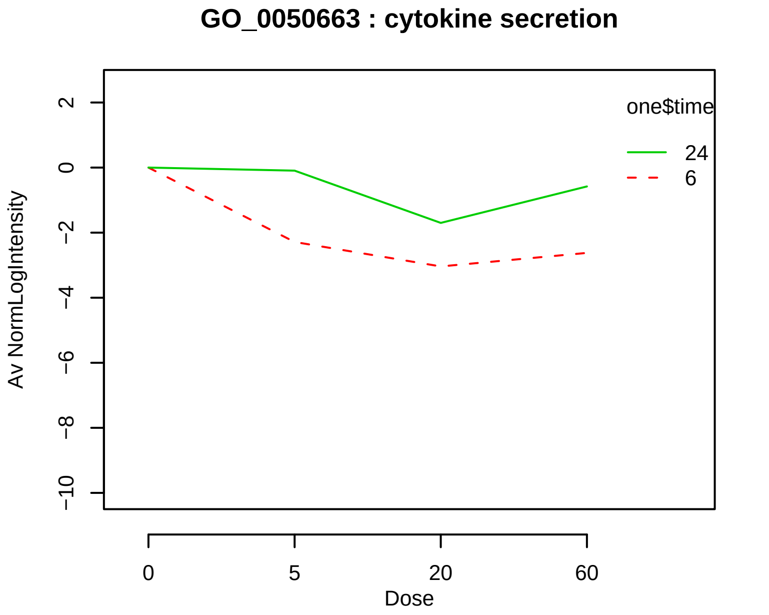
GO_0050663 : cytokine secretion
Av NormLogIntensity
Dose
one$time
24
6

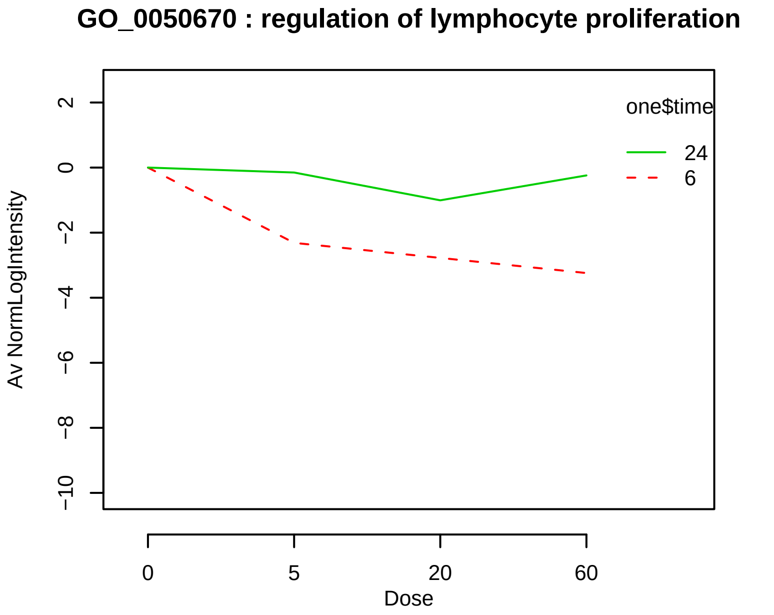
GO_0050670 : regulation of lymphocyte proliferation
Av NormLogIntensity
Dose
one$time
24
6

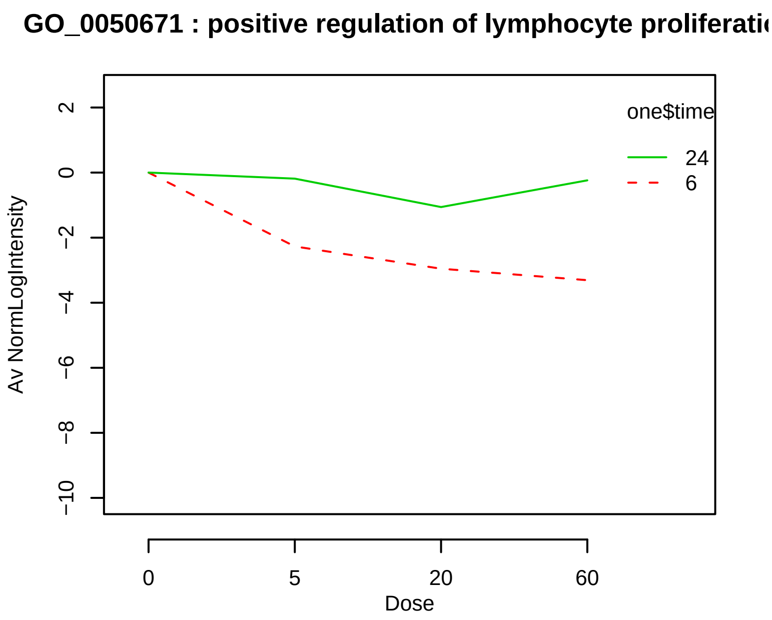
GO_0050671 : positive regulation of lymphocyte proliferati
Av NormLogIntensity
2
0
−2
−4
−6
−8
−10
0
5
20
60
Dose
one$time
24
6

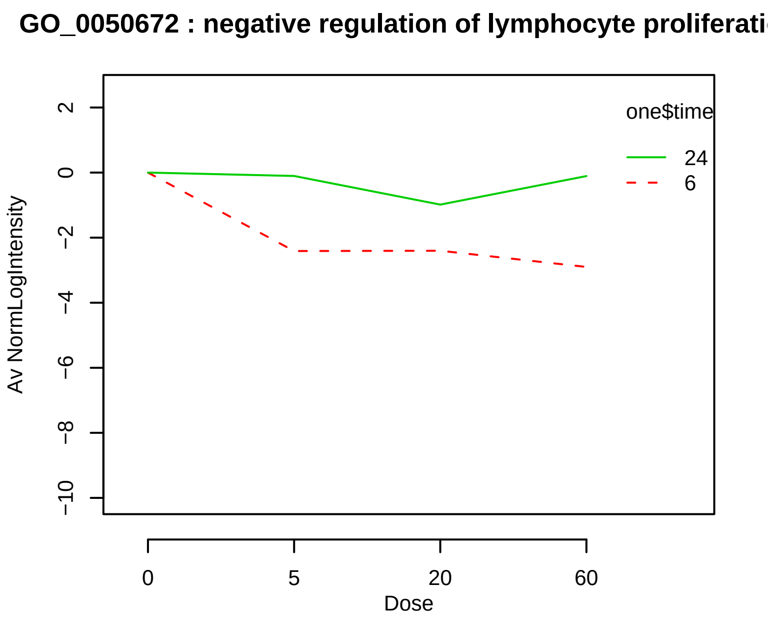
GO_0050672 : negative regulation of lymphocyte proliferati
Av NormLogIntensity
2
0
−2
−4
−6
−8
−10
0
5
20
60
Dose
one$time
24
6

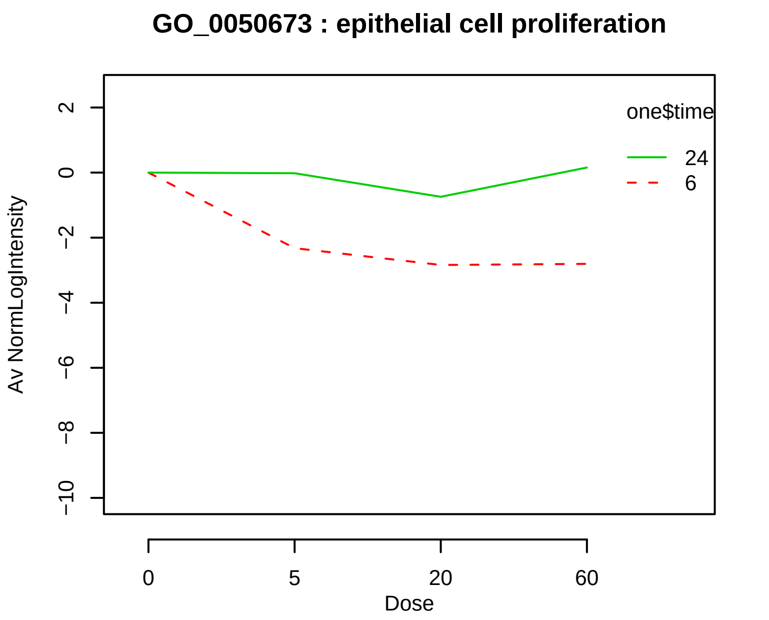
GO_0050673 : epithelial cell proliferation
Av NormLogIntensity
2
0
−2
−4
−6
−8
−10
0
5
20
60
Dose
one$time
24
6

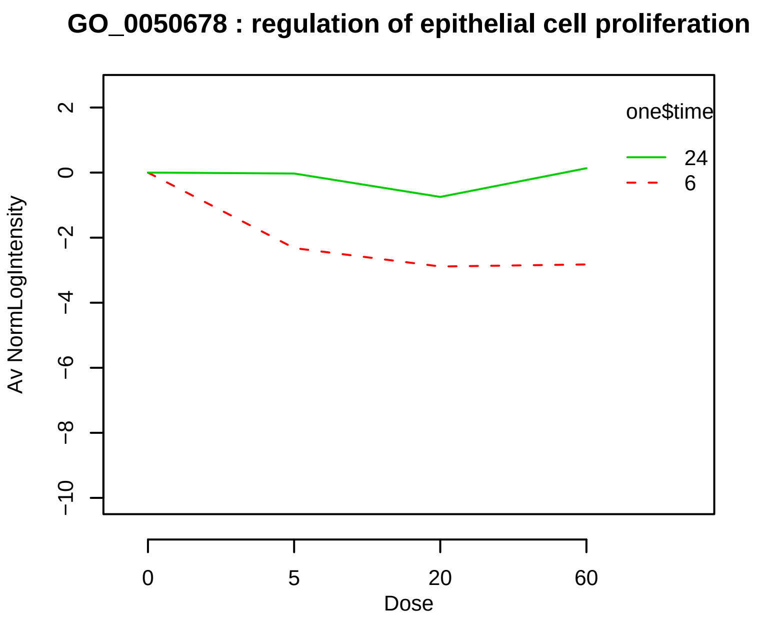
GO_0050678 : regulation of epithelial cell proliferation
Av NormLogIntensity
2
0
−2
−4
−6
−8
−10
0
5
20
60
Dose
one$time
24
6

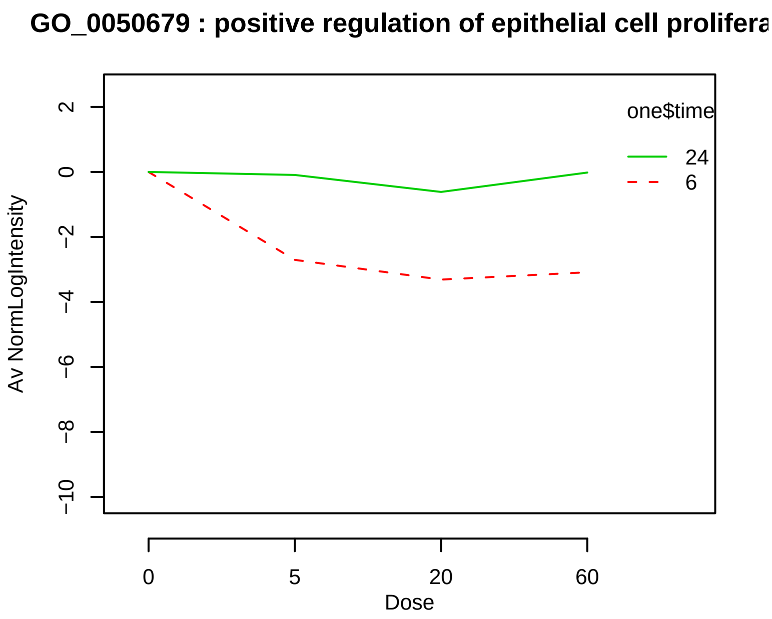
GO_0050679 : positive regulation of epithelial cell prolifera
Av NormLogIntensity
2
0
−2
−4
−6
−8
−10
0
5
20
60
Dose
one$time
24
6

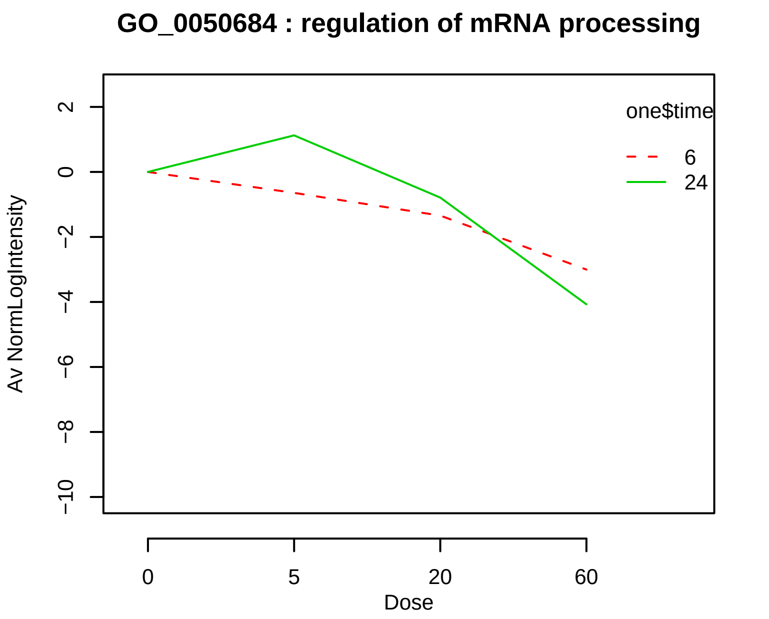
GO_0050684 : regulation of mRNA processing
Av NormLogIntensity
2
0
−2
−4
−6
−8
−10
0
5
20
60
Dose
one$time
6
24

GO_0050701 : interleukin−1 secretion

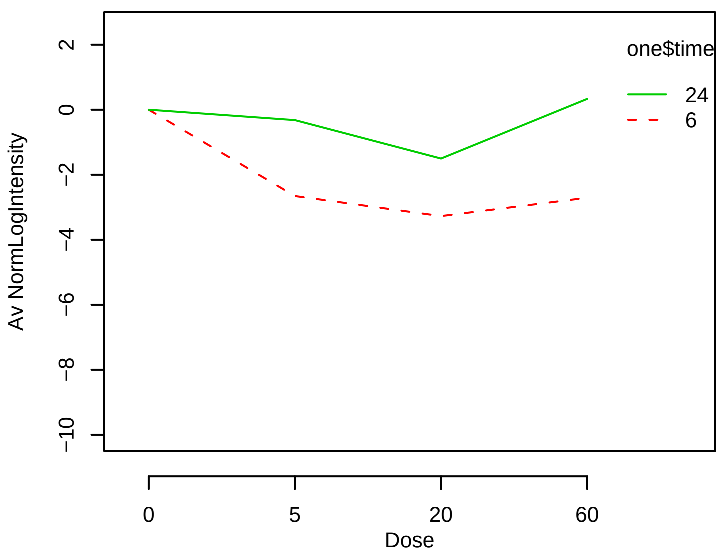

GO_0050702 : interleukin−1 beta secretion

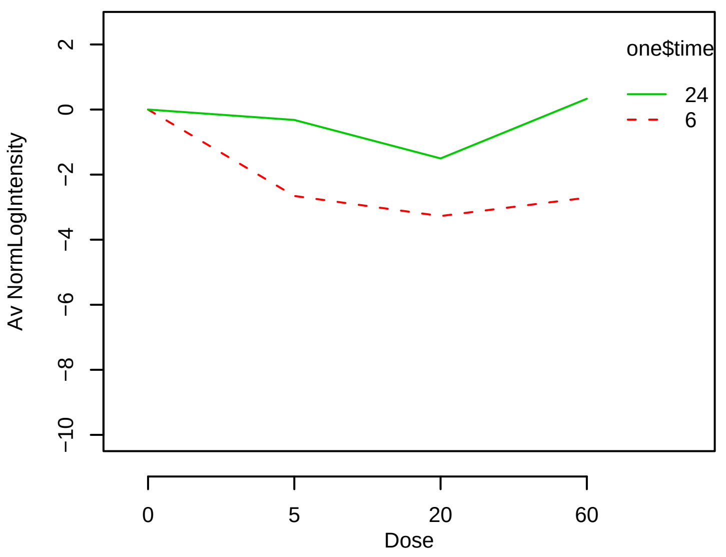

GO_0050704 : regulation of interleukin−1 secretion

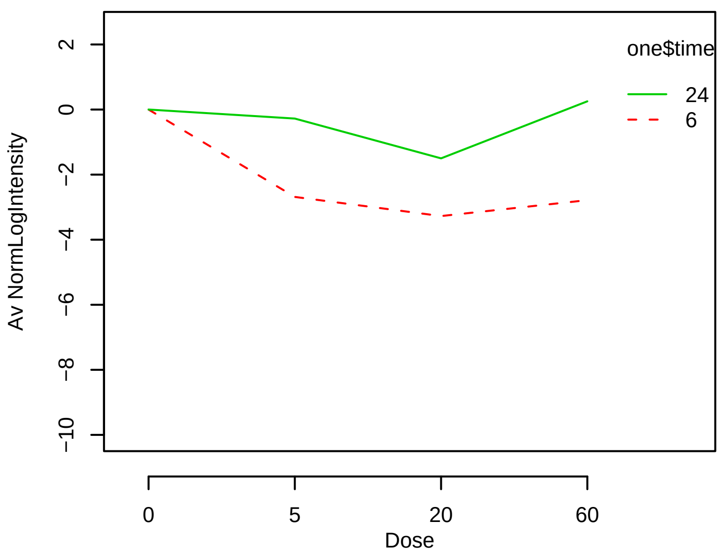

GO_0050706 : regulation of interleukin−1 beta secretion

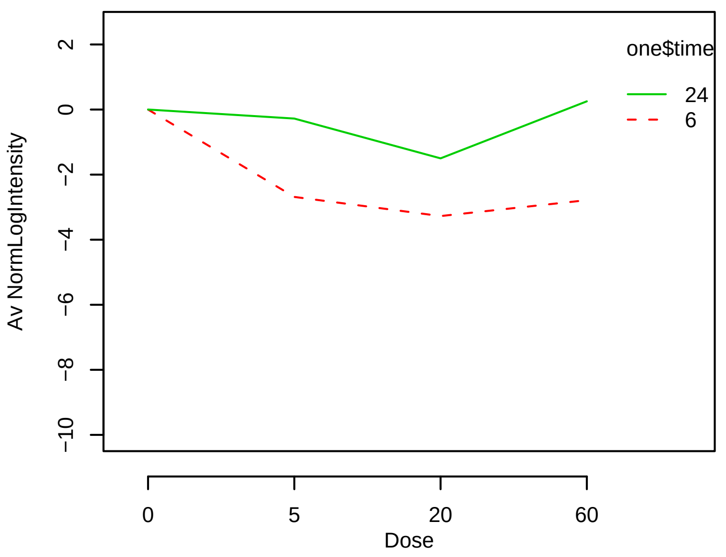

GO_0050707 : regulation of cytokine secretion

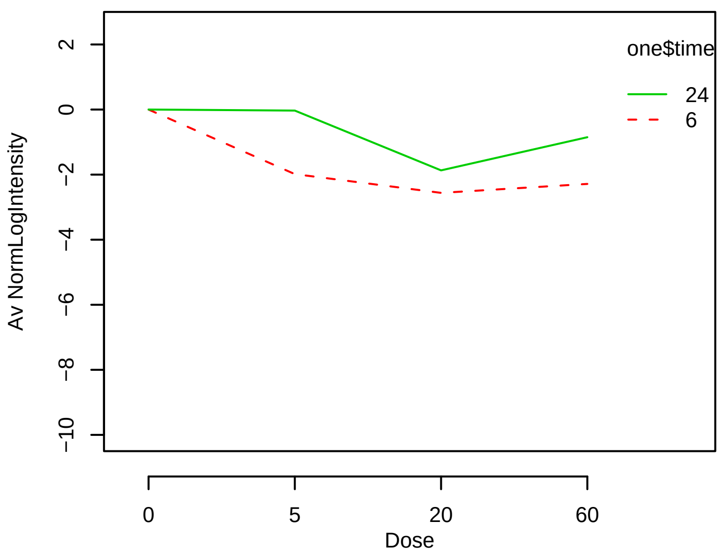

GO_0050708 : regulation of protein secretion

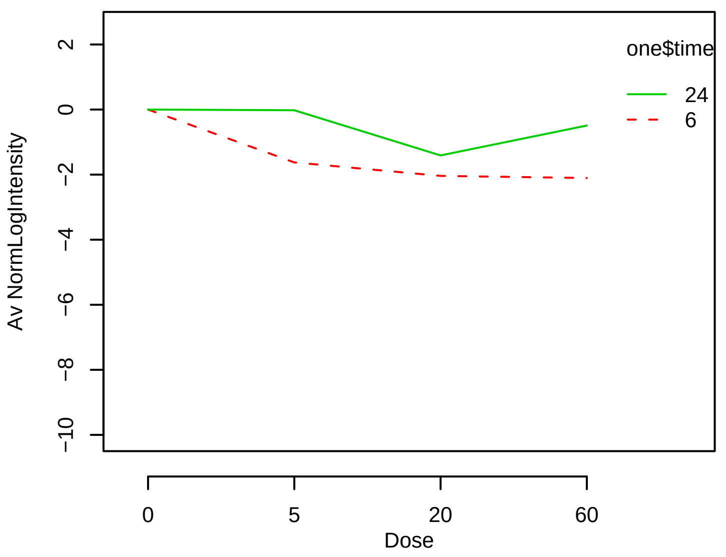

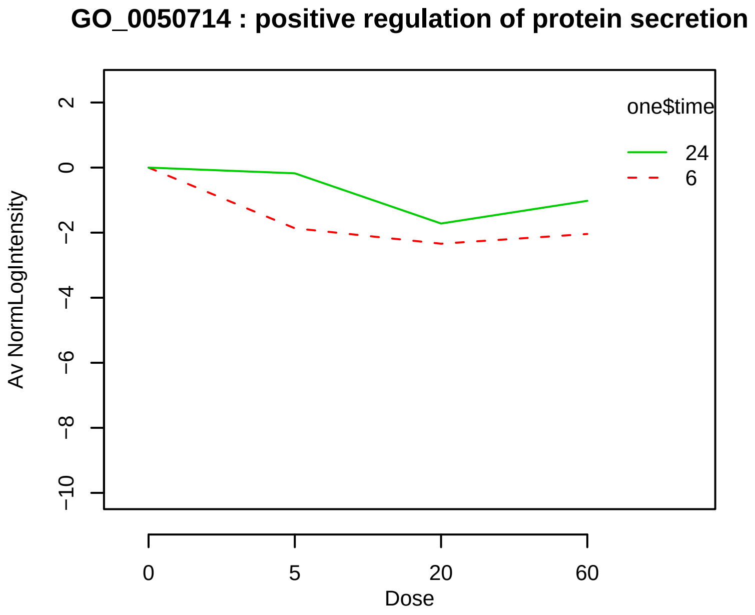
GO_0050714 : positive regulation of protein secretion
Av NormLogIntensity
Dose
one$time
24
6

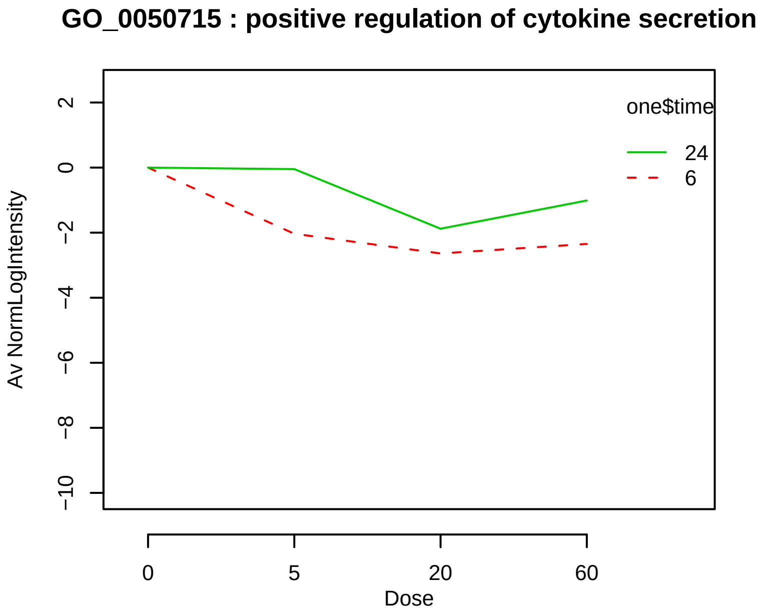
GO_0050715 : positive regulation of cytokine secretion
Av NormLogIntensity
Dose
one$time
24
6

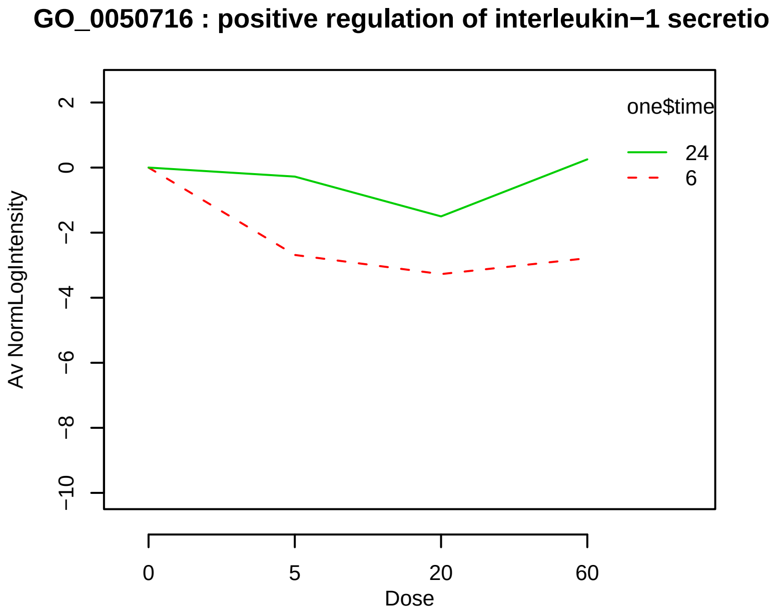
GO_0050716 : positive regulation of interleukin−1 secretio
Av NormLogIntensity
Dose
one$time
24
6

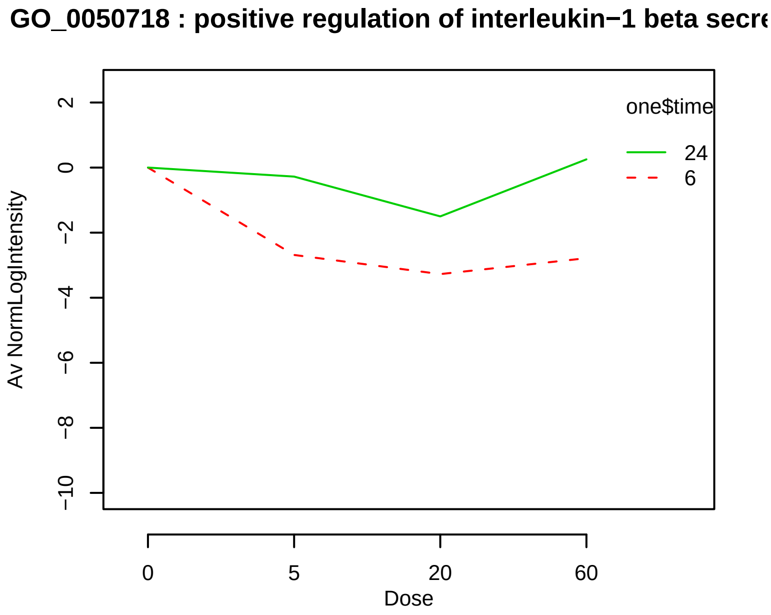
GO_0050718 : positive regulation of interleukin−1 beta secre
Av NormLogIntensity
Dose
one$time
24
6

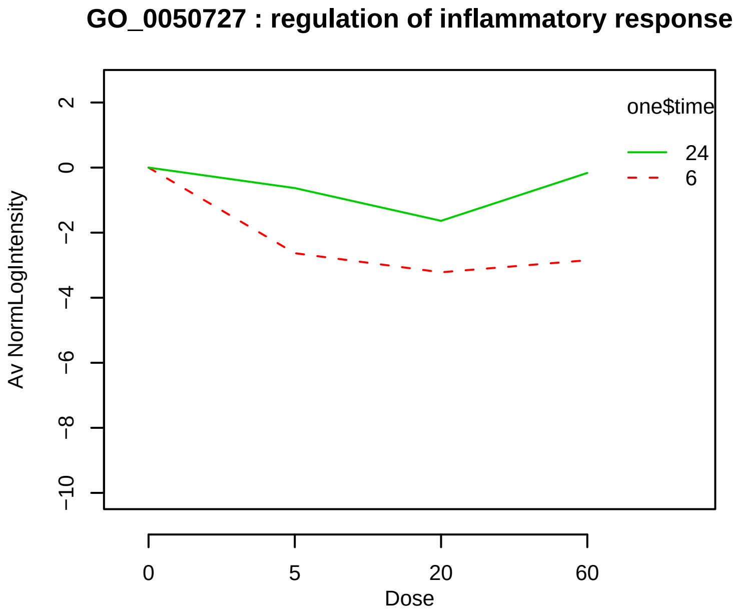
GO_0050727 : regulation of inflammatory response
Av NormLogIntensity
Dose
one$time
24
6

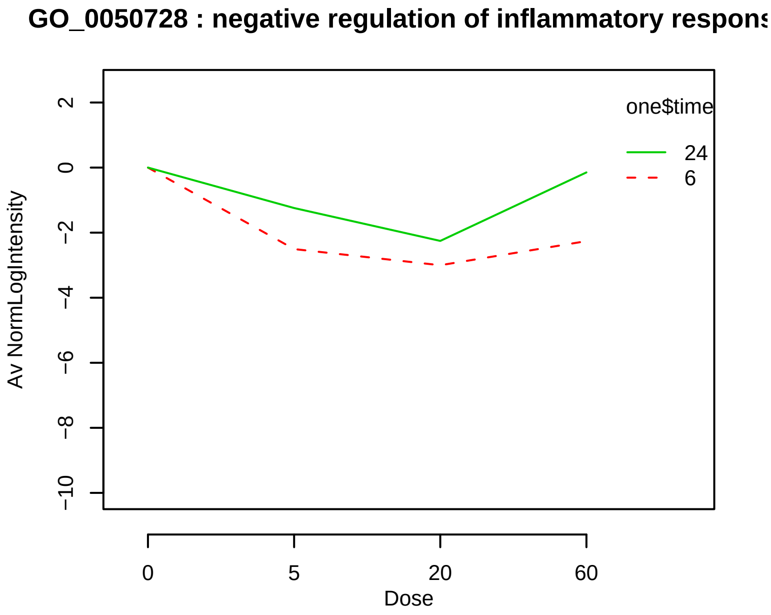
GO_0050728 : negative regulation of inflammatory respons
Av NormLogIntensity
Dose
one$time
24
6

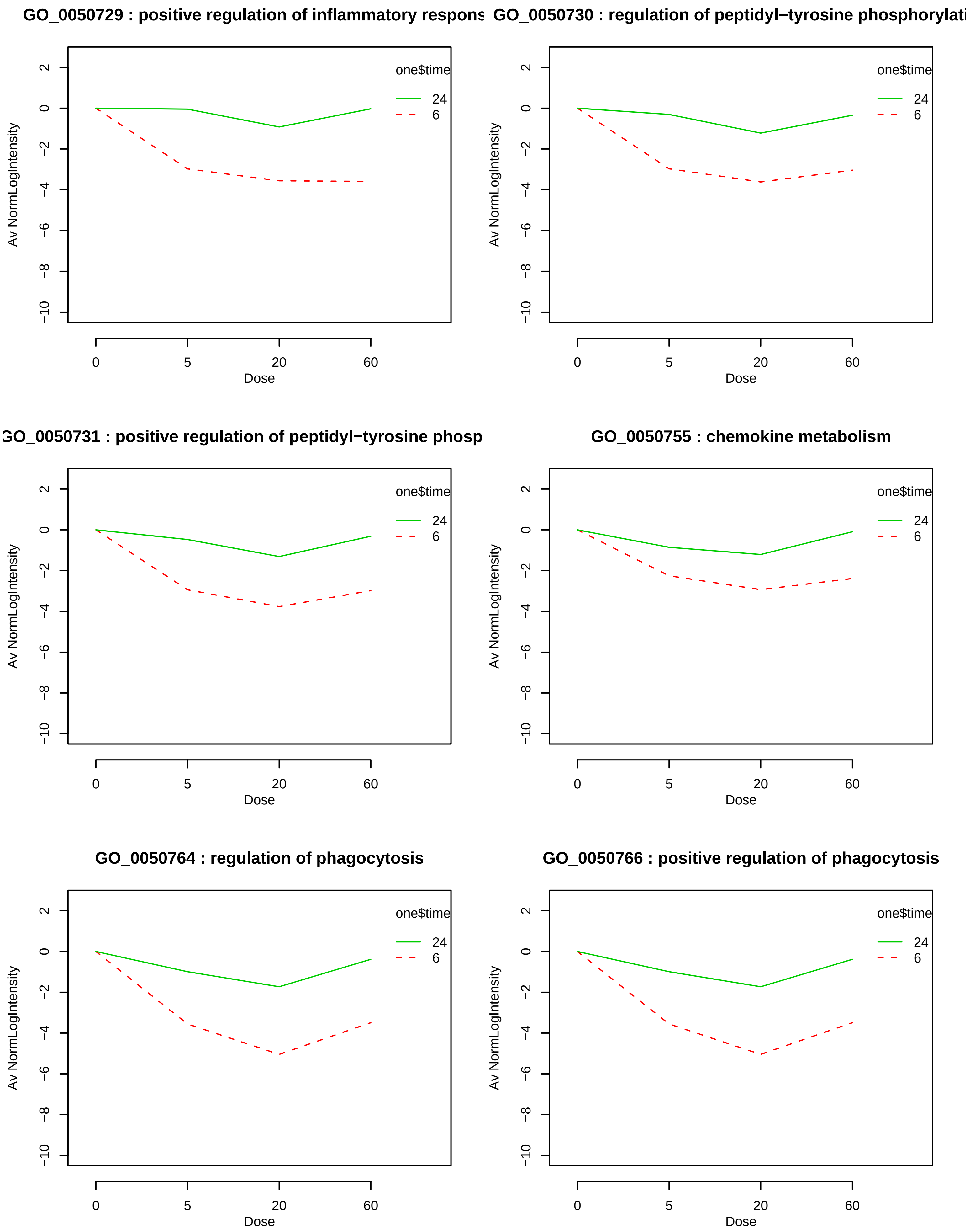
GO_0050729 : positive regulation of inflammatory respons
GO_0050730 : regulation of peptidyl−tyrosine phosphorylati
GO_0050731 : positive regulation of peptidyl−tyrosine phospl
GO_0050755 : chemokine metabolism
GO_0050764 : regulation of phagocytosis
GO_0050766 : positive regulation of phagocytosis
Av NormLogIntensity
Dose
one$time
24
6
0
5
20
60
2
0
−2
−4
−6
−8
−10

GO_0050767 : regulation of neurogenesis

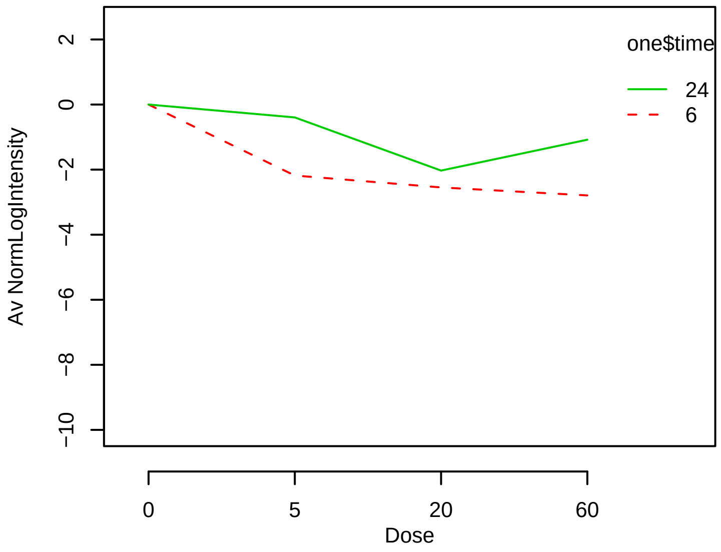

GO_0050768 : negative regulation of neurogenesis

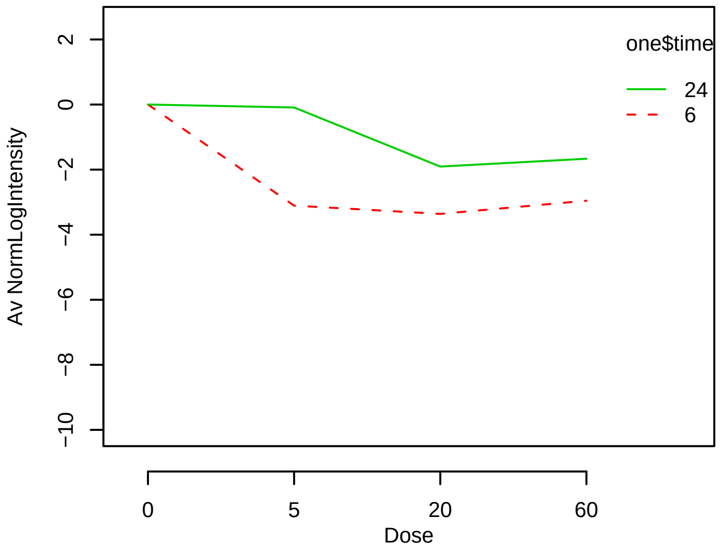

GO_0050769 : positive regulation of neurogenesis

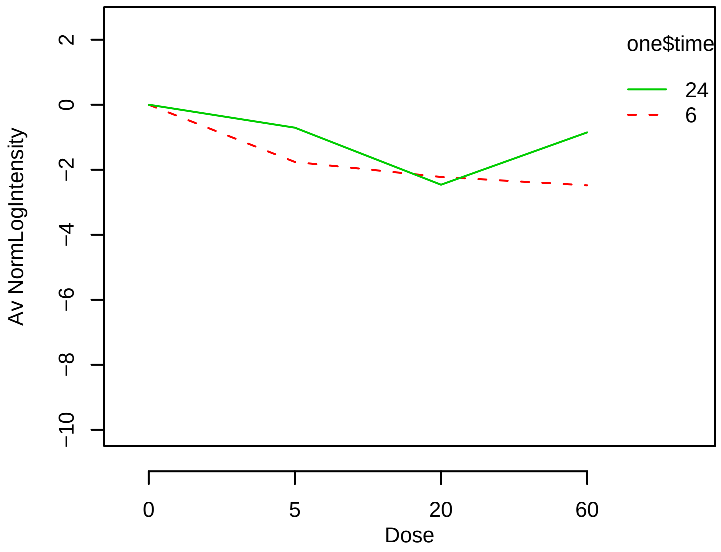

GO_0050770 : regulation of axonogenesis

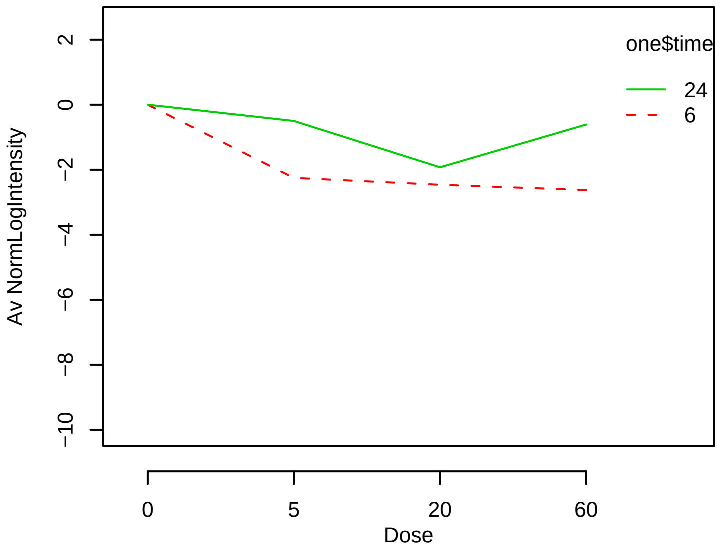

GO_0050771 : negative regulation of axonogenesis

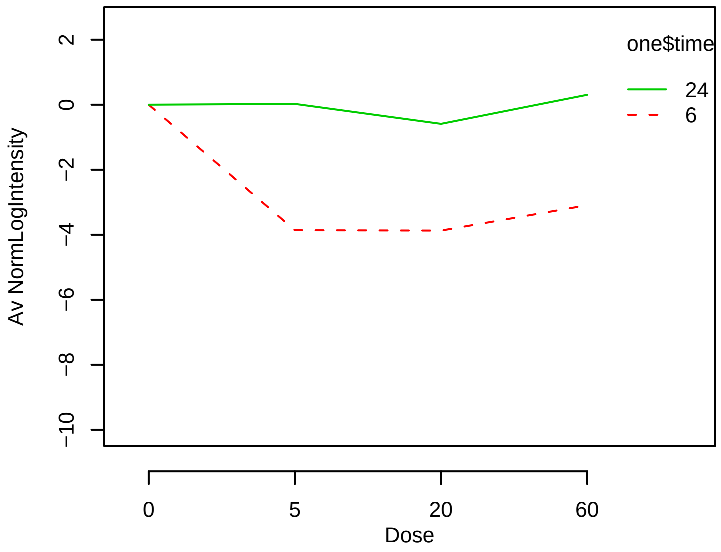

GO_0050772 : positive regulation of axonogenesis

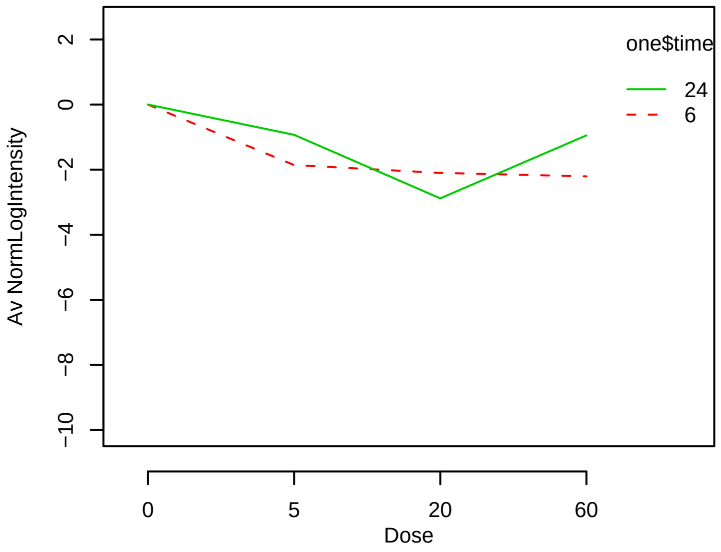

GO_0050776 : regulation of immune response

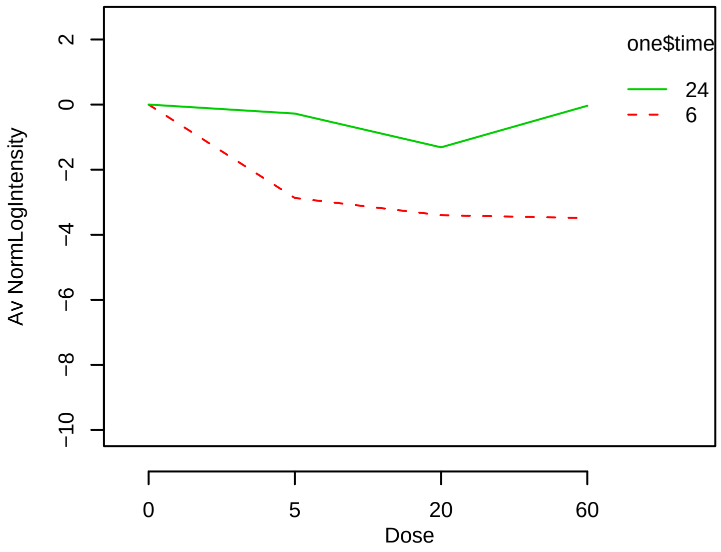

GO_0050777 : negative regulation of immune response

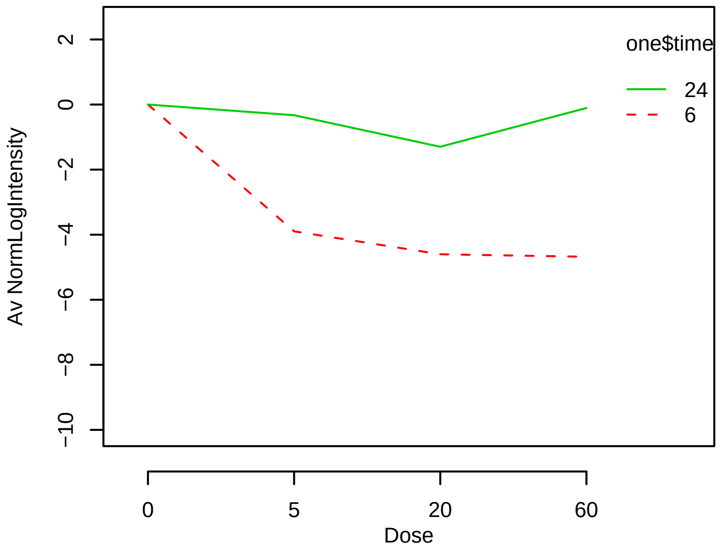

GO_0050778 : positive regulation of immune response

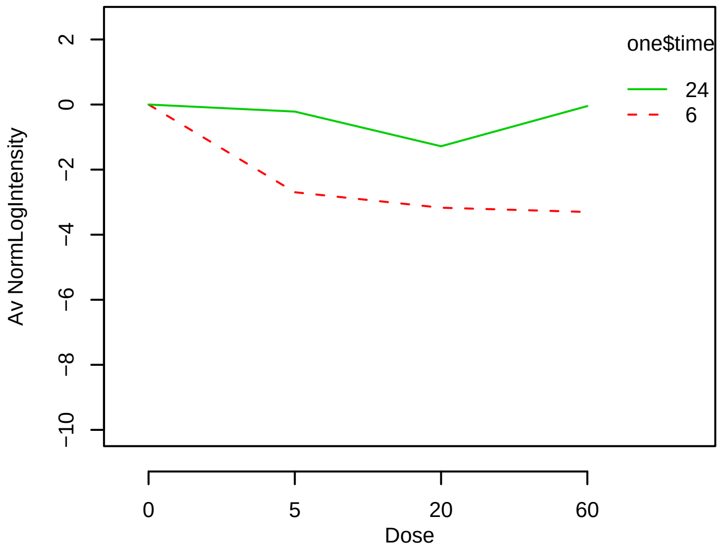

GO_0050792 : regulation of viral life cycle

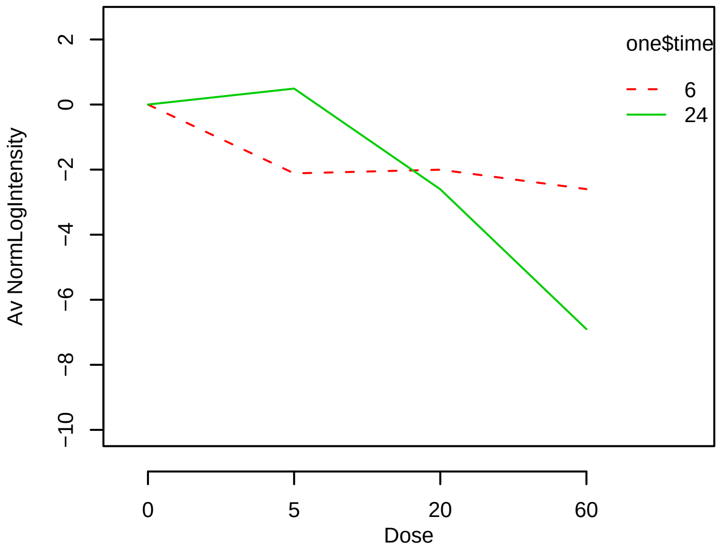

GO_0050793 : regulation of development

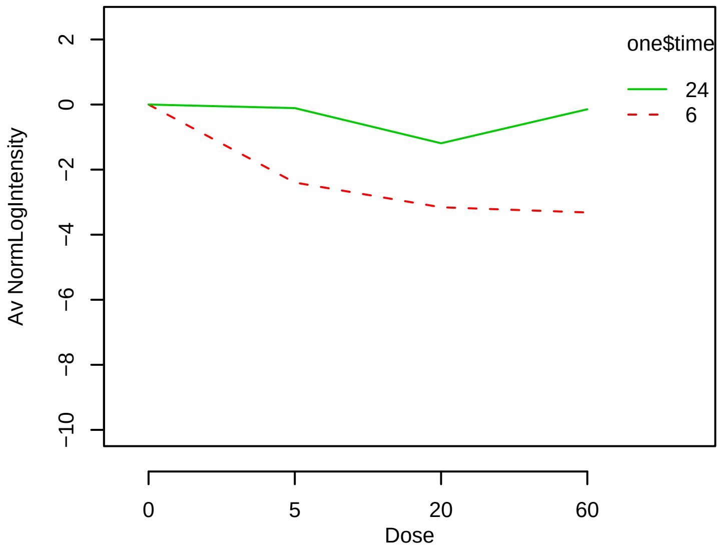

GO_0050796 : regulation of insulin secretion

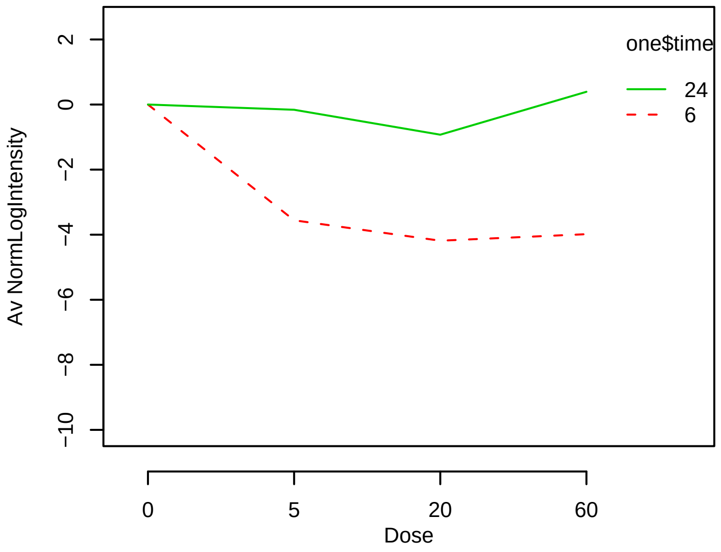

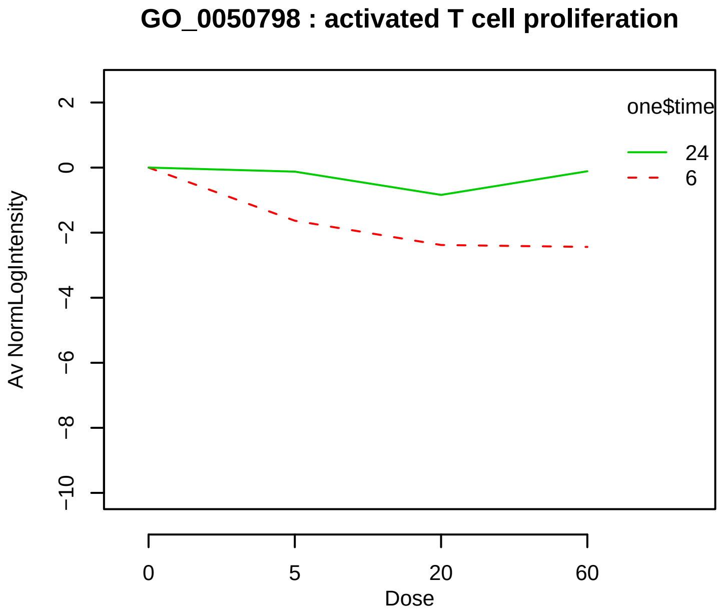
GO_0050798 : activated T cell proliferation
one$time
24
6
Av NormLogIntensity
2
0
−2
−4
−6
−8
−10
0
5
20
60
Dose

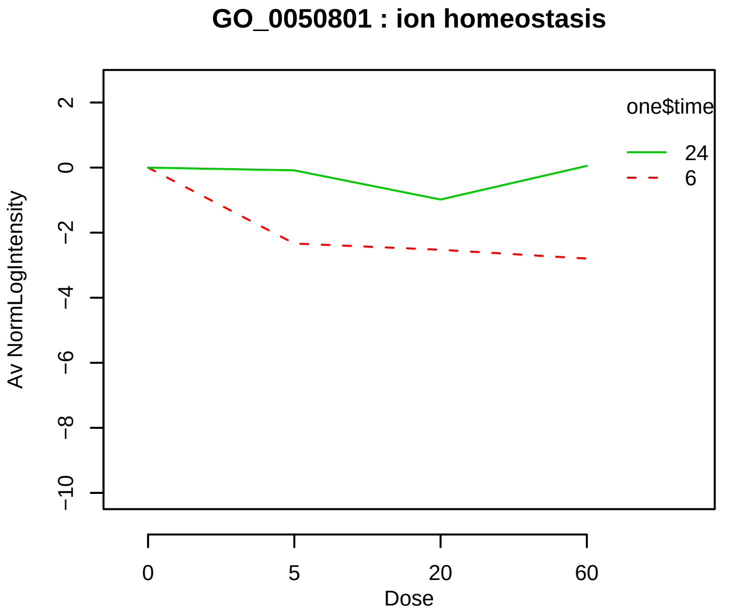
GO_0050801 : ion homeostasis
one$time
24
6
Av NormLogIntensity
2
0
−2
−4
−6
−8
−10
0
5
20
60
Dose

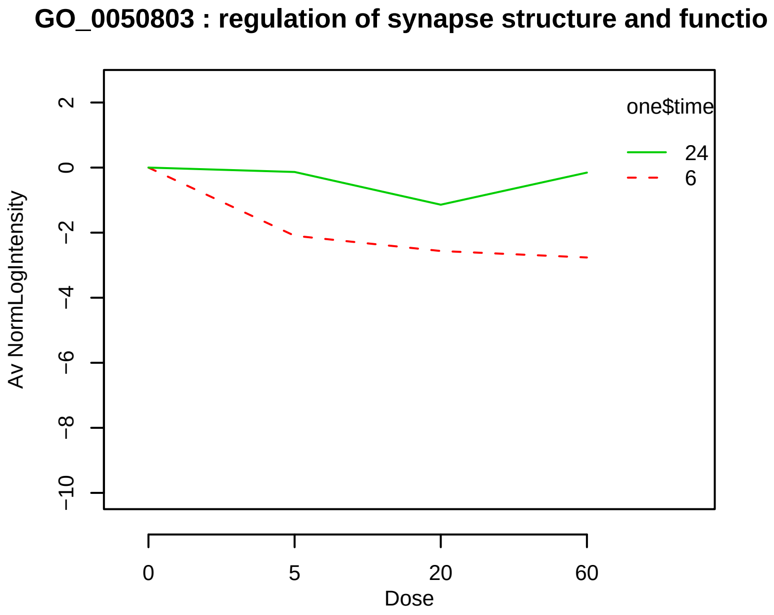
GO_0050803 : regulation of synapse structure and functio
one$time
24
6
Av NormLogIntensity
2
0
−2
−4
−6
−8
−10
0
5
20
60
Dose

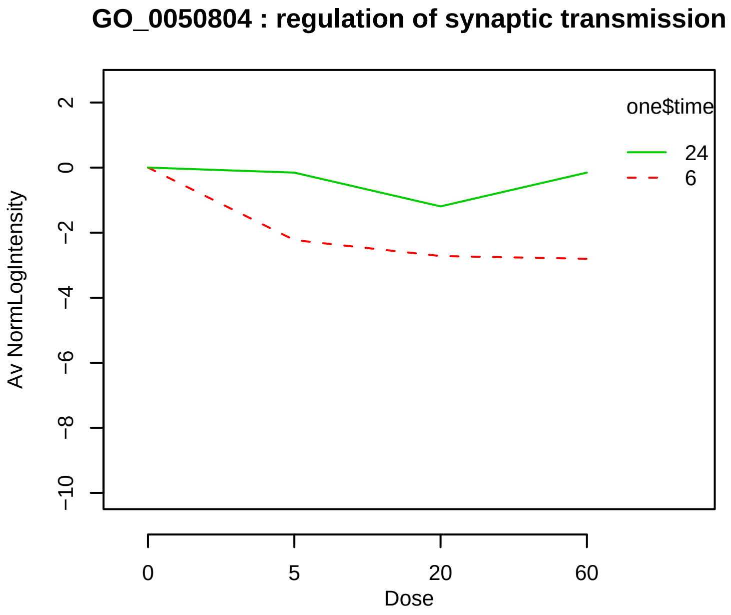
GO_0050804 : regulation of synaptic transmission
one$time
24
6
Av NormLogIntensity
2
0
−2
−4
−6
−8
−10
0
5
20
60
Dose

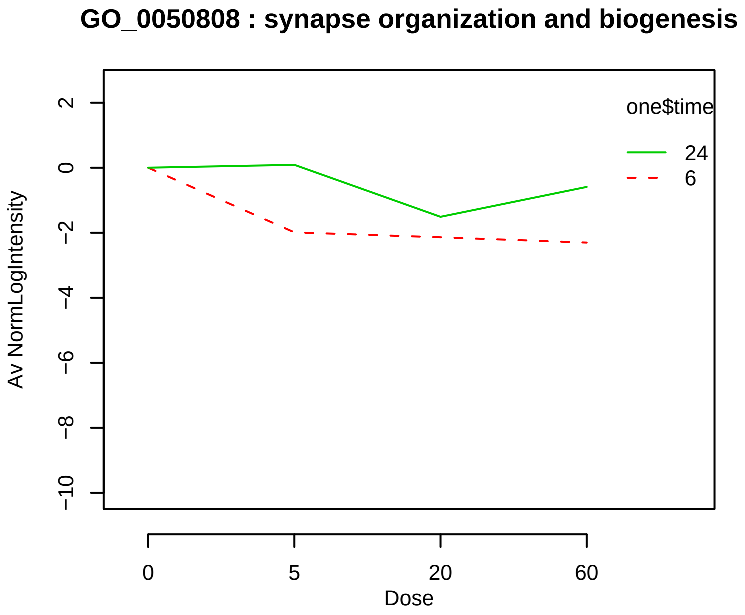
GO_0050808 : synapse organization and biogenesis
one$time
24
6
Av NormLogIntensity
2
0
−2
−4
−6
−8
−10
0
5
20
60
Dose

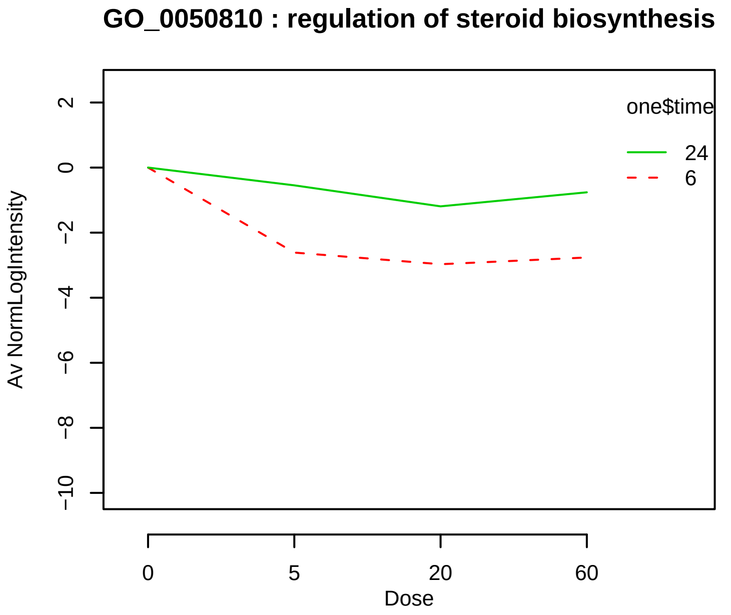
GO_0050810 : regulation of steroid biosynthesis
one$time
24
6
Av NormLogIntensity
2
0
−2
−4
−6
−8
−10
0
5
20
60
Dose

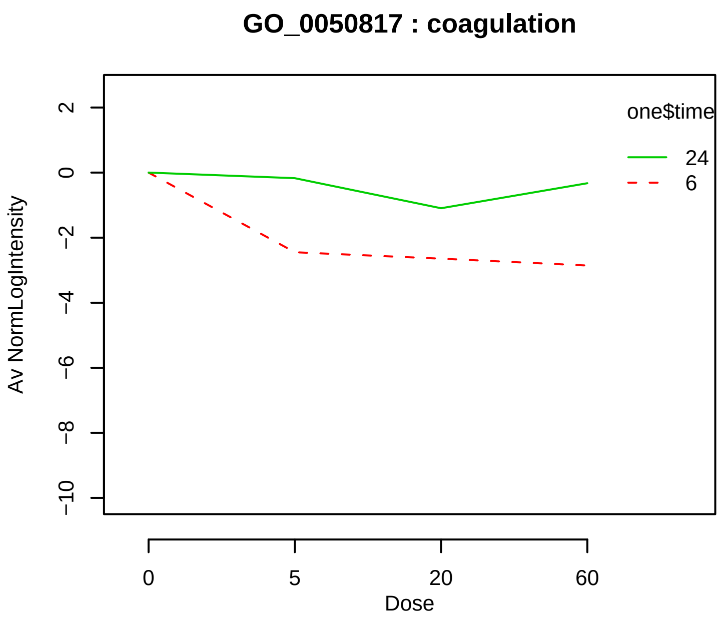
GO_0050817 : coagulation
one$time
24
6
Av NormLogIntensity
Dose

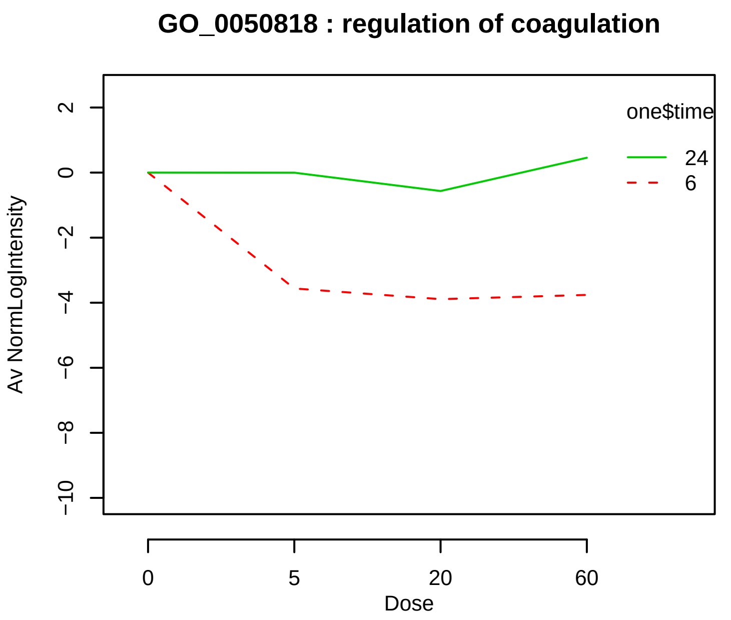
GO_0050818 : regulation of coagulation
one$time
24
6
Av NormLogIntensity
Dose

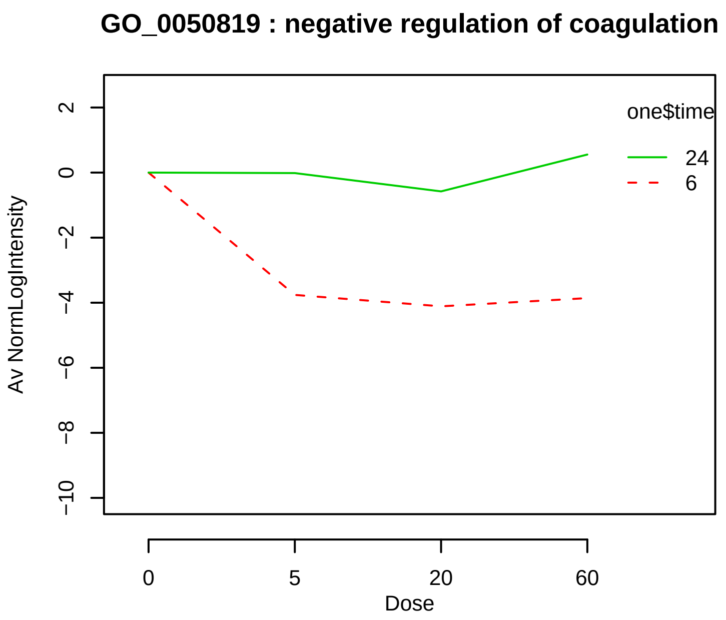
GO_0050819 : negative regulation of coagulation
one$time
24
6
Av NormLogIntensity
Dose

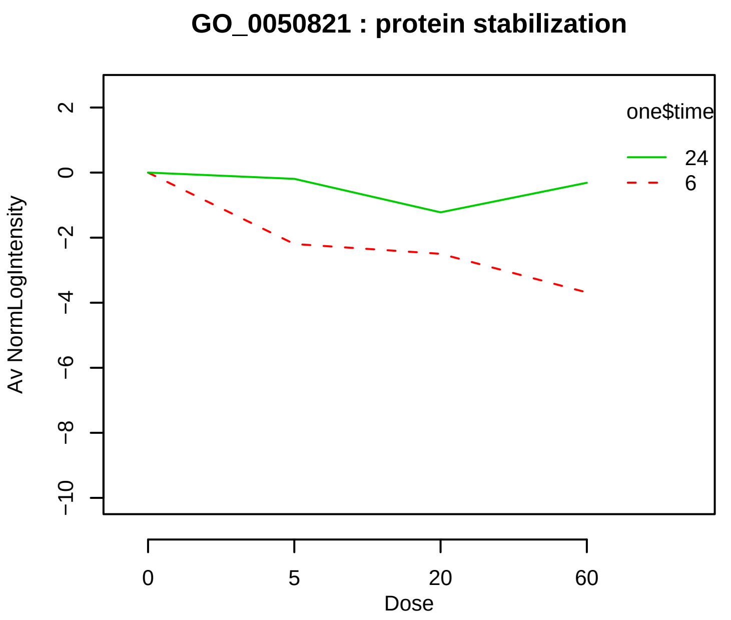
GO_0050821 : protein stabilization
one$time
24
6
Av NormLogIntensity
Dose

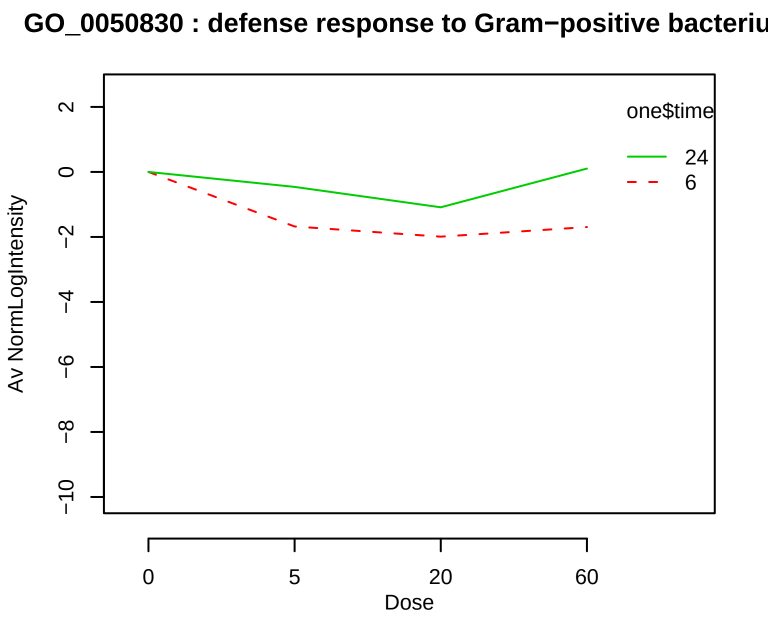
GO_0050830 : defense response to Gram−positive bacteriu
one$time
24
6
Av NormLogIntensity
Dose

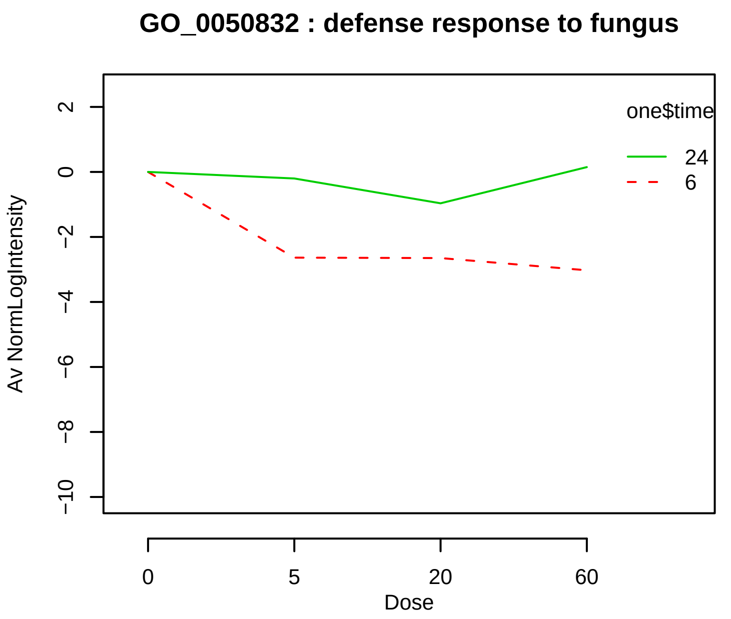
GO_0050832 : defense response to fungus
one$time
24
6
Av NormLogIntensity
Dose

GO_0050848 : regulation of calcium−mediated signaling

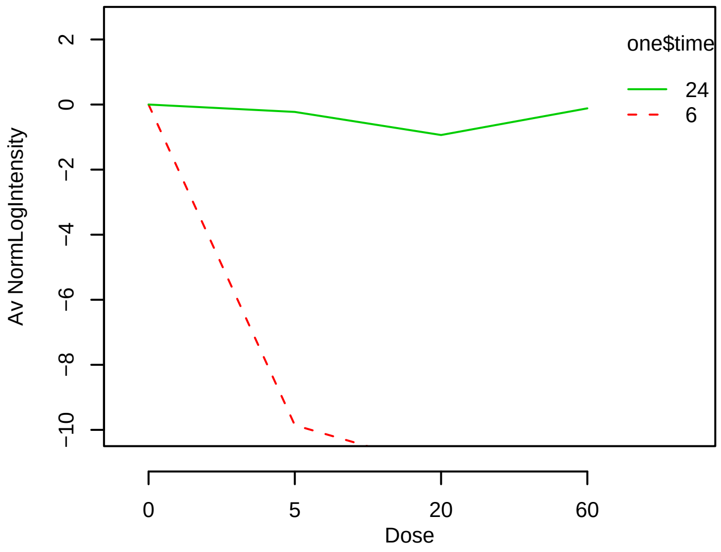

GO_0050850 : positive regulation of calcium−mediated signa

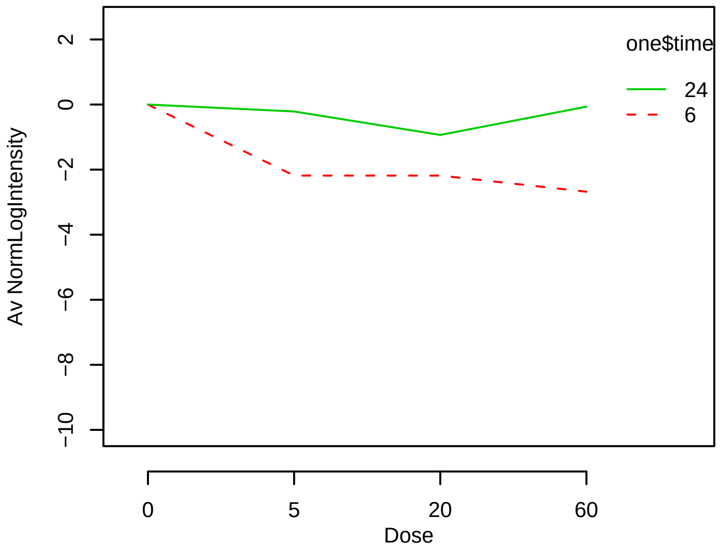

GO_0050851 : antigen receptor−mediated signaling pathwa

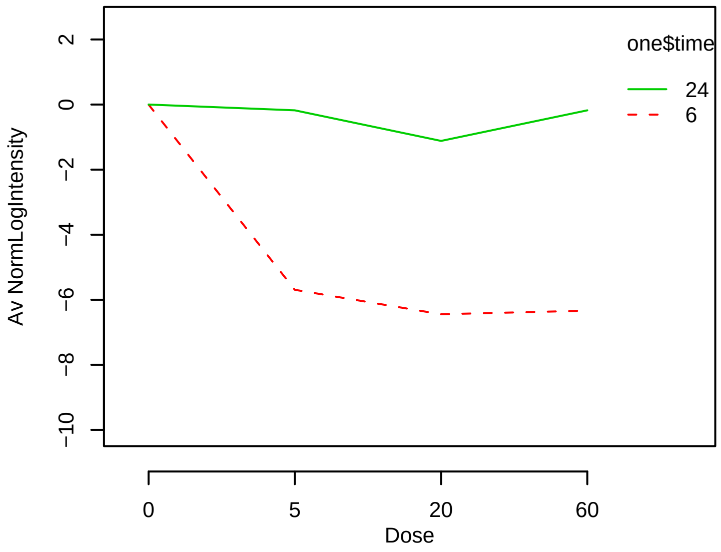

GO_0050852 : T cell receptor signaling pathway

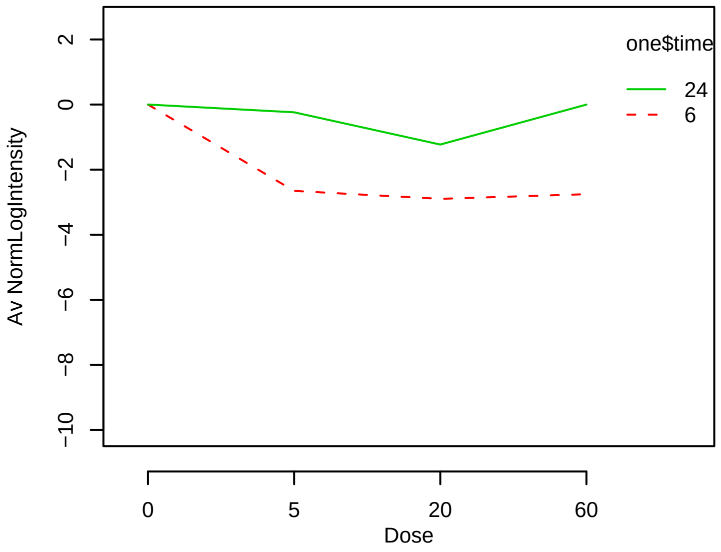

GO_0050853 : B cell receptor signaling pathway

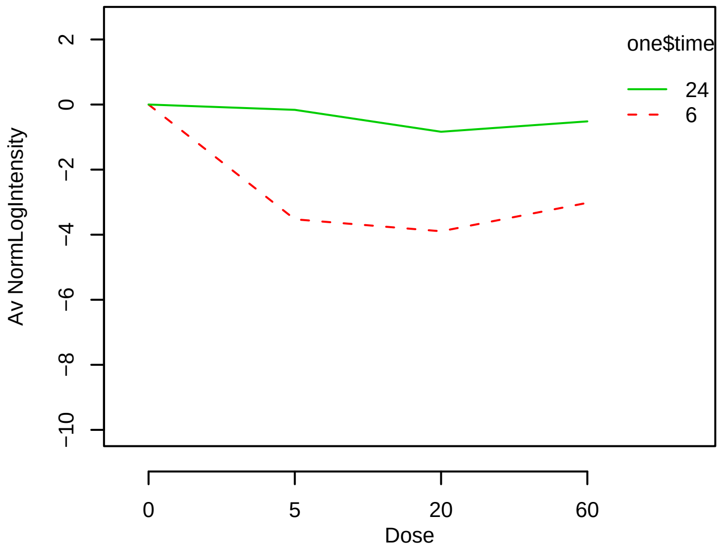

GO_0050854 : regulation of antigen receptor−mediated signa

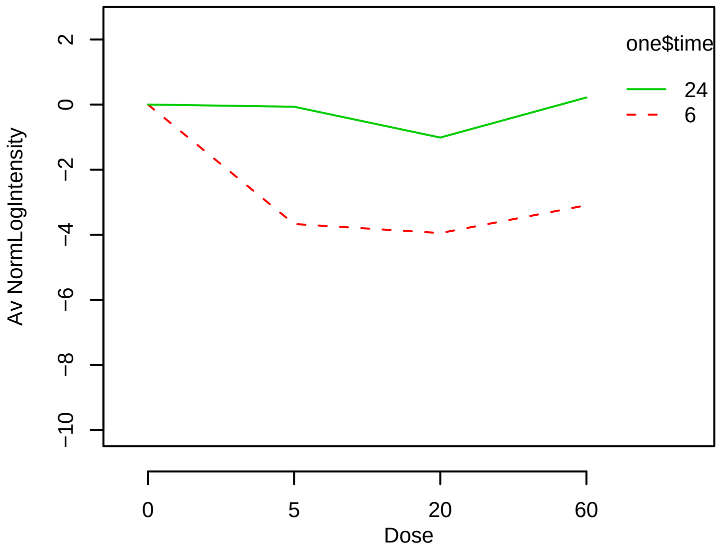

GO_0050856 : regulation of T cell receptor signaling pathw

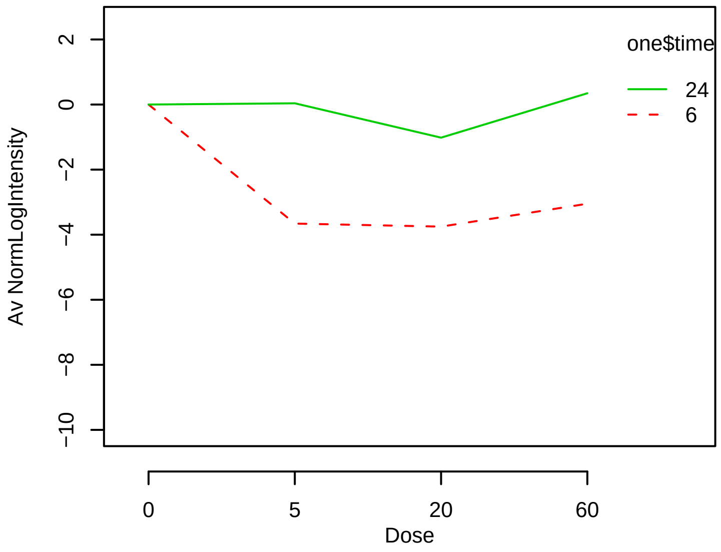

GO_0050858 : negative regulation of antigen receptor−mediat

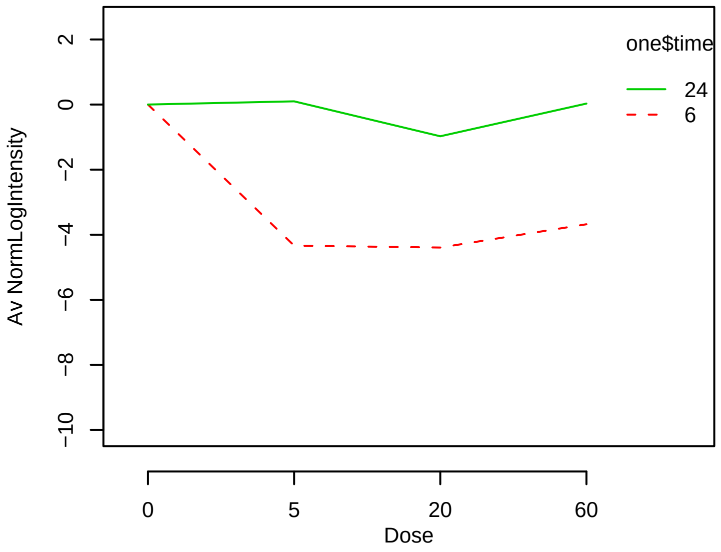

GO_0050860 : negative regulation of T cell receptor signalin

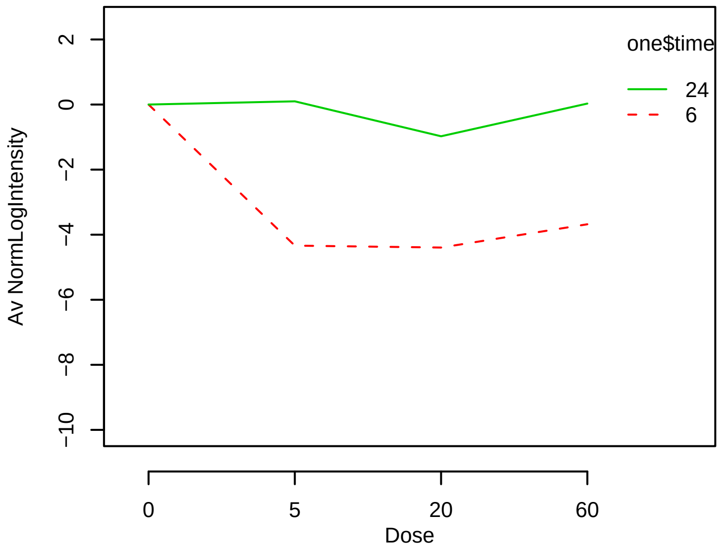

GO_0050863 : regulation of T cell activation

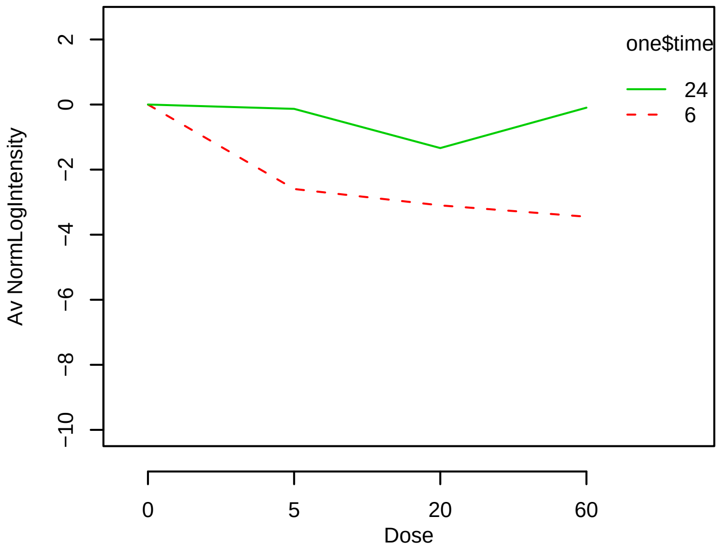

GO_0050864 : regulation of B cell activation

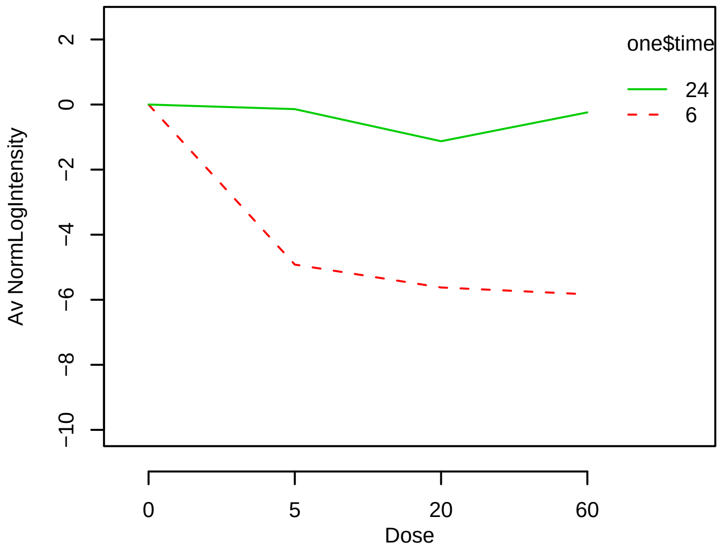

GO_0050865 : regulation of cell activation

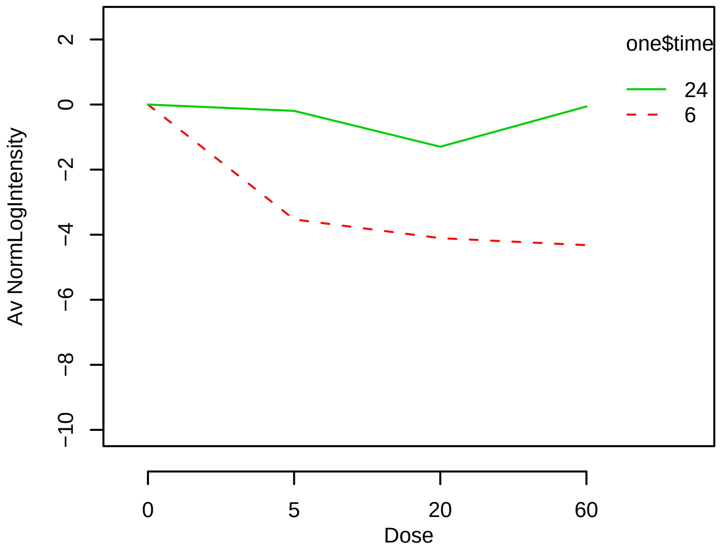

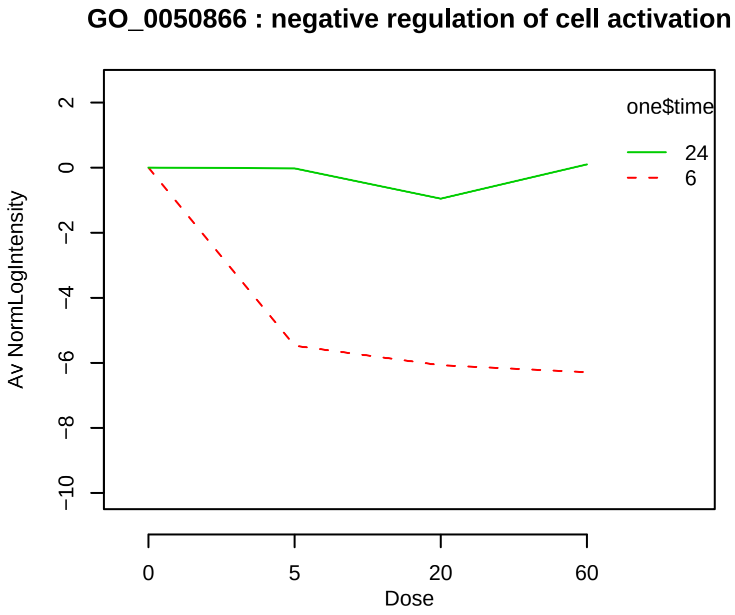
GO_0050866 : negative regulation of cell activation
one$time
24
6
Av NormLogIntensity
Dose
0
5
20
60

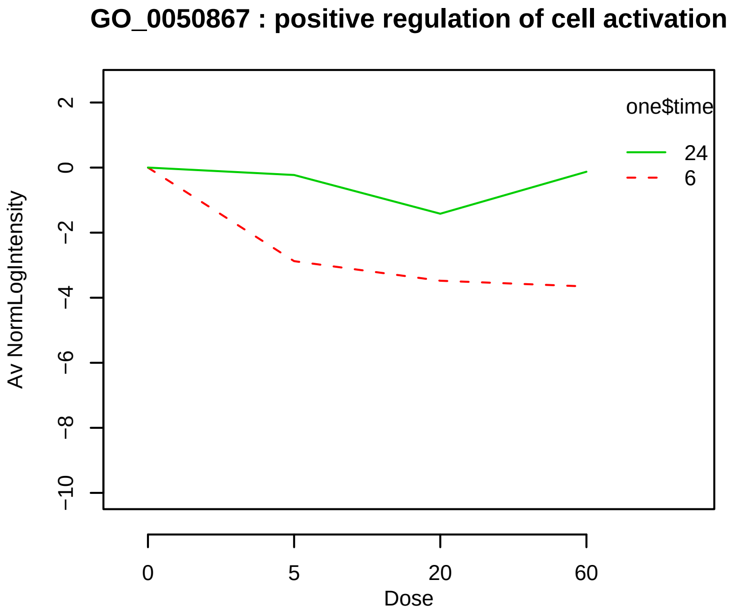
GO_0050867 : positive regulation of cell activation
one$time
24
6
Av NormLogIntensity
Dose
0
5
20
60

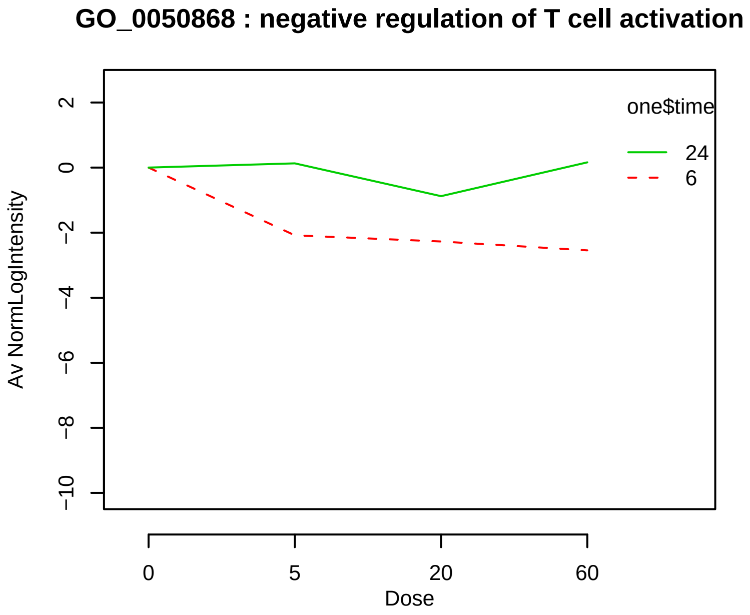
GO_0050868 : negative regulation of T cell activation
one$time
24
6
Av NormLogIntensity
Dose
0
5
20
60

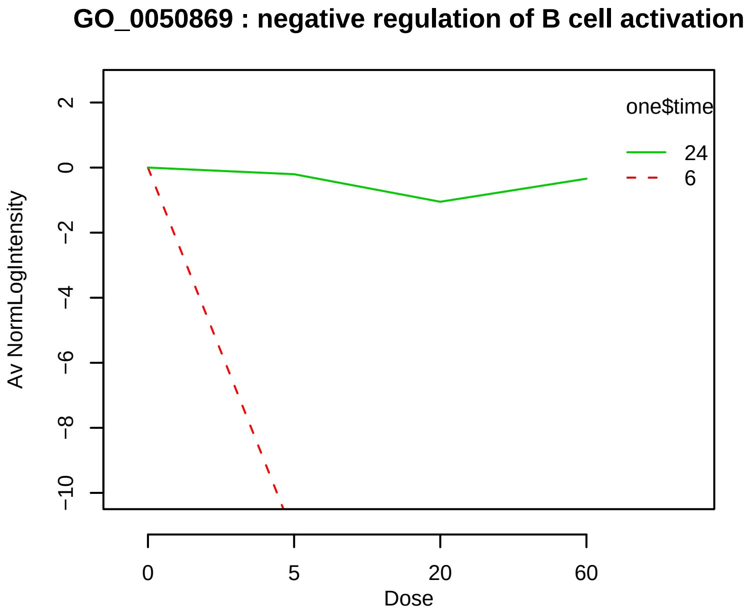
GO_0050869 : negative regulation of B cell activation
one$time
24
6
Av NormLogIntensity
Dose
0
5
20
60

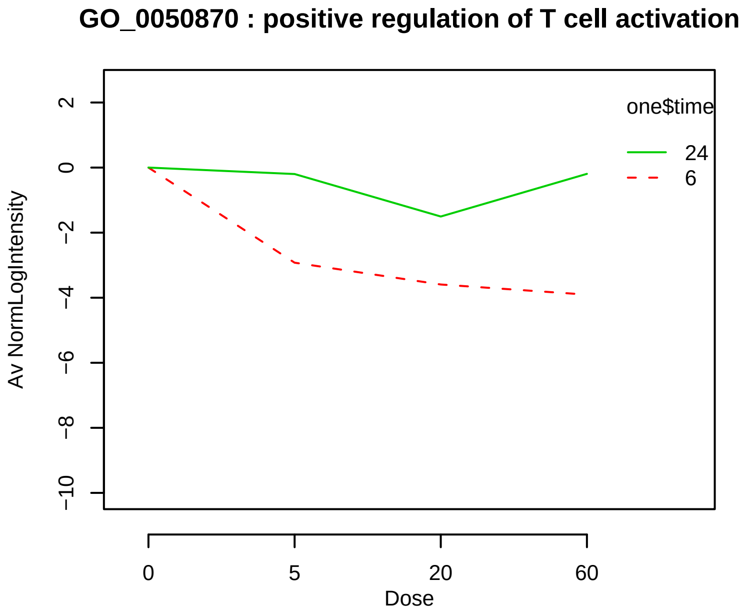
GO_0050870 : positive regulation of T cell activation
one$time
24
6
Av NormLogIntensity
Dose
0
5
20
60

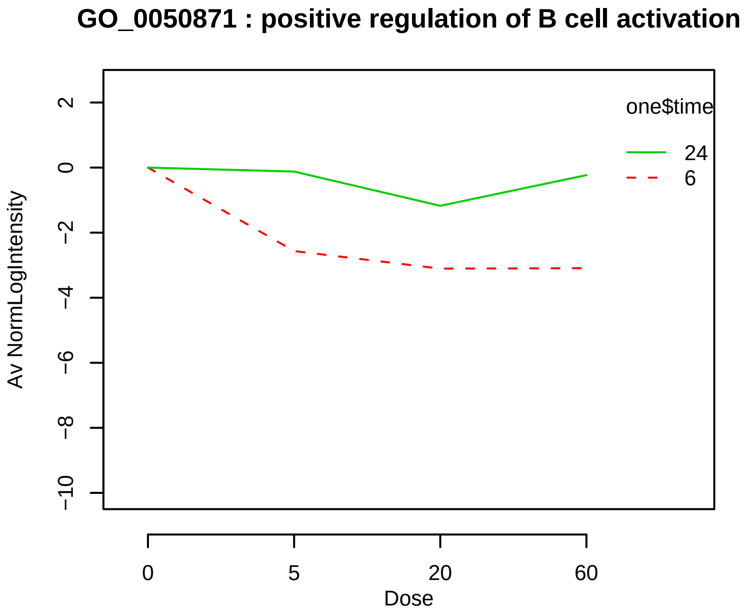
GO_0050871 : positive regulation of B cell activation
one$time
24
6
Av NormLogIntensity
Dose
0
5
20
60

GO_0050876 : reproductive physiological process

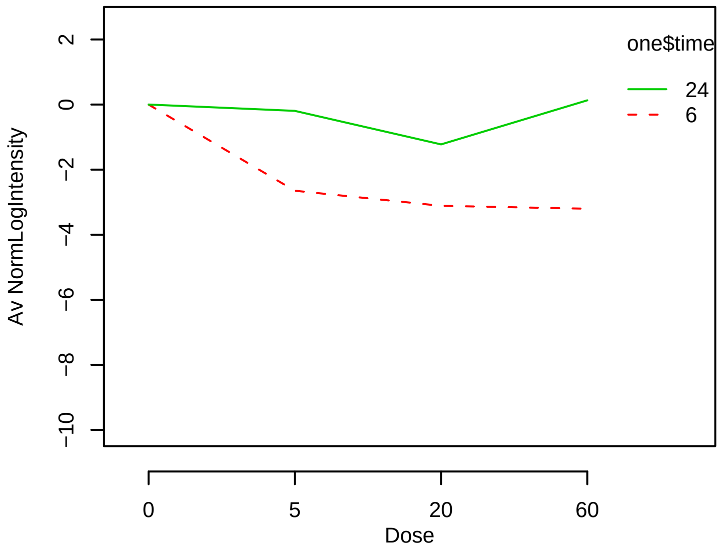

GO_0050878 : regulation of body fluids

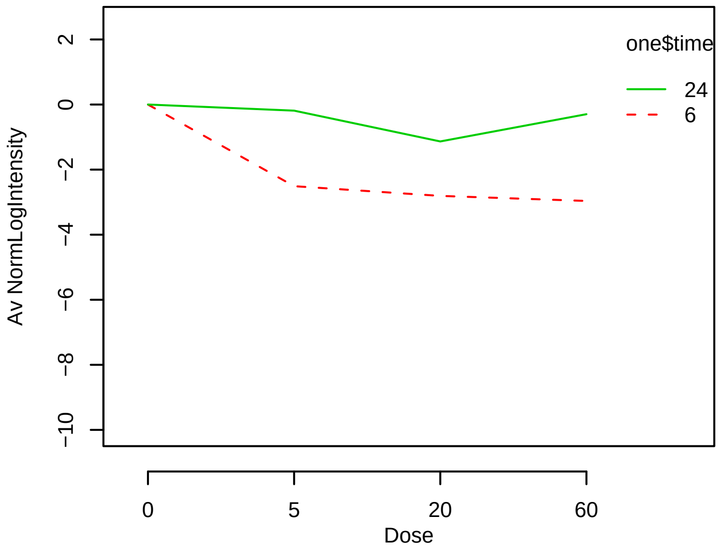

GO_0050879 : organismal movement

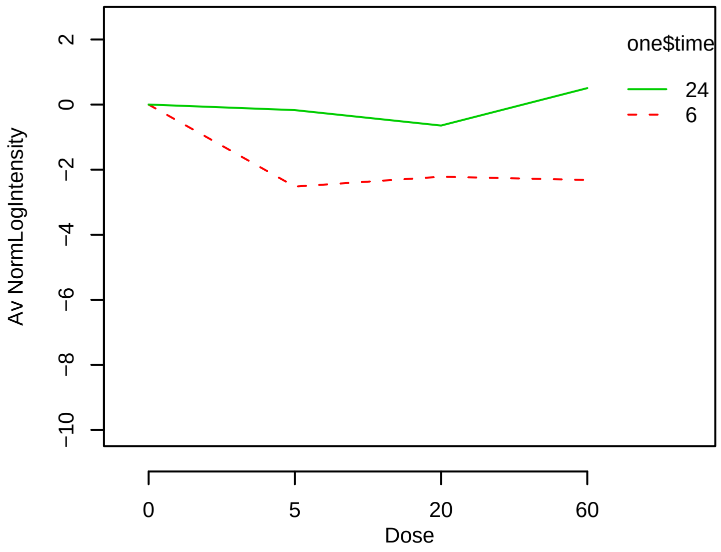

GO_0050880 : regulation of blood vessel size

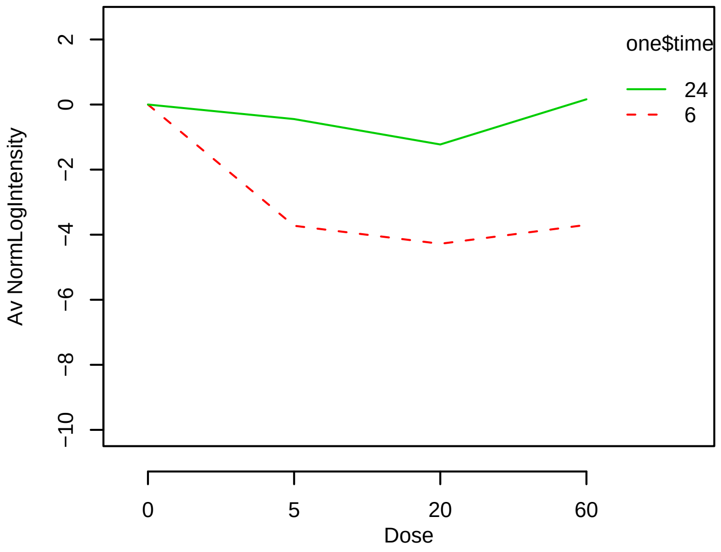

GO_0050881 : musculoskeletal movement

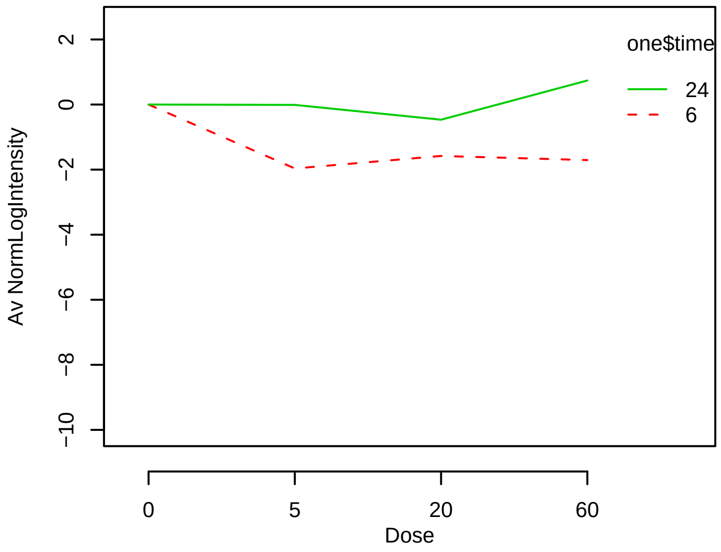

GO_0050885 : regulation of balance

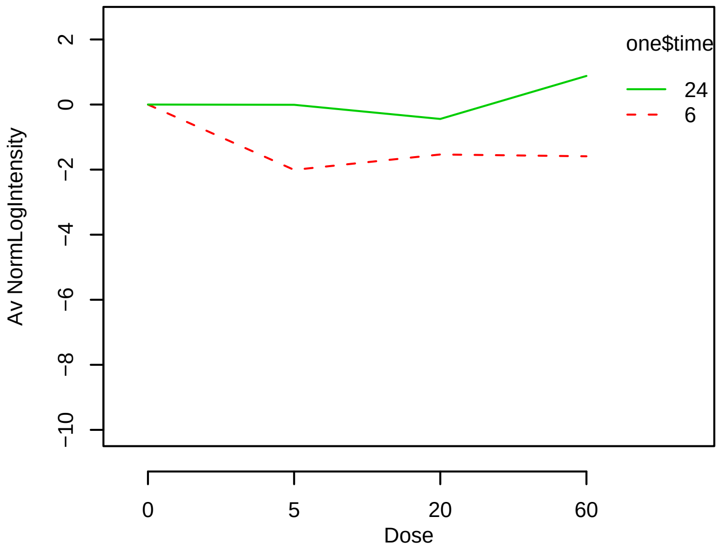

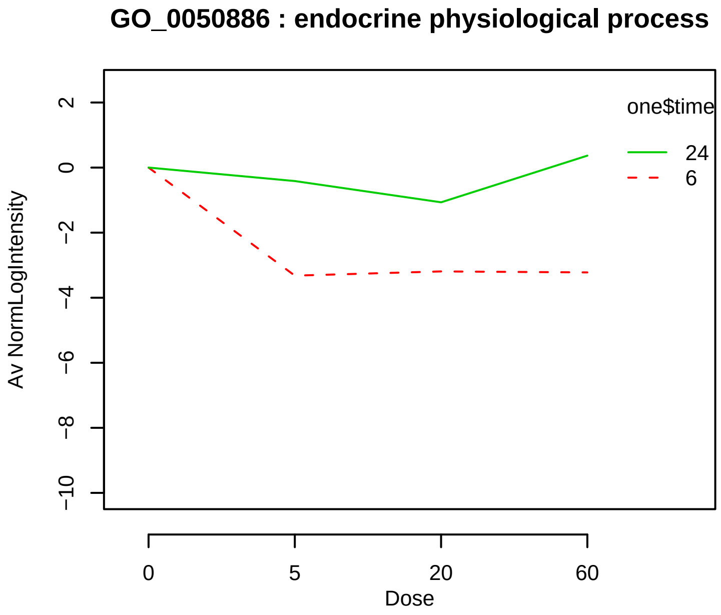
GO_0050886 : endocrine physiological process
Av NormLogIntensity
2
0
−2
−4
−6
−8
−10
one$time
24
6
0
5
20
60
Dose

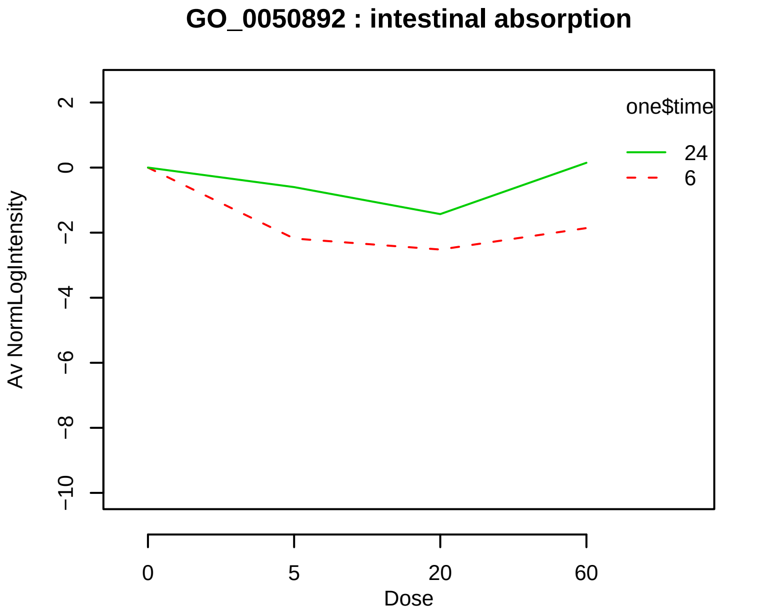
GO_0050892 : intestinal absorption
Av NormLogIntensity
2
0
−2
−4
−6
−8
−10
one$time
24
6
0
5
20
60
Dose

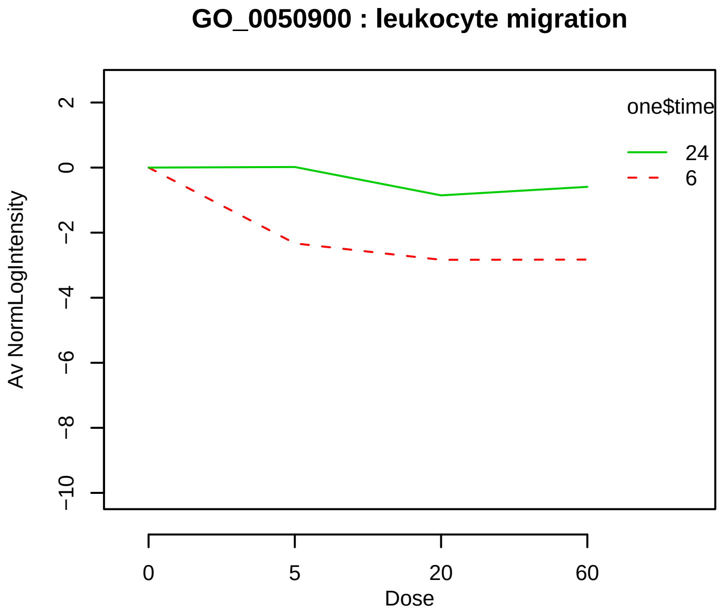
GO_0050900 : leukocyte migration
Av NormLogIntensity
2
0
−2
−4
−6
−8
−10
one$time
24
6
0
5
20
60
Dose

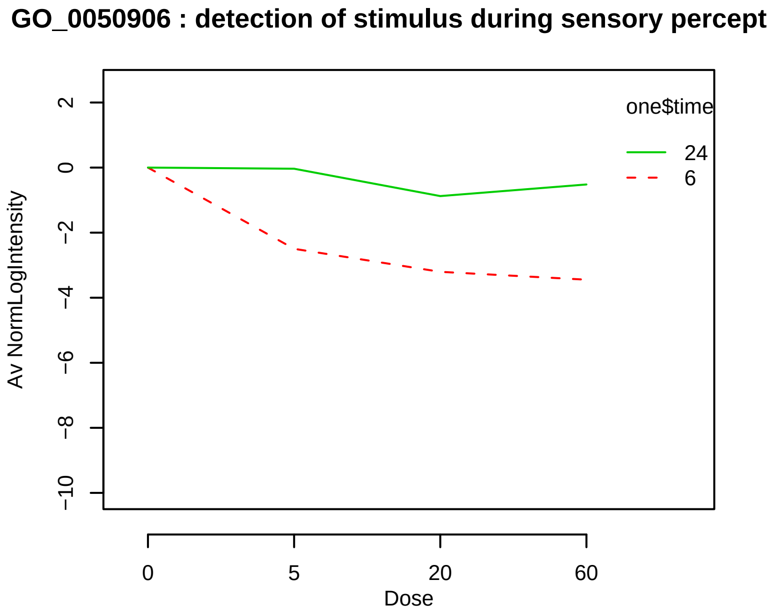
GO_0050906 : detection of stimulus during sensory percept
Av NormLogIntensity
2
0
−2
−4
−6
−8
−10
one$time
24
6
0
5
20
60
Dose

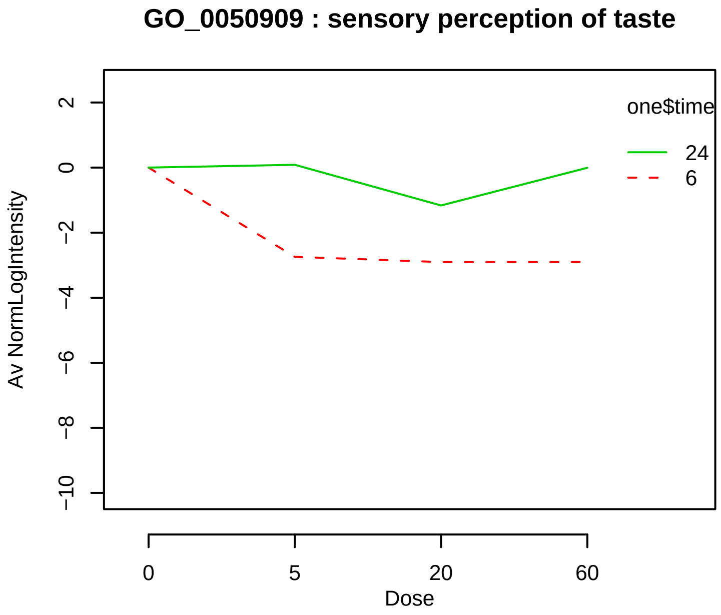
GO_0050909 : sensory perception of taste
Av NormLogIntensity
2
0
−2
−4
−6
−8
−10
one$time
24
6
0
5
20
60
Dose

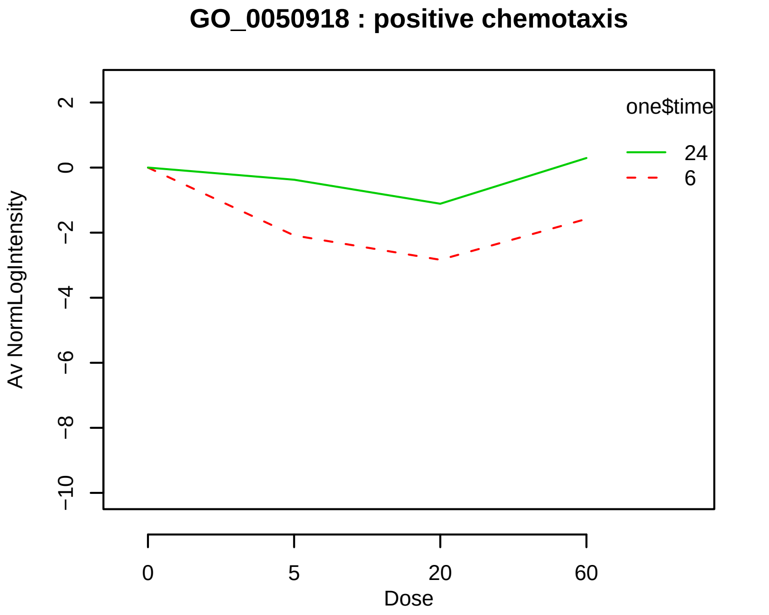
GO_0050918 : positive chemotaxis
Av NormLogIntensity
2
0
−2
−4
−6
−8
−10
one$time
24
6
0
5
20
60
Dose

GO_0050920 : regulation of chemotaxis

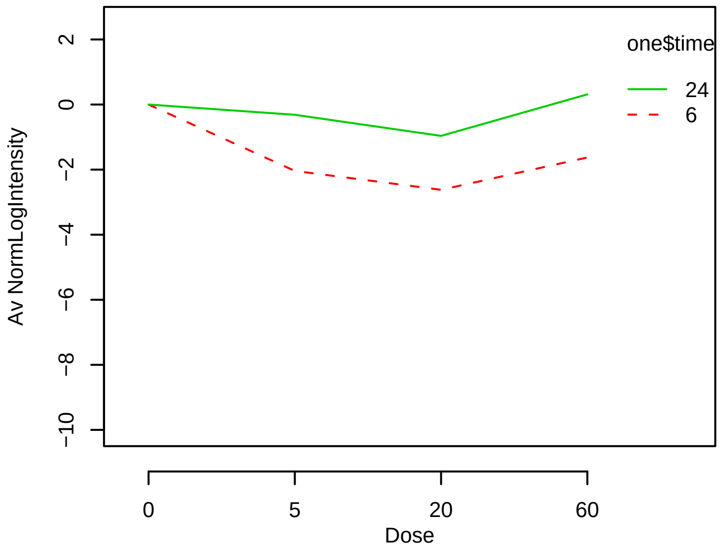

GO_0050921 : positive regulation of chemotaxis

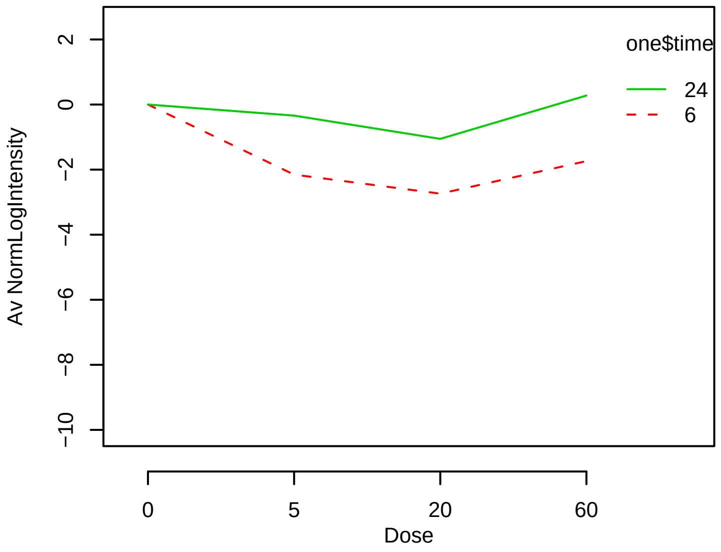

GO_0050926 : regulation of positive chemotaxis

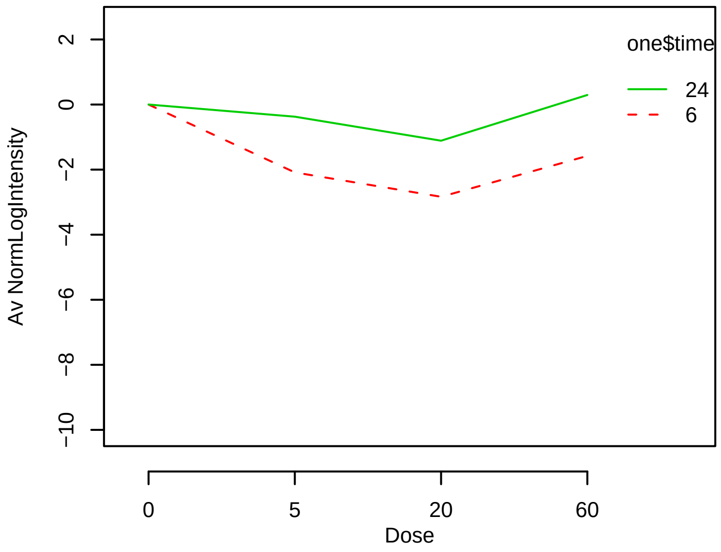

GO_0050927 : positive regulation of positive chemotaxis

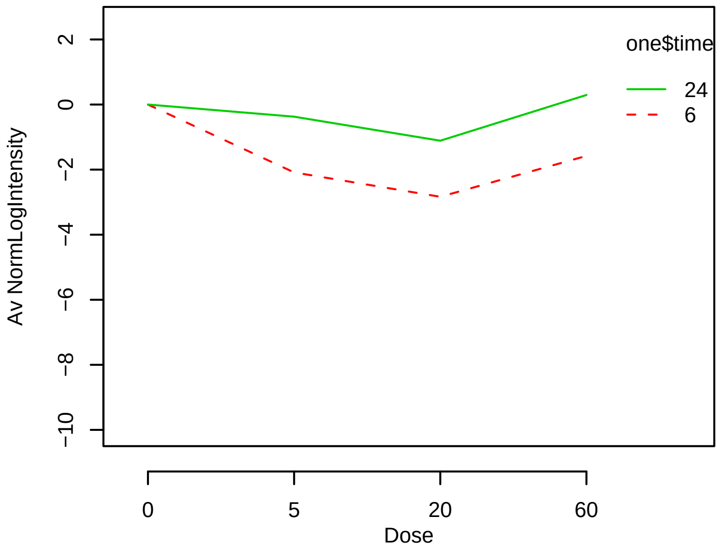

GO_0050930 : induction of positive chemotaxis

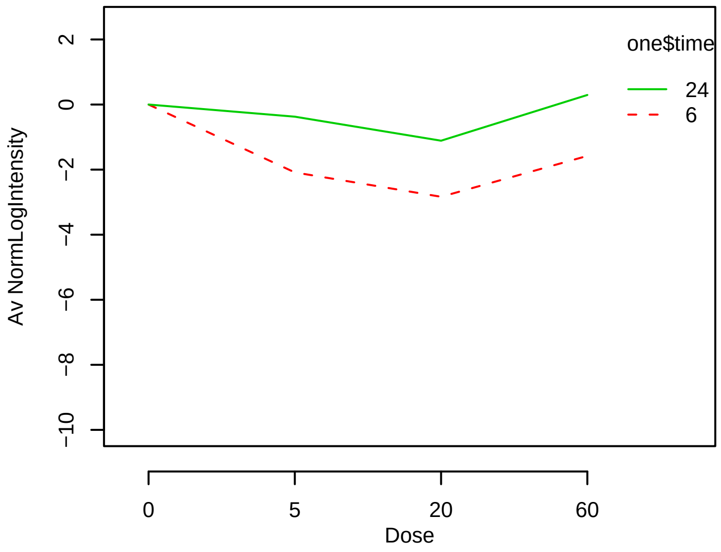

GO_0050931 : pigment cell differentiation

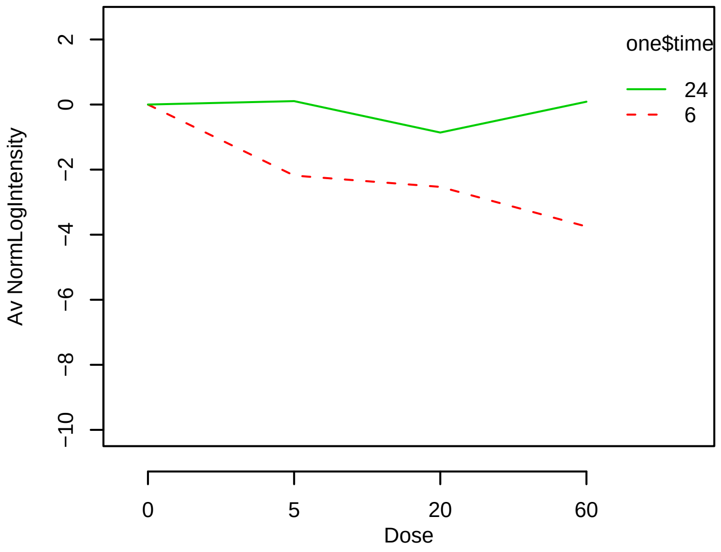

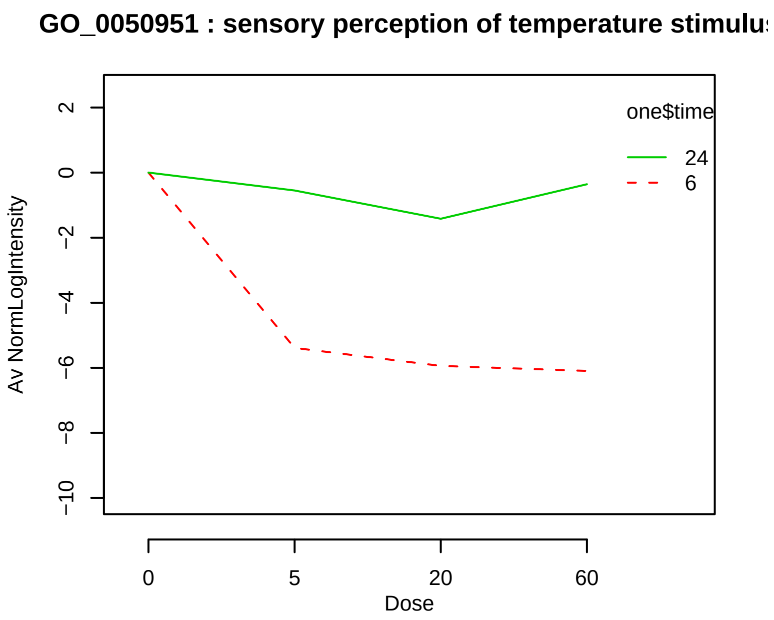
GO_0050951 : sensory perception of temperature stimulu
one$time
24
6
Av NormLogIntensity
2
0
−2
−4
−6
−8
−10
0
5
20
60
Dose

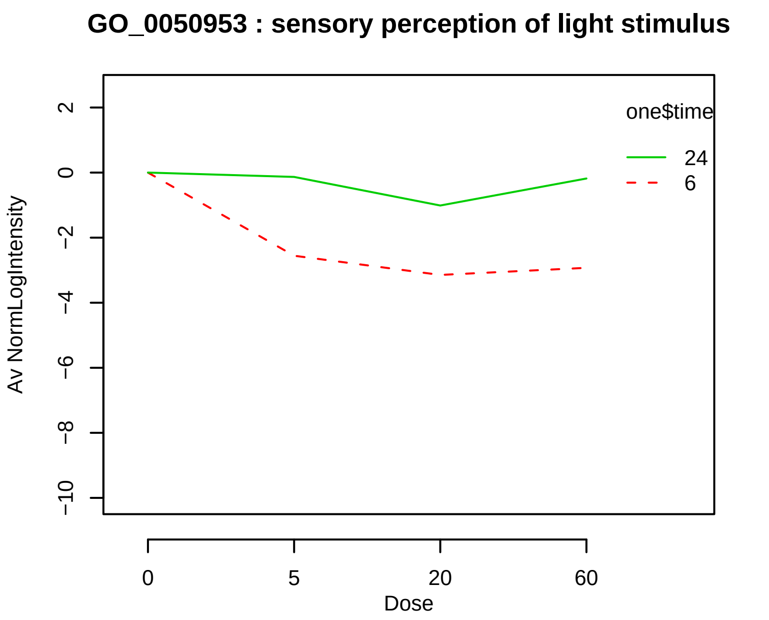
GO_0050953 : sensory perception of light stimulus
one$time
24
6
Av NormLogIntensity
2
0
−2
−4
−6
−8
−10
0
5
20
60
Dose

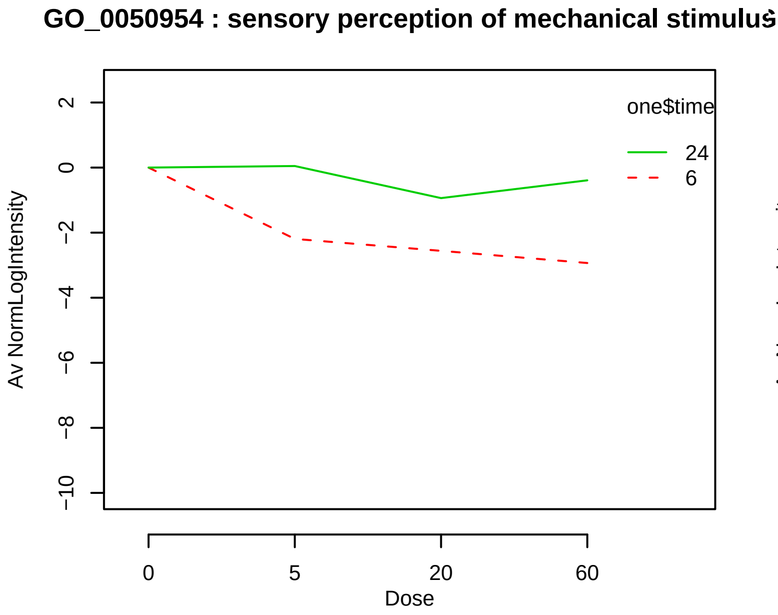
GO_0050954 : sensory perception of mechanical stimulus
one$time
24
6
Av NormLogIntensity
2
0
−2
−4
−6
−8
−10
0
5
20
60
Dose

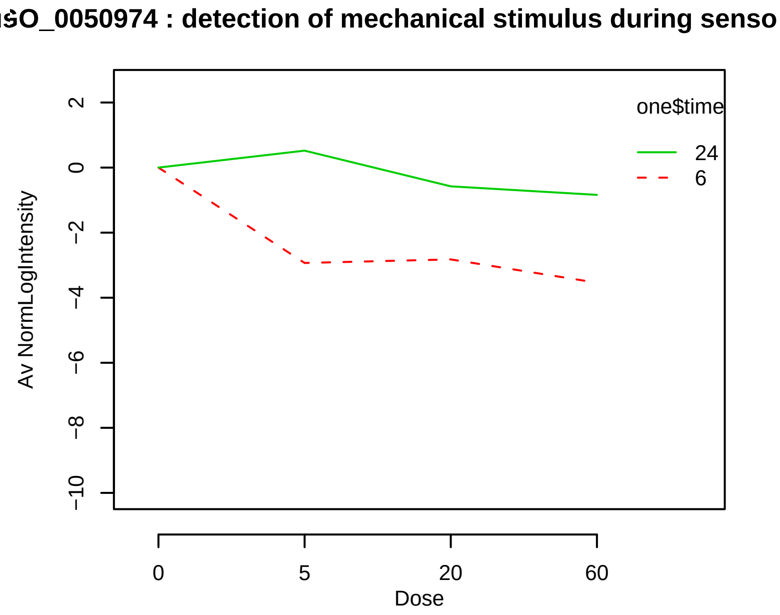
O_0050974 : detection of mechanical stimulus during senso
one$time
24
6
Av NormLogIntensity
2
0
−2
−4
−6
−8
−10
0
5
20
60
Dose

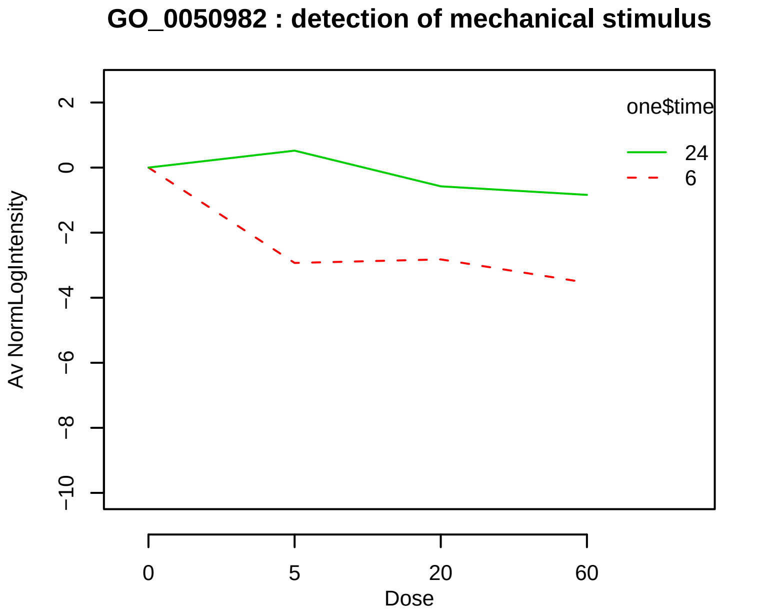
GO_0050982 : detection of mechanical stimulus
one$time
24
6
Av NormLogIntensity
2
0
−2
−4
−6
−8
−10
0
5
20
60
Dose

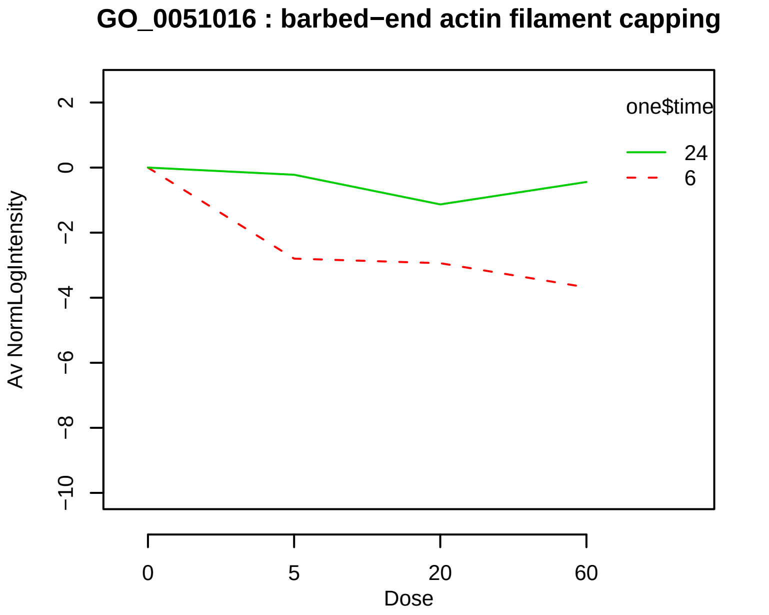
GO_0051016 : barbed−end actin filament capping
one$time
24
6
Av NormLogIntensity
2
0
−2
−4
−6
−8
−10
0
5
20
60
Dose

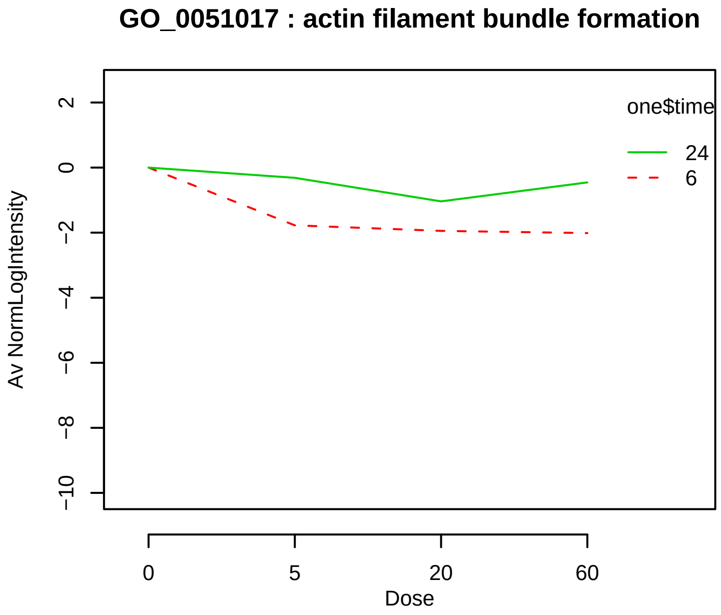
GO_0051017 : actin filament bundle formation
Av NormLogIntensity
2
0
−2
−4
−6
−8
−10
0
5
20
60
Dose
one$time
24
6

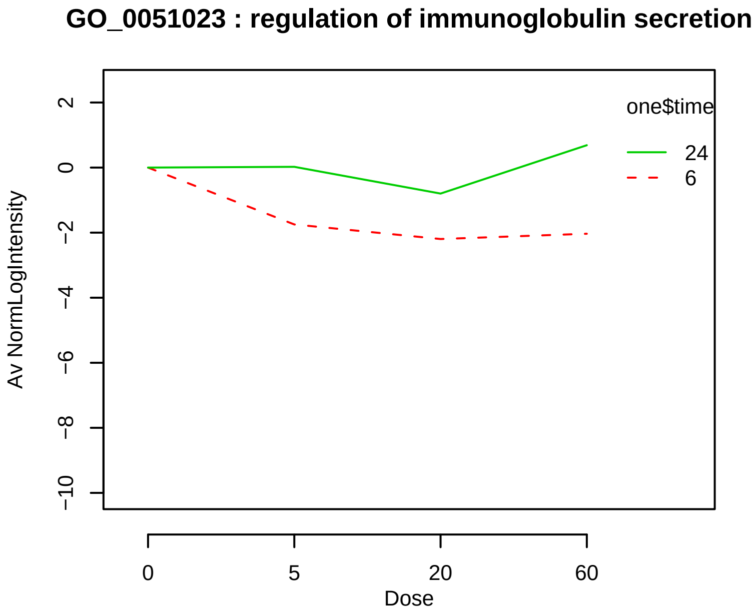
GO_0051023 : regulation of immunoglobulin secretion
Av NormLogIntensity
2
0
−2
−4
−6
−8
−10
0
5
20
60
Dose
one$time
24
6

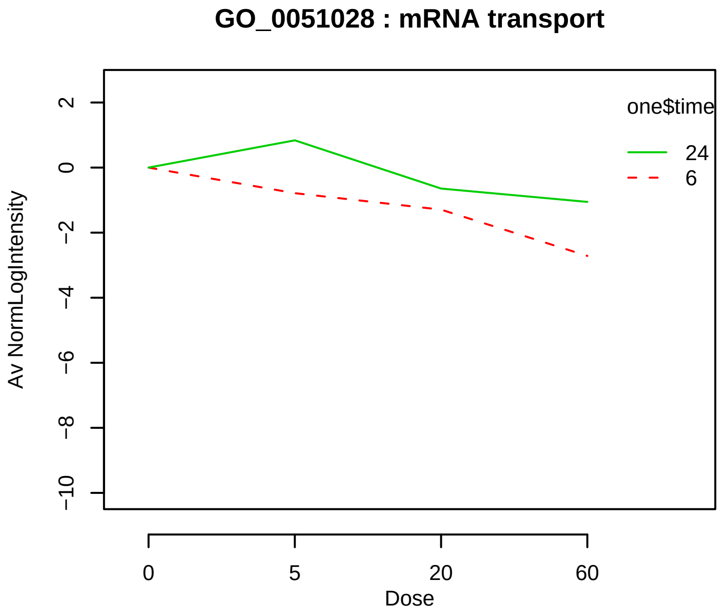
GO_0051028 : mRNA transport
Av NormLogIntensity
2
0
−2
−4
−6
−8
−10
0
5
20
60
Dose
one$time
24
6

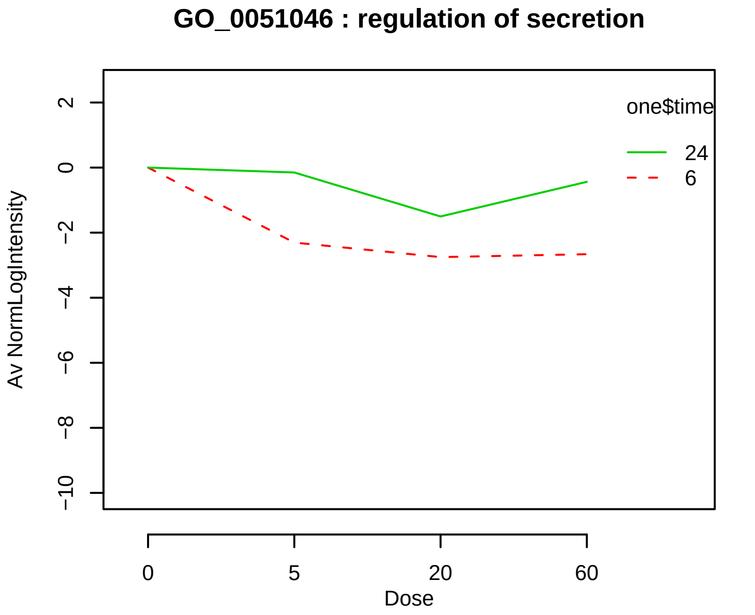
GO_0051046 : regulation of secretion
Av NormLogIntensity
2
0
−2
−4
−6
−8
−10
0
5
20
60
Dose
one$time
24
6

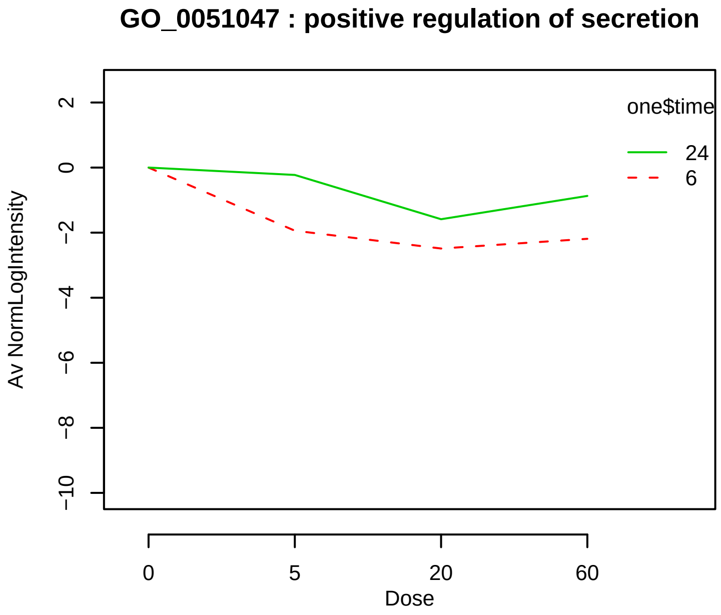
GO_0051047 : positive regulation of secretion
Av NormLogIntensity
2
0
−2
−4
−6
−8
−10
0
5
20
60
Dose
one$time
24
6

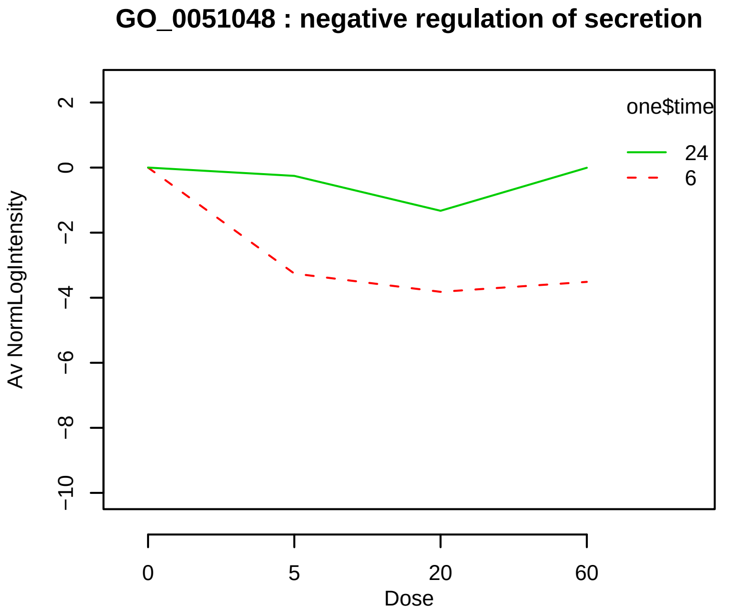
GO_0051048 : negative regulation of secretion
Av NormLogIntensity
2
0
−2
−4
−6
−8
−10
0
5
20
60
Dose
one$time
24
6

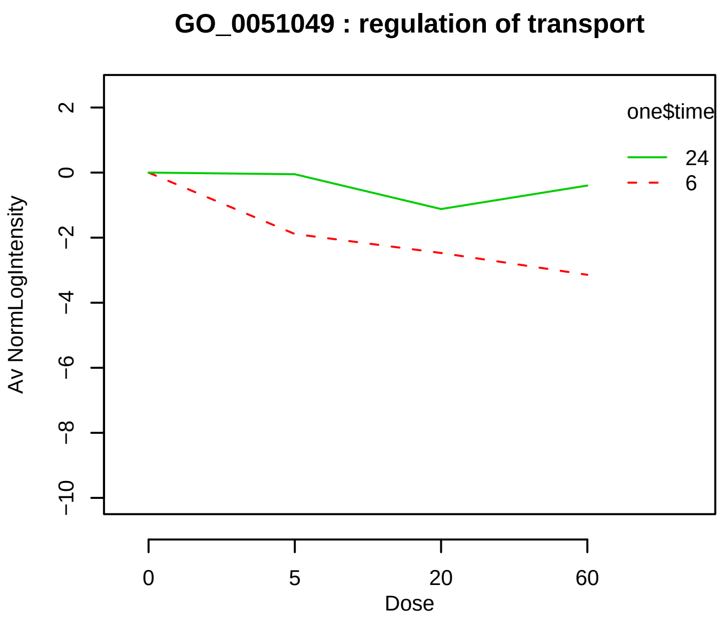
GO_0051049 : regulation of transport
one$time
24
6
Av NormLogIntensity
2
0
−2
−4
−6
−8
−10
0
5
20
60
Dose

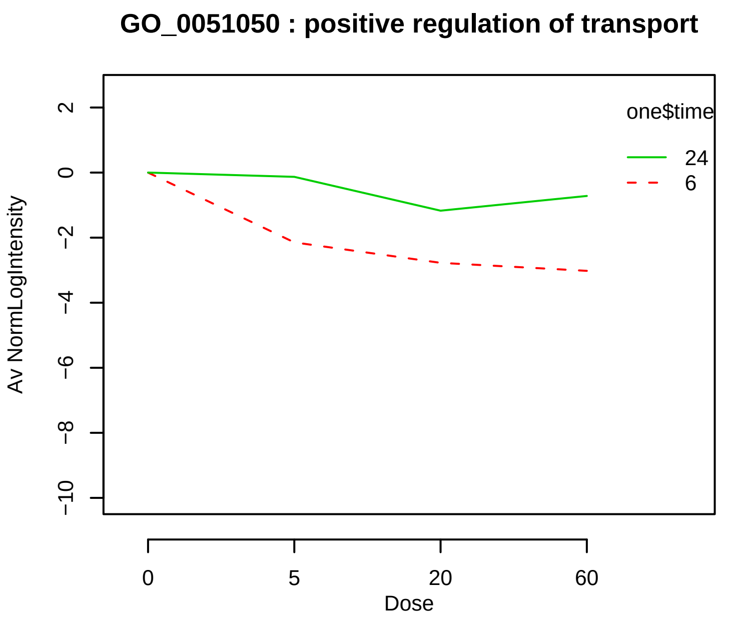
GO_0051050 : positive regulation of transport
one$time
24
6
Av NormLogIntensity
2
0
−2
−4
−6
−8
−10
0
5
20
60
Dose

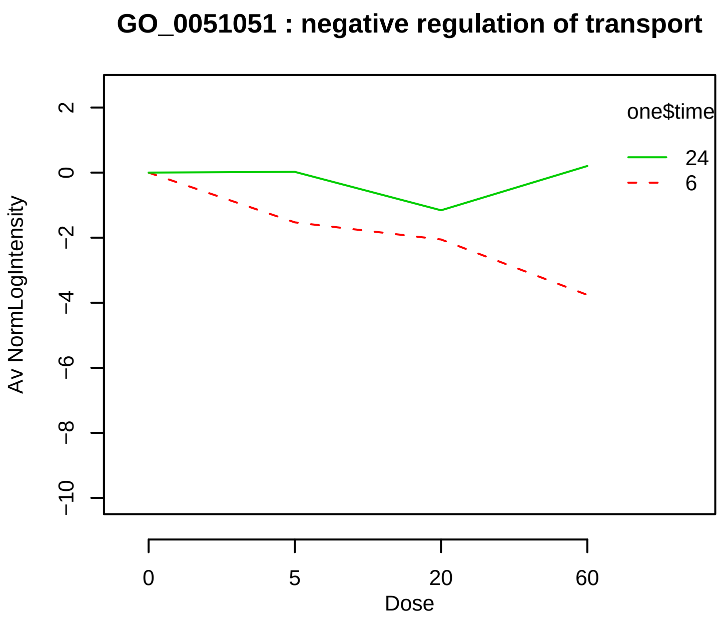
GO_0051051 : negative regulation of transport
one$time
24
6
Av NormLogIntensity
2
0
−2
−4
−6
−8
−10
0
5
20
60
Dose

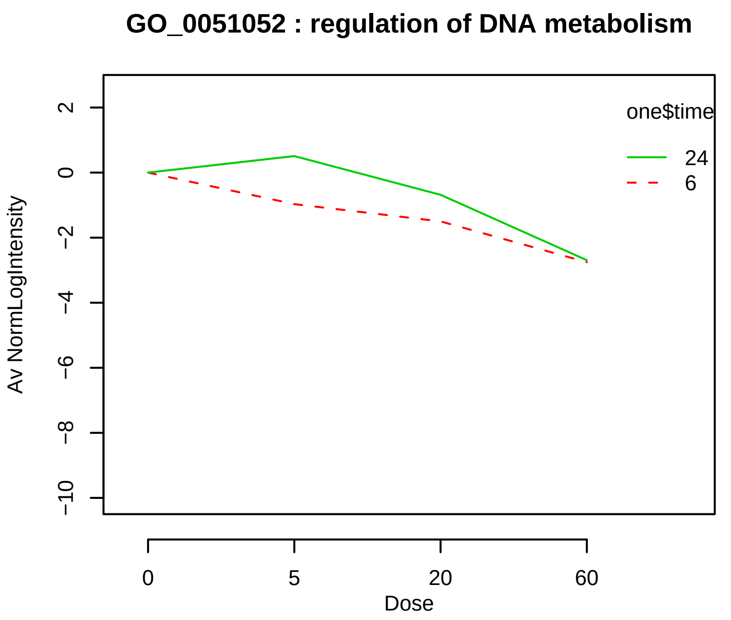
GO_0051052 : regulation of DNA metabolism
one$time
24
6
Av NormLogIntensity
2
0
−2
−4
−6
−8
−10
0
5
20
60
Dose

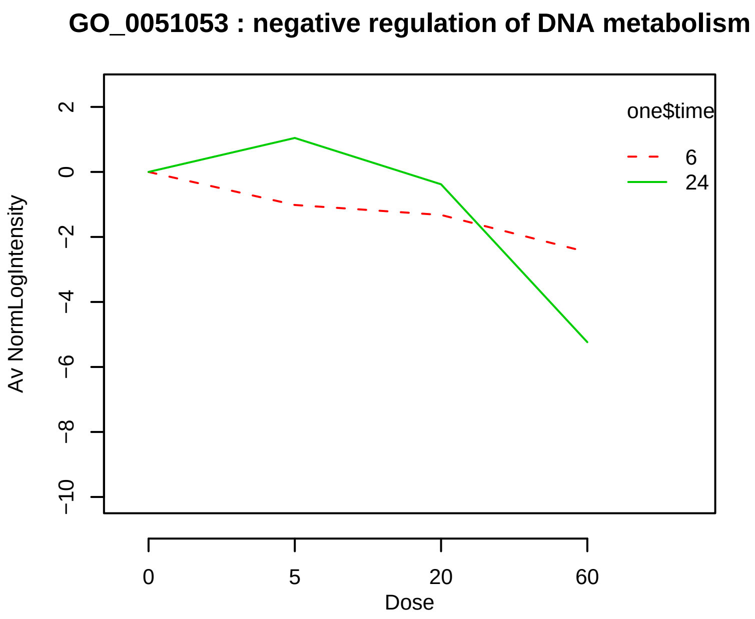
GO_0051053 : negative regulation of DNA metabolism
one$time
6
24
Av NormLogIntensity
2
0
−2
−4
−6
−8
−10
0
5
20
60
Dose

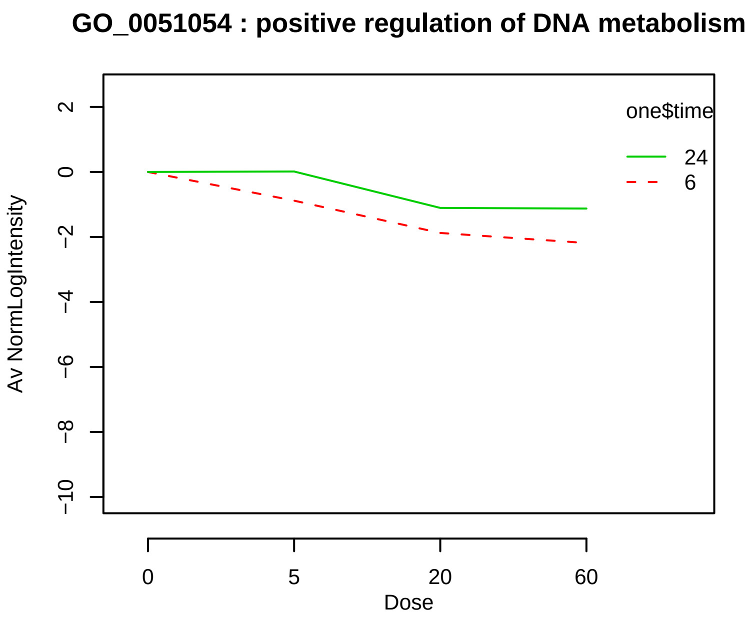
GO_0051054 : positive regulation of DNA metabolism
one$time
24
6
Av NormLogIntensity
2
0
−2
−4
−6
−8
−10
0
5
20
60
Dose

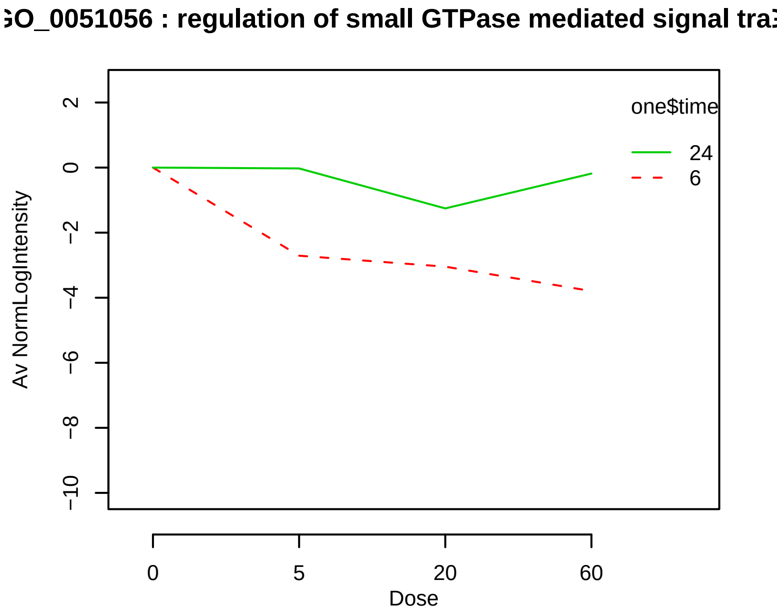

GO_0051056 : regulation of small GTPase mediated signal tra
one$time
24
6
Av NormLogIntensity
Dose

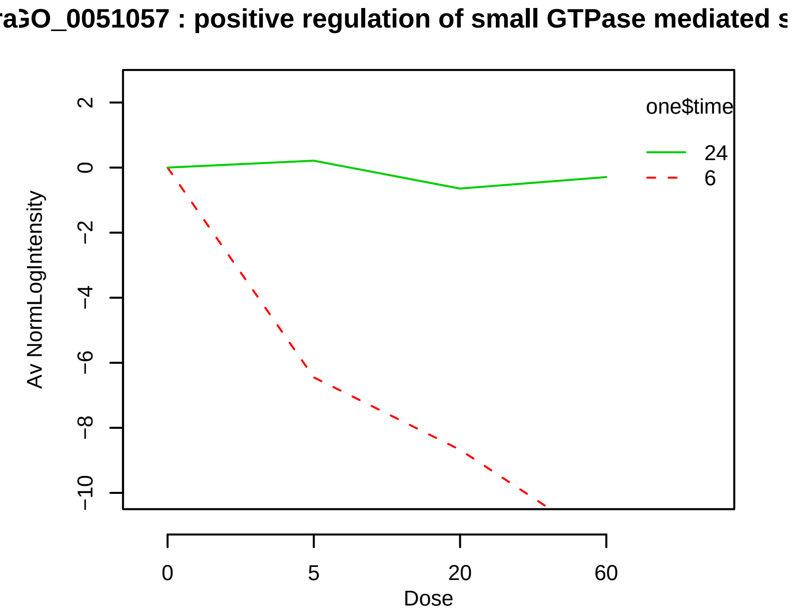

GO_0051057 : positive regulation of small GTPase mediated s
one$time
24
6
Av NormLogIntensity
Dose

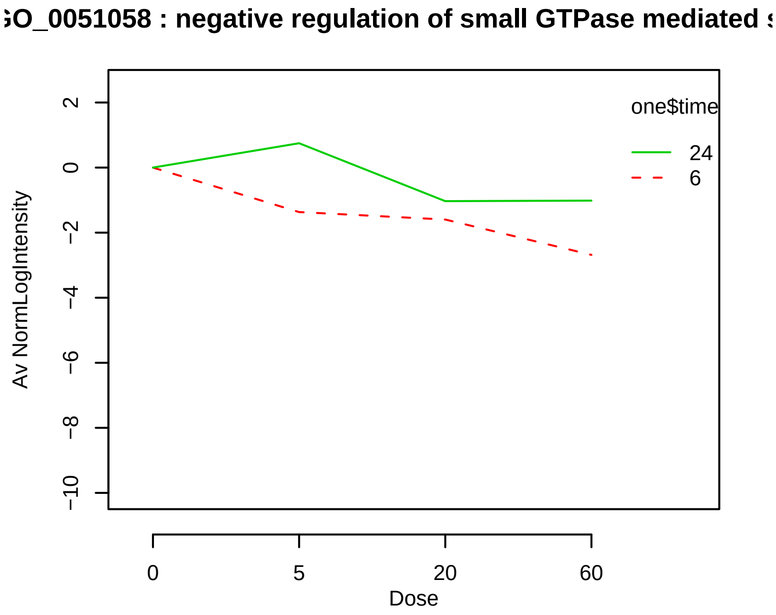

GO_0051058 : negative regulation of small GTPase mediated s
one$time
24
6
Av NormLogIntensity
Dose

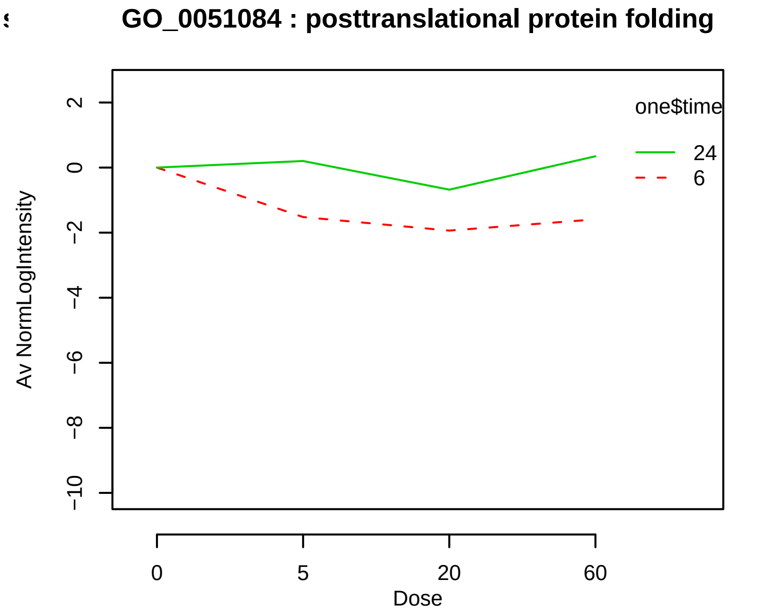

GO_0051084 : posttranslational protein folding
one$time
24
6
Av NormLogIntensity
Dose

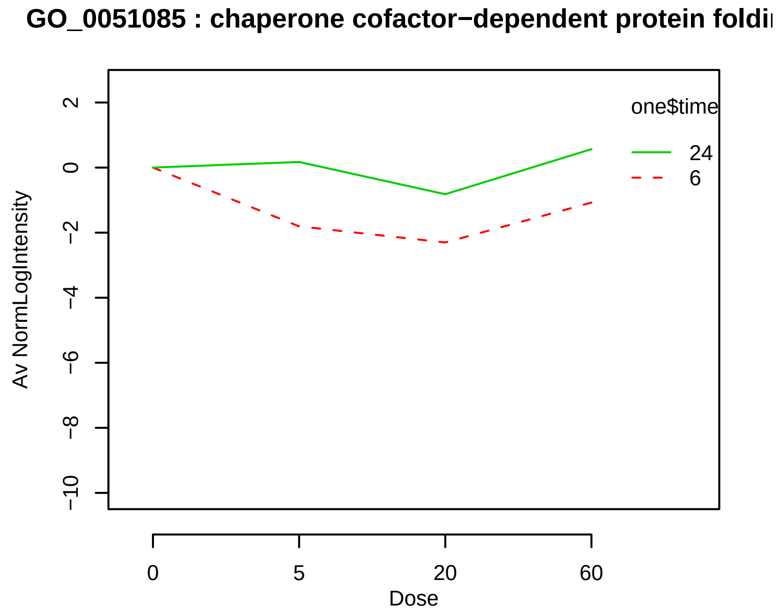

GO_0051085 : chaperone cofactor−dependent protein foldi
one$time
24
6
Av NormLogIntensity
Dose

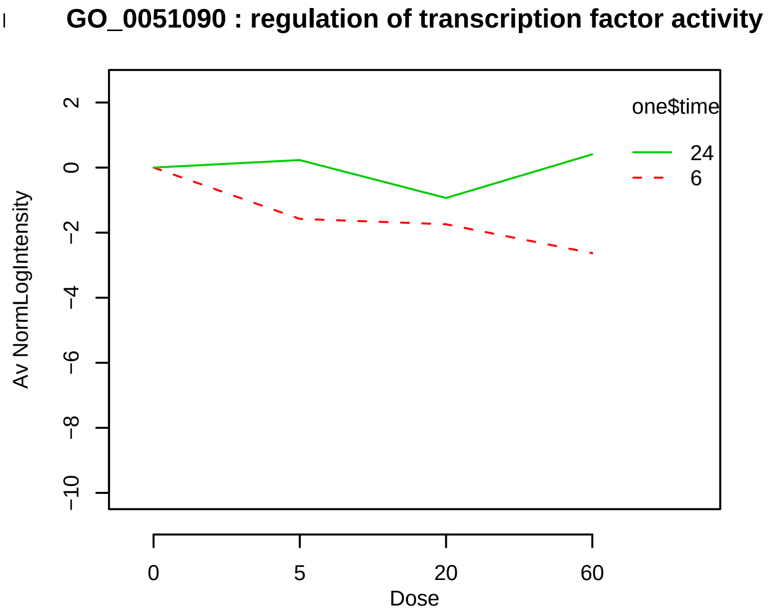

GO_0051090 : regulation of transcription factor activity
one$time
24
6
Av NormLogIntensity
Dose

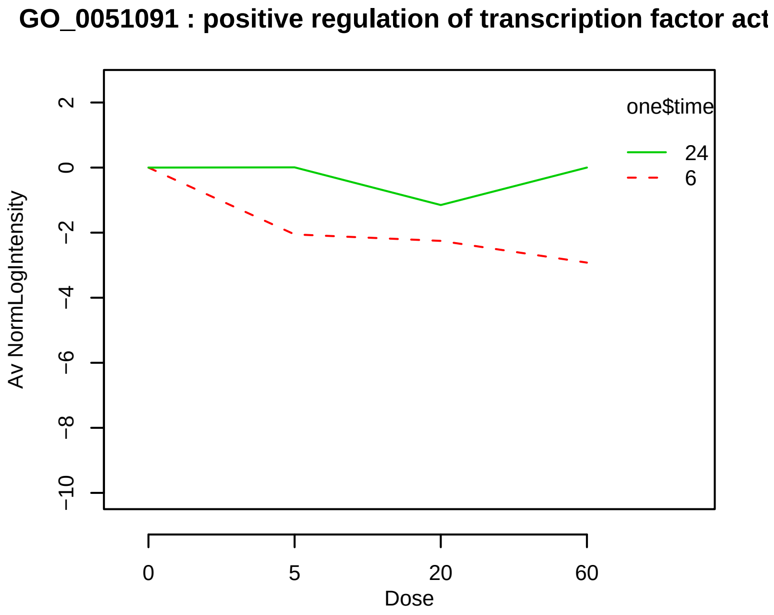
GO_0051091 : positive regulation of transcription factor act
one$time
24
6
Av NormLogIntensity
2
0
−2
−4
−6
−8
−10
0
5
20
60
Dose

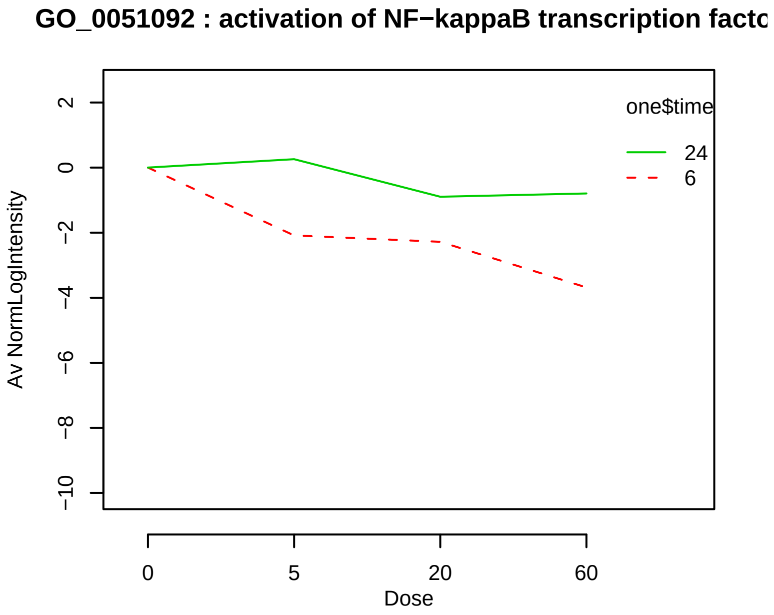
GO_0051092 : activation of NF−kappaB transcription facto
one$time
24
6
Av NormLogIntensity
2
0
−2
−4
−6
−8
−10
0
5
20
60
Dose

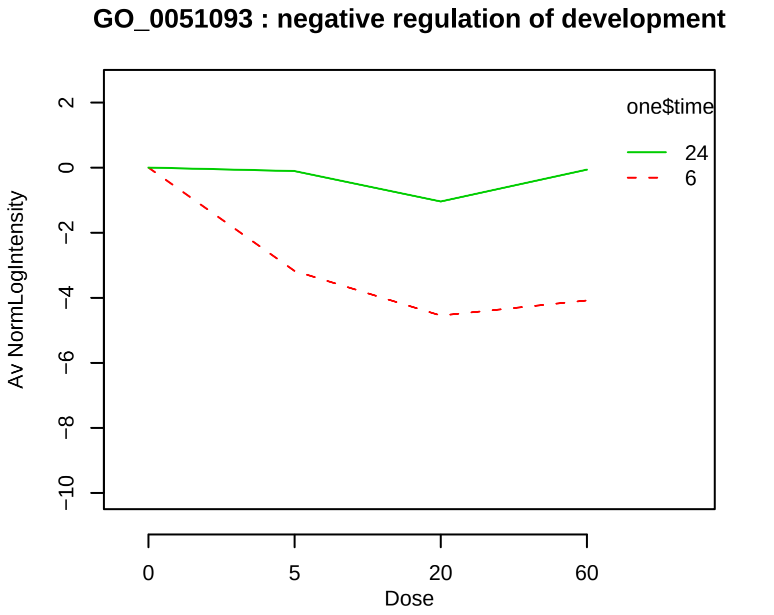
GO_0051093 : negative regulation of development
one$time
24
6
Av NormLogIntensity
2
0
−2
−4
−6
−8
−10
0
5
20
60
Dose

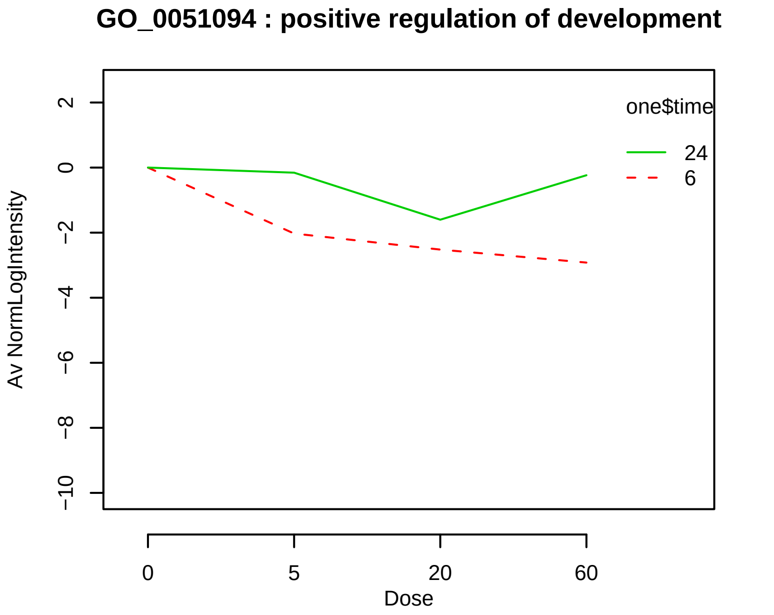
GO_0051094 : positive regulation of development
one$time
24
6
Av NormLogIntensity
2
0
−2
−4
−6
−8
−10
0
5
20
60
Dose

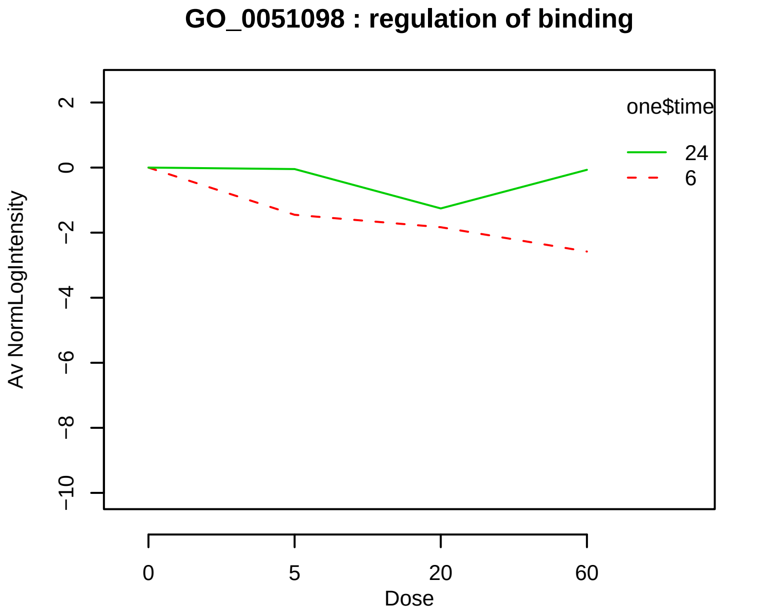
GO_0051098 : regulation of binding
one$time
24
6
Av NormLogIntensity
2
0
−2
−4
−6
−8
−10
0
5
20
60
Dose

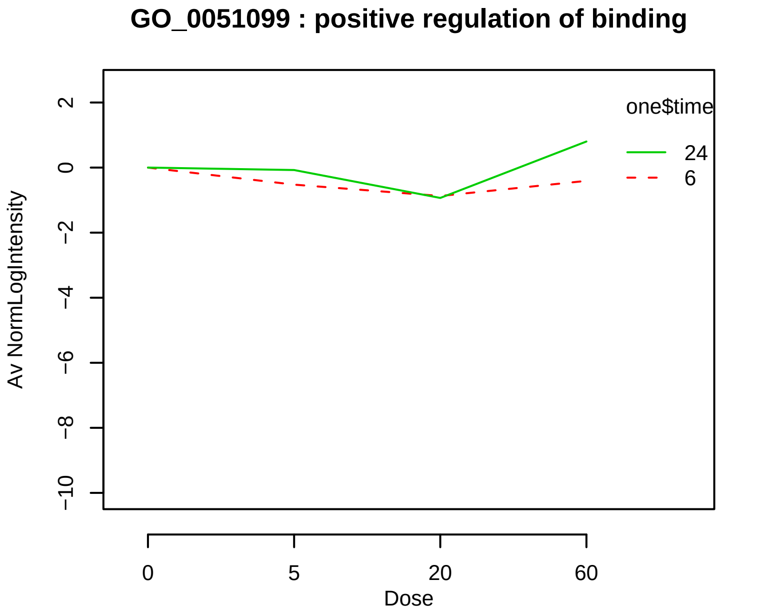
GO_0051099 : positive regulation of binding
one$time
24
6
Av NormLogIntensity
2
0
−2
−4
−6
−8
−10
0
5
20
60
Dose

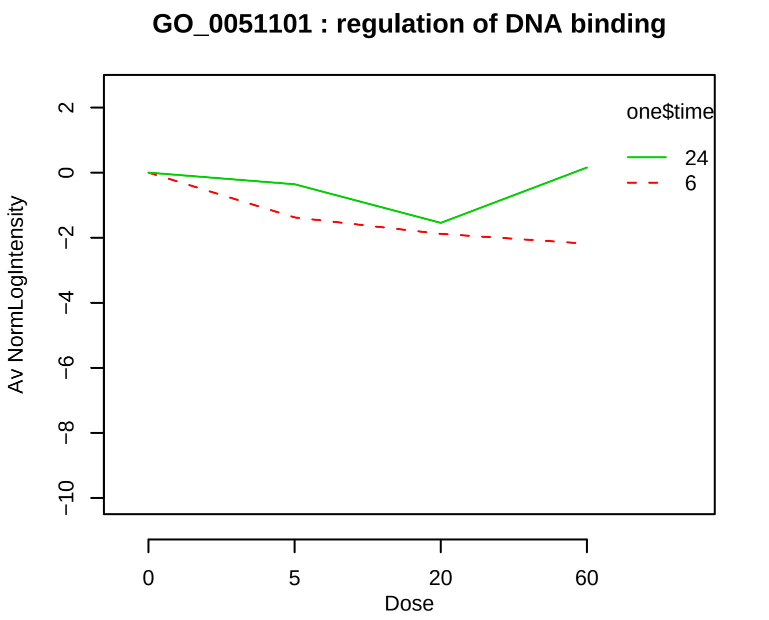
GO_0051101 : regulation of DNA binding
one$time
24
6
Av NormLogIntensity
Dose
0
5
20
60
2
0
−2
−4
−6
−8
−10

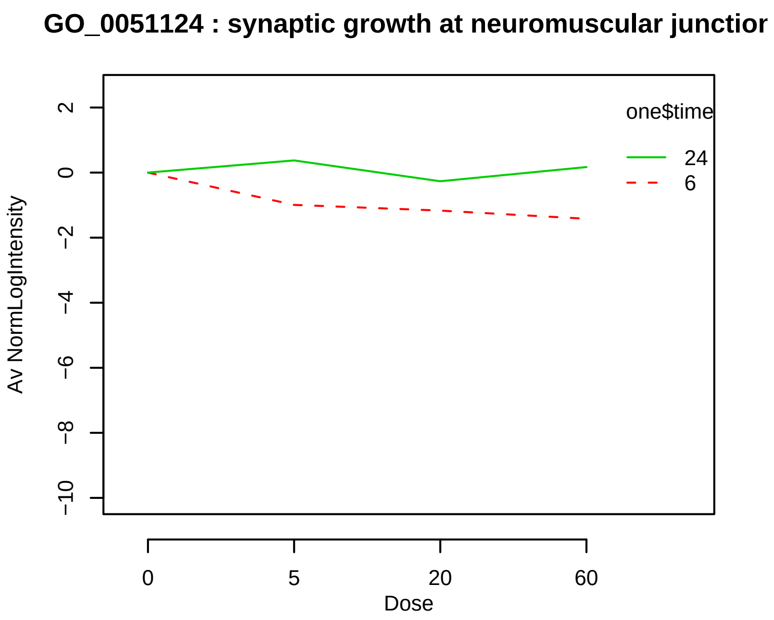
GO_0051124 : synaptic growth at neuromuscular junctior
one$time
24
6
Av NormLogIntensity
Dose
0
5
20
60
2
0
−2
−4
−6
−8
−10

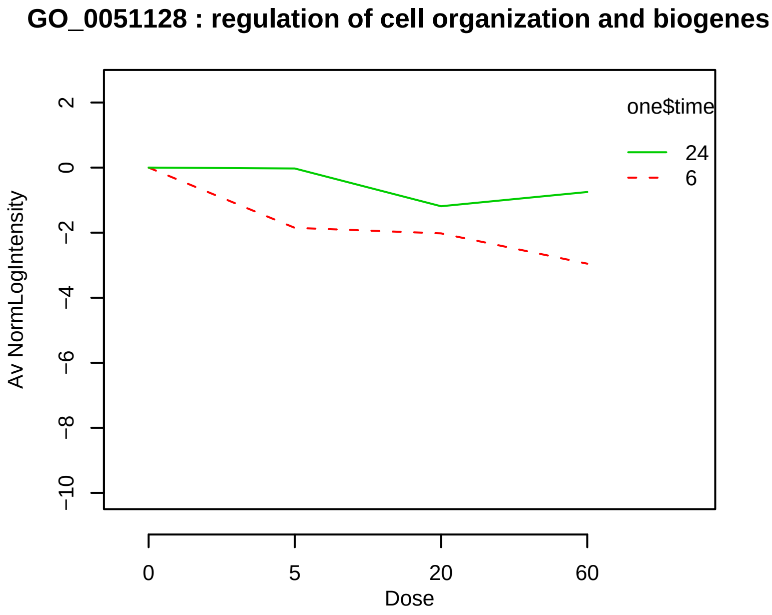
GO_0051128 : regulation of cell organization and biogenes
one$time
24
6
Av NormLogIntensity
Dose
0
5
20
60
2
0
−2
−4
−6
−8
−10

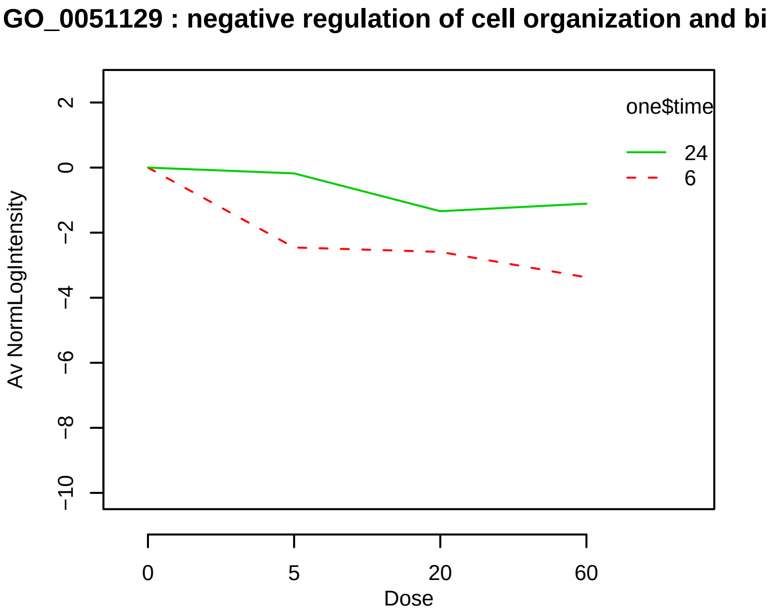
GO_0051129 : negative regulation of cell organization and bi
one$time
24
6
Av NormLogIntensity
Dose
0
5
20
60
2
0
−2
−4
−6
−8
−10

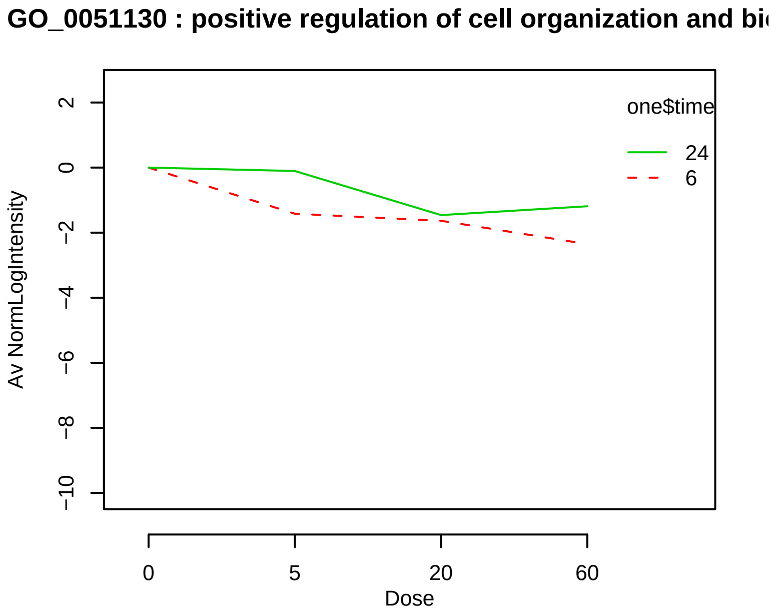
GO_0051130 : positive regulation of cell organization and bi
one$time
24
6
Av NormLogIntensity
Dose
0
5
20
60
2
0
−2
−4
−6
−8
−10

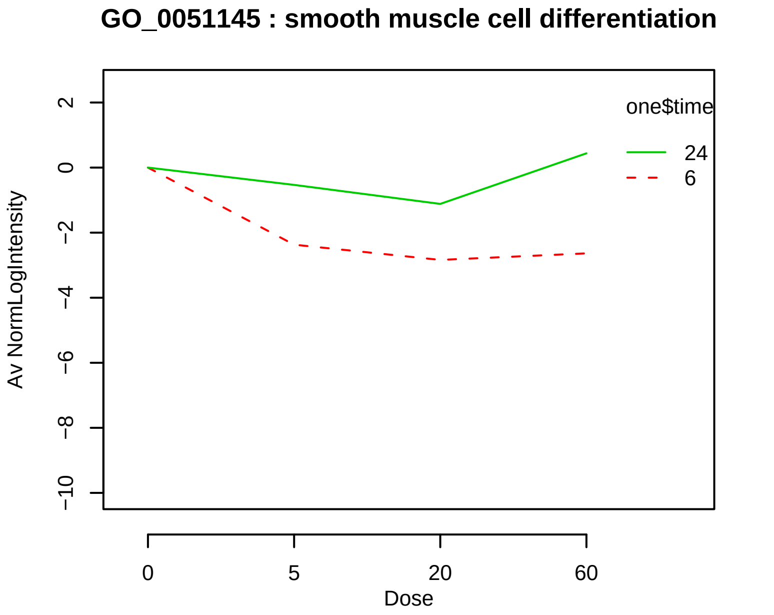
GO_0051145 : smooth muscle cell differentiation
one$time
24
6
Av NormLogIntensity
Dose
0
5
20
60
2
0
−2
−4
−6
−8
−10

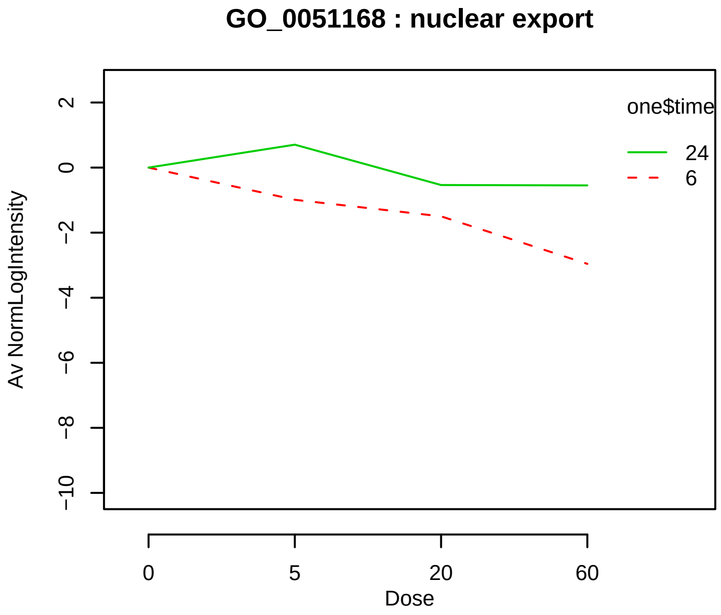
GO_0051168 : nuclear export
Av NormLogIntensity
Dose
one$time
24
6

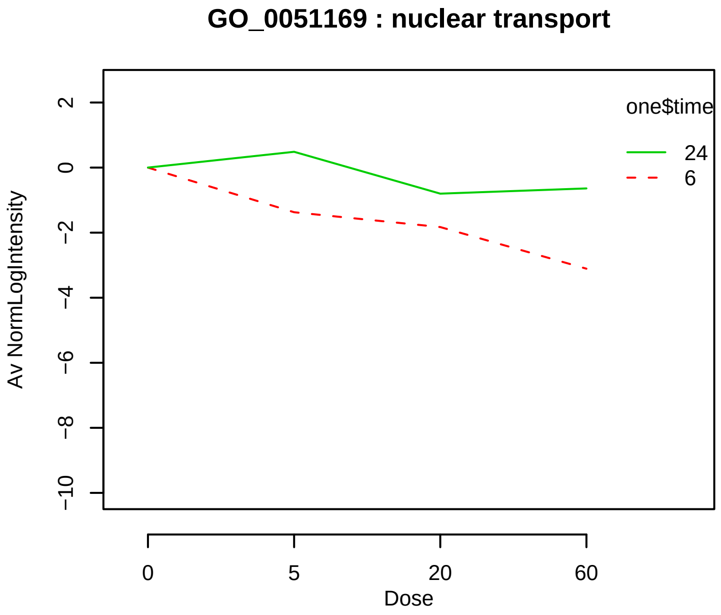
GO_0051169 : nuclear transport
Av NormLogIntensity
Dose
one$time
24
6

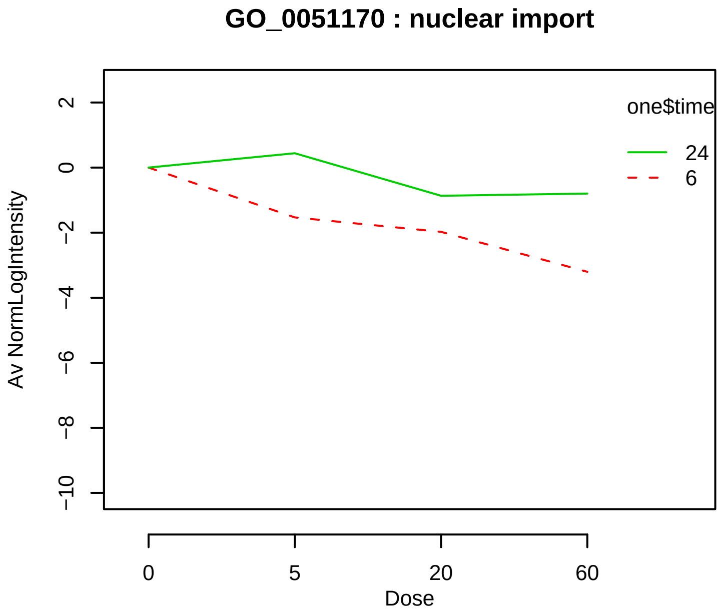
GO_0051170 : nuclear import
Av NormLogIntensity
Dose
one$time
24
6

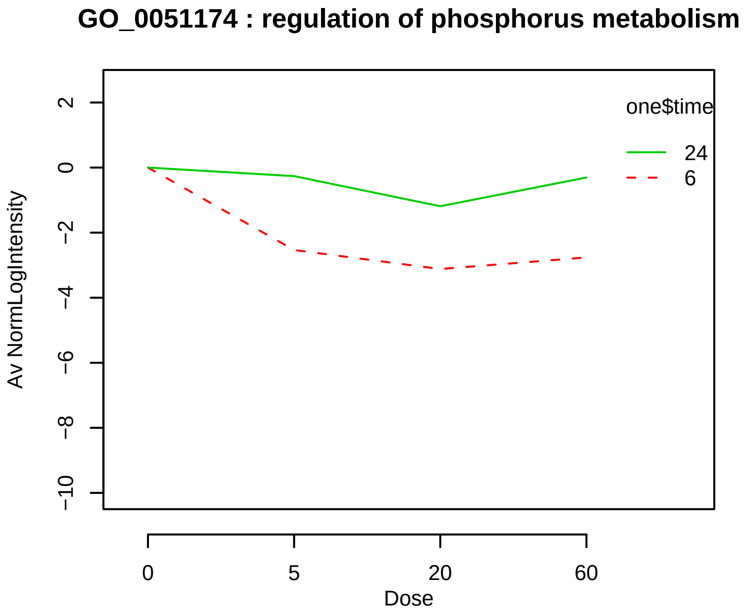
GO_0051174 : regulation of phosphorus metabolism
Av NormLogIntensity
Dose
one$time
24
6

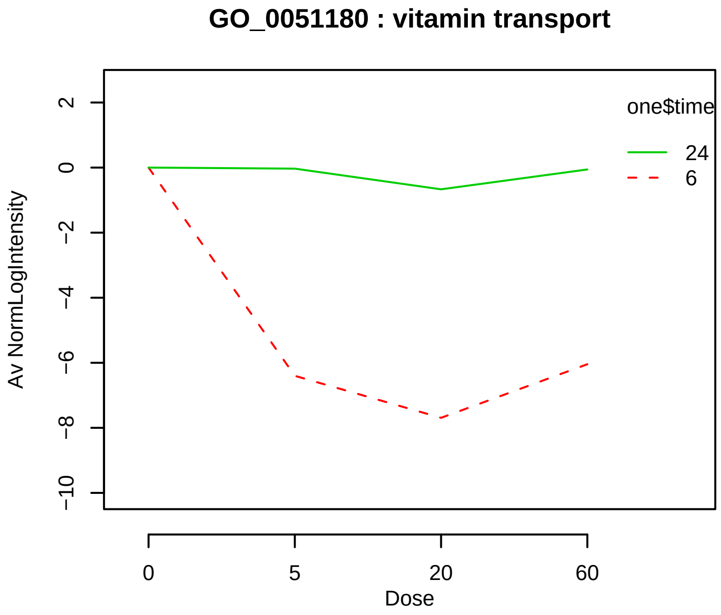
GO_0051180 : vitamin transport
Av NormLogIntensity
Dose
one$time
24
6

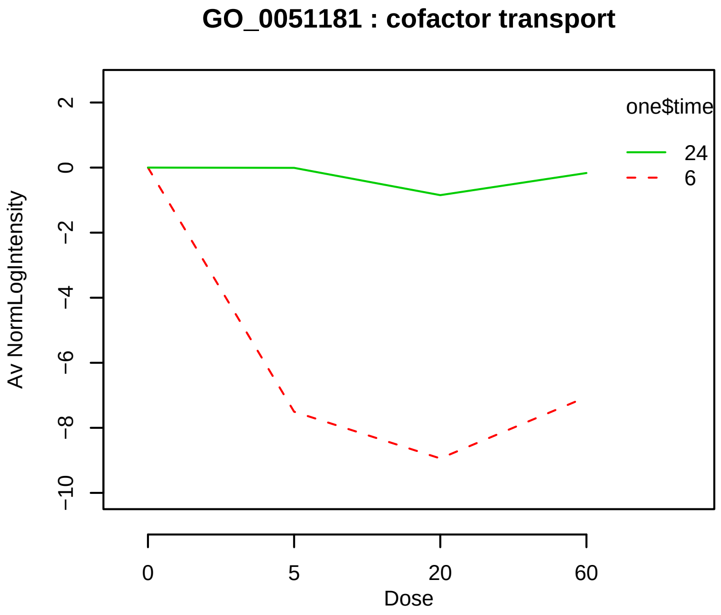
GO_0051181 : cofactor transport
Av NormLogIntensity
Dose
one$time
24
6

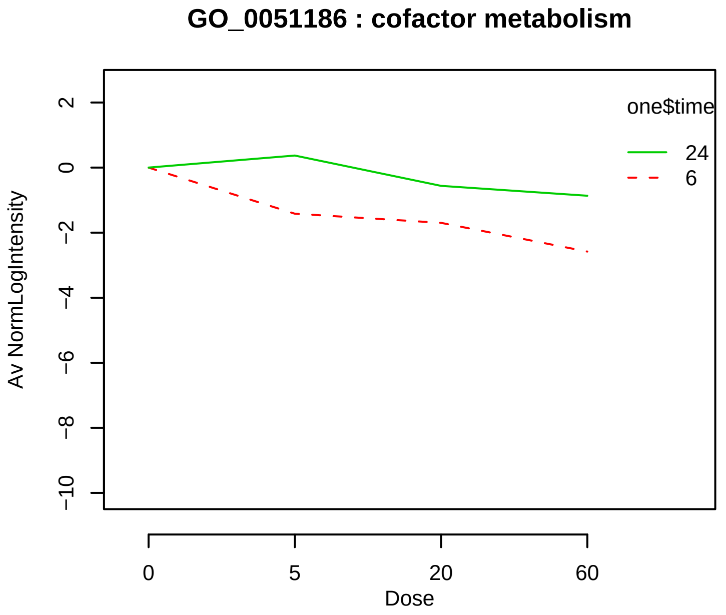
GO_0051186 : cofactor metabolism
one$time
24
6
Av NormLogIntensity
2
0
−2
−4
−6
−8
−10
0
5
20
60
Dose

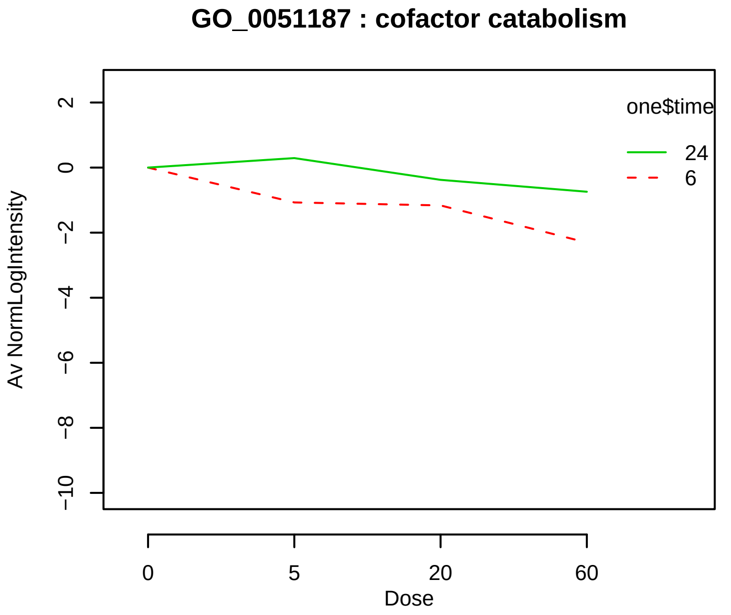
GO_0051187 : cofactor catabolism
one$time
24
6
Av NormLogIntensity
2
0
−2
−4
−6
−8
−10
0
5
20
60
Dose

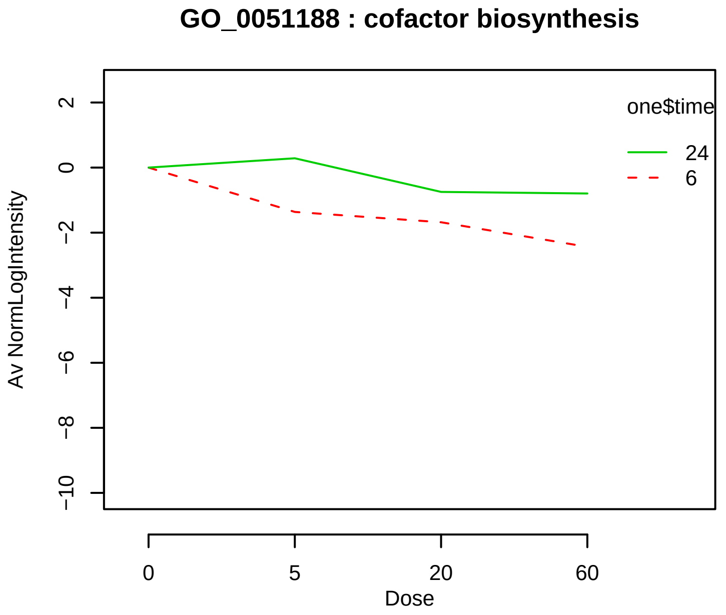
GO_0051188 : cofactor biosynthesis
one$time
24
6
Av NormLogIntensity
2
0
−2
−4
−6
−8
−10
0
5
20
60
Dose

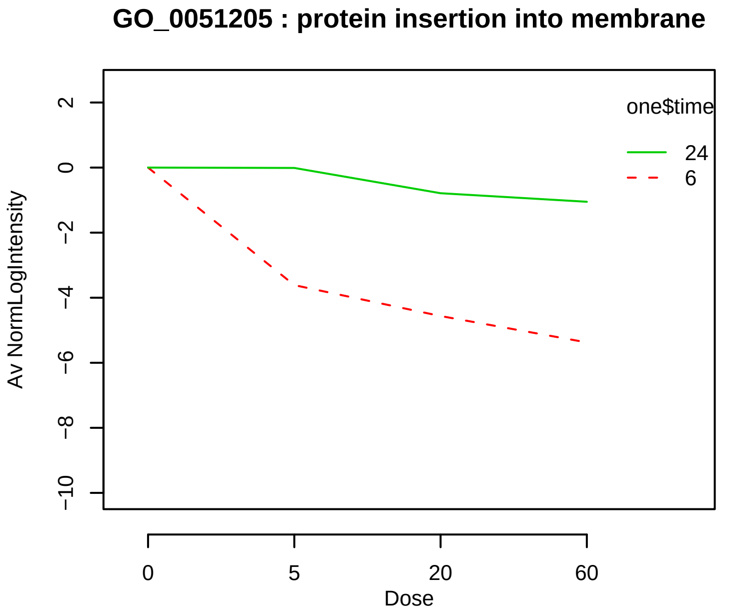
GO_0051205 : protein insertion into membrane
one$time
24
6
Av NormLogIntensity
2
0
−2
−4
−6
−8
−10
0
5
20
60
Dose

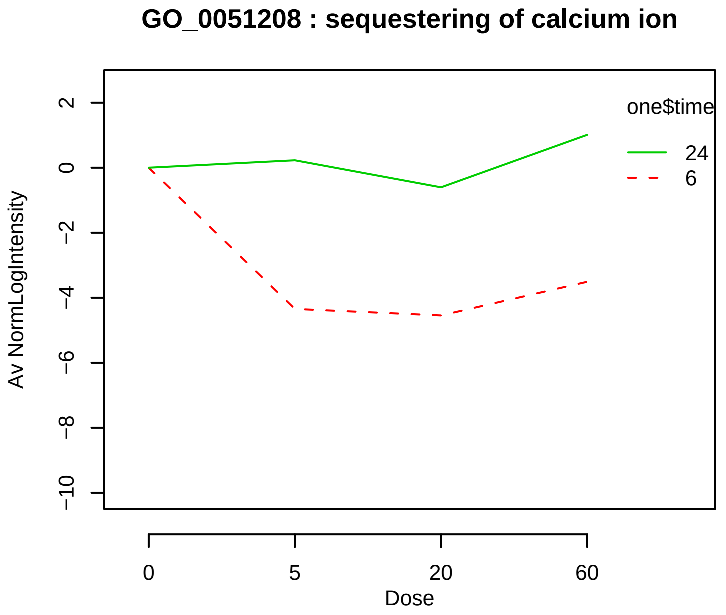
GO_0051208 : sequestering of calcium ion
one$time
24
6
Av NormLogIntensity
2
0
−2
−4
−6
−8
−10
0
5
20
60
Dose

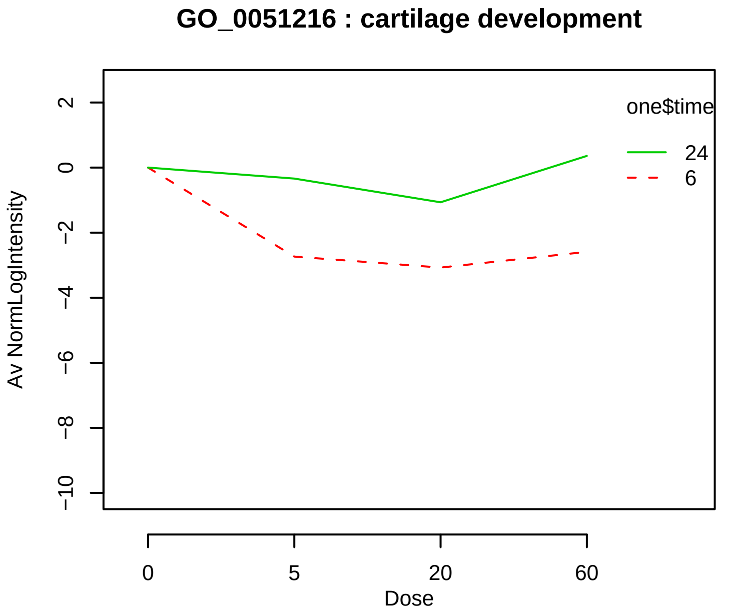
GO_0051216 : cartilage development
one$time
24
6
Av NormLogIntensity
2
0
−2
−4
−6
−8
−10
0
5
20
60
Dose

GO_0051220 : cytoplasmic sequestering of protein

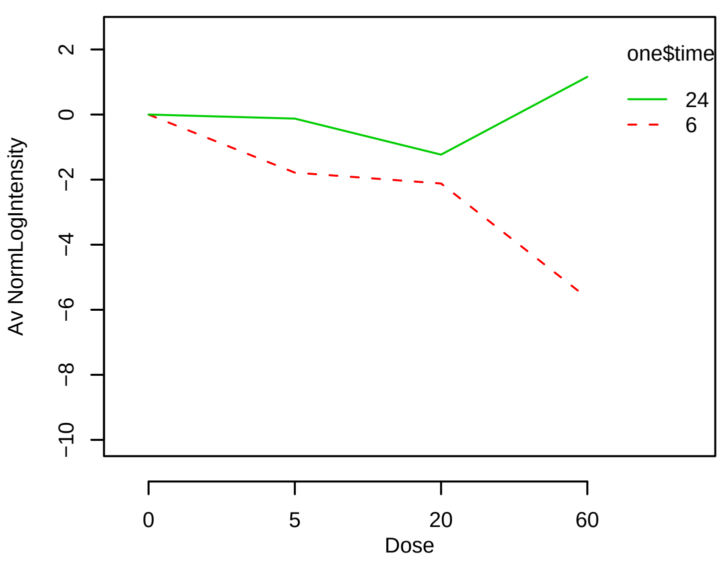

GO_0051222 : positive regulation of protein transport

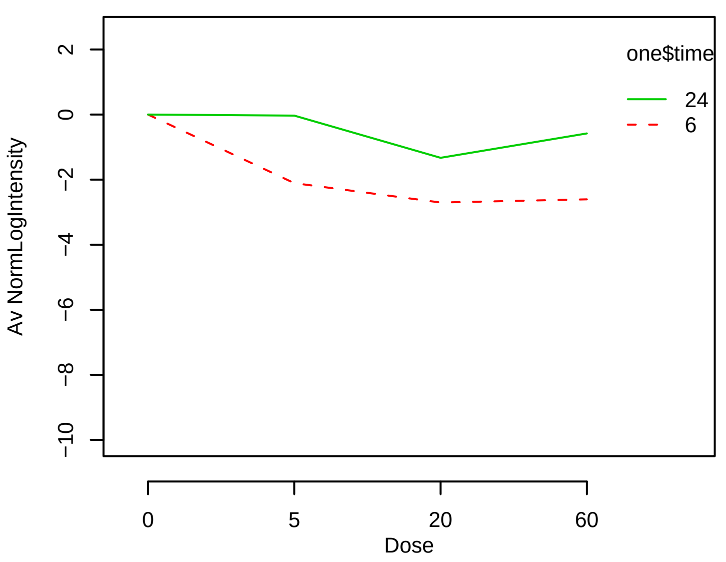

GO_0051223 : regulation of protein transport

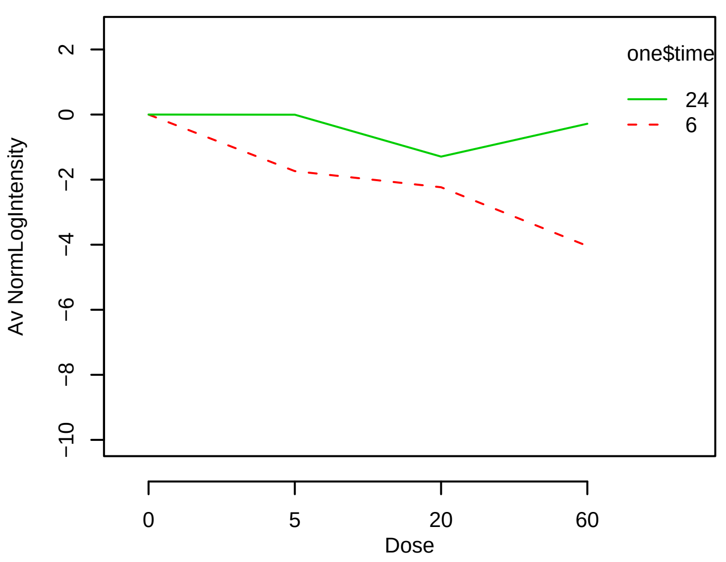

GO_0051224 : negative regulation of protein transport

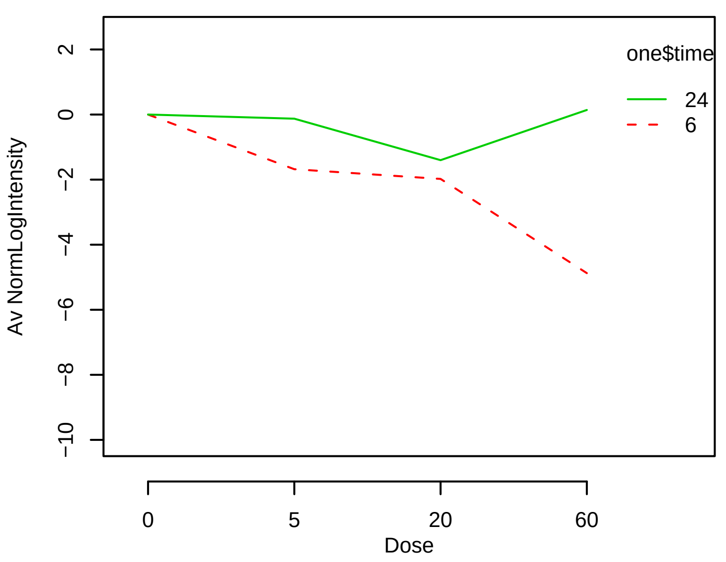

GO_0051235 : maintenance of localization

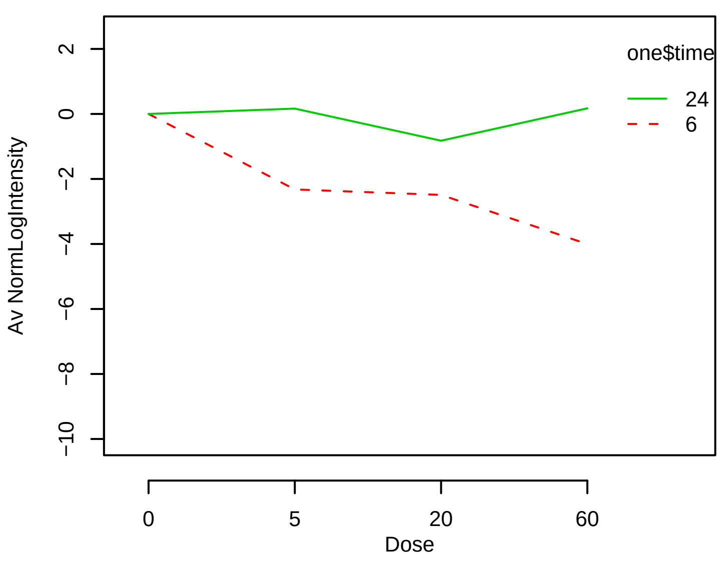

GO_0051236 : establishment of RNA localization

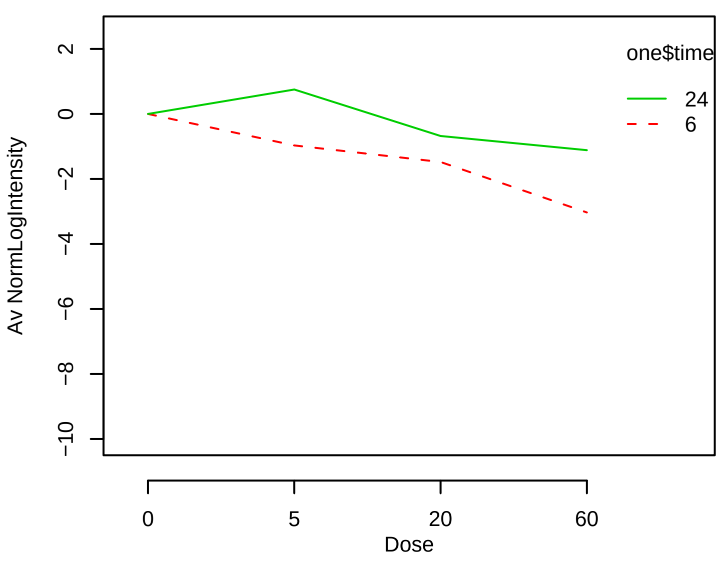

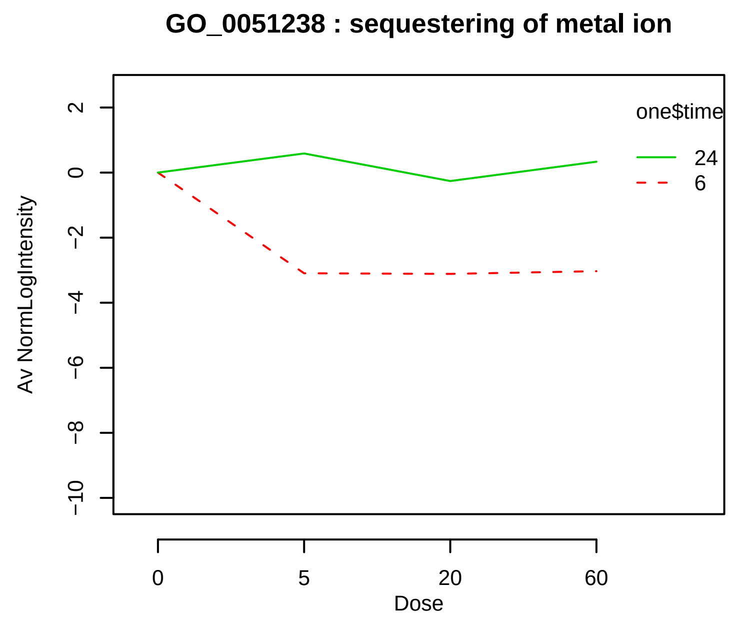

GO_0051238 : sequestering of metal ion
Av NormLogIntensity
Dose
one$time
24
6

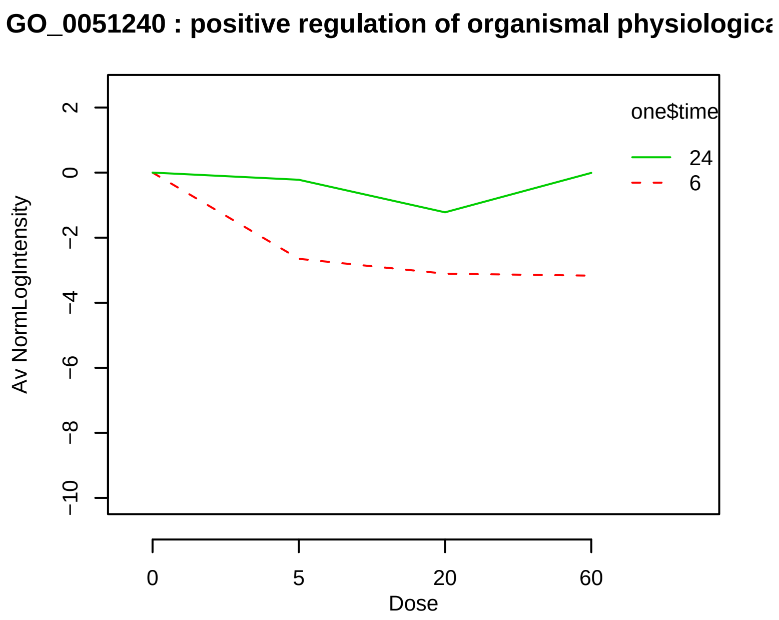

GO_0051240 : positive regulation of organismal physiologica
Av NormLogIntensity
Dose
one$time
24
6

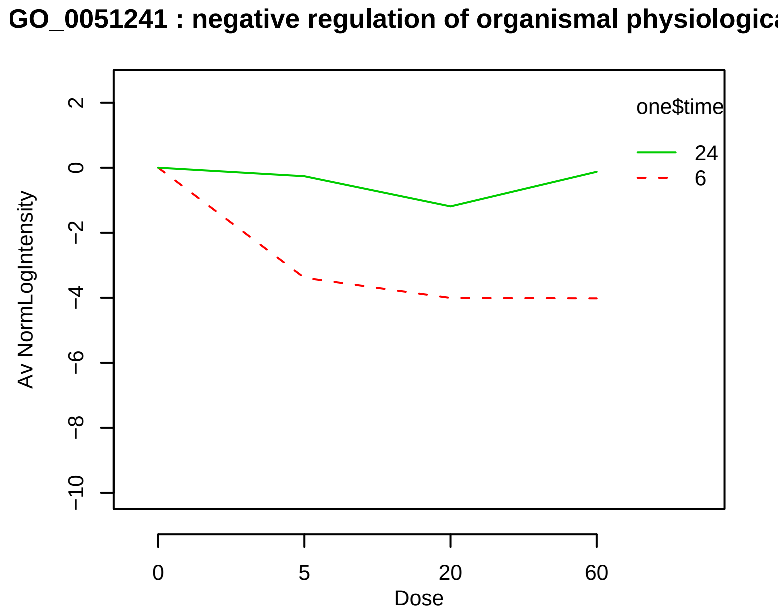

GO_0051241 : negative regulation of organismal physiologica
Av NormLogIntensity
Dose
one$time
24
6

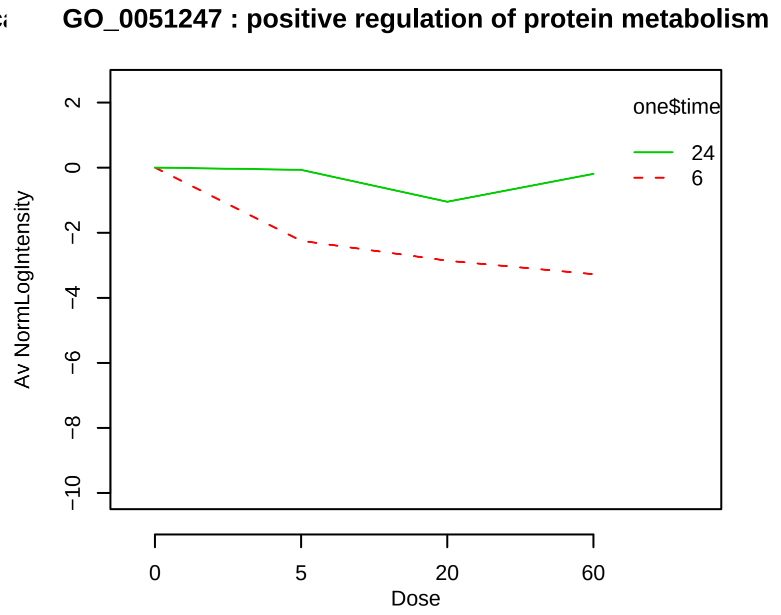

GO_0051247 : positive regulation of protein metabolism
Av NormLogIntensity
Dose
one$time
24
6

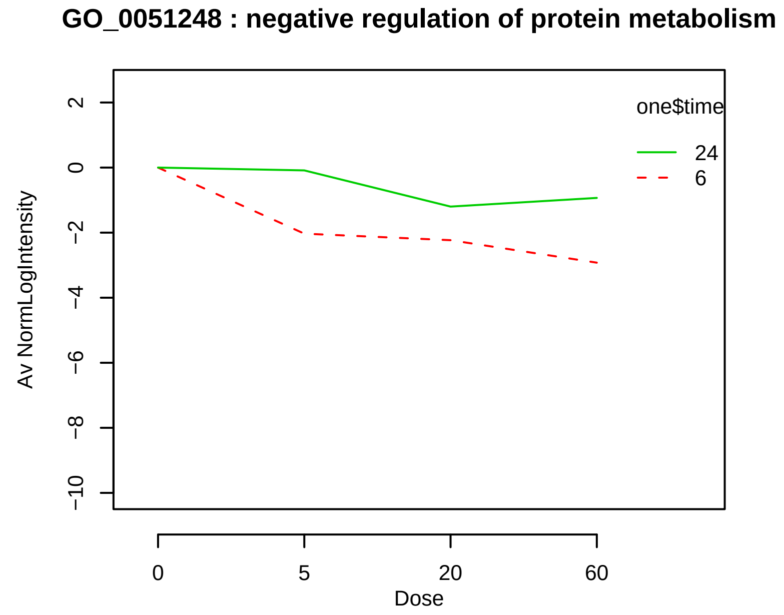

GO_0051248 : negative regulation of protein metabolism
Av NormLogIntensity
Dose
one$time
24
6

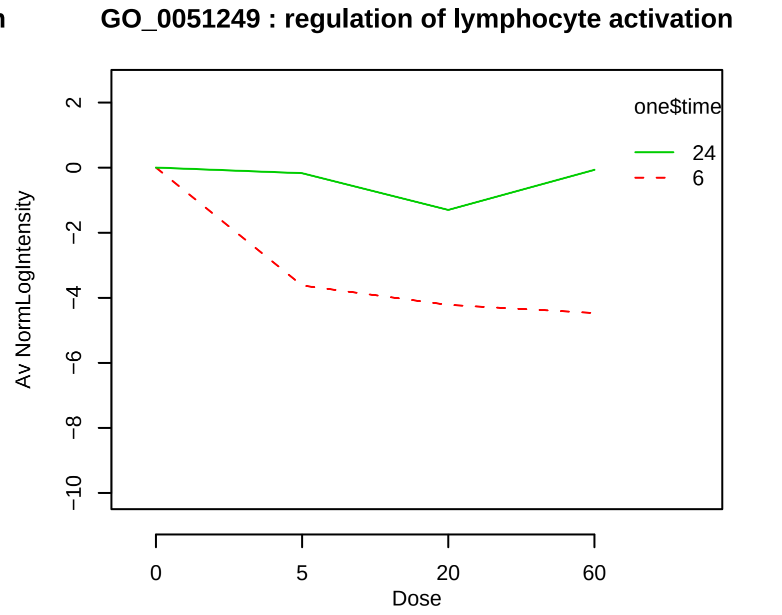

GO_0051249 : regulation of lymphocyte activation
Av NormLogIntensity
Dose
one$time
24
6

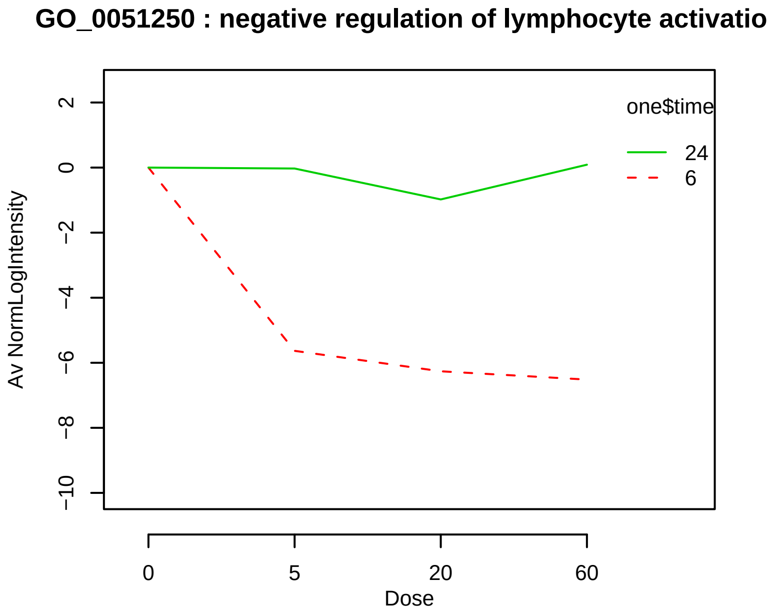
GO_0051250 : negative regulation of lymphocyte activatio
one$time
24
6
Av NormLogIntensity
2
0
−2
−4
−6
−8
−10
0
5
20
60
Dose
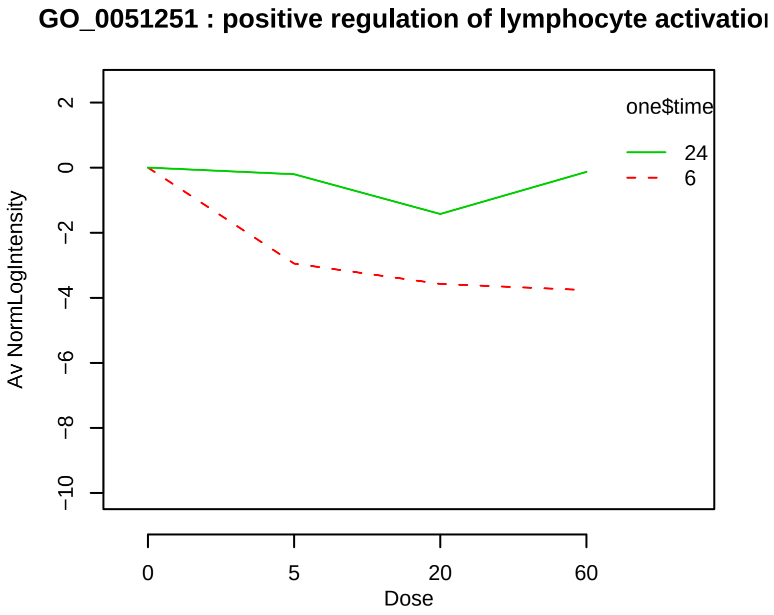
GO_0051251 : positive regulation of lymphocyte activatio
one$time
24
6
Av NormLogIntensity
2
0
−2
−4
−6
−8
−10
0
5
20
60
Dose
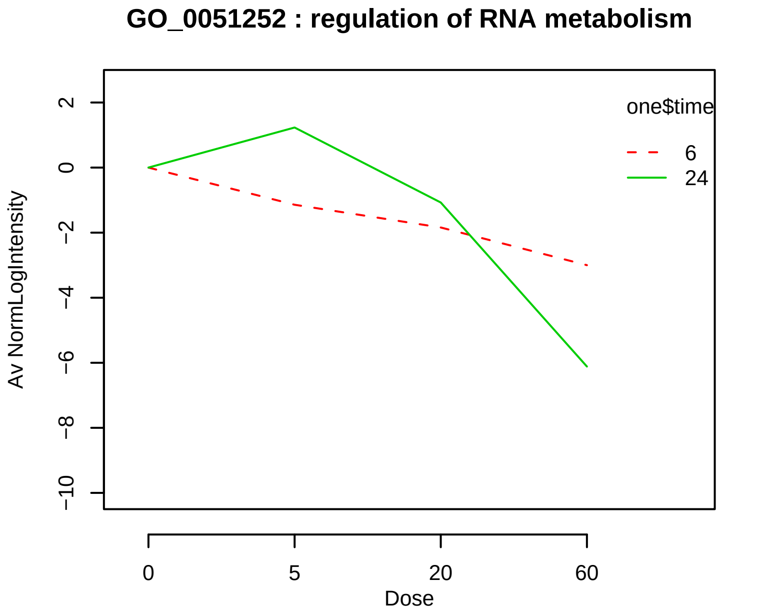
GO_0051252 : regulation of RNA metabolism
one$time
6
24
Av NormLogIntensity
2
0
−2
−4
−6
−8
−10
0
5
20
60
Dose
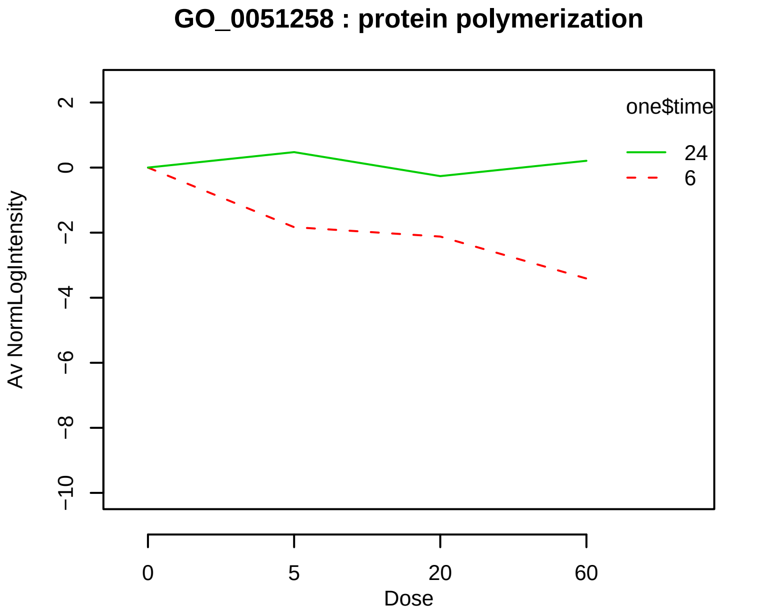
GO_0051258 : protein polymerization
one$time
24
6
Av NormLogIntensity
2
0
−2
−4
−6
−8
−10
0
5
20
60
Dose
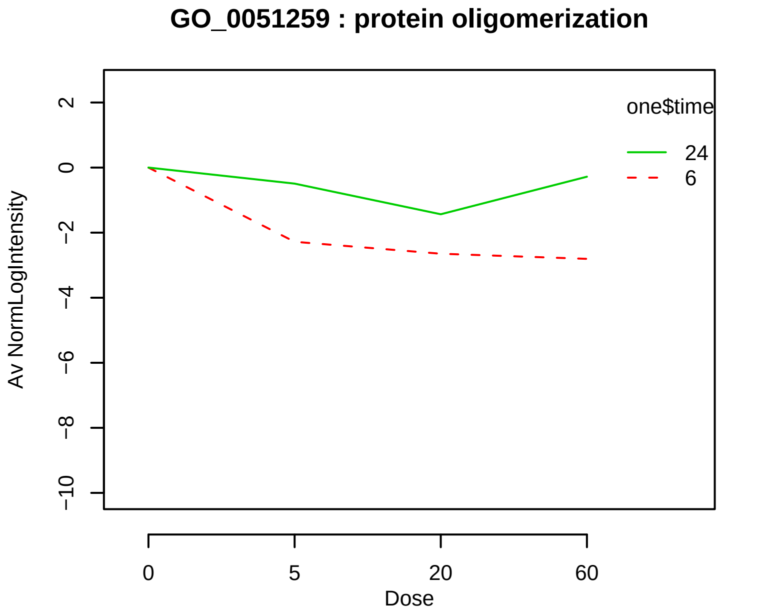
GO_0051259 : protein oligomerization
one$time
24
6
Av NormLogIntensity
2
0
−2
−4
−6
−8
−10
0
5
20
60
Dose
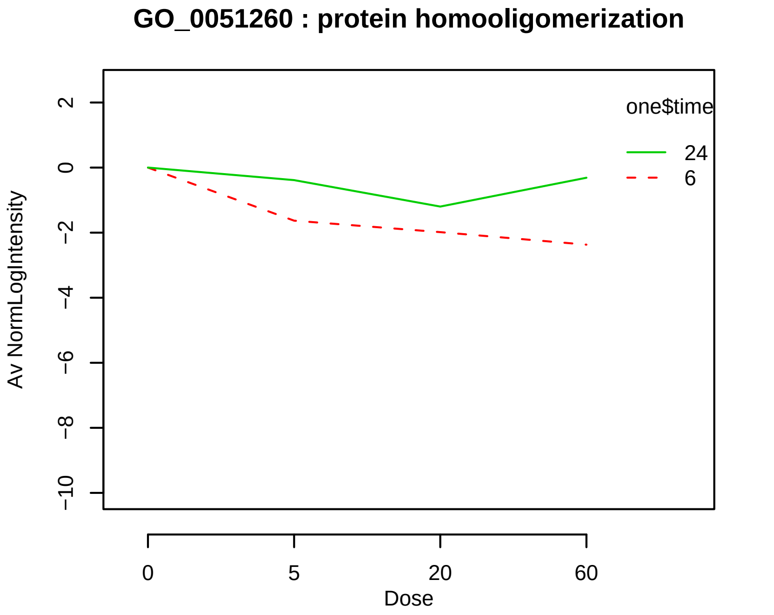
GO_0051260 : protein homooligomerization
one$time
24
6
Av NormLogIntensity
2
0
−2
−4
−6
−8
−10
0
5
20
60
Dose

GO_0051261 : protein depolymerization

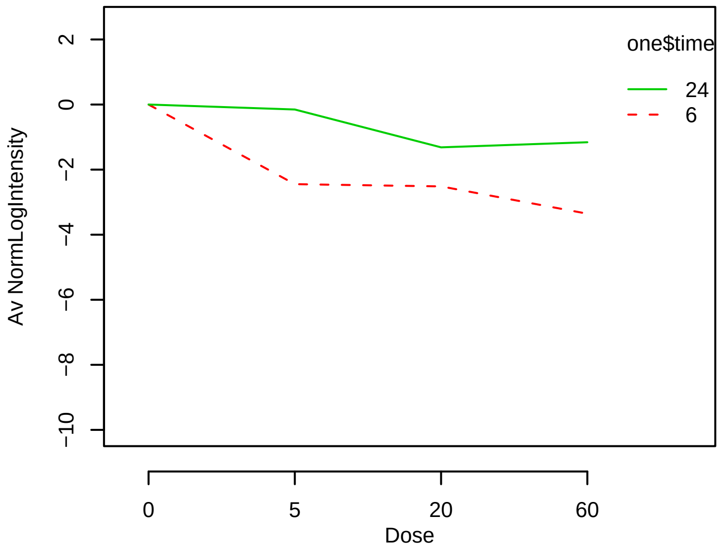

GO_0051262 : protein tetramerization

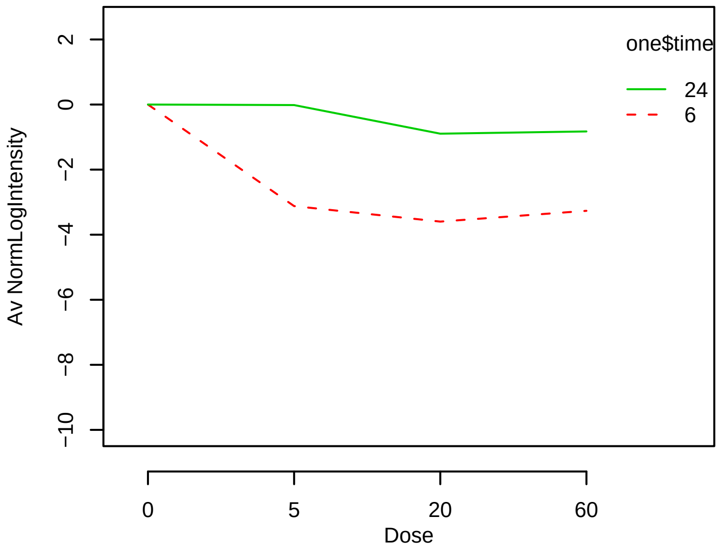

GO_0051270 : regulation of cell motility

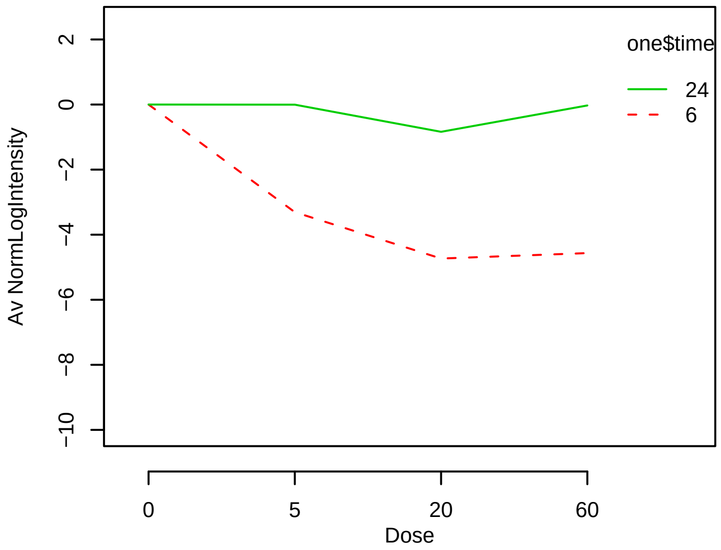

GO_0051271 : negative regulation of cell motility

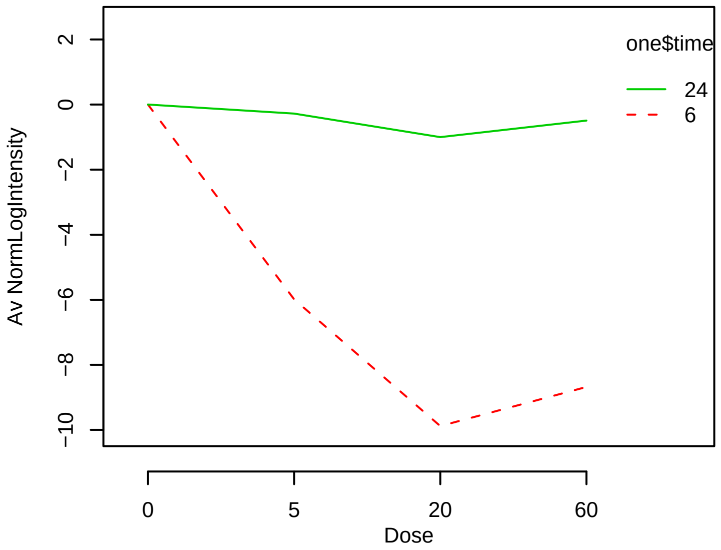

GO_0051272 : positive regulation of cell motility

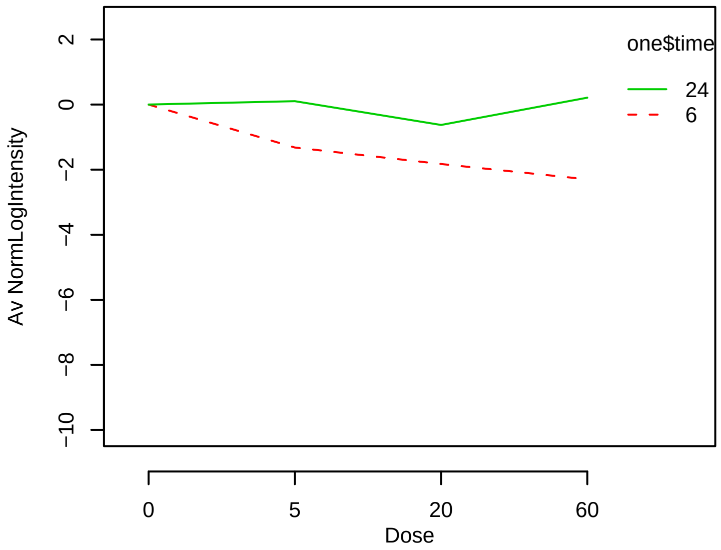

GO_0051289 : protein homotetramerization

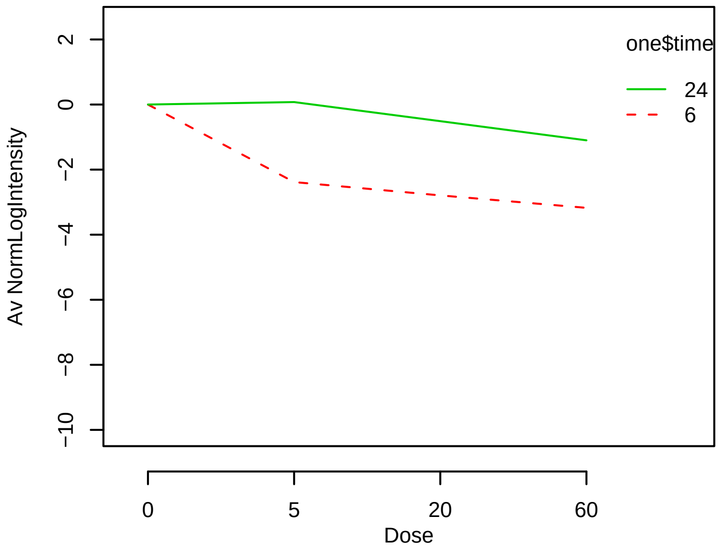

GO_0051291 : protein heterooligomerization

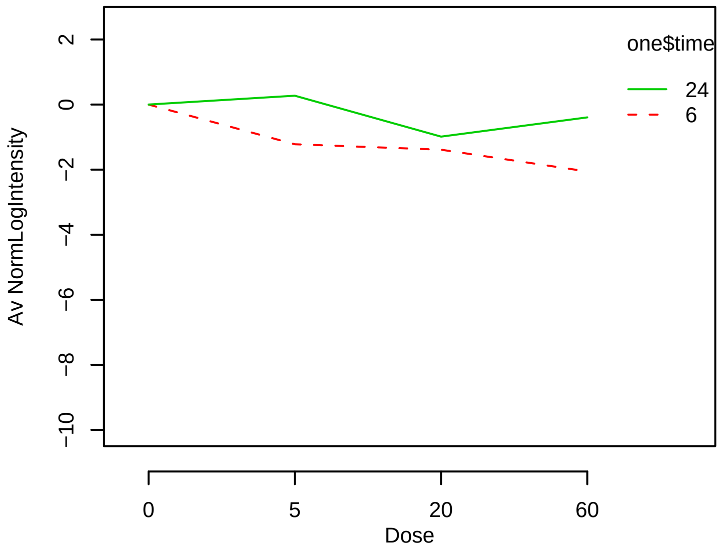

GO_0051297 : centrosome organization and biogenesis

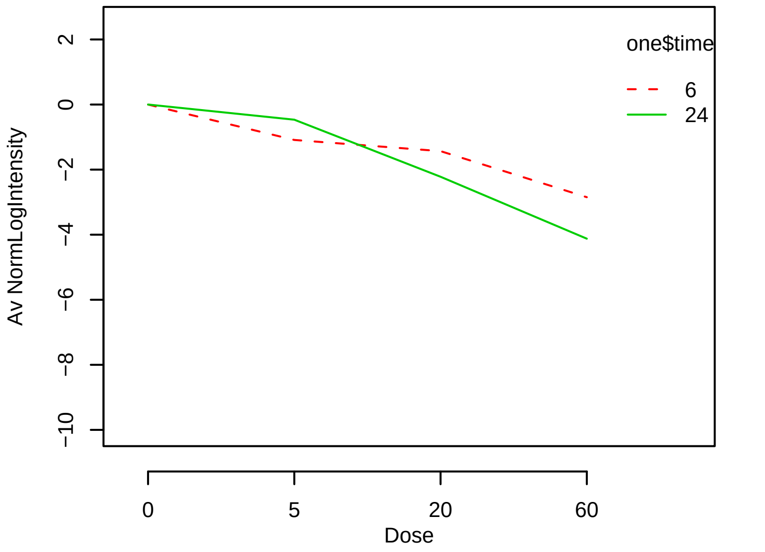

GO_0051298 : centrosome duplication

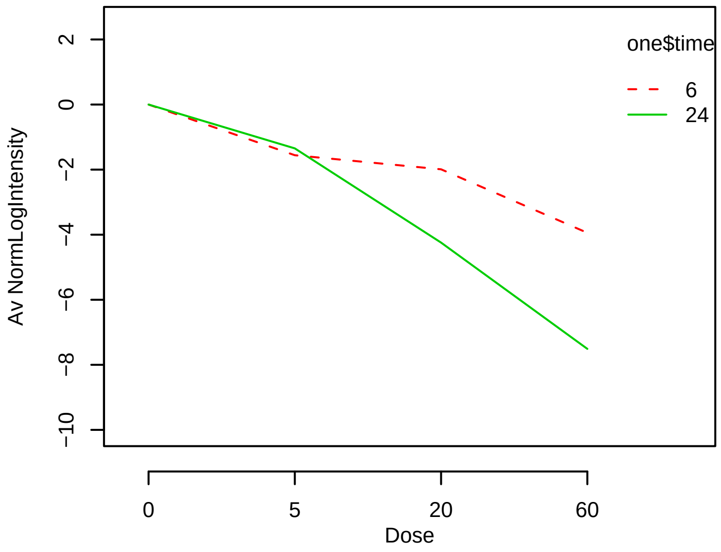

GO_0051301 : cell division

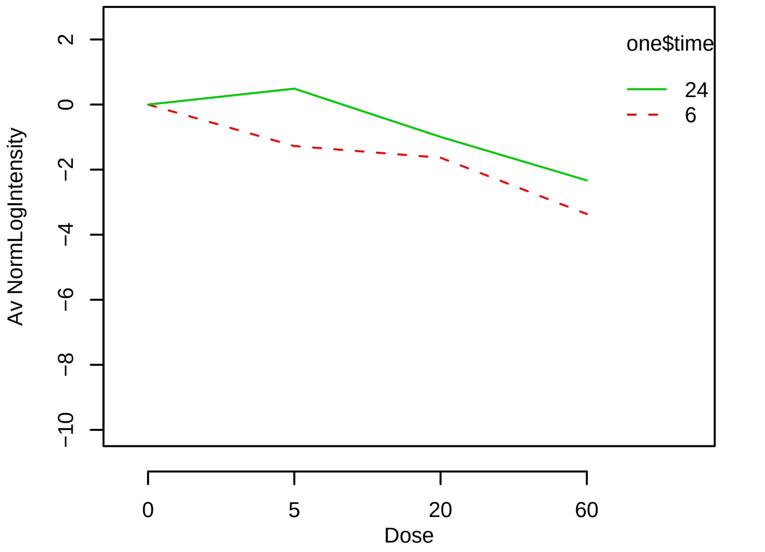

GO_0051318 : G1 phase

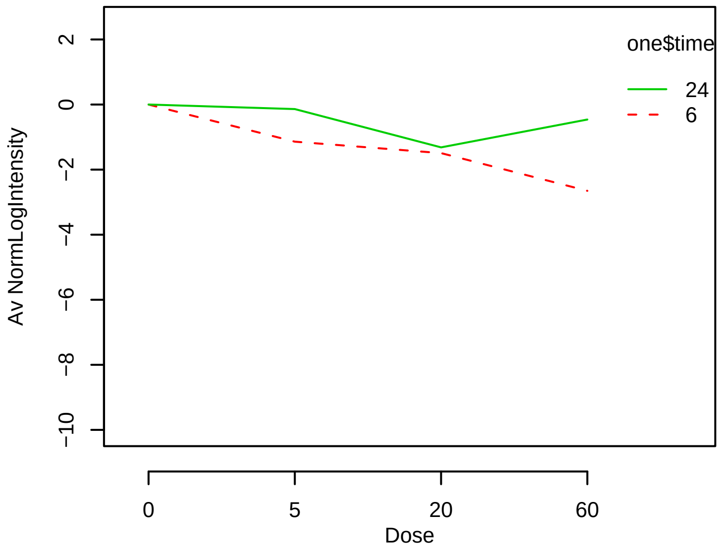

GO_0051319 : G2 phase

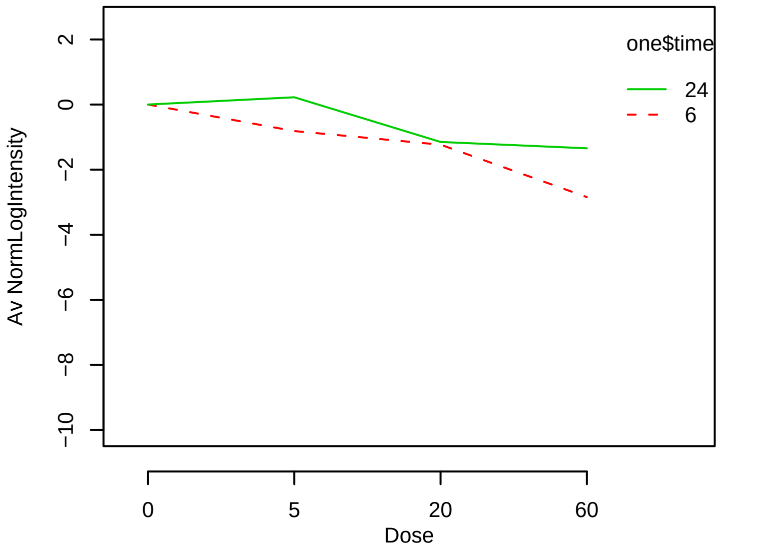

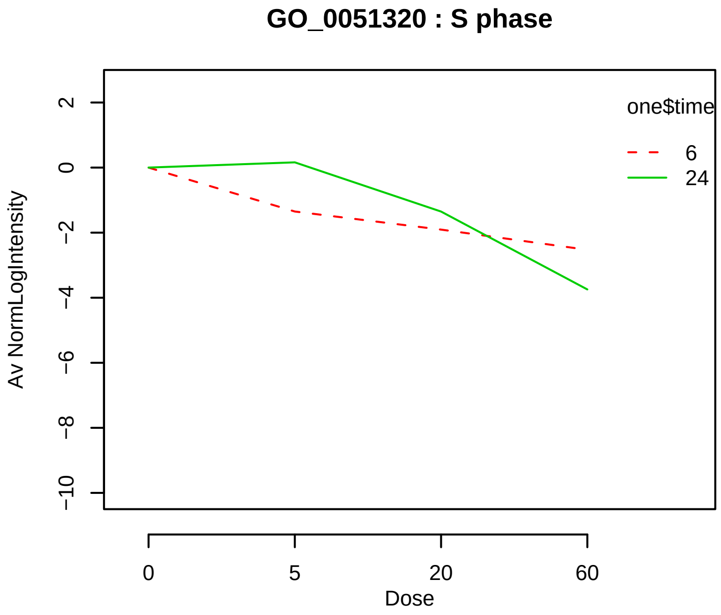
GO_0051320 : S phase
one$time
6
24
Av NormLogIntensity
2
0
−2
−4
−6
−8
−10
0
5
20
60
Dose

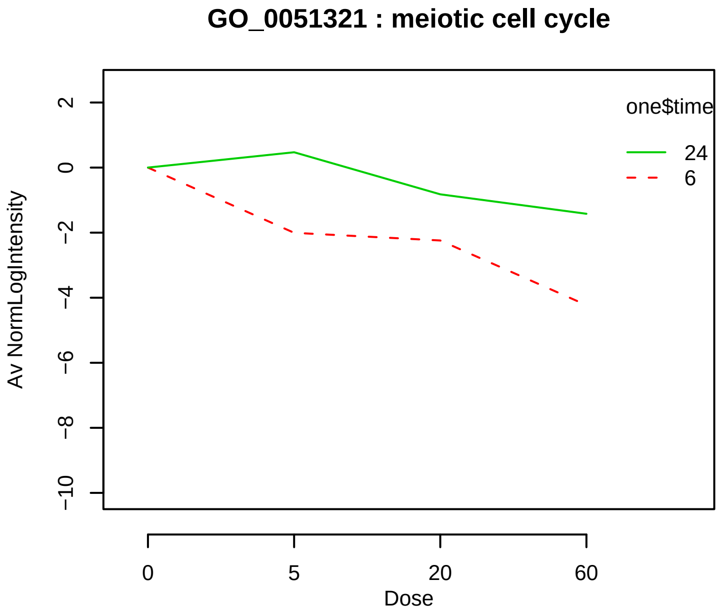
GO_0051321 : meiotic cell cycle
one$time
24
6
Av NormLogIntensity
2
0
−2
−4
−6
−8
−10
0
5
20
60
Dose

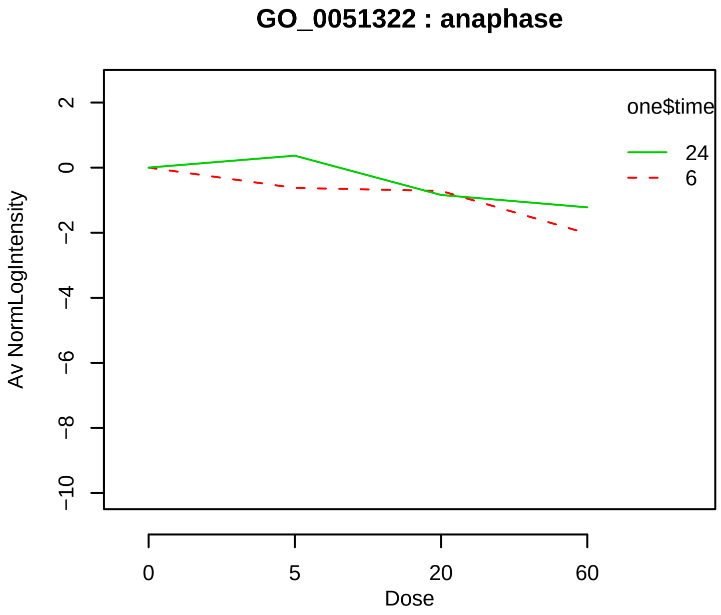
GO_0051322 : anaphase
one$time
24
6
Av NormLogIntensity
2
0
−2
−4
−6
−8
−10
0
5
20
60
Dose

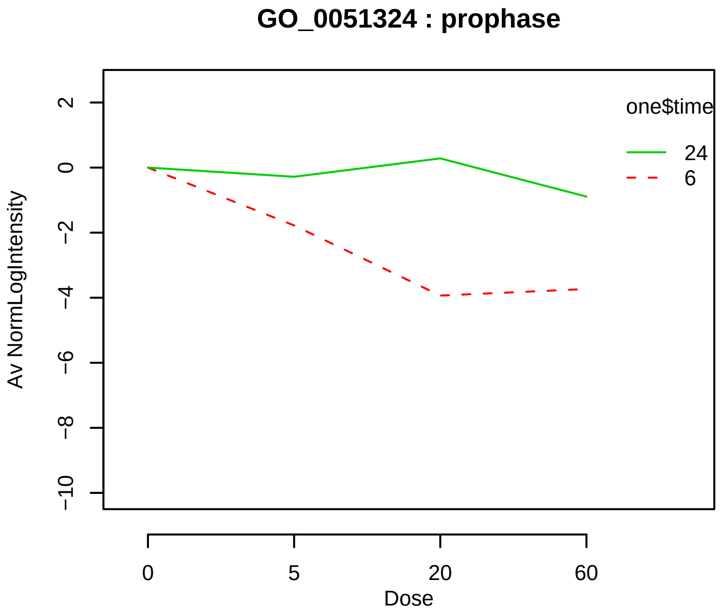
GO_0051324 : prophase
one$time
24
6
Av NormLogIntensity
2
0
−2
−4
−6
−8
−10
0
5
20
60
Dose

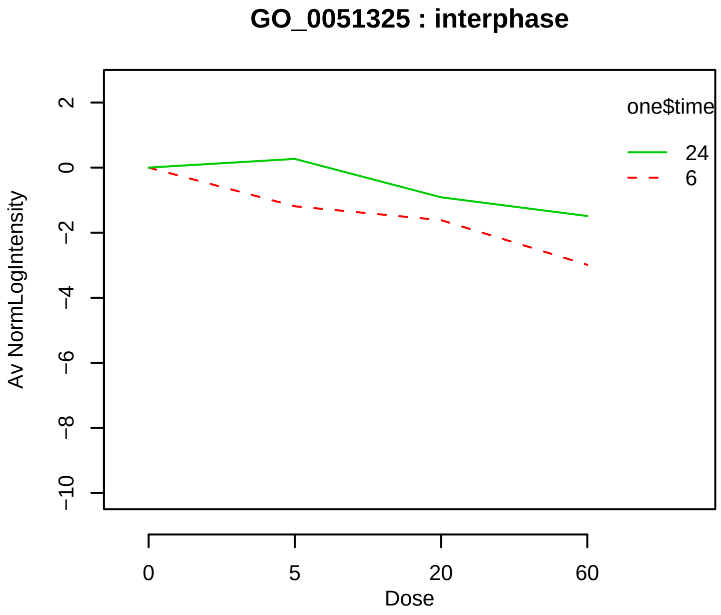
GO_0051325 : interphase
one$time
24
6
Av NormLogIntensity
2
0
−2
−4
−6
−8
−10
0
5
20
60
Dose

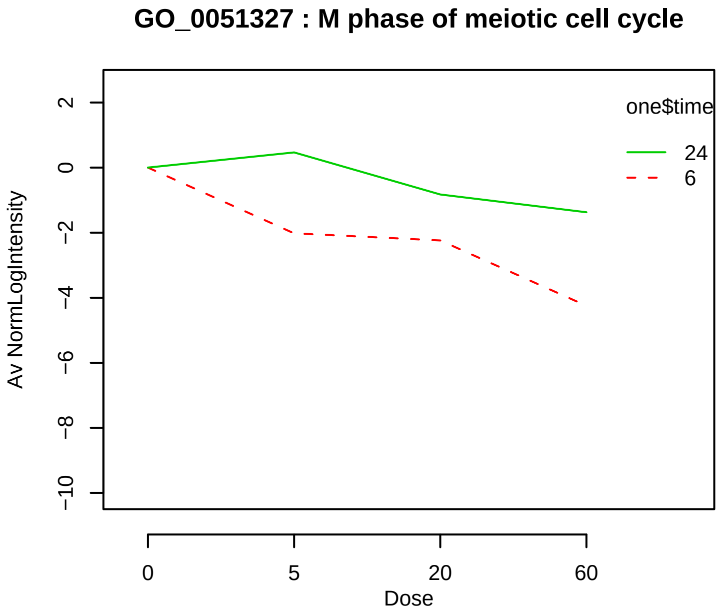
GO_0051327 : M phase of meiotic cell cycle
one$time
24
6
Av NormLogIntensity
2
0
−2
−4
−6
−8
−10
0
5
20
60
Dose

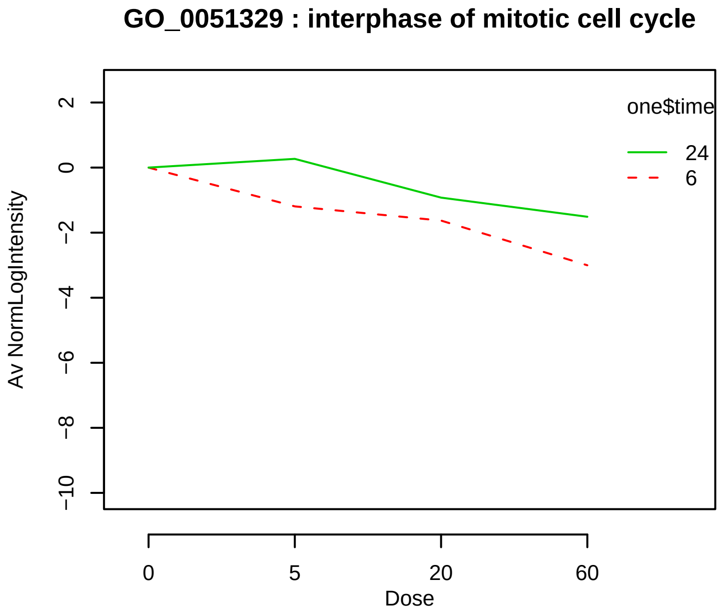
GO_0051329 : interphase of mitotic cell cycle
one$time
24
6
Av NormLogIntensity
2
0
−2
−4
−6
−8
−10
0
5
20
60
Dose

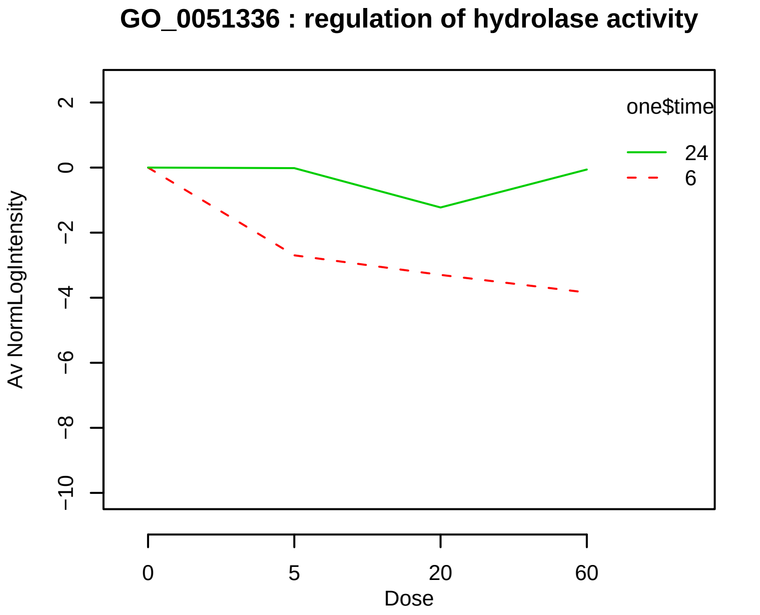
GO_0051336 : regulation of hydrolase activity
one$time
24
6
Av NormLogIntensity
2
0
−2
−4
−6
−8
−10
0
5
20
60
Dose

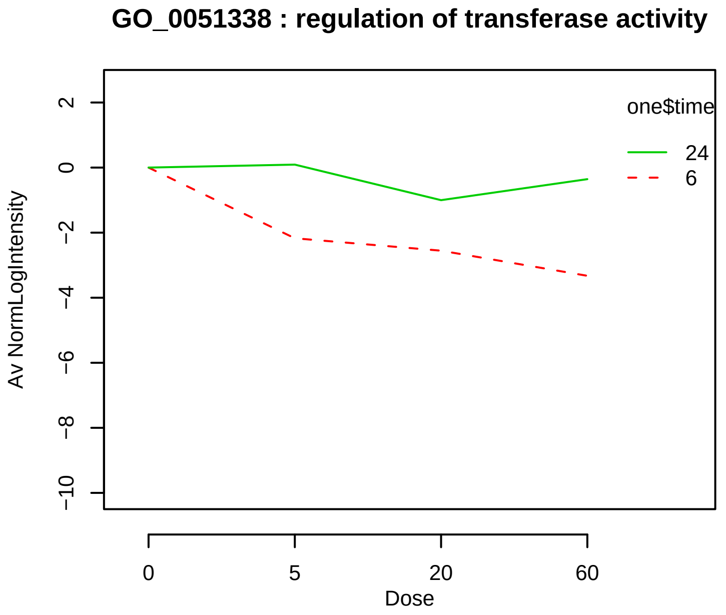
GO_0051338 : regulation of transferase activity
one$time
24
6
Av NormLogIntensity
2
0
−2
−4
−6
−8
−10
0
5
20
60
Dose

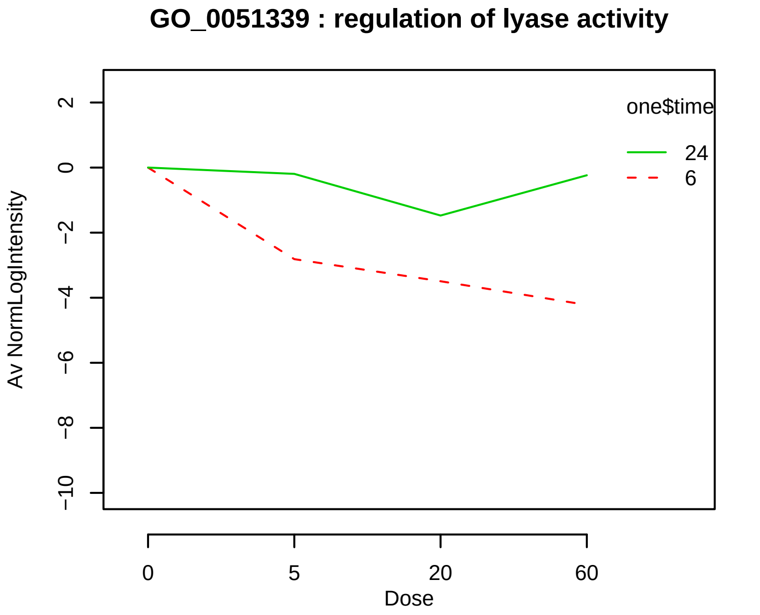
GO_0051339 : regulation of lyase activity
one$time
24
6
Av NormLogIntensity
2
0
−2
−4
−6
−8
−10
0
5
20
60
Dose

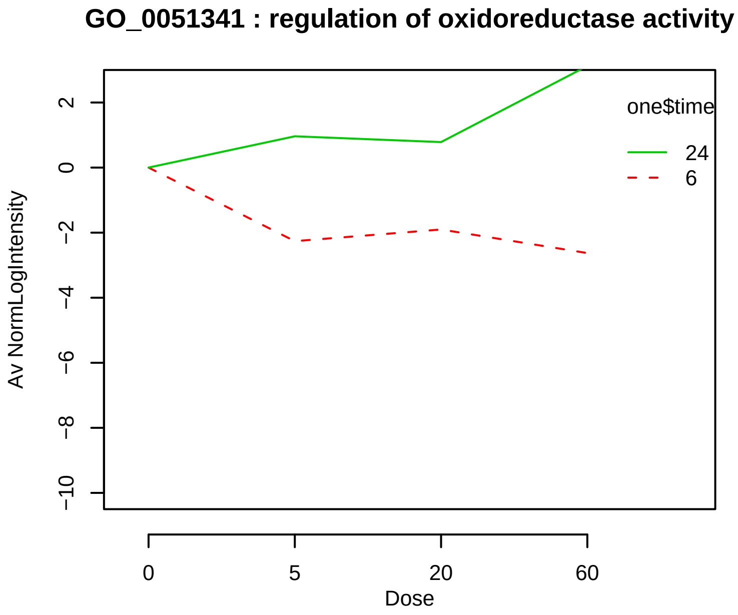
GO_0051341 : regulation of oxidoreductase activity
one$time
24
6
Av NormLogIntensity
2
0
−2
−4
−6
−8
−10
0
5
20
60
Dose

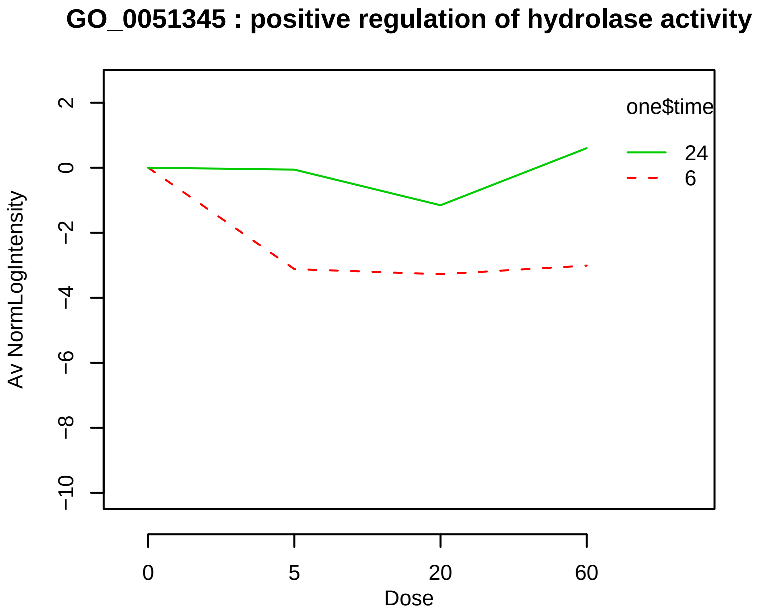
GO_0051345 : positive regulation of hydrolase activity
one$time
24
6
Av NormLogIntensity
2
0
−2
−4
−6
−8
−10
0
5
20
60
Dose

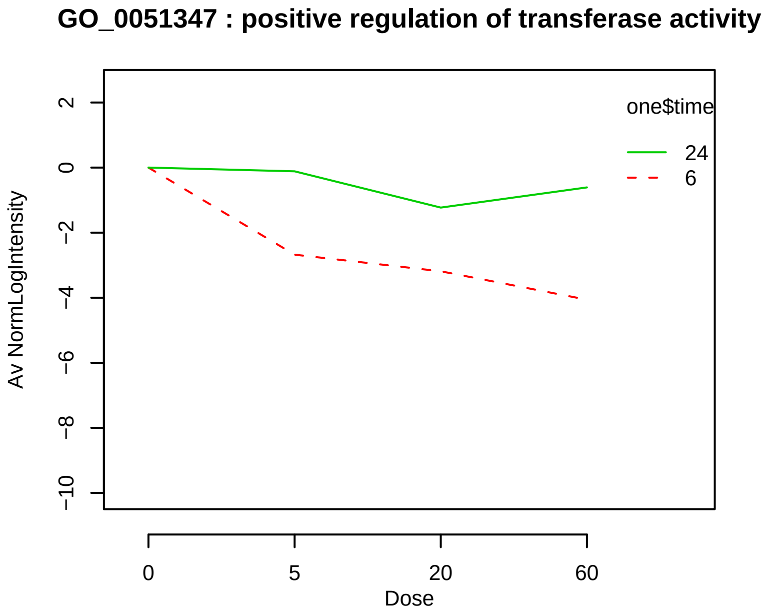
GO_0051347 : positive regulation of transferase activity
Av NormLogIntensity
Dose
one$time
24
6

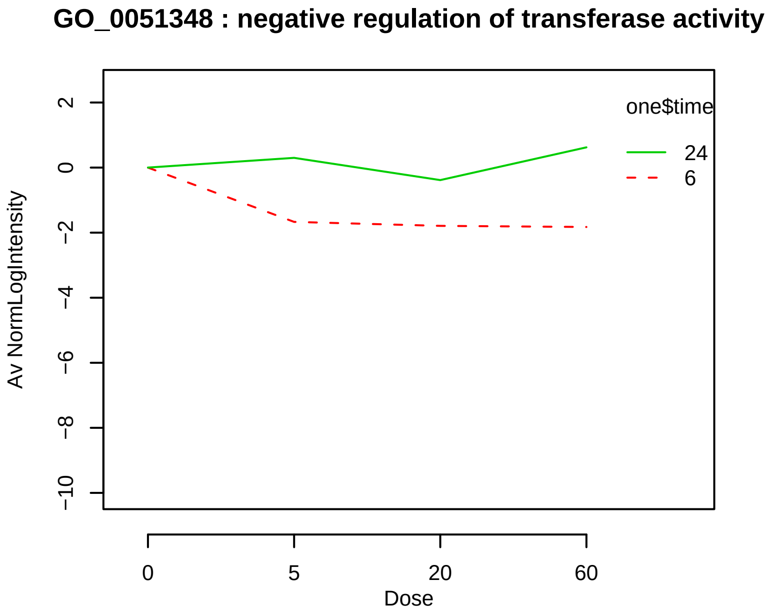
GO_0051348 : negative regulation of transferase activity
Av NormLogIntensity
Dose
one$time
24
6

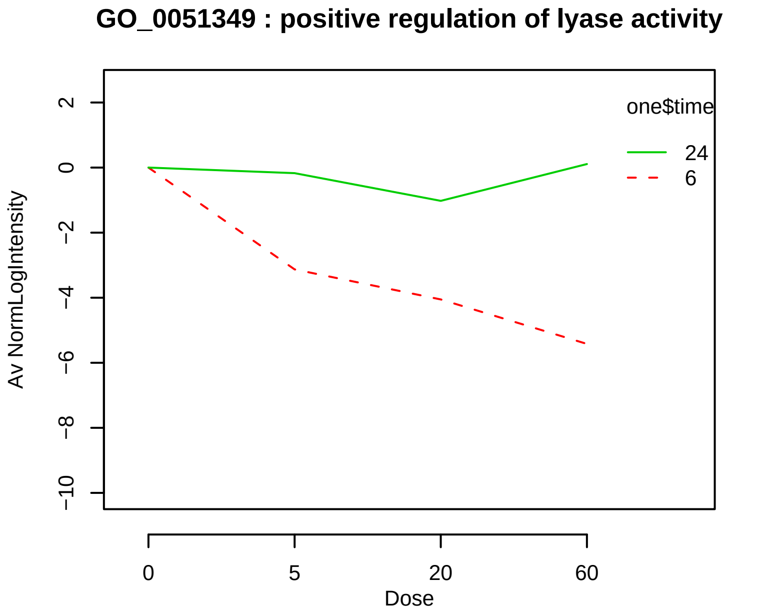
GO_0051349 : positive regulation of lyase activity
Av NormLogIntensity
Dose
one$time
24
6

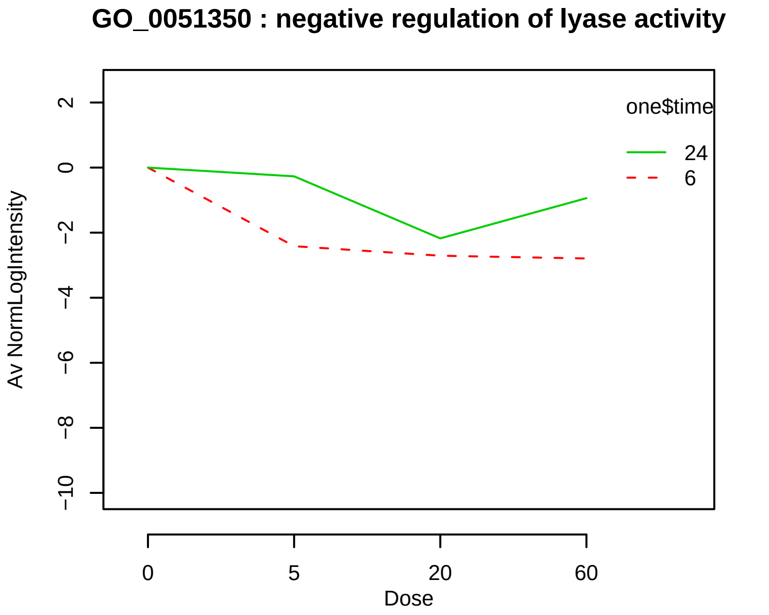
GO_0051350 : negative regulation of lyase activity
Av NormLogIntensity
Dose
one$time
24
6

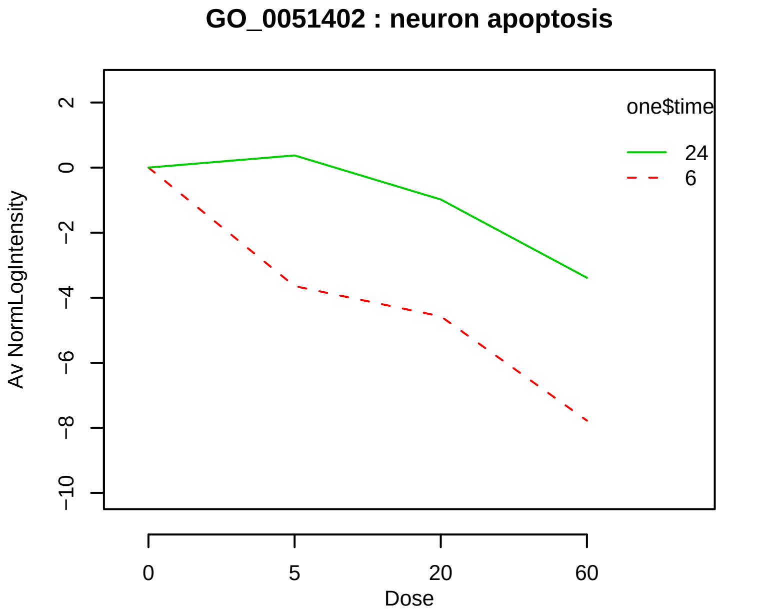
GO_0051402 : neuron apoptosis
Av NormLogIntensity
Dose
one$time
24
6

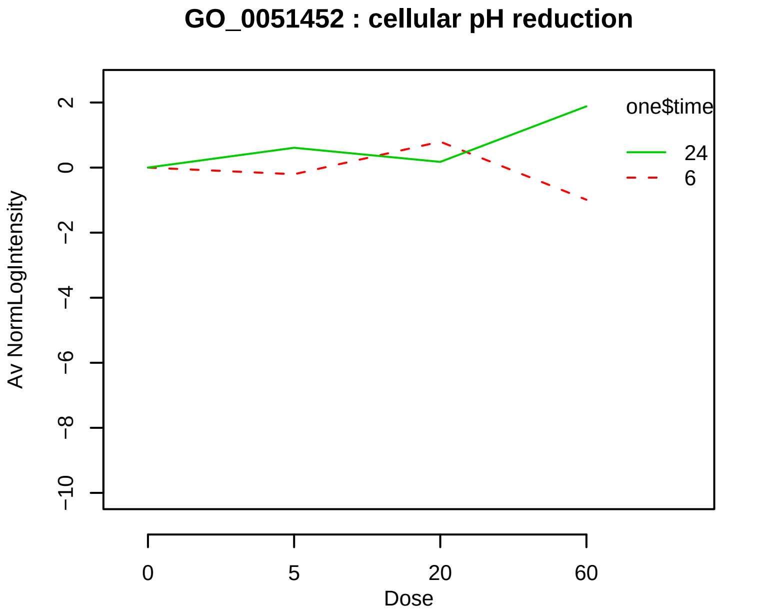
GO_0051452 : cellular pH reduction
Av NormLogIntensity
Dose
one$time
24
6

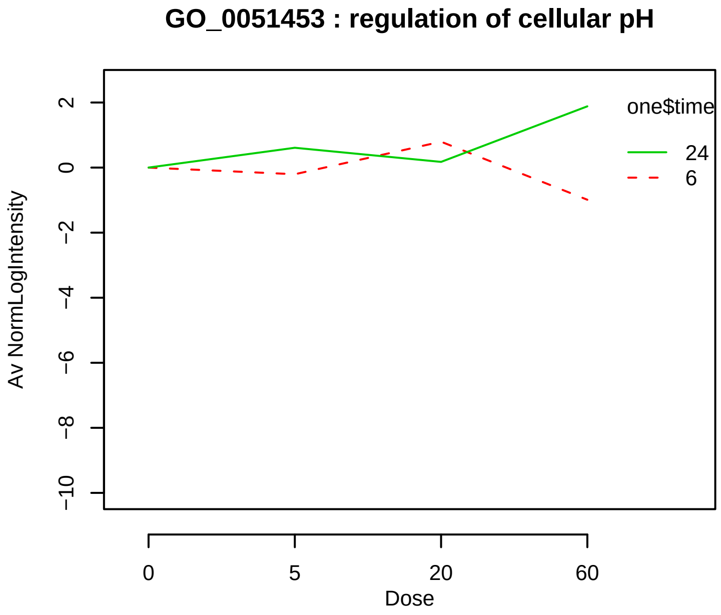
GO_0051453 : regulation of cellular pH
Av NormLogIntensity
Dose
one$time
24
6

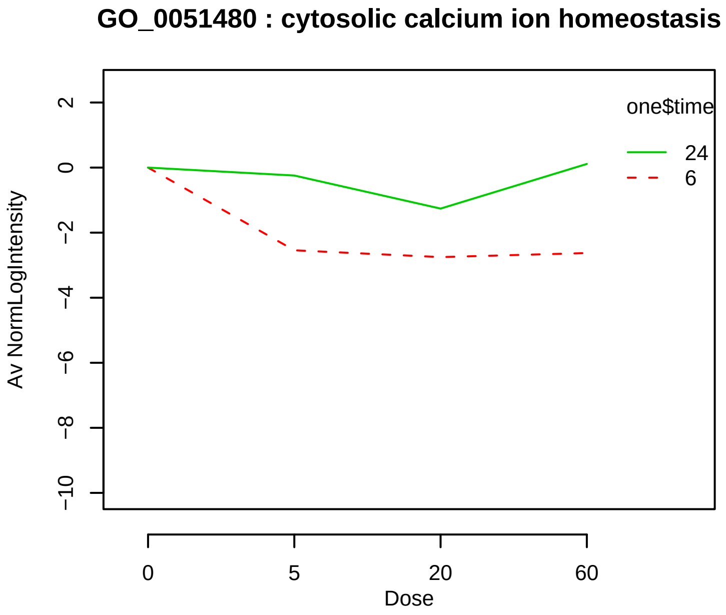
GO_0051480 : cytosolic calcium ion homeostasis
Av NormLogIntensity
Dose
one$time
24
6

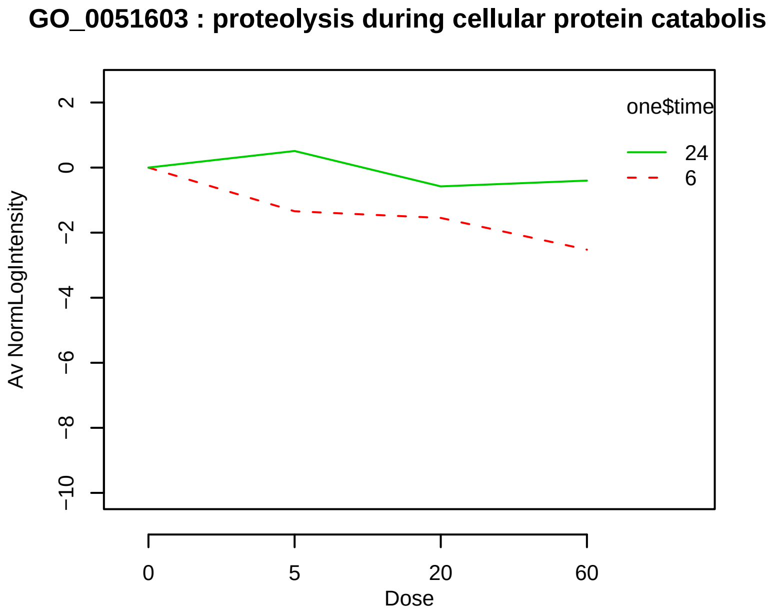
GO_0051603 : proteolysis during cellular protein catabolis
Av NormLogIntensity
Dose
one$time
24
6

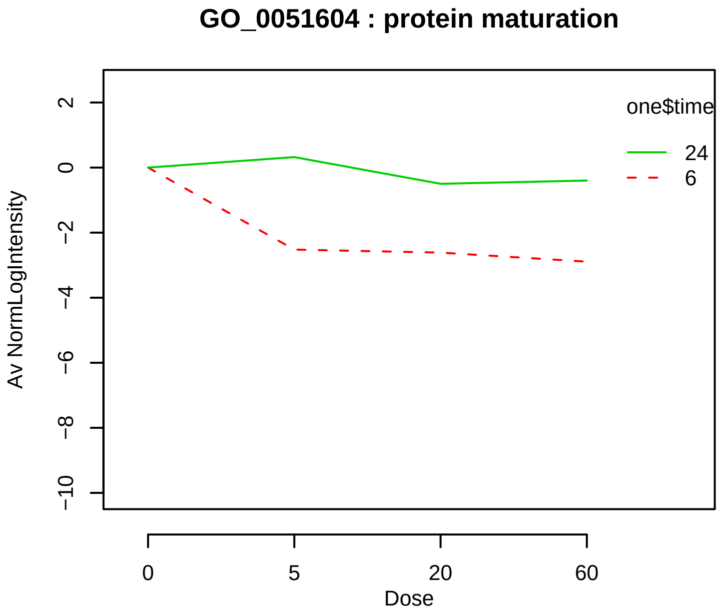
GO_0051604 : protein maturation
Av NormLogIntensity
Dose
one$time
24
6

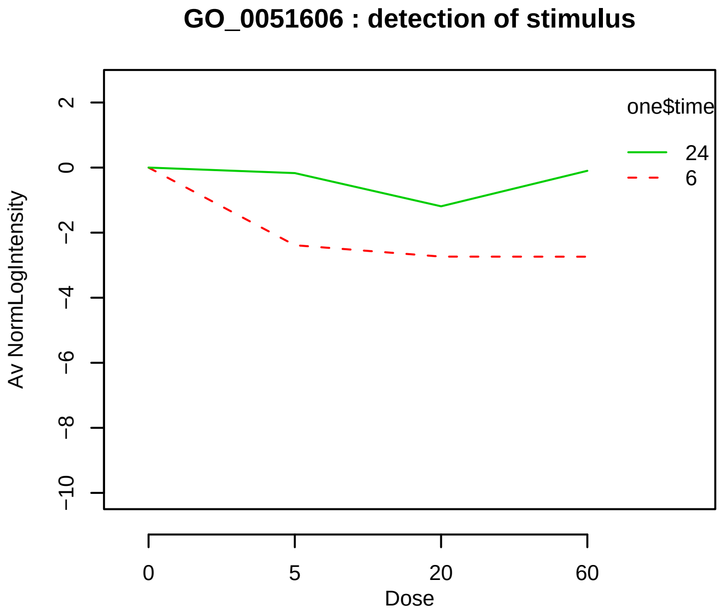
GO_0051606 : detection of stimulus
Av NormLogIntensity
Dose
one$time
24
6

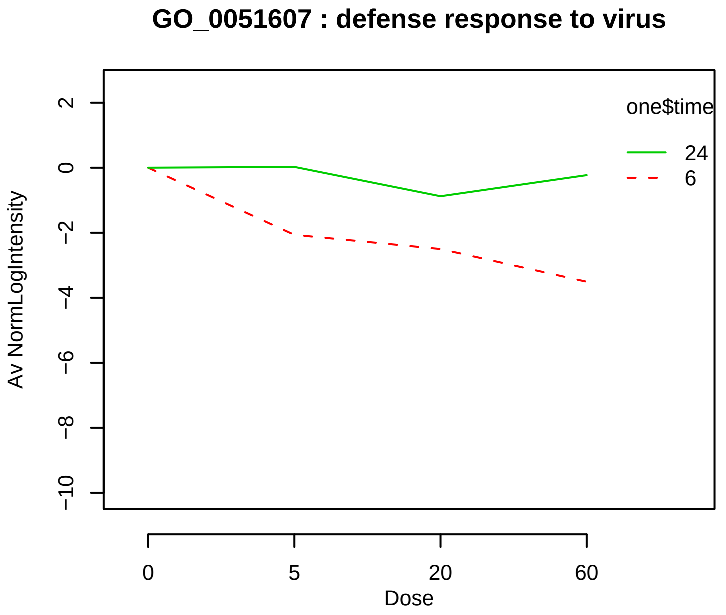
GO_0051607 : defense response to virus
Av NormLogIntensity
Dose
one$time
24
6

GO_0051640 : organelle localization

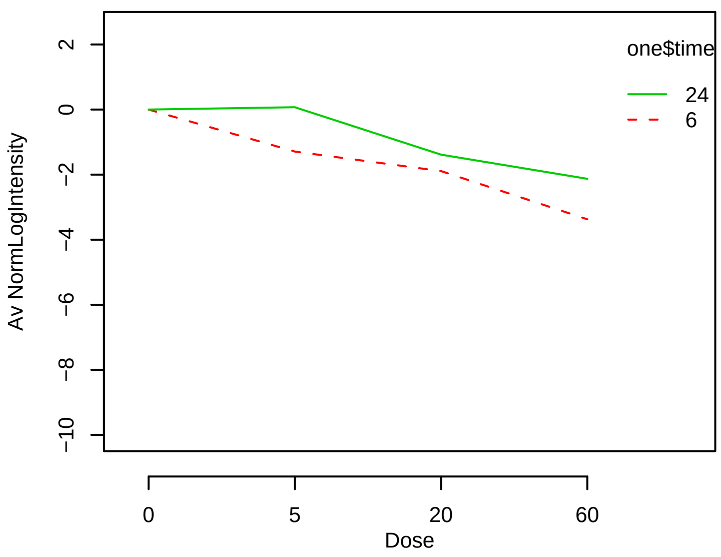

GO_0051647 : nucleus localization

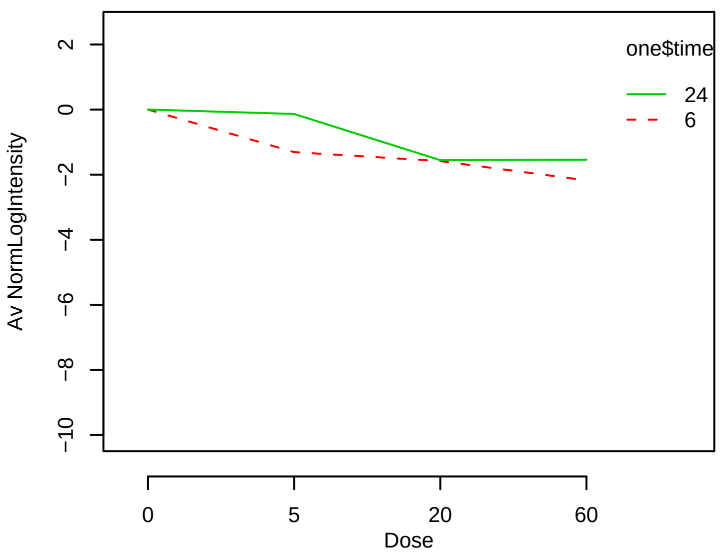

GO_0051656 : establishment of organelle localization

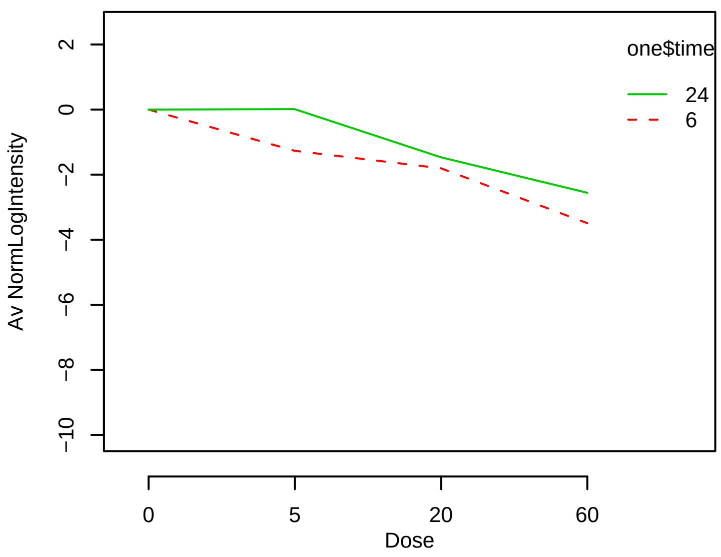

GO_0051668 : localization within membrane

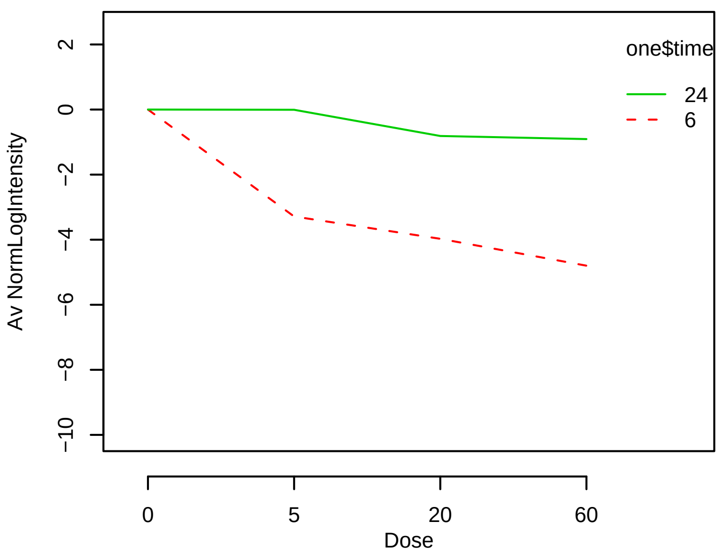

GO_0051693 : actin filament capping

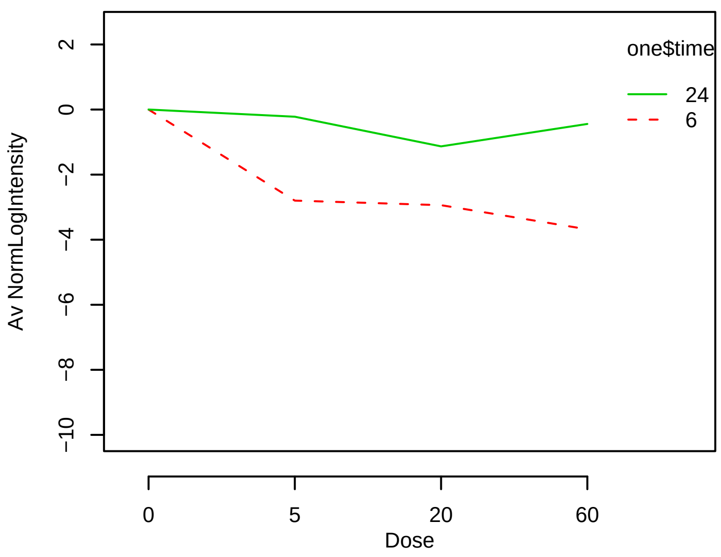

GO_0051701 : interaction with host

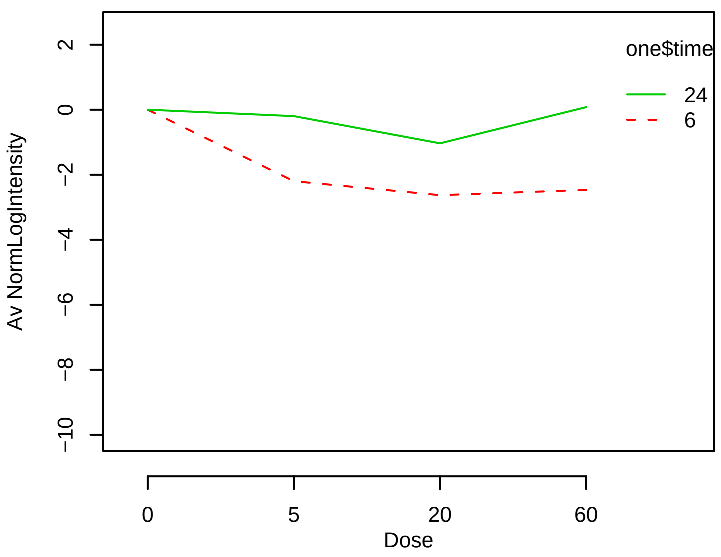

GO_0051704 : interaction between organisms

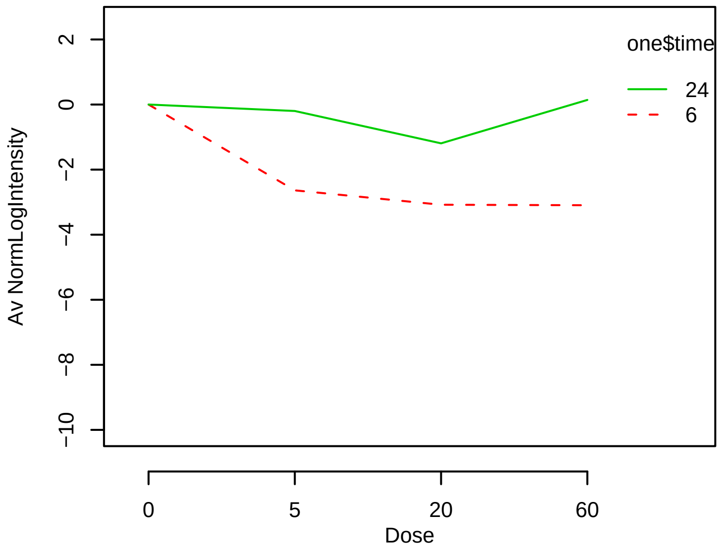

GO_0051705 : behavioral interaction between organisms

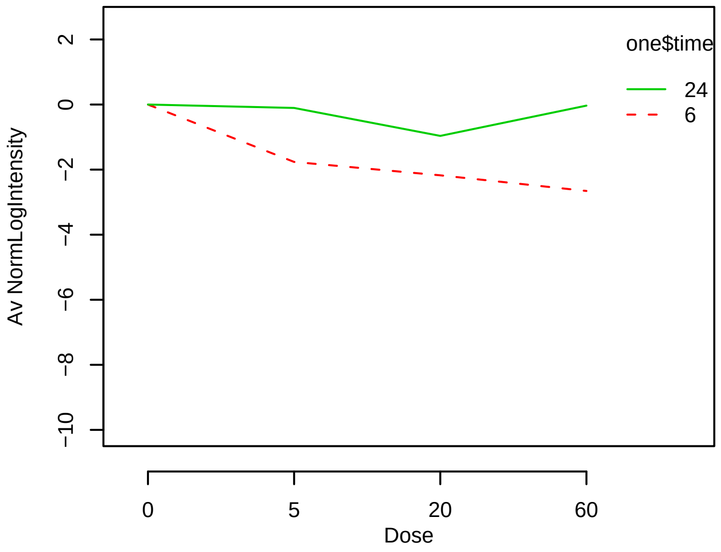

GO_0051706 : physiological interaction between organism

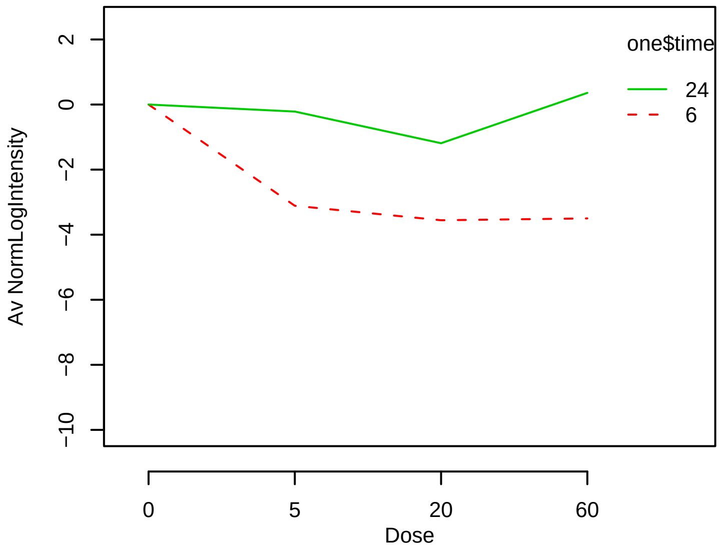

GO_0051716 : cellular response to stimulus

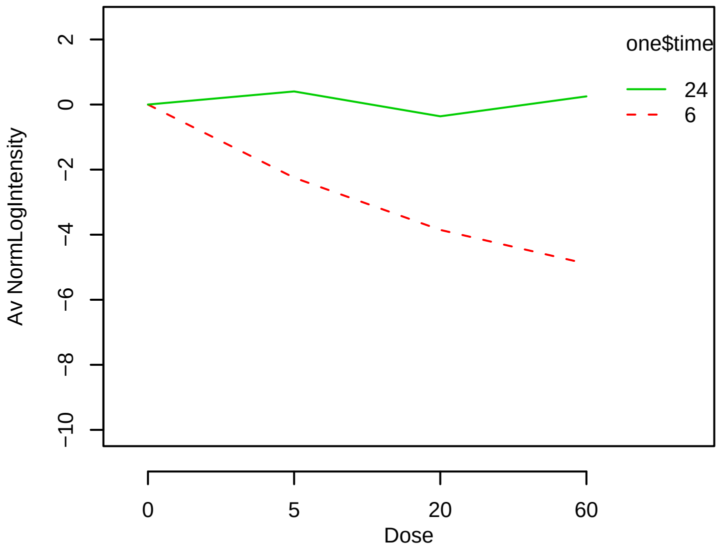

GO_0051789 : response to protein stimulus

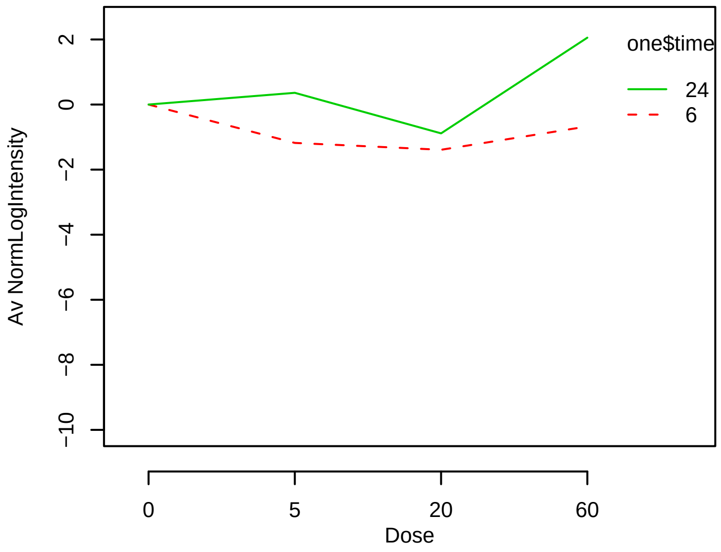

GO_0051806 : entry into cell of other organism during symbi

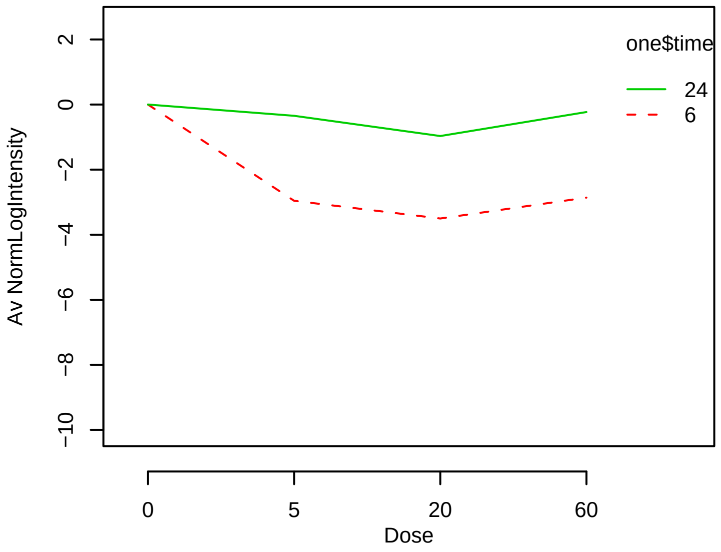

GO_0051828 : entry into other organism during symbiotic int

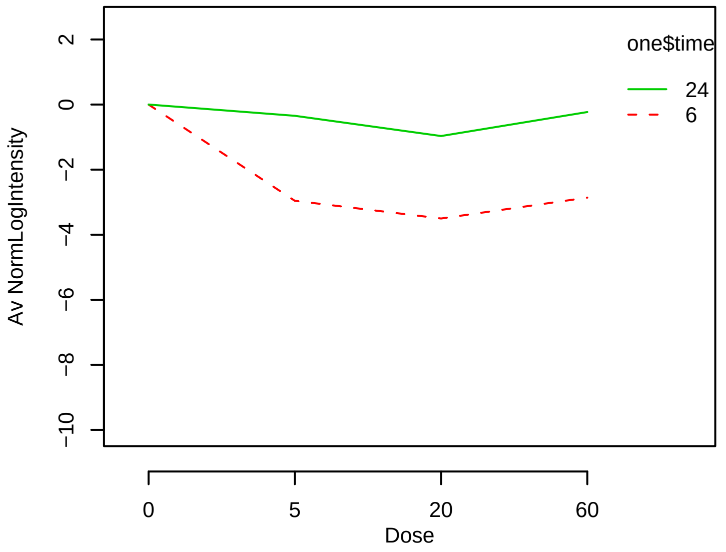

GO_0051923 : sulfation

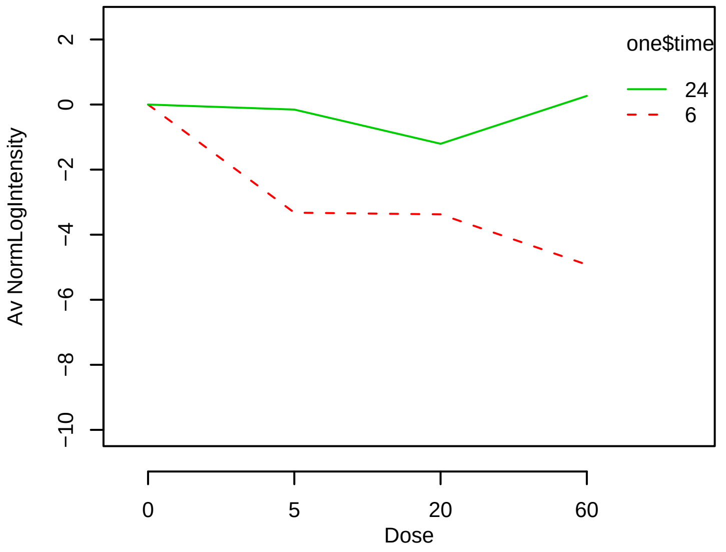

GO_0051924 : regulation of calcium ion transport

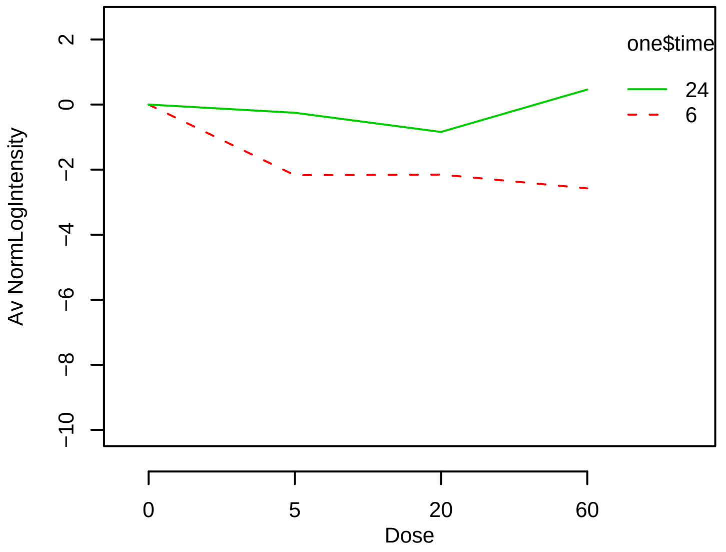

GO_0051969 : regulation of transmission of nerve impuls

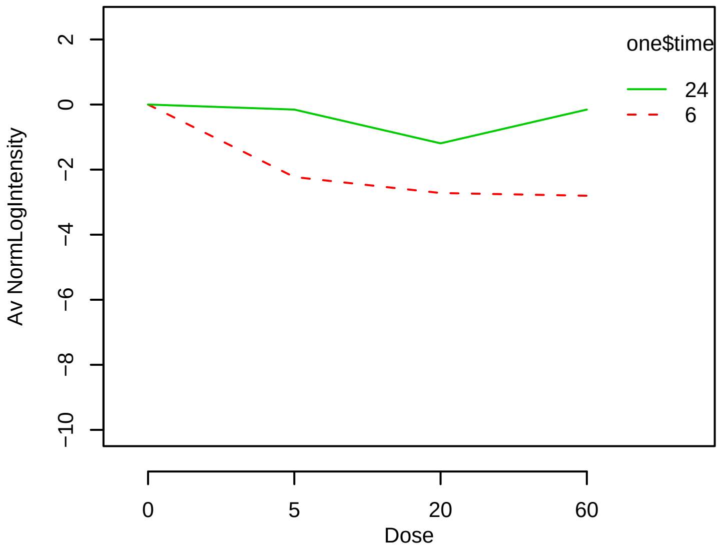

Supplement: Figure S1 — Average standardized gene expression values for each dose and each treatment duration. (0.77 MB PDF) [file pgen.1000115.s001.pdf]
